# Mouse models of immune dysfunction: their neuroanatomical differences reflect their anxiety-behavioural phenotype

**DOI:** 10.1038/s41380-022-01535-5

**Published:** 2022-04-14

**Authors:** Darren J. Fernandes, Shoshana Spring, Christina Corre, Andrew Tu, Lily R. Qiu, Christopher Hammill, Dulcie A. Vousden, T. Leigh Spencer Noakes, Brian J. Nieman, Dawn M. E. Bowdish, Jane A. Foster, Mark R. Palmert, Jason P. Lerch

**Affiliations:** 1 Mouse Imaging Centre, The Hospital for Sick Children, Toronto, ON Canada; 2 Department of Medical Biophysics, The University of Toronto, Toronto, ON Canada; 3 Department of Neurosciences and Mental Health, The Hospital for Sick Children, Toronto, ON Canada; 4 Division of Endocrinology, The Hospital for Sick Children, Toronto, ON Canada; 5 Department of Preclinical Imaging, Wellcome Centre for Integrative Neuroimaging, University of Oxford, Oxford, UK; 6 Translational Medicine, The Hospital for Sick Children, Toronto, ON Canada; 7 Ontario Institute for Cancer Research, Toronto, ON Canada; 8 Department of Medicine, McMaster University, Hamilton, ON Canada; 9 Department of Psychiatry and Behavioral Neurosciences, McMaster University, Hamilton, ON Canada; 10 Departments of Paediatrics and Physiology, The University of Toronto, Toronto, ON Canada

**Keywords:** Neuroscience, Genetics

## Abstract

Extensive evidence supports the role of the immune system in modulating brain function and behaviour. However, past studies have revealed striking heterogeneity in behavioural phenotypes produced from immune system dysfunction. Using magnetic resonance imaging, we studied the neuroanatomical differences among 11 distinct genetically modified mouse lines (*n* = 371), each deficient in a different element of the immune system. We found a significant and heterogeneous effect of immune dysfunction on the brains of both male and female mice. However, by imaging the whole brain and using Bayesian hierarchical modelling, we were able to identify patterns within the heterogeneous phenotype. Certain structures—such as the corpus callosum, midbrain, and thalamus—were more likely to be affected by immune dysfunction. A notable brain–behaviour relationship was identified with neuroanatomy endophenotypes across mouse models clustering according to anxiety-like behaviour phenotypes reported in literature, such as altered volume in brains regions associated with promoting fear response (e.g., the lateral septum and cerebellum). Interestingly, genes with preferential spatial expression in the most commonly affected regions are also associated with multiple sclerosis and other immune-mediated diseases. In total, our data suggest that the immune system modulates anxiety behaviour through well-established brain networks.

## Introduction

The immune system plays an important role in brain–behaviour interactions. During pregnancy, for example, maternal illness increases the risk of nervous system disorders in offspring [1–5] and maternal IL-6 cytokine concentration is associated with altered neurodevelopment in offspring [6–8]. In rodents, in utero exposure to maternal immune activation (MIA) affects brain structure and behaviour [9–11]. Immune system effects on the brain and, ultimately, behaviour are heterogenous and multifaceted. To better understand the complex pathophysiology responsible for immune dysregulation impacting brain development, the role of specific immune components has been investigated using targeted experiments in mice. For example, anxiety-like behaviour is modulated by the production of various cytokines [12–14], adaptive immune response [15–18], nitric oxide synthesis [19], and mast cell activity [20].

While the role of specific immune components is an area of active investigation, existing studies generally examine brain regions or immune components in isolation, thereby sacrificing the ability to observe broader patterns that may explain the observed heterogeneity. Structural magnetic resonance imaging (MRI) is a useful methodology for studying relationships between genetics, brain regions, and behaviour with several studies demonstrating a strong link between the volume of brain structures and organism behaviour [21, 22]. As structural MRI has the benefit of whole-brain coverage without the constraint to identify a region-of-interest (ROI) ahead of time, it allows the identification of novel, unanticipated phenotypes in mouse models of human disease. For example, we previously showed using ex vivo MRI that mice lacking all functional T cells demonstrated a loss of sexual dimorphism in many brain regions [15]. We corroborated this finding with a loss of sexual differentiation in activity-related behaviours, highlighting the importance of T cells for the development of sex differences in neuroanatomy and behaviour. However, such studies remain rare and the brain-wide effects of alterations in other elements of the immune system are unknown. High-throughput data acquisition and analysis of structural MRI allows for the investigation of similarities and differences across a range of immune system mutations.

Thus, here we used whole-brain MRI to explore the effect of immune system mutations on the brains of male and female mice. We imaged the brains of 11 genetically engineered mouse lines, each deficient in different elements of the immune system, using a standard pipeline for specimen preparation, data acquisition, and image registration. Brain structure was highly susceptible to immune dysfunction and we found regional variations in this susceptibility. The most susceptible regions tended to express genes associated with immune-mediated diseases. We also identified that strains with similar anxiety-behavioural phenotypes displayed analogous neuroanatomical effects. Lastly, we identified candidate brain regions and networks that may be involved in modulating the anxiety behaviours seen in mouse models of immune dysfunction.

## Methods

### Mice

The study consisted of 371 mice (Table 1) from 11 different genetically engineered strains (Jackson Labs, Bar Harbor, Maine, USA)—which encapsulate the different components of the immune system (Supplementary Fig. 1). Sample size was chosen to detect 4% hippocampal volume (Type I error = 0.05, power = 80%) [23]. All mice were housed in individually ventilated cages (10 air changes/hour at 20–22 °C), and provided with *ad libitum* water and diet#2918 (Envigo, Indianapolis, Indiana, USA). All breeding colonies were maintained using homozygous mutants with two exceptions: Cxcr2 colony was maintained using heterozygous mutants due to the fragility of homozygotes [24], and Rag2 mice were ordered directly from Jackson Labs. Genotypes were determined from tail biopsies using real-time PCR (Transnetyx, Cordova, Tennessee, USA) or fur colour in the case of the Kit strain. The control group consisted of C57Bl/6J mice and Cxcr2 wild-type littermates [25]. The two wild-type control strains were pooled for analyses as there were no significant neuroanatomical differences between them [26]. On P65±3 (mean±max range), mice were sacrificed for fixation (physical measurements reported in Supplementary Table 1 and Supplementary Fig. 2). Fixed brain samples were prepared for MRI using a previously described fixation protocol [27]. Briefly, mice were perfused with a solution of phosphate-buffered saline (Wisent Incorporated, Saint-Jean-Baptiste, Quebec, Canada), gadoteridol (Bracco Diagnostics Incorporated, Monroe Township, New Jersey, USA), and heparin (Sandoz Canada Incorporated, Mississauga, Ontario, Canada) followed by gadoteridol and 4% paraformaldehyde (PFA) (Electron Microscopy Sciences, Hatfield, Pennsylvania, USA). After decapitation, brains (kept within the skull) were stored at 4 °C in a solution of PFA, gadoteridol, and sodium trinitride (Fisher Scientific, Mississauga, Ontario, Canada). Brains were imaged 7–8 weeks postmortem [28].

**Table 1:** Information on strains used in this study.

Name	Strain	Allele type	Jackson Labs stock no	Number of female (F) and male (M) mice	Mutant strain details
CD4	B6.129S2- Cd4tm1Mak/J	Targeted (Null/Knockout)	002663	15F/15M	Significant block in CD4+ T cell development in homozygous mutants. Class II restricted deficit in helper T cell activity and other T cell responses. CD8+ T cells and myeloid component development is unaltered. Cytotoxic T cell activity against viruses remains in the normal range [76]
CD8	B6.129S2- Cd8atm1Mak/J	Targeted (Null/Knockout)	002665	15F/17M	Cytotoxic T cell population absent from thymus and lymph nodes of homozygous mutants resulting in a dramatic decrease in cytotoxic response of T lymphocytes against alloantigens and viral antigens. Helper T cell development and function appears unaltered [77]
Cxcr2	B6.129S2(C)- Cxcr2tm1Mwm/J	Targeted (Null/Knockout)	006848	15F/15M	Homozygous mutant mice display impaired neutrophil recruitment and decreased pathogen clearance during innate immune responses. Mutants may exhibit several abnormalities including splenomegaly, lymphadenopathy, neurological defects, impaired wound healing, impaired angiogenesis, altered growth of induced/implanted tumours and increased susceptibility to various pathogens [78]
Ighm	B6.129S2- Ighmtm1Cgn/J	Targeted (Null/Knockout)	002288	15F/15M	Absence of mature B cells in peripheral blood lymphocytes and spleen cells of homozygous mutant mice. Lack of membrane-bound IgM expression with arrested development at the stage of pre-B-cell maturation [79]
IL-6	B6.129S2- Il6tm1Kopf/J	Targeted (Null/Knockout)	002650	15F/15M	Homozygous mutant mice are deficient in IL-6 cytokines. Impaired viral infection control and compromised inflammatory responses to tissue damage or infection. Impaired T cell-dependent antibody response [80]
IL-10	B6.129P2- Il10tm1Cgn/J	Targeted (Null/Knockout)	002251	16F/15M	IL-10-deficient mice spontaneously develop generalised enterocolitis in conventional housing and milder intestinal inflammation under specific pathogen-free conditions. IL-10 deficiency is associated with elevated inflammatory markers, altered lymphocyte and myeloid profiles, altered responses to inflammatory stimuli, and elevated occurrence of colorectal adenocarcinoma [81, 82]
IL-18	B6.129P2- Il18tm1Aki/J	Targeted (Null/Knockout)	004130	16F/15M	IL-18-deficient mice exhibit reduced levels of IFNɣ in response to infection or lipopolysaccharide challenge, defective natural killer cell activity and impaired helper T cell response [83]
Kit	B6.Cg-KitW-sh/ HNihrJaeBsmGlliJ	Spontaneous	012861	15F/15M	Homozygous mutants are profoundly mast cell-deficient. Levels of other hematopoietic cells and lymphoid cells are normal [84–86]
Nos2	B6.129P2- Nos2tm1Lau/J	Targeted (Null/Knockout)	002609	15F/15M	Mutants are deficient in the inducible isoform of nitric oxide synthase. These mice lack a serum nitric oxide response implicated in the pathogenesis of septic shock. Nos2 deficiency results in altered responses to various infections and impairs wound healing properties of fibroblasts [87]
Rag1	B6.129S7- Rag1tm1Mom/J	Targeted (Null/Knockout)	002216	16F/14M	Recombinase Activating Gene (Rag) protein products are expressed in B and T lymphocytes and are required to generate the repertoire of immunoglobulins and T cell receptors by V(D)J recombination. Mice homozygous for the Rag1 mutation produce no mature T cells or B cells [88]
Rag2	B6(Cg)- Rag2tm1.1Cgn/J	Targeted (Null/Knockout)	008449	15F/15M	Rag2 knockout mice produce no mature T cells or B cells (as Rag1). The Rag1 and Rag2 protein sequences are not related but both are needed to form the complex necessary for V(D)J recombination [89, 90]
WT	C57BL6/J	–	000664	15F/15M	–
WT (Cxcr2 littermate controls)	B6.129S2(C)- Cxcr2tm1Mwm/J	–	006848	5F/5M	–

Only homozygous mutants were used.

All animal experiments were approved by The Centre for Phenogenomics (TCP) Animal Care Committee (AUP 17-0175H) in accordance with recommendations of the Canadian Council on Animal Care, the requirements under the Animals for Research Act, RSO 1980, and the TCP Committee Policies and Guidelines. As subjects were assigned to groups based on sex and strain, experimenters were not blinded to subject and subjects were not randomised.

### Image acquisition and registration

A multi-channel 7.0-T MRI scanner with a 40-cm diameter bore (Varian Incorporated, Palo Alto, California, USA) was used to acquire 40-micron-isotropic images of 16 brains concurrently [29] (parameters: T2W 3D FSE cylindrical k-space acquisition sequence, TR/TE/ETL = 350 ms/12 ms/6, two averages, FOV/matrix-size = 20 × 20 × 25 mm/504 × 504 × 630) [30]. All 371 brain images were registered together to create a consensus average (Supplementary Fig. 3) using a previously published pipeline [23] built on the mni_autoreg [31], ANTS [32], and pydpiper [33] toolkits. The MAGeT [34] pipeline was used to automatically segment each of the 371 brain images into 336 structures using previously published segmentations [35–39] in order to determine structure volumes. Assessment of registration convergence is illustrated in Supplementary Figs. 4 and 5. Data are available upon reasonable request.

### Frequentist statistics

For each structure, volume was fit using a linear model with predictors of sex, strain, and their interactions; and partial F-test was used to assess significance. Multiple comparisons were corrected using false-discovery rate (FDR) [40]. *Post hoc* simulations to assess power were also performed (Supplementary Fig. 6). Variances were similar between groups (Supplementary Table 4).

### Bayesian statistics

Structure volumes were standardised using Z-score. An anatomical hierarchy [41] was used to reduce the number of structures to 95 bilateral brain regions (Supplementary Figs. 7 and 8). A Bayesian hierarchical model (BHM) [42] was used to explore the effects of strain on neuroanatomical volumes of all 95 structures. It contained global and structure-specific predictors of sex, strain, and their interaction, and individual-specific intercept (priors and healthy model diagnostics given in Supplementary Table 2). Similar results were seen when refitting the model with different priors for the correlation matrices. For each structure, the posterior distribution was used to compute the effect-size (*d*) distribution.

The posterior also provided the distribution of neuroanatomy phenotype as beta-values for each structure and strain (referenced to wild-type). Hellinger distance was used to assess the dissimilarity of neuroanatomy phenotype between each pair of strains. Strains were annotated with anxiety phenotype based on existing literature (increased, decreased, or unchanged; Supplementary Table 3). Since Cxcr2 strain did not have an anxiety phenotype reported in the literature, it was assumed to be unchanged. To assess whether strains with similar anxiety phenotype have lower Hellinger distances, permutation testing was performed (10^5^ iterations). Similar results were seen after excluding the Cxcr2 strain and when using a bootstrap-based networking procedure [21]. To determine the effect of anxiety for each structure, the posterior distribution was used to calculate the anxiety effect size (*η*^2^) distribution. Hellinger distance, *η*^2^, and *d* statistics are illustrated in Supplementary Fig. 9.

### Preferential gene expression and disease enrichment analysis

Using previously published methods [43, 44] and the Allen Brain Institute (ABI) gene expression atlas [41], we explored the relationship between brain regions with altered volume and gene expression. Determined by the BHM, the top 25 brain structures with the highest absolute effect size (i.e., all structures with |*d*| > 1) constituted the ROI for this analysis. Preferential expression for each gene was evaluated using fold-change: defined as mean expression in ROI divided by mean expression in the brain.

For disease enrichment and Gene Ontology (GO) enrichment analysis, all genes were part of the background set, while the target set was the top 4000 genes with the highest preferential expression. Similar results were seen with target sets of the top 3000 and 5000 genes. For disease enrichment analysis, mouse genes were annotated with human diseases using two databases: NCBI [45] (mapping mouse genes to homologous human genes) and DisGeNET [46] (annotating human genes with associated diseases). GO enrichment analysis was conducted using GOrilla [47]. Significance was assessed using hypergeometric tests with FDR correction. Differences in probability density were assessed for significance using the Kolmogorov–Smirnov test. ABI developmental gene expression atlas [48] was used to investigate gene expression patterns through development. Expression was clustered using *k*-means with *k* = 4 determined by elbow-method [49].

## Results

### Immune dysfunction affects male and female neuroanatomy

**Fig. 1:**
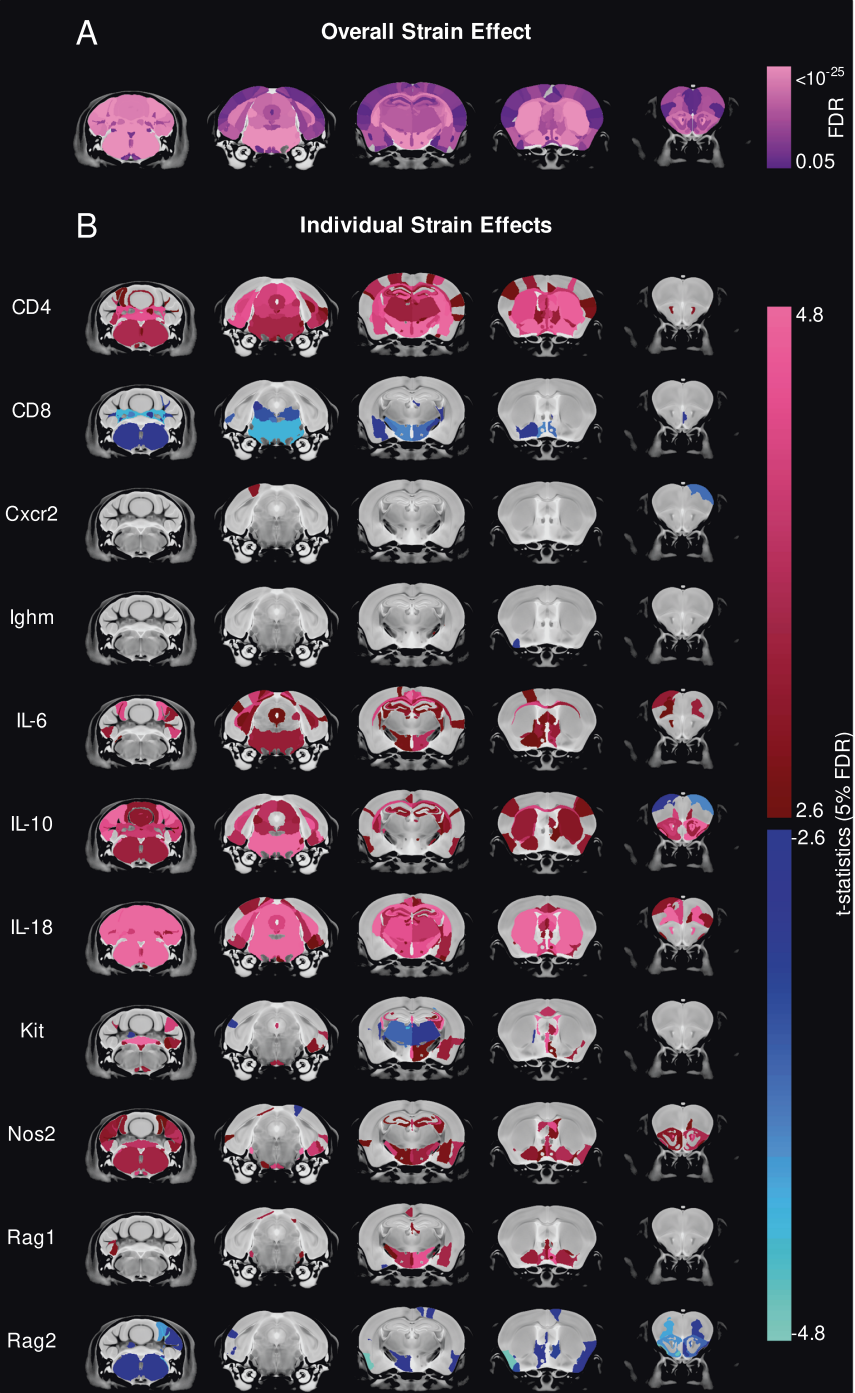
Immune system mutations have a highly heterogeneous effect on mouse brain anatomy. **A** Nearly all brain structures showed a significant effect of strain evaluated using F-statistics from ANOVA. **B** The directional effect in females of the various mutant strains relative to the wild-type strains is visualised using *t*-statistics and shows a heterogeneous neuroanatomical phenotype. Regions larger or smaller in mutants relative to wild-type are given maroon-pink and blue-turquoise colours, respectively, if effects are <5% FDR. Saturated colours represent effects <0.01% FDR.

A significant effect of strain was observed on the volumes of nearly all brain structures (Fig. 1A), and this effect was similar for male and female mice (Supplementary Fig. 10). While the effect of strain was highly significant, the magnitude and direction of these effects showed a great deal of heterogeneity (Fig. 1B for females, Supplementary Fig. 11 for males, Supplementary Table 4 for complete list). For example, mutants lacking cytokines IL-6, IL-10, and IL-18 had a similar phenotype of larger cerebellum compared to wild-type. However, they differed in phenotypes of the left frontal association cortex with IL-10 being smaller than wild-type, and IL-6 and IL-18 being larger.

**Fig. 2:**
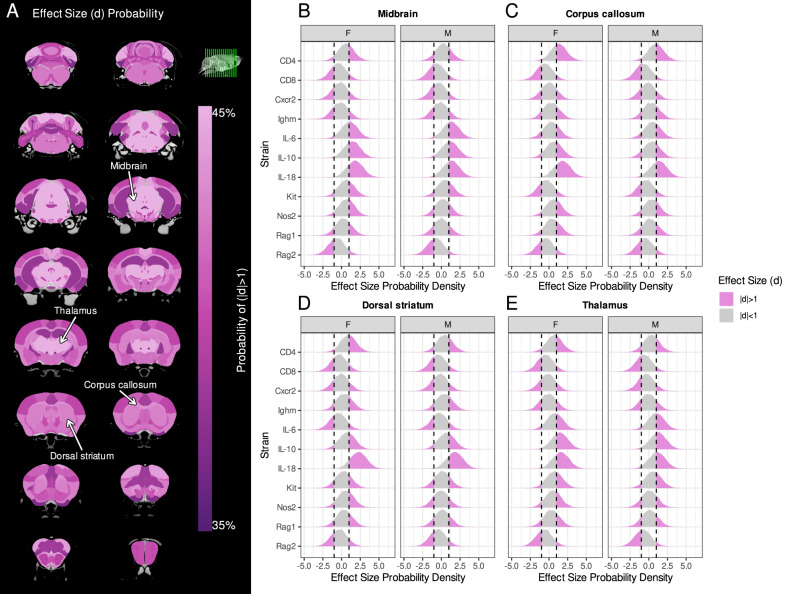
Brain regions showed variations in susceptibility to volume changes due to immune system mutations. **A** The probability of a brain region having a large effect size (*d*) magnitude in mutant strains. Probability of effect-sizes (*x*-axis) for various structures—**B** midbrain, **C** corpus callosum, **D** dorsal striatum, **E** thalamus—across the different mutant strains (*y*-axis), for both female (F) and male (M) mice. Vertical dashed lines represent effect sizes of ±1, and probability within this interval is shaded grey. Integrating the area of the probability density outside this interval (pink) provides the probability that immune system mutations result in a large effect-size magnitude. These four structures had the highest probability of large effect size magnitudes.

To find important patterns through these heterogeneous effects, a BHM was fitted to the neuroanatomy data. Consistent with the frequentist analysis (Fig. 1A), all brain structures had a high probability of having moderate effect-size magnitudes (95% credible interval exceeded 0.50 for all structures). However, certain structures were particularly sensitive to strain and had a high chance of having large effect sizes (Fig. 2A). For example, the midbrain (Fig. 2B), corpus callosum (Fig. 2C), dorsal striatum (Fig. 2D), and thalamus (Fig. 2E) all had among the highest probabilities of having large effect-sizes across the 11 strains evaluated (more examples in Supplementary Fig. 12).

### Neuroanatomical differences among strains cluster by anxiety phenotype

**Fig. 3:**
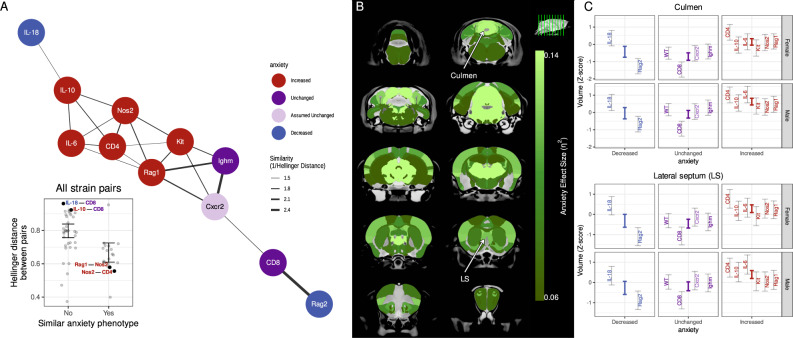
Mutant strains with similar anxiety-behavioural phenotypes have similar neuroanatomy endophenotypes. For each pair of strains, the dissimilarity of endophenotypes was assessed using Hellinger distance and visualised using a network (thicker edges imply greater similarity). **A** Strains with increased anxiety behaviours (red nodes) had similar endophenotypes and clustered together in the network. A similar pattern was seen for unchanged anxiety behaviours (dark purple), but not decreased anxiety behaviour (blue). Cxcr2 anxiety phenotype is not known and assumed unchanged (light purple). The inset plot shows that pairs of strains (represented as dots) with similar anxiety phenotype had similar neuroanatomy endophenotypes (*p* < 0.01 from permutation testing). **B** The effect size (*η*^2^) for the anxiety grouping was computed for each structure. The green colour bar represents at least medium effect sizes and saturates for large effect sizes. **C** The culmen and lateral septum were chosen as representative examples to illustrate large effect-size for anxiety grouping. The 95% credible interval of predicted volume for each strain (grey bars) and anxiety phenotype (coloured bars) are shown.

Given that immune strains had heterogeneous neuroanatomical phenotypes, we investigated whether clustering these phenotypes could reveal important underlying biology. Because prior literature has shown that immune dysregulation causes heterogeneous anxiety-like behaviours (Supplementary Table 3), we assessed whether there was a relationship between a strain’s neuroanatomical endophenotype and anxiety behaviour reported in literature. We found that strains with similar anxiety-behavioural phenotypes had similar neuroanatomical endophenotypes (Fig. 3A inset). This association is particularly driven by strains with increased anxiety behaviours, as visualised in a network plot (Fig. 3A). We then assessed which brain structures in particular drive this association with anxiety behaviour. Nearly every structure had at least a moderate effect-size of anxiety (Fig. 3B); the culmen and lateral septum were plotted as representative structures (Fig. 3C; more examples in Supplementary Fig. 13). As the Cxcr2 mutant strain did not have a documented anxiety phenotype, we assumed this strain had no anxiety-like behaviours for this analysis; repeating this analysis excluding this strain resulted in similarly strong grouping by anxiety phenotype.

### Affected neuroanatomy has preferential spatial expression of genes associated with immune-mediated disease

**Fig. 4:**
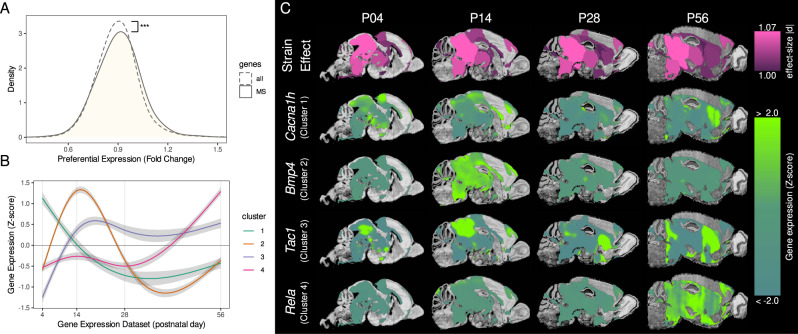
Brain regions susceptible to immune system mutations have a preferential spatial expression of genes involved with multiple sclerosis (MS). The top 25 brain structures with the highest effect-size magnitudes are shown in **C** (first row) and constitute the region-of-interest (ROI). For all genes in the mouse genome, preferential spatial expression was assessed using a fold-change measure (i.e., gene expression signal in ROI relative to the whole brain). **A** Genes associated with MS (solid line) had significantly higher expression in the ROI compared to the genome (two-sided KS test *D* = 0.05, *p* < 10^−3^). **B** MS genes showed four different clusters of temporal expression within the ROI. The shaded region represents the 95% confidence interval. **C** A representative example from each cluster was chosen to visualise gene expression signals within the ROI over the course of neurodevelopment. Each example was closest to its respective cluster’s centroid.

We sought to link the neuroanatomical findings with immune dysfunction by comparison with genome-wide gene expression patterns from the ABI database [41]. For this purpose, an ROI was defined from the MRI results by selecting the set of 25 structures with the highest median effect-size magnitude (shown in Fig. 4C first row). Spatial gene expression analysis [43, 44] was used to identify genes that were preferentially expressed within this ROI using a fold-change measure (average expression in ROI divided by average expression in the brain). Gene ontologies of preferentially expressed genes were enriched in many biological processes associated with white matter: ensheathment of neurons (GO:0007272, *p* < 10^−6^, FDR < 10^−3^), axon ensheathment (GO:0008366, *p* < 10^−6^, FDR < 10^−3^), and myelination (GO:0042552, *p* < 10^−6^, FDR < 10^−2^). Using the DisGeNet [46] and NCBI [45] databases, we then identified diseases in which these preferentially expressed genes have been implicated. Many of the disease terms with significant enrichment have known or suspected immune-mediated pathophysiology, such as Parkinson’s disease (*p* < 10^−8^, FDR < 10^−4^), Alzheimer’s disease (*p* < 10^−8^, FDR < 10^−4^), and multiple sclerosis (MS) (*p* < 10^−4^, FDR = 0.016).

As there is strong evidence that MS is an immune-mediated disease [50], we decided to explore it further. Consistent with the previous enrichment analysis, genes associated with MS tended to have higher expression in brain regions sensitive to immune dysfunction (Fig. 4A). We also clustered the expression patterns of MS-associated genes in the ROI through the course of postnatal development [48] and found four main clusters of genes (Fig. 4B). Two clusters had low expression in adulthood with expression peaks in early life: Cluster 1 had a peak at ~P4 and Cluster 2 peaked later ~P14. The other two clusters had high expression in adulthood: Cluster 3 and 4 had high expression starting from ~P14 and ~P56, respectively. All gene expression analysis results are provided in Supplementary Tables 5–8.

## Discussion

Using neuroanatomical imaging with ex vivo MRI, we confirm heterogeneous and widespread effects of the immune system on the brain using a selection of immune-related genetically engineered mouse strains. To identify patterns underlying this heterogeneity, we studied neuroanatomical phenotypes on three scales: analysing each strain individually, analysing groups of strains unified by a common anxiety-like behavioural phenotype, and aggregating across all strains. When analysing strains individually, we found results consistent with other studies. For example, we did not find altered morphology of the dorsal hippocampus in Rag1 mutants, which is consistent with histology studies [51]. We found thalamic changes in mast cell-deficient Kit mutants, consistent with mast cell migration into the thalamus during development [52]. Phenotypes also showed some degree of lateralisation that has previously been seen in autism models [53, 54]. MRI-detectable volume changes are poorly specific to evaluation of biological determinants: depending on the context, studies have implicated glutamate concentration [55], neurogenesis [56], cellular composition [57], and axonal/dendritic processes [58, 59]. By identifying phenotypes in each strain at mesoscale resolution, we hope targeted investigations can be performed in future studies to elucidate cellular mechanisms.

Analysis of the heterogeneous structural findings for association with behavioural phenotypes revealed important insights and suggests that neuroanatomical endophenotypes cluster significantly with anxiety-like behavioural phenotypes. Previous use of mouse models has revealed several neural circuits associated with producing anxiety behaviours [60, 61], but it is not known which of these neural circuits are responsible for driving anxiety behaviours in models of immune dysfunction. We found that alterations in the lateral septum and hypothalamus cluster with anxiety; and activation of this circuit can promote persistent anxiety [62]. We also found that the cerebellum and midbrain were correlated with anxiety. Studies have shown the important role of these structures in fear- and anxiety-related brain networks [63, 64]. It is unclear whether volume changes are associated with increased or decreased activation of a neural circuit [65]; however, these structures are promising candidates for future studies investigating anxiety behaviours in immune dysfunction. Interestingly, anxiety disorders are a notable comorbid condition in neurological disorders associated with immune dysfunction. For example, anxiety disorders are three times more prevalent in MS patients than in the general population [66]. The prevalence of anxiety disorders in Parkinson’s is 31% [67].

Immune dysfunction plays an important role in many neurological disorders, but the impact of the immune system is difficult to study because the aetiology is highly polygenic. For example, MS is associated with over 1000 genetic risk variants [46], but many of which are associated with immune function [68]. Although requiring further validation, our data provide a potential new avenue for examining the basis of these interactions. By studying the neuroanatomy of several mouse models, our methodology may be useful in finding patterns underlying polygenic immune dysfunction. The corpus callosum, thalamus, striatum, and midbrain were amongst the most-affected structures across all mouse models, which reflects neurological findings in autoimmune disorders [69–72]. Furthermore, several genes associated with myelination are expressed in these regions, which is consistent with molecular studies conducted on MIA model primates [73]. Preferentially expressed genes were also homologous to human genes associated with MS. This finding suggests that expression patterns of MS genes could be followed through neurodevelopment to identify when their expression peaks, pointing to possible developmental time-windows for further study. Our data support the hypothesis that, while there are many genetic variants associated with immune system dysregulation, certain brain regions may be more sensitive to alterations in the immune system. Thus, further study of mouse models with targeted immune system mutations could uncover potential insights regarding neurological symptoms in polygenic immune-mediated disorders.

There are limitations in our work that are important to discuss. Our study focuses solely on static loss-of-function mutations in isolated immune system components, which is a simplification used throughout the existing literature [12–16]. However, it is important to consider that triggering immune system dysfunction in different windows of neurodevelopment can have differing effects [11]. Thus, our work cannot identify when in the maturation process anatomical phenotypes emerge. To capture these dynamic effects, our ex vivo imaging study design could be extended to have a longitudinal in vivo imaging component. Although in vivo MRI generally has inferior performance compared to ex vivo MRI studies of the same animals [74], the additional information could help identify important periods in neurodevelopment where certain immune system components have the greatest effects. Our analysis of behaviour was limited to anxiety because it was best characterised by currently available literature. Social behaviour has also been shown to be reduced by MIA [75] that may suggest social avoidance as a behaviour to reduce susceptibility to pathogen infection. Initiatives like the International Mouse Phenotyping Consortium could be tremendously useful as they would have consistent behavioural assays for every single-gene mutation in the mouse [26]. This would allow future studies to not only investigate the association between immune mutations and anxiety, but social behaviours as well.

In summary, we imaged the neuroanatomy of 11 different mouse mutants, each deficient in particular components of the immune system. By using a consistent high-throughput protocol for data acquisition and analysis, we characterised the diverse effects of immune dysregulation on the brain and identified patterns underlying this heterogeneity. The observed neuroanatomical differences in the mouse models of immune dysfunction are clustered by their anxiety phenotype, recapitulating known brain networks implicated in modulating anxious behaviours. Exploratory analysis of gene expression shows that the brain regions that are most affected by immune dysfunction have a preferential spatial expression of genes associated with MS and other immune-mediated diseases.

## Supplementary information

Supplementary Figures

Supplementary Tables

## Data Availability

Analysis software is freely available online (https://github.com/Mouse-Imaging-Centre).

## References

[1] Estes ML, McAllister AK (2016). Maternal immune activation: implications for neuropsychiatric disorders. Science.

[2] Knuesel I, Chicha L, Britschgi M, Schobel SA, Bodmer M, Hellings JA (2014). Maternal immune activation and abnormal brain development across CNS disorders. Nat Rev Neurol.

[3] Graves JS, Chitnis T, Weinstock-Guttman B, Rubin J, Zelikovitch AS, Nourbakhsh B, et al. Maternal and perinatal exposures are associated with risk for pediatric-onset multiple sclerosis. Pediatrics. 2017;139:e20162838. 10.1542/peds.2016-2838. 10.1542/peds.2016-2838 PMC5369674 28562303

[4] Atladóttir HO, Thorsen P, Østergaard L, Schendel DE, Lemcke S, Abdallah M (2010). Maternal infection requiring hospitalization during pregnancy and autism spectrum disorders. J Autism Dev Disord.

[5] Brown AS (2012). Epidemiologic studies of exposure to prenatal infection and risk of schizophrenia and autism. Dev Neurobiol.

[6] Graham AM, Rasmussen JM, Rudolph MD, Heim CM, Gilmore JH, Styner M (2018). Maternal systemic interleukin-6 during pregnancy is associated with newborn amygdala phenotypes and subsequent behavior at 2 years of age. Biol Psychiatry.

[7] Rudolph MD, Graham AM, Feczko E, Miranda-Dominguez O, Rasmussen JM, Nardos R (2018). Maternal IL-6 during pregnancy can be estimated from newborn brain connectivity and predicts future working memory in offspring. Nat Neurosci.

[8] Rasmussen JM, Graham AM, Entringer S, Gilmore JH, Styner M, Fair DA (2019). Maternal Interleukin-6 concentration during pregnancy is associated with variation in frontolimbic white matter and cognitive development in early life. Neuroimage.

[9] Piontkewitz Y, Arad M, Weiner I (2011). Abnormal trajectories of neurodevelopment and behavior following in utero insult in the rat. Biol Psychiatry.

[10] Crum WR, Sawiak SJ, Chege W, Cooper JD, Williams SCR, Vernon AC (2017). Evolution of structural abnormalities in the rat brain following in utero exposure to maternal immune activation: a longitudinal in vivo MRI study. Brain Behav Immun.

[11] Guma E, Bordignon P, do C, Devenyi GA, Gallino D, Anastassiadis C (2021). Early or late gestational exposure to maternal immune activation alters neurodevelopmental trajectories in mice: an integrated neuroimaging, behavioral, and transcriptional study. Biol Psychiatry.

[12] Chourbaji S, Urani A, Inta I, Sanchis-Segura C, Brandwein C, Zink M (2006). IL-6 knockout mice exhibit resistance to stress-induced development of depression-like behaviors. Neurobiol Dis.

[13] Mesquita AR, Correia-Neves M, Roque S, Castro AG, Vieira P, Pedrosa J (2008). IL-10 modulates depressive-like behavior. J Psychiatr Res.

[14] Yaguchi T, Nagata T, Yang D, Nishizaki T (2010). Interleukin-18 regulates motor activity, anxiety and spatial learning without affecting synaptic plasticity. Behav Brain Res.

[15] Rilett KC, Friedel M, Ellegood J, MacKenzie RN, Lerch JP, Foster JA (2015). Loss of T cells influences sex differences in behavior and brain structure. Brain Behav Immun.

[16] Clark SM, Soroka JA, Song C, Li X, Tonelli LH (2016). CD4(+) T cells confer anxiolytic and antidepressant-like effects, but enhance fear memory processes in Rag2(-/-) mice. Stress.

[17] Sankar A, MacKenzie RN, Foster JA (2012). Loss of class I MHC function alters behavior and stress reactivity. J Neuroimmunol.

[18] Clark SM, Sand J, Francis TC, Nagaraju A, Michael KC, Keegan AD (2014). Immune status influences fear and anxiety responses in mice after acute stress exposure. Brain Behav Immun.

[19] Buskila Y, Abu-Ghanem Y, Levi Y, Moran A, Grauer E, Amitai Y (2007). Enhanced astrocytic nitric oxide production and neuronal modifications in the neocortex of a NOS2 mutant mouse. PLoS One.

[20] Nautiyal KM, Ribeiro AC, Pfaff DW, Silver R (2008). Brain mast cells link the immune system to anxiety-like behavior. Proc Natl Acad Sci USA.

[21] Ellegood J, Anagnostou E, Babineau BA, Crawley JN, Lin L, Genestine M (2015). Clustering autism: using neuroanatomical differences in 26 mouse models to gain insight into the heterogeneity. Mol Psychiatry.

[22] Anacker C, Scholz J, O’Donnell KJ, Allemang-Grand R, Diorio J, Bagot RC (2016). Neuroanatomic differences associated with stress susceptibility and resilience. Biol Psychiatry.

[23] Lerch JP, Yiu AP, Martinez-Canabal A, Pekar T, Bohbot VD, Frankland PW (2011). Maze training in mice induces MRI-detectable brain shape changes specific to the type of learning. Neuroimage.

[24] Cacalano G, Lee J, Kikly K, Ryan AM, Pitts-Meek S, Hultgren B (1994). Neutrophil and B cell expansion in mice that lack the murine IL-8 receptor homolog. Science.

[25] Holmdahl R, Malissen B (2012). The need for littermate controls. Eur J Immunol.

[26] de Angelis MH, Nicholson G, Selloum M, White J, Morgan H, Ramirez-Solis R (2015). Analysis of mammalian gene function through broad-based phenotypic screens across a consortium of mouse clinics. Nat Genet.

[27] Cahill LS, Laliberté CL, Ellegood J, Spring S, Gleave JA, van Eede MC (2012). Preparation of fixed mouse brains for MRI. Neuroimage.

[28] Guzman AE, de, de Guzman AE, Wong MD, Gleave JA, Nieman BJ (2016). Variations in post-perfusion immersion fixation and storage alter MRI measurements of mouse brain morphometry. NeuroImage.

[29] Dazai J, Spring S, Cahill LS, Henkelman RM. Multiple-mouse neuroanatomical magnetic resonance imaging. J Vis Exp. 2011:2497. 10.3791/2497. 10.3791/2497 PMC3339839 21829155

[30] Spencer Noakes TL, Henkelman RM, Nieman BJ. Partitioning k-space for cylindrical three-dimensional rapid acquisition with relaxation enhancement imaging in the mouse brain. NMR Biomed. 2017;30. 10.1002/nbm.3802. 10.1002/nbm.3802 28902423

[31] Collins DL, Neelin P, Peters TM, Evans AC (1994). Automatic 3D intersubject registration of MR volumetric data in standardized Talairach space. J Comput Assist Tomogr.

[32] Avants BB, Tustison NJ, Song G, Cook PA, Klein A, Gee JC (2011). A reproducible evaluation of ANTs similarity metric performance in brain image registration. Neuroimage.

[33] Friedel M, van Eede MC, Pipitone J, Chakravarty MM, Lerch JP (2014). Pydpiper: a flexible toolkit for constructing novel registration pipelines. Front Neuroinform.

[34] Chakravarty MM, Steadman P, van Eede MC, Calcott RD, Gu V, Shaw P (2013). Performing label-fusion-based segmentation using multiple automatically generated templates. Hum Brain Mapp.

[35] Dorr AE, Lerch JP, Spring S, Kabani N, Henkelman RM (2008). High resolution three-dimensional brain atlas using an average magnetic resonance image of 40 adult C57Bl/6J mice. Neuroimage.

[36] Steadman PE, Ellegood J, Szulc KU, Turnbull DH, Joyner AL, Henkelman RM (2014). Genetic effects on cerebellar structure across mouse models of autism using a magnetic resonance imaging atlas. Autism Res.

[37] Ullmann JFP, Watson C, Janke AL, Kurniawan ND, Reutens DC (2013). A segmentation protocol and MRI atlas of the C57BL/6J mouse neocortex. Neuroimage.

[38] Richards K, Watson C, Buckley RF, Kurniawan ND, Yang Z, Keller MD (2011). Segmentation of the mouse hippocampal formation in magnetic resonance images. Neuroimage.

[39] Qiu LR, Fernandes DJ, Szulc-Lerch KU, Dazai J, Nieman BJ, Turnbull DH (2018). Mouse MRI shows brain areas relatively larger in males emerge before those larger in females. Nat Commun.

[40] Genovese CR, Lazar NA, Nichols T (2002). Thresholding of statistical maps in functional neuroimaging using the false discovery rate. Neuroimage.

[41] Lein ES, Hawrylycz MJ, Ao N, Ayres M, Bensinger A, Bernard A (2007). Genome-wide atlas of gene expression in the adult mouse brain. Nature.

[42] Korner-Nievergelt F, Roth T, von Felten S, Guélat J, Almasi B, Korner-Nievergelt P. Bayesian data analysis in ecology using linear models with R, BUGS, and Stan. Academic Press; Cambridge, 2015.

[43] Fernandes DJ, Ellegood J, Askalan R, Blakely RD, Dicicco-Bloom E, Egan SE (2017). Spatial gene expression analysis of neuroanatomical differences in mouse models. Neuroimage.

[44] Fernandes DJ, Spring S, Roy AR, Qiu LR, Yee Y, Nieman BJ (2021). Exposure to maternal high-fat diet induces extensive changes in the brain of adult offspring. Transl Psychiatry.

[45] Wheeler DL (2004). Database resources of the National Center for Biotechnology Information. Nucleic Acids Res.

[46] Piñero J, Queralt-Rosinach N, Bravo À, Deu-Pons J, Bauer-Mehren A, Baron M (2015). DisGeNET: a discovery platform for the dynamical exploration of human diseases and their genes. Database.

[47] Eden E, Navon R, Steinfeld I, Lipson D, Yakhini Z (2009). GOrilla: a tool for discovery and visualization of enriched GO terms in ranked gene lists. BMC Bioinforma.

[48] Thompson CL, Ng L, Menon V, Martinez S, Lee C-K, Glattfelder K (2014). A high-resolution spatiotemporal atlas of gene expression of the developing mouse brain. Neuron.

[49] Thorndike RL (1953). Who belongs in the family?. Psychometrika.

[50] Wootla B, Eriguchi M, Rodriguez M (2012). Is multiple sclerosis an autoimmune disease?. Autoimmune Dis.

[51] Rattazzi L, Piras G, Ono M, Deacon R, Pariante CM, D’Acquisto F (2013). CD4^+^ but not CD8^+^ T cells revert the impaired emotional behavior of immunocompromised RAG-1-deficient mice. Transl Psychiatry.

[52] Wasielewska JM, Grönnert L, Rund N, Donix L, Rust R, Sykes AM (2017). Mast cells increase adult neural precursor proliferation and differentiation but this potential is not realized in vivo under physiological conditions. Sci Rep..

[53] Wang X, Bey AL, Katz BM, Badea A, Kim N, David LK, et al. Altered mGluR5-Homer scaffolds and corticostriatal connectivity in a Shank3 complete knockout model of autism. Nat Commun. 2016;7:11459. 10.1038/ncomms11459. 10.1038/ncomms11459 PMC4866051 27161151

[54] Wang S, Tan N, Zhu X, Yao M, Wang Y, Zhang X (2018). Sh3rf2 haploinsufficiency leads to unilateral neuronal development deficits and autistic-like behaviors in mice. Cell Rep..

[55] Biedermann S, Fuss J, Zheng L, Sartorius A, Falfán-Melgoza C, Demirakca T (2012). In vivo voxel based morphometry: detection of increased hippocampal volume and decreased glutamate levels in exercising mice. Neuroimage.

[56] Fuss J, Biedermann SV, Falfán-Melgoza C, Auer MK, Zheng L, Steinle J (2014). Exercise boosts hippocampal volume by preventing early age-related gray matter loss. Hippocampus.

[57] Asan L, Falfán-Melgoza C, Beretta CA, Sack M, Zheng L, Weber-Fahr W (2021). Cellular correlates of gray matter volume changes in magnetic resonance morphometry identified by two-photon microscopy. Sci Rep..

[58] Golub Y, Kaltwasser SF, Mauch CP, Herrmann L, Schmidt U, Holsboer F (2011). Reduced hippocampus volume in the mouse model of Posttraumatic Stress Disorder. J Psychiatr Res.

[59] Keifer OP, Hurt RC, Gutman DA, Keilholz SD, Gourley SL, Ressler KJ (2015). Voxel-based morphometry predicts shifts in dendritic spine density and morphology with auditory fear conditioning. Nat Commun.

[60] Calhoon GG, Tye KM (2015). Resolving the neural circuits of anxiety. Nat Neurosci.

[61] Apps R, Strata P (2015). Neuronal circuits for fear and anxiety – the missing link. Nat Rev Neurosci..

[62] Anthony TE, Dee N, Bernard A, Lerchner W, Heintz N, Anderson DJ (2014). Control of stress-induced persistent anxiety by an extra-amygdala septohypothalamic circuit. Cell.

[63] Moreno-Rius J (2018). The cerebellum in fear and anxiety-related disorders. Prog Neuropsychopharmacol Biol Psychiatry.

[64] Koutsikou S, Crook JJ, Earl EV, Leith JL, Watson TC, Lumb BM (2014). Neural substrates underlying fear-evoked freezing: the periaqueductal grey-cerebellar link. J Physiol.

[65] Jung H, Park H, Choi Y, Kang H, Lee E, Kweon H (2018). Sexually dimorphic behavior, neuronal activity, and gene expression in Chd8-mutant mice. Nat Neurosci.

[66] Silveira C, Guedes R, Maia D, Curral R, Coelho R (2019). Neuropsychiatric symptoms of multiple sclerosis: state of the art. Psychiatry Investig.

[67] Broen MPG, Narayen NE, Kuijf ML, Dissanayaka NNW, Leentjens AFG (2016). Prevalence of anxiety in Parkinson’s disease: a systematic review and meta-analysis. Mov Disord.

[68] Didonna A, Oksenberg JR. The genetics of multiple sclerosis. In: Zagon IS, McLaughlin PJ, editors. Multiple sclerosis: perspectives in treatment and pathogenesis. Codon Publications: Brisbane (AU); 2017.

[69] Mesaros S, Rocca MA, Riccitelli G, Pagani E, Rovaris M, Caputo D (2009). Corpus callosum damage and cognitive dysfunction in benign MS. Hum Brain Mapp.

[70] Harrison DM, Oh J, Roy S, Wood ET, Whetstone A, Seigo MA (2015). Thalamic lesions in multiple sclerosis by 7T MRI: clinical implications and relationship to cortical pathology. Mult Scler.

[71] Bermel RA, Innus MD, Tjoa CW, Bakshi R (2003). Selective caudate atrophy in multiple sclerosis: a 3D MRI parcellation study. Neuroreport.

[72] Quint DJ, Cornblath WT, Trobe JD (1993). Multiple sclerosis presenting as Parinaud syndrome. AJNR Am J Neuroradiol.

[73] Page NF, Gandal MJ, Estes ML, Cameron S, Buth J, Parhami S (2021). Alterations in retrotransposition, synaptic connectivity, and myelination implicated by transcriptomic changes following maternal immune activation in nonhuman primates. Biol Psychiatry.

[74] Ma D, Holmes HE, Cardoso MJ, Modat M, Harrison IF, Powell NM (2019). Study the longitudinal and cross-sectional brain volume difference for disease progression and treatment effect on mouse model of tauopathy using automated MRI structural parcellation. Front Neurosci.

[75] Mueller FS, Scarborough J, Schalbetter SM, Richetto J, Kim E, Couch A (2021). Behavioral, neuroanatomical, and molecular correlates of resilience and susceptibility to maternal immune activation. Mol Psychiatry.

[76] Rahemtulla A, Fung-Leung WP, Schilham MW, Kündig TM, Sambhara SR, Narendran A (1991). Normal development and function of CD8+ cells but markedly decreased helper cell activity in mice lacking CD4. Nature.

[77] Fung-Leung W-P, Schilham MW, Rahemtulla A, Kündig TM, Vollenweider M, Potter J (1991). CD8 is needed for development of cytotoxic T but not helper T cells. Cell..

[78] Keane MP, Belperio JA, Xue YY, Burdick MD, Strieter RM (2004). Depletion of CXCR2 inhibits tumor growth and angiogenesis in a murine model of lung cancer. J Immunol.

[79] Kitamura D, Roes J, Kühn R, Rajewsky KA (1991). B cell-deficient mouse by targeted disruption of the membrane exon of the immunoglobulin μ chain gene. Nature..

[80] Kopf M, Baumann H, Freer G, Freudenberg M, Lamers M, Kishimoto T (1994). Impaired immune and acute-phase responses in interleukin-6-deficient mice. Nature.

[81] Kühn R, Löhler J, Rennick D, Rajewsky K, Müller W (1993). Interleukin-10-deficient mice develop chronic enterocolitis. Cell.

[82] Berg DJ, Davidson N, Kühn R, Müller W, Menon S, Holland G (1996). Enterocolitis and colon cancer in interleukin-10-deficient mice are associated with aberrant cytokine production and CD4() TH1-like responses. J Clin Investig.

[83] Takeda K, Tsutsui H, Yoshimoto T, Adachi O, Yoshida N, Kishimoto T (1998). Defective NK cell activity and Th1 response in IL-18-deficient mice. Immunity.

[84] Lyon MF, Glenister PH (1982). A new allele sash (Wsh) at the W-locus and a spontaneous recessive lethal in mice. Genet Res.

[85] Grimbaldeston MA, Chen C-C, Piliponsky AM, Tsai M, Tam S-Y, Galli SJ (2005). Mast cell-deficient W-sash c-kit mutant Kit W-sh/W-sh mice as a model for investigating mast cell biology in vivo. Am J Pathol.

[86] Piliponsky AM, Chen C-C, Grimbaldeston MA, Burns-Guydish SM, Hardy J, Kalesnikoff J (2010). Mast cell-derived TNF can exacerbate mortality during severe bacterial infections in C57BL/6-KitW-sh/W-sh mice. Am J Pathol.

[87] Laubach VE, Shesely EG, Smithies O, Sherman PA (1995). Mice lacking inducible nitric oxide synthase are not resistant to lipopolysaccharide-induced death. Proc Natl Acad Sci USA.

[88] Mombaerts P, Iacomini J, Johnson RS, Herrup K, Tonegawa S, Papaioannou VE (1992). RAG-1-deficient mice have no mature B and T lymphocytes. Cell.

[89] Shinkai Y (1992). RAG-2-deficient mice lack mature lymphocytes owing to inability to initiate V(D)J rearrangement. Cell..

[90] Hao Z, Rajewsky K (2001). Homeostasis of peripheral B cells in the absence of B cell influx from the bone marrow. J Exp Med.

