# Supplementary material for: Mouse models of immune dysfunction: their neuroanatomical differences reflect their anxiety-behavioural phenotype
Source: Mol Psychiatry. 2022 Apr 14;27(7):3047–55. doi: 10.1038/s41380-022-01535-5 (PMC9205773; doi:10.1038/s41380-022-01535-5)
Supplement: Supplementary file 1 — Supplementary Figures [file 41380_2022_1535_MOESM1_ESM.pdf]

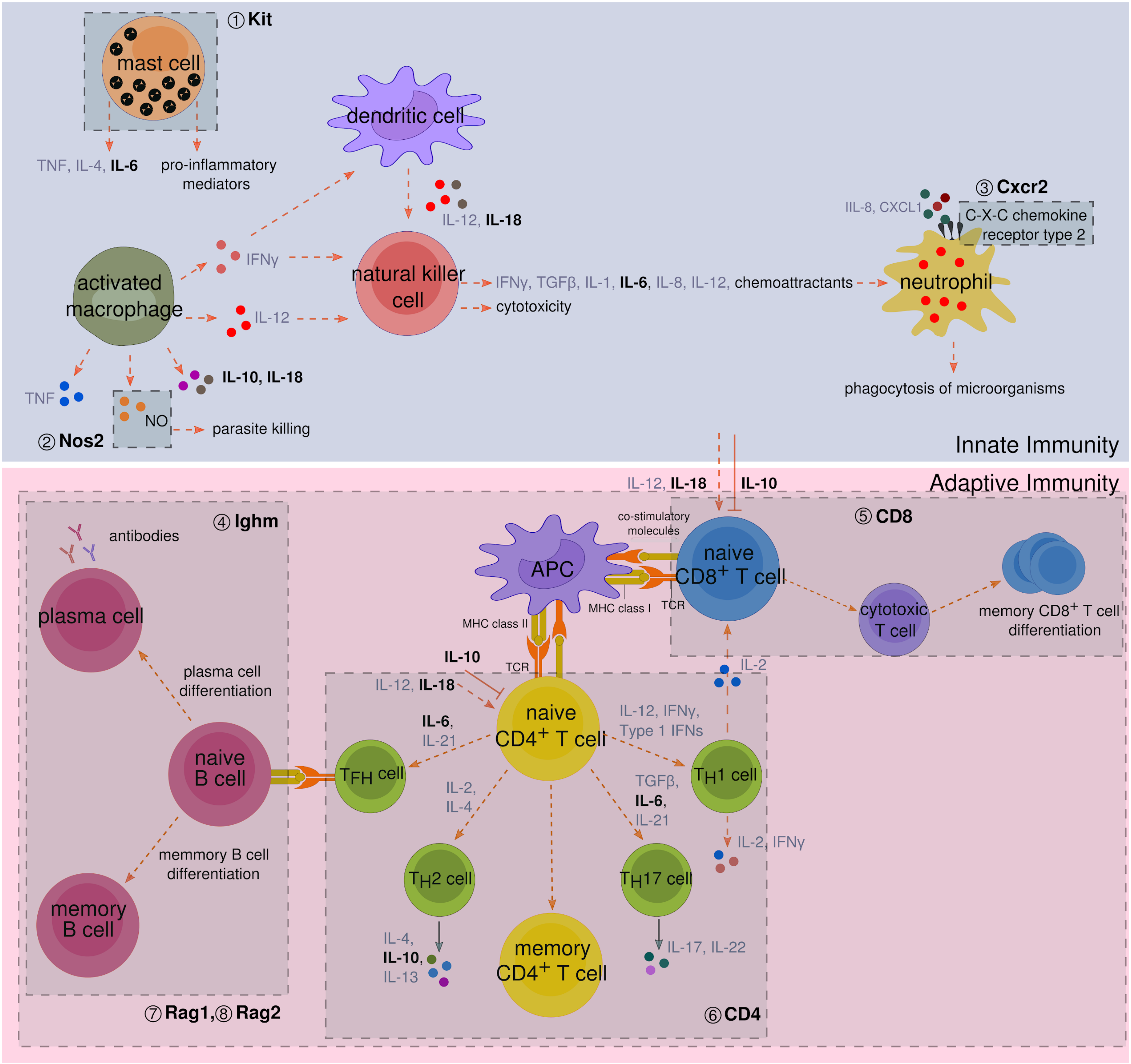

Supplementary Figure 1: Schematic representation depicting the functions of the various immune system components mutated in this study. Kit ① mice are depleted of mast cells soon after birth, affecting inflammatory responses and cytokine release. Nos2 ② mutants lack serum nitric oxide responses involved in parasite killing. Cxcr2 ③ mutants lack the chemokine receptor that binds CXCL2 and IL-8 leading to impaired neutrophil recruitment and decreased pathogen clearance. The Ighm ④ mutant lacks mature B cells impeding humoral immunity. CD8 ⑤ knockouts are deficient in functional cytotoxic T cells while CD4 ⑥ mutants have a significant block in CD4+ T cell development. Both Rag1 ⑦ and Rag2 ⑧ mutants lack mature, functional B and T cells and are therefore deficient in adaptive immune responses. The cytokines studied in this paper -- interleukin (IL)-6, IL-10 and IL-18 -- are shown in bold and act on multiple pathways. IL-6 is both a pro-inflammatory cytokine and anti-inflammatory myokine while IL-10 is an anti-inflammatory cytokine. IL-18 is a pro-inflammatory cytokine playing a key role in autoimmune, inflammatory and infectious diseases. This figure is not meant to be a comprehensive depiction of the immune system and all components. Other acronyms used in the figure: antigen presenting cell (APC), interferon (IFN), tumour necrosis factor (TNF), transforming growth factor (TGF), major histocompatibility complex (MHC), T cell receptor (TCR), T helper cell (T_H), T follicular helper cell (T_{FH}).

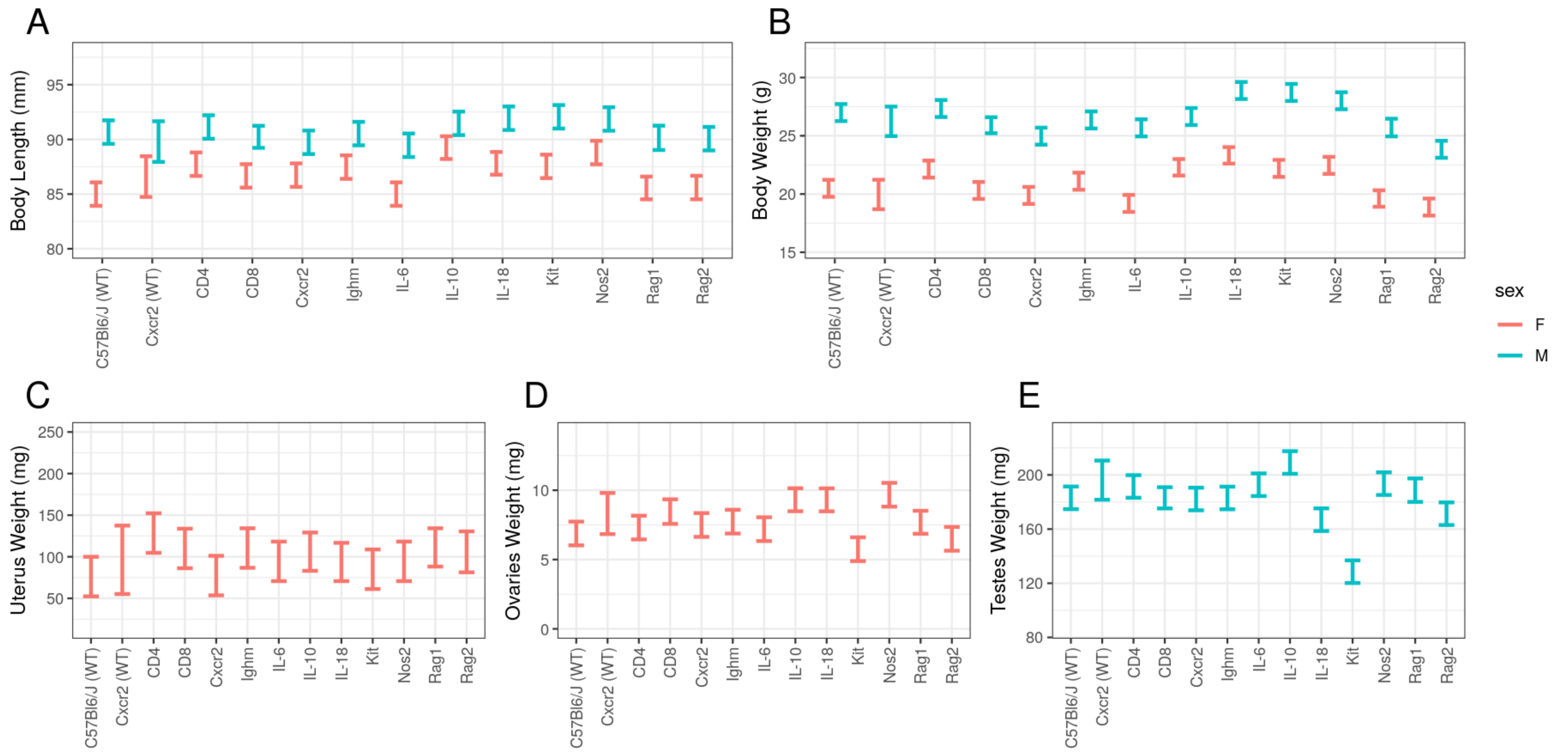

Supplementary Figure 2: Physical characteristics of each strain in study. (A) Body length, (B) body weight, (C) uterus weight, (D) ovaries weight, and (E) testes weight were measured. There was a significant effect of strain on body length ($F_{24,372} = 4.5, p < 10^{-10}$), body weight ($F_{24,372} = 14.9, p < 10^{-40}$), ovaries weight ($F_{12,186} = 7.2, p < 10^{-9}$), and testes weight ($F_{12,185} = 20.4, p < 10^{-27}$), but not uterus weight ($F_{12,186} = 1.5, p = 0.12$). There was no significant sex-strain interactions in the body length ($F_{12,360} = 1.43, p = 0.15$) and body weight ($F_{12,360} = 1.63, p = 0.08$) measures. Error bars represent the 95% confidence intervals.

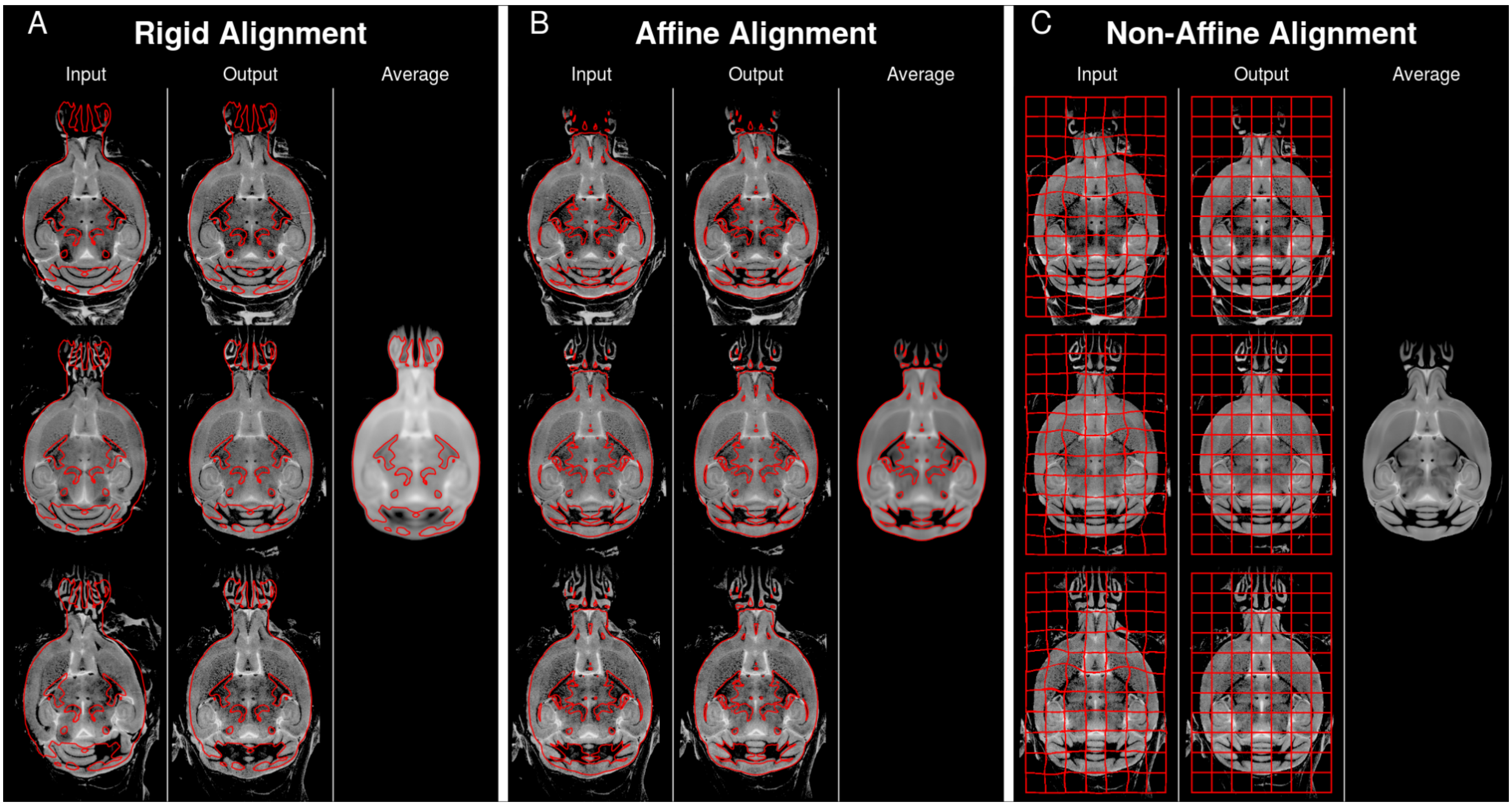

Supplementary Figure 3: Examples of images as they are processed through the registration pipeline.
(A) The first step was rigid alignment, where input images were rotated and translated to align with one another. The output images were averaged to create the rigid registration average. To assess registration quality, iso-intensity contours (marked in red) in the rigid registration average were overlaid on the output and input images.
(B) The second step in the pipeline was the affine alignment. In this step, the input images are scaled and sheared (in conjunction with being translated and rotated) to align with one another. The output images were averaged to create the affine registration average. To assess registration quality, iso-intensity contours (marked in red) in the affine registration average were overlaid on the input and output images.
(C) The third step in the pipeline was non-affine alignment. Input images were locally deformed to achieve optimal alignment. The output images were averaged to create the non-affine registration average, which is also the consensus average in this study. To visualise local deformations, grid-lines in the output images were back-transformed to input images. Iso-intensity contours (shown in Supplementary Figure 4) were used to assess registration quality.

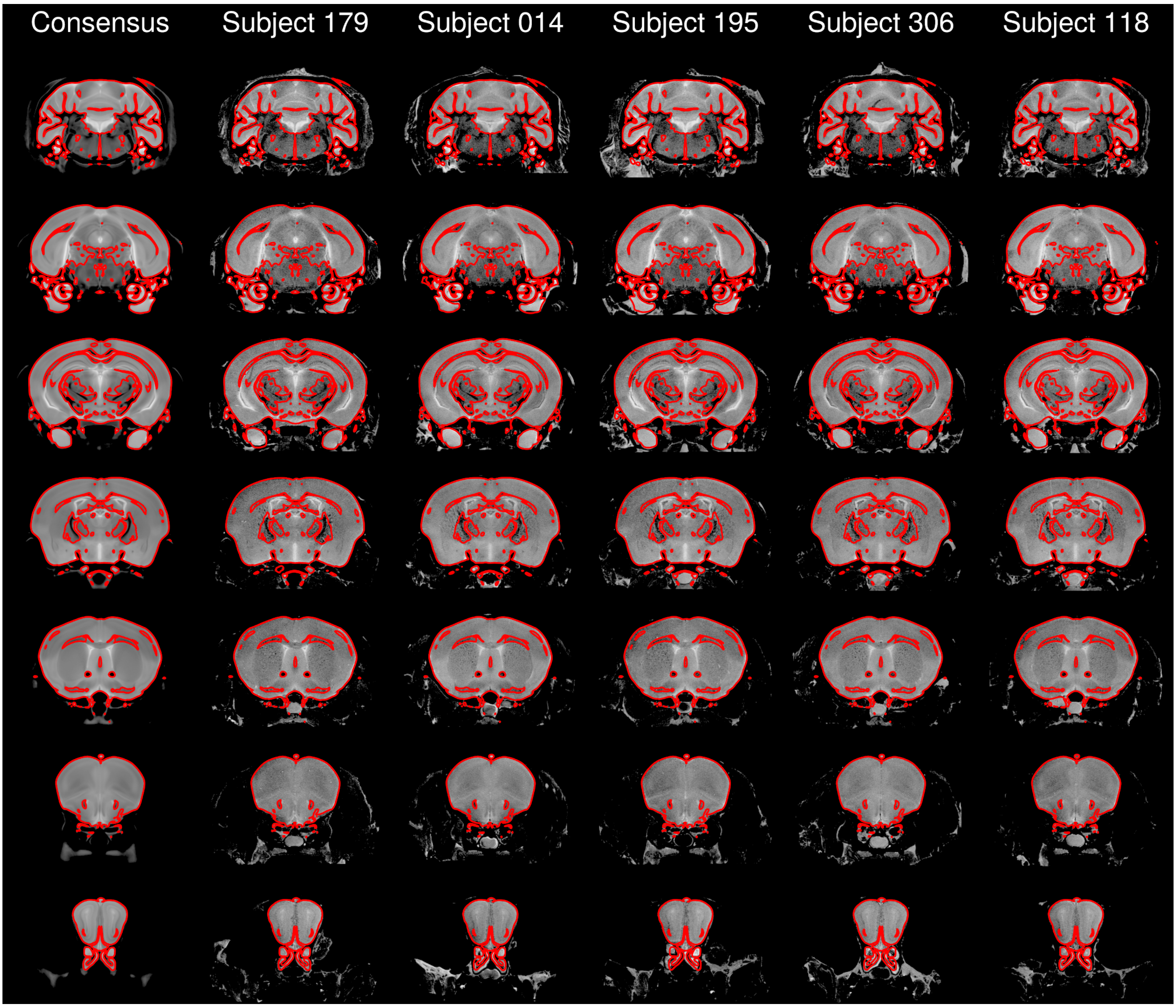

Supplementary Figure 4: Qualitative assessment of registration alignment. Iso-intensity contours in the consensus average (marked in red) were overlaid on all 371 images after they were processed by the registration pipeline. Registration accuracy was estimated by assessing if contours mark important landmarks in the brain; such as the parenchyma-CSF boundary and large white-matter tracts. Coronal slices for 5 random subjects are shown as an example of good registration accuracy. All 371 images had good alignment.

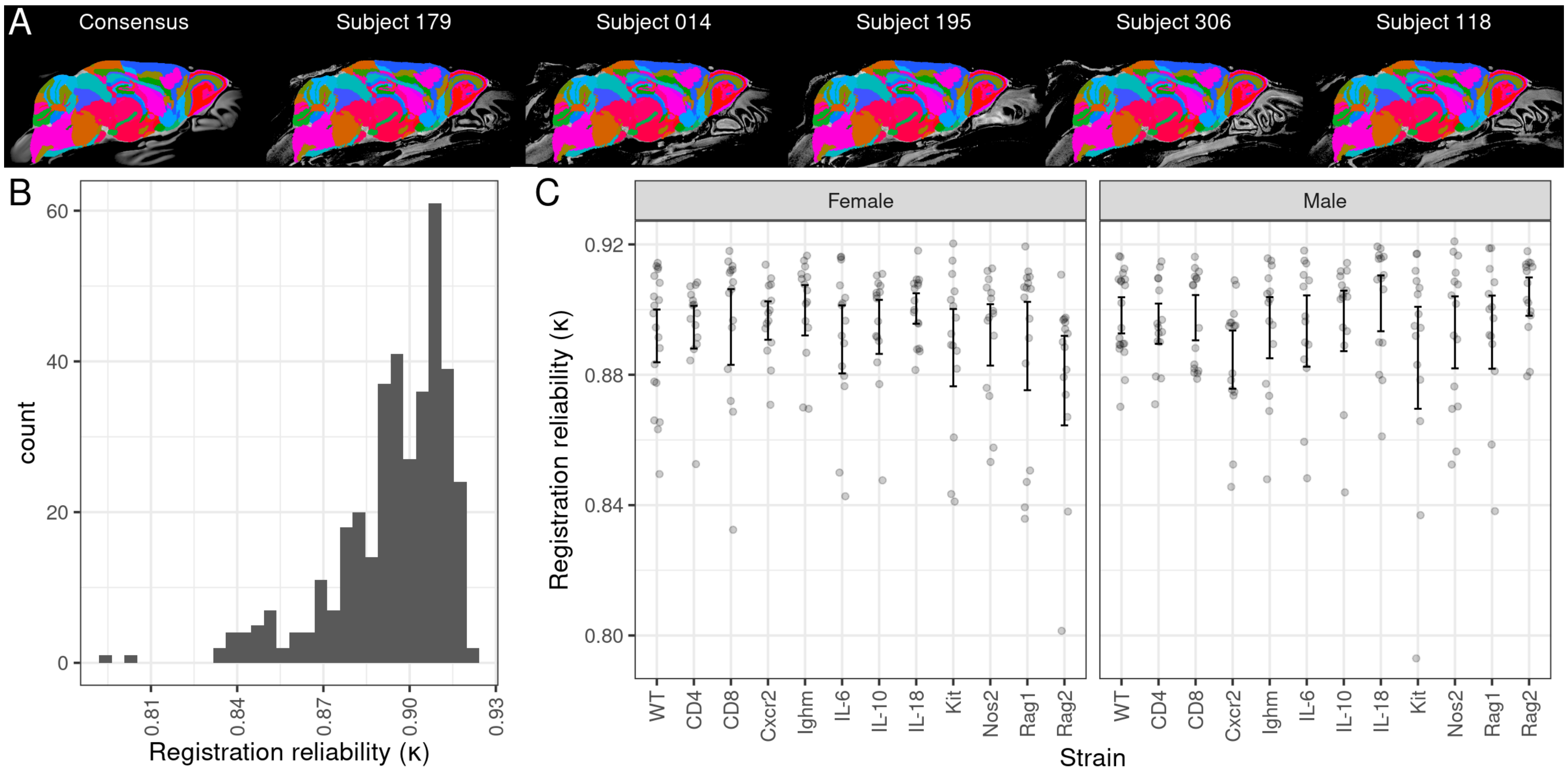

Supplementary Figure 5: Assessment of registration convergence. All strains had similar image intensities indicating similar uptake of MR-contrast agent (largest effect size was 0.17 between WT and IL-18 females). We registered atlas labels to the consensus average, shown in the left-most image subfigure (A). We also registered atlas labels to each subject's image using the MAGeT pipeline. As the subject images were all mapped to the consensus average, the subject labels were then transformed to the same space as the consensus average. 5 random examples of these transformed-subject-labels are shown in sub-figure (A). If all registrations achieved perfect convergence, then transformed subject labels should exactly match the consensus labels and the inter-rater reliability is high. Agreement between the consensus labels and transformed subject labels were measured using the Cohen's κ coefficient, which is routinely used to assess inter-rater reliability (McHugh 2012), using the formula below:

$$\kappa = 1 - \frac{1-p}{1-\sum_{k=1}^{336} q_k r_k}$$

p is the fraction of the brain volume where both registrations agree on label segmentation, k is the index for each of the 336 structures in the atlas. q_k represents the fraction of the brain encompassed by structure k in one registration, and r_k represents the same for the other registration. Values for κ can run between 0 and 1, indicating chance and perfect agreement between raters, respectively. (B) κ was calculated for the segmentations from each subject and all 371 κ values are shown as a histogram. Nearly all kappa scores exceeded 0.8, which represents the threshold for good reliability between registrations and is on par with the expected registration pipeline performance (Chakravarty et al. 2013). (C) κ scores were also not influenced by strain ($F_{22,347}$=1.2,P=0.25), sex ($F_{12,347}$=1.4,P=0.18), or their interaction ($F_{11,347}$=1.4,P=0.16) as assessed using ANOVA. Error bars represent 95% confidence intervals from parametric bootstrap. This indicates that registration convergence was not biased against any particular group.

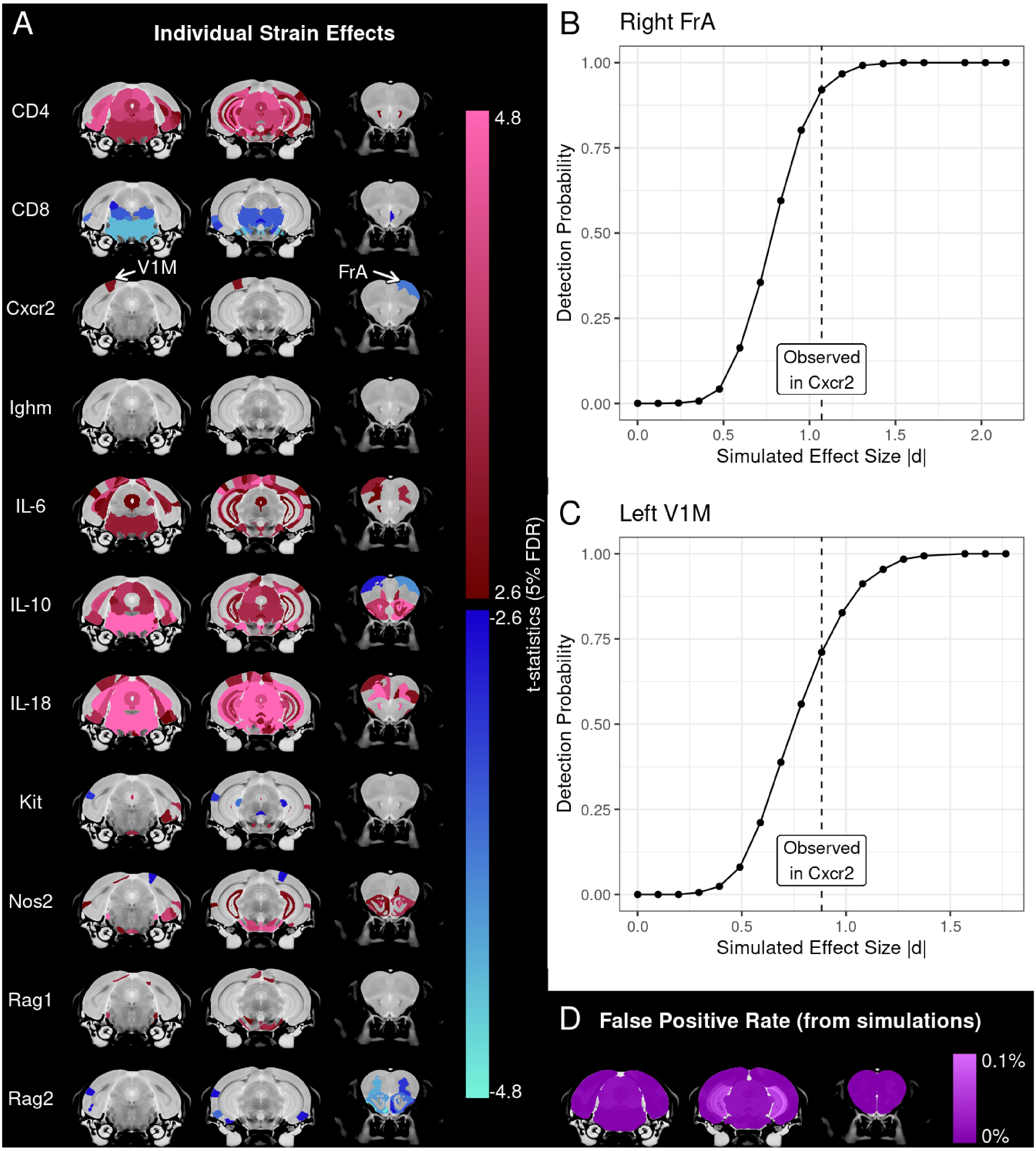

Supplementary Figure 6: *Post hoc* simulations to assess false-positives and detection probability in our study.
(A) The strain effects seen in our study (same as Figure 2 in the main paper) defined the ground truth for the simulation. Our goal was to create simulations to assess the power in detecting significant neuroanatomy, such as the left monocular area of the Primary visual cortex (V1M) and right Frontal association cortex (FrA) in the Cxcr2 strain. To create simulated data that has the same effect-size as the ground truth, we first performed the same frequentist statistics as described in the paper (i.e. fitting each structure with a linear model) and then bootstrapped the residuals. For each of the 1000 simulations, the same frequentist statistics methods were run again on simulated data (including the FDR-correction step). Detection probability (i.e. power) was assessed by calculating in what fraction of the 1000 iterations a significant effect (FDR>0.05) was detected. To assess how the detection probability is affected by the effect-size in the ground truth, additional simulations were run. In these simulations, the beta-coefficients associated with strain (from the linear models fitted to the ground truth data) were increased or decreased in magnitude -- which is associated with greater or lesser differences, respectively, between wild-type and mutant strains. The same method of bootstrapping residuals was used to generate simulated data from these modified beta-coefficients. Detection probability was plotted against the simulated effect-size magnitude (|*d*|) for (B) Left V1M and (C) Right FrA. For both structures, the actual observed effect-sizes are given as a dashed line, and the detection probability at these effect-sizes were 0.92 and 0.71, respectively. Detection probability decreases precipitously for lower effect-sizes and increases only slightly for higher effect-sizes.
To assess false-positive, beta-coefficients associated with strain (from the linear models fitted to the ground truth data) were fixed to be zero and bootstrapping residuals method was used to generate simulated data. For each structure, the fraction of 1000 simulations where significant strain effects were detected (despite beta-coefficients being 0, implying there are no differences between wild-type and mutants) represents the false-positive rate. (C) Low false-positive rates were seen for all structures using our statistical methodologies (highest value was 0.1%).

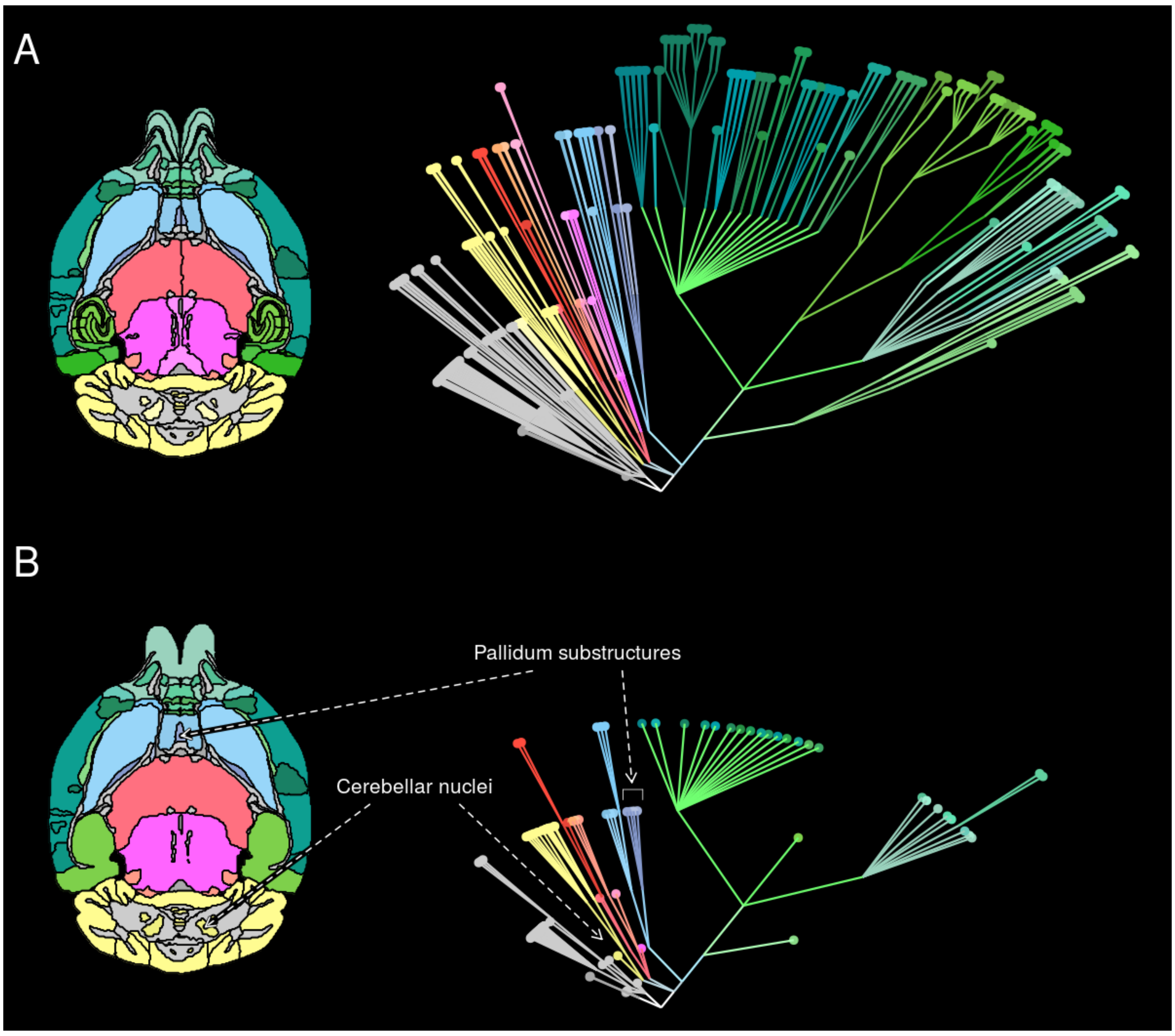

Supplementary Figure 7: Pruning the neuroanatomy hierarchy. The hierarchy can be found in this link: https://atlas.brain-map.org/atlas?atlas=1
(A) All 336 structures in the mouse brain atlas (left) can be organised into a developmental hierarchy (right). The first level of the hierarchy merges bilateral structures. In all subsequent levels, substructures merge together to form larger structures. The root of the tree represents the whole brain.
(B) Pruning the hierarchy reduces the number of total structures by merging substructures that have similar phenotypes across sex and strain. The **next** figure (Supplementary Figure 8) provides an illustration of how neuroanatomy phenotype determines when substructures are merged (ex cerebellar nuclei) or not merged (ex pallidum).

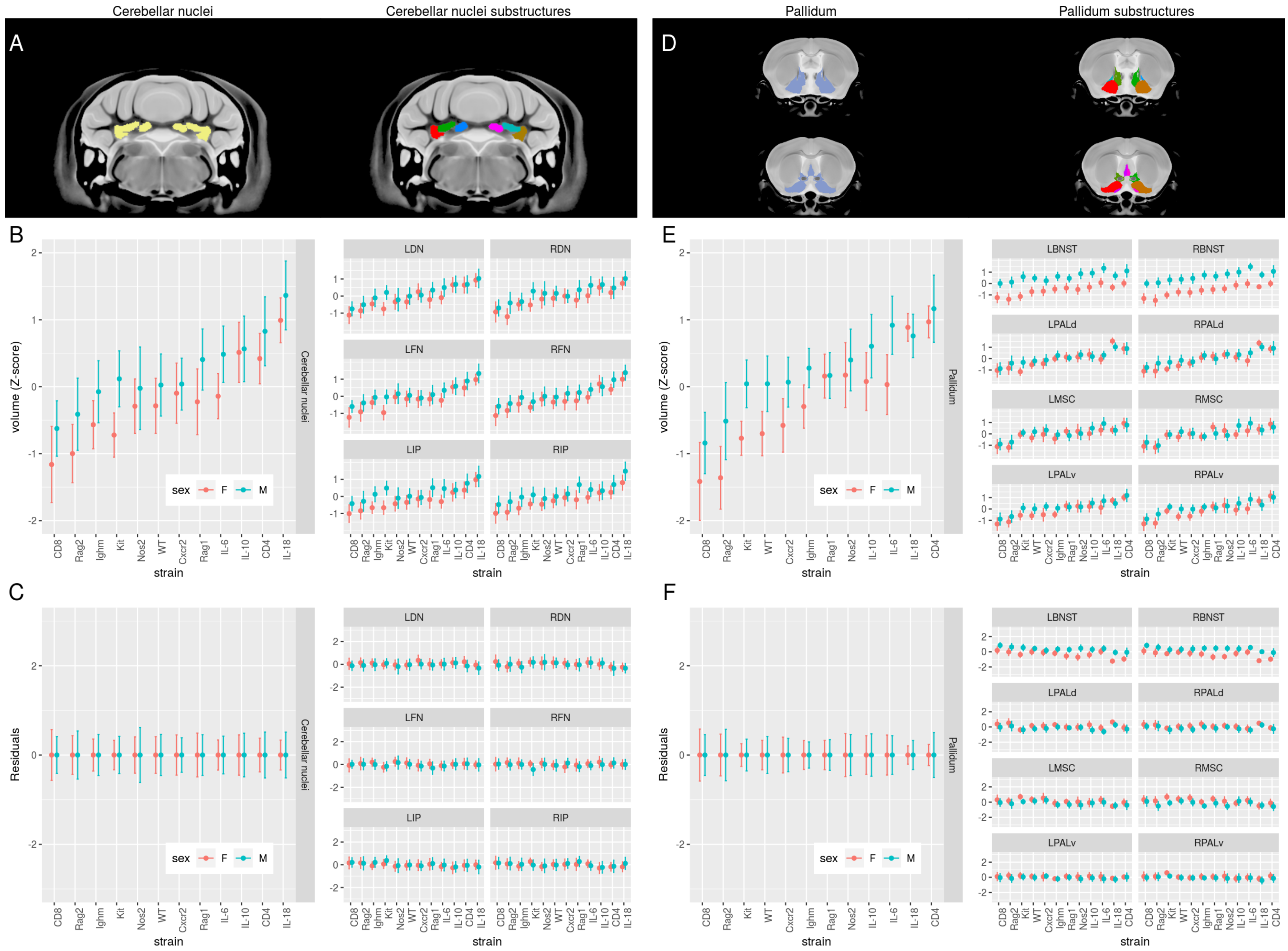

Supplementary Figure 8: Illustration of pruning neuroanatomy hierarchy.

(A) Segmentation of Cerebellar nuclei (Left) and its substructures (Right): Left Dentate Nucleus (LDN), Right Dentate Nucleus (RDN), Left Fastigial Nucleus (LFN), Right Fastigial Nucleus (LFN), Left Interposed Nucleus (LIP), and Right Interposed Nucleus (RIP). (B) Volume (as a Z-score) for the Cerebellar nuclei and each substructure. (C) Residuals from the model fitted to Cerebellar nuclei. Substructure residuals are not influenced by sex and strain, indicating that substructure response to sex and strain is well-accounted by the model fitted to the parent structure. This can be quantified rigorously using bayes factor comparing two models fitted to substructure residuals: intercept-only model vs sex and strain (with interaction) model. The intercept-only model is highly favoured (bayes factor for all substructures exceeded 10^{18}).

(D) Segmentation of Pallidum (Left) and its substructures (Right): Left Bed Nucleus of the Stria Terminalis (LBNST), Right Bed Nucleus of the Stria Terminalis (RBNST), Left Dorsal Pallidum (LPALd), Right Dorsal Pallidum (RPALd), Left Medial Septal Complex (LMSC), Right Medial Septal Complex (RMSC), Left Ventral Pallidum (LPALv), and Right Ventral Pallidum (RPALv). (E) Volume (as a Z-score) for the Pallidum and substructures. (F) Residuals from the model fitted to Pallidum. Substructure residuals are influenced by sex and strain, indicating that substructure response to sex and strain is not well-accounted by the model fitted to the parent structure: Bayes factor comparing two models fitted to substructures -- intercept-only model vs sex and strain (with interaction) model -- showed this to be the case: intercept-only model is highly unfavourable (bayes factor for RBNST is $<10^{-12}$).

This process was repeated for all 336 structures. If bayes-factor for the intercept-only model exceeded 100, the substructures were merged into the parent structure -- and the hierarchy was pruned. This resulted in 95 bilateral brain structures.

Error bars in all plots represent 95% confidence intervals.

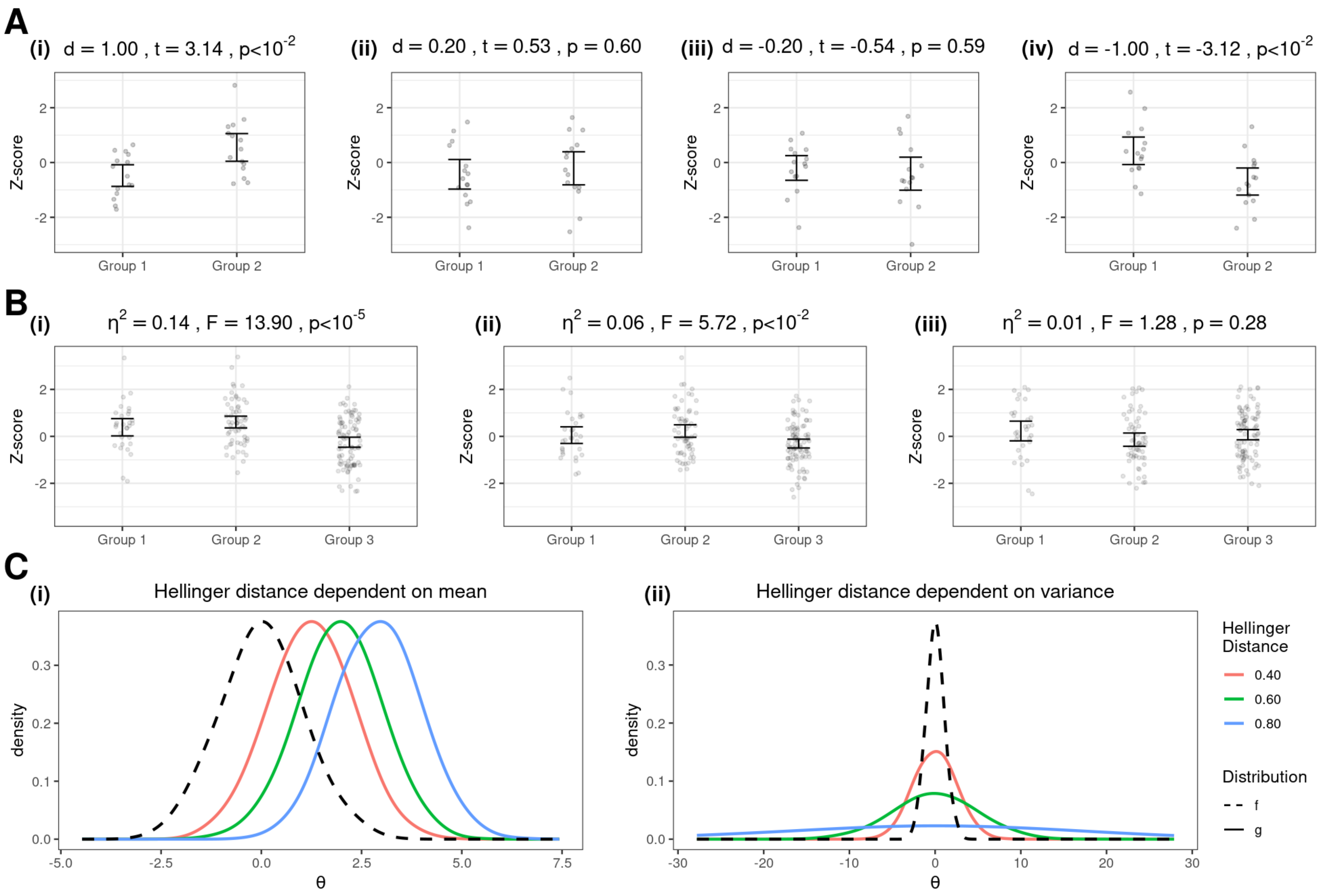

Supplementary Figure 9: Illustration of various statistics used in this manuscript. Simulated data was used throughout. We used approximately the same number of data points when illustrating the statistic as when the statistic was used in the manuscript. All error bars represent 95% confidence intervals for the group mean.

(A) Cohen's effect size (*d*) was used to assess differences between the mean of 2 groups (Lakens 2013). Points represent the 15 measurements per group which is approximately the same as when the statistic was used in the manuscript. To calculate *d*, the following equation was used:

$$d = \frac{\bar{x_2} - \bar{x_1}}{\sigma} = \bar{z_2} - \bar{z_1}$$

where $\bar{x_1}$ and $\bar{x_2}$ are the mean measurements in Group 1 and Group 2 respectively, σ is the pooled standard deviation, and $\bar{z_1}$ and $\bar{z_2}$ are the mean Z-score measurements for Group 1 and Group 2 respectively. Using simulated data, we show an example of (i) large positive effect size, (ii) weak positive effect size, (iii) weak negative effect size, and (iv) large negative effect size. We also provide the associated *t*-statistic and P-value for each example as comparison. Throughout the manuscript, 'Group 1' (i.e. the reference group) is the wild-type strain. Furthermore, as data was Z-transformed prior to fitting with BHM, the beta-coefficient associated with each strain is identical to the effect size for that strain versus the wild-type.

(B) η^2 effect size was used to assess how strongly measurements cluster in groups (Adams and Conway 2021). Points represent the volume measurements. The number of data points per group is approximately the same as when the statistic was used in the manuscript (15 measurements per strain with 2 strains for Group 1, 4 strains for Group 2, and 5 strains for Group 3). To calculate η^2, the following equation was used:

$$\eta^2 = \frac{\mathrm{SS_{group}}}{\mathrm{SS_{group}} + \mathrm{SS_{residuals}}}$$

where $\mathrm{SS_{group}}$ is the sum of squares for the group effect and $\mathrm{SS_{residuals}}$ is the sum of squares for the residuals. Using simulated data, we show an example of (i) large, (ii) medium, and (iii) small effect size. We also provide the associated *F*-statistic and P-value for each example as comparison.

(C) Hellinger distance (*H*) was used to assess the dissimilarity of two probability distributions (Boone, Merrick, and Krachey 2014). Hellinger distance runs between 0 and 1, with smaller values indicating more similarity in the probability distributions. Hellinger distance was calculated using the following equation:

$$H = \sqrt{1 - \int \mathrm{d}\theta \sqrt{f(\theta)\, g(\theta)}}$$

where f and g are the probability distributions being compared and θ is the argument for these distributions (can be multidimensional). In these plots, *f* (dashed line) is a standard normal distribution to which all other distributions *g* (solid line) are compared. Several *g* distributions are used to illustrate examples of how hellinger distance depends on the (i) mean and (ii) variance of the distributions being compared. The greater the similarity between 2 distributions, the lower the hellinger distance between them.

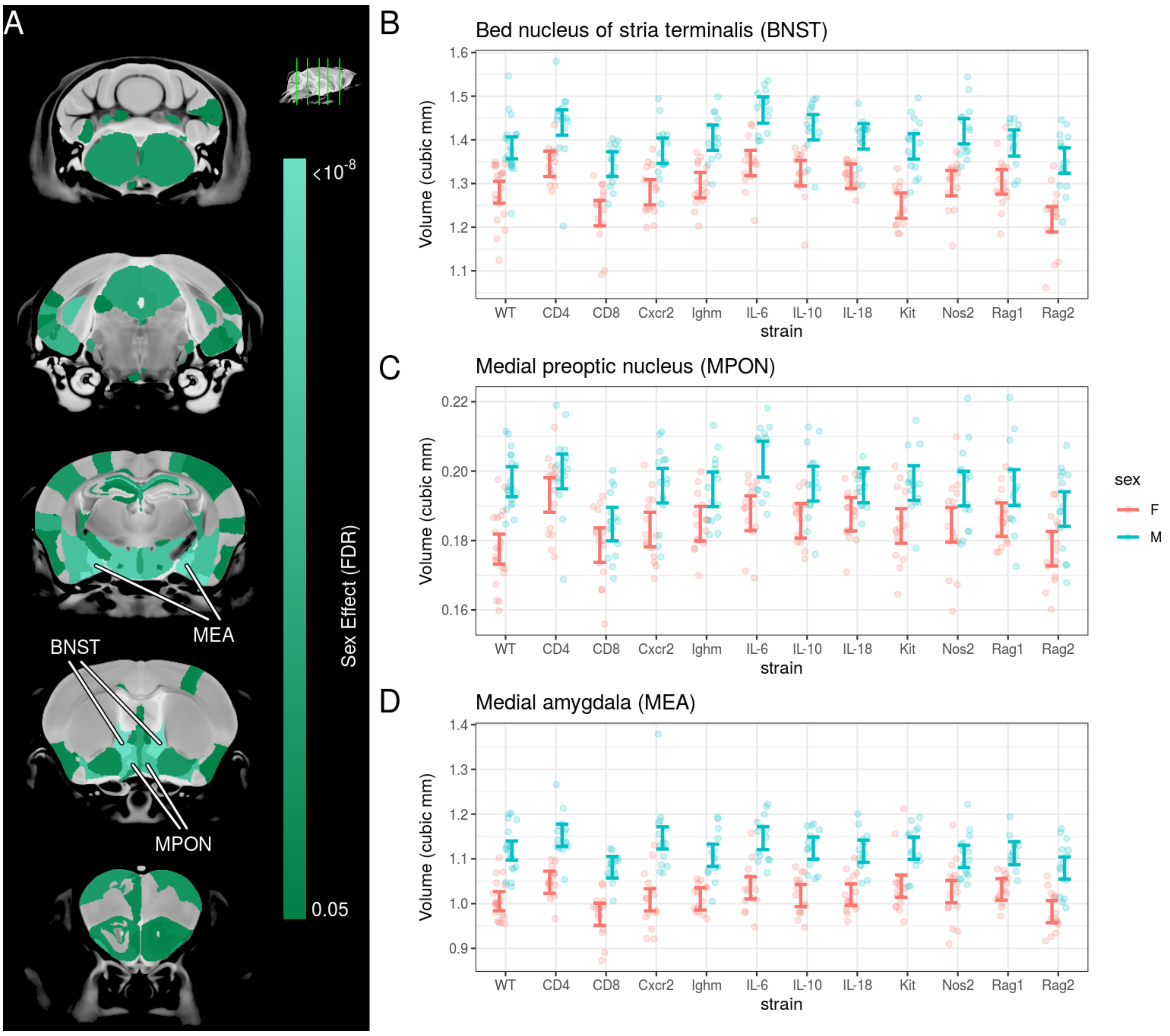

Supplementary Figure 10: Immune system mutations have a significant effect on the neuroanatomy of both sexes. (A) Sexual dimorphisms were also found throughout the brain, however no significant interactions between sex and the immune strains were identified. (B-D) The volume of canonical sexually dimorphic structures -- Bed nucleus of stria terminalis (BNST), Medial preoptic nucleus (MPON), Medial amygdala (MEA) -- in each strain are shown. These structures showed a significant effect of strain (BNST, $F_{22,369}$=6.87,$p<10^{-16}$; MPON, $F_{22,369}$=3.49,$p<10^{-6}$; MEA, $F_{22,369}$=3.24,$p<10^{-5}$) and sex (BNST, $F_{12,359}$=28.37,$p<10^{-43}$; MPON, $F_{12,359}$=11.29,$p<10^{-19}$; MEA, $F_{12,359}$=34.05, $p<10^{-50}$), but no significant sex-strain interaction (BNST, $F_{11,358}$=0.59,p=0.8; MPON, $F_{11,358}$=1.23,p=0.26; MEA, $F_{11,358}$=0.84,p=0.6). Colour maps show effects under 5% FDR and error bars represent 95% confidence intervals.

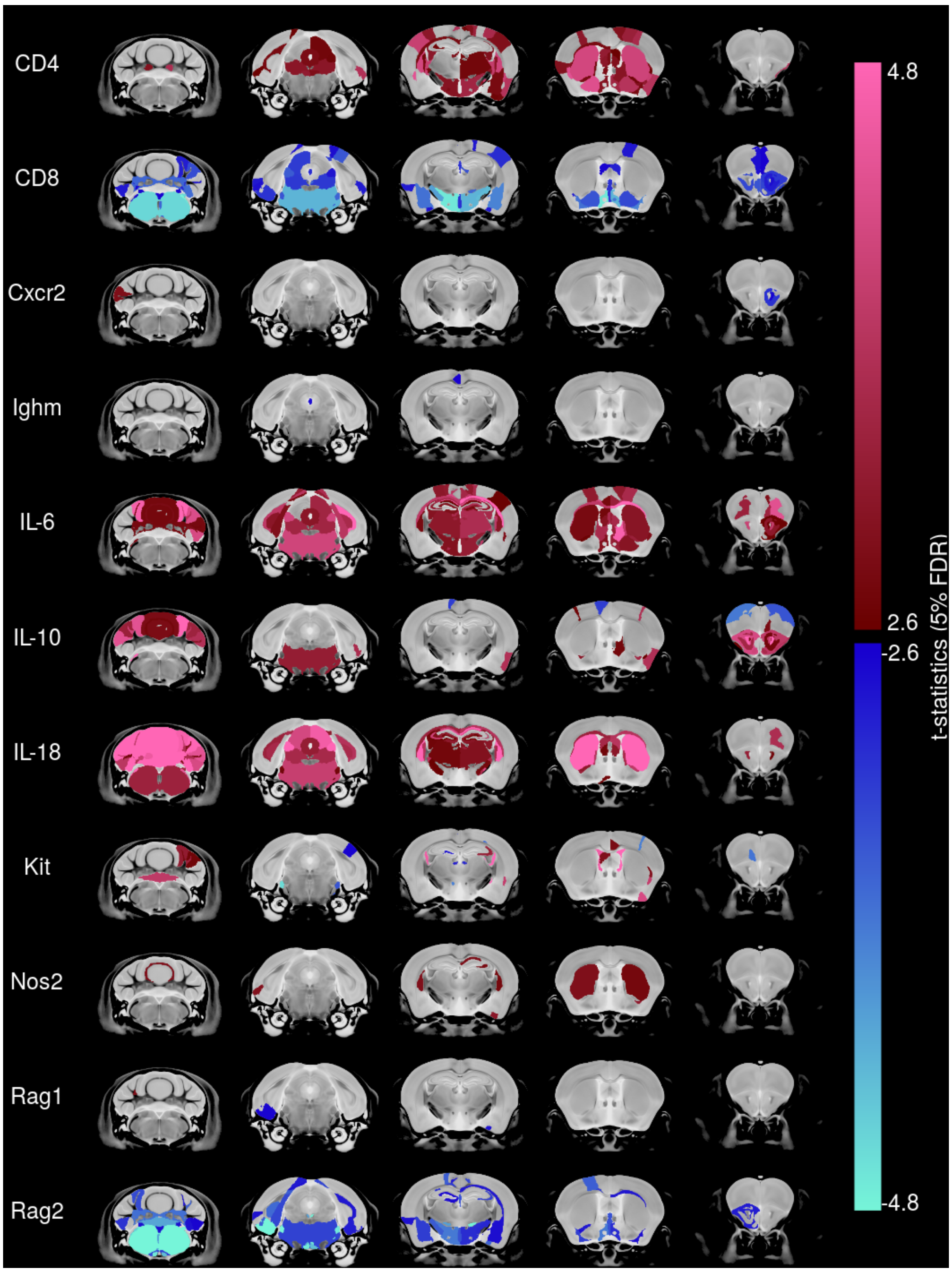

Supplementary Figure 11: Immune system mutations have a highly heterogeneous effect on mouse brain anatomy. The directional effect in males of the various mutant strains relative to the wild-type strains is visualized using t-statistics and shows a heterogeneous neuroanatomical phenotype. Regions larger or smaller in mutants relative to wild-type are given maroon-pink and blue-turquoise colours, respectively, if effects are <5% FDR. Saturated colours represent effects <0.01% FDR.

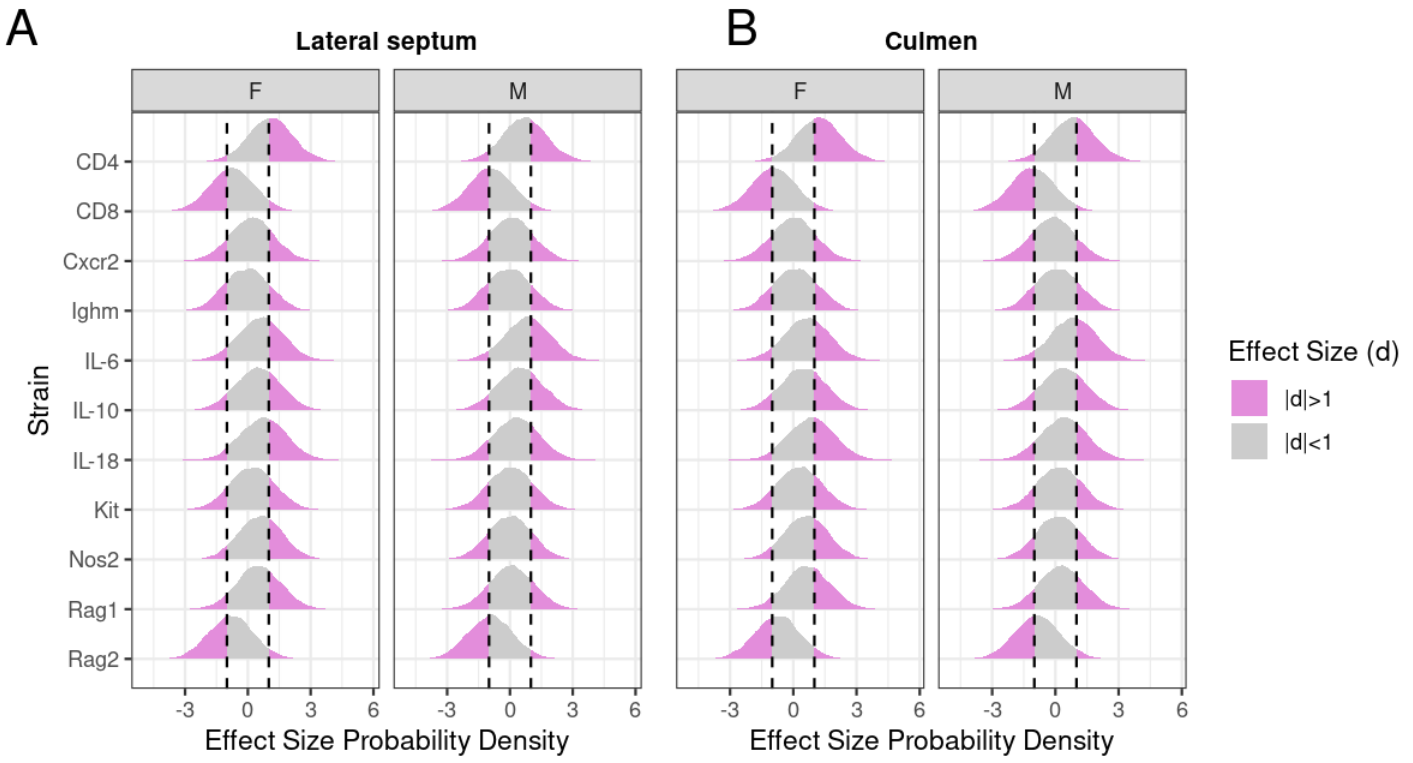

Supplementary Figure 12: Effect size (*d*) distributions for Lateral septum and Culmen determined using the BHM. This figure is an extension of Figure 2 in the main manuscript.

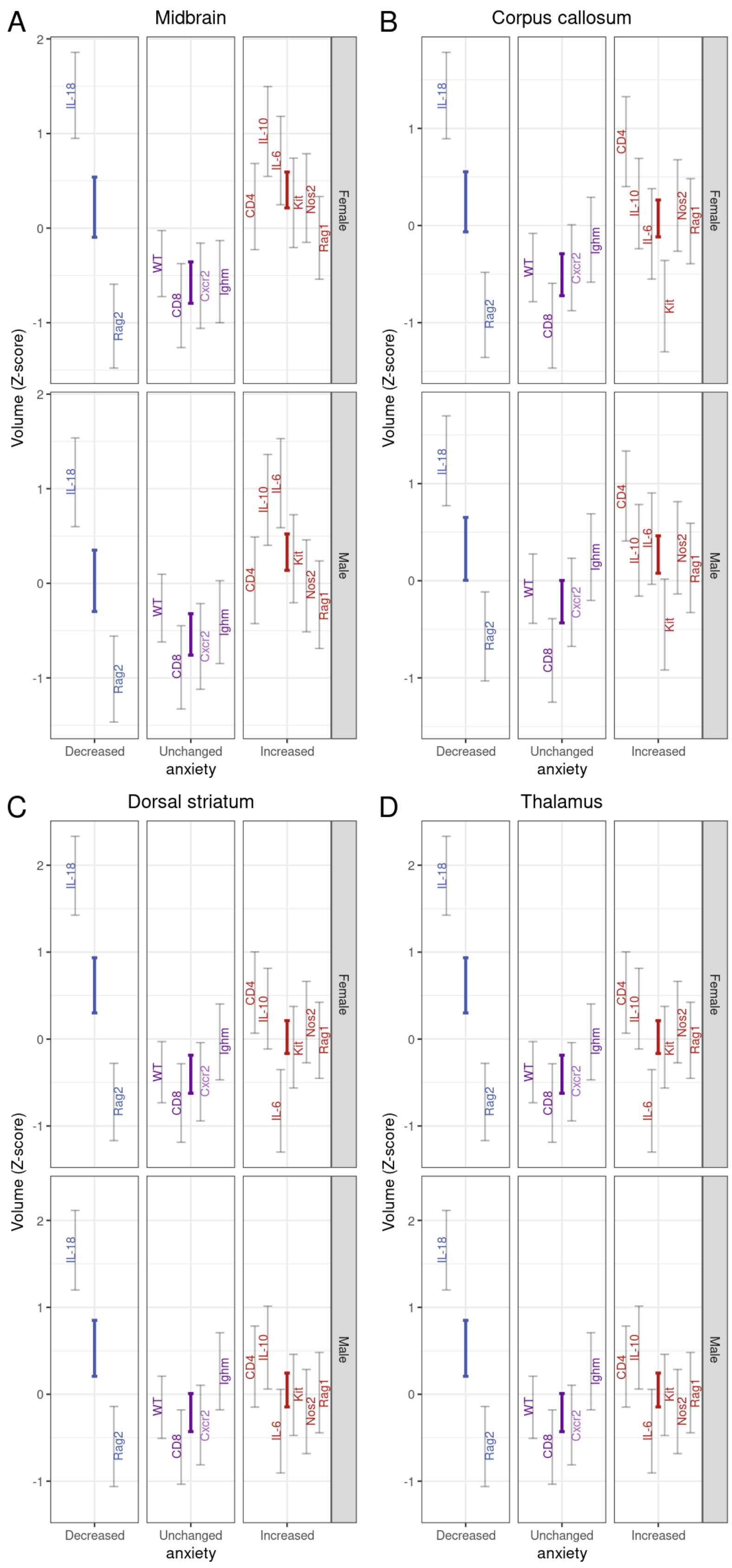

Supplementary Figure 13: Relationship between anxiety and volume of (A) Midbrain, (B) Corpus callosum, (C) Dorsal striatum, and (D) Thalamus as determined using the BHM. This figure is an extension of Figure 3C in the main manuscript.

References

Adams, Marc A., and Terry L. Conway. 2021. “Eta Squared.” *Encyclopedia of Quality of Life and Well-Being Research*. https://doi.org/10.1007/978-3-319-69909-7_918-2.

Boone, Edward L., Jason R. W. Merrick, and Matthew J. Krachey. 2014. “A Hellinger Distance Approach to MCMC Diagnostics.” *Journal of Statistical Computation and Simulation* 84 (4): 833–49.

Chakravarty, M. Mallar, Patrick Steadman, Matthijs C. van Eede, Rebecca D. Calcott, Victoria Gu, Philip Shaw, Armin Raznahan, D. Louis Collins, and Jason P. Lerch. 2013. “Performing Label-Fusion-Based Segmentation Using Multiple Automatically Generated Templates.” *Human Brain Mapping* 34 (10): 2635–54.

Lakens, Daniël. 2013. “Calculating and Reporting Effect Sizes to Facilitate Cumulative Science: A Practical Primer for T-Tests and ANOVAs.” *Frontiers in Psychology*. https://doi.org/10.3389/fpsyg.2013.00863.

McHugh, Mary L. 2012. “Interrater Reliability: The Kappa Statistic.” *Biochemia Medica: Casopis Hrvatskoga Drustva Medicinskih Biokemicara / HDMB* 22 (3): 276–82.
