# Supplementary material for: Mouse models of immune dysfunction: their neuroanatomical differences reflect their anxiety-behavioural phenotype
Source: Mol Psychiatry. 2022 Apr 14;27(7):3047–55. doi: 10.1038/s41380-022-01535-5 (PMC9205773; doi:10.1038/s41380-022-01535-5)
Supplement: Supplementary file 2 — Supplementary Tables [file 41380_2022_1535_MOESM2_ESM.zip › Supplementary Tables/Supplementary Tables.pdf]

Supplementary Table 1: Mean and Standard deviation of physical measures for all mouse strains and sex in our study.

strain	sex	measure	n	mean value	standard deviation
C57Bl6/J (WT)	F	body length (mm)	15	85.00	2.59
C57Bl6/J (WT)	F	body weight (g)	15	20.49	1.14
C57Bl6/J (WT)	F	gonad weight (mg)	15	6.88	0.85
C57Bl6/J (WT)	F	uterine weight (mg)	15	76.20	36.91
C57Bl6/J (WT)	M	body length (mm)	15	90.67	2.50
C57Bl6/J (WT)	M	body weight (g)	15	26.99	2.09
C57Bl6/J (WT)	M	gonad weight (mg)	15	183.06	16.68
Cxcr2 (WT)	F	body length (mm)	5	86.60	1.82
Cxcr2 (WT)	F	body weight (g)	5	19.96	1.99
Cxcr2 (WT)	F	gonad weight (mg)	5	8.32	2.05
Cxcr2 (WT)	F	uterine weight (mg)	5	96.32	38.13
Cxcr2 (WT)	M	body length (mm)	5	89.80	1.79
Cxcr2 (WT)	M	body weight (g)	5	26.24	1.21
Cxcr2 (WT)	M	gonad weight (mg)	5	196.14	8.65
CD4	F	body length (mm)	15	87.73	2.05
CD4	F	body weight (g)	15	22.14	1.17
CD4	F	gonad weight (mg)	15	7.31	1.14
CD4	F	uterine weight (mg)	15	128.50	49.26
CD4	M	body length (mm)	15	91.13	2.07
CD4	M	body weight (g)	15	27.34	1.30
CD4	M	gonad weight (mg)	15	191.47	18.39
CD8	F	body length (mm)	15	86.67	1.99
CD8	F	body weight (g)	15	20.31	1.36
CD8	F	gonad weight (mg)	14	8.46	2.11
CD8	F	uterine weight (mg)	15	109.98	42.40
CD8	M	body length (mm)	17	90.24	1.44
CD8	M	body weight (g)	17	25.91	1.55
CD8	M	gonad weight (mg)	17	183.08	17.63
Cxcr2	F	body length (mm)	15	86.73	2.63
Cxcr2	F	body weight (g)	15	19.88	1.48
Cxcr2	F	gonad weight (mg)	15	7.49	1.19
Cxcr2	F	uterine weight (mg)	15	77.39	34.29
Cxcr2	M	body length (mm)	15	89.73	1.87
Cxcr2	M	body weight (g)	15	24.97	1.88
Cxcr2	M	gonad weight (mg)	15	182.22	14.86
Ighm	F	body length (mm)	15	87.47	1.25
Ighm	F	body weight (g)	15	21.10	1.00
Ighm	F	gonad weight (mg)	15	7.73	1.72
Ighm	F	uterine weight (mg)	15	110.49	65.16
Ighm	M	body length (mm)	15	90.53	2.17
Ighm	M	body weight (g)	15	26.36	1.53
Ighm	M	gonad weight (mg)	15	182.97	11.06
IL-6	F	body length (mm)	15	85.00	2.14
IL-6	F	body weight (g)	15	19.19	1.44
IL-6	F	gonad weight (mg)	15	7.19	1.51
IL-6	F	uterine weight (mg)	15	94.47	44.88
IL-6	M	body length (mm)	15	89.47	1.51
IL-6	M	body weight (g)	15	25.68	1.37
IL-6	M	gonad weight (mg)	15	192.74	9.35
IL-10	F	body length (mm)	16	89.25	2.08
IL-10	F	body weight (g)	16	22.29	1.13
IL-10	F	gonad weight (mg)	16	9.31	1.94
IL-10	F	uterine weight (mg)	16	106.13	50.71
IL-10	M	body length (mm)	15	91.47	2.20
IL-10	M	body weight (g)	15	26.65	1.22
IL-10	M	gonad weight (mg)	15	209.18	14.71
IL-18	F	body length (mm)	16	87.81	2.34
IL-18	F	body weight (g)	16	23.32	1.52
IL-18	F	gonad weight (mg)	16	9.31	2.26
IL-18	F	uterine weight (mg)	16	93.72	32.82
IL-18	M	body length (mm)	15	91.93	1.79
IL-18	M	body weight (g)	15	28.89	1.54
IL-18	M	gonad weight (mg)	15	166.93	11.87
Kit	F	body length (mm)	15	87.53	2.97
Kit	F	body weight (g)	15	22.20	1.13
Kit	F	gonad weight (mg)	15	5.75	2.17
Kit	F	uterine weight (mg)	15	85.01	43.41
Kit	M	body length (mm)	15	92.07	2.87
Kit	M	body weight (g)	15	28.72	2.40
Kit	M	gonad weight (mg)	15	128.53	27.45
Nos2	F	body length (mm)	15	88.80	1.66
Nos2	F	body weight (g)	15	22.46	0.77
Nos2	F	gonad weight (mg)	15	9.67	1.91
Nos2	F	uterine weight (mg)	15	94.48	32.60
Nos2	M	body length (mm)	15	91.87	2.20
Nos2	M	body weight (g)	15	28.01	0.86
Nos2	M	gonad weight (mg)	15	193.53	8.67
Rag1	F	body length (mm)	16	85.56	2.03
Rag1	F	body weight (g)	16	19.61	1.10
Rag1	F	gonad weight (mg)	16	7.69	1.29
Rag1	F	uterine weight (mg)	16	111.24	55.05
Rag1	M	body length (mm)	14	90.14	2.44
Rag1	M	body weight (g)	14	25.71	1.70
Rag1	M	gonad weight (mg)	14	188.74	8.26
Rag2	F	body length (mm)	15	85.60	1.84
Rag2	F	body weight (g)	15	18.88	1.09
Rag2	F	gonad weight (mg)	15	6.49	1.20
Rag2	F	uterine weight (mg)	14	105.89	61.44
Rag2	M	body length (mm)	15	90.07	1.22
Rag2	M	body weight (g)	15	23.84	1.51
Rag2	M	gonad weight (mg)	15	171.36	25.47

Supplementary Table 2: Priors used in the bayesian hierarchical model

The priors used were centered and non-informative. Centered priors assume a priori that effect sizes between groups are 0. Non-informative priors assume *a priori* that a wide range of effect sizes are possible, but extremely large effect sizes are unlikely. By using centered and non-informative priors, any large effect sizes in the posterior distribution (which was used for inference) were due to patterns in the data and not a choice of the prior. Frequentist statistics implicitly assume a flat prior, which implies that small and infinite effect sizes are equally likely *a priori*. Stan was used to fit the BHM by running four parallel chains, each with 2500 discarded warm-up iteration and 2500 sampling iterations -- resulting in a total of 10 000 iterations. The following model diagnostics were seen to ensure that model fitting occurred properly: no iterations had divergences, no iteration saturated the maximum tree depth of 10, energy Bayesian fraction of missing information exceeded 0.2 for all predictors, all potential scale reduction factors were less than 1.013, number of effective samples exceeded 666. We also refit the data using different correlation matrix priors (LKJ with shape 1, 1000, and 100 000) and found similar predictions. This indicates models converged to a similar solution despite the different priors.

predictor/variable	level	group	class	prior distribution
			Sigma (standard deviation of y)	half student t (df = 3, location = 0, scale = 2.5)
			Global Intercept	student t (df = 3, location = 0, scale = 2.5)
sex	male		Global effect	student t (df = 3, location = 0, scale = 2.5)
sex-strain interaction	male, CD4		Global effect	student t (df = 3, location = 0, scale = 2.5)
sex-strain interaction	male, CD8		Global effect	student t (df = 3, location = 0, scale = 2.5)
sex-strain interaction	male, Cxcr2		Global effect	student t (df = 3, location = 0, scale = 2.5)
sex-strain interaction	male, IL-10		Global effect	student t (df = 3, location = 0, scale = 2.5)
sex-strain interaction	male, IL-18		Global effect	student t (df = 3, location = 0, scale = 2.5)
sex-strain interaction	male, IL-6		Global effect	student t (df = 3, location = 0, scale = 2.5)
sex-strain interaction	male, Ighm		Global effect	student t (df = 3, location = 0, scale = 2.5)
sex-strain interaction	male, Kit		Global effect	student t (df = 3, location = 0, scale = 2.5)
sex-strain interaction	male, Nos2		Global effect	student t (df = 3, location = 0, scale = 2.5)
sex-strain interaction	male, Rag2		Global effect	student t (df = 3, location = 0, scale = 2.5)
sex-strain interaction	male, Rag1		Global effect	student t (df = 3, location = 0, scale = 2.5)
strain	CD4		Global effect	student t (df = 3, location = 0, scale = 2.5)
strain	CD8		Global effect	student t (df = 3, location = 0, scale = 2.5)
strain	Cxcr2		Global effect	student t (df = 3, location = 0, scale = 2.5)
strain	IL-10		Global effect	student t (df = 3, location = 0, scale = 2.5)
strain	IL-18		Global effect	student t (df = 3, location = 0, scale = 2.5)
strain	IL-6		Global effect	student t (df = 3, location = 0, scale = 2.5)
strain	Ighm		Global effect	student t (df = 3, location = 0, scale = 2.5)
strain	Kit		Global effect	student t (df = 3, location = 0, scale = 2.5)
strain	Nos2		Global effect	student t (df = 3, location = 0, scale = 2.5)
strain	Rag2		Global effect	student t (df = 3, location = 0, scale = 2.5)
strain	Rag1		Global effect	student t (df = 3, location = 0, scale = 2.5)
intercept		mouse	Group standard deviations	student t (df = 3, location = 0, scale = 2.5)
correlation matrix		structure	Cholesky factor	LKJ density (shape = 10)
intercept		structure	Group standard deviations	half student t (df = 3, location = 0, scale = 2.5)
sex	male	structure	Group standard deviations	half student t (df = 3, location = 0, scale = 2.5)
sex-strain interaction	male, CD4	structure	Group standard deviations	half student t (df = 3, location = 0, scale = 2.5)
sex-strain interaction	male, CD8	structure	Group standard deviations	half student t (df = 3, location = 0, scale = 2.5)
sex-strain interaction	male, Cxcr2	structure	Group standard deviations	half student t (df = 3, location = 0, scale = 2.5)
sex-strain interaction	male, IL-10	structure	Group standard deviations	half student t (df = 3, location = 0, scale = 2.5)
sex-strain interaction	male, IL-18	structure	Group standard deviations	half student t (df = 3, location = 0, scale = 2.5)
sex-strain interaction	male, IL-6	structure	Group standard deviations	half student t (df = 3, location = 0, scale = 2.5)
sex-strain interaction	male, Ighm	structure	Group standard deviations	half student t (df = 3, location = 0, scale = 2.5)
sex-strain interaction	male, Kit	structure	Group standard deviations	half student t (df = 3, location = 0, scale = 2.5)
sex-strain interaction	male, Nos2	structure	Group standard deviations	half student t (df = 3, location = 0, scale = 2.5)
sex-strain interaction	male, Rag2	structure	Group standard deviations	half student t (df = 3, location = 0, scale = 2.5)
sex-strain interaction	male, Rag1	structure	Group standard deviations	half student t (df = 3, location = 0, scale = 2.5)
strain	CD4	structure	Group standard deviations	half student t (df = 3, location = 0, scale = 2.5)
strain	CD8	structure	Group standard deviations	half student t (df = 3, location = 0, scale = 2.5)
strain	Cxcr2	structure	Group standard deviations	half student t (df = 3, location = 0, scale = 2.5)
strain	IL-10	structure	Group standard deviations	half student t (df = 3, location = 0, scale = 2.5)
strain	IL-18	structure	Group standard deviations	half student t (df = 3, location = 0, scale = 2.5)
strain	IL-6	structure	Group standard deviations	half student t (df = 3, location = 0, scale = 2.5)
strain	Ighm	structure	Group standard deviations	half student t (df = 3, location = 0, scale = 2.5)
strain	Kit	structure	Group standard deviations	half student t (df = 3, location = 0, scale = 2.5)
strain	Nos2	structure	Group standard deviations	half student t (df = 3, location = 0, scale = 2.5)
strain	Rag2	structure	Group standard deviations	half student t (df = 3, location = 0, scale = 2.5)
strain	Rag1	structure	Group standard deviations	half student t (df = 3, location = 0, scale = 2.5)

Supplementary Table 3: Literature studies assessing the alterations of anxiety-like behaviour in response to dysregulation of specific elements in the immune system. OFT = open-field test, EPM = elevated-plus maze, L/D = light-dark test, HB = hole-board test, PA = passive avoidance test.

Immune system component	Experimental mouseline (nomenclature as in Reference)	Background strain and control mouseline	Sex	Age	Relevant test (s)	Effects	Notes	Anxiety-like behaviour (compared to control)	Reference
CD4	RAG-2^{-}/$^{-}$ reconstituted with CD4+ T cells	C57Bl/6	males only	10-14 weeks	OFT, EPM	Anxiety-like behaviour reduced in OFT and EPM of immune deficient RAG-2^{-}/$^{-}$ mice reconstituted with CD4+ T cells	No difference between wild-type and RAG-2^{-}/$^{-}$ mice in OFT and EPM but reduced anxiety-like behaviour in RAG-2^{-}/$^{-}$ mice reconstituted with CD4+ Tcells indicating anxiolytic effect of CD4+ T cells	Increased	[16]
CD8	RAG1^{-}/$^{-}$/OT-I (Rag1 deficient mutant expressing CD8+ T cells)	C57Bl/6	males only	5 weeks	OFT	No differences in OFT activity between RAG-1^{-}/$^{-}$ and RAG-1^{-}/$^{-}$/OT-I mutants	CD8+ T cells do not effect anxiety-like behaviours of RAG-1^{-}/$^{-}$ mutants	Unchanged	[51]
Cxcr2								No studies found	
Ighm	Ighm-/-	C57Bl/6	males and females	14-16 weeks	OFT, EPM, L/D	No genotype differences in OFT, EPM, L/D	Sex difference observed in TCR mice in L/D	Unchanged	[15]
IL-6	IL-6^{-}/$^{-}$	C57BL/6J	males only	3-6 months	OFT, L/D	IL-6^{-}/$^{-}$ mice display increased anxiety-like behaviour in the L/D with significantly decreased duration of stay inside the aversive lit compartment		Increased	[12]
IL-10	IL-10-/-	Balb/c	males and females	3 months	OFT, EPM	Male and female IL-10-/- mutants spent significantly less time in centre of OFT indicating increased anxiety-like behaviour	No differences found in EPM	Increased	[13]
IL-18	IL-18 KO	C57BL/6	males only	6 weeks	OFT, PA	IL-18 KO mice have shortened retention latency in PA indicating reduced anxiety-like behaviour	IL-18 KO mice show reduced rearing activity compared to wild-type in OFT indicating less exploratory behaviour in mutants compared to wild-type	Reduced	[14]
Kit	Sash^{-}/$^{-}$	C57BL/6	males only a	adult	OFT, EPM	Sash^{-}/$^{-}$ mice displayed increased latency in entering the centre square and entered the square fewer times than controls in OFT. Sash^{-}/$^{-}$ mice entered open arms fewer time and investigated entrances to open arms fewer times than wild-type controls in EPM indicating increased anxiety-like behaviours		Increased	[20]
Nos2	NOS2 mutant	B6; 129PF2/J	males only	adult	OFT, EPM, HB	NOS2 mutants showed reduced grooming in OFT and HB. NOS2 mutants fully avoided open arms, remained longer in the closed arms and exhibited significantly less head-dipping and stretch/attend posture in EPM indicating heightened anxiety	Behavioural phenotypes were unrelated to other systemic alterations such as changes in their blood pressure	Increased	[19]
Rag1	RAG-1^{-}/$^{-}$	C57Bl/6	males only 5	5 weeks	OFT	Reduced centre entries in OFT observed in RAG-1^{-}/$^{-}$ mutants indicating increased anxiety-like behaviour		Increased	[51]
Rag2	RAG-2^{-}/$^{-}$	BALB/c	males only	8-12 weeks	OFT, EPM	RAG-2^{-}/$^{-}$ exhibit reduced basal levels of anxiety-like behaviours and increased locomotor activity in OFT	No difference between wild-type and RAG-2^{-}/$^{-}$ mice in EPM	Reduced	[18]

Supplementary Table 4: Mean and Standard deviation of brain structure volumes for all mouse strains and sex in our study. Only 3/336 structures had significant unequal variance across groups evaluated using Breusch-Pagan Test (right lateral ventricle, FDR = 0.006; left habenular commissure, FDR = 0.03; left crus 1: ansiform lobule, FDR = 0.04; left Secondary auditory cortex: dorsal area, FDR = 0.04)

sex	strain	structure	mean volume (cubic mm)	standard deviation
F	WT	cerebral aqueduct	0.489	0.045
F	WT	fourth ventricle	0.920	0.073
F	WT	interpeduncular nucleus	0.272	0.014
F	WT	left Accessory olfactory bulb: glomerular, external plexiform and mitral cell layer	0.250	0.016
F	WT	left Accessory olfactory bulb: granule cell layer	0.120	0.009
F	WT	left amygdala	4.770	0.168
F	WT	left Amygdalopiriform transition area	0.530	0.039
F	WT	left anterior commissure: pars anterior	0.481	0.022
F	WT	left anterior commissure: pars posterior	0.206	0.012
F	WT	left anterior lobule (lobules 4-5)	0.843	0.044
F	WT	left anterior lobule white matter	0.043	0.004
F	WT	left Anterior olfactory nucleus	0.968	0.037
F	WT	left basal forebrain	2.539	0.081
F	WT	left bed nucleus of stria terminalis	0.643	0.036
F	WT	left CA10r	1.129	0.062
F	WT	left CA1Py	0.534	0.026
F	WT	left CA1Rad	1.209	0.041
F	WT	left CA20r	0.256	0.015
F	WT	left CA2Py	0.100	0.004
F	WT	left CA2Rad	0.202	0.007
F	WT	left CA30r	1.376	0.056
F	WT	left CA3Py Inner	0.075	0.004
F	WT	left CA3Py Outer	0.498	0.022
F	WT	left CA3Rad	0.889	0.039
F	WT	left Caudomedial entorhinal cortex	2.832	0.117
F	WT	left cerebellar peduncle: inferior	0.414	0.014
F	WT	left cerebellar peduncle: middle	0.566	0.028
F	WT	left cerebellar peduncle: superior	0.511	0.027
F	WT	left cerebral peduncle	1.008	0.028
F	WT	left Cingulate cortex: area 24a	0.918	0.059
F	WT	left Cingulate cortex: area 24a'	0.363	0.042
F	WT	left Cingulate cortex: area 24b	0.813	0.075
F	WT	left Cingulate cortex: area 24b'	0.304	0.020
F	WT	left Cingulate cortex: area 25	0.292	0.023
F	WT	left Cingulate cortex: area 29a	0.365	0.022
F	WT	left Cingulate cortex: area 29b	0.205	0.015
F	WT	left Cingulate cortex: area 29c	0.921	0.052
F	WT	left Cingulate cortex: area 30	1.433	0.093
F	WT	left Cingulate cortex: area 32	1.339	0.087
F	WT	left Cingulum	0.424	0.032
F	WT	left Claustrum	0.176	0.015
F	WT	left Claustrum: dorsal part	0.146	0.029
F	WT	left Claustrum: ventral part	0.263	0.032
F	WT	left colliculus: inferior	2.774	0.119
F	WT	left colliculus: superior	4.186	0.251
F	WT	left copula white matter	0.043	0.003
F	WT	left copula: pyramis (lobule 8)	1.135	0.096
F	WT	left corpus callosum	5.913	0.236
F	WT	left Cortex-amygdala transition zones	0.403	0.030
F	WT	left corticospinal tract/pyramids	0.845	0.056
F	WT	left crus 1 white matter	0.183	0.010
F	WT	left crus 1: ansiform lobule (lobule 6)	2.122	0.082
F	WT	left crus 2 white matter	0.126	0.008
F	WT	left crus 2: ansiform lobule (lobule 7)	1.944	0.113
F	WT	left cuneate nucleus	0.117	0.011
F	WT	left dentate nucleus	0.178	0.010
F	WT	left Dorsal intermediate entorhinal cortex	0.869	0.048
F	WT	left Dorsal nucleus of the endopiriform	0.719	0.034
F	WT	left Dorsal tenia tecta	0.487	0.022
F	WT	left Dorsolateral entorhinal cortex	1.199	0.086
F	WT	left Dorsolateral orbital cortex	0.443	0.019
F	WT	left Ectorhinal cortex	1.301	0.141
F	WT	left facial nerve (cranial nerve 7)	0.105	0.006
F	WT	left fasciculus retroflexus	0.128	0.007
F	WT	left fastigial nucleus	0.242	0.015
F	WT	left fimbria	1.617	0.086
F	WT	left flocculus (FL)	0.517	0.023
F	WT	left flocculus white matter	0.028	0.004
F	WT	left fornix	0.327	0.018
F	WT	left Frontal association cortex	3.589	0.132
F	WT	left Frontal cortex: area 3	0.356	0.022
F	WT	left fundus of striatum	0.072	0.008
F	WT	left globus pallidus	1.585	0.059
F	WT	left GrDG	0.666	0.022
F	WT	left habenular commissure	0.015	0.003
F	WT	left hypothalamus	5.133	0.159
F	WT	left inferior olivary complex	0.152	0.021
F	WT	left Insular region: not subdivided	3.769	0.370
F	WT	left Intermediate nucleus of the endopiriform claustrum	0.304	0.012
F	WT	left internal capsule	1.392	0.069
F	WT	left lateral olfactory tract	0.717	0.033
F	WT	left Lateral orbital cortex	1.782	0.090
F	WT	left Lateral parietal association cortex	0.150	0.014
F	WT	left lateral septum	1.495	0.082
F	WT	left lateral ventricle	1.803	0.225
F	WT	left LMol	1.158	0.031
F	WT	left mammillary bodies	0.242	0.024
F	WT	left mammilothalamic tract	0.123	0.007
F	WT	left Medial amygdala	0.491	0.023
F	WT	left Medial entorhinal cortex	0.348	0.017
F	WT	left medial lemniscus/medial longitudinal fasciculus	1.229	0.060
F	WT	left Medial orbital cortex	0.911	0.057
F	WT	left Medial parietal association cortex	0.233	0.029
F	WT	left Medial preoptic nucleus	0.092	0.007
F	WT	left medial septum	0.643	0.024
F	WT	left MoDG	2.126	0.059
F	WT	left nucleus accumbens	2.048	0.092
F	WT	left nucleus interpositus	0.223	0.014
F	WT	left Olfactory bulb: external plexiform layer	3.124	0.120
F	WT	left Olfactory bulb: glomerular layer	2.104	0.081
F	WT	left Olfactory bulb: granule cell layer	2.166	0.069
F	WT	left Olfactory bulb: internal plexiform layer	0.490	0.018
F	WT	left Olfactory bulb: mitral cell layer	0.621	0.020
F	WT	left olfactory peduncle	1.154	0.038
F	WT	left olfactory tubercle	1.631	0.143
F	WT	left optic tract	0.743	0.049
F	WT	left paraflocculus (PFL)	1.618	0.237
F	WT	left paraflocculus white matter	0.136	0.018
F	WT	left paramedian lobule	0.072	0.005
F	WT	left paramedian lobule (lobule 7)	1.987	0.104
F	WT	left Parietal cortex: posterior area: rostral part	0.070	0.009
F	WT	left Perirhinal cortex	1.239	0.083
F	WT	left Piriform cortex	5.105	0.199
F	WT	left PoDG	0.297	0.011
F	WT	left pontine nucleus	0.399	0.065
F	WT	left Posterolateral cortical amygdaloid area	0.404	0.027
F	WT	left Posteromedial cortical amygdaloid area	0.579	0.036
F	WT	left pre-para subiculum	1.116	0.040
F	WT	left Primary auditory cortex	0.753	0.051
F	WT	left Primary motor cortex	3.690	0.127
F	WT	left Primary somatosensory cortex	2.007	0.098
F	WT	left Primary somatosensory cortex: barrel field	4.714	0.208
F	WT	left Primary somatosensory cortex: dysgranular zone	0.170	0.016
F	WT	left Primary somatosensory cortex: forelimb region	2.078	0.122
F	WT	left Primary somatosensory cortex: hindlimb region	1.476	0.082
F	WT	left Primary somatosensory cortex: jaw region	0.271	0.032
F	WT	left Primary somatosensory cortex: shoulder region	0.089	0.009
F	WT	left Primary somatosensory cortex: trunk region	0.299	0.025
F	WT	left Primary somatosensory cortex: upper lip region	2.335	0.154
F	WT	left Primary visual cortex	1.164	0.104
F	WT	left Primary visual cortex: binocular area	1.002	0.059
F	WT	left Primary visual cortex: monocular area	1.138	0.087
F	WT	left Rostral amygdalopiriform area	0.196	0.015
F	WT	left Secondary auditory cortex: dorsal area	0.730	0.048
F	WT	left Secondary auditory cortex: ventral area	0.782	0.073
F	WT	left Secondary motor cortex	3.384	0.150
F	WT	left Secondary somatosensory cortex	3.347	0.389
F	WT	left Secondary visual cortex: lateral area	1.293	0.073
F	WT	left Secondary visual cortex: mediolateral area	0.589	0.067
F	WT	left Secondary visual cortex: mediomedial area	0.936	0.075
F	WT	left simple lobule (lobule 6)	2.319	0.118
F	WT	left simple lobule white matter	0.213	0.013
F	WT	left SLu	0.361	0.019
F	WT	left stria medullaris	0.321	0.013
F	WT	left stria terminalis	0.457	0.018
F	WT	left striatum	10.587	0.475
F	WT	left subependymale zone / rhinocele	0.028	0.002
F	WT	left subiculum	1.597	0.071
F	WT	left superior olivary complex	0.374	0.047
F	WT	left Temporal association area	1.392	0.132
F	WT	left thalamus	8.773	0.439
F	WT	left trunk of crus 2 and paramedian white matter	0.163	0.010
F	WT	left trunk of simple and crus 1 white matter	0.066	0.004
F	WT	left Ventral intermediate entorhinal cortex	0.556	0.033
F	WT	left Ventral nucleus of the endopiriform claustrum	0.248	0.030
F	WT	left Ventral orbital cortex	0.743	0.037
F	WT	left Ventral tenia tecta	0.056	0.004
F	WT	lobule 1-2 white matter	0.072	0.021
F	WT	lobule 10 white matter	0.081	0.006
F	WT	lobule 10: nodulus	1.314	0.081
F	WT	lobule 3 white matter	0.176	0.024
F	WT	lobule 3: central lobule (dorsal)	2.019	0.171
F	WT	lobule 6: declive	2.412	0.135
F	WT	lobule 7: tuber (or folium)	0.931	0.084
F	WT	lobule 8 white matter	0.153	0.011
F	WT	lobule 8: pyramis	1.560	0.099
F	WT	lobule 9 white matter	0.275	0.019
F	WT	lobule 9: uvula	2.641	0.175
F	WT	lobules 1-2: lingula and central lobule (ventral)	1.712	0.285
F	WT	lobules 4-5 white matter	0.555	0.034
F	WT	lobules 4-5: culmen (ventral and dorsal)	4.192	0.197
F	WT	lobules 6-7 white matter	0.681	0.049
F	WT	medulla	26.723	0.833
F	WT	midbrain	13.497	0.738
F	WT	periaqueductal grey	3.845	0.201
F	WT	pons	16.887	0.537
F	WT	posterior commissure	0.141	0.011
F	WT	right Accessory olfactory bulb: glomerular, external plexiform and mitral cell layer	0.240	0.013
F	WT	right Accessory olfactory bulb: granule cell layer	0.114	0.008
F	WT	right amygdala	4.407	0.172
F	WT	right Amygdalopiriform transition area	0.492	0.027
F	WT	right anterior commissure: pars anterior	0.473	0.022
F	WT	right anterior commissure: pars posterior	0.195	0.011
F	WT	right anterior lobule (lobules 4-5)	0.769	0.055
F	WT	right anterior lobule white matter	0.050	0.005
F	WT	right Anterior olfactory nucleus	0.990	0.044
F	WT	right basal forebrain	2.627	0.079
F	WT	right bed nucleus of stria terminalis	0.637	0.031
F	WT	right CA10r	0.978	0.053
F	WT	right CA1Py	0.537	0.026
F	WT	right CA1Rad	1.311	0.053
F	WT	right CA20r	0.208	0.013
F	WT	right CA2Py	0.095	0.003
F	WT	right CA2Rad	0.211	0.009
F	WT	right CA30r	1.351	0.067
F	WT	right CA3Py Inner	0.078	0.003
F	WT	right CA3Py Outer	0.492	0.029
F	WT	right CA3Rad	0.915	0.057
F	WT	right Caudomedial entorhinal cortex	2.750	0.150
F	WT	right cerebellar peduncle: inferior	0.384	0.015
F	WT	right cerebellar peduncle: middle	0.602	0.035
F	WT	right cerebellar peduncle: superior	0.488	0.031
F	WT	right cerebral peduncle	1.015	0.033
F	WT	right Cingulate cortex: area 24a	0.978	0.069
F	WT	right Cingulate cortex: area 24a'	0.348	0.037
F	WT	right Cingulate cortex: area 24b	0.784	0.071
F	WT	right Cingulate cortex: area 24b'	0.295	0.021
F	WT	right Cingulate cortex: area 25	0.300	0.020
F	WT	right Cingulate cortex: area 29a	0.356	0.023
F	WT	right Cingulate cortex: area 29b	0.197	0.015
F	WT	right Cingulate cortex: area 29c	0.893	0.059
F	WT	right Cingulate cortex: area 30	1.357	0.074
F	WT	right Cingulate cortex: area 32	1.090	0.100
F	WT	right Cingulum	0.429	0.025
F	WT	right Claustrum	0.147	0.011
F	WT	right Claustrum: dorsal part	0.139	0.016
F	WT	right Claustrum: ventral part	0.310	0.030
F	WT	right colliculus: inferior	2.644	0.113
F	WT	right colliculus: superior	4.283	0.261
F	WT	right copula white matter	0.034	0.002
F	WT	right copula: pyramis (lobule 8)	1.252	0.084
F	WT	right corpus callosum	5.649	0.342
F	WT	right Cortex-amygdala transition zones	0.425	0.042
F	WT	right corticospinal tract/pyramids	0.886	0.050
F	WT	right crus 1 white matter	0.179	0.008
F	WT	right crus 1: ansiform lobule (lobule 6)	2.126	0.081
F	WT	right crus 2 white matter	0.146	0.010
F	WT	right crus 2: ansiform lobule (lobule 7)	1.983	0.123
F	WT	right cuneate nucleus	0.124	0.013
F	WT	right dentate nucleus	0.176	0.012
F	WT	right Dorsal intermediate entorhinal cortex	0.897	0.047
F	WT	right Dorsal nucleus of the endopiriform	0.706	0.050
F	WT	right Dorsal tenia tecta	0.460	0.027
F	WT	right Dorsolateral entorhinal cortex	1.301	0.078
F	WT	right Dorsolateral orbital cortex	0.400	0.031
F	WT	right Ectorhinal cortex	1.316	0.119
F	WT	right facial nerve (cranial nerve 7)	0.109	0.005
F	WT	right fasciculus retroflexus	0.112	0.007
F	WT	right fastigial nucleus	0.229	0.013
F	WT	right fimbria	1.476	0.084
F	WT	right flocculus (FL)	0.496	0.032
F	WT	right flocculus white matter	0.038	0.003
F	WT	right fornix	0.289	0.013
F	WT	right Frontal association cortex	4.018	0.183
F	WT	right Frontal cortex: area 3	0.366	0.022
F	WT	right fundus of striatum	0.079	0.006
F	WT	right globus pallidus	1.599	0.072
F	WT	right GrDG	0.627	0.027
F	WT	right habenular commissure	0.015	0.003
F	WT	right hypothalamus	5.076	0.155
F	WT	right inferior olivary complex	0.170	0.024
F	WT	right Insular region: not subdivided	3.993	0.286
F	WT	right Intermediate nucleus of the endopiriform claustrum	0.281	0.019
F	WT	right internal capsule	1.479	0.071
F	WT	right lateral olfactory tract	0.634	0.031
F	WT	right Lateral orbital cortex	1.741	0.090
F	WT	right Lateral parietal association cortex	0.094	0.011
F	WT	right lateral septum	1.486	0.080
F	WT	right lateral ventricle	1.819	0.209
F	WT	right LMol	1.145	0.043
F	WT	right mammillary bodies	0.225	0.020
F	WT	right mammilothalamic tract	0.126	0.009

F	WT	right Medial amygdala	0.515	0.027
F	WT	right Medial entorhinal cortex	0.323	0.022
F	WT	right medial lemniscus/medial longitudinal fasciculus	1.313	0.055
F	WT	right Medial orbital cortex	0.835	0.043
F	WT	right Medial parietal association cortex	0.178	0.018
F	WT	right Medial preoptic nucleus	0.085	0.006
F	WT	right medial septum	0.604	0.024
F	WT	right MoDG	1.989	0.066
F	WT	right nucleus accumbens	2.034	0.084
F	WT	right nucleus interpositus	0.234	0.014
F	WT	right Olfactory bulb: external plexiform layer	3.104	0.154
F	WT	right Olfactory bulb: glomerular layer	2.165	0.085
F	WT	right Olfactory bulb: granule cell layer	2.207	0.088
F	WT	right Olfactory bulb: internal plexiform layer	0.480	0.032
F	WT	right Olfactory bulb: mitral cell layer	0.578	0.033
F	WT	right olfactory peduncle	1.113	0.040
F	WT	right olfactory tubercle	1.632	0.102
F	WT	right optic tract	0.705	0.031
F	WT	right paraflocculus (PFL)	1.613	0.200
F	WT	right paraflocculus white matter	0.157	0.019
F	WT	right paramedian lobule	0.079	0.007
F	WT	right paramedian lobule (lobule 7)	1.962	0.088
F	WT	right Parietal cortex: posterior area: rostral part	0.046	0.004
F	WT	right Perirhinal cortex	1.174	0.104
F	WT	right Piriform cortex	5.314	0.279
F	WT	right PoDG	0.314	0.013
F	WT	right pontine nucleus	0.382	0.053
F	WT	right Posterolateral cortical amygdaloid area	0.436	0.033
F	WT	right Posteromedial cortical amygdaloid area	0.443	0.027
F	WT	right pre-para subiculum	1.156	0.046
F	WT	right Primary auditory cortex	0.720	0.052
F	WT	right Primary motor cortex	3.206	0.140
F	WT	right Primary somatosensory cortex	2.061	0.121
F	WT	right Primary somatosensory cortex: barrel field	4.697	0.244
F	WT	right Primary somatosensory cortex: dysgranular zone	0.182	0.015
F	WT	right Primary somatosensory cortex: forelimb region	1.841	0.155
F	WT	right Primary somatosensory cortex: hindlimb region	1.235	0.110
F	WT	right Primary somatosensory cortex: jaw region	0.285	0.025
F	WT	right Primary somatosensory cortex: shoulder region	0.069	0.010
F	WT	right Primary somatosensory cortex: trunk region	0.202	0.017
F	WT	right Primary somatosensory cortex: upper lip region	2.752	0.173
F	WT	right Primary visual cortex	1.193	0.093
F	WT	right Primary visual cortex: binocular area	1.020	0.096
F	WT	right Primary visual cortex: monocular area	0.806	0.065
F	WT	right Rostral amygdalopiriform area	0.204	0.019
F	WT	right Secondary auditory cortex: dorsal area	0.695	0.054
F	WT	right Secondary auditory cortex: ventral area	0.961	0.089
F	WT	right Secondary motor cortex	3.313	0.169
F	WT	right Secondary somatosensory cortex	3.536	0.289
F	WT	right Secondary visual cortex: lateral area	1.459	0.101
F	WT	right Secondary visual cortex: mediolateral area	0.408	0.034
F	WT	right Secondary visual cortex: mediomedial area	0.794	0.046
F	WT	right simple lobule (lobule 6)	2.371	0.099
F	WT	right simple lobule white matter	0.177	0.010
F	WT	right SLu	0.356	0.027
F	WT	right stria medullaris	0.340	0.013
F	WT	right stria terminalis	0.384	0.015
F	WT	right striatum	10.450	0.487
F	WT	right subependymale zone / rhinocele	0.024	0.003
F	WT	right subiculum	1.580	0.075
F	WT	right superior olivary complex	0.397	0.062
F	WT	right Temporal association area	1.529	0.147
F	WT	right thalamus	8.696	0.453
F	WT	right trunk of crus 2 and paramedian white matter	0.174	0.010
F	WT	right trunk of simple and crus 1 white matter	0.082	0.005
F	WT	right Ventral intermediate entorhinal cortex	0.540	0.025
F	WT	right Ventral nucleus of the endopiriform claustrum	0.231	0.017
F	WT	right Ventral orbital cortex	0.684	0.037
F	WT	right Ventral tenia tecta	0.055	0.005
F	WT	third ventricle	1.235	0.096
F	WT	trunk of arbor vita	4.091	0.129
F	WT	trunk of lobules 1-3 white matter	0.121	0.011
F	WT	trunk of lobules 6-8 white matter	0.105	0.008
F	WT	ventral tegmental decussation	0.128	0.012
F	CD4	cerebral aqueduct	0.492	0.058
F	CD4	fourth ventricle	0.949	0.063
F	CD4	interpedunclar nucleus	0.280	0.013
F	CD4	left Accessory olfactory bulb: glomerular, external plexiform and mitral cell layer	0.258	0.014
F	CD4	left Accessory olfactory bulb: granule cell layer	0.120	0.007
F	CD4	left amygdala	5.026	0.161
F	CD4	left Amygdalopiriform transition area	0.549	0.031
F	CD4	left anterior commissure: pars anterior	0.502	0.023
F	CD4	left anterior commissure: pars posterior	0.225	0.011
F	CD4	left anterior lobule (lobules 4-5)	0.859	0.053
F	CD4	left anterior lobule white matter	0.042	0.003
F	CD4	left Anterior olfactory nucleus	1.001	0.046
F	CD4	left basal forebrain	2.725	0.060
F	CD4	left bed nucleus of stria terminalis	0.676	0.024
F	CD4	left CA10r	1.195	0.064
F	CD4	left CA1Py	0.562	0.022
F	CD4	left CA1Rad	1.262	0.039
F	CD4	left CA20r	0.270	0.011
F	CD4	left CA2Py	0.106	0.007
F	CD4	left CA2Rad	0.212	0.013
F	CD4	left CA30r	1.473	0.073
F	CD4	left CA3Py Inner	0.080	0.005
F	CD4	left CA3Py Outer	0.534	0.020
F	CD4	left CA3Rad	0.956	0.049
F	CD4	left Caudomedial entorhinal cortex	3.012	0.114
F	CD4	left cerebellar peduncle: inferior	0.440	0.018
F	CD4	left cerebellar peduncle: middle	0.604	0.030
F	CD4	left cerebellar peduncle: superior	0.547	0.026
F	CD4	left cerebral peduncle	1.075	0.040
F	CD4	left Cingulate cortex: area 24a	0.964	0.052
F	CD4	left Cingulate cortex: area 24a'	0.370	0.042
F	CD4	left Cingulate cortex: area 24b	0.865	0.091
F	CD4	left Cingulate cortex: area 24b'	0.324	0.022
F	CD4	left Cingulate cortex: area 25	0.307	0.013
F	CD4	left Cingulate cortex: area 29a	0.367	0.021
F	CD4	left Cingulate cortex: area 29b	0.216	0.015
F	CD4	left Cingulate cortex: area 29c	0.937	0.060
F	CD4	left Cingulate cortex: area 30	1.470	0.060
F	CD4	left Cingulate cortex: area 32	1.347	0.084
F	CD4	left Cingulum	0.451	0.022
F	CD4	left Claustrum	0.187	0.009
F	CD4	left Claustrum: dorsal part	0.157	0.019
F	CD4	left Claustrum: ventral part	0.291	0.028
F	CD4	left colliculus: inferior	2.929	0.085
F	CD4	left colliculus: superior	4.494	0.150
F	CD4	left copula white matter	0.047	0.004
F	CD4	left copula: pyramis (lobule 8)	1.237	0.082
F	CD4	left corpus callosum	6.300	0.183
F	CD4	left Cortex-amygdala transition zones	0.410	0.030
F	CD4	left corticospinal tract/pyramids	0.896	0.072
F	CD4	left crus 1 white matter	0.197	0.013
F	CD4	left crus 1: ansiform lobule (lobule 6)	2.207	0.083
F	CD4	left crus 2 white matter	0.134	0.010
F	CD4	left crus 2: ansiform lobule (lobule 7)	2.016	0.130
F	CD4	left cuneate nucleus	0.123	0.009
F	CD4	left dentate nucleus	0.190	0.010
F	CD4	left Dorsal intermediate entorhinal cortex	0.883	0.053
F	CD4	left Dorsal nucleus of the endopiriform	0.787	0.041
F	CD4	left Dorsal tenia tecta	0.500	0.032
F	CD4	left Dorsolateral entorhinal cortex	1.237	0.058
F	CD4	left Dorsolateral orbital cortex	0.433	0.029
F	CD4	left Ectorhinal cortex	1.317	0.084
F	CD4	left facial nerve (cranial nerve 7)	0.109	0.005
F	CD4	left fasciculus retroflexus	0.133	0.005
F	CD4	left fastigial nucleus	0.253	0.014
F	CD4	left fimbria	1.739	0.077
F	CD4	left flocculus (FL)	0.566	0.025
F	CD4	left flocculus white matter	0.031	0.003
F	CD4	left fornix	0.346	0.012
F	CD4	left Frontal association cortex	3.639	0.135
F	CD4	left Frontal cortex: area 3	0.368	0.020
F	CD4	left fundus of striatum	0.079	0.005
F	CD4	left globus pallidus	1.708	0.071
F	CD4	left GrDG	0.716	0.031
F	CD4	left habenular commissure	0.016	0.005
F	CD4	left hypothalamus	5.419	0.085
F	CD4	left inferior olivary complex	0.166	0.029
F	CD4	left Insular region: not subdivided	3.906	0.254
F	CD4	left Intermediate nucleus of the endopiriform claustrum	0.327	0.008
F	CD4	left internal capsule	1.486	0.051
F	CD4	left lateral olfactory tract	0.722	0.033
F	CD4	left Lateral orbital cortex	1.827	0.075
F	CD4	left Lateral parietal association cortex	0.160	0.014
F	CD4	left lateral septum	1.599	0.053
F	CD4	left lateral ventricle	2.043	0.247
F	CD4	left LMol	1.213	0.032
F	CD4	left mammillary bodies	0.248	0.023
F	CD4	left mammilothalamic tract	0.127	0.008
F	CD4	left Medial amygdala	0.512	0.027
F	CD4	left Medial entorhinal cortex	0.364	0.024
F	CD4	left medial lemniscus/medial longitudinal fasciculus	1.288	0.070
F	CD4	left Medial orbital cortex	0.944	0.033
F	CD4	left Medial parietal association cortex	0.243	0.016
F	CD4	left Medial preoptic nucleus	0.100	0.006
F	CD4	left medial septum	0.683	0.025
F	CD4	left MoDG	2.279	0.084
F	CD4	left nucleus accumbens	2.245	0.084
F	CD4	left nucleus interpositus	0.234	0.014
F	CD4	left Olfactory bulb: external plexiform layer	3.216	0.130
F	CD4	left Olfactory bulb: glomerular layer	2.168	0.079
F	CD4	left Olfactory bulb: granule cell layer	2.164	0.099
F	CD4	left Olfactory bulb: internal plexiform layer	0.499	0.025
F	CD4	left Olfactory bulb: mitral cell layer	0.635	0.031
F	CD4	left olfactory peduncle	1.177	0.037
F	CD4	left olfactory tubercle	1.656	0.059
F	CD4	left optic tract	0.797	0.022
F	CD4	left paraflocculus (PFL)	1.704	0.202
F	CD4	left paraflocculus white matter	0.146	0.015
F	CD4	left paramedian lobule	0.075	0.005
F	CD4	left paramedian lobule (lobule 7)	2.079	0.112
F	CD4	left Parietal cortex: posterior area: rostral part	0.074	0.006
F	CD4	left Perirhinal cortex	1.251	0.058
F	CD4	left Piriform cortex	5.261	0.166
F	CD4	left PoDG	0.315	0.015
F	CD4	left pontine nucleus	0.421	0.061
F	CD4	left Posterolateral cortical amygdaloid area	0.428	0.029
F	CD4	left Posteromedial cortical amygdaloid area	0.594	0.038
F	CD4	left pre-para subiculum	1.198	0.050
F	CD4	left Primary auditory cortex	0.746	0.042
F	CD4	left Primary motor cortex	3.850	0.190
F	CD4	left Primary somatosensory cortex	2.089	0.113
F	CD4	left Primary somatosensory cortex: barrel field	4.994	0.259
F	CD4	left Primary somatosensory cortex: dysgranular zone	0.171	0.013
F	CD4	left Primary somatosensory cortex: forelimb region	2.169	0.132
F	CD4	left Primary somatosensory cortex: hindlimb region	1.571	0.082
F	CD4	left Primary somatosensory cortex: jaw region	0.272	0.031
F	CD4	left Primary somatosensory cortex: shoulder region	0.092	0.007
F	CD4	left Primary somatosensory cortex: trunk region	0.319	0.019
F	CD4	left Primary somatosensory cortex: upper lip region	2.454	0.104
F	CD4	left Primary visual cortex	1.191	0.090
F	CD4	left Primary visual cortex: binocular area	1.050	0.087
F	CD4	left Primary visual cortex: monocular area	1.206	0.093
F	CD4	left Rostral amygdalopiriform area	0.201	0.018
F	CD4	left Secondary auditory cortex: dorsal area	0.746	0.044
F	CD4	left Secondary auditory cortex: ventral area	0.805	0.069
F	CD4	left Secondary motor cortex	3.512	0.127
F	CD4	left Secondary somatosensory cortex	3.412	0.279
F	CD4	left Secondary visual cortex: lateral area	1.343	0.069
F	CD4	left Secondary visual cortex: mediolateral area	0.608	0.041
F	CD4	left Secondary visual cortex: mediomedial area	0.950	0.039
F	CD4	left simple lobule (lobule 6)	2.421	0.146
F	CD4	left simple lobule white matter	0.225	0.020
F	CD4	left SLu	0.381	0.020
F	CD4	left stria medullaris	0.334	0.012
F	CD4	left stria terminalis	0.489	0.017
F	CD4	left striatum	11.215	0.448
F	CD4	left subependymale zone / rhinocele	0.029	0.002
F	CD4	left subiculum	1.686	0.049
F	CD4	left superior olivary complex	0.381	0.039
F	CD4	left Temporal association area	1.397	0.073
F	CD4	left thalamus	9.203	0.357
F	CD4	left trunk of crus 2 and paramedian white matter	0.171	0.017
F	CD4	left trunk of simple and crus 1 white matter	0.066	0.003
F	CD4	left Ventral intermediate entorhinal cortex	0.575	0.031
F	CD4	left Ventral nucleus of the endopiriform claustrum	0.268	0.030
F	CD4	left Ventral orbital cortex	0.776	0.036
F	CD4	left Ventral tenia tecta	0.053	0.005
F	CD4	lobule 1-2 white matter	0.065	0.015
F	CD4	lobule 10 white matter	0.083	0.005
F	CD4	lobule 10: nodulus	1.413	0.075
F	CD4	lobule 3 white matter	0.186	0.033
F	CD4	lobule 3: central lobule (dorsal)	2.172	0.179
F	CD4	lobule 6: declive	2.467	0.177
F	CD4	lobule 7: tuber (or folium)	0.968	0.105
F	CD4	lobule 8 white matter	0.167	0.012
F	CD4	lobule 8: pyramis	1.657	0.099
F	CD4	lobule 9 white matter	0.300	0.013
F	CD4	lobule 9: uvula	2.783	0.114
F	CD4	lobules 1-2: lingula and central lobule (ventral)	1.644	0.159
F	CD4	lobules 4-5 white matter	0.592	0.031
F	CD4	lobules 4-5: culmen (ventral and dorsal)	4.392	0.191
F	CD4	lobules 6-7 white matter	0.699	0.061
F	CD4	medulla	27.804	0.799
F	CD4	midbrain	14.351	0.497
F	CD4	periaqueductal grey	4.050	0.119
F	CD4	pons	17.568	0.346
F	CD4	posterior commissure	0.148	0.006
F	CD4	right Accessory olfactory bulb: glomerular, external plexiform and mitral cell layer	0.247	0.015
F	CD4	right Accessory olfactory bulb: granule cell layer	0.114	0.008
F	CD4	right amygdala	4.705	0.166
F	CD4	right Amygdalopiriform transition area	0.495	0.026
F	CD4	right anterior commissure: pars anterior	0.493	0.020
F	CD4	right anterior commissure: pars posterior	0.208	0.007
F	CD4	right anterior lobule (lobules 4-5)	0.781	0.039
F	CD4	right anterior lobule white matter	0.050	0.004
F	CD4	right Anterior olfactory nucleus	1.001	0.055
F	CD4	right basal forebrain	2.839	0.097
F	CD4	right bed nucleus of stria terminalis	0.669	0.021
F	CD4	right CA10r	1.038	0.040
F	CD4	right CA1Py	0.567	0.019
F	CD4	right CA1Rad	1.360	0.045
F	CD4	right CA20r	0.219	0.014
F	CD4	right CA2Py	0.103	0.009

F	CD4	right CA2Rad	0.226	0.025
F	CD4	right CA30r	1.513	0.161
F	CD4	right CA3Py Inner	0.083	0.008
F	CD4	right CA3Py Outer	0.553	0.075
F	CD4	right CA3Rad	1.017	0.179
F	CD4	right Caudomedial entorhinal cortex	2.919	0.171
F	CD4	right cerebellar peduncle: inferior	0.398	0.020
F	CD4	right cerebellar peduncle: middle	0.636	0.043
F	CD4	right cerebellar peduncle: superior	0.525	0.026
F	CD4	right cerebral peduncle	1.070	0.041
F	CD4	right Cingulate cortex: area 24a	1.037	0.072
F	CD4	right Cingulate cortex: area 24a'	0.351	0.040
F	CD4	right Cingulate cortex: area 24b	0.839	0.073
F	CD4	right Cingulate cortex: area 24b'	0.310	0.027
F	CD4	right Cingulate cortex: area 25	0.316	0.019
F	CD4	right Cingulate cortex: area 29a	0.341	0.018
F	CD4	right Cingulate cortex: area 29b	0.201	0.017
F	CD4	right Cingulate cortex: area 29c	0.877	0.050
F	CD4	right Cingulate cortex: area 30	1.381	0.066
F	CD4	right Cingulate cortex: area 32	1.066	0.058
F	CD4	right Cingulum	0.465	0.019
F	CD4	right Claustrum	0.156	0.009
F	CD4	right Claustrum: dorsal part	0.152	0.021
F	CD4	right Claustrum: ventral part	0.329	0.038
F	CD4	right colliculus: inferior	2.775	0.075
F	CD4	right colliculus: superior	4.586	0.175
F	CD4	right copula white matter	0.038	0.004
F	CD4	right copula: pyramis (lobule 8)	1.317	0.126
F	CD4	right corpus callosum	5.969	0.145
F	CD4	right Cortex-amygdala transition zones	0.430	0.027
F	CD4	right corticospinal tract/pyramids	0.911	0.062
F	CD4	right crus 1 white matter	0.189	0.011
F	CD4	right crus 1: ansiform lobule (lobule 6)	2.185	0.094
F	CD4	right crus 2 white matter	0.156	0.013
F	CD4	right crus 2: ansiform lobule (lobule 7)	2.056	0.166
F	CD4	right cuneate nucleus	0.126	0.010
F	CD4	right dentate nucleus	0.180	0.008
F	CD4	right Dorsal intermediate entorhinal cortex	0.921	0.053
F	CD4	right Dorsal nucleus of the endopiriform	0.766	0.044
F	CD4	right Dorsal tenia tecta	0.461	0.030
F	CD4	right Dorsolateral entorhinal cortex	1.383	0.071
F	CD4	right Dorsolateral orbital cortex	0.393	0.023
F	CD4	right Ectorhinal cortex	1.399	0.074
F	CD4	right facial nerve (cranial nerve 7)	0.111	0.004
F	CD4	right fasciculus retroflexus	0.118	0.008
F	CD4	right fastigial nucleus	0.240	0.009
F	CD4	right fimbria	1.635	0.092
F	CD4	right flocculus (FL)	0.516	0.028
F	CD4	right flocculus white matter	0.038	0.004
F	CD4	right fornix	0.308	0.008
F	CD4	right Frontal association cortex	3.901	0.159
F	CD4	right Frontal cortex: area 3	0.364	0.018
F	CD4	right fundus of striatum	0.086	0.006
F	CD4	right globus pallidus	1.730	0.046
F	CD4	right GrDG	0.679	0.040
F	CD4	right habenular commissure	0.015	0.005
F	CD4	right hypothalamus	5.373	0.087
F	CD4	right inferior olivary complex	0.174	0.021
F	CD4	right Insular region: not subdivided	4.252	0.120
F	CD4	right Intermediate nucleus of the endopiriform claustrum	0.290	0.014
F	CD4	right internal capsule	1.581	0.042
F	CD4	right lateral olfactory tract	0.640	0.028
F	CD4	right Lateral orbital cortex	1.795	0.067
F	CD4	right Lateral parietal association cortex	0.092	0.009
F	CD4	right lateral septum	1.600	0.048
F	CD4	right lateral ventricle	2.322	0.481
F	CD4	right LMol	1.192	0.041
F	CD4	right mammillary bodies	0.234	0.023
F	CD4	right mammilothalamic tract	0.133	0.007
F	CD4	right Medial amygdala	0.536	0.025
F	CD4	right Medial entorhinal cortex	0.344	0.020
F	CD4	right medial lemniscus/medial longitudinal fasciculus	1.362	0.062
F	CD4	right Medial orbital cortex	0.841	0.036
F	CD4	right Medial parietal association cortex	0.175	0.016
F	CD4	right Medial preoptic nucleus	0.093	0.007
F	CD4	right medial septum	0.638	0.026
F	CD4	right MoDG	2.132	0.095
F	CD4	right nucleus accumbens	2.193	0.045
F	CD4	right nucleus interpositus	0.242	0.011
F	CD4	right Olfactory bulb: external plexiform layer	3.195	0.140
F	CD4	right Olfactory bulb: glomerular layer	2.221	0.096
F	CD4	right Olfactory bulb: granule cell layer	2.194	0.108
F	CD4	right Olfactory bulb: internal plexiform layer	0.482	0.030
F	CD4	right Olfactory bulb: mitral cell layer	0.587	0.030
F	CD4	right olfactory peduncle	1.149	0.027
F	CD4	right olfactory tubercle	1.668	0.113
F	CD4	right optic tract	0.756	0.021
F	CD4	right paraflocculus (PFL)	1.724	0.189
F	CD4	right paraflocculus white matter	0.168	0.016
F	CD4	right paramedian lobule	0.082	0.008
F	CD4	right paramedian lobule (lobule 7)	2.003	0.124
F	CD4	right Parietal cortex: posterior area: rostral part	0.045	0.004
F	CD4	right Perirhinal cortex	1.239	0.090
F	CD4	right Piriform cortex	5.512	0.149
F	CD4	right PoDG	0.336	0.021
F	CD4	right pontine nucleus	0.406	0.050
F	CD4	right Posterolateral cortical amygdaloid area	0.454	0.027
F	CD4	right Posteromedial cortical amygdaloid area	0.476	0.030
F	CD4	right pre-para subiculum	1.195	0.062
F	CD4	right Primary auditory cortex	0.768	0.040
F	CD4	right Primary motor cortex	3.303	0.133
F	CD4	right Primary somatosensory cortex	2.166	0.060
F	CD4	right Primary somatosensory cortex: barrel field	4.850	0.293
F	CD4	right Primary somatosensory cortex: dysgranular zone	0.186	0.014
F	CD4	right Primary somatosensory cortex: forelimb region	1.996	0.134
F	CD4	right Primary somatosensory cortex: hindlimb region	1.311	0.093
F	CD4	right Primary somatosensory cortex: jaw region	0.309	0.037
F	CD4	right Primary somatosensory cortex: shoulder region	0.067	0.007
F	CD4	right Primary somatosensory cortex: trunk region	0.197	0.016
F	CD4	right Primary somatosensory cortex: upper lip region	2.860	0.154
F	CD4	right Primary visual cortex	1.223	0.070
F	CD4	right Primary visual cortex: binocular area	1.026	0.060
F	CD4	right Primary visual cortex: monocular area	0.799	0.050
F	CD4	right Rostral amygdalopiriform area	0.208	0.015
F	CD4	right Secondary auditory cortex: dorsal area	0.738	0.041
F	CD4	right Secondary auditory cortex: ventral area	1.039	0.075
F	CD4	right Secondary motor cortex	3.389	0.170
F	CD4	right Secondary somatosensory cortex	3.811	0.141
F	CD4	right Secondary visual cortex: lateral area	1.500	0.066
F	CD4	right Secondary visual cortex: mediolateral area	0.397	0.027
F	CD4	right Secondary visual cortex: mediomedial area	0.784	0.028
F	CD4	right simple lobule (lobule 6)	2.425	0.124
F	CD4	right simple lobule white matter	0.183	0.013
F	CD4	right SLu	0.381	0.040
F	CD4	right stria medullaris	0.359	0.012
F	CD4	right stria terminalis	0.418	0.017
F	CD4	right striatum	11.158	0.496
F	CD4	right subependymale zone / rhinocele	0.026	0.002
F	CD4	right subiculum	1.643	0.043
F	CD4	right superior olivary complex	0.400	0.047
F	CD4	right Temporal association area	1.582	0.084
F	CD4	right thalamus	9.159	0.314
F	CD4	right trunk of crus 2 and paramedian white matter	0.177	0.013
F	CD4	right trunk of simple and crus 1 white matter	0.084	0.006
F	CD4	right Ventral intermediate entorhinal cortex	0.546	0.028
F	CD4	right Ventral nucleus of the endopiriform claustrum	0.240	0.027
F	CD4	right Ventral orbital cortex	0.677	0.031
F	CD4	right Ventral tenia tecta	0.055	0.005
F	CD4	third ventricle	1.352	0.142
F	CD4	trunk of arbor vita	4.307	0.115
F	CD4	trunk of lobules 1-3 white matter	0.129	0.015
F	CD4	trunk of lobules 6-8 white matter	0.114	0.006
F	CD4	ventral tegmental decussation	0.134	0.009
F	CD8	cerebral aqueduct	0.463	0.030
F	CD8	fourth ventricle	0.883	0.046
F	CD8	interpeduncular nucleus	0.265	0.016
F	CD8	left Accessory olfactory bulb: glomerular, external plexiform and mitral cell layer	0.245	0.011
F	CD8	left Accessory olfactory bulb: granule cell layer	0.116	0.006
F	CD8	left amygdala	4.585	0.193
F	CD8	left Amygdalopiriform transition area	0.509	0.035
F	CD8	left anterior commissure: pars anterior	0.470	0.029
F	CD8	left anterior commissure: pars posterior	0.204	0.014
F	CD8	left anterior lobule (lobules 4-5)	0.824	0.078
F	CD8	left anterior lobule white matter	0.041	0.004
F	CD8	left Anterior olfactory nucleus	0.956	0.056
F	CD8	left basal forebrain	2.454	0.115
F	CD8	left bed nucleus of stria terminalis	0.620	0.036
F	CD8	left CA10r	1.139	0.058
F	CD8	left CA1Py	0.544	0.025
F	CD8	left CA1Rad	1.220	0.045
F	CD8	left CA20r	0.257	0.012
F	CD8	left CA2Py	0.100	0.005
F	CD8	left CA2Rad	0.202	0.012
F	CD8	left CA30r	1.324	0.066
F	CD8	left CA3Py Inner	0.073	0.004
F	CD8	left CA3Py Outer	0.493	0.023
F	CD8	left CA3Rad	0.877	0.038
F	CD8	left Caudomedial entorhinal cortex	2.836	0.137
F	CD8	left cerebellar peduncle: inferior	0.397	0.024
F	CD8	left cerebellar peduncle: middle	0.557	0.044
F	CD8	left cerebellar peduncle: superior	0.474	0.031
F	CD8	left cerebral peduncle	0.960	0.047
F	CD8	left Cingulate cortex: area 24a	0.956	0.045
F	CD8	left Cingulate cortex: area 24a'	0.378	0.046
F	CD8	left Cingulate cortex: area 24b	0.806	0.048
F	CD8	left Cingulate cortex: area 24b'	0.308	0.022
F	CD8	left Cingulate cortex: area 25	0.289	0.016
F	CD8	left Cingulate cortex: area 29a	0.360	0.029
F	CD8	left Cingulate cortex: area 29b	0.205	0.018
F	CD8	left Cingulate cortex: area 29c	0.925	0.077
F	CD8	left Cingulate cortex: area 30	1.477	0.100
F	CD8	left Cingulate cortex: area 32	1.326	0.091
F	CD8	left Cingulum	0.412	0.028
F	CD8	left Claustrum	0.174	0.013
F	CD8	left Claustrum: dorsal part	0.149	0.015
F	CD8	left Claustrum: ventral part	0.267	0.027
F	CD8	left colliculus: inferior	2.670	0.154
F	CD8	left colliculus: superior	4.036	0.297
F	CD8	left copula white matter	0.042	0.003
F	CD8	left copula: pyramis (lobule 8)	1.108	0.100
F	CD8	left corpus callosum	5.859	0.404
F	CD8	left Cortex-amygdala transition zones	0.382	0.034
F	CD8	left corticospinal tract/pyramids	0.851	0.047
F	CD8	left crus 1 white matter	0.175	0.015
F	CD8	left crus 1: ansiform lobule (lobule 6)	2.051	0.138
F	CD8	left crus 2 white matter	0.121	0.010
F	CD8	left crus 2: ansiform lobule (lobule 7)	1.860	0.092
F	CD8	left cuneate nucleus	0.115	0.012
F	CD8	left dentate nucleus	0.169	0.010
F	CD8	left Dorsal intermediate entorhinal cortex	0.820	0.052
F	CD8	left Dorsal nucleus of the endopiriform	0.708	0.036
F	CD8	left Dorsal tenia tecta	0.471	0.025
F	CD8	left Dorsolateral entorhinal cortex	1.116	0.071
F	CD8	left Dorsolateral orbital cortex	0.420	0.031
F	CD8	left Ectorhinal cortex	1.269	0.111
F	CD8	left facial nerve (cranial nerve 7)	0.100	0.007
F	CD8	left fasciculus retroflexus	0.123	0.008
F	CD8	left fastigial nucleus	0.225	0.017
F	CD8	left fimbria	1.557	0.112
F	CD8	left flocculus (FL)	0.527	0.044
F	CD8	left flocculus white matter	0.028	0.003
F	CD8	left fornix	0.316	0.022
F	CD8	left Frontal association cortex	3.532	0.160
F	CD8	left Frontal cortex: area 3	0.360	0.021
F	CD8	left fundus of striatum	0.070	0.007
F	CD8	left globus pallidus	1.542	0.091
F	CD8	left GrDG	0.649	0.027
F	CD8	left habenular commissure	0.013	0.002
F	CD8	left hypothalamus	4.915	0.215
F	CD8	left inferior olivary complex	0.158	0.019
F	CD8	left Insular region: not subdivided	3.770	0.264
F	CD8	left Intermediate nucleus of the endopiriform claustrum	0.299	0.016
F	CD8	left internal capsule	1.357	0.110
F	CD8	left lateral olfactory tract	0.706	0.030
F	CD8	left Lateral orbital cortex	1.774	0.088
F	CD8	left Lateral parietal association cortex	0.158	0.016
F	CD8	left lateral septum	1.444	0.074
F	CD8	left lateral ventricle	1.581	0.133
F	CD8	left LMol	1.164	0.044
F	CD8	left mammillary bodies	0.237	0.018
F	CD8	left mammilothalamic tract	0.119	0.008
F	CD8	left Medial amygdala	0.474	0.026
F	CD8	left Medial entorhinal cortex	0.347	0.026
F	CD8	left medial lemniscus/medial longitudinal fasciculus	1.194	0.067
F	CD8	left Medial orbital cortex	0.892	0.064
F	CD8	left Medial parietal association cortex	0.242	0.032
F	CD8	left Medial preoptic nucleus	0.093	0.006
F	CD8	left medial septum	0.619	0.035
F	CD8	left MoDG	2.089	0.086
F	CD8	left nucleus accumbens	2.024	0.087
F	CD8	left nucleus interpositus	0.212	0.015
F	CD8	left Olfactory bulb: external plexiform layer	3.105	0.140
F	CD8	left Olfactory bulb: glomerular layer	2.091	0.102
F	CD8	left Olfactory bulb: granule cell layer	2.128	0.130
F	CD8	left Olfactory bulb: internal plexiform layer	0.487	0.032
F	CD8	left Olfactory bulb: mitral cell layer	0.616	0.035
F	CD8	left olfactory peduncle	1.123	0.059
F	CD8	left olfactory tubercle	1.557	0.064
F	CD8	left optic tract	0.720	0.065
F	CD8	left paraflocculus (PFL)	1.631	0.251
F	CD8	left paraflocculus white matter	0.134	0.017
F	CD8	left paramedian lobule	0.072	0.006
F	CD8	left paramedian lobule (lobule 7)	1.953	0.148
F	CD8	left Parietal cortex: posterior area: rostral part	0.070	0.006
F	CD8	left Perirhinal cortex	1.199	0.099
F	CD8	left Piriform cortex	4.947	0.270
F	CD8	left PoDG	0.288	0.012
F	CD8	left pontine nucleus	0.380	0.039
F	CD8	left Posterolateral cortical amygdaloid area	0.389	0.029
F	CD8	left Posteromedial cortical amygdaloid area	0.545	0.024
F	CD8	left pre-para subiculum	1.110	0.055
F	CD8	left Primary auditory cortex	0.718	0.066
F	CD8	left Primary motor cortex	3.667	0.115
F	CD8	left Primary somatosensory cortex	2.034	0.111
F	CD8	left Primary somatosensory cortex: barrel field	4.630	0.295
F	CD8	left Primary somatosensory cortex: dysgranular zone	0.170	0.012
F	CD8	left Primary somatosensory cortex: forelimb region	2.051	0.132

F	CD8	left Primary somatosensory cortex: hindlimb region	1.511	0.092
F	CD8	left Primary somatosensory cortex: jaw region	0.272	0.024
F	CD8	left Primary somatosensory cortex: shoulder region	0.092	0.008
F	CD8	left Primary somatosensory cortex: trunk region	0.313	0.024
F	CD8	left Primary somatosensory cortex: upper lip region	2.314	0.148
F	CD8	left Primary visual cortex	1.194	0.087
F	CD8	left Primary visual cortex: binocular area	1.006	0.069
F	CD8	left Primary visual cortex: monocular area	1.162	0.051
F	CD8	left Rostral amygdalopiriform area	0.197	0.016
F	CD8	left Secondary auditory cortex: dorsal area	0.726	0.046
F	CD8	left Secondary auditory cortex: ventral area	0.777	0.092
F	CD8	left Secondary motor cortex	3.393	0.111
F	CD8	left Secondary somatosensory cortex	3.406	0.277
F	CD8	left Secondary visual cortex: lateral area	1.289	0.075
F	CD8	left Secondary visual cortex: mediolateral area	0.586	0.040
F	CD8	left Secondary visual cortex: mediomedial area	0.939	0.079
F	CD8	left simple lobule (lobule 6)	2.275	0.120
F	CD8	left simple lobule white matter	0.204	0.015
F	CD8	left SLu	0.362	0.018
F	CD8	left stria medullaris	0.310	0.017
F	CD8	left stria terminalis	0.439	0.032
F	CD8	left striatum	10.367	0.487
F	CD8	left subependymale zone / rhinocele	0.027	0.002
F	CD8	left subiculum	1.608	0.083
F	CD8	left superior olivary complex	0.361	0.028
F	CD8	left Temporal association area	1.318	0.130
F	CD8	left thalamus	8.590	0.488
F	CD8	left trunk of crus 2 and paramedian white matter	0.152	0.012
F	CD8	left trunk of simple and crus 1 white matter	0.064	0.003
F	CD8	left Ventral intermediate entorhinal cortex	0.541	0.035
F	CD8	left Ventral nucleus of the endopiriform claustrum	0.247	0.026
F	CD8	left Ventral orbital cortex	0.746	0.048
F	CD8	left Ventral tenia tecta	0.054	0.003
F	CD8	lobule 1-2 white matter	0.069	0.017
F	CD8	lobule 10 white matter	0.081	0.008
F	CD8	lobule 10: nodulus	1.337	0.092
F	CD8	lobule 3 white matter	0.169	0.022
F	CD8	lobule 3: central lobule (dorsal)	1.968	0.217
F	CD8	lobule 6: declive	2.402	0.162
F	CD8	lobule 7: tuber (or folium)	0.908	0.084
F	CD8	lobule 8 white matter	0.144	0.011
F	CD8	lobule 8: pyramis	1.480	0.108
F	CD8	lobule 9 white matter	0.269	0.025
F	CD8	lobule 9: uvula	2.592	0.193
F	CD8	lobules 1-2: lingula and central lobule (ventral)	1.627	0.223
F	CD8	lobules 4-5 white matter	0.546	0.030
F	CD8	lobules 4-5: culmen (ventral and dorsal)	4.230	0.280
F	CD8	lobules 6-7 white matter	0.665	0.051
F	CD8	medulla	25.874	1.380
F	CD8	midbrain	12.775	0.752
F	CD8	periaqueductal grey	3.699	0.185
F	CD8	pons	16.042	0.824
F	CD8	posterior commissure	0.133	0.012
F	CD8	right Accessory olfactory bulb: glomerular, external plexiform and mitral cell layer	0.236	0.013
F	CD8	right Accessory olfactory bulb: granule cell layer	0.111	0.008
F	CD8	right amygdala	4.328	0.218
F	CD8	right Amygdalopiriform transition area	0.491	0.031
F	CD8	right anterior commissure: pars anterior	0.469	0.023
F	CD8	right anterior commissure: pars posterior	0.193	0.012
F	CD8	right anterior lobule (lobules 4-5)	0.750	0.056
F	CD8	right anterior lobule white matter	0.048	0.005
F	CD8	right Anterior olfactory nucleus	0.978	0.044
F	CD8	right basal forebrain	2.554	0.123
F	CD8	right bed nucleus of stria terminalis	0.612	0.033
F	CD8	right CA10r	0.998	0.061
F	CD8	right CA1Py	0.548	0.028
F	CD8	right CA1Rad	1.327	0.043
F	CD8	right CA20r	0.212	0.011
F	CD8	right CA2Py	0.096	0.005
F	CD8	right CA2Rad	0.210	0.011
F	CD8	right CA30r	1.332	0.059
F	CD8	right CA3Py Inner	0.076	0.004
F	CD8	right CA3Py Outer	0.488	0.021
F	CD8	right CA3Rad	0.911	0.036
F	CD8	right Caudomedial entorhinal cortex	2.767	0.153
F	CD8	right cerebellar peduncle: inferior	0.369	0.021
F	CD8	right cerebellar peduncle: middle	0.588	0.046
F	CD8	right cerebellar peduncle: superior	0.463	0.032
F	CD8	right cerebral peduncle	0.974	0.052
F	CD8	right Cingulate cortex: area 24a	1.030	0.047
F	CD8	right Cingulate cortex: area 24a'	0.360	0.045
F	CD8	right Cingulate cortex: area 24b	0.774	0.047
F	CD8	right Cingulate cortex: area 24b'	0.291	0.021
F	CD8	right Cingulate cortex: area 25	0.292	0.017
F	CD8	right Cingulate cortex: area 29a	0.342	0.023
F	CD8	right Cingulate cortex: area 29b	0.195	0.021
F	CD8	right Cingulate cortex: area 29c	0.881	0.057
F	CD8	right Cingulate cortex: area 30	1.354	0.106
F	CD8	right Cingulate cortex: area 32	1.054	0.084
F	CD8	right Cingulum	0.421	0.030
F	CD8	right Claustrum	0.143	0.007
F	CD8	right Claustrum: dorsal part	0.142	0.010
F	CD8	right Claustrum: ventral part	0.301	0.021
F	CD8	right colliculus: inferior	2.565	0.140
F	CD8	right colliculus: superior	4.113	0.213
F	CD8	right copula white matter	0.033	0.003
F	CD8	right copula: pyramis (lobule 8)	1.224	0.110
F	CD8	right corpus callosum	5.538	0.308
F	CD8	right Cortex-amygdala transition zones	0.426	0.043
F	CD8	right corticospinal tract/pyramids	0.900	0.048
F	CD8	right crus 1 white matter	0.170	0.012
F	CD8	right crus 1: ansiform lobule (lobule 6)	2.086	0.127
F	CD8	right crus 2 white matter	0.141	0.007
F	CD8	right crus 2: ansiform lobule (lobule 7)	1.912	0.095
F	CD8	right cuneate nucleus	0.119	0.015
F	CD8	right dentate nucleus	0.167	0.012
F	CD8	right Dorsal intermediate entorhinal cortex	0.876	0.045
F	CD8	right Dorsal nucleus of the endopiriform	0.689	0.034
F	CD8	right Dorsal tenia tecta	0.436	0.020
F	CD8	right Dorsolateral entorhinal cortex	1.272	0.125
F	CD8	right Dorsolateral orbital cortex	0.398	0.038
F	CD8	right Ectorhinal cortex	1.327	0.119
F	CD8	right facial nerve (cranial nerve 7)	0.102	0.009
F	CD8	right fasciculus retroflexus	0.109	0.008
F	CD8	right fastigial nucleus	0.216	0.017
F	CD8	right fimbria	1.427	0.097
F	CD8	right flocculus (FL)	0.499	0.040
F	CD8	right flocculus white matter	0.036	0.003
F	CD8	right fornix	0.280	0.019
F	CD8	right Frontal association cortex	3.878	0.146
F	CD8	right Frontal cortex: area 3	0.353	0.028
F	CD8	right fundus of striatum	0.076	0.005
F	CD8	right globus pallidus	1.559	0.082
F	CD8	right GrDG	0.605	0.025
F	CD8	right habenular commissure	0.013	0.002
F	CD8	right hypothalamus	4.878	0.216
F	CD8	right inferior olivary complex	0.172	0.018
F	CD8	right Insular region: not subdivided	4.033	0.263
F	CD8	right Intermediate nucleus of the endopiriform claustrum	0.275	0.017
F	CD8	right internal capsule	1.446	0.091
F	CD8	right lateral olfactory tract	0.626	0.039
F	CD8	right Lateral orbital cortex	1.742	0.076
F	CD8	right Lateral parietal association cortex	0.088	0.011
F	CD8	right lateral septum	1.444	0.076
F	CD8	right lateral ventricle	1.645	0.148
F	CD8	right LMol	1.145	0.039
F	CD8	right mammillary bodies	0.217	0.018
F	CD8	right mammilothalamic tract	0.124	0.007
F	CD8	right Medial amygdala	0.503	0.027
F	CD8	right Medial entorhinal cortex	0.332	0.024
F	CD8	right medial lemniscus/medial longitudinal fasciculus	1.273	0.052
F	CD8	right Medial orbital cortex	0.809	0.058
F	CD8	right Medial parietal association cortex	0.170	0.022
F	CD8	right Medial preoptic nucleus	0.086	0.006
F	CD8	right medial septum	0.580	0.028
F	CD8	right MoDG	1.952	0.073
F	CD8	right nucleus accumbens	2.018	0.092
F	CD8	right nucleus interpositus	0.222	0.017
F	CD8	right Olfactory bulb: external plexiform layer	3.086	0.145
F	CD8	right Olfactory bulb: glomerular layer	2.140	0.119
F	CD8	right Olfactory bulb: granule cell layer	2.197	0.143
F	CD8	right Olfactory bulb: internal plexiform layer	0.484	0.028
F	CD8	right Olfactory bulb: mitral cell layer	0.585	0.031
F	CD8	right olfactory peduncle	1.104	0.056
F	CD8	right olfactory tubercle	1.577	0.090
F	CD8	right optic tract	0.682	0.040
F	CD8	right paraflocculus (PFL)	1.557	0.240
F	CD8	right paraflocculus white matter	0.151	0.022
F	CD8	right paramedian lobule	0.079	0.007
F	CD8	right paramedian lobule (lobule 7)	1.975	0.135
F	CD8	right Parietal cortex: posterior area: rostral part	0.044	0.007
F	CD8	right Perirhinal cortex	1.182	0.110
F	CD8	right Piriform cortex	5.225	0.344
F	CD8	right PoDG	0.302	0.012
F	CD8	right pontine nucleus	0.370	0.037
F	CD8	right Posterolateral cortical amygdaloid area	0.430	0.039
F	CD8	right Posteromedial cortical amygdaloid area	0.441	0.023
F	CD8	right pre-para subiculum	1.140	0.052
F	CD8	right Primary auditory cortex	0.734	0.048
F	CD8	right Primary motor cortex	3.110	0.119
F	CD8	right Primary somatosensory cortex	2.077	0.144
F	CD8	right Primary somatosensory cortex: barrel field	4.565	0.172
F	CD8	right Primary somatosensory cortex: dysgranular zone	0.177	0.008
F	CD8	right Primary somatosensory cortex: forelimb region	1.789	0.079
F	CD8	right Primary somatosensory cortex: hindlimb region	1.229	0.078
F	CD8	right Primary somatosensory cortex: jaw region	0.282	0.020
F	CD8	right Primary somatosensory cortex: shoulder region	0.064	0.009
F	CD8	right Primary somatosensory cortex: trunk region	0.193	0.024
F	CD8	right Primary somatosensory cortex: upper lip region	2.752	0.152
F	CD8	right Primary visual cortex	1.219	0.103
F	CD8	right Primary visual cortex: binocular area	1.003	0.045
F	CD8	right Primary visual cortex: monocular area	0.769	0.037
F	CD8	right Rostral amygdalopiriform area	0.205	0.017
F	CD8	right Secondary auditory cortex: dorsal area	0.714	0.044
F	CD8	right Secondary auditory cortex: ventral area	0.969	0.072
F	CD8	right Secondary motor cortex	3.238	0.156
F	CD8	right Secondary somatosensory cortex	3.732	0.285
F	CD8	right Secondary visual cortex: lateral area	1.466	0.076
F	CD8	right Secondary visual cortex: mediolateral area	0.381	0.036
F	CD8	right Secondary visual cortex: mediomedial area	0.769	0.070
F	CD8	right simple lobule (lobule 6)	2.277	0.125
F	CD8	right simple lobule white matter	0.165	0.012
F	CD8	right SLu	0.358	0.014
F	CD8	right stria medullaris	0.327	0.016
F	CD8	right stria terminalis	0.367	0.024
F	CD8	right striatum	10.240	0.518
F	CD8	right subependymale zone / rhinocele	0.025	0.002
F	CD8	right subiculum	1.596	0.075
F	CD8	right superior olivary complex	0.378	0.031
F	CD8	right Temporal association area	1.529	0.110
F	CD8	right thalamus	8.498	0.474
F	CD8	right trunk of crus 2 and paramedian white matter	0.162	0.013
F	CD8	right trunk of simple and crus 1 white matter	0.078	0.006
F	CD8	right Ventral intermediate entorhinal cortex	0.537	0.024
F	CD8	right Ventral nucleus of the endopiriform claustrum	0.226	0.017
F	CD8	right Ventral orbital cortex	0.685	0.038
F	CD8	right Ventral tenia tecta	0.055	0.005
F	CD8	third ventricle	1.170	0.072
F	CD8	trunk of arbor vita	3.876	0.212
F	CD8	trunk of lobules 1-3 white matter	0.120	0.011
F	CD8	trunk of lobules 6-8 white matter	0.102	0.009
F	CD8	ventral tegmental decussation	0.118	0.011
F	Cxcr2	cerebral aqueduct	0.524	0.078
F	Cxcr2	fourth ventricle	0.928	0.078
F	Cxcr2	interpeduncular nucleus	0.268	0.019
F	Cxcr2	left Accessory olfactory bulb: glomerular, external plexiform and mitral cell layer	0.251	0.015
F	Cxcr2	left Accessory olfactory bulb: granule cell layer	0.123	0.007
F	Cxcr2	left amygdala	4.771	0.223
F	Cxcr2	left Amygdalopiriform transition area	0.532	0.030
F	Cxcr2	left anterior commissure: pars anterior	0.479	0.024
F	Cxcr2	left anterior commissure: pars posterior	0.207	0.012
F	Cxcr2	left anterior lobule (lobules 4-5)	0.844	0.062
F	Cxcr2	left anterior lobule white matter	0.041	0.003
F	Cxcr2	left Anterior olfactory nucleus	0.948	0.044
F	Cxcr2	left basal forebrain	2.549	0.116
F	Cxcr2	left bed nucleus of stria terminalis	0.645	0.031
F	Cxcr2	left CA10r	1.116	0.048
F	Cxcr2	left CA1Py	0.531	0.025
F	Cxcr2	left CA1Rad	1.202	0.041
F	Cxcr2	left CA20r	0.251	0.008
F	Cxcr2	left CA2Py	0.099	0.004
F	Cxcr2	left CA2Rad	0.201	0.007
F	Cxcr2	left CA30r	1.383	0.068
F	Cxcr2	left CA3Py Inner	0.074	0.002
F	Cxcr2	left CA3Py Outer	0.502	0.023
F	Cxcr2	left CA3Rad	0.887	0.043
F	Cxcr2	left Caudomedial entorhinal cortex	2.881	0.118
F	Cxcr2	left cerebellar peduncle: inferior	0.411	0.017
F	Cxcr2	left cerebellar peduncle: middle	0.584	0.032
F	Cxcr2	left cerebellar peduncle: superior	0.505	0.037
F	Cxcr2	left cerebral peduncle	1.012	0.042
F	Cxcr2	left Cingulate cortex: area 24a	0.935	0.030
F	Cxcr2	left Cingulate cortex: area 24a'	0.361	0.029
F	Cxcr2	left Cingulate cortex: area 24b	0.787	0.073
F	Cxcr2	left Cingulate cortex: area 24b'	0.308	0.026
F	Cxcr2	left Cingulate cortex: area 25	0.291	0.022
F	Cxcr2	left Cingulate cortex: area 29a	0.382	0.014
F	Cxcr2	left Cingulate cortex: area 29b	0.213	0.019
F	Cxcr2	left Cingulate cortex: area 29c	0.944	0.044
F	Cxcr2	left Cingulate cortex: area 30	1.473	0.057
F	Cxcr2	left Cingulate cortex: area 32	1.317	0.094
F	Cxcr2	left Cingulum	0.430	0.025
F	Cxcr2	left Claustrum	0.182	0.015
F	Cxcr2	left Claustrum: dorsal part	0.150	0.024
F	Cxcr2	left Claustrum: ventral part	0.271	0.029
F	Cxcr2	left colliculus: inferior	2.780	0.110
F	Cxcr2	left colliculus: superior	4.275	0.203
F	Cxcr2	left copula white matter	0.042	0.003
F	Cxcr2	left copula: pyramis (lobule 8)	1.136	0.078
F	Cxcr2	left corpus callosum	5.962	0.275
F	Cxcr2	left Cortex-amygdala transition zones	0.398	0.028
F	Cxcr2	left corticospinal tract/pyramids	0.850	0.040
F	Cxcr2	left crus 1 white matter	0.184	0.010
F	Cxcr2	left crus 1: ansiform lobule (lobule 6)	2.105	0.100
F	Cxcr2	left crus 2 white matter	0.132	0.010
F	Cxcr2	left crus 2: ansiform lobule (lobule 7)	1.984	0.088
F	Cxcr2	left cuneate nucleus	0.120	0.011

F	Cxcr2	left dentate nucleus	0.185	0.010
F	Cxcr2	left Dorsal intermediate entorhinal cortex	0.864	0.042
F	Cxcr2	left Dorsal nucleus of the endopiriform	0.734	0.039
F	Cxcr2	left Dorsal tenia tecta	0.488	0.030
F	Cxcr2	left Dorsolateral entorhinal cortex	1.167	0.072
F	Cxcr2	left Dorsolateral orbital cortex	0.446	0.030
F	Cxcr2	left Ectorhinal cortex	1.284	0.081
F	Cxcr2	left facial nerve (cranial nerve 7)	0.104	0.005
F	Cxcr2	left fasciculus retroflexus	0.127	0.007
F	Cxcr2	left fastigial nucleus	0.242	0.014
F	Cxcr2	left fimbria	1.603	0.080
F	Cxcr2	left flocculus (FL)	0.550	0.037
F	Cxcr2	left flocculus white matter	0.030	0.003
F	Cxcr2	left fornix	0.325	0.017
F	Cxcr2	left Frontal association cortex	3.481	0.141
F	Cxcr2	left Frontal cortex: area 3	0.354	0.022
F	Cxcr2	left fundus of striatum	0.073	0.005
F	Cxcr2	left globus pallidus	1.593	0.067
F	Cxcr2	left GrDG	0.638	0.023
F	Cxcr2	left habenular commissure	0.015	0.003
F	Cxcr2	left hypothalamus	5.120	0.180
F	Cxcr2	left inferior olivary complex	0.156	0.018
F	Cxcr2	left Insular region: not subdivided	3.799	0.230
F	Cxcr2	left Intermediate nucleus of the endopiriform claustrum	0.308	0.015
F	Cxcr2	left internal capsule	1.413	0.068
F	Cxcr2	left lateral olfactory tract	0.722	0.029
F	Cxcr2	left Lateral orbital cortex	1.811	0.091
F	Cxcr2	left Lateral parietal association cortex	0.151	0.016
F	Cxcr2	left lateral septum	1.476	0.060
F	Cxcr2	left lateral ventricle	1.730	0.205
F	Cxcr2	left LMol	1.148	0.034
F	Cxcr2	left mammillary bodies	0.244	0.017
F	Cxcr2	left mammilothalamic tract	0.121	0.008
F	Cxcr2	left Medial amygdala	0.496	0.034
F	Cxcr2	left Medial entorhinal cortex	0.351	0.020
F	Cxcr2	left medial lemniscus/medial longitudinal fasciculus	1.231	0.055
F	Cxcr2	left Medial orbital cortex	0.915	0.040
F	Cxcr2	left Medial parietal association cortex	0.238	0.020
F	Cxcr2	left Medial preoptic nucleus	0.094	0.007
F	Cxcr2	left medial septum	0.652	0.035
F	Cxcr2	left MoDG	2.068	0.071
F	Cxcr2	left nucleus accumbens	2.009	0.072
F	Cxcr2	left nucleus interpositus	0.226	0.014
F	Cxcr2	left Olfactory bulb: external plexiform layer	3.118	0.106
F	Cxcr2	left Olfactory bulb: glomerular layer	2.120	0.079
F	Cxcr2	left Olfactory bulb: granule cell layer	2.177	0.123
F	Cxcr2	left Olfactory bulb: internal plexiform layer	0.496	0.028
F	Cxcr2	left Olfactory bulb: mitral cell layer	0.624	0.029
F	Cxcr2	left olfactory peduncle	1.141	0.043
F	Cxcr2	left olfactory tubercle	1.602	0.122
F	Cxcr2	left optic tract	0.751	0.037
F	Cxcr2	left paraflocculus (PFL)	1.700	0.228
F	Cxcr2	left paraflocculus white matter	0.146	0.013
F	Cxcr2	left paramedian lobule	0.073	0.006
F	Cxcr2	left paramedian lobule (lobule 7)	2.043	0.102
F	Cxcr2	left Parietal cortex: posterior area: rostral part	0.069	0.006
F	Cxcr2	left Perirhinal cortex	1.192	0.079
F	Cxcr2	left Piriform cortex	5.018	0.211
F	Cxcr2	left PoDG	0.283	0.011
F	Cxcr2	left pontine nucleus	0.405	0.050
F	Cxcr2	left Posterolateral cortical amygdaloid area	0.401	0.027
F	Cxcr2	left Posteromedial cortical amygdaloid area	0.570	0.026
F	Cxcr2	left pre-para subiculum	1.123	0.049
F	Cxcr2	left Primary auditory cortex	0.731	0.044
F	Cxcr2	left Primary motor cortex	3.718	0.163
F	Cxcr2	left Primary somatosensory cortex	2.013	0.104
F	Cxcr2	left Primary somatosensory cortex: barrel field	4.662	0.227
F	Cxcr2	left Primary somatosensory cortex: dysgranular zone	0.171	0.012
F	Cxcr2	left Primary somatosensory cortex: forelimb region	2.052	0.134
F	Cxcr2	left Primary somatosensory cortex: hindlimb region	1.498	0.088
F	Cxcr2	left Primary somatosensory cortex: jaw region	0.278	0.026
F	Cxcr2	left Primary somatosensory cortex: shoulder region	0.091	0.009
F	Cxcr2	left Primary somatosensory cortex: trunk region	0.301	0.020
F	Cxcr2	left Primary somatosensory cortex: upper lip region	2.338	0.154
F	Cxcr2	left Primary visual cortex	1.157	0.059
F	Cxcr2	left Primary visual cortex: binocular area	1.045	0.054
F	Cxcr2	left Primary visual cortex: monocular area	1.217	0.074
F	Cxcr2	left Rostral amygdalopiriform area	0.188	0.017
F	Cxcr2	left Secondary auditory cortex: dorsal area	0.769	0.041
F	Cxcr2	left Secondary auditory cortex: ventral area	0.782	0.077
F	Cxcr2	left Secondary motor cortex	3.379	0.179
F	Cxcr2	left Secondary somatosensory cortex	3.367	0.288
F	Cxcr2	left Secondary visual cortex: lateral area	1.299	0.059
F	Cxcr2	left Secondary visual cortex: mediolateral area	0.600	0.064
F	Cxcr2	left Secondary visual cortex: mediomedial area	0.950	0.060
F	Cxcr2	left simple lobule (lobule 6)	2.252	0.088
F	Cxcr2	left simple lobule white matter	0.204	0.014
F	Cxcr2	left SLu	0.368	0.019
F	Cxcr2	left stria medullaris	0.322	0.015
F	Cxcr2	left stria terminalis	0.461	0.020
F	Cxcr2	left striatum	10.469	0.512
F	Cxcr2	left subependymale zone / rhinocele	0.028	0.002
F	Cxcr2	left subiculum	1.599	0.063
F	Cxcr2	left superior olivary complex	0.365	0.028
F	Cxcr2	left Temporal association area	1.352	0.085
F	Cxcr2	left thalamus	8.708	0.390
F	Cxcr2	left trunk of crus 2 and paramedian white matter	0.163	0.011
F	Cxcr2	left trunk of simple and crus 1 white matter	0.067	0.004
F	Cxcr2	left Ventral intermediate entorhinal cortex	0.554	0.025
F	Cxcr2	left Ventral nucleus of the endopiriform claustrum	0.253	0.018
F	Cxcr2	left Ventral orbital cortex	0.759	0.030
F	Cxcr2	left Ventral tenia tecta	0.054	0.003
F	Cxcr2	lobule 1-2 white matter	0.061	0.017
F	Cxcr2	lobule 10 white matter	0.083	0.006
F	Cxcr2	lobule 10: nodulus	1.355	0.058
F	Cxcr2	lobule 3 white matter	0.162	0.034
F	Cxcr2	lobule 3: central lobule (dorsal)	1.962	0.211
F	Cxcr2	lobule 6: declive	2.409	0.145
F	Cxcr2	lobule 7: tuber (or folium)	0.951	0.096
F	Cxcr2	lobule 8 white matter	0.153	0.012
F	Cxcr2	lobule 8: pyramis	1.580	0.133
F	Cxcr2	lobule 9 white matter	0.269	0.011
F	Cxcr2	lobule 9: uvula	2.608	0.093
F	Cxcr2	lobules 1-2: lingula and central lobule (ventral)	1.627	0.183
F	Cxcr2	lobules 4-5 white matter	0.560	0.042
F	Cxcr2	lobules 4-5: culmen (ventral and dorsal)	4.182	0.248
F	Cxcr2	lobules 6-7 white matter	0.680	0.051
F	Cxcr2	medulla	26.767	0.760
F	Cxcr2	midbrain	13.536	0.685
F	Cxcr2	periaqueductal grey	3.906	0.165
F	Cxcr2	pons	16.926	0.712
F	Cxcr2	posterior commissure	0.141	0.009
F	Cxcr2	right Accessory olfactory bulb: glomerular, external plexiform and mitral cell layer	0.241	0.014
F	Cxcr2	right Accessory olfactory bulb: granule cell layer	0.114	0.007
F	Cxcr2	right amygdala	4.441	0.191
F	Cxcr2	right Amygdalopiriform transition area	0.499	0.029
F	Cxcr2	right anterior commissure: pars anterior	0.468	0.016
F	Cxcr2	right anterior commissure: pars posterior	0.194	0.011
F	Cxcr2	right anterior lobule (lobules 4-5)	0.757	0.074
F	Cxcr2	right anterior lobule white matter	0.048	0.006
F	Cxcr2	right Anterior olfactory nucleus	0.969	0.054
F	Cxcr2	right basal forebrain	2.631	0.074
F	Cxcr2	right bed nucleus of stria terminalis	0.635	0.031
F	Cxcr2	right CA10r	0.979	0.056
F	Cxcr2	right CA1Py	0.533	0.028
F	Cxcr2	right CA1Rad	1.290	0.052
F	Cxcr2	right CA20r	0.207	0.014
F	Cxcr2	right CA2Py	0.093	0.004
F	Cxcr2	right CA2Rad	0.211	0.011
F	Cxcr2	right CA30r	1.377	0.068
F	Cxcr2	right CA3Py Inner	0.075	0.003
F	Cxcr2	right CA3Py Outer	0.502	0.022
F	Cxcr2	right CA3Rad	0.915	0.036
F	Cxcr2	right Caudomedial entorhinal cortex	2.794	0.135
F	Cxcr2	right cerebellar peduncle: inferior	0.379	0.013
F	Cxcr2	right cerebellar peduncle: middle	0.614	0.034
F	Cxcr2	right cerebellar peduncle: superior	0.485	0.033
F	Cxcr2	right cerebral peduncle	1.013	0.047
F	Cxcr2	right Cingulate cortex: area 24a	0.991	0.050
F	Cxcr2	right Cingulate cortex: area 24a'	0.349	0.032
F	Cxcr2	right Cingulate cortex: area 24b	0.750	0.070
F	Cxcr2	right Cingulate cortex: area 24b'	0.292	0.022
F	Cxcr2	right Cingulate cortex: area 25	0.297	0.022
F	Cxcr2	right Cingulate cortex: area 29a	0.361	0.022
F	Cxcr2	right Cingulate cortex: area 29b	0.203	0.018
F	Cxcr2	right Cingulate cortex: area 29c	0.882	0.039
F	Cxcr2	right Cingulate cortex: area 30	1.376	0.052
F	Cxcr2	right Cingulate cortex: area 32	1.062	0.073
F	Cxcr2	right Cingulum	0.433	0.025
F	Cxcr2	right Claustrum	0.144	0.011
F	Cxcr2	right Claustrum: dorsal part	0.137	0.018
F	Cxcr2	right Claustrum: ventral part	0.291	0.036
F	Cxcr2	right colliculus: inferior	2.665	0.101
F	Cxcr2	right colliculus: superior	4.319	0.202
F	Cxcr2	right copula white matter	0.033	0.003
F	Cxcr2	right copula: pyramis (lobule 8)	1.265	0.099
F	Cxcr2	right corpus callosum	5.667	0.202
F	Cxcr2	right Cortex-amygdala transition zones	0.425	0.036
F	Cxcr2	right corticospinal tract/pyramids	0.874	0.043
F	Cxcr2	right crus 1 white matter	0.176	0.009
F	Cxcr2	right crus 1: ansiform lobule (lobule 6)	2.120	0.100
F	Cxcr2	right crus 2 white matter	0.153	0.013
F	Cxcr2	right crus 2: ansiform lobule (lobule 7)	2.085	0.123
F	Cxcr2	right cuneate nucleus	0.122	0.011
F	Cxcr2	right dentate nucleus	0.178	0.009
F	Cxcr2	right Dorsal intermediate entorhinal cortex	0.911	0.067
F	Cxcr2	right Dorsal nucleus of the endopiriform	0.696	0.042
F	Cxcr2	right Dorsal tenia tecta	0.456	0.026
F	Cxcr2	right Dorsolateral entorhinal cortex	1.302	0.084
F	Cxcr2	right Dorsolateral orbital cortex	0.405	0.036
F	Cxcr2	right Ectorhinal cortex	1.352	0.100
F	Cxcr2	right facial nerve (cranial nerve 7)	0.107	0.006
F	Cxcr2	right fasciculus retroflexus	0.113	0.008
F	Cxcr2	right fastigial nucleus	0.229	0.014
F	Cxcr2	right fimbria	1.488	0.073
F	Cxcr2	right flocculus (FL)	0.516	0.044
F	Cxcr2	right flocculus white matter	0.038	0.004
F	Cxcr2	right fornix	0.290	0.015
F	Cxcr2	right Frontal association cortex	3.787	0.186
F	Cxcr2	right Frontal cortex: area 3	0.351	0.019
F	Cxcr2	right fundus of striatum	0.077	0.008
F	Cxcr2	right globus pallidus	1.618	0.060
F	Cxcr2	right GrDG	0.605	0.027
F	Cxcr2	right habenular commissure	0.015	0.003
F	Cxcr2	right hypothalamus	5.093	0.192
F	Cxcr2	right inferior olivary complex	0.169	0.017
F	Cxcr2	right Insular region: not subdivided	4.047	0.309
F	Cxcr2	right Intermediate nucleus of the endopiriform claustrum	0.278	0.021
F	Cxcr2	right internal capsule	1.482	0.058
F	Cxcr2	right lateral olfactory tract	0.635	0.032
F	Cxcr2	right Lateral orbital cortex	1.756	0.098
F	Cxcr2	right Lateral parietal association cortex	0.089	0.008
F	Cxcr2	right lateral septum	1.486	0.066
F	Cxcr2	right lateral ventricle	1.862	0.231
F	Cxcr2	right LMol	1.128	0.038
F	Cxcr2	right mammillary bodies	0.224	0.020
F	Cxcr2	right mammilothalamic tract	0.126	0.010
F	Cxcr2	right Medial amygdala	0.513	0.032
F	Cxcr2	right Medial entorhinal cortex	0.339	0.021
F	Cxcr2	right medial lemniscus/medial longitudinal fasciculus	1.296	0.044
F	Cxcr2	right Medial orbital cortex	0.839	0.046
F	Cxcr2	right Medial parietal association cortex	0.169	0.013
F	Cxcr2	right Medial preoptic nucleus	0.089	0.004
F	Cxcr2	right medial septum	0.612	0.023
F	Cxcr2	right MoDG	1.943	0.081
F	Cxcr2	right nucleus accumbens	2.000	0.092
F	Cxcr2	right nucleus interpositus	0.237	0.014
F	Cxcr2	right Olfactory bulb: external plexiform layer	3.138	0.120
F	Cxcr2	right Olfactory bulb: glomerular layer	2.185	0.077
F	Cxcr2	right Olfactory bulb: granule cell layer	2.217	0.127
F	Cxcr2	right Olfactory bulb: internal plexiform layer	0.481	0.036
F	Cxcr2	right Olfactory bulb: mitral cell layer	0.581	0.032
F	Cxcr2	right olfactory peduncle	1.115	0.049
F	Cxcr2	right olfactory tubercle	1.596	0.100
F	Cxcr2	right optic tract	0.696	0.035
F	Cxcr2	right paraflocculus (PFL)	1.595	0.244
F	Cxcr2	right paraflocculus white matter	0.160	0.026
F	Cxcr2	right paramedian lobule	0.083	0.010
F	Cxcr2	right paramedian lobule (lobule 7)	2.020	0.153
F	Cxcr2	right Parietal cortex: posterior area: rostral part	0.041	0.004
F	Cxcr2	right Perirhinal cortex	1.176	0.058
F	Cxcr2	right Piriform cortex	5.240	0.292
F	Cxcr2	right PoDG	0.300	0.016
F	Cxcr2	right pontine nucleus	0.390	0.046
F	Cxcr2	right Posterolateral cortical amygdaloid area	0.429	0.024
F	Cxcr2	right Posteromedial cortical amygdaloid area	0.451	0.025
F	Cxcr2	right pre-para subiculum	1.155	0.060
F	Cxcr2	right Primary auditory cortex	0.728	0.038
F	Cxcr2	right Primary motor cortex	3.155	0.133
F	Cxcr2	right Primary somatosensory cortex	2.091	0.128
F	Cxcr2	right Primary somatosensory cortex: barrel field	4.584	0.237
F	Cxcr2	right Primary somatosensory cortex: dysgranular zone	0.186	0.010
F	Cxcr2	right Primary somatosensory cortex: forelimb region	1.840	0.084
F	Cxcr2	right Primary somatosensory cortex: hindlimb region	1.242	0.061
F	Cxcr2	right Primary somatosensory cortex: jaw region	0.293	0.031
F	Cxcr2	right Primary somatosensory cortex: shoulder region	0.065	0.011
F	Cxcr2	right Primary somatosensory cortex: trunk region	0.196	0.012
F	Cxcr2	right Primary somatosensory cortex: upper lip region	2.701	0.135
F	Cxcr2	right Primary visual cortex	1.185	0.085
F	Cxcr2	right Primary visual cortex: binocular area	1.030	0.066
F	Cxcr2	right Primary visual cortex: monocular area	0.778	0.050
F	Cxcr2	right Rostral amygdalopiriform area	0.195	0.009
F	Cxcr2	right Secondary auditory cortex: dorsal area	0.718	0.039
F	Cxcr2	right Secondary auditory cortex: ventral area	0.976	0.115
F	Cxcr2	right Secondary motor cortex	3.268	0.220
F	Cxcr2	right Secondary somatosensory cortex	3.646	0.391
F	Cxcr2	right Secondary visual cortex: lateral area	1.441	0.072
F	Cxcr2	right Secondary visual cortex: mediolateral area	0.389	0.025
F	Cxcr2	right Secondary visual cortex: mediomedial area	0.777	0.041
F	Cxcr2	right simple lobule (lobule 6)	2.311	0.117
F	Cxcr2	right simple lobule white matter	0.170	0.010
F	Cxcr2	right SLu	0.361	0.017
F	Cxcr2	right stria medullaris	0.340	0.018
F	Cxcr2	right stria terminalis	0.384	0.014
F	Cxcr2	right striatum	10.317	0.432
F	Cxcr2	right subependymale zone / rhinocele	0.025	0.002
F	Cxcr2	right subiculum	1.584	0.070

F	Cxcr2	right superior olivary complex	0.385	0.024
F	Cxcr2	right Temporal association area	1.518	0.127
F	Cxcr2	right thalamus	8.617	0.458
F	Cxcr2	right trunk of crus 2 and paramedian white matter	0.177	0.010
F	Cxcr2	right trunk of simple and crus 1 white matter	0.083	0.006
F	Cxcr2	right Ventral intermediate entorhinal cortex	0.543	0.032
F	Cxcr2	right Ventral nucleus of the endopiriform claustrum	0.223	0.020
F	Cxcr2	right Ventral orbital cortex	0.693	0.026
F	Cxcr2	right Ventral tenia tecta	0.055	0.006
F	Cxcr2	third ventricle	1.249	0.104
F	Cxcr2	trunk of arbor vita	4.101	0.157
F	Cxcr2	trunk of lobules 1-3 white matter	0.119	0.015
F	Cxcr2	trunk of lobules 6-8 white matter	0.103	0.005
F	Cxcr2	ventral tegmental decussation	0.127	0.011
F	Ighm	cerebral aqueduct	0.501	0.053
F	Ighm	fourth ventricle	0.927	0.070
F	Ighm	interpedunclar nucleus	0.270	0.013
F	Ighm	left Accessory olfactory bulb: glomerular, external plexiform and mitral cell layer	0.251	0.010
F	Ighm	left Accessory olfactory bulb: granule cell layer	0.118	0.004
F	Ighm	left amygdala	4.723	0.139
F	Ighm	left Amygdalopiriform transition area	0.515	0.028
F	Ighm	left anterior commissure: pars anterior	0.478	0.015
F	Ighm	left anterior commissure: pars posterior	0.203	0.012
F	Ighm	left anterior lobule (lobules 4-5)	0.836	0.049
F	Ighm	left anterior lobule white matter	0.041	0.004
F	Ighm	left Anterior olfactory nucleus	0.960	0.026
F	Ighm	left basal forebrain	2.551	0.055
F	Ighm	left bed nucleus of stria terminalis	0.653	0.026
F	Ighm	left CA10r	1.126	0.050
F	Ighm	left CA1Py	0.540	0.021
F	Ighm	left CA1Rad	1.206	0.036
F	Ighm	left CA20r	0.258	0.014
F	Ighm	left CA2Py	0.100	0.003
F	Ighm	left CA2Rad	0.200	0.007
F	Ighm	left CA30r	1.406	0.056
F	Ighm	left CA3Py Inner	0.075	0.003
F	Ighm	left CA3Py Outer	0.507	0.020
F	Ighm	left CA3Rad	0.887	0.038
F	Ighm	left Caudomedial entorhinal cortex	2.824	0.091
F	Ighm	left cerebellar peduncle: inferior	0.419	0.014
F	Ighm	left cerebellar peduncle: middle	0.580	0.030
F	Ighm	left cerebellar peduncle: superior	0.503	0.029
F	Ighm	left cerebral peduncle	1.015	0.034
F	Ighm	left Cingulate cortex: area 24a	0.938	0.041
F	Ighm	left Cingulate cortex: area 24a'	0.375	0.024
F	Ighm	left Cingulate cortex: area 24b	0.806	0.070
F	Ighm	left Cingulate cortex: area 24b'	0.301	0.021
F	Ighm	left Cingulate cortex: area 25	0.287	0.017
F	Ighm	left Cingulate cortex: area 29a	0.369	0.022
F	Ighm	left Cingulate cortex: area 29b	0.211	0.014
F	Ighm	left Cingulate cortex: area 29c	0.916	0.054
F	Ighm	left Cingulate cortex: area 30	1.403	0.061
F	Ighm	left Cingulate cortex: area 32	1.337	0.107
F	Ighm	left Cingulum	0.417	0.021
F	Ighm	left Claustrum	0.174	0.011
F	Ighm	left Claustrum: dorsal part	0.147	0.015
F	Ighm	left Claustrum: ventral part	0.272	0.025
F	Ighm	left colliculus: inferior	2.828	0.096
F	Ighm	left colliculus: superior	4.277	0.148
F	Ighm	left copula white matter	0.044	0.002
F	Ighm	left copula: pyramis (lobule 8)	1.182	0.085
F	Ighm	left corpus callosum	5.955	0.229
F	Ighm	left Cortex-amygdala transition zones	0.372	0.026
F	Ighm	left corticospinal tract/pyramids	0.848	0.051
F	Ighm	left crus 1 white matter	0.185	0.009
F	Ighm	left crus 1: ansiform lobule (lobule 6)	2.085	0.085
F	Ighm	left crus 2 white matter	0.129	0.010
F	Ighm	left crus 2: ansiform lobule (lobule 7)	1.945	0.105
F	Ighm	left cuneate nucleus	0.119	0.008
F	Ighm	left dentate nucleus	0.177	0.008
F	Ighm	left Dorsal intermediate entorhinal cortex	0.846	0.041
F	Ighm	left Dorsal nucleus of the endopiriform	0.729	0.028
F	Ighm	left Dorsal tenia tecta	0.500	0.020
F	Ighm	left Dorsolateral entorhinal cortex	1.181	0.060
F	Ighm	left Dorsolateral orbital cortex	0.430	0.017
F	Ighm	left Ectorhinal cortex	1.248	0.070
F	Ighm	left facial nerve (cranial nerve 7)	0.105	0.004
F	Ighm	left fasciculus retroflexus	0.127	0.006
F	Ighm	left fastigial nucleus	0.239	0.010
F	Ighm	left fimbria	1.633	0.078
F	Ighm	left flocculus (FL)	0.526	0.029
F	Ighm	left flocculus white matter	0.028	0.002
F	Ighm	left fornix	0.331	0.016
F	Ighm	left Frontal association cortex	3.534	0.166
F	Ighm	left Frontal cortex: area 3	0.352	0.019
F	Ighm	left fundus of striatum	0.071	0.005
F	Ighm	left globus pallidus	1.631	0.041
F	Ighm	left GrDG	0.665	0.016
F	Ighm	left habenular commissure	0.014	0.002
F	Ighm	left hypothalamus	5.192	0.135
F	Ighm	left inferior olivary complex	0.160	0.020
F	Ighm	left Insular region: not subdivided	3.699	0.169
F	Ighm	left Intermediate nucleus of the endopiriform claustrum	0.303	0.012
F	Ighm	left internal capsule	1.420	0.053
F	Ighm	left lateral olfactory tract	0.721	0.029
F	Ighm	left Lateral orbital cortex	1.772	0.044
F	Ighm	left Lateral parietal association cortex	0.143	0.012
F	Ighm	left lateral septum	1.499	0.055
F	Ighm	left lateral ventricle	1.887	0.213
F	Ighm	left LMol	1.143	0.036
F	Ighm	left mammillary bodies	0.241	0.019
F	Ighm	left mammilothalamic tract	0.123	0.007
F	Ighm	left Medial amygdala	0.492	0.017
F	Ighm	left Medial entorhinal cortex	0.339	0.015
F	Ighm	left medial lemniscus/medial longitudinal fasciculus	1.236	0.041
F	Ighm	left Medial orbital cortex	0.925	0.068
F	Ighm	left Medial parietal association cortex	0.227	0.022
F	Ighm	left Medial preoptic nucleus	0.095	0.004
F	Ighm	left medial septum	0.640	0.025
F	Ighm	left MoDG	2.131	0.065
F	Ighm	left nucleus accumbens	2.038	0.080
F	Ighm	left nucleus interpositus	0.218	0.010
F	Ighm	left Olfactory bulb: external plexiform layer	3.132	0.082
F	Ighm	left Olfactory bulb: glomerular layer	2.092	0.056
F	Ighm	left Olfactory bulb: granule cell layer	2.157	0.080
F	Ighm	left Olfactory bulb: internal plexiform layer	0.497	0.015
F	Ighm	left Olfactory bulb: mitral cell layer	0.627	0.015
F	Ighm	left olfactory peduncle	1.138	0.031
F	Ighm	left olfactory tubercle	1.592	0.080
F	Ighm	left optic tract	0.768	0.023
F	Ighm	left paraflocculus (PFL)	1.627	0.294
F	Ighm	left paraflocculus white matter	0.137	0.020
F	Ighm	left paramedian lobule	0.072	0.005
F	Ighm	left paramedian lobule (lobule 7)	1.993	0.130
F	Ighm	left Parietal cortex: posterior area: rostral part	0.070	0.005
F	Ighm	left Perirhinal cortex	1.217	0.049
F	Ighm	left Piriform cortex	4.922	0.190
F	Ighm	left PoDG	0.296	0.010
F	Ighm	left pontine nucleus	0.399	0.035
F	Ighm	left Posterolateral cortical amygdaloid area	0.385	0.021
F	Ighm	left Posteromedial cortical amygdaloid area	0.561	0.022
F	Ighm	left pre-para subiculum	1.098	0.053
F	Ighm	left Primary auditory cortex	0.721	0.051
F	Ighm	left Primary motor cortex	3.666	0.134
F	Ighm	left Primary somatosensory cortex	1.938	0.055
F	Ighm	left Primary somatosensory cortex: barrel field	4.665	0.179
F	Ighm	left Primary somatosensory cortex: dysgranular zone	0.166	0.009
F	Ighm	left Primary somatosensory cortex: forelimb region	2.016	0.107
F	Ighm	left Primary somatosensory cortex: hindlimb region	1.451	0.106
F	Ighm	left Primary somatosensory cortex: jaw region	0.259	0.021
F	Ighm	left Primary somatosensory cortex: shoulder region	0.091	0.007
F	Ighm	left Primary somatosensory cortex: trunk region	0.299	0.023
F	Ighm	left Primary somatosensory cortex: upper lip region	2.274	0.106
F	Ighm	left Primary visual cortex	1.130	0.051
F	Ighm	left Primary visual cortex: binocular area	0.976	0.033
F	Ighm	left Primary visual cortex: monocular area	1.120	0.060
F	Ighm	left Rostral amygdalopiriform area	0.184	0.021
F	Ighm	left Secondary auditory cortex: dorsal area	0.721	0.054
F	Ighm	left Secondary auditory cortex: ventral area	0.745	0.058
F	Ighm	left Secondary motor cortex	3.300	0.145
F	Ighm	left Secondary somatosensory cortex	3.238	0.187
F	Ighm	left Secondary visual cortex: lateral area	1.263	0.058
F	Ighm	left Secondary visual cortex: mediolateral area	0.570	0.045
F	Ighm	left Secondary visual cortex: mediomedial area	0.914	0.054
F	Ighm	left simple lobule (lobule 6)	2.270	0.105
F	Ighm	left simple lobule white matter	0.210	0.014
F	Ighm	left SLu	0.366	0.014
F	Ighm	left stria medullaris	0.320	0.014
F	Ighm	left stria terminalis	0.455	0.017
F	Ighm	left striatum	10.567	0.350
F	Ighm	left subependymale zone / rhinocele	0.028	0.001
F	Ighm	left subiculum	1.605	0.057
F	Ighm	left superior olivary complex	0.369	0.018
F	Ighm	left Temporal association area	1.316	0.076
F	Ighm	left thalamus	8.812	0.362
F	Ighm	left trunk of crus 2 and paramedian white matter	0.167	0.011
F	Ighm	left trunk of simple and crus 1 white matter	0.070	0.005
F	Ighm	left Ventral intermediate entorhinal cortex	0.536	0.022
F	Ighm	left Ventral nucleus of the endopiriform claustrum	0.238	0.020
F	Ighm	left Ventral orbital cortex	0.742	0.039
F	Ighm	left Ventral tenia tecta	0.055	0.003
F	Ighm	lobule 1-2 white matter	0.062	0.020
F	Ighm	lobule 10 white matter	0.077	0.005
F	Ighm	lobule 10: nodulus	1.302	0.054
F	Ighm	lobule 3 white matter	0.168	0.024
F	Ighm	lobule 3: central lobule (dorsal)	1.928	0.244
F	Ighm	lobule 6: declive	2.420	0.143
F	Ighm	lobule 7: tuber (or folium)	0.932	0.077
F	Ighm	lobule 8 white matter	0.157	0.009
F	Ighm	lobule 8: pyramis	1.560	0.090
F	Ighm	lobule 9 white matter	0.287	0.006
F	Ighm	lobule 9: uvula	2.713	0.103
F	Ighm	lobules 1-2: lingula and central lobule (ventral)	1.536	0.239
F	Ighm	lobules 4-5 white matter	0.546	0.036
F	Ighm	lobules 4-5: culmen (ventral and dorsal)	4.098	0.240
F	Ighm	lobules 6-7 white matter	0.686	0.032
F	Ighm	medulla	26.707	0.575
F	Ighm	midbrain	13.662	0.546
F	Ighm	periaqueductal grey	3.828	0.151
F	Ighm	pons	16.851	0.493
F	Ighm	posterior commissure	0.144	0.008
F	Ighm	right Accessory olfactory bulb: glomerular, external plexiform and mitral cell layer	0.241	0.010
F	Ighm	right Accessory olfactory bulb: granule cell layer	0.110	0.006
F	Ighm	right amygdala	4.463	0.141
F	Ighm	right Amygdalopiriform transition area	0.483	0.023
F	Ighm	right anterior commissure: pars anterior	0.478	0.021
F	Ighm	right anterior commissure: pars posterior	0.193	0.011
F	Ighm	right anterior lobule (lobules 4-5)	0.755	0.033
F	Ighm	right anterior lobule white matter	0.050	0.004
F	Ighm	right Anterior olfactory nucleus	0.991	0.050
F	Ighm	right basal forebrain	2.682	0.087
F	Ighm	right bed nucleus of stria terminalis	0.643	0.029
F	Ighm	right CA10r	0.991	0.040
F	Ighm	right CA1Py	0.545	0.018
F	Ighm	right CA1Rad	1.318	0.039
F	Ighm	right CA20r	0.209	0.010
F	Ighm	right CA2Py	0.095	0.004
F	Ighm	right CA2Rad	0.209	0.011
F	Ighm	right CA30r	1.394	0.049
F	Ighm	right CA3Py Inner	0.076	0.004
F	Ighm	right CA3Py Outer	0.505	0.017
F	Ighm	right CA3Rad	0.916	0.040
F	Ighm	right Caudomedial entorhinal cortex	2.785	0.143
F	Ighm	right cerebellar peduncle: inferior	0.390	0.015
F	Ighm	right cerebellar peduncle: middle	0.619	0.027
F	Ighm	right cerebellar peduncle: superior	0.479	0.025
F	Ighm	right cerebral peduncle	1.027	0.030
F	Ighm	right Cingulate cortex: area 24a	0.981	0.058
F	Ighm	right Cingulate cortex: area 24a'	0.348	0.030
F	Ighm	right Cingulate cortex: area 24b	0.770	0.080
F	Ighm	right Cingulate cortex: area 24b'	0.291	0.019
F	Ighm	right Cingulate cortex: area 25	0.296	0.014
F	Ighm	right Cingulate cortex: area 29a	0.357	0.021
F	Ighm	right Cingulate cortex: area 29b	0.204	0.015
F	Ighm	right Cingulate cortex: area 29c	0.869	0.036
F	Ighm	right Cingulate cortex: area 30	1.339	0.057
F	Ighm	right Cingulate cortex: area 32	1.094	0.074
F	Ighm	right Cingulum	0.423	0.025
F	Ighm	right Claustrum	0.144	0.010
F	Ighm	right Claustrum: dorsal part	0.140	0.012
F	Ighm	right Claustrum: ventral part	0.312	0.018
F	Ighm	right colliculus: inferior	2.699	0.087
F	Ighm	right colliculus: superior	4.323	0.165
F	Ighm	right copula white matter	0.036	0.003
F	Ighm	right copula: pyramis (lobule 8)	1.293	0.073
F	Ighm	right corpus callosum	5.695	0.191
F	Ighm	right Cortex-amygdala transition zones	0.396	0.030
F	Ighm	right corticospinal tract/pyramids	0.876	0.066
F	Ighm	right crus 1 white matter	0.178	0.012
F	Ighm	right crus 1: ansiform lobule (lobule 6)	2.107	0.088
F	Ighm	right crus 2 white matter	0.146	0.011
F	Ighm	right crus 2: ansiform lobule (lobule 7)	1.977	0.125
F	Ighm	right cuneate nucleus	0.124	0.011
F	Ighm	right dentate nucleus	0.172	0.008
F	Ighm	right Dorsal intermediate entorhinal cortex	0.907	0.051
F	Ighm	right Dorsal nucleus of the endopiriform	0.707	0.021
F	Ighm	right Dorsal tenia tecta	0.466	0.028
F	Ighm	right Dorsolateral entorhinal cortex	1.335	0.054
F	Ighm	right Dorsolateral orbital cortex	0.394	0.028
F	Ighm	right Ectorhinal cortex	1.325	0.107
F	Ighm	right facial nerve (cranial nerve 7)	0.109	0.004
F	Ighm	right fasciculus retroflexus	0.113	0.006
F	Ighm	right fastigial nucleus	0.227	0.013
F	Ighm	right fimbria	1.512	0.057
F	Ighm	right flocculus (FL)	0.503	0.025
F	Ighm	right flocculus white matter	0.037	0.005
F	Ighm	right fornix	0.293	0.012
F	Ighm	right Frontal association cortex	3.958	0.195
F	Ighm	right Frontal cortex: area 3	0.352	0.021
F	Ighm	right fundus of striatum	0.079	0.007
F	Ighm	right globus pallidus	1.663	0.063
F	Ighm	right GrDG	0.626	0.020
F	Ighm	right habenular commissure	0.014	0.002
F	Ighm	right hypothalamus	5.181	0.142
F	Ighm	right inferior olivary complex	0.171	0.017
F	Ighm	right Insular region: not subdivided	3.965	0.255
F	Ighm	right Intermediate nucleus of the endopiriform claustrum	0.280	0.017

F	Ighm	right internal capsule	1.513	0.054
F	Ighm	right lateral olfactory tract	0.636	0.024
F	Ighm	right Lateral orbital cortex	1.716	0.077
F	Ighm	right Lateral parietal association cortex	0.090	0.007
F	Ighm	right lateral septum	1.495	0.058
F	Ighm	right lateral ventricle	1.913	0.206
F	Ighm	right LMol	1.132	0.032
F	Ighm	right mammillary bodies	0.228	0.022
F	Ighm	right mammilothalamic tract	0.127	0.008
F	Ighm	right Medial amygdala	0.518	0.025
F	Ighm	right Medial entorhinal cortex	0.330	0.018
F	Ighm	right medial lemniscus/medial longitudinal fasciculus	1.307	0.057
F	Ighm	right Medial orbital cortex	0.854	0.059
F	Ighm	right Medial parietal association cortex	0.171	0.012
F	Ighm	right Medial preoptic nucleus	0.090	0.005
F	Ighm	right medial septum	0.605	0.018
F	Ighm	right MoDG	2.002	0.056
F	Ighm	right nucleus accumbens	2.045	0.064
F	Ighm	right nucleus interpositus	0.227	0.011
F	Ighm	right Olfactory bulb: external plexiform layer	3.156	0.058
F	Ighm	right Olfactory bulb: glomerular layer	2.183	0.053
F	Ighm	right Olfactory bulb: granule cell layer	2.213	0.094
F	Ighm	right Olfactory bulb: internal plexiform layer	0.492	0.023
F	Ighm	right Olfactory bulb: mitral cell layer	0.592	0.025
F	Ighm	right olfactory peduncle	1.117	0.042
F	Ighm	right olfactory tubercle	1.643	0.064
F	Ighm	right optic tract	0.715	0.022
F	Ighm	right paraflocculus (PFL)	1.570	0.260
F	Ighm	right paraflocculus white matter	0.153	0.026
F	Ighm	right paramedian lobule	0.081	0.005
F	Ighm	right paramedian lobule (lobule 7)	1.973	0.086
F	Ighm	right Parietal cortex: posterior area: rostral part	0.044	0.004
F	Ighm	right Perirhinal cortex	1.173	0.054
F	Ighm	right Piriform cortex	5.206	0.224
F	Ighm	right PoDG	0.309	0.011
F	Ighm	right pontine nucleus	0.393	0.034
F	Ighm	right Posterolateral cortical amygdaloid area	0.432	0.025
F	Ighm	right Posteromedial cortical amygdaloid area	0.463	0.050
F	Ighm	right pre-para subiculum	1.144	0.049
F	Ighm	right Primary auditory cortex	0.719	0.051
F	Ighm	right Primary motor cortex	3.123	0.121
F	Ighm	right Primary somatosensory cortex	2.029	0.117
F	Ighm	right Primary somatosensory cortex: barrel field	4.539	0.204
F	Ighm	right Primary somatosensory cortex: dysgranular zone	0.176	0.010
F	Ighm	right Primary somatosensory cortex: forelimb region	1.802	0.090
F	Ighm	right Primary somatosensory cortex: hindlimb region	1.200	0.059
F	Ighm	right Primary somatosensory cortex: jaw region	0.279	0.016
F	Ighm	right Primary somatosensory cortex: shoulder region	0.063	0.007
F	Ighm	right Primary somatosensory cortex: trunk region	0.201	0.017
F	Ighm	right Primary somatosensory cortex: upper lip region	2.653	0.144
F	Ighm	right Primary visual cortex	1.170	0.063
F	Ighm	right Primary visual cortex: binocular area	0.987	0.052
F	Ighm	right Primary visual cortex: monocular area	0.783	0.030
F	Ighm	right Rostral amygdalopiriform area	0.200	0.019
F	Ighm	right Secondary auditory cortex: dorsal area	0.691	0.052
F	Ighm	right Secondary auditory cortex: ventral area	0.943	0.071
F	Ighm	right Secondary motor cortex	3.205	0.113
F	Ighm	right Secondary somatosensory cortex	3.477	0.286
F	Ighm	right Secondary visual cortex: lateral area	1.413	0.087
F	Ighm	right Secondary visual cortex: mediolateral area	0.396	0.026
F	Ighm	right Secondary visual cortex: mediomedial area	0.781	0.034
F	Ighm	right simple lobule (lobule 6)	2.324	0.118
F	Ighm	right simple lobule white matter	0.175	0.011
F	Ighm	right SLu	0.361	0.017
F	Ighm	right stria medullaris	0.343	0.015
F	Ighm	right stria terminalis	0.386	0.016
F	Ighm	right striatum	10.501	0.395
F	Ighm	right subependymale zone / rhinocele	0.025	0.001
F	Ighm	right subiculum	1.606	0.068
F	Ighm	right superior olivary complex	0.396	0.022
F	Ighm	right Temporal association area	1.486	0.106
F	Ighm	right thalamus	8.739	0.420
F	Ighm	right trunk of crus 2 and paramedian white matter	0.178	0.007
F	Ighm	right trunk of simple and crus 1 white matter	0.080	0.004
F	Ighm	right Ventral intermediate entorhinal cortex	0.523	0.026
F	Ighm	right Ventral nucleus of the endopiriform claustrum	0.225	0.021
F	Ighm	right Ventral orbital cortex	0.674	0.034
F	Ighm	right Ventral tenia tecta	0.056	0.002
F	Ighm	third ventricle	1.249	0.076
F	Ighm	trunk of arbor vita	4.098	0.127
F	Ighm	trunk of lobules 1-3 white matter	0.121	0.008
F	Ighm	trunk of lobules 6-8 white matter	0.109	0.006
F	Ighm	ventral tegmental decussation	0.126	0.010
F	IL-6	cerebral aqueduct	0.496	0.056
F	IL-6	fourth ventricle	0.897	0.053
F	IL-6	interpeduncular nucleus	0.279	0.016
F	IL-6	left Accessory olfactory bulb: glomerular, external plexiform and mitral cell layer	0.257	0.011
F	IL-6	left Accessory olfactory bulb: granule cell layer	0.125	0.006
F	IL-6	left amygdala	4.803	0.178
F	IL-6	left Amygdalopiriform transition area	0.529	0.029
F	IL-6	left anterior commissure: pars anterior	0.500	0.026
F	IL-6	left anterior commissure: pars posterior	0.218	0.011
F	IL-6	left anterior lobule (lobules 4-5)	0.881	0.072
F	IL-6	left anterior lobule white matter	0.044	0.005
F	IL-6	left Anterior olfactory nucleus	0.986	0.039
F	IL-6	left basal forebrain	2.622	0.117
F	IL-6	left bed nucleus of stria terminalis	0.678	0.030
F	IL-6	left CA10r	1.193	0.050
F	IL-6	left CA1Py	0.571	0.024
F	IL-6	left CA1Rad	1.263	0.046
F	IL-6	left CA20r	0.274	0.012
F	IL-6	left CA2Py	0.106	0.004
F	IL-6	left CA2Rad	0.210	0.008
F	IL-6	left CA30r	1.442	0.063
F	IL-6	left CA3Py Inner	0.080	0.003
F	IL-6	left CA3Py Outer	0.529	0.017
F	IL-6	left CA3Rad	0.940	0.034
F	IL-6	left Caudomedial entorhinal cortex	2.906	0.138
F	IL-6	left cerebellar peduncle: inferior	0.430	0.019
F	IL-6	left cerebellar peduncle: middle	0.601	0.034
F	IL-6	left cerebellar peduncle: superior	0.525	0.024
F	IL-6	left cerebral peduncle	1.022	0.026
F	IL-6	left Cingulate cortex: area 24a	0.932	0.069
F	IL-6	left Cingulate cortex: area 24a'	0.386	0.030
F	IL-6	left Cingulate cortex: area 24b	0.828	0.088
F	IL-6	left Cingulate cortex: area 24b'	0.318	0.036
F	IL-6	left Cingulate cortex: area 25	0.297	0.020
F	IL-6	left Cingulate cortex: area 29a	0.395	0.017
F	IL-6	left Cingulate cortex: area 29b	0.220	0.019
F	IL-6	left Cingulate cortex: area 29c	1.013	0.076
F	IL-6	left Cingulate cortex: area 30	1.535	0.076
F	IL-6	left Cingulate cortex: area 32	1.394	0.083
F	IL-6	left Cingulum	0.456	0.029
F	IL-6	left Claustrum	0.178	0.010
F	IL-6	left Claustrum: dorsal part	0.149	0.019
F	IL-6	left Claustrum: ventral part	0.274	0.030
F	IL-6	left colliculus: inferior	2.843	0.105
F	IL-6	left colliculus: superior	4.372	0.245
F	IL-6	left copula white matter	0.045	0.002
F	IL-6	left copula: pyramis (lobule 8)	1.186	0.092
F	IL-6	left corpus callosum	6.288	0.238
F	IL-6	left Cortex-amygdala transition zones	0.389	0.038
F	IL-6	left corticospinal tract/pyramids	0.866	0.050
F	IL-6	left crus 1 white matter	0.190	0.013
F	IL-6	left crus 1: ansiform lobule (lobule 6)	2.172	0.116
F	IL-6	left crus 2 white matter	0.129	0.015
F	IL-6	left crus 2: ansiform lobule (lobule 7)	1.954	0.196
F	IL-6	left cuneate nucleus	0.118	0.008
F	IL-6	left dentate nucleus	0.181	0.008
F	IL-6	left Dorsal intermediate entorhinal cortex	0.881	0.053
F	IL-6	left Dorsal nucleus of the endopiriform	0.745	0.034
F	IL-6	left Dorsal tenia tecta	0.488	0.025
F	IL-6	left Dorsolateral entorhinal cortex	1.192	0.069
F	IL-6	left Dorsolateral orbital cortex	0.440	0.032
F	IL-6	left Ectorhinal cortex	1.266	0.105
F	IL-6	left facial nerve (cranial nerve 7)	0.109	0.005
F	IL-6	left fasciculus retroflexus	0.135	0.005
F	IL-6	left fastigial nucleus	0.241	0.011
F	IL-6	left fimbria	1.699	0.095
F	IL-6	left flocculus (FL)	0.545	0.029
F	IL-6	left flocculus white matter	0.030	0.003
F	IL-6	left fornix	0.343	0.016
F	IL-6	left Frontal association cortex	3.761	0.253
F	IL-6	left Frontal cortex: area 3	0.362	0.024
F	IL-6	left fundus of striatum	0.076	0.006
F	IL-6	left globus pallidus	1.625	0.059
F	IL-6	left GrDG	0.698	0.022
F	IL-6	left habenular commissure	0.016	0.003
F	IL-6	left hypothalamus	5.287	0.173
F	IL-6	left inferior olivary complex	0.155	0.012
F	IL-6	left Insular region: not subdivided	3.744	0.330
F	IL-6	left Intermediate nucleus of the endopiriform claustrum	0.314	0.013
F	IL-6	left internal capsule	1.449	0.045
F	IL-6	left lateral olfactory tract	0.728	0.048
F	IL-6	left Lateral orbital cortex	1.850	0.092
F	IL-6	left Lateral parietal association cortex	0.159	0.011
F	IL-6	left lateral septum	1.533	0.076
F	IL-6	left lateral ventricle	1.782	0.226
F	IL-6	left LMol	1.202	0.036
F	IL-6	left mammillary bodies	0.249	0.018
F	IL-6	left mammilothalamic tract	0.128	0.007
F	IL-6	left Medial amygdala	0.501	0.031
F	IL-6	left Medial entorhinal cortex	0.347	0.019
F	IL-6	left medial lemniscus/medial longitudinal fasciculus	1.248	0.045
F	IL-6	left Medial orbital cortex	0.923	0.050
F	IL-6	left Medial parietal association cortex	0.255	0.031
F	IL-6	left Medial preoptic nucleus	0.096	0.007
F	IL-6	left medial septum	0.664	0.025
F	IL-6	left MoDG	2.224	0.063
F	IL-6	left nucleus accumbens	2.091	0.089
F	IL-6	left nucleus interpositus	0.223	0.011
F	IL-6	left Olfactory bulb: external plexiform layer	3.172	0.171
F	IL-6	left Olfactory bulb: glomerular layer	2.135	0.119
F	IL-6	left Olfactory bulb: granule cell layer	2.169	0.132
F	IL-6	left Olfactory bulb: internal plexiform layer	0.493	0.033
F	IL-6	left Olfactory bulb: mitral cell layer	0.623	0.041
F	IL-6	left olfactory peduncle	1.156	0.048
F	IL-6	left olfactory tubercle	1.603	0.087
F	IL-6	left optic tract	0.753	0.062
F	IL-6	left paraflocculus (PFL)	1.555	0.217
F	IL-6	left paraflocculus white matter	0.132	0.013
F	IL-6	left paramedian lobule	0.076	0.004
F	IL-6	left paramedian lobule (lobule 7)	2.105	0.107
F	IL-6	left Parietal cortex: posterior area: rostral part	0.073	0.007
F	IL-6	left Perirhinal cortex	1.204	0.088
F	IL-6	left Piriform cortex	4.994	0.280
F	IL-6	left PoDG	0.309	0.012
F	IL-6	left pontine nucleus	0.406	0.030
F	IL-6	left Posterolateral cortical amygdaloid area	0.393	0.032
F	IL-6	left Posteromedial cortical amygdaloid area	0.563	0.032
F	IL-6	left pre-para subiculum	1.170	0.042
F	IL-6	left Primary auditory cortex	0.760	0.058
F	IL-6	left Primary motor cortex	3.826	0.137
F	IL-6	left Primary somatosensory cortex	2.024	0.117
F	IL-6	left Primary somatosensory cortex: barrel field	4.875	0.201
F	IL-6	left Primary somatosensory cortex: dysgranular zone	0.171	0.011
F	IL-6	left Primary somatosensory cortex: forelimb region	2.085	0.136
F	IL-6	left Primary somatosensory cortex: hindlimb region	1.526	0.086
F	IL-6	left Primary somatosensory cortex: jaw region	0.279	0.029
F	IL-6	left Primary somatosensory cortex: shoulder region	0.096	0.007
F	IL-6	left Primary somatosensory cortex: trunk region	0.321	0.016
F	IL-6	left Primary somatosensory cortex: upper lip region	2.331	0.139
F	IL-6	left Primary visual cortex	1.224	0.102
F	IL-6	left Primary visual cortex: binocular area	1.045	0.053
F	IL-6	left Primary visual cortex: monocular area	1.245	0.089
F	IL-6	left Rostral amygdalopiriform area	0.192	0.022
F	IL-6	left Secondary auditory cortex: dorsal area	0.811	0.074
F	IL-6	left Secondary auditory cortex: ventral area	0.768	0.067
F	IL-6	left Secondary motor cortex	3.472	0.132
F	IL-6	left Secondary somatosensory cortex	3.293	0.273
F	IL-6	left Secondary visual cortex: lateral area	1.359	0.061
F	IL-6	left Secondary visual cortex: mediolateral area	0.630	0.049
F	IL-6	left Secondary visual cortex: mediomedial area	0.999	0.047
F	IL-6	left simple lobule (lobule 6)	2.480	0.109
F	IL-6	left simple lobule white matter	0.229	0.016
F	IL-6	left SLu	0.386	0.014
F	IL-6	left stria medullaris	0.336	0.016
F	IL-6	left stria terminalis	0.474	0.017
F	IL-6	left striatum	10.754	0.357
F	IL-6	left subependymale zone / rhinocele	0.029	0.002
F	IL-6	left subiculum	1.655	0.064
F	IL-6	left superior olivary complex	0.373	0.025
F	IL-6	left Temporal association area	1.331	0.089
F	IL-6	left thalamus	9.068	0.396
F	IL-6	left trunk of crus 2 and paramedian white matter	0.172	0.015
F	IL-6	left trunk of simple and crus 1 white matter	0.068	0.006
F	IL-6	left Ventral intermediate entorhinal cortex	0.543	0.026
F	IL-6	left Ventral nucleus of the endopiriform claustrum	0.254	0.024
F	IL-6	left Ventral orbital cortex	0.774	0.036
F	IL-6	left Ventral tenia tecta	0.054	0.004
F	IL-6	lobule 1-2 white matter	0.074	0.012
F	IL-6	lobule 10 white matter	0.084	0.004
F	IL-6	lobule 10: nodulus	1.340	0.064
F	IL-6	lobule 3 white matter	0.195	0.012
F	IL-6	lobule 3: central lobule (dorsal)	2.178	0.164
F	IL-6	lobule 6: declive	2.532	0.115
F	IL-6	lobule 7: tuber (or folium)	0.962	0.078
F	IL-6	lobule 8 white matter	0.140	0.011
F	IL-6	lobule 8: pyramis	1.468	0.091
F	IL-6	lobule 9 white matter	0.264	0.019
F	IL-6	lobule 9: uvula	2.588	0.154
F	IL-6	lobules 1-2: lingula and central lobule (ventral)	1.746	0.172
F	IL-6	lobules 4-5 white matter	0.575	0.031
F	IL-6	lobules 4-5: culmen (ventral and dorsal)	4.313	0.280
F	IL-6	lobules 6-7 white matter	0.707	0.025
F	IL-6	medulla	27.152	0.904
F	IL-6	midbrain	13.988	0.563
F	IL-6	periaqueductal grey	3.996	0.123
F	IL-6	pons	17.530	0.473
F	IL-6	posterior commissure	0.148	0.008
F	IL-6	right Accessory olfactory bulb: glomerular, external plexiform and mitral cell layer	0.246	0.015
F	IL-6	right Accessory olfactory bulb: granule cell layer	0.117	0.005
F	IL-6	right amygdala	4.523	0.163
F	IL-6	right Amygdalopiriform transition area	0.497	0.036
F	IL-6	right anterior commissure: pars anterior	0.488	0.021
F	IL-6	right anterior commissure: pars posterior	0.204	0.010
F	IL-6	right anterior lobule (lobules 4-5)	0.812	0.049

F	IL-6	right anterior lobule white matter	0.053	0.005
F	IL-6	right Anterior olfactory nucleus	1.021	0.043
F	IL-6	right basal forebrain	2.707	0.094
F	IL-6	right bed nucleus of stria terminalis	0.669	0.028
F	IL-6	right CA10r	1.060	0.051
F	IL-6	right CA1Py	0.577	0.022
F	IL-6	right CA1Rad	1.357	0.038
F	IL-6	right CA20r	0.228	0.012
F	IL-6	right CA2Py	0.102	0.003
F	IL-6	right CA2Rad	0.222	0.009
F	IL-6	right CA30r	1.427	0.056
F	IL-6	right CA3Py Inner	0.080	0.003
F	IL-6	right CA3Py Outer	0.524	0.016
F	IL-6	right CA3Rad	0.964	0.030
F	IL-6	right Caudomedial entorhinal cortex	2.843	0.135
F	IL-6	right cerebellar peduncle: inferior	0.396	0.016
F	IL-6	right cerebellar peduncle: middle	0.644	0.029
F	IL-6	right cerebellar peduncle: superior	0.508	0.026
F	IL-6	right cerebral peduncle	1.048	0.036
F	IL-6	right Cingulate cortex: area 24a	1.007	0.068
F	IL-6	right Cingulate cortex: area 24a'	0.367	0.038
F	IL-6	right Cingulate cortex: area 24b	0.793	0.077
F	IL-6	right Cingulate cortex: area 24b'	0.306	0.032
F	IL-6	right Cingulate cortex: area 25	0.305	0.019
F	IL-6	right Cingulate cortex: area 29a	0.378	0.021
F	IL-6	right Cingulate cortex: area 29b	0.212	0.016
F	IL-6	right Cingulate cortex: area 29c	0.963	0.055
F	IL-6	right Cingulate cortex: area 30	1.464	0.127
F	IL-6	right Cingulate cortex: area 32	1.128	0.097
F	IL-6	right Cingulum	0.471	0.023
F	IL-6	right Claustrum	0.150	0.008
F	IL-6	right Claustrum: dorsal part	0.138	0.018
F	IL-6	right Claustrum: ventral part	0.301	0.030
F	IL-6	right colliculus: inferior	2.767	0.106
F	IL-6	right colliculus: superior	4.429	0.229
F	IL-6	right copula white matter	0.032	0.003
F	IL-6	right copula: pyramis (lobule 8)	1.308	0.055
F	IL-6	right corpus callosum	5.920	0.221
F	IL-6	right Cortex-amygdala transition zones	0.404	0.041
F	IL-6	right corticospinal tract/pyramids	0.899	0.055
F	IL-6	right crus 1 white matter	0.187	0.012
F	IL-6	right crus 1: ansiform lobule (lobule 6)	2.230	0.088
F	IL-6	right crus 2 white matter	0.150	0.009
F	IL-6	right crus 2: ansiform lobule (lobule 7)	2.032	0.089
F	IL-6	right cuneate nucleus	0.121	0.009
F	IL-6	right dentate nucleus	0.178	0.009
F	IL-6	right Dorsal intermediate entorhinal cortex	0.910	0.055
F	IL-6	right Dorsal nucleus of the endopiriform	0.711	0.040
F	IL-6	right Dorsal tenia tecta	0.452	0.022
F	IL-6	right Dorsolateral entorhinal cortex	1.356	0.061
F	IL-6	right Dorsolateral orbital cortex	0.413	0.022
F	IL-6	right Ectorhinal cortex	1.413	0.082
F	IL-6	right facial nerve (cranial nerve 7)	0.111	0.004
F	IL-6	right fasciculus retroflexus	0.119	0.007
F	IL-6	right fastigial nucleus	0.233	0.010
F	IL-6	right fimbria	1.552	0.080
F	IL-6	right flocculus (FL)	0.523	0.027
F	IL-6	right flocculus white matter	0.039	0.004
F	IL-6	right fornix	0.304	0.014
F	IL-6	right Frontal association cortex	4.137	0.307
F	IL-6	right Frontal cortex: area 3	0.367	0.018
F	IL-6	right fundus of striatum	0.080	0.009
F	IL-6	right globus pallidus	1.639	0.073
F	IL-6	right GrDG	0.654	0.026
F	IL-6	right habenular commissure	0.015	0.002
F	IL-6	right hypothalamus	5.275	0.165
F	IL-6	right inferior olivary complex	0.169	0.013
F	IL-6	right Insular region: not subdivided	4.059	0.206
F	IL-6	right Intermediate nucleus of the endopiriform claustrum	0.288	0.015
F	IL-6	right internal capsule	1.532	0.061
F	IL-6	right lateral olfactory tract	0.658	0.035
F	IL-6	right Lateral orbital cortex	1.825	0.096
F	IL-6	right Lateral parietal association cortex	0.099	0.032
F	IL-6	right lateral septum	1.540	0.071
F	IL-6	right lateral ventricle	1.837	0.175
F	IL-6	right LMol	1.181	0.028
F	IL-6	right mammillary bodies	0.231	0.022
F	IL-6	right mammilothalamic tract	0.133	0.005
F	IL-6	right Medial amygdala	0.535	0.037
F	IL-6	right Medial entorhinal cortex	0.335	0.018
F	IL-6	right medial lemniscus/medial longitudinal fasciculus	1.317	0.037
F	IL-6	right Medial orbital cortex	0.854	0.070
F	IL-6	right Medial parietal association cortex	0.190	0.048
F	IL-6	right Medial preoptic nucleus	0.092	0.005
F	IL-6	right medial septum	0.620	0.025
F	IL-6	right MoDG	2.082	0.065
F	IL-6	right nucleus accumbens	2.087	0.075
F	IL-6	right nucleus interpositus	0.237	0.010
F	IL-6	right Olfactory bulb: external plexiform layer	3.184	0.173
F	IL-6	right Olfactory bulb: glomerular layer	2.222	0.118
F	IL-6	right Olfactory bulb: granule cell layer	2.235	0.114
F	IL-6	right Olfactory bulb: internal plexiform layer	0.480	0.036
F	IL-6	right Olfactory bulb: mitral cell layer	0.584	0.042
F	IL-6	right olfactory peduncle	1.137	0.051
F	IL-6	right olfactory tubercle	1.647	0.100
F	IL-6	right optic tract	0.699	0.058
F	IL-6	right paraflocculus (PFL)	1.366	0.214
F	IL-6	right paraflocculus white matter	0.134	0.018
F	IL-6	right paramedian lobule	0.087	0.006
F	IL-6	right paramedian lobule (lobule 7)	2.124	0.112
F	IL-6	right Parietal cortex: posterior area: rostral part	0.050	0.017
F	IL-6	right Perirhinal cortex	1.233	0.073
F	IL-6	right Piriform cortex	5.289	0.274
F	IL-6	right PoDG	0.323	0.014
F	IL-6	right pontine nucleus	0.405	0.029
F	IL-6	right Posterolateral cortical amygdaloid area	0.421	0.037
F	IL-6	right Posteromedial cortical amygdaloid area	0.445	0.027
F	IL-6	right pre-para subiculum	1.186	0.042
F	IL-6	right Primary auditory cortex	0.763	0.070
F	IL-6	right Primary motor cortex	3.267	0.112
F	IL-6	right Primary somatosensory cortex	2.082	0.092
F	IL-6	right Primary somatosensory cortex: barrel field	4.817	0.424
F	IL-6	right Primary somatosensory cortex: dysgranular zone	0.182	0.015
F	IL-6	right Primary somatosensory cortex: forelimb region	1.857	0.183
F	IL-6	right Primary somatosensory cortex: hindlimb region	1.240	0.114
F	IL-6	right Primary somatosensory cortex: jaw region	0.285	0.025
F	IL-6	right Primary somatosensory cortex: shoulder region	0.074	0.016
F	IL-6	right Primary somatosensory cortex: trunk region	0.213	0.058
F	IL-6	right Primary somatosensory cortex: upper lip region	2.769	0.170
F	IL-6	right Primary visual cortex	1.262	0.108
F	IL-6	right Primary visual cortex: binocular area	1.079	0.076
F	IL-6	right Primary visual cortex: monocular area	0.846	0.115
F	IL-6	right Rostral amygdalopiriform area	0.194	0.018
F	IL-6	right Secondary auditory cortex: dorsal area	0.738	0.056
F	IL-6	right Secondary auditory cortex: ventral area	0.988	0.086
F	IL-6	right Secondary motor cortex	3.374	0.122
F	IL-6	right Secondary somatosensory cortex	3.597	0.253
F	IL-6	right Secondary visual cortex: lateral area	1.519	0.066
F	IL-6	right Secondary visual cortex: mediolateral area	0.436	0.079
F	IL-6	right Secondary visual cortex: mediomedial area	0.848	0.079
F	IL-6	right simple lobule (lobule 6)	2.546	0.101
F	IL-6	right simple lobule white matter	0.185	0.012
F	IL-6	right SLu	0.380	0.018
F	IL-6	right stria medullaris	0.356	0.016
F	IL-6	right stria terminalis	0.399	0.014
F	IL-6	right striatum	10.566	0.452
F	IL-6	right subependymale zone / rhinocele	0.026	0.002
F	IL-6	right subiculum	1.646	0.050
F	IL-6	right superior olivary complex	0.397	0.030
F	IL-6	right Temporal association area	1.585	0.076
F	IL-6	right thalamus	8.989	0.434
F	IL-6	right trunk of crus 2 and paramedian white matter	0.186	0.011
F	IL-6	right trunk of simple and crus 1 white matter	0.089	0.005
F	IL-6	right Ventral intermediate entorhinal cortex	0.541	0.030
F	IL-6	right Ventral nucleus of the endopiriform claustrum	0.227	0.019
F	IL-6	right Ventral orbital cortex	0.704	0.043
F	IL-6	right Ventral tenia tecta	0.055	0.004
F	IL-6	third ventricle	1.254	0.085
F	IL-6	trunk of arbor vita	4.220	0.117
F	IL-6	trunk of lobules 1-3 white matter	0.130	0.006
F	IL-6	trunk of lobules 6-8 white matter	0.102	0.008
F	IL-6	ventral tegmental decussation	0.135	0.008
F	IL-10	cerebral aqueduct	0.489	0.023
F	IL-10	fourth ventricle	0.937	0.050
F	IL-10	interpeduncular nucleus	0.282	0.015
F	IL-10	left Accessory olfactory bulb: glomerular, external plexiform and mitral cell layer	0.252	0.014
F	IL-10	left Accessory olfactory bulb: granule cell layer	0.126	0.005
F	IL-10	left amygdala	4.864	0.141
F	IL-10	left Amygdalopiriform transition area	0.543	0.023
F	IL-10	left anterior commissure: pars anterior	0.525	0.021
F	IL-10	left anterior commissure: pars posterior	0.220	0.012
F	IL-10	left anterior lobule (lobules 4-5)	0.899	0.069
F	IL-10	left anterior lobule white matter	0.045	0.004
F	IL-10	left Anterior olfactory nucleus	1.026	0.028
F	IL-10	left basal forebrain	2.633	0.101
F	IL-10	left bed nucleus of stria terminalis	0.661	0.032
F	IL-10	left CA10r	1.173	0.045
F	IL-10	left CA1Py	0.556	0.020
F	IL-10	left CA1Rad	1.247	0.033
F	IL-10	left CA20r	0.263	0.011
F	IL-10	left CA2Py	0.105	0.004
F	IL-10	left CA2Rad	0.214	0.007
F	IL-10	left CA30r	1.442	0.062
F	IL-10	left CA3Py Inner	0.081	0.004
F	IL-10	left CA3Py Outer	0.531	0.028
F	IL-10	left CA3Rad	0.949	0.046
F	IL-10	left Caudomedial entorhinal cortex	2.981	0.102
F	IL-10	left cerebellar peduncle: inferior	0.449	0.013
F	IL-10	left cerebellar peduncle: middle	0.618	0.020
F	IL-10	left cerebellar peduncle: superior	0.534	0.033
F	IL-10	left cerebral peduncle	1.039	0.031
F	IL-10	left Cingulate cortex: area 24a	0.938	0.054
F	IL-10	left Cingulate cortex: area 24a'	0.379	0.027
F	IL-10	left Cingulate cortex: area 24b	0.813	0.097
F	IL-10	left Cingulate cortex: area 24b'	0.310	0.021
F	IL-10	left Cingulate cortex: area 25	0.305	0.012
F	IL-10	left Cingulate cortex: area 29a	0.388	0.020
F	IL-10	left Cingulate cortex: area 29b	0.216	0.016
F	IL-10	left Cingulate cortex: area 29c	0.978	0.058
F	IL-10	left Cingulate cortex: area 30	1.500	0.054
F	IL-10	left Cingulate cortex: area 32	1.381	0.092
F	IL-10	left Cingulum	0.444	0.027
F	IL-10	left Claustrum	0.178	0.016
F	IL-10	left Claustrum: dorsal part	0.153	0.014
F	IL-10	left Claustrum: ventral part	0.282	0.026
F	IL-10	left colliculus: inferior	2.887	0.091
F	IL-10	left colliculus: superior	4.465	0.198
F	IL-10	left copula white matter	0.044	0.003
F	IL-10	left copula: pyramis (lobule 8)	1.185	0.097
F	IL-10	left corpus callosum	6.290	0.281
F	IL-10	left Cortex-amygdala transition zones	0.407	0.027
F	IL-10	left corticospinal tract/pyramids	0.867	0.058
F	IL-10	left crus 1 white matter	0.204	0.010
F	IL-10	left crus 1: ansiform lobule (lobule 6)	2.315	0.109
F	IL-10	left crus 2 white matter	0.139	0.009
F	IL-10	left crus 2: ansiform lobule (lobule 7)	2.117	0.095
F	IL-10	left cuneate nucleus	0.118	0.009
F	IL-10	left dentate nucleus	0.190	0.009
F	IL-10	left Dorsal intermediate entorhinal cortex	0.901	0.044
F	IL-10	left Dorsal nucleus of the endopiriform	0.739	0.038
F	IL-10	left Dorsal tenia tecta	0.500	0.024
F	IL-10	left Dorsolateral entorhinal cortex	1.199	0.058
F	IL-10	left Dorsolateral orbital cortex	0.443	0.030
F	IL-10	left Ectorhinal cortex	1.286	0.072
F	IL-10	left facial nerve (cranial nerve 7)	0.108	0.007
F	IL-10	left fasciculus retroflexus	0.134	0.007
F	IL-10	left fastigial nucleus	0.253	0.010
F	IL-10	left fimbria	1.690	0.063
F	IL-10	left flocculus (FL)	0.572	0.035
F	IL-10	left flocculus white matter	0.032	0.002
F	IL-10	left fornix	0.341	0.018
F	IL-10	left Frontal association cortex	3.436	0.209
F	IL-10	left Frontal cortex: area 3	0.365	0.020
F	IL-10	left fundus of striatum	0.081	0.005
F	IL-10	left globus pallidus	1.663	0.075
F	IL-10	left GrDG	0.699	0.022
F	IL-10	left habenular commissure	0.014	0.002
F	IL-10	left hypothalamus	5.247	0.140
F	IL-10	left inferior olivary complex	0.153	0.021
F	IL-10	left Insular region: not subdivided	3.858	0.184
F	IL-10	left Intermediate nucleus of the endopiriform claustrum	0.322	0.014
F	IL-10	left internal capsule	1.474	0.052
F	IL-10	left lateral olfactory tract	0.766	0.035
F	IL-10	left Lateral orbital cortex	1.846	0.071
F	IL-10	left Lateral parietal association cortex	0.151	0.011
F	IL-10	left lateral septum	1.526	0.059
F	IL-10	left lateral ventricle	1.755	0.137
F	IL-10	left LMol	1.205	0.030
F	IL-10	left mammillary bodies	0.242	0.016
F	IL-10	left mammilothalamic tract	0.129	0.007
F	IL-10	left Medial amygdala	0.499	0.019
F	IL-10	left Medial entorhinal cortex	0.374	0.022
F	IL-10	left medial lemniscus/medial longitudinal fasciculus	1.268	0.057
F	IL-10	left Medial orbital cortex	0.939	0.047
F	IL-10	left Medial parietal association cortex	0.248	0.026
F	IL-10	left Medial preoptic nucleus	0.097	0.005
F	IL-10	left medial septum	0.654	0.030
F	IL-10	left MoDG	2.227	0.059
F	IL-10	left nucleus accumbens	2.052	0.063
F	IL-10	left nucleus interpositus	0.232	0.015
F	IL-10	left Olfactory bulb: external plexiform layer	3.334	0.096
F	IL-10	left Olfactory bulb: glomerular layer	2.185	0.082
F	IL-10	left Olfactory bulb: granule cell layer	2.358	0.077
F	IL-10	left Olfactory bulb: internal plexiform layer	0.536	0.019
F	IL-10	left Olfactory bulb: mitral cell layer	0.674	0.022
F	IL-10	left olfactory peduncle	1.211	0.048
F	IL-10	left olfactory tubercle	1.654	0.105
F	IL-10	left optic tract	0.776	0.022
F	IL-10	left paraflocculus (PFL)	1.639	0.209
F	IL-10	left paraflocculus white matter	0.143	0.016
F	IL-10	left paramedian lobule	0.081	0.007
F	IL-10	left paramedian lobule (lobule 7)	2.159	0.143
F	IL-10	left Parietal cortex: posterior area: rostral part	0.069	0.007
F	IL-10	left Perirhinal cortex	1.229	0.066
F	IL-10	left Piriform cortex	5.320	0.178
F	IL-10	left PoDG	0.312	0.012
F	IL-10	left pontine nucleus	0.397	0.029

F	IL-10	left Posterolateral cortical amygdaloid area	0.418	0.031
F	IL-10	left Posteromedial cortical amygdaloid area	0.598	0.026
F	IL-10	left pre-para subiculum	1.159	0.057
F	IL-10	left Primary auditory cortex	0.764	0.044
F	IL-10	left Primary motor cortex	3.635	0.138
F	IL-10	left Primary somatosensory cortex	2.120	0.143
F	IL-10	left Primary somatosensory cortex: barrel field	4.850	0.252
F	IL-10	left Primary somatosensory cortex: dysgranular zone	0.181	0.014
F	IL-10	left Primary somatosensory cortex: forelimb region	2.115	0.095
F	IL-10	left Primary somatosensory cortex: hindlimb region	1.529	0.080
F	IL-10	left Primary somatosensory cortex: jaw region	0.287	0.034
F	IL-10	left Primary somatosensory cortex: shoulder region	0.096	0.009
F	IL-10	left Primary somatosensory cortex: trunk region	0.316	0.015
F	IL-10	left Primary somatosensory cortex: upper lip region	2.467	0.129
F	IL-10	left Primary visual cortex	1.237	0.054
F	IL-10	left Primary visual cortex: binocular area	1.029	0.043
F	IL-10	left Primary visual cortex: monocular area	1.188	0.057
F	IL-10	left Rostral amygdalopiriform area	0.198	0.019
F	IL-10	left Secondary auditory cortex: dorsal area	0.769	0.055
F	IL-10	left Secondary auditory cortex: ventral area	0.828	0.046
F	IL-10	left Secondary motor cortex	3.311	0.199
F	IL-10	left Secondary somatosensory cortex	3.498	0.243
F	IL-10	left Secondary visual cortex: lateral area	1.346	0.051
F	IL-10	left Secondary visual cortex: mediolateral area	0.609	0.034
F	IL-10	left Secondary visual cortex: mediomedial area	0.979	0.050
F	IL-10	left simple lobule (lobule 6)	2.558	0.102
F	IL-10	left simple lobule white matter	0.239	0.015
F	IL-10	left SLu	0.389	0.023
F	IL-10	left stria medullaris	0.329	0.013
F	IL-10	left stria terminalis	0.475	0.014
F	IL-10	left striatum	11.060	0.396
F	IL-10	left subependymale zone / rhinocele	0.030	0.002
F	IL-10	left subiculum	1.684	0.047
F	IL-10	left superior olivary complex	0.379	0.015
F	IL-10	left Temporal association area	1.397	0.066
F	IL-10	left thalamus	8.952	0.315
F	IL-10	left trunk of crus 2 and paramedian white matter	0.168	0.014
F	IL-10	left trunk of simple and crus 1 white matter	0.065	0.004
F	IL-10	left Ventral intermediate entorhinal cortex	0.570	0.017
F	IL-10	left Ventral nucleus of the endopiriform claustrum	0.258	0.022
F	IL-10	left Ventral orbital cortex	0.797	0.047
F	IL-10	left Ventral tenia tecta	0.061	0.005
F	IL-10	lobule 1-2 white matter	0.075	0.010
F	IL-10	lobule 10 white matter	0.087	0.008
F	IL-10	lobule 10: nodulus	1.403	0.059
F	IL-10	lobule 3 white matter	0.198	0.014
F	IL-10	lobule 3: central lobule (dorsal)	2.267	0.204
F	IL-10	lobule 6: declive	2.558	0.148
F	IL-10	lobule 7: tuber (or folium)	1.021	0.072
F	IL-10	lobule 8 white matter	0.160	0.010
F	IL-10	lobule 8: pyramis	1.640	0.099
F	IL-10	lobule 9 white matter	0.291	0.017
F	IL-10	lobule 9: uvula	2.823	0.124
F	IL-10	lobules 1-2: lingula and central lobule (ventral)	1.757	0.140
F	IL-10	lobules 4-5 white matter	0.585	0.050
F	IL-10	lobules 4-5: culmen (ventral and dorsal)	4.443	0.307
F	IL-10	lobules 6-7 white matter	0.695	0.036
F	IL-10	medulla	27.738	0.892
F	IL-10	midbrain	14.245	0.548
F	IL-10	periaqueductal grey	3.964	0.145
F	IL-10	pons	17.861	0.591
F	IL-10	posterior commissure	0.150	0.009
F	IL-10	right Accessory olfactory bulb: glomerular, external plexiform and mitral cell layer	0.242	0.011
F	IL-10	right Accessory olfactory bulb: granule cell layer	0.118	0.005
F	IL-10	right amygdala	4.529	0.200
F	IL-10	right Amygdalopiriform transition area	0.497	0.030
F	IL-10	right anterior commissure: pars anterior	0.514	0.015
F	IL-10	right anterior commissure: pars posterior	0.207	0.008
F	IL-10	right anterior lobule (lobules 4-5)	0.820	0.047
F	IL-10	right anterior lobule white matter	0.054	0.004
F	IL-10	right Anterior olfactory nucleus	1.045	0.039
F	IL-10	right basal forebrain	2.695	0.093
F	IL-10	right bed nucleus of stria terminalis	0.663	0.028
F	IL-10	right CA10r	1.039	0.056
F	IL-10	right CA1Py	0.563	0.023
F	IL-10	right CA1Rad	1.351	0.027
F	IL-10	right CA20r	0.222	0.016
F	IL-10	right CA2Py	0.100	0.003
F	IL-10	right CA2Rad	0.222	0.007
F	IL-10	right CA30r	1.417	0.053
F	IL-10	right CA3Py Inner	0.081	0.002
F	IL-10	right CA3Py Outer	0.524	0.023
F	IL-10	right CA3Rad	0.983	0.038
F	IL-10	right Caudomedial entorhinal cortex	2.905	0.116
F	IL-10	right cerebellar peduncle: inferior	0.409	0.013
F	IL-10	right cerebellar peduncle: middle	0.637	0.029
F	IL-10	right cerebellar peduncle: superior	0.510	0.017
F	IL-10	right cerebral peduncle	1.041	0.034
F	IL-10	right Cingulate cortex: area 24a	0.994	0.054
F	IL-10	right Cingulate cortex: area 24a'	0.356	0.033
F	IL-10	right Cingulate cortex: area 24b	0.771	0.076
F	IL-10	right Cingulate cortex: area 24b'	0.299	0.025
F	IL-10	right Cingulate cortex: area 25	0.305	0.013
F	IL-10	right Cingulate cortex: area 29a	0.373	0.016
F	IL-10	right Cingulate cortex: area 29b	0.208	0.017
F	IL-10	right Cingulate cortex: area 29c	0.917	0.037
F	IL-10	right Cingulate cortex: area 30	1.414	0.055
F	IL-10	right Cingulate cortex: area 32	1.104	0.082
F	IL-10	right Cingulum	0.448	0.024
F	IL-10	right Claustrum	0.150	0.007
F	IL-10	right Claustrum: dorsal part	0.144	0.016
F	IL-10	right Claustrum: ventral part	0.308	0.026
F	IL-10	right colliculus: inferior	2.762	0.140
F	IL-10	right colliculus: superior	4.518	0.133
F	IL-10	right copula white matter	0.033	0.002
F	IL-10	right copula: pyramis (lobule 8)	1.258	0.073
F	IL-10	right corpus callosum	5.965	0.216
F	IL-10	right Cortex-amygdala transition zones	0.440	0.034
F	IL-10	right corticospinal tract/pyramids	0.906	0.056
F	IL-10	right crus 1 white matter	0.193	0.009
F	IL-10	right crus 1: ansiform lobule (lobule 6)	2.309	0.100
F	IL-10	right crus 2 white matter	0.156	0.009
F	IL-10	right crus 2: ansiform lobule (lobule 7)	2.142	0.102
F	IL-10	right cuneate nucleus	0.123	0.009
F	IL-10	right dentate nucleus	0.184	0.010
F	IL-10	right Dorsal intermediate entorhinal cortex	0.925	0.035
F	IL-10	right Dorsal nucleus of the endopiriform	0.726	0.041
F	IL-10	right Dorsal tenia tecta	0.459	0.025
F	IL-10	right Dorsolateral entorhinal cortex	1.340	0.092
F	IL-10	right Dorsolateral orbital cortex	0.416	0.032
F	IL-10	right Ectorhinal cortex	1.367	0.107
F	IL-10	right facial nerve (cranial nerve 7)	0.114	0.006
F	IL-10	right fasciculus retroflexus	0.119	0.006
F	IL-10	right fastigial nucleus	0.246	0.014
F	IL-10	right fimbria	1.560	0.071
F	IL-10	right flocculus (FL)	0.528	0.026
F	IL-10	right flocculus white matter	0.038	0.003
F	IL-10	right fornix	0.299	0.014
F	IL-10	right Frontal association cortex	3.774	0.190
F	IL-10	right Frontal cortex: area 3	0.362	0.021
F	IL-10	right fundus of striatum	0.086	0.008
F	IL-10	right globus pallidus	1.666	0.061
F	IL-10	right GrDG	0.657	0.021
F	IL-10	right habenular commissure	0.014	0.002
F	IL-10	right hypothalamus	5.187	0.129
F	IL-10	right inferior olivary complex	0.173	0.026
F	IL-10	right Insular region: not subdivided	4.095	0.302
F	IL-10	right Intermediate nucleus of the endopiriform claustrum	0.292	0.016
F	IL-10	right internal capsule	1.544	0.053
F	IL-10	right lateral olfactory tract	0.678	0.030
F	IL-10	right Lateral orbital cortex	1.801	0.058
F	IL-10	right Lateral parietal association cortex	0.094	0.009
F	IL-10	right lateral septum	1.540	0.054
F	IL-10	right lateral ventricle	1.850	0.191
F	IL-10	right LMol	1.188	0.021
F	IL-10	right mammillary bodies	0.223	0.010
F	IL-10	right mammilothalamic tract	0.133	0.010
F	IL-10	right Medial amygdala	0.519	0.035
F	IL-10	right Medial entorhinal cortex	0.353	0.016
F	IL-10	right medial lemniscus/medial longitudinal fasciculus	1.334	0.044
F	IL-10	right Medial orbital cortex	0.856	0.057
F	IL-10	right Medial parietal association cortex	0.180	0.015
F	IL-10	right Medial preoptic nucleus	0.089	0.004
F	IL-10	right medial septum	0.611	0.020
F	IL-10	right MoDG	2.088	0.060
F	IL-10	right nucleus accumbens	2.027	0.063
F	IL-10	right nucleus interpositus	0.242	0.014
F	IL-10	right Olfactory bulb: external plexiform layer	3.311	0.106
F	IL-10	right Olfactory bulb: glomerular layer	2.259	0.088
F	IL-10	right Olfactory bulb: granule cell layer	2.399	0.089
F	IL-10	right Olfactory bulb: internal plexiform layer	0.520	0.022
F	IL-10	right Olfactory bulb: mitral cell layer	0.625	0.025
F	IL-10	right olfactory peduncle	1.182	0.046
F	IL-10	right olfactory tubercle	1.650	0.053
F	IL-10	right optic tract	0.720	0.025
F	IL-10	right paraflocculus (PFL)	1.775	0.211
F	IL-10	right paraflocculus white matter	0.172	0.017
F	IL-10	right paramedian lobule	0.086	0.005
F	IL-10	right paramedian lobule (lobule 7)	2.120	0.086
F	IL-10	right Parietal cortex: posterior area: rostral part	0.045	0.007
F	IL-10	right Perirhinal cortex	1.194	0.063
F	IL-10	right Piriform cortex	5.566	0.198
F	IL-10	right PoDG	0.327	0.011
F	IL-10	right pontine nucleus	0.387	0.024
F	IL-10	right Posterolateral cortical amygdaloid area	0.457	0.023
F	IL-10	right Posteromedial cortical amygdaloid area	0.469	0.029
F	IL-10	right pre-para subiculum	1.198	0.048
F	IL-10	right Primary auditory cortex	0.759	0.049
F	IL-10	right Primary motor cortex	3.103	0.132
F	IL-10	right Primary somatosensory cortex	2.139	0.101
F	IL-10	right Primary somatosensory cortex: barrel field	4.751	0.163
F	IL-10	right Primary somatosensory cortex: dysgranular zone	0.196	0.011
F	IL-10	right Primary somatosensory cortex: forelimb region	1.864	0.074
F	IL-10	right Primary somatosensory cortex: hindlimb region	1.212	0.064
F	IL-10	right Primary somatosensory cortex: jaw region	0.308	0.034
F	IL-10	right Primary somatosensory cortex: shoulder region	0.070	0.009
F	IL-10	right Primary somatosensory cortex: trunk region	0.205	0.016
F	IL-10	right Primary somatosensory cortex: upper lip region	2.875	0.090
F	IL-10	right Primary visual cortex	1.266	0.081
F	IL-10	right Primary visual cortex: binocular area	1.052	0.073
F	IL-10	right Primary visual cortex: monocular area	0.807	0.054
F	IL-10	right Rostral amygdalopiriform area	0.199	0.015
F	IL-10	right Secondary auditory cortex: dorsal area	0.742	0.047
F	IL-10	right Secondary auditory cortex: ventral area	1.004	0.072
F	IL-10	right Secondary motor cortex	3.208	0.123
F	IL-10	right Secondary somatosensory cortex	3.783	0.298
F	IL-10	right Secondary visual cortex: lateral area	1.531	0.082
F	IL-10	right Secondary visual cortex: mediolateral area	0.416	0.028
F	IL-10	right Secondary visual cortex: mediomedial area	0.823	0.028
F	IL-10	right simple lobule (lobule 6)	2.559	0.094
F	IL-10	right simple lobule white matter	0.192	0.009
F	IL-10	right SLu	0.385	0.021
F	IL-10	right stria medullaris	0.352	0.014
F	IL-10	right stria terminalis	0.398	0.015
F	IL-10	right striatum	10.904	0.408
F	IL-10	right subependymale zone / rhinocele	0.028	0.002
F	IL-10	right subiculum	1.680	0.051
F	IL-10	right superior olivary complex	0.404	0.025
F	IL-10	right Temporal association area	1.568	0.087
F	IL-10	right thalamus	8.883	0.316
F	IL-10	right trunk of crus 2 and paramedian white matter	0.171	0.010
F	IL-10	right trunk of simple and crus 1 white matter	0.080	0.006
F	IL-10	right Ventral intermediate entorhinal cortex	0.576	0.027
F	IL-10	right Ventral nucleus of the endopiriform claustrum	0.234	0.017
F	IL-10	right Ventral orbital cortex	0.723	0.035
F	IL-10	right Ventral tenia tecta	0.062	0.006
F	IL-10	third ventricle	1.237	0.055
F	IL-10	trunk of arbor vita	4.286	0.140
F	IL-10	trunk of lobules 1-3 white matter	0.135	0.009
F	IL-10	trunk of lobules 6-8 white matter	0.113	0.007
F	IL-10	ventral tegmental decussation	0.137	0.010
F	IL-18	cerebral aqueduct	0.503	0.036
F	IL-18	fourth ventricle	0.967	0.063
F	IL-18	interpedunclar nucleus	0.287	0.016
F	IL-18	left Accessory olfactory bulb: glomerular, external plexiform and mitral cell layer	0.261	0.013
F	IL-18	left Accessory olfactory bulb: granule cell layer	0.132	0.007
F	IL-18	left amygdala	4.889	0.174
F	IL-18	left Amygdalopiriform transition area	0.547	0.031
F	IL-18	left anterior commissure: pars anterior	0.515	0.016
F	IL-18	left anterior commissure: pars posterior	0.227	0.007
F	IL-18	left anterior lobule (lobules 4-5)	0.978	0.073
F	IL-18	left anterior lobule white matter	0.048	0.004
F	IL-18	left Anterior olfactory nucleus	1.003	0.032
F	IL-18	left basal forebrain	2.694	0.053
F	IL-18	left bed nucleus of stria terminalis	0.660	0.022
F	IL-18	left CA10r	1.227	0.042
F	IL-18	left CA1Py	0.584	0.020
F	IL-18	left CA1Rad	1.293	0.026
F	IL-18	left CA20r	0.276	0.015
F	IL-18	left CA2Py	0.109	0.004
F	IL-18	left CA2Rad	0.220	0.010
F	IL-18	left CA30r	1.470	0.070
F	IL-18	left CA3Py Inner	0.083	0.003
F	IL-18	left CA3Py Outer	0.543	0.019
F	IL-18	left CA3Rad	0.971	0.034
F	IL-18	left Caudomedial entorhinal cortex	2.976	0.109
F	IL-18	left cerebellar peduncle: inferior	0.458	0.016
F	IL-18	left cerebellar peduncle: middle	0.634	0.027
F	IL-18	left cerebellar peduncle: superior	0.533	0.021
F	IL-18	left cerebral peduncle	1.080	0.039
F	IL-18	left Cingulate cortex: area 24a	0.981	0.048
F	IL-18	left Cingulate cortex: area 24a'	0.394	0.052
F	IL-18	left Cingulate cortex: area 24b	0.815	0.048
F	IL-18	left Cingulate cortex: area 24b'	0.317	0.025
F	IL-18	left Cingulate cortex: area 25	0.300	0.014
F	IL-18	left Cingulate cortex: area 29a	0.412	0.017
F	IL-18	left Cingulate cortex: area 29b	0.228	0.012
F	IL-18	left Cingulate cortex: area 29c	0.999	0.062
F	IL-18	left Cingulate cortex: area 30	1.543	0.047
F	IL-18	left Cingulate cortex: area 32	1.412	0.079
F	IL-18	left Cingulum	0.452	0.025
F	IL-18	left Claustrum	0.184	0.007
F	IL-18	left Claustrum: dorsal part	0.154	0.013
F	IL-18	left Claustrum: ventral part	0.284	0.023
F	IL-18	left colliculus: inferior	3.044	0.088
F	IL-18	left colliculus: superior	4.621	0.166
F	IL-18	left copula white matter	0.052	0.004

F	IL-18	left copula: pyramis (lobule 8)	1.384	0.109
F	IL-18	left corpus callosum	6.403	0.151
F	IL-18	left Cortex-amygdala transition zones	0.393	0.031
F	IL-18	left corticospinal tract/pyramids	0.882	0.040
F	IL-18	left crus 1 white matter	0.208	0.009
F	IL-18	left crus 1: ansiform lobule (lobule 6)	2.393	0.097
F	IL-18	left crus 2 white matter	0.145	0.011
F	IL-18	left crus 2: ansiform lobule (lobule 7)	2.233	0.083
F	IL-18	left cuneate nucleus	0.130	0.009
F	IL-18	left dentate nucleus	0.193	0.008
F	IL-18	left Dorsal intermediate entorhinal cortex	0.877	0.037
F	IL-18	left Dorsal nucleus of the endopiriform	0.738	0.028
F	IL-18	left Dorsal tenia tecta	0.499	0.032
F	IL-18	left Dorsolateral entorhinal cortex	1.231	0.056
F	IL-18	left Dorsolateral orbital cortex	0.465	0.039
F	IL-18	left Ectorhinal cortex	1.330	0.075
F	IL-18	left facial nerve (cranial nerve 7)	0.111	0.006
F	IL-18	left fasciculus retroflexus	0.136	0.008
F	IL-18	left fastigial nucleus	0.260	0.010
F	IL-18	left fimbria	1.721	0.071
F	IL-18	left flocculus (FL)	0.599	0.036
F	IL-18	left flocculus white matter	0.032	0.004
F	IL-18	left fornix	0.344	0.011
F	IL-18	left Frontal association cortex	3.749	0.141
F	IL-18	left Frontal cortex: area 3	0.371	0.018
F	IL-18	left fundus of striatum	0.081	0.006
F	IL-18	left globus pallidus	1.765	0.042
F	IL-18	left GrDG	0.712	0.018
F	IL-18	left habenular commissure	0.015	0.003
F	IL-18	left hypothalamus	5.361	0.106
F	IL-18	left inferior olivary complex	0.167	0.022
F	IL-18	left Insular region: not subdivided	3.890	0.180
F	IL-18	left Intermediate nucleus of the endopiriform claustrum	0.319	0.018
F	IL-18	left internal capsule	1.525	0.039
F	IL-18	left lateral olfactory tract	0.746	0.024
F	IL-18	left Lateral orbital cortex	1.915	0.088
F	IL-18	left Lateral parietal association cortex	0.154	0.013
F	IL-18	left lateral septum	1.611	0.041
F	IL-18	left lateral ventricle	1.752	0.120
F	IL-18	left LMol	1.227	0.028
F	IL-18	left mammillary bodies	0.252	0.020
F	IL-18	left mammilothalamic tract	0.130	0.007
F	IL-18	left Medial amygdala	0.498	0.019
F	IL-18	left Medial entorhinal cortex	0.358	0.016
F	IL-18	left medial lemniscus/medial longitudinal fasciculus	1.299	0.042
F	IL-18	left Medial orbital cortex	0.982	0.042
F	IL-18	left Medial parietal association cortex	0.242	0.015
F	IL-18	left Medial preoptic nucleus	0.097	0.004
F	IL-18	left medial septum	0.664	0.018
F	IL-18	left MoDG	2.274	0.053
F	IL-18	left nucleus accumbens	2.219	0.036
F	IL-18	left nucleus interpositus	0.244	0.012
F	IL-18	left Olfactory bulb: external plexiform layer	3.325	0.066
F	IL-18	left Olfactory bulb: glomerular layer	2.226	0.056
F	IL-18	left Olfactory bulb: granule cell layer	2.299	0.068
F	IL-18	left Olfactory bulb: internal plexiform layer	0.528	0.015
F	IL-18	left Olfactory bulb: mitral cell layer	0.661	0.017
F	IL-18	left olfactory peduncle	1.174	0.027
F	IL-18	left olfactory tubercle	1.668	0.052
F	IL-18	left optic tract	0.799	0.021
F	IL-18	left paraflocculus (PFL)	1.709	0.250
F	IL-18	left paraflocculus white matter	0.150	0.017
F	IL-18	left paramedian lobule	0.084	0.006
F	IL-18	left paramedian lobule (lobule 7)	2.246	0.103
F	IL-18	left Parietal cortex: posterior area: rostral part	0.076	0.005
F	IL-18	left Perirhinal cortex	1.265	0.054
F	IL-18	left Piriform cortex	5.101	0.217
F	IL-18	left PoDG	0.318	0.013
F	IL-18	left pontine nucleus	0.434	0.041
F	IL-18	left Posterolateral cortical amygdaloid area	0.418	0.028
F	IL-18	left Posteromedial cortical amygdaloid area	0.604	0.035
F	IL-18	left pre-para subiculum	1.190	0.042
F	IL-18	left Primary auditory cortex	0.783	0.046
F	IL-18	left Primary motor cortex	3.786	0.191
F	IL-18	left Primary somatosensory cortex	2.095	0.074
F	IL-18	left Primary somatosensory cortex: barrel field	4.822	0.151
F	IL-18	left Primary somatosensory cortex: dysgranular zone	0.176	0.012
F	IL-18	left Primary somatosensory cortex: forelimb region	2.109	0.088
F	IL-18	left Primary somatosensory cortex: hindlimb region	1.504	0.071
F	IL-18	left Primary somatosensory cortex: jaw region	0.278	0.021
F	IL-18	left Primary somatosensory cortex: shoulder region	0.096	0.010
F	IL-18	left Primary somatosensory cortex: trunk region	0.323	0.022
F	IL-18	left Primary somatosensory cortex: upper lip region	2.425	0.087
F	IL-18	left Primary visual cortex	1.225	0.053
F	IL-18	left Primary visual cortex: binocular area	1.067	0.043
F	IL-18	left Primary visual cortex: monocular area	1.223	0.065
F	IL-18	left Rostral amygdalopiriform area	0.197	0.015
F	IL-18	left Secondary auditory cortex: dorsal area	0.768	0.044
F	IL-18	left Secondary auditory cortex: ventral area	0.821	0.059
F	IL-18	left Secondary motor cortex	3.463	0.134
F	IL-18	left Secondary somatosensory cortex	3.480	0.225
F	IL-18	left Secondary visual cortex: lateral area	1.349	0.060
F	IL-18	left Secondary visual cortex: mediolateral area	0.609	0.041
F	IL-18	left Secondary visual cortex: mediomedial area	0.991	0.033
F	IL-18	left simple lobule (lobule 6)	2.601	0.086
F	IL-18	left simple lobule white matter	0.236	0.015
F	IL-18	left SLu	0.397	0.014
F	IL-18	left stria medullaris	0.335	0.013
F	IL-18	left stria terminalis	0.483	0.016
F	IL-18	left striatum	11.597	0.229
F	IL-18	left subependymale zone / rhinocele	0.029	0.002
F	IL-18	left subiculum	1.735	0.056
F	IL-18	left superior olivary complex	0.397	0.017
F	IL-18	left Temporal association area	1.409	0.078
F	IL-18	left thalamus	9.334	0.312
F	IL-18	left trunk of crus 2 and paramedian white matter	0.178	0.012
F	IL-18	left trunk of simple and crus 1 white matter	0.074	0.005
F	IL-18	left Ventral intermediate entorhinal cortex	0.578	0.029
F	IL-18	left Ventral nucleus of the endopiriform claustrum	0.254	0.018
F	IL-18	left Ventral orbital cortex	0.809	0.035
F	IL-18	left Ventral tenia tecta	0.053	0.003
F	IL-18	lobule 1-2 white matter	0.077	0.013
F	IL-18	lobule 10 white matter	0.097	0.005
F	IL-18	lobule 10: nodulus	1.560	0.052
F	IL-18	lobule 3 white matter	0.225	0.017
F	IL-18	lobule 3: central lobule (dorsal)	2.470	0.165
F	IL-18	lobule 6: declive	2.686	0.131
F	IL-18	lobule 7: tuber (or folium)	1.043	0.062
F	IL-18	lobule 8 white matter	0.175	0.007
F	IL-18	lobule 8: pyramis	1.774	0.065
F	IL-18	lobule 9 white matter	0.331	0.015
F	IL-18	lobule 9: uvula	3.105	0.143
F	IL-18	lobules 1-2: lingula and central lobule (ventral)	1.839	0.194
F	IL-18	lobules 4-5 white matter	0.640	0.041
F	IL-18	lobules 4-5: culmen (ventral and dorsal)	4.735	0.246
F	IL-18	lobules 6-7 white matter	0.731	0.031
F	IL-18	medulla	28.527	0.727
F	IL-18	midbrain	14.522	0.552
F	IL-18	periaqueductal grey	4.081	0.130
F	IL-18	pons	18.039	0.513
F	IL-18	posterior commissure	0.149	0.008
F	IL-18	right Accessory olfactory bulb: glomerular, external plexiform and mitral cell layer	0.249	0.012
F	IL-18	right Accessory olfactory bulb: granule cell layer	0.123	0.006
F	IL-18	right amygdala	4.603	0.136
F	IL-18	right Amygdalopiriform transition area	0.508	0.023
F	IL-18	right anterior commissure: pars anterior	0.503	0.015
F	IL-18	right anterior commissure: pars posterior	0.218	0.011
F	IL-18	right anterior lobule (lobules 4-5)	0.892	0.061
F	IL-18	right anterior lobule white matter	0.059	0.006
F	IL-18	right Anterior olfactory nucleus	1.023	0.044
F	IL-18	right basal forebrain	2.788	0.076
F	IL-18	right bed nucleus of stria terminalis	0.657	0.014
F	IL-18	right CA10r	1.079	0.045
F	IL-18	right CA1Py	0.590	0.018
F	IL-18	right CA1Rad	1.398	0.035
F	IL-18	right CA20r	0.229	0.018
F	IL-18	right CA2Py	0.101	0.003
F	IL-18	right CA2Rad	0.221	0.012
F	IL-18	right CA30r	1.482	0.069
F	IL-18	right CA3Py Inner	0.083	0.003
F	IL-18	right CA3Py Outer	0.541	0.023
F	IL-18	right CA3Rad	0.999	0.037
F	IL-18	right Caudomedial entorhinal cortex	2.867	0.125
F	IL-18	right cerebellar peduncle: inferior	0.418	0.015
F	IL-18	right cerebellar peduncle: middle	0.658	0.035
F	IL-18	right cerebellar peduncle: superior	0.513	0.026
F	IL-18	right cerebral peduncle	1.082	0.037
F	IL-18	right Cingulate cortex: area 24a	1.043	0.055
F	IL-18	right Cingulate cortex: area 24a'	0.369	0.046
F	IL-18	right Cingulate cortex: area 24b	0.800	0.046
F	IL-18	right Cingulate cortex: area 24b'	0.308	0.026
F	IL-18	right Cingulate cortex: area 25	0.313	0.019
F	IL-18	right Cingulate cortex: area 29a	0.388	0.020
F	IL-18	right Cingulate cortex: area 29b	0.216	0.018
F	IL-18	right Cingulate cortex: area 29c	0.941	0.047
F	IL-18	right Cingulate cortex: area 30	1.437	0.072
F	IL-18	right Cingulate cortex: area 32	1.121	0.048
F	IL-18	right Cingulum	0.457	0.020
F	IL-18	right Claustrum	0.152	0.008
F	IL-18	right Claustrum: dorsal part	0.140	0.016
F	IL-18	right Claustrum: ventral part	0.315	0.026
F	IL-18	right colliculus: inferior	2.865	0.089
F	IL-18	right colliculus: superior	4.699	0.159
F	IL-18	right copula white matter	0.042	0.003
F	IL-18	right copula: pyramis (lobule 8)	1.494	0.117
F	IL-18	right corpus callosum	6.089	0.219
F	IL-18	right Cortex-amygdala transition zones	0.407	0.026
F	IL-18	right corticospinal tract/pyramids	0.931	0.038
F	IL-18	right crus 1 white matter	0.196	0.010
F	IL-18	right crus 1: ansiform lobule (lobule 6)	2.365	0.091
F	IL-18	right crus 2 white matter	0.161	0.009
F	IL-18	right crus 2: ansiform lobule (lobule 7)	2.199	0.110
F	IL-18	right cuneate nucleus	0.133	0.010
F	IL-18	right dentate nucleus	0.187	0.008
F	IL-18	right Dorsal intermediate entorhinal cortex	0.923	0.048
F	IL-18	right Dorsal nucleus of the endopiriform	0.733	0.023
F	IL-18	right Dorsal tenia tecta	0.458	0.028
F	IL-18	right Dorsolateral entorhinal cortex	1.328	0.065
F	IL-18	right Dorsolateral orbital cortex	0.428	0.028
F	IL-18	right Ectorhinal cortex	1.380	0.103
F	IL-18	right facial nerve (cranial nerve 7)	0.116	0.006
F	IL-18	right fasciculus retroflexus	0.122	0.008
F	IL-18	right fastigial nucleus	0.250	0.012
F	IL-18	right fimbria	1.592	0.075
F	IL-18	right flocculus (FL)	0.553	0.038
F	IL-18	right flocculus white matter	0.041	0.004
F	IL-18	right fornix	0.304	0.010
F	IL-18	right Frontal association cortex	4.081	0.170
F	IL-18	right Frontal cortex: area 3	0.373	0.014
F	IL-18	right fundus of striatum	0.086	0.007
F	IL-18	right globus pallidus	1.775	0.039
F	IL-18	right GrDG	0.669	0.023
F	IL-18	right habenular commissure	0.014	0.002
F	IL-18	right hypothalamus	5.310	0.109
F	IL-18	right inferior olivary complex	0.184	0.016
F	IL-18	right Insular region: not subdivided	4.117	0.237
F	IL-18	right Intermediate nucleus of the endopiriform claustrum	0.299	0.009
F	IL-18	right internal capsule	1.593	0.050
F	IL-18	right lateral olfactory tract	0.666	0.019
F	IL-18	right Lateral orbital cortex	1.857	0.030
F	IL-18	right Lateral parietal association cortex	0.092	0.008
F	IL-18	right lateral septum	1.621	0.037
F	IL-18	right lateral ventricle	1.908	0.140
F	IL-18	right LMol	1.197	0.033
F	IL-18	right mammillary bodies	0.234	0.022
F	IL-18	right mammilothalamic tract	0.139	0.010
F	IL-18	right Medial amygdala	0.522	0.027
F	IL-18	right Medial entorhinal cortex	0.343	0.020
F	IL-18	right medial lemniscus/medial longitudinal fasciculus	1.377	0.032
F	IL-18	right Medial orbital cortex	0.867	0.034
F	IL-18	right Medial parietal association cortex	0.178	0.014
F	IL-18	right Medial preoptic nucleus	0.091	0.005
F	IL-18	right medial septum	0.624	0.023
F	IL-18	right MoDG	2.120	0.059
F	IL-18	right nucleus accumbens	2.228	0.045
F	IL-18	right nucleus interpositus	0.251	0.013
F	IL-18	right Olfactory bulb: external plexiform layer	3.339	0.070
F	IL-18	right Olfactory bulb: glomerular layer	2.310	0.060
F	IL-18	right Olfactory bulb: granule cell layer	2.361	0.090
F	IL-18	right Olfactory bulb: internal plexiform layer	0.517	0.029
F	IL-18	right Olfactory bulb: mitral cell layer	0.621	0.025
F	IL-18	right olfactory peduncle	1.145	0.035
F	IL-18	right olfactory tubercle	1.703	0.057
F	IL-18	right optic tract	0.741	0.024
F	IL-18	right paraflocculus (PFL)	1.694	0.266
F	IL-18	right paraflocculus white matter	0.166	0.023
F	IL-18	right paramedian lobule	0.094	0.007
F	IL-18	right paramedian lobule (lobule 7)	2.242	0.101
F	IL-18	right Parietal cortex: posterior area: rostral part	0.044	0.005
F	IL-18	right Perirhinal cortex	1.221	0.065
F	IL-18	right Piriform cortex	5.420	0.145
F	IL-18	right PoDG	0.331	0.011
F	IL-18	right pontine nucleus	0.422	0.031
F	IL-18	right Posterolateral cortical amygdaloid area	0.464	0.022
F	IL-18	right Posteromedial cortical amygdaloid area	0.484	0.027
F	IL-18	right pre-para subiculum	1.216	0.035
F	IL-18	right Primary auditory cortex	0.744	0.035
F	IL-18	right Primary motor cortex	3.242	0.109
F	IL-18	right Primary somatosensory cortex	2.118	0.116
F	IL-18	right Primary somatosensory cortex: barrel field	4.728	0.220
F	IL-18	right Primary somatosensory cortex: dysgranular zone	0.188	0.010
F	IL-18	right Primary somatosensory cortex: forelimb region	1.843	0.087
F	IL-18	right Primary somatosensory cortex: hindlimb region	1.228	0.057
F	IL-18	right Primary somatosensory cortex: jaw region	0.288	0.021
F	IL-18	right Primary somatosensory cortex: shoulder region	0.064	0.010
F	IL-18	right Primary somatosensory cortex: trunk region	0.204	0.013
F	IL-18	right Primary somatosensory cortex: upper lip region	2.796	0.105
F	IL-18	right Primary visual cortex	1.229	0.075
F	IL-18	right Primary visual cortex: binocular area	1.062	0.107
F	IL-18	right Primary visual cortex: monocular area	0.812	0.057
F	IL-18	right Rostral amygdalopiriform area	0.207	0.016
F	IL-18	right Secondary auditory cortex: dorsal area	0.707	0.031
F	IL-18	right Secondary auditory cortex: ventral area	1.000	0.076
F	IL-18	right Secondary motor cortex	3.367	0.090
F	IL-18	right Secondary somatosensory cortex	3.664	0.305
F	IL-18	right Secondary visual cortex: lateral area	1.516	0.117
F	IL-18	right Secondary visual cortex: mediolateral area	0.406	0.022

F	IL-18	right Secondary visual cortex: mediomedial area	0.818	0.036
F	IL-18	right simple lobule (lobule 6)	2.622	0.092
F	IL-18	right simple lobule white matter	0.198	0.013
F	IL-18	right SLu	0.393	0.017
F	IL-18	right stria medullaris	0.354	0.013
F	IL-18	right stria terminalis	0.401	0.012
F	IL-18	right striatum	11.436	0.272
F	IL-18	right subependymale zone / rhinocele	0.026	0.001
F	IL-18	right subiculum	1.713	0.064
F	IL-18	right superior olivary complex	0.415	0.021
F	IL-18	right Temporal association area	1.585	0.110
F	IL-18	right thalamus	9.208	0.345
F	IL-18	right trunk of crus 2 and paramedian white matter	0.187	0.014
F	IL-18	right trunk of simple and crus 1 white matter	0.091	0.006
F	IL-18	right Ventral intermediate entorhinal cortex	0.562	0.030
F	IL-18	right Ventral nucleus of the endopiriform claustrum	0.234	0.014
F	IL-18	right Ventral orbital cortex	0.717	0.030
F	IL-18	right Ventral tenia tecta	0.056	0.003
F	IL-18	third ventricle	1.240	0.046
F	IL-18	trunk of arbor vita	4.520	0.130
F	IL-18	trunk of lobules 1-3 white matter	0.143	0.007
F	IL-18	trunk of lobules 6-8 white matter	0.114	0.008
F	IL-18	ventral tegmental decussation	0.138	0.007
F	Kit	cerebral aqueduct	0.553	0.067
F	Kit	fourth ventricle	0.988	0.083
F	Kit	interpedunclar nucleus	0.273	0.014
F	Kit	left Accessory olfactory bulb: glomerular, external plexiform and mitral cell layer	0.268	0.012
F	Kit	left Accessory olfactory bulb: granule cell layer	0.115	0.006
F	Kit	left amygdala	4.825	0.183
F	Kit	left Amygdalopiriform transition area	0.517	0.039
F	Kit	left anterior commissure: pars anterior	0.469	0.013
F	Kit	left anterior commissure: pars posterior	0.202	0.008
F	Kit	left anterior lobule (lobules 4-5)	0.834	0.033
F	Kit	left anterior lobule white matter	0.042	0.002
F	Kit	left Anterior olfactory nucleus	0.995	0.031
F	Kit	left basal forebrain	2.543	0.083
F	Kit	left bed nucleus of stria terminalis	0.625	0.028
F	Kit	left CA10r	1.106	0.164
F	Kit	left CA1Py	0.527	0.056
F	Kit	left CA1Rad	1.237	0.257
F	Kit	left CA20r	0.261	0.057
F	Kit	left CA2Py	0.109	0.032
F	Kit	left CA2Rad	0.219	0.062
F	Kit	left CA30r	1.486	0.165
F	Kit	left CA3Py Inner	0.082	0.022
F	Kit	left CA3Py Outer	0.518	0.056
F	Kit	left CA3Rad	0.930	0.185
F	Kit	left Caudomedial entorhinal cortex	2.925	0.104
F	Kit	left cerebellar peduncle: inferior	0.418	0.019
F	Kit	left cerebellar peduncle: middle	0.562	0.032
F	Kit	left cerebellar peduncle: superior	0.509	0.024
F	Kit	left cerebral peduncle	1.015	0.042
F	Kit	left Cingulate cortex: area 24a	0.986	0.077
F	Kit	left Cingulate cortex: area 24a'	0.362	0.033
F	Kit	left Cingulate cortex: area 24b	0.795	0.092
F	Kit	left Cingulate cortex: area 24b'	0.309	0.028
F	Kit	left Cingulate cortex: area 25	0.316	0.033
F	Kit	left Cingulate cortex: area 29a	0.354	0.025
F	Kit	left Cingulate cortex: area 29b	0.209	0.013
F	Kit	left Cingulate cortex: area 29c	0.929	0.040
F	Kit	left Cingulate cortex: area 30	1.462	0.092
F	Kit	left Cingulate cortex: area 32	1.383	0.092
F	Kit	left Cingulum	0.417	0.018
F	Kit	left Claustrum	0.171	0.018
F	Kit	left Claustrum: dorsal part	0.145	0.014
F	Kit	left Claustrum: ventral part	0.262	0.026
F	Kit	left colliculus: inferior	2.781	0.094
F	Kit	left colliculus: superior	4.187	0.163
F	Kit	left copula white matter	0.043	0.003
F	Kit	left copula: pyramis (lobule 8)	1.175	0.065
F	Kit	left corpus callosum	5.764	0.193
F	Kit	left Cortex-amygdala transition zones	0.412	0.017
F	Kit	left corticospinal tract/pyramids	0.900	0.042
F	Kit	left crus 1 white matter	0.196	0.010
F	Kit	left crus 1: ansiform lobule (lobule 6)	2.212	0.113
F	Kit	left crus 2 white matter	0.134	0.010
F	Kit	left crus 2: ansiform lobule (lobule 7)	2.029	0.118
F	Kit	left cuneate nucleus	0.119	0.009
F	Kit	left dentate nucleus	0.173	0.008
F	Kit	left Dorsal intermediate entorhinal cortex	0.869	0.059
F	Kit	left Dorsal nucleus of the endopiriform	0.749	0.033
F	Kit	left Dorsal tenia tecta	0.498	0.027
F	Kit	left Dorsolateral entorhinal cortex	1.139	0.074
F	Kit	left Dorsolateral orbital cortex	0.424	0.026
F	Kit	left Ectorhinal cortex	1.219	0.100
F	Kit	left facial nerve (cranial nerve 7)	0.102	0.005
F	Kit	left fasciculus retroflexus	0.118	0.005
F	Kit	left fastigial nucleus	0.229	0.012
F	Kit	left fimbria	1.677	0.131
F	Kit	left flocculus (FL)	0.543	0.025
F	Kit	left flocculus white matter	0.030	0.003
F	Kit	left fornix	0.318	0.017
F	Kit	left Frontal association cortex	3.506	0.207
F	Kit	left Frontal cortex: area 3	0.349	0.018
F	Kit	left fundus of striatum	0.074	0.004
F	Kit	left globus pallidus	1.532	0.050
F	Kit	left GrDG	0.669	0.118
F	Kit	left habenular commissure	0.018	0.002
F	Kit	left hypothalamus	5.182	0.168
F	Kit	left inferior olivary complex	0.171	0.023
F	Kit	left Insular region: not subdivided	3.778	0.209
F	Kit	left Intermediate nucleus of the endopiriform claustrum	0.308	0.012
F	Kit	left internal capsule	1.336	0.046
F	Kit	left lateral olfactory tract	0.720	0.025
F	Kit	left Lateral orbital cortex	1.782	0.084
F	Kit	left Lateral parietal association cortex	0.150	0.018
F	Kit	left lateral septum	1.553	0.151
F	Kit	left lateral ventricle	2.388	0.652
F	Kit	left LMol	1.187	0.218
F	Kit	left mammillary bodies	0.245	0.026
F	Kit	left mammilothalamic tract	0.114	0.006
F	Kit	left Medial amygdala	0.493	0.026
F	Kit	left Medial entorhinal cortex	0.345	0.020
F	Kit	left medial lemniscus/medial longitudinal fasciculus	1.239	0.033
F	Kit	left Medial orbital cortex	0.940	0.049
F	Kit	left Medial parietal association cortex	0.222	0.024
F	Kit	left Medial preoptic nucleus	0.094	0.006
F	Kit	left medial septum	0.652	0.018
F	Kit	left MoDG	2.151	0.319
F	Kit	left nucleus accumbens	2.086	0.063
F	Kit	left nucleus interpositus	0.218	0.011
F	Kit	left Olfactory bulb: external plexiform layer	3.010	0.108
F	Kit	left Olfactory bulb: glomerular layer	1.998	0.132
F	Kit	left Olfactory bulb: granule cell layer	2.182	0.105
F	Kit	left Olfactory bulb: internal plexiform layer	0.497	0.018
F	Kit	left Olfactory bulb: mitral cell layer	0.620	0.014
F	Kit	left olfactory peduncle	1.119	0.037
F	Kit	left olfactory tubercle	1.628	0.093
F	Kit	left optic tract	0.760	0.037
F	Kit	left paraflocculus (PFL)	1.643	0.157
F	Kit	left paraflocculus white matter	0.142	0.012
F	Kit	left paramedian lobule	0.074	0.006
F	Kit	left paramedian lobule (lobule 7)	2.037	0.089
F	Kit	left Parietal cortex: posterior area: rostral part	0.070	0.007
F	Kit	left Perirhinal cortex	1.185	0.114
F	Kit	left Piriform cortex	5.152	0.186
F	Kit	left PoDG	0.303	0.071
F	Kit	left pontine nucleus	0.412	0.053
F	Kit	left Posterolateral cortical amygdaloid area	0.399	0.025
F	Kit	left Posteromedial cortical amygdaloid area	0.570	0.025
F	Kit	left pre-para subiculum	1.093	0.062
F	Kit	left Primary auditory cortex	0.725	0.058
F	Kit	left Primary motor cortex	3.760	0.151
F	Kit	left Primary somatosensory cortex	1.945	0.108
F	Kit	left Primary somatosensory cortex: barrel field	4.728	0.236
F	Kit	left Primary somatosensory cortex: dysgranular zone	0.159	0.014
F	Kit	left Primary somatosensory cortex: forelimb region	2.072	0.123
F	Kit	left Primary somatosensory cortex: hindlimb region	1.510	0.083
F	Kit	left Primary somatosensory cortex: jaw region	0.248	0.020
F	Kit	left Primary somatosensory cortex: shoulder region	0.086	0.009
F	Kit	left Primary somatosensory cortex: trunk region	0.297	0.026
F	Kit	left Primary somatosensory cortex: upper lip region	2.282	0.169
F	Kit	left Primary visual cortex	1.185	0.056
F	Kit	left Primary visual cortex: binocular area	1.012	0.060
F	Kit	left Primary visual cortex: monocular area	1.159	0.086
F	Kit	left Rostral amygdalopiriform area	0.194	0.016
F	Kit	left Secondary auditory cortex: dorsal area	0.726	0.064
F	Kit	left Secondary auditory cortex: ventral area	0.740	0.076
F	Kit	left Secondary motor cortex	3.371	0.173
F	Kit	left Secondary somatosensory cortex	3.281	0.242
F	Kit	left Secondary visual cortex: lateral area	1.277	0.082
F	Kit	left Secondary visual cortex: mediolateral area	0.572	0.035
F	Kit	left Secondary visual cortex: mediomedial area	0.941	0.051
F	Kit	left simple lobule (lobule 6)	2.399	0.142
F	Kit	left simple lobule white matter	0.226	0.018
F	Kit	left SLu	0.351	0.022
F	Kit	left stria medullaris	0.315	0.012
F	Kit	left stria terminalis	0.447	0.019
F	Kit	left striatum	10.372	0.386
F	Kit	left subependymale zone / rhinocele	0.027	0.001
F	Kit	left subiculum	1.563	0.072
F	Kit	left superior olivary complex	0.380	0.029
F	Kit	left Temporal association area	1.288	0.084
F	Kit	left thalamus	8.306	0.293
F	Kit	left trunk of crus 2 and paramedian white matter	0.161	0.009
F	Kit	left trunk of simple and crus 1 white matter	0.064	0.003
F	Kit	left Ventral intermediate entorhinal cortex	0.535	0.042
F	Kit	left Ventral nucleus of the endopiriform claustrum	0.256	0.018
F	Kit	left Ventral orbital cortex	0.745	0.029
F	Kit	left Ventral tenia tecta	0.057	0.004
F	Kit	lobule 1-2 white matter	0.077	0.013
F	Kit	lobule 10 white matter	0.082	0.003
F	Kit	lobule 10: nodulus	1.444	0.067
F	Kit	lobule 3 white matter	0.193	0.011
F	Kit	lobule 3: central lobule (dorsal)	2.141	0.151
F	Kit	lobule 6: declive	2.541	0.182
F	Kit	lobule 7: tuber (or folium)	0.961	0.106
F	Kit	lobule 8 white matter	0.153	0.009
F	Kit	lobule 8: pyramis	1.551	0.080
F	Kit	lobule 9 white matter	0.283	0.017
F	Kit	lobule 9: uvula	2.760	0.151
F	Kit	lobules 1-2: lingula and central lobule (ventral)	1.752	0.180
F	Kit	lobules 4-5 white matter	0.542	0.035
F	Kit	lobules 4-5: culmen (ventral and dorsal)	4.088	0.213
F	Kit	lobules 6-7 white matter	0.692	0.055
F	Kit	medulla	27.191	0.682
F	Kit	midbrain	13.549	0.399
F	Kit	periaqueductal grey	3.818	0.143
F	Kit	pons	16.766	0.521
F	Kit	posterior commissure	0.140	0.007
F	Kit	right Accessory olfactory bulb: glomerular, external plexiform and mitral cell layer	0.262	0.013
F	Kit	right Accessory olfactory bulb: granule cell layer	0.110	0.007
F	Kit	right amygdala	4.599	0.158
F	Kit	right Amygdalopiriform transition area	0.496	0.031
F	Kit	right anterior commissure: pars anterior	0.467	0.014
F	Kit	right anterior commissure: pars posterior	0.193	0.005
F	Kit	right anterior lobule (lobules 4-5)	0.757	0.038
F	Kit	right anterior lobule white matter	0.050	0.003
F	Kit	right Anterior olfactory nucleus	1.033	0.035
F	Kit	right basal forebrain	2.685	0.052
F	Kit	right bed nucleus of stria terminalis	0.625	0.022
F	Kit	right CA10r	0.982	0.101
F	Kit	right CA1Py	0.547	0.082
F	Kit	right CA1Rad	1.376	0.348
F	Kit	right CA20r	0.215	0.029
F	Kit	right CA2Py	0.104	0.024
F	Kit	right CA2Rad	0.229	0.051
F	Kit	right CA30r	1.496	0.152
F	Kit	right CA3Py Inner	0.086	0.022
F	Kit	right CA3Py Outer	0.534	0.062
F	Kit	right CA3Rad	0.983	0.186
F	Kit	right Caudomedial entorhinal cortex	2.883	0.141
F	Kit	right cerebellar peduncle: inferior	0.398	0.016
F	Kit	right cerebellar peduncle: middle	0.590	0.042
F	Kit	right cerebellar peduncle: superior	0.489	0.024
F	Kit	right cerebral peduncle	1.032	0.034
F	Kit	right Cingulate cortex: area 24a	1.055	0.074
F	Kit	right Cingulate cortex: area 24a'	0.341	0.033
F	Kit	right Cingulate cortex: area 24b	0.751	0.059
F	Kit	right Cingulate cortex: area 24b'	0.294	0.024
F	Kit	right Cingulate cortex: area 25	0.316	0.029
F	Kit	right Cingulate cortex: area 29a	0.344	0.029
F	Kit	right Cingulate cortex: area 29b	0.206	0.010
F	Kit	right Cingulate cortex: area 29c	0.869	0.041
F	Kit	right Cingulate cortex: area 30	1.365	0.057
F	Kit	right Cingulate cortex: area 32	1.110	0.085
F	Kit	right Cingulum	0.432	0.021
F	Kit	right Claustrum	0.144	0.009
F	Kit	right Claustrum: dorsal part	0.140	0.019
F	Kit	right Claustrum: ventral part	0.310	0.039
F	Kit	right colliculus: inferior	2.715	0.081
F	Kit	right colliculus: superior	4.324	0.157
F	Kit	right copula white matter	0.036	0.003
F	Kit	right copula: pyramis (lobule 8)	1.338	0.105
F	Kit	right corpus callosum	5.591	0.210
F	Kit	right Cortex-amygdala transition zones	0.447	0.028
F	Kit	right corticospinal tract/pyramids	0.944	0.058
F	Kit	right crus 1 white matter	0.193	0.013
F	Kit	right crus 1: ansiform lobule (lobule 6)	2.264	0.118
F	Kit	right crus 2 white matter	0.151	0.014
F	Kit	right crus 2: ansiform lobule (lobule 7)	2.069	0.143
F	Kit	right cuneate nucleus	0.125	0.012
F	Kit	right dentate nucleus	0.172	0.008
F	Kit	right Dorsal intermediate entorhinal cortex	0.907	0.064
F	Kit	right Dorsal nucleus of the endopiriform	0.748	0.031
F	Kit	right Dorsal tenia tecta	0.460	0.031
F	Kit	right Dorsolateral entorhinal cortex	1.347	0.072
F	Kit	right Dorsolateral orbital cortex	0.408	0.032
F	Kit	right Ectorhinal cortex	1.401	0.083
F	Kit	right facial nerve (cranial nerve 7)	0.107	0.006
F	Kit	right fasciculus retroflexus	0.105	0.004
F	Kit	right fastigial nucleus	0.224	0.008
F	Kit	right fimbria	1.564	0.119
F	Kit	right flocculus (FL)	0.523	0.032
F	Kit	right flocculus white matter	0.042	0.004
F	Kit	right fornix	0.285	0.012
F	Kit	right Frontal association cortex	3.916	0.197

F	Kit	right Frontal cortex: area 3	0.361	0.018
F	Kit	right fundus of striatum	0.079	0.006
F	Kit	right globus pallidus	1.574	0.037
F	Kit	right GrDG	0.643	0.128
F	Kit	right habenular commissure	0.017	0.003
F	Kit	right hypothalamus	5.221	0.140
F	Kit	right inferior olivary complex	0.192	0.028
F	Kit	right Insular region: not subdivided	4.212	0.219
F	Kit	right Intermediate nucleus of the endopiriform claustrum	0.285	0.013
F	Kit	right internal capsule	1.446	0.046
F	Kit	right lateral olfactory tract	0.657	0.026
F	Kit	right Lateral orbital cortex	1.768	0.101
F	Kit	right Lateral parietal association cortex	0.083	0.009
F	Kit	right lateral septum	1.521	0.095
F	Kit	right lateral ventricle	2.541	0.715
F	Kit	right LMol	1.194	0.264
F	Kit	right mammillary bodies	0.230	0.023
F	Kit	right mammilothalamic tract	0.120	0.007
F	Kit	right Medial amygdala	0.546	0.067
F	Kit	right Medial entorhinal cortex	0.338	0.022
F	Kit	right medial lemniscus/medial longitudinal fasciculus	1.319	0.057
F	Kit	right Medial orbital cortex	0.844	0.041
F	Kit	right Medial parietal association cortex	0.164	0.015
F	Kit	right Medial preoptic nucleus	0.090	0.005
F	Kit	right medial septum	0.610	0.021
F	Kit	right MoDG	2.064	0.347
F	Kit	right nucleus accumbens	2.089	0.057
F	Kit	right nucleus interpositus	0.231	0.009
F	Kit	right Olfactory bulb: external plexiform layer	3.001	0.102
F	Kit	right Olfactory bulb: glomerular layer	2.058	0.098
F	Kit	right Olfactory bulb: granule cell layer	2.270	0.110
F	Kit	right Olfactory bulb: internal plexiform layer	0.488	0.026
F	Kit	right Olfactory bulb: mitral cell layer	0.582	0.023
F	Kit	right olfactory peduncle	1.110	0.033
F	Kit	right olfactory tubercle	1.690	0.077
F	Kit	right optic tract	0.716	0.036
F	Kit	right paraflocculus (PFL)	1.591	0.182
F	Kit	right paraflocculus white matter	0.155	0.016
F	Kit	right paramedian lobule	0.084	0.008
F	Kit	right paramedian lobule (lobule 7)	2.089	0.133
F	Kit	right Parietal cortex: posterior area: rostral part	0.041	0.004
F	Kit	right Perirhinal cortex	1.257	0.042
F	Kit	right Piriform cortex	5.435	0.208
F	Kit	right PoDG	0.316	0.072
F	Kit	right pontine nucleus	0.395	0.047
F	Kit	right Posterolateral cortical amygdaloid area	0.448	0.030
F	Kit	right Posteromedial cortical amygdaloid area	0.461	0.032
F	Kit	right pre-para subiculum	1.151	0.050
F	Kit	right Primary auditory cortex	0.758	0.033
F	Kit	right Primary motor cortex	3.244	0.129
F	Kit	right Primary somatosensory cortex	2.027	0.115
F	Kit	right Primary somatosensory cortex: barrel field	4.759	0.169
F	Kit	right Primary somatosensory cortex: dysgranular zone	0.180	0.009
F	Kit	right Primary somatosensory cortex: forelimb region	1.913	0.114
F	Kit	right Primary somatosensory cortex: hindlimb region	1.263	0.092
F	Kit	right Primary somatosensory cortex: jaw region	0.285	0.015
F	Kit	right Primary somatosensory cortex: shoulder region	0.056	0.009
F	Kit	right Primary somatosensory cortex: trunk region	0.184	0.016
F	Kit	right Primary somatosensory cortex: upper lip region	2.733	0.107
F	Kit	right Primary visual cortex	1.230	0.057
F	Kit	right Primary visual cortex: binocular area	1.020	0.065
F	Kit	right Primary visual cortex: monocular area	0.772	0.058
F	Kit	right Rostral amygdalopiriform area	0.204	0.014
F	Kit	right Secondary auditory cortex: dorsal area	0.720	0.038
F	Kit	right Secondary auditory cortex: ventral area	0.991	0.088
F	Kit	right Secondary motor cortex	3.284	0.184
F	Kit	right Secondary somatosensory cortex	3.628	0.181
F	Kit	right Secondary visual cortex: lateral area	1.443	0.068
F	Kit	right Secondary visual cortex: mediolateral area	0.379	0.036
F	Kit	right Secondary visual cortex: mediomedial area	0.779	0.034
F	Kit	right simple lobule (lobule 6)	2.467	0.136
F	Kit	right simple lobule white matter	0.184	0.009
F	Kit	right SLu	0.352	0.024
F	Kit	right stria medullaris	0.335	0.012
F	Kit	right stria terminalis	0.381	0.019
F	Kit	right striatum	10.467	0.391
F	Kit	right subependymale zone / rhinocele	0.026	0.002
F	Kit	right subiculum	1.602	0.120
F	Kit	right superior olivary complex	0.407	0.034
F	Kit	right Temporal association area	1.521	0.107
F	Kit	right thalamus	8.328	0.266
F	Kit	right trunk of crus 2 and paramedian white matter	0.171	0.011
F	Kit	right trunk of simple and crus 1 white matter	0.081	0.004
F	Kit	right Ventral intermediate entorhinal cortex	0.537	0.041
F	Kit	right Ventral nucleus of the endopiriform claustrum	0.223	0.020
F	Kit	right Ventral orbital cortex	0.662	0.023
F	Kit	right Ventral tenia tecta	0.055	0.004
F	Kit	third ventricle	1.371	0.134
F	Kit	trunk of arbor vita	4.006	0.136
F	Kit	trunk of lobules 1-3 white matter	0.131	0.009
F	Kit	trunk of lobules 6-8 white matter	0.106	0.007
F	Kit	ventral tegmental decussation	0.119	0.008
F	Nos2	cerebral aqueduct	0.513	0.066
F	Nos2	fourth ventricle	0.973	0.057
F	Nos2	interpeduncلar nucleus	0.281	0.019
F	Nos2	left Accessory olfactory bulb: glomerular, external plexiform and mitral cell layer	0.260	0.016
F	Nos2	left Accessory olfactory bulb: granule cell layer	0.126	0.007
F	Nos2	left amygdala	4.899	0.218
F	Nos2	left Amygdalopiriform transition area	0.552	0.026
F	Nos2	left anterior commissure: pars anterior	0.505	0.024
F	Nos2	left anterior commissure: pars posterior	0.214	0.013
F	Nos2	left anterior lobule (lobules 4-5)	0.859	0.049
F	Nos2	left anterior lobule white matter	0.042	0.003
F	Nos2	left Anterior olfactory nucleus	0.991	0.063
F	Nos2	left basal forebrain	2.636	0.108
F	Nos2	left bed nucleus of stria terminalis	0.652	0.034
F	Nos2	left CA10r	1.151	0.066
F	Nos2	left CA1Py	0.548	0.025
F	Nos2	left CA1Rad	1.246	0.035
F	Nos2	left CA20r	0.257	0.017
F	Nos2	left CA2Py	0.103	0.005
F	Nos2	left CA2Rad	0.214	0.018
F	Nos2	left CA30r	1.453	0.077
F	Nos2	left CA3Py Inner	0.079	0.006
F	Nos2	left CA3Py Outer	0.517	0.022
F	Nos2	left CA3Rad	0.911	0.036
F	Nos2	left Caudomedial entorhinal cortex	2.926	0.152
F	Nos2	left cerebellar peduncle: inferior	0.439	0.017
F	Nos2	left cerebellar peduncle: middle	0.618	0.041
F	Nos2	left cerebellar peduncle: superior	0.503	0.031
F	Nos2	left cerebral peduncle	1.060	0.050
F	Nos2	left Cingulate cortex: area 24a	0.962	0.066
F	Nos2	left Cingulate cortex: area 24a'	0.379	0.042
F	Nos2	left Cingulate cortex: area 24b	0.790	0.068
F	Nos2	left Cingulate cortex: area 24b'	0.300	0.027
F	Nos2	left Cingulate cortex: area 25	0.302	0.022
F	Nos2	left Cingulate cortex: area 29a	0.361	0.027
F	Nos2	left Cingulate cortex: area 29b	0.221	0.017
F	Nos2	left Cingulate cortex: area 29c	0.946	0.055
F	Nos2	left Cingulate cortex: area 30	1.486	0.096
F	Nos2	left Cingulate cortex: area 32	1.388	0.102
F	Nos2	left Cingulum	0.430	0.022
F	Nos2	left Claustrum	0.172	0.015
F	Nos2	left Claustrum: dorsal part	0.146	0.017
F	Nos2	left Claustrum: ventral part	0.273	0.023
F	Nos2	left colliculus: inferior	2.854	0.112
F	Nos2	left colliculus: superior	4.129	0.276
F	Nos2	left copula white matter	0.046	0.003
F	Nos2	left copula: pyramis (lobule 8)	1.185	0.097
F	Nos2	left corpus callosum	6.095	0.224
F	Nos2	left Cortex-amygdala transition zones	0.406	0.034
F	Nos2	left corticospinal tract/pyramids	0.914	0.055
F	Nos2	left crus 1 white matter	0.197	0.013
F	Nos2	left crus 1: ansiform lobule (lobule 6)	2.235	0.092
F	Nos2	left crus 2 white matter	0.136	0.012
F	Nos2	left crus 2: ansiform lobule (lobule 7)	2.064	0.116
F	Nos2	left cuneate nucleus	0.126	0.010
F	Nos2	left dentate nucleus	0.178	0.009
F	Nos2	left Dorsal intermediate entorhinal cortex	0.907	0.037
F	Nos2	left Dorsal nucleus of the endopiriform	0.747	0.035
F	Nos2	left Dorsal tenia tecta	0.512	0.036
F	Nos2	left Dorsolateral entorhinal cortex	1.214	0.093
F	Nos2	left Dorsolateral orbital cortex	0.440	0.028
F	Nos2	left Ectorhinal cortex	1.347	0.115
F	Nos2	left facial nerve (cranial nerve 7)	0.108	0.005
F	Nos2	left fasciculus retroflexus	0.128	0.007
F	Nos2	left fastigial nucleus	0.244	0.011
F	Nos2	left fimbria	1.687	0.102
F	Nos2	left flocculus (FL)	0.541	0.031
F	Nos2	left flocculus white matter	0.030	0.003
F	Nos2	left fornix	0.339	0.013
F	Nos2	left Frontal association cortex	3.532	0.145
F	Nos2	left Frontal cortex: area 3	0.378	0.019
F	Nos2	left fundus of striatum	0.075	0.005
F	Nos2	left globus pallidus	1.645	0.068
F	Nos2	left GrDG	0.693	0.032
F	Nos2	left habenular commissure	0.017	0.003
F	Nos2	left hypothalamus	5.286	0.240
F	Nos2	left inferior olivary complex	0.167	0.022
F	Nos2	left Insular region: not subdivided	3.832	0.269
F	Nos2	left Intermediate nucleus of the endopiriform claustrum	0.321	0.012
F	Nos2	left internal capsule	1.445	0.052
F	Nos2	left lateral olfactory tract	0.755	0.032
F	Nos2	left Lateral orbital cortex	1.793	0.090
F	Nos2	left Lateral parietal association cortex	0.153	0.013
F	Nos2	left lateral septum	1.587	0.113
F	Nos2	left lateral ventricle	1.985	0.456
F	Nos2	left LMol	1.200	0.034
F	Nos2	left mammillary bodies	0.265	0.026
F	Nos2	left mammilothalamic tract	0.123	0.010
F	Nos2	left Medial amygdala	0.496	0.030
F	Nos2	left Medial entorhinal cortex	0.348	0.029
F	Nos2	left medial lemniscus/medial longitudinal fasciculus	1.265	0.055
F	Nos2	left Medial orbital cortex	0.925	0.056
F	Nos2	left Medial parietal association cortex	0.235	0.018
F	Nos2	left Medial preoptic nucleus	0.095	0.007
F	Nos2	left medial septum	0.660	0.034
F	Nos2	left MoDG	2.215	0.065
F	Nos2	left nucleus accumbens	2.162	0.085
F	Nos2	left nucleus interpositus	0.221	0.012
F	Nos2	left Olfactory bulb: external plexiform layer	3.292	0.115
F	Nos2	left Olfactory bulb: glomerular layer	2.218	0.103
F	Nos2	left Olfactory bulb: granule cell layer	2.294	0.134
F	Nos2	left Olfactory bulb: internal plexiform layer	0.516	0.027
F	Nos2	left Olfactory bulb: mitral cell layer	0.652	0.028
F	Nos2	left olfactory peduncle	1.199	0.058
F	Nos2	left olfactory tubercle	1.698	0.084
F	Nos2	left optic tract	0.749	0.084
F	Nos2	left paraflocculus (PFL)	1.758	0.204
F	Nos2	left paraflocculus white matter	0.146	0.013
F	Nos2	left paramedian lobule	0.077	0.005
F	Nos2	left paramedian lobule (lobule 7)	2.119	0.112
F	Nos2	left Parietal cortex: posterior area: rostral part	0.073	0.010
F	Nos2	left Perirhinal cortex	1.304	0.085
F	Nos2	left Piriform cortex	5.233	0.247
F	Nos2	left PoDG	0.313	0.014
F	Nos2	left pontine nucleus	0.460	0.067
F	Nos2	left Posterolateral cortical amygdaloid area	0.407	0.028
F	Nos2	left Posteromedial cortical amygdaloid area	0.586	0.059
F	Nos2	left pre-para subiculum	1.148	0.043
F	Nos2	left Primary auditory cortex	0.739	0.055
F	Nos2	left Primary motor cortex	3.774	0.148
F	Nos2	left Primary somatosensory cortex	2.077	0.077
F	Nos2	left Primary somatosensory cortex: barrel field	4.804	0.268
F	Nos2	left Primary somatosensory cortex: dysgranular zone	0.174	0.013
F	Nos2	left Primary somatosensory cortex: forelimb region	2.115	0.149
F	Nos2	left Primary somatosensory cortex: hindlimb region	1.521	0.088
F	Nos2	left Primary somatosensory cortex: jaw region	0.283	0.020
F	Nos2	left Primary somatosensory cortex: shoulder region	0.092	0.008
F	Nos2	left Primary somatosensory cortex: trunk region	0.311	0.024
F	Nos2	left Primary somatosensory cortex: upper lip region	2.381	0.096
F	Nos2	left Primary visual cortex	1.201	0.106
F	Nos2	left Primary visual cortex: binocular area	0.989	0.074
F	Nos2	left Primary visual cortex: monocular area	1.140	0.079
F	Nos2	left Rostral amygdalopiriform area	0.199	0.018
F	Nos2	left Secondary auditory cortex: dorsal area	0.732	0.039
F	Nos2	left Secondary auditory cortex: ventral area	0.797	0.086
F	Nos2	left Secondary motor cortex	3.352	0.142
F	Nos2	left Secondary somatosensory cortex	3.439	0.249
F	Nos2	left Secondary visual cortex: lateral area	1.313	0.076
F	Nos2	left Secondary visual cortex: mediolateral area	0.568	0.041
F	Nos2	left Secondary visual cortex: mediomedial area	0.953	0.054
F	Nos2	left simple lobule (lobule 6)	2.434	0.112
F	Nos2	left simple lobule white matter	0.221	0.016
F	Nos2	left SLu	0.367	0.029
F	Nos2	left stria medullaris	0.324	0.013
F	Nos2	left stria terminalis	0.464	0.017
F	Nos2	left striatum	10.914	0.360
F	Nos2	left subependymale zone / rhinocele	0.032	0.006
F	Nos2	left subiculum	1.626	0.081
F	Nos2	left superior olivary complex	0.403	0.039
F	Nos2	left Temporal association area	1.403	0.130
F	Nos2	left thalamus	8.808	0.449
F	Nos2	left trunk of crus 2 and paramedian white matter	0.162	0.011
F	Nos2	left trunk of simple and crus 1 white matter	0.068	0.006
F	Nos2	left Ventral intermediate entorhinal cortex	0.568	0.026
F	Nos2	left Ventral nucleus of the endopiriform claustrum	0.258	0.022
F	Nos2	left Ventral orbital cortex	0.763	0.041
F	Nos2	left Ventral tenia tecta	0.057	0.004
F	Nos2	lobule 1-2 white matter	0.071	0.015
F	Nos2	lobule 10 white matter	0.082	0.004
F	Nos2	lobule 10: nodulus	1.399	0.057
F	Nos2	lobule 3 white matter	0.188	0.015
F	Nos2	lobule 3: central lobule (dorsal)	2.095	0.149
F	Nos2	lobule 6: declive	2.600	0.239
F	Nos2	lobule 7: tuber (or folium)	1.000	0.087
F	Nos2	lobule 8 white matter	0.158	0.007
F	Nos2	lobule 8: pyramis	1.616	0.091
F	Nos2	lobule 9 white matter	0.290	0.020
F	Nos2	lobule 9: uvula	2.789	0.202
F	Nos2	lobules 1-2: lingula and central lobule (ventral)	1.675	0.205
F	Nos2	lobules 4-5 white matter	0.556	0.035
F	Nos2	lobules 4-5: culmen (ventral and dorsal)	4.302	0.194
F	Nos2	lobules 6-7 white matter	0.736	0.091
F	Nos2	medulla	27.761	1.009
F	Nos2	midbrain	13.481	0.704
F	Nos2	periaqueductal grey	3.810	0.188

F	Nos2	pons	17.230	0.783
F	Nos2	posterior commissure	0.140	0.009
F	Nos2	right Accessory olfactory bulb: glomerular, external plexiform and mitral cell layer	0.251	0.011
F	Nos2	right Accessory olfactory bulb: granule cell layer	0.119	0.008
F	Nos2	right amygdala	4.574	0.146
F	Nos2	right Amygdalopiriform transition area	0.508	0.027
F	Nos2	right anterior commissure: pars anterior	0.500	0.021
F	Nos2	right anterior commissure: pars posterior	0.204	0.014
F	Nos2	right anterior lobule (lobules 4-5)	0.777	0.032
F	Nos2	right anterior lobule white matter	0.051	0.003
F	Nos2	right Anterior olfactory nucleus	1.033	0.042
F	Nos2	right basal forebrain	2.743	0.132
F	Nos2	right bed nucleus of stria terminalis	0.649	0.025
F	Nos2	right CA10r	1.001	0.053
F	Nos2	right CA1Py	0.554	0.025
F	Nos2	right CA1Rad	1.353	0.041
F	Nos2	right CA20r	0.215	0.008
F	Nos2	right CA2Py	0.100	0.006
F	Nos2	right CA2Rad	0.230	0.026
F	Nos2	right CA30r	1.462	0.087
F	Nos2	right CA3Py Inner	0.082	0.005
F	Nos2	right CA3Py Outer	0.529	0.026
F	Nos2	right CA3Rad	0.964	0.064
F	Nos2	right Caudomedial entorhinal cortex	2.929	0.153
F	Nos2	right cerebellar peduncle: inferior	0.401	0.017
F	Nos2	right cerebellar peduncle: middle	0.659	0.045
F	Nos2	right cerebellar peduncle: superior	0.486	0.035
F	Nos2	right cerebral peduncle	1.064	0.052
F	Nos2	right Cingulate cortex: area 24a	1.025	0.068
F	Nos2	right Cingulate cortex: area 24a'	0.365	0.038
F	Nos2	right Cingulate cortex: area 24b	0.769	0.056
F	Nos2	right Cingulate cortex: area 24b'	0.291	0.026
F	Nos2	right Cingulate cortex: area 25	0.305	0.020
F	Nos2	right Cingulate cortex: area 29a	0.355	0.028
F	Nos2	right Cingulate cortex: area 29b	0.204	0.017
F	Nos2	right Cingulate cortex: area 29c	0.902	0.058
F	Nos2	right Cingulate cortex: area 30	1.379	0.103
F	Nos2	right Cingulate cortex: area 32	1.127	0.085
F	Nos2	right Cingulum	0.441	0.023
F	Nos2	right Claustrum	0.150	0.010
F	Nos2	right Claustrum: dorsal part	0.136	0.019
F	Nos2	right Claustrum: ventral part	0.305	0.032
F	Nos2	right colliculus: inferior	2.685	0.094
F	Nos2	right colliculus: superior	4.228	0.291
F	Nos2	right copula white matter	0.035	0.002
F	Nos2	right copula: pyramis (lobule 8)	1.300	0.088
F	Nos2	right corpus callosum	5.861	0.227
F	Nos2	right Cortex-amygdala transition zones	0.429	0.032
F	Nos2	right corticospinal tract/pyramids	0.951	0.052
F	Nos2	right crus 1 white matter	0.190	0.008
F	Nos2	right crus 1: ansiform lobule (lobule 6)	2.240	0.109
F	Nos2	right crus 2 white matter	0.158	0.008
F	Nos2	right crus 2: ansiform lobule (lobule 7)	2.129	0.118
F	Nos2	right cuneate nucleus	0.132	0.012
F	Nos2	right dentate nucleus	0.176	0.011
F	Nos2	right Dorsal intermediate entorhinal cortex	0.939	0.041
F	Nos2	right Dorsal nucleus of the endopiriform	0.722	0.039
F	Nos2	right Dorsal tenia tecta	0.478	0.038
F	Nos2	right Dorsolateral entorhinal cortex	1.348	0.073
F	Nos2	right Dorsolateral orbital cortex	0.418	0.026
F	Nos2	right Ectorhinal cortex	1.377	0.078
F	Nos2	right facial nerve (cranial nerve 7)	0.112	0.007
F	Nos2	right fasciculus retroflexus	0.112	0.007
F	Nos2	right fastigial nucleus	0.231	0.012
F	Nos2	right fimbria	1.551	0.068
F	Nos2	right flocculus (FL)	0.524	0.040
F	Nos2	right flocculus white matter	0.040	0.004
F	Nos2	right fornix	0.298	0.014
F	Nos2	right Frontal association cortex	3.988	0.145
F	Nos2	right Frontal cortex: area 3	0.374	0.028
F	Nos2	right fundus of striatum	0.080	0.007
F	Nos2	right globus pallidus	1.685	0.054
F	Nos2	right GrDG	0.663	0.037
F	Nos2	right habenular commissure	0.017	0.003
F	Nos2	right hypothalamus	5.254	0.200
F	Nos2	right inferior olivary complex	0.179	0.016
F	Nos2	right Insular region: not subdivided	4.028	0.215
F	Nos2	right Intermediate nucleus of the endopiriform claustrum	0.286	0.022
F	Nos2	right internal capsule	1.531	0.065
F	Nos2	right lateral olfactory tract	0.663	0.019
F	Nos2	right Lateral orbital cortex	1.782	0.053
F	Nos2	right Lateral parietal association cortex	0.089	0.010
F	Nos2	right lateral septum	1.594	0.145
F	Nos2	right lateral ventricle	2.107	0.448
F	Nos2	right LMol	1.200	0.052
F	Nos2	right mammillary bodies	0.249	0.026
F	Nos2	right mammilothalamic tract	0.128	0.007
F	Nos2	right Medial amygdala	0.530	0.049
F	Nos2	right Medial entorhinal cortex	0.344	0.019
F	Nos2	right medial lemniscus/medial longitudinal fasciculus	1.346	0.052
F	Nos2	right Medial orbital cortex	0.869	0.068
F	Nos2	right Medial parietal association cortex	0.166	0.015
F	Nos2	right Medial preoptic nucleus	0.090	0.007
F	Nos2	right medial septum	0.621	0.031
F	Nos2	right MoDG	2.091	0.084
F	Nos2	right nucleus accumbens	2.146	0.086
F	Nos2	right nucleus interpositus	0.231	0.010
F	Nos2	right Olfactory bulb: external plexiform layer	3.295	0.125
F	Nos2	right Olfactory bulb: glomerular layer	2.278	0.092
F	Nos2	right Olfactory bulb: granule cell layer	2.356	0.097
F	Nos2	right Olfactory bulb: internal plexiform layer	0.514	0.034
F	Nos2	right Olfactory bulb: mitral cell layer	0.621	0.033
F	Nos2	right olfactory peduncle	1.164	0.027
F	Nos2	right olfactory tubercle	1.729	0.132
F	Nos2	right optic tract	0.710	0.069
F	Nos2	right paraflocculus (PFL)	1.723	0.155
F	Nos2	right paraflocculus white matter	0.169	0.018
F	Nos2	right paramedian lobule	0.085	0.008
F	Nos2	right paramedian lobule (lobule 7)	2.084	0.123
F	Nos2	right Parietal cortex: posterior area: rostral part	0.041	0.005
F	Nos2	right Perirhinal cortex	1.262	0.057
F	Nos2	right Piriform cortex	5.598	0.228
F	Nos2	right PoDG	0.332	0.021
F	Nos2	right pontine nucleus	0.440	0.053
F	Nos2	right Posterolateral cortical amygdaloid area	0.451	0.033
F	Nos2	right Posteromedial cortical amygdaloid area	0.473	0.042
F	Nos2	right pre-para subiculum	1.177	0.047
F	Nos2	right Primary auditory cortex	0.757	0.066
F	Nos2	right Primary motor cortex	3.287	0.136
F	Nos2	right Primary somatosensory cortex	2.086	0.118
F	Nos2	right Primary somatosensory cortex: barrel field	4.668	0.160
F	Nos2	right Primary somatosensory cortex: dysgranular zone	0.188	0.014
F	Nos2	right Primary somatosensory cortex: forelimb region	1.884	0.119
F	Nos2	right Primary somatosensory cortex: hindlimb region	1.263	0.086
F	Nos2	right Primary somatosensory cortex: jaw region	0.301	0.026
F	Nos2	right Primary somatosensory cortex: shoulder region	0.064	0.011
F	Nos2	right Primary somatosensory cortex: trunk region	0.192	0.023
F	Nos2	right Primary somatosensory cortex: upper lip region	2.817	0.111
F	Nos2	right Primary visual cortex	1.242	0.101
F	Nos2	right Primary visual cortex: binocular area	1.019	0.075
F	Nos2	right Primary visual cortex: monocular area	0.753	0.061
F	Nos2	right Rostral amygdalopiriform area	0.214	0.016
F	Nos2	right Secondary auditory cortex: dorsal area	0.720	0.068
F	Nos2	right Secondary auditory cortex: ventral area	1.013	0.069
F	Nos2	right Secondary motor cortex	3.281	0.118
F	Nos2	right Secondary somatosensory cortex	3.577	0.319
F	Nos2	right Secondary visual cortex: lateral area	1.478	0.106
F	Nos2	right Secondary visual cortex: mediolateral area	0.380	0.043
F	Nos2	right Secondary visual cortex: mediomedial area	0.779	0.060
F	Nos2	right simple lobule (lobule 6)	2.473	0.109
F	Nos2	right simple lobule white matter	0.180	0.007
F	Nos2	right SLu	0.367	0.025
F	Nos2	right stria medullaris	0.347	0.013
F	Nos2	right stria terminalis	0.393	0.011
F	Nos2	right striatum	10.816	0.407
F	Nos2	right subependymale zone / rhinocele	0.027	0.004
F	Nos2	right subiculum	1.615	0.072
F	Nos2	right superior olivary complex	0.415	0.034
F	Nos2	right Temporal association area	1.564	0.094
F	Nos2	right thalamus	8.726	0.429
F	Nos2	right trunk of crus 2 and paramedian white matter	0.175	0.008
F	Nos2	right trunk of simple and crus 1 white matter	0.082	0.004
F	Nos2	right Ventral intermediate entorhinal cortex	0.562	0.029
F	Nos2	right Ventral nucleus of the endopiriform claustrum	0.242	0.028
F	Nos2	right Ventral orbital cortex	0.717	0.048
F	Nos2	right Ventral tenia tecta	0.059	0.004
F	Nos2	third ventricle	1.346	0.121
F	Nos2	trunk of arbor vita	4.164	0.146
F	Nos2	trunk of lobules 1-3 white matter	0.128	0.011
F	Nos2	trunk of lobules 6-8 white matter	0.105	0.005
F	Nos2	ventral tegmental decussation	0.127	0.010
F	Rag1	cerebral aqueduct	0.526	0.047
F	Rag1	fourth ventricle	0.939	0.077
F	Rag1	interpeduncular nucleus	0.276	0.018
F	Rag1	left Accessory olfactory bulb: glomerular, external plexiform and mitral cell layer	0.261	0.014
F	Rag1	left Accessory olfactory bulb: granule cell layer	0.127	0.007
F	Rag1	left amygdala	4.892	0.191
F	Rag1	left Amygdalopiriform transition area	0.525	0.031
F	Rag1	left anterior commissure: pars anterior	0.496	0.016
F	Rag1	left anterior commissure: pars posterior	0.214	0.012
F	Rag1	left anterior lobule (lobules 4-5)	0.892	0.054
F	Rag1	left anterior lobule white matter	0.044	0.004
F	Rag1	left Anterior olfactory nucleus	0.994	0.036
F	Rag1	left basal forebrain	2.640	0.085
F	Rag1	left bed nucleus of stria terminalis	0.657	0.034
F	Rag1	left CA10r	1.141	0.061
F	Rag1	left CA1Py	0.542	0.028
F	Rag1	left CA1Rad	1.214	0.052
F	Rag1	left CA20r	0.262	0.009
F	Rag1	left CA2Py	0.102	0.003
F	Rag1	left CA2Rad	0.205	0.009
F	Rag1	left CA30r	1.440	0.054
F	Rag1	left CA3Py Inner	0.077	0.004
F	Rag1	left CA3Py Outer	0.511	0.024
F	Rag1	left CA3Rad	0.900	0.049
F	Rag1	left Caudomedial entorhinal cortex	2.828	0.198
F	Rag1	left cerebellar peduncle: inferior	0.423	0.014
F	Rag1	left cerebellar peduncle: middle	0.603	0.017
F	Rag1	left cerebellar peduncle: superior	0.520	0.034
F	Rag1	left cerebral peduncle	1.042	0.036
F	Rag1	left Cingulate cortex: area 24a	0.930	0.044
F	Rag1	left Cingulate cortex: area 24a'	0.372	0.033
F	Rag1	left Cingulate cortex: area 24b	0.803	0.057
F	Rag1	left Cingulate cortex: area 24b'	0.311	0.023
F	Rag1	left Cingulate cortex: area 25	0.298	0.014
F	Rag1	left Cingulate cortex: area 29a	0.383	0.027
F	Rag1	left Cingulate cortex: area 29b	0.223	0.020
F	Rag1	left Cingulate cortex: area 29c	0.983	0.041
F	Rag1	left Cingulate cortex: area 30	1.468	0.071
F	Rag1	left Cingulate cortex: area 32	1.389	0.102
F	Rag1	left Cingulum	0.430	0.022
F	Rag1	left Claustrum	0.176	0.013
F	Rag1	left Claustrum: dorsal part	0.146	0.016
F	Rag1	left Claustrum: ventral part	0.269	0.025
F	Rag1	left colliculus: inferior	2.760	0.090
F	Rag1	left colliculus: superior	4.293	0.262
F	Rag1	left copula white matter	0.045	0.003
F	Rag1	left copula: pyramis (lobule 8)	1.216	0.109
F	Rag1	left corpus callosum	6.007	0.262
F	Rag1	left Cortex-amygdala transition zones	0.394	0.025
F	Rag1	left corticospinal tract/pyramids	0.887	0.059
F	Rag1	left crus 1 white matter	0.189	0.009
F	Rag1	left crus 1: ansiform lobule (lobule 6)	2.136	0.072
F	Rag1	left crus 2 white matter	0.129	0.010
F	Rag1	left crus 2: ansiform lobule (lobule 7)	1.947	0.115
F	Rag1	left cuneate nucleus	0.124	0.009
F	Rag1	left dentate nucleus	0.180	0.011
F	Rag1	left Dorsal intermediate entorhinal cortex	0.854	0.025
F	Rag1	left Dorsal nucleus of the endopiriform	0.733	0.039
F	Rag1	left Dorsal tenia tecta	0.505	0.017
F	Rag1	left Dorsolateral entorhinal cortex	1.191	0.085
F	Rag1	left Dorsolateral orbital cortex	0.433	0.026
F	Rag1	left Ectorhinal cortex	1.274	0.121
F	Rag1	left facial nerve (cranial nerve 7)	0.105	0.005
F	Rag1	left fasciculus retroflexus	0.130	0.009
F	Rag1	left fastigial nucleus	0.242	0.012
F	Rag1	left fimbria	1.646	0.072
F	Rag1	left flocculus (FL)	0.538	0.027
F	Rag1	left flocculus white matter	0.029	0.003
F	Rag1	left fornix	0.334	0.016
F	Rag1	left Frontal association cortex	3.702	0.166
F	Rag1	left Frontal cortex: area 3	0.350	0.012
F	Rag1	left fundus of striatum	0.075	0.005
F	Rag1	left globus pallidus	1.644	0.065
F	Rag1	left GrDG	0.665	0.033
F	Rag1	left habenular commissure	0.017	0.003
F	Rag1	left hypothalamus	5.329	0.173
F	Rag1	left inferior olivary complex	0.162	0.019
F	Rag1	left Insular region: not subdivided	3.733	0.299
F	Rag1	left Intermediate nucleus of the endopiriform claustrum	0.308	0.018
F	Rag1	left internal capsule	1.428	0.062
F	Rag1	left lateral olfactory tract	0.734	0.029
F	Rag1	left Lateral orbital cortex	1.790	0.069
F	Rag1	left Lateral parietal association cortex	0.150	0.013
F	Rag1	left lateral septum	1.529	0.063
F	Rag1	left lateral ventricle	1.866	0.176
F	Rag1	left LMol	1.165	0.048
F	Rag1	left mammillary bodies	0.258	0.016
F	Rag1	left mammilothalamic tract	0.126	0.008
F	Rag1	left Medial amygdala	0.506	0.021
F	Rag1	left Medial entorhinal cortex	0.339	0.024
F	Rag1	left medial lemniscus/medial longitudinal fasciculus	1.252	0.047
F	Rag1	left Medial orbital cortex	0.935	0.049
F	Rag1	left Medial parietal association cortex	0.235	0.025
F	Rag1	left Medial preoptic nucleus	0.096	0.005
F	Rag1	left medial septum	0.661	0.018
F	Rag1	left MoDG	2.140	0.098
F	Rag1	left nucleus accumbens	2.091	0.098
F	Rag1	left nucleus interpositus	0.225	0.015
F	Rag1	left Olfactory bulb: external plexiform layer	3.174	0.068
F	Rag1	left Olfactory bulb: glomerular layer	2.121	0.085
F	Rag1	left Olfactory bulb: granule cell layer	2.231	0.099
F	Rag1	left Olfactory bulb: internal plexiform layer	0.496	0.018
F	Rag1	left Olfactory bulb: mitral cell layer	0.631	0.015
F	Rag1	left olfactory peduncle	1.143	0.052
F	Rag1	left olfactory tubercle	1.602	0.070
F	Rag1	left optic tract	0.768	0.033

F	Rag1	left paraflocculus (PFL)	1.598	0.278
F	Rag1	left paraflocculus white matter	0.135	0.017
F	Rag1	left paramedian lobule	0.071	0.006
F	Rag1	left paramedian lobule (lobule 7)	2.003	0.107
F	Rag1	left Parietal cortex: posterior area: rostral part	0.071	0.008
F	Rag1	left Perirhinal cortex	1.237	0.089
F	Rag1	left Piriform cortex	5.025	0.258
F	Rag1	left PoDG	0.297	0.015
F	Rag1	left pontine nucleus	0.428	0.049
F	Rag1	left Posterolateral cortical amygdaloid area	0.395	0.027
F	Rag1	left Posteromedial cortical amygdaloid area	0.545	0.026
F	Rag1	left pre-para subiculum	1.147	0.061
F	Rag1	left Primary auditory cortex	0.736	0.061
F	Rag1	left Primary motor cortex	3.673	0.152
F	Rag1	left Primary somatosensory cortex	1.974	0.122
F	Rag1	left Primary somatosensory cortex: barrel field	4.773	0.223
F	Rag1	left Primary somatosensory cortex: dysgranular zone	0.166	0.019
F	Rag1	left Primary somatosensory cortex: forelimb region	2.000	0.112
F	Rag1	left Primary somatosensory cortex: hindlimb region	1.447	0.076
F	Rag1	left Primary somatosensory cortex: jaw region	0.255	0.030
F	Rag1	left Primary somatosensory cortex: shoulder region	0.088	0.012
F	Rag1	left Primary somatosensory cortex: trunk region	0.302	0.023
F	Rag1	left Primary somatosensory cortex: upper lip region	2.281	0.126
F	Rag1	left Primary visual cortex	1.202	0.091
F	Rag1	left Primary visual cortex: binocular area	1.039	0.076
F	Rag1	left Primary visual cortex: monocular area	1.204	0.100
F	Rag1	left Rostral amygdalopiriform area	0.192	0.016
F	Rag1	left Secondary auditory cortex: dorsal area	0.762	0.042
F	Rag1	left Secondary auditory cortex: ventral area	0.745	0.070
F	Rag1	left Secondary motor cortex	3.344	0.109
F	Rag1	left Secondary somatosensory cortex	3.412	0.354
F	Rag1	left Secondary visual cortex: lateral area	1.318	0.105
F	Rag1	left Secondary visual cortex: mediolateral area	0.596	0.040
F	Rag1	left Secondary visual cortex: mediomedial area	0.951	0.043
F	Rag1	left simple lobule (lobule 6)	2.350	0.128
F	Rag1	left simple lobule white matter	0.215	0.015
F	Rag1	left SLu	0.369	0.025
F	Rag1	left stria medullaris	0.329	0.011
F	Rag1	left stria terminalis	0.469	0.016
F	Rag1	left striatum	10.632	0.423
F	Rag1	left subependymale zone / rhinocele	0.028	0.002
F	Rag1	left subiculum	1.605	0.067
F	Rag1	left superior olivary complex	0.385	0.036
F	Rag1	left Temporal association area	1.333	0.104
F	Rag1	left thalamus	8.843	0.489
F	Rag1	left trunk of crus 2 and paramedian white matter	0.176	0.014
F	Rag1	left trunk of simple and crus 1 white matter	0.068	0.004
F	Rag1	left Ventral intermediate entorhinal cortex	0.543	0.021
F	Rag1	left Ventral nucleus of the endopiriform claustrum	0.260	0.019
F	Rag1	left Ventral orbital cortex	0.761	0.039
F	Rag1	left Ventral tenia tecta	0.053	0.004
F	Rag1	lobule 1-2 white matter	0.072	0.017
F	Rag1	lobule 10 white matter	0.079	0.007
F	Rag1	lobule 10: nodulus	1.323	0.062
F	Rag1	lobule 3 white matter	0.177	0.033
F	Rag1	lobule 3: central lobule (dorsal)	1.982	0.253
F	Rag1	lobule 6: declive	2.465	0.206
F	Rag1	lobule 7: tuber (or folium)	0.906	0.083
F	Rag1	lobule 8 white matter	0.158	0.006
F	Rag1	lobule 8: pyramis	1.589	0.077
F	Rag1	lobule 9 white matter	0.283	0.012
F	Rag1	lobule 9: uvula	2.697	0.135
F	Rag1	lobules 1-2: lingula and central lobule (ventral)	1.727	0.213
F	Rag1	lobules 4-5 white matter	0.542	0.036
F	Rag1	lobules 4-5: culmen (ventral and dorsal)	4.134	0.256
F	Rag1	lobules 6-7 white matter	0.679	0.079
F	Rag1	medulla	27.152	0.688
F	Rag1	midbrain	13.759	0.782
F	Rag1	periaqueductal grey	3.927	0.207
F	Rag1	pons	17.242	0.615
F	Rag1	posterior commissure	0.145	0.009
F	Rag1	right Accessory olfactory bulb: glomerular, external plexiform and mitral cell layer	0.251	0.015
F	Rag1	right Accessory olfactory bulb: granule cell layer	0.121	0.005
F	Rag1	right amygdala	4.608	0.104
F	Rag1	right Amygdalopiriform transition area	0.481	0.023
F	Rag1	right anterior commissure: pars anterior	0.482	0.019
F	Rag1	right anterior commissure: pars posterior	0.206	0.010
F	Rag1	right anterior lobule (lobules 4-5)	0.816	0.082
F	Rag1	right anterior lobule white matter	0.055	0.007
F	Rag1	right Anterior olfactory nucleus	1.025	0.055
F	Rag1	right basal forebrain	2.732	0.082
F	Rag1	right bed nucleus of stria terminalis	0.646	0.030
F	Rag1	right CA10r	1.001	0.055
F	Rag1	right CA1Py	0.547	0.025
F	Rag1	right CA1Rad	1.319	0.044
F	Rag1	right CA20r	0.213	0.014
F	Rag1	right CA2Py	0.097	0.005
F	Rag1	right CA2Rad	0.215	0.013
F	Rag1	right CA30r	1.420	0.035
F	Rag1	right CA3Py Inner	0.078	0.003
F	Rag1	right CA3Py Outer	0.508	0.015
F	Rag1	right CA3Rad	0.928	0.051
F	Rag1	right Caudomedial entorhinal cortex	2.743	0.106
F	Rag1	right cerebellar peduncle: inferior	0.388	0.014
F	Rag1	right cerebellar peduncle: middle	0.636	0.020
F	Rag1	right cerebellar peduncle: superior	0.504	0.029
F	Rag1	right cerebral peduncle	1.044	0.033
F	Rag1	right Cingulate cortex: area 24a	1.001	0.070
F	Rag1	right Cingulate cortex: area 24a'	0.355	0.038
F	Rag1	right Cingulate cortex: area 24b	0.794	0.074
F	Rag1	right Cingulate cortex: area 24b'	0.300	0.025
F	Rag1	right Cingulate cortex: area 25	0.306	0.015
F	Rag1	right Cingulate cortex: area 29a	0.379	0.021
F	Rag1	right Cingulate cortex: area 29b	0.208	0.019
F	Rag1	right Cingulate cortex: area 29c	0.926	0.051
F	Rag1	right Cingulate cortex: area 30	1.361	0.065
F	Rag1	right Cingulate cortex: area 32	1.104	0.091
F	Rag1	right Cingulum	0.438	0.021
F	Rag1	right Claustrum	0.152	0.006
F	Rag1	right Claustrum: dorsal part	0.134	0.014
F	Rag1	right Claustrum: ventral part	0.309	0.026
F	Rag1	right colliculus: inferior	2.630	0.121
F	Rag1	right colliculus: superior	4.385	0.225
F	Rag1	right copula white matter	0.035	0.003
F	Rag1	right copula: pyramis (lobule 8)	1.324	0.082
F	Rag1	right corpus callosum	5.771	0.214
F	Rag1	right Cortex-amygdala transition zones	0.444	0.028
F	Rag1	right corticospinal tract/pyramids	0.912	0.063
F	Rag1	right crus 1 white matter	0.178	0.008
F	Rag1	right crus 1: ansiform lobule (lobule 6)	2.124	0.089
F	Rag1	right crus 2 white matter	0.148	0.012
F	Rag1	right crus 2: ansiform lobule (lobule 7)	1.972	0.127
F	Rag1	right cuneate nucleus	0.126	0.009
F	Rag1	right dentate nucleus	0.175	0.010
F	Rag1	right Dorsal intermediate entorhinal cortex	0.902	0.066
F	Rag1	right Dorsal nucleus of the endopiriform	0.728	0.029
F	Rag1	right Dorsal tenia tecta	0.472	0.026
F	Rag1	right Dorsolateral entorhinal cortex	1.316	0.059
F	Rag1	right Dorsolateral orbital cortex	0.408	0.034
F	Rag1	right Ectorhinal cortex	1.309	0.121
F	Rag1	right facial nerve (cranial nerve 7)	0.108	0.006
F	Rag1	right fasciculus retroflexus	0.117	0.006
F	Rag1	right fastigial nucleus	0.230	0.016
F	Rag1	right fimbria	1.510	0.076
F	Rag1	right flocculus (FL)	0.493	0.030
F	Rag1	right flocculus white matter	0.038	0.004
F	Rag1	right fornix	0.298	0.012
F	Rag1	right Frontal association cortex	4.095	0.207
F	Rag1	right Frontal cortex: area 3	0.362	0.025
F	Rag1	right fundus of striatum	0.083	0.005
F	Rag1	right globus pallidus	1.673	0.059
F	Rag1	right GrDG	0.630	0.022
F	Rag1	right habenular commissure	0.016	0.003
F	Rag1	right hypothalamus	5.310	0.148
F	Rag1	right inferior olivary complex	0.173	0.017
F	Rag1	right Insular region: not subdivided	3.977	0.305
F	Rag1	right Intermediate nucleus of the endopiriform claustrum	0.282	0.014
F	Rag1	right internal capsule	1.521	0.053
F	Rag1	right lateral olfactory tract	0.652	0.021
F	Rag1	right Lateral orbital cortex	1.759	0.061
F	Rag1	right Lateral parietal association cortex	0.091	0.012
F	Rag1	right lateral septum	1.518	0.060
F	Rag1	right lateral ventricle	1.928	0.169
F	Rag1	right LMol	1.147	0.038
F	Rag1	right mammillary bodies	0.246	0.017
F	Rag1	right mammilothalamic tract	0.128	0.009
F	Rag1	right Medial amygdala	0.526	0.023
F	Rag1	right Medial entorhinal cortex	0.324	0.018
F	Rag1	right medial lemniscus/medial longitudinal fasciculus	1.332	0.060
F	Rag1	right Medial orbital cortex	0.852	0.060
F	Rag1	right Medial parietal association cortex	0.173	0.015
F	Rag1	right Medial preoptic nucleus	0.090	0.005
F	Rag1	right medial septum	0.630	0.020
F	Rag1	right MoDG	2.013	0.070
F	Rag1	right nucleus accumbens	2.074	0.057
F	Rag1	right nucleus interpositus	0.235	0.016
F	Rag1	right Olfactory bulb: external plexiform layer	3.186	0.119
F	Rag1	right Olfactory bulb: glomerular layer	2.197	0.070
F	Rag1	right Olfactory bulb: granule cell layer	2.288	0.095
F	Rag1	right Olfactory bulb: internal plexiform layer	0.490	0.032
F	Rag1	right Olfactory bulb: mitral cell layer	0.593	0.033
F	Rag1	right olfactory peduncle	1.124	0.042
F	Rag1	right olfactory tubercle	1.659	0.117
F	Rag1	right optic tract	0.718	0.021
F	Rag1	right paraflocculus (PFL)	1.501	0.267
F	Rag1	right paraflocculus white matter	0.148	0.028
F	Rag1	right paramedian lobule	0.081	0.007
F	Rag1	right paramedian lobule (lobule 7)	2.006	0.100
F	Rag1	right Parietal cortex: posterior area: rostral part	0.044	0.007
F	Rag1	right Perirhinal cortex	1.191	0.046
F	Rag1	right Piriform cortex	5.431	0.190
F	Rag1	right PoDG	0.311	0.015
F	Rag1	right pontine nucleus	0.427	0.045
F	Rag1	right Posterolateral cortical amygdaloid area	0.436	0.024
F	Rag1	right Posteromedial cortical amygdaloid area	0.451	0.033
F	Rag1	right pre-para subiculum	1.163	0.050
F	Rag1	right Primary auditory cortex	0.738	0.047
F	Rag1	right Primary motor cortex	3.249	0.165
F	Rag1	right Primary somatosensory cortex	2.029	0.136
F	Rag1	right Primary somatosensory cortex: barrel field	4.628	0.219
F	Rag1	right Primary somatosensory cortex: dysgranular zone	0.176	0.009
F	Rag1	right Primary somatosensory cortex: forelimb region	1.810	0.116
F	Rag1	right Primary somatosensory cortex: hindlimb region	1.210	0.082
F	Rag1	right Primary somatosensory cortex: jaw region	0.277	0.018
F	Rag1	right Primary somatosensory cortex: shoulder region	0.065	0.009
F	Rag1	right Primary somatosensory cortex: trunk region	0.193	0.018
F	Rag1	right Primary somatosensory cortex: upper lip region	2.710	0.106
F	Rag1	right Primary visual cortex	1.216	0.094
F	Rag1	right Primary visual cortex: binocular area	1.057	0.056
F	Rag1	right Primary visual cortex: monocular area	0.804	0.067
F	Rag1	right Rostral amygdalopiriform area	0.202	0.014
F	Rag1	right Secondary auditory cortex: dorsal area	0.728	0.036
F	Rag1	right Secondary auditory cortex: ventral area	0.953	0.125
F	Rag1	right Secondary motor cortex	3.283	0.128
F	Rag1	right Secondary somatosensory cortex	3.543	0.401
F	Rag1	right Secondary visual cortex: lateral area	1.470	0.109
F	Rag1	right Secondary visual cortex: mediolateral area	0.402	0.039
F	Rag1	right Secondary visual cortex: mediomedial area	0.788	0.041
F	Rag1	right simple lobule (lobule 6)	2.375	0.139
F	Rag1	right simple lobule white matter	0.177	0.007
F	Rag1	right SLu	0.364	0.024
F	Rag1	right stria medullaris	0.353	0.013
F	Rag1	right stria terminalis	0.393	0.011
F	Rag1	right striatum	10.641	0.394
F	Rag1	right subependymale zone / rhinocele	0.025	0.002
F	Rag1	right subiculum	1.600	0.055
F	Rag1	right superior olivary complex	0.406	0.039
F	Rag1	right Temporal association area	1.486	0.130
F	Rag1	right thalamus	8.767	0.458
F	Rag1	right trunk of crus 2 and paramedian white matter	0.184	0.012
F	Rag1	right trunk of simple and crus 1 white matter	0.083	0.006
F	Rag1	right Ventral intermediate entorhinal cortex	0.543	0.026
F	Rag1	right Ventral nucleus of the endopiriform claustrum	0.243	0.019
F	Rag1	right Ventral orbital cortex	0.693	0.033
F	Rag1	right Ventral tenia tecta	0.055	0.004
F	Rag1	third ventricle	1.324	0.107
F	Rag1	trunk of arbor vita	4.202	0.149
F	Rag1	trunk of lobules 1-3 white matter	0.127	0.014
F	Rag1	trunk of lobules 6-8 white matter	0.110	0.006
F	Rag1	ventral tegmental decussation	0.131	0.011
F	Rag2	cerebral aqueduct	0.504	0.042
F	Rag2	fourth ventricle	0.926	0.075
F	Rag2	interpeduncular nucleus	0.264	0.014
F	Rag2	left Accessory olfactory bulb: glomerular, external plexiform and mitral cell layer	0.250	0.011
F	Rag2	left Accessory olfactory bulb: granule cell layer	0.118	0.006
F	Rag2	left amygdala	4.623	0.204
F	Rag2	left Amygdalopiriform transition area	0.505	0.036
F	Rag2	left anterior commissure: pars anterior	0.471	0.023
F	Rag2	left anterior commissure: pars posterior	0.203	0.009
F	Rag2	left anterior lobule (lobules 4-5)	0.816	0.066
F	Rag2	left anterior lobule white matter	0.041	0.005
F	Rag2	left Anterior olfactory nucleus	0.961	0.041
F	Rag2	left basal forebrain	2.476	0.092
F	Rag2	left bed nucleus of stria terminalis	0.613	0.038
F	Rag2	left CA10r	1.094	0.054
F	Rag2	left CA1Py	0.519	0.029
F	Rag2	left CA1Rad	1.177	0.057
F	Rag2	left CA20r	0.251	0.012
F	Rag2	left CA2Py	0.098	0.006
F	Rag2	left CA2Rad	0.200	0.013
F	Rag2	left CA30r	1.350	0.052
F	Rag2	left CA3Py Inner	0.074	0.006
F	Rag2	left CA3Py Outer	0.487	0.027
F	Rag2	left CA3Rad	0.867	0.053
F	Rag2	left Caudomedial entorhinal cortex	2.846	0.142
F	Rag2	left cerebellar peduncle: inferior	0.403	0.021
F	Rag2	left cerebellar peduncle: middle	0.551	0.028
F	Rag2	left cerebellar peduncle: superior	0.492	0.030
F	Rag2	left cerebral peduncle	0.979	0.037
F	Rag2	left Cingulate cortex: area 24a	0.937	0.063
F	Rag2	left Cingulate cortex: area 24a'	0.381	0.038
F	Rag2	left Cingulate cortex: area 24b	0.814	0.092
F	Rag2	left Cingulate cortex: area 24b'	0.318	0.020
F	Rag2	left Cingulate cortex: area 25	0.277	0.020
F	Rag2	left Cingulate cortex: area 29a	0.355	0.021
F	Rag2	left Cingulate cortex: area 29b	0.203	0.017
F	Rag2	left Cingulate cortex: area 29c	0.938	0.037

F	Rag2	left Cingulate cortex: area 30	1.407	0.091
F	Rag2	left Cingulate cortex: area 32	1.349	0.112
F	Rag2	left Cingulum	0.405	0.032
F	Rag2	left Claustrum	0.166	0.010
F	Rag2	left Claustrum: dorsal part	0.144	0.015
F	Rag2	left Claustrum: ventral part	0.265	0.032
F	Rag2	left colliculus: inferior	2.779	0.118
F	Rag2	left colliculus: superior	4.158	0.315
F	Rag2	left copula white matter	0.042	0.004
F	Rag2	left copula: pyramis (lobule 8)	1.139	0.093
F	Rag2	left corpus callosum	5.818	0.372
F	Rag2	left Cortex-amygdala transition zones	0.369	0.036
F	Rag2	left corticospinal tract/pyramids	0.833	0.057
F	Rag2	left crus 1 white matter	0.178	0.015
F	Rag2	left crus 1: ansiform lobule (lobule 6)	2.037	0.126
F	Rag2	left crus 2 white matter	0.124	0.012
F	Rag2	left crus 2: ansiform lobule (lobule 7)	1.875	0.130
F	Rag2	left cuneate nucleus	0.118	0.013
F	Rag2	left dentate nucleus	0.172	0.009
F	Rag2	left Dorsal intermediate entorhinal cortex	0.814	0.038
F	Rag2	left Dorsal nucleus of the endopiriform	0.692	0.037
F	Rag2	left Dorsal tenia tecta	0.468	0.022
F	Rag2	left Dorsolateral entorhinal cortex	1.145	0.054
F	Rag2	left Dorsolateral orbital cortex	0.429	0.039
F	Rag2	left Ectorhinal cortex	1.223	0.092
F	Rag2	left facial nerve (cranial nerve 7)	0.102	0.005
F	Rag2	left fasciculus retroflexus	0.125	0.009
F	Rag2	left fastigial nucleus	0.230	0.016
F	Rag2	left fimbria	1.560	0.079
F	Rag2	left flocculus (FL)	0.521	0.039
F	Rag2	left flocculus white matter	0.029	0.004
F	Rag2	left fornix	0.313	0.019
F	Rag2	left Frontal association cortex	3.580	0.183
F	Rag2	left Frontal cortex: area 3	0.341	0.019
F	Rag2	left fundus of striatum	0.069	0.005
F	Rag2	left globus pallidus	1.559	0.086
F	Rag2	left GrDG	0.658	0.033
F	Rag2	left habenular commissure	0.014	0.003
F	Rag2	left hypothalamus	4.972	0.189
F	Rag2	left inferior olivary complex	0.153	0.021
F	Rag2	left Insular region: not subdivided	3.574	0.277
F	Rag2	left Intermediate nucleus of the endopiriform claustrum	0.288	0.014
F	Rag2	left internal capsule	1.357	0.080
F	Rag2	left lateral olfactory tract	0.698	0.028
F	Rag2	left Lateral orbital cortex	1.675	0.084
F	Rag2	left Lateral parietal association cortex	0.147	0.010
F	Rag2	left lateral septum	1.434	0.093
F	Rag2	left lateral ventricle	1.743	0.133
F	Rag2	left LMol	1.138	0.049
F	Rag2	left mammillary bodies	0.234	0.020
F	Rag2	left mammilothalamic tract	0.118	0.009
F	Rag2	left Medial amygdala	0.473	0.029
F	Rag2	left Medial entorhinal cortex	0.328	0.019
F	Rag2	left medial lemniscus/medial longitudinal fasciculus	1.217	0.062
F	Rag2	left Medial orbital cortex	0.917	0.053
F	Rag2	left Medial parietal association cortex	0.219	0.014
F	Rag2	left Medial preoptic nucleus	0.092	0.008
F	Rag2	left medial septum	0.617	0.022
F	Rag2	left MoDG	2.096	0.084
F	Rag2	left nucleus accumbens	2.016	0.113
F	Rag2	left nucleus interpositus	0.215	0.014
F	Rag2	left Olfactory bulb: external plexiform layer	3.005	0.105
F	Rag2	left Olfactory bulb: glomerular layer	2.017	0.095
F	Rag2	left Olfactory bulb: granule cell layer	2.101	0.092
F	Rag2	left Olfactory bulb: internal plexiform layer	0.475	0.019
F	Rag2	left Olfactory bulb: mitral cell layer	0.602	0.021
F	Rag2	left olfactory peduncle	1.093	0.062
F	Rag2	left olfactory tubercle	1.585	0.098
F	Rag2	left optic tract	0.738	0.054
F	Rag2	left paraflocculus (PFL)	1.613	0.258
F	Rag2	left paraflocculus white matter	0.136	0.018
F	Rag2	left paramedian lobule	0.070	0.008
F	Rag2	left paramedian lobule (lobule 7)	1.950	0.124
F	Rag2	left Parietal cortex: posterior area: rostral part	0.071	0.005
F	Rag2	left Perirhinal cortex	1.212	0.065
F	Rag2	left Piriform cortex	4.751	0.199
F	Rag2	left PoDG	0.293	0.019
F	Rag2	left pontine nucleus	0.401	0.078
F	Rag2	left Posterolateral cortical amygdaloid area	0.382	0.037
F	Rag2	left Posteromedial cortical amygdaloid area	0.571	0.052
F	Rag2	left pre-para subiculum	1.112	0.068
F	Rag2	left Primary auditory cortex	0.711	0.038
F	Rag2	left Primary motor cortex	3.621	0.123
F	Rag2	left Primary somatosensory cortex	1.931	0.091
F	Rag2	left Primary somatosensory cortex: barrel field	4.816	0.362
F	Rag2	left Primary somatosensory cortex: dysgranular zone	0.172	0.014
F	Rag2	left Primary somatosensory cortex: forelimb region	2.010	0.128
F	Rag2	left Primary somatosensory cortex: hindlimb region	1.436	0.104
F	Rag2	left Primary somatosensory cortex: jaw region	0.282	0.032
F	Rag2	left Primary somatosensory cortex: shoulder region	0.091	0.009
F	Rag2	left Primary somatosensory cortex: trunk region	0.295	0.026
F	Rag2	left Primary somatosensory cortex: upper lip region	2.311	0.152
F	Rag2	left Primary visual cortex	1.181	0.093
F	Rag2	left Primary visual cortex: binocular area	1.030	0.087
F	Rag2	left Primary visual cortex: monocular area	1.188	0.116
F	Rag2	left Rostral amygdalopiriform area	0.182	0.015
F	Rag2	left Secondary auditory cortex: dorsal area	0.757	0.086
F	Rag2	left Secondary auditory cortex: ventral area	0.744	0.063
F	Rag2	left Secondary motor cortex	3.309	0.107
F	Rag2	left Secondary somatosensory cortex	3.179	0.266
F	Rag2	left Secondary visual cortex: lateral area	1.332	0.109
F	Rag2	left Secondary visual cortex: mediolateral area	0.577	0.038
F	Rag2	left Secondary visual cortex: mediomedial area	0.901	0.067
F	Rag2	left simple lobule (lobule 6)	2.255	0.125
F	Rag2	left simple lobule white matter	0.205	0.021
F	Rag2	left SLu	0.355	0.025
F	Rag2	left stria medullaris	0.310	0.015
F	Rag2	left stria terminalis	0.450	0.027
F	Rag2	left striatum	10.297	0.508
F	Rag2	left subependymale zone / rhinocele	0.026	0.003
F	Rag2	left subiculum	1.560	0.079
F	Rag2	left superior olivary complex	0.368	0.038
F	Rag2	left Temporal association area	1.299	0.083
F	Rag2	left thalamus	8.586	0.469
F	Rag2	left trunk of crus 2 and paramedian white matter	0.163	0.014
F	Rag2	left trunk of simple and crus 1 white matter	0.068	0.003
F	Rag2	left Ventral intermediate entorhinal cortex	0.536	0.037
F	Rag2	left Ventral nucleus of the endopiriform claustrum	0.244	0.026
F	Rag2	left Ventral orbital cortex	0.731	0.041
F	Rag2	left Ventral tenia tecta	0.049	0.005
F	Rag2	lobule 1-2 white matter	0.073	0.013
F	Rag2	lobule 10 white matter	0.077	0.006
F	Rag2	lobule 10: nodulus	1.283	0.086
F	Rag2	lobule 3 white matter	0.182	0.015
F	Rag2	lobule 3: central lobule (dorsal)	2.003	0.168
F	Rag2	lobule 6: declive	2.360	0.182
F	Rag2	lobule 7: tuber (or folium)	0.868	0.113
F	Rag2	lobule 8 white matter	0.145	0.010
F	Rag2	lobule 8: pyramis	1.481	0.111
F	Rag2	lobule 9 white matter	0.272	0.018
F	Rag2	lobule 9: uvula	2.572	0.156
F	Rag2	lobules 1-2: lingula and central lobule (ventral)	1.709	0.186
F	Rag2	lobules 4-5 white matter	0.527	0.029
F	Rag2	lobules 4-5: culmen (ventral and dorsal)	4.042	0.205
F	Rag2	lobules 6-7 white matter	0.655	0.049
F	Rag2	medulla	25.888	0.983
F	Rag2	midbrain	13.134	0.748
F	Rag2	periaqueductal grey	3.749	0.197
F	Rag2	pons	16.426	0.566
F	Rag2	posterior commissure	0.137	0.011
F	Rag2	right Accessory olfactory bulb: glomerular, external plexiform and mitral cell layer	0.239	0.015
F	Rag2	right Accessory olfactory bulb: granule cell layer	0.113	0.008
F	Rag2	right amygdala	4.316	0.128
F	Rag2	right Amygdalopiriform transition area	0.482	0.025
F	Rag2	right anterior commissure: pars anterior	0.462	0.027
F	Rag2	right anterior commissure: pars posterior	0.192	0.013
F	Rag2	right anterior lobule (lobules 4-5)	0.740	0.048
F	Rag2	right anterior lobule white matter	0.048	0.003
F	Rag2	right Anterior olfactory nucleus	0.979	0.075
F	Rag2	right basal forebrain	2.560	0.093
F	Rag2	right bed nucleus of stria terminalis	0.604	0.038
F	Rag2	right CA10r	0.955	0.047
F	Rag2	right CA1Py	0.522	0.020
F	Rag2	right CA1Rad	1.269	0.046
F	Rag2	right CA20r	0.213	0.017
F	Rag2	right CA2Py	0.095	0.006
F	Rag2	right CA2Rad	0.202	0.010
F	Rag2	right CA30r	1.336	0.052
F	Rag2	right CA3Py Inner	0.074	0.003
F	Rag2	right CA3Py Outer	0.483	0.023
F	Rag2	right CA3Rad	0.886	0.041
F	Rag2	right Caudomedial entorhinal cortex	2.687	0.134
F	Rag2	right cerebellar peduncle: inferior	0.361	0.017
F	Rag2	right cerebellar peduncle: middle	0.584	0.031
F	Rag2	right cerebellar peduncle: superior	0.472	0.037
F	Rag2	right cerebral peduncle	0.990	0.035
F	Rag2	right Cingulate cortex: area 24a	0.997	0.060
F	Rag2	right Cingulate cortex: area 24a'	0.370	0.046
F	Rag2	right Cingulate cortex: area 24b	0.784	0.101
F	Rag2	right Cingulate cortex: area 24b'	0.306	0.021
F	Rag2	right Cingulate cortex: area 25	0.285	0.019
F	Rag2	right Cingulate cortex: area 29a	0.346	0.014
F	Rag2	right Cingulate cortex: area 29b	0.199	0.016
F	Rag2	right Cingulate cortex: area 29c	0.881	0.033
F	Rag2	right Cingulate cortex: area 30	1.316	0.072
F	Rag2	right Cingulate cortex: area 32	1.060	0.106
F	Rag2	right Cingulum	0.413	0.034
F	Rag2	right Claustrum	0.137	0.007
F	Rag2	right Claustrum: dorsal part	0.130	0.016
F	Rag2	right Claustrum: ventral part	0.284	0.024
F	Rag2	right colliculus: inferior	2.630	0.125
F	Rag2	right colliculus: superior	4.236	0.270
F	Rag2	right copula white matter	0.032	0.004
F	Rag2	right copula: pyramis (lobule 8)	1.205	0.113
F	Rag2	right corpus callosum	5.471	0.287
F	Rag2	right Cortex-amygdala transition zones	0.395	0.033
F	Rag2	right corticospinal tract/pyramids	0.868	0.059
F	Rag2	right crus 1 white matter	0.163	0.010
F	Rag2	right crus 1: ansiform lobule (lobule 6)	2.026	0.113
F	Rag2	right crus 2 white matter	0.138	0.013
F	Rag2	right crus 2: ansiform lobule (lobule 7)	1.877	0.145
F	Rag2	right cuneate nucleus	0.121	0.016
F	Rag2	right dentate nucleus	0.164	0.010
F	Rag2	right Dorsal intermediate entorhinal cortex	0.849	0.049
F	Rag2	right Dorsal nucleus of the endopiriform	0.673	0.033
F	Rag2	right Dorsal tenia tecta	0.434	0.024
F	Rag2	right Dorsolateral entorhinal cortex	1.246	0.066
F	Rag2	right Dorsolateral orbital cortex	0.391	0.033
F	Rag2	right Ectorhinal cortex	1.282	0.107
F	Rag2	right facial nerve (cranial nerve 7)	0.106	0.007
F	Rag2	right fasciculus retroflexus	0.110	0.007
F	Rag2	right fastigial nucleus	0.221	0.012
F	Rag2	right fimbria	1.430	0.070
F	Rag2	right flocculus (FL)	0.477	0.043
F	Rag2	right flocculus white matter	0.036	0.005
F	Rag2	right fornix	0.278	0.016
F	Rag2	right Frontal association cortex	3.911	0.219
F	Rag2	right Frontal cortex: area 3	0.343	0.028
F	Rag2	right fundus of striatum	0.078	0.007
F	Rag2	right globus pallidus	1.560	0.091
F	Rag2	right GrDG	0.610	0.024
F	Rag2	right habenular commissure	0.014	0.004
F	Rag2	right hypothalamus	4.936	0.176
F	Rag2	right inferior olivary complex	0.165	0.025
F	Rag2	right Insular region: not subdivided	3.738	0.264
F	Rag2	right Intermediate nucleus of the endopiriform claustrum	0.264	0.016
F	Rag2	right internal capsule	1.430	0.077
F	Rag2	right lateral olfactory tract	0.622	0.034
F	Rag2	right Lateral orbital cortex	1.663	0.090
F	Rag2	right Lateral parietal association cortex	0.087	0.012
F	Rag2	right lateral septum	1.426	0.092
F	Rag2	right lateral ventricle	1.770	0.106
F	Rag2	right LMol	1.111	0.036
F	Rag2	right mammillary bodies	0.219	0.017
F	Rag2	right mammilothalamic tract	0.124	0.011
F	Rag2	right Medial amygdala	0.509	0.033
F	Rag2	right Medial entorhinal cortex	0.313	0.022
F	Rag2	right medial lemniscus/medial longitudinal fasciculus	1.275	0.061
F	Rag2	right Medial orbital cortex	0.823	0.044
F	Rag2	right Medial parietal association cortex	0.163	0.013
F	Rag2	right Medial preoptic nucleus	0.086	0.005
F	Rag2	right medial septum	0.577	0.029
F	Rag2	right MoDG	1.953	0.064
F	Rag2	right nucleus accumbens	1.986	0.090
F	Rag2	right nucleus interpositus	0.223	0.013
F	Rag2	right Olfactory bulb: external plexiform layer	3.011	0.135
F	Rag2	right Olfactory bulb: glomerular layer	2.105	0.114
F	Rag2	right Olfactory bulb: granule cell layer	2.152	0.095
F	Rag2	right Olfactory bulb: internal plexiform layer	0.454	0.035
F	Rag2	right Olfactory bulb: mitral cell layer	0.557	0.034
F	Rag2	right olfactory peduncle	1.061	0.056
F	Rag2	right olfactory tubercle	1.585	0.065
F	Rag2	right optic tract	0.685	0.026
F	Rag2	right paraflocculus (PFL)	1.556	0.243
F	Rag2	right paraflocculus white matter	0.148	0.020
F	Rag2	right paramedian lobule	0.077	0.009
F	Rag2	right paramedian lobule (lobule 7)	1.918	0.167
F	Rag2	right Parietal cortex: posterior area: rostral part	0.043	0.005
F	Rag2	right Perirhinal cortex	1.154	0.077
F	Rag2	right Piriform cortex	5.087	0.171
F	Rag2	right PoDG	0.300	0.015
F	Rag2	right pontine nucleus	0.383	0.052
F	Rag2	right Posterolateral cortical amygdaloid area	0.421	0.042
F	Rag2	right Posteromedial cortical amygdaloid area	0.435	0.031
F	Rag2	right pre-para subiculum	1.129	0.044
F	Rag2	right Primary auditory cortex	0.715	0.048
F	Rag2	right Primary motor cortex	3.092	0.147
F	Rag2	right Primary somatosensory cortex	1.967	0.154
F	Rag2	right Primary somatosensory cortex: barrel field	4.610	0.235
F	Rag2	right Primary somatosensory cortex: dysgranular zone	0.184	0.013
F	Rag2	right Primary somatosensory cortex: forelimb region	1.798	0.130
F	Rag2	right Primary somatosensory cortex: hindlimb region	1.159	0.057
F	Rag2	right Primary somatosensory cortex: jaw region	0.288	0.024
F	Rag2	right Primary somatosensory cortex: shoulder region	0.065	0.011
F	Rag2	right Primary somatosensory cortex: trunk region	0.188	0.021
F	Rag2	right Primary somatosensory cortex: upper lip region	2.649	0.139
F	Rag2	right Primary visual cortex	1.173	0.089

F	Rag2	right Primary visual cortex: binocular area	0.987	0.085
F	Rag2	right Primary visual cortex: monocular area	0.788	0.076
F	Rag2	right Rostral amygdalopiriform area	0.205	0.018
F	Rag2	right Secondary auditory cortex: dorsal area	0.698	0.042
F	Rag2	right Secondary auditory cortex: ventral area	0.911	0.081
F	Rag2	right Secondary motor cortex	3.150	0.174
F	Rag2	right Secondary somatosensory cortex	3.367	0.312
F	Rag2	right Secondary visual cortex: lateral area	1.434	0.098
F	Rag2	right Secondary visual cortex: mediolateral area	0.399	0.062
F	Rag2	right Secondary visual cortex: mediomedial area	0.777	0.063
F	Rag2	right simple lobule (lobule 6)	2.218	0.131
F	Rag2	right simple lobule white matter	0.162	0.012
F	Rag2	right SLu	0.348	0.019
F	Rag2	right stria medullaris	0.330	0.015
F	Rag2	right stria terminalis	0.375	0.016
F	Rag2	right striatum	10.069	0.485
F	Rag2	right subependymale zone / rhinocele	0.024	0.003
F	Rag2	right subiculum	1.535	0.059
F	Rag2	right superior olivary complex	0.386	0.039
F	Rag2	right Temporal association area	1.467	0.103
F	Rag2	right thalamus	8.448	0.492
F	Rag2	right trunk of crus 2 and paramedian white matter	0.166	0.012
F	Rag2	right trunk of simple and crus 1 white matter	0.081	0.005
F	Rag2	right Ventral intermediate entorhinal cortex	0.519	0.029
F	Rag2	right Ventral nucleus of the endopiriform claustrum	0.230	0.024
F	Rag2	right Ventral orbital cortex	0.655	0.036
F	Rag2	right Ventral tenia tecta	0.051	0.005
F	Rag2	third ventricle	1.236	0.107
F	Rag2	trunk of arbor vita	3.962	0.149
F	Rag2	trunk of lobules 1-3 white matter	0.125	0.010
F	Rag2	trunk of lobules 6-8 white matter	0.104	0.008
F	Rag2	ventral tegmental decussation	0.124	0.009
M	WT	cerebral aqueduct	0.523	0.055
M	WT	fourth ventricle	0.954	0.056
M	WT	interpeduncular nucleus	0.272	0.014
M	WT	left Accessory olfactory bulb: glomerular, external plexiform and mitral cell layer	0.263	0.013
M	WT	left Accessory olfactory bulb: granule cell layer	0.127	0.006
M	WT	left amygdala	4.957	0.188
M	WT	left Amygdalopiriform transition area	0.535	0.025
M	WT	left anterior commissure: pars anterior	0.492	0.023
M	WT	left anterior commissure: pars posterior	0.213	0.011
M	WT	left anterior lobule (lobules 4-5)	0.864	0.063
M	WT	left anterior lobule white matter	0.043	0.004
M	WT	left Anterior olfactory nucleus	0.996	0.043
M	WT	left basal forebrain	2.609	0.106
M	WT	left bed nucleus of stria terminalis	0.696	0.030
M	WT	left CA10r	1.176	0.048
M	WT	left CA1Py	0.562	0.021
M	WT	left CA1Rad	1.256	0.037
M	WT	left CA20r	0.262	0.014
M	WT	left CA2Py	0.103	0.004
M	WT	left CA2Rad	0.208	0.010
M	WT	left CA30r	1.416	0.084
M	WT	left CA3Py Inner	0.078	0.005
M	WT	left CA3Py Outer	0.516	0.030
M	WT	left CA3Rad	0.917	0.048
M	WT	left Caudomedial entorhinal cortex	2.982	0.142
M	WT	left cerebellar peduncle: inferior	0.420	0.015
M	WT	left cerebellar peduncle: middle	0.583	0.028
M	WT	left cerebellar peduncle: superior	0.509	0.036
M	WT	left cerebral peduncle	1.032	0.036
M	WT	left Cingulate cortex: area 24a	0.938	0.044
M	WT	left Cingulate cortex: area 24a'	0.386	0.041
M	WT	left Cingulate cortex: area 24b	0.826	0.099
M	WT	left Cingulate cortex: area 24b'	0.304	0.020
M	WT	left Cingulate cortex: area 25	0.293	0.021
M	WT	left Cingulate cortex: area 29a	0.380	0.018
M	WT	left Cingulate cortex: area 29b	0.218	0.020
M	WT	left Cingulate cortex: area 29c	0.954	0.060
M	WT	left Cingulate cortex: area 30	1.477	0.066
M	WT	left Cingulate cortex: area 32	1.346	0.115
M	WT	left Cingulum	0.443	0.026
M	WT	left Claustrum	0.171	0.012
M	WT	left Claustrum: dorsal part	0.144	0.022
M	WT	left Claustrum: ventral part	0.269	0.039
M	WT	left colliculus: inferior	2.836	0.108
M	WT	left colliculus: superior	4.366	0.237
M	WT	left copula white matter	0.044	0.003
M	WT	left copula: pyramis (lobule 8)	1.156	0.062
M	WT	left corpus callosum	6.070	0.325
M	WT	left Cortex-amygdala transition zones	0.398	0.025
M	WT	left corticospinal tract/pyramids	0.883	0.038
M	WT	left crus 1 white matter	0.185	0.012
M	WT	left crus 1: ansiform lobule (lobule 6)	2.149	0.100
M	WT	left crus 2 white matter	0.124	0.011
M	WT	left crus 2: ansiform lobule (lobule 7)	1.889	0.087
M	WT	left cuneate nucleus	0.123	0.008
M	WT	left dentate nucleus	0.182	0.012
M	WT	left Dorsal intermediate entorhinal cortex	0.867	0.049
M	WT	left Dorsal nucleus of the endopiriform	0.734	0.036
M	WT	left Dorsal tenia tecta	0.493	0.032
M	WT	left Dorsolateral entorhinal cortex	1.222	0.054
M	WT	left Dorsolateral orbital cortex	0.436	0.030
M	WT	left Ectorhinal cortex	1.355	0.093
M	WT	left facial nerve (cranial nerve 7)	0.107	0.006
M	WT	left fasciculus retroflexus	0.129	0.007
M	WT	left fastigial nucleus	0.245	0.014
M	WT	left fimbria	1.648	0.091
M	WT	left flocculus (FL)	0.545	0.037
M	WT	left flocculus white matter	0.029	0.004
M	WT	left fornix	0.337	0.021
M	WT	left Frontal association cortex	3.512	0.157
M	WT	left Frontal cortex: area 3	0.355	0.012
M	WT	left fundus of striatum	0.075	0.004
M	WT	left globus pallidus	1.614	0.070
M	WT	left GrDG	0.683	0.031
M	WT	left habenular commissure	0.015	0.002
M	WT	left hypothalamus	5.315	0.181
M	WT	left inferior olivary complex	0.170	0.023
M	WT	left Insular region: not subdivided	3.749	0.252
M	WT	left Intermediate nucleus of the endopiriform claustrum	0.316	0.016
M	WT	left internal capsule	1.429	0.057
M	WT	left lateral olfactory tract	0.749	0.030
M	WT	left Lateral orbital cortex	1.786	0.064
M	WT	left Lateral parietal association cortex	0.145	0.011
M	WT	left lateral septum	1.525	0.061
M	WT	left lateral ventricle	1.795	0.212
M	WT	left LMol	1.197	0.035
M	WT	left mammillary bodies	0.236	0.013
M	WT	left mammilothalamic tract	0.124	0.008
M	WT	left Medial amygdala	0.544	0.032
M	WT	left Medial entorhinal cortex	0.365	0.018
M	WT	left medial lemniscus/medial longitudinal fasciculus	1.252	0.056
M	WT	left Medial orbital cortex	0.923	0.068
M	WT	left Medial parietal association cortex	0.232	0.024
M	WT	left Medial preoptic nucleus	0.103	0.004
M	WT	left medial septum	0.660	0.026
M	WT	left MoDG	2.196	0.080
M	WT	left nucleus accumbens	2.102	0.068
M	WT	left nucleus interpositus	0.228	0.015
M	WT	left Olfactory bulb: external plexiform layer	3.284	0.094
M	WT	left Olfactory bulb: glomerular layer	2.247	0.074
M	WT	left Olfactory bulb: granule cell layer	2.317	0.089
M	WT	left Olfactory bulb: internal plexiform layer	0.524	0.027
M	WT	left Olfactory bulb: mitral cell layer	0.657	0.026
M	WT	left olfactory peduncle	1.176	0.034
M	WT	left olfactory tubercle	1.661	0.101
M	WT	left optic tract	0.778	0.036
M	WT	left paraflocculus (PFL)	1.703	0.219
M	WT	left paraflocculus white matter	0.141	0.015
M	WT	left paramedian lobule	0.072	0.005
M	WT	left paramedian lobule (lobule 7)	2.046	0.087
M	WT	left Parietal cortex: posterior area: rostral part	0.070	0.005
M	WT	left Perirhinal cortex	1.282	0.066
M	WT	left Piriform cortex	5.173	0.181
M	WT	left PoDG	0.308	0.014
M	WT	left pontine nucleus	0.387	0.040
M	WT	left Posterolateral cortical amygdaloid area	0.425	0.023
M	WT	left Posteromedial cortical amygdaloid area	0.614	0.041
M	WT	left pre-para subiculum	1.147	0.049
M	WT	left Primary auditory cortex	0.714	0.051
M	WT	left Primary motor cortex	3.678	0.139
M	WT	left Primary somatosensory cortex	1.989	0.114
M	WT	left Primary somatosensory cortex: barrel field	4.589	0.216
M	WT	left Primary somatosensory cortex: dysgranular zone	0.165	0.015
M	WT	left Primary somatosensory cortex: forelimb region	2.015	0.132
M	WT	left Primary somatosensory cortex: hindlimb region	1.454	0.079
M	WT	left Primary somatosensory cortex: jaw region	0.259	0.034
M	WT	left Primary somatosensory cortex: shoulder region	0.090	0.008
M	WT	left Primary somatosensory cortex: trunk region	0.298	0.023
M	WT	left Primary somatosensory cortex: upper lip region	2.330	0.139
M	WT	left Primary visual cortex	1.190	0.087
M	WT	left Primary visual cortex: binocular area	0.995	0.054
M	WT	left Primary visual cortex: monocular area	1.169	0.075
M	WT	left Rostral amygdalopiriform area	0.198	0.015
M	WT	left Secondary auditory cortex: dorsal area	0.698	0.048
M	WT	left Secondary auditory cortex: ventral area	0.773	0.085
M	WT	left Secondary motor cortex	3.389	0.182
M	WT	left Secondary somatosensory cortex	3.331	0.246
M	WT	left Secondary visual cortex: lateral area	1.313	0.065
M	WT	left Secondary visual cortex: mediolateral area	0.583	0.030
M	WT	left Secondary visual cortex: mediomedial area	0.967	0.047
M	WT	left simple lobule (lobule 6)	2.346	0.095
M	WT	left simple lobule white matter	0.213	0.012
M	WT	left SLu	0.376	0.025
M	WT	left stria medullaris	0.324	0.012
M	WT	left stria terminalis	0.471	0.022
M	WT	left striatum	10.551	0.427
M	WT	left subependymale zone / rhinocele	0.029	0.003
M	WT	left subiculum	1.661	0.057
M	WT	left superior olivary complex	0.374	0.025
M	WT	left Temporal association area	1.416	0.112
M	WT	left thalamus	8.827	0.347
M	WT	left trunk of crus 2 and paramedian white matter	0.161	0.011
M	WT	left trunk of simple and crus 1 white matter	0.065	0.004
M	WT	left Ventral intermediate entorhinal cortex	0.569	0.029
M	WT	left Ventral nucleus of the endopiriform claustrum	0.253	0.022
M	WT	left Ventral orbital cortex	0.771	0.044
M	WT	left Ventral tenia tecta	0.057	0.004
M	WT	lobule 1-2 white matter	0.068	0.019
M	WT	lobule 10 white matter	0.082	0.007
M	WT	lobule 10: nodulus	1.362	0.060
M	WT	lobule 3 white matter	0.182	0.018
M	WT	lobule 3: central lobule (dorsal)	2.137	0.179
M	WT	lobule 6: declive	2.353	0.134
M	WT	lobule 7: tuber (or folium)	0.912	0.054
M	WT	lobule 8 white matter	0.151	0.009
M	WT	lobule 8: pyramis	1.563	0.078
M	WT	lobule 9 white matter	0.280	0.017
M	WT	lobule 9: uvula	2.720	0.133
M	WT	lobules 1-2: lingula and central lobule (ventral)	1.678	0.234
M	WT	lobules 4-5 white matter	0.549	0.043
M	WT	lobules 4-5: culmen (ventral and dorsal)	4.246	0.275
M	WT	lobules 6-7 white matter	0.654	0.046
M	WT	medulla	27.578	0.767
M	WT	midbrain	13.693	0.632
M	WT	periaqueductal grey	3.950	0.183
M	WT	pons	17.105	0.584
M	WT	posterior commissure	0.142	0.009
M	WT	right Accessory olfactory bulb: glomerular, external plexiform and mitral cell layer	0.252	0.013
M	WT	right Accessory olfactory bulb: granule cell layer	0.120	0.007
M	WT	right amygdala	4.632	0.202
M	WT	right Amygdalopiriform transition area	0.489	0.026
M	WT	right anterior commissure: pars anterior	0.488	0.019
M	WT	right anterior commissure: pars posterior	0.201	0.009
M	WT	right anterior lobule (lobules 4-5)	0.770	0.058
M	WT	right anterior lobule white matter	0.050	0.005
M	WT	right Anterior olfactory nucleus	1.021	0.043
M	WT	right basal forebrain	2.706	0.094
M	WT	right bed nucleus of stria terminalis	0.686	0.037
M	WT	right CA10r	1.025	0.063
M	WT	right CA1Py	0.563	0.031
M	WT	right CA1Rad	1.357	0.047
M	WT	right CA20r	0.213	0.010
M	WT	right CA2Py	0.099	0.003
M	WT	right CA2Rad	0.220	0.008
M	WT	right CA30r	1.420	0.076
M	WT	right CA3Py Inner	0.080	0.003
M	WT	right CA3Py Outer	0.518	0.025
M	WT	right CA3Rad	0.952	0.042
M	WT	right Caudomedial entorhinal cortex	2.871	0.101
M	WT	right cerebellar peduncle: inferior	0.396	0.013
M	WT	right cerebellar peduncle: middle	0.612	0.029
M	WT	right cerebellar peduncle: superior	0.496	0.027
M	WT	right cerebral peduncle	1.041	0.044
M	WT	right Cingulate cortex: area 24a	0.987	0.048
M	WT	right Cingulate cortex: area 24a'	0.362	0.041
M	WT	right Cingulate cortex: area 24b	0.775	0.075
M	WT	right Cingulate cortex: area 24b'	0.297	0.020
M	WT	right Cingulate cortex: area 25	0.303	0.021
M	WT	right Cingulate cortex: area 29a	0.363	0.016
M	WT	right Cingulate cortex: area 29b	0.205	0.019
M	WT	right Cingulate cortex: area 29c	0.906	0.039
M	WT	right Cingulate cortex: area 30	1.382	0.093
M	WT	right Cingulate cortex: area 32	1.063	0.072
M	WT	right Cingulum	0.447	0.027
M	WT	right Claustrum	0.142	0.007
M	WT	right Claustrum: dorsal part	0.133	0.015
M	WT	right Claustrum: ventral part	0.303	0.030
M	WT	right colliculus: inferior	2.702	0.110
M	WT	right colliculus: superior	4.413	0.223
M	WT	right copula white matter	0.034	0.002
M	WT	right copula: pyramis (lobule 8)	1.287	0.074
M	WT	right corpus callosum	5.795	0.214
M	WT	right Cortex-amygdala transition zones	0.429	0.027
M	WT	right corticospinal tract/pyramids	0.915	0.044
M	WT	right crus 1 white matter	0.180	0.008
M	WT	right crus 1: ansiform lobule (lobule 6)	2.162	0.077
M	WT	right crus 2 white matter	0.145	0.008
M	WT	right crus 2: ansiform lobule (lobule 7)	1.957	0.085
M	WT	right cuneate nucleus	0.130	0.009
M	WT	right dentate nucleus	0.180	0.010
M	WT	right Dorsal intermediate entorhinal cortex	0.892	0.045
M	WT	right Dorsal nucleus of the endopiriform	0.711	0.030
M	WT	right Dorsal tenia tecta	0.458	0.028
M	WT	right Dorsolateral entorhinal cortex	1.360	0.088
M	WT	right Dorsolateral orbital cortex	0.398	0.029

M	WT	right Ectorhinal cortex	1.405	0.109
M	WT	right facial nerve (cranial nerve 7)	0.110	0.003
M	WT	right fasciculus retroflexus	0.114	0.006
M	WT	right fastigial nucleus	0.233	0.017
M	WT	right fimbria	1.525	0.094
M	WT	right flocculus (FL)	0.513	0.025
M	WT	right flocculus white matter	0.039	0.004
M	WT	right fornix	0.299	0.015
M	WT	right Frontal association cortex	3.849	0.144
M	WT	right Frontal cortex: area 3	0.363	0.016
M	WT	right fundus of striatum	0.081	0.006
M	WT	right globus pallidus	1.642	0.085
M	WT	right GrDG	0.645	0.024
M	WT	right habenular commissure	0.014	0.003
M	WT	right hypothalamus	5.264	0.161
M	WT	right inferior olivary complex	0.184	0.022
M	WT	right Insular region: not subdivided	3.990	0.328
M	WT	right Intermediate nucleus of the endopiriform claustrum	0.284	0.013
M	WT	right internal capsule	1.505	0.062
M	WT	right lateral olfactory tract	0.661	0.035
M	WT	right Lateral orbital cortex	1.737	0.092
M	WT	right Lateral parietal association cortex	0.088	0.009
M	WT	right lateral septum	1.531	0.078
M	WT	right lateral ventricle	1.959	0.227
M	WT	right LMol	1.176	0.029
M	WT	right mammillary bodies	0.216	0.013
M	WT	right mammilothalamic tract	0.127	0.009
M	WT	right Medial amygdala	0.574	0.026
M	WT	right Medial entorhinal cortex	0.337	0.015
M	WT	right medial lemniscus/medial longitudinal fasciculus	1.351	0.046
M	WT	right Medial orbital cortex	0.830	0.053
M	WT	right Medial parietal association cortex	0.176	0.026
M	WT	right Medial preoptic nucleus	0.094	0.006
M	WT	right medial septum	0.618	0.025
M	WT	right MoDG	2.049	0.069
M	WT	right nucleus accumbens	2.074	0.084
M	WT	right nucleus interpositus	0.238	0.017
M	WT	right Olfactory bulb: external plexiform layer	3.280	0.085
M	WT	right Olfactory bulb: glomerular layer	2.326	0.089
M	WT	right Olfactory bulb: granule cell layer	2.361	0.095
M	WT	right Olfactory bulb: internal plexiform layer	0.511	0.024
M	WT	right Olfactory bulb: mitral cell layer	0.617	0.024
M	WT	right olfactory peduncle	1.144	0.038
M	WT	right olfactory tubercle	1.693	0.089
M	WT	right optic tract	0.737	0.028
M	WT	right paraflocculus (PFL)	1.607	0.225
M	WT	right paraflocculus white matter	0.155	0.018
M	WT	right paramedian lobule	0.081	0.008
M	WT	right paramedian lobule (lobule 7)	2.014	0.094
M	WT	right Parietal cortex: posterior area: rostral part	0.044	0.005
M	WT	right Perirhinal cortex	1.223	0.064
M	WT	right Piriform cortex	5.379	0.198
M	WT	right PoDG	0.321	0.012
M	WT	right pontine nucleus	0.376	0.035
M	WT	right Posterolateral cortical amygdaloid area	0.454	0.029
M	WT	right Posteromedial cortical amygdaloid area	0.493	0.044
M	WT	right pre-para subiculum	1.166	0.042
M	WT	right Primary auditory cortex	0.727	0.045
M	WT	right Primary motor cortex	3.150	0.123
M	WT	right Primary somatosensory cortex	2.062	0.065
M	WT	right Primary somatosensory cortex: barrel field	4.594	0.151
M	WT	right Primary somatosensory cortex: dysgranular zone	0.179	0.014
M	WT	right Primary somatosensory cortex: forelimb region	1.800	0.104
M	WT	right Primary somatosensory cortex: hindlimb region	1.191	0.081
M	WT	right Primary somatosensory cortex: jaw region	0.284	0.021
M	WT	right Primary somatosensory cortex: shoulder region	0.069	0.007
M	WT	right Primary somatosensory cortex: trunk region	0.199	0.019
M	WT	right Primary somatosensory cortex: upper lip region	2.767	0.110
M	WT	right Primary visual cortex	1.198	0.081
M	WT	right Primary visual cortex: binocular area	1.031	0.054
M	WT	right Primary visual cortex: monocular area	0.784	0.062
M	WT	right Rostral amygdalopiriform area	0.196	0.015
M	WT	right Secondary auditory cortex: dorsal area	0.690	0.047
M	WT	right Secondary auditory cortex: ventral area	0.967	0.061
M	WT	right Secondary motor cortex	3.225	0.159
M	WT	right Secondary somatosensory cortex	3.471	0.319
M	WT	right Secondary visual cortex: lateral area	1.483	0.077
M	WT	right Secondary visual cortex: mediolateral area	0.395	0.046
M	WT	right Secondary visual cortex: mediomedial area	0.803	0.067
M	WT	right simple lobule (lobule 6)	2.356	0.098
M	WT	right simple lobule white matter	0.176	0.008
M	WT	right SLu	0.374	0.021
M	WT	right stria medullaris	0.347	0.014
M	WT	right stria terminalis	0.398	0.020
M	WT	right striatum	10.456	0.478
M	WT	right subependymale zone / rhinocele	0.026	0.002
M	WT	right subiculum	1.637	0.056
M	WT	right superior olivary complex	0.396	0.030
M	WT	right Temporal association area	1.593	0.114
M	WT	right thalamus	8.702	0.442
M	WT	right trunk of crus 2 and paramedian white matter	0.172	0.008
M	WT	right trunk of simple and crus 1 white matter	0.081	0.004
M	WT	right Ventral intermediate entorhinal cortex	0.546	0.021
M	WT	right Ventral nucleus of the endopiriform claustrum	0.234	0.020
M	WT	right Ventral orbital cortex	0.691	0.035
M	WT	right Ventral tenia tecta	0.058	0.004
M	WT	third ventricle	1.252	0.075
M	WT	trunk of arbor vita	4.152	0.158
M	WT	trunk of lobules 1-3 white matter	0.122	0.012
M	WT	trunk of lobules 6-8 white matter	0.104	0.007
M	WT	ventral tegmental decussation	0.126	0.009
M	CD4	cerebral aqueduct	0.484	0.029
M	CD4	fourth ventricle	0.937	0.060
M	CD4	interpeduncular nucleus	0.281	0.015
M	CD4	left Accessory olfactory bulb: glomerular, external plexiform and mitral cell layer	0.261	0.018
M	CD4	left Accessory olfactory bulb: granule cell layer	0.126	0.007
M	CD4	left amygdala	5.115	0.159
M	CD4	left Amygdalopiriform transition area	0.554	0.034
M	CD4	left anterior commissure: pars anterior	0.509	0.029
M	CD4	left anterior commissure: pars posterior	0.224	0.014
M	CD4	left anterior lobule (lobules 4-5)	0.871	0.057
M	CD4	left anterior lobule white matter	0.043	0.003
M	CD4	left Anterior olfactory nucleus	1.004	0.052
M	CD4	left basal forebrain	2.745	0.112
M	CD4	left bed nucleus of stria terminalis	0.724	0.043
M	CD4	left CA10r	1.216	0.058
M	CD4	left CA1Py	0.577	0.022
M	CD4	left CA1Rad	1.285	0.035
M	CD4	left CA20r	0.265	0.012
M	CD4	left CA2Py	0.108	0.006
M	CD4	left CA2Rad	0.215	0.009
M	CD4	left CA30r	1.452	0.079
M	CD4	left CA3Py Inner	0.082	0.004
M	CD4	left CA3Py Outer	0.539	0.035
M	CD4	left CA3Rad	0.968	0.055
M	CD4	left Caudomedial entorhinal cortex	3.078	0.102
M	CD4	left cerebellar peduncle: inferior	0.426	0.013
M	CD4	left cerebellar peduncle: middle	0.579	0.034
M	CD4	left cerebellar peduncle: superior	0.541	0.030
M	CD4	left cerebral peduncle	1.037	0.031
M	CD4	left Cingulate cortex: area 24a	0.965	0.036
M	CD4	left Cingulate cortex: area 24a'	0.392	0.027
M	CD4	left Cingulate cortex: area 24b	0.868	0.109
M	CD4	left Cingulate cortex: area 24b'	0.334	0.032
M	CD4	left Cingulate cortex: area 25	0.307	0.024
M	CD4	left Cingulate cortex: area 29a	0.372	0.027
M	CD4	left Cingulate cortex: area 29b	0.206	0.019
M	CD4	left Cingulate cortex: area 29c	0.985	0.060
M	CD4	left Cingulate cortex: area 30	1.445	0.069
M	CD4	left Cingulate cortex: area 32	1.369	0.105
M	CD4	left Cingulum	0.463	0.023
M	CD4	left Claustrum	0.181	0.018
M	CD4	left Claustrum: dorsal part	0.164	0.024
M	CD4	left Claustrum: ventral part	0.311	0.034
M	CD4	left colliculus: inferior	2.926	0.085
M	CD4	left colliculus: superior	4.548	0.206
M	CD4	left copula white matter	0.045	0.002
M	CD4	left copula: pyramis (lobule 8)	1.174	0.072
M	CD4	left corpus callosum	6.322	0.192
M	CD4	left Cortex-amygdala transition zones	0.397	0.043
M	CD4	left corticospinal tract/pyramids	0.873	0.046
M	CD4	left crus 1 white matter	0.194	0.012
M	CD4	left crus 1: ansiform lobule (lobule 6)	2.196	0.079
M	CD4	left crus 2 white matter	0.129	0.013
M	CD4	left crus 2: ansiform lobule (lobule 7)	1.988	0.111
M	CD4	left cuneate nucleus	0.120	0.013
M	CD4	left dentate nucleus	0.190	0.011
M	CD4	left Dorsal intermediate entorhinal cortex	0.890	0.050
M	CD4	left Dorsal nucleus of the endopiriform	0.787	0.051
M	CD4	left Dorsal tenia tecta	0.488	0.037
M	CD4	left Dorsolateral entorhinal cortex	1.285	0.064
M	CD4	left Dorsolateral orbital cortex	0.429	0.031
M	CD4	left Ectorhinal cortex	1.350	0.080
M	CD4	left facial nerve (cranial nerve 7)	0.107	0.005
M	CD4	left fasciculus retroflexus	0.135	0.009
M	CD4	left fastigial nucleus	0.259	0.015
M	CD4	left fimbria	1.741	0.067
M	CD4	left flocculus (FL)	0.546	0.032
M	CD4	left flocculus white matter	0.029	0.003
M	CD4	left fornix	0.352	0.016
M	CD4	left Frontal association cortex	3.601	0.207
M	CD4	left Frontal cortex: area 3	0.366	0.019
M	CD4	left fundus of striatum	0.078	0.006
M	CD4	left globus pallidus	1.708	0.080
M	CD4	left GrDG	0.709	0.022
M	CD4	left habenular commissure	0.014	0.004
M	CD4	left hypothalamus	5.484	0.181
M	CD4	left inferior olivary complex	0.161	0.025
M	CD4	left Insular region: not subdivided	4.005	0.160
M	CD4	left Intermediate nucleus of the endopiriform claustrum	0.330	0.026
M	CD4	left internal capsule	1.487	0.059
M	CD4	left lateral olfactory tract	0.740	0.027
M	CD4	left Lateral orbital cortex	1.808	0.093
M	CD4	left Lateral parietal association cortex	0.155	0.012
M	CD4	left lateral septum	1.608	0.108
M	CD4	left lateral ventricle	2.005	0.275
M	CD4	left LMol	1.223	0.034
M	CD4	left mammillary bodies	0.236	0.018
M	CD4	left mammilothalamic tract	0.128	0.008
M	CD4	left Medial amygdala	0.560	0.029
M	CD4	left Medial entorhinal cortex	0.370	0.019
M	CD4	left medial lemniscus/medial longitudinal fasciculus	1.278	0.052
M	CD4	left Medial orbital cortex	0.917	0.049
M	CD4	left Medial parietal association cortex	0.245	0.024
M	CD4	left Medial preoptic nucleus	0.104	0.008
M	CD4	left medial septum	0.678	0.033
M	CD4	left MoDG	2.255	0.062
M	CD4	left nucleus accumbens	2.234	0.123
M	CD4	left nucleus interpositus	0.240	0.015
M	CD4	left Olfactory bulb: external plexiform layer	3.234	0.117
M	CD4	left Olfactory bulb: glomerular layer	2.200	0.087
M	CD4	left Olfactory bulb: granule cell layer	2.275	0.102
M	CD4	left Olfactory bulb: internal plexiform layer	0.513	0.027
M	CD4	left Olfactory bulb: mitral cell layer	0.645	0.027
M	CD4	left olfactory peduncle	1.196	0.047
M	CD4	left olfactory tubercle	1.653	0.083
M	CD4	left optic tract	0.807	0.032
M	CD4	left paraflocculus (PFL)	1.751	0.196
M	CD4	left paraflocculus white matter	0.145	0.013
M	CD4	left paramedian lobule	0.072	0.007
M	CD4	left paramedian lobule (lobule 7)	2.000	0.097
M	CD4	left Parietal cortex: posterior area: rostral part	0.074	0.004
M	CD4	left Perirhinal cortex	1.276	0.068
M	CD4	left Piriform cortex	5.366	0.266
M	CD4	left PoDG	0.314	0.012
M	CD4	left pontine nucleus	0.384	0.059
M	CD4	left Posterolateral cortical amygdaloid area	0.430	0.029
M	CD4	left Posteromedial cortical amygdaloid area	0.615	0.037
M	CD4	left pre-para subiculum	1.183	0.047
M	CD4	left Primary auditory cortex	0.766	0.044
M	CD4	left Primary motor cortex	3.786	0.168
M	CD4	left Primary somatosensory cortex	2.080	0.070
M	CD4	left Primary somatosensory cortex: barrel field	4.893	0.258
M	CD4	left Primary somatosensory cortex: dysgranular zone	0.171	0.011
M	CD4	left Primary somatosensory cortex: forelimb region	2.144	0.144
M	CD4	left Primary somatosensory cortex: hindlimb region	1.513	0.095
M	CD4	left Primary somatosensory cortex: jaw region	0.277	0.030
M	CD4	left Primary somatosensory cortex: shoulder region	0.087	0.008
M	CD4	left Primary somatosensory cortex: trunk region	0.311	0.018
M	CD4	left Primary somatosensory cortex: upper lip region	2.436	0.132
M	CD4	left Primary visual cortex	1.186	0.079
M	CD4	left Primary visual cortex: binocular area	1.026	0.053
M	CD4	left Primary visual cortex: monocular area	1.199	0.079
M	CD4	left Rostral amygdalopiriform area	0.195	0.014
M	CD4	left Secondary auditory cortex: dorsal area	0.739	0.042
M	CD4	left Secondary auditory cortex: ventral area	0.819	0.070
M	CD4	left Secondary motor cortex	3.491	0.182
M	CD4	left Secondary somatosensory cortex	3.472	0.265
M	CD4	left Secondary visual cortex: lateral area	1.346	0.057
M	CD4	left Secondary visual cortex: mediolateral area	0.622	0.044
M	CD4	left Secondary visual cortex: mediomedial area	0.979	0.046
M	CD4	left simple lobule (lobule 6)	2.376	0.109
M	CD4	left simple lobule white matter	0.221	0.020
M	CD4	left SLu	0.390	0.026
M	CD4	left stria medullaris	0.337	0.011
M	CD4	left stria terminalis	0.499	0.014
M	CD4	left striatum	11.158	0.588
M	CD4	left subependymale zone / rhinocele	0.030	0.003
M	CD4	left subiculum	1.702	0.057
M	CD4	left superior olivary complex	0.372	0.044
M	CD4	left Temporal association area	1.409	0.083
M	CD4	left thalamus	9.135	0.369
M	CD4	left trunk of crus 2 and paramedian white matter	0.163	0.011
M	CD4	left trunk of simple and crus 1 white matter	0.068	0.005
M	CD4	left Ventral intermediate entorhinal cortex	0.575	0.031
M	CD4	left Ventral nucleus of the endopiriform claustrum	0.272	0.046
M	CD4	left Ventral orbital cortex	0.763	0.038
M	CD4	left Ventral tenia tecta	0.054	0.006
M	CD4	lobule 1-2 white matter	0.069	0.014
M	CD4	lobule 10 white matter	0.085	0.006
M	CD4	lobule 10: nodulus	1.413	0.065
M	CD4	lobule 3 white matter	0.197	0.020
M	CD4	lobule 3: central lobule (dorsal)	2.251	0.186
M	CD4	lobule 6: declive	2.401	0.076
M	CD4	lobule 7: tuber (or folium)	0.932	0.052
M	CD4	lobule 8 white matter	0.159	0.007
M	CD4	lobule 8: pyramis	1.617	0.063

M	CD4	lobule 9 white matter	0.289	0.012
M	CD4	lobule 9: uvula	2.779	0.177
M	CD4	lobules 1-2: lingula and central lobule (ventral)	1.666	0.195
M	CD4	lobules 4-5 white matter	0.577	0.028
M	CD4	lobules 4-5: culmen (ventral and dorsal)	4.384	0.253
M	CD4	lobules 6-7 white matter	0.669	0.028
M	CD4	medulla	27.583	0.899
M	CD4	midbrain	14.347	0.697
M	CD4	periaqueductal grey	4.114	0.203
M	CD4	pons	17.464	0.501
M	CD4	posterior commissure	0.149	0.008
M	CD4	right Accessory olfactory bulb: glomerular, external plexiform and mitral cell layer	0.251	0.010
M	CD4	right Accessory olfactory bulb: granule cell layer	0.120	0.007
M	CD4	right amygdala	4.800	0.220
M	CD4	right Amygdalopiriform transition area	0.510	0.031
M	CD4	right anterior commissure: pars anterior	0.492	0.020
M	CD4	right anterior commissure: pars posterior	0.211	0.011
M	CD4	right anterior lobule (lobules 4-5)	0.792	0.041
M	CD4	right anterior lobule white matter	0.051	0.003
M	CD4	right Anterior olfactory nucleus	1.008	0.050
M	CD4	right basal forebrain	2.825	0.107
M	CD4	right bed nucleus of stria terminalis	0.716	0.039
M	CD4	right CA10r	1.062	0.048
M	CD4	right CA1Py	0.582	0.022
M	CD4	right CA1Rad	1.388	0.049
M	CD4	right CA20r	0.215	0.007
M	CD4	right CA2Py	0.100	0.004
M	CD4	right CA2Rad	0.221	0.010
M	CD4	right CA30r	1.452	0.058
M	CD4	right CA3Py Inner	0.082	0.006
M	CD4	right CA3Py Outer	0.536	0.026
M	CD4	right CA3Rad	0.993	0.051
M	CD4	right Caudomedial entorhinal cortex	2.986	0.143
M	CD4	right cerebellar peduncle: inferior	0.395	0.014
M	CD4	right cerebellar peduncle: middle	0.608	0.032
M	CD4	right cerebellar peduncle: superior	0.524	0.031
M	CD4	right cerebral peduncle	1.057	0.035
M	CD4	right Cingulate cortex: area 24a	1.030	0.046
M	CD4	right Cingulate cortex: area 24a'	0.369	0.027
M	CD4	right Cingulate cortex: area 24b	0.818	0.094
M	CD4	right Cingulate cortex: area 24b'	0.318	0.020
M	CD4	right Cingulate cortex: area 25	0.305	0.017
M	CD4	right Cingulate cortex: area 29a	0.352	0.028
M	CD4	right Cingulate cortex: area 29b	0.195	0.015
M	CD4	right Cingulate cortex: area 29c	0.922	0.054
M	CD4	right Cingulate cortex: area 30	1.338	0.058
M	CD4	right Cingulate cortex: area 32	1.110	0.088
M	CD4	right Cingulum	0.468	0.023
M	CD4	right Claustrum	0.150	0.010
M	CD4	right Claustrum: dorsal part	0.151	0.026
M	CD4	right Claustrum: ventral part	0.331	0.045
M	CD4	right colliculus: inferior	2.799	0.091
M	CD4	right colliculus: superior	4.602	0.229
M	CD4	right copula white matter	0.036	0.002
M	CD4	right copula: pyramis (lobule 8)	1.273	0.067
M	CD4	right corpus callosum	5.999	0.161
M	CD4	right Cortex-amygdala transition zones	0.428	0.030
M	CD4	right corticospinal tract/pyramids	0.898	0.035
M	CD4	right crus 1 white matter	0.187	0.007
M	CD4	right crus 1: ansiform lobule (lobule 6)	2.209	0.083
M	CD4	right crus 2 white matter	0.143	0.011
M	CD4	right crus 2: ansiform lobule (lobule 7)	1.946	0.109
M	CD4	right cuneate nucleus	0.123	0.010
M	CD4	right dentate nucleus	0.184	0.013
M	CD4	right Dorsal intermediate entorhinal cortex	0.935	0.060
M	CD4	right Dorsal nucleus of the endopiriform	0.760	0.048
M	CD4	right Dorsal tenia tecta	0.443	0.024
M	CD4	right Dorsolateral entorhinal cortex	1.457	0.081
M	CD4	right Dorsolateral orbital cortex	0.396	0.024
M	CD4	right Ectorhinal cortex	1.390	0.100
M	CD4	right facial nerve (cranial nerve 7)	0.110	0.006
M	CD4	right fasciculus retroflexus	0.119	0.008
M	CD4	right fastigial nucleus	0.250	0.013
M	CD4	right fimbria	1.604	0.049
M	CD4	right flocculus (FL)	0.519	0.023
M	CD4	right flocculus white matter	0.038	0.004
M	CD4	right fornix	0.308	0.016
M	CD4	right Frontal association cortex	3.991	0.174
M	CD4	right Frontal cortex: area 3	0.372	0.020
M	CD4	right fundus of striatum	0.087	0.007
M	CD4	right globus pallidus	1.734	0.091
M	CD4	right GrDG	0.677	0.030
M	CD4	right habenular commissure	0.014	0.004
M	CD4	right hypothalamus	5.447	0.172
M	CD4	right inferior olivary complex	0.175	0.025
M	CD4	right Insular region: not subdivided	4.213	0.316
M	CD4	right Intermediate nucleus of the endopiriform claustrum	0.301	0.021
M	CD4	right internal capsule	1.570	0.062
M	CD4	right lateral olfactory tract	0.662	0.038
M	CD4	right Lateral orbital cortex	1.789	0.094
M	CD4	right Lateral parietal association cortex	0.092	0.009
M	CD4	right lateral septum	1.597	0.098
M	CD4	right lateral ventricle	2.129	0.287
M	CD4	right LMol	1.206	0.034
M	CD4	right mammillary bodies	0.217	0.016
M	CD4	right mammilothalamic tract	0.133	0.008
M	CD4	right Medial amygdala	0.593	0.029
M	CD4	right Medial entorhinal cortex	0.353	0.015
M	CD4	right medial lemniscus/medial longitudinal fasciculus	1.354	0.045
M	CD4	right Medial orbital cortex	0.844	0.054
M	CD4	right Medial parietal association cortex	0.172	0.013
M	CD4	right Medial preoptic nucleus	0.096	0.005
M	CD4	right medial septum	0.630	0.027
M	CD4	right MoDG	2.123	0.077
M	CD4	right nucleus accumbens	2.198	0.112
M	CD4	right nucleus interpositus	0.249	0.016
M	CD4	right Olfactory bulb: external plexiform layer	3.216	0.113
M	CD4	right Olfactory bulb: glomerular layer	2.277	0.090
M	CD4	right Olfactory bulb: granule cell layer	2.337	0.102
M	CD4	right Olfactory bulb: internal plexiform layer	0.499	0.022
M	CD4	right Olfactory bulb: mitral cell layer	0.607	0.018
M	CD4	right olfactory peduncle	1.154	0.040
M	CD4	right olfactory tubercle	1.663	0.075
M	CD4	right optic tract	0.751	0.031
M	CD4	right paraflocculus (PFL)	1.782	0.169
M	CD4	right paraflocculus white matter	0.168	0.016
M	CD4	right paramedian lobule	0.077	0.007
M	CD4	right paramedian lobule (lobule 7)	1.967	0.106
M	CD4	right Parietal cortex: posterior area: rostral part	0.045	0.004
M	CD4	right Perirhinal cortex	1.227	0.091
M	CD4	right Piriform cortex	5.676	0.300
M	CD4	right PoDG	0.334	0.016
M	CD4	right pontine nucleus	0.372	0.048
M	CD4	right Posterolateral cortical amygdaloid area	0.483	0.031
M	CD4	right Posteromedial cortical amygdaloid area	0.480	0.022
M	CD4	right pre-para subiculum	1.182	0.050
M	CD4	right Primary auditory cortex	0.752	0.044
M	CD4	right Primary motor cortex	3.305	0.126
M	CD4	right Primary somatosensory cortex	2.110	0.085
M	CD4	right Primary somatosensory cortex: barrel field	4.808	0.174
M	CD4	right Primary somatosensory cortex: dysgranular zone	0.180	0.015
M	CD4	right Primary somatosensory cortex: forelimb region	1.932	0.099
M	CD4	right Primary somatosensory cortex: hindlimb region	1.236	0.067
M	CD4	right Primary somatosensory cortex: jaw region	0.285	0.030
M	CD4	right Primary somatosensory cortex: shoulder region	0.063	0.010
M	CD4	right Primary somatosensory cortex: trunk region	0.199	0.020
M	CD4	right Primary somatosensory cortex: upper lip region	2.835	0.123
M	CD4	right Primary visual cortex	1.191	0.075
M	CD4	right Primary visual cortex: binocular area	1.030	0.070
M	CD4	right Primary visual cortex: monocular area	0.794	0.043
M	CD4	right Rostral amygdalopiriform area	0.211	0.020
M	CD4	right Secondary auditory cortex: dorsal area	0.705	0.030
M	CD4	right Secondary auditory cortex: ventral area	1.016	0.092
M	CD4	right Secondary motor cortex	3.381	0.185
M	CD4	right Secondary somatosensory cortex	3.637	0.278
M	CD4	right Secondary visual cortex: lateral area	1.452	0.080
M	CD4	right Secondary visual cortex: mediolateral area	0.414	0.029
M	CD4	right Secondary visual cortex: mediomedial area	0.808	0.036
M	CD4	right simple lobule (lobule 6)	2.414	0.098
M	CD4	right simple lobule white matter	0.181	0.006
M	CD4	right SLu	0.384	0.026
M	CD4	right stria medullaris	0.358	0.010
M	CD4	right stria terminalis	0.420	0.014
M	CD4	right striatum	11.072	0.594
M	CD4	right subependymale zone / rhinocele	0.026	0.002
M	CD4	right subiculum	1.667	0.055
M	CD4	right superior olivary complex	0.395	0.048
M	CD4	right Temporal association area	1.597	0.126
M	CD4	right thalamus	9.070	0.415
M	CD4	right trunk of crus 2 and paramedian white matter	0.173	0.010
M	CD4	right trunk of simple and crus 1 white matter	0.081	0.005
M	CD4	right Ventral intermediate entorhinal cortex	0.561	0.029
M	CD4	right Ventral nucleus of the endopiriform claustrum	0.245	0.026
M	CD4	right Ventral orbital cortex	0.700	0.042
M	CD4	right Ventral tenia tecta	0.054	0.005
M	CD4	third ventricle	1.305	0.107
M	CD4	trunk of arbor vita	4.275	0.116
M	CD4	trunk of lobules 1-3 white matter	0.129	0.010
M	CD4	trunk of lobules 6-8 white matter	0.106	0.007
M	CD4	ventral tegmental decussation	0.135	0.010
M	CD8	cerebral aqueduct	0.474	0.042
M	CD8	fourth ventricle	0.898	0.070
M	CD8	interpeduncular nucleus	0.267	0.017
M	CD8	left Accessory olfactory bulb: glomerular, external plexiform and mitral cell layer	0.242	0.013
M	CD8	left Accessory olfactory bulb: granule cell layer	0.121	0.007
M	CD8	left amygdala	4.738	0.187
M	CD8	left Amygdalopiriform transition area	0.509	0.020
M	CD8	left anterior commissure: pars anterior	0.482	0.027
M	CD8	left anterior commissure: pars posterior	0.206	0.016
M	CD8	left anterior lobule (lobules 4-5)	0.818	0.058
M	CD8	left anterior lobule white matter	0.040	0.004
M	CD8	left Anterior olfactory nucleus	0.949	0.050
M	CD8	left basal forebrain	2.505	0.116
M	CD8	left bed nucleus of stria terminalis	0.675	0.029
M	CD8	left CA10r	1.178	0.062
M	CD8	left CA1Py	0.561	0.027
M	CD8	left CA1Rad	1.249	0.052
M	CD8	left CA20r	0.256	0.018
M	CD8	left CA2Py	0.100	0.006
M	CD8	left CA2Rad	0.203	0.012
M	CD8	left CA30r	1.355	0.074
M	CD8	left CA3Py Inner	0.075	0.006
M	CD8	left CA3Py Outer	0.501	0.030
M	CD8	left CA3Rad	0.893	0.050
M	CD8	left Caudomedial entorhinal cortex	2.858	0.108
M	CD8	left cerebellar peduncle: inferior	0.405	0.020
M	CD8	left cerebellar peduncle: middle	0.549	0.040
M	CD8	left cerebellar peduncle: superior	0.492	0.026
M	CD8	left cerebral peduncle	0.981	0.043
M	CD8	left Cingulate cortex: area 24a	0.902	0.038
M	CD8	left Cingulate cortex: area 24a'	0.351	0.023
M	CD8	left Cingulate cortex: area 24b	0.791	0.084
M	CD8	left Cingulate cortex: area 24b'	0.290	0.023
M	CD8	left Cingulate cortex: area 25	0.285	0.012
M	CD8	left Cingulate cortex: area 29a	0.362	0.019
M	CD8	left Cingulate cortex: area 29b	0.207	0.020
M	CD8	left Cingulate cortex: area 29c	0.923	0.069
M	CD8	left Cingulate cortex: area 30	1.441	0.067
M	CD8	left Cingulate cortex: area 32	1.312	0.108
M	CD8	left Cingulum	0.421	0.022
M	CD8	left Claustrum	0.169	0.011
M	CD8	left Claustrum: dorsal part	0.147	0.022
M	CD8	left Claustrum: ventral part	0.265	0.033
M	CD8	left colliculus: inferior	2.710	0.124
M	CD8	left colliculus: superior	4.137	0.240
M	CD8	left copula white matter	0.043	0.003
M	CD8	left copula: pyramis (lobule 8)	1.091	0.079
M	CD8	left corpus callosum	5.916	0.300
M	CD8	left Cortex-amygdala transition zones	0.387	0.029
M	CD8	left corticospinal tract/pyramids	0.846	0.052
M	CD8	left crus 1 white matter	0.181	0.016
M	CD8	left crus 1: ansiform lobule (lobule 6)	2.106	0.135
M	CD8	left crus 2 white matter	0.120	0.012
M	CD8	left crus 2: ansiform lobule (lobule 7)	1.862	0.128
M	CD8	left cuneate nucleus	0.116	0.010
M	CD8	left dentate nucleus	0.173	0.009
M	CD8	left Dorsal intermediate entorhinal cortex	0.836	0.057
M	CD8	left Dorsal nucleus of the endopiriform	0.705	0.033
M	CD8	left Dorsal tenia tecta	0.462	0.032
M	CD8	left Dorsolateral entorhinal cortex	1.159	0.068
M	CD8	left Dorsolateral orbital cortex	0.421	0.019
M	CD8	left Ectorhinal cortex	1.299	0.093
M	CD8	left facial nerve (cranial nerve 7)	0.101	0.007
M	CD8	left fasciculus retroflexus	0.125	0.008
M	CD8	left fastigial nucleus	0.235	0.010
M	CD8	left fimbria	1.598	0.062
M	CD8	left flocculus (FL)	0.531	0.039
M	CD8	left flocculus white matter	0.029	0.003
M	CD8	left fornix	0.330	0.014
M	CD8	left Frontal association cortex	3.445	0.116
M	CD8	left Frontal cortex: area 3	0.355	0.015
M	CD8	left fundus of striatum	0.071	0.005
M	CD8	left globus pallidus	1.556	0.084
M	CD8	left GrDG	0.665	0.038
M	CD8	left habenular commissure	0.013	0.002
M	CD8	left hypothalamus	5.057	0.197
M	CD8	left inferior olivary complex	0.154	0.019
M	CD8	left Insular region: not subdivided	3.760	0.254
M	CD8	left Intermediate nucleus of the endopiriform claustrum	0.302	0.017
M	CD8	left internal capsule	1.394	0.065
M	CD8	left lateral olfactory tract	0.725	0.026
M	CD8	left Lateral orbital cortex	1.732	0.072
M	CD8	left Lateral parietal association cortex	0.148	0.009
M	CD8	left lateral septum	1.458	0.059
M	CD8	left lateral ventricle	1.662	0.131
M	CD8	left LMol	1.179	0.049
M	CD8	left mammillary bodies	0.230	0.028
M	CD8	left mammilothalamic tract	0.120	0.007
M	CD8	left Medial amygdala	0.523	0.021
M	CD8	left Medial entorhinal cortex	0.356	0.015
M	CD8	left medial lemniscus/medial longitudinal fasciculus	1.209	0.070
M	CD8	left Medial orbital cortex	0.876	0.049
M	CD8	left Medial parietal association cortex	0.239	0.027
M	CD8	left Medial preoptic nucleus	0.098	0.007
M	CD8	left medial septum	0.626	0.029
M	CD8	left MoDG	2.137	0.108
M	CD8	left nucleus accumbens	2.041	0.084

M	CD8	left nucleus interpositus	0.221	0.012
M	CD8	left Olfactory bulb: external plexiform layer	3.182	0.111
M	CD8	left Olfactory bulb: glomerular layer	2.181	0.092
M	CD8	left Olfactory bulb: granule cell layer	2.249	0.125
M	CD8	left Olfactory bulb: internal plexiform layer	0.508	0.029
M	CD8	left Olfactory bulb: mitral cell layer	0.637	0.029
M	CD8	left olfactory peduncle	1.138	0.049
M	CD8	left olfactory tubercle	1.600	0.074
M	CD8	left optic tract	0.758	0.030
M	CD8	left paraflocculus (PFL)	1.705	0.234
M	CD8	left paraflocculus white matter	0.140	0.016
M	CD8	left paramedian lobule	0.069	0.006
M	CD8	left paramedian lobule (lobule 7)	1.943	0.090
M	CD8	left Parietal cortex: posterior area: rostral part	0.068	0.006
M	CD8	left Perirhinal cortex	1.213	0.073
M	CD8	left Piriform cortex	5.051	0.205
M	CD8	left PoDG	0.298	0.018
M	CD8	left pontine nucleus	0.360	0.049
M	CD8	left Posterolateral cortical amygdaloid area	0.410	0.033
M	CD8	left Posteromedial cortical amygdaloid area	0.579	0.021
M	CD8	left pre-para subiculum	1.104	0.081
M	CD8	left Primary auditory cortex	0.695	0.045
M	CD8	left Primary motor cortex	3.558	0.142
M	CD8	left Primary somatosensory cortex	1.969	0.087
M	CD8	left Primary somatosensory cortex: barrel field	4.489	0.152
M	CD8	left Primary somatosensory cortex: dysgranular zone	0.164	0.012
M	CD8	left Primary somatosensory cortex: forelimb region	1.953	0.125
M	CD8	left Primary somatosensory cortex: hindlimb region	1.421	0.095
M	CD8	left Primary somatosensory cortex: jaw region	0.256	0.025
M	CD8	left Primary somatosensory cortex: shoulder region	0.088	0.006
M	CD8	left Primary somatosensory cortex: trunk region	0.296	0.014
M	CD8	left Primary somatosensory cortex: upper lip region	2.306	0.088
M	CD8	left Primary visual cortex	1.173	0.095
M	CD8	left Primary visual cortex: binocular area	0.991	0.057
M	CD8	left Primary visual cortex: monocular area	1.105	0.067
M	CD8	left Rostral amygdalopiriform area	0.195	0.014
M	CD8	left Secondary auditory cortex: dorsal area	0.687	0.049
M	CD8	left Secondary auditory cortex: ventral area	0.762	0.073
M	CD8	left Secondary motor cortex	3.326	0.136
M	CD8	left Secondary somatosensory cortex	3.341	0.251
M	CD8	left Secondary visual cortex: lateral area	1.281	0.055
M	CD8	left Secondary visual cortex: mediolateral area	0.560	0.035
M	CD8	left Secondary visual cortex: mediomedial area	0.917	0.042
M	CD8	left simple lobule (lobule 6)	2.263	0.122
M	CD8	left simple lobule white matter	0.205	0.017
M	CD8	left SLu	0.369	0.022
M	CD8	left stria medullaris	0.313	0.016
M	CD8	left stria terminalis	0.456	0.013
M	CD8	left striatum	10.238	0.462
M	CD8	left subependymale zone / rhinocele	0.028	0.002
M	CD8	left subiculum	1.639	0.066
M	CD8	left superior olivary complex	0.368	0.032
M	CD8	left Temporal association area	1.360	0.103
M	CD8	left thalamus	8.629	0.344
M	CD8	left trunk of crus 2 and paramedian white matter	0.154	0.011
M	CD8	left trunk of simple and crus 1 white matter	0.064	0.005
M	CD8	left Ventral intermediate entorhinal cortex	0.563	0.029
M	CD8	left Ventral nucleus of the endopiriform claustrum	0.247	0.031
M	CD8	left Ventral orbital cortex	0.749	0.039
M	CD8	left Ventral tenia tecta	0.056	0.004
M	CD8	lobule 1-2 white matter	0.062	0.014
M	CD8	lobule 10 white matter	0.083	0.005
M	CD8	lobule 10: nodulus	1.361	0.065
M	CD8	lobule 3 white matter	0.176	0.021
M	CD8	lobule 3: central lobule (dorsal)	2.077	0.159
M	CD8	lobule 6: declive	2.315	0.113
M	CD8	lobule 7: tuber (or folium)	0.893	0.077
M	CD8	lobule 8 white matter	0.149	0.012
M	CD8	lobule 8: pyramis	1.522	0.089
M	CD8	lobule 9 white matter	0.273	0.017
M	CD8	lobule 9: uvula	2.665	0.137
M	CD8	lobules 1-2: lingula and central lobule (ventral)	1.560	0.192
M	CD8	lobules 4-5 white matter	0.563	0.032
M	CD8	lobules 4-5: culmen (ventral and dorsal)	4.272	0.208
M	CD8	lobules 6-7 white matter	0.647	0.032
M	CD8	medulla	26.220	1.215
M	CD8	midbrain	13.041	0.654
M	CD8	periaqueductal grey	3.837	0.165
M	CD8	pons	16.280	0.743
M	CD8	posterior commissure	0.136	0.009
M	CD8	right Accessory olfactory bulb: glomerular, external plexiform and mitral cell layer	0.234	0.006
M	CD8	right Accessory olfactory bulb: granule cell layer	0.116	0.007
M	CD8	right amygdala	4.412	0.139
M	CD8	right Amygdalopiriform transition area	0.484	0.028
M	CD8	right anterior commissure: pars anterior	0.470	0.020
M	CD8	right anterior commissure: pars posterior	0.193	0.011
M	CD8	right anterior lobule (lobules 4-5)	0.764	0.054
M	CD8	right anterior lobule white matter	0.051	0.006
M	CD8	right Anterior olfactory nucleus	0.974	0.060
M	CD8	right basal forebrain	2.604	0.098
M	CD8	right bed nucleus of stria terminalis	0.669	0.025
M	CD8	right CA10r	1.034	0.042
M	CD8	right CA1Py	0.567	0.021
M	CD8	right CA1Rad	1.352	0.051
M	CD8	right CA20r	0.211	0.018
M	CD8	right CA2Py	0.095	0.005
M	CD8	right CA2Rad	0.213	0.010
M	CD8	right CA30r	1.352	0.068
M	CD8	right CA3Py Inner	0.077	0.004
M	CD8	right CA3Py Outer	0.499	0.024
M	CD8	right CA3Rad	0.927	0.043
M	CD8	right Caudomedial entorhinal cortex	2.783	0.100
M	CD8	right cerebellar peduncle: inferior	0.366	0.018
M	CD8	right cerebellar peduncle: middle	0.572	0.040
M	CD8	right cerebellar peduncle: superior	0.477	0.025
M	CD8	right cerebral peduncle	0.985	0.051
M	CD8	right Cingulate cortex: area 24a	0.966	0.041
M	CD8	right Cingulate cortex: area 24a'	0.332	0.023
M	CD8	right Cingulate cortex: area 24b	0.762	0.066
M	CD8	right Cingulate cortex: area 24b'	0.281	0.019
M	CD8	right Cingulate cortex: area 25	0.295	0.014
M	CD8	right Cingulate cortex: area 29a	0.339	0.022
M	CD8	right Cingulate cortex: area 29b	0.198	0.020
M	CD8	right Cingulate cortex: area 29c	0.865	0.035
M	CD8	right Cingulate cortex: area 30	1.347	0.059
M	CD8	right Cingulate cortex: area 32	1.062	0.061
M	CD8	right Cingulum	0.427	0.021
M	CD8	right Claustrum	0.141	0.012
M	CD8	right Claustrum: dorsal part	0.142	0.015
M	CD8	right Claustrum: ventral part	0.309	0.034
M	CD8	right colliculus: inferior	2.604	0.113
M	CD8	right colliculus: superior	4.253	0.198
M	CD8	right copula white matter	0.033	0.003
M	CD8	right copula: pyramis (lobule 8)	1.177	0.101
M	CD8	right corpus callosum	5.632	0.200
M	CD8	right Cortex-amygdala transition zones	0.421	0.030
M	CD8	right corticospinal tract/pyramids	0.886	0.035
M	CD8	right crus 1 white matter	0.172	0.009
M	CD8	right crus 1: ansiform lobule (lobule 6)	2.053	0.090
M	CD8	right crus 2 white matter	0.135	0.009
M	CD8	right crus 2: ansiform lobule (lobule 7)	1.859	0.114
M	CD8	right cuneate nucleus	0.121	0.011
M	CD8	right dentate nucleus	0.170	0.010
M	CD8	right Dorsal intermediate entorhinal cortex	0.849	0.038
M	CD8	right Dorsal nucleus of the endopiriform	0.683	0.029
M	CD8	right Dorsal tenia tecta	0.429	0.027
M	CD8	right Dorsolateral entorhinal cortex	1.286	0.073
M	CD8	right Dorsolateral orbital cortex	0.391	0.021
M	CD8	right Ectorhinal cortex	1.376	0.077
M	CD8	right facial nerve (cranial nerve 7)	0.106	0.008
M	CD8	right fasciculus retroflexus	0.112	0.008
M	CD8	right fastigial nucleus	0.225	0.013
M	CD8	right fimbria	1.473	0.068
M	CD8	right flocculus (FL)	0.495	0.034
M	CD8	right flocculus white matter	0.037	0.004
M	CD8	right fornix	0.292	0.016
M	CD8	right Frontal association cortex	3.741	0.135
M	CD8	right Frontal cortex: area 3	0.357	0.018
M	CD8	right fundus of striatum	0.078	0.005
M	CD8	right globus pallidus	1.587	0.070
M	CD8	right GrDG	0.627	0.032
M	CD8	right habenular commissure	0.013	0.002
M	CD8	right hypothalamus	5.040	0.198
M	CD8	right inferior olivary complex	0.175	0.030
M	CD8	right Insular region: not subdivided	4.025	0.193
M	CD8	right Intermediate nucleus of the endopiriform claustrum	0.274	0.011
M	CD8	right internal capsule	1.476	0.052
M	CD8	right lateral olfactory tract	0.629	0.022
M	CD8	right Lateral orbital cortex	1.698	0.051
M	CD8	right Lateral parietal association cortex	0.089	0.008
M	CD8	right lateral septum	1.460	0.069
M	CD8	right lateral ventricle	1.723	0.143
M	CD8	right LMol	1.160	0.039
M	CD8	right mammillary bodies	0.210	0.020
M	CD8	right mammilothalamic tract	0.126	0.009
M	CD8	right Medial amygdala	0.558	0.017
M	CD8	right Medial entorhinal cortex	0.335	0.017
M	CD8	right medial lemniscus/medial longitudinal fasciculus	1.289	0.050
M	CD8	right Medial orbital cortex	0.786	0.036
M	CD8	right Medial parietal association cortex	0.172	0.011
M	CD8	right Medial preoptic nucleus	0.087	0.004
M	CD8	right medial septum	0.590	0.035
M	CD8	right MoDG	2.004	0.090
M	CD8	right nucleus accumbens	1.997	0.064
M	CD8	right nucleus interpositus	0.230	0.015
M	CD8	right Olfactory bulb: external plexiform layer	3.165	0.092
M	CD8	right Olfactory bulb: glomerular layer	2.226	0.103
M	CD8	right Olfactory bulb: granule cell layer	2.267	0.110
M	CD8	right Olfactory bulb: internal plexiform layer	0.493	0.029
M	CD8	right Olfactory bulb: mitral cell layer	0.592	0.028
M	CD8	right olfactory peduncle	1.100	0.047
M	CD8	right olfactory tubercle	1.593	0.110
M	CD8	right optic tract	0.711	0.028
M	CD8	right paraflocculus (PFL)	1.710	0.216
M	CD8	right paraflocculus white matter	0.162	0.020
M	CD8	right paramedian lobule	0.076	0.006
M	CD8	right paramedian lobule (lobule 7)	1.915	0.132
M	CD8	right Parietal cortex: posterior area: rostral part	0.044	0.005
M	CD8	right Perirhinal cortex	1.182	0.077
M	CD8	right Piriform cortex	5.240	0.182
M	CD8	right PoDG	0.312	0.018
M	CD8	right pontine nucleus	0.348	0.037
M	CD8	right Posterolateral cortical amygdaloid area	0.442	0.029
M	CD8	right Posteromedial cortical amygdaloid area	0.461	0.036
M	CD8	right pre-para subiculum	1.133	0.061
M	CD8	right Primary auditory cortex	0.718	0.049
M	CD8	right Primary motor cortex	3.019	0.112
M	CD8	right Primary somatosensory cortex	2.065	0.073
M	CD8	right Primary somatosensory cortex: barrel field	4.377	0.152
M	CD8	right Primary somatosensory cortex: dysgranular zone	0.174	0.009
M	CD8	right Primary somatosensory cortex: forelimb region	1.721	0.075
M	CD8	right Primary somatosensory cortex: hindlimb region	1.151	0.051
M	CD8	right Primary somatosensory cortex: jaw region	0.274	0.017
M	CD8	right Primary somatosensory cortex: shoulder region	0.065	0.006
M	CD8	right Primary somatosensory cortex: trunk region	0.197	0.013
M	CD8	right Primary somatosensory cortex: upper lip region	2.694	0.130
M	CD8	right Primary visual cortex	1.198	0.069
M	CD8	right Primary visual cortex: binocular area	0.952	0.041
M	CD8	right Primary visual cortex: monocular area	0.732	0.036
M	CD8	right Rostral amygdalopiriform area	0.201	0.015
M	CD8	right Secondary auditory cortex: dorsal area	0.688	0.032
M	CD8	right Secondary auditory cortex: ventral area	0.965	0.056
M	CD8	right Secondary motor cortex	3.146	0.141
M	CD8	right Secondary somatosensory cortex	3.582	0.188
M	CD8	right Secondary visual cortex: lateral area	1.436	0.067
M	CD8	right Secondary visual cortex: mediolateral area	0.379	0.031
M	CD8	right Secondary visual cortex: mediomedial area	0.768	0.038
M	CD8	right simple lobule (lobule 6)	2.248	0.119
M	CD8	right simple lobule white matter	0.169	0.011
M	CD8	right SLu	0.365	0.018
M	CD8	right stria medullaris	0.332	0.017
M	CD8	right stria terminalis	0.384	0.016
M	CD8	right striatum	10.166	0.360
M	CD8	right subependymale zone / rhinocele	0.025	0.002
M	CD8	right subiculum	1.622	0.061
M	CD8	right superior olivary complex	0.383	0.050
M	CD8	right Temporal association area	1.549	0.117
M	CD8	right thalamus	8.581	0.347
M	CD8	right trunk of crus 2 and paramedian white matter	0.165	0.017
M	CD8	right trunk of simple and crus 1 white matter	0.080	0.007
M	CD8	right Ventral intermediate entorhinal cortex	0.543	0.024
M	CD8	right Ventral nucleus of the endopiriform claustrum	0.225	0.020
M	CD8	right Ventral orbital cortex	0.666	0.029
M	CD8	right Ventral tenia tecta	0.056	0.004
M	CD8	third ventricle	1.162	0.112
M	CD8	trunk of arbor vita	3.980	0.162
M	CD8	trunk of lobules 1-3 white matter	0.120	0.010
M	CD8	trunk of lobules 6-8 white matter	0.105	0.009
M	CD8	ventral tegmental decussation	0.122	0.010
M	Cxcr2	cerebral aqueduct	0.506	0.061
M	Cxcr2	fourth ventricle	0.932	0.056
M	Cxcr2	interpeduncular nucleus	0.280	0.017
M	Cxcr2	left Accessory olfactory bulb: glomerular, external plexiform and mitral cell layer	0.256	0.014
M	Cxcr2	left Accessory olfactory bulb: granule cell layer	0.123	0.006
M	Cxcr2	left amygdala	4.875	0.139
M	Cxcr2	left Amygdalopiriform transition area	0.532	0.038
M	Cxcr2	left anterior commissure: pars anterior	0.485	0.022
M	Cxcr2	left anterior commissure: pars posterior	0.209	0.012
M	Cxcr2	left anterior lobule (lobules 4-5)	0.837	0.034
M	Cxcr2	left anterior lobule white matter	0.041	0.002
M	Cxcr2	left Anterior olfactory nucleus	0.960	0.043
M	Cxcr2	left basal forebrain	2.633	0.069
M	Cxcr2	left bed nucleus of stria terminalis	0.686	0.030
M	Cxcr2	left CA10r	1.148	0.064
M	Cxcr2	left CA1Py	0.552	0.027
M	Cxcr2	left CA1Rad	1.243	0.060
M	Cxcr2	left CA20r	0.262	0.018
M	Cxcr2	left CA2Py	0.103	0.006
M	Cxcr2	left CA2Rad	0.213	0.012
M	Cxcr2	left CA30r	1.370	0.048
M	Cxcr2	left CA3Py Inner	0.078	0.005
M	Cxcr2	left CA3Py Outer	0.505	0.018
M	Cxcr2	left CA3Rad	0.912	0.041
M	Cxcr2	left Caudomedial entorhinal cortex	2.914	0.085
M	Cxcr2	left cerebellar peduncle: inferior	0.414	0.015
M	Cxcr2	left cerebellar peduncle: middle	0.581	0.037
M	Cxcr2	left cerebellar peduncle: superior	0.512	0.027

M	Cxcr2	left cerebral peduncle	1.022	0.041
M	Cxcr2	left Cingulate cortex: area 24a	0.915	0.071
M	Cxcr2	left Cingulate cortex: area 24a'	0.364	0.035
M	Cxcr2	left Cingulate cortex: area 24b	0.798	0.088
M	Cxcr2	left Cingulate cortex: area 24b'	0.300	0.024
M	Cxcr2	left Cingulate cortex: area 25	0.292	0.012
M	Cxcr2	left Cingulate cortex: area 29a	0.385	0.026
M	Cxcr2	left Cingulate cortex: area 29b	0.216	0.014
M	Cxcr2	left Cingulate cortex: area 29c	0.977	0.056
M	Cxcr2	left Cingulate cortex: area 30	1.453	0.070
M	Cxcr2	left Cingulate cortex: area 32	1.290	0.097
M	Cxcr2	left Cingulum	0.432	0.031
M	Cxcr2	left Claustrum	0.171	0.010
M	Cxcr2	left Claustrum: dorsal part	0.143	0.019
M	Cxcr2	left Claustrum: ventral part	0.273	0.026
M	Cxcr2	left colliculus: inferior	2.773	0.060
M	Cxcr2	left colliculus: superior	4.354	0.169
M	Cxcr2	left copula white matter	0.042	0.003
M	Cxcr2	left copula: pyramis (lobule 8)	1.126	0.072
M	Cxcr2	left corpus callosum	6.048	0.278
M	Cxcr2	left Cortex-amygdala transition zones	0.410	0.029
M	Cxcr2	left corticospinal tract/pyramids	0.868	0.046
M	Cxcr2	left crus 1 white matter	0.185	0.006
M	Cxcr2	left crus 1: ansiform lobule (lobule 6)	2.093	0.068
M	Cxcr2	left crus 2 white matter	0.130	0.012
M	Cxcr2	left crus 2: ansiform lobule (lobule 7)	2.004	0.101
M	Cxcr2	left cuneate nucleus	0.116	0.008
M	Cxcr2	left dentate nucleus	0.183	0.009
M	Cxcr2	left Dorsal intermediate entorhinal cortex	0.850	0.042
M	Cxcr2	left Dorsal nucleus of the endopiriform	0.719	0.027
M	Cxcr2	left Dorsal tenia tecta	0.492	0.033
M	Cxcr2	left Dorsolateral entorhinal cortex	1.164	0.040
M	Cxcr2	left Dorsolateral orbital cortex	0.438	0.025
M	Cxcr2	left Ectorhinal cortex	1.295	0.107
M	Cxcr2	left facial nerve (cranial nerve 7)	0.105	0.005
M	Cxcr2	left fasciculus retroflexus	0.129	0.007
M	Cxcr2	left fastigial nucleus	0.243	0.012
M	Cxcr2	left fimbria	1.617	0.080
M	Cxcr2	left flocculus (FL)	0.553	0.038
M	Cxcr2	left flocculus white matter	0.029	0.003
M	Cxcr2	left fornix	0.335	0.016
M	Cxcr2	left Frontal association cortex	3.380	0.137
M	Cxcr2	left Frontal cortex: area 3	0.360	0.031
M	Cxcr2	left fundus of striatum	0.074	0.004
M	Cxcr2	left globus pallidus	1.621	0.076
M	Cxcr2	left GrDG	0.671	0.034
M	Cxcr2	left habenular commissure	0.014	0.002
M	Cxcr2	left hypothalamus	5.337	0.135
M	Cxcr2	left inferior olivary complex	0.162	0.021
M	Cxcr2	left Insular region: not subdivided	3.730	0.218
M	Cxcr2	left Intermediate nucleus of the endopiriform claustrum	0.312	0.016
M	Cxcr2	left internal capsule	1.411	0.055
M	Cxcr2	left lateral olfactory tract	0.727	0.042
M	Cxcr2	left Lateral orbital cortex	1.752	0.065
M	Cxcr2	left Lateral parietal association cortex	0.143	0.013
M	Cxcr2	left lateral septum	1.494	0.060
M	Cxcr2	left lateral ventricle	1.667	0.117
M	Cxcr2	left LMol	1.191	0.042
M	Cxcr2	left mammillary bodies	0.243	0.020
M	Cxcr2	left mammilothalamic tract	0.121	0.005
M	Cxcr2	left Medial amygdala	0.557	0.040
M	Cxcr2	left Medial entorhinal cortex	0.348	0.020
M	Cxcr2	left medial lemniscus/medial longitudinal fasciculus	1.249	0.046
M	Cxcr2	left Medial orbital cortex	0.904	0.052
M	Cxcr2	left Medial parietal association cortex	0.234	0.027
M	Cxcr2	left Medial preoptic nucleus	0.104	0.007
M	Cxcr2	left medial septum	0.664	0.026
M	Cxcr2	left MoDG	2.160	0.079
M	Cxcr2	left nucleus accumbens	2.040	0.063
M	Cxcr2	left nucleus interpositus	0.227	0.012
M	Cxcr2	left Olfactory bulb: external plexiform layer	3.204	0.134
M	Cxcr2	left Olfactory bulb: glomerular layer	2.176	0.087
M	Cxcr2	left Olfactory bulb: granule cell layer	2.190	0.107
M	Cxcr2	left Olfactory bulb: internal plexiform layer	0.502	0.024
M	Cxcr2	left Olfactory bulb: mitral cell layer	0.634	0.026
M	Cxcr2	left olfactory peduncle	1.153	0.042
M	Cxcr2	left olfactory tubercle	1.632	0.085
M	Cxcr2	left optic tract	0.772	0.023
M	Cxcr2	left paraflocculus (PFL)	1.708	0.232
M	Cxcr2	left paraflocculus white matter	0.144	0.016
M	Cxcr2	left paramedian lobule	0.072	0.005
M	Cxcr2	left paramedian lobule (lobule 7)	2.024	0.111
M	Cxcr2	left Parietal cortex: posterior area: rostral part	0.067	0.006
M	Cxcr2	left Perirhinal cortex	1.222	0.069
M	Cxcr2	left Piriform cortex	4.979	0.229
M	Cxcr2	left PoDG	0.297	0.018
M	Cxcr2	left pontine nucleus	0.399	0.048
M	Cxcr2	left Posterolateral cortical amygdaloid area	0.409	0.031
M	Cxcr2	left Posteromedial cortical amygdaloid area	0.603	0.040
M	Cxcr2	left pre-para subiculum	1.151	0.076
M	Cxcr2	left Primary auditory cortex	0.707	0.035
M	Cxcr2	left Primary motor cortex	3.642	0.077
M	Cxcr2	left Primary somatosensory cortex	2.001	0.097
M	Cxcr2	left Primary somatosensory cortex: barrel field	4.502	0.184
M	Cxcr2	left Primary somatosensory cortex: dysgranular zone	0.164	0.013
M	Cxcr2	left Primary somatosensory cortex: forelimb region	1.964	0.132
M	Cxcr2	left Primary somatosensory cortex: hindlimb region	1.412	0.108
M	Cxcr2	left Primary somatosensory cortex: jaw region	0.263	0.025
M	Cxcr2	left Primary somatosensory cortex: shoulder region	0.091	0.006
M	Cxcr2	left Primary somatosensory cortex: trunk region	0.300	0.019
M	Cxcr2	left Primary somatosensory cortex: upper lip region	2.264	0.114
M	Cxcr2	left Primary visual cortex	1.144	0.069
M	Cxcr2	left Primary visual cortex: binocular area	1.025	0.070
M	Cxcr2	left Primary visual cortex: monocular area	1.179	0.065
M	Cxcr2	left Rostral amygdalopiriform area	0.189	0.018
M	Cxcr2	left Secondary auditory cortex: dorsal area	0.704	0.054
M	Cxcr2	left Secondary auditory cortex: ventral area	0.759	0.055
M	Cxcr2	left Secondary motor cortex	3.286	0.145
M	Cxcr2	left Secondary somatosensory cortex	3.253	0.248
M	Cxcr2	left Secondary visual cortex: lateral area	1.254	0.058
M	Cxcr2	left Secondary visual cortex: mediolateral area	0.572	0.029
M	Cxcr2	left Secondary visual cortex: mediomedial area	0.921	0.046
M	Cxcr2	left simple lobule (lobule 6)	2.274	0.080
M	Cxcr2	left simple lobule white matter	0.210	0.011
M	Cxcr2	left SLu	0.370	0.017
M	Cxcr2	left stria medullaris	0.320	0.013
M	Cxcr2	left stria terminalis	0.464	0.017
M	Cxcr2	left striatum	10.477	0.423
M	Cxcr2	left subependymale zone / rhinocele	0.028	0.002
M	Cxcr2	left subiculum	1.636	0.061
M	Cxcr2	left superior olivary complex	0.377	0.041
M	Cxcr2	left Temporal association area	1.336	0.109
M	Cxcr2	left thalamus	8.656	0.316
M	Cxcr2	left trunk of crus 2 and paramedian white matter	0.163	0.012
M	Cxcr2	left trunk of simple and crus 1 white matter	0.067	0.004
M	Cxcr2	left Ventral intermediate entorhinal cortex	0.556	0.028
M	Cxcr2	left Ventral nucleus of the endopiriform claustrum	0.257	0.018
M	Cxcr2	left Ventral orbital cortex	0.762	0.044
M	Cxcr2	left Ventral tenia tecta	0.055	0.005
M	Cxcr2	lobule 1-2 white matter	0.060	0.012
M	Cxcr2	lobule 10 white matter	0.083	0.004
M	Cxcr2	lobule 10: nodulus	1.345	0.059
M	Cxcr2	lobule 3 white matter	0.181	0.013
M	Cxcr2	lobule 3: central lobule (dorsal)	2.093	0.151
M	Cxcr2	lobule 6: declive	2.421	0.138
M	Cxcr2	lobule 7: tuber (or folium)	0.952	0.064
M	Cxcr2	lobule 8 white matter	0.153	0.009
M	Cxcr2	lobule 8: pyramis	1.582	0.082
M	Cxcr2	lobule 9 white matter	0.279	0.014
M	Cxcr2	lobule 9: uvula	2.687	0.141
M	Cxcr2	lobules 1-2: lingula and central lobule (ventral)	1.582	0.178
M	Cxcr2	lobules 4-5 white matter	0.551	0.026
M	Cxcr2	lobules 4-5: culmen (ventral and dorsal)	4.181	0.167
M	Cxcr2	lobules 6-7 white matter	0.676	0.036
M	Cxcr2	medulla	27.318	0.938
M	Cxcr2	midbrain	13.680	0.563
M	Cxcr2	periaqueductal grey	3.976	0.193
M	Cxcr2	pons	17.080	0.498
M	Cxcr2	posterior commissure	0.141	0.009
M	Cxcr2	right Accessory olfactory bulb: glomerular, external plexiform and mitral cell layer	0.242	0.014
M	Cxcr2	right Accessory olfactory bulb: granule cell layer	0.116	0.008
M	Cxcr2	right amygdala	4.650	0.186
M	Cxcr2	right Amygdalopiriform transition area	0.491	0.031
M	Cxcr2	right anterior commissure: pars anterior	0.470	0.019
M	Cxcr2	right anterior commissure: pars posterior	0.200	0.014
M	Cxcr2	right anterior lobule (lobules 4-5)	0.765	0.039
M	Cxcr2	right anterior lobule white matter	0.049	0.004
M	Cxcr2	right Anterior olfactory nucleus	0.970	0.055
M	Cxcr2	right basal forebrain	2.704	0.076
M	Cxcr2	right bed nucleus of stria terminalis	0.689	0.034
M	Cxcr2	right CA10r	1.026	0.063
M	Cxcr2	right CA1Py	0.558	0.033
M	Cxcr2	right CA1Rad	1.344	0.061
M	Cxcr2	right CA20r	0.223	0.016
M	Cxcr2	right CA2Py	0.100	0.006
M	Cxcr2	right CA2Rad	0.222	0.013
M	Cxcr2	right CA30r	1.389	0.044
M	Cxcr2	right CA3Py Inner	0.078	0.005
M	Cxcr2	right CA3Py Outer	0.509	0.015
M	Cxcr2	right CA3Rad	0.939	0.042
M	Cxcr2	right Caudomedial entorhinal cortex	2.891	0.177
M	Cxcr2	right cerebellar peduncle: inferior	0.387	0.014
M	Cxcr2	right cerebellar peduncle: middle	0.608	0.041
M	Cxcr2	right cerebellar peduncle: superior	0.488	0.025
M	Cxcr2	right cerebral peduncle	1.046	0.049
M	Cxcr2	right Cingulate cortex: area 24a	0.976	0.080
M	Cxcr2	right Cingulate cortex: area 24a'	0.343	0.040
M	Cxcr2	right Cingulate cortex: area 24b	0.762	0.055
M	Cxcr2	right Cingulate cortex: area 24b'	0.282	0.020
M	Cxcr2	right Cingulate cortex: area 25	0.297	0.014
M	Cxcr2	right Cingulate cortex: area 29a	0.372	0.030
M	Cxcr2	right Cingulate cortex: area 29b	0.204	0.013
M	Cxcr2	right Cingulate cortex: area 29c	0.918	0.068
M	Cxcr2	right Cingulate cortex: area 30	1.345	0.054
M	Cxcr2	right Cingulate cortex: area 32	1.027	0.086
M	Cxcr2	right Cingulum	0.435	0.021
M	Cxcr2	right Claustrum	0.141	0.009
M	Cxcr2	right Claustrum: dorsal part	0.131	0.023
M	Cxcr2	right Claustrum: ventral part	0.284	0.033
M	Cxcr2	right colliculus: inferior	2.689	0.093
M	Cxcr2	right colliculus: superior	4.354	0.190
M	Cxcr2	right copula white matter	0.033	0.002
M	Cxcr2	right copula: pyramis (lobule 8)	1.242	0.081
M	Cxcr2	right corpus callosum	5.699	0.272
M	Cxcr2	right Cortex-amygdala transition zones	0.438	0.028
M	Cxcr2	right corticospinal tract/pyramids	0.894	0.049
M	Cxcr2	right crus 1 white matter	0.178	0.007
M	Cxcr2	right crus 1: ansiform lobule (lobule 6)	2.133	0.056
M	Cxcr2	right crus 2 white matter	0.148	0.011
M	Cxcr2	right crus 2: ansiform lobule (lobule 7)	2.026	0.091
M	Cxcr2	right cuneate nucleus	0.122	0.009
M	Cxcr2	right dentate nucleus	0.178	0.008
M	Cxcr2	right Dorsal intermediate entorhinal cortex	0.877	0.043
M	Cxcr2	right Dorsal nucleus of the endopiriform	0.686	0.029
M	Cxcr2	right Dorsal tenia tecta	0.451	0.024
M	Cxcr2	right Dorsolateral entorhinal cortex	1.331	0.062
M	Cxcr2	right Dorsolateral orbital cortex	0.407	0.028
M	Cxcr2	right Ectorhinal cortex	1.352	0.107
M	Cxcr2	right facial nerve (cranial nerve 7)	0.107	0.004
M	Cxcr2	right fasciculus retroflexus	0.113	0.008
M	Cxcr2	right fastigial nucleus	0.237	0.012
M	Cxcr2	right fimbria	1.496	0.066
M	Cxcr2	right flocculus (FL)	0.520	0.034
M	Cxcr2	right flocculus white matter	0.039	0.004
M	Cxcr2	right fornix	0.297	0.015
M	Cxcr2	right Frontal association cortex	3.750	0.202
M	Cxcr2	right Frontal cortex: area 3	0.358	0.021
M	Cxcr2	right fundus of striatum	0.082	0.006
M	Cxcr2	right globus pallidus	1.631	0.067
M	Cxcr2	right GrDG	0.633	0.039
M	Cxcr2	right habenular commissure	0.014	0.002
M	Cxcr2	right hypothalamus	5.252	0.158
M	Cxcr2	right inferior olivary complex	0.175	0.019
M	Cxcr2	right Insular region: not subdivided	3.955	0.317
M	Cxcr2	right Intermediate nucleus of the endopiriform claustrum	0.281	0.013
M	Cxcr2	right internal capsule	1.492	0.057
M	Cxcr2	right lateral olfactory tract	0.642	0.054
M	Cxcr2	right Lateral orbital cortex	1.730	0.086
M	Cxcr2	right Lateral parietal association cortex	0.087	0.007
M	Cxcr2	right lateral septum	1.503	0.053
M	Cxcr2	right lateral ventricle	1.790	0.107
M	Cxcr2	right LMol	1.171	0.054
M	Cxcr2	right mammillary bodies	0.224	0.017
M	Cxcr2	right mammilothalamic tract	0.127	0.008
M	Cxcr2	right Medial amygdala	0.590	0.085
M	Cxcr2	right Medial entorhinal cortex	0.337	0.014
M	Cxcr2	right medial lemniscus/medial longitudinal fasciculus	1.317	0.066
M	Cxcr2	right Medial orbital cortex	0.834	0.057
M	Cxcr2	right Medial parietal association cortex	0.169	0.016
M	Cxcr2	right Medial preoptic nucleus	0.092	0.006
M	Cxcr2	right medial septum	0.614	0.024
M	Cxcr2	right MoDG	2.023	0.089
M	Cxcr2	right nucleus accumbens	2.033	0.055
M	Cxcr2	right nucleus interpositus	0.240	0.012
M	Cxcr2	right Olfactory bulb: external plexiform layer	3.142	0.307
M	Cxcr2	right Olfactory bulb: glomerular layer	2.227	0.214
M	Cxcr2	right Olfactory bulb: granule cell layer	2.220	0.196
M	Cxcr2	right Olfactory bulb: internal plexiform layer	0.468	0.062
M	Cxcr2	right Olfactory bulb: mitral cell layer	0.571	0.068
M	Cxcr2	right olfactory peduncle	1.113	0.058
M	Cxcr2	right olfactory tubercle	1.686	0.133
M	Cxcr2	right optic tract	0.724	0.030
M	Cxcr2	right paraflocculus (PFL)	1.739	0.158
M	Cxcr2	right paraflocculus white matter	0.167	0.015
M	Cxcr2	right paramedian lobule	0.076	0.006
M	Cxcr2	right paramedian lobule (lobule 7)	1.989	0.115
M	Cxcr2	right Parietal cortex: posterior area: rostral part	0.042	0.004
M	Cxcr2	right Perirhinal cortex	1.206	0.055
M	Cxcr2	right Piriform cortex	5.409	0.232
M	Cxcr2	right PoDG	0.313	0.019
M	Cxcr2	right pontine nucleus	0.380	0.043
M	Cxcr2	right Posterolateral cortical amygdaloid area	0.481	0.038
M	Cxcr2	right Posteromedial cortical amygdaloid area	0.479	0.040
M	Cxcr2	right pre-para subiculum	1.167	0.043
M	Cxcr2	right Primary auditory cortex	0.731	0.037
M	Cxcr2	right Primary motor cortex	3.104	0.139
M	Cxcr2	right Primary somatosensory cortex	2.047	0.099

M	Cxcr2	right Primary somatosensory cortex: barrel field	4.471	0.196
M	Cxcr2	right Primary somatosensory cortex: dysgranular zone	0.179	0.011
M	Cxcr2	right Primary somatosensory cortex: forelimb region	1.739	0.096
M	Cxcr2	right Primary somatosensory cortex: hindlimb region	1.139	0.082
M	Cxcr2	right Primary somatosensory cortex: jaw region	0.277	0.018
M	Cxcr2	right Primary somatosensory cortex: shoulder region	0.066	0.011
M	Cxcr2	right Primary somatosensory cortex: trunk region	0.194	0.015
M	Cxcr2	right Primary somatosensory cortex: upper lip region	2.717	0.140
M	Cxcr2	right Primary visual cortex	1.156	0.055
M	Cxcr2	right Primary visual cortex: binocular area	1.008	0.074
M	Cxcr2	right Primary visual cortex: monocular area	0.802	0.083
M	Cxcr2	right Rostral amygdalopiriform area	0.204	0.015
M	Cxcr2	right Secondary auditory cortex: dorsal area	0.706	0.040
M	Cxcr2	right Secondary auditory cortex: ventral area	0.945	0.069
M	Cxcr2	right Secondary motor cortex	3.143	0.125
M	Cxcr2	right Secondary somatosensory cortex	3.547	0.335
M	Cxcr2	right Secondary visual cortex: lateral area	1.450	0.077
M	Cxcr2	right Secondary visual cortex: mediolateral area	0.397	0.026
M	Cxcr2	right Secondary visual cortex: mediomedial area	0.778	0.033
M	Cxcr2	right simple lobule (lobule 6)	2.299	0.083
M	Cxcr2	right simple lobule white matter	0.171	0.007
M	Cxcr2	right SLu	0.365	0.016
M	Cxcr2	right stria medullaris	0.343	0.014
M	Cxcr2	right stria terminalis	0.394	0.013
M	Cxcr2	right striatum	10.315	0.407
M	Cxcr2	right subependymale zone / rhinocele	0.025	0.002
M	Cxcr2	right subiculum	1.623	0.062
M	Cxcr2	right superior olivary complex	0.398	0.029
M	Cxcr2	right Temporal association area	1.499	0.114
M	Cxcr2	right thalamus	8.603	0.373
M	Cxcr2	right trunk of crus 2 and paramedian white matter	0.178	0.016
M	Cxcr2	right trunk of simple and crus 1 white matter	0.083	0.005
M	Cxcr2	right Ventral intermediate entorhinal cortex	0.538	0.024
M	Cxcr2	right Ventral nucleus of the endopiriform claustrum	0.231	0.031
M	Cxcr2	right Ventral orbital cortex	0.703	0.046
M	Cxcr2	right Ventral tenia tecta	0.056	0.006
M	Cxcr2	third ventricle	1.230	0.077
M	Cxcr2	trunk of arbor vita	4.110	0.098
M	Cxcr2	trunk of lobules 1-3 white matter	0.119	0.012
M	Cxcr2	trunk of lobules 6-8 white matter	0.102	0.006
M	Cxcr2	ventral tegmental decussation	0.129	0.009
M	Ighm	cerebral aqueduct	0.477	0.024
M	Ighm	fourth ventricle	0.916	0.068
M	Ighm	interpedunclar nucleus	0.268	0.014
M	Ighm	left Accessory olfactory bulb: glomerular, external plexiform and mitral cell layer	0.259	0.011
M	Ighm	left Accessory olfactory bulb: granule cell layer	0.126	0.006
M	Ighm	left amygdala	4.899	0.192
M	Ighm	left Amygdalopiriform transition area	0.531	0.032
M	Ighm	left anterior commissure: pars anterior	0.490	0.018
M	Ighm	left anterior commissure: pars posterior	0.208	0.013
M	Ighm	left anterior lobule (lobules 4-5)	0.871	0.062
M	Ighm	left anterior lobule white matter	0.043	0.005
M	Ighm	left Anterior olfactory nucleus	0.983	0.034
M	Ighm	left basal forebrain	2.615	0.070
M	Ighm	left bed nucleus of stria terminalis	0.703	0.029
M	Ighm	left CA10r	1.174	0.037
M	Ighm	left CA1Py	0.563	0.015
M	Ighm	left CA1Rad	1.256	0.028
M	Ighm	left CA20r	0.262	0.012
M	Ighm	left CA2Py	0.102	0.003
M	Ighm	left CA2Rad	0.207	0.010
M	Ighm	left CA30r	1.432	0.075
M	Ighm	left CA3Py Inner	0.076	0.003
M	Ighm	left CA3Py Outer	0.524	0.022
M	Ighm	left CA3Rad	0.918	0.034
M	Ighm	left Caudomedial entorhinal cortex	2.960	0.104
M	Ighm	left cerebellar peduncle: inferior	0.426	0.013
M	Ighm	left cerebellar peduncle: middle	0.568	0.017
M	Ighm	left cerebellar peduncle: superior	0.519	0.030
M	Ighm	left cerebral peduncle	1.029	0.035
M	Ighm	left Cingulate cortex: area 24a	0.929	0.045
M	Ighm	left Cingulate cortex: area 24a'	0.369	0.033
M	Ighm	left Cingulate cortex: area 24b	0.803	0.065
M	Ighm	left Cingulate cortex: area 24b'	0.304	0.024
M	Ighm	left Cingulate cortex: area 25	0.296	0.013
M	Ighm	left Cingulate cortex: area 29a	0.363	0.024
M	Ighm	left Cingulate cortex: area 29b	0.211	0.012
M	Ighm	left Cingulate cortex: area 29c	0.904	0.045
M	Ighm	left Cingulate cortex: area 30	1.444	0.083
M	Ighm	left Cingulate cortex: area 32	1.334	0.055
M	Ighm	left Cingulum	0.433	0.032
M	Ighm	left Claustrum	0.175	0.011
M	Ighm	left Claustrum: dorsal part	0.151	0.020
M	Ighm	left Claustrum: ventral part	0.278	0.029
M	Ighm	left colliculus: inferior	2.886	0.098
M	Ighm	left colliculus: superior	4.404	0.188
M	Ighm	left copula white matter	0.045	0.002
M	Ighm	left copula: pyramis (lobule 8)	1.172	0.059
M	Ighm	left corpus callosum	6.101	0.261
M	Ighm	left Cortex-amygdala transition zones	0.373	0.031
M	Ighm	left corticospinal tract/pyramids	0.848	0.034
M	Ighm	left crus 1 white matter	0.189	0.012
M	Ighm	left crus 1: ansiform lobule (lobule 6)	2.157	0.085
M	Ighm	left crus 2 white matter	0.126	0.011
M	Ighm	left crus 2: ansiform lobule (lobule 7)	1.980	0.118
M	Ighm	left cuneate nucleus	0.119	0.006
M	Ighm	left dentate nucleus	0.181	0.011
M	Ighm	left Dorsal intermediate entorhinal cortex	0.846	0.032
M	Ighm	left Dorsal nucleus of the endopiriform	0.737	0.035
M	Ighm	left Dorsal tenia tecta	0.499	0.024
M	Ighm	left Dorsolateral entorhinal cortex	1.238	0.041
M	Ighm	left Dorsolateral orbital cortex	0.422	0.020
M	Ighm	left Ectorhinal cortex	1.316	0.089
M	Ighm	left facial nerve (cranial nerve 7)	0.106	0.006
M	Ighm	left fasciculus retroflexus	0.132	0.006
M	Ighm	left fastigial nucleus	0.243	0.012
M	Ighm	left fimbria	1.648	0.071
M	Ighm	left flocculus (FL)	0.543	0.037
M	Ighm	left flocculus white matter	0.028	0.003
M	Ighm	left fornix	0.338	0.013
M	Ighm	left Frontal association cortex	3.507	0.116
M	Ighm	left Frontal cortex: area 3	0.348	0.020
M	Ighm	left fundus of striatum	0.074	0.004
M	Ighm	left globus pallidus	1.656	0.060
M	Ighm	left GrDG	0.685	0.017
M	Ighm	left habenular commissure	0.013	0.002
M	Ighm	left hypothalamus	5.318	0.134
M	Ighm	left inferior olivary complex	0.158	0.017
M	Ighm	left Insular region: not subdivided	3.784	0.319
M	Ighm	left Intermediate nucleus of the endopiriform claustrum	0.312	0.020
M	Ighm	left internal capsule	1.454	0.061
M	Ighm	left lateral olfactory tract	0.748	0.030
M	Ighm	left Lateral orbital cortex	1.783	0.060
M	Ighm	left Lateral parietal association cortex	0.148	0.011
M	Ighm	left lateral septum	1.531	0.039
M	Ighm	left lateral ventricle	1.798	0.183
M	Ighm	left LMol	1.189	0.026
M	Ighm	left mammillary bodies	0.230	0.015
M	Ighm	left mammilothalamic tract	0.124	0.007
M	Ighm	left Medial amygdala	0.541	0.039
M	Ighm	left Medial entorhinal cortex	0.355	0.026
M	Ighm	left medial lemniscus/medial longitudinal fasciculus	1.250	0.037
M	Ighm	left Medial orbital cortex	0.921	0.050
M	Ighm	left Medial parietal association cortex	0.236	0.024
M	Ighm	left Medial preoptic nucleus	0.101	0.005
M	Ighm	left medial septum	0.651	0.022
M	Ighm	left MoDG	2.201	0.050
M	Ighm	left nucleus accumbens	2.090	0.067
M	Ighm	left nucleus interpositus	0.230	0.013
M	Ighm	left Olfactory bulb: external plexiform layer	3.277	0.106
M	Ighm	left Olfactory bulb: glomerular layer	2.208	0.088
M	Ighm	left Olfactory bulb: granule cell layer	2.307	0.064
M	Ighm	left Olfactory bulb: internal plexiform layer	0.529	0.019
M	Ighm	left Olfactory bulb: mitral cell layer	0.661	0.022
M	Ighm	left olfactory peduncle	1.176	0.042
M	Ighm	left olfactory tubercle	1.614	0.059
M	Ighm	left optic tract	0.781	0.040
M	Ighm	left paraflocculus (PFL)	1.781	0.238
M	Ighm	left paraflocculus white matter	0.145	0.015
M	Ighm	left paramedian lobule	0.070	0.004
M	Ighm	left paramedian lobule (lobule 7)	1.958	0.074
M	Ighm	left Parietal cortex: posterior area: rostral part	0.068	0.005
M	Ighm	left Perirhinal cortex	1.232	0.058
M	Ighm	left Piriform cortex	5.017	0.221
M	Ighm	left PoDG	0.304	0.008
M	Ighm	left pontine nucleus	0.376	0.031
M	Ighm	left Posterolateral cortical amygdaloid area	0.418	0.024
M	Ighm	left Posteromedial cortical amygdaloid area	0.595	0.030
M	Ighm	left pre-para subiculum	1.134	0.064
M	Ighm	left Primary auditory cortex	0.742	0.054
M	Ighm	left Primary motor cortex	3.634	0.120
M	Ighm	left Primary somatosensory cortex	1.988	0.115
M	Ighm	left Primary somatosensory cortex: barrel field	4.594	0.182
M	Ighm	left Primary somatosensory cortex: dysgranular zone	0.161	0.013
M	Ighm	left Primary somatosensory cortex: forelimb region	2.021	0.124
M	Ighm	left Primary somatosensory cortex: hindlimb region	1.448	0.099
M	Ighm	left Primary somatosensory cortex: jaw region	0.258	0.026
M	Ighm	left Primary somatosensory cortex: shoulder region	0.089	0.009
M	Ighm	left Primary somatosensory cortex: trunk region	0.300	0.021
M	Ighm	left Primary somatosensory cortex: upper lip region	2.343	0.163
M	Ighm	left Primary visual cortex	1.143	0.074
M	Ighm	left Primary visual cortex: binocular area	0.991	0.048
M	Ighm	left Primary visual cortex: monocular area	1.133	0.068
M	Ighm	left Rostral amygdalopiriform area	0.190	0.013
M	Ighm	left Secondary auditory cortex: dorsal area	0.713	0.029
M	Ighm	left Secondary auditory cortex: ventral area	0.765	0.048
M	Ighm	left Secondary motor cortex	3.299	0.142
M	Ighm	left Secondary somatosensory cortex	3.332	0.306
M	Ighm	left Secondary visual cortex: lateral area	1.299	0.067
M	Ighm	left Secondary visual cortex: mediolateral area	0.581	0.049
M	Ighm	left Secondary visual cortex: mediomedial area	0.951	0.051
M	Ighm	left simple lobule (lobule 6)	2.310	0.105
M	Ighm	left simple lobule white matter	0.213	0.015
M	Ighm	left SLu	0.379	0.017
M	Ighm	left stria medullaris	0.326	0.017
M	Ighm	left stria terminalis	0.475	0.023
M	Ighm	left striatum	10.727	0.387
M	Ighm	left subependymale zone / rhinocele	0.029	0.002
M	Ighm	left subiculum	1.673	0.060
M	Ighm	left superior olivary complex	0.373	0.023
M	Ighm	left Temporal association area	1.364	0.088
M	Ighm	left thalamus	8.913	0.342
M	Ighm	left trunk of crus 2 and paramedian white matter	0.166	0.010
M	Ighm	left trunk of simple and crus 1 white matter	0.069	0.005
M	Ighm	left Ventral intermediate entorhinal cortex	0.559	0.024
M	Ighm	left Ventral nucleus of the endopiriform claustrum	0.247	0.019
M	Ighm	left Ventral orbital cortex	0.764	0.039
M	Ighm	left Ventral tenia tecta	0.055	0.004
M	Ighm	lobule 1-2 white matter	0.075	0.023
M	Ighm	lobule 10 white matter	0.082	0.005
M	Ighm	lobule 10: nodulus	1.348	0.055
M	Ighm	lobule 3 white matter	0.171	0.029
M	Ighm	lobule 3: central lobule (dorsal)	1.985	0.185
M	Ighm	lobule 6: declive	2.412	0.158
M	Ighm	lobule 7: tuber (or folium)	0.930	0.049
M	Ighm	lobule 8 white matter	0.158	0.006
M	Ighm	lobule 8: pyramis	1.581	0.053
M	Ighm	lobule 9 white matter	0.294	0.011
M	Ighm	lobule 9: uvula	2.804	0.113
M	Ighm	lobules 1-2: lingula and central lobule (ventral)	1.717	0.336
M	Ighm	lobules 4-5 white matter	0.555	0.053
M	Ighm	lobules 4-5: culmen (ventral and dorsal)	4.214	0.317
M	Ighm	lobules 6-7 white matter	0.685	0.045
M	Ighm	medulla	27.436	0.859
M	Ighm	midbrain	13.974	0.527
M	Ighm	periaqueductal grey	3.977	0.172
M	Ighm	pons	17.126	0.473
M	Ighm	posterior commissure	0.145	0.009
M	Ighm	right Accessory olfactory bulb: glomerular, external plexiform and mitral cell layer	0.251	0.010
M	Ighm	right Accessory olfactory bulb: granule cell layer	0.119	0.005
M	Ighm	right amygdala	4.571	0.181
M	Ighm	right Amygdalopiriform transition area	0.480	0.022
M	Ighm	right anterior commissure: pars anterior	0.483	0.017
M	Ighm	right anterior commissure: pars posterior	0.200	0.012
M	Ighm	right anterior lobule (lobules 4-5)	0.776	0.050
M	Ighm	right anterior lobule white matter	0.050	0.005
M	Ighm	right Anterior olfactory nucleus	1.013	0.041
M	Ighm	right basal forebrain	2.728	0.056
M	Ighm	right bed nucleus of stria terminalis	0.702	0.027
M	Ighm	right CA10r	1.028	0.038
M	Ighm	right CA1Py	0.569	0.019
M	Ighm	right CA1Rad	1.368	0.033
M	Ighm	right CA20r	0.217	0.010
M	Ighm	right CA2Py	0.099	0.004
M	Ighm	right CA2Rad	0.220	0.010
M	Ighm	right CA30r	1.425	0.061
M	Ighm	right CA3Py Inner	0.081	0.004
M	Ighm	right CA3Py Outer	0.522	0.018
M	Ighm	right CA3Rad	0.957	0.034
M	Ighm	right Caudomedial entorhinal cortex	2.889	0.087
M	Ighm	right cerebellar peduncle: inferior	0.395	0.014
M	Ighm	right cerebellar peduncle: middle	0.602	0.030
M	Ighm	right cerebellar peduncle: superior	0.502	0.024
M	Ighm	right cerebral peduncle	1.042	0.039
M	Ighm	right Cingulate cortex: area 24a	0.993	0.045
M	Ighm	right Cingulate cortex: area 24a'	0.346	0.028
M	Ighm	right Cingulate cortex: area 24b	0.775	0.045
M	Ighm	right Cingulate cortex: area 24b'	0.286	0.021
M	Ighm	right Cingulate cortex: area 25	0.301	0.010
M	Ighm	right Cingulate cortex: area 29a	0.349	0.023
M	Ighm	right Cingulate cortex: area 29b	0.201	0.012
M	Ighm	right Cingulate cortex: area 29c	0.865	0.057
M	Ighm	right Cingulate cortex: area 30	1.365	0.060
M	Ighm	right Cingulate cortex: area 32	1.080	0.051
M	Ighm	right Cingulum	0.438	0.030
M	Ighm	right Claustrum	0.147	0.007
M	Ighm	right Claustrum: dorsal part	0.142	0.017
M	Ighm	right Claustrum: ventral part	0.311	0.030
M	Ighm	right colliculus: inferior	2.743	0.100
M	Ighm	right colliculus: superior	4.472	0.162
M	Ighm	right copula white matter	0.036	0.002
M	Ighm	right copula: pyramis (lobule 8)	1.291	0.082
M	Ighm	right corpus callosum	5.861	0.232
M	Ighm	right Cortex-amygdala transition zones	0.407	0.023
M	Ighm	right corticospinal tract/pyramids	0.896	0.037
M	Ighm	right crus 1 white matter	0.182	0.011
M	Ighm	right crus 1: ansiform lobule (lobule 6)	2.187	0.097

M	Ighm	right crus 2 white matter	0.144	0.012
M	Ighm	right crus 2: ansiform lobule (lobule 7)	1.971	0.153
M	Ighm	right cuneate nucleus	0.126	0.009
M	Ighm	right dentate nucleus	0.174	0.011
M	Ighm	right Dorsal intermediate entorhinal cortex	0.878	0.028
M	Ighm	right Dorsal nucleus of the endopiriform	0.730	0.037
M	Ighm	right Dorsal tenia tecta	0.462	0.025
M	Ighm	right Dorsolateral entorhinal cortex	1.370	0.066
M	Ighm	right Dorsolateral orbital cortex	0.389	0.031
M	Ighm	right Ectorhinal cortex	1.361	0.098
M	Ighm	right facial nerve (cranial nerve 7)	0.110	0.005
M	Ighm	right fasciculus retroflexus	0.116	0.006
M	Ighm	right fastigial nucleus	0.233	0.012
M	Ighm	right fimbria	1.527	0.060
M	Ighm	right flocculus (FL)	0.515	0.032
M	Ighm	right flocculus white matter	0.038	0.003
M	Ighm	right fornix	0.298	0.014
M	Ighm	right Frontal association cortex	3.873	0.134
M	Ighm	right Frontal cortex: area 3	0.352	0.018
M	Ighm	right fundus of striatum	0.083	0.007
M	Ighm	right globus pallidus	1.679	0.058
M	Ighm	right GrDG	0.648	0.022
M	Ighm	right habenular commissure	0.013	0.001
M	Ighm	right hypothalamus	5.252	0.126
M	Ighm	right inferior olivary complex	0.174	0.019
M	Ighm	right Insular region: not subdivided	4.053	0.265
M	Ighm	right Intermediate nucleus of the endopiriform claustrum	0.291	0.015
M	Ighm	right internal capsule	1.545	0.059
M	Ighm	right lateral olfactory tract	0.665	0.023
M	Ighm	right Lateral orbital cortex	1.745	0.052
M	Ighm	right Lateral parietal association cortex	0.089	0.010
M	Ighm	right lateral septum	1.529	0.041
M	Ighm	right lateral ventricle	1.907	0.197
M	Ighm	right LMol	1.179	0.025
M	Ighm	right mammillary bodies	0.212	0.016
M	Ighm	right mammilothalamic tract	0.128	0.007
M	Ighm	right Medial amygdala	0.567	0.027
M	Ighm	right Medial entorhinal cortex	0.344	0.020
M	Ighm	right medial lemniscus/medial longitudinal fasciculus	1.347	0.056
M	Ighm	right Medial orbital cortex	0.832	0.034
M	Ighm	right Medial parietal association cortex	0.168	0.015
M	Ighm	right Medial preoptic nucleus	0.093	0.007
M	Ighm	right medial septum	0.606	0.016
M	Ighm	right MoDG	2.054	0.057
M	Ighm	right nucleus accumbens	2.093	0.052
M	Ighm	right nucleus interpositus	0.237	0.016
M	Ighm	right Olfactory bulb: external plexiform layer	3.270	0.102
M	Ighm	right Olfactory bulb: glomerular layer	2.288	0.065
M	Ighm	right Olfactory bulb: granule cell layer	2.377	0.068
M	Ighm	right Olfactory bulb: internal plexiform layer	0.526	0.020
M	Ighm	right Olfactory bulb: mitral cell layer	0.625	0.024
M	Ighm	right olfactory peduncle	1.143	0.039
M	Ighm	right olfactory tubercle	1.655	0.072
M	Ighm	right optic tract	0.736	0.028
M	Ighm	right paraflocculus (PFL)	1.696	0.214
M	Ighm	right paraflocculus white matter	0.163	0.015
M	Ighm	right paramedian lobule	0.078	0.006
M	Ighm	right paramedian lobule (lobule 7)	1.978	0.130
M	Ighm	right Parietal cortex: posterior area: rostral part	0.045	0.004
M	Ighm	right Perirhinal cortex	1.189	0.058
M	Ighm	right Piriform cortex	5.333	0.175
M	Ighm	right PoDG	0.320	0.012
M	Ighm	right pontine nucleus	0.367	0.029
M	Ighm	right Posterolateral cortical amygdaloid area	0.466	0.024
M	Ighm	right Posteromedial cortical amygdaloid area	0.481	0.040
M	Ighm	right pre-para subiculum	1.160	0.040
M	Ighm	right Primary auditory cortex	0.726	0.048
M	Ighm	right Primary motor cortex	3.137	0.145
M	Ighm	right Primary somatosensory cortex	2.042	0.092
M	Ighm	right Primary somatosensory cortex: barrel field	4.577	0.148
M	Ighm	right Primary somatosensory cortex: dysgranular zone	0.177	0.011
M	Ighm	right Primary somatosensory cortex: forelimb region	1.805	0.079
M	Ighm	right Primary somatosensory cortex: hindlimb region	1.189	0.079
M	Ighm	right Primary somatosensory cortex: jaw region	0.286	0.020
M	Ighm	right Primary somatosensory cortex: shoulder region	0.063	0.006
M	Ighm	right Primary somatosensory cortex: trunk region	0.192	0.016
M	Ighm	right Primary somatosensory cortex: upper lip region	2.744	0.107
M	Ighm	right Primary visual cortex	1.171	0.074
M	Ighm	right Primary visual cortex: binocular area	1.016	0.075
M	Ighm	right Primary visual cortex: monocular area	0.774	0.047
M	Ighm	right Rostral amygdalopiriform area	0.203	0.018
M	Ighm	right Secondary auditory cortex: dorsal area	0.697	0.065
M	Ighm	right Secondary auditory cortex: ventral area	0.972	0.069
M	Ighm	right Secondary motor cortex	3.238	0.128
M	Ighm	right Secondary somatosensory cortex	3.564	0.302
M	Ighm	right Secondary visual cortex: lateral area	1.455	0.105
M	Ighm	right Secondary visual cortex: mediolateral area	0.398	0.032
M	Ighm	right Secondary visual cortex: mediomedial area	0.801	0.035
M	Ighm	right simple lobule (lobule 6)	2.346	0.097
M	Ighm	right simple lobule white matter	0.176	0.014
M	Ighm	right SLu	0.372	0.018
M	Ighm	right stria medullaris	0.348	0.016
M	Ighm	right stria terminalis	0.402	0.016
M	Ighm	right striatum	10.610	0.301
M	Ighm	right subependymale zone / rhinocele	0.027	0.001
M	Ighm	right subiculum	1.658	0.047
M	Ighm	right superior olivary complex	0.391	0.026
M	Ighm	right Temporal association area	1.566	0.110
M	Ighm	right thalamus	8.823	0.347
M	Ighm	right trunk of crus 2 and paramedian white matter	0.176	0.015
M	Ighm	right trunk of simple and crus 1 white matter	0.083	0.007
M	Ighm	right Ventral intermediate entorhinal cortex	0.542	0.030
M	Ighm	right Ventral nucleus of the endopiriform claustrum	0.233	0.016
M	Ighm	right Ventral orbital cortex	0.686	0.027
M	Ighm	right Ventral tenia tecta	0.055	0.003
M	Ighm	third ventricle	1.222	0.052
M	Ighm	trunk of arbor vita	4.165	0.164
M	Ighm	trunk of lobules 1-3 white matter	0.122	0.012
M	Ighm	trunk of lobules 6-8 white matter	0.111	0.007
M	Ighm	ventral tegmental decussation	0.129	0.011
M	IL-6	cerebral aqueduct	0.493	0.038
M	IL-6	fourth ventricle	0.913	0.048
M	IL-6	interpedunclar nucleus	0.282	0.011
M	IL-6	left Accessory olfactory bulb: glomerular, external plexiform and mitral cell layer	0.262	0.012
M	IL-6	left Accessory olfactory bulb: granule cell layer	0.130	0.007
M	IL-6	left amygdala	5.017	0.204
M	IL-6	left Amygdalopiriform transition area	0.531	0.040
M	IL-6	left anterior commissure: pars anterior	0.522	0.019
M	IL-6	left anterior commissure: pars posterior	0.224	0.007
M	IL-6	left anterior lobule (lobules 4-5)	0.929	0.038
M	IL-6	left anterior lobule white matter	0.045	0.002
M	IL-6	left Anterior olfactory nucleus	1.032	0.047
M	IL-6	left basal forebrain	2.690	0.104
M	IL-6	left bed nucleus of stria terminalis	0.734	0.030
M	IL-6	left CA10r	1.252	0.055
M	IL-6	left CA1Py	0.600	0.025
M	IL-6	left CA1Rad	1.316	0.048
M	IL-6	left CA20r	0.283	0.015
M	IL-6	left CA2Py	0.112	0.006
M	IL-6	left CA2Rad	0.219	0.010
M	IL-6	left CA30r	1.479	0.079
M	IL-6	left CA3Py Inner	0.080	0.003
M	IL-6	left CA3Py Outer	0.548	0.024
M	IL-6	left CA3Rad	0.967	0.032
M	IL-6	left Caudomedial entorhinal cortex	3.025	0.122
M	IL-6	left cerebellar peduncle: inferior	0.438	0.018
M	IL-6	left cerebellar peduncle: middle	0.600	0.020
M	IL-6	left cerebellar peduncle: superior	0.532	0.017
M	IL-6	left cerebral peduncle	1.051	0.040
M	IL-6	left Cingulate cortex: area 24a	0.990	0.062
M	IL-6	left Cingulate cortex: area 24a'	0.382	0.039
M	IL-6	left Cingulate cortex: area 24b	0.856	0.063
M	IL-6	left Cingulate cortex: area 24b'	0.307	0.024
M	IL-6	left Cingulate cortex: area 25	0.306	0.016
M	IL-6	left Cingulate cortex: area 29a	0.397	0.016
M	IL-6	left Cingulate cortex: area 29b	0.234	0.019
M	IL-6	left Cingulate cortex: area 29c	1.000	0.043
M	IL-6	left Cingulate cortex: area 30	1.577	0.072
M	IL-6	left Cingulate cortex: area 32	1.379	0.078
M	IL-6	left Cingulum	0.481	0.023
M	IL-6	left Claustrum	0.181	0.011
M	IL-6	left Claustrum: dorsal part	0.148	0.013
M	IL-6	left Claustrum: ventral part	0.273	0.022
M	IL-6	left colliculus: inferior	2.993	0.141
M	IL-6	left colliculus: superior	4.557	0.197
M	IL-6	left copula white matter	0.045	0.003
M	IL-6	left copula: pyramis (lobule 8)	1.178	0.083
M	IL-6	left corpus callosum	6.419	0.183
M	IL-6	left Cortex-amygdala transition zones	0.398	0.028
M	IL-6	left corticospinal tract/pyramids	0.889	0.053
M	IL-6	left crus 1 white matter	0.193	0.010
M	IL-6	left crus 1: ansiform lobule (lobule 6)	2.240	0.090
M	IL-6	left crus 2 white matter	0.128	0.008
M	IL-6	left crus 2: ansiform lobule (lobule 7)	1.982	0.119
M	IL-6	left cuneate nucleus	0.125	0.010
M	IL-6	left dentate nucleus	0.188	0.010
M	IL-6	left Dorsal intermediate entorhinal cortex	0.846	0.044
M	IL-6	left Dorsal nucleus of the endopiriform	0.762	0.028
M	IL-6	left Dorsal tenia tecta	0.510	0.028
M	IL-6	left Dorsolateral entorhinal cortex	1.247	0.073
M	IL-6	left Dorsolateral orbital cortex	0.445	0.020
M	IL-6	left Ectorhinal cortex	1.412	0.078
M	IL-6	left facial nerve (cranial nerve 7)	0.111	0.005
M	IL-6	left fasciculus retroflexus	0.138	0.007
M	IL-6	left fastigial nucleus	0.250	0.012
M	IL-6	left fimbria	1.740	0.079
M	IL-6	left flocculus (FL)	0.560	0.027
M	IL-6	left flocculus white matter	0.029	0.004
M	IL-6	left fornix	0.357	0.012
M	IL-6	left Frontal association cortex	3.566	0.106
M	IL-6	left Frontal cortex: area 3	0.367	0.016
M	IL-6	left fundus of striatum	0.077	0.003
M	IL-6	left globus pallidus	1.658	0.051
M	IL-6	left GrDG	0.713	0.028
M	IL-6	left habenular commissure	0.015	0.002
M	IL-6	left hypothalamus	5.485	0.159
M	IL-6	left inferior olivary complex	0.169	0.023
M	IL-6	left Insular region: not subdivided	3.928	0.191
M	IL-6	left Intermediate nucleus of the endopiriform claustrum	0.326	0.017
M	IL-6	left internal capsule	1.475	0.037
M	IL-6	left lateral olfactory tract	0.770	0.025
M	IL-6	left Lateral orbital cortex	1.873	0.062
M	IL-6	left Lateral parietal association cortex	0.157	0.013
M	IL-6	left lateral septum	1.597	0.071
M	IL-6	left lateral ventricle	1.890	0.303
M	IL-6	left LMol	1.247	0.037
M	IL-6	left mammillary bodies	0.243	0.010
M	IL-6	left mammilothalamic tract	0.128	0.006
M	IL-6	left Medial amygdala	0.548	0.021
M	IL-6	left Medial entorhinal cortex	0.359	0.027
M	IL-6	left medial lemniscus/medial longitudinal fasciculus	1.279	0.047
M	IL-6	left Medial orbital cortex	0.924	0.061
M	IL-6	left Medial parietal association cortex	0.260	0.011
M	IL-6	left Medial preoptic nucleus	0.106	0.005
M	IL-6	left medial septum	0.682	0.028
M	IL-6	left MoDG	2.293	0.091
M	IL-6	left nucleus accumbens	2.155	0.089
M	IL-6	left nucleus interpositus	0.235	0.014
M	IL-6	left Olfactory bulb: external plexiform layer	3.420	0.088
M	IL-6	left Olfactory bulb: glomerular layer	2.308	0.076
M	IL-6	left Olfactory bulb: granule cell layer	2.333	0.095
M	IL-6	left Olfactory bulb: internal plexiform layer	0.528	0.018
M	IL-6	left Olfactory bulb: mitral cell layer	0.669	0.019
M	IL-6	left olfactory peduncle	1.216	0.050
M	IL-6	left olfactory tubercle	1.661	0.051
M	IL-6	left optic tract	0.779	0.031
M	IL-6	left paraflocculus (PFL)	1.653	0.218
M	IL-6	left paraflocculus white matter	0.135	0.012
M	IL-6	left paramedian lobule	0.076	0.007
M	IL-6	left paramedian lobule (lobule 7)	2.131	0.100
M	IL-6	left Parietal cortex: posterior area: rostral part	0.071	0.007
M	IL-6	left Perirhinal cortex	1.312	0.058
M	IL-6	left Piriform cortex	5.268	0.188
M	IL-6	left PoDG	0.317	0.013
M	IL-6	left pontine nucleus	0.404	0.020
M	IL-6	left Posterolateral cortical amygdaloid area	0.415	0.031
M	IL-6	left Posteromedial cortical amygdaloid area	0.607	0.040
M	IL-6	left pre-para subiculum	1.201	0.049
M	IL-6	left Primary auditory cortex	0.747	0.069
M	IL-6	left Primary motor cortex	3.841	0.194
M	IL-6	left Primary somatosensory cortex	2.015	0.107
M	IL-6	left Primary somatosensory cortex: barrel field	4.758	0.243
M	IL-6	left Primary somatosensory cortex: dysgranular zone	0.171	0.013
M	IL-6	left Primary somatosensory cortex: forelimb region	2.099	0.104
M	IL-6	left Primary somatosensory cortex: hindlimb region	1.550	0.088
M	IL-6	left Primary somatosensory cortex: jaw region	0.260	0.021
M	IL-6	left Primary somatosensory cortex: shoulder region	0.095	0.010
M	IL-6	left Primary somatosensory cortex: trunk region	0.320	0.018
M	IL-6	left Primary somatosensory cortex: upper lip region	2.424	0.116
M	IL-6	left Primary visual cortex	1.315	0.122
M	IL-6	left Primary visual cortex: binocular area	1.033	0.060
M	IL-6	left Primary visual cortex: monocular area	1.196	0.099
M	IL-6	left Rostral amygdalopiriform area	0.197	0.019
M	IL-6	left Secondary auditory cortex: dorsal area	0.722	0.052
M	IL-6	left Secondary auditory cortex: ventral area	0.778	0.061
M	IL-6	left Secondary motor cortex	3.411	0.136
M	IL-6	left Secondary somatosensory cortex	3.509	0.214
M	IL-6	left Secondary visual cortex: lateral area	1.372	0.070
M	IL-6	left Secondary visual cortex: mediolateral area	0.610	0.032
M	IL-6	left Secondary visual cortex: mediomedial area	1.027	0.053
M	IL-6	left simple lobule (lobule 6)	2.526	0.114
M	IL-6	left simple lobule white matter	0.228	0.016
M	IL-6	left SLu	0.396	0.014
M	IL-6	left stria medullaris	0.341	0.011
M	IL-6	left stria terminalis	0.482	0.019
M	IL-6	left striatum	10.972	0.436
M	IL-6	left subependymale zone / rhinocele	0.030	0.002
M	IL-6	left subiculum	1.729	0.061
M	IL-6	left superior olivary complex	0.388	0.022
M	IL-6	left Temporal association area	1.435	0.063
M	IL-6	left thalamus	9.240	0.368
M	IL-6	left trunk of crus 2 and paramedian white matter	0.165	0.011
M	IL-6	left trunk of simple and crus 1 white matter	0.066	0.003
M	IL-6	left Ventral intermediate entorhinal cortex	0.544	0.029
M	IL-6	left Ventral nucleus of the endopiriform claustrum	0.252	0.020
M	IL-6	left Ventral orbital cortex	0.802	0.036
M	IL-6	left Ventral tenia tecta	0.059	0.005

M	IL-6	lobule 1-2 white matter	0.075	0.014
M	IL-6	lobule 10 white matter	0.085	0.004
M	IL-6	lobule 10: nodulus	1.377	0.052
M	IL-6	lobule 3 white matter	0.189	0.010
M	IL-6	lobule 3: central lobule (dorsal)	2.188	0.119
M	IL-6	lobule 6: declive	2.529	0.153
M	IL-6	lobule 7: tuber (or folium)	0.972	0.071
M	IL-6	lobule 8 white matter	0.148	0.009
M	IL-6	lobule 8: pyramis	1.535	0.082
M	IL-6	lobule 9 white matter	0.275	0.010
M	IL-6	lobule 9: uvula	2.682	0.119
M	IL-6	lobules 1-2: lingula and central lobule (ventral)	1.792	0.171
M	IL-6	lobules 4-5 white matter	0.584	0.028
M	IL-6	lobules 4-5: culmen (ventral and dorsal)	4.475	0.206
M	IL-6	lobules 6-7 white matter	0.695	0.036
M	IL-6	medulla	28.376	0.859
M	IL-6	midbrain	14.430	0.538
M	IL-6	periaqueductal grey	4.130	0.137
M	IL-6	pons	17.936	0.575
M	IL-6	posterior commissure	0.151	0.007
M	IL-6	right Accessory olfactory bulb: glomerular, external plexiform and mitral cell layer	0.259	0.013
M	IL-6	right Accessory olfactory bulb: granule cell layer	0.128	0.009
M	IL-6	right amygdala	4.691	0.164
M	IL-6	right Amygdalopiriform transition area	0.493	0.030
M	IL-6	right anterior commissure: pars anterior	0.514	0.020
M	IL-6	right anterior commissure: pars posterior	0.214	0.012
M	IL-6	right anterior lobule (lobules 4-5)	0.848	0.045
M	IL-6	right anterior lobule white matter	0.054	0.003
M	IL-6	right Anterior olfactory nucleus	1.073	0.058
M	IL-6	right basal forebrain	2.804	0.108
M	IL-6	right bed nucleus of stria terminalis	0.734	0.022
M	IL-6	right CA10r	1.099	0.041
M	IL-6	right CA1Py	0.607	0.023
M	IL-6	right CA1Rad	1.436	0.062
M	IL-6	right CA20r	0.236	0.013
M	IL-6	right CA2Py	0.107	0.005
M	IL-6	right CA2Rad	0.236	0.010
M	IL-6	right CA30r	1.491	0.071
M	IL-6	right CA3Py Inner	0.083	0.005
M	IL-6	right CA3Py Outer	0.553	0.029
M	IL-6	right CA3Rad	1.008	0.039
M	IL-6	right Caudomedial entorhinal cortex	2.970	0.122
M	IL-6	right cerebellar peduncle: inferior	0.399	0.021
M	IL-6	right cerebellar peduncle: middle	0.641	0.022
M	IL-6	right cerebellar peduncle: superior	0.516	0.023
M	IL-6	right cerebral peduncle	1.059	0.030
M	IL-6	right Cingulate cortex: area 24a	1.056	0.070
M	IL-6	right Cingulate cortex: area 24a'	0.367	0.037
M	IL-6	right Cingulate cortex: area 24b	0.816	0.064
M	IL-6	right Cingulate cortex: area 24b'	0.300	0.025
M	IL-6	right Cingulate cortex: area 25	0.317	0.014
M	IL-6	right Cingulate cortex: area 29a	0.377	0.020
M	IL-6	right Cingulate cortex: area 29b	0.213	0.012
M	IL-6	right Cingulate cortex: area 29c	0.967	0.055
M	IL-6	right Cingulate cortex: area 30	1.465	0.065
M	IL-6	right Cingulate cortex: area 32	1.142	0.066
M	IL-6	right Cingulum	0.481	0.024
M	IL-6	right Claustrum	0.149	0.005
M	IL-6	right Claustrum: dorsal part	0.134	0.014
M	IL-6	right Claustrum: ventral part	0.302	0.023
M	IL-6	right colliculus: inferior	2.853	0.112
M	IL-6	right colliculus: superior	4.652	0.186
M	IL-6	right copula white matter	0.033	0.002
M	IL-6	right copula: pyramis (lobule 8)	1.323	0.069
M	IL-6	right corpus callosum	6.209	0.195
M	IL-6	right Cortex-amygdala transition zones	0.431	0.026
M	IL-6	right corticospinal tract/pyramids	0.919	0.040
M	IL-6	right crus 1 white matter	0.192	0.008
M	IL-6	right crus 1: ansiform lobule (lobule 6)	2.308	0.103
M	IL-6	right crus 2 white matter	0.155	0.010
M	IL-6	right crus 2: ansiform lobule (lobule 7)	2.069	0.081
M	IL-6	right cuneate nucleus	0.125	0.011
M	IL-6	right dentate nucleus	0.186	0.010
M	IL-6	right Dorsal intermediate entorhinal cortex	0.880	0.063
M	IL-6	right Dorsal nucleus of the endopiriform	0.748	0.043
M	IL-6	right Dorsal tenia tecta	0.475	0.031
M	IL-6	right Dorsolateral entorhinal cortex	1.365	0.036
M	IL-6	right Dorsolateral orbital cortex	0.401	0.024
M	IL-6	right Ectorhinal cortex	1.447	0.095
M	IL-6	right facial nerve (cranial nerve 7)	0.114	0.004
M	IL-6	right fasciculus retroflexus	0.122	0.005
M	IL-6	right fastigial nucleus	0.240	0.010
M	IL-6	right fimbria	1.621	0.065
M	IL-6	right flocculus (FL)	0.542	0.027
M	IL-6	right flocculus white matter	0.039	0.003
M	IL-6	right fornix	0.315	0.013
M	IL-6	right Frontal association cortex	4.008	0.218
M	IL-6	right Frontal cortex: area 3	0.366	0.024
M	IL-6	right fundus of striatum	0.086	0.006
M	IL-6	right globus pallidus	1.699	0.060
M	IL-6	right GrDG	0.685	0.032
M	IL-6	right habenular commissure	0.015	0.002
M	IL-6	right hypothalamus	5.442	0.173
M	IL-6	right inferior olivary complex	0.185	0.019
M	IL-6	right Insular region: not subdivided	4.027	0.196
M	IL-6	right Intermediate nucleus of the endopiriform claustrum	0.297	0.019
M	IL-6	right internal capsule	1.588	0.044
M	IL-6	right lateral olfactory tract	0.680	0.023
M	IL-6	right Lateral orbital cortex	1.851	0.084
M	IL-6	right Lateral parietal association cortex	0.091	0.006
M	IL-6	right lateral septum	1.587	0.051
M	IL-6	right lateral ventricle	1.994	0.282
M	IL-6	right LMol	1.232	0.052
M	IL-6	right mammillary bodies	0.227	0.013
M	IL-6	right mammilothalamic tract	0.136	0.006
M	IL-6	right Medial amygdala	0.598	0.041
M	IL-6	right Medial entorhinal cortex	0.343	0.022
M	IL-6	right medial lemniscus/medial longitudinal fasciculus	1.369	0.048
M	IL-6	right Medial orbital cortex	0.865	0.063
M	IL-6	right Medial parietal association cortex	0.179	0.008
M	IL-6	right Medial preoptic nucleus	0.098	0.007
M	IL-6	right medial septum	0.640	0.026
M	IL-6	right MoDG	2.176	0.088
M	IL-6	right nucleus accumbens	2.153	0.079
M	IL-6	right nucleus interpositus	0.245	0.011
M	IL-6	right Olfactory bulb: external plexiform layer	3.445	0.094
M	IL-6	right Olfactory bulb: glomerular layer	2.368	0.048
M	IL-6	right Olfactory bulb: granule cell layer	2.383	0.079
M	IL-6	right Olfactory bulb: internal plexiform layer	0.528	0.019
M	IL-6	right Olfactory bulb: mitral cell layer	0.635	0.023
M	IL-6	right olfactory peduncle	1.183	0.042
M	IL-6	right olfactory tubercle	1.689	0.054
M	IL-6	right optic tract	0.748	0.021
M	IL-6	right paraflocculus (PFL)	1.512	0.301
M	IL-6	right paraflocculus white matter	0.147	0.027
M	IL-6	right paramedian lobule	0.088	0.008
M	IL-6	right paramedian lobule (lobule 7)	2.184	0.114
M	IL-6	right Parietal cortex: posterior area: rostral part	0.045	0.005
M	IL-6	right Perirhinal cortex	1.251	0.066
M	IL-6	right Piriform cortex	5.498	0.275
M	IL-6	right PoDG	0.341	0.016
M	IL-6	right pontine nucleus	0.400	0.019
M	IL-6	right Posterolateral cortical amygdaloid area	0.451	0.036
M	IL-6	right Posteromedial cortical amygdaloid area	0.492	0.041
M	IL-6	right pre-para subiculum	1.232	0.073
M	IL-6	right Primary auditory cortex	0.767	0.054
M	IL-6	right Primary motor cortex	3.319	0.158
M	IL-6	right Primary somatosensory cortex	2.082	0.095
M	IL-6	right Primary somatosensory cortex: barrel field	4.790	0.178
M	IL-6	right Primary somatosensory cortex: dysgranular zone	0.179	0.014
M	IL-6	right Primary somatosensory cortex: forelimb region	1.872	0.111
M	IL-6	right Primary somatosensory cortex: hindlimb region	1.257	0.092
M	IL-6	right Primary somatosensory cortex: jaw region	0.286	0.026
M	IL-6	right Primary somatosensory cortex: shoulder region	0.067	0.010
M	IL-6	right Primary somatosensory cortex: trunk region	0.198	0.009
M	IL-6	right Primary somatosensory cortex: upper lip region	2.819	0.117
M	IL-6	right Primary visual cortex	1.314	0.081
M	IL-6	right Primary visual cortex: binocular area	1.060	0.078
M	IL-6	right Primary visual cortex: monocular area	0.819	0.054
M	IL-6	right Rostral amygdalopiriform area	0.195	0.013
M	IL-6	right Secondary auditory cortex: dorsal area	0.749	0.053
M	IL-6	right Secondary auditory cortex: ventral area	1.029	0.103
M	IL-6	right Secondary motor cortex	3.396	0.157
M	IL-6	right Secondary somatosensory cortex	3.587	0.252
M	IL-6	right Secondary visual cortex: lateral area	1.550	0.102
M	IL-6	right Secondary visual cortex: mediolateral area	0.417	0.027
M	IL-6	right Secondary visual cortex: mediomedial area	0.861	0.043
M	IL-6	right simple lobule (lobule 6)	2.582	0.105
M	IL-6	right simple lobule white matter	0.191	0.010
M	IL-6	right SLu	0.399	0.017
M	IL-6	right stria medullaris	0.365	0.013
M	IL-6	right stria terminalis	0.412	0.011
M	IL-6	right striatum	10.927	0.420
M	IL-6	right subependymale zone / rhinocele	0.027	0.002
M	IL-6	right subiculum	1.716	0.073
M	IL-6	right superior olivary complex	0.402	0.019
M	IL-6	right Temporal association area	1.630	0.111
M	IL-6	right thalamus	9.192	0.362
M	IL-6	right trunk of crus 2 and paramedian white matter	0.183	0.008
M	IL-6	right trunk of simple and crus 1 white matter	0.083	0.003
M	IL-6	right Ventral intermediate entorhinal cortex	0.538	0.037
M	IL-6	right Ventral nucleus of the endopiriform claustrum	0.245	0.024
M	IL-6	right Ventral orbital cortex	0.732	0.039
M	IL-6	right Ventral tenia tecta	0.061	0.005
M	IL-6	third ventricle	1.257	0.060
M	IL-6	trunk of arbor vita	4.295	0.112
M	IL-6	trunk of lobules 1-3 white matter	0.131	0.006
M	IL-6	trunk of lobules 6-8 white matter	0.106	0.007
M	IL-6	ventral tegmental decussation	0.136	0.006
M	IL-10	cerebral aqueduct	0.520	0.051
M	IL-10	fourth ventricle	0.967	0.078
M	IL-10	interpedunclar nucleus	0.281	0.012
M	IL-10	left Accessory olfactory bulb: glomerular, external plexiform and mitral cell layer	0.265	0.015
M	IL-10	left Accessory olfactory bulb: granule cell layer	0.132	0.007
M	IL-10	left amygdala	4.950	0.158
M	IL-10	left Amygdalopiriform transition area	0.546	0.018
M	IL-10	left anterior commissure: pars anterior	0.533	0.017
M	IL-10	left anterior commissure: pars posterior	0.220	0.011
M	IL-10	left anterior lobule (lobules 4-5)	0.921	0.047
M	IL-10	left anterior lobule white matter	0.045	0.003
M	IL-10	left Anterior olfactory nucleus	1.046	0.036
M	IL-10	left basal forebrain	2.667	0.089
M	IL-10	left bed nucleus of stria terminalis	0.716	0.030
M	IL-10	left CA10r	1.189	0.048
M	IL-10	left CA1Py	0.567	0.014
M	IL-10	left CA1Rad	1.267	0.022
M	IL-10	left CA20r	0.269	0.015
M	IL-10	left CA2Py	0.107	0.003
M	IL-10	left CA2Rad	0.218	0.006
M	IL-10	left CA30r	1.432	0.051
M	IL-10	left CA3Py Inner	0.082	0.003
M	IL-10	left CA3Py Outer	0.533	0.012
M	IL-10	left CA3Rad	0.948	0.033
M	IL-10	left Caudomedial entorhinal cortex	3.069	0.087
M	IL-10	left cerebellar peduncle: inferior	0.451	0.012
M	IL-10	left cerebellar peduncle: middle	0.603	0.015
M	IL-10	left cerebellar peduncle: superior	0.526	0.030
M	IL-10	left cerebral peduncle	1.056	0.043
M	IL-10	left Cingulate cortex: area 24a	0.953	0.046
M	IL-10	left Cingulate cortex: area 24a'	0.391	0.026
M	IL-10	left Cingulate cortex: area 24b	0.792	0.064
M	IL-10	left Cingulate cortex: area 24b'	0.321	0.020
M	IL-10	left Cingulate cortex: area 25	0.307	0.012
M	IL-10	left Cingulate cortex: area 29a	0.382	0.022
M	IL-10	left Cingulate cortex: area 29b	0.217	0.012
M	IL-10	left Cingulate cortex: area 29c	0.969	0.064
M	IL-10	left Cingulate cortex: area 30	1.481	0.071
M	IL-10	left Cingulate cortex: area 32	1.355	0.090
M	IL-10	left Cingulum	0.437	0.016
M	IL-10	left Claustrum	0.173	0.008
M	IL-10	left Claustrum: dorsal part	0.145	0.020
M	IL-10	left Claustrum: ventral part	0.273	0.033
M	IL-10	left colliculus: inferior	2.893	0.083
M	IL-10	left colliculus: superior	4.472	0.141
M	IL-10	left copula white matter	0.044	0.003
M	IL-10	left copula: pyramis (lobule 8)	1.139	0.076
M	IL-10	left corpus callosum	6.277	0.208
M	IL-10	left Cortex-amygdala transition zones	0.394	0.037
M	IL-10	left corticospinal tract/pyramids	0.898	0.050
M	IL-10	left crus 1 white matter	0.201	0.009
M	IL-10	left crus 1: ansiform lobule (lobule 6)	2.266	0.123
M	IL-10	left crus 2 white matter	0.139	0.009
M	IL-10	left crus 2: ansiform lobule (lobule 7)	2.062	0.105
M	IL-10	left cuneate nucleus	0.123	0.012
M	IL-10	left dentate nucleus	0.190	0.010
M	IL-10	left Dorsal intermediate entorhinal cortex	0.868	0.034
M	IL-10	left Dorsal nucleus of the endopiriform	0.732	0.029
M	IL-10	left Dorsal tenia tecta	0.515	0.026
M	IL-10	left Dorsolateral entorhinal cortex	1.196	0.056
M	IL-10	left Dorsolateral orbital cortex	0.430	0.024
M	IL-10	left Ectorhinal cortex	1.297	0.097
M	IL-10	left facial nerve (cranial nerve 7)	0.111	0.004
M	IL-10	left fasciculus retroflexus	0.133	0.005
M	IL-10	left fastigial nucleus	0.254	0.014
M	IL-10	left fimbria	1.668	0.058
M	IL-10	left flocculus (FL)	0.548	0.034
M	IL-10	left flocculus white matter	0.030	0.002
M	IL-10	left fornix	0.346	0.012
M	IL-10	left Frontal association cortex	3.313	0.123
M	IL-10	left Frontal cortex: area 3	0.346	0.018
M	IL-10	left fundus of striatum	0.080	0.007
M	IL-10	left globus pallidus	1.648	0.066
M	IL-10	left GrDG	0.709	0.021
M	IL-10	left habenular commissure	0.015	0.003
M	IL-10	left hypothalamus	5.390	0.131
M	IL-10	left inferior olivary complex	0.168	0.020
M	IL-10	left Insular region: not subdivided	3.712	0.251
M	IL-10	left Intermediate nucleus of the endopiriform claustrum	0.320	0.010
M	IL-10	left internal capsule	1.477	0.049
M	IL-10	left lateral olfactory tract	0.777	0.029
M	IL-10	left Lateral orbital cortex	1.816	0.064
M	IL-10	left Lateral parietal association cortex	0.155	0.009
M	IL-10	left lateral septum	1.557	0.052
M	IL-10	left lateral ventricle	1.772	0.175
M	IL-10	left LMol	1.215	0.018
M	IL-10	left mammillary bodies	0.247	0.021
M	IL-10	left mammilothalamic tract	0.126	0.005

M	IL-10	left Medial amygdala	0.541	0.029
M	IL-10	left Medial entorhinal cortex	0.374	0.019
M	IL-10	left medial lemniscus/medial longitudinal fasciculus	1.282	0.041
M	IL-10	left Medial orbital cortex	0.914	0.044
M	IL-10	left Medial parietal association cortex	0.241	0.023
M	IL-10	left Medial preoptic nucleus	0.103	0.007
M	IL-10	left medial septum	0.669	0.029
M	IL-10	left MoDG	2.259	0.045
M	IL-10	left nucleus accumbens	2.047	0.052
M	IL-10	left nucleus interpositus	0.234	0.013
M	IL-10	left Olfactory bulb: external plexiform layer	3.388	0.097
M	IL-10	left Olfactory bulb: glomerular layer	2.261	0.094
M	IL-10	left Olfactory bulb: granule cell layer	2.455	0.088
M	IL-10	left Olfactory bulb: internal plexiform layer	0.550	0.019
M	IL-10	left Olfactory bulb: mitral cell layer	0.686	0.024
M	IL-10	left olfactory peduncle	1.240	0.041
M	IL-10	left olfactory tubercle	1.675	0.082
M	IL-10	left optic tract	0.788	0.018
M	IL-10	left paraflocculus (PFL)	1.789	0.189
M	IL-10	left paraflocculus white matter	0.149	0.010
M	IL-10	left paramedian lobule	0.079	0.006
M	IL-10	left paramedian lobule (lobule 7)	2.108	0.087
M	IL-10	left Parietal cortex: posterior area: rostral part	0.071	0.005
M	IL-10	left Perirhinal cortex	1.240	0.065
M	IL-10	left Piriform cortex	5.313	0.201
M	IL-10	left PoDG	0.318	0.011
M	IL-10	left pontine nucleus	0.407	0.046
M	IL-10	left Posterolateral cortical amygdaloid area	0.435	0.022
M	IL-10	left Posteromedial cortical amygdaloid area	0.629	0.027
M	IL-10	left pre-para subiculum	1.163	0.041
M	IL-10	left Primary auditory cortex	0.728	0.041
M	IL-10	left Primary motor cortex	3.558	0.126
M	IL-10	left Primary somatosensory cortex	2.011	0.120
M	IL-10	left Primary somatosensory cortex: barrel field	4.667	0.166
M	IL-10	left Primary somatosensory cortex: dysgranular zone	0.177	0.014
M	IL-10	left Primary somatosensory cortex: forelimb region	2.094	0.124
M	IL-10	left Primary somatosensory cortex: hindlimb region	1.477	0.078
M	IL-10	left Primary somatosensory cortex: jaw region	0.265	0.037
M	IL-10	left Primary somatosensory cortex: shoulder region	0.093	0.007
M	IL-10	left Primary somatosensory cortex: trunk region	0.315	0.016
M	IL-10	left Primary somatosensory cortex: upper lip region	2.364	0.086
M	IL-10	left Primary visual cortex	1.209	0.075
M	IL-10	left Primary visual cortex: binocular area	1.014	0.059
M	IL-10	left Primary visual cortex: monocular area	1.187	0.072
M	IL-10	left Rostral amygdalopiriform area	0.202	0.016
M	IL-10	left Secondary auditory cortex: dorsal area	0.703	0.023
M	IL-10	left Secondary auditory cortex: ventral area	0.771	0.073
M	IL-10	left Secondary motor cortex	3.227	0.126
M	IL-10	left Secondary somatosensory cortex	3.306	0.279
M	IL-10	left Secondary visual cortex: lateral area	1.299	0.068
M	IL-10	left Secondary visual cortex: mediolateral area	0.594	0.032
M	IL-10	left Secondary visual cortex: mediomedial area	0.988	0.066
M	IL-10	left simple lobule (lobule 6)	2.511	0.111
M	IL-10	left simple lobule white matter	0.236	0.013
M	IL-10	left SLu	0.389	0.012
M	IL-10	left stria medullaris	0.331	0.010
M	IL-10	left stria terminalis	0.478	0.016
M	IL-10	left striatum	10.872	0.416
M	IL-10	left subependymale zone / rhinocele	0.031	0.002
M	IL-10	left subiculum	1.707	0.041
M	IL-10	left superior olivary complex	0.399	0.037
M	IL-10	left Temporal association area	1.351	0.097
M	IL-10	left thalamus	8.749	0.261
M	IL-10	left trunk of crus 2 and paramedian white matter	0.165	0.012
M	IL-10	left trunk of simple and crus 1 white matter	0.064	0.003
M	IL-10	left Ventral intermediate entorhinal cortex	0.573	0.017
M	IL-10	left Ventral nucleus of the endopiriform claustrum	0.257	0.019
M	IL-10	left Ventral orbital cortex	0.797	0.039
M	IL-10	left Ventral tenia tecta	0.063	0.005
M	IL-10	lobule 1-2 white matter	0.073	0.011
M	IL-10	lobule 10 white matter	0.083	0.008
M	IL-10	lobule 10: nodulus	1.396	0.066
M	IL-10	lobule 3 white matter	0.194	0.020
M	IL-10	lobule 3: central lobule (dorsal)	2.258	0.240
M	IL-10	lobule 6: declive	2.506	0.152
M	IL-10	lobule 7: tuber (or folium)	1.008	0.058
M	IL-10	lobule 8 white matter	0.164	0.009
M	IL-10	lobule 8: pyramis	1.660	0.104
M	IL-10	lobule 9 white matter	0.298	0.018
M	IL-10	lobule 9: uvula	2.895	0.157
M	IL-10	lobules 1-2: lingula and central lobule (ventral)	1.686	0.157
M	IL-10	lobules 4-5 white matter	0.582	0.033
M	IL-10	lobules 4-5: culmen (ventral and dorsal)	4.477	0.201
M	IL-10	lobules 6-7 white matter	0.675	0.041
M	IL-10	medulla	28.244	0.612
M	IL-10	midbrain	14.122	0.459
M	IL-10	periaqueductal grey	3.951	0.123
M	IL-10	pons	17.746	0.389
M	IL-10	posterior commissure	0.146	0.007
M	IL-10	right Accessory olfactory bulb: glomerular, external plexiform and mitral cell layer	0.251	0.015
M	IL-10	right Accessory olfactory bulb: granule cell layer	0.126	0.007
M	IL-10	right amygdala	4.647	0.134
M	IL-10	right Amygdalopiriform transition area	0.494	0.023
M	IL-10	right anterior commissure: pars anterior	0.524	0.020
M	IL-10	right anterior commissure: pars posterior	0.211	0.010
M	IL-10	right anterior lobule (lobules 4-5)	0.814	0.055
M	IL-10	right anterior lobule white matter	0.053	0.005
M	IL-10	right Anterior olfactory nucleus	1.066	0.045
M	IL-10	right basal forebrain	2.763	0.116
M	IL-10	right bed nucleus of stria terminalis	0.713	0.031
M	IL-10	right CA10r	1.043	0.044
M	IL-10	right CA1Py	0.570	0.022
M	IL-10	right CA1Rad	1.368	0.028
M	IL-10	right CA20r	0.219	0.012
M	IL-10	right CA2Py	0.101	0.005
M	IL-10	right CA2Rad	0.227	0.009
M	IL-10	right CA30r	1.455	0.054
M	IL-10	right CA3Py Inner	0.083	0.004
M	IL-10	right CA3Py Outer	0.538	0.015
M	IL-10	right CA3Rad	0.997	0.032
M	IL-10	right Caudomedial entorhinal cortex	2.938	0.093
M	IL-10	right cerebellar peduncle: inferior	0.408	0.011
M	IL-10	right cerebellar peduncle: middle	0.632	0.033
M	IL-10	right cerebellar peduncle: superior	0.503	0.023
M	IL-10	right cerebral peduncle	1.067	0.033
M	IL-10	right Cingulate cortex: area 24a	1.015	0.056
M	IL-10	right Cingulate cortex: area 24a'	0.364	0.027
M	IL-10	right Cingulate cortex: area 24b	0.757	0.064
M	IL-10	right Cingulate cortex: area 24b'	0.296	0.024
M	IL-10	right Cingulate cortex: area 25	0.319	0.014
M	IL-10	right Cingulate cortex: area 29a	0.369	0.017
M	IL-10	right Cingulate cortex: area 29b	0.206	0.016
M	IL-10	right Cingulate cortex: area 29c	0.916	0.057
M	IL-10	right Cingulate cortex: area 30	1.392	0.061
M	IL-10	right Cingulate cortex: area 32	1.069	0.072
M	IL-10	right Cingulum	0.453	0.018
M	IL-10	right Claustrum	0.148	0.008
M	IL-10	right Claustrum: dorsal part	0.136	0.017
M	IL-10	right Claustrum: ventral part	0.306	0.030
M	IL-10	right colliculus: inferior	2.752	0.095
M	IL-10	right colliculus: superior	4.535	0.136
M	IL-10	right copula white matter	0.034	0.003
M	IL-10	right copula: pyramis (lobule 8)	1.252	0.093
M	IL-10	right corpus callosum	5.983	0.184
M	IL-10	right Cortex-amygdala transition zones	0.432	0.018
M	IL-10	right corticospinal tract/pyramids	0.936	0.052
M	IL-10	right crus 1 white matter	0.192	0.009
M	IL-10	right crus 1: ansiform lobule (lobule 6)	2.274	0.113
M	IL-10	right crus 2 white matter	0.156	0.011
M	IL-10	right crus 2: ansiform lobule (lobule 7)	2.100	0.130
M	IL-10	right cuneate nucleus	0.127	0.012
M	IL-10	right dentate nucleus	0.186	0.009
M	IL-10	right Dorsal intermediate entorhinal cortex	0.887	0.052
M	IL-10	right Dorsal nucleus of the endopiriform	0.732	0.025
M	IL-10	right Dorsal tenia tecta	0.474	0.031
M	IL-10	right Dorsolateral entorhinal cortex	1.367	0.065
M	IL-10	right Dorsolateral orbital cortex	0.401	0.026
M	IL-10	right Ectorhinal cortex	1.392	0.104
M	IL-10	right facial nerve (cranial nerve 7)	0.114	0.004
M	IL-10	right fasciculus retroflexus	0.118	0.006
M	IL-10	right fastigial nucleus	0.243	0.015
M	IL-10	right fimbria	1.558	0.048
M	IL-10	right flocculus (FL)	0.522	0.026
M	IL-10	right flocculus white matter	0.040	0.004
M	IL-10	right fornix	0.305	0.012
M	IL-10	right Frontal association cortex	3.640	0.133
M	IL-10	right Frontal cortex: area 3	0.352	0.020
M	IL-10	right fundus of striatum	0.088	0.007
M	IL-10	right globus pallidus	1.685	0.070
M	IL-10	right GrDG	0.670	0.027
M	IL-10	right habenular commissure	0.014	0.003
M	IL-10	right hypothalamus	5.336	0.134
M	IL-10	right inferior olivary complex	0.181	0.021
M	IL-10	right Insular region: not subdivided	4.046	0.322
M	IL-10	right Intermediate nucleus of the endopiriform claustrum	0.298	0.013
M	IL-10	right internal capsule	1.556	0.052
M	IL-10	right lateral olfactory tract	0.701	0.028
M	IL-10	right Lateral orbital cortex	1.780	0.067
M	IL-10	right Lateral parietal association cortex	0.089	0.008
M	IL-10	right lateral septum	1.556	0.058
M	IL-10	right lateral ventricle	1.957	0.271
M	IL-10	right LMol	1.199	0.016
M	IL-10	right mammillary bodies	0.233	0.020
M	IL-10	right mammilothalamic tract	0.132	0.009
M	IL-10	right Medial amygdala	0.583	0.035
M	IL-10	right Medial entorhinal cortex	0.360	0.015
M	IL-10	right medial lemniscus/medial longitudinal fasciculus	1.377	0.065
M	IL-10	right Medial orbital cortex	0.820	0.033
M	IL-10	right Medial parietal association cortex	0.173	0.016
M	IL-10	right Medial preoptic nucleus	0.093	0.004
M	IL-10	right medial septum	0.634	0.030
M	IL-10	right MoDG	2.108	0.057
M	IL-10	right nucleus accumbens	2.034	0.063
M	IL-10	right nucleus interpositus	0.244	0.015
M	IL-10	right Olfactory bulb: external plexiform layer	3.397	0.101
M	IL-10	right Olfactory bulb: glomerular layer	2.343	0.098
M	IL-10	right Olfactory bulb: granule cell layer	2.523	0.092
M	IL-10	right Olfactory bulb: internal plexiform layer	0.541	0.014
M	IL-10	right Olfactory bulb: mitral cell layer	0.645	0.015
M	IL-10	right olfactory peduncle	1.200	0.044
M	IL-10	right olfactory tubercle	1.692	0.065
M	IL-10	right optic tract	0.745	0.034
M	IL-10	right paraflocculus (PFL)	1.738	0.185
M	IL-10	right paraflocculus white matter	0.168	0.016
M	IL-10	right paramedian lobule	0.087	0.007
M	IL-10	right paramedian lobule (lobule 7)	2.117	0.097
M	IL-10	right Parietal cortex: posterior area: rostral part	0.044	0.003
M	IL-10	right Perirhinal cortex	1.231	0.065
M	IL-10	right Piriform cortex	5.670	0.218
M	IL-10	right PoDG	0.335	0.015
M	IL-10	right pontine nucleus	0.398	0.044
M	IL-10	right Posterolateral cortical amygdaloid area	0.466	0.033
M	IL-10	right Posteromedial cortical amygdaloid area	0.488	0.025
M	IL-10	right pre-para subiculum	1.179	0.045
M	IL-10	right Primary auditory cortex	0.739	0.041
M	IL-10	right Primary motor cortex	3.101	0.091
M	IL-10	right Primary somatosensory cortex	2.105	0.114
M	IL-10	right Primary somatosensory cortex: barrel field	4.640	0.144
M	IL-10	right Primary somatosensory cortex: dysgranular zone	0.193	0.015
M	IL-10	right Primary somatosensory cortex: forelimb region	1.853	0.103
M	IL-10	right Primary somatosensory cortex: hindlimb region	1.220	0.080
M	IL-10	right Primary somatosensory cortex: jaw region	0.314	0.028
M	IL-10	right Primary somatosensory cortex: shoulder region	0.069	0.009
M	IL-10	right Primary somatosensory cortex: trunk region	0.199	0.017
M	IL-10	right Primary somatosensory cortex: upper lip region	2.847	0.153
M	IL-10	right Primary visual cortex	1.189	0.055
M	IL-10	right Primary visual cortex: binocular area	1.029	0.043
M	IL-10	right Primary visual cortex: monocular area	0.776	0.050
M	IL-10	right Rostral amygdalopiriform area	0.201	0.012
M	IL-10	right Secondary auditory cortex: dorsal area	0.709	0.047
M	IL-10	right Secondary auditory cortex: ventral area	0.987	0.082
M	IL-10	right Secondary motor cortex	3.117	0.116
M	IL-10	right Secondary somatosensory cortex	3.629	0.355
M	IL-10	right Secondary visual cortex: lateral area	1.454	0.074
M	IL-10	right Secondary visual cortex: mediolateral area	0.398	0.027
M	IL-10	right Secondary visual cortex: mediomedial area	0.821	0.044
M	IL-10	right simple lobule (lobule 6)	2.541	0.129
M	IL-10	right simple lobule white matter	0.194	0.013
M	IL-10	right SLu	0.390	0.013
M	IL-10	right stria medullaris	0.354	0.014
M	IL-10	right stria terminalis	0.405	0.014
M	IL-10	right striatum	10.832	0.512
M	IL-10	right subependymale zone / rhinocele	0.029	0.001
M	IL-10	right subiculum	1.675	0.050
M	IL-10	right superior olivary complex	0.410	0.037
M	IL-10	right Temporal association area	1.545	0.107
M	IL-10	right thalamus	8.671	0.342
M	IL-10	right trunk of crus 2 and paramedian white matter	0.173	0.013
M	IL-10	right trunk of simple and crus 1 white matter	0.079	0.005
M	IL-10	right Ventral intermediate entorhinal cortex	0.563	0.029
M	IL-10	right Ventral nucleus of the endopiriform claustrum	0.236	0.022
M	IL-10	right Ventral orbital cortex	0.725	0.043
M	IL-10	right Ventral tenia tecta	0.064	0.005
M	IL-10	third ventricle	1.259	0.087
M	IL-10	trunk of arbor vita	4.266	0.125
M	IL-10	trunk of lobules 1-3 white matter	0.128	0.009
M	IL-10	trunk of lobules 6-8 white matter	0.118	0.009
M	IL-10	ventral tegmental decussation	0.135	0.009
M	IL-18	cerebral aqueduct	0.498	0.020
M	IL-18	fourth ventricle	0.956	0.042
M	IL-18	interpeduncLar nucleus	0.279	0.013
M	IL-18	left Accessory olfactory bulb: glomerular, external plexiform and mitral cell layer	0.265	0.013
M	IL-18	left Accessory olfactory bulb: granule cell layer	0.134	0.007
M	IL-18	left amygdala	4.981	0.109
M	IL-18	left Amygdalopiriform transition area	0.540	0.027
M	IL-18	left anterior commissure: pars anterior	0.516	0.021
M	IL-18	left anterior commissure: pars posterior	0.225	0.008
M	IL-18	left anterior lobule (lobules 4-5)	0.981	0.051
M	IL-18	left anterior lobule white matter	0.048	0.003
M	IL-18	left Anterior olfactory nucleus	1.002	0.042
M	IL-18	left basal forebrain	2.664	0.083
M	IL-18	left bed nucleus of stria terminalis	0.705	0.026
M	IL-18	left CA10r	1.260	0.042
M	IL-18	left CA1Py	0.601	0.020
M	IL-18	left CA1Rad	1.311	0.030
M	IL-18	left CA20r	0.279	0.011
M	IL-18	left CA2Py	0.110	0.004

M	IL-18	left CA2Rad	0.216	0.010
M	IL-18	left CA30r	1.490	0.042
M	IL-18	left CA3Py Inner	0.082	0.003
M	IL-18	left CA3Py Outer	0.552	0.018
M	IL-18	left CA3Rad	0.972	0.035
M	IL-18	left Caudomedial entorhinal cortex	3.026	0.095
M	IL-18	left cerebellar peduncle: inferior	0.451	0.019
M	IL-18	left cerebellar peduncle: middle	0.612	0.029
M	IL-18	left cerebellar peduncle: superior	0.531	0.025
M	IL-18	left cerebral peduncle	1.066	0.024
M	IL-18	left Cingulate cortex: area 24a	0.946	0.059
M	IL-18	left Cingulate cortex: area 24a'	0.386	0.036
M	IL-18	left Cingulate cortex: area 24b	0.823	0.078
M	IL-18	left Cingulate cortex: area 24b'	0.313	0.022
M	IL-18	left Cingulate cortex: area 25	0.294	0.012
M	IL-18	left Cingulate cortex: area 29a	0.406	0.019
M	IL-18	left Cingulate cortex: area 29b	0.228	0.016
M	IL-18	left Cingulate cortex: area 29c	1.000	0.067
M	IL-18	left Cingulate cortex: area 30	1.513	0.080
M	IL-18	left Cingulate cortex: area 32	1.345	0.063
M	IL-18	left Cingulum	0.451	0.028
M	IL-18	left Claustrum	0.178	0.012
M	IL-18	left Claustrum: dorsal part	0.145	0.017
M	IL-18	left Claustrum: ventral part	0.273	0.026
M	IL-18	left colliculus: inferior	3.028	0.106
M	IL-18	left colliculus: superior	4.674	0.191
M	IL-18	left copula white matter	0.050	0.002
M	IL-18	left copula: pyramis (lobule 8)	1.294	0.075
M	IL-18	left corpus callosum	6.369	0.263
M	IL-18	left Cortex-amygdala transition zones	0.396	0.035
M	IL-18	left corticospinal tract/pyramids	0.877	0.028
M	IL-18	left crus 1 white matter	0.207	0.007
M	IL-18	left crus 1: ansiform lobule (lobule 6)	2.371	0.055
M	IL-18	left crus 2 white matter	0.138	0.007
M	IL-18	left crus 2: ansiform lobule (lobule 7)	2.133	0.072
M	IL-18	left cuneate nucleus	0.128	0.007
M	IL-18	left dentate nucleus	0.194	0.012
M	IL-18	left Dorsal intermediate entorhinal cortex	0.849	0.030
M	IL-18	left Dorsal nucleus of the endopiriform	0.747	0.024
M	IL-18	left Dorsal tenia tecta	0.488	0.024
M	IL-18	left Dorsolateral entorhinal cortex	1.259	0.062
M	IL-18	left Dorsolateral orbital cortex	0.439	0.029
M	IL-18	left Ectorhinal cortex	1.358	0.068
M	IL-18	left facial nerve (cranial nerve 7)	0.110	0.005
M	IL-18	left fasciculus retroflexus	0.135	0.008
M	IL-18	left fastigial nucleus	0.266	0.016
M	IL-18	left fimbria	1.729	0.073
M	IL-18	left flocculus (FL)	0.594	0.042
M	IL-18	left flocculus white matter	0.032	0.003
M	IL-18	left fornix	0.345	0.015
M	IL-18	left Frontal association cortex	3.587	0.176
M	IL-18	left Frontal cortex: area 3	0.355	0.011
M	IL-18	left fundus of striatum	0.081	0.005
M	IL-18	left globus pallidus	1.721	0.052
M	IL-18	left GrDG	0.716	0.022
M	IL-18	left habenular commissure	0.014	0.001
M	IL-18	left hypothalamus	5.366	0.119
M	IL-18	left inferior olivary complex	0.169	0.015
M	IL-18	left Insular region: not subdivided	3.742	0.188
M	IL-18	left Intermediate nucleus of the endopiriform claustrum	0.316	0.011
M	IL-18	left internal capsule	1.507	0.062
M	IL-18	left lateral olfactory tract	0.749	0.018
M	IL-18	left Lateral orbital cortex	1.838	0.070
M	IL-18	left Lateral parietal association cortex	0.154	0.012
M	IL-18	left lateral septum	1.614	0.062
M	IL-18	left lateral ventricle	1.826	0.157
M	IL-18	left LMol	1.237	0.026
M	IL-18	left mammillary bodies	0.232	0.010
M	IL-18	left mammilothalamic tract	0.128	0.007
M	IL-18	left Medial amygdala	0.541	0.020
M	IL-18	left Medial entorhinal cortex	0.365	0.018
M	IL-18	left medial lemniscus/medial longitudinal fasciculus	1.278	0.024
M	IL-18	left Medial orbital cortex	0.911	0.026
M	IL-18	left Medial parietal association cortex	0.251	0.020
M	IL-18	left Medial preoptic nucleus	0.103	0.004
M	IL-18	left medial septum	0.663	0.024
M	IL-18	left MoDG	2.299	0.048
M	IL-18	left nucleus accumbens	2.182	0.084
M	IL-18	left nucleus interpositus	0.247	0.017
M	IL-18	left Olfactory bulb: external plexiform layer	3.432	0.115
M	IL-18	left Olfactory bulb: glomerular layer	2.315	0.048
M	IL-18	left Olfactory bulb: granule cell layer	2.361	0.075
M	IL-18	left Olfactory bulb: internal plexiform layer	0.540	0.024
M	IL-18	left Olfactory bulb: mitral cell layer	0.679	0.028
M	IL-18	left olfactory peduncle	1.175	0.045
M	IL-18	left olfactory tubercle	1.653	0.054
M	IL-18	left optic tract	0.814	0.030
M	IL-18	left paraflocculus (PFL)	1.900	0.278
M	IL-18	left paraflocculus white matter	0.156	0.016
M	IL-18	left paramedian lobule	0.081	0.005
M	IL-18	left paramedian lobule (lobule 7)	2.185	0.098
M	IL-18	left Parietal cortex: posterior area: rostral part	0.073	0.005
M	IL-18	left Perirhinal cortex	1.285	0.048
M	IL-18	left Piriform cortex	5.116	0.179
M	IL-18	left PoDG	0.317	0.012
M	IL-18	left pontine nucleus	0.395	0.019
M	IL-18	left Posterolateral cortical amygdaloid area	0.414	0.022
M	IL-18	left Posteromedial cortical amygdaloid area	0.632	0.047
M	IL-18	left pre-para subiculum	1.190	0.039
M	IL-18	left Primary auditory cortex	0.759	0.051
M	IL-18	left Primary motor cortex	3.672	0.137
M	IL-18	left Primary somatosensory cortex	1.999	0.103
M	IL-18	left Primary somatosensory cortex: barrel field	4.675	0.210
M	IL-18	left Primary somatosensory cortex: dysgranular zone	0.165	0.012
M	IL-18	left Primary somatosensory cortex: forelimb region	2.020	0.112
M	IL-18	left Primary somatosensory cortex: hindlimb region	1.445	0.063
M	IL-18	left Primary somatosensory cortex: jaw region	0.264	0.028
M	IL-18	left Primary somatosensory cortex: shoulder region	0.091	0.011
M	IL-18	left Primary somatosensory cortex: trunk region	0.307	0.015
M	IL-18	left Primary somatosensory cortex: upper lip region	2.358	0.105
M	IL-18	left Primary visual cortex	1.176	0.088
M	IL-18	left Primary visual cortex: binocular area	1.022	0.063
M	IL-18	left Primary visual cortex: monocular area	1.184	0.096
M	IL-18	left Rostral amygdalopiriform area	0.188	0.010
M	IL-18	left Secondary auditory cortex: dorsal area	0.742	0.052
M	IL-18	left Secondary auditory cortex: ventral area	0.804	0.086
M	IL-18	left Secondary motor cortex	3.427	0.161
M	IL-18	left Secondary somatosensory cortex	3.340	0.224
M	IL-18	left Secondary visual cortex: lateral area	1.348	0.075
M	IL-18	left Secondary visual cortex: mediolateral area	0.597	0.045
M	IL-18	left Secondary visual cortex: mediomedial area	0.975	0.068
M	IL-18	left simple lobule (lobule 6)	2.553	0.111
M	IL-18	left simple lobule white matter	0.233	0.017
M	IL-18	left SLu	0.405	0.015
M	IL-18	left stria medullaris	0.335	0.010
M	IL-18	left stria terminalis	0.487	0.022
M	IL-18	left striatum	11.376	0.385
M	IL-18	left subependymale zone / rhinocele	0.030	0.002
M	IL-18	left subiculum	1.736	0.047
M	IL-18	left superior olivary complex	0.387	0.017
M	IL-18	left Temporal association area	1.429	0.076
M	IL-18	left thalamus	9.184	0.322
M	IL-18	left trunk of crus 2 and paramedian white matter	0.172	0.016
M	IL-18	left trunk of simple and crus 1 white matter	0.074	0.004
M	IL-18	left Ventral intermediate entorhinal cortex	0.575	0.019
M	IL-18	left Ventral nucleus of the endopiriform claustrum	0.259	0.021
M	IL-18	left Ventral orbital cortex	0.790	0.038
M	IL-18	left Ventral tenia tecta	0.054	0.004
M	IL-18	lobule 1-2 white matter	0.076	0.014
M	IL-18	lobule 10 white matter	0.093	0.004
M	IL-18	lobule 10: nodulus	1.526	0.058
M	IL-18	lobule 3 white matter	0.217	0.032
M	IL-18	lobule 3: central lobule (dorsal)	2.482	0.248
M	IL-18	lobule 6: declive	2.573	0.098
M	IL-18	lobule 7: tuber (or folium)	1.020	0.054
M	IL-18	lobule 8 white matter	0.172	0.008
M	IL-18	lobule 8: pyramis	1.759	0.067
M	IL-18	lobule 9 white matter	0.317	0.011
M	IL-18	lobule 9: uvula	3.027	0.112
M	IL-18	lobules 1-2: lingula and central lobule (ventral)	1.830	0.214
M	IL-18	lobules 4-5 white matter	0.635	0.034
M	IL-18	lobules 4-5: culmen (ventral and dorsal)	4.747	0.209
M	IL-18	lobules 6-7 white matter	0.708	0.024
M	IL-18	medulla	28.597	0.655
M	IL-18	midbrain	14.437	0.531
M	IL-18	periaqueductal grey	4.135	0.150
M	IL-18	pons	17.892	0.465
M	IL-18	posterior commissure	0.147	0.008
M	IL-18	right Accessory olfactory bulb: glomerular, external plexiform and mitral cell layer	0.256	0.011
M	IL-18	right Accessory olfactory bulb: granule cell layer	0.127	0.008
M	IL-18	right amygdala	4.633	0.155
M	IL-18	right Amygdalopiriform transition area	0.497	0.024
M	IL-18	right anterior commissure: pars anterior	0.506	0.017
M	IL-18	right anterior commissure: pars posterior	0.216	0.009
M	IL-18	right anterior lobule (lobules 4-5)	0.889	0.033
M	IL-18	right anterior lobule white matter	0.059	0.002
M	IL-18	right Anterior olfactory nucleus	1.030	0.044
M	IL-18	right basal forebrain	2.745	0.096
M	IL-18	right bed nucleus of stria terminalis	0.703	0.020
M	IL-18	right CA10r	1.114	0.035
M	IL-18	right CA1Py	0.607	0.018
M	IL-18	right CA1Rad	1.420	0.044
M	IL-18	right CA20r	0.228	0.011
M	IL-18	right CA2Py	0.102	0.004
M	IL-18	right CA2Rad	0.223	0.009
M	IL-18	right CA30r	1.488	0.054
M	IL-18	right CA3Py Inner	0.084	0.005
M	IL-18	right CA3Py Outer	0.548	0.020
M	IL-18	right CA3Rad	1.005	0.037
M	IL-18	right Caudomedial entorhinal cortex	2.932	0.109
M	IL-18	right cerebellar peduncle: inferior	0.421	0.010
M	IL-18	right cerebellar peduncle: middle	0.644	0.031
M	IL-18	right cerebellar peduncle: superior	0.513	0.026
M	IL-18	right cerebral peduncle	1.079	0.028
M	IL-18	right Cingulate cortex: area 24a	1.010	0.068
M	IL-18	right Cingulate cortex: area 24a'	0.363	0.034
M	IL-18	right Cingulate cortex: area 24b	0.786	0.075
M	IL-18	right Cingulate cortex: area 24b'	0.299	0.020
M	IL-18	right Cingulate cortex: area 25	0.303	0.014
M	IL-18	right Cingulate cortex: area 29a	0.387	0.021
M	IL-18	right Cingulate cortex: area 29b	0.217	0.012
M	IL-18	right Cingulate cortex: area 29c	0.940	0.069
M	IL-18	right Cingulate cortex: area 30	1.432	0.056
M	IL-18	right Cingulate cortex: area 32	1.075	0.086
M	IL-18	right Cingulum	0.463	0.027
M	IL-18	right Claustrum	0.149	0.008
M	IL-18	right Claustrum: dorsal part	0.139	0.014
M	IL-18	right Claustrum: ventral part	0.309	0.026
M	IL-18	right colliculus: inferior	2.884	0.088
M	IL-18	right colliculus: superior	4.731	0.186
M	IL-18	right copula white matter	0.041	0.002
M	IL-18	right copula: pyramis (lobule 8)	1.451	0.107
M	IL-18	right corpus callosum	6.103	0.214
M	IL-18	right Cortex-amygdala transition zones	0.422	0.035
M	IL-18	right corticospinal tract/pyramids	0.920	0.040
M	IL-18	right crus 1 white matter	0.190	0.008
M	IL-18	right crus 1: ansiform lobule (lobule 6)	2.345	0.083
M	IL-18	right crus 2 white matter	0.156	0.009
M	IL-18	right crus 2: ansiform lobule (lobule 7)	2.130	0.069
M	IL-18	right cuneate nucleus	0.134	0.008
M	IL-18	right dentate nucleus	0.190	0.009
M	IL-18	right Dorsal intermediate entorhinal cortex	0.895	0.056
M	IL-18	right Dorsal nucleus of the endopiriform	0.718	0.035
M	IL-18	right Dorsal tenia tecta	0.449	0.023
M	IL-18	right Dorsolateral entorhinal cortex	1.409	0.092
M	IL-18	right Dorsolateral orbital cortex	0.409	0.019
M	IL-18	right Ectorhinal cortex	1.399	0.086
M	IL-18	right facial nerve (cranial nerve 7)	0.113	0.005
M	IL-18	right fasciculus retroflexus	0.121	0.006
M	IL-18	right fastigial nucleus	0.256	0.013
M	IL-18	right fimbria	1.608	0.078
M	IL-18	right flocculus (FL)	0.547	0.024
M	IL-18	right flocculus white matter	0.039	0.003
M	IL-18	right fornix	0.303	0.010
M	IL-18	right Frontal association cortex	3.974	0.165
M	IL-18	right Frontal cortex: area 3	0.356	0.022
M	IL-18	right fundus of striatum	0.084	0.006
M	IL-18	right globus pallidus	1.744	0.050
M	IL-18	right GrDG	0.679	0.023
M	IL-18	right habenular commissure	0.014	0.001
M	IL-18	right hypothalamus	5.311	0.129
M	IL-18	right inferior olivary complex	0.185	0.016
M	IL-18	right Insular region: not subdivided	3.991	0.229
M	IL-18	right Intermediate nucleus of the endopiriform claustrum	0.289	0.014
M	IL-18	right internal capsule	1.579	0.058
M	IL-18	right lateral olfactory tract	0.675	0.028
M	IL-18	right Lateral orbital cortex	1.829	0.055
M	IL-18	right Lateral parietal association cortex	0.098	0.006
M	IL-18	right lateral septum	1.625	0.051
M	IL-18	right lateral ventricle	1.997	0.179
M	IL-18	right LMol	1.209	0.038
M	IL-18	right mammillary bodies	0.217	0.008
M	IL-18	right mammilothalamic tract	0.135	0.007
M	IL-18	right Medial amygdala	0.577	0.032
M	IL-18	right Medial entorhinal cortex	0.346	0.012
M	IL-18	right medial lemniscus/medial longitudinal fasciculus	1.356	0.044
M	IL-18	right Medial orbital cortex	0.812	0.036
M	IL-18	right Medial parietal association cortex	0.187	0.008
M	IL-18	right Medial preoptic nucleus	0.093	0.005
M	IL-18	right medial septum	0.622	0.023
M	IL-18	right MoDG	2.155	0.062
M	IL-18	right nucleus accumbens	2.172	0.071
M	IL-18	right nucleus interpositus	0.262	0.016
M	IL-18	right Olfactory bulb: external plexiform layer	3.435	0.102
M	IL-18	right Olfactory bulb: glomerular layer	2.403	0.061
M	IL-18	right Olfactory bulb: granule cell layer	2.422	0.077
M	IL-18	right Olfactory bulb: internal plexiform layer	0.529	0.023
M	IL-18	right Olfactory bulb: mitral cell layer	0.637	0.023
M	IL-18	right olfactory peduncle	1.150	0.030
M	IL-18	right olfactory tubercle	1.706	0.085
M	IL-18	right optic tract	0.761	0.032
M	IL-18	right paraflocculus (PFL)	1.783	0.207
M	IL-18	right paraflocculus white matter	0.172	0.020
M	IL-18	right paramedian lobule	0.091	0.006
M	IL-18	right paramedian lobule (lobule 7)	2.181	0.090
M	IL-18	right Parietal cortex: posterior area: rostral part	0.047	0.005
M	IL-18	right Perirhinal cortex	1.227	0.056

M	IL-18	right Piriform cortex	5.344	0.180
M	IL-18	right PoDG	0.335	0.012
M	IL-18	right pontine nucleus	0.387	0.020
M	IL-18	right Posterolateral cortical amygdaloid area	0.450	0.039
M	IL-18	right Posteromedial cortical amygdaloid area	0.471	0.026
M	IL-18	right pre-para subiculum	1.209	0.032
M	IL-18	right Primary auditory cortex	0.743	0.037
M	IL-18	right Primary motor cortex	3.200	0.139
M	IL-18	right Primary somatosensory cortex	2.044	0.123
M	IL-18	right Primary somatosensory cortex: barrel field	4.566	0.234
M	IL-18	right Primary somatosensory cortex: dysgranular zone	0.181	0.011
M	IL-18	right Primary somatosensory cortex: forelimb region	1.819	0.085
M	IL-18	right Primary somatosensory cortex: hindlimb region	1.225	0.063
M	IL-18	right Primary somatosensory cortex: jaw region	0.268	0.021
M	IL-18	right Primary somatosensory cortex: shoulder region	0.068	0.007
M	IL-18	right Primary somatosensory cortex: trunk region	0.214	0.013
M	IL-18	right Primary somatosensory cortex: upper lip region	2.754	0.136
M	IL-18	right Primary visual cortex	1.193	0.096
M	IL-18	right Primary visual cortex: binocular area	1.030	0.077
M	IL-18	right Primary visual cortex: monocular area	0.784	0.038
M	IL-18	right Rostral amygdalopiriform area	0.202	0.016
M	IL-18	right Secondary auditory cortex: dorsal area	0.698	0.048
M	IL-18	right Secondary auditory cortex: ventral area	0.994	0.066
M	IL-18	right Secondary motor cortex	3.328	0.110
M	IL-18	right Secondary somatosensory cortex	3.570	0.235
M	IL-18	right Secondary visual cortex: lateral area	1.465	0.110
M	IL-18	right Secondary visual cortex: mediolateral area	0.407	0.027
M	IL-18	right Secondary visual cortex: mediomedial area	0.834	0.045
M	IL-18	right simple lobule (lobule 6)	2.534	0.070
M	IL-18	right simple lobule white matter	0.191	0.008
M	IL-18	right SLu	0.401	0.014
M	IL-18	right stria medullaris	0.357	0.010
M	IL-18	right stria terminalis	0.402	0.016
M	IL-18	right striatum	11.283	0.338
M	IL-18	right subependymale zone / rhinocele	0.026	0.001
M	IL-18	right subiculum	1.714	0.057
M	IL-18	right superior olivary complex	0.408	0.020
M	IL-18	right Temporal association area	1.595	0.099
M	IL-18	right thalamus	9.100	0.289
M	IL-18	right trunk of crus 2 and paramedian white matter	0.186	0.011
M	IL-18	right trunk of simple and crus 1 white matter	0.087	0.004
M	IL-18	right Ventral intermediate entorhinal cortex	0.558	0.032
M	IL-18	right Ventral nucleus of the endopiriform claustrum	0.234	0.020
M	IL-18	right Ventral orbital cortex	0.717	0.036
M	IL-18	right Ventral tenia tecta	0.057	0.003
M	IL-18	third ventricle	1.233	0.037
M	IL-18	trunk of arbor vita	4.491	0.158
M	IL-18	trunk of lobules 1-3 white matter	0.140	0.015
M	IL-18	trunk of lobules 6-8 white matter	0.111	0.005
M	IL-18	ventral tegmental decussation	0.137	0.009
M	Kit	cerebral aqueduct	0.515	0.060
M	Kit	fourth ventricle	0.968	0.050
M	Kit	interpeduncular nucleus	0.279	0.009
M	Kit	left Accessory olfactory bulb: glomerular, external plexiform and mitral cell layer	0.266	0.010
M	Kit	left Accessory olfactory bulb: granule cell layer	0.120	0.005
M	Kit	left amygdala	5.047	0.258
M	Kit	left Amygdalopiriform transition area	0.528	0.040
M	Kit	left anterior commissure: pars anterior	0.480	0.019
M	Kit	left anterior commissure: pars posterior	0.209	0.011
M	Kit	left anterior lobule (lobules 4-5)	0.858	0.053
M	Kit	left anterior lobule white matter	0.042	0.003
M	Kit	left Anterior olfactory nucleus	0.997	0.038
M	Kit	left basal forebrain	2.618	0.080
M	Kit	left bed nucleus of stria terminalis	0.701	0.033
M	Kit	left CA10r	1.144	0.057
M	Kit	left CA1Py	0.546	0.023
M	Kit	left CA1Rad	1.221	0.051
M	Kit	left CA20r	0.254	0.011
M	Kit	left CA2Py	0.102	0.007
M	Kit	left CA2Rad	0.202	0.011
M	Kit	left CA30r	1.471	0.105
M	Kit	left CA3Py Inner	0.080	0.006
M	Kit	left CA3Py Outer	0.526	0.034
M	Kit	left CA3Rad	0.929	0.053
M	Kit	left Caudomedial entorhinal cortex	2.993	0.121
M	Kit	left cerebellar peduncle: inferior	0.415	0.019
M	Kit	left cerebellar peduncle: middle	0.536	0.024
M	Kit	left cerebellar peduncle: superior	0.519	0.025
M	Kit	left cerebral peduncle	1.052	0.031
M	Kit	left Cingulate cortex: area 24a	0.967	0.061
M	Kit	left Cingulate cortex: area 24a'	0.352	0.022
M	Kit	left Cingulate cortex: area 24b	0.860	0.082
M	Kit	left Cingulate cortex: area 24b'	0.299	0.027
M	Kit	left Cingulate cortex: area 25	0.320	0.018
M	Kit	left Cingulate cortex: area 29a	0.364	0.020
M	Kit	left Cingulate cortex: area 29b	0.215	0.014
M	Kit	left Cingulate cortex: area 29c	0.926	0.060
M	Kit	left Cingulate cortex: area 30	1.452	0.105
M	Kit	left Cingulate cortex: area 32	1.347	0.076
M	Kit	left Cingulum	0.440	0.026
M	Kit	left Claustrum	0.176	0.014
M	Kit	left Claustrum: dorsal part	0.155	0.022
M	Kit	left Claustrum: ventral part	0.277	0.030
M	Kit	left colliculus: inferior	2.851	0.086
M	Kit	left colliculus: superior	4.390	0.217
M	Kit	left copula white matter	0.044	0.003
M	Kit	left copula: pyramis (lobule 8)	1.145	0.074
M	Kit	left corpus callosum	5.890	0.275
M	Kit	left Cortex-amygdala transition zones	0.411	0.030
M	Kit	left corticospinal tract/pyramids	0.882	0.040
M	Kit	left crus 1 white matter	0.195	0.011
M	Kit	left crus 1: ansiform lobule (lobule 6)	2.231	0.146
M	Kit	left crus 2 white matter	0.126	0.010
M	Kit	left crus 2: ansiform lobule (lobule 7)	1.984	0.176
M	Kit	left cuneate nucleus	0.118	0.011
M	Kit	left dentate nucleus	0.185	0.008
M	Kit	left Dorsal intermediate entorhinal cortex	0.841	0.068
M	Kit	left Dorsal nucleus of the endopiriform	0.755	0.050
M	Kit	left Dorsal tenia tecta	0.473	0.025
M	Kit	left Dorsolateral entorhinal cortex	1.214	0.104
M	Kit	left Dorsolateral orbital cortex	0.436	0.026
M	Kit	left Ectorhinal cortex	1.353	0.103
M	Kit	left facial nerve (cranial nerve 7)	0.104	0.004
M	Kit	left fasciculus retroflexus	0.122	0.007
M	Kit	left fastigial nucleus	0.244	0.012
M	Kit	left fimbria	1.694	0.110
M	Kit	left flocculus (FL)	0.553	0.032
M	Kit	left flocculus white matter	0.028	0.003
M	Kit	left fornix	0.330	0.022
M	Kit	left Frontal association cortex	3.532	0.099
M	Kit	left Frontal cortex: area 3	0.358	0.021
M	Kit	left fundus of striatum	0.076	0.005
M	Kit	left globus pallidus	1.603	0.075
M	Kit	left GrDG	0.656	0.034
M	Kit	left habenular commissure	0.016	0.002
M	Kit	left hypothalamus	5.330	0.143
M	Kit	left inferior olivary complex	0.180	0.028
M	Kit	left Insular region: not subdivided	3.923	0.309
M	Kit	left Intermediate nucleus of the endopiriform claustrum	0.311	0.016
M	Kit	left internal capsule	1.398	0.079
M	Kit	left lateral olfactory tract	0.743	0.018
M	Kit	left Lateral orbital cortex	1.793	0.083
M	Kit	left Lateral parietal association cortex	0.145	0.015
M	Kit	left lateral septum	1.600	0.081
M	Kit	left lateral ventricle	2.229	0.435
M	Kit	left LMol	1.171	0.044
M	Kit	left mammillary bodies	0.230	0.013
M	Kit	left mammilothalamic tract	0.115	0.005
M	Kit	left Medial amygdala	0.535	0.027
M	Kit	left Medial entorhinal cortex	0.364	0.025
M	Kit	left medial lemniscus/medial longitudinal fasciculus	1.251	0.035
M	Kit	left Medial orbital cortex	0.895	0.053
M	Kit	left Medial parietal association cortex	0.232	0.024
M	Kit	left Medial preoptic nucleus	0.102	0.007
M	Kit	left medial septum	0.657	0.017
M	Kit	left MoDG	2.121	0.076
M	Kit	left nucleus accumbens	2.138	0.062
M	Kit	left nucleus interpositus	0.236	0.012
M	Kit	left Olfactory bulb: external plexiform layer	3.142	0.111
M	Kit	left Olfactory bulb: glomerular layer	2.152	0.085
M	Kit	left Olfactory bulb: granule cell layer	2.255	0.114
M	Kit	left Olfactory bulb: internal plexiform layer	0.501	0.023
M	Kit	left Olfactory bulb: mitral cell layer	0.631	0.023
M	Kit	left olfactory peduncle	1.142	0.045
M	Kit	left olfactory tubercle	1.675	0.080
M	Kit	left optic tract	0.778	0.034
M	Kit	left paraflocculus (PFL)	1.745	0.208
M	Kit	left paraflocculus white matter	0.141	0.012
M	Kit	left paramedian lobule	0.073	0.005
M	Kit	left paramedian lobule (lobule 7)	2.029	0.112
M	Kit	left Parietal cortex: posterior area: rostral part	0.070	0.009
M	Kit	left Perirhinal cortex	1.250	0.060
M	Kit	left Piriform cortex	5.295	0.374
M	Kit	left PoDG	0.289	0.016
M	Kit	left pontine nucleus	0.369	0.031
M	Kit	left Posterolateral cortical amygdaloid area	0.423	0.027
M	Kit	left Posteromedial cortical amygdaloid area	0.621	0.040
M	Kit	left pre-para subiculum	1.111	0.038
M	Kit	left Primary auditory cortex	0.707	0.051
M	Kit	left Primary motor cortex	3.731	0.185
M	Kit	left Primary somatosensory cortex	1.960	0.130
M	Kit	left Primary somatosensory cortex: barrel field	4.631	0.164
M	Kit	left Primary somatosensory cortex: dysgranular zone	0.157	0.008
M	Kit	left Primary somatosensory cortex: forelimb region	2.016	0.145
M	Kit	left Primary somatosensory cortex: hindlimb region	1.458	0.101
M	Kit	left Primary somatosensory cortex: jaw region	0.246	0.020
M	Kit	left Primary somatosensory cortex: shoulder region	0.082	0.009
M	Kit	left Primary somatosensory cortex: trunk region	0.289	0.028
M	Kit	left Primary somatosensory cortex: upper lip region	2.333	0.130
M	Kit	left Primary visual cortex	1.194	0.103
M	Kit	left Primary visual cortex: binocular area	0.975	0.040
M	Kit	left Primary visual cortex: monocular area	1.128	0.041
M	Kit	left Rostral amygdalopiriform area	0.195	0.019
M	Kit	left Secondary auditory cortex: dorsal area	0.697	0.048
M	Kit	left Secondary auditory cortex: ventral area	0.740	0.081
M	Kit	left Secondary motor cortex	3.348	0.198
M	Kit	left Secondary somatosensory cortex	3.490	0.371
M	Kit	left Secondary visual cortex: lateral area	1.295	0.084
M	Kit	left Secondary visual cortex: mediolateral area	0.576	0.045
M	Kit	left Secondary visual cortex: mediomedial area	0.954	0.062
M	Kit	left simple lobule (lobule 6)	2.421	0.091
M	Kit	left simple lobule white matter	0.226	0.014
M	Kit	left SLu	0.373	0.025
M	Kit	left stria medullaris	0.319	0.011
M	Kit	left stria terminalis	0.466	0.031
M	Kit	left striatum	10.716	0.584
M	Kit	left subependymale zone / rhinocele	0.029	0.002
M	Kit	left subiculum	1.640	0.063
M	Kit	left superior olivary complex	0.368	0.020
M	Kit	left Temporal association area	1.361	0.118
M	Kit	left thalamus	8.603	0.418
M	Kit	left trunk of crus 2 and paramedian white matter	0.162	0.009
M	Kit	left trunk of simple and crus 1 white matter	0.066	0.004
M	Kit	left Ventral intermediate entorhinal cortex	0.559	0.037
M	Kit	left Ventral nucleus of the endopiriform claustrum	0.251	0.016
M	Kit	left Ventral orbital cortex	0.723	0.035
M	Kit	left Ventral tenia tecta	0.057	0.005
M	Kit	lobule 1-2 white matter	0.069	0.018
M	Kit	lobule 10 white matter	0.086	0.005
M	Kit	lobule 10: nodulus	1.448	0.073
M	Kit	lobule 3 white matter	0.181	0.039
M	Kit	lobule 3: central lobule (dorsal)	2.134	0.197
M	Kit	lobule 6: declive	2.446	0.146
M	Kit	lobule 7: tuber (or folium)	0.917	0.077
M	Kit	lobule 8 white matter	0.152	0.009
M	Kit	lobule 8: pyramis	1.520	0.094
M	Kit	lobule 9 white matter	0.285	0.019
M	Kit	lobule 9: uvula	2.763	0.150
M	Kit	lobules 1-2: lingula and central lobule (ventral)	1.717	0.200
M	Kit	lobules 4-5 white matter	0.560	0.036
M	Kit	lobules 4-5: culmen (ventral and dorsal)	4.283	0.247
M	Kit	lobules 6-7 white matter	0.657	0.041
M	Kit	medulla	27.857	0.787
M	Kit	midbrain	14.046	0.544
M	Kit	periaqueductal grey	3.963	0.134
M	Kit	pons	17.011	0.409
M	Kit	posterior commissure	0.141	0.008
M	Kit	right Accessory olfactory bulb: glomerular, external plexiform and mitral cell layer	0.262	0.011
M	Kit	right Accessory olfactory bulb: granule cell layer	0.113	0.007
M	Kit	right amygdala	4.709	0.269
M	Kit	right Amygdalopiriform transition area	0.496	0.029
M	Kit	right anterior commissure: pars anterior	0.479	0.020
M	Kit	right anterior commissure: pars posterior	0.201	0.011
M	Kit	right anterior lobule (lobules 4-5)	0.784	0.060
M	Kit	right anterior lobule white matter	0.051	0.005
M	Kit	right Anterior olfactory nucleus	1.042	0.036
M	Kit	right basal forebrain	2.728	0.054
M	Kit	right bed nucleus of stria terminalis	0.683	0.033
M	Kit	right CA10r	0.999	0.055
M	Kit	right CA1Py	0.551	0.030
M	Kit	right CA1Rad	1.337	0.052
M	Kit	right CA20r	0.206	0.012
M	Kit	right CA2Py	0.100	0.009
M	Kit	right CA2Rad	0.220	0.020
M	Kit	right CA30r	1.515	0.139
M	Kit	right CA3Py Inner	0.083	0.008
M	Kit	right CA3Py Outer	0.554	0.048
M	Kit	right CA3Rad	0.987	0.071
M	Kit	right Caudomedial entorhinal cortex	2.884	0.113
M	Kit	right cerebellar peduncle: inferior	0.397	0.017
M	Kit	right cerebellar peduncle: middle	0.572	0.024
M	Kit	right cerebellar peduncle: superior	0.507	0.029
M	Kit	right cerebral peduncle	1.058	0.033
M	Kit	right Cingulate cortex: area 24a	1.046	0.074
M	Kit	right Cingulate cortex: area 24a'	0.333	0.022
M	Kit	right Cingulate cortex: area 24b	0.823	0.095
M	Kit	right Cingulate cortex: area 24b'	0.290	0.026
M	Kit	right Cingulate cortex: area 25	0.326	0.021
M	Kit	right Cingulate cortex: area 29a	0.349	0.025
M	Kit	right Cingulate cortex: area 29b	0.204	0.015
M	Kit	right Cingulate cortex: area 29c	0.882	0.042
M	Kit	right Cingulate cortex: area 30	1.348	0.094
M	Kit	right Cingulate cortex: area 32	1.100	0.062
M	Kit	right Cingulum	0.452	0.026
M	Kit	right Claustrum	0.145	0.011
M	Kit	right Claustrum: dorsal part	0.151	0.026
M	Kit	right Claustrum: ventral part	0.335	0.044

M	Kit	right colliculus: inferior	2.724	0.099
M	Kit	right colliculus: superior	4.512	0.133
M	Kit	right copula white matter	0.034	0.003
M	Kit	right copula: pyramis (lobule 8)	1.268	0.085
M	Kit	right corpus callosum	5.746	0.257
M	Kit	right Cortex-amygdala transition zones	0.476	0.043
M	Kit	right corticospinal tract/pyramids	0.930	0.045
M	Kit	right crus 1 white matter	0.191	0.009
M	Kit	right crus 1: ansiform lobule (lobule 6)	2.252	0.122
M	Kit	right crus 2 white matter	0.144	0.008
M	Kit	right crus 2: ansiform lobule (lobule 7)	1.955	0.101
M	Kit	right cuneate nucleus	0.124	0.012
M	Kit	right dentate nucleus	0.181	0.010
M	Kit	right Dorsal intermediate entorhinal cortex	0.898	0.056
M	Kit	right Dorsal nucleus of the endopiriform	0.759	0.049
M	Kit	right Dorsal tenia tecta	0.447	0.029
M	Kit	right Dorsolateral entorhinal cortex	1.374	0.107
M	Kit	right Dorsolateral orbital cortex	0.391	0.023
M	Kit	right Ectorhinal cortex	1.419	0.148
M	Kit	right facial nerve (cranial nerve 7)	0.109	0.004
M	Kit	right fasciculus retroflexus	0.107	0.006
M	Kit	right fastigial nucleus	0.229	0.015
M	Kit	right fimbria	1.585	0.093
M	Kit	right flocculus (FL)	0.517	0.029
M	Kit	right flocculus white matter	0.039	0.002
M	Kit	right fornix	0.292	0.014
M	Kit	right Frontal association cortex	3.918	0.171
M	Kit	right Frontal cortex: area 3	0.353	0.018
M	Kit	right fundus of striatum	0.085	0.004
M	Kit	right globus pallidus	1.627	0.083
M	Kit	right GrDG	0.629	0.027
M	Kit	right habenular commissure	0.015	0.002
M	Kit	right hypothalamus	5.321	0.109
M	Kit	right inferior olivary complex	0.198	0.025
M	Kit	right Insular region: not subdivided	4.116	0.349
M	Kit	right Intermediate nucleus of the endopiriform claustrum	0.285	0.014
M	Kit	right internal capsule	1.478	0.079
M	Kit	right lateral olfactory tract	0.645	0.020
M	Kit	right Lateral orbital cortex	1.762	0.061
M	Kit	right Lateral parietal association cortex	0.083	0.007
M	Kit	right lateral septum	1.565	0.084
M	Kit	right lateral ventricle	2.487	0.481
M	Kit	right LMol	1.167	0.046
M	Kit	right mammillary bodies	0.214	0.013
M	Kit	right mammilothalamic tract	0.121	0.007
M	Kit	right Medial amygdala	0.589	0.032
M	Kit	right Medial entorhinal cortex	0.344	0.018
M	Kit	right medial lemniscus/medial longitudinal fasciculus	1.343	0.032
M	Kit	right Medial orbital cortex	0.830	0.047
M	Kit	right Medial parietal association cortex	0.163	0.014
M	Kit	right Medial preoptic nucleus	0.094	0.004
M	Kit	right medial septum	0.611	0.019
M	Kit	right MoDG	2.021	0.066
M	Kit	right nucleus accumbens	2.118	0.070
M	Kit	right nucleus interpositus	0.240	0.013
M	Kit	right Olfactory bulb: external plexiform layer	3.170	0.115
M	Kit	right Olfactory bulb: glomerular layer	2.200	0.063
M	Kit	right Olfactory bulb: granule cell layer	2.315	0.080
M	Kit	right Olfactory bulb: internal plexiform layer	0.500	0.024
M	Kit	right Olfactory bulb: mitral cell layer	0.599	0.028
M	Kit	right olfactory peduncle	1.116	0.041
M	Kit	right olfactory tubercle	1.675	0.082
M	Kit	right optic tract	0.730	0.024
M	Kit	right paraflocculus (PFL)	1.702	0.149
M	Kit	right paraflocculus white matter	0.162	0.011
M	Kit	right paramedian lobule	0.077	0.006
M	Kit	right paramedian lobule (lobule 7)	2.067	0.136
M	Kit	right Parietal cortex: posterior area: rostral part	0.040	0.004
M	Kit	right Perirhinal cortex	1.224	0.077
M	Kit	right Piriform cortex	5.553	0.359
M	Kit	right PoDG	0.307	0.015
M	Kit	right pontine nucleus	0.356	0.025
M	Kit	right Posterolateral cortical amygdaloid area	0.454	0.041
M	Kit	right Posteromedial cortical amygdaloid area	0.477	0.041
M	Kit	right pre-para subiculum	1.161	0.047
M	Kit	right Primary auditory cortex	0.720	0.063
M	Kit	right Primary motor cortex	3.238	0.179
M	Kit	right Primary somatosensory cortex	2.052	0.124
M	Kit	right Primary somatosensory cortex: barrel field	4.615	0.194
M	Kit	right Primary somatosensory cortex: dysgranular zone	0.163	0.011
M	Kit	right Primary somatosensory cortex: forelimb region	1.828	0.143
M	Kit	right Primary somatosensory cortex: hindlimb region	1.200	0.102
M	Kit	right Primary somatosensory cortex: jaw region	0.266	0.015
M	Kit	right Primary somatosensory cortex: shoulder region	0.058	0.014
M	Kit	right Primary somatosensory cortex: trunk region	0.182	0.016
M	Kit	right Primary somatosensory cortex: upper lip region	2.678	0.120
M	Kit	right Primary visual cortex	1.210	0.080
M	Kit	right Primary visual cortex: binocular area	0.991	0.077
M	Kit	right Primary visual cortex: monocular area	0.761	0.033
M	Kit	right Rostral amygdalopiriform area	0.201	0.017
M	Kit	right Secondary auditory cortex: dorsal area	0.677	0.050
M	Kit	right Secondary auditory cortex: ventral area	0.980	0.113
M	Kit	right Secondary motor cortex	3.321	0.247
M	Kit	right Secondary somatosensory cortex	3.584	0.437
M	Kit	right Secondary visual cortex: lateral area	1.405	0.089
M	Kit	right Secondary visual cortex: mediolateral area	0.379	0.028
M	Kit	right Secondary visual cortex: mediomedial area	0.782	0.034
M	Kit	right simple lobule (lobule 6)	2.477	0.096
M	Kit	right simple lobule white matter	0.186	0.011
M	Kit	right SLu	0.379	0.023
M	Kit	right stria medullaris	0.342	0.014
M	Kit	right stria terminalis	0.394	0.023
M	Kit	right striatum	10.762	0.621
M	Kit	right subependymale zone / rhinocele	0.027	0.002
M	Kit	right subiculum	1.614	0.060
M	Kit	right superior olivary complex	0.386	0.026
M	Kit	right Temporal association area	1.551	0.183
M	Kit	right thalamus	8.511	0.387
M	Kit	right trunk of crus 2 and paramedian white matter	0.173	0.013
M	Kit	right trunk of simple and crus 1 white matter	0.079	0.005
M	Kit	right Ventral intermediate entorhinal cortex	0.545	0.034
M	Kit	right Ventral nucleus of the endopiriform claustrum	0.246	0.028
M	Kit	right Ventral orbital cortex	0.671	0.037
M	Kit	right Ventral tenia tecta	0.057	0.005
M	Kit	third ventricle	1.295	0.062
M	Kit	trunk of arbor vita	4.073	0.149
M	Kit	trunk of lobules 1-3 white matter	0.123	0.014
M	Kit	trunk of lobules 6-8 white matter	0.112	0.006
M	Kit	ventral tegmental decussation	0.124	0.007
M	Nos2	cerebral aqueduct	0.497	0.038
M	Nos2	fourth ventricle	0.977	0.041
M	Nos2	interpeduncular nucleus	0.271	0.021
M	Nos2	left Accessory olfactory bulb: glomerular, external plexiform and mitral cell layer	0.266	0.007
M	Nos2	left Accessory olfactory bulb: granule cell layer	0.132	0.005
M	Nos2	left amygdala	4.989	0.178
M	Nos2	left Amygdalopiriform transition area	0.554	0.036
M	Nos2	left anterior commissure: pars anterior	0.500	0.018
M	Nos2	left anterior commissure: pars posterior	0.214	0.011
M	Nos2	left anterior lobule (lobules 4-5)	0.871	0.057
M	Nos2	left anterior lobule white matter	0.042	0.004
M	Nos2	left Anterior olfactory nucleus	0.976	0.042
M	Nos2	left basal forebrain	2.628	0.093
M	Nos2	left bed nucleus of stria terminalis	0.713	0.035
M	Nos2	left CA10r	1.216	0.056
M	Nos2	left CA1Py	0.578	0.024
M	Nos2	left CA1Rad	1.286	0.044
M	Nos2	left CA20r	0.270	0.010
M	Nos2	left CA2Py	0.107	0.006
M	Nos2	left CA2Rad	0.213	0.005
M	Nos2	left CA30r	1.454	0.177
M	Nos2	left CA3Py Inner	0.079	0.006
M	Nos2	left CA3Py Outer	0.531	0.044
M	Nos2	left CA3Rad	0.951	0.056
M	Nos2	left Caudomedial entorhinal cortex	3.022	0.078
M	Nos2	left cerebellar peduncle: inferior	0.426	0.014
M	Nos2	left cerebellar peduncle: middle	0.580	0.024
M	Nos2	left cerebellar peduncle: superior	0.511	0.033
M	Nos2	left cerebral peduncle	1.027	0.036
M	Nos2	left Cingulate cortex: area 24a	0.961	0.057
M	Nos2	left Cingulate cortex: area 24a'	0.380	0.037
M	Nos2	left Cingulate cortex: area 24b	0.854	0.073
M	Nos2	left Cingulate cortex: area 24b'	0.316	0.020
M	Nos2	left Cingulate cortex: area 25	0.303	0.031
M	Nos2	left Cingulate cortex: area 29a	0.382	0.020
M	Nos2	left Cingulate cortex: area 29b	0.220	0.016
M	Nos2	left Cingulate cortex: area 29c	0.969	0.034
M	Nos2	left Cingulate cortex: area 30	1.487	0.075
M	Nos2	left Cingulate cortex: area 32	1.395	0.089
M	Nos2	left Cingulum	0.439	0.028
M	Nos2	left Claustrum	0.179	0.015
M	Nos2	left Claustrum: dorsal part	0.155	0.018
M	Nos2	left Claustrum: ventral part	0.287	0.032
M	Nos2	left colliculus: inferior	2.918	0.149
M	Nos2	left colliculus: superior	4.411	0.207
M	Nos2	left copula white matter	0.043	0.003
M	Nos2	left copula: pyramis (lobule 8)	1.102	0.064
M	Nos2	left corpus callosum	6.203	0.218
M	Nos2	left Cortex-amygdala transition zones	0.389	0.014
M	Nos2	left corticospinal tract/pyramids	0.859	0.034
M	Nos2	left crus 1 white matter	0.193	0.006
M	Nos2	left crus 1: ansiform lobule (lobule 6)	2.199	0.076
M	Nos2	left crus 2 white matter	0.130	0.012
M	Nos2	left crus 2: ansiform lobule (lobule 7)	1.975	0.088
M	Nos2	left cuneate nucleus	0.122	0.008
M	Nos2	left dentate nucleus	0.180	0.014
M	Nos2	left Dorsal intermediate entorhinal cortex	0.888	0.040
M	Nos2	left Dorsal nucleus of the endopiriform	0.741	0.027
M	Nos2	left Dorsal tenia tecta	0.487	0.022
M	Nos2	left Dorsolateral entorhinal cortex	1.292	0.091
M	Nos2	left Dorsolateral orbital cortex	0.435	0.023
M	Nos2	left Ectorhinal cortex	1.414	0.108
M	Nos2	left facial nerve (cranial nerve 7)	0.105	0.007
M	Nos2	left fasciculus retroflexus	0.129	0.009
M	Nos2	left fastigial nucleus	0.247	0.017
M	Nos2	left fimbria	1.681	0.149
M	Nos2	left flocculus (FL)	0.562	0.033
M	Nos2	left flocculus white matter	0.029	0.003
M	Nos2	left fornix	0.341	0.014
M	Nos2	left Frontal association cortex	3.515	0.132
M	Nos2	left Frontal cortex: area 3	0.368	0.021
M	Nos2	left fundus of striatum	0.078	0.007
M	Nos2	left globus pallidus	1.661	0.060
M	Nos2	left GrDG	0.689	0.022
M	Nos2	left habenular commissure	0.014	0.001
M	Nos2	left hypothalamus	5.316	0.168
M	Nos2	left inferior olivary complex	0.160	0.015
M	Nos2	left Insular region: not subdivided	3.919	0.232
M	Nos2	left Intermediate nucleus of the endopiriform claustrum	0.323	0.013
M	Nos2	left internal capsule	1.460	0.053
M	Nos2	left lateral olfactory tract	0.771	0.030
M	Nos2	left Lateral orbital cortex	1.846	0.058
M	Nos2	left Lateral parietal association cortex	0.152	0.008
M	Nos2	left lateral septum	1.580	0.093
M	Nos2	left lateral ventricle	1.888	0.578
M	Nos2	left LMol	1.226	0.036
M	Nos2	left mammillary bodies	0.232	0.016
M	Nos2	left mammilothalamic tract	0.124	0.007
M	Nos2	left Medial amygdala	0.533	0.026
M	Nos2	left Medial entorhinal cortex	0.371	0.018
M	Nos2	left medial lemniscus/medial longitudinal fasciculus	1.251	0.044
M	Nos2	left Medial orbital cortex	0.920	0.048
M	Nos2	left Medial parietal association cortex	0.241	0.019
M	Nos2	left Medial preoptic nucleus	0.103	0.008
M	Nos2	left medial septum	0.655	0.030
M	Nos2	left MoDG	2.222	0.076
M	Nos2	left nucleus accumbens	2.175	0.069
M	Nos2	left nucleus interpositus	0.226	0.017
M	Nos2	left Olfactory bulb: external plexiform layer	3.297	0.116
M	Nos2	left Olfactory bulb: glomerular layer	2.240	0.075
M	Nos2	left Olfactory bulb: granule cell layer	2.401	0.103
M	Nos2	left Olfactory bulb: internal plexiform layer	0.543	0.018
M	Nos2	left Olfactory bulb: mitral cell layer	0.676	0.021
M	Nos2	left olfactory peduncle	1.193	0.042
M	Nos2	left olfactory tubercle	1.640	0.109
M	Nos2	left optic tract	0.794	0.033
M	Nos2	left paraflocculus (PFL)	1.851	0.201
M	Nos2	left paraflocculus white matter	0.147	0.017
M	Nos2	left paramedian lobule	0.072	0.007
M	Nos2	left paramedian lobule (lobule 7)	2.015	0.115
M	Nos2	left Parietal cortex: posterior area: rostral part	0.071	0.004
M	Nos2	left Perirhinal cortex	1.295	0.052
M	Nos2	left Piriform cortex	5.274	0.196
M	Nos2	left PoDG	0.307	0.009
M	Nos2	left pontine nucleus	0.381	0.017
M	Nos2	left Posterolateral cortical amygdaloid area	0.440	0.023
M	Nos2	left Posteromedial cortical amygdaloid area	0.637	0.064
M	Nos2	left pre-para subiculum	1.146	0.065
M	Nos2	left Primary auditory cortex	0.765	0.047
M	Nos2	left Primary motor cortex	3.702	0.135
M	Nos2	left Primary somatosensory cortex	2.031	0.074
M	Nos2	left Primary somatosensory cortex: barrel field	4.703	0.224
M	Nos2	left Primary somatosensory cortex: dysgranular zone	0.170	0.009
M	Nos2	left Primary somatosensory cortex: forelimb region	2.049	0.098
M	Nos2	left Primary somatosensory cortex: hindlimb region	1.465	0.108
M	Nos2	left Primary somatosensory cortex: jaw region	0.271	0.025
M	Nos2	left Primary somatosensory cortex: shoulder region	0.090	0.011
M	Nos2	left Primary somatosensory cortex: trunk region	0.305	0.018
M	Nos2	left Primary somatosensory cortex: upper lip region	2.386	0.124
M	Nos2	left Primary visual cortex	1.205	0.117
M	Nos2	left Primary visual cortex: binocular area	1.003	0.055
M	Nos2	left Primary visual cortex: monocular area	1.173	0.064
M	Nos2	left Rostral amygdalopiriform area	0.194	0.012
M	Nos2	left Secondary auditory cortex: dorsal area	0.713	0.060
M	Nos2	left Secondary auditory cortex: ventral area	0.830	0.066
M	Nos2	left Secondary motor cortex	3.419	0.120
M	Nos2	left Secondary somatosensory cortex	3.530	0.256
M	Nos2	left Secondary visual cortex: lateral area	1.339	0.112
M	Nos2	left Secondary visual cortex: mediolateral area	0.603	0.036
M	Nos2	left Secondary visual cortex: mediomedial area	0.983	0.048
M	Nos2	left simple lobule (lobule 6)	2.376	0.092
M	Nos2	left simple lobule white matter	0.216	0.009
M	Nos2	left SLu	0.384	0.014
M	Nos2	left stria medullaris	0.327	0.013
M	Nos2	left stria terminalis	0.477	0.020
M	Nos2	left striatum	10.980	0.422
M	Nos2	left subependymale zone / rhinocele	0.030	0.002
M	Nos2	left subiculum	1.705	0.066

M	Nos2	left superior olivary complex	0.372	0.020
M	Nos2	left Temporal association area	1.485	0.137
M	Nos2	left thalamus	9.010	0.343
M	Nos2	left trunk of crus 2 and paramedian white matter	0.158	0.010
M	Nos2	left trunk of simple and crus 1 white matter	0.068	0.004
M	Nos2	left Ventral intermediate entorhinal cortex	0.576	0.023
M	Nos2	left Ventral nucleus of the endopiriform claustrum	0.253	0.017
M	Nos2	left Ventral orbital cortex	0.774	0.035
M	Nos2	left Ventral tenia tecta	0.054	0.003
M	Nos2	lobule 1-2 white matter	0.059	0.017
M	Nos2	lobule 10 white matter	0.085	0.006
M	Nos2	lobule 10: nodulus	1.386	0.046
M	Nos2	lobule 3 white matter	0.173	0.037
M	Nos2	lobule 3: central lobule (dorsal)	2.109	0.209
M	Nos2	lobule 6: declive	2.412	0.090
M	Nos2	lobule 7: tuber (or folium)	0.923	0.049
M	Nos2	lobule 8 white matter	0.151	0.012
M	Nos2	lobule 8: pyramis	1.550	0.052
M	Nos2	lobule 9 white matter	0.280	0.014
M	Nos2	lobule 9: uvula	2.715	0.103
M	Nos2	lobules 1-2: lingula and central lobule (ventral)	1.563	0.221
M	Nos2	lobules 4-5 white matter	0.584	0.033
M	Nos2	lobules 4-5: culmen (ventral and dorsal)	4.436	0.227
M	Nos2	lobules 6-7 white matter	0.673	0.024
M	Nos2	medulla	27.474	1.050
M	Nos2	midbrain	13.866	0.729
M	Nos2	periaqueductal grey	3.975	0.210
M	Nos2	pons	17.120	0.769
M	Nos2	posterior commissure	0.143	0.009
M	Nos2	right Accessory olfactory bulb: glomerular, external plexiform and mitral cell layer	0.256	0.007
M	Nos2	right Accessory olfactory bulb: granule cell layer	0.128	0.006
M	Nos2	right amygdala	4.695	0.177
M	Nos2	right Amygdalopiriform transition area	0.507	0.021
M	Nos2	right anterior commissure: pars anterior	0.492	0.020
M	Nos2	right anterior commissure: pars posterior	0.205	0.011
M	Nos2	right anterior lobule (lobules 4-5)	0.786	0.043
M	Nos2	right anterior lobule white matter	0.051	0.004
M	Nos2	right Anterior olfactory nucleus	1.009	0.052
M	Nos2	right basal forebrain	2.735	0.123
M	Nos2	right bed nucleus of stria terminalis	0.706	0.029
M	Nos2	right CA10r	1.077	0.068
M	Nos2	right CA1Py	0.593	0.048
M	Nos2	right CA1Rad	1.413	0.097
M	Nos2	right CA20r	0.216	0.013
M	Nos2	right CA2Py	0.104	0.019
M	Nos2	right CA2Rad	0.236	0.061
M	Nos2	right CA30r	1.464	0.146
M	Nos2	right CA3Py Inner	0.083	0.018
M	Nos2	right CA3Py Outer	0.546	0.080
M	Nos2	right CA3Rad	1.022	0.150
M	Nos2	right Caudomedial entorhinal cortex	2.941	0.135
M	Nos2	right cerebellar peduncle: inferior	0.392	0.016
M	Nos2	right cerebellar peduncle: middle	0.622	0.024
M	Nos2	right cerebellar peduncle: superior	0.497	0.036
M	Nos2	right cerebral peduncle	1.035	0.034
M	Nos2	right Cingulate cortex: area 24a	1.028	0.063
M	Nos2	right Cingulate cortex: area 24a'	0.365	0.043
M	Nos2	right Cingulate cortex: area 24b	0.817	0.066
M	Nos2	right Cingulate cortex: area 24b'	0.305	0.024
M	Nos2	right Cingulate cortex: area 25	0.312	0.033
M	Nos2	right Cingulate cortex: area 29a	0.368	0.028
M	Nos2	right Cingulate cortex: area 29b	0.206	0.014
M	Nos2	right Cingulate cortex: area 29c	0.912	0.057
M	Nos2	right Cingulate cortex: area 30	1.394	0.077
M	Nos2	right Cingulate cortex: area 32	1.124	0.054
M	Nos2	right Cingulum	0.449	0.032
M	Nos2	right Claustrum	0.151	0.009
M	Nos2	right Claustrum: dorsal part	0.143	0.020
M	Nos2	right Claustrum: ventral part	0.318	0.038
M	Nos2	right colliculus: inferior	2.752	0.114
M	Nos2	right colliculus: superior	4.495	0.211
M	Nos2	right copula white matter	0.033	0.002
M	Nos2	right copula: pyramis (lobule 8)	1.247	0.075
M	Nos2	right corpus callosum	5.898	0.261
M	Nos2	right Cortex-amygdala transition zones	0.417	0.020
M	Nos2	right corticospinal tract/pyramids	0.918	0.046
M	Nos2	right crus 1 white matter	0.185	0.008
M	Nos2	right crus 1: ansiform lobule (lobule 6)	2.225	0.073
M	Nos2	right crus 2 white matter	0.152	0.010
M	Nos2	right crus 2: ansiform lobule (lobule 7)	2.033	0.082
M	Nos2	right cuneate nucleus	0.127	0.006
M	Nos2	right dentate nucleus	0.180	0.014
M	Nos2	right Dorsal intermediate entorhinal cortex	0.918	0.045
M	Nos2	right Dorsal nucleus of the endopiriform	0.726	0.035
M	Nos2	right Dorsal tenia tecta	0.444	0.020
M	Nos2	right Dorsolateral entorhinal cortex	1.428	0.086
M	Nos2	right Dorsolateral orbital cortex	0.398	0.023
M	Nos2	right Ectorhinal cortex	1.485	0.098
M	Nos2	right facial nerve (cranial nerve 7)	0.108	0.005
M	Nos2	right fasciculus retroflexus	0.115	0.007
M	Nos2	right fastigial nucleus	0.234	0.016
M	Nos2	right fimbria	1.539	0.088
M	Nos2	right flocculus (FL)	0.525	0.032
M	Nos2	right flocculus white matter	0.039	0.003
M	Nos2	right fornix	0.303	0.014
M	Nos2	right Frontal association cortex	3.848	0.171
M	Nos2	right Frontal cortex: area 3	0.356	0.018
M	Nos2	right fundus of striatum	0.087	0.007
M	Nos2	right globus pallidus	1.690	0.056
M	Nos2	right GrDG	0.664	0.048
M	Nos2	right habenular commissure	0.013	0.001
M	Nos2	right hypothalamus	5.292	0.169
M	Nos2	right inferior olivary complex	0.180	0.014
M	Nos2	right Insular region: not subdivided	4.137	0.262
M	Nos2	right Intermediate nucleus of the endopiriform claustrum	0.292	0.008
M	Nos2	right internal capsule	1.550	0.055
M	Nos2	right lateral olfactory tract	0.677	0.018
M	Nos2	right Lateral orbital cortex	1.779	0.060
M	Nos2	right Lateral parietal association cortex	0.091	0.007
M	Nos2	right lateral septum	1.568	0.088
M	Nos2	right lateral ventricle	1.965	0.480
M	Nos2	right LMol	1.232	0.092
M	Nos2	right mammillary bodies	0.216	0.013
M	Nos2	right mammilothalamic tract	0.130	0.008
M	Nos2	right Medial amygdala	0.573	0.036
M	Nos2	right Medial entorhinal cortex	0.348	0.022
M	Nos2	right medial lemniscus/medial longitudinal fasciculus	1.325	0.055
M	Nos2	right Medial orbital cortex	0.832	0.044
M	Nos2	right Medial parietal association cortex	0.173	0.015
M	Nos2	right Medial preoptic nucleus	0.092	0.006
M	Nos2	right medial septum	0.608	0.024
M	Nos2	right MoDG	2.122	0.135
M	Nos2	right nucleus accumbens	2.159	0.080
M	Nos2	right nucleus interpositus	0.236	0.018
M	Nos2	right Olfactory bulb: external plexiform layer	3.289	0.127
M	Nos2	right Olfactory bulb: glomerular layer	2.303	0.066
M	Nos2	right Olfactory bulb: granule cell layer	2.474	0.090
M	Nos2	right Olfactory bulb: internal plexiform layer	0.540	0.026
M	Nos2	right Olfactory bulb: mitral cell layer	0.640	0.029
M	Nos2	right olfactory peduncle	1.150	0.045
M	Nos2	right olfactory tubercle	1.659	0.073
M	Nos2	right optic tract	0.745	0.049
M	Nos2	right paraflocculus (PFL)	1.696	0.212
M	Nos2	right paraflocculus white matter	0.161	0.017
M	Nos2	right paramedian lobule	0.083	0.009
M	Nos2	right paramedian lobule (lobule 7)	2.054	0.094
M	Nos2	right Parietal cortex: posterior area: rostral part	0.043	0.003
M	Nos2	right Perirhinal cortex	1.268	0.044
M	Nos2	right Piriform cortex	5.523	0.191
M	Nos2	right PoDG	0.332	0.027
M	Nos2	right pontine nucleus	0.372	0.021
M	Nos2	right Posterolateral cortical amygdaloid area	0.487	0.022
M	Nos2	right Posteromedial cortical amygdaloid area	0.498	0.041
M	Nos2	right pre-para subiculum	1.174	0.056
M	Nos2	right Primary auditory cortex	0.736	0.052
M	Nos2	right Primary motor cortex	3.185	0.177
M	Nos2	right Primary somatosensory cortex	2.074	0.110
M	Nos2	right Primary somatosensory cortex: barrel field	4.590	0.275
M	Nos2	right Primary somatosensory cortex: dysgranular zone	0.181	0.010
M	Nos2	right Primary somatosensory cortex: forelimb region	1.796	0.095
M	Nos2	right Primary somatosensory cortex: hindlimb region	1.216	0.089
M	Nos2	right Primary somatosensory cortex: jaw region	0.279	0.022
M	Nos2	right Primary somatosensory cortex: shoulder region	0.071	0.009
M	Nos2	right Primary somatosensory cortex: trunk region	0.198	0.012
M	Nos2	right Primary somatosensory cortex: upper lip region	2.786	0.140
M	Nos2	right Primary visual cortex	1.244	0.081
M	Nos2	right Primary visual cortex: binocular area	1.008	0.077
M	Nos2	right Primary visual cortex: monocular area	0.768	0.046
M	Nos2	right Rostral amygdalopiriform area	0.205	0.010
M	Nos2	right Secondary auditory cortex: dorsal area	0.708	0.055
M	Nos2	right Secondary auditory cortex: ventral area	1.019	0.079
M	Nos2	right Secondary motor cortex	3.346	0.170
M	Nos2	right Secondary somatosensory cortex	3.718	0.325
M	Nos2	right Secondary visual cortex: lateral area	1.519	0.076
M	Nos2	right Secondary visual cortex: mediolateral area	0.402	0.029
M	Nos2	right Secondary visual cortex: mediomedial area	0.815	0.055
M	Nos2	right simple lobule (lobule 6)	2.392	0.100
M	Nos2	right simple lobule white matter	0.179	0.010
M	Nos2	right SLu	0.387	0.019
M	Nos2	right stria medullaris	0.349	0.014
M	Nos2	right stria terminalis	0.403	0.023
M	Nos2	right striatum	10.874	0.472
M	Nos2	right subependymale zone / rhinocele	0.027	0.002
M	Nos2	right subiculum	1.675	0.057
M	Nos2	right superior olivary complex	0.385	0.014
M	Nos2	right Temporal association area	1.687	0.111
M	Nos2	right thalamus	8.949	0.373
M	Nos2	right trunk of crus 2 and paramedian white matter	0.170	0.008
M	Nos2	right trunk of simple and crus 1 white matter	0.083	0.005
M	Nos2	right Ventral intermediate entorhinal cortex	0.559	0.024
M	Nos2	right Ventral nucleus of the endopiriform claustrum	0.230	0.021
M	Nos2	right Ventral orbital cortex	0.693	0.026
M	Nos2	right Ventral tenia tecta	0.054	0.004
M	Nos2	third ventricle	1.216	0.070
M	Nos2	trunk of arbor vita	4.141	0.152
M	Nos2	trunk of lobules 1-3 white matter	0.115	0.014
M	Nos2	trunk of lobules 6-8 white matter	0.106	0.007
M	Nos2	ventral tegmental decussation	0.128	0.013
M	Rag1	cerebral aqueduct	0.506	0.063
M	Rag1	fourth ventricle	0.927	0.070
M	Rag1	interpeduncular nucleus	0.280	0.012
M	Rag1	left Accessory olfactory bulb: glomerular, external plexiform and mitral cell layer	0.265	0.012
M	Rag1	left Accessory olfactory bulb: granule cell layer	0.133	0.006
M	Rag1	left amygdala	4.919	0.100
M	Rag1	left Amygdalopiriform transition area	0.533	0.027
M	Rag1	left anterior commissure: pars anterior	0.493	0.021
M	Rag1	left anterior commissure: pars posterior	0.219	0.006
M	Rag1	left anterior lobule (lobules 4-5)	0.892	0.066
M	Rag1	left anterior lobule white matter	0.044	0.005
M	Rag1	left Anterior olfactory nucleus	0.971	0.047
M	Rag1	left basal forebrain	2.626	0.074
M	Rag1	left bed nucleus of stria terminalis	0.695	0.025
M	Rag1	left CA10r	1.169	0.058
M	Rag1	left CA1Py	0.560	0.022
M	Rag1	left CA1Rad	1.257	0.040
M	Rag1	left CA20r	0.261	0.015
M	Rag1	left CA2Py	0.103	0.004
M	Rag1	left CA2Rad	0.210	0.006
M	Rag1	left CA30r	1.412	0.031
M	Rag1	left CA3Py Inner	0.079	0.003
M	Rag1	left CA3Py Outer	0.518	0.018
M	Rag1	left CA3Rad	0.933	0.028
M	Rag1	left Caudomedial entorhinal cortex	2.871	0.065
M	Rag1	left cerebellar peduncle: inferior	0.420	0.016
M	Rag1	left cerebellar peduncle: middle	0.572	0.030
M	Rag1	left cerebellar peduncle: superior	0.531	0.023
M	Rag1	left cerebral peduncle	1.029	0.031
M	Rag1	left Cingulate cortex: area 24a	0.934	0.040
M	Rag1	left Cingulate cortex: area 24a'	0.368	0.033
M	Rag1	left Cingulate cortex: area 24b	0.816	0.073
M	Rag1	left Cingulate cortex: area 24b'	0.304	0.022
M	Rag1	left Cingulate cortex: area 25	0.292	0.016
M	Rag1	left Cingulate cortex: area 29a	0.382	0.013
M	Rag1	left Cingulate cortex: area 29b	0.217	0.016
M	Rag1	left Cingulate cortex: area 29c	0.952	0.037
M	Rag1	left Cingulate cortex: area 30	1.472	0.063
M	Rag1	left Cingulate cortex: area 32	1.330	0.113
M	Rag1	left Cingulum	0.446	0.026
M	Rag1	left Claustrum	0.178	0.012
M	Rag1	left Claustrum: dorsal part	0.159	0.020
M	Rag1	left Claustrum: ventral part	0.289	0.024
M	Rag1	left colliculus: inferior	2.807	0.120
M	Rag1	left colliculus: superior	4.442	0.132
M	Rag1	left copula white matter	0.044	0.002
M	Rag1	left copula: pyramis (lobule 8)	1.151	0.063
M	Rag1	left corpus callosum	6.139	0.183
M	Rag1	left Cortex-amygdala transition zones	0.399	0.019
M	Rag1	left corticospinal tract/pyramids	0.863	0.051
M	Rag1	left crus 1 white matter	0.189	0.009
M	Rag1	left crus 1: ansiform lobule (lobule 6)	2.120	0.059
M	Rag1	left crus 2 white matter	0.127	0.008
M	Rag1	left crus 2: ansiform lobule (lobule 7)	1.948	0.082
M	Rag1	left cuneate nucleus	0.114	0.009
M	Rag1	left dentate nucleus	0.186	0.009
M	Rag1	left Dorsal intermediate entorhinal cortex	0.837	0.046
M	Rag1	left Dorsal nucleus of the endopiriform	0.738	0.028
M	Rag1	left Dorsal tenia tecta	0.490	0.028
M	Rag1	left Dorsolateral entorhinal cortex	1.216	0.032
M	Rag1	left Dorsolateral orbital cortex	0.430	0.018
M	Rag1	left Ectorhinal cortex	1.325	0.064
M	Rag1	left facial nerve (cranial nerve 7)	0.106	0.004
M	Rag1	left fasciculus retroflexus	0.133	0.007
M	Rag1	left fastigial nucleus	0.246	0.015
M	Rag1	left fimbria	1.659	0.054
M	Rag1	left flocculus (FL)	0.543	0.025
M	Rag1	left flocculus white matter	0.028	0.003
M	Rag1	left fornix	0.344	0.010
M	Rag1	left Frontal association cortex	3.568	0.168
M	Rag1	left Frontal cortex: area 3	0.361	0.012
M	Rag1	left fundus of striatum	0.077	0.004
M	Rag1	left globus pallidus	1.636	0.039
M	Rag1	left GrDG	0.685	0.020
M	Rag1	left habenular commissure	0.014	0.002
M	Rag1	left hypothalamus	5.361	0.103
M	Rag1	left inferior olivary complex	0.154	0.022
M	Rag1	left Insular region: not subdivided	3.894	0.183
M	Rag1	left Intermediate nucleus of the endopiriform claustrum	0.314	0.008

M	Rag1	left internal capsule	1.443	0.049
M	Rag1	left lateral olfactory tract	0.745	0.025
M	Rag1	left Lateral orbital cortex	1.776	0.053
M	Rag1	left Lateral parietal association cortex	0.150	0.015
M	Rag1	left lateral septum	1.526	0.048
M	Rag1	left lateral ventricle	1.742	0.079
M	Rag1	left LMol	1.200	0.027
M	Rag1	left mammillary bodies	0.237	0.018
M	Rag1	left mammilothalamic tract	0.125	0.006
M	Rag1	left Medial amygdala	0.539	0.017
M	Rag1	left Medial entorhinal cortex	0.347	0.018
M	Rag1	left medial lemniscus/medial longitudinal fasciculus	1.253	0.049
M	Rag1	left Medial orbital cortex	0.901	0.038
M	Rag1	left Medial parietal association cortex	0.234	0.027
M	Rag1	left Medial preoptic nucleus	0.102	0.007
M	Rag1	left medial septum	0.649	0.024
M	Rag1	left MoDG	2.186	0.046
M	Rag1	left nucleus accumbens	2.086	0.049
M	Rag1	left nucleus interpositus	0.236	0.014
M	Rag1	left Olfactory bulb: external plexiform layer	3.283	0.103
M	Rag1	left Olfactory bulb: glomerular layer	2.215	0.051
M	Rag1	left Olfactory bulb: granule cell layer	2.288	0.075
M	Rag1	left Olfactory bulb: internal plexiform layer	0.518	0.027
M	Rag1	left Olfactory bulb: mitral cell layer	0.652	0.030
M	Rag1	left olfactory peduncle	1.150	0.035
M	Rag1	left olfactory tubercle	1.621	0.081
M	Rag1	left optic tract	0.788	0.025
M	Rag1	left paraflocculus (PFL)	1.671	0.235
M	Rag1	left paraflocculus white matter	0.139	0.016
M	Rag1	left paramedian lobule	0.070	0.006
M	Rag1	left paramedian lobule (lobule 7)	1.959	0.107
M	Rag1	left Parietal cortex: posterior area: rostral part	0.070	0.005
M	Rag1	left Perirhinal cortex	1.226	0.048
M	Rag1	left Piriform cortex	5.097	0.132
M	Rag1	left PoDG	0.307	0.011
M	Rag1	left pontine nucleus	0.382	0.039
M	Rag1	left Posterolateral cortical amygdaloid area	0.425	0.030
M	Rag1	left Posteromedial cortical amygdaloid area	0.591	0.027
M	Rag1	left pre-para subiculum	1.148	0.047
M	Rag1	left Primary auditory cortex	0.722	0.037
M	Rag1	left Primary motor cortex	3.682	0.113
M	Rag1	left Primary somatosensory cortex	2.017	0.064
M	Rag1	left Primary somatosensory cortex: barrel field	4.574	0.132
M	Rag1	left Primary somatosensory cortex: dysgranular zone	0.165	0.013
M	Rag1	left Primary somatosensory cortex: forelimb region	1.977	0.071
M	Rag1	left Primary somatosensory cortex: hindlimb region	1.412	0.069
M	Rag1	left Primary somatosensory cortex: jaw region	0.252	0.023
M	Rag1	left Primary somatosensory cortex: shoulder region	0.092	0.010
M	Rag1	left Primary somatosensory cortex: trunk region	0.302	0.022
M	Rag1	left Primary somatosensory cortex: upper lip region	2.358	0.092
M	Rag1	left Primary visual cortex	1.173	0.080
M	Rag1	left Primary visual cortex: binocular area	1.035	0.052
M	Rag1	left Primary visual cortex: monocular area	1.207	0.068
M	Rag1	left Rostral amygdalopiriform area	0.196	0.017
M	Rag1	left Secondary auditory cortex: dorsal area	0.725	0.048
M	Rag1	left Secondary auditory cortex: ventral area	0.770	0.057
M	Rag1	left Secondary motor cortex	3.377	0.138
M	Rag1	left Secondary somatosensory cortex	3.489	0.256
M	Rag1	left Secondary visual cortex: lateral area	1.299	0.054
M	Rag1	left Secondary visual cortex: mediolateral area	0.594	0.044
M	Rag1	left Secondary visual cortex: mediomedial area	0.948	0.033
M	Rag1	left simple lobule (lobule 6)	2.355	0.065
M	Rag1	left simple lobule white matter	0.222	0.015
M	Rag1	left SLu	0.380	0.014
M	Rag1	left stria medullaris	0.326	0.013
M	Rag1	left stria terminalis	0.480	0.018
M	Rag1	left striatum	10.621	0.239
M	Rag1	left subependymale zone / rhinocele	0.028	0.001
M	Rag1	left subiculum	1.646	0.052
M	Rag1	left superior olivary complex	0.366	0.026
M	Rag1	left Temporal association area	1.356	0.096
M	Rag1	left thalamus	8.871	0.343
M	Rag1	left trunk of crus 2 and paramedian white matter	0.171	0.010
M	Rag1	left trunk of simple and crus 1 white matter	0.070	0.003
M	Rag1	left Ventral intermediate entorhinal cortex	0.556	0.019
M	Rag1	left Ventral nucleus of the endopiriform claustrum	0.256	0.023
M	Rag1	left Ventral orbital cortex	0.764	0.032
M	Rag1	left Ventral tenia tecta	0.057	0.004
M	Rag1	lobule 1-2 white matter	0.063	0.016
M	Rag1	lobule 10 white matter	0.082	0.004
M	Rag1	lobule 10: nodulus	1.328	0.045
M	Rag1	lobule 3 white matter	0.183	0.015
M	Rag1	lobule 3: central lobule (dorsal)	2.128	0.163
M	Rag1	lobule 6: declive	2.343	0.122
M	Rag1	lobule 7: tuber (or folium)	0.874	0.054
M	Rag1	lobule 8 white matter	0.155	0.010
M	Rag1	lobule 8: pyramis	1.546	0.063
M	Rag1	lobule 9 white matter	0.289	0.012
M	Rag1	lobule 9: uvula	2.721	0.110
M	Rag1	lobules 1-2: lingula and central lobule (ventral)	1.598	0.188
M	Rag1	lobules 4-5 white matter	0.570	0.045
M	Rag1	lobules 4-5: culmen (ventral and dorsal)	4.292	0.258
M	Rag1	lobules 6-7 white matter	0.646	0.038
M	Rag1	medulla	27.117	0.871
M	Rag1	midbrain	14.033	0.538
M	Rag1	periaqueductal grey	4.011	0.142
M	Rag1	pons	17.264	0.482
M	Rag1	posterior commissure	0.146	0.007
M	Rag1	right Accessory olfactory bulb: glomerular, external plexiform and mitral cell layer	0.254	0.011
M	Rag1	right Accessory olfactory bulb: granule cell layer	0.126	0.007
M	Rag1	right amygdala	4.671	0.141
M	Rag1	right Amygdalopiriform transition area	0.480	0.023
M	Rag1	right anterior commissure: pars anterior	0.485	0.015
M	Rag1	right anterior commissure: pars posterior	0.206	0.011
M	Rag1	right anterior lobule (lobules 4-5)	0.810	0.042
M	Rag1	right anterior lobule white matter	0.054	0.004
M	Rag1	right Anterior olfactory nucleus	0.994	0.039
M	Rag1	right basal forebrain	2.717	0.086
M	Rag1	right bed nucleus of stria terminalis	0.697	0.028
M	Rag1	right CA10r	1.035	0.058
M	Rag1	right CA1Py	0.571	0.027
M	Rag1	right CA1Rad	1.371	0.057
M	Rag1	right CA20r	0.216	0.008
M	Rag1	right CA2Py	0.099	0.002
M	Rag1	right CA2Rad	0.219	0.010
M	Rag1	right CA30r	1.417	0.049
M	Rag1	right CA3Py Inner	0.078	0.003
M	Rag1	right CA3Py Outer	0.518	0.024
M	Rag1	right CA3Rad	0.955	0.050
M	Rag1	right Caudomedial entorhinal cortex	2.805	0.126
M	Rag1	right cerebellar peduncle: inferior	0.385	0.010
M	Rag1	right cerebellar peduncle: middle	0.593	0.039
M	Rag1	right cerebellar peduncle: superior	0.508	0.034
M	Rag1	right cerebral peduncle	1.043	0.027
M	Rag1	right Cingulate cortex: area 24a	0.988	0.029
M	Rag1	right Cingulate cortex: area 24a'	0.345	0.034
M	Rag1	right Cingulate cortex: area 24b	0.779	0.061
M	Rag1	right Cingulate cortex: area 24b'	0.289	0.020
M	Rag1	right Cingulate cortex: area 25	0.301	0.019
M	Rag1	right Cingulate cortex: area 29a	0.361	0.017
M	Rag1	right Cingulate cortex: area 29b	0.202	0.011
M	Rag1	right Cingulate cortex: area 29c	0.889	0.031
M	Rag1	right Cingulate cortex: area 30	1.348	0.054
M	Rag1	right Cingulate cortex: area 32	1.075	0.071
M	Rag1	right Cingulum	0.449	0.024
M	Rag1	right Claustrum	0.151	0.009
M	Rag1	right Claustrum: dorsal part	0.146	0.019
M	Rag1	right Claustrum: ventral part	0.314	0.031
M	Rag1	right colliculus: inferior	2.688	0.078
M	Rag1	right colliculus: superior	4.492	0.174
M	Rag1	right copula white matter	0.035	0.002
M	Rag1	right copula: pyramis (lobule 8)	1.274	0.080
M	Rag1	right corpus callosum	5.771	0.300
M	Rag1	right Cortex-amygdala transition zones	0.444	0.041
M	Rag1	right corticospinal tract/pyramids	0.899	0.052
M	Rag1	right crus 1 white matter	0.179	0.010
M	Rag1	right crus 1: ansiform lobule (lobule 6)	2.149	0.087
M	Rag1	right crus 2 white matter	0.141	0.012
M	Rag1	right crus 2: ansiform lobule (lobule 7)	1.925	0.082
M	Rag1	right cuneate nucleus	0.121	0.009
M	Rag1	right dentate nucleus	0.182	0.011
M	Rag1	right Dorsal intermediate entorhinal cortex	0.872	0.036
M	Rag1	right Dorsal nucleus of the endopiriform	0.723	0.024
M	Rag1	right Dorsal tenia tecta	0.454	0.022
M	Rag1	right Dorsolateral entorhinal cortex	1.358	0.070
M	Rag1	right Dorsolateral orbital cortex	0.410	0.024
M	Rag1	right Ectorhinal cortex	1.389	0.076
M	Rag1	right facial nerve (cranial nerve 7)	0.109	0.004
M	Rag1	right fasciculus retroflexus	0.118	0.007
M	Rag1	right fastigial nucleus	0.238	0.012
M	Rag1	right fimbria	1.534	0.075
M	Rag1	right flocculus (FL)	0.499	0.038
M	Rag1	right flocculus white matter	0.036	0.003
M	Rag1	right fornix	0.303	0.011
M	Rag1	right Frontal association cortex	3.944	0.152
M	Rag1	right Frontal cortex: area 3	0.371	0.010
M	Rag1	right fundus of striatum	0.085	0.006
M	Rag1	right globus pallidus	1.653	0.073
M	Rag1	right GrDG	0.644	0.023
M	Rag1	right habenular commissure	0.013	0.001
M	Rag1	right hypothalamus	5.307	0.115
M	Rag1	right inferior olivary complex	0.168	0.021
M	Rag1	right Insular region: not subdivided	4.156	0.270
M	Rag1	right Intermediate nucleus of the endopiriform claustrum	0.290	0.010
M	Rag1	right internal capsule	1.511	0.055
M	Rag1	right lateral olfactory tract	0.663	0.032
M	Rag1	right Lateral orbital cortex	1.774	0.053
M	Rag1	right Lateral parietal association cortex	0.090	0.010
M	Rag1	right lateral septum	1.535	0.052
M	Rag1	right lateral ventricle	1.863	0.138
M	Rag1	right LMol	1.183	0.035
M	Rag1	right mammillary bodies	0.222	0.018
M	Rag1	right mammilothalamic tract	0.130	0.006
M	Rag1	right Medial amygdala	0.574	0.027
M	Rag1	right Medial entorhinal cortex	0.339	0.016
M	Rag1	right medial lemniscus/medial longitudinal fasciculus	1.335	0.048
M	Rag1	right Medial orbital cortex	0.833	0.030
M	Rag1	right Medial parietal association cortex	0.173	0.014
M	Rag1	right Medial preoptic nucleus	0.093	0.007
M	Rag1	right medial septum	0.613	0.026
M	Rag1	right MoDG	2.048	0.058
M	Rag1	right nucleus accumbens	2.077	0.066
M	Rag1	right nucleus interpositus	0.249	0.013
M	Rag1	right Olfactory bulb: external plexiform layer	3.296	0.086
M	Rag1	right Olfactory bulb: glomerular layer	2.330	0.066
M	Rag1	right Olfactory bulb: granule cell layer	2.352	0.080
M	Rag1	right Olfactory bulb: internal plexiform layer	0.503	0.020
M	Rag1	right Olfactory bulb: mitral cell layer	0.606	0.021
M	Rag1	right olfactory peduncle	1.121	0.025
M	Rag1	right olfactory tubercle	1.658	0.128
M	Rag1	right optic tract	0.736	0.013
M	Rag1	right paraflocculus (PFL)	1.700	0.209
M	Rag1	right paraflocculus white matter	0.162	0.018
M	Rag1	right paramedian lobule	0.078	0.006
M	Rag1	right paramedian lobule (lobule 7)	1.957	0.076
M	Rag1	right Parietal cortex: posterior area: rostral part	0.045	0.005
M	Rag1	right Perirhinal cortex	1.190	0.060
M	Rag1	right Piriform cortex	5.481	0.240
M	Rag1	right PoDG	0.319	0.015
M	Rag1	right pontine nucleus	0.374	0.039
M	Rag1	right Posterolateral cortical amygdaloid area	0.468	0.021
M	Rag1	right Posteromedial cortical amygdaloid area	0.461	0.020
M	Rag1	right pre-para subiculum	1.173	0.050
M	Rag1	right Primary auditory cortex	0.710	0.038
M	Rag1	right Primary motor cortex	3.134	0.106
M	Rag1	right Primary somatosensory cortex	2.055	0.095
M	Rag1	right Primary somatosensory cortex: barrel field	4.454	0.169
M	Rag1	right Primary somatosensory cortex: dysgranular zone	0.173	0.008
M	Rag1	right Primary somatosensory cortex: forelimb region	1.728	0.087
M	Rag1	right Primary somatosensory cortex: hindlimb region	1.162	0.064
M	Rag1	right Primary somatosensory cortex: jaw region	0.263	0.015
M	Rag1	right Primary somatosensory cortex: shoulder region	0.066	0.010
M	Rag1	right Primary somatosensory cortex: trunk region	0.197	0.022
M	Rag1	right Primary somatosensory cortex: upper lip region	2.737	0.105
M	Rag1	right Primary visual cortex	1.169	0.091
M	Rag1	right Primary visual cortex: binocular area	1.022	0.053
M	Rag1	right Primary visual cortex: monocular area	0.777	0.034
M	Rag1	right Rostral amygdalopiriform area	0.195	0.017
M	Rag1	right Secondary auditory cortex: dorsal area	0.677	0.021
M	Rag1	right Secondary auditory cortex: ventral area	0.951	0.070
M	Rag1	right Secondary motor cortex	3.231	0.161
M	Rag1	right Secondary somatosensory cortex	3.716	0.213
M	Rag1	right Secondary visual cortex: lateral area	1.447	0.045
M	Rag1	right Secondary visual cortex: mediolateral area	0.399	0.020
M	Rag1	right Secondary visual cortex: mediomedial area	0.790	0.023
M	Rag1	right simple lobule (lobule 6)	2.408	0.102
M	Rag1	right simple lobule white matter	0.182	0.011
M	Rag1	right SLu	0.375	0.023
M	Rag1	right stria medullaris	0.351	0.011
M	Rag1	right stria terminalis	0.402	0.010
M	Rag1	right striatum	10.466	0.428
M	Rag1	right subependymale zone / rhinocele	0.025	0.002
M	Rag1	right subiculum	1.646	0.065
M	Rag1	right superior olivary complex	0.392	0.043
M	Rag1	right Temporal association area	1.551	0.077
M	Rag1	right thalamus	8.768	0.347
M	Rag1	right trunk of crus 2 and paramedian white matter	0.176	0.012
M	Rag1	right trunk of simple and crus 1 white matter	0.083	0.006
M	Rag1	right Ventral intermediate entorhinal cortex	0.546	0.023
M	Rag1	right Ventral nucleus of the endopiriform claustrum	0.235	0.019
M	Rag1	right Ventral orbital cortex	0.684	0.032
M	Rag1	right Ventral tenia tecta	0.057	0.005
M	Rag1	third ventricle	1.215	0.070
M	Rag1	trunk of arbor vita	4.231	0.147
M	Rag1	trunk of lobules 1-3 white matter	0.120	0.009
M	Rag1	trunk of lobules 6-8 white matter	0.109	0.007
M	Rag1	ventral tegmental decussation	0.134	0.009
M	Rag2	cerebral aqueduct	0.497	0.052
M	Rag2	fourth ventricle	0.895	0.066
M	Rag2	interpeduncular nucleus	0.270	0.008
M	Rag2	left Accessory olfactory bulb: glomerular, external plexiform and mitral cell layer	0.249	0.011
M	Rag2	left Accessory olfactory bulb: granule cell layer	0.127	0.008
M	Rag2	left amygdala	4.762	0.207
M	Rag2	left Amygdalopiriform transition area	0.508	0.031
M	Rag2	left anterior commissure: pars anterior	0.478	0.024
M	Rag2	left anterior commissure: pars posterior	0.202	0.011
M	Rag2	left anterior lobule (lobules 4-5)	0.815	0.062

M	Rag2	left anterior lobule white matter	0.041	0.003
M	Rag2	left Anterior olfactory nucleus	0.994	0.051
M	Rag2	left basal forebrain	2.530	0.105
M	Rag2	left bed nucleus of stria terminalis	0.680	0.035
M	Rag2	left CA10r	1.121	0.055
M	Rag2	left CA1Py	0.543	0.020
M	Rag2	left CA1Rad	1.236	0.042
M	Rag2	left CA20r	0.244	0.013
M	Rag2	left CA2Py	0.098	0.005
M	Rag2	left CA2Rad	0.205	0.010
M	Rag2	left CA30r	1.348	0.078
M	Rag2	left CA3Py Inner	0.074	0.004
M	Rag2	left CA3Py Outer	0.494	0.027
M	Rag2	left CA3Rad	0.894	0.046
M	Rag2	left Caudomedial entorhinal cortex	2.765	0.148
M	Rag2	left cerebellar peduncle: inferior	0.394	0.030
M	Rag2	left cerebellar peduncle: middle	0.526	0.042
M	Rag2	left cerebellar peduncle: superior	0.500	0.035
M	Rag2	left cerebral peduncle	0.988	0.054
M	Rag2	left Cingulate cortex: area 24a	0.921	0.043
M	Rag2	left Cingulate cortex: area 24a'	0.372	0.030
M	Rag2	left Cingulate cortex: area 24b	0.822	0.080
M	Rag2	left Cingulate cortex: area 24b'	0.310	0.016
M	Rag2	left Cingulate cortex: area 25	0.288	0.011
M	Rag2	left Cingulate cortex: area 29a	0.355	0.023
M	Rag2	left Cingulate cortex: area 29b	0.202	0.016
M	Rag2	left Cingulate cortex: area 29c	0.942	0.056
M	Rag2	left Cingulate cortex: area 30	1.402	0.077
M	Rag2	left Cingulate cortex: area 32	1.320	0.097
M	Rag2	left Cingulum	0.415	0.033
M	Rag2	left Claustrum	0.173	0.010
M	Rag2	left Claustrum: dorsal part	0.156	0.018
M	Rag2	left Claustrum: ventral part	0.280	0.024
M	Rag2	left colliculus: inferior	2.799	0.147
M	Rag2	left colliculus: superior	4.307	0.241
M	Rag2	left copula white matter	0.041	0.004
M	Rag2	left copula: pyramis (lobule 8)	1.100	0.098
M	Rag2	left corpus callosum	5.844	0.372
M	Rag2	left Cortex-amygdala transition zones	0.386	0.025
M	Rag2	left corticospinal tract/pyramids	0.803	0.062
M	Rag2	left crus 1 white matter	0.178	0.014
M	Rag2	left crus 1: ansiform lobule (lobule 6)	2.080	0.173
M	Rag2	left crus 2 white matter	0.120	0.008
M	Rag2	left crus 2: ansiform lobule (lobule 7)	1.874	0.137
M	Rag2	left cuneate nucleus	0.113	0.013
M	Rag2	left dentate nucleus	0.176	0.010
M	Rag2	left Dorsal intermediate entorhinal cortex	0.802	0.048
M	Rag2	left Dorsal nucleus of the endopiriform	0.710	0.039
M	Rag2	left Dorsal tenia tecta	0.481	0.031
M	Rag2	left Dorsolateral entorhinal cortex	1.186	0.083
M	Rag2	left Dorsolateral orbital cortex	0.423	0.029
M	Rag2	left Ectorhinal cortex	1.296	0.137
M	Rag2	left facial nerve (cranial nerve 7)	0.100	0.005
M	Rag2	left fasciculus retroflexus	0.129	0.008
M	Rag2	left fastigial nucleus	0.238	0.014
M	Rag2	left fimbria	1.578	0.094
M	Rag2	left flocculus (FL)	0.509	0.043
M	Rag2	left flocculus white matter	0.027	0.005
M	Rag2	left fornix	0.327	0.016
M	Rag2	left Frontal association cortex	3.498	0.116
M	Rag2	left Frontal cortex: area 3	0.341	0.029
M	Rag2	left fundus of striatum	0.070	0.004
M	Rag2	left globus pallidus	1.597	0.098
M	Rag2	left GrDG	0.660	0.032
M	Rag2	left habenular commissure	0.013	0.001
M	Rag2	left hypothalamus	5.109	0.214
M	Rag2	left inferior olivary complex	0.147	0.027
M	Rag2	left Insular region: not subdivided	3.713	0.281
M	Rag2	left Intermediate nucleus of the endopiriform claustrum	0.303	0.017
M	Rag2	left internal capsule	1.376	0.088
M	Rag2	left lateral olfactory tract	0.725	0.038
M	Rag2	left Lateral orbital cortex	1.724	0.075
M	Rag2	left Lateral parietal association cortex	0.146	0.016
M	Rag2	left lateral septum	1.478	0.061
M	Rag2	left lateral ventricle	1.677	0.133
M	Rag2	left LMol	1.175	0.046
M	Rag2	left mammillary bodies	0.217	0.012
M	Rag2	left mammilothalamic tract	0.120	0.008
M	Rag2	left Medial amygdala	0.524	0.027
M	Rag2	left Medial entorhinal cortex	0.340	0.028
M	Rag2	left medial lemniscus/medial longitudinal fasciculus	1.180	0.066
M	Rag2	left Medial orbital cortex	0.900	0.051
M	Rag2	left Medial parietal association cortex	0.228	0.027
M	Rag2	left Medial preoptic nucleus	0.100	0.007
M	Rag2	left medial septum	0.631	0.034
M	Rag2	left MoDG	2.108	0.088
M	Rag2	left nucleus accumbens	2.075	0.082
M	Rag2	left nucleus interpositus	0.224	0.017
M	Rag2	left Olfactory bulb: external plexiform layer	3.085	0.173
M	Rag2	left Olfactory bulb: glomerular layer	2.038	0.118
M	Rag2	left Olfactory bulb: granule cell layer	2.264	0.135
M	Rag2	left Olfactory bulb: internal plexiform layer	0.511	0.036
M	Rag2	left Olfactory bulb: mitral cell layer	0.635	0.042
M	Rag2	left olfactory peduncle	1.132	0.050
M	Rag2	left olfactory tubercle	1.626	0.069
M	Rag2	left optic tract	0.760	0.032
M	Rag2	left paraflocculus (PFL)	1.725	0.212
M	Rag2	left paraflocculus white matter	0.138	0.013
M	Rag2	left paramedian lobule	0.068	0.006
M	Rag2	left paramedian lobule (lobule 7)	1.933	0.115
M	Rag2	left Parietal cortex: posterior area: rostral part	0.070	0.008
M	Rag2	left Perirhinal cortex	1.214	0.082
M	Rag2	left Piriform cortex	4.979	0.204
M	Rag2	left PoDG	0.296	0.016
M	Rag2	left pontine nucleus	0.351	0.039
M	Rag2	left Posterolateral cortical amygdaloid area	0.402	0.027
M	Rag2	left Posteromedial cortical amygdaloid area	0.594	0.038
M	Rag2	left pre-para subiculum	1.087	0.045
M	Rag2	left Primary auditory cortex	0.728	0.040
M	Rag2	left Primary motor cortex	3.505	0.169
M	Rag2	left Primary somatosensory cortex	1.940	0.140
M	Rag2	left Primary somatosensory cortex: barrel field	4.584	0.251
M	Rag2	left Primary somatosensory cortex: dysgranular zone	0.165	0.016
M	Rag2	left Primary somatosensory cortex: forelimb region	1.992	0.113
M	Rag2	left Primary somatosensory cortex: hindlimb region	1.390	0.087
M	Rag2	left Primary somatosensory cortex: jaw region	0.263	0.036
M	Rag2	left Primary somatosensory cortex: shoulder region	0.087	0.006
M	Rag2	left Primary somatosensory cortex: trunk region	0.297	0.024
M	Rag2	left Primary somatosensory cortex: upper lip region	2.319	0.183
M	Rag2	left Primary visual cortex	1.165	0.060
M	Rag2	left Primary visual cortex: binocular area	0.986	0.070
M	Rag2	left Primary visual cortex: monocular area	1.138	0.082
M	Rag2	left Rostral amygdalopiriform area	0.187	0.015
M	Rag2	left Secondary auditory cortex: dorsal area	0.712	0.045
M	Rag2	left Secondary auditory cortex: ventral area	0.756	0.057
M	Rag2	left Secondary motor cortex	3.263	0.173
M	Rag2	left Secondary somatosensory cortex	3.326	0.323
M	Rag2	left Secondary visual cortex: lateral area	1.282	0.081
M	Rag2	left Secondary visual cortex: mediolateral area	0.570	0.051
M	Rag2	left Secondary visual cortex: mediomedial area	0.915	0.052
M	Rag2	left simple lobule (lobule 6)	2.222	0.136
M	Rag2	left simple lobule white matter	0.201	0.013
M	Rag2	left SLu	0.366	0.021
M	Rag2	left stria medullaris	0.312	0.015
M	Rag2	left stria terminalis	0.456	0.032
M	Rag2	left striatum	10.364	0.426
M	Rag2	left subependymale zone / rhinocele	0.029	0.002
M	Rag2	left subiculum	1.584	0.076
M	Rag2	left superior olivary complex	0.353	0.032
M	Rag2	left Temporal association area	1.360	0.099
M	Rag2	left thalamus	8.679	0.444
M	Rag2	left trunk of crus 2 and paramedian white matter	0.151	0.011
M	Rag2	left trunk of simple and crus 1 white matter	0.069	0.006
M	Rag2	left Ventral intermediate entorhinal cortex	0.530	0.032
M	Rag2	left Ventral nucleus of the endopiriform claustrum	0.240	0.016
M	Rag2	left Ventral orbital cortex	0.743	0.037
M	Rag2	left Ventral tenia tecta	0.054	0.004
M	Rag2	lobule 1-2 white matter	0.066	0.017
M	Rag2	lobule 10 white matter	0.077	0.006
M	Rag2	lobule 10: nodulus	1.271	0.081
M	Rag2	lobule 3 white matter	0.184	0.035
M	Rag2	lobule 3: central lobule (dorsal)	2.169	0.152
M	Rag2	lobule 6: declive	2.344	0.130
M	Rag2	lobule 7: tuber (or folium)	0.859	0.094
M	Rag2	lobule 8 white matter	0.144	0.015
M	Rag2	lobule 8: pyramis	1.511	0.143
M	Rag2	lobule 9 white matter	0.270	0.018
M	Rag2	lobule 9: uvula	2.628	0.157
M	Rag2	lobules 1-2: lingula and central lobule (ventral)	1.637	0.226
M	Rag2	lobules 4-5 white matter	0.542	0.024
M	Rag2	lobules 4-5: culmen (ventral and dorsal)	4.109	0.188
M	Rag2	lobules 6-7 white matter	0.650	0.037
M	Rag2	medulla	26.032	1.269
M	Rag2	midbrain	13.507	0.732
M	Rag2	periaqueductal grey	3.902	0.183
M	Rag2	pons	16.468	0.754
M	Rag2	posterior commissure	0.142	0.010
M	Rag2	right Accessory olfactory bulb: glomerular, external plexiform and mitral cell layer	0.247	0.012
M	Rag2	right Accessory olfactory bulb: granule cell layer	0.123	0.008
M	Rag2	right amygdala	4.470	0.185
M	Rag2	right Amygdalopiriform transition area	0.482	0.028
M	Rag2	right anterior commissure: pars anterior	0.472	0.021
M	Rag2	right anterior commissure: pars posterior	0.194	0.013
M	Rag2	right anterior lobule (lobules 4-5)	0.763	0.058
M	Rag2	right anterior lobule white matter	0.050	0.005
M	Rag2	right Anterior olfactory nucleus	1.024	0.038
M	Rag2	right basal forebrain	2.653	0.109
M	Rag2	right bed nucleus of stria terminalis	0.672	0.033
M	Rag2	right CA10r	0.993	0.048
M	Rag2	right CA1Py	0.553	0.023
M	Rag2	right CA1Rad	1.341	0.044
M	Rag2	right CA20r	0.206	0.010
M	Rag2	right CA2Py	0.094	0.005
M	Rag2	right CA2Rad	0.212	0.008
M	Rag2	right CA30r	1.332	0.080
M	Rag2	right CA3Py Inner	0.077	0.004
M	Rag2	right CA3Py Outer	0.491	0.027
M	Rag2	right CA3Rad	0.935	0.042
M	Rag2	right Caudomedial entorhinal cortex	2.742	0.154
M	Rag2	right cerebellar peduncle: inferior	0.364	0.028
M	Rag2	right cerebellar peduncle: middle	0.557	0.049
M	Rag2	right cerebellar peduncle: superior	0.483	0.038
M	Rag2	right cerebral peduncle	1.002	0.054
M	Rag2	right Cingulate cortex: area 24a	0.979	0.050
M	Rag2	right Cingulate cortex: area 24a'	0.351	0.025
M	Rag2	right Cingulate cortex: area 24b	0.794	0.075
M	Rag2	right Cingulate cortex: area 24b'	0.299	0.022
M	Rag2	right Cingulate cortex: area 25	0.291	0.013
M	Rag2	right Cingulate cortex: area 29a	0.345	0.024
M	Rag2	right Cingulate cortex: area 29b	0.193	0.016
M	Rag2	right Cingulate cortex: area 29c	0.884	0.056
M	Rag2	right Cingulate cortex: area 30	1.301	0.074
M	Rag2	right Cingulate cortex: area 32	1.067	0.083
M	Rag2	right Cingulum	0.425	0.037
M	Rag2	right Claustrum	0.144	0.011
M	Rag2	right Claustrum: dorsal part	0.147	0.018
M	Rag2	right Claustrum: ventral part	0.319	0.028
M	Rag2	right colliculus: inferior	2.699	0.111
M	Rag2	right colliculus: superior	4.387	0.200
M	Rag2	right copula white matter	0.033	0.004
M	Rag2	right copula: pyramis (lobule 8)	1.201	0.113
M	Rag2	right corpus callosum	5.560	0.394
M	Rag2	right Cortex-amygdala transition zones	0.415	0.042
M	Rag2	right corticospinal tract/pyramids	0.841	0.062
M	Rag2	right crus 1 white matter	0.168	0.013
M	Rag2	right crus 1: ansiform lobule (lobule 6)	2.088	0.150
M	Rag2	right crus 2 white matter	0.138	0.012
M	Rag2	right crus 2: ansiform lobule (lobule 7)	1.903	0.133
M	Rag2	right cuneate nucleus	0.117	0.010
M	Rag2	right dentate nucleus	0.173	0.013
M	Rag2	right Dorsal intermediate entorhinal cortex	0.867	0.041
M	Rag2	right Dorsal nucleus of the endopiriform	0.694	0.028
M	Rag2	right Dorsal tenia tecta	0.443	0.027
M	Rag2	right Dorsolateral entorhinal cortex	1.320	0.082
M	Rag2	right Dorsolateral orbital cortex	0.404	0.023
M	Rag2	right Ectorhinal cortex	1.372	0.103
M	Rag2	right facial nerve (cranial nerve 7)	0.106	0.007
M	Rag2	right fasciculus retroflexus	0.114	0.008
M	Rag2	right fastigial nucleus	0.227	0.014
M	Rag2	right fimbria	1.462	0.084
M	Rag2	right flocculus (FL)	0.476	0.047
M	Rag2	right flocculus white matter	0.035	0.004
M	Rag2	right fornix	0.288	0.017
M	Rag2	right Frontal association cortex	3.923	0.206
M	Rag2	right Frontal cortex: area 3	0.348	0.020
M	Rag2	right fundus of striatum	0.078	0.004
M	Rag2	right globus pallidus	1.620	0.097
M	Rag2	right GrDG	0.623	0.028
M	Rag2	right habenular commissure	0.012	0.002
M	Rag2	right hypothalamus	5.102	0.207
M	Rag2	right inferior olivary complex	0.161	0.029
M	Rag2	right Insular region: not subdivided	3.975	0.255
M	Rag2	right Intermediate nucleus of the endopiriform claustrum	0.275	0.014
M	Rag2	right internal capsule	1.478	0.094
M	Rag2	right lateral olfactory tract	0.651	0.035
M	Rag2	right Lateral orbital cortex	1.716	0.082
M	Rag2	right Lateral parietal association cortex	0.086	0.010
M	Rag2	right lateral septum	1.475	0.063
M	Rag2	right lateral ventricle	1.732	0.165
M	Rag2	right LMol	1.156	0.042
M	Rag2	right mammillary bodies	0.209	0.015
M	Rag2	right mammilothalamic tract	0.127	0.007
M	Rag2	right Medial amygdala	0.556	0.033
M	Rag2	right Medial entorhinal cortex	0.330	0.019
M	Rag2	right medial lemniscus/medial longitudinal fasciculus	1.252	0.069
M	Rag2	right Medial orbital cortex	0.808	0.053
M	Rag2	right Medial parietal association cortex	0.168	0.019
M	Rag2	right Medial preoptic nucleus	0.089	0.005
M	Rag2	right medial septum	0.582	0.030
M	Rag2	right MoDG	1.975	0.081
M	Rag2	right nucleus accumbens	2.053	0.081
M	Rag2	right nucleus interpositus	0.233	0.016
M	Rag2	right Olfactory bulb: external plexiform layer	3.078	0.146
M	Rag2	right Olfactory bulb: glomerular layer	2.110	0.097
M	Rag2	right Olfactory bulb: granule cell layer	2.325	0.122
M	Rag2	right Olfactory bulb: internal plexiform layer	0.502	0.026
M	Rag2	right Olfactory bulb: mitral cell layer	0.601	0.030

M	Rag2	right olfactory peduncle	1.114	0.046
M	Rag2	right olfactory tubercle	1.612	0.065
M	Rag2	right optic tract	0.710	0.034
M	Rag2	right paraflocculus (PFL)	1.729	0.201
M	Rag2	right paraflocculus white matter	0.160	0.016
M	Rag2	right paramedian lobule	0.074	0.008
M	Rag2	right paramedian lobule (lobule 7)	1.909	0.147
M	Rag2	right Parietal cortex: posterior area: rostral part	0.044	0.004
M	Rag2	right Perirhinal cortex	1.170	0.074
M	Rag2	right Piriform cortex	5.251	0.217
M	Rag2	right PoDG	0.311	0.016
M	Rag2	right pontine nucleus	0.343	0.037
M	Rag2	right Posterolateral cortical amygdaloid area	0.446	0.025
M	Rag2	right Posteromedial cortical amygdaloid area	0.453	0.027
M	Rag2	right pre-para subiculum	1.127	0.059
M	Rag2	right Primary auditory cortex	0.723	0.040
M	Rag2	right Primary motor cortex	3.060	0.198
M	Rag2	right Primary somatosensory cortex	1.996	0.084
M	Rag2	right Primary somatosensory cortex: barrel field	4.550	0.201
M	Rag2	right Primary somatosensory cortex: dysgranular zone	0.174	0.011
M	Rag2	right Primary somatosensory cortex: forelimb region	1.761	0.110
M	Rag2	right Primary somatosensory cortex: hindlimb region	1.138	0.072
M	Rag2	right Primary somatosensory cortex: jaw region	0.275	0.021
M	Rag2	right Primary somatosensory cortex: shoulder region	0.065	0.008
M	Rag2	right Primary somatosensory cortex: trunk region	0.193	0.022
M	Rag2	right Primary somatosensory cortex: upper lip region	2.655	0.126
M	Rag2	right Primary visual cortex	1.176	0.055
M	Rag2	right Primary visual cortex: binocular area	0.982	0.054
M	Rag2	right Primary visual cortex: monocular area	0.756	0.050
M	Rag2	right Rostral amygdalopiriform area	0.204	0.017
M	Rag2	right Secondary auditory cortex: dorsal area	0.681	0.033
M	Rag2	right Secondary auditory cortex: ventral area	0.961	0.085
M	Rag2	right Secondary motor cortex	3.183	0.171
M	Rag2	right Secondary somatosensory cortex	3.529	0.253
M	Rag2	right Secondary visual cortex: lateral area	1.444	0.064
M	Rag2	right Secondary visual cortex: mediolateral area	0.391	0.030
M	Rag2	right Secondary visual cortex: mediomedial area	0.771	0.048
M	Rag2	right simple lobule (lobule 6)	2.264	0.163
M	Rag2	right simple lobule white matter	0.166	0.013
M	Rag2	right SLu	0.362	0.019
M	Rag2	right stria medullaris	0.336	0.015
M	Rag2	right stria terminalis	0.384	0.023
M	Rag2	right striatum	10.317	0.394
M	Rag2	right subependymale zone / rhinocele	0.025	0.002
M	Rag2	right subiculum	1.592	0.063
M	Rag2	right superior olivary complex	0.371	0.042
M	Rag2	right Temporal association area	1.564	0.106
M	Rag2	right thalamus	8.648	0.381
M	Rag2	right trunk of crus 2 and paramedian white matter	0.165	0.014
M	Rag2	right trunk of simple and crus 1 white matter	0.084	0.007
M	Rag2	right Ventral intermediate entorhinal cortex	0.537	0.029
M	Rag2	right Ventral nucleus of the endopiriform claustrum	0.223	0.014
M	Rag2	right Ventral orbital cortex	0.666	0.039
M	Rag2	right Ventral tenia tecta	0.054	0.004
M	Rag2	third ventricle	1.151	0.076
M	Rag2	trunk of arbor vita	3.972	0.213
M	Rag2	trunk of lobules 1-3 white matter	0.119	0.016
M	Rag2	trunk of lobules 6-8 white matter	0.100	0.006
M	Rag2	ventral tegmental decussation	0.128	0.012

Supplementary Table 5: Preferential expression of all genes in brain regions strongly affected by immune dysfunction. Fold change is calculated as average gene expression signal in brain regions divided by average gene expression signal in whole brain.

Gene	Fold Change	URL
Tcf7l2	2.048	http://api.brain-map.org/grid_data/download/72339557?include=energy
Slitrk6	2.034	http://api.brain-map.org/grid_data/download/73992918?include=energy
Plekhg1	1.975	http://api.brain-map.org/grid_data/download/71670687?include=energy
Gpr6	1.879	http://api.brain-map.org/grid_data/download/71247615?include=energy
Cd4	1.815	http://api.brain-map.org/grid_data/download/74819249?include=energy
Rarb	1.808	http://api.brain-map.org/grid_data/download/75038442?include=energy
Lef1	1.786	http://api.brain-map.org/grid_data/download/77866900?include=energy
Synpo2	1.765	http://api.brain-map.org/grid_data/download/75144656?include=energy
Tnnt1	1.735	http://api.brain-map.org/grid_data/download/77280576?include=energy
Amotl1	1.727	http://api.brain-map.org/grid_data/download/75038463?include=energy
Vmn1r65	1.698	http://api.brain-map.org/grid_data/download/74056185?include=energy
Adora2a	1.686	http://api.brain-map.org/grid_data/download/72109410?include=energy
Pde10a	1.681	http://api.brain-map.org/grid_data/download/71924204?include=energy
Pde7b	1.666	http://api.brain-map.org/grid_data/download/71670683?include=energy
Pde1b	1.659	http://api.brain-map.org/grid_data/download/1257?include=energy
Gm261	1.641	http://api.brain-map.org/grid_data/download/73636032?include=energy
Ntsr1	1.636	http://api.brain-map.org/grid_data/download/73519704?include=energy
Tgfa	1.615	http://api.brain-map.org/grid_data/download/69980304?include=energy
Fam173b	1.609	http://api.brain-map.org/grid_data/download/532757?include=energy
Sema3f	1.608	http://api.brain-map.org/grid_data/download/72007563?include=energy
Serpina9	1.6	http://api.brain-map.org/grid_data/download/71670728?include=energy
Ppp2r2a	1.592	http://api.brain-map.org/grid_data/download/73512208?include=energy
Ric8b	1.583	http://api.brain-map.org/grid_data/download/70301277?include=energy
Gjc1	1.582	http://api.brain-map.org/grid_data/download/77887876?include=energy
4930468A15Rik	1.58	http://api.brain-map.org/grid_data/download/71891737?include=energy
Rgs16	1.576	http://api.brain-map.org/grid_data/download/1567?include=energy
Rgs9	1.558	http://api.brain-map.org/grid_data/download/73521819?include=energy
Tpd52l1	1.555	http://api.brain-map.org/grid_data/download/74357570?include=energy
Rasd2	1.554	http://api.brain-map.org/grid_data/download/73636089?include=energy
Ocm	1.549	http://api.brain-map.org/grid_data/download/71668984?include=energy
Rasgrp2	1.549	http://api.brain-map.org/grid_data/download/76115743?include=energy
Tacr1	1.542	http://api.brain-map.org/grid_data/download/1295?include=energy
Syt9	1.542	http://api.brain-map.org/grid_data/download/73817439?include=energy
Slc17a6	1.536	http://api.brain-map.org/grid_data/download/73818754?include=energy
Gprin3	1.533	http://api.brain-map.org/grid_data/download/73520968?include=energy
Prkcd	1.528	http://api.brain-map.org/grid_data/download/70301274?include=energy
Slc10a4	1.524	http://api.brain-map.org/grid_data/download/71670742?include=energy
Ddc	1.523	http://api.brain-map.org/grid_data/download/79556614?include=energy
Zfhx3	1.519	http://api.brain-map.org/grid_data/download/74641308?include=energy
Oplah	1.516	http://api.brain-map.org/grid_data/download/68203497?include=energy
Vangl1	1.512	http://api.brain-map.org/grid_data/download/71670709?include=energy
Mme	1.499	http://api.brain-map.org/grid_data/download/77371755?include=energy
1810041L15Rik	1.498	http://api.brain-map.org/grid_data/download/72340125?include=energy
Tal1	1.485	http://api.brain-map.org/grid_data/download/72080006?include=energy
Rab37	1.481	http://api.brain-map.org/grid_data/download/73931643?include=energy
4930550L24Rik	1.475	http://api.brain-map.org/grid_data/download/73513275?include=energy
Fcrl1	1.474	http://api.brain-map.org/grid_data/download/69014812?include=energy
Slc18a2	1.473	http://api.brain-map.org/grid_data/download/79591703?include=energy
Gm5	1.458	http://api.brain-map.org/grid_data/download/71529851?include=energy
Slc6a3	1.456	http://api.brain-map.org/grid_data/download/79591365?include=energy
Calml4	1.456	http://api.brain-map.org/grid_data/download/68080684?include=energy
Mrvi1	1.456	http://api.brain-map.org/grid_data/download/71210520?include=energy
Ramp3	1.455	http://api.brain-map.org/grid_data/download/1818?include=energy
Chrna6	1.45	http://api.brain-map.org/grid_data/download/75551461?include=energy
Csrp1	1.449	http://api.brain-map.org/grid_data/download/2129?include=energy
Drd1a	1.445	http://api.brain-map.org/grid_data/download/352?include=energy
Adm2	1.444	http://api.brain-map.org/grid_data/download/71569298?include=energy
Ccdc103	1.439	http://api.brain-map.org/grid_data/download/74634853?include=energy
En1	1.435	http://api.brain-map.org/grid_data/download/74578600?include=energy
Olfr6	1.424	http://api.brain-map.org/grid_data/download/74724241?include=energy
Cd83	1.424	http://api.brain-map.org/grid_data/download/69540623?include=energy
Fign	1.422	http://api.brain-map.org/grid_data/download/72081550?include=energy
Gucy2c	1.416	http://api.brain-map.org/grid_data/download/73992911?include=energy
Rgs2	1.413	http://api.brain-map.org/grid_data/download/73635771?include=energy
Scn4b	1.412	http://api.brain-map.org/grid_data/download/73636111?include=energy
Tktl2	1.409	http://api.brain-map.org/grid_data/download/70724451?include=energy
Gch1	1.408	http://api.brain-map.org/grid_data/download/70813921?include=energy
Strn	1.406	http://api.brain-map.org/grid_data/download/73521824?include=energy
Tph2	1.405	http://api.brain-map.org/grid_data/download/74511925?include=energy
Ephb1	1.403	http://api.brain-map.org/grid_data/download/72077478?include=energy
Ptchd3	1.401	http://api.brain-map.org/grid_data/download/71891731?include=energy
Actl9	1.395	http://api.brain-map.org/grid_data/download/74635011?include=energy
Nup37	1.391	http://api.brain-map.org/grid_data/download/68795447?include=energy
Gpr115	1.388	http://api.brain-map.org/grid_data/download/71587931?include=energy
Epha1	1.388	http://api.brain-map.org/grid_data/download/74640882?include=energy
Lass2	1.387	http://api.brain-map.org/grid_data/download/68196984?include=energy
Inpp4b	1.387	http://api.brain-map.org/grid_data/download/72080054?include=energy
Foxp2	1.387	http://api.brain-map.org/grid_data/download/72079884?include=energy
Thbs4	1.385	http://api.brain-map.org/grid_data/download/112651416?include=energy
Wfdc2	1.384	http://api.brain-map.org/grid_data/download/71919260?include=energy
E030025P04Rik	1.384	http://api.brain-map.org/grid_data/download/71809482?include=energy
9130014G24Rik	1.382	http://api.brain-map.org/grid_data/download/71569115?include=energy
Zdhhc22	1.381	http://api.brain-map.org/grid_data/download/71587745?include=energy
Awat2	1.378	http://api.brain-map.org/grid_data/download/71020992?include=energy
Atp2c1	1.377	http://api.brain-map.org/grid_data/download/71495825?include=energy
mCG113542.1	1.375	http://api.brain-map.org/grid_data/download/73635381?include=energy
Lrtm2	1.372	http://api.brain-map.org/grid_data/download/74819475?include=energy
4632434I11Rik	1.371	http://api.brain-map.org/grid_data/download/73584777?include=energy
Gabra4	1.371	http://api.brain-map.org/grid_data/download/71924402?include=energy
Kcna4	1.368	http://api.brain-map.org/grid_data/download/71529965?include=energy
Ptpn3	1.368	http://api.brain-map.org/grid_data/download/74511860?include=energy
Frmd8	1.367	http://api.brain-map.org/grid_data/download/67978501?include=energy
C130021I20Rik	1.366	http://api.brain-map.org/grid_data/download/74511997?include=energy
TC1541413	1.365	http://api.brain-map.org/grid_data/download/74641209?include=energy
Gbx2	1.365	http://api.brain-map.org/grid_data/download/77868786?include=energy
Lrrk2	1.36	http://api.brain-map.org/grid_data/download/73497609?include=energy
Adcy5	1.36	http://api.brain-map.org/grid_data/download/79?include=energy
Col6a6	1.356	http://api.brain-map.org/grid_data/download/71809521?include=energy
Exd1	1.356	http://api.brain-map.org/grid_data/download/72183009?include=energy
Ddah1	1.355	http://api.brain-map.org/grid_data/download/70431283?include=energy
1700094D03Rik	1.354	http://api.brain-map.org/grid_data/download/74357917?include=energy
Samd5	1.354	http://api.brain-map.org/grid_data/download/69671750?include=energy
Itga5	1.353	http://api.brain-map.org/grid_data/download/74882636?include=energy
Gtf3a	1.352	http://api.brain-map.org/grid_data/download/68162213?include=energy
Gm16294	1.352	http://api.brain-map.org/grid_data/download/70611419?include=energy
Klhl13	1.35	http://api.brain-map.org/grid_data/download/69735861?include=energy
Prc1	1.349	http://api.brain-map.org/grid_data/download/80514004?include=energy
1500015O10Rik	1.349	http://api.brain-map.org/grid_data/download/70429477?include=energy
Wdr52	1.347	http://api.brain-map.org/grid_data/download/70305670?include=energy
Ptprv	1.346	http://api.brain-map.org/grid_data/download/73817436?include=energy
Cachd1	1.345	http://api.brain-map.org/grid_data/download/72119579?include=energy
Tbc1d8	1.345	http://api.brain-map.org/grid_data/download/71836747?include=energy
Cdc42ep2	1.344	http://api.brain-map.org/grid_data/download/68545893?include=energy
Opalin	1.343	http://api.brain-map.org/grid_data/download/69116504?include=energy
Acp5	1.342	http://api.brain-map.org/grid_data/download/70528186?include=energy
Sema6a	1.341	http://api.brain-map.org/grid_data/download/71358651?include=energy
Cdk1	1.341	http://api.brain-map.org/grid_data/download/74431284?include=energy
Slc35f4	1.34	http://api.brain-map.org/grid_data/download/71211495?include=energy
2610018G03Rik	1.339	http://api.brain-map.org/grid_data/download/74047829?include=energy
Lrrc36	1.338	http://api.brain-map.org/grid_data/download/74562528?include=energy
Trpm6	1.337	http://api.brain-map.org/grid_data/download/73907502?include=energy
Cxcl5	1.337	http://api.brain-map.org/grid_data/download/70542966?include=energy
5033414D02Rik	1.334	http://api.brain-map.org/grid_data/download/71763711?include=energy
Slc22a16	1.332	http://api.brain-map.org/grid_data/download/73424226?include=energy
Htr2c	1.332	http://api.brain-map.org/grid_data/download/73636098?include=energy
Ak7	1.33	http://api.brain-map.org/grid_data/download/73870641?include=energy
Txndc8	1.33	http://api.brain-map.org/grid_data/download/71891665?include=energy
Nat8	1.329	http://api.brain-map.org/grid_data/download/74735301?include=energy
Cyld	1.329	http://api.brain-map.org/grid_data/download/73992938?include=energy
Smcr7	1.328	http://api.brain-map.org/grid_data/download/74431047?include=energy
Gpr155	1.328	http://api.brain-map.org/grid_data/download/70562055?include=energy
I920003L04Rik*	1.328	http://api.brain-map.org/grid_data/download/73731346?include=energy
C130023O10Rik	1.327	http://api.brain-map.org/grid_data/download/71808558?include=energy
Drd2	1.326	http://api.brain-map.org/grid_data/download/357?include=energy
A930025A13Rik	1.325	http://api.brain-map.org/grid_data/download/71021007?include=energy
U2af1	1.323	http://api.brain-map.org/grid_data/download/70194019?include=energy
2810416G20Rik	1.323	http://api.brain-map.org/grid_data/download/74580948?include=energy
Pmch	1.321	http://api.brain-map.org/grid_data/download/55?include=energy
Gatm	1.32	http://api.brain-map.org/grid_data/download/1205?include=energy
Iyd	1.319	http://api.brain-map.org/grid_data/download/71015205?include=energy
Kcnj2	1.319	http://api.brain-map.org/grid_data/download/79490118?include=energy
Chmp1b	1.317	http://api.brain-map.org/grid_data/download/76098347?include=energy
Hbb-y	1.317	http://api.brain-map.org/grid_data/download/69734929?include=energy
Gm5606	1.316	http://api.brain-map.org/grid_data/download/70787660?include=energy
Klhl1	1.316	http://api.brain-map.org/grid_data/download/73520995?include=energy
Ano2	1.316	http://api.brain-map.org/grid_data/download/71587834?include=energy
Mog	1.315	http://api.brain-map.org/grid_data/download/70946633?include=energy
Pigl	1.314	http://api.brain-map.org/grid_data/download/71920495?include=energy
Cldn11	1.309	http://api.brain-map.org/grid_data/download/77280580?include=energy
Olfr131	1.307	http://api.brain-map.org/grid_data/download/73635580?include=energy
Gabrd	1.306	http://api.brain-map.org/grid_data/download/80342297?include=energy
A630082N15Rik	1.303	http://api.brain-map.org/grid_data/download/71494702?include=energy
Zfhx4	1.301	http://api.brain-map.org/grid_data/download/74988739?include=energy
Dach1	1.3	http://api.brain-map.org/grid_data/download/74047769?include=energy
Epha8	1.298	http://api.brain-map.org/grid_data/download/72472794?include=energy
Cryge	1.298	http://api.brain-map.org/grid_data/download/68919970?include=energy
Krtap5-2	1.298	http://api.brain-map.org/grid_data/download/71891575?include=energy
Zfp384	1.297	http://api.brain-map.org/grid_data/download/74510152?include=energy
Slc6a4	1.295	http://api.brain-map.org/grid_data/download/79591679?include=energy
Chrnb3	1.293	http://api.brain-map.org/grid_data/download/70436747?include=energy
Bcl11b	1.293	http://api.brain-map.org/grid_data/download/74990505?include=energy
Bnc2	1.291	http://api.brain-map.org/grid_data/download/73636164?include=energy
mCG1049263	1.29	http://api.brain-map.org/grid_data/download/71022942?include=energy
Rfpl3s	1.29	http://api.brain-map.org/grid_data/download/71891970?include=energy
Tmprss3	1.289	http://api.brain-map.org/grid_data/download/77464850?include=energy
Crebzf	1.288	http://api.brain-map.org/grid_data/download/70813359?include=energy
Vmn2r7	1.287	http://api.brain-map.org/grid_data/download/73606464?include=energy
Rrm2	1.287	http://api.brain-map.org/grid_data/download/74272039?include=energy
Sytl5	1.287	http://api.brain-map.org/grid_data/download/73930809?include=energy
Angpt4	1.287	http://api.brain-map.org/grid_data/download/72471743?include=energy
Il18rap	1.287	http://api.brain-map.org/grid_data/download/71529941?include=energy
Adcy7	1.285	http://api.brain-map.org/grid_data/download/79488953?include=energy
Ntng1	1.284	http://api.brain-map.org/grid_data/download/71924185?include=energy
Gast	1.283	http://api.brain-map.org/grid_data/download/68743895?include=energy
Gm16516	1.283	http://api.brain-map.org/grid_data/download/68444660?include=energy
Pard3	1.282	http://api.brain-map.org/grid_data/download/73992915?include=energy
Carhsp1	1.282	http://api.brain-map.org/grid_data/download/73512493?include=energy
F830209M14Rik*	1.282	http://api.brain-map.org/grid_data/download/73748704?include=energy
Znrf3	1.281	http://api.brain-map.org/grid_data/download/70231254?include=energy
Gpr88	1.281	http://api.brain-map.org/grid_data/download/79567811?include=energy
Spock3	1.281	http://api.brain-map.org/grid_data/download/70634298?include=energy
G530126L13Rik*	1.28	http://api.brain-map.org/grid_data/download/73767356?include=energy
LOC433198	1.28	http://api.brain-map.org/grid_data/download/70723002?include=energy
Tas2r129	1.28	http://api.brain-map.org/grid_data/download/70302372?include=energy
C130060K24Rik	1.278	http://api.brain-map.org/grid_data/download/1164?include=energy
Ntrk1	1.278	http://api.brain-map.org/grid_data/download/71670679?include=energy
Iah1	1.278	http://api.brain-map.org/grid_data/download/69116560?include=energy
Slc29a1	1.276	http://api.brain-map.org/grid_data/download/1838?include=energy
1110059M19Rik	1.276	http://api.brain-map.org/grid_data/download/71232593?include=energy
Gckr	1.276	http://api.brain-map.org/grid_data/download/74274683?include=energy
Kif6	1.275	http://api.brain-map.org/grid_data/download/69735807?include=energy
Gpx1	1.274	http://api.brain-map.org/grid_data/download/74003380?include=energy
Ergic2	1.274	http://api.brain-map.org/grid_data/download/70919621?include=energy
Ctnna1	1.274	http://api.brain-map.org/grid_data/download/77455062?include=energy
Phlda3	1.273	http://api.brain-map.org/grid_data/download/70473945?include=energy
Gsto2	1.273	http://api.brain-map.org/grid_data/download/68162209?include=energy
Grb10	1.272	http://api.brain-map.org/grid_data/download/74750075?include=energy
4930451C15Rik	1.272	http://api.brain-map.org/grid_data/download/74750011?include=energy
Plekhh1	1.271	http://api.brain-map.org/grid_data/download/70300857?include=energy
1600029I14Rik	1.27	http://api.brain-map.org/grid_data/download/71919212?include=energy
Tbc1d25	1.27	http://api.brain-map.org/grid_data/download/69782527?include=energy
F2r	1.269	http://api.brain-map.org/grid_data/download/1711?include=energy
Akap2	1.269	http://api.brain-map.org/grid_data/download/1448?include=energy
Dbndd1	1.268	http://api.brain-map.org/grid_data/download/68745251?include=energy
Maoa	1.268	http://api.brain-map.org/grid_data/download/74750015?include=energy
1700029I15Rik	1.267	http://api.brain-map.org/grid_data/download/74749223?include=energy
Vmn1r14	1.265	http://api.brain-map.org/grid_data/download/70785273?include=energy
Necab2	1.264	http://api.brain-map.org/grid_data/download/73788010?include=energy
Gm766	1.263	http://api.brain-map.org/grid_data/download/71764443?include=energy
LOC383700	1.263	http://api.brain-map.org/grid_data/download/74736141?include=energy
Speer3	1.262	http://api.brain-map.org/grid_data/download/71892119?include=energy
Tmem56	1.261	http://api.brain-map.org/grid_data/download/68666667?include=energy
Fam109b	1.261	http://api.brain-map.org/grid_data/download/71021052?include=energy
Cdh6	1.261	http://api.brain-map.org/grid_data/download/238?include=energy
Hcrtr1	1.259	http://api.brain-map.org/grid_data/download/77868713?include=energy
Cd82	1.258	http://api.brain-map.org/grid_data/download/69735676?include=energy
4921531P07Rik	1.258	http://api.brain-map.org/grid_data/download/71670715?include=energy
Galnt6	1.258	http://api.brain-map.org/grid_data/download/74882595?include=energy
LOC435511	1.258	http://api.brain-map.org/grid_data/download/70725652?include=energy
A530079E22Rik	1.258	http://api.brain-map.org/grid_data/download/71250305?include=energy
D630033O11Rik	1.257	http://api.brain-map.org/grid_data/download/70238704?include=energy
Ace	1.256	http://api.brain-map.org/grid_data/download/72109489?include=energy
Prr11	1.256	http://api.brain-map.org/grid_data/download/68161471?include=energy
Dlx6	1.256	http://api.brain-map.org/grid_data/download/77800885?include=energy
Fhdc1	1.255	http://api.brain-map.org/grid_data/download/73925717?include=energy
Isl1	1.254	http://api.brain-map.org/grid_data/download/596?include=energy
Mfap5	1.254	http://api.brain-map.org/grid_data/download/71735836?include=energy
Spats1	1.253	http://api.brain-map.org/grid_data/download/77924440?include=energy
B630019K06Rik	1.253	http://api.brain-map.org/grid_data/download/74881160?include=energy
Rps6ka5	1.252	http://api.brain-map.org/grid_data/download/79904751?include=energy
Vmn1r87	1.252	http://api.brain-map.org/grid_data/download/70785714?include=energy
Ermn	1.252	http://api.brain-map.org/grid_data/download/69448632?include=energy
Slc17a8	1.251	http://api.brain-map.org/grid_data/download/71587918?include=energy
Grid2ip	1.251	http://api.brain-map.org/grid_data/download/71670479?include=energy
Lrdd	1.25	http://api.brain-map.org/grid_data/download/71920430?include=energy
Hspb8	1.25	http://api.brain-map.org/grid_data/download/1181?include=energy
Aspa	1.25	http://api.brain-map.org/grid_data/download/77371803?include=energy
Plekhb1	1.25	http://api.brain-map.org/grid_data/download/71836842?include=energy
Krt9	1.249	http://api.brain-map.org/grid_data/download/71670723?include=energy
Mal	1.248	http://api.brain-map.org/grid_data/download/386243?include=energy
Map2k6	1.248	http://api.brain-map.org/grid_data/download/73817433?include=energy
1700003F12Rik	1.247	http://api.brain-map.org/grid_data/download/74357797?include=energy
Apol10b	1.247	http://api.brain-map.org/grid_data/download/70724484?include=energy
Cox18	1.247	http://api.brain-map.org/grid_data/download/71495938?include=energy
Chrna4	1.247	http://api.brain-map.org/grid_data/download/1173?include=energy
H60a	1.245	http://api.brain-map.org/grid_data/download/71723802?include=energy
Avp	1.245	http://api.brain-map.org/grid_data/download/131?include=energy
Lbp	1.244	http://api.brain-map.org/grid_data/download/68191384?include=energy
Htr1b	1.244	http://api.brain-map.org/grid_data/download/584?include=energy
Lrrc23	1.244	http://api.brain-map.org/grid_data/download/69114516?include=energy
Lhfpl3	1.242	http://api.brain-map.org/grid_data/download/73635972?include=energy
Ero1lb	1.242	http://api.brain-map.org/grid_data/download/72340145?include=energy
Chst3	1.242	http://api.brain-map.org/grid_data/download/69014500?include=energy
Acaa2	1.241	http://api.brain-map.org/grid_data/download/71924375?include=energy
Vwa3a	1.241	http://api.brain-map.org/grid_data/download/72472789?include=energy
Zswim6	1.241	http://api.brain-map.org/grid_data/download/75146003?include=energy
LOC381980	1.241	http://api.brain-map.org/grid_data/download/70596155?include=energy
Krtap5-3	1.24	http://api.brain-map.org/grid_data/download/70228322?include=energy
Mobp	1.239	http://api.brain-map.org/grid_data/download/69201510?include=energy
Cog8	1.239	http://api.brain-map.org/grid_data/download/68157409?include=energy
Srgap1	1.239	http://api.brain-map.org/grid_data/download/74047745?include=energy
Surf6	1.239	http://api.brain-map.org/grid_data/download/678719?include=energy
Foxo1	1.238	http://api.brain-map.org/grid_data/download/71249055?include=energy

Mag	1.238	http://api.brain-map.org/grid_data/download/69736418?include=energy
B4galt1	1.237	http://api.brain-map.org/grid_data/download/71063735?include=energy
Slc25a13	1.236	http://api.brain-map.org/grid_data/download/78534504?include=energy
Oca2	1.236	http://api.brain-map.org/grid_data/download/71381097?include=energy
Lhx8	1.235	http://api.brain-map.org/grid_data/download/2379?include=energy
Nipsnap3a	1.235	http://api.brain-map.org/grid_data/download/74635035?include=energy
A730049H05Rik	1.234	http://api.brain-map.org/grid_data/download/69446274?include=energy
Oprm1	1.234	http://api.brain-map.org/grid_data/download/79912572?include=energy
Tmsb15l	1.233	http://api.brain-map.org/grid_data/download/71764892?include=energy
A730089K16Rik	1.233	http://api.brain-map.org/grid_data/download/69449008?include=energy
Adamts19	1.233	http://api.brain-map.org/grid_data/download/73635973?include=energy
Acbd7	1.233	http://api.brain-map.org/grid_data/download/71892430?include=energy
Slc4a2	1.232	http://api.brain-map.org/grid_data/download/74583129?include=energy
6030429G01Rik	1.232	http://api.brain-map.org/grid_data/download/72471746?include=energy
Arhgef40	1.232	http://api.brain-map.org/grid_data/download/69671720?include=energy
Ifi27l2b	1.231	http://api.brain-map.org/grid_data/download/70723644?include=energy
Olfr820	1.23	http://api.brain-map.org/grid_data/download/74734950?include=energy
6330503K22Rik	1.23	http://api.brain-map.org/grid_data/download/77280311?include=energy
Bnip2	1.23	http://api.brain-map.org/grid_data/download/68301216?include=energy
9130401M01Rik	1.229	http://api.brain-map.org/grid_data/download/70717374?include=energy
Krtap2-4	1.229	http://api.brain-map.org/grid_data/download/74580817?include=energy
Zfp945	1.229	http://api.brain-map.org/grid_data/download/70717976?include=energy
Ggh	1.228	http://api.brain-map.org/grid_data/download/69112406?include=energy
Nrsn2	1.228	http://api.brain-map.org/grid_data/download/79912548?include=energy
S1pr5	1.228	http://api.brain-map.org/grid_data/download/112651200?include=energy
Otx2	1.227	http://api.brain-map.org/grid_data/download/112194935?include=energy
Slc12a2	1.227	http://api.brain-map.org/grid_data/download/74882926?include=energy
Wnk3-ps	1.227	http://api.brain-map.org/grid_data/download/69817342?include=energy
Rasd1	1.227	http://api.brain-map.org/grid_data/download/2521?include=energy
Serpinb1a	1.226	http://api.brain-map.org/grid_data/download/68302350?include=energy
Abhd12b	1.226	http://api.brain-map.org/grid_data/download/75043599?include=energy
Brix1	1.226	http://api.brain-map.org/grid_data/download/71152485?include=energy
Kcne4	1.226	http://api.brain-map.org/grid_data/download/74819245?include=energy
Zfp672	1.226	http://api.brain-map.org/grid_data/download/71891826?include=energy
4930562A09Rik	1.226	http://api.brain-map.org/grid_data/download/70218384?include=energy
Mm.247024	1.226	http://api.brain-map.org/grid_data/download/71456144?include=energy
Gpr4	1.225	http://api.brain-map.org/grid_data/download/74724680?include=energy
Rsph9	1.225	http://api.brain-map.org/grid_data/download/671226?include=energy
Cyb561d2	1.225	http://api.brain-map.org/grid_data/download/68633473?include=energy
Ankrd24	1.225	http://api.brain-map.org/grid_data/download/80343396?include=energy
Maob	1.225	http://api.brain-map.org/grid_data/download/71670489?include=energy
Trim12a	1.225	http://api.brain-map.org/grid_data/download/74056145?include=energy
Pacs2	1.224	http://api.brain-map.org/grid_data/download/73635816?include=energy
Pglyrp1	1.224	http://api.brain-map.org/grid_data/download/104595?include=energy
LOC433436	1.224	http://api.brain-map.org/grid_data/download/73636096?include=energy
Mapk15	1.224	http://api.brain-map.org/grid_data/download/69835585?include=energy
Tug1	1.223	http://api.brain-map.org/grid_data/download/73712774?include=energy
Gpr116	1.223	http://api.brain-map.org/grid_data/download/73907496?include=energy
Slc44a1	1.223	http://api.brain-map.org/grid_data/download/77925099?include=energy
Postn	1.223	http://api.brain-map.org/grid_data/download/77371799?include=energy
Lss	1.223	http://api.brain-map.org/grid_data/download/68444636?include=energy
Lgi3	1.223	http://api.brain-map.org/grid_data/download/73520996?include=energy
Olfr314	1.223	http://api.brain-map.org/grid_data/download/74720985?include=energy
Murc	1.222	http://api.brain-map.org/grid_data/download/74635343?include=energy
Ttc21a	1.222	http://api.brain-map.org/grid_data/download/69407893?include=energy
Nrip2	1.221	http://api.brain-map.org/grid_data/download/69816655?include=energy
Olfr1184	1.221	http://api.brain-map.org/grid_data/download/73635550?include=energy
Gm568	1.221	http://api.brain-map.org/grid_data/download/71529857?include=energy
2610019F03Rik	1.221	http://api.brain-map.org/grid_data/download/74724511?include=energy
Tmem63a	1.221	http://api.brain-map.org/grid_data/download/68637838?include=energy
Olfr649	1.22	http://api.brain-map.org/grid_data/download/74734788?include=energy
Gm98	1.22	http://api.brain-map.org/grid_data/download/70295805?include=energy
4933417A18Rik	1.219	http://api.brain-map.org/grid_data/download/71892071?include=energy
2010107H07Rik	1.218	http://api.brain-map.org/grid_data/download/73512517?include=energy
Fa2h	1.218	http://api.brain-map.org/grid_data/download/74882584?include=energy
Cpxm2	1.217	http://api.brain-map.org/grid_data/download/532761?include=energy
Sla2	1.217	http://api.brain-map.org/grid_data/download/74300687?include=energy
Extl3	1.216	http://api.brain-map.org/grid_data/download/411?include=energy
Dnaic1	1.216	http://api.brain-map.org/grid_data/download/70238902?include=energy
Olfr558	1.216	http://api.brain-map.org/grid_data/download/73653440?include=energy
Tmem164	1.215	http://api.brain-map.org/grid_data/download/68844343?include=energy
Ttyh2	1.215	http://api.brain-map.org/grid_data/download/70946147?include=energy
Sun2	1.214	http://api.brain-map.org/grid_data/download/74658032?include=energy
LOC239690	1.214	http://api.brain-map.org/grid_data/download/74736075?include=energy
Insl6	1.214	http://api.brain-map.org/grid_data/download/69549175?include=energy
Pde3a	1.214	http://api.brain-map.org/grid_data/download/71717083?include=energy
Tprn	1.214	http://api.brain-map.org/grid_data/download/69531007?include=energy
Gpr151	1.214	http://api.brain-map.org/grid_data/download/74724649?include=energy
Pkib	1.213	http://api.brain-map.org/grid_data/download/69783238?include=energy
Meis2	1.213	http://api.brain-map.org/grid_data/download/1231?include=energy
Mpl	1.213	http://api.brain-map.org/grid_data/download/69289405?include=energy
Acvr1c	1.212	http://api.brain-map.org/grid_data/download/71250307?include=energy
LOC381355	1.212	http://api.brain-map.org/grid_data/download/71924491?include=energy
Car13	1.212	http://api.brain-map.org/grid_data/download/71021066?include=energy
Gpr83	1.212	http://api.brain-map.org/grid_data/download/72338696?include=energy
1700018L24Rik	1.212	http://api.brain-map.org/grid_data/download/74634791?include=energy
Rgs3	1.211	http://api.brain-map.org/grid_data/download/1822?include=energy
4930404H21Rik	1.211	http://api.brain-map.org/grid_data/download/71891659?include=energy
Plxnb3	1.211	http://api.brain-map.org/grid_data/download/1265?include=energy
Sh3d19	1.211	http://api.brain-map.org/grid_data/download/74425516?include=energy
Glra3	1.211	http://api.brain-map.org/grid_data/download/73788474?include=energy
LOC436359	1.21	http://api.brain-map.org/grid_data/download/74736336?include=energy
Psat1	1.21	http://api.brain-map.org/grid_data/download/76081936?include=energy
Plekha7	1.21	http://api.brain-map.org/grid_data/download/73992710?include=energy
Snx29	1.21	http://api.brain-map.org/grid_data/download/71892238?include=energy
Rhox10	1.21	http://api.brain-map.org/grid_data/download/71764506?include=energy
Pou3f4	1.21	http://api.brain-map.org/grid_data/download/77924474?include=energy
Ccdc88c	1.209	http://api.brain-map.org/grid_data/download/74363346?include=energy
Gm6792	1.209	http://api.brain-map.org/grid_data/download/73870605?include=energy
4930420G21Rik	1.209	http://api.brain-map.org/grid_data/download/71809509?include=energy
Gm1587	1.208	http://api.brain-map.org/grid_data/download/74578371?include=energy
LOC433354	1.208	http://api.brain-map.org/grid_data/download/70723187?include=energy
Mtrr	1.208	http://api.brain-map.org/grid_data/download/2413?include=energy
4933411O13Rik	1.208	http://api.brain-map.org/grid_data/download/74265231?include=energy
Amtn	1.208	http://api.brain-map.org/grid_data/download/71892256?include=energy
Slc39a4	1.208	http://api.brain-map.org/grid_data/download/68203442?include=energy
Sox14	1.208	http://api.brain-map.org/grid_data/download/73636036?include=energy
Gpr82	1.207	http://api.brain-map.org/grid_data/download/77464858?include=energy
Arsg	1.207	http://api.brain-map.org/grid_data/download/308054556?include=energy
Gng7	1.207	http://api.brain-map.org/grid_data/download/1734?include=energy
Ankrd44	1.207	http://api.brain-map.org/grid_data/download/74277688?include=energy
Entpd3	1.206	http://api.brain-map.org/grid_data/download/74511804?include=energy
Crybb2	1.206	http://api.brain-map.org/grid_data/download/69608082?include=energy
Arhgef10	1.205	http://api.brain-map.org/grid_data/download/77280329?include=energy
Id4	1.205	http://api.brain-map.org/grid_data/download/74357547?include=energy
Vav3	1.205	http://api.brain-map.org/grid_data/download/76085746?include=energy
Olfr655	1.205	http://api.brain-map.org/grid_data/download/74734800?include=energy
Acy1	1.204	http://api.brain-map.org/grid_data/download/100146049?include=energy
Prss8	1.204	http://api.brain-map.org/grid_data/download/68443274?include=energy
LOC433388	1.204	http://api.brain-map.org/grid_data/download/74431506?include=energy
Tspan2	1.203	http://api.brain-map.org/grid_data/download/75144618?include=energy
LOC433252	1.203	http://api.brain-map.org/grid_data/download/70725531?include=energy
Nudt10	1.202	http://api.brain-map.org/grid_data/download/71022900?include=energy
Ndc80	1.201	http://api.brain-map.org/grid_data/download/71763441?include=energy
2810021J22Rik	1.201	http://api.brain-map.org/grid_data/download/68918970?include=energy
6330410L21Rik	1.2	http://api.brain-map.org/grid_data/download/71210472?include=energy
1700063K16Rik	1.2	http://api.brain-map.org/grid_data/download/73497711?include=energy
Grm1	1.2	http://api.brain-map.org/grid_data/download/537?include=energy
Rgs20	1.2	http://api.brain-map.org/grid_data/download/73817437?include=energy
Vmn2r-ps11	1.199	http://api.brain-map.org/grid_data/download/71764904?include=energy
Sfmbt2	1.199	http://api.brain-map.org/grid_data/download/73929593?include=energy
Stk32a	1.199	http://api.brain-map.org/grid_data/download/71920510?include=energy
Mthfs	1.199	http://api.brain-map.org/grid_data/download/71763810?include=energy
1700026L06Rik	1.199	http://api.brain-map.org/grid_data/download/69116373?include=energy
Agt	1.199	http://api.brain-map.org/grid_data/download/87?include=energy
Pik3c2b	1.198	http://api.brain-map.org/grid_data/download/71809067?include=energy
Trim59	1.198	http://api.brain-map.org/grid_data/download/79544788?include=energy
LOC384276	1.198	http://api.brain-map.org/grid_data/download/70725133?include=energy
B230380O10Rik*	1.198	http://api.brain-map.org/grid_data/download/73811591?include=energy
Abcb4	1.198	http://api.brain-map.org/grid_data/download/69514588?include=energy
Vmn1r73	1.198	http://api.brain-map.org/grid_data/download/70785654?include=energy
Dppa1	1.197	http://api.brain-map.org/grid_data/download/71587589?include=energy
Cnst	1.197	http://api.brain-map.org/grid_data/download/70445562?include=energy
Qdpr	1.197	http://api.brain-map.org/grid_data/download/75042261?include=energy
Ndrg1	1.196	http://api.brain-map.org/grid_data/download/70429303?include=energy
Zswim1	1.196	http://api.brain-map.org/grid_data/download/68080696?include=energy
Srpk3	1.196	http://api.brain-map.org/grid_data/download/71488343?include=energy
Evi2a	1.196	http://api.brain-map.org/grid_data/download/68546137?include=energy
Clcnka	1.196	http://api.brain-map.org/grid_data/download/68191300?include=energy
Prlr	1.196	http://api.brain-map.org/grid_data/download/1268?include=energy
Obox1	1.196	http://api.brain-map.org/grid_data/download/81790668?include=energy
LOC436028	1.195	http://api.brain-map.org/grid_data/download/70784564?include=energy
B230369F24Rik	1.194	http://api.brain-map.org/grid_data/download/74822575?include=energy
Ugt8a	1.194	http://api.brain-map.org/grid_data/download/655116?include=energy
Vrk1	1.193	http://api.brain-map.org/grid_data/download/71724756?include=energy
A330079A07Rik*	1.193	http://api.brain-map.org/grid_data/download/73808558?include=energy
TC1413271	1.192	http://api.brain-map.org/grid_data/download/70793989?include=energy
9130230L23Rik	1.192	http://api.brain-map.org/grid_data/download/71809031?include=energy
Mycn	1.191	http://api.brain-map.org/grid_data/download/79762720?include=energy
Fmn1	1.191	http://api.brain-map.org/grid_data/download/74635029?include=energy
4833408D11Rik	1.191	http://api.brain-map.org/grid_data/download/73635401?include=energy
Lpcat2b	1.191	http://api.brain-map.org/grid_data/download/70205656?include=energy
Ttll10	1.191	http://api.brain-map.org/grid_data/download/72472452?include=energy
Dbh	1.191	http://api.brain-map.org/grid_data/download/326?include=energy
BC002230	1.19	http://api.brain-map.org/grid_data/download/68161469?include=energy
Ffar2	1.19	http://api.brain-map.org/grid_data/download/79544822?include=energy
LOC545665	1.189	http://api.brain-map.org/grid_data/download/74277733?include=energy
Pawr	1.189	http://api.brain-map.org/grid_data/download/71211395?include=energy
Foxp1	1.189	http://api.brain-map.org/grid_data/download/71249056?include=energy
9430097D07Rik	1.189	http://api.brain-map.org/grid_data/download/69446917?include=energy
Nxt2	1.189	http://api.brain-map.org/grid_data/download/69782521?include=energy
Ssxb2	1.189	http://api.brain-map.org/grid_data/download/71662901?include=energy
Col8a1	1.189	http://api.brain-map.org/grid_data/download/70431241?include=energy
Traip	1.188	http://api.brain-map.org/grid_data/download/77887842?include=energy
Dydc1	1.188	http://api.brain-map.org/grid_data/download/72081671?include=energy
Slc6a11	1.188	http://api.brain-map.org/grid_data/download/72472802?include=energy
Zcchc12	1.187	http://api.brain-map.org/grid_data/download/73817424?include=energy
Lcn4	1.187	http://api.brain-map.org/grid_data/download/69736070?include=energy
LOC547047	1.187	http://api.brain-map.org/grid_data/download/74736522?include=energy
Sult2a2	1.186	http://api.brain-map.org/grid_data/download/69887457?include=energy
Acer3	1.186	http://api.brain-map.org/grid_data/download/68161478?include=energy
Pmp22	1.186	http://api.brain-map.org/grid_data/download/825?include=energy
Cntn2	1.186	http://api.brain-map.org/grid_data/download/73497519?include=energy
Gm10413	1.186	http://api.brain-map.org/grid_data/download/73929885?include=energy
Klk1b7-ps	1.186	http://api.brain-map.org/grid_data/download/71920231?include=energy
Gsn	1.185	http://api.brain-map.org/grid_data/download/72081395?include=energy
Nlrp12	1.185	http://api.brain-map.org/grid_data/download/71668983?include=energy
Rnf13	1.185	http://api.brain-map.org/grid_data/download/72109423?include=energy
E030042O20Rik	1.185	http://api.brain-map.org/grid_data/download/71809494?include=energy
Ckap2	1.185	http://api.brain-map.org/grid_data/download/74363353?include=energy
Tinagl1	1.185	http://api.brain-map.org/grid_data/download/68269290?include=energy
B3gnt2	1.185	http://api.brain-map.org/grid_data/download/75988600?include=energy
Eps8l2	1.185	http://api.brain-map.org/grid_data/download/74658244?include=energy
LOC433567	1.184	http://api.brain-map.org/grid_data/download/70785792?include=energy
Gltp	1.184	http://api.brain-map.org/grid_data/download/68341597?include=energy
Klk6	1.183	http://api.brain-map.org/grid_data/download/74988616?include=energy
1700027A23Rik	1.183	http://api.brain-map.org/grid_data/download/69114608?include=energy
Npc1	1.183	http://api.brain-map.org/grid_data/download/71234644?include=energy
Myl6b	1.182	http://api.brain-map.org/grid_data/download/68545875?include=energy
G630005L12Rik*	1.182	http://api.brain-map.org/grid_data/download/73731277?include=energy
Spin4	1.182	http://api.brain-map.org/grid_data/download/71763729?include=energy
2210009G21Rik	1.182	http://api.brain-map.org/grid_data/download/71492940?include=energy
Rab30	1.181	http://api.brain-map.org/grid_data/download/68342288?include=energy
Rwdd2a	1.18	http://api.brain-map.org/grid_data/download/74511895?include=energy
Col11a2	1.18	http://api.brain-map.org/grid_data/download/74581187?include=energy
Ampd3	1.18	http://api.brain-map.org/grid_data/download/77371807?include=energy
Coch	1.179	http://api.brain-map.org/grid_data/download/71717614?include=energy
Olfr551	1.179	http://api.brain-map.org/grid_data/download/74724157?include=energy
Sh3kbp1	1.179	http://api.brain-map.org/grid_data/download/74272906?include=energy
D930014E17Rik	1.179	http://api.brain-map.org/grid_data/download/71529929?include=energy
Dpy19l2	1.178	http://api.brain-map.org/grid_data/download/73808186?include=energy
LOC433611	1.178	http://api.brain-map.org/grid_data/download/70785822?include=energy
H2-Q8	1.178	http://api.brain-map.org/grid_data/download/73716584?include=energy
Glipr2	1.178	http://api.brain-map.org/grid_data/download/71529839?include=energy
Zmat4	1.177	http://api.brain-map.org/grid_data/download/71249750?include=energy
Zim1	1.177	http://api.brain-map.org/grid_data/download/71670692?include=energy
Slc6a16	1.177	http://api.brain-map.org/grid_data/download/78534546?include=energy
Fads6	1.176	http://api.brain-map.org/grid_data/download/69529137?include=energy
Ccm2	1.176	http://api.brain-map.org/grid_data/download/71608344?include=energy
Sncg	1.175	http://api.brain-map.org/grid_data/download/72081426?include=energy
Riiad1	1.175	http://api.brain-map.org/grid_data/download/74749189?include=energy
Lypd6b	1.175	http://api.brain-map.org/grid_data/download/73635774?include=energy
6820408C15Rik	1.175	http://api.brain-map.org/grid_data/download/74363339?include=energy
Il1f5	1.175	http://api.brain-map.org/grid_data/download/71529947?include=energy
Itga3	1.175	http://api.brain-map.org/grid_data/download/598?include=energy
Zfp712	1.174	http://api.brain-map.org/grid_data/download/73635433?include=energy
Zfp618	1.174	http://api.brain-map.org/grid_data/download/71670712?include=energy
Ache	1.173	http://api.brain-map.org/grid_data/download/70301082?include=energy
S100pbp	1.173	http://api.brain-map.org/grid_data/download/74819247?include=energy
Gm520	1.173	http://api.brain-map.org/grid_data/download/74511966?include=energy
Me2	1.173	http://api.brain-map.org/grid_data/download/74658090?include=energy
Sult1c2	1.173	http://api.brain-map.org/grid_data/download/68498651?include=energy
Pla2g2a	1.173	http://api.brain-map.org/grid_data/download/68924521?include=energy
Fbll1	1.172	http://api.brain-map.org/grid_data/download/71764150?include=energy
D730005E14Rik	1.172	http://api.brain-map.org/grid_data/download/74300753?include=energy
Slc8a3	1.172	http://api.brain-map.org/grid_data/download/74357546?include=energy
Klhdc7a	1.172	http://api.brain-map.org/grid_data/download/69527806?include=energy
Paqr5	1.172	http://api.brain-map.org/grid_data/download/69236706?include=energy
B3galt5	1.172	http://api.brain-map.org/grid_data/download/77280578?include=energy
Ppp1r14a	1.171	http://api.brain-map.org/grid_data/download/70546384?include=energy
Plekhg3	1.171	http://api.brain-map.org/grid_data/download/69173232?include=energy
Scd1	1.171	http://api.brain-map.org/grid_data/download/71836746?include=energy
Gm266	1.171	http://api.brain-map.org/grid_data/download/71307358?include=energy
9130227L01Rik	1.17	http://api.brain-map.org/grid_data/download/71809019?include=energy
B230104C08Rik	1.17	http://api.brain-map.org/grid_data/download/71495861?include=energy
Stk24	1.17	http://api.brain-map.org/grid_data/download/73635773?include=energy
Clmn	1.17	http://api.brain-map.org/grid_data/download/73931626?include=energy
A330043C09Rik	1.169	http://api.brain-map.org/grid_data/download/71494672?include=energy
A230065C20Rik	1.169	http://api.brain-map.org/grid_data/download/70228376?include=energy
C230090A06Rik*	1.169	http://api.brain-map.org/grid_data/download/73811687?include=energy
Rab11a	1.169	http://api.brain-map.org/grid_data/download/71724017?include=energy
Gm5553	1.169	http://api.brain-map.org/grid_data/download/74736255?include=energy
A630001O12Rik	1.168	http://api.brain-map.org/grid_data/download/73748710?include=energy
Mcat	1.168	http://api.brain-map.org/grid_data/download/71574516?include=energy
Plch1	1.168	http://api.brain-map.org/grid_data/download/71717604?include=energy
Iqca	1.168	http://api.brain-map.org/grid_data/download/74579987?include=energy
LOC546299	1.168	http://api.brain-map.org/grid_data/download/70218259?include=energy
mCG22988.1	1.168	http://api.brain-map.org/grid_data/download/71213262?include=energy
Tmem88b	1.167	http://api.brain-map.org/grid_data/download/69447978?include=energy
Ptger2	1.167	http://api.brain-map.org/grid_data/download/79534953?include=energy
Apod	1.167	http://api.brain-map.org/grid_data/download/71380893?include=energy
Pgcp	1.167	http://api.brain-map.org/grid_data/download/378547?include=energy
Eif5a2	1.167	http://api.brain-map.org/grid_data/download/73992947?include=energy
F830205C13Rik*	1.166	http://api.brain-map.org/grid_data/download/73731436?include=energy
Galr1	1.166	http://api.brain-map.org/grid_data/download/72283790?include=energy
E130304F04Rik	1.166	http://api.brain-map.org/grid_data/download/69671738?include=energy
Cnp	1.166	http://api.brain-map.org/grid_data/download/77371841?include=energy

Dusp27	1.165	http://api.brain-map.org/grid_data/download/69530846?include=energy
Ifnb1	1.165	http://api.brain-map.org/grid_data/download/69735240?include=energy
Corin	1.165	http://api.brain-map.org/grid_data/download/100144928?include=energy
Ccl1	1.165	http://api.brain-map.org/grid_data/download/69540441?include=energy
Plek2	1.165	http://api.brain-map.org/grid_data/download/68632136?include=energy
Dsg4	1.165	http://api.brain-map.org/grid_data/download/77800909?include=energy
Anxa10	1.164	http://api.brain-map.org/grid_data/download/73606663?include=energy
Olfr424	1.164	http://api.brain-map.org/grid_data/download/73653422?include=energy
Wdr96	1.164	http://api.brain-map.org/grid_data/download/74426862?include=energy
Gtf2ird1	1.164	http://api.brain-map.org/grid_data/download/74510284?include=energy
Pdcd1	1.163	http://api.brain-map.org/grid_data/download/69782897?include=energy
Spata17	1.163	http://api.brain-map.org/grid_data/download/71891838?include=energy
Gnal	1.163	http://api.brain-map.org/grid_data/download/71670716?include=energy
LOC432562	1.163	http://api.brain-map.org/grid_data/download/72076527?include=energy
Hdac10	1.163	http://api.brain-map.org/grid_data/download/74431834?include=energy
Vmn1r78	1.163	http://api.brain-map.org/grid_data/download/70785684?include=energy
Lefty2	1.163	http://api.brain-map.org/grid_data/download/77620828?include=energy
Olfr429	1.163	http://api.brain-map.org/grid_data/download/73653428?include=energy
Arhgap18	1.163	http://api.brain-map.org/grid_data/download/68498597?include=energy
Prkcq	1.163	http://api.brain-map.org/grid_data/download/71670693?include=energy
Trim12c	1.162	http://api.brain-map.org/grid_data/download/68795432?include=energy
Batf	1.162	http://api.brain-map.org/grid_data/download/100145359?include=energy
Olfr2	1.162	http://api.brain-map.org/grid_data/download/74720898?include=energy
Tlr3	1.162	http://api.brain-map.org/grid_data/download/72004438?include=energy
C230029D21Rik	1.162	http://api.brain-map.org/grid_data/download/71496083?include=energy
Sssca1	1.162	http://api.brain-map.org/grid_data/download/2633?include=energy
Tgm2	1.162	http://api.brain-map.org/grid_data/download/69980316?include=energy
Elmo1	1.161	http://api.brain-map.org/grid_data/download/70743907?include=energy
H2-M10.6	1.161	http://api.brain-map.org/grid_data/download/70724804?include=energy
Glrx2	1.161	http://api.brain-map.org/grid_data/download/73513786?include=energy
Mzt1	1.161	http://api.brain-map.org/grid_data/download/69854164?include=energy
4632408I12Rik*	1.161	http://api.brain-map.org/grid_data/download/73870521?include=energy
Prr18	1.161	http://api.brain-map.org/grid_data/download/69447782?include=energy
Rbms3	1.16	http://api.brain-map.org/grid_data/download/75931565?include=energy
Gcnt2	1.16	http://api.brain-map.org/grid_data/download/69838554?include=energy
Olfr1404	1.16	http://api.brain-map.org/grid_data/download/73653308?include=energy
Cldnd2	1.16	http://api.brain-map.org/grid_data/download/74635090?include=energy
Gm4759	1.16	http://api.brain-map.org/grid_data/download/70565903?include=energy
1700006E09Rik	1.16	http://api.brain-map.org/grid_data/download/71919242?include=energy
Slc7a13	1.16	http://api.brain-map.org/grid_data/download/69015673?include=energy
Poc1b	1.16	http://api.brain-map.org/grid_data/download/71486989?include=energy
Isl2	1.16	http://api.brain-map.org/grid_data/download/69289237?include=energy
Cd59a	1.16	http://api.brain-map.org/grid_data/download/73992928?include=energy
Bcl7c	1.159	http://api.brain-map.org/grid_data/download/633284?include=energy
Gjc3	1.159	http://api.brain-map.org/grid_data/download/69257355?include=energy
Tmem17	1.159	http://api.brain-map.org/grid_data/download/68162210?include=energy
LOC638024	1.159	http://api.brain-map.org/grid_data/download/71211401?include=energy
1700008I05Rik	1.159	http://api.brain-map.org/grid_data/download/71919439?include=energy
Ttn	1.158	http://api.brain-map.org/grid_data/download/74047746?include=energy
Acvrl1	1.158	http://api.brain-map.org/grid_data/download/79488929?include=energy
3110082I17Rik	1.158	http://api.brain-map.org/grid_data/download/71656695?include=energy
Mm.173068	1.158	http://api.brain-map.org/grid_data/download/71152210?include=energy
A730024G14Rik*	1.158	http://api.brain-map.org/grid_data/download/74359622?include=energy
Slc18a3	1.158	http://api.brain-map.org/grid_data/download/73521822?include=energy
Adam28	1.158	http://api.brain-map.org/grid_data/download/68798049?include=energy
Ccnd3	1.158	http://api.brain-map.org/grid_data/download/68191468?include=energy
Car2	1.157	http://api.brain-map.org/grid_data/download/72103831?include=energy
Rem2	1.157	http://api.brain-map.org/grid_data/download/70439099?include=energy
A530010F05Rik	1.157	http://api.brain-map.org/grid_data/download/71809271?include=energy
Egflam	1.156	http://api.brain-map.org/grid_data/download/74047441?include=energy
Cpne5	1.156	http://api.brain-map.org/grid_data/download/544709?include=energy
1700040F17Rik	1.156	http://api.brain-map.org/grid_data/download/74357881?include=energy
Lama5	1.156	http://api.brain-map.org/grid_data/download/69736004?include=energy
Cecr2	1.156	http://api.brain-map.org/grid_data/download/71579861?include=energy
Parp11	1.156	http://api.brain-map.org/grid_data/download/68203503?include=energy
A430048G15Rik	1.155	http://api.brain-map.org/grid_data/download/70228412?include=energy
Nags	1.155	http://api.brain-map.org/grid_data/download/69028575?include=energy
Bcr	1.155	http://api.brain-map.org/grid_data/download/69529286?include=energy
1810006K21Rik	1.155	http://api.brain-map.org/grid_data/download/74635126?include=energy
Tmed11	1.155	http://api.brain-map.org/grid_data/download/73818857?include=energy
Guk1	1.155	http://api.brain-map.org/grid_data/download/2279?include=energy
Ugt1a7c	1.155	http://api.brain-map.org/grid_data/download/70302896?include=energy
4932418E24Rik	1.155	http://api.brain-map.org/grid_data/download/73635776?include=energy
Gjd3	1.155	http://api.brain-map.org/grid_data/download/100146044?include=energy
Plod1	1.155	http://api.brain-map.org/grid_data/download/77792637?include=energy
Gm5172	1.154	http://api.brain-map.org/grid_data/download/70725085?include=energy
Arrdc3	1.154	http://api.brain-map.org/grid_data/download/77280426?include=energy
Med29	1.154	http://api.brain-map.org/grid_data/download/199204?include=energy
B230311B06Rik	1.154	http://api.brain-map.org/grid_data/download/72283563?include=energy
Tubb4a	1.154	http://api.brain-map.org/grid_data/download/1081?include=energy
Socs6	1.154	http://api.brain-map.org/grid_data/download/70634236?include=energy
9330178D15Rik	1.153	http://api.brain-map.org/grid_data/download/70305162?include=energy
Mcam	1.153	http://api.brain-map.org/grid_data/download/71836841?include=energy
A730049K10Rik*	1.153	http://api.brain-map.org/grid_data/download/74000498?include=energy
Lrrc16b	1.153	http://api.brain-map.org/grid_data/download/69529071?include=energy
Gpatch4	1.153	http://api.brain-map.org/grid_data/download/587434?include=energy
Gpr110	1.153	http://api.brain-map.org/grid_data/download/79632204?include=energy
Tomm70a	1.153	http://api.brain-map.org/grid_data/download/320?include=energy
H2-M10.4	1.152	http://api.brain-map.org/grid_data/download/70724798?include=energy
Gpr165	1.152	http://api.brain-map.org/grid_data/download/70560278?include=energy
Sema4d	1.152	http://api.brain-map.org/grid_data/download/945?include=energy
2210415F13Rik	1.151	http://api.brain-map.org/grid_data/download/70445437?include=energy
Gchfr	1.151	http://api.brain-map.org/grid_data/download/74511806?include=energy
Msmb	1.151	http://api.brain-map.org/grid_data/download/71020834?include=energy
Sntb1	1.151	http://api.brain-map.org/grid_data/download/71670741?include=energy
Cryab	1.151	http://api.brain-map.org/grid_data/download/74357575?include=energy
Rab25	1.151	http://api.brain-map.org/grid_data/download/633300?include=energy
Gm38	1.151	http://api.brain-map.org/grid_data/download/70295996?include=energy
Ebf3	1.151	http://api.brain-map.org/grid_data/download/73712894?include=energy
LOC432456	1.151	http://api.brain-map.org/grid_data/download/70608529?include=energy
Gad2	1.151	http://api.brain-map.org/grid_data/download/79591669?include=energy
St6galnac2	1.151	http://api.brain-map.org/grid_data/download/71763876?include=energy
Vmn1r83	1.151	http://api.brain-map.org/grid_data/download/70785690?include=energy
Erp27	1.151	http://api.brain-map.org/grid_data/download/70201582?include=energy
Clcn5	1.151	http://api.brain-map.org/grid_data/download/75749741?include=energy
Olig1	1.15	http://api.brain-map.org/grid_data/download/70437828?include=energy
S100a6	1.15	http://api.brain-map.org/grid_data/download/77278967?include=energy
Casc5	1.15	http://api.brain-map.org/grid_data/download/70218703?include=energy
Atf2	1.15	http://api.brain-map.org/grid_data/download/68845807?include=energy
Lect1	1.15	http://api.brain-map.org/grid_data/download/68924853?include=energy
Defb21	1.15	http://api.brain-map.org/grid_data/download/71764898?include=energy
Avpi1	1.15	http://api.brain-map.org/grid_data/download/68744480?include=energy
Hcn2	1.15	http://api.brain-map.org/grid_data/download/73512185?include=energy
Fnta	1.15	http://api.brain-map.org/grid_data/download/2226?include=energy
Nkd1	1.15	http://api.brain-map.org/grid_data/download/74586666?include=energy
Ripk3	1.149	http://api.brain-map.org/grid_data/download/275676?include=energy
1110065P20Rik	1.149	http://api.brain-map.org/grid_data/download/71492790?include=energy
Gzma	1.149	http://api.brain-map.org/grid_data/download/71764119?include=energy
Myo1d	1.149	http://api.brain-map.org/grid_data/download/69782007?include=energy
Slc35d3	1.149	http://api.brain-map.org/grid_data/download/73929607?include=energy
Prdx4	1.148	http://api.brain-map.org/grid_data/download/74819229?include=energy
Plp1	1.148	http://api.brain-map.org/grid_data/download/79556704?include=energy
Sycp1	1.148	http://api.brain-map.org/grid_data/download/72471745?include=energy
Ttr	1.148	http://api.brain-map.org/grid_data/download/68632172?include=energy
4930513O06Rik	1.148	http://api.brain-map.org/grid_data/download/74580026?include=energy
Col25a1	1.147	http://api.brain-map.org/grid_data/download/71924301?include=energy
Nup214	1.147	http://api.brain-map.org/grid_data/download/71161399?include=energy
Tmem110	1.147	http://api.brain-map.org/grid_data/download/71763996?include=energy
Cryba2	1.147	http://api.brain-map.org/grid_data/download/75831743?include=energy
Ece2	1.147	http://api.brain-map.org/grid_data/download/77332686?include=energy
Vat1l	1.147	http://api.brain-map.org/grid_data/download/75650860?include=energy
Igsf10	1.147	http://api.brain-map.org/grid_data/download/69735293?include=energy
Commd1	1.147	http://api.brain-map.org/grid_data/download/70720253?include=energy
Ttf2	1.147	http://api.brain-map.org/grid_data/download/70302860?include=energy
Serpine2	1.147	http://api.brain-map.org/grid_data/download/74047768?include=energy
Klrb1b	1.147	http://api.brain-map.org/grid_data/download/69735891?include=energy
Aatk	1.147	http://api.brain-map.org/grid_data/download/71213880?include=energy
TC1460196	1.146	http://api.brain-map.org/grid_data/download/70795742?include=energy
Ccnd2	1.146	http://api.brain-map.org/grid_data/download/205?include=energy
Nhedc2	1.146	http://api.brain-map.org/grid_data/download/69540248?include=energy
Gm1592	1.146	http://api.brain-map.org/grid_data/download/70298441?include=energy
Gm5092	1.146	http://api.brain-map.org/grid_data/download/70724457?include=energy
Defb1	1.146	http://api.brain-map.org/grid_data/download/73718051?include=energy
Folr1	1.145	http://api.brain-map.org/grid_data/download/71919056?include=energy
Il33	1.145	http://api.brain-map.org/grid_data/download/512763?include=energy
Vmn1r66	1.145	http://api.brain-map.org/grid_data/download/70785291?include=energy
Klrg1	1.145	http://api.brain-map.org/grid_data/download/69735903?include=energy
Olfr610	1.145	http://api.brain-map.org/grid_data/download/74724253?include=energy
Cldn16	1.145	http://api.brain-map.org/grid_data/download/71579891?include=energy
Pitpnc1	1.145	http://api.brain-map.org/grid_data/download/75144644?include=energy
Lamp3	1.145	http://api.brain-map.org/grid_data/download/100144409?include=energy
LOC432915	1.144	http://api.brain-map.org/grid_data/download/70611383?include=energy
5330438D12Rik	1.144	http://api.brain-map.org/grid_data/download/74431393?include=energy
Gpx8	1.144	http://api.brain-map.org/grid_data/download/68410255?include=energy
Grap	1.144	http://api.brain-map.org/grid_data/download/71529875?include=energy
Tmem212	1.144	http://api.brain-map.org/grid_data/download/72076503?include=energy
Cmtm5	1.144	http://api.brain-map.org/grid_data/download/69113692?include=energy
LOC433011	1.144	http://api.brain-map.org/grid_data/download/74431191?include=energy
Slc5a7	1.144	http://api.brain-map.org/grid_data/download/73929608?include=energy
Wscd1	1.144	http://api.brain-map.org/grid_data/download/71920517?include=energy
Fsd1	1.144	http://api.brain-map.org/grid_data/download/69113644?include=energy
Ptgds	1.143	http://api.brain-map.org/grid_data/download/79567709?include=energy
Ap1s2	1.143	http://api.brain-map.org/grid_data/download/71764053?include=energy
Rg9mtd1	1.143	http://api.brain-map.org/grid_data/download/68444648?include=energy
Fam186a	1.143	http://api.brain-map.org/grid_data/download/70298447?include=energy
Rfx6	1.143	http://api.brain-map.org/grid_data/download/74822943?include=energy
Crygd	1.143	http://api.brain-map.org/grid_data/download/72471802?include=energy
Pitx2	1.143	http://api.brain-map.org/grid_data/download/79556607?include=energy
4933404G15Rik	1.143	http://api.brain-map.org/grid_data/download/71892059?include=energy
Riok2	1.142	http://api.brain-map.org/grid_data/download/68302380?include=energy
Abo	1.142	http://api.brain-map.org/grid_data/download/71569292?include=energy
Serpinb1c	1.142	http://api.brain-map.org/grid_data/download/74511967?include=energy
Neurl2	1.142	http://api.brain-map.org/grid_data/download/74581122?include=energy
Phlpp1	1.142	http://api.brain-map.org/grid_data/download/70546366?include=energy
Sftpd	1.142	http://api.brain-map.org/grid_data/download/323165?include=energy
Tppp3	1.142	http://api.brain-map.org/grid_data/download/70813926?include=energy
4833427G06Rik	1.142	http://api.brain-map.org/grid_data/download/70525769?include=energy
Mrgprb8	1.141	http://api.brain-map.org/grid_data/download/70784654?include=energy
Igfals	1.141	http://api.brain-map.org/grid_data/download/69735275?include=energy
Spag4	1.141	http://api.brain-map.org/grid_data/download/72002064?include=energy
Gm626	1.141	http://api.brain-map.org/grid_data/download/73931634?include=energy
Zpbp2	1.141	http://api.brain-map.org/grid_data/download/73423663?include=energy
Zfp467	1.141	http://api.brain-map.org/grid_data/download/68203498?include=energy
Pax5	1.14	http://api.brain-map.org/grid_data/download/69289677?include=energy
Mgp	1.14	http://api.brain-map.org/grid_data/download/1229?include=energy
Mtmr2	1.14	http://api.brain-map.org/grid_data/download/69549617?include=energy
Lrrc9	1.139	http://api.brain-map.org/grid_data/download/71891647?include=energy
Myom1	1.139	http://api.brain-map.org/grid_data/download/77455013?include=energy
Olfr681	1.139	http://api.brain-map.org/grid_data/download/74734836?include=energy
1700009P17Rik	1.139	http://api.brain-map.org/grid_data/download/69174064?include=energy
Pcp4	1.139	http://api.brain-map.org/grid_data/download/772?include=energy
Gna12	1.139	http://api.brain-map.org/grid_data/download/71529869?include=energy
4930588K23Rik	1.139	http://api.brain-map.org/grid_data/download/73906533?include=energy
Rgs17	1.139	http://api.brain-map.org/grid_data/download/73512204?include=energy
LOC432871	1.139	http://api.brain-map.org/grid_data/download/70611353?include=energy
Vmn1r54	1.138	http://api.brain-map.org/grid_data/download/74056166?include=energy
Plin3	1.138	http://api.brain-map.org/grid_data/download/71763468?include=energy
Ccdc78	1.138	http://api.brain-map.org/grid_data/download/70298483?include=energy
Arrdc1	1.138	http://api.brain-map.org/grid_data/download/68341710?include=energy
Gpr37	1.138	http://api.brain-map.org/grid_data/download/2271?include=energy
Gpr156	1.138	http://api.brain-map.org/grid_data/download/74641322?include=energy
Timp3	1.138	http://api.brain-map.org/grid_data/download/73769324?include=energy
LOC385068	1.138	http://api.brain-map.org/grid_data/download/74821974?include=energy
Wfdc9	1.138	http://api.brain-map.org/grid_data/download/73927745?include=energy
Trim8	1.138	http://api.brain-map.org/grid_data/download/72004448?include=energy
S100a16	1.137	http://api.brain-map.org/grid_data/download/77371787?include=energy
Zcchc10	1.137	http://api.brain-map.org/grid_data/download/71724475?include=energy
Vcam1	1.137	http://api.brain-map.org/grid_data/download/77414897?include=energy
Lpar1	1.137	http://api.brain-map.org/grid_data/download/79556595?include=energy
LOC432648	1.137	http://api.brain-map.org/grid_data/download/72081621?include=energy
Gm609	1.137	http://api.brain-map.org/grid_data/download/70546239?include=energy
9030407P20Rik	1.137	http://api.brain-map.org/grid_data/download/71212156?include=energy
Ddr1	1.137	http://api.brain-map.org/grid_data/download/72472787?include=energy
1190028D05Rik	1.136	http://api.brain-map.org/grid_data/download/74581169?include=energy
Fam189a2	1.136	http://api.brain-map.org/grid_data/download/73512353?include=energy
Olfr750	1.136	http://api.brain-map.org/grid_data/download/74734938?include=energy
Dnali1	1.136	http://api.brain-map.org/grid_data/download/71021169?include=energy
Maged2	1.136	http://api.brain-map.org/grid_data/download/71836892?include=energy
Pdzd8	1.136	http://api.brain-map.org/grid_data/download/74047716?include=energy
Fah	1.136	http://api.brain-map.org/grid_data/download/67765699?include=energy
LOC381076	1.136	http://api.brain-map.org/grid_data/download/72338724?include=energy
Pdcl2	1.135	http://api.brain-map.org/grid_data/download/69114471?include=energy
9930038B18Rik	1.135	http://api.brain-map.org/grid_data/download/71668372?include=energy
Bcas1	1.135	http://api.brain-map.org/grid_data/download/77371831?include=energy
Limk2	1.135	http://api.brain-map.org/grid_data/download/61002?include=energy
Rassf1	1.135	http://api.brain-map.org/grid_data/download/68076559?include=energy
Xylt2	1.134	http://api.brain-map.org/grid_data/download/67815956?include=energy
Ebf4	1.134	http://api.brain-map.org/grid_data/download/71020443?include=energy
Ccr2	1.134	http://api.brain-map.org/grid_data/download/69237011?include=energy
Kdr	1.134	http://api.brain-map.org/grid_data/download/2347?include=energy
Shb	1.134	http://api.brain-map.org/grid_data/download/71670695?include=energy
9330003I22Rik*	1.134	http://api.brain-map.org/grid_data/download/73949424?include=energy
Ufsp1	1.134	http://api.brain-map.org/grid_data/download/72007932?include=energy
Frmd4b	1.134	http://api.brain-map.org/grid_data/download/69174541?include=energy
Gbp6	1.134	http://api.brain-map.org/grid_data/download/69028659?include=energy
A830029E22Rik	1.134	http://api.brain-map.org/grid_data/download/70717979?include=energy
Cd209d	1.133	http://api.brain-map.org/grid_data/download/70724637?include=energy
4933402J07Rik	1.133	http://api.brain-map.org/grid_data/download/74514055?include=energy
G530133B12Rik*	1.133	http://api.brain-map.org/grid_data/download/73808165?include=energy
Gm9871	1.133	http://api.brain-map.org/grid_data/download/72004515?include=energy
7230401B09Rik*	1.133	http://api.brain-map.org/grid_data/download/73927676?include=energy
Hist1h1e	1.133	http://api.brain-map.org/grid_data/download/74735343?include=energy
Adam23	1.133	http://api.brain-map.org/grid_data/download/69514768?include=energy
Zswim5	1.133	http://api.brain-map.org/grid_data/download/72004505?include=energy
2810407A14Rik	1.133	http://api.brain-map.org/grid_data/download/71809313?include=energy
9530002B09Rik	1.132	http://api.brain-map.org/grid_data/download/71892406?include=energy
Pdyn	1.132	http://api.brain-map.org/grid_data/download/71717084?include=energy
Rnf180	1.132	http://api.brain-map.org/grid_data/download/68744450?include=energy
4930538K18Rik	1.132	http://api.brain-map.org/grid_data/download/71763700?include=energy
C2cd2	1.132	http://api.brain-map.org/grid_data/download/1986?include=energy
Fshr	1.132	http://api.brain-map.org/grid_data/download/71307340?include=energy
LOC436568	1.131	http://api.brain-map.org/grid_data/download/74736384?include=energy
LOC433555	1.131	http://api.brain-map.org/grid_data/download/70785780?include=energy
Ucp2	1.131	http://api.brain-map.org/grid_data/download/68300980?include=energy
Trpd52l3	1.131	http://api.brain-map.org/grid_data/download/70218474?include=energy
Cdnf	1.131	http://api.brain-map.org/grid_data/download/70305156?include=energy
Hs6st2	1.131	http://api.brain-map.org/grid_data/download/72129255?include=energy
Kcnj12	1.131	http://api.brain-map.org/grid_data/download/74882646?include=energy
Vmn1r80	1.131	http://api.brain-map.org/grid_data/download/70785660?include=energy
Gm884	1.131	http://api.brain-map.org/grid_data/download/70725061?include=energy
Plcxd3	1.131	http://api.brain-map.org/grid_data/download/69527758?include=energy
Otos	1.131	http://api.brain-map.org/grid_data/download/69782611?include=energy
C230081A13Rik	1.13	http://api.brain-map.org/grid_data/download/73931624?include=energy
4931429P17Rik	1.13	http://api.brain-map.org/grid_data/download/74265219?include=energy
Pros1	1.13	http://api.brain-map.org/grid_data/download/69817360?include=energy
Sema4g	1.13	http://api.brain-map.org/grid_data/download/71587856?include=energy
C1qtnf2	1.13	http://api.brain-map.org/grid_data/download/68162211?include=energy

Nap1l2	1.13	http://api.brain-map.org/grid_data/download/71234531?include=energy
5530401A14Rik	1.13	http://api.brain-map.org/grid_data/download/71892286?include=energy
Onecut1	1.13	http://api.brain-map.org/grid_data/download/71213292?include=energy
4921517D22Rik	1.13	http://api.brain-map.org/grid_data/download/70205644?include=energy
Sept4	1.13	http://api.brain-map.org/grid_data/download/69113542?include=energy
Fzd4	1.13	http://api.brain-map.org/grid_data/download/2241?include=energy
Olfr722	1.13	http://api.brain-map.org/grid_data/download/73653500?include=energy
Comt	1.13	http://api.brain-map.org/grid_data/download/68301371?include=energy
Eps15l1	1.13	http://api.brain-map.org/grid_data/download/2193?include=energy
Bdh2	1.129	http://api.brain-map.org/grid_data/download/70806049?include=energy
Ambn	1.129	http://api.brain-map.org/grid_data/download/69526576?include=energy
Sdc2	1.129	http://api.brain-map.org/grid_data/download/72080155?include=energy
Atp11c	1.129	http://api.brain-map.org/grid_data/download/74047410?include=energy
Pi15	1.129	http://api.brain-map.org/grid_data/download/69783130?include=energy
Vmn2r10	1.129	http://api.brain-map.org/grid_data/download/70194880?include=energy
Apoc4	1.129	http://api.brain-map.org/grid_data/download/68632142?include=energy
Plcb4	1.129	http://api.brain-map.org/grid_data/download/72008304?include=energy
Axin2	1.129	http://api.brain-map.org/grid_data/download/74819487?include=energy
Ttc39a	1.129	http://api.brain-map.org/grid_data/download/75828175?include=energy
Gpc5	1.129	http://api.brain-map.org/grid_data/download/74047712?include=energy
LOC434271	1.129	http://api.brain-map.org/grid_data/download/70787654?include=energy
Pbx3	1.129	http://api.brain-map.org/grid_data/download/76085744?include=energy
LOC433583	1.128	http://api.brain-map.org/grid_data/download/70785804?include=energy
2410015M20Rik	1.128	http://api.brain-map.org/grid_data/download/68844217?include=energy
Fryl	1.128	http://api.brain-map.org/grid_data/download/70431430?include=energy
Lrrc10b	1.128	http://api.brain-map.org/grid_data/download/70595823?include=energy
Sept10	1.128	http://api.brain-map.org/grid_data/download/75694304?include=energy
6720463M24Rik	1.127	http://api.brain-map.org/grid_data/download/68797816?include=energy
Aldh3b2	1.127	http://api.brain-map.org/grid_data/download/70723577?include=energy
F830010L01Rik*	1.126	http://api.brain-map.org/grid_data/download/73731424?include=energy
Gm13624	1.126	http://api.brain-map.org/grid_data/download/74736237?include=energy
Tas2r106	1.126	http://api.brain-map.org/grid_data/download/73424344?include=energy
Ryr3	1.126	http://api.brain-map.org/grid_data/download/77904836?include=energy
LOC434199	1.126	http://api.brain-map.org/grid_data/download/70787573?include=energy
A030009H04Rik	1.126	http://api.brain-map.org/grid_data/download/74750014?include=energy
Bend6	1.126	http://api.brain-map.org/grid_data/download/77280313?include=energy
Cdhr4	1.126	http://api.brain-map.org/grid_data/download/71809331?include=energy
Ngfr	1.126	http://api.brain-map.org/grid_data/download/705?include=energy
4930519H02Rik	1.126	http://api.brain-map.org/grid_data/download/71891820?include=energy
Slc15a4	1.125	http://api.brain-map.org/grid_data/download/78534506?include=energy
Cbr3	1.125	http://api.brain-map.org/grid_data/download/2077?include=energy
Slc37a2	1.125	http://api.brain-map.org/grid_data/download/72001996?include=energy
Gata3	1.125	http://api.brain-map.org/grid_data/download/73931427?include=energy
Tm4sf1	1.125	http://api.brain-map.org/grid_data/download/74882950?include=energy
C130018E23Rik*	1.125	http://api.brain-map.org/grid_data/download/75144623?include=energy
Sohlh2	1.125	http://api.brain-map.org/grid_data/download/71892089?include=energy
Kcnab1	1.125	http://api.brain-map.org/grid_data/download/70301087?include=energy
Qprt	1.125	http://api.brain-map.org/grid_data/download/71617020?include=energy
Epn3	1.124	http://api.brain-map.org/grid_data/download/74357584?include=energy
4732415M23Rik	1.124	http://api.brain-map.org/grid_data/download/69407648?include=energy
Ctrc	1.124	http://api.brain-map.org/grid_data/download/71991666?include=energy
Abcc12	1.124	http://api.brain-map.org/grid_data/download/76003341?include=energy
Ninj2	1.124	http://api.brain-map.org/grid_data/download/69782270?include=energy
Gpr12	1.124	http://api.brain-map.org/grid_data/download/73817425?include=energy
Kcnc2	1.123	http://api.brain-map.org/grid_data/download/73512364?include=energy
Gm426	1.123	http://api.brain-map.org/grid_data/download/73712792?include=energy
Chrm2	1.123	http://api.brain-map.org/grid_data/download/70560343?include=energy
Anln	1.123	http://api.brain-map.org/grid_data/download/33?include=energy
Gpr179	1.123	http://api.brain-map.org/grid_data/download/73712768?include=energy
Olfr1388	1.123	http://api.brain-map.org/grid_data/download/73653278?include=energy
Cit	1.123	http://api.brain-map.org/grid_data/download/75079801?include=energy
Xlr	1.123	http://api.brain-map.org/grid_data/download/633305?include=energy
2010106E10Rik	1.123	http://api.brain-map.org/grid_data/download/73818842?include=energy
Dlec1	1.123	http://api.brain-map.org/grid_data/download/71809424?include=energy
Tacr3	1.123	http://api.brain-map.org/grid_data/download/1297?include=energy
Grk1	1.122	http://api.brain-map.org/grid_data/download/71587770?include=energy
Cyp17a1	1.122	http://api.brain-map.org/grid_data/download/71380995?include=energy
Olfr32	1.122	http://api.brain-map.org/grid_data/download/73653392?include=energy
Trim13	1.122	http://api.brain-map.org/grid_data/download/70192045?include=energy
Gpc2	1.122	http://api.brain-map.org/grid_data/download/72472808?include=energy
F930045I16Rik*	1.122	http://api.brain-map.org/grid_data/download/73808141?include=energy
LOC238771	1.122	http://api.brain-map.org/grid_data/download/70566235?include=energy
Abat	1.122	http://api.brain-map.org/grid_data/download/72079931?include=energy
Arhgap24	1.122	http://api.brain-map.org/grid_data/download/68151345?include=energy
Mki67	1.122	http://api.brain-map.org/grid_data/download/1381?include=energy
Abca2	1.122	http://api.brain-map.org/grid_data/download/69514530?include=energy
Gm4166	1.122	http://api.brain-map.org/grid_data/download/70565464?include=energy
Olfr629	1.122	http://api.brain-map.org/grid_data/download/73653464?include=energy
Defb19	1.122	http://api.brain-map.org/grid_data/download/69113829?include=energy
F530110H13Rik*	1.122	http://api.brain-map.org/grid_data/download/73767374?include=energy
Strc	1.122	http://api.brain-map.org/grid_data/download/73424317?include=energy
Tpst1	1.122	http://api.brain-map.org/grid_data/download/69080019?include=energy
A430086B14Rik*	1.122	http://api.brain-map.org/grid_data/download/73949671?include=energy
LOC433707	1.122	http://api.brain-map.org/grid_data/download/70786713?include=energy
Wrb	1.121	http://api.brain-map.org/grid_data/download/1117?include=energy
Fam82a1	1.121	http://api.brain-map.org/grid_data/download/69236652?include=energy
Heatr8	1.121	http://api.brain-map.org/grid_data/download/70299899?include=energy
Pla2g16	1.121	http://api.brain-map.org/grid_data/download/112650769?include=energy
2210020M01Rik	1.121	http://api.brain-map.org/grid_data/download/77790736?include=energy
Cenpw	1.121	http://api.brain-map.org/grid_data/download/69202872?include=energy
Aloxe3	1.121	http://api.brain-map.org/grid_data/download/74579455?include=energy
Zdhhc12	1.12	http://api.brain-map.org/grid_data/download/532759?include=energy
Ppm1n	1.12	http://api.brain-map.org/grid_data/download/71574600?include=energy
4930545L08Rik	1.12	http://api.brain-map.org/grid_data/download/70218318?include=energy
Ugt3a1	1.12	http://api.brain-map.org/grid_data/download/70593126?include=energy
Gucy2g	1.12	http://api.brain-map.org/grid_data/download/74277641?include=energy
Nrn1l	1.12	http://api.brain-map.org/grid_data/download/71764407?include=energy
Slc25a41	1.12	http://api.brain-map.org/grid_data/download/72182965?include=energy
Ugt1a5	1.12	http://api.brain-map.org/grid_data/download/70302902?include=energy
Tmem53	1.12	http://api.brain-map.org/grid_data/download/68341824?include=energy
TC1410973	1.12	http://api.brain-map.org/grid_data/download/73512191?include=energy
Agr3	1.12	http://api.brain-map.org/grid_data/download/74822939?include=energy
Rit2	1.12	http://api.brain-map.org/grid_data/download/70429399?include=energy
Mep1b	1.119	http://api.brain-map.org/grid_data/download/69752179?include=energy
Gfra4	1.119	http://api.brain-map.org/grid_data/download/74359748?include=energy
Tas2r144	1.119	http://api.brain-map.org/grid_data/download/71670733?include=energy
Coq7	1.119	http://api.brain-map.org/grid_data/download/68546248?include=energy
1600022D10Rik	1.119	http://api.brain-map.org/grid_data/download/71570753?include=energy
Ttc16	1.119	http://api.brain-map.org/grid_data/download/70192117?include=energy
Ncr1	1.118	http://api.brain-map.org/grid_data/download/71234534?include=energy
Rptn	1.118	http://api.brain-map.org/grid_data/download/74635382?include=energy
4930553J12Rik	1.118	http://api.brain-map.org/grid_data/download/74579416?include=energy
Rtkn	1.118	http://api.brain-map.org/grid_data/download/80471950?include=energy
LOC433434	1.118	http://api.brain-map.org/grid_data/download/70723241?include=energy
Gm15688	1.118	http://api.brain-map.org/grid_data/download/70786634?include=energy
Dopey1	1.118	http://api.brain-map.org/grid_data/download/73949532?include=energy
Cftr	1.118	http://api.brain-map.org/grid_data/download/69548870?include=energy
Olfr1019	1.118	http://api.brain-map.org/grid_data/download/74720549?include=energy
4930564C03Rik	1.118	http://api.brain-map.org/grid_data/download/70218402?include=energy
Plac8l1	1.117	http://api.brain-map.org/grid_data/download/71919285?include=energy
Paip2b	1.117	http://api.brain-map.org/grid_data/download/71612166?include=energy
Slc4a9	1.117	http://api.brain-map.org/grid_data/download/71808624?include=energy
Slc22a26	1.117	http://api.brain-map.org/grid_data/download/69015691?include=energy
Sox21	1.117	http://api.brain-map.org/grid_data/download/73496995?include=energy
Cntn4	1.117	http://api.brain-map.org/grid_data/download/73992718?include=energy
Olfr700	1.117	http://api.brain-map.org/grid_data/download/73653488?include=energy
LOC433411	1.117	http://api.brain-map.org/grid_data/download/74578359?include=energy
Zfp800	1.117	http://api.brain-map.org/grid_data/download/71495167?include=energy
Gtpbp3	1.117	http://api.brain-map.org/grid_data/download/71529881?include=energy
AI836003	1.117	http://api.brain-map.org/grid_data/download/71250313?include=energy
Abca9	1.117	http://api.brain-map.org/grid_data/download/78534532?include=energy
Elovl1	1.117	http://api.brain-map.org/grid_data/download/74882527?include=energy
9030404E10Rik	1.117	http://api.brain-map.org/grid_data/download/70305138?include=energy
2410076I21Rik	1.116	http://api.brain-map.org/grid_data/download/74635556?include=energy
Alas1	1.116	http://api.brain-map.org/grid_data/download/199198?include=energy
1700024P16Rik	1.116	http://api.brain-map.org/grid_data/download/74581199?include=energy
Mab21l1	1.116	http://api.brain-map.org/grid_data/download/76098357?include=energy
Dennd1b	1.116	http://api.brain-map.org/grid_data/download/69529113?include=energy
Mark4	1.116	http://api.brain-map.org/grid_data/download/71764553?include=energy
2410066E13Rik	1.116	http://api.brain-map.org/grid_data/download/71587888?include=energy
Daam2	1.116	http://api.brain-map.org/grid_data/download/74511880?include=energy
4930441O14Rik	1.116	http://api.brain-map.org/grid_data/download/69407983?include=energy
Secisbp2l	1.116	http://api.brain-map.org/grid_data/download/70203615?include=energy
Glra2	1.115	http://api.brain-map.org/grid_data/download/73636121?include=energy
Endod1	1.115	http://api.brain-map.org/grid_data/download/71015195?include=energy
Olfr1288	1.115	http://api.brain-map.org/grid_data/download/74720687?include=energy
Kif11	1.115	http://api.brain-map.org/grid_data/download/74509588?include=energy
Fam176b	1.115	http://api.brain-map.org/grid_data/download/71724473?include=energy
Antxr1	1.115	http://api.brain-map.org/grid_data/download/70230945?include=energy
Gamt	1.115	http://api.brain-map.org/grid_data/download/69262341?include=energy
Prrg2	1.115	http://api.brain-map.org/grid_data/download/276063?include=energy
Pcsk1n	1.115	http://api.brain-map.org/grid_data/download/777?include=energy
6430710M23Rik	1.115	http://api.brain-map.org/grid_data/download/100145390?include=energy
B130016D09Rik	1.115	http://api.brain-map.org/grid_data/download/70784558?include=energy
Prame	1.114	http://api.brain-map.org/grid_data/download/71891880?include=energy
Stard10	1.114	http://api.brain-map.org/grid_data/download/72002106?include=energy
Kremen2	1.114	http://api.brain-map.org/grid_data/download/69735915?include=energy
E130003G02Rik	1.114	http://api.brain-map.org/grid_data/download/74514001?include=energy
Thsd7a	1.114	http://api.brain-map.org/grid_data/download/71924155?include=energy
Arpm1	1.114	http://api.brain-map.org/grid_data/download/74822578?include=energy
Nek3	1.114	http://api.brain-map.org/grid_data/download/71763498?include=energy
Sema3b	1.114	http://api.brain-map.org/grid_data/download/79544848?include=energy
Snx16	1.114	http://api.brain-map.org/grid_data/download/671228?include=energy
Tmem22	1.114	http://api.brain-map.org/grid_data/download/74511956?include=energy
Mm.94153	1.114	http://api.brain-map.org/grid_data/download/71210484?include=energy
Ubr4	1.113	http://api.brain-map.org/grid_data/download/71232607?include=energy
Sgk1	1.113	http://api.brain-map.org/grid_data/download/79903782?include=energy
Pgpep1l	1.113	http://api.brain-map.org/grid_data/download/71919020?include=energy
Ttc18	1.113	http://api.brain-map.org/grid_data/download/70302854?include=energy
Plk1s1	1.113	http://api.brain-map.org/grid_data/download/70295882?include=energy
4931419H13Rik	1.113	http://api.brain-map.org/grid_data/download/71892035?include=energy
Accn4	1.113	http://api.brain-map.org/grid_data/download/71924089?include=energy
F830218M08Rik*	1.112	http://api.brain-map.org/grid_data/download/73731460?include=energy
Nsmaf	1.112	http://api.brain-map.org/grid_data/download/71234677?include=energy
Mm.35159	1.112	http://api.brain-map.org/grid_data/download/71161135?include=energy
Bves	1.112	http://api.brain-map.org/grid_data/download/74511935?include=energy
Ret	1.112	http://api.brain-map.org/grid_data/download/81657693?include=energy
Gm6420	1.112	http://api.brain-map.org/grid_data/download/73716644?include=energy
Arhgef15	1.112	http://api.brain-map.org/grid_data/download/71281517?include=energy
Parvb	1.111	http://api.brain-map.org/grid_data/download/77332713?include=energy
Cables2	1.111	http://api.brain-map.org/grid_data/download/72339555?include=energy
Crisp4	1.111	http://api.brain-map.org/grid_data/download/71892418?include=energy
H2-Ea-ps	1.111	http://api.brain-map.org/grid_data/download/71764812?include=energy
Ube2e1	1.111	http://api.brain-map.org/grid_data/download/71763628?include=energy
Nfe2l3	1.111	http://api.brain-map.org/grid_data/download/68910962?include=energy
Ighg3	1.111	http://api.brain-map.org/grid_data/download/71569356?include=energy
Speer7-ps1	1.111	http://api.brain-map.org/grid_data/download/75197785?include=energy
A830019P07Rik	1.11	http://api.brain-map.org/grid_data/download/69449032?include=energy
Hdac11	1.11	http://api.brain-map.org/grid_data/download/69080509?include=energy
Fnbp1	1.11	http://api.brain-map.org/grid_data/download/73906077?include=energy
Apbb2	1.11	http://api.brain-map.org/grid_data/download/72128788?include=energy
mCG145435	1.11	http://api.brain-map.org/grid_data/download/71212174?include=energy
Dcaf5	1.11	http://api.brain-map.org/grid_data/download/71161519?include=energy
Coro2a	1.11	http://api.brain-map.org/grid_data/download/71496324?include=energy
A330008L17Rik	1.11	http://api.brain-map.org/grid_data/download/71494660?include=energy
Phf11	1.11	http://api.brain-map.org/grid_data/download/71892137?include=energy
Ppp1r32	1.11	http://api.brain-map.org/grid_data/download/69442404?include=energy
LOC277860	1.11	http://api.brain-map.org/grid_data/download/74581425?include=energy
Agxt2l2	1.11	http://api.brain-map.org/grid_data/download/70543028?include=energy
Gfra1	1.109	http://api.brain-map.org/grid_data/download/71064217?include=energy
Cdkl3	1.109	http://api.brain-map.org/grid_data/download/71161006?include=energy
Tm7sf3	1.109	http://api.brain-map.org/grid_data/download/1543?include=energy
Jub	1.109	http://api.brain-map.org/grid_data/download/323182?include=energy
BC030476	1.109	http://api.brain-map.org/grid_data/download/68162208?include=energy
1500035N22Rik	1.109	http://api.brain-map.org/grid_data/download/74357785?include=energy
Prl7a2	1.109	http://api.brain-map.org/grid_data/download/74581235?include=energy
Unc5b	1.108	http://api.brain-map.org/grid_data/download/74274687?include=energy
Apoh	1.108	http://api.brain-map.org/grid_data/download/671239?include=energy
4930432M17Rik	1.108	http://api.brain-map.org/grid_data/download/73906539?include=energy
Mia2	1.108	http://api.brain-map.org/grid_data/download/70300336?include=energy
Sik3	1.108	http://api.brain-map.org/grid_data/download/74881867?include=energy
A730069N07Rik	1.108	http://api.brain-map.org/grid_data/download/69448996?include=energy
A130014H13Rik	1.108	http://api.brain-map.org/grid_data/download/75041574?include=energy
Edn1	1.108	http://api.brain-map.org/grid_data/download/71763759?include=energy
4921513H07Rik	1.108	http://api.brain-map.org/grid_data/download/74579944?include=energy
Spint3	1.108	http://api.brain-map.org/grid_data/download/73927784?include=energy
Pqbp1	1.108	http://api.brain-map.org/grid_data/download/1520?include=energy
D230025D16Rik	1.108	http://api.brain-map.org/grid_data/download/68269300?include=energy
Cxcl1	1.108	http://api.brain-map.org/grid_data/download/75233725?include=energy
9330132A10Rik	1.108	http://api.brain-map.org/grid_data/download/71493867?include=energy
Zfp959	1.107	http://api.brain-map.org/grid_data/download/71724828?include=energy
A430084P05Rik	1.107	http://api.brain-map.org/grid_data/download/70595835?include=energy
LOC435576	1.107	http://api.brain-map.org/grid_data/download/70725658?include=energy
Zfp458	1.107	http://api.brain-map.org/grid_data/download/76081042?include=energy
Cyp4a10	1.107	http://api.brain-map.org/grid_data/download/71627752?include=energy
Fan1	1.107	http://api.brain-map.org/grid_data/download/72182971?include=energy
Edil3	1.107	http://api.brain-map.org/grid_data/download/76003522?include=energy
Smtn	1.107	http://api.brain-map.org/grid_data/download/74819233?include=energy
Nacc2	1.107	http://api.brain-map.org/grid_data/download/70743871?include=energy
Sspn	1.106	http://api.brain-map.org/grid_data/download/671249?include=energy
Anxa2	1.106	http://api.brain-map.org/grid_data/download/70928384?include=energy
Tdrd7	1.106	http://api.brain-map.org/grid_data/download/77800951?include=energy
Kif13a	1.106	http://api.brain-map.org/grid_data/download/70295410?include=energy
LOC434427	1.106	http://api.brain-map.org/grid_data/download/71022322?include=energy
Ube2j1	1.106	http://api.brain-map.org/grid_data/download/68666720?include=energy
Clic4	1.106	http://api.brain-map.org/grid_data/download/73769125?include=energy
Dtnb	1.106	http://api.brain-map.org/grid_data/download/72716?include=energy
Obsl1	1.106	http://api.brain-map.org/grid_data/download/71495849?include=energy
Fstl1	1.105	http://api.brain-map.org/grid_data/download/71016596?include=energy
D830047O19Rik*	1.105	http://api.brain-map.org/grid_data/download/74076066?include=energy
4930414F18Rik	1.105	http://api.brain-map.org/grid_data/download/71891653?include=energy
Gm1582	1.105	http://api.brain-map.org/grid_data/download/70298417?include=energy
Mbp	1.105	http://api.brain-map.org/grid_data/download/112202838?include=energy
Cubn	1.105	http://api.brain-map.org/grid_data/download/75041430?include=energy
Spock2	1.105	http://api.brain-map.org/grid_data/download/70812774?include=energy
Pramel4	1.105	http://api.brain-map.org/grid_data/download/70300899?include=energy
Nol12	1.105	http://api.brain-map.org/grid_data/download/71616350?include=energy
Prps1l3	1.105	http://api.brain-map.org/grid_data/download/70795811?include=energy
Olfr1356	1.105	http://api.brain-map.org/grid_data/download/74735056?include=energy
Olfr609	1.104	http://api.brain-map.org/grid_data/download/73653458?include=energy
Shc3	1.104	http://api.brain-map.org/grid_data/download/69873732?include=energy
9430008C03Rik	1.104	http://api.brain-map.org/grid_data/download/69446887?include=energy
Kcnq1ot1	1.104	http://api.brain-map.org/grid_data/download/71152252?include=energy
Cdr2	1.104	http://api.brain-map.org/grid_data/download/73992716?include=energy
Fam110b	1.104	http://api.brain-map.org/grid_data/download/70523673?include=energy
Lyrm4	1.104	http://api.brain-map.org/grid_data/download/69169947?include=energy
Arrdc5	1.103	http://api.brain-map.org/grid_data/download/74581343?include=energy
Olfr30	1.103	http://api.brain-map.org/grid_data/download/74720952?include=energy
Rpl24	1.103	http://api.brain-map.org/grid_data/download/69262115?include=energy
Zfp956	1.103	http://api.brain-map.org/grid_data/download/70230873?include=energy
Ppp4r4	1.103	http://api.brain-map.org/grid_data/download/71249744?include=energy
Cthrc1	1.103	http://api.brain-map.org/grid_data/download/72472772?include=energy
Dkk2	1.103	http://api.brain-map.org/grid_data/download/77464854?include=energy
Otor	1.103	http://api.brain-map.org/grid_data/download/71668988?include=energy
Nid2	1.103	http://api.brain-map.org/grid_data/download/69837532?include=energy
Micalcl	1.103	http://api.brain-map.org/grid_data/download/68410566?include=energy
2700081L22Rik	1.103	http://api.brain-map.org/grid_data/download/74635701?include=energy

Txnip	1.103	http://api.brain-map.org/grid_data/download/1084?include=energy
Clec4e	1.103	http://api.brain-map.org/grid_data/download/67979047?include=energy
LOC433878	1.102	http://api.brain-map.org/grid_data/download/70787044?include=energy
Fbxl13	1.102	http://api.brain-map.org/grid_data/download/71764830?include=energy
Gm7120	1.102	http://api.brain-map.org/grid_data/download/70918964?include=energy
D830046C22Rik	1.102	http://api.brain-map.org/grid_data/download/71021167?include=energy
Cib2	1.102	http://api.brain-map.org/grid_data/download/71836803?include=energy
Mn1	1.102	http://api.brain-map.org/grid_data/download/70787119?include=energy
Ttc23l	1.102	http://api.brain-map.org/grid_data/download/71891605?include=energy
Fank1	1.101	http://api.brain-map.org/grid_data/download/68745239?include=energy
Anxa1	1.101	http://api.brain-map.org/grid_data/download/70813897?include=energy
Tmem41a	1.101	http://api.brain-map.org/grid_data/download/70743869?include=energy
A530086A05Rik*	1.101	http://api.brain-map.org/grid_data/download/73949734?include=energy
Tnni1	1.101	http://api.brain-map.org/grid_data/download/75774109?include=energy
Mm.372079	1.1	http://api.brain-map.org/grid_data/download/71210043?include=energy
D830028D21Rik*	1.1	http://api.brain-map.org/grid_data/download/73834457?include=energy
Fbln7	1.1	http://api.brain-map.org/grid_data/download/73929875?include=energy
Col6a4	1.1	http://api.brain-map.org/grid_data/download/72081722?include=energy
mCG147019	1.1	http://api.brain-map.org/grid_data/download/71212500?include=energy
Atp1b3	1.1	http://api.brain-map.org/grid_data/download/69526952?include=energy
L3mbtl4	1.1	http://api.brain-map.org/grid_data/download/72472779?include=energy
Wrn	1.1	http://api.brain-map.org/grid_data/download/70195024?include=energy
G730044K14Rik*	1.1	http://api.brain-map.org/grid_data/download/73716770?include=energy
Kif9	1.1	http://api.brain-map.org/grid_data/download/69735819?include=energy
Cd63	1.1	http://api.brain-map.org/grid_data/download/73992715?include=energy
Gm11346	1.1	http://api.brain-map.org/grid_data/download/74580823?include=energy
9630005C17Rik	1.1	http://api.brain-map.org/grid_data/download/71161036?include=energy
LOC435226	1.099	http://api.brain-map.org/grid_data/download/70725634?include=energy
Shc1	1.099	http://api.brain-map.org/grid_data/download/74431597?include=energy
Adamts4	1.099	http://api.brain-map.org/grid_data/download/71836845?include=energy
Cryba1	1.099	http://api.brain-map.org/grid_data/download/69608076?include=energy
Fmnl3	1.099	http://api.brain-map.org/grid_data/download/69236640?include=energy
D630036G22Rik	1.099	http://api.brain-map.org/grid_data/download/73834445?include=energy
Spp1	1.099	http://api.brain-map.org/grid_data/download/70436740?include=energy
Pbld1	1.099	http://api.brain-map.org/grid_data/download/71627844?include=energy
Gm5067	1.099	http://api.brain-map.org/grid_data/download/70595805?include=energy
Lif	1.099	http://api.brain-map.org/grid_data/download/69736124?include=energy
Dbi	1.099	http://api.brain-map.org/grid_data/download/71836880?include=energy
Ifna11	1.099	http://api.brain-map.org/grid_data/download/72283617?include=energy
F5	1.099	http://api.brain-map.org/grid_data/download/73992763?include=energy
Bok	1.099	http://api.brain-map.org/grid_data/download/71064032?include=energy
Vmn1r123	1.099	http://api.brain-map.org/grid_data/download/73617642?include=energy
Gip	1.098	http://api.brain-map.org/grid_data/download/71991684?include=energy
Odf3b	1.098	http://api.brain-map.org/grid_data/download/73606588?include=energy
Hepacam	1.098	http://api.brain-map.org/grid_data/download/35?include=energy
Tsr2	1.098	http://api.brain-map.org/grid_data/download/69113632?include=energy
Gpr1	1.098	http://api.brain-map.org/grid_data/download/68744743?include=energy
Wdr65	1.098	http://api.brain-map.org/grid_data/download/70200866?include=energy
Mcm2	1.098	http://api.brain-map.org/grid_data/download/69752125?include=energy
Pkm	1.098	http://api.brain-map.org/grid_data/download/68269268?include=energy
4921524J17Rik	1.097	http://api.brain-map.org/grid_data/download/68667168?include=energy
Cd9	1.097	http://api.brain-map.org/grid_data/download/73520971?include=energy
Gipr	1.097	http://api.brain-map.org/grid_data/download/71307352?include=energy
Klhdc8a	1.097	http://api.brain-map.org/grid_data/download/71610854?include=energy
Adarb2	1.097	http://api.brain-map.org/grid_data/download/73925721?include=energy
Rgs8	1.097	http://api.brain-map.org/grid_data/download/71020445?include=energy
Plcl1	1.097	http://api.brain-map.org/grid_data/download/75081396?include=energy
Rcan2	1.097	http://api.brain-map.org/grid_data/download/71064278?include=energy
Sgk3	1.097	http://api.brain-map.org/grid_data/download/75650862?include=energy
Rbmy1a1	1.096	http://api.brain-map.org/grid_data/download/74749179?include=energy
Hemk1	1.096	http://api.brain-map.org/grid_data/download/564757?include=energy
Cox7b	1.096	http://api.brain-map.org/grid_data/download/67870085?include=energy
Osbpl9	1.096	http://api.brain-map.org/grid_data/download/77371759?include=energy
Nacad	1.096	http://api.brain-map.org/grid_data/download/70565867?include=energy
LOC329248	1.096	http://api.brain-map.org/grid_data/download/70595865?include=energy
Gm12692	1.096	http://api.brain-map.org/grid_data/download/74736147?include=energy
D830030K20Rik	1.096	http://api.brain-map.org/grid_data/download/73930807?include=energy
Ppp3r1	1.096	http://api.brain-map.org/grid_data/download/32386?include=energy
Moxd2	1.096	http://api.brain-map.org/grid_data/download/70565470?include=energy
Olfr958	1.096	http://api.brain-map.org/grid_data/download/74277397?include=energy
Gm6878	1.096	http://api.brain-map.org/grid_data/download/73927643?include=energy
Ereg	1.096	http://api.brain-map.org/grid_data/download/177149?include=energy
Atp5f1	1.096	http://api.brain-map.org/grid_data/download/69113524?include=energy
Immt	1.096	http://api.brain-map.org/grid_data/download/69735449?include=energy
Rora	1.096	http://api.brain-map.org/grid_data/download/2537?include=energy
4930463O16Rik	1.096	http://api.brain-map.org/grid_data/download/71891701?include=energy
Rap1gap	1.096	http://api.brain-map.org/grid_data/download/68798974?include=energy
Crygc	1.096	http://api.brain-map.org/grid_data/download/69608088?include=energy
Arfgap2	1.096	http://api.brain-map.org/grid_data/download/512773?include=energy
Map4k2	1.095	http://api.brain-map.org/grid_data/download/69752035?include=energy
LOC433379	1.095	http://api.brain-map.org/grid_data/download/74562504?include=energy
Mm.331983	1.095	http://api.brain-map.org/grid_data/download/73635445?include=energy
E030002O03Rik	1.095	http://api.brain-map.org/grid_data/download/68798163?include=energy
Sgk2	1.095	http://api.brain-map.org/grid_data/download/69170312?include=energy
Rps12	1.095	http://api.brain-map.org/grid_data/download/70928395?include=energy
Mmp8	1.095	http://api.brain-map.org/grid_data/download/68920827?include=energy
Flt3	1.095	http://api.brain-map.org/grid_data/download/71232693?include=energy
Gm11292	1.095	http://api.brain-map.org/grid_data/download/70565873?include=energy
Krt18	1.095	http://api.brain-map.org/grid_data/download/69735939?include=energy
Drd5	1.095	http://api.brain-map.org/grid_data/download/81790710?include=energy
Rab3c	1.095	http://api.brain-map.org/grid_data/download/73521805?include=energy
Cd3eap	1.095	http://api.brain-map.org/grid_data/download/248561?include=energy
Npas1	1.095	http://api.brain-map.org/grid_data/download/74821618?include=energy
Pmvk	1.095	http://api.brain-map.org/grid_data/download/68522491?include=energy
Nanos3	1.095	http://api.brain-map.org/grid_data/download/70300517?include=energy
Olfr1270	1.094	http://api.brain-map.org/grid_data/download/73635562?include=energy
Lsm5	1.094	http://api.brain-map.org/grid_data/download/69117164?include=energy
Eif2c4	1.094	http://api.brain-map.org/grid_data/download/75931524?include=energy
LOC436099	1.094	http://api.brain-map.org/grid_data/download/73512350?include=energy
Trim30b	1.094	http://api.brain-map.org/grid_data/download/69448692?include=energy
C030006K11Rik	1.094	http://api.brain-map.org/grid_data/download/71612222?include=energy
BC016423	1.094	http://api.brain-map.org/grid_data/download/73906239?include=energy
Atp7a	1.094	http://api.brain-map.org/grid_data/download/69526982?include=energy
Fbxo3	1.094	http://api.brain-map.org/grid_data/download/69103916?include=energy
Pabpc4l	1.094	http://api.brain-map.org/grid_data/download/70566316?include=energy
Mm.343429	1.094	http://api.brain-map.org/grid_data/download/71161060?include=energy
Cyp2s1	1.094	http://api.brain-map.org/grid_data/download/74821670?include=energy
Fcgr2b	1.094	http://api.brain-map.org/grid_data/download/119698?include=energy
1600020E01Rik	1.093	http://api.brain-map.org/grid_data/download/74581289?include=energy
Apeh	1.093	http://api.brain-map.org/grid_data/download/68498854?include=energy
Hnrnpd	1.093	http://api.brain-map.org/grid_data/download/68301939?include=energy
Gm410	1.093	http://api.brain-map.org/grid_data/download/70298136?include=energy
Wdr64	1.093	http://api.brain-map.org/grid_data/download/71891802?include=energy
Utp14b	1.093	http://api.brain-map.org/grid_data/download/71924249?include=energy
Rad9b	1.093	http://api.brain-map.org/grid_data/download/68795450?include=energy
Dnajc12	1.093	http://api.brain-map.org/grid_data/download/73718054?include=energy
Zfand1	1.093	http://api.brain-map.org/grid_data/download/70474905?include=energy
LOC432858	1.093	http://api.brain-map.org/grid_data/download/71764824?include=energy
Cyth4	1.093	http://api.brain-map.org/grid_data/download/69549626?include=energy
Cdk18	1.093	http://api.brain-map.org/grid_data/download/69782879?include=energy
Slco2a1	1.093	http://api.brain-map.org/grid_data/download/69169917?include=energy
Gga1	1.092	http://api.brain-map.org/grid_data/download/532690?include=energy
Olfr686	1.092	http://api.brain-map.org/grid_data/download/74734854?include=energy
Adh1	1.092	http://api.brain-map.org/grid_data/download/68341519?include=energy
Gabrr1	1.092	http://api.brain-map.org/grid_data/download/75551470?include=energy
Ngly1	1.092	http://api.brain-map.org/grid_data/download/68151451?include=energy
Tac1	1.092	http://api.brain-map.org/grid_data/download/1038?include=energy
Kif5b	1.092	http://api.brain-map.org/grid_data/download/2353?include=energy
Mak	1.092	http://api.brain-map.org/grid_data/download/75831733?include=energy
Sult4a1	1.092	http://api.brain-map.org/grid_data/download/69113650?include=energy
Asz1	1.092	http://api.brain-map.org/grid_data/download/70430830?include=energy
Evl	1.092	http://api.brain-map.org/grid_data/download/69838381?include=energy
Gnb5	1.092	http://api.brain-map.org/grid_data/download/1456?include=energy
Trnp1	1.092	http://api.brain-map.org/grid_data/download/72080175?include=energy
I0C0021L08Rik*	1.091	http://api.brain-map.org/grid_data/download/73716722?include=energy
Zbbx	1.091	http://api.brain-map.org/grid_data/download/69529155?include=energy
B3galtl	1.091	http://api.brain-map.org/grid_data/download/69672904?include=energy
Tulp4	1.091	http://api.brain-map.org/grid_data/download/2699?include=energy
Olfr656	1.091	http://api.brain-map.org/grid_data/download/74734806?include=energy
Pigp	1.091	http://api.brain-map.org/grid_data/download/2165?include=energy
Rrp8	1.091	http://api.brain-map.org/grid_data/download/532671?include=energy
Cd69	1.091	http://api.brain-map.org/grid_data/download/74821820?include=energy
A330041K01	1.091	http://api.brain-map.org/grid_data/download/71161147?include=energy
Npc2	1.091	http://api.brain-map.org/grid_data/download/75774115?include=energy
Rab31	1.091	http://api.brain-map.org/grid_data/download/79544800?include=energy
Rnd1	1.091	http://api.brain-map.org/grid_data/download/69113620?include=energy
Gm1323	1.09	http://api.brain-map.org/grid_data/download/70608358?include=energy
Qk	1.09	http://api.brain-map.org/grid_data/download/68797858?include=energy
Spsb2	1.09	http://api.brain-map.org/grid_data/download/68269058?include=energy
Hhip	1.09	http://api.brain-map.org/grid_data/download/71358556?include=energy
7420422B15Rik*	1.09	http://api.brain-map.org/grid_data/download/73906119?include=energy
Kazn	1.09	http://api.brain-map.org/grid_data/download/72007937?include=energy
E030019B06Rik	1.09	http://api.brain-map.org/grid_data/download/74510170?include=energy
Ephb2	1.09	http://api.brain-map.org/grid_data/download/69672096?include=energy
Rhog	1.09	http://api.brain-map.org/grid_data/download/68745114?include=energy
Krtap7-1	1.09	http://api.brain-map.org/grid_data/download/74359640?include=energy
Pcdhb4	1.09	http://api.brain-map.org/grid_data/download/69837413?include=energy
1700108M19Rik	1.09	http://api.brain-map.org/grid_data/download/71920225?include=energy
Ppig	1.089	http://api.brain-map.org/grid_data/download/73512516?include=energy
5730420D15Rik	1.089	http://api.brain-map.org/grid_data/download/74580811?include=energy
Ankrd34c	1.089	http://api.brain-map.org/grid_data/download/72472747?include=energy
Eppk1	1.089	http://api.brain-map.org/grid_data/download/69672108?include=energy
Fam98c	1.089	http://api.brain-map.org/grid_data/download/68410614?include=energy
Kl	1.089	http://api.brain-map.org/grid_data/download/72119590?include=energy
Ppp2r1a	1.089	http://api.brain-map.org/grid_data/download/68667946?include=energy
Pdxk	1.089	http://api.brain-map.org/grid_data/download/788?include=energy
Gm5485	1.089	http://api.brain-map.org/grid_data/download/74736342?include=energy
Mm.347488	1.089	http://api.brain-map.org/grid_data/download/71161072?include=energy
Nudt1	1.089	http://api.brain-map.org/grid_data/download/68410488?include=energy
Trabd	1.088	http://api.brain-map.org/grid_data/download/71616334?include=energy
Fkbp9	1.088	http://api.brain-map.org/grid_data/download/68562926?include=energy
Lrrc46	1.088	http://api.brain-map.org/grid_data/download/76135594?include=energy
Mm.125817	1.088	http://api.brain-map.org/grid_data/download/74509586?include=energy
Zic3	1.088	http://api.brain-map.org/grid_data/download/69817133?include=energy
Mm.121392	1.088	http://api.brain-map.org/grid_data/download/71147998?include=energy
Trp63	1.088	http://api.brain-map.org/grid_data/download/69290692?include=energy
Gm1399	1.088	http://api.brain-map.org/grid_data/download/71924184?include=energy
Cphx	1.087	http://api.brain-map.org/grid_data/download/70919800?include=energy
Irg1	1.087	http://api.brain-map.org/grid_data/download/69735526?include=energy
Pptc7	1.087	http://api.brain-map.org/grid_data/download/74881503?include=energy
Tgfbr3	1.087	http://api.brain-map.org/grid_data/download/72004415?include=energy
Ripply2	1.087	http://api.brain-map.org/grid_data/download/72076557?include=energy
Trnau1ap	1.087	http://api.brain-map.org/grid_data/download/71211665?include=energy
Mageb18	1.087	http://api.brain-map.org/grid_data/download/70231194?include=energy
Ube2t	1.087	http://api.brain-map.org/grid_data/download/276069?include=energy
Slc36a2	1.087	http://api.brain-map.org/grid_data/download/1069?include=energy
Neil3	1.087	http://api.brain-map.org/grid_data/download/74658247?include=energy
Olfr1112	1.087	http://api.brain-map.org/grid_data/download/74720609?include=energy
Spats2	1.087	http://api.brain-map.org/grid_data/download/68080693?include=energy
E330010G22Rik*	1.087	http://api.brain-map.org/grid_data/download/73906436?include=energy
Gm6034	1.087	http://api.brain-map.org/grid_data/download/74743327?include=energy
Kitl	1.086	http://api.brain-map.org/grid_data/download/616?include=energy
Myo15	1.086	http://api.brain-map.org/grid_data/download/74059212?include=energy
Fam3b	1.086	http://api.brain-map.org/grid_data/download/74580865?include=energy
Arhgap23	1.086	http://api.brain-map.org/grid_data/download/74431685?include=energy
Gm428	1.086	http://api.brain-map.org/grid_data/download/72471839?include=energy
Slc25a1	1.086	http://api.brain-map.org/grid_data/download/2587?include=energy
Nova1	1.086	http://api.brain-map.org/grid_data/download/73931640?include=energy
Slc39a14	1.086	http://api.brain-map.org/grid_data/download/1846?include=energy
Grem2	1.086	http://api.brain-map.org/grid_data/download/73992910?include=energy
Dnajc1	1.086	http://api.brain-map.org/grid_data/download/71496453?include=energy
Trim50	1.086	http://api.brain-map.org/grid_data/download/74056155?include=energy
Gm5065	1.086	http://api.brain-map.org/grid_data/download/74736099?include=energy
4930568M16Rik*	1.086	http://api.brain-map.org/grid_data/download/73906485?include=energy
Bbs10	1.086	http://api.brain-map.org/grid_data/download/100144865?include=energy
Calcb	1.086	http://api.brain-map.org/grid_data/download/72338699?include=energy
Tshz2	1.086	http://api.brain-map.org/grid_data/download/72129294?include=energy
Ndufs1	1.086	http://api.brain-map.org/grid_data/download/2421?include=energy
Scn2b	1.086	http://api.brain-map.org/grid_data/download/73573157?include=energy
Serpinf1	1.086	http://api.brain-map.org/grid_data/download/2559?include=energy
Scml2	1.086	http://api.brain-map.org/grid_data/download/73992917?include=energy
Smc4	1.086	http://api.brain-map.org/grid_data/download/69816900?include=energy
4930555F03Rik	1.085	http://api.brain-map.org/grid_data/download/71891892?include=energy
Enpp6	1.085	http://api.brain-map.org/grid_data/download/74882555?include=energy
Commd6	1.085	http://api.brain-map.org/grid_data/download/74580978?include=energy
Nr3c1	1.085	http://api.brain-map.org/grid_data/download/727?include=energy
Vamp5	1.085	http://api.brain-map.org/grid_data/download/70194898?include=energy
Cdkl1	1.085	http://api.brain-map.org/grid_data/download/69540701?include=energy
Sox10	1.085	http://api.brain-map.org/grid_data/download/79556652?include=energy
Gm4791	1.085	http://api.brain-map.org/grid_data/download/70565458?include=energy
Ces2f	1.085	http://api.brain-map.org/grid_data/download/70218697?include=energy
Lmnb1	1.085	http://api.brain-map.org/grid_data/download/69736142?include=energy
Lingo3	1.085	http://api.brain-map.org/grid_data/download/100143455?include=energy
LOC433479	1.085	http://api.brain-map.org/grid_data/download/71832812?include=energy
Efs	1.084	http://api.brain-map.org/grid_data/download/71664143?include=energy
Tgds	1.084	http://api.brain-map.org/grid_data/download/77620499?include=energy
Naglu	1.084	http://api.brain-map.org/grid_data/download/68861870?include=energy
Twist2	1.084	http://api.brain-map.org/grid_data/download/74821628?include=energy
Inpp5f	1.084	http://api.brain-map.org/grid_data/download/69133785?include=energy
Flnb	1.084	http://api.brain-map.org/grid_data/download/69672480?include=energy
Aox1	1.084	http://api.brain-map.org/grid_data/download/74425525?include=energy
Cma1	1.084	http://api.brain-map.org/grid_data/download/81790714?include=energy
Cxx1c	1.084	http://api.brain-map.org/grid_data/download/71764023?include=energy
Kcnk2	1.084	http://api.brain-map.org/grid_data/download/75147764?include=energy
Slc23a1	1.083	http://api.brain-map.org/grid_data/download/323828?include=energy
Sh3pxd2b	1.083	http://api.brain-map.org/grid_data/download/71735074?include=energy
Rnase13	1.083	http://api.brain-map.org/grid_data/download/73514905?include=energy
Defb12	1.083	http://api.brain-map.org/grid_data/download/69626837?include=energy
Bcap29	1.083	http://api.brain-map.org/grid_data/download/104826?include=energy
Ush2a	1.083	http://api.brain-map.org/grid_data/download/71210944?include=energy
Cd99l2	1.083	http://api.brain-map.org/grid_data/download/68637898?include=energy
2810032G03Rik	1.083	http://api.brain-map.org/grid_data/download/71493075?include=energy
mCG148246	1.083	http://api.brain-map.org/grid_data/download/71212632?include=energy
LOC433649	1.083	http://api.brain-map.org/grid_data/download/71456126?include=energy
Trappc4	1.083	http://api.brain-map.org/grid_data/download/71662899?include=energy
S100a11	1.082	http://api.brain-map.org/grid_data/download/104600?include=energy
Efcab8	1.082	http://api.brain-map.org/grid_data/download/71809319?include=energy
Sly	1.082	http://api.brain-map.org/grid_data/download/69114180?include=energy
Alas2	1.082	http://api.brain-map.org/grid_data/download/71723851?include=energy
9430028L06Rik	1.082	http://api.brain-map.org/grid_data/download/71717154?include=energy
Lrrc43	1.082	http://api.brain-map.org/grid_data/download/70299953?include=energy
Nefh	1.082	http://api.brain-map.org/grid_data/download/74512048?include=energy
Baiap3	1.082	http://api.brain-map.org/grid_data/download/75081206?include=energy
Npb	1.082	http://api.brain-map.org/grid_data/download/74059213?include=energy
Mocs3	1.082	http://api.brain-map.org/grid_data/download/74581361?include=energy
Slc7a3	1.081	http://api.brain-map.org/grid_data/download/73817929?include=energy
9430062P05Rik	1.081	http://api.brain-map.org/grid_data/download/71212117?include=energy
Shd	1.081	http://api.brain-map.org/grid_data/download/72001882?include=energy
Selenbp2	1.081	http://api.brain-map.org/grid_data/download/69261401?include=energy
Rarres2	1.081	http://api.brain-map.org/grid_data/download/75077281?include=energy
Pold4	1.081	http://api.brain-map.org/grid_data/download/71764195?include=energy
Ccdc144b	1.081	http://api.brain-map.org/grid_data/download/69114803?include=energy
Ssty1	1.081	http://api.brain-map.org/grid_data/download/69885774?include=energy
F730205C23Rik*	1.08	http://api.brain-map.org/grid_data/download/73767199?include=energy
Trpm2	1.08	http://api.brain-map.org/grid_data/download/69288417?include=energy
Fam111a	1.08	http://api.brain-map.org/grid_data/download/69095976?include=energy
Zmynd17	1.08	http://api.brain-map.org/grid_data/download/71833046?include=energy

Cd81	1.08	http://api.brain-map.org/grid_data/download/226?include=energy
Svs4	1.08	http://api.brain-map.org/grid_data/download/69887487?include=energy
Mmel1	1.08	http://api.brain-map.org/grid_data/download/76135804?include=energy
Ltb4r1	1.08	http://api.brain-map.org/grid_data/download/68744188?include=energy
Ddhd1	1.08	http://api.brain-map.org/grid_data/download/69838392?include=energy
Ybey	1.08	http://api.brain-map.org/grid_data/download/77454729?include=energy
Vmn1r26	1.08	http://api.brain-map.org/grid_data/download/70785230?include=energy
Fam75d3	1.08	http://api.brain-map.org/grid_data/download/70201253?include=energy
Mapk3	1.079	http://api.brain-map.org/grid_data/download/32576?include=energy
Olfr19	1.079	http://api.brain-map.org/grid_data/download/73653344?include=energy
5031439G07Rik	1.079	http://api.brain-map.org/grid_data/download/76003377?include=energy
Slc28a1	1.079	http://api.brain-map.org/grid_data/download/71213862?include=energy
Ntsr2	1.079	http://api.brain-map.org/grid_data/download/81600566?include=energy
Serpinb2	1.079	http://api.brain-map.org/grid_data/download/75084144?include=energy
Nrtn	1.079	http://api.brain-map.org/grid_data/download/741?include=energy
Ccdc105	1.079	http://api.brain-map.org/grid_data/download/69442434?include=energy
Sesn2	1.079	http://api.brain-map.org/grid_data/download/386017?include=energy
Nit2	1.079	http://api.brain-map.org/grid_data/download/68341818?include=energy
Ap1s3	1.079	http://api.brain-map.org/grid_data/download/74581163?include=energy
Gm1566	1.079	http://api.brain-map.org/grid_data/download/74578278?include=energy
Stag2	1.079	http://api.brain-map.org/grid_data/download/72002105?include=energy
Atp6v0b	1.079	http://api.brain-map.org/grid_data/download/293682?include=energy
Perp	1.078	http://api.brain-map.org/grid_data/download/68795445?include=energy
Gyltl1b	1.078	http://api.brain-map.org/grid_data/download/71529887?include=energy
Efnb3	1.078	http://api.brain-map.org/grid_data/download/79556601?include=energy
Cep63	1.078	http://api.brain-map.org/grid_data/download/68797786?include=energy
Inpp5a	1.078	http://api.brain-map.org/grid_data/download/73994713?include=energy
Smarcd3	1.078	http://api.brain-map.org/grid_data/download/100143575?include=energy
LOC434442	1.078	http://api.brain-map.org/grid_data/download/70920147?include=energy
Crabp2	1.078	http://api.brain-map.org/grid_data/download/68076913?include=energy
Crebl2	1.078	http://api.brain-map.org/grid_data/download/70305425?include=energy
mCG13005.1	1.078	http://api.brain-map.org/grid_data/download/71211365?include=energy
Slc32a1	1.078	http://api.brain-map.org/grid_data/download/72081554?include=energy
Nfkbib	1.078	http://api.brain-map.org/grid_data/download/69549166?include=energy
Sptlc2	1.078	http://api.brain-map.org/grid_data/download/74658175?include=energy
G270054J13Rik*	1.077	http://api.brain-map.org/grid_data/download/73716686?include=energy
AW549877	1.077	http://api.brain-map.org/grid_data/download/69059819?include=energy
Dnm2	1.077	http://api.brain-map.org/grid_data/download/386015?include=energy
Tagln2	1.077	http://api.brain-map.org/grid_data/download/68269294?include=energy
Gab1	1.077	http://api.brain-map.org/grid_data/download/71735832?include=energy
Glra1	1.077	http://api.brain-map.org/grid_data/download/72108824?include=energy
LOC545328	1.077	http://api.brain-map.org/grid_data/download/77621743?include=energy
A130023E24Rik	1.077	http://api.brain-map.org/grid_data/download/70228346?include=energy
Xpnpep2	1.077	http://api.brain-map.org/grid_data/download/80342131?include=energy
Enah	1.077	http://api.brain-map.org/grid_data/download/70920003?include=energy
4930470P17Rik	1.077	http://api.brain-map.org/grid_data/download/71891862?include=energy
C030016D13Rik	1.077	http://api.brain-map.org/grid_data/download/69530720?include=energy
E330037G11Rik	1.077	http://api.brain-map.org/grid_data/download/72182946?include=energy
Stag3	1.076	http://api.brain-map.org/grid_data/download/69887319?include=energy
Olfr1104	1.076	http://api.brain-map.org/grid_data/download/74720591?include=energy
A230074B11Rik	1.076	http://api.brain-map.org/grid_data/download/69447984?include=energy
Nsun4	1.076	http://api.brain-map.org/grid_data/download/68445036?include=energy
Gaa	1.076	http://api.brain-map.org/grid_data/download/356090?include=energy
Prss41	1.076	http://api.brain-map.org/grid_data/download/74580727?include=energy
E130203B14Rik	1.076	http://api.brain-map.org/grid_data/download/70238997?include=energy
Fam158a	1.076	http://api.brain-map.org/grid_data/download/68632160?include=energy
Zfp410	1.076	http://api.brain-map.org/grid_data/download/68410476?include=energy
5730588L14Rik	1.076	http://api.brain-map.org/grid_data/download/71020962?include=energy
LOC435515	1.076	http://api.brain-map.org/grid_data/download/74822603?include=energy
Dnahc12	1.076	http://api.brain-map.org/grid_data/download/74988273?include=energy
Pias4	1.076	http://api.brain-map.org/grid_data/download/77414895?include=energy
Ctnnal1	1.076	http://api.brain-map.org/grid_data/download/68667097?include=energy
Acyp2	1.076	http://api.brain-map.org/grid_data/download/73930820?include=energy
Bmp5	1.076	http://api.brain-map.org/grid_data/download/73817428?include=energy
Golga7	1.076	http://api.brain-map.org/grid_data/download/68321904?include=energy
Naca	1.075	http://api.brain-map.org/grid_data/download/69170150?include=energy
LOC432978	1.075	http://api.brain-map.org/grid_data/download/70611443?include=energy
Smad4	1.075	http://api.brain-map.org/grid_data/download/71656698?include=energy
4930544M13Rik	1.075	http://api.brain-map.org/grid_data/download/70218306?include=energy
Pllp	1.075	http://api.brain-map.org/grid_data/download/1880?include=energy
Cldn2	1.075	http://api.brain-map.org/grid_data/download/75694900?include=energy
Gpr137c	1.075	http://api.brain-map.org/grid_data/download/70298423?include=energy
9330154K18Rik	1.075	http://api.brain-map.org/grid_data/download/69446643?include=energy
Agxt2	1.075	http://api.brain-map.org/grid_data/download/74579461?include=energy
Padi2	1.074	http://api.brain-map.org/grid_data/download/69013438?include=energy
Sbno2	1.074	http://api.brain-map.org/grid_data/download/68744104?include=energy
BC005764	1.074	http://api.brain-map.org/grid_data/download/73925724?include=energy
Sypl	1.074	http://api.brain-map.org/grid_data/download/75233089?include=energy
LOC381279	1.074	http://api.brain-map.org/grid_data/download/70596023?include=energy
Spag16	1.074	http://api.brain-map.org/grid_data/download/75080199?include=energy
Rffl	1.074	http://api.brain-map.org/grid_data/download/73584861?include=energy
Slc22a23	1.074	http://api.brain-map.org/grid_data/download/72128754?include=energy
Pip5kl1	1.074	http://api.brain-map.org/grid_data/download/71381103?include=energy
Csf1r	1.074	http://api.brain-map.org/grid_data/download/69608106?include=energy
Rpl6	1.074	http://api.brain-map.org/grid_data/download/69818317?include=energy
Xaf1	1.074	http://api.brain-map.org/grid_data/download/70595841?include=energy
0610012H03Rik	1.073	http://api.brain-map.org/grid_data/download/69028635?include=energy
Pdha1	1.073	http://api.brain-map.org/grid_data/download/323397?include=energy
Ublcp1	1.073	http://api.brain-map.org/grid_data/download/70634406?include=energy
Tcn2	1.073	http://api.brain-map.org/grid_data/download/71924389?include=energy
Kctd12b	1.073	http://api.brain-map.org/grid_data/download/71529983?include=energy
Birc7	1.073	http://api.brain-map.org/grid_data/download/69529322?include=energy
Stat6	1.073	http://api.brain-map.org/grid_data/download/69290549?include=energy
Nkx6-2	1.073	http://api.brain-map.org/grid_data/download/71325276?include=energy
Tpsab1	1.073	http://api.brain-map.org/grid_data/download/71616784?include=energy
Itgb4	1.073	http://api.brain-map.org/grid_data/download/73514695?include=energy
Kctd9	1.073	http://api.brain-map.org/grid_data/download/70813924?include=energy
Zyg11a	1.073	http://api.brain-map.org/grid_data/download/71574510?include=energy
Sh2d5	1.073	http://api.brain-map.org/grid_data/download/71380914?include=energy
H2afz	1.073	http://api.brain-map.org/grid_data/download/69734875?include=energy
Peg3	1.073	http://api.brain-map.org/grid_data/download/69289693?include=energy
Kcnip4	1.073	http://api.brain-map.org/grid_data/download/71924387?include=energy
Gja1	1.072	http://api.brain-map.org/grid_data/download/2255?include=energy
Col11a1	1.072	http://api.brain-map.org/grid_data/download/71064195?include=energy
Gdap1	1.072	http://api.brain-map.org/grid_data/download/69104747?include=energy
Edn3	1.072	http://api.brain-map.org/grid_data/download/69671893?include=energy
Gpx4	1.072	http://api.brain-map.org/grid_data/download/69734572?include=energy
Bcas3	1.072	http://api.brain-map.org/grid_data/download/69529256?include=energy
Gm836	1.072	http://api.brain-map.org/grid_data/download/70298340?include=energy
9430020K01Rik	1.072	http://api.brain-map.org/grid_data/download/75988537?include=energy
Slc6a13	1.072	http://api.brain-map.org/grid_data/download/78534514?include=energy
Nt5c2	1.072	http://api.brain-map.org/grid_data/download/69134634?include=energy
Impact	1.072	http://api.brain-map.org/grid_data/download/594?include=energy
A930017M01Rik	1.072	http://api.brain-map.org/grid_data/download/71809049?include=energy
Stk39	1.072	http://api.brain-map.org/grid_data/download/68911013?include=energy
Zfp804a	1.072	http://api.brain-map.org/grid_data/download/73817426?include=energy
Txnrd1	1.072	http://api.brain-map.org/grid_data/download/74988746?include=energy
Atp8b1	1.072	http://api.brain-map.org/grid_data/download/71574449?include=energy
Gpr33	1.072	http://api.brain-map.org/grid_data/download/71307376?include=energy
Irx3	1.072	http://api.brain-map.org/grid_data/download/81654099?include=energy
Bbs4	1.072	http://api.brain-map.org/grid_data/download/69528094?include=energy
Tmem47	1.072	http://api.brain-map.org/grid_data/download/75081394?include=energy
Nsg2	1.072	http://api.brain-map.org/grid_data/download/67767440?include=energy
Gstp1	1.071	http://api.brain-map.org/grid_data/download/68910954?include=energy
AI452102	1.071	http://api.brain-map.org/grid_data/download/73818824?include=energy
A530080P10	1.071	http://api.brain-map.org/grid_data/download/70305305?include=energy
Aktip	1.071	http://api.brain-map.org/grid_data/download/1355?include=energy
Mus81	1.071	http://api.brain-map.org/grid_data/download/199202?include=energy
Slc22a15	1.071	http://api.brain-map.org/grid_data/download/72005995?include=energy
Tmem132e	1.071	http://api.brain-map.org/grid_data/download/69734446?include=energy
Armcx6	1.071	http://api.brain-map.org/grid_data/download/69169582?include=energy
Tnfsf11	1.071	http://api.brain-map.org/grid_data/download/69981169?include=energy
Zfp709	1.071	http://api.brain-map.org/grid_data/download/70430864?include=energy
Olfr574	1.071	http://api.brain-map.org/grid_data/download/74724211?include=energy
Cep57l1	1.071	http://api.brain-map.org/grid_data/download/70474935?include=energy
LOC433134	1.071	http://api.brain-map.org/grid_data/download/70722940?include=energy
Klk1b9	1.071	http://api.brain-map.org/grid_data/download/79904776?include=energy
Bex1	1.071	http://api.brain-map.org/grid_data/download/69116584?include=energy
Lrsam1	1.071	http://api.brain-map.org/grid_data/download/74583161?include=energy
Serpinh1	1.071	http://api.brain-map.org/grid_data/download/69873588?include=energy
Ceacam14	1.071	http://api.brain-map.org/grid_data/download/75080228?include=energy
Il1b	1.071	http://api.brain-map.org/grid_data/download/75694738?include=energy
A930024C03Rik*	1.071	http://api.brain-map.org/grid_data/download/74000504?include=energy
Pax6	1.07	http://api.brain-map.org/grid_data/download/764?include=energy
Spata1	1.07	http://api.brain-map.org/grid_data/download/69014428?include=energy
Atp11a	1.07	http://api.brain-map.org/grid_data/download/75043137?include=energy
Gm949	1.07	http://api.brain-map.org/grid_data/download/71764351?include=energy
Ccdc157	1.07	http://api.brain-map.org/grid_data/download/70528061?include=energy
Unc13c	1.07	http://api.brain-map.org/grid_data/download/2707?include=energy
Slc6a6	1.07	http://api.brain-map.org/grid_data/download/1850?include=energy
Mpst	1.07	http://api.brain-map.org/grid_data/download/69080002?include=energy
Eno3	1.07	http://api.brain-map.org/grid_data/download/69059972?include=energy
Mmp15	1.07	http://api.brain-map.org/grid_data/download/1383?include=energy
B230216G23Rik	1.07	http://api.brain-map.org/grid_data/download/74277638?include=energy
Mapk8ip1	1.07	http://api.brain-map.org/grid_data/download/69752059?include=energy
Tmbim4	1.07	http://api.brain-map.org/grid_data/download/69549176?include=energy
H2-T3	1.07	http://api.brain-map.org/grid_data/download/69734887?include=energy
Dync1h1	1.07	http://api.brain-map.org/grid_data/download/69627003?include=energy
TC1504563	1.07	http://api.brain-map.org/grid_data/download/70813083?include=energy
Tsku	1.07	http://api.brain-map.org/grid_data/download/69446935?include=energy
Abca3	1.07	http://api.brain-map.org/grid_data/download/69514536?include=energy
Cul4b	1.07	http://api.brain-map.org/grid_data/download/100142517?include=energy
Olfr640	1.07	http://api.brain-map.org/grid_data/download/73653470?include=energy
Adcy10	1.07	http://api.brain-map.org/grid_data/download/71281268?include=energy
Tpra1	1.069	http://api.brain-map.org/grid_data/download/71654155?include=energy
AW046396	1.069	http://api.brain-map.org/grid_data/download/72340236?include=energy
Lcn14	1.069	http://api.brain-map.org/grid_data/download/70608352?include=energy
Cyp51	1.069	http://api.brain-map.org/grid_data/download/75934914?include=energy
Slc3a1	1.069	http://api.brain-map.org/grid_data/download/323825?include=energy
Pcolce2	1.069	http://api.brain-map.org/grid_data/download/68797601?include=energy
Mrps18a	1.069	http://api.brain-map.org/grid_data/download/71662900?include=energy
Plec	1.069	http://api.brain-map.org/grid_data/download/68499093?include=energy
Rab4a	1.069	http://api.brain-map.org/grid_data/download/69817880?include=energy
Palld	1.069	http://api.brain-map.org/grid_data/download/76135666?include=energy
Slc18a1	1.069	http://api.brain-map.org/grid_data/download/2583?include=energy
Ptpn5	1.069	http://api.brain-map.org/grid_data/download/74743293?include=energy
Enpp3	1.069	http://api.brain-map.org/grid_data/download/68269292?include=energy
Slc2a1	1.069	http://api.brain-map.org/grid_data/download/74882936?include=energy
Lpin1	1.069	http://api.brain-map.org/grid_data/download/74819460?include=energy
Syde2	1.069	http://api.brain-map.org/grid_data/download/69531019?include=energy
Serinc5	1.069	http://api.brain-map.org/grid_data/download/77278973?include=energy
Tmem163	1.069	http://api.brain-map.org/grid_data/download/71670711?include=energy
Mdh1	1.069	http://api.brain-map.org/grid_data/download/69752155?include=energy
1700061G19Rik	1.069	http://api.brain-map.org/grid_data/download/70201474?include=energy
Pmel	1.069	http://api.brain-map.org/grid_data/download/69873749?include=energy
8430410A17Rik	1.069	http://api.brain-map.org/grid_data/download/199201?include=energy
Crxos1	1.069	http://api.brain-map.org/grid_data/download/81790680?include=energy
Chrna3	1.068	http://api.brain-map.org/grid_data/download/72119655?include=energy
Ctif	1.068	http://api.brain-map.org/grid_data/download/68920413?include=energy
Prkra	1.068	http://api.brain-map.org/grid_data/download/564761?include=energy
Orc3	1.068	http://api.brain-map.org/grid_data/download/69174433?include=energy
Olfr1325	1.068	http://api.brain-map.org/grid_data/download/74720729?include=energy
Slain1	1.068	http://api.brain-map.org/grid_data/download/68080699?include=energy
Cntln	1.068	http://api.brain-map.org/grid_data/download/74431414?include=energy
Osmr	1.068	http://api.brain-map.org/grid_data/download/69782587?include=energy
Ccdc114	1.068	http://api.brain-map.org/grid_data/download/69528124?include=energy
Sostdc1	1.068	http://api.brain-map.org/grid_data/download/71836808?include=energy
Rpl36	1.068	http://api.brain-map.org/grid_data/download/68410536?include=energy
Pmfbp1	1.068	http://api.brain-map.org/grid_data/download/71717105?include=energy
Golgb1	1.068	http://api.brain-map.org/grid_data/download/71587757?include=energy
Spo11	1.068	http://api.brain-map.org/grid_data/download/70565270?include=energy
E030034C22Rik	1.068	http://api.brain-map.org/grid_data/download/68203499?include=energy
Smc1b	1.067	http://api.brain-map.org/grid_data/download/73424295?include=energy
Dek	1.067	http://api.brain-map.org/grid_data/download/69014860?include=energy
Sult1a1	1.067	http://api.brain-map.org/grid_data/download/75774114?include=energy
Lhx4	1.067	http://api.brain-map.org/grid_data/download/2377?include=energy
Fndc5	1.067	http://api.brain-map.org/grid_data/download/73635989?include=energy
Agpat9	1.067	http://api.brain-map.org/grid_data/download/72006001?include=energy
Dlgap5	1.067	http://api.brain-map.org/grid_data/download/69262256?include=energy
Ech1	1.067	http://api.brain-map.org/grid_data/download/69671863?include=energy
Stard9	1.067	http://api.brain-map.org/grid_data/download/75043136?include=energy
Slfn4	1.067	http://api.brain-map.org/grid_data/download/70523810?include=energy
Zfp81	1.067	http://api.brain-map.org/grid_data/download/70303600?include=energy
TC1523473	1.067	http://api.brain-map.org/grid_data/download/76003318?include=energy
Prss54	1.067	http://api.brain-map.org/grid_data/download/72283462?include=energy
Pus1	1.067	http://api.brain-map.org/grid_data/download/532760?include=energy
Trpa1	1.067	http://api.brain-map.org/grid_data/download/70230939?include=energy
Gm953	1.067	http://api.brain-map.org/grid_data/download/2419?include=energy
Samt3	1.067	http://api.brain-map.org/grid_data/download/71920189?include=energy
Mdn1	1.067	http://api.brain-map.org/grid_data/download/70565939?include=energy
Gpr128	1.067	http://api.brain-map.org/grid_data/download/71764886?include=energy
Penk	1.067	http://api.brain-map.org/grid_data/download/74881286?include=energy
Ropn1	1.067	http://api.brain-map.org/grid_data/download/69113865?include=energy
Hjurp	1.067	http://api.brain-map.org/grid_data/download/69838337?include=energy
Mm.240073	1.066	http://api.brain-map.org/grid_data/download/74431420?include=energy
Olfr143	1.066	http://api.brain-map.org/grid_data/download/73653326?include=energy
Spn	1.066	http://api.brain-map.org/grid_data/download/72002080?include=energy
Tmem8b	1.066	http://api.brain-map.org/grid_data/download/74431315?include=energy
Relt	1.066	http://api.brain-map.org/grid_data/download/77869869?include=energy
Puf60	1.066	http://api.brain-map.org/grid_data/download/69202675?include=energy
Tk1	1.066	http://api.brain-map.org/grid_data/download/71616166?include=energy
Folh1	1.066	http://api.brain-map.org/grid_data/download/73512554?include=energy
Bcl2	1.066	http://api.brain-map.org/grid_data/download/69529274?include=energy
Phf14	1.066	http://api.brain-map.org/grid_data/download/199216?include=energy
Tcf25	1.066	http://api.brain-map.org/grid_data/download/293034?include=energy
Otop1	1.065	http://api.brain-map.org/grid_data/download/71210583?include=energy
Cd93	1.065	http://api.brain-map.org/grid_data/download/69530852?include=energy
Nalcn	1.065	http://api.brain-map.org/grid_data/download/69448686?include=energy
Aim1	1.065	http://api.brain-map.org/grid_data/download/71250315?include=energy
Gm682	1.065	http://api.brain-map.org/grid_data/download/71668561?include=energy
Fcgbp	1.065	http://api.brain-map.org/grid_data/download/69448662?include=energy
1700112H15Rik	1.065	http://api.brain-map.org/grid_data/download/71920195?include=energy
Serpinb7	1.065	http://api.brain-map.org/grid_data/download/71656736?include=energy
Atp8a2	1.065	http://api.brain-map.org/grid_data/download/73925723?include=energy
Zswim2	1.065	http://api.brain-map.org/grid_data/download/70303522?include=energy
Bckdhb	1.065	http://api.brain-map.org/grid_data/download/71836890?include=energy
Sox11	1.065	http://api.brain-map.org/grid_data/download/79912528?include=energy
Olfr1024	1.065	http://api.brain-map.org/grid_data/download/74720555?include=energy
Actbl2	1.065	http://api.brain-map.org/grid_data/download/72183012?include=energy
Enox1	1.065	http://api.brain-map.org/grid_data/download/68744438?include=energy
Iscu	1.065	http://api.brain-map.org/grid_data/download/74819565?include=energy
Lamp2	1.065	http://api.brain-map.org/grid_data/download/69736028?include=energy
Six3	1.065	http://api.brain-map.org/grid_data/download/81654113?include=energy
Rcn1	1.065	http://api.brain-map.org/grid_data/download/79591573?include=energy
Hgd	1.065	http://api.brain-map.org/grid_data/download/75990703?include=energy
St8sia6	1.065	http://api.brain-map.org/grid_data/download/73929596?include=energy
Dennd4a	1.065	http://api.brain-map.org/grid_data/download/69524473?include=energy
Reg3b	1.065	http://api.brain-map.org/grid_data/download/74277401?include=energy
Fam161b	1.064	http://api.brain-map.org/grid_data/download/74277676?include=energy
Exosc5	1.064	http://api.brain-map.org/grid_data/download/71160967?include=energy
Scube3	1.064	http://api.brain-map.org/grid_data/download/74988656?include=energy
Olfr31	1.064	http://api.brain-map.org/grid_data/download/74720979?include=energy
4921507P07Rik	1.064	http://api.brain-map.org/grid_data/download/72076641?include=energy
Pdcd6ip	1.064	http://api.brain-map.org/grid_data/download/68445042?include=energy
Serpina1f	1.064	http://api.brain-map.org/grid_data/download/71919086?include=energy
Gabrr2	1.064	http://api.brain-map.org/grid_data/download/79589524?include=energy
Ttll8	1.064	http://api.brain-map.org/grid_data/download/71808802?include=energy

Traf4	1.064	http://api.brain-map.org/grid_data/download/70192033?include=energy
Gzmk	1.064	http://api.brain-map.org/grid_data/download/69734869?include=energy
Fam40b	1.064	http://api.brain-map.org/grid_data/download/72283809?include=energy
Eif2b1	1.064	http://api.brain-map.org/grid_data/download/72727?include=energy
Tfdp2	1.064	http://api.brain-map.org/grid_data/download/74509589?include=energy
Papolb	1.064	http://api.brain-map.org/grid_data/download/69782718?include=energy
Enkur	1.064	http://api.brain-map.org/grid_data/download/71892173?include=energy
TC1447992	1.064	http://api.brain-map.org/grid_data/download/72472383?include=energy
Mm.120298	1.064	http://api.brain-map.org/grid_data/download/71147966?include=energy
Nr0b1	1.063	http://api.brain-map.org/grid_data/download/77464954?include=energy
Wwtr1	1.063	http://api.brain-map.org/grid_data/download/70429501?include=energy
Unkl	1.063	http://api.brain-map.org/grid_data/download/69353209?include=energy
Sln	1.063	http://api.brain-map.org/grid_data/download/74425522?include=energy
Fbxo32	1.063	http://api.brain-map.org/grid_data/download/68637473?include=energy
Olfr1260	1.063	http://api.brain-map.org/grid_data/download/73635556?include=energy
F630111L10Rik	1.063	http://api.brain-map.org/grid_data/download/69672210?include=energy
Pikfyve	1.063	http://api.brain-map.org/grid_data/download/73949701?include=energy
Jam3	1.063	http://api.brain-map.org/grid_data/download/77465024?include=energy
Gm16853	1.063	http://api.brain-map.org/grid_data/download/72005997?include=energy
Tmem130	1.063	http://api.brain-map.org/grid_data/download/73930834?include=energy
Sgpp1	1.063	http://api.brain-map.org/grid_data/download/71210241?include=energy
Prrxl1	1.063	http://api.brain-map.org/grid_data/download/71325372?include=energy
Epb4.1l3	1.063	http://api.brain-map.org/grid_data/download/72283605?include=energy
Slc34a1	1.063	http://api.brain-map.org/grid_data/download/1842?include=energy
Fgf17	1.063	http://api.brain-map.org/grid_data/download/71529797?include=energy
Etnk1	1.063	http://api.brain-map.org/grid_data/download/69672168?include=energy
S100b	1.063	http://api.brain-map.org/grid_data/download/923?include=energy
Sdsl	1.063	http://api.brain-map.org/grid_data/download/67815939?include=energy
Cplx1	1.062	http://api.brain-map.org/grid_data/download/67752308?include=energy
Rab3gap1	1.062	http://api.brain-map.org/grid_data/download/68924661?include=energy
Hadha	1.062	http://api.brain-map.org/grid_data/download/68744988?include=energy
Clca5	1.062	http://api.brain-map.org/grid_data/download/73584843?include=energy
Areg	1.062	http://api.brain-map.org/grid_data/download/104610?include=energy
Esrra	1.062	http://api.brain-map.org/grid_data/download/1351?include=energy
Mdm4	1.062	http://api.brain-map.org/grid_data/download/512777?include=energy
Ppapdc1a	1.062	http://api.brain-map.org/grid_data/download/74881927?include=energy
Xkr9	1.062	http://api.brain-map.org/grid_data/download/71668565?include=energy
Ptpn4	1.062	http://api.brain-map.org/grid_data/download/74743283?include=energy
Tex16	1.062	http://api.brain-map.org/grid_data/download/70302461?include=energy
Cyc1	1.062	http://api.brain-map.org/grid_data/download/357103?include=energy
Chrnb2	1.062	http://api.brain-map.org/grid_data/download/2097?include=energy
Stx16	1.062	http://api.brain-map.org/grid_data/download/70302288?include=energy
Ptbp1	1.062	http://api.brain-map.org/grid_data/download/356857?include=energy
Cidea	1.062	http://api.brain-map.org/grid_data/download/73992929?include=energy
Phospho2	1.062	http://api.brain-map.org/grid_data/download/67787356?include=energy
Pparg	1.062	http://api.brain-map.org/grid_data/download/2495?include=energy
Ppnr	1.062	http://api.brain-map.org/grid_data/download/76003315?include=energy
Ttc12	1.061	http://api.brain-map.org/grid_data/download/79393966?include=energy
Cdh1	1.061	http://api.brain-map.org/grid_data/download/70918862?include=energy
Olfr923	1.061	http://api.brain-map.org/grid_data/download/74734991?include=energy
5330403O16Rik*	1.061	http://api.brain-map.org/grid_data/download/73870545?include=energy
Myo6	1.061	http://api.brain-map.org/grid_data/download/71789879?include=energy
Fsip2	1.061	http://api.brain-map.org/grid_data/download/71587685?include=energy
Oosp1	1.061	http://api.brain-map.org/grid_data/download/71668985?include=energy
Dnm3	1.061	http://api.brain-map.org/grid_data/download/74641219?include=energy
Rhob	1.061	http://api.brain-map.org/grid_data/download/70429393?include=energy
AI987944	1.061	http://api.brain-map.org/grid_data/download/73513398?include=energy
Tmigd1	1.061	http://api.brain-map.org/grid_data/download/70201630?include=energy
Cdc42ep3	1.061	http://api.brain-map.org/grid_data/download/75146001?include=energy
H2-M9	1.061	http://api.brain-map.org/grid_data/download/70724810?include=energy
Arxes1	1.061	http://api.brain-map.org/grid_data/download/45841?include=energy
1810064F22Rik	1.061	http://api.brain-map.org/grid_data/download/74000696?include=energy
Rps24	1.061	http://api.brain-map.org/grid_data/download/68668078?include=energy
Cacna1a	1.06	http://api.brain-map.org/grid_data/download/79761029?include=energy
Rpl36a	1.06	http://api.brain-map.org/grid_data/download/68744648?include=energy
5530400C07Rik*	1.06	http://api.brain-map.org/grid_data/download/73808180?include=energy
Ctps	1.06	http://api.brain-map.org/grid_data/download/1185?include=energy
Elf5	1.06	http://api.brain-map.org/grid_data/download/119684?include=energy
Fam19a5	1.06	http://api.brain-map.org/grid_data/download/69059974?include=energy
Alox8	1.06	http://api.brain-map.org/grid_data/download/73718055?include=energy
LOC546392	1.06	http://api.brain-map.org/grid_data/download/73584867?include=energy
Itgb1bp1	1.06	http://api.brain-map.org/grid_data/download/71015806?include=energy
F730046H10Rik	1.06	http://api.brain-map.org/grid_data/download/71764419?include=energy
1700022A21Rik	1.06	http://api.brain-map.org/grid_data/download/70723632?include=energy
9830137J09Rik	1.06	http://api.brain-map.org/grid_data/download/70608535?include=energy
Pcsk9	1.06	http://api.brain-map.org/grid_data/download/75693192?include=energy
Glul	1.06	http://api.brain-map.org/grid_data/download/74988768?include=energy
Gstk1	1.06	http://api.brain-map.org/grid_data/download/72129245?include=energy
Ap2m1	1.059	http://api.brain-map.org/grid_data/download/101?include=energy
Gm4836	1.059	http://api.brain-map.org/grid_data/download/69174439?include=energy
Crot	1.059	http://api.brain-map.org/grid_data/download/71616361?include=energy
Dbndd2	1.059	http://api.brain-map.org/grid_data/download/45668?include=energy
Cgn	1.059	http://api.brain-map.org/grid_data/download/71579885?include=energy
Snhg7	1.059	http://api.brain-map.org/grid_data/download/1968?include=energy
Oog1	1.059	http://api.brain-map.org/grid_data/download/77904883?include=energy
Gjb1	1.059	http://api.brain-map.org/grid_data/download/69257343?include=energy
Irx2	1.059	http://api.brain-map.org/grid_data/download/1752?include=energy
H2-Oa	1.059	http://api.brain-map.org/grid_data/download/74003381?include=energy
Otoa	1.059	http://api.brain-map.org/grid_data/download/69782599?include=energy
Ppard	1.059	http://api.brain-map.org/grid_data/download/77924442?include=energy
Aldh1a1	1.059	http://api.brain-map.org/grid_data/download/72103809?include=energy
Ndufb8	1.059	http://api.brain-map.org/grid_data/download/74743256?include=energy
1700009J07Rik	1.059	http://api.brain-map.org/grid_data/download/69116978?include=energy
Nedd4	1.059	http://api.brain-map.org/grid_data/download/70813143?include=energy
LOC545458	1.059	http://api.brain-map.org/grid_data/download/71574455?include=energy
Resp18	1.059	http://api.brain-map.org/grid_data/download/71015810?include=energy
Marco	1.059	http://api.brain-map.org/grid_data/download/71789789?include=energy
Rnd2	1.059	http://api.brain-map.org/grid_data/download/73512201?include=energy
Bphl	1.059	http://api.brain-map.org/grid_data/download/71608345?include=energy
C87499	1.059	http://api.brain-map.org/grid_data/download/68667649?include=energy
A230006K03Rik	1.059	http://api.brain-map.org/grid_data/download/71210025?include=energy
Rfk	1.058	http://api.brain-map.org/grid_data/download/71064080?include=energy
Mrpl20	1.058	http://api.brain-map.org/grid_data/download/68341603?include=energy
Pstpip1	1.058	http://api.brain-map.org/grid_data/download/71717135?include=energy
Gm5600	1.058	http://api.brain-map.org/grid_data/download/71213868?include=energy
Gm1335	1.058	http://api.brain-map.org/grid_data/download/74988767?include=energy
Emb	1.058	http://api.brain-map.org/grid_data/download/73930836?include=energy
Akr1e1	1.058	http://api.brain-map.org/grid_data/download/356093?include=energy
Omd	1.058	http://api.brain-map.org/grid_data/download/69782569?include=energy
Adh4	1.058	http://api.brain-map.org/grid_data/download/75988240?include=energy
Ctsq	1.058	http://api.brain-map.org/grid_data/download/71919206?include=energy
Icam4	1.058	http://api.brain-map.org/grid_data/download/70277997?include=energy
Hars	1.058	http://api.brain-map.org/grid_data/download/554?include=energy
LOC432612	1.058	http://api.brain-map.org/grid_data/download/74431008?include=energy
LOC433207	1.058	http://api.brain-map.org/grid_data/download/74431254?include=energy
Mrpl51	1.058	http://api.brain-map.org/grid_data/download/68410452?include=energy
Fam149a	1.058	http://api.brain-map.org/grid_data/download/100146089?include=energy
Cpsf3	1.058	http://api.brain-map.org/grid_data/download/68637629?include=energy
Lin28b	1.058	http://api.brain-map.org/grid_data/download/70595925?include=energy
Bcat1	1.058	http://api.brain-map.org/grid_data/download/71016665?include=energy
Surf2	1.057	http://api.brain-map.org/grid_data/download/71764583?include=energy
1700019J19Rik	1.057	http://api.brain-map.org/grid_data/download/71919547?include=energy
Zfp954	1.057	http://api.brain-map.org/grid_data/download/69837606?include=energy
Fv1	1.057	http://api.brain-map.org/grid_data/download/68667970?include=energy
Uaca	1.057	http://api.brain-map.org/grid_data/download/74800883?include=energy
Olfr412	1.057	http://api.brain-map.org/grid_data/download/74724085?include=energy
LOC434372	1.057	http://api.brain-map.org/grid_data/download/70920141?include=energy
Tmem107	1.057	http://api.brain-map.org/grid_data/download/70303731?include=energy
Myt1	1.057	http://api.brain-map.org/grid_data/download/71924077?include=energy
Hfe2	1.057	http://api.brain-map.org/grid_data/download/77800929?include=energy
Rln1	1.057	http://api.brain-map.org/grid_data/download/71020890?include=energy
Klk5	1.057	http://api.brain-map.org/grid_data/download/74300873?include=energy
1700066J24Rik	1.057	http://api.brain-map.org/grid_data/download/74357911?include=energy
Mrc1	1.057	http://api.brain-map.org/grid_data/download/74581428?include=energy
Gm832	1.057	http://api.brain-map.org/grid_data/download/70298334?include=energy
Plxnb1	1.057	http://api.brain-map.org/grid_data/download/1561?include=energy
Cda	1.057	http://api.brain-map.org/grid_data/download/74425539?include=energy
Mastl	1.057	http://api.brain-map.org/grid_data/download/69752083?include=energy
Pank4	1.057	http://api.brain-map.org/grid_data/download/69549312?include=energy
Phgdh	1.056	http://api.brain-map.org/grid_data/download/100143511?include=energy
Nkain3	1.056	http://api.brain-map.org/grid_data/download/70239009?include=energy
Tmod2	1.056	http://api.brain-map.org/grid_data/download/69116685?include=energy
Nfasc	1.056	http://api.brain-map.org/grid_data/download/68844086?include=energy
Enpp2	1.056	http://api.brain-map.org/grid_data/download/70613967?include=energy
Dpy19l3	1.056	http://api.brain-map.org/grid_data/download/71836886?include=energy
BC006779	1.056	http://api.brain-map.org/grid_data/download/68743925?include=energy
Slc39a13	1.056	http://api.brain-map.org/grid_data/download/69169505?include=energy
Mblac2	1.056	http://api.brain-map.org/grid_data/download/72283390?include=energy
Sqstm1	1.056	http://api.brain-map.org/grid_data/download/293263?include=energy
Slc9a3r1	1.056	http://api.brain-map.org/grid_data/download/69874066?include=energy
Usp33	1.056	http://api.brain-map.org/grid_data/download/71764757?include=energy
Coro7	1.056	http://api.brain-map.org/grid_data/download/69116572?include=energy
Agxt2l1	1.056	http://api.brain-map.org/grid_data/download/75079777?include=energy
1700001K19Rik	1.056	http://api.brain-map.org/grid_data/download/72081777?include=energy
Exosc9	1.056	http://api.brain-map.org/grid_data/download/633301?include=energy
4921539E11Rik	1.056	http://api.brain-map.org/grid_data/download/71891593?include=energy
Ptf1a	1.056	http://api.brain-map.org/grid_data/download/81657673?include=energy
Kidins220	1.056	http://api.brain-map.org/grid_data/download/69816512?include=energy
Vcl	1.055	http://api.brain-map.org/grid_data/download/71763927?include=energy
Fam134b	1.055	http://api.brain-map.org/grid_data/download/70634224?include=energy
Rassf4	1.055	http://api.brain-map.org/grid_data/download/74988636?include=energy
Adamtsl3	1.055	http://api.brain-map.org/grid_data/download/73925720?include=energy
Clcf1	1.055	http://api.brain-map.org/grid_data/download/75197654?include=energy
Irf8	1.055	http://api.brain-map.org/grid_data/download/79536566?include=energy
Nfatc2	1.055	http://api.brain-map.org/grid_data/download/70437816?include=energy
Steap1	1.055	http://api.brain-map.org/grid_data/download/70302276?include=energy
Cacna2d2	1.055	http://api.brain-map.org/grid_data/download/72119650?include=energy
Pdp1	1.055	http://api.brain-map.org/grid_data/download/74357581?include=energy
Prcp	1.055	http://api.brain-map.org/grid_data/download/74819671?include=energy
Itm2a	1.055	http://api.brain-map.org/grid_data/download/74881146?include=energy
Olig2	1.055	http://api.brain-map.org/grid_data/download/2448?include=energy
Efcab10	1.055	http://api.brain-map.org/grid_data/download/71891832?include=energy
Idh1	1.055	http://api.brain-map.org/grid_data/download/74511808?include=energy
Eif4e	1.055	http://api.brain-map.org/grid_data/download/73906527?include=energy
L1cam	1.054	http://api.brain-map.org/grid_data/download/79591595?include=energy
Calcoco1	1.054	http://api.brain-map.org/grid_data/download/69407086?include=energy
Sf1	1.054	http://api.brain-map.org/grid_data/download/68668024?include=energy
Rpl11	1.054	http://api.brain-map.org/grid_data/download/276067?include=energy
Ghsr	1.054	http://api.brain-map.org/grid_data/download/74511739?include=energy
Gprc5b	1.054	http://api.brain-map.org/grid_data/download/71836881?include=energy
Cyp2f2	1.054	http://api.brain-map.org/grid_data/download/100142527?include=energy
Lig1	1.054	http://api.brain-map.org/grid_data/download/637?include=energy
Il17rc	1.054	http://api.brain-map.org/grid_data/download/2319?include=energy
Creld1	1.054	http://api.brain-map.org/grid_data/download/69059968?include=energy
Slc12a3	1.054	http://api.brain-map.org/grid_data/download/2573?include=energy
Grk5	1.054	http://api.brain-map.org/grid_data/download/77371851?include=energy
Msi1	1.054	http://api.brain-map.org/grid_data/download/74509595?include=energy
Fpr-rs4	1.054	http://api.brain-map.org/grid_data/download/70723447?include=energy
Tnc	1.054	http://api.brain-map.org/grid_data/download/71717115?include=energy
Csf1	1.054	http://api.brain-map.org/grid_data/download/77932787?include=energy
Gramd1a	1.054	http://api.brain-map.org/grid_data/download/67979016?include=energy
Kdm4b	1.054	http://api.brain-map.org/grid_data/download/2331?include=energy
Gys2	1.053	http://api.brain-map.org/grid_data/download/77800927?include=energy
Pip	1.053	http://api.brain-map.org/grid_data/download/68632014?include=energy
Sipa1	1.053	http://api.brain-map.org/grid_data/download/69014404?include=energy
Fyttd1	1.053	http://api.brain-map.org/grid_data/download/71723827?include=energy
Ccdc150	1.053	http://api.brain-map.org/grid_data/download/74580020?include=energy
LOC432409	1.053	http://api.brain-map.org/grid_data/download/70300156?include=energy
Rrad	1.053	http://api.brain-map.org/grid_data/download/75080239?include=energy
Tpi1	1.053	http://api.brain-map.org/grid_data/download/70191997?include=energy
2600001M11Rik	1.053	http://api.brain-map.org/grid_data/download/70812853?include=energy
Uhrf1	1.053	http://api.brain-map.org/grid_data/download/68301150?include=energy
Cygb	1.053	http://api.brain-map.org/grid_data/download/72103830?include=energy
3110043A19Rik	1.053	http://api.brain-map.org/grid_data/download/70203652?include=energy
Uba52	1.053	http://api.brain-map.org/grid_data/download/69013702?include=energy
Mapk8ip2	1.053	http://api.brain-map.org/grid_data/download/68148737?include=energy
Retsat	1.053	http://api.brain-map.org/grid_data/download/68302011?include=energy
LOC433191	1.053	http://api.brain-map.org/grid_data/download/70722990?include=energy
Dennd5a	1.053	http://api.brain-map.org/grid_data/download/1526?include=energy
Olfr1324	1.053	http://api.brain-map.org/grid_data/download/74720723?include=energy
Ndufa6	1.053	http://api.brain-map.org/grid_data/download/69114998?include=energy
Bcam	1.053	http://api.brain-map.org/grid_data/download/71764144?include=energy
Gss	1.053	http://api.brain-map.org/grid_data/download/74273307?include=energy
Rpl10	1.053	http://api.brain-map.org/grid_data/download/68666661?include=energy
Wipi1	1.053	http://api.brain-map.org/grid_data/download/69261400?include=energy
LOC432413	1.053	http://api.brain-map.org/grid_data/download/70608460?include=energy
Slc25a12	1.052	http://api.brain-map.org/grid_data/download/68798133?include=energy
Yipf7	1.052	http://api.brain-map.org/grid_data/download/70218685?include=energy
2700060E02Rik	1.052	http://api.brain-map.org/grid_data/download/70527975?include=energy
2510009E07Rik	1.052	http://api.brain-map.org/grid_data/download/69202723?include=energy
Pcdh9	1.052	http://api.brain-map.org/grid_data/download/10?include=energy
Cox6b1	1.052	http://api.brain-map.org/grid_data/download/71724561?include=energy
2310016D23Rik	1.052	http://api.brain-map.org/grid_data/download/71991762?include=energy
Cd47	1.052	http://api.brain-map.org/grid_data/download/357100?include=energy
Gpr108	1.052	http://api.brain-map.org/grid_data/download/1619?include=energy
Ppef1	1.052	http://api.brain-map.org/grid_data/download/70300881?include=energy
Ostf1	1.052	http://api.brain-map.org/grid_data/download/73907505?include=energy
Kcns2	1.052	http://api.brain-map.org/grid_data/download/71924259?include=energy
Lamb2	1.052	http://api.brain-map.org/grid_data/download/68562975?include=energy
Ik	1.052	http://api.brain-map.org/grid_data/download/68443351?include=energy
Ino80d	1.052	http://api.brain-map.org/grid_data/download/71574504?include=energy
Mid2	1.052	http://api.brain-map.org/grid_data/download/77800893?include=energy
8030423J24Rik	1.052	http://api.brain-map.org/grid_data/download/73818845?include=energy
Bbs2	1.052	http://api.brain-map.org/grid_data/download/69028378?include=energy
Dusp26	1.052	http://api.brain-map.org/grid_data/download/68637706?include=energy
P2ry1	1.052	http://api.brain-map.org/grid_data/download/69863246?include=energy
Rasgef1b	1.052	http://api.brain-map.org/grid_data/download/71358553?include=energy
Pltp	1.052	http://api.brain-map.org/grid_data/download/74581394?include=energy
Bmper	1.052	http://api.brain-map.org/grid_data/download/77465060?include=energy
Gm17749	1.052	http://api.brain-map.org/grid_data/download/71992109?include=energy
Arl2bp	1.052	http://api.brain-map.org/grid_data/download/68910953?include=energy
Vwc2	1.052	http://api.brain-map.org/grid_data/download/77332096?include=energy
4930447C04Rik	1.052	http://api.brain-map.org/grid_data/download/71891761?include=energy
Slc30a9	1.051	http://api.brain-map.org/grid_data/download/73573181?include=energy
Bex2	1.051	http://api.brain-map.org/grid_data/download/68545917?include=energy
Rfx5	1.051	http://api.brain-map.org/grid_data/download/70813125?include=energy
Syngr2	1.051	http://api.brain-map.org/grid_data/download/72731?include=energy
Cdca3	1.051	http://api.brain-map.org/grid_data/download/68191462?include=energy
Stim1	1.051	http://api.brain-map.org/grid_data/download/68562994?include=energy
Rdx	1.051	http://api.brain-map.org/grid_data/download/75851020?include=energy
Pard3b	1.051	http://api.brain-map.org/grid_data/download/69816416?include=energy
Polr3f	1.051	http://api.brain-map.org/grid_data/download/68269694?include=energy
Gap43	1.051	http://api.brain-map.org/grid_data/download/70928270?include=energy
2410131K14Rik	1.051	http://api.brain-map.org/grid_data/download/69202711?include=energy
Gng11	1.051	http://api.brain-map.org/grid_data/download/1732?include=energy
Ppap2c	1.051	http://api.brain-map.org/grid_data/download/68269550?include=energy
LOC432928	1.051	http://api.brain-map.org/grid_data/download/73931424?include=energy
4930555G01Rik	1.05	http://api.brain-map.org/grid_data/download/70218360?include=energy
Vps39	1.05	http://api.brain-map.org/grid_data/download/74047842?include=energy
Slc28a3	1.05	http://api.brain-map.org/grid_data/download/70927425?include=energy
9330169L03Rik	1.05	http://api.brain-map.org/grid_data/download/69446667?include=energy
Uqcrc1	1.05	http://api.brain-map.org/grid_data/download/68744540?include=energy
Sepp1	1.05	http://api.brain-map.org/grid_data/download/79903768?include=energy
Reep1	1.05	http://api.brain-map.org/grid_data/download/74821680?include=energy
Card14	1.05	http://api.brain-map.org/grid_data/download/68444654?include=energy
Ifng	1.05	http://api.brain-map.org/grid_data/download/69735251?include=energy
Zwint	1.05	http://api.brain-map.org/grid_data/download/68157385?include=energy

Zfp462	1.05	http://api.brain-map.org/grid_data/download/71670736?include=energy
Mmp23	1.05	http://api.brain-map.org/grid_data/download/71764431?include=energy
Acss2	1.05	http://api.brain-map.org/grid_data/download/69013516?include=energy
Taldo1	1.05	http://api.brain-map.org/grid_data/download/74988562?include=energy
Leng1	1.05	http://api.brain-map.org/grid_data/download/76098383?include=energy
Speg	1.05	http://api.brain-map.org/grid_data/download/69526743?include=energy
5430402E10Rik	1.05	http://api.brain-map.org/grid_data/download/70218908?include=energy
Gabrq	1.05	http://api.brain-map.org/grid_data/download/79591569?include=energy
1700010L04Rik	1.05	http://api.brain-map.org/grid_data/download/72081783?include=energy
Xpo4	1.05	http://api.brain-map.org/grid_data/download/70303022?include=energy
Vmn1r230	1.05	http://api.brain-map.org/grid_data/download/69442229?include=energy
Ube2w	1.05	http://api.brain-map.org/grid_data/download/71211389?include=energy
Gm5514	1.05	http://api.brain-map.org/grid_data/download/74736213?include=energy
Akp3	1.05	http://api.brain-map.org/grid_data/download/70724565?include=energy
Sgol1	1.05	http://api.brain-map.org/grid_data/download/72001868?include=energy
LOC433493	1.049	http://api.brain-map.org/grid_data/download/70723319?include=energy
Phb2	1.049	http://api.brain-map.org/grid_data/download/69529250?include=energy
Mvk	1.049	http://api.brain-map.org/grid_data/download/79539594?include=energy
Serinc3	1.049	http://api.brain-map.org/grid_data/download/71616360?include=energy
A330049M08Rik	1.049	http://api.brain-map.org/grid_data/download/69059926?include=energy
Sez6l2	1.049	http://api.brain-map.org/grid_data/download/1407?include=energy
Slc38a10	1.049	http://api.brain-map.org/grid_data/download/69201696?include=energy
Glt25d1	1.049	http://api.brain-map.org/grid_data/download/70812762?include=energy
Slc25a39	1.049	http://api.brain-map.org/grid_data/download/74988740?include=energy
Slc25a42	1.049	http://api.brain-map.org/grid_data/download/69236646?include=energy
Zfp773	1.049	http://api.brain-map.org/grid_data/download/74821604?include=energy
Gbe1	1.049	http://api.brain-map.org/grid_data/download/75042250?include=energy
Ddx24	1.049	http://api.brain-map.org/grid_data/download/69014542?include=energy
Mbnl1	1.049	http://api.brain-map.org/grid_data/download/75934934?include=energy
Rft1	1.049	http://api.brain-map.org/grid_data/download/70565452?include=energy
Zfp558	1.049	http://api.brain-map.org/grid_data/download/74581307?include=energy
H2afj	1.049	http://api.brain-map.org/grid_data/download/71789757?include=energy
Trpc2	1.049	http://api.brain-map.org/grid_data/download/73592539?include=energy
Sept7	1.049	http://api.brain-map.org/grid_data/download/961?include=energy
Alcam	1.049	http://api.brain-map.org/grid_data/download/70928271?include=energy
Ciita	1.049	http://api.brain-map.org/grid_data/download/69530953?include=energy
Ak8	1.049	http://api.brain-map.org/grid_data/download/71232594?include=energy
Phlda1	1.049	http://api.brain-map.org/grid_data/download/68080675?include=energy
Pcgf5	1.049	http://api.brain-map.org/grid_data/download/70227802?include=energy
Wbp2nl	1.048	http://api.brain-map.org/grid_data/download/71891844?include=energy
1700011E24Rik	1.048	http://api.brain-map.org/grid_data/download/72081659?include=energy
Hoga1	1.048	http://api.brain-map.org/grid_data/download/70524282?include=energy
Olfr547	1.048	http://api.brain-map.org/grid_data/download/74724151?include=energy
9030419F21Rik	1.048	http://api.brain-map.org/grid_data/download/73635465?include=energy
Rnf219	1.048	http://api.brain-map.org/grid_data/download/276273?include=energy
Ick	1.048	http://api.brain-map.org/grid_data/download/68445065?include=energy
Klhdc9	1.048	http://api.brain-map.org/grid_data/download/69353152?include=energy
Ccr8	1.048	http://api.brain-map.org/grid_data/download/70593129?include=energy
Habp4	1.048	http://api.brain-map.org/grid_data/download/69734893?include=energy
Nsfl1c	1.048	http://api.brain-map.org/grid_data/download/69782413?include=energy
Gabbr1	1.048	http://api.brain-map.org/grid_data/download/68862120?include=energy
Olfr76	1.048	http://api.brain-map.org/grid_data/download/74277395?include=energy
Myh10	1.048	http://api.brain-map.org/grid_data/download/69781971?include=energy
Gnrhr	1.048	http://api.brain-map.org/grid_data/download/70593007?include=energy
Tgfbi	1.048	http://api.brain-map.org/grid_data/download/69980310?include=energy
LOC432531	1.048	http://api.brain-map.org/grid_data/download/70608598?include=energy
4930511M11Rik	1.048	http://api.brain-map.org/grid_data/download/71891635?include=energy
Vma21	1.048	http://api.brain-map.org/grid_data/download/71891545?include=energy
Olfr125	1.048	http://api.brain-map.org/grid_data/download/74720663?include=energy
Rbm28	1.048	http://api.brain-map.org/grid_data/download/74359431?include=energy
C230071H21Rik*	1.048	http://api.brain-map.org/grid_data/download/73811681?include=energy
Kntc1	1.048	http://api.brain-map.org/grid_data/download/70295440?include=energy
Slc4a3	1.048	http://api.brain-map.org/grid_data/download/69874000?include=energy
Prom1	1.048	http://api.brain-map.org/grid_data/download/79756306?include=energy
Mpnd	1.048	http://api.brain-map.org/grid_data/download/75146005?include=energy
Cox7a2	1.048	http://api.brain-map.org/grid_data/download/68301443?include=energy
Tradd	1.048	http://api.brain-map.org/grid_data/download/69134193?include=energy
Olfr323	1.048	http://api.brain-map.org/grid_data/download/74720991?include=energy
Sparc	1.047	http://api.brain-map.org/grid_data/download/73788443?include=energy
Vmn1r75	1.047	http://api.brain-map.org/grid_data/download/70785678?include=energy
1100001G20Rik	1.047	http://api.brain-map.org/grid_data/download/74427075?include=energy
Zcchc17	1.047	http://api.brain-map.org/grid_data/download/69015434?include=energy
Rnf152	1.047	http://api.brain-map.org/grid_data/download/75043151?include=energy
Igf2	1.047	http://api.brain-map.org/grid_data/download/2313?include=energy
Foxj1	1.047	http://api.brain-map.org/grid_data/download/81790662?include=energy
Fsip1	1.047	http://api.brain-map.org/grid_data/download/71892143?include=energy
Ncdn	1.047	http://api.brain-map.org/grid_data/download/684?include=energy
Usp54	1.047	http://api.brain-map.org/grid_data/download/70194660?include=energy
Tmem29	1.047	http://api.brain-map.org/grid_data/download/75080656?include=energy
Dnajc15	1.047	http://api.brain-map.org/grid_data/download/68744683?include=energy
Limk1	1.047	http://api.brain-map.org/grid_data/download/79908802?include=energy
Rpl4	1.047	http://api.brain-map.org/grid_data/download/69095958?include=energy
D10Jhu81e	1.047	http://api.brain-map.org/grid_data/download/68341412?include=energy
Htt	1.047	http://api.brain-map.org/grid_data/download/69734971?include=energy
Nr2e3	1.047	http://api.brain-map.org/grid_data/download/2434?include=energy
B3galt4	1.047	http://api.brain-map.org/grid_data/download/69527902?include=energy
Esd	1.047	http://api.brain-map.org/grid_data/download/69672144?include=energy
Enpp5	1.047	http://api.brain-map.org/grid_data/download/69080062?include=energy
Adamdec1	1.047	http://api.brain-map.org/grid_data/download/68924763?include=energy
Slc25a37	1.046	http://api.brain-map.org/grid_data/download/68445000?include=energy
Gm9925	1.046	http://api.brain-map.org/grid_data/download/70723008?include=energy
Reep5	1.046	http://api.brain-map.org/grid_data/download/2159?include=energy
Gm1571	1.046	http://api.brain-map.org/grid_data/download/74821862?include=energy
Gm9949	1.046	http://api.brain-map.org/grid_data/download/71808490?include=energy
Ascl1	1.046	http://api.brain-map.org/grid_data/download/73817423?include=energy
Nanos1	1.046	http://api.brain-map.org/grid_data/download/70546245?include=energy
Gata5	1.046	http://api.brain-map.org/grid_data/download/71307346?include=energy
Crat	1.046	http://api.brain-map.org/grid_data/download/2119?include=energy
Tbl3	1.046	http://api.brain-map.org/grid_data/download/248328?include=energy
Tmem41b	1.046	http://api.brain-map.org/grid_data/download/75750294?include=energy
Pou1f1	1.046	http://api.brain-map.org/grid_data/download/69117158?include=energy
Tm6sf2	1.046	http://api.brain-map.org/grid_data/download/69173337?include=energy
Napsa	1.046	http://api.brain-map.org/grid_data/download/69549295?include=energy
Gm597	1.046	http://api.brain-map.org/grid_data/download/70295620?include=energy
D430040D24Rik	1.045	http://api.brain-map.org/grid_data/download/71160316?include=energy
Vmn1r76	1.045	http://api.brain-map.org/grid_data/download/70785666?include=energy
Zswim4	1.045	http://api.brain-map.org/grid_data/download/70523674?include=energy
Npdc1	1.045	http://api.brain-map.org/grid_data/download/715?include=energy
Fignl1	1.045	http://api.brain-map.org/grid_data/download/69672468?include=energy
LOC433117	1.045	http://api.brain-map.org/grid_data/download/71808534?include=energy
Caly	1.045	http://api.brain-map.org/grid_data/download/75831782?include=energy
Pstpip2	1.045	http://api.brain-map.org/grid_data/download/80514000?include=energy
Sdha	1.045	http://api.brain-map.org/grid_data/download/67815548?include=energy
Sephs1	1.045	http://api.brain-map.org/grid_data/download/69873528?include=energy
Cyp2j6	1.045	http://api.brain-map.org/grid_data/download/77278979?include=energy
Ctsw	1.045	http://api.brain-map.org/grid_data/download/75041420?include=energy
Ndufa13	1.045	http://api.brain-map.org/grid_data/download/69838573?include=energy
Clec2f-ps	1.045	http://api.brain-map.org/grid_data/download/74735289?include=energy
Fam96b	1.045	http://api.brain-map.org/grid_data/download/74819685?include=energy
Synj2bp	1.045	http://api.brain-map.org/grid_data/download/77414884?include=energy
Wdr72	1.045	http://api.brain-map.org/grid_data/download/74277303?include=energy
Nat1	1.045	http://api.brain-map.org/grid_data/download/71234532?include=energy
Rassf2	1.045	http://api.brain-map.org/grid_data/download/68744432?include=energy
Mm.208428	1.045	http://api.brain-map.org/grid_data/download/71152384?include=energy
1600027N09Rik	1.045	http://api.brain-map.org/grid_data/download/69353318?include=energy
Fam120b	1.045	http://api.brain-map.org/grid_data/download/71724792?include=energy
Efhc2	1.045	http://api.brain-map.org/grid_data/download/70717365?include=energy
Slc24a6	1.045	http://api.brain-map.org/grid_data/download/78634029?include=energy
Rpl28	1.045	http://api.brain-map.org/grid_data/download/69173436?include=energy
Zfp592	1.044	http://api.brain-map.org/grid_data/download/73870668?include=energy
A330074K22Rik	1.044	http://api.brain-map.org/grid_data/download/70300475?include=energy
Prkaca	1.044	http://api.brain-map.org/grid_data/download/532575?include=energy
Shfm1	1.044	http://api.brain-map.org/grid_data/download/79544852?include=energy
Scin	1.044	http://api.brain-map.org/grid_data/download/69873445?include=energy
Mtap4	1.044	http://api.brain-map.org/grid_data/download/71587919?include=energy
Mum1	1.044	http://api.brain-map.org/grid_data/download/69169923?include=energy
1700028P14Rik	1.044	http://api.brain-map.org/grid_data/download/69114328?include=energy
Olfr523	1.044	http://api.brain-map.org/grid_data/download/74724145?include=energy
Mapk9	1.044	http://api.brain-map.org/grid_data/download/68563000?include=energy
Blvra	1.044	http://api.brain-map.org/grid_data/download/75850398?include=energy
Tmtc4	1.044	http://api.brain-map.org/grid_data/download/76081876?include=energy
Tnfaip6	1.044	http://api.brain-map.org/grid_data/download/513012?include=energy
Mttp	1.044	http://api.brain-map.org/grid_data/download/323183?include=energy
LOC436018	1.044	http://api.brain-map.org/grid_data/download/74431327?include=energy
Pydc3	1.044	http://api.brain-map.org/grid_data/download/71808409?include=energy
Clcn6	1.044	http://api.brain-map.org/grid_data/download/69237143?include=energy
Ccl28	1.044	http://api.brain-map.org/grid_data/download/75831761?include=energy
Tprgl	1.044	http://api.brain-map.org/grid_data/download/69080068?include=energy
Scd3	1.044	http://api.brain-map.org/grid_data/download/74882915?include=energy
Apc2	1.044	http://api.brain-map.org/grid_data/download/69526731?include=energy
LOC328644	1.044	http://api.brain-map.org/grid_data/download/71765034?include=energy
Nefm	1.044	http://api.brain-map.org/grid_data/download/73817434?include=energy
Ccdc109b	1.044	http://api.brain-map.org/grid_data/download/72129249?include=energy
G370041N07Rik*	1.044	http://api.brain-map.org/grid_data/download/73716692?include=energy
5230400M03Rik	1.044	http://api.brain-map.org/grid_data/download/72182981?include=energy
Adamts14	1.044	http://api.brain-map.org/grid_data/download/71489809?include=energy
Aebp2	1.044	http://api.brain-map.org/grid_data/download/68442859?include=energy
Cxx1a	1.044	http://api.brain-map.org/grid_data/download/71724768?include=energy
9630041A04Rik	1.043	http://api.brain-map.org/grid_data/download/73617594?include=energy
Commd2	1.043	http://api.brain-map.org/grid_data/download/70720254?include=energy
2010002M12Rik	1.043	http://api.brain-map.org/grid_data/download/70203411?include=energy
Acss1	1.043	http://api.brain-map.org/grid_data/download/67881763?include=energy
Col9a3	1.043	http://api.brain-map.org/grid_data/download/70928304?include=energy
Dbpht1	1.043	http://api.brain-map.org/grid_data/download/71580216?include=energy
Npm3	1.043	http://api.brain-map.org/grid_data/download/71763516?include=energy
Chchd10	1.043	http://api.brain-map.org/grid_data/download/75197594?include=energy
Mrgprd	1.043	http://api.brain-map.org/grid_data/download/76003353?include=energy
Pcdhb17	1.043	http://api.brain-map.org/grid_data/download/1908?include=energy
Fkbp11	1.043	http://api.brain-map.org/grid_data/download/68191450?include=energy
Tm2d3	1.043	http://api.brain-map.org/grid_data/download/77414123?include=energy
Gngt2	1.043	http://api.brain-map.org/grid_data/download/75860991?include=energy
Dnahc3	1.043	http://api.brain-map.org/grid_data/download/71587559?include=energy
Slc16a13	1.043	http://api.brain-map.org/grid_data/download/119699?include=energy
Mut	1.043	http://api.brain-map.org/grid_data/download/76098394?include=energy
Inpp1	1.043	http://api.brain-map.org/grid_data/download/67870434?include=energy
A130030D18Rik	1.043	http://api.brain-map.org/grid_data/download/74277353?include=energy
2010015L04Rik	1.043	http://api.brain-map.org/grid_data/download/73497738?include=energy
LOC433088	1.043	http://api.brain-map.org/grid_data/download/71924504?include=energy
Acta2	1.043	http://api.brain-map.org/grid_data/download/68743877?include=energy
Rbfox1	1.043	http://api.brain-map.org/grid_data/download/71280790?include=energy
Adamts1	1.043	http://api.brain-map.org/grid_data/download/69?include=energy
Olfr1271	1.043	http://api.brain-map.org/grid_data/download/74720675?include=energy
Add1	1.042	http://api.brain-map.org/grid_data/download/68631935?include=energy
Cebpg	1.042	http://api.brain-map.org/grid_data/download/70539130?include=energy
Mtap1b	1.042	http://api.brain-map.org/grid_data/download/73834355?include=energy
Anapc4	1.042	http://api.brain-map.org/grid_data/download/68342324?include=energy
Gabrb2	1.042	http://api.brain-map.org/grid_data/download/76115725?include=energy
Sult1d1	1.042	http://api.brain-map.org/grid_data/download/69169445?include=energy
Npepl1	1.042	http://api.brain-map.org/grid_data/download/69782342?include=energy
Sgpp2	1.042	http://api.brain-map.org/grid_data/download/70301275?include=energy
2310002J15Rik	1.042	http://api.brain-map.org/grid_data/download/70723656?include=energy
Fam198a	1.042	http://api.brain-map.org/grid_data/download/71021041?include=energy
Clic6	1.042	http://api.brain-map.org/grid_data/download/1984?include=energy
1700026J12Rik	1.042	http://api.brain-map.org/grid_data/download/74634802?include=energy
Magee1	1.042	http://api.brain-map.org/grid_data/download/69262114?include=energy
Cacna1c	1.042	http://api.brain-map.org/grid_data/download/69236891?include=energy
Reg4	1.042	http://api.brain-map.org/grid_data/download/75694911?include=energy
Bcl2l15	1.042	http://api.brain-map.org/grid_data/download/69672958?include=energy
Ndufs3	1.042	http://api.brain-map.org/grid_data/download/67767465?include=energy
Sox13	1.042	http://api.brain-map.org/grid_data/download/71724551?include=energy
Nbas	1.042	http://api.brain-map.org/grid_data/download/68862114?include=energy
Pstk	1.042	http://api.brain-map.org/grid_data/download/72182972?include=energy
Smpd3	1.042	http://api.brain-map.org/grid_data/download/74822931?include=energy
Ankrd55	1.042	http://api.brain-map.org/grid_data/download/74821606?include=energy
Aurka	1.042	http://api.brain-map.org/grid_data/download/2643?include=energy
Rsrc1	1.041	http://api.brain-map.org/grid_data/download/68546101?include=energy
LOC434265	1.041	http://api.brain-map.org/grid_data/download/70787648?include=energy
Trim36	1.041	http://api.brain-map.org/grid_data/download/74657930?include=energy
Tfpi	1.041	http://api.brain-map.org/grid_data/download/69170276?include=energy
Gm591	1.041	http://api.brain-map.org/grid_data/download/70295596?include=energy
Ribc1	1.041	http://api.brain-map.org/grid_data/download/70474965?include=energy
BC026585	1.041	http://api.brain-map.org/grid_data/download/74431197?include=energy
Acbd6	1.041	http://api.brain-map.org/grid_data/download/69134169?include=energy
Mm.131059	1.041	http://api.brain-map.org/grid_data/download/71148097?include=energy
Tdrd1	1.041	http://api.brain-map.org/grid_data/download/74431321?include=energy
Phf1	1.041	http://api.brain-map.org/grid_data/download/356287?include=energy
Tas2r115	1.041	http://api.brain-map.org/grid_data/download/70302342?include=energy
2900097C17Rik	1.041	http://api.brain-map.org/grid_data/download/71764607?include=energy
Mnat1	1.041	http://api.brain-map.org/grid_data/download/71325264?include=energy
Scamp1	1.041	http://api.brain-map.org/grid_data/download/69169714?include=energy
D530049N12Rik	1.041	http://api.brain-map.org/grid_data/download/74300741?include=energy
LOC384610	1.041	http://api.brain-map.org/grid_data/download/70725447?include=energy
Ctbp1	1.041	http://api.brain-map.org/grid_data/download/69059817?include=energy
Nob1	1.041	http://api.brain-map.org/grid_data/download/69353620?include=energy
nnyRab5a	1.041	http://api.brain-map.org/grid_data/download/67978388?include=energy
Cep192	1.041	http://api.brain-map.org/grid_data/download/73497786?include=energy
Gpr158	1.04	http://api.brain-map.org/grid_data/download/73497162?include=energy
Ccdc82	1.04	http://api.brain-map.org/grid_data/download/69202478?include=energy
LOC432742	1.04	http://api.brain-map.org/grid_data/download/70611252?include=energy
Pex7	1.04	http://api.brain-map.org/grid_data/download/69783011?include=energy
Dnajc18	1.04	http://api.brain-map.org/grid_data/download/69095955?include=energy
Atp5b	1.04	http://api.brain-map.org/grid_data/download/70528264?include=energy
Slc25a33	1.04	http://api.brain-map.org/grid_data/download/70430738?include=energy
Eml2	1.04	http://api.brain-map.org/grid_data/download/69014854?include=energy
Yars2	1.04	http://api.brain-map.org/grid_data/download/75197552?include=energy
1700001C19Rik	1.04	http://api.brain-map.org/grid_data/download/68442877?include=energy
Serpinb3a	1.04	http://api.brain-map.org/grid_data/download/73818791?include=energy
Hbxip	1.04	http://api.brain-map.org/grid_data/download/69134163?include=energy
Ltbp3	1.04	http://api.brain-map.org/grid_data/download/76115733?include=energy
Acad8	1.04	http://api.brain-map.org/grid_data/download/678718?include=energy
Cel	1.04	http://api.brain-map.org/grid_data/download/68269293?include=energy
Adipor2	1.04	http://api.brain-map.org/grid_data/download/69549165?include=energy
Stk30	1.04	http://api.brain-map.org/grid_data/download/69170198?include=energy
Ccdc48	1.04	http://api.brain-map.org/grid_data/download/71495191?include=energy
Kcnj10	1.04	http://api.brain-map.org/grid_data/download/69735700?include=energy
Scd2	1.04	http://api.brain-map.org/grid_data/download/74819250?include=energy
Mtap7	1.04	http://api.brain-map.org/grid_data/download/69013278?include=energy
1700095B22Rik	1.04	http://api.brain-map.org/grid_data/download/74359544?include=energy
Dip2a	1.04	http://api.brain-map.org/grid_data/download/2147?include=energy
Snhg10	1.04	http://api.brain-map.org/grid_data/download/73512519?include=energy
Pex16	1.04	http://api.brain-map.org/grid_data/download/393220?include=energy
1600021P15Rik	1.04	http://api.brain-map.org/grid_data/download/71924290?include=energy
Gpr139	1.04	http://api.brain-map.org/grid_data/download/79490116?include=energy
Spag9	1.04	http://api.brain-map.org/grid_data/download/70302246?include=energy
Olfr1277	1.04	http://api.brain-map.org/grid_data/download/74720681?include=energy
Nsg1	1.039	http://api.brain-map.org/grid_data/download/70429327?include=energy
Gpr17	1.039	http://api.brain-map.org/grid_data/download/74641313?include=energy
Shox2	1.039	http://api.brain-map.org/grid_data/download/71325456?include=energy
Lyrm5	1.039	http://api.brain-map.org/grid_data/download/71612177?include=energy
Lonp2	1.039	http://api.brain-map.org/grid_data/download/71764547?include=energy
Svs6	1.039	http://api.brain-map.org/grid_data/download/71892382?include=energy
Dhx35	1.039	http://api.brain-map.org/grid_data/download/68795399?include=energy
Hc	1.039	http://api.brain-map.org/grid_data/download/73992912?include=energy
Sox8	1.039	http://api.brain-map.org/grid_data/download/74988736?include=energy
Ednra	1.039	http://api.brain-map.org/grid_data/download/100146092?include=energy
Spink3	1.039	http://api.brain-map.org/grid_data/download/75234121?include=energy
Pafah1b1	1.039	http://api.brain-map.org/grid_data/download/2460?include=energy

Ankrd29	1.039	http://api.brain-map.org/grid_data/download/71495765?include=energy
Fundc2	1.039	http://api.brain-map.org/grid_data/download/70523684?include=energy
Tnfrsf14	1.039	http://api.brain-map.org/grid_data/download/75990422?include=energy
Myoc	1.039	http://api.brain-map.org/grid_data/download/71234527?include=energy
Diras1	1.039	http://api.brain-map.org/grid_data/download/72103819?include=energy
Cypt1	1.039	http://api.brain-map.org/grid_data/download/74822942?include=energy
Lbr	1.038	http://api.brain-map.org/grid_data/download/71763462?include=energy
Uqcrh	1.038	http://api.brain-map.org/grid_data/download/77340502?include=energy
Gm1396	1.038	http://api.brain-map.org/grid_data/download/70300097?include=energy
1700111E14Rik	1.038	http://api.brain-map.org/grid_data/download/74359311?include=energy
Esrrb	1.038	http://api.brain-map.org/grid_data/download/77464890?include=energy
Unc45b	1.038	http://api.brain-map.org/grid_data/download/76003343?include=energy
Gad1	1.038	http://api.brain-map.org/grid_data/download/479?include=energy
Arhgdib	1.038	http://api.brain-map.org/grid_data/download/68443369?include=energy
Rps14	1.038	http://api.brain-map.org/grid_data/download/69012777?include=energy
Tor1aip2	1.038	http://api.brain-map.org/grid_data/download/81600562?include=energy
E330012B07Rik	1.038	http://api.brain-map.org/grid_data/download/74000516?include=energy
Olfr1344	1.038	http://api.brain-map.org/grid_data/download/74720735?include=energy
Pnma1	1.038	http://api.brain-map.org/grid_data/download/69816368?include=energy
Sv2c	1.038	http://api.brain-map.org/grid_data/download/74357583?include=energy
Myl6	1.038	http://api.brain-map.org/grid_data/download/68633312?include=energy
Ap1s1	1.038	http://api.brain-map.org/grid_data/download/69526707?include=energy
Clu	1.038	http://api.brain-map.org/grid_data/download/275?include=energy
Creb5	1.038	http://api.brain-map.org/grid_data/download/75990533?include=energy
Tuba1b	1.038	http://api.brain-map.org/grid_data/download/77465048?include=energy
0610005G16Rik*	1.038	http://api.brain-map.org/grid_data/download/74357755?include=energy
Trim32	1.038	http://api.brain-map.org/grid_data/download/75774651?include=energy
Tmem120a	1.038	http://api.brain-map.org/grid_data/download/71210938?include=energy
Rps3	1.038	http://api.brain-map.org/grid_data/download/68269604?include=energy
0610009I12Rik*	1.038	http://api.brain-map.org/grid_data/download/74357773?include=energy
Ralgds	1.038	http://api.brain-map.org/grid_data/download/75214940?include=energy
LOC385446	1.038	http://api.brain-map.org/grid_data/download/74736159?include=energy
Mmgt1	1.038	http://api.brain-map.org/grid_data/download/77414585?include=energy
Snurf	1.038	http://api.brain-map.org/grid_data/download/70302222?include=energy
Zcchc18	1.038	http://api.brain-map.org/grid_data/download/583555?include=energy
Fes	1.037	http://api.brain-map.org/grid_data/download/71764775?include=energy
S1pr2	1.037	http://api.brain-map.org/grid_data/download/69237215?include=energy
Zan	1.037	http://api.brain-map.org/grid_data/download/72004460?include=energy
Mrfap1	1.037	http://api.brain-map.org/grid_data/download/69446589?include=energy
Olfr1420	1.037	http://api.brain-map.org/grid_data/download/73653320?include=energy
1600029O10Rik	1.037	http://api.brain-map.org/grid_data/download/68445955?include=energy
Gm6288	1.037	http://api.brain-map.org/grid_data/download/73716776?include=energy
Dnajc27	1.037	http://api.brain-map.org/grid_data/download/68845639?include=energy
Sv2a	1.037	http://api.brain-map.org/grid_data/download/69012765?include=energy
Ttll13	1.037	http://api.brain-map.org/grid_data/download/70201504?include=energy
Rbm34	1.037	http://api.brain-map.org/grid_data/download/69626564?include=energy
Slc9a10	1.037	http://api.brain-map.org/grid_data/download/73423681?include=energy
Dlx3	1.037	http://api.brain-map.org/grid_data/download/350?include=energy
Cdca7	1.037	http://api.brain-map.org/grid_data/download/26?include=energy
Rpl37	1.037	http://api.brain-map.org/grid_data/download/70918802?include=energy
Nadsyn1	1.037	http://api.brain-map.org/grid_data/download/678703?include=energy
Nsf	1.037	http://api.brain-map.org/grid_data/download/356092?include=energy
Hoxd9	1.037	http://api.brain-map.org/grid_data/download/1623?include=energy
Rnase6	1.037	http://api.brain-map.org/grid_data/download/71832868?include=energy
Lhfp	1.037	http://api.brain-map.org/grid_data/download/73769323?include=energy
Mlf2	1.037	http://api.brain-map.org/grid_data/download/513491?include=energy
Xpo1	1.037	http://api.brain-map.org/grid_data/download/68498956?include=energy
Stmn4	1.037	http://api.brain-map.org/grid_data/download/1006?include=energy
C1ql4	1.037	http://api.brain-map.org/grid_data/download/74735271?include=energy
Eif2b3	1.037	http://api.brain-map.org/grid_data/download/69671935?include=energy
1110034B05Rik	1.037	http://api.brain-map.org/grid_data/download/71492766?include=energy
Lynx1	1.037	http://api.brain-map.org/grid_data/download/655?include=energy
St3gal4	1.037	http://api.brain-map.org/grid_data/download/69080504?include=energy
Txndc5	1.037	http://api.brain-map.org/grid_data/download/68342378?include=energy
Tpsb2	1.037	http://api.brain-map.org/grid_data/download/71489795?include=energy
Fam169b	1.037	http://api.brain-map.org/grid_data/download/74743483?include=energy
Abcb9	1.037	http://api.brain-map.org/grid_data/download/68798753?include=energy
Klc1	1.037	http://api.brain-map.org/grid_data/download/68844845?include=energy
Gtdc2	1.037	http://api.brain-map.org/grid_data/download/74658171?include=energy
Serpina4-ps1	1.036	http://api.brain-map.org/grid_data/download/74276876?include=energy
4930525F21Rik	1.036	http://api.brain-map.org/grid_data/download/74265147?include=energy
Adipor1	1.036	http://api.brain-map.org/grid_data/download/61210?include=energy
Cdk19	1.036	http://api.brain-map.org/grid_data/download/70203549?include=energy
Tmed10	1.036	http://api.brain-map.org/grid_data/download/68910987?include=energy
C4b	1.036	http://api.brain-map.org/grid_data/download/74047758?include=energy
Tuba1a	1.036	http://api.brain-map.org/grid_data/download/68844164?include=energy
9630041I06Rik	1.036	http://api.brain-map.org/grid_data/download/71147894?include=energy
1810022K09Rik	1.036	http://api.brain-map.org/grid_data/download/70609184?include=energy
Pla2r1	1.036	http://api.brain-map.org/grid_data/download/69012653?include=energy
4931428L18Rik	1.036	http://api.brain-map.org/grid_data/download/74822595?include=energy
Canx	1.036	http://api.brain-map.org/grid_data/download/69080016?include=energy
Tmem91	1.036	http://api.brain-map.org/grid_data/download/75651161?include=energy
Hsf2bp	1.036	http://api.brain-map.org/grid_data/download/77464978?include=energy
Mcf2	1.036	http://api.brain-map.org/grid_data/download/76003702?include=energy
Notum	1.036	http://api.brain-map.org/grid_data/download/73732151?include=energy
Hmgn2	1.036	http://api.brain-map.org/grid_data/download/71764565?include=energy
Bclaf1	1.036	http://api.brain-map.org/grid_data/download/68498325?include=energy
Ppp2r2d	1.036	http://api.brain-map.org/grid_data/download/70784988?include=energy
Cct2	1.036	http://api.brain-map.org/grid_data/download/356082?include=energy
Crybb3	1.036	http://api.brain-map.org/grid_data/download/77620495?include=energy
LOC384644	1.036	http://api.brain-map.org/grid_data/download/70725453?include=energy
Slc22a19	1.036	http://api.brain-map.org/grid_data/download/80342217?include=energy
Tmbim1	1.036	http://api.brain-map.org/grid_data/download/72182992?include=energy
D130023B06Rik*	1.036	http://api.brain-map.org/grid_data/download/73834343?include=energy
Edaradd	1.035	http://api.brain-map.org/grid_data/download/68744474?include=energy
Gm5563	1.035	http://api.brain-map.org/grid_data/download/72076521?include=energy
Ppie	1.035	http://api.brain-map.org/grid_data/download/69012723?include=energy
Sfxn2	1.035	http://api.brain-map.org/grid_data/download/68410339?include=energy
Dnai2	1.035	http://api.brain-map.org/grid_data/download/69626951?include=energy
Smug1	1.035	http://api.brain-map.org/grid_data/download/73992919?include=energy
Apbb1	1.035	http://api.brain-map.org/grid_data/download/2033?include=energy
Pappa2	1.035	http://api.brain-map.org/grid_data/download/276066?include=energy
Npas3	1.035	http://api.brain-map.org/grid_data/download/69816643?include=energy
Slc45a4	1.035	http://api.brain-map.org/grid_data/download/69446673?include=energy
Ebpl	1.035	http://api.brain-map.org/grid_data/download/112650122?include=energy
Kcnq4	1.035	http://api.brain-map.org/grid_data/download/73635775?include=energy
Chaf1a	1.035	http://api.brain-map.org/grid_data/download/69548891?include=energy
Chi3l4	1.035	http://api.brain-map.org/grid_data/download/71021150?include=energy
Snx6	1.035	http://api.brain-map.org/grid_data/download/69117026?include=energy
4930412F15Rik	1.035	http://api.brain-map.org/grid_data/download/72182945?include=energy
Atp1a2	1.035	http://api.brain-map.org/grid_data/download/69173256?include=energy
Batf3	1.035	http://api.brain-map.org/grid_data/download/70723337?include=energy
Slc25a4	1.035	http://api.brain-map.org/grid_data/download/68797490?include=energy
Jag1	1.035	http://api.brain-map.org/grid_data/download/69735646?include=energy
Anapc2	1.035	http://api.brain-map.org/grid_data/download/69526617?include=energy
Btc	1.035	http://api.brain-map.org/grid_data/download/75988324?include=energy
Dctn2	1.035	http://api.brain-map.org/grid_data/download/545090?include=energy
Rabepk	1.035	http://api.brain-map.org/grid_data/download/68410440?include=energy
B9d1	1.035	http://api.brain-map.org/grid_data/download/71991965?include=energy
Ube2d2a	1.035	http://api.brain-map.org/grid_data/download/73573295?include=energy
Pcdhb11	1.035	http://api.brain-map.org/grid_data/download/69782796?include=energy
Xlr4a	1.035	http://api.brain-map.org/grid_data/download/74743396?include=energy
Gtf3c6	1.034	http://api.brain-map.org/grid_data/download/69113536?include=energy
Kif5a	1.034	http://api.brain-map.org/grid_data/download/79556603?include=energy
E030025L23Rik*	1.034	http://api.brain-map.org/grid_data/download/73834505?include=energy
Fn3k	1.034	http://api.brain-map.org/grid_data/download/69261426?include=energy
Lox	1.034	http://api.brain-map.org/grid_data/download/68076968?include=energy
Lpp	1.034	http://api.brain-map.org/grid_data/download/73834451?include=energy
Speer8-ps1	1.034	http://api.brain-map.org/grid_data/download/71892125?include=energy
Gzmb	1.034	http://api.brain-map.org/grid_data/download/512751?include=energy
AB124611	1.034	http://api.brain-map.org/grid_data/download/74578242?include=energy
Olfr1145	1.034	http://api.brain-map.org/grid_data/download/73635538?include=energy
Gprasp2	1.034	http://api.brain-map.org/grid_data/download/70218896?include=energy
Nipal3	1.034	http://api.brain-map.org/grid_data/download/70436746?include=energy
Tmem126a	1.034	http://api.brain-map.org/grid_data/download/77414913?include=energy
Olfr282	1.034	http://api.brain-map.org/grid_data/download/73653362?include=energy
Nxf1	1.034	http://api.brain-map.org/grid_data/download/356089?include=energy
Tubb2b	1.034	http://api.brain-map.org/grid_data/download/69854163?include=energy
4921511H03Rik	1.034	http://api.brain-map.org/grid_data/download/69114229?include=energy
Slc35e1	1.034	http://api.brain-map.org/grid_data/download/70919788?include=energy
Skp1a	1.034	http://api.brain-map.org/grid_data/download/73573175?include=energy
Dynll2	1.034	http://api.brain-map.org/grid_data/download/564758?include=energy
Rgs7bp	1.034	http://api.brain-map.org/grid_data/download/73931629?include=energy
Ndst4	1.034	http://api.brain-map.org/grid_data/download/74511837?include=energy
Tspan3	1.034	http://api.brain-map.org/grid_data/download/71489783?include=energy
ND3	1.034	http://api.brain-map.org/grid_data/download/675?include=energy
Dnajb2	1.034	http://api.brain-map.org/grid_data/download/564753?include=energy
Phldb1	1.034	http://api.brain-map.org/grid_data/download/69169851?include=energy
Tmem2	1.034	http://api.brain-map.org/grid_data/download/1409?include=energy
Cish	1.034	http://api.brain-map.org/grid_data/download/70634310?include=energy
Ankrd2	1.033	http://api.brain-map.org/grid_data/download/69526647?include=energy
Col3a1	1.033	http://api.brain-map.org/grid_data/download/70719653?include=energy
Tmem120b	1.033	http://api.brain-map.org/grid_data/download/71764577?include=energy
Nkain1	1.033	http://api.brain-map.org/grid_data/download/71991923?include=energy
Bin1	1.033	http://api.brain-map.org/grid_data/download/69529292?include=energy
3300005D01Rik	1.033	http://api.brain-map.org/grid_data/download/71493141?include=energy
Kremen1	1.033	http://api.brain-map.org/grid_data/download/81600576?include=energy
Pcdhb3	1.033	http://api.brain-map.org/grid_data/download/69782838?include=energy
Lta4h	1.033	http://api.brain-map.org/grid_data/download/77455036?include=energy
Ccdc50	1.033	http://api.brain-map.org/grid_data/download/71832844?include=energy
Lrrc49	1.033	http://api.brain-map.org/grid_data/download/70634108?include=energy
Adrm1	1.033	http://api.brain-map.org/grid_data/download/2019?include=energy
Prkg2	1.033	http://api.brain-map.org/grid_data/download/71670688?include=energy
Thy1	1.033	http://api.brain-map.org/grid_data/download/68798025?include=energy
Aldh1a2	1.033	http://api.brain-map.org/grid_data/download/2025?include=energy
Tmem55b	1.033	http://api.brain-map.org/grid_data/download/69529095?include=energy
Fxyd3	1.033	http://api.brain-map.org/grid_data/download/73513768?include=energy
Rps8	1.033	http://api.brain-map.org/grid_data/download/69549280?include=energy
Txn1	1.033	http://api.brain-map.org/grid_data/download/73573289?include=energy
Srp72	1.033	http://api.brain-map.org/grid_data/download/69885750?include=energy
Mbtps1	1.033	http://api.brain-map.org/grid_data/download/69015164?include=energy
Bcl2l1	1.033	http://api.brain-map.org/grid_data/download/69529280?include=energy
LOC546316	1.033	http://api.brain-map.org/grid_data/download/71213755?include=energy
Erbb3	1.033	http://api.brain-map.org/grid_data/download/71496565?include=energy
Ogt	1.033	http://api.brain-map.org/grid_data/download/758?include=energy
Bpgm	1.033	http://api.brain-map.org/grid_data/download/77414641?include=energy
Ccl7	1.033	http://api.brain-map.org/grid_data/download/71662902?include=energy
Wdr35	1.033	http://api.brain-map.org/grid_data/download/70565425?include=energy
F430209L04Rik*	1.033	http://api.brain-map.org/grid_data/download/73767296?include=energy
Zpld1	1.033	http://api.brain-map.org/grid_data/download/71020981?include=energy
Snx1	1.032	http://api.brain-map.org/grid_data/download/69885606?include=energy
Csmd3	1.032	http://api.brain-map.org/grid_data/download/70611425?include=energy
Defb45	1.032	http://api.brain-map.org/grid_data/download/71991977?include=energy
Rln3	1.032	http://api.brain-map.org/grid_data/download/73929581?include=energy
Kndc1	1.032	http://api.brain-map.org/grid_data/download/71213183?include=energy
Smad2	1.032	http://api.brain-map.org/grid_data/download/70439147?include=energy
Raver1	1.032	http://api.brain-map.org/grid_data/download/72081771?include=energy
Gm5751	1.032	http://api.brain-map.org/grid_data/download/74736312?include=energy
4930524O08Rik	1.032	http://api.brain-map.org/grid_data/download/71891868?include=energy
Rps3a	1.032	http://api.brain-map.org/grid_data/download/68191186?include=energy
Tmsb10	1.032	http://api.brain-map.org/grid_data/download/73521810?include=energy
Azi2	1.032	http://api.brain-map.org/grid_data/download/81599640?include=energy
Clstn3	1.032	http://api.brain-map.org/grid_data/download/69013572?include=energy
Adamts15	1.032	http://api.brain-map.org/grid_data/download/77332098?include=energy
C1qb	1.032	http://api.brain-map.org/grid_data/download/71487058?include=energy
Foxd2	1.032	http://api.brain-map.org/grid_data/download/81790678?include=energy
Hmga1	1.032	http://api.brain-map.org/grid_data/download/2295?include=energy
Cops7a	1.032	http://api.brain-map.org/grid_data/download/512762?include=energy
Zscan22	1.032	http://api.brain-map.org/grid_data/download/247974?include=energy
Bcl10	1.032	http://api.brain-map.org/grid_data/download/75861854?include=energy
Atp5d	1.032	http://api.brain-map.org/grid_data/download/81600110?include=energy
N28178	1.032	http://api.brain-map.org/grid_data/download/71234529?include=energy
Phf12	1.032	http://api.brain-map.org/grid_data/download/69170180?include=energy
Npbwr1	1.032	http://api.brain-map.org/grid_data/download/70565993?include=energy
Fbln1	1.032	http://api.brain-map.org/grid_data/download/2205?include=energy
Olfr3	1.032	http://api.brain-map.org/grid_data/download/73653380?include=energy
9130204K15Rik	1.032	http://api.brain-map.org/grid_data/download/74514007?include=energy
Aqp3	1.032	http://api.brain-map.org/grid_data/download/293692?include=energy
LOC433163	1.032	http://api.brain-map.org/grid_data/download/70722952?include=energy
Lemd2	1.032	http://api.brain-map.org/grid_data/download/69173262?include=energy
Scrt1	1.031	http://api.brain-map.org/grid_data/download/73636035?include=energy
Prkcdbp	1.031	http://api.brain-map.org/grid_data/download/69080013?include=energy
Plcl2	1.031	http://api.brain-map.org/grid_data/download/68445889?include=energy
Macc1	1.031	http://api.brain-map.org/grid_data/download/71808989?include=energy
Slc25a5	1.031	http://api.brain-map.org/grid_data/download/2591?include=energy
Asl	1.031	http://api.brain-map.org/grid_data/download/74990535?include=energy
Rdh1	1.031	http://api.brain-map.org/grid_data/download/70301007?include=energy
Gm101	1.031	http://api.brain-map.org/grid_data/download/71021173?include=energy
4930448K20Rik	1.031	http://api.brain-map.org/grid_data/download/70218151?include=energy
Arl5c	1.031	http://api.brain-map.org/grid_data/download/77620439?include=energy
Dlk1	1.031	http://api.brain-map.org/grid_data/download/71587885?include=energy
Brms1l	1.031	http://api.brain-map.org/grid_data/download/68744671?include=energy
Serpinb6b	1.031	http://api.brain-map.org/grid_data/download/69133611?include=energy
Ppp1ca	1.031	http://api.brain-map.org/grid_data/download/69015542?include=energy
Tmem19	1.031	http://api.brain-map.org/grid_data/download/69059976?include=energy
Syt11	1.031	http://api.brain-map.org/grid_data/download/2649?include=energy
Prdm2	1.031	http://api.brain-map.org/grid_data/download/73906125?include=energy
Olfr123	1.031	http://api.brain-map.org/grid_data/download/74720639?include=energy
Eef2	1.031	http://api.brain-map.org/grid_data/download/67936204?include=energy
Anxa5	1.031	http://api.brain-map.org/grid_data/download/79488933?include=energy
Isyna1	1.031	http://api.brain-map.org/grid_data/download/75145997?include=energy
Troap	1.031	http://api.brain-map.org/grid_data/download/73712720?include=energy
Ankrd34b	1.03	http://api.brain-map.org/grid_data/download/72128747?include=energy
Pja2	1.03	http://api.brain-map.org/grid_data/download/583577?include=energy
Msx1	1.03	http://api.brain-map.org/grid_data/download/70918934?include=energy
Cnga1	1.03	http://api.brain-map.org/grid_data/download/71380718?include=energy
EG433662	1.03	http://api.brain-map.org/grid_data/download/70786640?include=energy
Slc16a9	1.03	http://api.brain-map.org/grid_data/download/69014931?include=energy
Insrr	1.03	http://api.brain-map.org/grid_data/download/71670480?include=energy
Fam184b	1.03	http://api.brain-map.org/grid_data/download/73636151?include=energy
Rpl13a	1.03	http://api.brain-map.org/grid_data/download/71764512?include=energy
LOC432503	1.03	http://api.brain-map.org/grid_data/download/70608592?include=energy
Serpind1	1.03	http://api.brain-map.org/grid_data/download/71656108?include=energy
Klc3	1.03	http://api.brain-map.org/grid_data/download/583557?include=energy
Kifap3	1.03	http://api.brain-map.org/grid_data/download/67765709?include=energy
Robo1	1.03	http://api.brain-map.org/grid_data/download/73521820?include=energy
Pofut2	1.03	http://api.brain-map.org/grid_data/download/147?include=energy
AU022252	1.03	http://api.brain-map.org/grid_data/download/71210253?include=energy
Fen1	1.03	http://api.brain-map.org/grid_data/download/77620497?include=energy
A430002G09Rik*	1.03	http://api.brain-map.org/grid_data/download/73808552?include=energy
Pnma5	1.03	http://api.brain-map.org/grid_data/download/77620283?include=energy
Abcg3	1.03	http://api.brain-map.org/grid_data/download/71808612?include=energy
Igsf11	1.03	http://api.brain-map.org/grid_data/download/72103820?include=energy
Snrpa1	1.03	http://api.brain-map.org/grid_data/download/512761?include=energy
C030019I05Rik	1.029	http://api.brain-map.org/grid_data/download/69530732?include=energy
Timm8a1	1.029	http://api.brain-map.org/grid_data/download/69079985?include=energy
Gm5069	1.029	http://api.brain-map.org/grid_data/download/69014842?include=energy
Ivns1abp	1.029	http://api.brain-map.org/grid_data/download/70813898?include=energy
Mmachc	1.029	http://api.brain-map.org/grid_data/download/70201588?include=energy
0610006F12Rik*	1.029	http://api.brain-map.org/grid_data/download/74357761?include=energy
Polg2	1.029	http://api.brain-map.org/grid_data/download/71920497?include=energy
Nadkd1	1.029	http://api.brain-map.org/grid_data/download/69352938?include=energy
Calca	1.029	http://api.brain-map.org/grid_data/download/79587715?include=energy
Rhox6	1.029	http://api.brain-map.org/grid_data/download/77464894?include=energy
D130079A08Rik	1.029	http://api.brain-map.org/grid_data/download/70238615?include=energy
Fam102a	1.029	http://api.brain-map.org/grid_data/download/75826580?include=energy
Abca14	1.029	http://api.brain-map.org/grid_data/download/78534524?include=energy

Bcan	1.029	http://api.brain-map.org/grid_data/download/154?include=energy
1810030J14Rik	1.029	http://api.brain-map.org/grid_data/download/76098398?include=energy
Zfp53	1.029	http://api.brain-map.org/grid_data/download/68924709?include=energy
Aim1l	1.029	http://api.brain-map.org/grid_data/download/71574377?include=energy
Dhrs13	1.029	http://api.brain-map.org/grid_data/download/69202914?include=energy
Fez1	1.029	http://api.brain-map.org/grid_data/download/68632051?include=energy
Krt2	1.029	http://api.brain-map.org/grid_data/download/71920416?include=energy
D19Ertd737e	1.029	http://api.brain-map.org/grid_data/download/70523780?include=energy
Oaz1	1.029	http://api.brain-map.org/grid_data/download/69782533?include=energy
LOC215538	1.029	http://api.brain-map.org/grid_data/download/70565951?include=energy
Llgl1	1.029	http://api.brain-map.org/grid_data/download/645?include=energy
Hist1h1t	1.029	http://api.brain-map.org/grid_data/download/70724828?include=energy
Il13ra1	1.029	http://api.brain-map.org/grid_data/download/74819256?include=energy
Rangap1	1.029	http://api.brain-map.org/grid_data/download/2519?include=energy
Trim39	1.029	http://api.brain-map.org/grid_data/download/68631875?include=energy
Pih1d2	1.029	http://api.brain-map.org/grid_data/download/68920707?include=energy
Slc5a12	1.029	http://api.brain-map.org/grid_data/download/70230831?include=energy
Btbd2	1.029	http://api.brain-map.org/grid_data/download/69169469?include=energy
Rnf208	1.029	http://api.brain-map.org/grid_data/download/68545794?include=energy
Atp1b1	1.029	http://api.brain-map.org/grid_data/download/70919203?include=energy
Sepw1	1.029	http://api.brain-map.org/grid_data/download/69873540?include=energy
Prss34	1.029	http://api.brain-map.org/grid_data/download/70300935?include=energy
Als2cl	1.029	http://api.brain-map.org/grid_data/download/71281098?include=energy
Klrd1	1.029	http://api.brain-map.org/grid_data/download/69735897?include=energy
Nxn	1.029	http://api.brain-map.org/grid_data/download/72007747?include=energy
Rabgap1l	1.029	http://api.brain-map.org/grid_data/download/69134220?include=energy
Abl1	1.029	http://api.brain-map.org/grid_data/download/68844474?include=energy
Cst3	1.028	http://api.brain-map.org/grid_data/download/69120555?include=energy
Cacna2d4	1.028	http://api.brain-map.org/grid_data/download/71808777?include=energy
Sat1	1.028	http://api.brain-map.org/grid_data/download/69873409?include=energy
Csnk1g3	1.028	http://api.brain-map.org/grid_data/download/71723900?include=energy
Wwc2	1.028	http://api.brain-map.org/grid_data/download/71735080?include=energy
Eif3i	1.028	http://api.brain-map.org/grid_data/download/68637694?include=energy
Slc29a3	1.028	http://api.brain-map.org/grid_data/download/76081033?include=energy
Slc25a17	1.028	http://api.brain-map.org/grid_data/download/69059768?include=energy
Gm15217	1.028	http://api.brain-map.org/grid_data/download/70595990?include=energy
Cyp2c50	1.028	http://api.brain-map.org/grid_data/download/77620489?include=energy
Acsl1	1.028	http://api.brain-map.org/grid_data/download/100144473?include=energy
Acsl3	1.028	http://api.brain-map.org/grid_data/download/75042266?include=energy
Scarb2	1.028	http://api.brain-map.org/grid_data/download/72081482?include=energy
Myo5a	1.028	http://api.brain-map.org/grid_data/download/70918838?include=energy
Kank4	1.028	http://api.brain-map.org/grid_data/download/76085741?include=energy
Chat	1.028	http://api.brain-map.org/grid_data/download/252?include=energy
Aff3	1.028	http://api.brain-map.org/grid_data/download/71656693?include=energy
Wdr5b	1.028	http://api.brain-map.org/grid_data/download/68744146?include=energy
Olfr1387	1.028	http://api.brain-map.org/grid_data/download/73635616?include=energy
Gje1	1.028	http://api.brain-map.org/grid_data/download/71892460?include=energy
Krt33b	1.028	http://api.brain-map.org/grid_data/download/74735355?include=energy
Samd14	1.027	http://api.brain-map.org/grid_data/download/73930839?include=energy
Rps6kl1	1.027	http://api.brain-map.org/grid_data/download/68148754?include=energy
Slc1a7	1.027	http://api.brain-map.org/grid_data/download/71763589?include=energy
Sfxn1	1.027	http://api.brain-map.org/grid_data/download/100146066?include=energy
Unc5cl	1.027	http://api.brain-map.org/grid_data/download/68498723?include=energy
Sh3gl3	1.027	http://api.brain-map.org/grid_data/download/79632219?include=energy
Timp2	1.027	http://api.brain-map.org/grid_data/download/75988524?include=energy
Rexo4	1.027	http://api.brain-map.org/grid_data/download/69672916?include=energy
Nrip3	1.027	http://api.brain-map.org/grid_data/download/73520999?include=energy
Capza1	1.027	http://api.brain-map.org/grid_data/download/68342264?include=energy
Rab3il1	1.027	http://api.brain-map.org/grid_data/download/68341722?include=energy
A930001N09Rik	1.027	http://api.brain-map.org/grid_data/download/69514404?include=energy
Sh3rf2	1.027	http://api.brain-map.org/grid_data/download/71670729?include=energy
Rad51l1	1.027	http://api.brain-map.org/grid_data/download/69028593?include=energy
Fgfr1op	1.027	http://api.brain-map.org/grid_data/download/69672450?include=energy
Vat1	1.027	http://api.brain-map.org/grid_data/download/74357572?include=energy
Dfna5	1.027	http://api.brain-map.org/grid_data/download/77904877?include=energy
Myh11	1.027	http://api.brain-map.org/grid_data/download/68562932?include=energy
C230052I12Rik	1.027	http://api.brain-map.org/grid_data/download/69530905?include=energy
C530008M17Rik	1.027	http://api.brain-map.org/grid_data/download/75080646?include=energy
Rap2c	1.027	http://api.brain-map.org/grid_data/download/69170108?include=energy
Lbh	1.027	http://api.brain-map.org/grid_data/download/69015422?include=energy
Clec5a	1.027	http://api.brain-map.org/grid_data/download/72471812?include=energy
Lamtor2	1.027	http://api.brain-map.org/grid_data/download/70546111?include=energy
Rps4x	1.026	http://api.brain-map.org/grid_data/download/69873295?include=energy
Cmpk2	1.026	http://api.brain-map.org/grid_data/download/68844803?include=energy
1700069B07Rik	1.026	http://api.brain-map.org/grid_data/download/74357929?include=energy
3000002C10Rik	1.026	http://api.brain-map.org/grid_data/download/69407397?include=energy
Rdh5	1.026	http://api.brain-map.org/grid_data/download/75042255?include=energy
BC055111	1.026	http://api.brain-map.org/grid_data/download/69114153?include=energy
Hcls1	1.026	http://api.brain-map.org/grid_data/download/68443122?include=energy
Pcdh12	1.026	http://api.brain-map.org/grid_data/download/69782778?include=energy
LOC237229	1.026	http://api.brain-map.org/grid_data/download/70566187?include=energy
Nme5	1.026	http://api.brain-map.org/grid_data/download/75850264?include=energy
Sos1	1.026	http://api.brain-map.org/grid_data/download/69885660?include=energy
Psapl1	1.026	http://api.brain-map.org/grid_data/download/71493008?include=energy
Kcnn3	1.026	http://api.brain-map.org/grid_data/download/69258071?include=energy
Mns1	1.026	http://api.brain-map.org/grid_data/download/68745522?include=energy
Vamp1	1.026	http://api.brain-map.org/grid_data/download/75651233?include=energy
Osbpl1a	1.026	http://api.brain-map.org/grid_data/download/71763540?include=energy
0610008A24Rik*	1.026	http://api.brain-map.org/grid_data/download/74357767?include=energy
Amigo2	1.026	http://api.brain-map.org/grid_data/download/71250310?include=energy
Sorbs3	1.026	http://api.brain-map.org/grid_data/download/77620501?include=energy
4921528I01Rik	1.026	http://api.brain-map.org/grid_data/download/71808784?include=energy
Cep110	1.026	http://api.brain-map.org/grid_data/download/71213228?include=energy
1110003E01Rik	1.026	http://api.brain-map.org/grid_data/download/69352852?include=energy
Ppa1	1.026	http://api.brain-map.org/grid_data/download/74635292?include=energy
Zdhhc20	1.026	http://api.brain-map.org/grid_data/download/68341902?include=energy
Rab11fip3	1.026	http://api.brain-map.org/grid_data/download/68410554?include=energy
Eid3	1.026	http://api.brain-map.org/grid_data/download/70201385?include=energy
Sdr39u1	1.026	http://api.brain-map.org/grid_data/download/100144110?include=energy
Ido2	1.026	http://api.brain-map.org/grid_data/download/67815952?include=energy
mCG1030251	1.026	http://api.brain-map.org/grid_data/download/71022846?include=energy
Id3	1.026	http://api.brain-map.org/grid_data/download/74724763?include=energy
Erlin1	1.026	http://api.brain-map.org/grid_data/download/68302368?include=energy
Riok1	1.026	http://api.brain-map.org/grid_data/download/68076544?include=energy
Pip4k2c	1.026	http://api.brain-map.org/grid_data/download/799?include=energy
Idi2	1.026	http://api.brain-map.org/grid_data/download/72182938?include=energy
3110040N11Rik	1.026	http://api.brain-map.org/grid_data/download/77910816?include=energy
Abhd14b	1.025	http://api.brain-map.org/grid_data/download/68637641?include=energy
Nfkbia	1.025	http://api.brain-map.org/grid_data/download/69782222?include=energy
Rpl7	1.025	http://api.brain-map.org/grid_data/download/68410596?include=energy
Zfp68	1.025	http://api.brain-map.org/grid_data/download/68797493?include=energy
Samt4	1.025	http://api.brain-map.org/grid_data/download/71891916?include=energy
Tifa	1.025	http://api.brain-map.org/grid_data/download/71210932?include=energy
Cab39l	1.025	http://api.brain-map.org/grid_data/download/77371789?include=energy
Dbr1	1.025	http://api.brain-map.org/grid_data/download/2141?include=energy
Fam76b	1.025	http://api.brain-map.org/grid_data/download/68797792?include=energy
Cd48	1.025	http://api.brain-map.org/grid_data/download/69174340?include=energy
Echs1	1.025	http://api.brain-map.org/grid_data/download/69671869?include=energy
Scg2	1.025	http://api.brain-map.org/grid_data/download/934?include=energy
Muc20	1.025	http://api.brain-map.org/grid_data/download/70546233?include=energy
Arhgap12	1.025	http://api.brain-map.org/grid_data/download/71836846?include=energy
Gm12866	1.025	http://api.brain-map.org/grid_data/download/70786749?include=energy
Slc6a9	1.025	http://api.brain-map.org/grid_data/download/1420?include=energy
Ctsc	1.025	http://api.brain-map.org/grid_data/download/69608220?include=energy
Prmt2	1.025	http://api.brain-map.org/grid_data/download/577?include=energy
1810034E14Rik	1.025	http://api.brain-map.org/grid_data/download/73767380?include=energy
Krt32	1.025	http://api.brain-map.org/grid_data/download/70724888?include=energy
Krtdap	1.025	http://api.brain-map.org/grid_data/download/74357779?include=energy
Pppde2	1.025	http://api.brain-map.org/grid_data/download/68862048?include=energy
Rgs6	1.025	http://api.brain-map.org/grid_data/download/74272893?include=energy
Urod	1.025	http://api.brain-map.org/grid_data/download/74581008?include=energy
Rgs7	1.025	http://api.brain-map.org/grid_data/download/69113608?include=energy
Gucy1a3	1.025	http://api.brain-map.org/grid_data/download/77866848?include=energy
Mbnl2	1.025	http://api.brain-map.org/grid_data/download/71022744?include=energy
Clec3a	1.025	http://api.brain-map.org/grid_data/download/74735295?include=energy
Sptlc3	1.025	http://api.brain-map.org/grid_data/download/70231857?include=energy
Deb1	1.025	http://api.brain-map.org/grid_data/download/68545450?include=energy
Crybg3	1.025	http://api.brain-map.org/grid_data/download/69529131?include=energy
Gm497	1.025	http://api.brain-map.org/grid_data/download/74359664?include=energy
Cox5b	1.025	http://api.brain-map.org/grid_data/download/74743435?include=energy
Prelid1	1.024	http://api.brain-map.org/grid_data/download/81600090?include=energy
Rps6	1.024	http://api.brain-map.org/grid_data/download/69873301?include=energy
Aco2	1.024	http://api.brain-map.org/grid_data/download/67978674?include=energy
Nipa1	1.024	http://api.brain-map.org/grid_data/download/70546275?include=energy
Magee2	1.024	http://api.brain-map.org/grid_data/download/69736424?include=energy
Mapk1	1.024	http://api.brain-map.org/grid_data/download/68744976?include=energy
LOC435346	1.024	http://api.brain-map.org/grid_data/download/71022475?include=energy
Minpp1	1.024	http://api.brain-map.org/grid_data/download/68193046?include=energy
Tmem48	1.024	http://api.brain-map.org/grid_data/download/68446063?include=energy
Trim63	1.024	http://api.brain-map.org/grid_data/download/70786788?include=energy
Phax	1.024	http://api.brain-map.org/grid_data/download/69113578?include=energy
Col4a3bp	1.024	http://api.brain-map.org/grid_data/download/68193060?include=energy
Hrsp12	1.024	http://api.brain-map.org/grid_data/download/76003665?include=energy
Prr9	1.024	http://api.brain-map.org/grid_data/download/70228310?include=energy
Sarnp	1.024	http://api.brain-map.org/grid_data/download/68744618?include=energy
Olfr124	1.024	http://api.brain-map.org/grid_data/download/74720657?include=energy
Tgoln2	1.024	http://api.brain-map.org/grid_data/download/69980340?include=energy
1110014N23Rik	1.024	http://api.brain-map.org/grid_data/download/71147765?include=energy
Cyp2j9	1.024	http://api.brain-map.org/grid_data/download/69608372?include=energy
BC035947	1.024	http://api.brain-map.org/grid_data/download/69173274?include=energy
Ppp1r42	1.024	http://api.brain-map.org/grid_data/download/68498735?include=energy
Prepl	1.024	http://api.brain-map.org/grid_data/download/70431259?include=energy
4930415O20Rik	1.024	http://api.brain-map.org/grid_data/download/69114174?include=energy
Ptma	1.024	http://api.brain-map.org/grid_data/download/69816721?include=energy
Slc12a4	1.024	http://api.brain-map.org/grid_data/download/69873797?include=energy
Gm5134	1.024	http://api.brain-map.org/grid_data/download/71281825?include=energy
4921508D12Rik	1.024	http://api.brain-map.org/grid_data/download/71891587?include=energy
Stk25	1.024	http://api.brain-map.org/grid_data/download/68667823?include=energy
Scml1	1.024	http://api.brain-map.org/grid_data/download/70565879?include=energy
Adamtsl4	1.024	http://api.brain-map.org/grid_data/download/69838742?include=energy
Ugp2	1.024	http://api.brain-map.org/grid_data/download/2705?include=energy
Stau2	1.024	http://api.brain-map.org/grid_data/download/248566?include=energy
1600002D24Rik	1.024	http://api.brain-map.org/grid_data/download/74581277?include=energy
Erp44	1.024	http://api.brain-map.org/grid_data/download/68342015?include=energy
Nfx1	1.024	http://api.brain-map.org/grid_data/download/68667443?include=energy
Rhcg	1.024	http://api.brain-map.org/grid_data/download/78634041?include=energy
1700020N15Rik	1.024	http://api.brain-map.org/grid_data/download/76081051?include=energy
Tbr1	1.024	http://api.brain-map.org/grid_data/download/1041?include=energy
Pigy	1.024	http://api.brain-map.org/grid_data/download/276271?include=energy
Sytl1	1.024	http://api.brain-map.org/grid_data/download/71764625?include=energy
Slc15a1	1.024	http://api.brain-map.org/grid_data/download/81600570?include=energy
Eci3	1.024	http://api.brain-map.org/grid_data/download/68666625?include=energy
Six3os1	1.024	http://api.brain-map.org/grid_data/download/75041431?include=energy
Cstb	1.024	http://api.brain-map.org/grid_data/download/1343?include=energy
LOC432826	1.024	http://api.brain-map.org/grid_data/download/72076653?include=energy
Tmed1	1.023	http://api.brain-map.org/grid_data/download/79544818?include=energy
Abcc2	1.023	http://api.brain-map.org/grid_data/download/69514600?include=energy
Mdga1	1.023	http://api.brain-map.org/grid_data/download/69752149?include=energy
Gnat2	1.023	http://api.brain-map.org/grid_data/download/79360255?include=energy
4930443G12Rik	1.023	http://api.brain-map.org/grid_data/download/72076647?include=energy
Fbxw10	1.023	http://api.brain-map.org/grid_data/download/71232681?include=energy
Srgap2	1.023	http://api.brain-map.org/grid_data/download/69672533?include=energy
Lmo3	1.023	http://api.brain-map.org/grid_data/download/73616037?include=energy
Cpsf2	1.023	http://api.brain-map.org/grid_data/download/71610841?include=energy
Gabarapl2	1.023	http://api.brain-map.org/grid_data/download/68637778?include=energy
Alad	1.023	http://api.brain-map.org/grid_data/download/75826639?include=energy
Lmod3	1.023	http://api.brain-map.org/grid_data/download/70295512?include=energy
Klc2	1.023	http://api.brain-map.org/grid_data/download/618?include=energy
Xkr6	1.023	http://api.brain-map.org/grid_data/download/70542954?include=energy
AW822252	1.023	http://api.brain-map.org/grid_data/download/71808588?include=energy
Rnf113a2	1.023	http://api.brain-map.org/grid_data/download/69202394?include=energy
Ndrg2	1.023	http://api.brain-map.org/grid_data/download/1237?include=energy
Mospd2	1.023	http://api.brain-map.org/grid_data/download/70539191?include=energy
Kcnj13	1.023	http://api.brain-map.org/grid_data/download/100144419?include=energy
Zfp566	1.023	http://api.brain-map.org/grid_data/download/71213286?include=energy
AI854517	1.023	http://api.brain-map.org/grid_data/download/70230867?include=energy
Pdgfrb	1.023	http://api.brain-map.org/grid_data/download/77278981?include=energy
1700014N06Rik	1.023	http://api.brain-map.org/grid_data/download/69816404?include=energy
Pldn	1.023	http://api.brain-map.org/grid_data/download/69169457?include=energy
LOC432451	1.023	http://api.brain-map.org/grid_data/download/74578284?include=energy
Eml1	1.023	http://api.brain-map.org/grid_data/download/73992948?include=energy
Ppia	1.023	http://api.brain-map.org/grid_data/download/69817199?include=energy
B830004H01Rik	1.023	http://api.brain-map.org/grid_data/download/69527974?include=energy
Casd1	1.023	http://api.brain-map.org/grid_data/download/77620473?include=energy
Cdh8	1.023	http://api.brain-map.org/grid_data/download/79568026?include=energy
Tlr9	1.023	http://api.brain-map.org/grid_data/download/75990421?include=energy
Wdr73	1.023	http://api.brain-map.org/grid_data/download/71212205?include=energy
Begain	1.023	http://api.brain-map.org/grid_data/download/69529340?include=energy
Rnf14	1.023	http://api.brain-map.org/grid_data/download/69013560?include=energy
Vsig10l	1.023	http://api.brain-map.org/grid_data/download/71492970?include=energy
4930533I22Rik	1.022	http://api.brain-map.org/grid_data/download/71764910?include=energy
Susd2	1.022	http://api.brain-map.org/grid_data/download/75146000?include=energy
Sirt2	1.022	http://api.brain-map.org/grid_data/download/75651224?include=energy
Mad2l1	1.022	http://api.brain-map.org/grid_data/download/74821701?include=energy
Rpl14	1.022	http://api.brain-map.org/grid_data/download/70946135?include=energy
Kcnd3	1.022	http://api.brain-map.org/grid_data/download/75147766?include=energy
Smad7	1.022	http://api.brain-map.org/grid_data/download/69290501?include=energy
B3gnt9-ps	1.022	http://api.brain-map.org/grid_data/download/69531109?include=energy
Prss27	1.022	http://api.brain-map.org/grid_data/download/71281313?include=energy
Rnd3	1.022	http://api.brain-map.org/grid_data/download/71724557?include=energy
Rnf111	1.022	http://api.brain-map.org/grid_data/download/75931453?include=energy
Arhgef2	1.022	http://api.brain-map.org/grid_data/download/2043?include=energy
Rps6ka4	1.022	http://api.brain-map.org/grid_data/download/75826620?include=energy
Qrich2	1.022	http://api.brain-map.org/grid_data/download/71992001?include=energy
1700080E11Rik	1.022	http://api.brain-map.org/grid_data/download/69116423?include=energy
9330182L06Rik	1.022	http://api.brain-map.org/grid_data/download/75081005?include=energy
9530091C08Rik	1.022	http://api.brain-map.org/grid_data/download/71249747?include=energy
Parp4	1.022	http://api.brain-map.org/grid_data/download/72076587?include=energy
Slc9a7	1.022	http://api.brain-map.org/grid_data/download/77278977?include=energy
Slc7a11	1.022	http://api.brain-map.org/grid_data/download/69874048?include=energy
Lrrc1	1.022	http://api.brain-map.org/grid_data/download/74581426?include=energy
Xlr3c	1.022	http://api.brain-map.org/grid_data/download/79591575?include=energy
2310016M24Rik	1.022	http://api.brain-map.org/grid_data/download/69202388?include=energy
Fgd6	1.022	http://api.brain-map.org/grid_data/download/67792194?include=energy
Rnase9	1.022	http://api.brain-map.org/grid_data/download/75931569?include=energy
2410022L05Rik	1.022	http://api.brain-map.org/grid_data/download/74357594?include=energy
Gbp9	1.022	http://api.brain-map.org/grid_data/download/69101786?include=energy
Aes	1.022	http://api.brain-map.org/grid_data/download/69524404?include=energy
D630013G24Rik	1.022	http://api.brain-map.org/grid_data/download/74510122?include=energy
Cyfip2	1.022	http://api.brain-map.org/grid_data/download/74357791?include=energy
LOC228862	1.022	http://api.brain-map.org/grid_data/download/74635779?include=energy
Musk	1.022	http://api.brain-map.org/grid_data/download/71234525?include=energy
Aplp2	1.021	http://api.brain-map.org/grid_data/download/68799083?include=energy
4933402J10Rik	1.021	http://api.brain-map.org/grid_data/download/74580733?include=energy
Wdr86	1.021	http://api.brain-map.org/grid_data/download/73617564?include=energy
Pitpna	1.021	http://api.brain-map.org/grid_data/download/68844295?include=energy
1110059E24Rik	1.021	http://api.brain-map.org/grid_data/download/71492778?include=energy
AI852640	1.021	http://api.brain-map.org/grid_data/download/74958645?include=energy
Rab11b	1.021	http://api.brain-map.org/grid_data/download/69816739?include=energy
5033417F24Rik	1.021	http://api.brain-map.org/grid_data/download/71892262?include=energy
March5	1.021	http://api.brain-map.org/grid_data/download/68743883?include=energy
Slc5a3	1.021	http://api.brain-map.org/grid_data/download/976?include=energy
Utp11l	1.021	http://api.brain-map.org/grid_data/download/69117008?include=energy
9430053O09Rik	1.021	http://api.brain-map.org/grid_data/download/74000612?include=energy
Eprs	1.021	http://api.brain-map.org/grid_data/download/69672114?include=energy
Fus	1.021	http://api.brain-map.org/grid_data/download/68301512?include=energy
Asb1	1.021	http://api.brain-map.org/grid_data/download/71210502?include=energy

Ube2cbp	1.021	http://api.brain-map.org/grid_data/download/69202806?include=energy
Rassf9	1.021	http://api.brain-map.org/grid_data/download/68744468?include=energy
Flywch1	1.021	http://api.brain-map.org/grid_data/download/385773?include=energy
2010001M06Rik	1.021	http://api.brain-map.org/grid_data/download/74635198?include=energy
Snx27	1.021	http://api.brain-map.org/grid_data/download/70812954?include=energy
LOC381948	1.021	http://api.brain-map.org/grid_data/download/70596149?include=energy
Ndufab1	1.021	http://api.brain-map.org/grid_data/download/69174341?include=energy
Ppp1r2	1.021	http://api.brain-map.org/grid_data/download/73521813?include=energy
Igfbpl1	1.021	http://api.brain-map.org/grid_data/download/1974?include=energy
Asna1	1.021	http://api.brain-map.org/grid_data/download/67767438?include=energy
Eif3h	1.021	http://api.brain-map.org/grid_data/download/67765724?include=energy
Hapln4	1.021	http://api.brain-map.org/grid_data/download/71717078?include=energy
Otub1	1.021	http://api.brain-map.org/grid_data/download/68080680?include=energy
Paip2	1.021	http://api.brain-map.org/grid_data/download/357095?include=energy
Cfb	1.02	http://api.brain-map.org/grid_data/download/323167?include=energy
Pcdh11x	1.02	http://api.brain-map.org/grid_data/download/73521002?include=energy
Esrrg	1.02	http://api.brain-map.org/grid_data/download/73616033?include=energy
Ppp1r7	1.02	http://api.brain-map.org/grid_data/download/832?include=energy
Slc2a2	1.02	http://api.brain-map.org/grid_data/download/69015679?include=energy
Cybb	1.02	http://api.brain-map.org/grid_data/download/71380989?include=energy
Itga9	1.02	http://api.brain-map.org/grid_data/download/70278093?include=energy
Pls1	1.02	http://api.brain-map.org/grid_data/download/71495698?include=energy
1700049L16Rik	1.02	http://api.brain-map.org/grid_data/download/75851040?include=energy
Aldoa	1.02	http://api.brain-map.org/grid_data/download/77620804?include=energy
Rpl31-ps4	1.02	http://api.brain-map.org/grid_data/download/74736300?include=energy
Lipa	1.02	http://api.brain-map.org/grid_data/download/72129290?include=energy
Spcs1	1.02	http://api.brain-map.org/grid_data/download/69262338?include=energy
Cbx7	1.02	http://api.brain-map.org/grid_data/download/68446081?include=energy
Fabp4	1.02	http://api.brain-map.org/grid_data/download/74821969?include=energy
Tubb3	1.02	http://api.brain-map.org/grid_data/download/68196914?include=energy
Spns1	1.02	http://api.brain-map.org/grid_data/download/532670?include=energy
A230028O05Rik	1.02	http://api.brain-map.org/grid_data/download/72283534?include=energy
Hmmr	1.02	http://api.brain-map.org/grid_data/download/79632217?include=energy
Slc46a2	1.02	http://api.brain-map.org/grid_data/download/70302836?include=energy
4930519P11Rik	1.02	http://api.brain-map.org/grid_data/download/73870674?include=energy
Oog4	1.02	http://api.brain-map.org/grid_data/download/73818827?include=energy
Snrpn	1.02	http://api.brain-map.org/grid_data/download/68563043?include=energy
Ren2	1.02	http://api.brain-map.org/grid_data/download/79568057?include=energy
Ywhab	1.02	http://api.brain-map.org/grid_data/download/2737?include=energy
Haus7	1.02	http://api.brain-map.org/grid_data/download/68157414?include=energy
Crct1	1.02	http://api.brain-map.org/grid_data/download/68798346?include=energy
Zc3hav1	1.02	http://api.brain-map.org/grid_data/download/73767418?include=energy
Acat3	1.02	http://api.brain-map.org/grid_data/download/69262321?include=energy
Rpl7a	1.02	http://api.brain-map.org/grid_data/download/81600152?include=energy
B230208H17Rik	1.02	http://api.brain-map.org/grid_data/download/71211353?include=energy
Dtx3	1.02	http://api.brain-map.org/grid_data/download/69671636?include=energy
0610005I03Rik*	1.02	http://api.brain-map.org/grid_data/download/74357749?include=energy
Dnm1l	1.02	http://api.brain-map.org/grid_data/download/77465026?include=energy
A330089M16Rik*	1.02	http://api.brain-map.org/grid_data/download/73808546?include=energy
Adap2	1.019	http://api.brain-map.org/grid_data/download/71579873?include=energy
Slc26a11	1.019	http://api.brain-map.org/grid_data/download/71570749?include=energy
Vegfb	1.019	http://api.brain-map.org/grid_data/download/75079746?include=energy
Dscc1	1.019	http://api.brain-map.org/grid_data/download/71991690?include=energy
Ints4	1.019	http://api.brain-map.org/grid_data/download/69169409?include=energy
Cdk7	1.019	http://api.brain-map.org/grid_data/download/545092?include=energy
Chmp4c	1.019	http://api.brain-map.org/grid_data/download/70203489?include=energy
Clasp2	1.019	http://api.brain-map.org/grid_data/download/71211477?include=energy
Tns1	1.019	http://api.brain-map.org/grid_data/download/73512354?include=energy
1110051M20Rik	1.019	http://api.brain-map.org/grid_data/download/67936384?include=energy
LOC214238	1.019	http://api.brain-map.org/grid_data/download/74988751?include=energy
Bche	1.019	http://api.brain-map.org/grid_data/download/74881889?include=energy
Zfp551	1.019	http://api.brain-map.org/grid_data/download/71211464?include=energy
Sgms2	1.019	http://api.brain-map.org/grid_data/download/70218643?include=energy
Il2rg	1.019	http://api.brain-map.org/grid_data/download/80341157?include=energy
Foxl1	1.019	http://api.brain-map.org/grid_data/download/100142544?include=energy
Meox2	1.019	http://api.brain-map.org/grid_data/download/79632234?include=energy
Flg2	1.019	http://api.brain-map.org/grid_data/download/70566035?include=energy
Med12l	1.019	http://api.brain-map.org/grid_data/download/73808618?include=energy
Rasl11a	1.019	http://api.brain-map.org/grid_data/download/75988636?include=energy
1700019D03Rik	1.019	http://api.brain-map.org/grid_data/download/70474845?include=energy
Kpna4	1.019	http://api.brain-map.org/grid_data/download/2359?include=energy
Phex	1.019	http://api.brain-map.org/grid_data/download/74821723?include=energy
Fam193b	1.019	http://api.brain-map.org/grid_data/download/68161454?include=energy
Dnajb12	1.019	http://api.brain-map.org/grid_data/download/2153?include=energy
Prap1	1.019	http://api.brain-map.org/grid_data/download/75831747?include=energy
Foxa2	1.019	http://api.brain-map.org/grid_data/download/71307316?include=energy
Srrt	1.019	http://api.brain-map.org/grid_data/download/69169660?include=energy
Psma2	1.019	http://api.brain-map.org/grid_data/download/68797492?include=energy
Fgf18	1.019	http://api.brain-map.org/grid_data/download/69672426?include=energy
Hdc	1.019	http://api.brain-map.org/grid_data/download/71016663?include=energy
Mrpl42	1.019	http://api.brain-map.org/grid_data/download/69173343?include=energy
Ep400	1.019	http://api.brain-map.org/grid_data/download/69672060?include=energy
4930431F12Rik	1.019	http://api.brain-map.org/grid_data/download/71891677?include=energy
Lmtk3	1.019	http://api.brain-map.org/grid_data/download/81654127?include=energy
Nr1d2	1.019	http://api.brain-map.org/grid_data/download/1245?include=energy
Dram2	1.019	http://api.brain-map.org/grid_data/download/69170318?include=energy
Slc45a1	1.019	http://api.brain-map.org/grid_data/download/69626997?include=energy
B230312C02Rik	1.019	http://api.brain-map.org/grid_data/download/74363336?include=energy
Ppp2r5c	1.019	http://api.brain-map.org/grid_data/download/73927607?include=energy
Banf2	1.019	http://api.brain-map.org/grid_data/download/69114168?include=energy
4932438H23Rik	1.019	http://api.brain-map.org/grid_data/download/1321?include=energy
Picalm	1.019	http://api.brain-map.org/grid_data/download/68301539?include=energy
Thap4	1.019	http://api.brain-map.org/grid_data/download/76097726?include=energy
Polr2m	1.019	http://api.brain-map.org/grid_data/download/1740?include=energy
Mef2a	1.019	http://api.brain-map.org/grid_data/download/199392?include=energy
Dip2b	1.018	http://api.brain-map.org/grid_data/download/73606642?include=energy
Atp6v0e2	1.018	http://api.brain-map.org/grid_data/download/69059973?include=energy
F11	1.018	http://api.brain-map.org/grid_data/download/71627819?include=energy
Mm.353395	1.018	http://api.brain-map.org/grid_data/download/74509494?include=energy
Pde6d	1.018	http://api.brain-map.org/grid_data/download/69059804?include=energy
Acta1	1.018	http://api.brain-map.org/grid_data/download/60?include=energy
Ube2i	1.018	http://api.brain-map.org/grid_data/download/45666?include=energy
H2afb1	1.018	http://api.brain-map.org/grid_data/download/71763978?include=energy
LOC433188	1.018	http://api.brain-map.org/grid_data/download/71919451?include=energy
Tsga13	1.018	http://api.brain-map.org/grid_data/download/70302842?include=energy
As3mt	1.018	http://api.brain-map.org/grid_data/download/69169451?include=energy
D930021N14	1.018	http://api.brain-map.org/grid_data/download/70565446?include=energy
Trpc4ap	1.018	http://api.brain-map.org/grid_data/download/68633261?include=energy
E230008N13Rik	1.018	http://api.brain-map.org/grid_data/download/73513723?include=energy
6430567L13Rik*	1.018	http://api.brain-map.org/grid_data/download/73870581?include=energy
Apol7a	1.018	http://api.brain-map.org/grid_data/download/70724469?include=energy
Tmem143	1.018	http://api.brain-map.org/grid_data/download/68410219?include=energy
Des	1.018	http://api.brain-map.org/grid_data/download/68666619?include=energy
Zfand5	1.018	http://api.brain-map.org/grid_data/download/1140?include=energy
Rinl	1.018	http://api.brain-map.org/grid_data/download/69838405?include=energy
Gtpbp8	1.018	http://api.brain-map.org/grid_data/download/71919080?include=energy
Ssr2	1.018	http://api.brain-map.org/grid_data/download/121097?include=energy
Col4a4	1.018	http://api.brain-map.org/grid_data/download/69568872?include=energy
LOC433316	1.018	http://api.brain-map.org/grid_data/download/70723092?include=energy
Filip1	1.018	http://api.brain-map.org/grid_data/download/73635796?include=energy
Swi5	1.018	http://api.brain-map.org/grid_data/download/68546452?include=energy
Slc39a12	1.018	http://api.brain-map.org/grid_data/download/70302168?include=energy
Syde1	1.018	http://api.brain-map.org/grid_data/download/72081750?include=energy
Tubgcp3	1.018	http://api.brain-map.org/grid_data/download/69174523?include=energy
Ahcyl1	1.017	http://api.brain-map.org/grid_data/download/69838759?include=energy
Slc38a2	1.017	http://api.brain-map.org/grid_data/download/68845729?include=energy
1700041A03Rik*	1.017	http://api.brain-map.org/grid_data/download/73716650?include=energy
E130016E03Rik	1.017	http://api.brain-map.org/grid_data/download/1488?include=energy
Slc10a1	1.017	http://api.brain-map.org/grid_data/download/74743423?include=energy
Mycbpap	1.017	http://api.brain-map.org/grid_data/download/67787350?include=energy
Set	1.017	http://api.brain-map.org/grid_data/download/68301831?include=energy
Prune2	1.017	http://api.brain-map.org/grid_data/download/74363335?include=energy
Cbx4	1.017	http://api.brain-map.org/grid_data/download/73497017?include=energy
Rims3	1.017	http://api.brain-map.org/grid_data/download/74512027?include=energy
Sbp	1.017	http://api.brain-map.org/grid_data/download/69114759?include=energy
D930017J03Rik	1.017	http://api.brain-map.org/grid_data/download/71160283?include=energy
Krt71	1.017	http://api.brain-map.org/grid_data/download/69735962?include=energy
Txnrd3	1.017	http://api.brain-map.org/grid_data/download/71670706?include=energy
Inf2	1.017	http://api.brain-map.org/grid_data/download/69202902?include=energy
BC024537	1.017	http://api.brain-map.org/grid_data/download/69236670?include=energy
Lrrtm1	1.017	http://api.brain-map.org/grid_data/download/70805896?include=energy
Rtn3	1.017	http://api.brain-map.org/grid_data/download/83161?include=energy
Prr5l	1.017	http://api.brain-map.org/grid_data/download/68797510?include=energy
Olfr459	1.017	http://api.brain-map.org/grid_data/download/73653434?include=energy
Ttc6	1.017	http://api.brain-map.org/grid_data/download/71670724?include=energy
4930548F15Rik	1.017	http://api.brain-map.org/grid_data/download/74580159?include=energy
St8sia1	1.017	http://api.brain-map.org/grid_data/download/73907494?include=energy
Fbrs	1.017	http://api.brain-map.org/grid_data/download/2207?include=energy
Wnt11	1.017	http://api.brain-map.org/grid_data/download/2727?include=energy
Fam83g	1.017	http://api.brain-map.org/grid_data/download/69202460?include=energy
Psmg2	1.017	http://api.brain-map.org/grid_data/download/67765704?include=energy
Olfr1395	1.017	http://api.brain-map.org/grid_data/download/73653290?include=energy
Sbf1	1.017	http://api.brain-map.org/grid_data/download/276264?include=energy
Nkx2-1	1.017	http://api.brain-map.org/grid_data/download/74988460?include=energy
Sar1b	1.017	http://api.brain-map.org/grid_data/download/69873397?include=energy
Bpifb6	1.017	http://api.brain-map.org/grid_data/download/75831707?include=energy
Rhobtb2	1.017	http://api.brain-map.org/grid_data/download/68193036?include=energy
Zfp114	1.017	http://api.brain-map.org/grid_data/download/74957922?include=energy
Gfap	1.016	http://api.brain-map.org/grid_data/download/1357?include=energy
Etfa	1.016	http://api.brain-map.org/grid_data/download/74581380?include=energy
Ppp2r3a	1.016	http://api.brain-map.org/grid_data/download/100142518?include=energy
Acadvl	1.016	http://api.brain-map.org/grid_data/download/227161?include=energy
Reep2	1.016	http://api.brain-map.org/grid_data/download/583550?include=energy
4930430F08Rik	1.016	http://api.brain-map.org/grid_data/download/71493204?include=energy
Edem1	1.016	http://api.brain-map.org/grid_data/download/68633068?include=energy
Rbm6	1.016	http://api.brain-map.org/grid_data/download/68562988?include=energy
Ms4a8a	1.016	http://api.brain-map.org/grid_data/download/76081024?include=energy
Tkt	1.016	http://api.brain-map.org/grid_data/download/71393286?include=energy
Zfp612	1.016	http://api.brain-map.org/grid_data/download/69012944?include=energy
2310014F06Rik	1.016	http://api.brain-map.org/grid_data/download/70218679?include=energy
Arap3	1.016	http://api.brain-map.org/grid_data/download/69134280?include=energy
Chga	1.016	http://api.brain-map.org/grid_data/download/2093?include=energy
Gm4814	1.016	http://api.brain-map.org/grid_data/download/71808600?include=energy
Lag3	1.016	http://api.brain-map.org/grid_data/download/71920417?include=energy
Gm5103	1.016	http://api.brain-map.org/grid_data/download/71808478?include=energy
F830022J16Rik*	1.016	http://api.brain-map.org/grid_data/download/73731418?include=energy
Gm8766	1.016	http://api.brain-map.org/grid_data/download/80342275?include=energy
Lrp10	1.016	http://api.brain-map.org/grid_data/download/69014482?include=energy
Rasgrp3	1.016	http://api.brain-map.org/grid_data/download/77620467?include=energy
6030422M02Rik	1.016	http://api.brain-map.org/grid_data/download/70227866?include=energy
1600016N20Rik	1.016	http://api.brain-map.org/grid_data/download/74581271?include=energy
E130307A14Rik	1.016	http://api.brain-map.org/grid_data/download/70239003?include=energy
Cct3	1.016	http://api.brain-map.org/grid_data/download/69540543?include=energy
Tmem50b	1.016	http://api.brain-map.org/grid_data/download/133?include=energy
Glrb	1.016	http://api.brain-map.org/grid_data/download/498?include=energy
Cd200r1	1.016	http://api.brain-map.org/grid_data/download/68667748?include=energy
Gm655	1.016	http://api.brain-map.org/grid_data/download/71832901?include=energy
E130306D19Rik	1.016	http://api.brain-map.org/grid_data/download/69671744?include=energy
Fam195a	1.016	http://api.brain-map.org/grid_data/download/1996?include=energy
Fam20b	1.016	http://api.brain-map.org/grid_data/download/68158082?include=energy
Dnpep	1.016	http://api.brain-map.org/grid_data/download/69627039?include=energy
Pbld2	1.016	http://api.brain-map.org/grid_data/download/70203664?include=energy
Adprh	1.016	http://api.brain-map.org/grid_data/download/71608214?include=energy
Zfp941	1.016	http://api.brain-map.org/grid_data/download/74578594?include=energy
Jakmip1	1.015	http://api.brain-map.org/grid_data/download/71281710?include=energy
Rimkla	1.015	http://api.brain-map.org/grid_data/download/70523726?include=energy
Rell1	1.015	http://api.brain-map.org/grid_data/download/72007746?include=energy
Ahcy	1.015	http://api.brain-map.org/grid_data/download/68341495?include=energy
Trpt1	1.015	http://api.brain-map.org/grid_data/download/68744725?include=energy
Plekha6	1.015	http://api.brain-map.org/grid_data/download/70546360?include=energy
Trim14	1.015	http://api.brain-map.org/grid_data/download/72004445?include=energy
Stk38	1.015	http://api.brain-map.org/grid_data/download/512772?include=energy
Zc3h10	1.015	http://api.brain-map.org/grid_data/download/67815959?include=energy
Adm	1.015	http://api.brain-map.org/grid_data/download/74819251?include=energy
Car1	1.015	http://api.brain-map.org/grid_data/download/71617032?include=energy
Srsf12	1.015	http://api.brain-map.org/grid_data/download/71210920?include=energy
Itm2b	1.015	http://api.brain-map.org/grid_data/download/532581?include=energy
Gm1110	1.015	http://api.brain-map.org/grid_data/download/74578254?include=energy
Chst11	1.015	http://api.brain-map.org/grid_data/download/73931625?include=energy
Sprr2d	1.015	http://api.brain-map.org/grid_data/download/75198202?include=energy
Abcd1	1.015	http://api.brain-map.org/grid_data/download/356086?include=energy
2900041M22Rik	1.015	http://api.brain-map.org/grid_data/download/72076581?include=energy
4022404D01Rik*	1.015	http://api.brain-map.org/grid_data/download/73906461?include=energy
Chst2	1.015	http://api.brain-map.org/grid_data/download/266?include=energy
Slc45a3	1.015	http://api.brain-map.org/grid_data/download/68410608?include=energy
Thnsl1	1.015	http://api.brain-map.org/grid_data/download/74988343?include=energy
Cox4nb	1.015	http://api.brain-map.org/grid_data/download/76003321?include=energy
1700123O20Rik	1.015	http://api.brain-map.org/grid_data/download/564644?include=energy
Cend1	1.015	http://api.brain-map.org/grid_data/download/68196909?include=energy
Zfp869	1.015	http://api.brain-map.org/grid_data/download/70593123?include=energy
Agtr2	1.015	http://api.brain-map.org/grid_data/download/71608203?include=energy
Il1f9	1.015	http://api.brain-map.org/grid_data/download/70278039?include=energy
Krt85	1.015	http://api.brain-map.org/grid_data/download/73497588?include=energy
Ly6k	1.015	http://api.brain-map.org/grid_data/download/69114626?include=energy
Txk	1.015	http://api.brain-map.org/grid_data/download/69817055?include=energy
Arl2	1.015	http://api.brain-map.org/grid_data/download/74819344?include=energy
C030039L03Rik	1.014	http://api.brain-map.org/grid_data/download/69837840?include=energy
Tfap2a	1.014	http://api.brain-map.org/grid_data/download/2661?include=energy
Ghitm	1.014	http://api.brain-map.org/grid_data/download/1207?include=energy
1700029J07Rik	1.014	http://api.brain-map.org/grid_data/download/74634897?include=energy
Ndufb10	1.014	http://api.brain-map.org/grid_data/download/71608213?include=energy
Naip1	1.014	http://api.brain-map.org/grid_data/download/76003500?include=energy
Mcm3	1.014	http://api.brain-map.org/grid_data/download/71656071?include=energy
Rps11	1.014	http://api.brain-map.org/grid_data/download/100143572?include=energy
Cd36	1.014	http://api.brain-map.org/grid_data/download/69540587?include=energy
Aldh3b1	1.014	http://api.brain-map.org/grid_data/download/75828203?include=energy
St8sia3	1.014	http://api.brain-map.org/grid_data/download/73929595?include=energy
Gm1125	1.014	http://api.brain-map.org/grid_data/download/71717570?include=energy
Nlrc5	1.014	http://api.brain-map.org/grid_data/download/73731481?include=energy
1700029F09Rik	1.014	http://api.brain-map.org/grid_data/download/70609151?include=energy
Csnk1e	1.014	http://api.brain-map.org/grid_data/download/68563006?include=energy
Ninj1	1.014	http://api.brain-map.org/grid_data/download/71670490?include=energy
Psmd5	1.014	http://api.brain-map.org/grid_data/download/75146002?include=energy
Rtdr1	1.014	http://api.brain-map.org/grid_data/download/70301937?include=energy
Igha	1.014	http://api.brain-map.org/grid_data/download/74743447?include=energy
Krtap12-1	1.014	http://api.brain-map.org/grid_data/download/1367?include=energy
Gigyf2	1.014	http://api.brain-map.org/grid_data/download/73906233?include=energy
Pld6	1.014	http://api.brain-map.org/grid_data/download/71832894?include=energy
1700120K04Rik	1.014	http://api.brain-map.org/grid_data/download/100144874?include=energy
Eif3g	1.014	http://api.brain-map.org/grid_data/download/293259?include=energy
Arl6ip1	1.014	http://api.brain-map.org/grid_data/download/69526874?include=energy
Dclk3	1.014	http://api.brain-map.org/grid_data/download/72079921?include=energy
G630082H20Rik*	1.014	http://api.brain-map.org/grid_data/download/73731265?include=energy
Sacs	1.014	http://api.brain-map.org/grid_data/download/74743372?include=energy
LOC436390	1.014	http://api.brain-map.org/grid_data/download/72076533?include=energy
Pnmal2	1.014	http://api.brain-map.org/grid_data/download/70787426?include=energy
Kbtbd11	1.014	http://api.brain-map.org/grid_data/download/69288518?include=energy
Cidec	1.014	http://api.brain-map.org/grid_data/download/69548952?include=energy
Lpo	1.014	http://api.brain-map.org/grid_data/download/68795451?include=energy
Xirp2	1.014	http://api.brain-map.org/grid_data/download/70203483?include=energy
Ctsl	1.014	http://api.brain-map.org/grid_data/download/1187?include=energy
Tbx22	1.014	http://api.brain-map.org/grid_data/download/73606561?include=energy
Suclg1	1.014	http://api.brain-map.org/grid_data/download/564780?include=energy
Nup155	1.014	http://api.brain-map.org/grid_data/download/69782479?include=energy
Itga2	1.014	http://api.brain-map.org/grid_data/download/69735562?include=energy
Npy2r	1.013	http://api.brain-map.org/grid_data/download/69258196?include=energy
Krtap1-5	1.013	http://api.brain-map.org/grid_data/download/70303779?include=energy

Niacr1	1.013	http://api.brain-map.org/grid_data/download/100144869?include=energy
S100a1	1.013	http://api.brain-map.org/grid_data/download/70429411?include=energy
Tuba3a	1.013	http://api.brain-map.org/grid_data/download/69114908?include=energy
Ddo	1.013	http://api.brain-map.org/grid_data/download/68203462?include=energy
4732418C07Rik	1.013	http://api.brain-map.org/grid_data/download/68844731?include=energy
Snrnp70	1.013	http://api.brain-map.org/grid_data/download/68076882?include=energy
Ankrd36	1.013	http://api.brain-map.org/grid_data/download/70609148?include=energy
Mospd3	1.013	http://api.brain-map.org/grid_data/download/75198070?include=energy
Sez6	1.013	http://api.brain-map.org/grid_data/download/71063725?include=energy
Chd4	1.013	http://api.brain-map.org/grid_data/download/68745036?include=energy
1700017G19Rik	1.013	http://api.brain-map.org/grid_data/download/71919487?include=energy
Aph1c	1.013	http://api.brain-map.org/grid_data/download/69352730?include=energy
LOC432670	1.013	http://api.brain-map.org/grid_data/download/68797559?include=energy
Ccdc46	1.013	http://api.brain-map.org/grid_data/download/73716495?include=energy
Gm628	1.013	http://api.brain-map.org/grid_data/download/74514073?include=energy
Acsl6	1.013	http://api.brain-map.org/grid_data/download/71836810?include=energy
Krt16	1.013	http://api.brain-map.org/grid_data/download/69735933?include=energy
Scn1a	1.013	http://api.brain-map.org/grid_data/download/77340528?include=energy
Tmem106c	1.013	http://api.brain-map.org/grid_data/download/68745456?include=energy
Ttk	1.013	http://api.brain-map.org/grid_data/download/70565357?include=energy
Slc6a18	1.013	http://api.brain-map.org/grid_data/download/78634002?include=energy
Mm.145671	1.013	http://api.brain-map.org/grid_data/download/71148184?include=energy
Trpv4	1.013	http://api.brain-map.org/grid_data/download/73992707?include=energy
2900060N12Rik	1.013	http://api.brain-map.org/grid_data/download/72283324?include=energy
A930033C23Rik	1.013	http://api.brain-map.org/grid_data/download/74300717?include=energy
Hdac5	1.013	http://api.brain-map.org/grid_data/download/69734965?include=energy
Slc25a10	1.013	http://api.brain-map.org/grid_data/download/74047855?include=energy
Ict1	1.013	http://api.brain-map.org/grid_data/download/68744827?include=energy
Sema6d	1.013	http://api.brain-map.org/grid_data/download/74750032?include=energy
4930579E07Rik*	1.013	http://api.brain-map.org/grid_data/download/73906521?include=energy
4921509C19Rik	1.013	http://api.brain-map.org/grid_data/download/70205614?include=energy
Olfr522	1.013	http://api.brain-map.org/grid_data/download/74724139?include=energy
Rab3a	1.013	http://api.brain-map.org/grid_data/download/68924503?include=energy
Ifi205	1.013	http://api.brain-map.org/grid_data/download/75079787?include=energy
G3bp2	1.013	http://api.brain-map.org/grid_data/download/75198076?include=energy
Pon2	1.013	http://api.brain-map.org/grid_data/download/68162244?include=energy
Prpf19	1.012	http://api.brain-map.org/grid_data/download/68191420?include=energy
Aknad1	1.012	http://api.brain-map.org/grid_data/download/72005994?include=energy
Lyz1	1.012	http://api.brain-map.org/grid_data/download/74819693?include=energy
Grhpr	1.012	http://api.brain-map.org/grid_data/download/68269712?include=energy
Aldh18a1	1.012	http://api.brain-map.org/grid_data/download/68080702?include=energy
Flcn	1.012	http://api.brain-map.org/grid_data/download/227163?include=energy
Rbck1	1.012	http://api.brain-map.org/grid_data/download/68632166?include=energy
Isca1	1.012	http://api.brain-map.org/grid_data/download/67978773?include=energy
Car14	1.012	http://api.brain-map.org/grid_data/download/69012735?include=energy
Chd6	1.012	http://api.brain-map.org/grid_data/download/77340492?include=energy
Gnat3	1.012	http://api.brain-map.org/grid_data/download/71832888?include=energy
Qars	1.012	http://api.brain-map.org/grid_data/download/69169893?include=energy
Fbxl7	1.012	http://api.brain-map.org/grid_data/download/73834367?include=energy
Stard4	1.012	http://api.brain-map.org/grid_data/download/69059820?include=energy
Tifab	1.012	http://api.brain-map.org/grid_data/download/68410590?include=energy
Ccdc158	1.012	http://api.brain-map.org/grid_data/download/71808790?include=energy
Tnrc6a	1.012	http://api.brain-map.org/grid_data/download/314?include=energy
Ercc5	1.012	http://api.brain-map.org/grid_data/download/69672132?include=energy
Tomm20	1.012	http://api.brain-map.org/grid_data/download/74819230?include=energy
Defb29	1.012	http://api.brain-map.org/grid_data/download/71919062?include=energy
Kbtbd3	1.012	http://api.brain-map.org/grid_data/download/74988611?include=energy
Ropn1l	1.012	http://api.brain-map.org/grid_data/download/69174481?include=energy
Ppp1r3a	1.012	http://api.brain-map.org/grid_data/download/74749168?include=energy
Prdx1	1.012	http://api.brain-map.org/grid_data/download/836?include=energy
Zfp385a	1.012	http://api.brain-map.org/grid_data/download/76098306?include=energy
4933401J08Rik*	1.012	http://api.brain-map.org/grid_data/download/73870539?include=energy
Clgn	1.012	http://api.brain-map.org/grid_data/download/74425567?include=energy
Phox2b	1.012	http://api.brain-map.org/grid_data/download/2480?include=energy
Slc25a43	1.012	http://api.brain-map.org/grid_data/download/78634043?include=energy
Slc6a20a	1.012	http://api.brain-map.org/grid_data/download/71210956?include=energy
Birc3	1.012	http://api.brain-map.org/grid_data/download/75694726?include=energy
Pou3f2	1.012	http://api.brain-map.org/grid_data/download/81655548?include=energy
Ergic1	1.012	http://api.brain-map.org/grid_data/download/1952?include=energy
Dnajc8	1.011	http://api.brain-map.org/grid_data/download/81600136?include=energy
Hilpda	1.011	http://api.brain-map.org/grid_data/download/71627817?include=energy
Cdkl4	1.011	http://api.brain-map.org/grid_data/download/70298489?include=energy
Ngb	1.011	http://api.brain-map.org/grid_data/download/79556712?include=energy
Spire2	1.011	http://api.brain-map.org/grid_data/download/70439171?include=energy
Cd79b	1.011	http://api.brain-map.org/grid_data/download/75694762?include=energy
A430005L14Rik	1.011	http://api.brain-map.org/grid_data/download/69113757?include=energy
Tekt5	1.011	http://api.brain-map.org/grid_data/download/74635749?include=energy
A930006J02Rik	1.011	http://api.brain-map.org/grid_data/download/74580942?include=energy
Olfr616	1.011	http://api.brain-map.org/grid_data/download/74734746?include=energy
Krt26	1.011	http://api.brain-map.org/grid_data/download/70205352?include=energy
Prrt4	1.011	http://api.brain-map.org/grid_data/download/74822583?include=energy
Tecr	1.011	http://api.brain-map.org/grid_data/download/544902?include=energy
Zfp651	1.011	http://api.brain-map.org/grid_data/download/73584717?include=energy
Nap1l5	1.011	http://api.brain-map.org/grid_data/download/72080123?include=energy
Slc23a2	1.011	http://api.brain-map.org/grid_data/download/68797840?include=energy
Olfr214	1.011	http://api.brain-map.org/grid_data/download/74720910?include=energy
Spdef	1.011	http://api.brain-map.org/grid_data/download/81654083?include=energy
Myl9	1.011	http://api.brain-map.org/grid_data/download/69838564?include=energy
Uchl1	1.011	http://api.brain-map.org/grid_data/download/79556674?include=energy
Hrasls	1.011	http://api.brain-map.org/grid_data/download/69114890?include=energy
Pnpo	1.011	http://api.brain-map.org/grid_data/download/70743879?include=energy
Slc7a10	1.011	http://api.brain-map.org/grid_data/download/69874042?include=energy
Gcg	1.011	http://api.brain-map.org/grid_data/download/75749416?include=energy
Dtd1	1.011	http://api.brain-map.org/grid_data/download/1742?include=energy
Klhl2	1.011	http://api.brain-map.org/grid_data/download/73584837?include=energy
Armcx2	1.011	http://api.brain-map.org/grid_data/download/69013585?include=energy
Sufu	1.011	http://api.brain-map.org/grid_data/download/71808552?include=energy
D17H6S56E-3	1.011	http://api.brain-map.org/grid_data/download/71496381?include=energy
A230069K02Rik*	1.011	http://api.brain-map.org/grid_data/download/73808528?include=energy
Dap3	1.011	http://api.brain-map.org/grid_data/download/68342075?include=energy
Mrpl3	1.011	http://api.brain-map.org/grid_data/download/70919884?include=energy
She	1.011	http://api.brain-map.org/grid_data/download/70305180?include=energy
Dok7	1.011	http://api.brain-map.org/grid_data/download/71569247?include=energy
Jun	1.011	http://api.brain-map.org/grid_data/download/2333?include=energy
Ppp2r4	1.011	http://api.brain-map.org/grid_data/download/68668048?include=energy
9430025C20Rik	1.011	http://api.brain-map.org/grid_data/download/70305186?include=energy
Afg3l1	1.011	http://api.brain-map.org/grid_data/download/75831742?include=energy
Tgtp1	1.011	http://api.brain-map.org/grid_data/download/71656098?include=energy
Spag7	1.01	http://api.brain-map.org/grid_data/download/69014949?include=energy
Tgfbr2	1.01	http://api.brain-map.org/grid_data/download/74819696?include=energy
Ripply3	1.01	http://api.brain-map.org/grid_data/download/367?include=energy
Ins2	1.01	http://api.brain-map.org/grid_data/download/71920273?include=energy
Wdr66	1.01	http://api.brain-map.org/grid_data/download/71569102?include=energy
Etos1	1.01	http://api.brain-map.org/grid_data/download/70276765?include=energy
Itgae	1.01	http://api.brain-map.org/grid_data/download/69735592?include=energy
Ndufb7	1.01	http://api.brain-map.org/grid_data/download/68545312?include=energy
Gsg1l	1.01	http://api.brain-map.org/grid_data/download/73636097?include=energy
4930588G17Rik	1.01	http://api.brain-map.org/grid_data/download/71493675?include=energy
Usp29	1.01	http://api.brain-map.org/grid_data/download/70194636?include=energy
Catsperb	1.01	http://api.brain-map.org/grid_data/download/72183011?include=energy
Slco3a1	1.01	http://api.brain-map.org/grid_data/download/293691?include=energy
Gm4827	1.01	http://api.brain-map.org/grid_data/download/73818814?include=energy
Ufsp2	1.01	http://api.brain-map.org/grid_data/download/71763990?include=energy
Gdi1	1.01	http://api.brain-map.org/grid_data/download/74357589?include=energy
Mm.297635	1.01	http://api.brain-map.org/grid_data/download/71161489?include=energy
Ttc19	1.01	http://api.brain-map.org/grid_data/download/68795425?include=energy
Manba	1.01	http://api.brain-map.org/grid_data/download/72007562?include=energy
Msx2	1.01	http://api.brain-map.org/grid_data/download/70723504?include=energy
Pthlh	1.01	http://api.brain-map.org/grid_data/download/73592531?include=energy
Hsp90aa1	1.01	http://api.brain-map.org/grid_data/download/69012681?include=energy
Snx3	1.01	http://api.brain-map.org/grid_data/download/69885624?include=energy
Cope	1.01	http://api.brain-map.org/grid_data/download/68269664?include=energy
Sacm1l	1.01	http://api.brain-map.org/grid_data/download/74882832?include=energy
Hgsnat	1.01	http://api.brain-map.org/grid_data/download/68341949?include=energy
LOC433944	1.01	http://api.brain-map.org/grid_data/download/71456127?include=energy
Rps9	1.01	http://api.brain-map.org/grid_data/download/68563128?include=energy
Prkar2b	1.01	http://api.brain-map.org/grid_data/download/74583119?include=energy
Kank1	1.01	http://api.brain-map.org/grid_data/download/69133686?include=energy
Polb	1.01	http://api.brain-map.org/grid_data/download/69174511?include=energy
Noc3l	1.01	http://api.brain-map.org/grid_data/download/69170294?include=energy
Serpinb8	1.01	http://api.brain-map.org/grid_data/download/293668?include=energy
Atad5	1.01	http://api.brain-map.org/grid_data/download/70296026?include=energy
LOC384865	1.009	http://api.brain-map.org/grid_data/download/71456086?include=energy
Oasl1	1.009	http://api.brain-map.org/grid_data/download/68446009?include=energy
Trim2	1.009	http://api.brain-map.org/grid_data/download/69838371?include=energy
Fam19a2	1.009	http://api.brain-map.org/grid_data/download/73636033?include=energy
Ckap5	1.009	http://api.brain-map.org/grid_data/download/69407959?include=energy
Ovgp1	1.009	http://api.brain-map.org/grid_data/download/71670680?include=energy
Rpl34	1.009	http://api.brain-map.org/grid_data/download/68744869?include=energy
Nptn	1.009	http://api.brain-map.org/grid_data/download/74581026?include=energy
0610007C21Rik	1.009	http://api.brain-map.org/grid_data/download/69113518?include=energy
Gdf11	1.009	http://api.brain-map.org/grid_data/download/74272478?include=energy
Syt3	1.009	http://api.brain-map.org/grid_data/download/1026?include=energy
Gm1630	1.009	http://api.brain-map.org/grid_data/download/70298567?include=energy
Tpx2	1.009	http://api.brain-map.org/grid_data/download/70192015?include=energy
Flywch2	1.009	http://api.brain-map.org/grid_data/download/68632008?include=energy
Ndufv3	1.009	http://api.brain-map.org/grid_data/download/12?include=energy
Ank1	1.009	http://api.brain-map.org/grid_data/download/70560276?include=energy
Stat5a	1.009	http://api.brain-map.org/grid_data/download/77413690?include=energy
Card9	1.009	http://api.brain-map.org/grid_data/download/71662904?include=energy
Galnt3	1.009	http://api.brain-map.org/grid_data/download/68920689?include=energy
F830220E19Rik*	1.009	http://api.brain-map.org/grid_data/download/73748782?include=energy
Plekha8	1.009	http://api.brain-map.org/grid_data/download/69114186?include=energy
Eif4a1	1.009	http://api.brain-map.org/grid_data/download/68924419?include=energy
Tbc1d2	1.009	http://api.brain-map.org/grid_data/download/71809292?include=energy
Galk2	1.009	http://api.brain-map.org/grid_data/download/69672748?include=energy
4931429L15Rik	1.009	http://api.brain-map.org/grid_data/download/71892047?include=energy
Praf2	1.009	http://api.brain-map.org/grid_data/download/69549607?include=energy
Lamp1	1.009	http://api.brain-map.org/grid_data/download/69736022?include=energy
Phactr4	1.009	http://api.brain-map.org/grid_data/download/71924214?include=energy
Rcsd1	1.009	http://api.brain-map.org/grid_data/download/75934525?include=energy
Fam122b	1.009	http://api.brain-map.org/grid_data/download/69407612?include=energy
Snhg11	1.009	http://api.brain-map.org/grid_data/download/73992946?include=energy
Slc10a6	1.009	http://api.brain-map.org/grid_data/download/69446280?include=energy
Fbp2	1.009	http://api.brain-map.org/grid_data/download/68301347?include=energy
Plin5	1.009	http://api.brain-map.org/grid_data/download/176775?include=energy
Glycam1	1.009	http://api.brain-map.org/grid_data/download/69672898?include=energy
Ndrg4	1.009	http://api.brain-map.org/grid_data/download/69782109?include=energy
4933427E13Rik	1.009	http://api.brain-map.org/grid_data/download/71892161?include=energy
Dnajc13	1.009	http://api.brain-map.org/grid_data/download/81600134?include=energy
C030048B08Rik	1.008	http://api.brain-map.org/grid_data/download/176782?include=energy
Slc40a1	1.008	http://api.brain-map.org/grid_data/download/79904732?include=energy
F630015J07Rik*	1.008	http://api.brain-map.org/grid_data/download/73748836?include=energy
Nol7	1.008	http://api.brain-map.org/grid_data/download/69782306?include=energy
Plscr5	1.008	http://api.brain-map.org/grid_data/download/70595883?include=energy
4930470H14Rik	1.008	http://api.brain-map.org/grid_data/download/71891749?include=energy
Cst12	1.008	http://api.brain-map.org/grid_data/download/76081045?include=energy
Plk1	1.008	http://api.brain-map.org/grid_data/download/2487?include=energy
Arhgap10	1.008	http://api.brain-map.org/grid_data/download/74509482?include=energy
Heatr7a	1.008	http://api.brain-map.org/grid_data/download/73992940?include=energy
Mib2	1.008	http://api.brain-map.org/grid_data/download/69262258?include=energy
Rcan3	1.008	http://api.brain-map.org/grid_data/download/70946481?include=energy
Dctn1	1.008	http://api.brain-map.org/grid_data/download/70946413?include=energy
Mta3	1.008	http://api.brain-map.org/grid_data/download/130537?include=energy
Gm97	1.008	http://api.brain-map.org/grid_data/download/71832988?include=energy
Map4k4	1.008	http://api.brain-map.org/grid_data/download/69752041?include=energy
Olfr362	1.008	http://api.brain-map.org/grid_data/download/74724049?include=energy
Olfr365	1.008	http://api.brain-map.org/grid_data/download/74724055?include=energy
Rps27a	1.008	http://api.brain-map.org/grid_data/download/100143502?include=energy
Samhd1	1.008	http://api.brain-map.org/grid_data/download/75694483?include=energy
4933400A11Rik	1.008	http://api.brain-map.org/grid_data/download/71892041?include=energy
Spg11	1.008	http://api.brain-map.org/grid_data/download/71809007?include=energy
Tnfrsf23	1.008	http://api.brain-map.org/grid_data/download/100146135?include=energy
Wdr67	1.008	http://api.brain-map.org/grid_data/download/69549313?include=energy
Mdh2	1.008	http://api.brain-map.org/grid_data/download/68080709?include=energy
Hdac3	1.008	http://api.brain-map.org/grid_data/download/71232781?include=energy
7420498E04Rik*	1.008	http://api.brain-map.org/grid_data/download/75043148?include=energy
Sympk	1.008	http://api.brain-map.org/grid_data/download/68924467?include=energy
A830031A19Rik	1.008	http://api.brain-map.org/grid_data/download/74277746?include=energy
Ppbp	1.008	http://api.brain-map.org/grid_data/download/76081001?include=energy
Eif3f	1.008	http://api.brain-map.org/grid_data/download/69671953?include=energy
Surf1	1.008	http://api.brain-map.org/grid_data/download/69080040?include=energy
Dhrs9	1.008	http://api.brain-map.org/grid_data/download/70238854?include=energy
Apobec2	1.008	http://api.brain-map.org/grid_data/download/71656091?include=energy
Col6a3	1.008	http://api.brain-map.org/grid_data/download/75081393?include=energy
Grm6	1.008	http://api.brain-map.org/grid_data/download/68845777?include=energy
Ifrd1	1.008	http://api.brain-map.org/grid_data/download/75991121?include=energy
Dync1i2	1.008	http://api.brain-map.org/grid_data/download/81600138?include=energy
Snx14	1.008	http://api.brain-map.org/grid_data/download/71789921?include=energy
Akap11	1.008	http://api.brain-map.org/grid_data/download/69526516?include=energy
Bsg	1.008	http://api.brain-map.org/grid_data/download/166?include=energy
Gm680	1.007	http://api.brain-map.org/grid_data/download/70298220?include=energy
5830411J07Rik	1.007	http://api.brain-map.org/grid_data/download/70227836?include=energy
Auh	1.007	http://api.brain-map.org/grid_data/download/71764059?include=energy
Fmr1nb	1.007	http://api.brain-map.org/grid_data/download/69672521?include=energy
Osbpl7	1.007	http://api.brain-map.org/grid_data/download/74580775?include=energy
Vps37c	1.007	http://api.brain-map.org/grid_data/download/68410620?include=energy
Phactr3	1.007	http://api.brain-map.org/grid_data/download/69837922?include=energy
Vmn2r57	1.007	http://api.brain-map.org/grid_data/download/74510116?include=energy
Gfpt1	1.007	http://api.brain-map.org/grid_data/download/69080046?include=energy
Galntl5	1.007	http://api.brain-map.org/grid_data/download/71919266?include=energy
A530053G22Rik	1.007	http://api.brain-map.org/grid_data/download/70305299?include=energy
Fam72a	1.007	http://api.brain-map.org/grid_data/download/70542930?include=energy
Wac	1.007	http://api.brain-map.org/grid_data/download/71281111?include=energy
Olfr1396	1.007	http://api.brain-map.org/grid_data/download/74720811?include=energy
Ccdc47	1.007	http://api.brain-map.org/grid_data/download/69202896?include=energy
A430057M04Rik	1.007	http://api.brain-map.org/grid_data/download/69448638?include=energy
Diap2	1.007	http://api.brain-map.org/grid_data/download/69626891?include=energy
Lrrcc1	1.007	http://api.brain-map.org/grid_data/download/71764916?include=energy
Slc6a2	1.007	http://api.brain-map.org/grid_data/download/73615562?include=energy
Grid1	1.007	http://api.brain-map.org/grid_data/download/74724739?include=energy
Cntnap1	1.007	http://api.brain-map.org/grid_data/download/73992931?include=energy
Aldh1l1	1.007	http://api.brain-map.org/grid_data/download/1724?include=energy
Zdhhc9	1.007	http://api.brain-map.org/grid_data/download/74425545?include=energy
Carns1	1.007	http://api.brain-map.org/grid_data/download/68157418?include=energy
Tspyl2	1.007	http://api.brain-map.org/grid_data/download/73817931?include=energy
Stmn3	1.007	http://api.brain-map.org/grid_data/download/1004?include=energy
Ikzf1	1.007	http://api.brain-map.org/grid_data/download/1144?include=energy
Pate4	1.007	http://api.brain-map.org/grid_data/download/70302300?include=energy
Slc39a7	1.007	http://api.brain-map.org/grid_data/download/69873953?include=energy
C230030N03Rik	1.007	http://api.brain-map.org/grid_data/download/73992924?include=energy
Prpf40b	1.007	http://api.brain-map.org/grid_data/download/71493050?include=energy
Rsl1d1	1.007	http://api.brain-map.org/grid_data/download/69169594?include=energy
Sh3pxd2a	1.007	http://api.brain-map.org/grid_data/download/69873720?include=energy
2200002K05Rik	1.007	http://api.brain-map.org/grid_data/download/68844672?include=energy
Vwf	1.007	http://api.brain-map.org/grid_data/download/73617540?include=energy
Barhl2	1.007	http://api.brain-map.org/grid_data/download/73497128?include=energy
C730034F03Rik	1.006	http://api.brain-map.org/grid_data/download/77904875?include=energy
Oit3	1.006	http://api.brain-map.org/grid_data/download/71808759?include=energy
B020018I07Rik*	1.006	http://api.brain-map.org/grid_data/download/74000510?include=energy
Tas2r131	1.006	http://api.brain-map.org/grid_data/download/70302384?include=energy
Cltc	1.006	http://api.brain-map.org/grid_data/download/69549024?include=energy
Mm.40896	1.006	http://api.brain-map.org/grid_data/download/71210259?include=energy
Mm.95774	1.006	http://api.brain-map.org/grid_data/download/71210490?include=energy
Gm923	1.006	http://api.brain-map.org/grid_data/download/70298453?include=energy
Actr3	1.006	http://api.brain-map.org/grid_data/download/69514702?include=energy
Clec14a	1.006	http://api.brain-map.org/grid_data/download/71627823?include=energy

Elp4	1.006	http://api.brain-map.org/grid_data/download/73712834?include=energy
Gnl3l	1.006	http://api.brain-map.org/grid_data/download/68920425?include=energy
Ndufb5	1.006	http://api.brain-map.org/grid_data/download/71763831?include=energy
Ankrd28	1.006	http://api.brain-map.org/grid_data/download/69526653?include=energy
Gnao1	1.006	http://api.brain-map.org/grid_data/download/72081553?include=energy
Osbpl8	1.006	http://api.brain-map.org/grid_data/download/74641162?include=energy
9530053H22	1.006	http://api.brain-map.org/grid_data/download/71569133?include=energy
1700049J03Rik	1.006	http://api.brain-map.org/grid_data/download/74357875?include=energy
Comtd1	1.006	http://api.brain-map.org/grid_data/download/69113668?include=energy
Cp	1.006	http://api.brain-map.org/grid_data/download/70920009?include=energy
Alk	1.006	http://api.brain-map.org/grid_data/download/77371829?include=energy
Nrbp2	1.006	http://api.brain-map.org/grid_data/download/75774686?include=energy
Pbxip1	1.006	http://api.brain-map.org/grid_data/download/70919633?include=energy
mCG146126	1.006	http://api.brain-map.org/grid_data/download/71212410?include=energy
Pold1	1.006	http://api.brain-map.org/grid_data/download/75694340?include=energy
F830005K03Rik	1.006	http://api.brain-map.org/grid_data/download/70276789?include=energy
Fam73a	1.006	http://api.brain-map.org/grid_data/download/71496013?include=energy
Tas2r104	1.006	http://api.brain-map.org/grid_data/download/70785146?include=energy
Prkd1	1.006	http://api.brain-map.org/grid_data/download/69817318?include=energy
Coro1b	1.006	http://api.brain-map.org/grid_data/download/69568962?include=energy
mCG148349	1.006	http://api.brain-map.org/grid_data/download/71212653?include=energy
Parp12	1.006	http://api.brain-map.org/grid_data/download/74821638?include=energy
Dcaf12l2	1.006	http://api.brain-map.org/grid_data/download/73679128?include=energy
Reep4	1.006	http://api.brain-map.org/grid_data/download/68744761?include=energy
Usp48	1.006	http://api.brain-map.org/grid_data/download/149?include=energy
Tcf7	1.006	http://api.brain-map.org/grid_data/download/77464880?include=energy
Pdhb	1.006	http://api.brain-map.org/grid_data/download/69080069?include=energy
LOC546142	1.006	http://api.brain-map.org/grid_data/download/77621657?include=energy
B230312A22Rik	1.006	http://api.brain-map.org/grid_data/download/71808771?include=energy
Atp5o	1.006	http://api.brain-map.org/grid_data/download/1158?include=energy
Spryd3	1.006	http://api.brain-map.org/grid_data/download/69528112?include=energy
F2rl1	1.006	http://api.brain-map.org/grid_data/download/199391?include=energy
Patl2	1.006	http://api.brain-map.org/grid_data/download/73818830?include=energy
Cntn5	1.006	http://api.brain-map.org/grid_data/download/74578317?include=energy
Hspa8	1.006	http://api.brain-map.org/grid_data/download/75694450?include=energy
Esam	1.006	http://api.brain-map.org/grid_data/download/69262331?include=energy
Irf6	1.006	http://api.brain-map.org/grid_data/download/79539656?include=energy
Ddx4	1.006	http://api.brain-map.org/grid_data/download/70946444?include=energy
Pnkd	1.006	http://api.brain-map.org/grid_data/download/73769326?include=energy
Stip1	1.006	http://api.brain-map.org/grid_data/download/413807?include=energy
Sult5a1	1.005	http://api.brain-map.org/grid_data/download/74579491?include=energy
Cfh	1.005	http://api.brain-map.org/grid_data/download/69169738?include=energy
Ifnar2	1.005	http://api.brain-map.org/grid_data/download/69169301?include=energy
LOC385660	1.005	http://api.brain-map.org/grid_data/download/71211296?include=energy
Rcn2	1.005	http://api.brain-map.org/grid_data/download/73997151?include=energy
Rad51l3	1.005	http://api.brain-map.org/grid_data/download/74821547?include=energy
Wdr6	1.005	http://api.brain-map.org/grid_data/download/77465028?include=energy
Tm4sf20	1.005	http://api.brain-map.org/grid_data/download/73818843?include=energy
Hp1bp3	1.005	http://api.brain-map.org/grid_data/download/81599666?include=energy
Rtkn2	1.005	http://api.brain-map.org/grid_data/download/74513896?include=energy
BC026590	1.005	http://api.brain-map.org/grid_data/download/69529017?include=energy
Ndufs2	1.005	http://api.brain-map.org/grid_data/download/74818622?include=energy
Arx	1.005	http://api.brain-map.org/grid_data/download/118?include=energy
AI646023	1.005	http://api.brain-map.org/grid_data/download/69526390?include=energy
Mrgprb2	1.005	http://api.brain-map.org/grid_data/download/73606516?include=energy
Dgkb	1.005	http://api.brain-map.org/grid_data/download/73787952?include=energy
Sfpq	1.005	http://api.brain-map.org/grid_data/download/69873624?include=energy
Sugt1	1.005	http://api.brain-map.org/grid_data/download/69080005?include=energy
Tanc1	1.005	http://api.brain-map.org/grid_data/download/72081515?include=energy
Cenpf	1.005	http://api.brain-map.org/grid_data/download/1631?include=energy
Eogt	1.005	http://api.brain-map.org/grid_data/download/69013098?include=energy
Foxm1	1.005	http://api.brain-map.org/grid_data/download/77924478?include=energy
Il6ra	1.005	http://api.brain-map.org/grid_data/download/74300711?include=energy
Krtcap3	1.005	http://api.brain-map.org/grid_data/download/68161455?include=energy
LOC433348	1.005	http://api.brain-map.org/grid_data/download/70723161?include=energy
E2f1	1.005	http://api.brain-map.org/grid_data/download/2171?include=energy
Kctd10	1.005	http://api.brain-map.org/grid_data/download/80514006?include=energy
Adal	1.005	http://api.brain-map.org/grid_data/download/69442377?include=energy
9230020A06Rik	1.005	http://api.brain-map.org/grid_data/download/73906173?include=energy
Tnfsf14	1.005	http://api.brain-map.org/grid_data/download/70302569?include=energy
Tjp3	1.005	http://api.brain-map.org/grid_data/download/69095972?include=energy
Abhd3	1.005	http://api.brain-map.org/grid_data/download/70744514?include=energy
Necap1	1.005	http://api.brain-map.org/grid_data/download/69169421?include=energy
Ppa2	1.005	http://api.brain-map.org/grid_data/download/69080009?include=energy
Ndufb9	1.005	http://api.brain-map.org/grid_data/download/68631911?include=energy
Klhl11	1.005	http://api.brain-map.org/grid_data/download/69735855?include=energy
Ssh3	1.005	http://api.brain-map.org/grid_data/download/68637467?include=energy
Gal3st2	1.004	http://api.brain-map.org/grid_data/download/77800905?include=energy
Dalrd3	1.004	http://api.brain-map.org/grid_data/download/69169439?include=energy
LOC212970	1.004	http://api.brain-map.org/grid_data/download/70565927?include=energy
Gkap1	1.004	http://api.brain-map.org/grid_data/download/69116329?include=energy
Pla2g7	1.004	http://api.brain-map.org/grid_data/download/74047841?include=energy
Dnaja3	1.004	http://api.brain-map.org/grid_data/download/68443441?include=energy
Gltscr1	1.004	http://api.brain-map.org/grid_data/download/70566310?include=energy
BC030867	1.004	http://api.brain-map.org/grid_data/download/68498615?include=energy
Nkx3-2	1.004	http://api.brain-map.org/grid_data/download/81790684?include=energy
1700052I22Rik	1.004	http://api.brain-map.org/grid_data/download/74635023?include=energy
Prl7c1	1.004	http://api.brain-map.org/grid_data/download/71832994?include=energy
Pik3ca	1.004	http://api.brain-map.org/grid_data/download/70813341?include=energy
LOC433696	1.004	http://api.brain-map.org/grid_data/download/72471829?include=energy
Rpl17	1.004	http://api.brain-map.org/grid_data/download/69014530?include=energy
LOC432885	1.004	http://api.brain-map.org/grid_data/download/71789969?include=energy
Gabrg3	1.004	http://api.brain-map.org/grid_data/download/71587875?include=energy
LOC433773	1.004	http://api.brain-map.org/grid_data/download/71991935?include=energy
LOC380889	1.004	http://api.brain-map.org/grid_data/download/74988754?include=energy
Eif1	1.004	http://api.brain-map.org/grid_data/download/77791992?include=energy
Tbc1d22a	1.004	http://api.brain-map.org/grid_data/download/68444684?include=energy
Zfp493	1.004	http://api.brain-map.org/grid_data/download/73497086?include=energy
Mpzl1	1.004	http://api.brain-map.org/grid_data/download/77332711?include=energy
4930456L15Rik	1.004	http://api.brain-map.org/grid_data/download/71891778?include=energy
Esco2	1.004	http://api.brain-map.org/grid_data/download/68498259?include=energy
BC052040	1.004	http://api.brain-map.org/grid_data/download/71393322?include=energy
Lpcat4	1.004	http://api.brain-map.org/grid_data/download/73606648?include=energy
A730017C20Rik	1.004	http://api.brain-map.org/grid_data/download/69448948?include=energy
Gclc	1.004	http://api.brain-map.org/grid_data/download/68410303?include=energy
Scoc	1.004	http://api.brain-map.org/grid_data/download/81599674?include=energy
Slc41a1	1.004	http://api.brain-map.org/grid_data/download/69873982?include=energy
Trp53i13	1.004	http://api.brain-map.org/grid_data/download/71991870?include=energy
1700008H02Rik	1.004	http://api.brain-map.org/grid_data/download/71808618?include=energy
Myo3b	1.004	http://api.brain-map.org/grid_data/download/71809247?include=energy
Zfp395	1.004	http://api.brain-map.org/grid_data/download/69835564?include=energy
Tulp1	1.004	http://api.brain-map.org/grid_data/download/69261411?include=energy
Myh14	1.003	http://api.brain-map.org/grid_data/download/100144898?include=energy
1700034J05Rik	1.003	http://api.brain-map.org/grid_data/download/74357893?include=energy
Mm.246905	1.003	http://api.brain-map.org/grid_data/download/71456143?include=energy
Ints1	1.003	http://api.brain-map.org/grid_data/download/68743990?include=energy
Got1	1.003	http://api.brain-map.org/grid_data/download/75695658?include=energy
Zfp276	1.003	http://api.brain-map.org/grid_data/download/68798537?include=energy
9330104G04Rik	1.003	http://api.brain-map.org/grid_data/download/71493855?include=energy
Nkx2-6	1.003	http://api.brain-map.org/grid_data/download/77932777?include=energy
Plek	1.003	http://api.brain-map.org/grid_data/download/75084080?include=energy
Pdzd2	1.003	http://api.brain-map.org/grid_data/download/74988286?include=energy
Wdr95	1.003	http://api.brain-map.org/grid_data/download/69407977?include=energy
Hirip3	1.003	http://api.brain-map.org/grid_data/download/69837911?include=energy
Thbs1	1.003	http://api.brain-map.org/grid_data/download/80473473?include=energy
Zcchc24	1.003	http://api.brain-map.org/grid_data/download/69202490?include=energy
Tonsl	1.003	http://api.brain-map.org/grid_data/download/69407284?include=energy
Gbx1	1.003	http://api.brain-map.org/grid_data/download/81654137?include=energy
1300010F03Rik	1.003	http://api.brain-map.org/grid_data/download/70200920?include=energy
Klhl7	1.003	http://api.brain-map.org/grid_data/download/69059850?include=energy
Rab6b	1.003	http://api.brain-map.org/grid_data/download/69817886?include=energy
2700029M09Rik	1.003	http://api.brain-map.org/grid_data/download/75990648?include=energy
Msl1	1.003	http://api.brain-map.org/grid_data/download/71656894?include=energy
Rhou	1.003	http://api.brain-map.org/grid_data/download/69169475?include=energy
Cngb1	1.003	http://api.brain-map.org/grid_data/download/69568782?include=energy
Caskin2	1.003	http://api.brain-map.org/grid_data/download/68744444?include=energy
Map6d1	1.003	http://api.brain-map.org/grid_data/download/70565891?include=energy
Gdpd2	1.003	http://api.brain-map.org/grid_data/download/74047789?include=energy
Rtel1	1.003	http://api.brain-map.org/grid_data/download/71495843?include=energy
Lypd6	1.003	http://api.brain-map.org/grid_data/download/77332684?include=energy
Hspa9	1.003	http://api.brain-map.org/grid_data/download/69735144?include=energy
Ckb	1.003	http://api.brain-map.org/grid_data/download/70927826?include=energy
4930515B02Rik	1.003	http://api.brain-map.org/grid_data/download/72076659?include=energy
Siah1b	1.003	http://api.brain-map.org/grid_data/download/68667437?include=energy
Homez	1.003	http://api.brain-map.org/grid_data/download/70303594?include=energy
6330439K17Rik	1.003	http://api.brain-map.org/grid_data/download/71808860?include=energy
Dennd2c	1.003	http://api.brain-map.org/grid_data/download/69514422?include=energy
Gm16515	1.003	http://api.brain-map.org/grid_data/download/68080694?include=energy
C530028O21Rik	1.003	http://api.brain-map.org/grid_data/download/69531043?include=energy
D5Ertd577e	1.003	http://api.brain-map.org/grid_data/download/73818828?include=energy
Blm	1.003	http://api.brain-map.org/grid_data/download/69529334?include=energy
Ndn	1.002	http://api.brain-map.org/grid_data/download/688?include=energy
Csrp2	1.002	http://api.brain-map.org/grid_data/download/73513654?include=energy
Gm1043	1.002	http://api.brain-map.org/grid_data/download/70299923?include=energy
Ccdc6	1.002	http://api.brain-map.org/grid_data/download/77620271?include=energy
Tlr6	1.002	http://api.brain-map.org/grid_data/download/68668072?include=energy
TC1414896	1.002	http://api.brain-map.org/grid_data/download/70795202?include=energy
Myo15b	1.002	http://api.brain-map.org/grid_data/download/72076545?include=energy
Fabp5	1.002	http://api.brain-map.org/grid_data/download/70634396?include=energy
Fam114a1	1.002	http://api.brain-map.org/grid_data/download/67767473?include=energy
Pnma3	1.002	http://api.brain-map.org/grid_data/download/71763553?include=energy
Mansc1	1.002	http://api.brain-map.org/grid_data/download/53?include=energy
Gm5427	1.002	http://api.brain-map.org/grid_data/download/72076539?include=energy
Ankmy1	1.002	http://api.brain-map.org/grid_data/download/71574401?include=energy
Daam1	1.002	http://api.brain-map.org/grid_data/download/75197516?include=energy
Sowahd	1.002	http://api.brain-map.org/grid_data/download/71809307?include=energy
Gapdh	1.002	http://api.brain-map.org/grid_data/download/70276861?include=energy
Cstf2	1.002	http://api.brain-map.org/grid_data/download/2131?include=energy
Snrnp48	1.002	http://api.brain-map.org/grid_data/download/532763?include=energy
1600012H06Rik	1.002	http://api.brain-map.org/grid_data/download/69353306?include=energy
Sord	1.002	http://api.brain-map.org/grid_data/download/69549661?include=energy
2310046O06Rik	1.002	http://api.brain-map.org/grid_data/download/73679122?include=energy
D630003M21Rik	1.002	http://api.brain-map.org/grid_data/download/75990492?include=energy
Nars	1.002	http://api.brain-map.org/grid_data/download/68667829?include=energy
Rnf130	1.002	http://api.brain-map.org/grid_data/download/68637868?include=energy
Efcab1	1.002	http://api.brain-map.org/grid_data/download/69443403?include=energy
Pold2	1.002	http://api.brain-map.org/grid_data/download/67979050?include=energy
Cx3cl1	1.002	http://api.brain-map.org/grid_data/download/2135?include=energy
Crlf2	1.002	http://api.brain-map.org/grid_data/download/1882?include=energy
Med28	1.002	http://api.brain-map.org/grid_data/download/69095943?include=energy
Prcc	1.002	http://api.brain-map.org/grid_data/download/68799143?include=energy
Cdk4	1.002	http://api.brain-map.org/grid_data/download/79677345?include=energy
Tuba3b	1.002	http://api.brain-map.org/grid_data/download/69114334?include=energy
Ctxn2	1.002	http://api.brain-map.org/grid_data/download/74000672?include=energy
Glt25d2	1.002	http://api.brain-map.org/grid_data/download/69524527?include=energy
Mtap1a	1.002	http://api.brain-map.org/grid_data/download/76115734?include=energy
Calcr	1.002	http://api.brain-map.org/grid_data/download/75080999?include=energy
Stoml2	1.002	http://api.brain-map.org/grid_data/download/74581379?include=energy
Kifc3	1.002	http://api.brain-map.org/grid_data/download/71717081?include=energy
Gars	1.002	http://api.brain-map.org/grid_data/download/68410297?include=energy
Cgnl1	1.002	http://api.brain-map.org/grid_data/download/72008123?include=energy
Tmem59	1.002	http://api.brain-map.org/grid_data/download/69015793?include=energy
Atp13a5	1.002	http://api.brain-map.org/grid_data/download/74658053?include=energy
Keap1	1.002	http://api.brain-map.org/grid_data/download/75831721?include=energy
Nek11	1.002	http://api.brain-map.org/grid_data/download/69782180?include=energy
Mgat5b	1.002	http://api.brain-map.org/grid_data/download/73616036?include=energy
Mfsd7c	1.002	http://api.brain-map.org/grid_data/download/68301963?include=energy
Cox5a	1.002	http://api.brain-map.org/grid_data/download/70445676?include=energy
Rcbtb1	1.002	http://api.brain-map.org/grid_data/download/69289787?include=energy
Gp49a	1.002	http://api.brain-map.org/grid_data/download/69734512?include=energy
9230106D20Rik	1.002	http://api.brain-map.org/grid_data/download/71809013?include=energy
Copg	1.002	http://api.brain-map.org/grid_data/download/67779873?include=energy
Idh3g	1.002	http://api.brain-map.org/grid_data/download/81600144?include=energy
Pdgfa	1.002	http://api.brain-map.org/grid_data/download/1516?include=energy
Bcap31	1.002	http://api.brain-map.org/grid_data/download/79544798?include=energy
Vps37a	1.002	http://api.brain-map.org/grid_data/download/69170264?include=energy
Dnajc11	1.002	http://api.brain-map.org/grid_data/download/71496459?include=energy
Pef1	1.002	http://api.brain-map.org/grid_data/download/545095?include=energy
Dnm3os	1.002	http://api.brain-map.org/grid_data/download/73617462?include=energy
Eif4a2	1.001	http://api.brain-map.org/grid_data/download/71381013?include=energy
Tpbpb	1.001	http://api.brain-map.org/grid_data/download/70785206?include=energy
Edf1	1.001	http://api.brain-map.org/grid_data/download/68117986?include=energy
Cir1	1.001	http://api.brain-map.org/grid_data/download/69353633?include=energy
Fdps	1.001	http://api.brain-map.org/grid_data/download/76085745?include=energy
C2cd3	1.001	http://api.brain-map.org/grid_data/download/69527030?include=energy
5730528L13Rik	1.001	http://api.brain-map.org/grid_data/download/70946197?include=energy
Spag8	1.001	http://api.brain-map.org/grid_data/download/74514067?include=energy
Angptl6	1.001	http://api.brain-map.org/grid_data/download/74580835?include=energy
Gtf3c1	1.001	http://api.brain-map.org/grid_data/download/69734809?include=energy
Ubl3	1.001	http://api.brain-map.org/grid_data/download/1884?include=energy
Zfa	1.001	http://api.brain-map.org/grid_data/download/69817109?include=energy
Klf2	1.001	http://api.brain-map.org/grid_data/download/74635803?include=energy
Cd248	1.001	http://api.brain-map.org/grid_data/download/68745510?include=energy
Kirrel	1.001	http://api.brain-map.org/grid_data/download/71613657?include=energy
S100a8	1.001	http://api.brain-map.org/grid_data/download/73573151?include=energy
Hvcn1	1.001	http://api.brain-map.org/grid_data/download/70445371?include=energy
Cct7	1.001	http://api.brain-map.org/grid_data/download/413791?include=energy
Ap4b1	1.001	http://api.brain-map.org/grid_data/download/70230951?include=energy
Clic5	1.001	http://api.brain-map.org/grid_data/download/73636094?include=energy
Anks4b	1.001	http://api.brain-map.org/grid_data/download/71280889?include=energy
Vsx1	1.001	http://api.brain-map.org/grid_data/download/70445299?include=energy
Snrpd2	1.001	http://api.brain-map.org/grid_data/download/68744002?include=energy
Cdk5r2	1.001	http://api.brain-map.org/grid_data/download/70812811?include=energy
Pycr2	1.001	http://api.brain-map.org/grid_data/download/77923131?include=energy
Amfr	1.001	http://api.brain-map.org/grid_data/download/68546524?include=energy
Cyp4f17	1.001	http://api.brain-map.org/grid_data/download/70724965?include=energy
Cox6a2	1.001	http://api.brain-map.org/grid_data/download/71836876?include=energy
Fam83d	1.001	http://api.brain-map.org/grid_data/download/69202352?include=energy
1700026D08Rik	1.001	http://api.brain-map.org/grid_data/download/69116727?include=energy
Olfr71	1.001	http://api.brain-map.org/grid_data/download/74734872?include=energy
Dmp1	1.001	http://api.brain-map.org/grid_data/download/74511936?include=energy
Cd97	1.001	http://api.brain-map.org/grid_data/download/112646460?include=energy
Gm5531	1.001	http://api.brain-map.org/grid_data/download/74743471?include=energy
Tmem67	1.001	http://api.brain-map.org/grid_data/download/69443292?include=energy
Svs2	1.001	http://api.brain-map.org/grid_data/download/69887493?include=energy
Rsph4a	1.001	http://api.brain-map.org/grid_data/download/69873331?include=energy
Aptx	1.001	http://api.brain-map.org/grid_data/download/69169630?include=energy
Psme2b-ps	1.001	http://api.brain-map.org/grid_data/download/75990413?include=energy
Rgs5	1.001	http://api.brain-map.org/grid_data/download/74357571?include=energy
Ptcd1	1.001	http://api.brain-map.org/grid_data/download/74882764?include=energy
Ptgr2	1.001	http://api.brain-map.org/grid_data/download/76098389?include=energy
Tspyl4	1.001	http://api.brain-map.org/grid_data/download/67936212?include=energy
Epas1	1.001	http://api.brain-map.org/grid_data/download/69015278?include=energy
Ifitm6	1.001	http://api.brain-map.org/grid_data/download/73617672?include=energy
Hsf5	1	http://api.brain-map.org/grid_data/download/74578236?include=energy
Nedd8	1	http://api.brain-map.org/grid_data/download/393259?include=energy
Sgms1	1	http://api.brain-map.org/grid_data/download/77910765?include=energy
Ctsll3	1	http://api.brain-map.org/grid_data/download/71991816?include=energy
Itgb1	1	http://api.brain-map.org/grid_data/download/76115730?include=energy
Sucla2	1	http://api.brain-map.org/grid_data/download/69887439?include=energy
Sco1	1	http://api.brain-map.org/grid_data/download/73635395?include=energy
Nudc	1	http://api.brain-map.org/grid_data/download/2442?include=energy
2410091C18Rik	1	http://api.brain-map.org/grid_data/download/70527963?include=energy
Lrrfip2	1	http://api.brain-map.org/grid_data/download/68666732?include=energy
I830007O11Rik*	1	http://api.brain-map.org/grid_data/download/73767235?include=energy
Tfpt	1	http://api.brain-map.org/grid_data/download/69170270?include=energy
9530085C10Rik*	1	http://api.brain-map.org/grid_data/download/75080269?include=energy

Gpihbp1	1	http://api.brain-map.org/grid_data/download/69117122?include=energy
Mogs	1	http://api.brain-map.org/grid_data/download/69837471?include=energy
Clpx	1	http://api.brain-map.org/grid_data/download/69116697?include=energy
Coro6	1	http://api.brain-map.org/grid_data/download/77280432?include=energy
Cbx8	1	http://api.brain-map.org/grid_data/download/69838623?include=energy
Ccbe1	1	http://api.brain-map.org/grid_data/download/74513995?include=energy
Vmn1r235	1	http://api.brain-map.org/grid_data/download/74056214?include=energy
Cul2	1	http://api.brain-map.org/grid_data/download/68203492?include=energy
Gpld1	1	http://api.brain-map.org/grid_data/download/74509585?include=energy
Bmpr2	1	http://api.brain-map.org/grid_data/download/69529382?include=energy
Rbm25	1	http://api.brain-map.org/grid_data/download/68341250?include=energy
Slc25a11	1	http://api.brain-map.org/grid_data/download/81599614?include=energy
G630027G22Rik*	1	http://api.brain-map.org/grid_data/download/73731271?include=energy
BC040756	1	http://api.brain-map.org/grid_data/download/71212590?include=energy
Rgs10	1	http://api.brain-map.org/grid_data/download/74511849?include=energy
Ggct	1	http://api.brain-map.org/grid_data/download/69447894?include=energy
Cks2	1	http://api.brain-map.org/grid_data/download/74512038?include=energy
1700008F21Rik	1	http://api.brain-map.org/grid_data/download/69116910?include=energy
Dcn	1	http://api.brain-map.org/grid_data/download/329?include=energy
Litaf	1	http://api.brain-map.org/grid_data/download/68269586?include=energy
Pcp4l1	1	http://api.brain-map.org/grid_data/download/75084520?include=energy
Aifm1	1	http://api.brain-map.org/grid_data/download/513245?include=energy
Pde6c	1	http://api.brain-map.org/grid_data/download/70546341?include=energy
Olfr648	1	http://api.brain-map.org/grid_data/download/73653476?include=energy
Tgm3	0.999	http://api.brain-map.org/grid_data/download/69980322?include=energy
Higd1b	0.999	http://api.brain-map.org/grid_data/download/71616335?include=energy
Frs3	0.999	http://api.brain-map.org/grid_data/download/2234?include=energy
Rpf2	0.999	http://api.brain-map.org/grid_data/download/293465?include=energy
Olr1	0.999	http://api.brain-map.org/grid_data/download/71920458?include=energy
Dpysl2	0.999	http://api.brain-map.org/grid_data/download/74510104?include=energy
Leprel4	0.999	http://api.brain-map.org/grid_data/download/2547?include=energy
Dand5	0.999	http://api.brain-map.org/grid_data/download/71281852?include=energy
Sgcz	0.999	http://api.brain-map.org/grid_data/download/74749942?include=energy
TC1442825	0.999	http://api.brain-map.org/grid_data/download/70795485?include=energy
Pacrg	0.999	http://api.brain-map.org/grid_data/download/74743258?include=energy
Fam187a	0.999	http://api.brain-map.org/grid_data/download/74821788?include=energy
2310067B10Rik	0.999	http://api.brain-map.org/grid_data/download/71656916?include=energy
E130303B06Rik	0.999	http://api.brain-map.org/grid_data/download/68744080?include=energy
Exosc3	0.999	http://api.brain-map.org/grid_data/download/68744026?include=energy
Pank1	0.999	http://api.brain-map.org/grid_data/download/68441526?include=energy
Il20ra	0.999	http://api.brain-map.org/grid_data/download/69015200?include=energy
Rhbdd2	0.999	http://api.brain-map.org/grid_data/download/68301682?include=energy
Wnt8a	0.999	http://api.brain-map.org/grid_data/download/1115?include=energy
Prkcz	0.999	http://api.brain-map.org/grid_data/download/841?include=energy
Zbtb49	0.999	http://api.brain-map.org/grid_data/download/74509506?include=energy
Olfr543	0.999	http://api.brain-map.org/grid_data/download/74735027?include=energy
4933434C23Rik	0.999	http://api.brain-map.org/grid_data/download/71892191?include=energy
Gsto1	0.999	http://api.brain-map.org/grid_data/download/74511807?include=energy
Tcea1	0.999	http://api.brain-map.org/grid_data/download/227534?include=energy
Atp2b3	0.999	http://api.brain-map.org/grid_data/download/71281325?include=energy
Tubb2a	0.999	http://api.brain-map.org/grid_data/download/74819689?include=energy
Ndufa4	0.999	http://api.brain-map.org/grid_data/download/69782139?include=energy
Fibp	0.999	http://api.brain-map.org/grid_data/download/68633279?include=energy
4933435E02Rik	0.999	http://api.brain-map.org/grid_data/download/71892197?include=energy
Slc9a1	0.999	http://api.brain-map.org/grid_data/download/72080207?include=energy
Tatdn2	0.999	http://api.brain-map.org/grid_data/download/71764518?include=energy
5330426L24Rik	0.999	http://api.brain-map.org/grid_data/download/69443373?include=energy
Ddx17	0.999	http://api.brain-map.org/grid_data/download/77620754?include=energy
Arhgef4	0.999	http://api.brain-map.org/grid_data/download/308054630?include=energy
Zfp352	0.999	http://api.brain-map.org/grid_data/download/70785732?include=energy
Cst13	0.999	http://api.brain-map.org/grid_data/download/75860943?include=energy
Triobp	0.999	http://api.brain-map.org/grid_data/download/69262263?include=energy
Akap3	0.999	http://api.brain-map.org/grid_data/download/76080980?include=energy
C230029F24Rik	0.999	http://api.brain-map.org/grid_data/download/71808576?include=energy
Mccc2	0.999	http://api.brain-map.org/grid_data/download/68544973?include=energy
Pcyt2	0.999	http://api.brain-map.org/grid_data/download/69095961?include=energy
Tspan7	0.999	http://api.brain-map.org/grid_data/download/72718?include=energy
Hnrnpl	0.999	http://api.brain-map.org/grid_data/download/532678?include=energy
Ptprcap	0.999	http://api.brain-map.org/grid_data/download/121091?include=energy
P4hb	0.999	http://api.brain-map.org/grid_data/download/74658245?include=energy
Mrpl22	0.999	http://api.brain-map.org/grid_data/download/69116909?include=energy
Madd	0.999	http://api.brain-map.org/grid_data/download/69236700?include=energy
1110004E09Rik	0.999	http://api.brain-map.org/grid_data/download/68342003?include=energy
1700019E19Rik	0.999	http://api.brain-map.org/grid_data/download/68920773?include=energy
Wdr78	0.998	http://api.brain-map.org/grid_data/download/68498717?include=energy
Podxl2	0.998	http://api.brain-map.org/grid_data/download/74047921?include=energy
Rgs13	0.998	http://api.brain-map.org/grid_data/download/74426037?include=energy
Rbm33	0.998	http://api.brain-map.org/grid_data/download/69444915?include=energy
Oprl1	0.998	http://api.brain-map.org/grid_data/download/75077232?include=energy
Mettl4	0.998	http://api.brain-map.org/grid_data/download/73584765?include=energy
Rpgrip1	0.998	http://api.brain-map.org/grid_data/download/69169540?include=energy
A230050P20Rik	0.998	http://api.brain-map.org/grid_data/download/68920737?include=energy
Stxbp4	0.998	http://api.brain-map.org/grid_data/download/69261410?include=energy
Ncs1	0.998	http://api.brain-map.org/grid_data/download/77332082?include=energy
Fxn	0.998	http://api.brain-map.org/grid_data/download/69672575?include=energy
Reep3	0.998	http://api.brain-map.org/grid_data/download/633686?include=energy
Dock4	0.998	http://api.brain-map.org/grid_data/download/70238931?include=energy
Psap	0.998	http://api.brain-map.org/grid_data/download/846?include=energy
mCG1027388	0.998	http://api.brain-map.org/grid_data/download/71022816?include=energy
I830077J02Rik	0.998	http://api.brain-map.org/grid_data/download/71456124?include=energy
Slbp	0.998	http://api.brain-map.org/grid_data/download/68546434?include=energy
Alpl	0.998	http://api.brain-map.org/grid_data/download/100145313?include=energy
Cycs	0.998	http://api.brain-map.org/grid_data/download/68546299?include=energy
Acer2	0.998	http://api.brain-map.org/grid_data/download/71281021?include=energy
Hspa5	0.998	http://api.brain-map.org/grid_data/download/69735138?include=energy
1700021F07Rik	0.998	http://api.brain-map.org/grid_data/download/74357845?include=energy
Nsun5	0.998	http://api.brain-map.org/grid_data/download/71764589?include=energy
Ndufc2	0.998	http://api.brain-map.org/grid_data/download/81599620?include=energy
LOC544975	0.998	http://api.brain-map.org/grid_data/download/75144616?include=energy
Renbp	0.998	http://api.brain-map.org/grid_data/download/69028264?include=energy
Lmo7	0.998	http://api.brain-map.org/grid_data/download/71920429?include=energy
Bpifb1	0.998	http://api.brain-map.org/grid_data/download/70194462?include=energy
Mrps15	0.998	http://api.brain-map.org/grid_data/download/68545366?include=energy
Rtn4	0.998	http://api.brain-map.org/grid_data/download/70805906?include=energy
Idh3b	0.998	http://api.brain-map.org/grid_data/download/81600142?include=energy
Chd3	0.998	http://api.brain-map.org/grid_data/download/385790?include=energy
Pik3r2	0.998	http://api.brain-map.org/grid_data/download/512986?include=energy
C330006K01Rik	0.998	http://api.brain-map.org/grid_data/download/70231923?include=energy
Arhgef10l	0.998	http://api.brain-map.org/grid_data/download/75831723?include=energy
Pyroxd2	0.998	http://api.brain-map.org/grid_data/download/71493165?include=energy
1810026B05Rik	0.998	http://api.brain-map.org/grid_data/download/74635174?include=energy
Akt1	0.998	http://api.brain-map.org/grid_data/download/75988255?include=energy
BC057079	0.998	http://api.brain-map.org/grid_data/download/74881879?include=energy
Thsd1	0.998	http://api.brain-map.org/grid_data/download/72004434?include=energy
Kcnh8	0.998	http://api.brain-map.org/grid_data/download/70562113?include=energy
Rundc3b	0.998	http://api.brain-map.org/grid_data/download/71656696?include=energy
Tbc1d10c	0.998	http://api.brain-map.org/grid_data/download/69201678?include=energy
Angptl2	0.998	http://api.brain-map.org/grid_data/download/69526635?include=energy
Zfp36l3	0.998	http://api.brain-map.org/grid_data/download/73513486?include=energy
Rps20	0.998	http://api.brain-map.org/grid_data/download/69873289?include=energy
Armc3	0.998	http://api.brain-map.org/grid_data/download/69526880?include=energy
Sept12	0.997	http://api.brain-map.org/grid_data/download/71892113?include=energy
Prss37	0.997	http://api.brain-map.org/grid_data/download/71919397?include=energy
Il13ra2	0.997	http://api.brain-map.org/grid_data/download/79544794?include=energy
Hnrnph1	0.997	http://api.brain-map.org/grid_data/download/69014464?include=energy
Lrrc3b	0.997	http://api.brain-map.org/grid_data/download/74881145?include=energy
Hnrnpm	0.997	http://api.brain-map.org/grid_data/download/69735072?include=energy
1300017J02Rik	0.997	http://api.brain-map.org/grid_data/download/75828169?include=energy
Prdm9	0.997	http://api.brain-map.org/grid_data/download/67765700?include=energy
Rab24	0.997	http://api.brain-map.org/grid_data/download/68341842?include=energy
Cyhr1	0.997	http://api.brain-map.org/grid_data/download/77414565?include=energy
4930458L03Rik	0.997	http://api.brain-map.org/grid_data/download/73513263?include=energy
Dgat2	0.997	http://api.brain-map.org/grid_data/download/72077467?include=energy
Rmnd5a	0.997	http://api.brain-map.org/grid_data/download/75831719?include=energy
Cep350	0.997	http://api.brain-map.org/grid_data/download/70218673?include=energy
Uba3	0.997	http://api.brain-map.org/grid_data/download/413804?include=energy
Ttc22	0.997	http://api.brain-map.org/grid_data/download/70205566?include=energy
2610316D01Rik	0.997	http://api.brain-map.org/grid_data/download/70725543?include=energy
Hspa4l	0.997	http://api.brain-map.org/grid_data/download/75749390?include=energy
Rmi1	0.997	http://api.brain-map.org/grid_data/download/378551?include=energy
Acox1	0.997	http://api.brain-map.org/grid_data/download/68844450?include=energy
Lgmn	0.997	http://api.brain-map.org/grid_data/download/69736118?include=energy
Spanxn4	0.997	http://api.brain-map.org/grid_data/download/74635041?include=energy
Rasgrf1	0.997	http://api.brain-map.org/grid_data/download/1274?include=energy
Nox4	0.997	http://api.brain-map.org/grid_data/download/68446069?include=energy
Cnr1	0.997	http://api.brain-map.org/grid_data/download/283?include=energy
Amy2a5	0.997	http://api.brain-map.org/grid_data/download/69526611?include=energy
Skap2	0.997	http://api.brain-map.org/grid_data/download/512776?include=energy
Gm16517	0.997	http://api.brain-map.org/grid_data/download/70724768?include=energy
Scgb3a2	0.997	http://api.brain-map.org/grid_data/download/73424208?include=energy
Zcchc11	0.997	http://api.brain-map.org/grid_data/download/74277333?include=energy
4933400C23	0.997	http://api.brain-map.org/grid_data/download/72006000?include=energy
Pdcd11	0.997	http://api.brain-map.org/grid_data/download/69133749?include=energy
Acly	0.997	http://api.brain-map.org/grid_data/download/77620447?include=energy
1110031I02Rik	0.997	http://api.brain-map.org/grid_data/download/69170306?include=energy
Got2	0.997	http://api.brain-map.org/grid_data/download/76115728?include=energy
Spna2	0.997	http://api.brain-map.org/grid_data/download/69885690?include=energy
Ogdh	0.997	http://api.brain-map.org/grid_data/download/68193022?include=energy
2610507B11Rik	0.997	http://api.brain-map.org/grid_data/download/69202932?include=energy
Slc22a3	0.997	http://api.brain-map.org/grid_data/download/73931644?include=energy
Ccdc109a	0.997	http://api.brain-map.org/grid_data/download/72472776?include=energy
Ndufv1	0.997	http://api.brain-map.org/grid_data/download/69015572?include=energy
Sncaip	0.997	http://api.brain-map.org/grid_data/download/76081913?include=energy
Olfr1018	0.997	http://api.brain-map.org/grid_data/download/74720543?include=energy
Arhgef5	0.997	http://api.brain-map.org/grid_data/download/74881537?include=energy
Tnfsf10	0.997	http://api.brain-map.org/grid_data/download/69981163?include=energy
Ano1	0.997	http://api.brain-map.org/grid_data/download/1472?include=energy
Ppp1r26	0.997	http://api.brain-map.org/grid_data/download/73497581?include=energy
Kars	0.997	http://api.brain-map.org/grid_data/download/68546272?include=energy
Clk2	0.997	http://api.brain-map.org/grid_data/download/356286?include=energy
Tspan9	0.997	http://api.brain-map.org/grid_data/download/68744056?include=energy
Muted	0.997	http://api.brain-map.org/grid_data/download/71763816?include=energy
Zfp353	0.997	http://api.brain-map.org/grid_data/download/71808540?include=energy
Cct5	0.997	http://api.brain-map.org/grid_data/download/69540549?include=energy
Abca5	0.996	http://api.brain-map.org/grid_data/download/69514542?include=energy
Gm5084	0.996	http://api.brain-map.org/grid_data/download/71569145?include=energy
Mrgprh	0.996	http://api.brain-map.org/grid_data/download/69258172?include=energy
Pigc	0.996	http://api.brain-map.org/grid_data/download/413816?include=energy
Opn1mw	0.996	http://api.brain-map.org/grid_data/download/79360270?include=energy
Ywhaz	0.996	http://api.brain-map.org/grid_data/download/1133?include=energy
Mm.39446	0.996	http://api.brain-map.org/grid_data/download/71210217?include=energy
Cacybp	0.996	http://api.brain-map.org/grid_data/download/75144703?include=energy
Zrsr1	0.996	http://api.brain-map.org/grid_data/download/72004453?include=energy
Inpp5b	0.996	http://api.brain-map.org/grid_data/download/69015530?include=energy
Idnk	0.996	http://api.brain-map.org/grid_data/download/68666517?include=energy
Il7	0.996	http://api.brain-map.org/grid_data/download/74513989?include=energy
Jak1	0.996	http://api.brain-map.org/grid_data/download/74581365?include=energy
LOC434129	0.996	http://api.brain-map.org/grid_data/download/70787432?include=energy
Gpam	0.996	http://api.brain-map.org/grid_data/download/68193048?include=energy
Kif22	0.996	http://api.brain-map.org/grid_data/download/72726?include=energy
5830454E08Rik	0.996	http://api.brain-map.org/grid_data/download/69444278?include=energy
6030419C18Rik	0.996	http://api.brain-map.org/grid_data/download/73497059?include=energy
Pld5	0.996	http://api.brain-map.org/grid_data/download/77280438?include=energy
Entpd7	0.996	http://api.brain-map.org/grid_data/download/69672054?include=energy
Gnpat	0.996	http://api.brain-map.org/grid_data/download/68562982?include=energy
LOC435143	0.996	http://api.brain-map.org/grid_data/download/70725621?include=energy
Plcxd1	0.996	http://api.brain-map.org/grid_data/download/69170246?include=energy
Atg5	0.996	http://api.brain-map.org/grid_data/download/77792004?include=energy
Gnb2	0.996	http://api.brain-map.org/grid_data/download/512?include=energy
LOC432930	0.996	http://api.brain-map.org/grid_data/download/70611401?include=energy
Prss33	0.996	http://api.brain-map.org/grid_data/download/73514851?include=energy
Emx1	0.996	http://api.brain-map.org/grid_data/download/100145374?include=energy
Nfat5	0.996	http://api.brain-map.org/grid_data/download/73496959?include=energy
Prep	0.996	http://api.brain-map.org/grid_data/download/69059821?include=energy
Rabac1	0.996	http://api.brain-map.org/grid_data/download/75750398?include=energy
Ppp2r3d	0.996	http://api.brain-map.org/grid_data/download/77414925?include=energy
Fam73b	0.996	http://api.brain-map.org/grid_data/download/69013326?include=energy
Prdx5	0.996	http://api.brain-map.org/grid_data/download/76115740?include=energy
Spcs2	0.995	http://api.brain-map.org/grid_data/download/68667312?include=energy
Ctns	0.995	http://api.brain-map.org/grid_data/download/275689?include=energy
Ptgfrn	0.995	http://api.brain-map.org/grid_data/download/69817643?include=energy
Rab3gap2	0.995	http://api.brain-map.org/grid_data/download/69015302?include=energy
Ywhae	0.995	http://api.brain-map.org/grid_data/download/2739?include=energy
Ncaph2	0.995	http://api.brain-map.org/grid_data/download/355887?include=energy
Fasn	0.995	http://api.brain-map.org/grid_data/download/74425543?include=energy
B4galt6	0.995	http://api.brain-map.org/grid_data/download/2055?include=energy
Sowaha	0.995	http://api.brain-map.org/grid_data/download/71250306?include=energy
Srrm1	0.995	http://api.brain-map.org/grid_data/download/69885756?include=energy
Lmo4	0.995	http://api.brain-map.org/grid_data/download/2385?include=energy
Ncl	0.995	http://api.brain-map.org/grid_data/download/68442979?include=energy
Slc43a2	0.995	http://api.brain-map.org/grid_data/download/74425544?include=energy
Elovl6	0.995	http://api.brain-map.org/grid_data/download/74425551?include=energy
A1bg	0.995	http://api.brain-map.org/grid_data/download/100144171?include=energy
Mrps31	0.995	http://api.brain-map.org/grid_data/download/68341639?include=energy
Rfxank	0.995	http://api.brain-map.org/grid_data/download/67881545?include=energy
Zfp644	0.995	http://api.brain-map.org/grid_data/download/68498546?include=energy
Cib1	0.995	http://api.brain-map.org/grid_data/download/68191564?include=energy
Tcp10a	0.995	http://api.brain-map.org/grid_data/download/69980220?include=energy
Apoe	0.995	http://api.brain-map.org/grid_data/download/79490110?include=energy
Atn1	0.995	http://api.brain-map.org/grid_data/download/68911027?include=energy
Eral1	0.995	http://api.brain-map.org/grid_data/download/69672120?include=energy
Camk2g	0.995	http://api.brain-map.org/grid_data/download/189?include=energy
Tas2r121	0.995	http://api.brain-map.org/grid_data/download/70785170?include=energy
Gabarap	0.995	http://api.brain-map.org/grid_data/download/2245?include=energy
Ccne1	0.995	http://api.brain-map.org/grid_data/download/69540513?include=energy
5730416O20Rik	0.995	http://api.brain-map.org/grid_data/download/74265321?include=energy
Fahd2a	0.995	http://api.brain-map.org/grid_data/download/556986?include=energy
Tceal7	0.995	http://api.brain-map.org/grid_data/download/71492760?include=energy
Abcf2	0.995	http://api.brain-map.org/grid_data/download/75695665?include=energy
Tbxas1	0.995	http://api.brain-map.org/grid_data/download/68410644?include=energy
Ip6k2	0.995	http://api.brain-map.org/grid_data/download/69170240?include=energy
Usf1	0.995	http://api.brain-map.org/grid_data/download/77454705?include=energy
Zfp219	0.995	http://api.brain-map.org/grid_data/download/69290818?include=energy
D9Ertd402e	0.995	http://api.brain-map.org/grid_data/download/70300007?include=energy
Tmem154	0.995	http://api.brain-map.org/grid_data/download/69447888?include=energy
Tmem25	0.995	http://api.brain-map.org/grid_data/download/71670700?include=energy
Myeov2	0.995	http://api.brain-map.org/grid_data/download/74581062?include=energy
LOC545421	0.995	http://api.brain-map.org/grid_data/download/74736438?include=energy
Wdr44	0.995	http://api.brain-map.org/grid_data/download/70542889?include=energy
Tmprss11a	0.995	http://api.brain-map.org/grid_data/download/75931610?include=energy
Nhsl1	0.995	http://api.brain-map.org/grid_data/download/74265315?include=energy
Pgk1	0.995	http://api.brain-map.org/grid_data/download/69783047?include=energy
Ywhag	0.995	http://api.brain-map.org/grid_data/download/1124?include=energy
Adssl1	0.995	http://api.brain-map.org/grid_data/download/72104185?include=energy
Parp2	0.995	http://api.brain-map.org/grid_data/download/69835612?include=energy
Nop56	0.995	http://api.brain-map.org/grid_data/download/68193039?include=energy
Gm485	0.995	http://api.brain-map.org/grid_data/download/70295578?include=energy
Pira1	0.995	http://api.brain-map.org/grid_data/download/69783220?include=energy
Wsb1	0.995	http://api.brain-map.org/grid_data/download/68666589?include=energy
Slc22a22	0.995	http://api.brain-map.org/grid_data/download/69261398?include=energy
Swap70	0.995	http://api.brain-map.org/grid_data/download/71393274?include=energy
Cstl1	0.995	http://api.brain-map.org/grid_data/download/69114298?include=energy
LOC432948	0.994	http://api.brain-map.org/grid_data/download/70725525?include=energy
Dbf4	0.994	http://api.brain-map.org/grid_data/download/70430790?include=energy
Cyp2d9	0.994	http://api.brain-map.org/grid_data/download/77620818?include=energy
Mefv	0.994	http://api.brain-map.org/grid_data/download/73423774?include=energy
Ccdc89	0.994	http://api.brain-map.org/grid_data/download/69353572?include=energy

Pga5	0.994	http://api.brain-map.org/grid_data/download/75931496?include=energy
LOC432740	0.994	http://api.brain-map.org/grid_data/download/70611246?include=energy
Abcg8	0.994	http://api.brain-map.org/grid_data/download/71569286?include=energy
Cited2	0.994	http://api.brain-map.org/grid_data/download/81790672?include=energy
Pias2	0.994	http://api.brain-map.org/grid_data/download/356088?include=energy
C630007G15Rik*	0.994	http://api.brain-map.org/grid_data/download/73906370?include=energy
Nt5c3	0.994	http://api.brain-map.org/grid_data/download/69059851?include=energy
LOC66376	0.994	http://api.brain-map.org/grid_data/download/293468?include=energy
Mm.273113	0.994	http://api.brain-map.org/grid_data/download/72182978?include=energy
Atp6ap2	0.994	http://api.brain-map.org/grid_data/download/69015578?include=energy
Fam151b	0.994	http://api.brain-map.org/grid_data/download/74426888?include=energy
Ppp1r3d	0.994	http://api.brain-map.org/grid_data/download/71213671?include=energy
Atxn2	0.994	http://api.brain-map.org/grid_data/download/69873421?include=energy
Pla2g1b	0.994	http://api.brain-map.org/grid_data/download/74635168?include=energy
Ifnar1	0.994	http://api.brain-map.org/grid_data/download/588?include=energy
Tsc1	0.994	http://api.brain-map.org/grid_data/download/72004449?include=energy
Zscan29	0.994	http://api.brain-map.org/grid_data/download/69174016?include=energy
Crygf	0.994	http://api.brain-map.org/grid_data/download/68919868?include=energy
Cfd	0.994	http://api.brain-map.org/grid_data/download/71764745?include=energy
Prrc2a	0.994	http://api.brain-map.org/grid_data/download/69013308?include=energy
Ndfip1	0.994	http://api.brain-map.org/grid_data/download/72715?include=energy
Msto1	0.994	http://api.brain-map.org/grid_data/download/1379?include=energy
Pvr	0.994	http://api.brain-map.org/grid_data/download/119504?include=energy
Hydin	0.994	http://api.brain-map.org/grid_data/download/71529923?include=energy
Phf10	0.994	http://api.brain-map.org/grid_data/download/68269066?include=energy
Cct8	0.994	http://api.brain-map.org/grid_data/download/2079?include=energy
Id1	0.994	http://api.brain-map.org/grid_data/download/71608229?include=energy
LOC436542	0.994	http://api.brain-map.org/grid_data/download/70784618?include=energy
H2-T10	0.994	http://api.brain-map.org/grid_data/download/68795452?include=energy
Cyp27a1	0.994	http://api.brain-map.org/grid_data/download/70634300?include=energy
F830212M06Rik*	0.994	http://api.brain-map.org/grid_data/download/73731454?include=energy
LOC436442	0.994	http://api.brain-map.org/grid_data/download/74736354?include=energy
Fkbp6	0.994	http://api.brain-map.org/grid_data/download/2222?include=energy
Sin3b	0.994	http://api.brain-map.org/grid_data/download/69169397?include=energy
Cacnb2	0.994	http://api.brain-map.org/grid_data/download/72119651?include=energy
Traf7	0.994	http://api.brain-map.org/grid_data/download/2527?include=energy
A130050O07Rik	0.994	http://api.brain-map.org/grid_data/download/81600158?include=energy
Cfdp1	0.994	http://api.brain-map.org/grid_data/download/70431229?include=energy
Dpysl5	0.994	http://api.brain-map.org/grid_data/download/73635770?include=energy
BC003266	0.993	http://api.brain-map.org/grid_data/download/69080042?include=energy
Tm7sf2	0.993	http://api.brain-map.org/grid_data/download/69015494?include=energy
Eif4a3	0.993	http://api.brain-map.org/grid_data/download/2143?include=energy
Rhox5	0.993	http://api.brain-map.org/grid_data/download/77454707?include=energy
Olfr411	0.993	http://api.brain-map.org/grid_data/download/73653416?include=energy
Olfr553	0.993	http://api.brain-map.org/grid_data/download/74724169?include=energy
Tiprl	0.993	http://api.brain-map.org/grid_data/download/70445431?include=energy
Acsl4	0.993	http://api.brain-map.org/grid_data/download/71495215?include=energy
Vwa5b1	0.993	http://api.brain-map.org/grid_data/download/71493681?include=energy
P2ry2	0.993	http://api.brain-map.org/grid_data/download/75144693?include=energy
Actr1a	0.993	http://api.brain-map.org/grid_data/download/321206?include=energy
Pdlim7	0.993	http://api.brain-map.org/grid_data/download/69113566?include=energy
Pnpla6	0.993	http://api.brain-map.org/grid_data/download/71280688?include=energy
Ankrd53	0.993	http://api.brain-map.org/grid_data/download/71283534?include=energy
Pip4k2a	0.993	http://api.brain-map.org/grid_data/download/75146004?include=energy
Nmral1	0.993	http://api.brain-map.org/grid_data/download/69549178?include=energy
Ppp1cc	0.993	http://api.brain-map.org/grid_data/download/513023?include=energy
Cat	0.993	http://api.brain-map.org/grid_data/download/70919370?include=energy
C330013J21Rik	0.993	http://api.brain-map.org/grid_data/download/69530971?include=energy
Dync1i1	0.993	http://api.brain-map.org/grid_data/download/69837160?include=energy
A430093F15Rik	0.993	http://api.brain-map.org/grid_data/download/70228436?include=energy
Anpep	0.993	http://api.brain-map.org/grid_data/download/68443160?include=energy
8430423G03Rik	0.993	http://api.brain-map.org/grid_data/download/71892340?include=energy
Gm595	0.993	http://api.brain-map.org/grid_data/download/74562558?include=energy
Pgrmc1	0.993	http://api.brain-map.org/grid_data/download/2478?include=energy
Prss40	0.993	http://api.brain-map.org/grid_data/download/69980280?include=energy
Cox4i1	0.993	http://api.brain-map.org/grid_data/download/73992933?include=energy
LOC436460	0.993	http://api.brain-map.org/grid_data/download/70784612?include=energy
Huwe1	0.993	http://api.brain-map.org/grid_data/download/70445293?include=energy
Gm382	0.993	http://api.brain-map.org/grid_data/download/70295626?include=energy
Nup93	0.993	http://api.brain-map.org/grid_data/download/75774679?include=energy
Gmnn	0.993	http://api.brain-map.org/grid_data/download/75827097?include=energy
Cplx2	0.993	http://api.brain-map.org/grid_data/download/308054482?include=energy
Mocs1	0.993	http://api.brain-map.org/grid_data/download/323590?include=energy
Dtx3l	0.993	http://api.brain-map.org/grid_data/download/70303695?include=energy
Prrc2c	0.993	http://api.brain-map.org/grid_data/download/72081816?include=energy
Pdcd2	0.993	http://api.brain-map.org/grid_data/download/69782909?include=energy
Aqp1	0.993	http://api.brain-map.org/grid_data/download/79534912?include=energy
Acot7	0.993	http://api.brain-map.org/grid_data/download/145?include=energy
Supt16h	0.993	http://api.brain-map.org/grid_data/download/69838741?include=energy
LOC433947	0.993	http://api.brain-map.org/grid_data/download/70787137?include=energy
2310004N24Rik	0.992	http://api.brain-map.org/grid_data/download/70474899?include=energy
Lhx1	0.992	http://api.brain-map.org/grid_data/download/633?include=energy
Xbp1	0.992	http://api.brain-map.org/grid_data/download/1119?include=energy
Cyth2	0.992	http://api.brain-map.org/grid_data/download/73514857?include=energy
Reps1	0.992	http://api.brain-map.org/grid_data/download/71764709?include=energy
LOC433656	0.992	http://api.brain-map.org/grid_data/download/70786628?include=energy
Ndufa2	0.992	http://api.brain-map.org/grid_data/download/81599652?include=energy
Dynlt3	0.992	http://api.brain-map.org/grid_data/download/74425518?include=energy
6330403K07Rik	0.992	http://api.brain-map.org/grid_data/download/69444795?include=energy
2700023E23Rik	0.992	http://api.brain-map.org/grid_data/download/71211377?include=energy
Nol6	0.992	http://api.brain-map.org/grid_data/download/69169517?include=energy
Odf1	0.992	http://api.brain-map.org/grid_data/download/71920457?include=energy
Ppcdc	0.992	http://api.brain-map.org/grid_data/download/68744044?include=energy
LOC545854	0.992	http://api.brain-map.org/grid_data/download/73732153?include=energy
Clspn	0.992	http://api.brain-map.org/grid_data/download/68666959?include=energy
B230215H11Rik*	0.992	http://api.brain-map.org/grid_data/download/73906355?include=energy
Elp3	0.992	http://api.brain-map.org/grid_data/download/69838597?include=energy
Kif18b	0.992	http://api.brain-map.org/grid_data/download/68844434?include=energy
Ccdc136	0.992	http://api.brain-map.org/grid_data/download/324214?include=energy
Gas6	0.992	http://api.brain-map.org/grid_data/download/72008122?include=energy
D130047A11Rik*	0.992	http://api.brain-map.org/grid_data/download/73834337?include=energy
Epb4.1l4a	0.992	http://api.brain-map.org/grid_data/download/81599644?include=energy
Ccna2	0.992	http://api.brain-map.org/grid_data/download/77464888?include=energy
Fastk	0.992	http://api.brain-map.org/grid_data/download/71610819?include=energy
Rgp1	0.992	http://api.brain-map.org/grid_data/download/71281241?include=energy
Pgam1	0.992	http://api.brain-map.org/grid_data/download/513469?include=energy
Fam155a	0.992	http://api.brain-map.org/grid_data/download/73927682?include=energy
Olfr653	0.992	http://api.brain-map.org/grid_data/download/74734794?include=energy
Klraq1	0.992	http://api.brain-map.org/grid_data/download/69352920?include=energy
Hp	0.992	http://api.brain-map.org/grid_data/download/70277955?include=energy
Gabarapl1	0.992	http://api.brain-map.org/grid_data/download/654925?include=energy
Zfp36	0.992	http://api.brain-map.org/grid_data/download/68445925?include=energy
Bco2	0.992	http://api.brain-map.org/grid_data/download/69529262?include=energy
Pigyl	0.992	http://api.brain-map.org/grid_data/download/199214?include=energy
Lingo4	0.992	http://api.brain-map.org/grid_data/download/76080954?include=energy
Eno4	0.992	http://api.brain-map.org/grid_data/download/67792195?include=energy
Slc16a12	0.992	http://api.brain-map.org/grid_data/download/69527728?include=energy
Polr3b	0.992	http://api.brain-map.org/grid_data/download/69816691?include=energy
Maf1	0.992	http://api.brain-map.org/grid_data/download/69080508?include=energy
Fam13c	0.992	http://api.brain-map.org/grid_data/download/72081756?include=energy
Olfr685	0.992	http://api.brain-map.org/grid_data/download/74734848?include=energy
Trim25	0.992	http://api.brain-map.org/grid_data/download/1545?include=energy
Artn	0.992	http://api.brain-map.org/grid_data/download/71495819?include=energy
Arsa	0.992	http://api.brain-map.org/grid_data/download/75749415?include=energy
Cenpa	0.992	http://api.brain-map.org/grid_data/download/68301156?include=energy
Rnasel	0.992	http://api.brain-map.org/grid_data/download/73817438?include=energy
Hsph1	0.992	http://api.brain-map.org/grid_data/download/69735126?include=energy
Snap47	0.992	http://api.brain-map.org/grid_data/download/3?include=energy
Tnfrsf17	0.992	http://api.brain-map.org/grid_data/download/68744719?include=energy
Kansl1	0.992	http://api.brain-map.org/grid_data/download/68667772?include=energy
Clvs1	0.992	http://api.brain-map.org/grid_data/download/69443106?include=energy
Helb	0.992	http://api.brain-map.org/grid_data/download/69734977?include=energy
LOC433253	0.991	http://api.brain-map.org/grid_data/download/71669000?include=energy
Stxbp1	0.991	http://api.brain-map.org/grid_data/download/1009?include=energy
Prkch	0.991	http://api.brain-map.org/grid_data/download/73520406?include=energy
Meg3	0.991	http://api.brain-map.org/grid_data/download/71281027?include=energy
Usp40	0.991	http://api.brain-map.org/grid_data/download/71789939?include=energy
Wdr54	0.991	http://api.brain-map.org/grid_data/download/71724774?include=energy
Serpina7	0.991	http://api.brain-map.org/grid_data/download/74579428?include=energy
Lzts2	0.991	http://api.brain-map.org/grid_data/download/69736394?include=energy
1200007C13Rik	0.991	http://api.brain-map.org/grid_data/download/70200902?include=energy
LOC432635	0.991	http://api.brain-map.org/grid_data/download/70611144?include=energy
Ptpn11	0.991	http://api.brain-map.org/grid_data/download/77620471?include=energy
Smurf1	0.991	http://api.brain-map.org/grid_data/download/67767472?include=energy
Serinc1	0.991	http://api.brain-map.org/grid_data/download/67752306?include=energy
Nisch	0.991	http://api.brain-map.org/grid_data/download/69134550?include=energy
LOC381597	0.991	http://api.brain-map.org/grid_data/download/70596095?include=energy
Neu3	0.991	http://api.brain-map.org/grid_data/download/74003385?include=energy
Col15a1	0.991	http://api.brain-map.org/grid_data/download/74511791?include=energy
Banf1	0.991	http://api.brain-map.org/grid_data/download/68910955?include=energy
Bbs9	0.991	http://api.brain-map.org/grid_data/download/69262294?include=energy
Hspd1	0.991	http://api.brain-map.org/grid_data/download/69169385?include=energy
Gtf2ird2	0.991	http://api.brain-map.org/grid_data/download/69353548?include=energy
Atp5g3	0.991	http://api.brain-map.org/grid_data/download/69526970?include=energy
Nsun2	0.991	http://api.brain-map.org/grid_data/download/68301784?include=energy
Eef1a1	0.991	http://api.brain-map.org/grid_data/download/73817934?include=energy
Copb1	0.991	http://api.brain-map.org/grid_data/download/68498609?include=energy
LOC435887	0.991	http://api.brain-map.org/grid_data/download/71022609?include=energy
Ctsb	0.991	http://api.brain-map.org/grid_data/download/67779877?include=energy
Rnf144a	0.991	http://api.brain-map.org/grid_data/download/68157416?include=energy
Zfp626	0.991	http://api.brain-map.org/grid_data/download/69261406?include=energy
Cdk17	0.991	http://api.brain-map.org/grid_data/download/69262342?include=energy
Clip4	0.991	http://api.brain-map.org/grid_data/download/75774680?include=energy
Espl1	0.991	http://api.brain-map.org/grid_data/download/69672150?include=energy
Ckmt1	0.991	http://api.brain-map.org/grid_data/download/100144550?include=energy
Tm9sf1	0.991	http://api.brain-map.org/grid_data/download/641528?include=energy
Olfr981	0.991	http://api.brain-map.org/grid_data/download/74735015?include=energy
2700007P21Rik	0.991	http://api.brain-map.org/grid_data/download/69835563?include=energy
Ckm	0.991	http://api.brain-map.org/grid_data/download/76115727?include=energy
Mtx2	0.991	http://api.brain-map.org/grid_data/download/77464914?include=energy
LOC208429	0.991	http://api.brain-map.org/grid_data/download/70565897?include=energy
Gprasp1	0.991	http://api.brain-map.org/grid_data/download/70743888?include=energy
Ablim2	0.991	http://api.brain-map.org/grid_data/download/73636039?include=energy
Psmc2	0.991	http://api.brain-map.org/grid_data/download/356850?include=energy
Pbp2	0.991	http://api.brain-map.org/grid_data/download/71919523?include=energy
Psmb11	0.991	http://api.brain-map.org/grid_data/download/100144866?include=energy
F430212H09Rik*	0.991	http://api.brain-map.org/grid_data/download/73767368?include=energy
Lrrc24	0.991	http://api.brain-map.org/grid_data/download/70305096?include=energy
Aff2	0.991	http://api.brain-map.org/grid_data/download/76003633?include=energy
Adc	0.991	http://api.brain-map.org/grid_data/download/72182963?include=energy
Hba-a1	0.991	http://api.brain-map.org/grid_data/download/77332708?include=energy
Bzrap1	0.99	http://api.brain-map.org/grid_data/download/69530696?include=energy
Aldh2	0.99	http://api.brain-map.org/grid_data/download/74819240?include=energy
Rian	0.99	http://api.brain-map.org/grid_data/download/74580805?include=energy
Mrps27	0.99	http://api.brain-map.org/grid_data/download/68797514?include=energy
Nanog	0.99	http://api.brain-map.org/grid_data/download/69289465?include=energy
Ylpm1	0.99	http://api.brain-map.org/grid_data/download/69134352?include=energy
Nfkb1	0.99	http://api.brain-map.org/grid_data/download/77454688?include=energy
2900011O08Rik	0.99	http://api.brain-map.org/grid_data/download/67815971?include=energy
LOC195357	0.99	http://api.brain-map.org/grid_data/download/71764131?include=energy
Tmem177	0.99	http://api.brain-map.org/grid_data/download/69202328?include=energy
Osbpl5	0.99	http://api.brain-map.org/grid_data/download/73997146?include=energy
Dtl	0.99	http://api.brain-map.org/grid_data/download/69816428?include=energy
Tctex1d1	0.99	http://api.brain-map.org/grid_data/download/71891743?include=energy
Add2	0.99	http://api.brain-map.org/grid_data/download/70230801?include=energy
Hook2	0.99	http://api.brain-map.org/grid_data/download/68342033?include=energy
1300001I01Rik	0.99	http://api.brain-map.org/grid_data/download/71380477?include=energy
Cmas	0.99	http://api.brain-map.org/grid_data/download/67767470?include=energy
Bcs1l	0.99	http://api.brain-map.org/grid_data/download/68637892?include=energy
D130040H23Rik	0.99	http://api.brain-map.org/grid_data/download/71808606?include=energy
Spnb4	0.99	http://api.brain-map.org/grid_data/download/81657679?include=energy
Tmx2	0.99	http://api.brain-map.org/grid_data/download/71836805?include=energy
Rpl13	0.99	http://api.brain-map.org/grid_data/download/69028599?include=energy
Trem2	0.99	http://api.brain-map.org/grid_data/download/67870433?include=energy
Eif2ak2	0.99	http://api.brain-map.org/grid_data/download/73424017?include=energy
Pitx1	0.99	http://api.brain-map.org/grid_data/download/199398?include=energy
Hist1h2bc	0.99	http://api.brain-map.org/grid_data/download/560?include=energy
Ndufa5	0.99	http://api.brain-map.org/grid_data/download/68522460?include=energy
Bnipl	0.99	http://api.brain-map.org/grid_data/download/79536604?include=energy
LOC544874	0.99	http://api.brain-map.org/grid_data/download/74736396?include=energy
Arhgap42	0.99	http://api.brain-map.org/grid_data/download/71668369?include=energy
Mast3	0.99	http://api.brain-map.org/grid_data/download/69236658?include=energy
Rab13	0.99	http://api.brain-map.org/grid_data/download/68637581?include=energy
Aspscr1	0.99	http://api.brain-map.org/grid_data/download/545113?include=energy
Mm.48776	0.99	http://api.brain-map.org/grid_data/download/71210316?include=energy
9930104L06Rik	0.99	http://api.brain-map.org/grid_data/download/69447876?include=energy
Ctrl	0.99	http://api.brain-map.org/grid_data/download/75934519?include=energy
Hip1r	0.99	http://api.brain-map.org/grid_data/download/69735018?include=energy
Rai1	0.99	http://api.brain-map.org/grid_data/download/71148190?include=energy
Srsf1	0.99	http://api.brain-map.org/grid_data/download/70795778?include=energy
Lilra5	0.99	http://api.brain-map.org/grid_data/download/71587613?include=energy
Hnf1a	0.99	http://api.brain-map.org/grid_data/download/77464918?include=energy
Fhad1	0.99	http://api.brain-map.org/grid_data/download/68546125?include=energy
Vapb	0.99	http://api.brain-map.org/grid_data/download/68745305?include=energy
Mm.358838	0.99	http://api.brain-map.org/grid_data/download/75991186?include=energy
Slc22a17	0.99	http://api.brain-map.org/grid_data/download/74635072?include=energy
Setx	0.99	http://api.brain-map.org/grid_data/download/73808331?include=energy
9830128D06Rik*	0.99	http://api.brain-map.org/grid_data/download/73808486?include=energy
Cops2	0.99	http://api.brain-map.org/grid_data/download/68563056?include=energy
Myom3	0.989	http://api.brain-map.org/grid_data/download/70786794?include=energy
Cpne9	0.989	http://api.brain-map.org/grid_data/download/308054780?include=energy
Ube4b	0.989	http://api.brain-map.org/grid_data/download/74750010?include=energy
Hnrnpu	0.989	http://api.brain-map.org/grid_data/download/386235?include=energy
A230046K03Rik	0.989	http://api.brain-map.org/grid_data/download/69447924?include=energy
Ddx39	0.989	http://api.brain-map.org/grid_data/download/68341728?include=energy
Fbxo21	0.989	http://api.brain-map.org/grid_data/download/70634119?include=energy
Camk1d	0.989	http://api.brain-map.org/grid_data/download/73992927?include=energy
Tmem207	0.989	http://api.brain-map.org/grid_data/download/70565981?include=energy
Ring1	0.989	http://api.brain-map.org/grid_data/download/69079988?include=energy
2010107E04Rik	0.989	http://api.brain-map.org/grid_data/download/69116709?include=energy
Pldi	0.989	http://api.brain-map.org/grid_data/download/71920219?include=energy
Sept3	0.989	http://api.brain-map.org/grid_data/download/69838258?include=energy
Dmap1	0.989	http://api.brain-map.org/grid_data/download/68745426?include=energy
Gm1199	0.989	http://api.brain-map.org/grid_data/download/70300055?include=energy
Dusp11	0.989	http://api.brain-map.org/grid_data/download/69838738?include=energy
1700021A07Rik	0.989	http://api.brain-map.org/grid_data/download/71377987?include=energy
Matn4	0.989	http://api.brain-map.org/grid_data/download/77371795?include=energy
Otud6b	0.989	http://api.brain-map.org/grid_data/download/83158?include=energy
LOC436449	0.989	http://api.brain-map.org/grid_data/download/74736360?include=energy
Ppapdc1b	0.989	http://api.brain-map.org/grid_data/download/74635180?include=energy
Fam13a	0.989	http://api.brain-map.org/grid_data/download/71668374?include=energy
Agfg1	0.989	http://api.brain-map.org/grid_data/download/69173488?include=energy
Xk	0.989	http://api.brain-map.org/grid_data/download/71670735?include=energy
Tbc1d16	0.989	http://api.brain-map.org/grid_data/download/69014824?include=energy
Man1a	0.989	http://api.brain-map.org/grid_data/download/69120618?include=energy
Rps23	0.989	http://api.brain-map.org/grid_data/download/77465042?include=energy
LOC545698	0.989	http://api.brain-map.org/grid_data/download/70302998?include=energy
Unc79	0.989	http://api.brain-map.org/grid_data/download/69446310?include=energy
9230119M08Rik*	0.989	http://api.brain-map.org/grid_data/download/73870599?include=energy
Wdr75	0.989	http://api.brain-map.org/grid_data/download/69202687?include=energy
1810026J23Rik	0.989	http://api.brain-map.org/grid_data/download/70527951?include=energy
Thtpa	0.989	http://api.brain-map.org/grid_data/download/69170102?include=energy
Cxcr7	0.989	http://api.brain-map.org/grid_data/download/79543540?include=energy
Olfr354	0.989	http://api.brain-map.org/grid_data/download/74721018?include=energy
Gm362	0.989	http://api.brain-map.org/grid_data/download/70296002?include=energy
C230057A21Rik	0.989	http://api.brain-map.org/grid_data/download/74509587?include=energy
Casq1	0.989	http://api.brain-map.org/grid_data/download/74819254?include=energy

Fam83c	0.989	http://api.brain-map.org/grid_data/download/74638863?include=energy
Tmcc2	0.989	http://api.brain-map.org/grid_data/download/69835579?include=energy
Cnih	0.989	http://api.brain-map.org/grid_data/download/278?include=energy
Styxl1	0.989	http://api.brain-map.org/grid_data/download/68745432?include=energy
Pnrc1	0.989	http://api.brain-map.org/grid_data/download/2501?include=energy
Paf1	0.989	http://api.brain-map.org/grid_data/download/293469?include=energy
Ptp4a3	0.989	http://api.brain-map.org/grid_data/download/71735828?include=energy
Jund	0.989	http://api.brain-map.org/grid_data/download/606?include=energy
Rnase12	0.989	http://api.brain-map.org/grid_data/download/74743366?include=energy
mCG145979	0.989	http://api.brain-map.org/grid_data/download/71212386?include=energy
Zbtb8b	0.989	http://api.brain-map.org/grid_data/download/68158083?include=energy
Msrb3	0.989	http://api.brain-map.org/grid_data/download/71808693?include=energy
Vmn1r232	0.989	http://api.brain-map.org/grid_data/download/70785618?include=energy
Gm14354	0.989	http://api.brain-map.org/grid_data/download/71891623?include=energy
Rnf122	0.988	http://api.brain-map.org/grid_data/download/275875?include=energy
TC1484629	0.988	http://api.brain-map.org/grid_data/download/71456128?include=energy
E2f6	0.988	http://api.brain-map.org/grid_data/download/2173?include=energy
Ubfd1	0.988	http://api.brain-map.org/grid_data/download/70431265?include=energy
Lrrc14	0.988	http://api.brain-map.org/grid_data/download/71612178?include=energy
Trex2	0.988	http://api.brain-map.org/grid_data/download/77799047?include=energy
Rpl31	0.988	http://api.brain-map.org/grid_data/download/68861982?include=energy
Slc25a48	0.988	http://api.brain-map.org/grid_data/download/71809515?include=energy
Rnf220	0.988	http://api.brain-map.org/grid_data/download/71924364?include=energy
Sertad4	0.988	http://api.brain-map.org/grid_data/download/71920519?include=energy
C030013D06Rik	0.988	http://api.brain-map.org/grid_data/download/71148046?include=energy
LOC433019	0.988	http://api.brain-map.org/grid_data/download/70611455?include=energy
5830433M19Rik	0.988	http://api.brain-map.org/grid_data/download/68341830?include=energy
Unc45a	0.988	http://api.brain-map.org/grid_data/download/70919627?include=energy
Kcnc1	0.988	http://api.brain-map.org/grid_data/download/72108825?include=energy
1700003H04Rik	0.988	http://api.brain-map.org/grid_data/download/74581259?include=energy
Plekhb2	0.988	http://api.brain-map.org/grid_data/download/69028402?include=energy
Epm2a	0.988	http://api.brain-map.org/grid_data/download/69672102?include=energy
Hs3st1	0.988	http://api.brain-map.org/grid_data/download/2305?include=energy
Zfp579	0.988	http://api.brain-map.org/grid_data/download/73584705?include=energy
Ngfrap1	0.988	http://api.brain-map.org/grid_data/download/1786?include=energy
LOC319574	0.988	http://api.brain-map.org/grid_data/download/74277653?include=energy
Slc29a2	0.988	http://api.brain-map.org/grid_data/download/74357657?include=energy
Invs	0.988	http://api.brain-map.org/grid_data/download/69289207?include=energy
Klhl21	0.988	http://api.brain-map.org/grid_data/download/76003364?include=energy
Kansl1l	0.988	http://api.brain-map.org/grid_data/download/69531013?include=energy
Psma6	0.988	http://api.brain-map.org/grid_data/download/69817408?include=energy
Olfr284	0.988	http://api.brain-map.org/grid_data/download/73653374?include=energy
F13b	0.988	http://api.brain-map.org/grid_data/download/68446033?include=energy
Eif4g3	0.988	http://api.brain-map.org/grid_data/download/69671971?include=energy
Dgat1	0.988	http://api.brain-map.org/grid_data/download/69095971?include=energy
Ahnak	0.988	http://api.brain-map.org/grid_data/download/70812687?include=energy
Grik1	0.988	http://api.brain-map.org/grid_data/download/527?include=energy
Eri2	0.988	http://api.brain-map.org/grid_data/download/70813053?include=energy
Dus1l	0.988	http://api.brain-map.org/grid_data/download/68341884?include=energy
Adra2b	0.988	http://api.brain-map.org/grid_data/download/74724532?include=energy
Tmem106b	0.988	http://api.brain-map.org/grid_data/download/71920509?include=energy
Mrpl4	0.988	http://api.brain-map.org/grid_data/download/68845765?include=energy
D630040G17Rik	0.988	http://api.brain-map.org/grid_data/download/72283587?include=energy
Gm2511	0.988	http://api.brain-map.org/grid_data/download/70787567?include=energy
Xrra1	0.988	http://api.brain-map.org/grid_data/download/73584771?include=energy
Rnaseh2b	0.988	http://api.brain-map.org/grid_data/download/70203543?include=energy
Atp6v1c1	0.988	http://api.brain-map.org/grid_data/download/70539160?include=energy
Fbxo10	0.988	http://api.brain-map.org/grid_data/download/69672318?include=energy
Trim21	0.988	http://api.brain-map.org/grid_data/download/76135656?include=energy
Tmem174	0.987	http://api.brain-map.org/grid_data/download/69261397?include=energy
Atxn10	0.987	http://api.brain-map.org/grid_data/download/544899?include=energy
Pax1	0.987	http://api.brain-map.org/grid_data/download/69289661?include=energy
LOC435411	0.987	http://api.brain-map.org/grid_data/download/71022499?include=energy
Slc26a9	0.987	http://api.brain-map.org/grid_data/download/70302123?include=energy
Pvalb	0.987	http://api.brain-map.org/grid_data/download/868?include=energy
Rapgef6	0.987	http://api.brain-map.org/grid_data/download/75041528?include=energy
Fam180a	0.987	http://api.brain-map.org/grid_data/download/72283801?include=energy
Pom121l2	0.987	http://api.brain-map.org/grid_data/download/70295554?include=energy
Dct	0.987	http://api.brain-map.org/grid_data/download/69237185?include=energy
Ndufa10	0.987	http://api.brain-map.org/grid_data/download/74658173?include=energy
Myh4	0.987	http://api.brain-map.org/grid_data/download/68744086?include=energy
Rps26	0.987	http://api.brain-map.org/grid_data/download/68191282?include=energy
Dctn3	0.987	http://api.brain-map.org/grid_data/download/69174493?include=energy
Gja6	0.987	http://api.brain-map.org/grid_data/download/69257331?include=energy
D030024E09Rik	0.987	http://api.brain-map.org/grid_data/download/74514061?include=energy
A930006D01Rik	0.987	http://api.brain-map.org/grid_data/download/100144402?include=energy
Wdfy3	0.987	http://api.brain-map.org/grid_data/download/69174559?include=energy
Kat2b	0.987	http://api.brain-map.org/grid_data/download/76003323?include=energy
LOC545053	0.987	http://api.brain-map.org/grid_data/download/69290770?include=energy
Atp8b2	0.987	http://api.brain-map.org/grid_data/download/69527006?include=energy
D930019A15Rik*	0.987	http://api.brain-map.org/grid_data/download/74076078?include=energy
Sh2d3c	0.987	http://api.brain-map.org/grid_data/download/70302057?include=energy
Uvrag	0.987	http://api.brain-map.org/grid_data/download/69173286?include=energy
5031410I06Rik	0.986	http://api.brain-map.org/grid_data/download/72472398?include=energy
Zfp759	0.986	http://api.brain-map.org/grid_data/download/678694?include=energy
Spg7	0.986	http://api.brain-map.org/grid_data/download/72002079?include=energy
Ctnnb1	0.986	http://api.brain-map.org/grid_data/download/79591729?include=energy
LOC225475	0.986	http://api.brain-map.org/grid_data/download/74562290?include=energy
Tctex1d4	0.986	http://api.brain-map.org/grid_data/download/75985970?include=energy
Anks1b	0.986	http://api.brain-map.org/grid_data/download/73513603?include=energy
Serbp1	0.986	http://api.brain-map.org/grid_data/download/532664?include=energy
Zfp616	0.986	http://api.brain-map.org/grid_data/download/74736105?include=energy
Pkd2	0.986	http://api.brain-map.org/grid_data/download/69863247?include=energy
Epb4.9	0.986	http://api.brain-map.org/grid_data/download/79544804?include=energy
Grin2d	0.986	http://api.brain-map.org/grid_data/download/75551479?include=energy
LOC545297	0.986	http://api.brain-map.org/grid_data/download/74736426?include=energy
Cyb5d1	0.986	http://api.brain-map.org/grid_data/download/70298274?include=energy
A2ld1	0.986	http://api.brain-map.org/grid_data/download/79539638?include=energy
Tmem59l	0.986	http://api.brain-map.org/grid_data/download/68843883?include=energy
Tpm1	0.986	http://api.brain-map.org/grid_data/download/69817031?include=energy
Tex11	0.986	http://api.brain-map.org/grid_data/download/69980286?include=energy
Lyve1	0.986	http://api.brain-map.org/grid_data/download/71015813?include=energy
Fam116b	0.986	http://api.brain-map.org/grid_data/download/69353669?include=energy
Tmem234	0.986	http://api.brain-map.org/grid_data/download/68632202?include=energy
1700012B09Rik	0.986	http://api.brain-map.org/grid_data/download/70723626?include=energy
Glrp1	0.986	http://api.brain-map.org/grid_data/download/69672892?include=energy
Tcf20	0.986	http://api.brain-map.org/grid_data/download/70598360?include=energy
Nod2	0.986	http://api.brain-map.org/grid_data/download/75038483?include=energy
Pex6	0.986	http://api.brain-map.org/grid_data/download/72725?include=energy
Zmpste24	0.986	http://api.brain-map.org/grid_data/download/70200806?include=energy
Csl	0.986	http://api.brain-map.org/grid_data/download/69116484?include=energy
BC021891	0.986	http://api.brain-map.org/grid_data/download/73906149?include=energy
Fcrl5	0.986	http://api.brain-map.org/grid_data/download/75197498?include=energy
Sulf1	0.986	http://api.brain-map.org/grid_data/download/75041501?include=energy
Uty	0.986	http://api.brain-map.org/grid_data/download/69290716?include=energy
Zfp870	0.986	http://api.brain-map.org/grid_data/download/69529226?include=energy
Rnf112	0.986	http://api.brain-map.org/grid_data/download/393241?include=energy
Olfr807	0.986	http://api.brain-map.org/grid_data/download/74735033?include=energy
Nckap1	0.986	http://api.brain-map.org/grid_data/download/69782073?include=energy
Ankrd11	0.986	http://api.brain-map.org/grid_data/download/70203603?include=energy
Obscn	0.986	http://api.brain-map.org/grid_data/download/69262301?include=energy
Nap1l3	0.986	http://api.brain-map.org/grid_data/download/583576?include=energy
Sars	0.986	http://api.brain-map.org/grid_data/download/69873403?include=energy
1700018A14Rik	0.986	http://api.brain-map.org/grid_data/download/74357821?include=energy
Ttc9c	0.986	http://api.brain-map.org/grid_data/download/69133719?include=energy
Kti12	0.986	http://api.brain-map.org/grid_data/download/68410464?include=energy
Mm.188298	0.986	http://api.brain-map.org/grid_data/download/71152270?include=energy
Commd5	0.986	http://api.brain-map.org/grid_data/download/68080687?include=energy
Esyt1	0.986	http://api.brain-map.org/grid_data/download/112202410?include=energy
Rab15	0.986	http://api.brain-map.org/grid_data/download/69080008?include=energy
Ddrgk1	0.986	http://api.brain-map.org/grid_data/download/74821558?include=energy
Brsk1	0.986	http://api.brain-map.org/grid_data/download/100144096?include=energy
Gstt4	0.986	http://api.brain-map.org/grid_data/download/70946170?include=energy
Pmm2	0.986	http://api.brain-map.org/grid_data/download/69012843?include=energy
Arl6ip6	0.986	http://api.brain-map.org/grid_data/download/71627821?include=energy
Asphd2	0.986	http://api.brain-map.org/grid_data/download/68745516?include=energy
Mm.12964	0.986	http://api.brain-map.org/grid_data/download/71148064?include=energy
Atp5a1	0.986	http://api.brain-map.org/grid_data/download/1579?include=energy
Sry	0.986	http://api.brain-map.org/grid_data/download/77932785?include=energy
Cox7a2l	0.986	http://api.brain-map.org/grid_data/download/81599622?include=energy
Gkn3	0.985	http://api.brain-map.org/grid_data/download/74957933?include=energy
Agmo	0.985	http://api.brain-map.org/grid_data/download/69448680?include=energy
Mettl2	0.985	http://api.brain-map.org/grid_data/download/71789819?include=energy
Grm7	0.985	http://api.brain-map.org/grid_data/download/81790704?include=energy
Cc2d2a	0.985	http://api.brain-map.org/grid_data/download/678687?include=energy
LOC432787	0.985	http://api.brain-map.org/grid_data/download/74822590?include=energy
Tmem39a	0.985	http://api.brain-map.org/grid_data/download/75750276?include=energy
Mm.240247	0.985	http://api.brain-map.org/grid_data/download/71152635?include=energy
Pfpl	0.985	http://api.brain-map.org/grid_data/download/71832907?include=energy
Sesn3	0.985	http://api.brain-map.org/grid_data/download/69873594?include=energy
Vit	0.985	http://api.brain-map.org/grid_data/download/76081041?include=energy
Cyp11a1	0.985	http://api.brain-map.org/grid_data/download/69608327?include=energy
Zfp454	0.985	http://api.brain-map.org/grid_data/download/68798585?include=energy
Hpgd	0.985	http://api.brain-map.org/grid_data/download/70918952?include=energy
Nhlh2	0.985	http://api.brain-map.org/grid_data/download/74657929?include=energy
Hbb	0.985	http://api.brain-map.org/grid_data/download/77340464?include=energy
Mb	0.985	http://api.brain-map.org/grid_data/download/75750351?include=energy
Serpina3a	0.985	http://api.brain-map.org/grid_data/download/75860996?include=energy
Rttn	0.985	http://api.brain-map.org/grid_data/download/74513890?include=energy
Tfcp2l1	0.985	http://api.brain-map.org/grid_data/download/71488858?include=energy
Gtf2a1l	0.985	http://api.brain-map.org/grid_data/download/70431331?include=energy
Shank3	0.985	http://api.brain-map.org/grid_data/download/69816840?include=energy
Adhfe1	0.985	http://api.brain-map.org/grid_data/download/69170300?include=energy
Med19	0.985	http://api.brain-map.org/grid_data/download/69202651?include=energy
Rars	0.985	http://api.brain-map.org/grid_data/download/68341788?include=energy
Erv3	0.985	http://api.brain-map.org/grid_data/download/69353312?include=energy
Rab14	0.985	http://api.brain-map.org/grid_data/download/70539158?include=energy
Ahsa2	0.985	http://api.brain-map.org/grid_data/download/71358552?include=energy
Slc48a1	0.985	http://api.brain-map.org/grid_data/download/532687?include=energy
Ttc33	0.985	http://api.brain-map.org/grid_data/download/70527999?include=energy
LOC546168	0.985	http://api.brain-map.org/grid_data/download/71212093?include=energy
Pcdhb21	0.985	http://api.brain-map.org/grid_data/download/69782826?include=energy
Car12	0.985	http://api.brain-map.org/grid_data/download/72081406?include=energy
Ebag9	0.985	http://api.brain-map.org/grid_data/download/69671839?include=energy
Cox6a1	0.985	http://api.brain-map.org/grid_data/download/69080022?include=energy
Disp1	0.985	http://api.brain-map.org/grid_data/download/69626897?include=energy
Fem1c	0.985	http://api.brain-map.org/grid_data/download/69288968?include=energy
Trip11	0.985	http://api.brain-map.org/grid_data/download/69444759?include=energy
AI182371	0.985	http://api.brain-map.org/grid_data/download/71569325?include=energy
Mrpl54	0.985	http://api.brain-map.org/grid_data/download/74819464?include=energy
Cdh13	0.985	http://api.brain-map.org/grid_data/download/234?include=energy
Tmem81	0.985	http://api.brain-map.org/grid_data/download/69407953?include=energy
F630006H17Rik*	0.985	http://api.brain-map.org/grid_data/download/73748842?include=energy
Gm5524	0.985	http://api.brain-map.org/grid_data/download/70723056?include=energy
Hspe1	0.985	http://api.brain-map.org/grid_data/download/68499485?include=energy
Eaf1	0.985	http://api.brain-map.org/grid_data/download/69671827?include=energy
Onecut3	0.985	http://api.brain-map.org/grid_data/download/81655213?include=energy
Arhgef17	0.984	http://api.brain-map.org/grid_data/download/74821649?include=energy
Taf4a	0.984	http://api.brain-map.org/grid_data/download/69290573?include=energy
5930416I19Rik	0.984	http://api.brain-map.org/grid_data/download/69444729?include=energy
Gm11541	0.984	http://api.brain-map.org/grid_data/download/74635830?include=energy
Acsm2	0.984	http://api.brain-map.org/grid_data/download/69170336?include=energy
Hap1	0.984	http://api.brain-map.org/grid_data/download/72007561?include=energy
Cap1	0.984	http://api.brain-map.org/grid_data/download/323396?include=energy
Ttll7	0.984	http://api.brain-map.org/grid_data/download/72128755?include=energy
Nap1l4	0.984	http://api.brain-map.org/grid_data/download/71789891?include=energy
Tln2	0.984	http://api.brain-map.org/grid_data/download/71022286?include=energy
Mrpl13	0.984	http://api.brain-map.org/grid_data/download/69116960?include=energy
Arhgap32	0.984	http://api.brain-map.org/grid_data/download/71281516?include=energy
Rpf1	0.984	http://api.brain-map.org/grid_data/download/70303528?include=energy
Gtf2f2	0.984	http://api.brain-map.org/grid_data/download/68631929?include=energy
Eif3a	0.984	http://api.brain-map.org/grid_data/download/69671947?include=energy
Rpl19	0.984	http://api.brain-map.org/grid_data/download/100143495?include=energy
4930432E11Rik	0.984	http://api.brain-map.org/grid_data/download/71808847?include=energy
Zfp420	0.984	http://api.brain-map.org/grid_data/download/70528282?include=energy
BC029214	0.984	http://api.brain-map.org/grid_data/download/77923129?include=energy
Zbtb2	0.984	http://api.brain-map.org/grid_data/download/71213222?include=energy
Ank3	0.984	http://api.brain-map.org/grid_data/download/74047925?include=energy
Nrg1	0.984	http://api.brain-map.org/grid_data/download/74743257?include=energy
BC030870	0.984	http://api.brain-map.org/grid_data/download/74359532?include=energy
Tmem8c	0.984	http://api.brain-map.org/grid_data/download/68798358?include=energy
Gm5547	0.984	http://api.brain-map.org/grid_data/download/72471825?include=energy
Secisbp2	0.984	http://api.brain-map.org/grid_data/download/69873499?include=energy
Krt8	0.984	http://api.brain-map.org/grid_data/download/74822596?include=energy
Mchr1	0.984	http://api.brain-map.org/grid_data/download/74724669?include=energy
Sumo2	0.984	http://api.brain-map.org/grid_data/download/74990518?include=energy
Nrl	0.984	http://api.brain-map.org/grid_data/download/79544826?include=energy
Apitd1	0.984	http://api.brain-map.org/grid_data/download/71664116?include=energy
Akr1a1	0.984	http://api.brain-map.org/grid_data/download/68191138?include=energy
Fkbp1a	0.984	http://api.brain-map.org/grid_data/download/68269071?include=energy
Nhs	0.984	http://api.brain-map.org/grid_data/download/70595756?include=energy
4931400O07Rik	0.984	http://api.brain-map.org/grid_data/download/71891964?include=energy
Sort1	0.984	http://api.brain-map.org/grid_data/download/69288327?include=energy
Dhx30	0.984	http://api.brain-map.org/grid_data/download/68342366?include=energy
Anapc5	0.984	http://api.brain-map.org/grid_data/download/69838210?include=energy
Manea	0.984	http://api.brain-map.org/grid_data/download/74882656?include=energy
A130066N16Rik	0.984	http://api.brain-map.org/grid_data/download/81600160?include=energy
2610029G23Rik	0.984	http://api.brain-map.org/grid_data/download/69117032?include=energy
Actb	0.984	http://api.brain-map.org/grid_data/download/74881516?include=energy
AI597468	0.984	http://api.brain-map.org/grid_data/download/73513392?include=energy
Ankrd46	0.984	http://api.brain-map.org/grid_data/download/71232592?include=energy
Atg16l2	0.984	http://api.brain-map.org/grid_data/download/71764619?include=energy
Fam193a	0.984	http://api.brain-map.org/grid_data/download/69529077?include=energy
Pir	0.984	http://api.brain-map.org/grid_data/download/67870113?include=energy
Ttc1	0.984	http://api.brain-map.org/grid_data/download/70429465?include=energy
Arhgap21	0.984	http://api.brain-map.org/grid_data/download/69526809?include=energy
Moap1	0.984	http://api.brain-map.org/grid_data/download/68666553?include=energy
Gm5441	0.984	http://api.brain-map.org/grid_data/download/71808454?include=energy
Rbx1	0.983	http://api.brain-map.org/grid_data/download/69262239?include=energy
Atat1	0.983	http://api.brain-map.org/grid_data/download/74425517?include=energy
Tmem88	0.983	http://api.brain-map.org/grid_data/download/70542887?include=energy
Ilk	0.983	http://api.brain-map.org/grid_data/download/68443136?include=energy
Mc5r	0.983	http://api.brain-map.org/grid_data/download/69258142?include=energy
Taf1a	0.983	http://api.brain-map.org/grid_data/download/100144468?include=energy
Ran	0.983	http://api.brain-map.org/grid_data/download/2517?include=energy
2310022B05Rik	0.983	http://api.brain-map.org/grid_data/download/70474911?include=energy
Paqr4	0.983	http://api.brain-map.org/grid_data/download/248335?include=energy
Cpe	0.983	http://api.brain-map.org/grid_data/download/1691?include=energy
Specc1	0.983	http://api.brain-map.org/grid_data/download/70611048?include=energy
Vcp	0.983	http://api.brain-map.org/grid_data/download/70194910?include=energy
Prkar2a	0.983	http://api.brain-map.org/grid_data/download/77332735?include=energy
Ghr	0.983	http://api.brain-map.org/grid_data/download/69838740?include=energy
Ythdc2	0.983	http://api.brain-map.org/grid_data/download/70303028?include=energy
4930451G09Rik	0.983	http://api.brain-map.org/grid_data/download/71808741?include=energy
Paqr8	0.983	http://api.brain-map.org/grid_data/download/74724530?include=energy
Dgcr2	0.983	http://api.brain-map.org/grid_data/download/69134238?include=energy
Mapre3	0.983	http://api.brain-map.org/grid_data/download/69015218?include=energy
Imp3	0.983	http://api.brain-map.org/grid_data/download/512518?include=energy
Ppp1r18	0.983	http://api.brain-map.org/grid_data/download/69202370?include=energy
Sh3bgrl2	0.983	http://api.brain-map.org/grid_data/download/71717686?include=energy
Reg2	0.983	http://api.brain-map.org/grid_data/download/71991672?include=energy
Antxr2	0.983	http://api.brain-map.org/grid_data/download/69526659?include=energy
Bccip	0.983	http://api.brain-map.org/grid_data/download/69105321?include=energy
Kansl3	0.983	http://api.brain-map.org/grid_data/download/74988741?include=energy
Zfpm2	0.983	http://api.brain-map.org/grid_data/download/75651162?include=energy
Kifc2	0.983	http://api.brain-map.org/grid_data/download/614?include=energy
Lmbr1	0.983	http://api.brain-map.org/grid_data/download/68342159?include=energy

Clec9a	0.983	http://api.brain-map.org/grid_data/download/69447828?include=energy
Snrnp35	0.983	http://api.brain-map.org/grid_data/download/69444885?include=energy
4930506M07Rik	0.983	http://api.brain-map.org/grid_data/download/322956?include=energy
Olfml1	0.983	http://api.brain-map.org/grid_data/download/68797607?include=energy
Nt5c1b	0.983	http://api.brain-map.org/grid_data/download/75860962?include=energy
Sfta2	0.983	http://api.brain-map.org/grid_data/download/72076509?include=energy
Samd4	0.983	http://api.brain-map.org/grid_data/download/69114866?include=energy
Gbas	0.983	http://api.brain-map.org/grid_data/download/74819681?include=energy
Acsl5	0.983	http://api.brain-map.org/grid_data/download/73931635?include=energy
Pla2g4e	0.983	http://api.brain-map.org/grid_data/download/69202412?include=energy
Lca5	0.983	http://api.brain-map.org/grid_data/download/77465018?include=energy
Miox	0.983	http://api.brain-map.org/grid_data/download/68341394?include=energy
Sele	0.983	http://api.brain-map.org/grid_data/download/69873505?include=energy
Hist3h2ba	0.983	http://api.brain-map.org/grid_data/download/68798669?include=energy
Tacc3	0.983	http://api.brain-map.org/grid_data/download/75651179?include=energy
Olfr1494	0.983	http://api.brain-map.org/grid_data/download/73653332?include=energy
Shank2	0.983	http://api.brain-map.org/grid_data/download/70302075?include=energy
Capns1	0.982	http://api.brain-map.org/grid_data/download/75934915?include=energy
Slc30a5	0.982	http://api.brain-map.org/grid_data/download/78534510?include=energy
Ctsh	0.982	http://api.brain-map.org/grid_data/download/68269287?include=energy
Kat5	0.982	http://api.brain-map.org/grid_data/download/69735174?include=energy
Usp9x	0.982	http://api.brain-map.org/grid_data/download/70194678?include=energy
Gm11568	0.982	http://api.brain-map.org/grid_data/download/71789945?include=energy
Grik2	0.982	http://api.brain-map.org/grid_data/download/71247618?include=energy
AA066038	0.982	http://api.brain-map.org/grid_data/download/73870707?include=energy
Lamc3	0.982	http://api.brain-map.org/grid_data/download/69736016?include=energy
Yars	0.982	http://api.brain-map.org/grid_data/download/1122?include=energy
Ifna4	0.982	http://api.brain-map.org/grid_data/download/70724840?include=energy
D330004B08Rik*	0.982	http://api.brain-map.org/grid_data/download/73834385?include=energy
Fbxo2	0.982	http://api.brain-map.org/grid_data/download/2210?include=energy
Pigb	0.982	http://api.brain-map.org/grid_data/download/69549329?include=energy
Ctf2	0.982	http://api.brain-map.org/grid_data/download/76081096?include=energy
1700015F17Rik	0.982	http://api.brain-map.org/grid_data/download/73497699?include=energy
Rpa2	0.982	http://api.brain-map.org/grid_data/download/70812768?include=energy
Fam13b	0.982	http://api.brain-map.org/grid_data/download/70474959?include=energy
Rin2	0.982	http://api.brain-map.org/grid_data/download/67767452?include=energy
Mm.31198	0.982	http://api.brain-map.org/grid_data/download/74431387?include=energy
St6galnac1	0.982	http://api.brain-map.org/grid_data/download/69873755?include=energy
Armc6	0.982	http://api.brain-map.org/grid_data/download/69526886?include=energy
Cdc16	0.982	http://api.brain-map.org/grid_data/download/69173980?include=energy
1500032L24Rik	0.982	http://api.brain-map.org/grid_data/download/75651168?include=energy
Shisa9	0.982	http://api.brain-map.org/grid_data/download/74363334?include=energy
Chaf1b	0.982	http://api.brain-map.org/grid_data/download/80341135?include=energy
Slc38a1	0.982	http://api.brain-map.org/grid_data/download/1844?include=energy
Pms1	0.982	http://api.brain-map.org/grid_data/download/75990557?include=energy
Bbc3	0.982	http://api.brain-map.org/grid_data/download/68798043?include=energy
Brs3	0.982	http://api.brain-map.org/grid_data/download/70560328?include=energy
Rps25	0.982	http://api.brain-map.org/grid_data/download/68637521?include=energy
Nr1i2	0.982	http://api.brain-map.org/grid_data/download/71325288?include=energy
Bmp8b	0.982	http://api.brain-map.org/grid_data/download/69529370?include=energy
B230325I23Rik*	0.982	http://api.brain-map.org/grid_data/download/73811585?include=energy
Tigd2	0.982	http://api.brain-map.org/grid_data/download/72004435?include=energy
8030498B09Rik	0.982	http://api.brain-map.org/grid_data/download/74958606?include=energy
Fbxo22	0.982	http://api.brain-map.org/grid_data/download/69015482?include=energy
Smarcd1	0.982	http://api.brain-map.org/grid_data/download/69169726?include=energy
Hsf3	0.982	http://api.brain-map.org/grid_data/download/77904821?include=energy
F730050M04Rik*	0.982	http://api.brain-map.org/grid_data/download/73767166?include=energy
Tmeff2	0.982	http://api.brain-map.org/grid_data/download/71015807?include=energy
Eln	0.982	http://api.brain-map.org/grid_data/download/69262291?include=energy
Neurod4	0.982	http://api.brain-map.org/grid_data/download/77455008?include=energy
Pmaip1	0.982	http://api.brain-map.org/grid_data/download/823?include=energy
Gfi1b	0.982	http://api.brain-map.org/grid_data/download/68667557?include=energy
Calr	0.982	http://api.brain-map.org/grid_data/download/67978478?include=energy
Mpp1	0.982	http://api.brain-map.org/grid_data/download/75826682?include=energy
Kif24	0.982	http://api.brain-map.org/grid_data/download/69735783?include=energy
Rasip1	0.982	http://api.brain-map.org/grid_data/download/68523180?include=energy
Gm454	0.982	http://api.brain-map.org/grid_data/download/71764673?include=energy
Atp6v1g2	0.982	http://api.brain-map.org/grid_data/download/583575?include=energy
Cd80	0.982	http://api.brain-map.org/grid_data/download/75988360?include=energy
Baz1b	0.982	http://api.brain-map.org/grid_data/download/69288680?include=energy
Scamp5	0.982	http://api.brain-map.org/grid_data/download/81599672?include=energy
Zfp329	0.982	http://api.brain-map.org/grid_data/download/71809391?include=energy
Gm19849	0.982	http://api.brain-map.org/grid_data/download/70565921?include=energy
Ephx1	0.981	http://api.brain-map.org/grid_data/download/68498247?include=energy
Vamp8	0.981	http://api.brain-map.org/grid_data/download/69059818?include=energy
Olfr536	0.981	http://api.brain-map.org/grid_data/download/74357588?include=energy
Arhgap28	0.981	http://api.brain-map.org/grid_data/download/77620437?include=energy
1110018G07Rik	0.981	http://api.brain-map.org/grid_data/download/69352914?include=energy
BC107364	0.981	http://api.brain-map.org/grid_data/download/70429076?include=energy
Gpr75	0.981	http://api.brain-map.org/grid_data/download/69257648?include=energy
Pdgfd	0.981	http://api.brain-map.org/grid_data/download/1910?include=energy
Arf4	0.981	http://api.brain-map.org/grid_data/download/69526785?include=energy
Spop	0.981	http://api.brain-map.org/grid_data/download/69885702?include=energy
Atp2a2	0.981	http://api.brain-map.org/grid_data/download/74881549?include=energy
Nudt21	0.981	http://api.brain-map.org/grid_data/download/2115?include=energy
Tgfbr1	0.981	http://api.brain-map.org/grid_data/download/1054?include=energy
Frmd4a	0.981	http://api.brain-map.org/grid_data/download/69672581?include=energy
Lrrc45	0.981	http://api.brain-map.org/grid_data/download/67810609?include=energy
Cox6c	0.981	http://api.brain-map.org/grid_data/download/74641307?include=energy
Flrt3	0.981	http://api.brain-map.org/grid_data/download/73931404?include=energy
Rpsa	0.981	http://api.brain-map.org/grid_data/download/77620255?include=energy
F630119A15Rik*	0.981	http://api.brain-map.org/grid_data/download/73748923?include=energy
Tmbim6	0.981	http://api.brain-map.org/grid_data/download/2663?include=energy
Ubr2	0.981	http://api.brain-map.org/grid_data/download/71608349?include=energy
Oat	0.981	http://api.brain-map.org/grid_data/download/79539640?include=energy
Dmrt3	0.981	http://api.brain-map.org/grid_data/download/74819342?include=energy
Nipsnap1	0.981	http://api.brain-map.org/grid_data/download/69059970?include=energy
AA474331	0.981	http://api.brain-map.org/grid_data/download/74735367?include=energy
2310040G07Rik	0.981	http://api.brain-map.org/grid_data/download/74359383?include=energy
LOC545133	0.981	http://api.brain-map.org/grid_data/download/69262305?include=energy
Mif4gd	0.981	http://api.brain-map.org/grid_data/download/68498295?include=energy
2210010C04Rik	0.981	http://api.brain-map.org/grid_data/download/69202244?include=energy
Slc15a2	0.981	http://api.brain-map.org/grid_data/download/68743943?include=energy
Klf6	0.981	http://api.brain-map.org/grid_data/download/70431247?include=energy
Agbl1	0.981	http://api.brain-map.org/grid_data/download/71580204?include=energy
Rhbdl2	0.981	http://api.brain-map.org/grid_data/download/75990430?include=energy
Syp	0.981	http://api.brain-map.org/grid_data/download/74431646?include=energy
Psmb5	0.981	http://api.brain-map.org/grid_data/download/81599656?include=energy
Prm3	0.981	http://api.brain-map.org/grid_data/download/74822868?include=energy
LOC280640	0.981	http://api.brain-map.org/grid_data/download/74743459?include=energy
Igdcc4	0.981	http://api.brain-map.org/grid_data/download/71234631?include=energy
4930547M16Rik	0.981	http://api.brain-map.org/grid_data/download/73731229?include=energy
Adora3	0.981	http://api.brain-map.org/grid_data/download/69236801?include=energy
Ffar3	0.981	http://api.brain-map.org/grid_data/download/74822610?include=energy
Mycbp2	0.981	http://api.brain-map.org/grid_data/download/71234742?include=energy
Pcmtd1	0.981	http://api.brain-map.org/grid_data/download/68743859?include=energy
Vamp3	0.981	http://api.brain-map.org/grid_data/download/70919812?include=energy
2410004P03Rik	0.98	http://api.brain-map.org/grid_data/download/71493020?include=energy
Pex10	0.98	http://api.brain-map.org/grid_data/download/70295912?include=energy
Calm1	0.98	http://api.brain-map.org/grid_data/download/179?include=energy
Ndufaf2	0.98	http://api.brain-map.org/grid_data/download/74988362?include=energy
Trex1	0.98	http://api.brain-map.org/grid_data/download/68302323?include=energy
Tdrkh	0.98	http://api.brain-map.org/grid_data/download/69012894?include=energy
Dync2li1	0.98	http://api.brain-map.org/grid_data/download/378552?include=energy
Olfr846	0.98	http://api.brain-map.org/grid_data/download/74735039?include=energy
App	0.98	http://api.brain-map.org/grid_data/download/79591705?include=energy
Ins1	0.98	http://api.brain-map.org/grid_data/download/70724852?include=energy
Macf1	0.98	http://api.brain-map.org/grid_data/download/69736400?include=energy
Alg8	0.98	http://api.brain-map.org/grid_data/download/69174046?include=energy
Lman2	0.98	http://api.brain-map.org/grid_data/download/73718047?include=energy
Neurl3	0.98	http://api.brain-map.org/grid_data/download/69015182?include=energy
Clrn3	0.98	http://api.brain-map.org/grid_data/download/2023?include=energy
Scrib	0.98	http://api.brain-map.org/grid_data/download/69837873?include=energy
Tspo	0.98	http://api.brain-map.org/grid_data/download/79556668?include=energy
Prkar1a	0.98	http://api.brain-map.org/grid_data/download/70429369?include=energy
Aplp1	0.98	http://api.brain-map.org/grid_data/download/69015613?include=energy
Eng	0.98	http://api.brain-map.org/grid_data/download/68637957?include=energy
Mpzl3	0.98	http://api.brain-map.org/grid_data/download/69448710?include=energy
Amd1	0.98	http://api.brain-map.org/grid_data/download/75826627?include=energy
Cylc1	0.98	http://api.brain-map.org/grid_data/download/74579963?include=energy
Dgki	0.98	http://api.brain-map.org/grid_data/download/74658043?include=energy
Ddt	0.98	http://api.brain-map.org/grid_data/download/75081209?include=energy
Zfp568	0.98	http://api.brain-map.org/grid_data/download/70566337?include=energy
AK129341	0.98	http://api.brain-map.org/grid_data/download/69526492?include=energy
Cntn6	0.98	http://api.brain-map.org/grid_data/download/74882147?include=energy
Uckl1	0.98	http://api.brain-map.org/grid_data/download/68341579?include=energy
Stam2	0.98	http://api.brain-map.org/grid_data/download/564651?include=energy
Arl3	0.98	http://api.brain-map.org/grid_data/download/75043143?include=energy
Bmf	0.98	http://api.brain-map.org/grid_data/download/69529346?include=energy
Gm17250	0.98	http://api.brain-map.org/grid_data/download/70295859?include=energy
Hras1	0.98	http://api.brain-map.org/grid_data/download/67979018?include=energy
Hadhb	0.98	http://api.brain-map.org/grid_data/download/76115729?include=energy
Trap1	0.98	http://api.brain-map.org/grid_data/download/79556700?include=energy
Pid1	0.98	http://api.brain-map.org/grid_data/download/73635974?include=energy
Nhp2	0.98	http://api.brain-map.org/grid_data/download/68563134?include=energy
Gm4958	0.98	http://api.brain-map.org/grid_data/download/71489811?include=energy
1700003M02Rik	0.98	http://api.brain-map.org/grid_data/download/68798097?include=energy
Ubqln1	0.98	http://api.brain-map.org/grid_data/download/67814217?include=energy
Dclk2	0.98	http://api.brain-map.org/grid_data/download/68862030?include=energy
Srxn1	0.98	http://api.brain-map.org/grid_data/download/70429315?include=energy
Casp9	0.98	http://api.brain-map.org/grid_data/download/1681?include=energy
Gpi1	0.98	http://api.brain-map.org/grid_data/download/100144120?include=energy
Atp1a4	0.98	http://api.brain-map.org/grid_data/download/74881548?include=energy
D630021H01Rik	0.98	http://api.brain-map.org/grid_data/download/69608995?include=energy
Fkbp2	0.98	http://api.brain-map.org/grid_data/download/70528665?include=energy
Epo	0.98	http://api.brain-map.org/grid_data/download/70239123?include=energy
Gpr149	0.979	http://api.brain-map.org/grid_data/download/71283586?include=energy
Rps21	0.979	http://api.brain-map.org/grid_data/download/74425523?include=energy
Icam2	0.979	http://api.brain-map.org/grid_data/download/68162269?include=energy
4930563I02Rik	0.979	http://api.brain-map.org/grid_data/download/72076671?include=energy
Slc4a5	0.979	http://api.brain-map.org/grid_data/download/71496239?include=energy
Rims2	0.979	http://api.brain-map.org/grid_data/download/73424184?include=energy
Fcho1	0.979	http://api.brain-map.org/grid_data/download/68161462?include=energy
Gata2	0.979	http://api.brain-map.org/grid_data/download/100?include=energy
Mettl16	0.979	http://api.brain-map.org/grid_data/download/69262327?include=energy
Plk5	0.979	http://api.brain-map.org/grid_data/download/74988743?include=energy
Lzic	0.979	http://api.brain-map.org/grid_data/download/276064?include=energy
Ubl4b	0.979	http://api.brain-map.org/grid_data/download/73818790?include=energy
Cd84	0.979	http://api.brain-map.org/grid_data/download/69540629?include=energy
Azi1	0.979	http://api.brain-map.org/grid_data/download/72472487?include=energy
Arl8a	0.979	http://api.brain-map.org/grid_data/download/71495807?include=energy
Trpc3	0.979	http://api.brain-map.org/grid_data/download/74821603?include=energy
Klb	0.979	http://api.brain-map.org/grid_data/download/70231086?include=energy
Gm829	0.979	http://api.brain-map.org/grid_data/download/71764332?include=energy
Itih3	0.979	http://api.brain-map.org/grid_data/download/600?include=energy
Pcdha1	0.979	http://api.brain-map.org/grid_data/download/79354800?include=energy
Olfr390	0.979	http://api.brain-map.org/grid_data/download/73653404?include=energy
Pex5l	0.979	http://api.brain-map.org/grid_data/download/74581447?include=energy
D930016D06Rik	0.979	http://api.brain-map.org/grid_data/download/73906376?include=energy
Ppp1r27	0.979	http://api.brain-map.org/grid_data/download/74581092?include=energy
Snx9	0.979	http://api.brain-map.org/grid_data/download/69885636?include=energy
1700010H22Rik	0.979	http://api.brain-map.org/grid_data/download/75934507?include=energy
Cd5	0.979	http://api.brain-map.org/grid_data/download/68301981?include=energy
Fnbp1l	0.979	http://api.brain-map.org/grid_data/download/70560266?include=energy
H2afy	0.979	http://api.brain-map.org/grid_data/download/68269502?include=energy
Phka1	0.979	http://api.brain-map.org/grid_data/download/70795409?include=energy
Atxn2l	0.979	http://api.brain-map.org/grid_data/download/68797948?include=energy
Treh	0.979	http://api.brain-map.org/grid_data/download/68446015?include=energy
Ccdc113	0.979	http://api.brain-map.org/grid_data/download/69838722?include=energy
Csde1	0.979	http://api.brain-map.org/grid_data/download/72182999?include=energy
Dhx32	0.979	http://api.brain-map.org/grid_data/download/68193037?include=energy
Rchy1	0.979	http://api.brain-map.org/grid_data/download/68444708?include=energy
LOC434366	0.979	http://api.brain-map.org/grid_data/download/70920129?include=energy
Mbd6	0.979	http://api.brain-map.org/grid_data/download/69608441?include=energy
Lgals3bp	0.979	http://api.brain-map.org/grid_data/download/69736088?include=energy
Fbxo31	0.979	http://api.brain-map.org/grid_data/download/74300843?include=energy
Olfr745	0.979	http://api.brain-map.org/grid_data/download/74734932?include=energy
Cnksr3	0.979	http://api.brain-map.org/grid_data/download/74273121?include=energy
Gpm6a	0.979	http://api.brain-map.org/grid_data/download/68080704?include=energy
Olfr1052	0.979	http://api.brain-map.org/grid_data/download/74720585?include=energy
Lim2	0.979	http://api.brain-map.org/grid_data/download/71832976?include=energy
Mup5	0.979	http://api.brain-map.org/grid_data/download/73997144?include=energy
Hspb7	0.979	http://api.brain-map.org/grid_data/download/579?include=energy
Pisd	0.979	http://api.brain-map.org/grid_data/download/69446316?include=energy
Adam11	0.979	http://api.brain-map.org/grid_data/download/69837407?include=energy
Atxn7l2	0.979	http://api.brain-map.org/grid_data/download/69202956?include=energy
Fbxo9	0.979	http://api.brain-map.org/grid_data/download/74800954?include=energy
Lrrc7	0.979	http://api.brain-map.org/grid_data/download/69526468?include=energy
Nrbp1	0.979	http://api.brain-map.org/grid_data/download/71763528?include=energy
Cnfn	0.979	http://api.brain-map.org/grid_data/download/100146167?include=energy
Srpr	0.979	http://api.brain-map.org/grid_data/download/1593?include=energy
Cwf19l2	0.979	http://api.brain-map.org/grid_data/download/70720258?include=energy
Gm5082	0.979	http://api.brain-map.org/grid_data/download/71569151?include=energy
Hist2h2bb	0.979	http://api.brain-map.org/grid_data/download/69735048?include=energy
LOC434320	0.978	http://api.brain-map.org/grid_data/download/74359706?include=energy
4931403M11Rik	0.978	http://api.brain-map.org/grid_data/download/70218456?include=energy
Atp2b2	0.978	http://api.brain-map.org/grid_data/download/70724583?include=energy
Tmprss4	0.978	http://api.brain-map.org/grid_data/download/2684?include=energy
Defb20	0.978	http://api.brain-map.org/grid_data/download/72183001?include=energy
Ccdc81	0.978	http://api.brain-map.org/grid_data/download/75986049?include=energy
Uba6	0.978	http://api.brain-map.org/grid_data/download/74819486?include=energy
Gys1	0.978	http://api.brain-map.org/grid_data/download/69734863?include=energy
Zfp78	0.978	http://api.brain-map.org/grid_data/download/74821639?include=energy
Chid1	0.978	http://api.brain-map.org/grid_data/download/69059977?include=energy
Ceacam16	0.978	http://api.brain-map.org/grid_data/download/71212392?include=energy
Chchd3	0.978	http://api.brain-map.org/grid_data/download/67870084?include=energy
Atp1b2	0.978	http://api.brain-map.org/grid_data/download/74819255?include=energy
Rbm4b	0.978	http://api.brain-map.org/grid_data/download/638701?include=energy
Rufy3	0.978	http://api.brain-map.org/grid_data/download/73817983?include=energy
Cox19	0.978	http://api.brain-map.org/grid_data/download/75828190?include=energy
Cops4	0.978	http://api.brain-map.org/grid_data/download/74882166?include=energy
Gm255	0.978	http://api.brain-map.org/grid_data/download/70295733?include=energy
Il1rl1	0.978	http://api.brain-map.org/grid_data/download/75694311?include=energy
Gpr123	0.978	http://api.brain-map.org/grid_data/download/71924056?include=energy
Ece1	0.978	http://api.brain-map.org/grid_data/download/69671851?include=energy
Tesc	0.978	http://api.brain-map.org/grid_data/download/70634149?include=energy
Zdbf2	0.978	http://api.brain-map.org/grid_data/download/74047400?include=energy
Pcx	0.978	http://api.brain-map.org/grid_data/download/69014512?include=energy
E030010A14Rik	0.978	http://api.brain-map.org/grid_data/download/70565482?include=energy
BC037034	0.978	http://api.brain-map.org/grid_data/download/378548?include=energy
LOC434354	0.978	http://api.brain-map.org/grid_data/download/70725585?include=energy
Pcdh10	0.978	http://api.brain-map.org/grid_data/download/71668995?include=energy
4930571K11Rik*	0.978	http://api.brain-map.org/grid_data/download/73870533?include=energy
Morc2b	0.978	http://api.brain-map.org/grid_data/download/70218540?include=energy
Efhc1	0.978	http://api.brain-map.org/grid_data/download/70239087?include=energy
Abhd12	0.978	http://api.brain-map.org/grid_data/download/70445532?include=energy
4833415N18Rik	0.978	http://api.brain-map.org/grid_data/download/71022912?include=energy
Dolpp1	0.978	http://api.brain-map.org/grid_data/download/69173268?include=energy
Lrrn1	0.978	http://api.brain-map.org/grid_data/download/72129293?include=energy
Actg2	0.978	http://api.brain-map.org/grid_data/download/77620463?include=energy
LOC433816	0.978	http://api.brain-map.org/grid_data/download/70786839?include=energy
Lphn3	0.978	http://api.brain-map.org/grid_data/download/74514294?include=energy
Ccdc104	0.978	http://api.brain-map.org/grid_data/download/76081944?include=energy
LOC435303	0.978	http://api.brain-map.org/grid_data/download/70725640?include=energy
Ttc14	0.978	http://api.brain-map.org/grid_data/download/74265183?include=energy
Eef1g	0.978	http://api.brain-map.org/grid_data/download/74819238?include=energy
Rad50	0.978	http://api.brain-map.org/grid_data/download/69817904?include=energy

Slc43a3	0.978	http://api.brain-map.org/grid_data/download/69873988?include=energy
2810046L04Rik	0.978	http://api.brain-map.org/grid_data/download/71232628?include=energy
Mamld1	0.977	http://api.brain-map.org/grid_data/download/70276843?include=energy
4930402H24Rik	0.977	http://api.brain-map.org/grid_data/download/70218085?include=energy
Snapin	0.977	http://api.brain-map.org/grid_data/download/69549659?include=energy
Ajap1	0.977	http://api.brain-map.org/grid_data/download/71717560?include=energy
Itk	0.977	http://api.brain-map.org/grid_data/download/69735634?include=energy
Rps2	0.977	http://api.brain-map.org/grid_data/download/100146146?include=energy
Trav3-3	0.977	http://api.brain-map.org/grid_data/download/71670720?include=energy
Msl3l2	0.977	http://api.brain-map.org/grid_data/download/71724780?include=energy
BC034902	0.977	http://api.brain-map.org/grid_data/download/71656090?include=energy
Msi2	0.977	http://api.brain-map.org/grid_data/download/73616034?include=energy
Mm.359809	0.977	http://api.brain-map.org/grid_data/download/71209940?include=energy
Zmynd19	0.977	http://api.brain-map.org/grid_data/download/79393531?include=energy
Rpl23a	0.977	http://api.brain-map.org/grid_data/download/100144177?include=energy
Gnas	0.977	http://api.brain-map.org/grid_data/download/509?include=energy
Pcdhb8	0.977	http://api.brain-map.org/grid_data/download/70784860?include=energy
Kbtbd4	0.977	http://api.brain-map.org/grid_data/download/68203495?include=energy
Chdh	0.977	http://api.brain-map.org/grid_data/download/68910964?include=energy
Fam176a	0.977	http://api.brain-map.org/grid_data/download/68666649?include=energy
Scyl1	0.977	http://api.brain-map.org/grid_data/download/68563080?include=energy
Csn1s2b	0.977	http://api.brain-map.org/grid_data/download/532593?include=energy
Klkb1	0.977	http://api.brain-map.org/grid_data/download/76098400?include=energy
Gpaa1	0.977	http://api.brain-map.org/grid_data/download/79539606?include=energy
Dbx1	0.977	http://api.brain-map.org/grid_data/download/73497113?include=energy
Tmem150c	0.977	http://api.brain-map.org/grid_data/download/77340478?include=energy
Crispld2	0.977	http://api.brain-map.org/grid_data/download/69201654?include=energy
Morc3	0.977	http://api.brain-map.org/grid_data/download/1311?include=energy
Araf	0.977	http://api.brain-map.org/grid_data/download/130514?include=energy
Pcsk7	0.977	http://api.brain-map.org/grid_data/download/69059805?include=energy
LOC433666	0.977	http://api.brain-map.org/grid_data/download/70786646?include=energy
Fam168a	0.977	http://api.brain-map.org/grid_data/download/69527980?include=energy
G0s2	0.977	http://api.brain-map.org/grid_data/download/71232703?include=energy
Scrn1	0.977	http://api.brain-map.org/grid_data/download/74357551?include=energy
Rabgef1	0.977	http://api.brain-map.org/grid_data/download/67765714?include=energy
Ifi27l1	0.977	http://api.brain-map.org/grid_data/download/74581074?include=energy
Adam6a	0.977	http://api.brain-map.org/grid_data/download/74735103?include=energy
Sim2	0.977	http://api.brain-map.org/grid_data/download/71325462?include=energy
5031425E22Rik	0.977	http://api.brain-map.org/grid_data/download/70787008?include=energy
Mob1b	0.977	http://api.brain-map.org/grid_data/download/70200854?include=energy
Ptges3	0.977	http://api.brain-map.org/grid_data/download/75214937?include=energy
Wdr45	0.977	http://api.brain-map.org/grid_data/download/75198190?include=energy
Umps	0.977	http://api.brain-map.org/grid_data/download/69080001?include=energy
Mrps7	0.977	http://api.brain-map.org/grid_data/download/69105164?include=energy
Pfdn2	0.977	http://api.brain-map.org/grid_data/download/68666601?include=energy
LOC434519	0.977	http://api.brain-map.org/grid_data/download/74736261?include=energy
Ppip5k2	0.977	http://api.brain-map.org/grid_data/download/68667479?include=energy
Fgf20	0.977	http://api.brain-map.org/grid_data/download/434?include=energy
Rwdd4a	0.977	http://api.brain-map.org/grid_data/download/74277687?include=energy
Igsf5	0.977	http://api.brain-map.org/grid_data/download/592?include=energy
4933424B01Rik	0.977	http://api.brain-map.org/grid_data/download/68193034?include=energy
Ubqln2	0.977	http://api.brain-map.org/grid_data/download/76003322?include=energy
St7l	0.977	http://api.brain-map.org/grid_data/download/68798079?include=energy
Tars2	0.977	http://api.brain-map.org/grid_data/download/71832963?include=energy
Hsdl1	0.977	http://api.brain-map.org/grid_data/download/70542931?include=energy
Nefl	0.977	http://api.brain-map.org/grid_data/download/73512198?include=energy
Mm.171079	0.977	http://api.brain-map.org/grid_data/download/71152198?include=energy
9230109C12Rik*	0.977	http://api.brain-map.org/grid_data/download/73870587?include=energy
Rrs1	0.977	http://api.brain-map.org/grid_data/download/67978480?include=energy
Trim68	0.977	http://api.brain-map.org/grid_data/download/68633449?include=energy
Ift57	0.977	http://api.brain-map.org/grid_data/download/69672156?include=energy
4732419C18Rik	0.976	http://api.brain-map.org/grid_data/download/70300481?include=energy
Heatr5b	0.976	http://api.brain-map.org/grid_data/download/70560265?include=energy
Cd74	0.976	http://api.brain-map.org/grid_data/download/67979029?include=energy
Dynlt1b	0.976	http://api.brain-map.org/grid_data/download/71489780?include=energy
Itga1	0.976	http://api.brain-map.org/grid_data/download/73949740?include=energy
Gdpd5	0.976	http://api.brain-map.org/grid_data/download/80514099?include=energy
Tjap1	0.976	http://api.brain-map.org/grid_data/download/77800915?include=energy
Bst2	0.976	http://api.brain-map.org/grid_data/download/69529430?include=energy
Map3k8	0.976	http://api.brain-map.org/grid_data/download/69752029?include=energy
LOC436474	0.976	http://api.brain-map.org/grid_data/download/74736372?include=energy
Flna	0.976	http://api.brain-map.org/grid_data/download/70565563?include=energy
Chordc1	0.976	http://api.brain-map.org/grid_data/download/69015763?include=energy
Rslcan18	0.976	http://api.brain-map.org/grid_data/download/70611281?include=energy
Fam195b	0.976	http://api.brain-map.org/grid_data/download/69528100?include=energy
Smarca1	0.976	http://api.brain-map.org/grid_data/download/69816888?include=energy
Ehd2	0.976	http://api.brain-map.org/grid_data/download/71735070?include=energy
Uck1	0.976	http://api.brain-map.org/grid_data/download/293666?include=energy
LOC436342	0.976	http://api.brain-map.org/grid_data/download/74635662?include=energy
Fam132a	0.976	http://api.brain-map.org/grid_data/download/71283428?include=energy
Mast1	0.976	http://api.brain-map.org/grid_data/download/68798903?include=energy
Actr5	0.976	http://api.brain-map.org/grid_data/download/69837477?include=energy
5730403B10Rik	0.976	http://api.brain-map.org/grid_data/download/413811?include=energy
Dcstamp	0.976	http://api.brain-map.org/grid_data/download/74579957?include=energy
Hdac2	0.976	http://api.brain-map.org/grid_data/download/100145444?include=energy
Slc38a3	0.976	http://api.brain-map.org/grid_data/download/70436729?include=energy
Idh2	0.976	http://api.brain-map.org/grid_data/download/69028683?include=energy
Elmod1	0.976	http://api.brain-map.org/grid_data/download/71924269?include=energy
Lbx2	0.976	http://api.brain-map.org/grid_data/download/69816613?include=energy
4930534B04Rik	0.976	http://api.brain-map.org/grid_data/download/75986070?include=energy
Myo1f	0.976	http://api.brain-map.org/grid_data/download/68845347?include=energy
Bcl2l2	0.976	http://api.brain-map.org/grid_data/download/67765710?include=energy
E230001N04Rik	0.976	http://api.brain-map.org/grid_data/download/69671774?include=energy
Armc4	0.976	http://api.brain-map.org/grid_data/download/74076210?include=energy
Ddit3	0.976	http://api.brain-map.org/grid_data/download/564779?include=energy
Sharpin	0.976	http://api.brain-map.org/grid_data/download/68193064?include=energy
Atp13a4	0.976	http://api.brain-map.org/grid_data/download/69838517?include=energy
Inpp5j	0.976	http://api.brain-map.org/grid_data/download/77869865?include=energy
Insig1	0.976	http://api.brain-map.org/grid_data/download/75041453?include=energy
Cttnbp2nl	0.976	http://api.brain-map.org/grid_data/download/532609?include=energy
Mm.325302	0.976	http://api.brain-map.org/grid_data/download/71158964?include=energy
Eif4g1	0.976	http://api.brain-map.org/grid_data/download/70920063?include=energy
Tgoln1	0.976	http://api.brain-map.org/grid_data/download/69549673?include=energy
Ctsd	0.976	http://api.brain-map.org/grid_data/download/69028479?include=energy
Itgb7	0.976	http://api.brain-map.org/grid_data/download/86359?include=energy
Cxcl13	0.976	http://api.brain-map.org/grid_data/download/74819234?include=energy
Setd8	0.976	http://api.brain-map.org/grid_data/download/73584831?include=energy
Wnt3a	0.976	http://api.brain-map.org/grid_data/download/77800899?include=energy
Pkmyt1	0.976	http://api.brain-map.org/grid_data/download/76135614?include=energy
Ptprn	0.976	http://api.brain-map.org/grid_data/download/71381133?include=energy
Med31	0.976	http://api.brain-map.org/grid_data/download/68410249?include=energy
2610002J02Rik	0.976	http://api.brain-map.org/grid_data/download/70812972?include=energy
Tlr4	0.976	http://api.brain-map.org/grid_data/download/378542?include=energy
Atp5j2	0.976	http://api.brain-map.org/grid_data/download/75198094?include=energy
Wnt3	0.976	http://api.brain-map.org/grid_data/download/2731?include=energy
Inca1	0.976	http://api.brain-map.org/grid_data/download/69526414?include=energy
Tmem125	0.975	http://api.brain-map.org/grid_data/download/68545977?include=energy
Supv3l1	0.975	http://api.brain-map.org/grid_data/download/69887475?include=energy
Fh1	0.975	http://api.brain-map.org/grid_data/download/355890?include=energy
A530057D15Rik*	0.975	http://api.brain-map.org/grid_data/download/75042238?include=energy
Por	0.975	http://api.brain-map.org/grid_data/download/69549250?include=energy
Rgl3	0.975	http://api.brain-map.org/grid_data/download/74003386?include=energy
Syt15	0.975	http://api.brain-map.org/grid_data/download/71808516?include=energy
Dbnl	0.975	http://api.brain-map.org/grid_data/download/74819497?include=energy
Klrb1f	0.975	http://api.brain-map.org/grid_data/download/73513365?include=energy
Cbs	0.975	http://api.brain-map.org/grid_data/download/1335?include=energy
Lbx1	0.975	http://api.brain-map.org/grid_data/download/70723492?include=energy
Cib4	0.975	http://api.brain-map.org/grid_data/download/71378651?include=energy
Il10	0.975	http://api.brain-map.org/grid_data/download/76003673?include=energy
Rnf145	0.975	http://api.brain-map.org/grid_data/download/69407540?include=energy
Naaladl1	0.975	http://api.brain-map.org/grid_data/download/70596017?include=energy
Kcnd2	0.975	http://api.brain-map.org/grid_data/download/71020432?include=energy
Gm1094	0.975	http://api.brain-map.org/grid_data/download/75692945?include=energy
Rcc2	0.975	http://api.brain-map.org/grid_data/download/69202938?include=energy
LOC433896	0.975	http://api.brain-map.org/grid_data/download/70787071?include=energy
Rpl39l	0.975	http://api.brain-map.org/grid_data/download/70723710?include=energy
5330416C01Rik	0.975	http://api.brain-map.org/grid_data/download/69443355?include=energy
D4Wsu53e	0.975	http://api.brain-map.org/grid_data/download/74819463?include=energy
Narf	0.975	http://api.brain-map.org/grid_data/download/583563?include=energy
Cdc42	0.975	http://api.brain-map.org/grid_data/download/71021145?include=energy
B230209K01Rik	0.975	http://api.brain-map.org/grid_data/download/71809061?include=energy
Zfp507	0.975	http://api.brain-map.org/grid_data/download/70527945?include=energy
Btbd11	0.975	http://api.brain-map.org/grid_data/download/73769325?include=energy
Tro	0.975	http://api.brain-map.org/grid_data/download/74819332?include=energy
Rpl8	0.975	http://api.brain-map.org/grid_data/download/74749928?include=energy
Tmsb4x	0.975	http://api.brain-map.org/grid_data/download/77792010?include=energy
Ruvbl1	0.975	http://api.brain-map.org/grid_data/download/70429405?include=energy
Rc3h1	0.975	http://api.brain-map.org/grid_data/download/71161525?include=energy
Il6st	0.975	http://api.brain-map.org/grid_data/download/69735431?include=energy
Mgrn1	0.975	http://api.brain-map.org/grid_data/download/74427051?include=energy
En2	0.975	http://api.brain-map.org/grid_data/download/74988733?include=energy
Zfp319	0.975	http://api.brain-map.org/grid_data/download/73927664?include=energy
F830010A05Rik*	0.975	http://api.brain-map.org/grid_data/download/73731364?include=energy
Phtf1	0.975	http://api.brain-map.org/grid_data/download/68798507?include=energy
Podxl	0.975	http://api.brain-map.org/grid_data/download/69816392?include=energy
Fam203a	0.975	http://api.brain-map.org/grid_data/download/69174391?include=energy
Rdh14	0.975	http://api.brain-map.org/grid_data/download/75043157?include=energy
Bcmo1	0.975	http://api.brain-map.org/grid_data/download/70231809?include=energy
Itfg1	0.975	http://api.brain-map.org/grid_data/download/112194079?include=energy
Cox8a	0.975	http://api.brain-map.org/grid_data/download/69568986?include=energy
7530420F21Rik	0.974	http://api.brain-map.org/grid_data/download/69445433?include=energy
Shroom4	0.974	http://api.brain-map.org/grid_data/download/71808637?include=energy
Ccdc58	0.974	http://api.brain-map.org/grid_data/download/69236694?include=energy
Ttc3	0.974	http://api.brain-map.org/grid_data/download/1079?include=energy
Tsx	0.974	http://api.brain-map.org/grid_data/download/75850261?include=energy
Ranbp1	0.974	http://api.brain-map.org/grid_data/download/69174298?include=energy
Wbp7	0.974	http://api.brain-map.org/grid_data/download/70203513?include=energy
Narg2	0.974	http://api.brain-map.org/grid_data/download/69169463?include=energy
Atp5c1	0.974	http://api.brain-map.org/grid_data/download/73513447?include=energy
Siglec5	0.974	http://api.brain-map.org/grid_data/download/71808753?include=energy
Mt1	0.974	http://api.brain-map.org/grid_data/download/67767450?include=energy
Mcoln2	0.974	http://api.brain-map.org/grid_data/download/248562?include=energy
A730009L09Rik	0.974	http://api.brain-map.org/grid_data/download/70305323?include=energy
LOC433975	0.974	http://api.brain-map.org/grid_data/download/72076551?include=energy
Zscan5b	0.974	http://api.brain-map.org/grid_data/download/70303492?include=energy
LOC434238	0.974	http://api.brain-map.org/grid_data/download/70787615?include=energy
Nfam1	0.974	http://api.brain-map.org/grid_data/download/71493171?include=energy
Clic3	0.974	http://api.brain-map.org/grid_data/download/70543074?include=energy
B230118H07Rik	0.974	http://api.brain-map.org/grid_data/download/71763735?include=energy
Cacng5	0.974	http://api.brain-map.org/grid_data/download/70560341?include=energy
Pisd-ps1	0.974	http://api.brain-map.org/grid_data/download/69174529?include=energy
Gm14206	0.974	http://api.brain-map.org/grid_data/download/70725055?include=energy
Myd88	0.974	http://api.brain-map.org/grid_data/download/68798151?include=energy
LOC544685	0.974	http://api.brain-map.org/grid_data/download/74736390?include=energy
Carkd	0.974	http://api.brain-map.org/grid_data/download/69080067?include=energy
Cast	0.974	http://api.brain-map.org/grid_data/download/70928199?include=energy
Fam163a	0.974	http://api.brain-map.org/grid_data/download/70305245?include=energy
Ncam1	0.974	http://api.brain-map.org/grid_data/download/80342134?include=energy
D330028D13Rik	0.974	http://api.brain-map.org/grid_data/download/69014991?include=energy
Ccnt2	0.974	http://api.brain-map.org/grid_data/download/68666840?include=energy
1110004F10Rik	0.974	http://api.brain-map.org/grid_data/download/69352872?include=energy
4930595M18Rik	0.974	http://api.brain-map.org/grid_data/download/70218444?include=energy
Gadd45a	0.974	http://api.brain-map.org/grid_data/download/576421?include=energy
Taar5	0.974	http://api.brain-map.org/grid_data/download/100144941?include=energy
Strbp	0.974	http://api.brain-map.org/grid_data/download/71724726?include=energy
Prss50	0.974	http://api.brain-map.org/grid_data/download/76003338?include=energy
1700092C17Rik	0.974	http://api.brain-map.org/grid_data/download/71920159?include=energy
Ssbp2	0.974	http://api.brain-map.org/grid_data/download/70928385?include=energy
Rnaset2b	0.974	http://api.brain-map.org/grid_data/download/74988459?include=energy
Eef1a2	0.974	http://api.brain-map.org/grid_data/download/79544840?include=energy
Acad11	0.974	http://api.brain-map.org/grid_data/download/69549625?include=energy
Upk3b	0.974	http://api.brain-map.org/grid_data/download/72004456?include=energy
Fastkd1	0.974	http://api.brain-map.org/grid_data/download/71281331?include=energy
1010001N08Rik	0.974	http://api.brain-map.org/grid_data/download/70200842?include=energy
Tekt2	0.974	http://api.brain-map.org/grid_data/download/79544786?include=energy
Il22ra1	0.974	http://api.brain-map.org/grid_data/download/71832913?include=energy
Diap1	0.974	http://api.brain-map.org/grid_data/download/69626885?include=energy
Ido1	0.974	http://api.brain-map.org/grid_data/download/68924407?include=energy
Ldb1	0.974	http://api.brain-map.org/grid_data/download/69059844?include=energy
Rad23b	0.974	http://api.brain-map.org/grid_data/download/881?include=energy
Il4i1	0.974	http://api.brain-map.org/grid_data/download/69735407?include=energy
Ocln	0.974	http://api.brain-map.org/grid_data/download/79394328?include=energy
Phkg2	0.974	http://api.brain-map.org/grid_data/download/70795397?include=energy
1810046K07Rik	0.974	http://api.brain-map.org/grid_data/download/70201600?include=energy
Zfp952	0.974	http://api.brain-map.org/grid_data/download/68844863?include=energy
Nsdhl	0.974	http://api.brain-map.org/grid_data/download/72007933?include=energy
Snap25	0.974	http://api.brain-map.org/grid_data/download/1854?include=energy
Ippk	0.973	http://api.brain-map.org/grid_data/download/69201648?include=energy
4930572J05Rik	0.973	http://api.brain-map.org/grid_data/download/72008306?include=energy
Bfsp2	0.973	http://api.brain-map.org/grid_data/download/71495968?include=energy
Tsnaxip1	0.973	http://api.brain-map.org/grid_data/download/70302848?include=energy
Abcc10	0.973	http://api.brain-map.org/grid_data/download/70305377?include=energy
Gjd4	0.973	http://api.brain-map.org/grid_data/download/100144859?include=energy
Zfp160	0.973	http://api.brain-map.org/grid_data/download/71627750?include=energy
Rbm5	0.973	http://api.brain-map.org/grid_data/download/69818030?include=energy
3010003L10Rik	0.973	http://api.brain-map.org/grid_data/download/73497771?include=energy
Uqcrb	0.973	http://api.brain-map.org/grid_data/download/323595?include=energy
Mad1l1	0.973	http://api.brain-map.org/grid_data/download/70928294?include=energy
Cdr2l	0.973	http://api.brain-map.org/grid_data/download/69608402?include=energy
Arih1	0.973	http://api.brain-map.org/grid_data/download/70430818?include=energy
Gzmf	0.973	http://api.brain-map.org/grid_data/download/70724774?include=energy
Bsph2	0.973	http://api.brain-map.org/grid_data/download/74580907?include=energy
Psma3	0.973	http://api.brain-map.org/grid_data/download/69817402?include=energy
Rusc2	0.973	http://api.brain-map.org/grid_data/download/69104579?include=energy
Timm8a2	0.973	http://api.brain-map.org/grid_data/download/70565975?include=energy
Dnajb8	0.973	http://api.brain-map.org/grid_data/download/75860950?include=energy
Nkrf	0.973	http://api.brain-map.org/grid_data/download/71283580?include=energy
Cer1	0.973	http://api.brain-map.org/grid_data/download/2091?include=energy
Klhdc3	0.973	http://api.brain-map.org/grid_data/download/68637491?include=energy
BC005624	0.973	http://api.brain-map.org/grid_data/download/67936203?include=energy
Skil	0.973	http://api.brain-map.org/grid_data/download/69262315?include=energy
Mettl15	0.973	http://api.brain-map.org/grid_data/download/69352762?include=energy
Rnf6	0.973	http://api.brain-map.org/grid_data/download/69353170?include=energy
Serpinb10	0.973	http://api.brain-map.org/grid_data/download/68633422?include=energy
Foxred1	0.973	http://api.brain-map.org/grid_data/download/68157408?include=energy
Pygl	0.973	http://api.brain-map.org/grid_data/download/75826661?include=energy
Grsf1	0.973	http://api.brain-map.org/grid_data/download/73994670?include=energy
Zfyve21	0.973	http://api.brain-map.org/grid_data/download/76097729?include=energy
Tnfrsf26	0.973	http://api.brain-map.org/grid_data/download/70302563?include=energy
Ilvbl	0.973	http://api.brain-map.org/grid_data/download/68844229?include=energy
Frk	0.973	http://api.brain-map.org/grid_data/download/68443268?include=energy
Pou2af1	0.973	http://api.brain-map.org/grid_data/download/68743739?include=energy
Ccin	0.973	http://api.brain-map.org/grid_data/download/76003320?include=energy
Glrx5	0.973	http://api.brain-map.org/grid_data/download/69262236?include=energy
9630040P08Rik*	0.973	http://api.brain-map.org/grid_data/download/73808480?include=energy
Il17rb	0.973	http://api.brain-map.org/grid_data/download/2317?include=energy
Taf15	0.973	http://api.brain-map.org/grid_data/download/69980154?include=energy
Cacng4	0.973	http://api.brain-map.org/grid_data/download/72119652?include=energy
Slc38a4	0.973	http://api.brain-map.org/grid_data/download/2617?include=energy
Sncb	0.973	http://api.brain-map.org/grid_data/download/100144408?include=energy
AI854703	0.973	http://api.brain-map.org/grid_data/download/69288608?include=energy
LOC434391	0.973	http://api.brain-map.org/grid_data/download/71022268?include=energy
Neb	0.973	http://api.brain-map.org/grid_data/download/70300523?include=energy
Srp19	0.973	http://api.brain-map.org/grid_data/download/68798950?include=energy
Arl10	0.973	http://api.brain-map.org/grid_data/download/74658019?include=energy
Mpdu1	0.973	http://api.brain-map.org/grid_data/download/68637665?include=energy
F630018O14Rik*	0.973	http://api.brain-map.org/grid_data/download/73748854?include=energy

Rpl27	0.973	http://api.brain-map.org/grid_data/download/69261404?include=energy
Olfr1322	0.973	http://api.brain-map.org/grid_data/download/74720711?include=energy
H2afv	0.972	http://api.brain-map.org/grid_data/download/74821957?include=energy
Adarb1	0.972	http://api.brain-map.org/grid_data/download/72109379?include=energy
Slfn5	0.972	http://api.brain-map.org/grid_data/download/70302204?include=energy
Rps16	0.972	http://api.brain-map.org/grid_data/download/69818379?include=energy
2700046G09Rik	0.972	http://api.brain-map.org/grid_data/download/69202980?include=energy
Plscr3	0.972	http://api.brain-map.org/grid_data/download/68341734?include=energy
C130026I21Rik	0.972	http://api.brain-map.org/grid_data/download/74578389?include=energy
A930011O12Rik	0.972	http://api.brain-map.org/grid_data/download/69514434?include=energy
Tmem229b	0.972	http://api.brain-map.org/grid_data/download/72128773?include=energy
Mtmr4	0.972	http://api.brain-map.org/grid_data/download/68862084?include=energy
Josd1	0.972	http://api.brain-map.org/grid_data/download/69079990?include=energy
Disp2	0.972	http://api.brain-map.org/grid_data/download/74511801?include=energy
Anxa7	0.972	http://api.brain-map.org/grid_data/download/74562606?include=energy
Syn1	0.972	http://api.brain-map.org/grid_data/download/227540?include=energy
Foxr2	0.972	http://api.brain-map.org/grid_data/download/71022678?include=energy
Dock11	0.972	http://api.brain-map.org/grid_data/download/74580793?include=energy
Gna13	0.972	http://api.brain-map.org/grid_data/download/69014764?include=energy
C1qtnf5	0.972	http://api.brain-map.org/grid_data/download/293664?include=energy
1700016K19Rik	0.972	http://api.brain-map.org/grid_data/download/69114594?include=energy
Nr2e1	0.972	http://api.brain-map.org/grid_data/download/77464920?include=energy
Rpl5	0.972	http://api.brain-map.org/grid_data/download/68633062?include=energy
Cstf1	0.972	http://api.brain-map.org/grid_data/download/70918820?include=energy
F830001A07Rik	0.972	http://api.brain-map.org/grid_data/download/74431552?include=energy
Rgl2	0.972	http://api.brain-map.org/grid_data/download/69818113?include=energy
Pgs1	0.972	http://api.brain-map.org/grid_data/download/70919465?include=energy
Cnot10	0.972	http://api.brain-map.org/grid_data/download/67767469?include=energy
Slc25a3	0.972	http://api.brain-map.org/grid_data/download/972?include=energy
Nudcd3	0.972	http://api.brain-map.org/grid_data/download/69529005?include=energy
Acsf3	0.972	http://api.brain-map.org/grid_data/download/77800945?include=energy
F830218P16Rik*	0.972	http://api.brain-map.org/grid_data/download/73748743?include=energy
TC1516517	0.972	http://api.brain-map.org/grid_data/download/70813209?include=energy
Psmb7	0.972	http://api.brain-map.org/grid_data/download/69014818?include=energy
LOC433089	0.972	http://api.brain-map.org/grid_data/download/71789951?include=energy
Tuft1	0.972	http://api.brain-map.org/grid_data/download/68446003?include=energy
Shisa2	0.972	http://api.brain-map.org/grid_data/download/75043156?include=energy
Opa1	0.972	http://api.brain-map.org/grid_data/download/77620792?include=energy
Ppp2r5d	0.972	http://api.brain-map.org/grid_data/download/74047908?include=energy
Leprotl1	0.972	http://api.brain-map.org/grid_data/download/2373?include=energy
Cyp46a1	0.972	http://api.brain-map.org/grid_data/download/70613977?include=energy
Krt15	0.972	http://api.brain-map.org/grid_data/download/70524284?include=energy
Ccdc63	0.972	http://api.brain-map.org/grid_data/download/70303875?include=energy
D11Ertd759e	0.972	http://api.brain-map.org/grid_data/download/74881365?include=energy
Pramef12	0.972	http://api.brain-map.org/grid_data/download/71891934?include=energy
Zfyve16	0.972	http://api.brain-map.org/grid_data/download/75831734?include=energy
Tex15	0.972	http://api.brain-map.org/grid_data/download/69980298?include=energy
Il3	0.972	http://api.brain-map.org/grid_data/download/69735395?include=energy
Psmb1	0.972	http://api.brain-map.org/grid_data/download/74059243?include=energy
Lax1	0.972	http://api.brain-map.org/grid_data/download/69262302?include=energy
Hint3	0.972	http://api.brain-map.org/grid_data/download/199218?include=energy
Cdk2	0.972	http://api.brain-map.org/grid_data/download/76081209?include=energy
Slc6a17	0.972	http://api.brain-map.org/grid_data/download/72001997?include=energy
Spns3	0.972	http://api.brain-map.org/grid_data/download/74580924?include=energy
Nfatc1	0.972	http://api.brain-map.org/grid_data/download/68745167?include=energy
Kcnk16	0.972	http://api.brain-map.org/grid_data/download/71393473?include=energy
Dnahc7	0.972	http://api.brain-map.org/grid_data/download/70298561?include=energy
Gde1	0.972	http://api.brain-map.org/grid_data/download/356858?include=energy
Slc16a1	0.972	http://api.brain-map.org/grid_data/download/68203467?include=energy
Fut4-ps1	0.972	http://api.brain-map.org/grid_data/download/74265261?include=energy
Prkcsh	0.972	http://api.brain-map.org/grid_data/download/67870479?include=energy
AA388235	0.971	http://api.brain-map.org/grid_data/download/73635413?include=energy
Myl3	0.971	http://api.brain-map.org/grid_data/download/70543064?include=energy
Prss3	0.971	http://api.brain-map.org/grid_data/download/71210822?include=energy
Sh2b1	0.971	http://api.brain-map.org/grid_data/download/68799022?include=energy
Stk11	0.971	http://api.brain-map.org/grid_data/download/69887349?include=energy
Cops6	0.971	http://api.brain-map.org/grid_data/download/75695664?include=energy
1190002H23Rik	0.971	http://api.brain-map.org/grid_data/download/69114097?include=energy
N4bp2l2	0.971	http://api.brain-map.org/grid_data/download/68637742?include=energy
Wnk1	0.971	http://api.brain-map.org/grid_data/download/70300923?include=energy
5730422E09Rik	0.971	http://api.brain-map.org/grid_data/download/70787191?include=energy
Armc9	0.971	http://api.brain-map.org/grid_data/download/68441586?include=energy
Tmem218	0.971	http://api.brain-map.org/grid_data/download/1960?include=energy
Arf2	0.971	http://api.brain-map.org/grid_data/download/70717993?include=energy
Gpat2	0.971	http://api.brain-map.org/grid_data/download/69448704?include=energy
Mier1	0.971	http://api.brain-map.org/grid_data/download/74580769?include=energy
Ggt7	0.971	http://api.brain-map.org/grid_data/download/75145998?include=energy
Eif3c	0.971	http://api.brain-map.org/grid_data/download/68499549?include=energy
4932407I05	0.971	http://api.brain-map.org/grid_data/download/70305012?include=energy
Brwd3	0.971	http://api.brain-map.org/grid_data/download/70231827?include=energy
Scn11a	0.971	http://api.brain-map.org/grid_data/download/69288291?include=energy
Csad	0.971	http://api.brain-map.org/grid_data/download/71496342?include=energy
Adcy8	0.971	http://api.brain-map.org/grid_data/download/77280540?include=energy
Alkbh6	0.971	http://api.brain-map.org/grid_data/download/77414882?include=energy
Psmb6	0.971	http://api.brain-map.org/grid_data/download/69080020?include=energy
Pwwp2b	0.971	http://api.brain-map.org/grid_data/download/70300965?include=energy
Sfi1	0.971	http://api.brain-map.org/grid_data/download/69012783?include=energy
Tef	0.971	http://api.brain-map.org/grid_data/download/393201?include=energy
Ccl20	0.971	http://api.brain-map.org/grid_data/download/68744654?include=energy
Tubgcp5	0.971	http://api.brain-map.org/grid_data/download/68442865?include=energy
AF119384	0.971	http://api.brain-map.org/grid_data/download/70724547?include=energy
Cep97	0.971	http://api.brain-map.org/grid_data/download/77620445?include=energy
Ubxn10	0.971	http://api.brain-map.org/grid_data/download/77800953?include=energy
Gng12	0.971	http://api.brain-map.org/grid_data/download/73512194?include=energy
Gm799	0.971	http://api.brain-map.org/grid_data/download/73818855?include=energy
Dnajc4	0.971	http://api.brain-map.org/grid_data/download/69262343?include=energy
LOC433906	0.971	http://api.brain-map.org/grid_data/download/70787089?include=energy
Arhgap19	0.971	http://api.brain-map.org/grid_data/download/75988276?include=energy
Ddx50	0.971	http://api.brain-map.org/grid_data/download/81600132?include=energy
Btn1a1	0.971	http://api.brain-map.org/grid_data/download/68076975?include=energy
Unc80	0.971	http://api.brain-map.org/grid_data/download/69530726?include=energy
Mknk1	0.971	http://api.brain-map.org/grid_data/download/71610882?include=energy
Nkx2-9	0.971	http://api.brain-map.org/grid_data/download/75934936?include=energy
Jup	0.971	http://api.brain-map.org/grid_data/download/74425547?include=energy
Calb2	0.971	http://api.brain-map.org/grid_data/download/79556662?include=energy
Tmx4	0.971	http://api.brain-map.org/grid_data/download/74882217?include=energy
Gm979	0.971	http://api.brain-map.org/grid_data/download/70298549?include=energy
Garnl3	0.971	http://api.brain-map.org/grid_data/download/73994668?include=energy
Ndufs6	0.971	http://api.brain-map.org/grid_data/download/75214950?include=energy
Tmem45a	0.971	http://api.brain-map.org/grid_data/download/69133623?include=energy
Sap30l	0.971	http://api.brain-map.org/grid_data/download/73907495?include=energy
I1C0048O07Rik*	0.97	http://api.brain-map.org/grid_data/download/73716728?include=energy
Defb36	0.97	http://api.brain-map.org/grid_data/download/73423687?include=energy
Anp32e	0.97	http://api.brain-map.org/grid_data/download/71610840?include=energy
Tob1	0.97	http://api.brain-map.org/grid_data/download/69817019?include=energy
4832428D23Rik	0.97	http://api.brain-map.org/grid_data/download/70205572?include=energy
Ap3m1	0.97	http://api.brain-map.org/grid_data/download/68498337?include=energy
Fkbp15	0.97	http://api.brain-map.org/grid_data/download/71021021?include=energy
Car3	0.97	http://api.brain-map.org/grid_data/download/75990502?include=energy
Igf1r	0.97	http://api.brain-map.org/grid_data/download/69735263?include=energy
Olfr140	0.97	http://api.brain-map.org/grid_data/download/73653296?include=energy
Aars	0.97	http://api.brain-map.org/grid_data/download/74641317?include=energy
Ogfr	0.97	http://api.brain-map.org/grid_data/download/67809268?include=energy
Smpdl3a	0.97	http://api.brain-map.org/grid_data/download/75198088?include=energy
Zfp180	0.97	http://api.brain-map.org/grid_data/download/70445329?include=energy
Vmn1r45	0.97	http://api.brain-map.org/grid_data/download/69442216?include=energy
9430041J12Rik	0.97	http://api.brain-map.org/grid_data/download/74300669?include=energy
Iqcf3	0.97	http://api.brain-map.org/grid_data/download/72081789?include=energy
Trp53bp1	0.97	http://api.brain-map.org/grid_data/download/71836834?include=energy
Arhgdia	0.97	http://api.brain-map.org/grid_data/download/75774677?include=energy
Gm2716	0.97	http://api.brain-map.org/grid_data/download/72182967?include=energy
BC002163	0.97	http://api.brain-map.org/grid_data/download/68076547?include=energy
Morf4l2	0.97	http://api.brain-map.org/grid_data/download/70946634?include=energy
Rbm12	0.97	http://api.brain-map.org/grid_data/download/71212072?include=energy
Ttc29	0.97	http://api.brain-map.org/grid_data/download/75990628?include=energy
Slc25a27	0.97	http://api.brain-map.org/grid_data/download/74988334?include=energy
Cdc37l1	0.97	http://api.brain-map.org/grid_data/download/69015781?include=energy
Map1lc3b	0.97	http://api.brain-map.org/grid_data/download/69751980?include=energy
Fyb	0.97	http://api.brain-map.org/grid_data/download/74003378?include=energy
Caprin1	0.97	http://api.brain-map.org/grid_data/download/71232750?include=energy
Il28ra	0.97	http://api.brain-map.org/grid_data/download/70946158?include=energy
Sp1	0.97	http://api.brain-map.org/grid_data/download/70593390?include=energy
Cnot2	0.97	http://api.brain-map.org/grid_data/download/71496312?include=energy
Fnbp4	0.97	http://api.brain-map.org/grid_data/download/69672539?include=energy
Actc1	0.97	http://api.brain-map.org/grid_data/download/62?include=energy
Gabpb1	0.97	http://api.brain-map.org/grid_data/download/121098?include=energy
Tmub2	0.97	http://api.brain-map.org/grid_data/download/293027?include=energy
C1galt1c1	0.97	http://api.brain-map.org/grid_data/download/75931466?include=energy
H2-DMb2	0.97	http://api.brain-map.org/grid_data/download/71764691?include=energy
Coasy	0.97	http://api.brain-map.org/grid_data/download/69169487?include=energy
Lgi2	0.97	http://api.brain-map.org/grid_data/download/71670485?include=energy
BC068157	0.97	http://api.brain-map.org/grid_data/download/71022726?include=energy
Hist1h1b	0.97	http://api.brain-map.org/grid_data/download/74272040?include=energy
Malt1	0.97	http://api.brain-map.org/grid_data/download/100144931?include=energy
Tyrobp	0.97	http://api.brain-map.org/grid_data/download/70523695?include=energy
Dcaf13	0.97	http://api.brain-map.org/grid_data/download/69028641?include=energy
Acy3	0.97	http://api.brain-map.org/grid_data/download/71617959?include=energy
Serpina11	0.97	http://api.brain-map.org/grid_data/download/76081910?include=energy
Cdhr2	0.97	http://api.brain-map.org/grid_data/download/69529107?include=energy
B230369O20Rik*	0.97	http://api.brain-map.org/grid_data/download/73906349?include=energy
Slc35b2	0.97	http://api.brain-map.org/grid_data/download/2613?include=energy
Ear6	0.97	http://api.brain-map.org/grid_data/download/70724696?include=energy
Sergef	0.97	http://api.brain-map.org/grid_data/download/71764482?include=energy
Ccpg1	0.97	http://api.brain-map.org/grid_data/download/69059940?include=energy
A330035P11Rik	0.97	http://api.brain-map.org/grid_data/download/69448014?include=energy
Cyb5b	0.969	http://api.brain-map.org/grid_data/download/69102359?include=energy
Kif13b	0.969	http://api.brain-map.org/grid_data/download/72283647?include=energy
Csrp2bp	0.969	http://api.brain-map.org/grid_data/download/276275?include=energy
Olfml2a	0.969	http://api.brain-map.org/grid_data/download/74277664?include=energy
Scg5	0.969	http://api.brain-map.org/grid_data/download/75773511?include=energy
Cldn5	0.969	http://api.brain-map.org/grid_data/download/79490120?include=energy
Xiap	0.969	http://api.brain-map.org/grid_data/download/69529310?include=energy
Crocc	0.969	http://api.brain-map.org/grid_data/download/70546069?include=energy
Ptprb	0.969	http://api.brain-map.org/grid_data/download/71378418?include=energy
LOC215539	0.969	http://api.brain-map.org/grid_data/download/70565957?include=energy
Ubqlnl	0.969	http://api.brain-map.org/grid_data/download/71570754?include=energy
Gm177	0.969	http://api.brain-map.org/grid_data/download/70295954?include=energy
Atxn7	0.969	http://api.brain-map.org/grid_data/download/70301979?include=energy
Ints3	0.969	http://api.brain-map.org/grid_data/download/100144709?include=energy
1110020A21Rik	0.969	http://api.brain-map.org/grid_data/download/71919104?include=energy
Vtn	0.969	http://api.brain-map.org/grid_data/download/67752116?include=energy
Sumf2	0.969	http://api.brain-map.org/grid_data/download/72002107?include=energy
Zcwpw2	0.969	http://api.brain-map.org/grid_data/download/71891671?include=energy
Ecel1	0.969	http://api.brain-map.org/grid_data/download/70231305?include=energy
2500004C02Rik	0.969	http://api.brain-map.org/grid_data/download/71211620?include=energy
Sbsn	0.969	http://api.brain-map.org/grid_data/download/69133755?include=energy
Dpt	0.969	http://api.brain-map.org/grid_data/download/68920884?include=energy
Atic	0.969	http://api.brain-map.org/grid_data/download/72077466?include=energy
Supt5h	0.969	http://api.brain-map.org/grid_data/download/2647?include=energy
Slc10a5	0.969	http://api.brain-map.org/grid_data/download/70296092?include=energy
Fgf23	0.969	http://api.brain-map.org/grid_data/download/81655572?include=energy
Erc2	0.969	http://api.brain-map.org/grid_data/download/68797661?include=energy
Smap2	0.969	http://api.brain-map.org/grid_data/download/69407122?include=energy
Slc16a6	0.969	http://api.brain-map.org/grid_data/download/2581?include=energy
Rbm38	0.969	http://api.brain-map.org/grid_data/download/2535?include=energy
Wdr7	0.969	http://api.brain-map.org/grid_data/download/70303004?include=energy
Grb7	0.969	http://api.brain-map.org/grid_data/download/112204995?include=energy
Mvd	0.969	http://api.brain-map.org/grid_data/download/79536365?include=energy
Ccdc39	0.969	http://api.brain-map.org/grid_data/download/69608941?include=energy
Arid3b	0.969	http://api.brain-map.org/grid_data/download/68797798?include=energy
Alg11	0.969	http://api.brain-map.org/grid_data/download/69262233?include=energy
Smarcb1	0.969	http://api.brain-map.org/grid_data/download/68522368?include=energy
Tpst2	0.969	http://api.brain-map.org/grid_data/download/69080033?include=energy
Heca	0.969	http://api.brain-map.org/grid_data/download/74821831?include=energy
Spink8	0.969	http://api.brain-map.org/grid_data/download/72129253?include=energy
Rusc1	0.969	http://api.brain-map.org/grid_data/download/68844080?include=energy
2700089E24Rik	0.969	http://api.brain-map.org/grid_data/download/71022918?include=energy
Mtnr1b	0.969	http://api.brain-map.org/grid_data/download/69258178?include=energy
Rad23a	0.969	http://api.brain-map.org/grid_data/download/71992007?include=energy
1810031K17Rik	0.969	http://api.brain-map.org/grid_data/download/227165?include=energy
Eci2	0.969	http://api.brain-map.org/grid_data/download/100144173?include=energy
LOC545795	0.969	http://api.brain-map.org/grid_data/download/74427049?include=energy
Usp39	0.969	http://api.brain-map.org/grid_data/download/74357633?include=energy
Btnl1	0.969	http://api.brain-map.org/grid_data/download/69013080?include=energy
Atp5h	0.969	http://api.brain-map.org/grid_data/download/77371785?include=energy
Rragc	0.969	http://api.brain-map.org/grid_data/download/79534965?include=energy
Eif5a	0.969	http://api.brain-map.org/grid_data/download/2183?include=energy
7420419J09Rik*	0.969	http://api.brain-map.org/grid_data/download/73906107?include=energy
Acat1	0.969	http://api.brain-map.org/grid_data/download/71836840?include=energy
Mark3	0.969	http://api.brain-map.org/grid_data/download/67881765?include=energy
4932416K20Rik	0.969	http://api.brain-map.org/grid_data/download/74431309?include=energy
A630085K21	0.969	http://api.brain-map.org/grid_data/download/71735066?include=energy
Nqo2	0.969	http://api.brain-map.org/grid_data/download/74819244?include=energy
Tiam1	0.969	http://api.brain-map.org/grid_data/download/1059?include=energy
Mbd3	0.969	http://api.brain-map.org/grid_data/download/69080515?include=energy
Lrpprc	0.969	http://api.brain-map.org/grid_data/download/70546087?include=energy
A830021M18Rik	0.969	http://api.brain-map.org/grid_data/download/70305347?include=energy
Hsp90ab1	0.969	http://api.brain-map.org/grid_data/download/69735168?include=energy
Smarcc2	0.969	http://api.brain-map.org/grid_data/download/69444723?include=energy
Eif3d	0.968	http://api.brain-map.org/grid_data/download/69671959?include=energy
Usp5	0.968	http://api.brain-map.org/grid_data/download/72004457?include=energy
Fto	0.968	http://api.brain-map.org/grid_data/download/79749595?include=energy
4921525O09Rik	0.968	http://api.brain-map.org/grid_data/download/71493192?include=energy
Nde1	0.968	http://api.brain-map.org/grid_data/download/67779875?include=energy
Wbp1	0.968	http://api.brain-map.org/grid_data/download/69080018?include=energy
9330159M07Rik	0.968	http://api.brain-map.org/grid_data/download/71152282?include=energy
Bid	0.968	http://api.brain-map.org/grid_data/download/71836838?include=energy
Suv39h1	0.968	http://api.brain-map.org/grid_data/download/68157405?include=energy
Spag6	0.968	http://api.brain-map.org/grid_data/download/72002077?include=energy
Rps28	0.968	http://api.brain-map.org/grid_data/download/69112407?include=energy
Hnrnpa1	0.968	http://api.brain-map.org/grid_data/download/71232800?include=energy
Polr1e	0.968	http://api.brain-map.org/grid_data/download/75774108?include=energy
Znrf1	0.968	http://api.brain-map.org/grid_data/download/74581382?include=energy
Haghl	0.968	http://api.brain-map.org/grid_data/download/75259440?include=energy
Denr	0.968	http://api.brain-map.org/grid_data/download/68845705?include=energy
Vmp1	0.968	http://api.brain-map.org/grid_data/download/532588?include=energy
Zfp358	0.968	http://api.brain-map.org/grid_data/download/2743?include=energy
C130073F10Rik	0.968	http://api.brain-map.org/grid_data/download/70231887?include=energy
Epb4.1l4b	0.968	http://api.brain-map.org/grid_data/download/75988402?include=energy
Tmem30c	0.968	http://api.brain-map.org/grid_data/download/72076677?include=energy
Krtap16-10b	0.968	http://api.brain-map.org/grid_data/download/70231110?include=energy
Mob3b	0.968	http://api.brain-map.org/grid_data/download/70814343?include=energy
Zfp772	0.968	http://api.brain-map.org/grid_data/download/79544876?include=energy
4933407L21Rik	0.968	http://api.brain-map.org/grid_data/download/69443142?include=energy
2310044H10Rik	0.968	http://api.brain-map.org/grid_data/download/71486931?include=energy
Dnahc2	0.968	http://api.brain-map.org/grid_data/download/69608917?include=energy
Zfyve20	0.968	http://api.brain-map.org/grid_data/download/81600098?include=energy
Sep15	0.968	http://api.brain-map.org/grid_data/download/671225?include=energy
Zmym3	0.968	http://api.brain-map.org/grid_data/download/70598369?include=energy
Hmgn5	0.968	http://api.brain-map.org/grid_data/download/73423729?include=energy
Tmco3	0.968	http://api.brain-map.org/grid_data/download/71608433?include=energy
Fam89a	0.968	http://api.brain-map.org/grid_data/download/69202418?include=energy
Mtap6	0.968	http://api.brain-map.org/grid_data/download/74580829?include=energy
Glb1l2	0.968	http://api.brain-map.org/grid_data/download/678705?include=energy
Sema4a	0.968	http://api.brain-map.org/grid_data/download/942?include=energy
Fzd7	0.968	http://api.brain-map.org/grid_data/download/77932779?include=energy
Znhit1	0.968	http://api.brain-map.org/grid_data/download/69134256?include=energy

Tmem127	0.968	http://api.brain-map.org/grid_data/download/72081906?include=energy
Tmprss11g	0.968	http://api.brain-map.org/grid_data/download/72471694?include=energy
Eif3l	0.968	http://api.brain-map.org/grid_data/download/69174022?include=energy
Mrap2	0.968	http://api.brain-map.org/grid_data/download/71924480?include=energy
Agap1	0.968	http://api.brain-map.org/grid_data/download/69548840?include=energy
Gpr153	0.968	http://api.brain-map.org/grid_data/download/79360308?include=energy
BC024659	0.968	http://api.brain-map.org/grid_data/download/69529011?include=energy
Rbm18	0.968	http://api.brain-map.org/grid_data/download/356083?include=energy
Bex4	0.968	http://api.brain-map.org/grid_data/download/69201534?include=energy
5330417C22Rik	0.968	http://api.brain-map.org/grid_data/download/69443361?include=energy
Fam55c	0.967	http://api.brain-map.org/grid_data/download/74658081?include=energy
Naa50	0.967	http://api.brain-map.org/grid_data/download/70546105?include=energy
Wnt9b	0.967	http://api.brain-map.org/grid_data/download/1892?include=energy
Ifi35	0.967	http://api.brain-map.org/grid_data/download/69735234?include=energy
Pla2g4c	0.967	http://api.brain-map.org/grid_data/download/79912601?include=energy
1810046J19Rik	0.967	http://api.brain-map.org/grid_data/download/678683?include=energy
Mill2	0.967	http://api.brain-map.org/grid_data/download/74743354?include=energy
Lrrc59	0.967	http://api.brain-map.org/grid_data/download/69079980?include=energy
Foxi3	0.967	http://api.brain-map.org/grid_data/download/74736063?include=energy
Prop1	0.967	http://api.brain-map.org/grid_data/download/71325366?include=energy
4931412M21	0.967	http://api.brain-map.org/grid_data/download/72005998?include=energy
Ric8	0.967	http://api.brain-map.org/grid_data/download/69080052?include=energy
Ercc1	0.967	http://api.brain-map.org/grid_data/download/576431?include=energy
Ppap2a	0.967	http://api.brain-map.org/grid_data/download/830?include=energy
Micu1	0.967	http://api.brain-map.org/grid_data/download/74658238?include=energy
Oas1h	0.967	http://api.brain-map.org/grid_data/download/76081026?include=energy
Dna2	0.967	http://api.brain-map.org/grid_data/download/69626927?include=energy
Gm816	0.967	http://api.brain-map.org/grid_data/download/71832856?include=energy
Smtnl2	0.967	http://api.brain-map.org/grid_data/download/71616125?include=energy
Gm430	0.967	http://api.brain-map.org/grid_data/download/70298142?include=energy
Pnoc	0.967	http://api.brain-map.org/grid_data/download/75038402?include=energy
Colec11	0.967	http://api.brain-map.org/grid_data/download/74581014?include=energy
AU021034	0.967	http://api.brain-map.org/grid_data/download/69527036?include=energy
Slc17a3	0.967	http://api.brain-map.org/grid_data/download/69015715?include=energy
Cdx1	0.967	http://api.brain-map.org/grid_data/download/130534?include=energy
Epdr1	0.967	http://api.brain-map.org/grid_data/download/74881156?include=energy
Arfrp1	0.967	http://api.brain-map.org/grid_data/download/69080006?include=energy
Stbd1	0.967	http://api.brain-map.org/grid_data/download/69608977?include=energy
Apln	0.967	http://api.brain-map.org/grid_data/download/67850953?include=energy
Nr1h2	0.967	http://api.brain-map.org/grid_data/download/72182970?include=energy
Chfr	0.967	http://api.brain-map.org/grid_data/download/68798525?include=energy
Cd247	0.967	http://api.brain-map.org/grid_data/download/68667898?include=energy
Iars	0.967	http://api.brain-map.org/grid_data/download/69735192?include=energy
LOC433841	0.967	http://api.brain-map.org/grid_data/download/70787002?include=energy
A330044P14Rik	0.967	http://api.brain-map.org/grid_data/download/71808448?include=energy
Ets1	0.967	http://api.brain-map.org/grid_data/download/2199?include=energy
Lrrc3	0.967	http://api.brain-map.org/grid_data/download/112649047?include=energy
Sdf4	0.967	http://api.brain-map.org/grid_data/download/69873457?include=energy
Myo1c	0.967	http://api.brain-map.org/grid_data/download/68410446?include=energy
Wdr5	0.967	http://api.brain-map.org/grid_data/download/227159?include=energy
D5Ertd579e	0.967	http://api.brain-map.org/grid_data/download/70429064?include=energy
Zfp296	0.967	http://api.brain-map.org/grid_data/download/71489793?include=energy
Armcx1	0.967	http://api.brain-map.org/grid_data/download/68445131?include=energy
Dnaja2	0.967	http://api.brain-map.org/grid_data/download/71763753?include=energy
Ypel2	0.967	http://api.brain-map.org/grid_data/download/71213322?include=energy
1700025O08Rik	0.967	http://api.brain-map.org/grid_data/download/71919619?include=energy
Tfcp2	0.967	http://api.brain-map.org/grid_data/download/68410494?include=energy
Stxbp3a	0.967	http://api.brain-map.org/grid_data/download/69887421?include=energy
Tmem222	0.967	http://api.brain-map.org/grid_data/download/69059877?include=energy
Hist1h1c	0.967	http://api.brain-map.org/grid_data/download/69735030?include=energy
Traf3ip2	0.967	http://api.brain-map.org/grid_data/download/75931448?include=energy
Rnf25	0.967	http://api.brain-map.org/grid_data/download/70523811?include=energy
Tspan33	0.967	http://api.brain-map.org/grid_data/download/70743905?include=energy
Plac9	0.967	http://api.brain-map.org/grid_data/download/68545009?include=energy
Ndufa12	0.967	http://api.brain-map.org/grid_data/download/70526031?include=energy
Sppl3	0.967	http://api.brain-map.org/grid_data/download/68443453?include=energy
Zmym5	0.967	http://api.brain-map.org/grid_data/download/75774661?include=energy
Morn2	0.967	http://api.brain-map.org/grid_data/download/71210514?include=energy
Ogfod2	0.966	http://api.brain-map.org/grid_data/download/69169797?include=energy
Magohb	0.966	http://api.brain-map.org/grid_data/download/77464996?include=energy
Rhbdl1	0.966	http://api.brain-map.org/grid_data/download/583574?include=energy
1700017N19Rik	0.966	http://api.brain-map.org/grid_data/download/71919493?include=energy
Ggnbp2	0.966	http://api.brain-map.org/grid_data/download/70303498?include=energy
Adam5	0.966	http://api.brain-map.org/grid_data/download/75988238?include=energy
Fbxo44	0.966	http://api.brain-map.org/grid_data/download/77340498?include=energy
Tmc1	0.966	http://api.brain-map.org/grid_data/download/75041514?include=energy
I0C0044D17Rik	0.966	http://api.brain-map.org/grid_data/download/75043659?include=energy
Rxrg	0.966	http://api.brain-map.org/grid_data/download/917?include=energy
Zbtb7a	0.966	http://api.brain-map.org/grid_data/download/71161477?include=energy
Arhgap44	0.966	http://api.brain-map.org/grid_data/download/70231074?include=energy
Slc35b4	0.966	http://api.brain-map.org/grid_data/download/75774107?include=energy
Cldn18	0.966	http://api.brain-map.org/grid_data/download/70237944?include=energy
2610028E06Rik	0.966	http://api.brain-map.org/grid_data/download/71020430?include=energy
Slc35f3	0.966	http://api.brain-map.org/grid_data/download/75084156?include=energy
1700037H04Rik	0.966	http://api.brain-map.org/grid_data/download/324211?include=energy
Izumo4	0.966	http://api.brain-map.org/grid_data/download/69028611?include=energy
Zfp101	0.966	http://api.brain-map.org/grid_data/download/70919436?include=energy
Znrf4	0.966	http://api.brain-map.org/grid_data/download/72004494?include=energy
Trim34a	0.966	http://api.brain-map.org/grid_data/download/70302824?include=energy
Usmg5	0.966	http://api.brain-map.org/grid_data/download/67862431?include=energy
Rundc1	0.966	http://api.brain-map.org/grid_data/download/69873349?include=energy
Samd3	0.966	http://api.brain-map.org/grid_data/download/1375?include=energy
Nsun7	0.966	http://api.brain-map.org/grid_data/download/77332090?include=energy
Snapc4	0.966	http://api.brain-map.org/grid_data/download/68797822?include=energy
Scnm1	0.966	http://api.brain-map.org/grid_data/download/68666595?include=energy
Tmem186	0.966	http://api.brain-map.org/grid_data/download/67767462?include=energy
Mprip	0.966	http://api.brain-map.org/grid_data/download/68844235?include=energy
Rpe65	0.966	http://api.brain-map.org/grid_data/download/70301073?include=energy
Aox3	0.966	http://api.brain-map.org/grid_data/download/69526689?include=energy
Prss42	0.966	http://api.brain-map.org/grid_data/download/69174445?include=energy
Gm156	0.966	http://api.brain-map.org/grid_data/download/72471826?include=energy
Neto2	0.966	http://api.brain-map.org/grid_data/download/75084453?include=energy
Khdrbs1	0.966	http://api.brain-map.org/grid_data/download/532636?include=energy
Ahi1	0.966	http://api.brain-map.org/grid_data/download/69549642?include=energy
Plin4	0.966	http://api.brain-map.org/grid_data/download/71280778?include=energy
Limd2	0.966	http://api.brain-map.org/grid_data/download/69352750?include=energy
Tbc1d9b	0.966	http://api.brain-map.org/grid_data/download/70542909?include=energy
Fig4	0.966	http://api.brain-map.org/grid_data/download/68341525?include=energy
Rela	0.966	http://api.brain-map.org/grid_data/download/2523?include=energy
Dyx1c1	0.966	http://api.brain-map.org/grid_data/download/68498729?include=energy
9330109E03Rik	0.966	http://api.brain-map.org/grid_data/download/71493861?include=energy
Dnajc9	0.966	http://api.brain-map.org/grid_data/download/69095973?include=energy
LOC433402	0.966	http://api.brain-map.org/grid_data/download/73931425?include=energy
1110032A04Rik	0.966	http://api.brain-map.org/grid_data/download/68498540?include=energy
Hyal1	0.966	http://api.brain-map.org/grid_data/download/68301132?include=energy
Col4a5	0.966	http://api.brain-map.org/grid_data/download/73992932?include=energy
Pdlim4	0.966	http://api.brain-map.org/grid_data/download/68545735?include=energy
Rlim	0.966	http://api.brain-map.org/grid_data/download/71763577?include=energy
Adk	0.966	http://api.brain-map.org/grid_data/download/70928196?include=energy
Notch3	0.966	http://api.brain-map.org/grid_data/download/79908817?include=energy
1700040L02Rik	0.966	http://api.brain-map.org/grid_data/download/69353693?include=energy
Sec11c	0.966	http://api.brain-map.org/grid_data/download/385777?include=energy
Arg2	0.966	http://api.brain-map.org/grid_data/download/70928338?include=energy
Srsf3	0.965	http://api.brain-map.org/grid_data/download/69873648?include=energy
F730314M05Rik*	0.965	http://api.brain-map.org/grid_data/download/73767205?include=energy
Psmd14	0.965	http://api.brain-map.org/grid_data/download/69080012?include=energy
Gcdh	0.965	http://api.brain-map.org/grid_data/download/69289039?include=energy
Ear5	0.965	http://api.brain-map.org/grid_data/download/70724690?include=energy
Lctl	0.965	http://api.brain-map.org/grid_data/download/68523312?include=energy
Gcap14	0.965	http://api.brain-map.org/grid_data/download/71493123?include=energy
Baat1	0.965	http://api.brain-map.org/grid_data/download/69514512?include=energy
Tppp	0.965	http://api.brain-map.org/grid_data/download/74724775?include=energy
Tns3	0.965	http://api.brain-map.org/grid_data/download/75080694?include=energy
Nol8	0.965	http://api.brain-map.org/grid_data/download/69134322?include=energy
Apoa1bp	0.965	http://api.brain-map.org/grid_data/download/68862156?include=energy
Ccdc102a	0.965	http://api.brain-map.org/grid_data/download/69529029?include=energy
1700012P22Rik	0.965	http://api.brain-map.org/grid_data/download/71919403?include=energy
Igf2bp2	0.965	http://api.brain-map.org/grid_data/download/68843751?include=energy
Gm1441	0.965	http://api.brain-map.org/grid_data/download/72109262?include=energy
Pkhd1l1	0.965	http://api.brain-map.org/grid_data/download/71325336?include=energy
Ppm1g	0.965	http://api.brain-map.org/grid_data/download/2499?include=energy
Tmem26	0.965	http://api.brain-map.org/grid_data/download/69981091?include=energy
Pnp	0.965	http://api.brain-map.org/grid_data/download/68191558?include=energy
Col6a1	0.965	http://api.brain-map.org/grid_data/download/290?include=energy
Hist1h1a	0.965	http://api.brain-map.org/grid_data/download/74988764?include=energy
Kpna2	0.965	http://api.brain-map.org/grid_data/download/73584741?include=energy
Zfyve28	0.965	http://api.brain-map.org/grid_data/download/75934919?include=energy
Syce2	0.965	http://api.brain-map.org/grid_data/download/70609150?include=energy
Setd1b	0.965	http://api.brain-map.org/grid_data/download/74000642?include=energy
Gm4861	0.965	http://api.brain-map.org/grid_data/download/74510290?include=energy
Mrpl21	0.965	http://api.brain-map.org/grid_data/download/68523129?include=energy
Hapln2	0.965	http://api.brain-map.org/grid_data/download/71529905?include=energy
9230110C19Rik	0.965	http://api.brain-map.org/grid_data/download/71493849?include=energy
Col8a2	0.965	http://api.brain-map.org/grid_data/download/70720251?include=energy
Cela2a	0.965	http://api.brain-map.org/grid_data/download/68499326?include=energy
Fam46d	0.965	http://api.brain-map.org/grid_data/download/70295667?include=energy
Car9	0.965	http://api.brain-map.org/grid_data/download/74988271?include=energy
Rpl35a	0.965	http://api.brain-map.org/grid_data/download/68637784?include=energy
Camk4	0.965	http://api.brain-map.org/grid_data/download/75038464?include=energy
Colec10	0.965	http://api.brain-map.org/grid_data/download/100147510?include=energy
Exosc7	0.965	http://api.brain-map.org/grid_data/download/68667539?include=energy
Mkl2	0.965	http://api.brain-map.org/grid_data/download/69289363?include=energy
Ctnnbl1	0.965	http://api.brain-map.org/grid_data/download/71724858?include=energy
Hr	0.965	http://api.brain-map.org/grid_data/download/68924509?include=energy
Pex13	0.965	http://api.brain-map.org/grid_data/download/74658240?include=energy
Hbq1a	0.965	http://api.brain-map.org/grid_data/download/69734935?include=energy
Cbfb	0.965	http://api.brain-map.org/grid_data/download/1168?include=energy
D030034H08	0.965	http://api.brain-map.org/grid_data/download/71213794?include=energy
Rgs4	0.965	http://api.brain-map.org/grid_data/download/74511884?include=energy
Mrps18b	0.965	http://api.brain-map.org/grid_data/download/68410428?include=energy
Arid1a	0.965	http://api.brain-map.org/grid_data/download/69134208?include=energy
Syt2	0.965	http://api.brain-map.org/grid_data/download/1023?include=energy
Icmt	0.965	http://api.brain-map.org/grid_data/download/585?include=energy
Bpifb3	0.965	http://api.brain-map.org/grid_data/download/75831709?include=energy
Zfp106	0.965	http://api.brain-map.org/grid_data/download/70195102?include=energy
Pard6a	0.965	http://api.brain-map.org/grid_data/download/69116578?include=energy
Abcc5	0.964	http://api.brain-map.org/grid_data/download/69236628?include=energy
1700029F12Rik	0.964	http://api.brain-map.org/grid_data/download/69114486?include=energy
Eea1	0.964	http://api.brain-map.org/grid_data/download/70239081?include=energy
Gpr68	0.964	http://api.brain-map.org/grid_data/download/69257624?include=energy
Slitrk3	0.964	http://api.brain-map.org/grid_data/download/69874150?include=energy
Speer5-ps1	0.964	http://api.brain-map.org/grid_data/download/75931623?include=energy
1200014J11Rik	0.964	http://api.brain-map.org/grid_data/download/69353176?include=energy
Prickle2	0.964	http://api.brain-map.org/grid_data/download/70300917?include=energy
Npy6r	0.964	http://api.brain-map.org/grid_data/download/69258208?include=energy
Flrt1	0.964	http://api.brain-map.org/grid_data/download/71587667?include=energy
Gtpbp1	0.964	http://api.brain-map.org/grid_data/download/70919221?include=energy
Mtap7d1	0.964	http://api.brain-map.org/grid_data/download/70429166?include=energy
Aldh5a1	0.964	http://api.brain-map.org/grid_data/download/77925007?include=energy
Aspdh	0.964	http://api.brain-map.org/grid_data/download/68341991?include=energy
Rasgrp1	0.964	http://api.brain-map.org/grid_data/download/76115742?include=energy
Mmp10	0.964	http://api.brain-map.org/grid_data/download/70946570?include=energy
Eaf2	0.964	http://api.brain-map.org/grid_data/download/633673?include=energy
Rbm27	0.964	http://api.brain-map.org/grid_data/download/68797518?include=energy
Thrap3	0.964	http://api.brain-map.org/grid_data/download/71210166?include=energy
Atp5s	0.964	http://api.brain-map.org/grid_data/download/323589?include=energy
LOC545135	0.964	http://api.brain-map.org/grid_data/download/77621745?include=energy
Tnnt3	0.964	http://api.brain-map.org/grid_data/download/61411?include=energy
Nucb2	0.964	http://api.brain-map.org/grid_data/download/75774683?include=energy
Gsta4	0.964	http://api.brain-map.org/grid_data/download/77278951?include=energy
Wnt6	0.964	http://api.brain-map.org/grid_data/download/2735?include=energy
Hmox2	0.964	http://api.brain-map.org/grid_data/download/75774681?include=energy
Gjb3	0.964	http://api.brain-map.org/grid_data/download/75861847?include=energy
Mapt	0.964	http://api.brain-map.org/grid_data/download/79556696?include=energy
Wwp2	0.964	http://api.brain-map.org/grid_data/download/386002?include=energy
Tmem184b	0.964	http://api.brain-map.org/grid_data/download/68745534?include=energy
Map3k4	0.964	http://api.brain-map.org/grid_data/download/71724870?include=energy
Gnl2	0.964	http://api.brain-map.org/grid_data/download/513480?include=energy
Luc7l2	0.964	http://api.brain-map.org/grid_data/download/74578167?include=energy
Myo1b	0.964	http://api.brain-map.org/grid_data/download/72080133?include=energy
Il10rb	0.964	http://api.brain-map.org/grid_data/download/77464934?include=energy
March6	0.964	http://api.brain-map.org/grid_data/download/69838566?include=energy
Slc39a11	0.964	http://api.brain-map.org/grid_data/download/78633993?include=energy
4930448N21Rik	0.964	http://api.brain-map.org/grid_data/download/73818831?include=energy
Tsg101	0.964	http://api.brain-map.org/grid_data/download/74047878?include=energy
Il12b	0.964	http://api.brain-map.org/grid_data/download/69735347?include=energy
Ube2v1	0.964	http://api.brain-map.org/grid_data/download/67979036?include=energy
Rint1	0.964	http://api.brain-map.org/grid_data/download/69353266?include=energy
Zranb1	0.964	http://api.brain-map.org/grid_data/download/70303516?include=energy
Gli2	0.964	http://api.brain-map.org/grid_data/download/69289063?include=energy
Dnhd1	0.964	http://api.brain-map.org/grid_data/download/71493837?include=energy
Mical3	0.964	http://api.brain-map.org/grid_data/download/71281123?include=energy
Bend3	0.964	http://api.brain-map.org/grid_data/download/71495729?include=energy
Glp1r	0.964	http://api.brain-map.org/grid_data/download/74511737?include=energy
Lsm10	0.964	http://api.brain-map.org/grid_data/download/71735827?include=energy
Hdgfrp2	0.964	http://api.brain-map.org/grid_data/download/513472?include=energy
Pcnxl3	0.964	http://api.brain-map.org/grid_data/download/71920491?include=energy
Mapk6	0.964	http://api.brain-map.org/grid_data/download/68745546?include=energy
Mrpl41	0.964	http://api.brain-map.org/grid_data/download/74300849?include=energy
Mau2	0.964	http://api.brain-map.org/grid_data/download/71158472?include=energy
Cntfr	0.964	http://api.brain-map.org/grid_data/download/75651138?include=energy
Hbb-bh1	0.964	http://api.brain-map.org/grid_data/download/68798891?include=energy
Pcsk2	0.964	http://api.brain-map.org/grid_data/download/72080025?include=energy
2210404J11Rik	0.964	http://api.brain-map.org/grid_data/download/71808496?include=energy
Angptl3	0.964	http://api.brain-map.org/grid_data/download/75827099?include=energy
LOC547273	0.964	http://api.brain-map.org/grid_data/download/100144395?include=energy
2310003L22Rik	0.964	http://api.brain-map.org/grid_data/download/72283749?include=energy
Tyrp1	0.964	http://api.brain-map.org/grid_data/download/100144880?include=energy
Uqcrfs1	0.963	http://api.brain-map.org/grid_data/download/71924399?include=energy
Olfml2b	0.963	http://api.brain-map.org/grid_data/download/1980?include=energy
Tdrd9	0.963	http://api.brain-map.org/grid_data/download/70302449?include=energy
Olfr976	0.963	http://api.brain-map.org/grid_data/download/74735009?include=energy
Rad21	0.963	http://api.brain-map.org/grid_data/download/69817898?include=energy
Mm.351348	0.963	http://api.brain-map.org/grid_data/download/71161129?include=energy
Tpt1	0.963	http://api.brain-map.org/grid_data/download/71378554?include=energy
Pfdn5	0.963	http://api.brain-map.org/grid_data/download/68498271?include=energy
Vps52	0.963	http://api.brain-map.org/grid_data/download/69174469?include=energy
LOC545748	0.963	http://api.brain-map.org/grid_data/download/75043665?include=energy
Birc6	0.963	http://api.brain-map.org/grid_data/download/69529316?include=energy
Eml4	0.963	http://api.brain-map.org/grid_data/download/70239105?include=energy
H2-Bl	0.963	http://api.brain-map.org/grid_data/download/69015410?include=energy
Expi	0.963	http://api.brain-map.org/grid_data/download/68301222?include=energy
Cyp1a2	0.963	http://api.brain-map.org/grid_data/download/68302293?include=energy
Tbce	0.963	http://api.brain-map.org/grid_data/download/68667198?include=energy
Pdia6	0.963	http://api.brain-map.org/grid_data/download/69079989?include=energy
Tfeb	0.963	http://api.brain-map.org/grid_data/download/69290650?include=energy
Arhgap27	0.963	http://api.brain-map.org/grid_data/download/75774664?include=energy
Zfp30	0.963	http://api.brain-map.org/grid_data/download/68911021?include=energy
Glipr1l2	0.963	http://api.brain-map.org/grid_data/download/71891581?include=energy
Atp7b	0.963	http://api.brain-map.org/grid_data/download/69526988?include=energy
2310006M14Rik	0.963	http://api.brain-map.org/grid_data/download/74635400?include=energy
Sox9	0.963	http://api.brain-map.org/grid_data/download/1422?include=energy
Smpdl3b	0.963	http://api.brain-map.org/grid_data/download/68269298?include=energy
P2ry13	0.963	http://api.brain-map.org/grid_data/download/69258280?include=energy
LOC433126	0.963	http://api.brain-map.org/grid_data/download/75693109?include=energy
Atp5l	0.963	http://api.brain-map.org/grid_data/download/71724816?include=energy
Cog1	0.963	http://api.brain-map.org/grid_data/download/68844092?include=energy

9230019H11Rik	0.963	http://api.brain-map.org/grid_data/download/74514198?include=energy
Rpl26	0.963	http://api.brain-map.org/grid_data/download/69816792?include=energy
B020004J07Rik	0.963	http://api.brain-map.org/grid_data/download/70786731?include=energy
Spon2	0.963	http://api.brain-map.org/grid_data/download/68410225?include=energy
Ctnna3	0.963	http://api.brain-map.org/grid_data/download/75988355?include=energy
Gal3st1	0.963	http://api.brain-map.org/grid_data/download/68193027?include=energy
Psg16	0.963	http://api.brain-map.org/grid_data/download/655307?include=energy
Slk	0.963	http://api.brain-map.org/grid_data/download/69874168?include=energy
Srpk1	0.963	http://api.brain-map.org/grid_data/download/513000?include=energy
Fam33a	0.963	http://api.brain-map.org/grid_data/download/69352840?include=energy
Gm4934	0.963	http://api.brain-map.org/grid_data/download/70566217?include=energy
Anxa4	0.963	http://api.brain-map.org/grid_data/download/70436730?include=energy
Gm5463	0.963	http://api.brain-map.org/grid_data/download/70611347?include=energy
Atp1a3	0.963	http://api.brain-map.org/grid_data/download/74881263?include=energy
Lime1	0.963	http://api.brain-map.org/grid_data/download/68148758?include=energy
Rpl27a	0.963	http://api.brain-map.org/grid_data/download/69262228?include=energy
Ppfibp1	0.963	http://api.brain-map.org/grid_data/download/74272480?include=energy
Lypd2	0.963	http://api.brain-map.org/grid_data/download/76081044?include=energy
Fam19a3	0.963	http://api.brain-map.org/grid_data/download/71281800?include=energy
Ppp6r1	0.963	http://api.brain-map.org/grid_data/download/68911023?include=energy
Prss56	0.963	http://api.brain-map.org/grid_data/download/70201379?include=energy
Adamts16	0.963	http://api.brain-map.org/grid_data/download/276267?include=energy
Cdadc1	0.963	http://api.brain-map.org/grid_data/download/70525807?include=energy
Pmpcb	0.963	http://api.brain-map.org/grid_data/download/69816338?include=energy
Gm1661	0.963	http://api.brain-map.org/grid_data/download/70299905?include=energy
Casc1	0.963	http://api.brain-map.org/grid_data/download/75038484?include=energy
Ldhc	0.963	http://api.brain-map.org/grid_data/download/69116522?include=energy
Ddx42	0.963	http://api.brain-map.org/grid_data/download/81600130?include=energy
Fgf1	0.963	http://api.brain-map.org/grid_data/download/79556599?include=energy
Cd160	0.963	http://api.brain-map.org/grid_data/download/68410482?include=energy
Arhgef18	0.962	http://api.brain-map.org/grid_data/download/69080007?include=energy
Gm12	0.962	http://api.brain-map.org/grid_data/download/70295524?include=energy
Rad1	0.962	http://api.brain-map.org/grid_data/download/68798621?include=energy
LOC545465	0.962	http://api.brain-map.org/grid_data/download/70919418?include=energy
4933434E20Rik	0.962	http://api.brain-map.org/grid_data/download/69443250?include=energy
Gm4969	0.962	http://api.brain-map.org/grid_data/download/70305168?include=energy
Chrac1	0.962	http://api.brain-map.org/grid_data/download/69174274?include=energy
Ttbk2	0.962	http://api.brain-map.org/grid_data/download/75147759?include=energy
Pkp2	0.962	http://api.brain-map.org/grid_data/download/73997149?include=energy
Stk3	0.962	http://api.brain-map.org/grid_data/download/70918940?include=energy
Tmem52	0.962	http://api.brain-map.org/grid_data/download/71991798?include=energy
Epb4.1l1	0.962	http://api.brain-map.org/grid_data/download/74750065?include=energy
2700097O09Rik	0.962	http://api.brain-map.org/grid_data/download/68844020?include=energy
Slc16a4	0.962	http://api.brain-map.org/grid_data/download/2579?include=energy
Spata4	0.962	http://api.brain-map.org/grid_data/download/69114587?include=energy
Akap10	0.962	http://api.brain-map.org/grid_data/download/68667383?include=energy
1700010I14Rik	0.962	http://api.brain-map.org/grid_data/download/69116763?include=energy
Glud1	0.962	http://api.brain-map.org/grid_data/download/503?include=energy
Tmem30a	0.962	http://api.brain-map.org/grid_data/download/81600550?include=energy
Nudt3	0.962	http://api.brain-map.org/grid_data/download/393247?include=energy
Tmem72	0.962	http://api.brain-map.org/grid_data/download/73992925?include=energy
Vdac3	0.962	http://api.brain-map.org/grid_data/download/1549?include=energy
Lin37	0.962	http://api.brain-map.org/grid_data/download/393212?include=energy
Acad9	0.962	http://api.brain-map.org/grid_data/download/76098388?include=energy
Cdh19	0.962	http://api.brain-map.org/grid_data/download/74359646?include=energy
Msn	0.962	http://api.brain-map.org/grid_data/download/74658085?include=energy
Mrps9	0.962	http://api.brain-map.org/grid_data/download/69169745?include=energy
Zfyve9	0.962	http://api.brain-map.org/grid_data/download/73521827?include=energy
Ttc4	0.962	http://api.brain-map.org/grid_data/download/545098?include=energy
Cdc25c	0.962	http://api.brain-map.org/grid_data/download/77793151?include=energy
LOC434070	0.962	http://api.brain-map.org/grid_data/download/70787390?include=energy
Six2	0.962	http://api.brain-map.org/grid_data/download/71325468?include=energy
Lrp11	0.962	http://api.brain-map.org/grid_data/download/69837962?include=energy
Tmem192	0.962	http://api.brain-map.org/grid_data/download/71610898?include=energy
LOC433473	0.962	http://api.brain-map.org/grid_data/download/70723277?include=energy
Cttnbp2	0.962	http://api.brain-map.org/grid_data/download/70305634?include=energy
Pes1	0.962	http://api.brain-map.org/grid_data/download/77910701?include=energy
Fgfbp1	0.962	http://api.brain-map.org/grid_data/download/77455051?include=energy
Leng8	0.962	http://api.brain-map.org/grid_data/download/77620457?include=energy
Ltv1	0.962	http://api.brain-map.org/grid_data/download/69202830?include=energy
Senp2	0.962	http://api.brain-map.org/grid_data/download/276274?include=energy
Pdia3	0.962	http://api.brain-map.org/grid_data/download/71015817?include=energy
G530019E01Rik*	0.962	http://api.brain-map.org/grid_data/download/73808159?include=energy
E130311K13Rik	0.962	http://api.brain-map.org/grid_data/download/70565518?include=energy
Ubn2	0.962	http://api.brain-map.org/grid_data/download/69013104?include=energy
Psmb9	0.962	http://api.brain-map.org/grid_data/download/71377822?include=energy
Gpr19	0.962	http://api.brain-map.org/grid_data/download/100144124?include=energy
Rad54l2	0.962	http://api.brain-map.org/grid_data/download/71280925?include=energy
St6gal2	0.962	http://api.brain-map.org/grid_data/download/69290537?include=energy
A930005L06Rik*	0.962	http://api.brain-map.org/grid_data/download/73808312?include=energy
Krtcap2	0.962	http://api.brain-map.org/grid_data/download/67767459?include=energy
Sdhb	0.962	http://api.brain-map.org/grid_data/download/74658140?include=energy
Ctss	0.962	http://api.brain-map.org/grid_data/download/77371797?include=energy
Il12a	0.962	http://api.brain-map.org/grid_data/download/69735341?include=energy
Itpk1	0.962	http://api.brain-map.org/grid_data/download/71923998?include=energy
AI593442	0.962	http://api.brain-map.org/grid_data/download/71250312?include=energy
Pcdhga2	0.962	http://api.brain-map.org/grid_data/download/74277412?include=energy
Wasf3	0.962	http://api.brain-map.org/grid_data/download/74058647?include=energy
Hdlbp	0.962	http://api.brain-map.org/grid_data/download/74819232?include=energy
F830225E14Rik*	0.961	http://api.brain-map.org/grid_data/download/73748776?include=energy
Ganc	0.961	http://api.brain-map.org/grid_data/download/69444080?include=energy
2610524H06Rik	0.961	http://api.brain-map.org/grid_data/download/69202950?include=energy
Zkscan16	0.961	http://api.brain-map.org/grid_data/download/74750043?include=energy
Rec8	0.961	http://api.brain-map.org/grid_data/download/77278975?include=energy
Plekhm2	0.961	http://api.brain-map.org/grid_data/download/70300869?include=energy
4931431F19Rik	0.961	http://api.brain-map.org/grid_data/download/70218498?include=energy
Gfod2	0.961	http://api.brain-map.org/grid_data/download/77454589?include=energy
Fam64a	0.961	http://api.brain-map.org/grid_data/download/68444964?include=energy
Gm9903	0.961	http://api.brain-map.org/grid_data/download/71213855?include=energy
BC034090	0.961	http://api.brain-map.org/grid_data/download/69529065?include=energy
Fndc4	0.961	http://api.brain-map.org/grid_data/download/293665?include=energy
Mllt11	0.961	http://api.brain-map.org/grid_data/download/89?include=energy
Nrg2	0.961	http://api.brain-map.org/grid_data/download/81654141?include=energy
Nr1h3	0.961	http://api.brain-map.org/grid_data/download/112205214?include=energy
Mirg	0.961	http://api.brain-map.org/grid_data/download/70946557?include=energy
Anapc7	0.961	http://api.brain-map.org/grid_data/download/69526623?include=energy
Pkd2l1	0.961	http://api.brain-map.org/grid_data/download/74881878?include=energy
Pdia4	0.961	http://api.brain-map.org/grid_data/download/69540272?include=energy
Wfdc8	0.961	http://api.brain-map.org/grid_data/download/74578272?include=energy
Gm10010	0.961	http://api.brain-map.org/grid_data/download/70787402?include=energy
Dynll1	0.961	http://api.brain-map.org/grid_data/download/81600140?include=energy
Megf8	0.961	http://api.brain-map.org/grid_data/download/71496546?include=energy
Cyp2c70	0.961	http://api.brain-map.org/grid_data/download/68499069?include=energy
Eif2c2	0.961	http://api.brain-map.org/grid_data/download/69169941?include=energy
Spink2	0.961	http://api.brain-map.org/grid_data/download/70302252?include=energy
Peg10	0.961	http://api.brain-map.org/grid_data/download/74357549?include=energy
H2-D1	0.961	http://api.brain-map.org/grid_data/download/77620277?include=energy
Olfr137	0.961	http://api.brain-map.org/grid_data/download/74720793?include=energy
Mycs	0.961	http://api.brain-map.org/grid_data/download/75214945?include=energy
1110006O24Rik	0.961	http://api.brain-map.org/grid_data/download/69116948?include=energy
Emd	0.961	http://api.brain-map.org/grid_data/download/69672001?include=energy
E030030I06Rik	0.961	http://api.brain-map.org/grid_data/download/70238973?include=energy
Fgf13	0.961	http://api.brain-map.org/grid_data/download/427?include=energy
Nog	0.961	http://api.brain-map.org/grid_data/download/75084476?include=energy
Nipal4	0.961	http://api.brain-map.org/grid_data/download/70717377?include=energy
Ypel5	0.961	http://api.brain-map.org/grid_data/download/70195084?include=energy
Kcnk7	0.961	http://api.brain-map.org/grid_data/download/73496923?include=energy
Slc9a3r2	0.961	http://api.brain-map.org/grid_data/download/74657980?include=energy
Phyh	0.961	http://api.brain-map.org/grid_data/download/74800914?include=energy
Vdac2	0.961	http://api.brain-map.org/grid_data/download/1888?include=energy
F8	0.961	http://api.brain-map.org/grid_data/download/75988413?include=energy
Olfr315	0.961	http://api.brain-map.org/grid_data/download/73653386?include=energy
Skiv2l2	0.961	http://api.brain-map.org/grid_data/download/322964?include=energy
Txndc16	0.961	http://api.brain-map.org/grid_data/download/69013149?include=energy
Gm5820	0.961	http://api.brain-map.org/grid_data/download/75043629?include=energy
Gsdmd	0.961	http://api.brain-map.org/grid_data/download/71724472?include=energy
Dguok	0.961	http://api.brain-map.org/grid_data/download/69626861?include=energy
Cyp39a1	0.961	http://api.brain-map.org/grid_data/download/71836847?include=energy
9330169B04Rik	0.961	http://api.brain-map.org/grid_data/download/71147936?include=energy
Tsfm	0.961	http://api.brain-map.org/grid_data/download/72129246?include=energy
LOC382174	0.961	http://api.brain-map.org/grid_data/download/71764344?include=energy
Dopey2	0.961	http://api.brain-map.org/grid_data/download/31?include=energy
Acad12	0.961	http://api.brain-map.org/grid_data/download/69446631?include=energy
Gas8	0.961	http://api.brain-map.org/grid_data/download/74990538?include=energy
Ctr9	0.961	http://api.brain-map.org/grid_data/download/71668371?include=energy
Zdhhc15	0.961	http://api.brain-map.org/grid_data/download/70598365?include=energy
Prpf38b	0.961	http://api.brain-map.org/grid_data/download/73907503?include=energy
Wnt2	0.961	http://api.brain-map.org/grid_data/download/81654143?include=energy
Drg1	0.961	http://api.brain-map.org/grid_data/download/68861649?include=energy
Def8	0.961	http://api.brain-map.org/grid_data/download/69095968?include=energy
Elovl5	0.961	http://api.brain-map.org/grid_data/download/74273118?include=energy
Camk2d	0.961	http://api.brain-map.org/grid_data/download/71016676?include=energy
Kif16b	0.961	http://api.brain-map.org/grid_data/download/70723289?include=energy
Tph1	0.961	http://api.brain-map.org/grid_data/download/72004442?include=energy
F3	0.96	http://api.brain-map.org/grid_data/download/77280341?include=energy
Hexdc	0.96	http://api.brain-map.org/grid_data/download/73423787?include=energy
Timm22	0.96	http://api.brain-map.org/grid_data/download/68844026?include=energy
A530013C23Rik	0.96	http://api.brain-map.org/grid_data/download/71494690?include=energy
Gm4755	0.96	http://api.brain-map.org/grid_data/download/70565551?include=energy
Cyb5r4	0.96	http://api.brain-map.org/grid_data/download/532762?include=energy
Rab28	0.96	http://api.brain-map.org/grid_data/download/81600102?include=energy
Sc4mol	0.96	http://api.brain-map.org/grid_data/download/74750020?include=energy
Fbf1	0.96	http://api.brain-map.org/grid_data/download/71497000?include=energy
Gna14	0.96	http://api.brain-map.org/grid_data/download/71723949?include=energy
Tmco5b	0.96	http://api.brain-map.org/grid_data/download/69114346?include=energy
Rab9b	0.96	http://api.brain-map.org/grid_data/download/69817892?include=energy
2610305D13Rik	0.96	http://api.brain-map.org/grid_data/download/71764011?include=energy
Klhl10	0.96	http://api.brain-map.org/grid_data/download/75828200?include=energy
Trit1	0.96	http://api.brain-map.org/grid_data/download/671233?include=energy
Gm333	0.96	http://api.brain-map.org/grid_data/download/80514151?include=energy
Slc11a1	0.96	http://api.brain-map.org/grid_data/download/69816870?include=energy
Emilin1	0.96	http://api.brain-map.org/grid_data/download/68442925?include=energy
Rbm20	0.96	http://api.brain-map.org/grid_data/download/69352926?include=energy
Nudt7	0.96	http://api.brain-map.org/grid_data/download/74427085?include=energy
Pttg1ip	0.96	http://api.brain-map.org/grid_data/download/1956?include=energy
Snph	0.96	http://api.brain-map.org/grid_data/download/69885582?include=energy
Ppp4r1	0.96	http://api.brain-map.org/grid_data/download/67787349?include=energy
Hps3	0.96	http://api.brain-map.org/grid_data/download/68798073?include=energy
Zbtb7b	0.96	http://api.brain-map.org/grid_data/download/68920815?include=energy
Galc	0.96	http://api.brain-map.org/grid_data/download/71280505?include=energy
Atp6v0a2	0.96	http://api.brain-map.org/grid_data/download/357094?include=energy
Cyp2d10	0.96	http://api.brain-map.org/grid_data/download/77465044?include=energy
Atl2	0.96	http://api.brain-map.org/grid_data/download/513010?include=energy
1110018J18Rik	0.96	http://api.brain-map.org/grid_data/download/71764541?include=energy
C030014I23Rik	0.96	http://api.brain-map.org/grid_data/download/71022876?include=energy
Itlnb	0.96	http://api.brain-map.org/grid_data/download/81655574?include=energy
Apip	0.96	http://api.brain-map.org/grid_data/download/68522510?include=energy
Kcnj8	0.96	http://api.brain-map.org/grid_data/download/2343?include=energy
Pcgf2	0.96	http://api.brain-map.org/grid_data/download/69015488?include=energy
Naaa	0.96	http://api.brain-map.org/grid_data/download/70445598?include=energy
Paqr7	0.96	http://api.brain-map.org/grid_data/download/69015601?include=energy
Tceal6	0.96	http://api.brain-map.org/grid_data/download/71724762?include=energy
1700109H08Rik	0.96	http://api.brain-map.org/grid_data/download/72283707?include=energy
Daf2	0.96	http://api.brain-map.org/grid_data/download/72472783?include=energy
0610010K14Rik	0.96	http://api.brain-map.org/grid_data/download/69134379?include=energy
Atrn	0.96	http://api.brain-map.org/grid_data/download/69527012?include=energy
9130409J20Rik	0.96	http://api.brain-map.org/grid_data/download/71892358?include=energy
LOC432980	0.96	http://api.brain-map.org/grid_data/download/71282556?include=energy
Ash1l	0.96	http://api.brain-map.org/grid_data/download/69526916?include=energy
Men1	0.96	http://api.brain-map.org/grid_data/download/71489796?include=energy
Taar3	0.96	http://api.brain-map.org/grid_data/download/73573235?include=energy
A730023A08Rik*	0.96	http://api.brain-map.org/grid_data/download/73811525?include=energy
Oaf	0.96	http://api.brain-map.org/grid_data/download/68499081?include=energy
F830212F20Rik*	0.96	http://api.brain-map.org/grid_data/download/73748716?include=energy
Mbd5	0.96	http://api.brain-map.org/grid_data/download/71789801?include=energy
Ccndbp1	0.96	http://api.brain-map.org/grid_data/download/532590?include=energy
Oscp1	0.96	http://api.brain-map.org/grid_data/download/68745341?include=energy
Foxh1	0.96	http://api.brain-map.org/grid_data/download/71307322?include=energy
Akap12	0.96	http://api.brain-map.org/grid_data/download/72128778?include=energy
Msr1	0.959	http://api.brain-map.org/grid_data/download/79360306?include=energy
1700016C15Rik	0.959	http://api.brain-map.org/grid_data/download/70201277?include=energy
Fam101b	0.959	http://api.brain-map.org/grid_data/download/71492814?include=energy
Tmem236	0.959	http://api.brain-map.org/grid_data/download/73731325?include=energy
Ptrh1	0.959	http://api.brain-map.org/grid_data/download/69202250?include=energy
Ccdc97	0.959	http://api.brain-map.org/grid_data/download/69609067?include=energy
Trnt1	0.959	http://api.brain-map.org/grid_data/download/67765722?include=energy
Vps37b	0.959	http://api.brain-map.org/grid_data/download/68637682?include=energy
4933408J17Rik	0.959	http://api.brain-map.org/grid_data/download/71892131?include=energy
Arhgef9	0.959	http://api.brain-map.org/grid_data/download/70231011?include=energy
Lrrc48	0.959	http://api.brain-map.org/grid_data/download/68633267?include=energy
Mlana	0.959	http://api.brain-map.org/grid_data/download/70300348?include=energy
Slc17a9	0.959	http://api.brain-map.org/grid_data/download/81790702?include=energy
Ces5a	0.959	http://api.brain-map.org/grid_data/download/70276927?include=energy
Cmpk1	0.959	http://api.brain-map.org/grid_data/download/67765697?include=energy
Slco4c1	0.959	http://api.brain-map.org/grid_data/download/69874102?include=energy
Camta1	0.959	http://api.brain-map.org/grid_data/download/69540333?include=energy
Pnn	0.959	http://api.brain-map.org/grid_data/download/70919603?include=energy
A330070K13Rik	0.959	http://api.brain-map.org/grid_data/download/69448215?include=energy
Clip3	0.959	http://api.brain-map.org/grid_data/download/71380483?include=energy
Ddx5	0.959	http://api.brain-map.org/grid_data/download/340?include=energy
Igfbp7	0.959	http://api.brain-map.org/grid_data/download/81654139?include=energy
Sprr3	0.959	http://api.brain-map.org/grid_data/download/69885714?include=energy
Cbr1	0.959	http://api.brain-map.org/grid_data/download/68301431?include=energy
Upk1a	0.959	http://api.brain-map.org/grid_data/download/70302914?include=energy
Cul1	0.959	http://api.brain-map.org/grid_data/download/68545132?include=energy
Gm933	0.959	http://api.brain-map.org/grid_data/download/71570762?include=energy
Dync1li2	0.959	http://api.brain-map.org/grid_data/download/69627027?include=energy
8430419L09Rik	0.959	http://api.brain-map.org/grid_data/download/69446286?include=energy
Hmgcs2	0.959	http://api.brain-map.org/grid_data/download/68666714?include=energy
Sstr3	0.959	http://api.brain-map.org/grid_data/download/69288339?include=energy
Ccdc85b	0.959	http://api.brain-map.org/grid_data/download/71574362?include=energy
Fchsd2	0.959	http://api.brain-map.org/grid_data/download/70276819?include=energy
Rhoc	0.959	http://api.brain-map.org/grid_data/download/68191354?include=energy
A930012M17	0.959	http://api.brain-map.org/grid_data/download/70305371?include=energy
4732414G09Rik	0.959	http://api.brain-map.org/grid_data/download/70300168?include=energy
Ndufs7	0.959	http://api.brain-map.org/grid_data/download/70526029?include=energy
Cyp2c55	0.959	http://api.brain-map.org/grid_data/download/71627811?include=energy
Hexb	0.959	http://api.brain-map.org/grid_data/download/77340466?include=energy
Hes3	0.959	http://api.brain-map.org/grid_data/download/2287?include=energy
Otof	0.959	http://api.brain-map.org/grid_data/download/73788043?include=energy
Lamb3	0.959	http://api.brain-map.org/grid_data/download/76081212?include=energy
Ptger3	0.959	http://api.brain-map.org/grid_data/download/71325384?include=energy
Fads2	0.959	http://api.brain-map.org/grid_data/download/69015290?include=energy
H3f3a	0.959	http://api.brain-map.org/grid_data/download/632507?include=energy
Slc16a5	0.959	http://api.brain-map.org/grid_data/download/73424220?include=energy
Cd164l2	0.959	http://api.brain-map.org/grid_data/download/71991828?include=energy
2310044G17Rik	0.959	http://api.brain-map.org/grid_data/download/70919782?include=energy
Ulk4	0.959	http://api.brain-map.org/grid_data/download/69443070?include=energy
Mcph1	0.959	http://api.brain-map.org/grid_data/download/68843907?include=energy
Ehd4	0.959	http://api.brain-map.org/grid_data/download/71724556?include=energy
Slc14a1	0.959	http://api.brain-map.org/grid_data/download/76003324?include=energy
Zfp706	0.959	http://api.brain-map.org/grid_data/download/69838238?include=energy
Ecsit	0.959	http://api.brain-map.org/grid_data/download/67767444?include=energy
Ebi3	0.959	http://api.brain-map.org/grid_data/download/104608?include=energy
Asxl2	0.959	http://api.brain-map.org/grid_data/download/74580691?include=energy
Ddx6	0.959	http://api.brain-map.org/grid_data/download/70445688?include=energy
Tbc1d10b	0.959	http://api.brain-map.org/grid_data/download/68151349?include=energy

Thoc3	0.959	http://api.brain-map.org/grid_data/download/68410231?include=energy
LOC545143	0.959	http://api.brain-map.org/grid_data/download/70812705?include=energy
Trpm4	0.959	http://api.brain-map.org/grid_data/download/69863273?include=energy
Matr3	0.959	http://api.brain-map.org/grid_data/download/67752309?include=energy
1810024B03Rik	0.959	http://api.brain-map.org/grid_data/download/70609194?include=energy
Igfbp2	0.959	http://api.brain-map.org/grid_data/download/70431385?include=energy
Rab33a	0.959	http://api.brain-map.org/grid_data/download/70785000?include=energy
Slc7a12	0.959	http://api.brain-map.org/grid_data/download/73818809?include=energy
Hrh3	0.959	http://api.brain-map.org/grid_data/download/73636034?include=energy
Pacsin1	0.959	http://api.brain-map.org/grid_data/download/74047926?include=energy
Tbcd	0.959	http://api.brain-map.org/grid_data/download/69980178?include=energy
Lrpap1	0.958	http://api.brain-map.org/grid_data/download/69736262?include=energy
Oas1g	0.958	http://api.brain-map.org/grid_data/download/68341615?include=energy
Sec61g	0.958	http://api.brain-map.org/grid_data/download/76098393?include=energy
Tmc8	0.958	http://api.brain-map.org/grid_data/download/71587631?include=energy
Nub1	0.958	http://api.brain-map.org/grid_data/download/69549670?include=energy
Rasl10b	0.958	http://api.brain-map.org/grid_data/download/74509536?include=energy
Zfp71-rs1	0.958	http://api.brain-map.org/grid_data/download/634461?include=energy
Spsb4	0.958	http://api.brain-map.org/grid_data/download/75773701?include=energy
Atp6v0c	0.958	http://api.brain-map.org/grid_data/download/74881559?include=energy
Chmp2a	0.958	http://api.brain-map.org/grid_data/download/76097722?include=energy
Cdc42bpa	0.958	http://api.brain-map.org/grid_data/download/73513621?include=energy
Gm973	0.958	http://api.brain-map.org/grid_data/download/74988753?include=energy
Neo1	0.958	http://api.brain-map.org/grid_data/download/68843817?include=energy
Ccdc87	0.958	http://api.brain-map.org/grid_data/download/69442440?include=energy
Coro1c	0.958	http://api.brain-map.org/grid_data/download/75651191?include=energy
mCG1029207	0.958	http://api.brain-map.org/grid_data/download/74579485?include=energy
Mat2a	0.958	http://api.brain-map.org/grid_data/download/67978473?include=energy
C030003K17Rik*	0.958	http://api.brain-map.org/grid_data/download/73808373?include=energy
Plag1	0.958	http://api.brain-map.org/grid_data/download/71212524?include=energy
Lrrc10	0.958	http://api.brain-map.org/grid_data/download/76081022?include=energy
Matk	0.958	http://api.brain-map.org/grid_data/download/76135823?include=energy
Arf5	0.958	http://api.brain-map.org/grid_data/download/69526791?include=energy
Fmo9	0.958	http://api.brain-map.org/grid_data/download/70303863?include=energy
Itgb2	0.958	http://api.brain-map.org/grid_data/download/77464984?include=energy
Trank1	0.958	http://api.brain-map.org/grid_data/download/70295984?include=energy
Vldlr	0.958	http://api.brain-map.org/grid_data/download/77413688?include=energy
Ifrd2	0.958	http://api.brain-map.org/grid_data/download/678702?include=energy
Mthfd1	0.958	http://api.brain-map.org/grid_data/download/71763804?include=energy
Mrto4	0.958	http://api.brain-map.org/grid_data/download/512988?include=energy
Gpr98	0.958	http://api.brain-map.org/grid_data/download/72081561?include=energy
Rbm4	0.958	http://api.brain-map.org/grid_data/download/888?include=energy
Taar9	0.958	http://api.brain-map.org/grid_data/download/77620287?include=energy
Mcm4	0.958	http://api.brain-map.org/grid_data/download/68076912?include=energy
LOC433390	0.958	http://api.brain-map.org/grid_data/download/70723205?include=energy
Sf3b2	0.958	http://api.brain-map.org/grid_data/download/74988735?include=energy
Vars	0.958	http://api.brain-map.org/grid_data/download/68668018?include=energy
Cybrd1	0.958	http://api.brain-map.org/grid_data/download/68523342?include=energy
Ptn	0.958	http://api.brain-map.org/grid_data/download/2507?include=energy
Vwc2l	0.958	http://api.brain-map.org/grid_data/download/69449014?include=energy
Adprhl1	0.958	http://api.brain-map.org/grid_data/download/69837099?include=energy
Hnrnpa2b1	0.958	http://api.brain-map.org/grid_data/download/71232819?include=energy
March2	0.958	http://api.brain-map.org/grid_data/download/68076533?include=energy
Cd200	0.958	http://api.brain-map.org/grid_data/download/71358579?include=energy
Ccdc22	0.958	http://api.brain-map.org/grid_data/download/68301951?include=energy
Zp1	0.958	http://api.brain-map.org/grid_data/download/100144098?include=energy
Mybph	0.958	http://api.brain-map.org/grid_data/download/71280694?include=energy
Rtn1	0.958	http://api.brain-map.org/grid_data/download/70928293?include=energy
Dhrs7	0.958	http://api.brain-map.org/grid_data/download/75081208?include=energy
Pcyox1	0.958	http://api.brain-map.org/grid_data/download/67767456?include=energy
Cltb	0.958	http://api.brain-map.org/grid_data/download/71496300?include=energy
Ndufs8	0.958	http://api.brain-map.org/grid_data/download/74819243?include=energy
Zfp790	0.958	http://api.brain-map.org/grid_data/download/356855?include=energy
Slc24a3	0.958	http://api.brain-map.org/grid_data/download/70927810?include=energy
3110057O12Rik	0.958	http://api.brain-map.org/grid_data/download/69407493?include=energy
Nans	0.958	http://api.brain-map.org/grid_data/download/67979040?include=energy
Dhrs3	0.958	http://api.brain-map.org/grid_data/download/112650553?include=energy
Pja1	0.958	http://api.brain-map.org/grid_data/download/74581229?include=energy
Gpr137	0.958	http://api.brain-map.org/grid_data/download/75651149?include=energy
Trim30d	0.958	http://api.brain-map.org/grid_data/download/71764138?include=energy
Lad1	0.958	http://api.brain-map.org/grid_data/download/77910759?include=energy
Gsk3b	0.958	http://api.brain-map.org/grid_data/download/68269508?include=energy
Zfand6	0.958	http://api.brain-map.org/grid_data/download/68269634?include=energy
Prodh2	0.958	http://api.brain-map.org/grid_data/download/69549266?include=energy
Bbx	0.958	http://api.brain-map.org/grid_data/download/77620241?include=energy
Pigr	0.958	http://api.brain-map.org/grid_data/download/104607?include=energy
Rnf34	0.958	http://api.brain-map.org/grid_data/download/71608202?include=energy
LOC432424	0.958	http://api.brain-map.org/grid_data/download/70608484?include=energy
Phc3	0.957	http://api.brain-map.org/grid_data/download/70300780?include=energy
Gm4793	0.957	http://api.brain-map.org/grid_data/download/71765022?include=energy
Gm10103	0.957	http://api.brain-map.org/grid_data/download/70596233?include=energy
Tmc5	0.957	http://api.brain-map.org/grid_data/download/69981067?include=energy
E130308A19Rik	0.957	http://api.brain-map.org/grid_data/download/71789765?include=energy
Nf2	0.957	http://api.brain-map.org/grid_data/download/68442973?include=energy
Tnfrsf11a	0.957	http://api.brain-map.org/grid_data/download/199393?include=energy
Gm1724	0.957	http://api.brain-map.org/grid_data/download/70300049?include=energy
Slc25a25	0.957	http://api.brain-map.org/grid_data/download/544516?include=energy
Dnmbp	0.957	http://api.brain-map.org/grid_data/download/71587571?include=energy
Sparcl1	0.957	http://api.brain-map.org/grid_data/download/74750016?include=energy
Scamp3	0.957	http://api.brain-map.org/grid_data/download/75695653?include=energy
Ppat	0.957	http://api.brain-map.org/grid_data/download/71211272?include=energy
Med4	0.957	http://api.brain-map.org/grid_data/download/71612184?include=energy
Ank2	0.957	http://api.brain-map.org/grid_data/download/71924087?include=energy
Acrbp	0.957	http://api.brain-map.org/grid_data/download/77910651?include=energy
B4galt5	0.957	http://api.brain-map.org/grid_data/download/73635976?include=energy
Nfs1	0.957	http://api.brain-map.org/grid_data/download/67870467?include=energy
C230004F18Rik	0.957	http://api.brain-map.org/grid_data/download/71496048?include=energy
Tdp2	0.957	http://api.brain-map.org/grid_data/download/69817049?include=energy
Mmrn1	0.957	http://api.brain-map.org/grid_data/download/70946603?include=energy
Tas2r118	0.957	http://api.brain-map.org/grid_data/download/70302348?include=energy
Ube2d1	0.957	http://api.brain-map.org/grid_data/download/75991144?include=energy
Ccdc116	0.957	http://api.brain-map.org/grid_data/download/68919000?include=energy
Mybl1	0.957	http://api.brain-map.org/grid_data/download/100146091?include=energy
Vdac1	0.957	http://api.brain-map.org/grid_data/download/74957912?include=energy
Gm5887	0.957	http://api.brain-map.org/grid_data/download/73949632?include=energy
Wtap	0.957	http://api.brain-map.org/grid_data/download/70565431?include=energy
3300002I08Rik	0.957	http://api.brain-map.org/grid_data/download/75934523?include=energy
Dnajc25	0.957	http://api.brain-map.org/grid_data/download/69202238?include=energy
Cpa3	0.957	http://api.brain-map.org/grid_data/download/69568992?include=energy
Fxr1	0.957	http://api.brain-map.org/grid_data/download/69838756?include=energy
Elf1	0.957	http://api.brain-map.org/grid_data/download/77456056?include=energy
2810002N01Rik	0.957	http://api.brain-map.org/grid_data/download/69236538?include=energy
Fam183b	0.957	http://api.brain-map.org/grid_data/download/69407415?include=energy
Gpcpd1	0.957	http://api.brain-map.org/grid_data/download/71489800?include=energy
Ankrd10	0.957	http://api.brain-map.org/grid_data/download/2027?include=energy
Gnai2	0.957	http://api.brain-map.org/grid_data/download/2263?include=energy
Dhx36	0.957	http://api.brain-map.org/grid_data/download/69626867?include=energy
Drd3	0.957	http://api.brain-map.org/grid_data/download/75038431?include=energy
Kcnt1	0.957	http://api.brain-map.org/grid_data/download/71211407?include=energy
Ankmy2	0.957	http://api.brain-map.org/grid_data/download/68301790?include=energy
Mppe1	0.957	http://api.brain-map.org/grid_data/download/68745414?include=energy
Atmin	0.957	http://api.brain-map.org/grid_data/download/69529185?include=energy
Sod1	0.957	http://api.brain-map.org/grid_data/download/994?include=energy
Morn1	0.957	http://api.brain-map.org/grid_data/download/71764338?include=energy
Pdap1	0.957	http://api.brain-map.org/grid_data/download/69782891?include=energy
Papola	0.957	http://api.brain-map.org/grid_data/download/1251?include=energy
LOC433295	0.957	http://api.brain-map.org/grid_data/download/70723074?include=energy
Alg13	0.957	http://api.brain-map.org/grid_data/download/73617636?include=energy
Pfkfb3	0.957	http://api.brain-map.org/grid_data/download/74657950?include=energy
1110049F12Rik	0.957	http://api.brain-map.org/grid_data/download/71232582?include=energy
Grina	0.957	http://api.brain-map.org/grid_data/download/1948?include=energy
Zfp934	0.957	http://api.brain-map.org/grid_data/download/69134328?include=energy
Irs3	0.957	http://api.brain-map.org/grid_data/download/69735538?include=energy
Hdhd2	0.957	http://api.brain-map.org/grid_data/download/69028557?include=energy
Sf3a1	0.957	http://api.brain-map.org/grid_data/download/68546398?include=energy
Ppic	0.957	http://api.brain-map.org/grid_data/download/70546378?include=energy
Cacng7	0.957	http://api.brain-map.org/grid_data/download/74750054?include=energy
Ncoa1	0.957	http://api.brain-map.org/grid_data/download/69289471?include=energy
Dus3l	0.957	http://api.brain-map.org/grid_data/download/69013155?include=energy
Ms4a6b	0.957	http://api.brain-map.org/grid_data/download/293474?include=energy
Capn1	0.957	http://api.brain-map.org/grid_data/download/70528444?include=energy
Tpm4	0.957	http://api.brain-map.org/grid_data/download/69817037?include=energy
Olfr1339	0.957	http://api.brain-map.org/grid_data/download/73635592?include=energy
Vmo1	0.956	http://api.brain-map.org/grid_data/download/70298280?include=energy
Usp8	0.956	http://api.brain-map.org/grid_data/download/70194672?include=energy
Wdr63	0.956	http://api.brain-map.org/grid_data/download/76080942?include=energy
LOC432588	0.956	http://api.brain-map.org/grid_data/download/70611066?include=energy
Mapre2	0.956	http://api.brain-map.org/grid_data/download/659?include=energy
Opn4	0.956	http://api.brain-map.org/grid_data/download/100145386?include=energy
Usp27x	0.956	http://api.brain-map.org/grid_data/download/71393292?include=energy
Ethe1	0.956	http://api.brain-map.org/grid_data/download/69672162?include=energy
Sod2	0.956	http://api.brain-map.org/grid_data/download/634450?include=energy
Ushbp1	0.956	http://api.brain-map.org/grid_data/download/76135471?include=energy
Lpar2	0.956	http://api.brain-map.org/grid_data/download/69237209?include=energy
Ube1y1	0.956	http://api.brain-map.org/grid_data/download/74821753?include=energy
Hipk1	0.956	http://api.brain-map.org/grid_data/download/69735024?include=energy
Tmem30b	0.956	http://api.brain-map.org/grid_data/download/69981097?include=energy
Zfp423	0.956	http://api.brain-map.org/grid_data/download/1607?include=energy
Fxr2	0.956	http://api.brain-map.org/grid_data/download/68862108?include=energy
Nlrc4	0.956	http://api.brain-map.org/grid_data/download/75988354?include=energy
Ccdc25	0.956	http://api.brain-map.org/grid_data/download/75990518?include=energy
4930504O13Rik	0.956	http://api.brain-map.org/grid_data/download/69113907?include=energy
Aldoc	0.956	http://api.brain-map.org/grid_data/download/73512352?include=energy
Pard6g	0.956	http://api.brain-map.org/grid_data/download/678712?include=energy
Cd1d1	0.956	http://api.brain-map.org/grid_data/download/219?include=energy
Pnpla5	0.956	http://api.brain-map.org/grid_data/download/71891563?include=energy
LOC435957	0.956	http://api.brain-map.org/grid_data/download/70787495?include=energy
Ica1l	0.956	http://api.brain-map.org/grid_data/download/75934928?include=energy
Drap1	0.956	http://api.brain-map.org/grid_data/download/70431295?include=energy
Trpm1	0.956	http://api.brain-map.org/grid_data/download/75081002?include=energy
Akap8l	0.956	http://api.brain-map.org/grid_data/download/68924596?include=energy
Vgf	0.956	http://api.brain-map.org/grid_data/download/71924165?include=energy
LOC240906	0.956	http://api.brain-map.org/grid_data/download/74514327?include=energy
Stk32c	0.956	http://api.brain-map.org/grid_data/download/70744516?include=energy
1700106N22Rik	0.956	http://api.brain-map.org/grid_data/download/71764631?include=energy
Zfp641	0.956	http://api.brain-map.org/grid_data/download/71380363?include=energy
Ly6g6c	0.956	http://api.brain-map.org/grid_data/download/74427074?include=energy
Nudt11	0.956	http://api.brain-map.org/grid_data/download/71656792?include=energy
Gm824	0.956	http://api.brain-map.org/grid_data/download/74514345?include=energy
Dsg2	0.956	http://api.brain-map.org/grid_data/download/76135549?include=energy
4932412H11Rik	0.956	http://api.brain-map.org/grid_data/download/70218564?include=energy
Gng2	0.956	http://api.brain-map.org/grid_data/download/67936006?include=energy
Gm12830	0.956	http://api.brain-map.org/grid_data/download/70786743?include=energy
Itpripl1	0.956	http://api.brain-map.org/grid_data/download/71492868?include=energy
1110002N22Rik	0.956	http://api.brain-map.org/grid_data/download/69352846?include=energy
Mtap7d2	0.956	http://api.brain-map.org/grid_data/download/74272052?include=energy
Mlph	0.956	http://api.brain-map.org/grid_data/download/73423832?include=energy
Mob2	0.956	http://api.brain-map.org/grid_data/download/70445461?include=energy
Slc24a2	0.956	http://api.brain-map.org/grid_data/download/71924238?include=energy
Trim47	0.956	http://api.brain-map.org/grid_data/download/72004447?include=energy
Pdgfra	0.956	http://api.brain-map.org/grid_data/download/77280309?include=energy
Atcay	0.956	http://api.brain-map.org/grid_data/download/69012837?include=energy
Pfkm	0.956	http://api.brain-map.org/grid_data/download/67870466?include=energy
Grlf1	0.955	http://api.brain-map.org/grid_data/download/71281194?include=energy
Psenen	0.955	http://api.brain-map.org/grid_data/download/69173331?include=energy
Ceacam1	0.955	http://api.brain-map.org/grid_data/download/79544802?include=energy
Clcn3	0.955	http://api.brain-map.org/grid_data/download/70305610?include=energy
Hes6	0.955	http://api.brain-map.org/grid_data/download/1504?include=energy
Tmem242	0.955	http://api.brain-map.org/grid_data/download/68410345?include=energy
Bax	0.955	http://api.brain-map.org/grid_data/download/71616139?include=energy
Fam161a	0.955	http://api.brain-map.org/grid_data/download/100145370?include=energy
Olfr641	0.955	http://api.brain-map.org/grid_data/download/74734776?include=energy
Pkd1	0.955	http://api.brain-map.org/grid_data/download/71325330?include=energy
Npsr1	0.955	http://api.brain-map.org/grid_data/download/70560288?include=energy
Rfx1	0.955	http://api.brain-map.org/grid_data/download/895?include=energy
Cyp4f39	0.955	http://api.brain-map.org/grid_data/download/75931562?include=energy
Ccdc127	0.955	http://api.brain-map.org/grid_data/download/67978709?include=energy
Pank3	0.955	http://api.brain-map.org/grid_data/download/68161467?include=energy
Luc7l	0.955	http://api.brain-map.org/grid_data/download/68080678?include=energy
Lpcat1	0.955	http://api.brain-map.org/grid_data/download/70539129?include=energy
Spata18	0.955	http://api.brain-map.org/grid_data/download/69353753?include=energy
Ndufv2	0.955	http://api.brain-map.org/grid_data/download/77371763?include=energy
2410017P09Rik	0.955	http://api.brain-map.org/grid_data/download/71022972?include=energy
Faim2	0.955	http://api.brain-map.org/grid_data/download/385775?include=energy
Fgf10	0.955	http://api.brain-map.org/grid_data/download/74513971?include=energy
Coq5	0.955	http://api.brain-map.org/grid_data/download/69104585?include=energy
Fam116a	0.955	http://api.brain-map.org/grid_data/download/71212162?include=energy
Ivd	0.955	http://api.brain-map.org/grid_data/download/71610855?include=energy
2310061I04Rik	0.955	http://api.brain-map.org/grid_data/download/69202555?include=energy
Nup160	0.955	http://api.brain-map.org/grid_data/download/77465020?include=energy
Prnp	0.955	http://api.brain-map.org/grid_data/download/1405?include=energy
I0C0017H17Rik*	0.955	http://api.brain-map.org/grid_data/download/73716716?include=energy
Optc	0.955	http://api.brain-map.org/grid_data/download/73423881?include=energy
Tlr11	0.955	http://api.brain-map.org/grid_data/download/70302515?include=energy
Trappc6b	0.955	http://api.brain-map.org/grid_data/download/68637862?include=energy
Mthfr	0.955	http://api.brain-map.org/grid_data/download/68745438?include=energy
5730585A16Rik	0.955	http://api.brain-map.org/grid_data/download/74265327?include=energy
Vti1b	0.955	http://api.brain-map.org/grid_data/download/67978762?include=energy
Prodh	0.955	http://api.brain-map.org/grid_data/download/75694299?include=energy
Slc6a20b	0.955	http://api.brain-map.org/grid_data/download/77371783?include=energy
Ctnnbip1	0.955	http://api.brain-map.org/grid_data/download/70805935?include=energy
Gm12580	0.955	http://api.brain-map.org/grid_data/download/70786725?include=energy
BC023829	0.955	http://api.brain-map.org/grid_data/download/69528993?include=energy
3110009E18Rik	0.955	http://api.brain-map.org/grid_data/download/68523264?include=energy
Mid1	0.955	http://api.brain-map.org/grid_data/download/73927724?include=energy
Atxn1	0.955	http://api.brain-map.org/grid_data/download/932?include=energy
9430091E24Rik	0.955	http://api.brain-map.org/grid_data/download/70920117?include=energy
AY074887	0.955	http://api.brain-map.org/grid_data/download/73423759?include=energy
Prom2	0.955	http://api.brain-map.org/grid_data/download/68498519?include=energy
Ddx59	0.955	http://api.brain-map.org/grid_data/download/68795405?include=energy
Cuta	0.955	http://api.brain-map.org/grid_data/download/71610894?include=energy
Gm6658	0.955	http://api.brain-map.org/grid_data/download/74822588?include=energy
Adad1	0.955	http://api.brain-map.org/grid_data/download/69980250?include=energy
Eif3e	0.955	http://api.brain-map.org/grid_data/download/68545201?include=energy
Thap3	0.955	http://api.brain-map.org/grid_data/download/69853996?include=energy
5830415F09Rik	0.955	http://api.brain-map.org/grid_data/download/69444074?include=energy
Car11	0.955	http://api.brain-map.org/grid_data/download/68797501?include=energy
Ap3b2	0.955	http://api.brain-map.org/grid_data/download/74514117?include=energy
Il17re	0.955	http://api.brain-map.org/grid_data/download/69169720?include=energy
Micall2	0.955	http://api.brain-map.org/grid_data/download/68861772?include=energy
Metap1d	0.955	http://api.brain-map.org/grid_data/download/68744062?include=energy
Vangl2	0.955	http://api.brain-map.org/grid_data/download/68666834?include=energy
Pdpr	0.955	http://api.brain-map.org/grid_data/download/69407899?include=energy
Csgalnact2	0.955	http://api.brain-map.org/grid_data/download/69838748?include=energy
Lhx5	0.955	http://api.brain-map.org/grid_data/download/1369?include=energy
Dlgap3	0.955	http://api.brain-map.org/grid_data/download/70528605?include=energy
Uqcr11	0.955	http://api.brain-map.org/grid_data/download/67870133?include=energy
Kynu	0.955	http://api.brain-map.org/grid_data/download/71833014?include=energy
Mtmr6	0.955	http://api.brain-map.org/grid_data/download/74658087?include=energy
1810049H13Rik	0.955	http://api.brain-map.org/grid_data/download/70723650?include=energy
Prkar1b	0.955	http://api.brain-map.org/grid_data/download/70616006?include=energy
Uhmk1	0.955	http://api.brain-map.org/grid_data/download/69735831?include=energy
Ccdc18	0.955	http://api.brain-map.org/grid_data/download/69442461?include=energy
Plxnb2	0.955	http://api.brain-map.org/grid_data/download/1263?include=energy
Cyb5r2	0.955	http://api.brain-map.org/grid_data/download/71809418?include=energy
Sfrs18	0.955	http://api.brain-map.org/grid_data/download/70218944?include=energy
Zfp788	0.955	http://api.brain-map.org/grid_data/download/1466?include=energy

Zfp354b	0.955	http://api.brain-map.org/grid_data/download/73606576?include=energy
BC057022	0.955	http://api.brain-map.org/grid_data/download/72077480?include=energy
Pdia2	0.954	http://api.brain-map.org/grid_data/download/71920279?include=energy
Agl	0.954	http://api.brain-map.org/grid_data/download/76003446?include=energy
Dexi	0.954	http://api.brain-map.org/grid_data/download/73930841?include=energy
LOC381358	0.954	http://api.brain-map.org/grid_data/download/70596059?include=energy
Hdac8	0.954	http://api.brain-map.org/grid_data/download/69174397?include=energy
Capn10	0.954	http://api.brain-map.org/grid_data/download/77792631?include=energy
Tnfsf18	0.954	http://api.brain-map.org/grid_data/download/71489790?include=energy
Psmd2	0.954	http://api.brain-map.org/grid_data/download/68157415?include=energy
Blmh	0.954	http://api.brain-map.org/grid_data/download/2064?include=energy
Rhoa	0.954	http://api.brain-map.org/grid_data/download/73606534?include=energy
Gm442	0.954	http://api.brain-map.org/grid_data/download/70298148?include=energy
Cd164	0.954	http://api.brain-map.org/grid_data/download/75749771?include=energy
4632415L05Rik	0.954	http://api.brain-map.org/grid_data/download/75774660?include=energy
Rnmt	0.954	http://api.brain-map.org/grid_data/download/69080481?include=energy
Oma1	0.954	http://api.brain-map.org/grid_data/download/68342330?include=energy
Atp6ap1	0.954	http://api.brain-map.org/grid_data/download/69549631?include=energy
Cwc22	0.954	http://api.brain-map.org/grid_data/download/69080497?include=energy
Exoc5	0.954	http://api.brain-map.org/grid_data/download/71152306?include=energy
LOC432596	0.954	http://api.brain-map.org/grid_data/download/70611090?include=energy
Txlna	0.954	http://api.brain-map.org/grid_data/download/73573283?include=energy
Trim44	0.954	http://api.brain-map.org/grid_data/download/71763607?include=energy
Tial1	0.954	http://api.brain-map.org/grid_data/download/634440?include=energy
Mrgprb3	0.954	http://api.brain-map.org/grid_data/download/73635477?include=energy
Dbn1	0.954	http://api.brain-map.org/grid_data/download/70928198?include=energy
Zfp398	0.954	http://api.brain-map.org/grid_data/download/70303666?include=energy
Gltpd2	0.954	http://api.brain-map.org/grid_data/download/71724467?include=energy
Zfp523	0.954	http://api.brain-map.org/grid_data/download/74581205?include=energy
Fam162b	0.954	http://api.brain-map.org/grid_data/download/71892424?include=energy
Ergic3	0.954	http://api.brain-map.org/grid_data/download/68920713?include=energy
Slc2a5	0.954	http://api.brain-map.org/grid_data/download/2602?include=energy
Tubg2	0.954	http://api.brain-map.org/grid_data/download/68632978?include=energy
Frat2	0.954	http://api.brain-map.org/grid_data/download/1553?include=energy
Ccdc125	0.954	http://api.brain-map.org/grid_data/download/71281779?include=energy
Pigz	0.954	http://api.brain-map.org/grid_data/download/69672204?include=energy
Dus2l	0.954	http://api.brain-map.org/grid_data/download/69202382?include=energy
3632451O06Rik	0.954	http://api.brain-map.org/grid_data/download/70928306?include=energy
Rev1	0.954	http://api.brain-map.org/grid_data/download/68919940?include=energy
Emcn	0.954	http://api.brain-map.org/grid_data/download/68076551?include=energy
Stc1	0.954	http://api.brain-map.org/grid_data/download/2639?include=energy
Uros	0.954	http://api.brain-map.org/grid_data/download/70194600?include=energy
Dos	0.954	http://api.brain-map.org/grid_data/download/74882515?include=energy
Ubr5	0.954	http://api.brain-map.org/grid_data/download/69014937?include=energy
Mm.5547	0.954	http://api.brain-map.org/grid_data/download/73635471?include=energy
Reps2	0.954	http://api.brain-map.org/grid_data/download/76135799?include=energy
LOC433761	0.954	http://api.brain-map.org/grid_data/download/70786767?include=energy
Ndufa9	0.954	http://api.brain-map.org/grid_data/download/70523706?include=energy
Txndc9	0.954	http://api.brain-map.org/grid_data/download/68546416?include=energy
Foxi2	0.954	http://api.brain-map.org/grid_data/download/70231122?include=energy
Saa4	0.954	http://api.brain-map.org/grid_data/download/77800919?include=energy
Serhl	0.954	http://api.brain-map.org/grid_data/download/69014866?include=energy
Tslp	0.954	http://api.brain-map.org/grid_data/download/75931601?include=energy
Rap1gds1	0.954	http://api.brain-map.org/grid_data/download/654924?include=energy
Ppp3r2	0.954	http://api.brain-map.org/grid_data/download/75084112?include=energy
AI413582	0.954	http://api.brain-map.org/grid_data/download/68522518?include=energy
D730039F16Rik	0.954	http://api.brain-map.org/grid_data/download/68341997?include=energy
Lpcat2	0.954	http://api.brain-map.org/grid_data/download/70305251?include=energy
Iqgap1	0.954	http://api.brain-map.org/grid_data/download/69262296?include=energy
Ninl	0.954	http://api.brain-map.org/grid_data/download/73512540?include=energy
Athl1	0.954	http://api.brain-map.org/grid_data/download/68444946?include=energy
H1foo	0.954	http://api.brain-map.org/grid_data/download/71832824?include=energy
Ttll5	0.954	http://api.brain-map.org/grid_data/download/68157391?include=energy
Lmo2	0.954	http://api.brain-map.org/grid_data/download/2383?include=energy
Ide	0.954	http://api.brain-map.org/grid_data/download/68845759?include=energy
Mm.360537	0.954	http://api.brain-map.org/grid_data/download/71209994?include=energy
Rnf10	0.954	http://api.brain-map.org/grid_data/download/67936005?include=energy
Rcn3	0.954	http://api.brain-map.org/grid_data/download/68498896?include=energy
Fermt1	0.954	http://api.brain-map.org/grid_data/download/68443531?include=energy
Rhox13	0.954	http://api.brain-map.org/grid_data/download/74635108?include=energy
Calcrl	0.954	http://api.brain-map.org/grid_data/download/74988670?include=energy
Abcc4	0.954	http://api.brain-map.org/grid_data/download/75042241?include=energy
Psip1	0.954	http://api.brain-map.org/grid_data/download/70565135?include=energy
Man2c1	0.954	http://api.brain-map.org/grid_data/download/77620493?include=energy
P2ry14	0.954	http://api.brain-map.org/grid_data/download/76135748?include=energy
Cpa2	0.954	http://api.brain-map.org/grid_data/download/70566100?include=energy
Fam108c	0.954	http://api.brain-map.org/grid_data/download/68341609?include=energy
Gm1540	0.954	http://api.brain-map.org/grid_data/download/74821910?include=energy
Rsad1	0.954	http://api.brain-map.org/grid_data/download/69529167?include=energy
Dmrtc1c2	0.953	http://api.brain-map.org/grid_data/download/71892203?include=energy
Cldn13	0.953	http://api.brain-map.org/grid_data/download/70237932?include=energy
Bnip3	0.953	http://api.brain-map.org/grid_data/download/75077193?include=energy
Iqcj	0.953	http://api.brain-map.org/grid_data/download/74272918?include=energy
Chmp3	0.953	http://api.brain-map.org/grid_data/download/69013477?include=energy
Son	0.953	http://api.brain-map.org/grid_data/download/996?include=energy
Camk2b	0.953	http://api.brain-map.org/grid_data/download/70719036?include=energy
Ascl2	0.953	http://api.brain-map.org/grid_data/download/1156?include=energy
Tpm3	0.953	http://api.brain-map.org/grid_data/download/275873?include=energy
Vps53	0.953	http://api.brain-map.org/grid_data/download/68563092?include=energy
Sfxn4	0.953	http://api.brain-map.org/grid_data/download/71570747?include=energy
Sox15	0.953	http://api.brain-map.org/grid_data/download/73606540?include=energy
Fbxl2	0.953	http://api.brain-map.org/grid_data/download/69672312?include=energy
Ifi203	0.953	http://api.brain-map.org/grid_data/download/71487118?include=energy
Irgm1	0.953	http://api.brain-map.org/grid_data/download/69735228?include=energy
1810033B17Rik	0.953	http://api.brain-map.org/grid_data/download/70201576?include=energy
Uts2	0.953	http://api.brain-map.org/grid_data/download/100144180?include=energy
Sept1	0.953	http://api.brain-map.org/grid_data/download/100144886?include=energy
A530075A22Rik*	0.953	http://api.brain-map.org/grid_data/download/73808594?include=energy
Stmn2	0.953	http://api.brain-map.org/grid_data/download/74819695?include=energy
5830403L16Rik	0.953	http://api.brain-map.org/grid_data/download/69526462?include=energy
Lhfpl4	0.953	http://api.brain-map.org/grid_data/download/70231170?include=energy
Fkbp4	0.953	http://api.brain-map.org/grid_data/download/67978476?include=energy
Epha2	0.953	http://api.brain-map.org/grid_data/download/69134601?include=energy
Gm53	0.953	http://api.brain-map.org/grid_data/download/70295530?include=energy
Gm16897	0.953	http://api.brain-map.org/grid_data/download/80342165?include=energy
Akna	0.953	http://api.brain-map.org/grid_data/download/81657671?include=energy
5430414B19Rik	0.953	http://api.brain-map.org/grid_data/download/74580799?include=energy
Pkdrej	0.953	http://api.brain-map.org/grid_data/download/75985900?include=energy
Cpm	0.953	http://api.brain-map.org/grid_data/download/71021163?include=energy
Gusb	0.953	http://api.brain-map.org/grid_data/download/69134262?include=energy
Plekha5	0.953	http://api.brain-map.org/grid_data/download/70743870?include=energy
Stac2	0.953	http://api.brain-map.org/grid_data/download/293472?include=energy
Map3k12	0.953	http://api.brain-map.org/grid_data/download/71656802?include=energy
Aasdhppt	0.953	http://api.brain-map.org/grid_data/download/68924545?include=energy
Kif1a	0.953	http://api.brain-map.org/grid_data/download/69735759?include=energy
Sorcs1	0.953	http://api.brain-map.org/grid_data/download/74800882?include=energy
Tsen34	0.953	http://api.brain-map.org/grid_data/download/1944?include=energy
A330017A19Rik	0.953	http://api.brain-map.org/grid_data/download/70228388?include=energy
Grin1	0.953	http://api.brain-map.org/grid_data/download/1585?include=energy
Nedd9	0.953	http://api.brain-map.org/grid_data/download/77790732?include=energy
Jph4	0.953	http://api.brain-map.org/grid_data/download/75831724?include=energy
Metrn	0.953	http://api.brain-map.org/grid_data/download/69838736?include=energy
Gpr56	0.953	http://api.brain-map.org/grid_data/download/71015815?include=energy
Hnrnph3	0.953	http://api.brain-map.org/grid_data/download/70608547?include=energy
Plk4	0.953	http://api.brain-map.org/grid_data/download/75828170?include=energy
Ppp1r10	0.953	http://api.brain-map.org/grid_data/download/68911019?include=energy
Phb	0.953	http://api.brain-map.org/grid_data/download/69783071?include=energy
Wdr20b	0.953	http://api.brain-map.org/grid_data/download/70565419?include=energy
Uqcc	0.953	http://api.brain-map.org/grid_data/download/69202609?include=energy
LOC235953	0.953	http://api.brain-map.org/grid_data/download/73931419?include=energy
Ina	0.953	http://api.brain-map.org/grid_data/download/71836833?include=energy
Cdh5	0.953	http://api.brain-map.org/grid_data/download/77931975?include=energy
Cdk16	0.953	http://api.brain-map.org/grid_data/download/70429345?include=energy
Cbr2	0.953	http://api.brain-map.org/grid_data/download/68341424?include=energy
Galp	0.953	http://api.brain-map.org/grid_data/download/79556611?include=energy
LOC436350	0.953	http://api.brain-map.org/grid_data/download/77620847?include=energy
Slc22a30	0.953	http://api.brain-map.org/grid_data/download/69262323?include=energy
Vmn1r185	0.953	http://api.brain-map.org/grid_data/download/70785297?include=energy
Apon	0.953	http://api.brain-map.org/grid_data/download/68499087?include=energy
Cyp4f41-ps	0.953	http://api.brain-map.org/grid_data/download/70227860?include=energy
Pabpn1	0.953	http://api.brain-map.org/grid_data/download/74431266?include=energy
Clta	0.952	http://api.brain-map.org/grid_data/download/74819690?include=energy
Ift122	0.952	http://api.brain-map.org/grid_data/download/70194976?include=energy
Rab21	0.952	http://api.brain-map.org/grid_data/download/69013678?include=energy
Tssk2	0.952	http://api.brain-map.org/grid_data/download/69116823?include=energy
Cpt1a	0.952	http://api.brain-map.org/grid_data/download/1478?include=energy
Nodal	0.952	http://api.brain-map.org/grid_data/download/69782288?include=energy
Nr1i3	0.952	http://api.brain-map.org/grid_data/download/69170186?include=energy
9030203C11Rik	0.952	http://api.brain-map.org/grid_data/download/69446304?include=energy
Slc6a7	0.952	http://api.brain-map.org/grid_data/download/71016653?include=energy
Kcnab3	0.952	http://api.brain-map.org/grid_data/download/73817925?include=energy
Ace2	0.952	http://api.brain-map.org/grid_data/download/69015769?include=energy
Spint2	0.952	http://api.brain-map.org/grid_data/download/74047774?include=energy
Slc17a2	0.952	http://api.brain-map.org/grid_data/download/68302017?include=energy
Agpat6	0.952	http://api.brain-map.org/grid_data/download/69080049?include=energy
LOC433946	0.952	http://api.brain-map.org/grid_data/download/70787131?include=energy
Pepd	0.952	http://api.brain-map.org/grid_data/download/69782975?include=energy
Trp73	0.952	http://api.brain-map.org/grid_data/download/73929579?include=energy
Dnahc9	0.952	http://api.brain-map.org/grid_data/download/72472774?include=energy
Cgref1	0.952	http://api.brain-map.org/grid_data/download/532681?include=energy
9230109A22Rik	0.952	http://api.brain-map.org/grid_data/download/70717375?include=energy
Cadm4	0.952	http://api.brain-map.org/grid_data/download/69735299?include=energy
6330407J23Rik	0.952	http://api.brain-map.org/grid_data/download/1988?include=energy
Fam110c	0.952	http://api.brain-map.org/grid_data/download/75197630?include=energy
Dnajc5	0.952	http://api.brain-map.org/grid_data/download/68795442?include=energy
LOC432605	0.952	http://api.brain-map.org/grid_data/download/70611102?include=energy
Sh2b2	0.952	http://api.brain-map.org/grid_data/download/69838287?include=energy
Ccdc7	0.952	http://api.brain-map.org/grid_data/download/71891910?include=energy
1700084K02Rik	0.952	http://api.brain-map.org/grid_data/download/74357923?include=energy
Nat8l	0.952	http://api.brain-map.org/grid_data/download/74958656?include=energy
Guca2a	0.952	http://api.brain-map.org/grid_data/download/69095980?include=energy
Bhlhe22	0.952	http://api.brain-map.org/grid_data/download/1162?include=energy
Ei24	0.952	http://api.brain-map.org/grid_data/download/2179?include=energy
Wwp1	0.952	http://api.brain-map.org/grid_data/download/69817079?include=energy
Mapk8ip3	0.952	http://api.brain-map.org/grid_data/download/69752065?include=energy
Tbkbp1	0.952	http://api.brain-map.org/grid_data/download/68861858?include=energy
Utrn	0.952	http://api.brain-map.org/grid_data/download/73929872?include=energy
Tmem205	0.952	http://api.brain-map.org/grid_data/download/68341466?include=energy
Trim7	0.952	http://api.brain-map.org/grid_data/download/70302830?include=energy
3830406C13Rik	0.952	http://api.brain-map.org/grid_data/download/77414919?include=energy
Itpkb	0.952	http://api.brain-map.org/grid_data/download/73520992?include=energy
Taf7	0.952	http://api.brain-map.org/grid_data/download/71656061?include=energy
D19Wsu162e	0.952	http://api.brain-map.org/grid_data/download/68498552?include=energy
Spag1	0.952	http://api.brain-map.org/grid_data/download/68745221?include=energy
Cst8	0.952	http://api.brain-map.org/grid_data/download/70525777?include=energy
Cdo1	0.952	http://api.brain-map.org/grid_data/download/74583197?include=energy
Fbxw26	0.952	http://api.brain-map.org/grid_data/download/77620858?include=energy
Ythdf3	0.952	http://api.brain-map.org/grid_data/download/69028420?include=energy
9230014I20Rik*	0.952	http://api.brain-map.org/grid_data/download/73906167?include=energy
4930465K10Rik	0.952	http://api.brain-map.org/grid_data/download/70218175?include=energy
Mapk1ip1	0.952	http://api.brain-map.org/grid_data/download/71493002?include=energy
Mm.349813	0.952	http://api.brain-map.org/grid_data/download/71161102?include=energy
Nmrk1	0.952	http://api.brain-map.org/grid_data/download/68666607?include=energy
1700008P02Rik	0.952	http://api.brain-map.org/grid_data/download/71919421?include=energy
Kif26b	0.952	http://api.brain-map.org/grid_data/download/71717582?include=energy
Pld2	0.952	http://api.brain-map.org/grid_data/download/68745396?include=energy
Rps18	0.952	http://api.brain-map.org/grid_data/download/69873283?include=energy
Sdad1	0.952	http://api.brain-map.org/grid_data/download/69262282?include=energy
Mrpl19	0.951	http://api.brain-map.org/grid_data/download/68845747?include=energy
Pnmt	0.951	http://api.brain-map.org/grid_data/download/76081013?include=energy
Trip4	0.951	http://api.brain-map.org/grid_data/download/1924?include=energy
Gm10339	0.951	http://api.brain-map.org/grid_data/download/74427103?include=energy
Stra8	0.951	http://api.brain-map.org/grid_data/download/69887385?include=energy
Prpsap2	0.951	http://api.brain-map.org/grid_data/download/293663?include=energy
Spnb2	0.951	http://api.brain-map.org/grid_data/download/69885696?include=energy
Ccdc38	0.951	http://api.brain-map.org/grid_data/download/74513923?include=energy
Hlf	0.951	http://api.brain-map.org/grid_data/download/565?include=energy
Cacna1s	0.951	http://api.brain-map.org/grid_data/download/70723167?include=energy
Barx2	0.951	http://api.brain-map.org/grid_data/download/2057?include=energy
Ms4a6c	0.951	http://api.brain-map.org/grid_data/download/68744158?include=energy
Gpr84	0.951	http://api.brain-map.org/grid_data/download/1583?include=energy
2310035C23Rik	0.951	http://api.brain-map.org/grid_data/download/69202454?include=energy
Ftsj1	0.951	http://api.brain-map.org/grid_data/download/68302172?include=energy
4930550G17Rik	0.951	http://api.brain-map.org/grid_data/download/70218348?include=energy
Rbbp6	0.951	http://api.brain-map.org/grid_data/download/170?include=energy
Ctsa	0.951	http://api.brain-map.org/grid_data/download/512767?include=energy
Hectd2	0.951	http://api.brain-map.org/grid_data/download/2002?include=energy
4930539E08Rik	0.951	http://api.brain-map.org/grid_data/download/70218277?include=energy
Ccdc155	0.951	http://api.brain-map.org/grid_data/download/68667162?include=energy
Slco1a1	0.951	http://api.brain-map.org/grid_data/download/69838239?include=energy
LOC433345	0.951	http://api.brain-map.org/grid_data/download/70723128?include=energy
Olfr1443	0.951	http://api.brain-map.org/grid_data/download/74720838?include=energy
Smn1	0.951	http://api.brain-map.org/grid_data/download/68924632?include=energy
Gm702	0.951	http://api.brain-map.org/grid_data/download/70298268?include=energy
4931414P19Rik	0.951	http://api.brain-map.org/grid_data/download/100142523?include=energy
Jakmip3	0.951	http://api.brain-map.org/grid_data/download/71892298?include=energy
Arpp21	0.951	http://api.brain-map.org/grid_data/download/71587781?include=energy
Cenpc1	0.951	http://api.brain-map.org/grid_data/download/69548801?include=energy
Nes	0.951	http://api.brain-map.org/grid_data/download/1387?include=energy
Klk9	0.951	http://api.brain-map.org/grid_data/download/75931618?include=energy
2610005L07Rik	0.951	http://api.brain-map.org/grid_data/download/74635608?include=energy
Adam34	0.951	http://api.brain-map.org/grid_data/download/76080969?include=energy
Arrb2	0.951	http://api.brain-map.org/grid_data/download/71992067?include=energy
Cobra1	0.951	http://api.brain-map.org/grid_data/download/69095969?include=energy
4930510E17Rik	0.951	http://api.brain-map.org/grid_data/download/71570759?include=energy
LOC432657	0.951	http://api.brain-map.org/grid_data/download/71765040?include=energy
Sav1	0.951	http://api.brain-map.org/grid_data/download/68410291?include=energy
Bop1	0.951	http://api.brain-map.org/grid_data/download/69059816?include=energy
Cyp2r1	0.951	http://api.brain-map.org/grid_data/download/71579960?include=energy
Arhgef3	0.951	http://api.brain-map.org/grid_data/download/69080048?include=energy
Erdr1	0.951	http://api.brain-map.org/grid_data/download/378?include=energy
Tyk2	0.951	http://api.brain-map.org/grid_data/download/70192171?include=energy
9530004P13Rik	0.951	http://api.brain-map.org/grid_data/download/74300675?include=energy
Fancb	0.951	http://api.brain-map.org/grid_data/download/67815963?include=energy
Zfat	0.951	http://api.brain-map.org/grid_data/download/71656705?include=energy
Asb11	0.951	http://api.brain-map.org/grid_data/download/71836891?include=energy
Plp2	0.951	http://api.brain-map.org/grid_data/download/71377711?include=energy
Zfp536	0.951	http://api.brain-map.org/grid_data/download/71836801?include=energy
Rplp0	0.951	http://api.brain-map.org/grid_data/download/74047927?include=energy
Nlgn3	0.951	http://api.brain-map.org/grid_data/download/70300559?include=energy
Atp10d	0.951	http://api.brain-map.org/grid_data/download/70593127?include=energy
Nphp3	0.951	http://api.brain-map.org/grid_data/download/76003713?include=energy
Ysk4	0.951	http://api.brain-map.org/grid_data/download/75988578?include=energy
4931406P16Rik	0.951	http://api.brain-map.org/grid_data/download/70218468?include=energy
Upb1	0.951	http://api.brain-map.org/grid_data/download/68445961?include=energy
Fbxo25	0.951	http://api.brain-map.org/grid_data/download/69015685?include=energy
Gnpnat1	0.951	http://api.brain-map.org/grid_data/download/2267?include=energy
Clcnkb	0.951	http://api.brain-map.org/grid_data/download/71627818?include=energy
St3gal3	0.951	http://api.brain-map.org/grid_data/download/2567?include=energy
Rab7	0.951	http://api.brain-map.org/grid_data/download/532633?include=energy
Isoc1	0.951	http://api.brain-map.org/grid_data/download/73732145?include=energy
Cd53	0.951	http://api.brain-map.org/grid_data/download/68445203?include=energy
Xlr3a	0.951	http://api.brain-map.org/grid_data/download/75650861?include=energy
Abcg1	0.951	http://api.brain-map.org/grid_data/download/69514624?include=energy
Mtss1	0.95	http://api.brain-map.org/grid_data/download/1776?include=energy
Iapp	0.95	http://api.brain-map.org/grid_data/download/68744665?include=energy
Tdrd5	0.95	http://api.brain-map.org/grid_data/download/74562296?include=energy

Fcna	0.95	http://api.brain-map.org/grid_data/download/68193044?include=energy
Prdx2	0.95	http://api.brain-map.org/grid_data/download/75233570?include=energy
Gm543	0.95	http://api.brain-map.org/grid_data/download/70295775?include=energy
C1qtnf3	0.95	http://api.brain-map.org/grid_data/download/75041550?include=energy
Sftpc	0.95	http://api.brain-map.org/grid_data/download/69873654?include=energy
F530204I15Rik*	0.95	http://api.brain-map.org/grid_data/download/73767392?include=energy
Ngdn	0.95	http://api.brain-map.org/grid_data/download/68743966?include=energy
Atg4b	0.95	http://api.brain-map.org/grid_data/download/532689?include=energy
Il27ra	0.95	http://api.brain-map.org/grid_data/download/69549170?include=energy
Actl6b	0.95	http://api.brain-map.org/grid_data/download/69105315?include=energy
Gga2	0.95	http://api.brain-map.org/grid_data/download/68843931?include=energy
Cops8	0.95	http://api.brain-map.org/grid_data/download/67765696?include=energy
Ano6	0.95	http://api.brain-map.org/grid_data/download/71924067?include=energy
5830477G23Rik	0.95	http://api.brain-map.org/grid_data/download/71892304?include=energy
Rhox9	0.95	http://api.brain-map.org/grid_data/download/81655580?include=energy
Nmnat3	0.95	http://api.brain-map.org/grid_data/download/77792022?include=energy
LOC434184	0.95	http://api.brain-map.org/grid_data/download/72471791?include=energy
Synj1	0.95	http://api.brain-map.org/grid_data/download/1287?include=energy
Kdm3a	0.95	http://api.brain-map.org/grid_data/download/68844570?include=energy
2700062C07Rik	0.95	http://api.brain-map.org/grid_data/download/75750457?include=energy
Gm14296	0.95	http://api.brain-map.org/grid_data/download/68798825?include=energy
Mdga2	0.95	http://api.brain-map.org/grid_data/download/70300276?include=energy
Capzb	0.95	http://api.brain-map.org/grid_data/download/75080633?include=energy
Syt1	0.95	http://api.brain-map.org/grid_data/download/1014?include=energy
C030007I09Rik	0.95	http://api.brain-map.org/grid_data/download/69530708?include=energy
Spast	0.95	http://api.brain-map.org/grid_data/download/71161435?include=energy
Josd2	0.95	http://api.brain-map.org/grid_data/download/69015817?include=energy
4933427G23Rik	0.95	http://api.brain-map.org/grid_data/download/71764879?include=energy
Rnh1	0.95	http://api.brain-map.org/grid_data/download/74800925?include=energy
Ppfia3	0.95	http://api.brain-map.org/grid_data/download/69202693?include=energy
Olfr561	0.95	http://api.brain-map.org/grid_data/download/73653446?include=energy
Sec24a	0.95	http://api.brain-map.org/grid_data/download/69873493?include=energy
Cd8b1	0.95	http://api.brain-map.org/grid_data/download/73732149?include=energy
Dcpp1	0.95	http://api.brain-map.org/grid_data/download/76098358?include=energy
LOC434284	0.95	http://api.brain-map.org/grid_data/download/71992013?include=energy
Nt5m	0.95	http://api.brain-map.org/grid_data/download/74658088?include=energy
Nfatc3	0.95	http://api.brain-map.org/grid_data/download/75934935?include=energy
G830039O19Rik*	0.95	http://api.brain-map.org/grid_data/download/73716782?include=energy
Arl16	0.95	http://api.brain-map.org/grid_data/download/73817933?include=energy
Calr3	0.95	http://api.brain-map.org/grid_data/download/69540296?include=energy
4933427I22Rik	0.95	http://api.brain-map.org/grid_data/download/69443232?include=energy
Ciao1	0.95	http://api.brain-map.org/grid_data/download/2051?include=energy
Fam160b2	0.95	http://api.brain-map.org/grid_data/download/69817922?include=energy
F630050F06Rik*	0.95	http://api.brain-map.org/grid_data/download/73731379?include=energy
Taf9	0.95	http://api.brain-map.org/grid_data/download/81654147?include=energy
Gp1bb	0.95	http://api.brain-map.org/grid_data/download/75990859?include=energy
Cops5	0.95	http://api.brain-map.org/grid_data/download/69568944?include=energy
Gm667	0.95	http://api.brain-map.org/grid_data/download/71668560?include=energy
2900092C05Rik	0.95	http://api.brain-map.org/grid_data/download/71991941?include=energy
Pick1	0.95	http://api.brain-map.org/grid_data/download/68924560?include=energy
Rnf170	0.95	http://api.brain-map.org/grid_data/download/68080700?include=energy
Gtf2f1	0.95	http://api.brain-map.org/grid_data/download/67870080?include=energy
2610021A01Rik	0.95	http://api.brain-map.org/grid_data/download/69202836?include=energy
Scaf1	0.95	http://api.brain-map.org/grid_data/download/73497253?include=energy
Cacna1f	0.95	http://api.brain-map.org/grid_data/download/69236903?include=energy
LOC436226	0.95	http://api.brain-map.org/grid_data/download/70784600?include=energy
Capn5	0.95	http://api.brain-map.org/grid_data/download/69015476?include=energy
Fermt2	0.95	http://api.brain-map.org/grid_data/download/68499479?include=energy
Aimp2	0.95	http://api.brain-map.org/grid_data/download/67814204?include=energy
Pgrmc2	0.95	http://api.brain-map.org/grid_data/download/74882697?include=energy
Phf17	0.949	http://api.brain-map.org/grid_data/download/68117987?include=energy
Fcgrt	0.949	http://api.brain-map.org/grid_data/download/79544790?include=energy
Hdgf	0.949	http://api.brain-map.org/grid_data/download/2285?include=energy
Ccdc30	0.949	http://api.brain-map.org/grid_data/download/70201432?include=energy
Agrn	0.949	http://api.brain-map.org/grid_data/download/73925722?include=energy
Mrpl12	0.949	http://api.brain-map.org/grid_data/download/70743841?include=energy
Pfdn4	0.949	http://api.brain-map.org/grid_data/download/74882686?include=energy
Cbx1	0.949	http://api.brain-map.org/grid_data/download/77454690?include=energy
Cpeb2	0.949	http://api.brain-map.org/grid_data/download/74510236?include=energy
Dnajc24	0.949	http://api.brain-map.org/grid_data/download/68545905?include=energy
Cnn2	0.949	http://api.brain-map.org/grid_data/download/76081897?include=energy
Bbs7	0.949	http://api.brain-map.org/grid_data/download/67767447?include=energy
Vipr2	0.949	http://api.brain-map.org/grid_data/download/1104?include=energy
Mettl13	0.949	http://api.brain-map.org/grid_data/download/70445496?include=energy
Tep1	0.949	http://api.brain-map.org/grid_data/download/69980262?include=energy
Rbbp7	0.949	http://api.brain-map.org/grid_data/download/512775?include=energy
Scly	0.949	http://api.brain-map.org/grid_data/download/68445943?include=energy
Sgcb	0.949	http://api.brain-map.org/grid_data/download/69549654?include=energy
Gm12666	0.949	http://api.brain-map.org/grid_data/download/68341854?include=energy
Dll4	0.949	http://api.brain-map.org/grid_data/download/100144503?include=energy
Atp6v1h	0.949	http://api.brain-map.org/grid_data/download/70525808?include=energy
Rasa1	0.949	http://api.brain-map.org/grid_data/download/71610829?include=energy
Ppp1r9b	0.949	http://api.brain-map.org/grid_data/download/74271623?include=energy
Gm9517	0.949	http://api.brain-map.org/grid_data/download/76003374?include=energy
Cep78	0.949	http://api.brain-map.org/grid_data/download/322963?include=energy
Mtmr7	0.949	http://api.brain-map.org/grid_data/download/79567753?include=energy
Traf3	0.949	http://api.brain-map.org/grid_data/download/70192021?include=energy
Mtg1	0.949	http://api.brain-map.org/grid_data/download/69169624?include=energy
Mrm1	0.949	http://api.brain-map.org/grid_data/download/71494696?include=energy
Actn2	0.949	http://api.brain-map.org/grid_data/download/70724523?include=energy
Ly6g6d	0.949	http://api.brain-map.org/grid_data/download/74743333?include=energy
Nudt18	0.949	http://api.brain-map.org/grid_data/download/69095974?include=energy
D7Ertd443e	0.949	http://api.brain-map.org/grid_data/download/70717361?include=energy
Dhcr24	0.949	http://api.brain-map.org/grid_data/download/71016721?include=energy
Il18r1	0.949	http://api.brain-map.org/grid_data/download/75831726?include=energy
Hrasls5	0.949	http://api.brain-map.org/grid_data/download/75860955?include=energy
Ccdc122	0.949	http://api.brain-map.org/grid_data/download/70218786?include=energy
Slu7	0.949	http://api.brain-map.org/grid_data/download/73513678?include=energy
Agpat4	0.949	http://api.brain-map.org/grid_data/download/73718048?include=energy
Trim17	0.949	http://api.brain-map.org/grid_data/download/69113919?include=energy
Gm614	0.949	http://api.brain-map.org/grid_data/download/71991983?include=energy
LOC435959	0.949	http://api.brain-map.org/grid_data/download/71022627?include=energy
Vps33b	0.949	http://api.brain-map.org/grid_data/download/69173250?include=energy
Vps35	0.949	http://api.brain-map.org/grid_data/download/355884?include=energy
Btbd6	0.949	http://api.brain-map.org/grid_data/download/69236682?include=energy
Zfp949	0.949	http://api.brain-map.org/grid_data/download/69407929?include=energy
P4ha2	0.949	http://api.brain-map.org/grid_data/download/73732152?include=energy
Gm1611	0.949	http://api.brain-map.org/grid_data/download/71570763?include=energy
Acyp1	0.949	http://api.brain-map.org/grid_data/download/74988760?include=energy
Calm2	0.949	http://api.brain-map.org/grid_data/download/182?include=energy
Kazald1	0.949	http://api.brain-map.org/grid_data/download/71764637?include=energy
Eno2	0.948	http://api.brain-map.org/grid_data/download/112197625?include=energy
Acan	0.948	http://api.brain-map.org/grid_data/download/72128777?include=energy
Caln1	0.948	http://api.brain-map.org/grid_data/download/69540290?include=energy
Slc4a4	0.948	http://api.brain-map.org/grid_data/download/78534526?include=energy
Pfkp	0.948	http://api.brain-map.org/grid_data/download/79490112?include=energy
Flt4	0.948	http://api.brain-map.org/grid_data/download/69672498?include=energy
Asb13	0.948	http://api.brain-map.org/grid_data/download/72081558?include=energy
Gpc3	0.948	http://api.brain-map.org/grid_data/download/71020431?include=energy
Crb2	0.948	http://api.brain-map.org/grid_data/download/69529125?include=energy
Evi2b	0.948	http://api.brain-map.org/grid_data/download/68410189?include=energy
Vmn1r32	0.948	http://api.brain-map.org/grid_data/download/75931579?include=energy
2010109A12Rik	0.948	http://api.brain-map.org/grid_data/download/69854021?include=energy
LOC433812	0.948	http://api.brain-map.org/grid_data/download/70786827?include=energy
Eif4h	0.948	http://api.brain-map.org/grid_data/download/513227?include=energy
Olfr77	0.948	http://api.brain-map.org/grid_data/download/71764168?include=energy
Rps6kb2	0.948	http://api.brain-map.org/grid_data/download/69169349?include=energy
Tecta	0.948	http://api.brain-map.org/grid_data/download/74882940?include=energy
Zfp113	0.948	http://api.brain-map.org/grid_data/download/70445317?include=energy
Gm51	0.948	http://api.brain-map.org/grid_data/download/71789963?include=energy
Tmem38a	0.948	http://api.brain-map.org/grid_data/download/71612429?include=energy
LOC433077	0.948	http://api.brain-map.org/grid_data/download/70611485?include=energy
Zfp931	0.948	http://api.brain-map.org/grid_data/download/70813137?include=energy
A730027D23Rik*	0.948	http://api.brain-map.org/grid_data/download/74000474?include=energy
Tmem179	0.948	http://api.brain-map.org/grid_data/download/76097701?include=energy
LOC433695	0.948	http://api.brain-map.org/grid_data/download/74562582?include=energy
Smg7	0.948	http://api.brain-map.org/grid_data/download/75197642?include=energy
Wbp5	0.948	http://api.brain-map.org/grid_data/download/71717602?include=energy
Tnnc2	0.948	http://api.brain-map.org/grid_data/download/69236688?include=energy
AU019823	0.948	http://api.brain-map.org/grid_data/download/77465050?include=energy
1700001G17Rik	0.948	http://api.brain-map.org/grid_data/download/74581301?include=energy
Ube2c	0.948	http://api.brain-map.org/grid_data/download/70194504?include=energy
Maged1	0.948	http://api.brain-map.org/grid_data/download/69015619?include=energy
Atp13a2	0.948	http://api.brain-map.org/grid_data/download/68160844?include=energy
Vmn1r74	0.948	http://api.brain-map.org/grid_data/download/70785672?include=energy
Srcrb4d	0.948	http://api.brain-map.org/grid_data/download/70302258?include=energy
Rhpn2	0.948	http://api.brain-map.org/grid_data/download/100146042?include=energy
Clec10a	0.948	http://api.brain-map.org/grid_data/download/69015637?include=energy
Kiss1	0.948	http://api.brain-map.org/grid_data/download/79912608?include=energy
Ppp2ca	0.948	http://api.brain-map.org/grid_data/download/71836836?include=energy
Srrm2	0.948	http://api.brain-map.org/grid_data/download/77620766?include=energy
T	0.948	http://api.brain-map.org/grid_data/download/69290561?include=energy
LOC545780	0.948	http://api.brain-map.org/grid_data/download/68667461?include=energy
Ccr10	0.948	http://api.brain-map.org/grid_data/download/74724659?include=energy
Ccdc163	0.948	http://api.brain-map.org/grid_data/download/67881750?include=energy
Gpr37l1	0.948	http://api.brain-map.org/grid_data/download/74724670?include=energy
AW554918	0.948	http://api.brain-map.org/grid_data/download/76003630?include=energy
Cela1	0.948	http://api.brain-map.org/grid_data/download/68302362?include=energy
Rpl23	0.948	http://api.brain-map.org/grid_data/download/74819237?include=energy
A930005I04Rik	0.948	http://api.brain-map.org/grid_data/download/71021006?include=energy
Psmd4	0.948	http://api.brain-map.org/grid_data/download/2503?include=energy
D030018L15Rik	0.948	http://api.brain-map.org/grid_data/download/70238052?include=energy
Pppde1	0.948	http://api.brain-map.org/grid_data/download/70445514?include=energy
Scrt2	0.948	http://api.brain-map.org/grid_data/download/71211383?include=energy
6030408B16Rik	0.948	http://api.brain-map.org/grid_data/download/73617588?include=energy
Cog2	0.948	http://api.brain-map.org/grid_data/download/68301796?include=energy
Tex101	0.948	http://api.brain-map.org/grid_data/download/69113889?include=energy
Asb7	0.948	http://api.brain-map.org/grid_data/download/77465064?include=energy
Rasgrp4	0.948	http://api.brain-map.org/grid_data/download/76135807?include=energy
Naf1	0.948	http://api.brain-map.org/grid_data/download/69529161?include=energy
Rogdi	0.948	http://api.brain-map.org/grid_data/download/76081947?include=energy
Trim26	0.948	http://api.brain-map.org/grid_data/download/70565345?include=energy
Zfp692	0.947	http://api.brain-map.org/grid_data/download/70946145?include=energy
Mcpt9	0.947	http://api.brain-map.org/grid_data/download/74743339?include=energy
4732491K20Rik	0.947	http://api.brain-map.org/grid_data/download/69407726?include=energy
Pax3	0.947	http://api.brain-map.org/grid_data/download/2464?include=energy
Ostn	0.947	http://api.brain-map.org/grid_data/download/74581440?include=energy
5330439B14Rik	0.947	http://api.brain-map.org/grid_data/download/71493741?include=energy
Slc7a2	0.947	http://api.brain-map.org/grid_data/download/71570750?include=energy
Ttpa	0.947	http://api.brain-map.org/grid_data/download/72004451?include=energy
Sval2	0.947	http://api.brain-map.org/grid_data/download/70785140?include=energy
Mark1	0.947	http://api.brain-map.org/grid_data/download/71789795?include=energy
Psmd1	0.947	http://api.brain-map.org/grid_data/download/74059253?include=energy
Cdca7l	0.947	http://api.brain-map.org/grid_data/download/633692?include=energy
Cyp2j11	0.947	http://api.brain-map.org/grid_data/download/71764787?include=energy
Nt5dc3	0.947	http://api.brain-map.org/grid_data/download/73992926?include=energy
Ppap2b	0.947	http://api.brain-map.org/grid_data/download/75749384?include=energy
Abhd10	0.947	http://api.brain-map.org/grid_data/download/69262229?include=energy
Tnks	0.947	http://api.brain-map.org/grid_data/download/72472775?include=energy
LOC434413	0.947	http://api.brain-map.org/grid_data/download/71022298?include=energy
Afg3l2	0.947	http://api.brain-map.org/grid_data/download/70445568?include=energy
Agtr1a	0.947	http://api.brain-map.org/grid_data/download/2021?include=energy
4930473A06Rik	0.947	http://api.brain-map.org/grid_data/download/75080180?include=energy
Ndor1	0.947	http://api.brain-map.org/grid_data/download/68797924?include=energy
Fgf14	0.947	http://api.brain-map.org/grid_data/download/100145371?include=energy
Msx3	0.947	http://api.brain-map.org/grid_data/download/69289417?include=energy
Ecm1	0.947	http://api.brain-map.org/grid_data/download/69671875?include=energy
Copz2	0.947	http://api.brain-map.org/grid_data/download/275874?include=energy
Snapc3	0.947	http://api.brain-map.org/grid_data/download/68797876?include=energy
Fam134a	0.947	http://api.brain-map.org/grid_data/download/386010?include=energy
Shkbp1	0.947	http://api.brain-map.org/grid_data/download/68798232?include=energy
Cct4	0.947	http://api.brain-map.org/grid_data/download/71380971?include=energy
Dnlz	0.947	http://api.brain-map.org/grid_data/download/69170168?include=energy
Tmem161a	0.947	http://api.brain-map.org/grid_data/download/69080054?include=energy
Far1	0.947	http://api.brain-map.org/grid_data/download/74990480?include=energy
Hist1h4i	0.947	http://api.brain-map.org/grid_data/download/69169564?include=energy
Sorcs2	0.947	http://api.brain-map.org/grid_data/download/74047717?include=energy
Hmgb4	0.947	http://api.brain-map.org/grid_data/download/75861016?include=energy
9230110F15Rik	0.947	http://api.brain-map.org/grid_data/download/71892412?include=energy
Vezf1	0.947	http://api.brain-map.org/grid_data/download/75931470?include=energy
Pkig	0.947	http://api.brain-map.org/grid_data/download/2483?include=energy
Wdr11	0.947	http://api.brain-map.org/grid_data/download/75990608?include=energy
Dyrk1b	0.947	http://api.brain-map.org/grid_data/download/71717583?include=energy
Socs4	0.947	http://api.brain-map.org/grid_data/download/69885648?include=energy
Ehbp1	0.947	http://api.brain-map.org/grid_data/download/70239099?include=energy
LOC545466	0.947	http://api.brain-map.org/grid_data/download/75043641?include=energy
Mettl8	0.947	http://api.brain-map.org/grid_data/download/393256?include=energy
Zfp324	0.947	http://api.brain-map.org/grid_data/download/68798807?include=energy
C2cd4c	0.947	http://api.brain-map.org/grid_data/download/71493687?include=energy
Hhex	0.947	http://api.brain-map.org/grid_data/download/70431355?include=energy
Ripk4	0.947	http://api.brain-map.org/grid_data/download/77455011?include=energy
1810035L17Rik	0.947	http://api.brain-map.org/grid_data/download/75198184?include=energy
Atp6v0a1	0.947	http://api.brain-map.org/grid_data/download/126?include=energy
Gm6377	0.947	http://api.brain-map.org/grid_data/download/73906304?include=energy
Mtap2	0.947	http://api.brain-map.org/grid_data/download/74272037?include=energy
Zfp12	0.947	http://api.brain-map.org/grid_data/download/68845197?include=energy
Aimp1	0.947	http://api.brain-map.org/grid_data/download/73573163?include=energy
Cd209c	0.947	http://api.brain-map.org/grid_data/download/71021133?include=energy
Irak1	0.947	http://api.brain-map.org/grid_data/download/2323?include=energy
1700029P11Rik	0.947	http://api.brain-map.org/grid_data/download/70201397?include=energy
Gps1	0.947	http://api.brain-map.org/grid_data/download/67978711?include=energy
S100a13	0.947	http://api.brain-map.org/grid_data/download/71735833?include=energy
Mis12	0.947	http://api.brain-map.org/grid_data/download/68498355?include=energy
AI427809	0.947	http://api.brain-map.org/grid_data/download/74430948?include=energy
Tmem150b	0.947	http://api.brain-map.org/grid_data/download/70528318?include=energy
Bub3	0.947	http://api.brain-map.org/grid_data/download/68522380?include=energy
Fam160b1	0.947	http://api.brain-map.org/grid_data/download/69169959?include=energy
Lpxn	0.947	http://api.brain-map.org/grid_data/download/69261407?include=energy
Chchd2	0.947	http://api.brain-map.org/grid_data/download/74272036?include=energy
Klk12	0.946	http://api.brain-map.org/grid_data/download/68798310?include=energy
4930452B06Rik	0.946	http://api.brain-map.org/grid_data/download/75831714?include=energy
TC1498347	0.946	http://api.brain-map.org/grid_data/download/70812984?include=energy
Agap3	0.946	http://api.brain-map.org/grid_data/download/2089?include=energy
Gcnt3	0.946	http://api.brain-map.org/grid_data/download/71617022?include=energy
Ccdc91	0.946	http://api.brain-map.org/grid_data/download/74818623?include=energy
Creb3	0.946	http://api.brain-map.org/grid_data/download/69080032?include=energy
Rassf8	0.946	http://api.brain-map.org/grid_data/download/71249739?include=energy
Sec14l3	0.946	http://api.brain-map.org/grid_data/download/70595931?include=energy
Adh5	0.946	http://api.brain-map.org/grid_data/download/75988241?include=energy
Tor1aip1	0.946	http://api.brain-map.org/grid_data/download/512990?include=energy
Gm1568	0.946	http://api.brain-map.org/grid_data/download/73497575?include=energy
Vwa5a	0.946	http://api.brain-map.org/grid_data/download/71836830?include=energy
Ywhaq	0.946	http://api.brain-map.org/grid_data/download/1130?include=energy
Gja10	0.946	http://api.brain-map.org/grid_data/download/69672886?include=energy
Hmbs	0.946	http://api.brain-map.org/grid_data/download/75990578?include=energy
Krt222	0.946	http://api.brain-map.org/grid_data/download/71249742?include=energy
1700061J05Rik	0.946	http://api.brain-map.org/grid_data/download/72081689?include=energy
2210010C17Rik	0.946	http://api.brain-map.org/grid_data/download/71213060?include=energy
Slc10a2	0.946	http://api.brain-map.org/grid_data/download/78534548?include=energy
BC030336	0.946	http://api.brain-map.org/grid_data/download/678689?include=energy
Vegfa	0.946	http://api.brain-map.org/grid_data/download/74988747?include=energy
6330408A02Rik	0.946	http://api.brain-map.org/grid_data/download/71380526?include=energy
Olfr1368	0.946	http://api.brain-map.org/grid_data/download/74720787?include=energy
Vps11	0.946	http://api.brain-map.org/grid_data/download/68148721?include=energy
Ipo9	0.946	http://api.brain-map.org/grid_data/download/70278081?include=energy
Zfp366	0.946	http://api.brain-map.org/grid_data/download/70200782?include=energy
Nop10	0.946	http://api.brain-map.org/grid_data/download/75042268?include=energy

Alg5	0.946	http://api.brain-map.org/grid_data/download/75774105?include=energy
Mm.247158	0.946	http://api.brain-map.org/grid_data/download/71456145?include=energy
Sprr2g	0.946	http://api.brain-map.org/grid_data/download/72472379?include=energy
Katnal1	0.946	http://api.brain-map.org/grid_data/download/386005?include=energy
Rxra	0.946	http://api.brain-map.org/grid_data/download/100145350?include=energy
St7	0.946	http://api.brain-map.org/grid_data/download/69095947?include=energy
LOC433005	0.946	http://api.brain-map.org/grid_data/download/71283352?include=energy
Olfr557	0.946	http://api.brain-map.org/grid_data/download/74724187?include=energy
Snx18	0.946	http://api.brain-map.org/grid_data/download/76081036?include=energy
Cabp4	0.946	http://api.brain-map.org/grid_data/download/70523800?include=energy
Usp30	0.946	http://api.brain-map.org/grid_data/download/70194642?include=energy
Enam	0.946	http://api.brain-map.org/grid_data/download/77799003?include=energy
Gm14920	0.946	http://api.brain-map.org/grid_data/download/74821864?include=energy
Mafk	0.946	http://api.brain-map.org/grid_data/download/79544896?include=energy
Spaca7	0.946	http://api.brain-map.org/grid_data/download/76135583?include=energy
F830212C03Rik*	0.946	http://api.brain-map.org/grid_data/download/73731448?include=energy
Tapbpl	0.946	http://api.brain-map.org/grid_data/download/68342402?include=energy
Ndufa7	0.946	http://api.brain-map.org/grid_data/download/69838286?include=energy
Tssk1	0.946	http://api.brain-map.org/grid_data/download/70565288?include=energy
Fam168b	0.946	http://api.brain-map.org/grid_data/download/69529119?include=energy
Mgat1	0.946	http://api.brain-map.org/grid_data/download/68300968?include=energy
Klhl9	0.946	http://api.brain-map.org/grid_data/download/74988762?include=energy
1110007C09Rik	0.946	http://api.brain-map.org/grid_data/download/74357561?include=energy
Gm1690	0.946	http://api.brain-map.org/grid_data/download/70596119?include=energy
Pcdhb19	0.946	http://api.brain-map.org/grid_data/download/74743270?include=energy
Sc5d	0.946	http://api.brain-map.org/grid_data/download/227160?include=energy
Dync2h1	0.946	http://api.brain-map.org/grid_data/download/69627009?include=energy
Crygn	0.946	http://api.brain-map.org/grid_data/download/70238010?include=energy
E330032C10Rik	0.946	http://api.brain-map.org/grid_data/download/70565539?include=energy
Skint10	0.945	http://api.brain-map.org/grid_data/download/71765010?include=energy
Dpep1	0.945	http://api.brain-map.org/grid_data/download/67979038?include=energy
Akr1cl	0.945	http://api.brain-map.org/grid_data/download/75850418?include=energy
Thada	0.945	http://api.brain-map.org/grid_data/download/74510302?include=energy
Emid2	0.945	http://api.brain-map.org/grid_data/download/69134511?include=energy
Ubp1	0.945	http://api.brain-map.org/grid_data/download/69290704?include=energy
Rnf103	0.945	http://api.brain-map.org/grid_data/download/69818212?include=energy
Smo	0.945	http://api.brain-map.org/grid_data/download/69838516?include=energy
Entpd5	0.945	http://api.brain-map.org/grid_data/download/67765698?include=energy
Isca2	0.945	http://api.brain-map.org/grid_data/download/74581032?include=energy
Shcbp1	0.945	http://api.brain-map.org/grid_data/download/69873738?include=energy
Accn2	0.945	http://api.brain-map.org/grid_data/download/71654154?include=energy
Herpud1	0.945	http://api.brain-map.org/grid_data/download/686221?include=energy
Pnpla1	0.945	http://api.brain-map.org/grid_data/download/70722904?include=energy
LOC434002	0.945	http://api.brain-map.org/grid_data/download/74581371?include=energy
Cno	0.945	http://api.brain-map.org/grid_data/download/70528527?include=energy
Traf5	0.945	http://api.brain-map.org/grid_data/download/2693?include=energy
Tmem176a	0.945	http://api.brain-map.org/grid_data/download/75650863?include=energy
Anapc16	0.945	http://api.brain-map.org/grid_data/download/293463?include=energy
Lhx9	0.945	http://api.brain-map.org/grid_data/download/112646029?include=energy
Hsd17b1	0.945	http://api.brain-map.org/grid_data/download/69735120?include=energy
Commd3	0.945	http://api.brain-map.org/grid_data/download/69262340?include=energy
Myef2	0.945	http://api.brain-map.org/grid_data/download/71022840?include=energy
Rac3	0.945	http://api.brain-map.org/grid_data/download/70785006?include=energy
Slc9a6	0.945	http://api.brain-map.org/grid_data/download/76115744?include=energy
4930549D04Rik*	0.945	http://api.brain-map.org/grid_data/download/73906509?include=energy
2500003M10Rik	0.945	http://api.brain-map.org/grid_data/download/69134556?include=energy
Lrp3	0.945	http://api.brain-map.org/grid_data/download/71022633?include=energy
Egfl8	0.945	http://api.brain-map.org/grid_data/download/69671923?include=energy
AW557046	0.945	http://api.brain-map.org/grid_data/download/74427122?include=energy
Trim35	0.945	http://api.brain-map.org/grid_data/download/70192057?include=energy
Hnrnpab	0.945	http://api.brain-map.org/grid_data/download/73716596?include=energy
Dnm1	0.945	http://api.brain-map.org/grid_data/download/47983?include=energy
Aamp	0.945	http://api.brain-map.org/grid_data/download/67792196?include=energy
Urgcp	0.945	http://api.brain-map.org/grid_data/download/70203417?include=energy
Col9a2	0.945	http://api.brain-map.org/grid_data/download/74750022?include=energy
Glyat	0.945	http://api.brain-map.org/grid_data/download/77932821?include=energy
Otud6a	0.945	http://api.brain-map.org/grid_data/download/71489808?include=energy
Fbxo18	0.945	http://api.brain-map.org/grid_data/download/293462?include=energy
Me1	0.945	http://api.brain-map.org/grid_data/download/75695655?include=energy
Aph1a	0.945	http://api.brain-map.org/grid_data/download/1990?include=energy
Pik3c3	0.945	http://api.brain-map.org/grid_data/download/68498253?include=energy
Clasrp	0.945	http://api.brain-map.org/grid_data/download/73716555?include=energy
Hk3	0.945	http://api.brain-map.org/grid_data/download/74578305?include=energy
Zfp385b	0.945	http://api.brain-map.org/grid_data/download/72128752?include=energy
Gabrg1	0.945	http://api.brain-map.org/grid_data/download/75551469?include=energy
Mmp27	0.945	http://api.brain-map.org/grid_data/download/74431020?include=energy
Has3	0.945	http://api.brain-map.org/grid_data/download/71529911?include=energy
Gpm6b	0.945	http://api.brain-map.org/grid_data/download/544520?include=energy
Mmp13	0.945	http://api.brain-map.org/grid_data/download/70946571?include=energy
Tln1	0.945	http://api.brain-map.org/grid_data/download/71489782?include=energy
Tmem68	0.945	http://api.brain-map.org/grid_data/download/583558?include=energy
Rg9mtd3	0.945	http://api.brain-map.org/grid_data/download/69549281?include=energy
LOC433740	0.945	http://api.brain-map.org/grid_data/download/77340490?include=energy
Dusp7	0.945	http://api.brain-map.org/grid_data/download/69549657?include=energy
Sh3glb2	0.945	http://api.brain-map.org/grid_data/download/69549263?include=energy
Prl6a1	0.945	http://api.brain-map.org/grid_data/download/69817354?include=energy
1700128F08Rik	0.945	http://api.brain-map.org/grid_data/download/71920243?include=energy
Zfp74	0.945	http://api.brain-map.org/grid_data/download/75991080?include=energy
Alx4	0.945	http://api.brain-map.org/grid_data/download/77454703?include=energy
Adora2b	0.945	http://api.brain-map.org/grid_data/download/69855773?include=energy
Gm801	0.945	http://api.brain-map.org/grid_data/download/70298286?include=energy
Sap130	0.945	http://api.brain-map.org/grid_data/download/68844253?include=energy
Gga3	0.945	http://api.brain-map.org/grid_data/download/71529827?include=energy
Usp26	0.945	http://api.brain-map.org/grid_data/download/70302932?include=energy
Il20rb	0.945	http://api.brain-map.org/grid_data/download/76003625?include=energy
Tmco6	0.945	http://api.brain-map.org/grid_data/download/70542876?include=energy
Zfp473	0.945	http://api.brain-map.org/grid_data/download/71377741?include=energy
Aurkb	0.945	http://api.brain-map.org/grid_data/download/633668?include=energy
Hmg20b	0.945	http://api.brain-map.org/grid_data/download/68301819?include=energy
Lnpep	0.945	http://api.brain-map.org/grid_data/download/77465066?include=energy
Itsn1	0.945	http://api.brain-map.org/grid_data/download/1365?include=energy
Lrig1	0.945	http://api.brain-map.org/grid_data/download/1371?include=energy
Arid4b	0.944	http://api.brain-map.org/grid_data/download/69169905?include=energy
Creld2	0.944	http://api.brain-map.org/grid_data/download/68920878?include=energy
Pkia	0.944	http://api.brain-map.org/grid_data/download/806?include=energy
Gm12680	0.944	http://api.brain-map.org/grid_data/download/74047747?include=energy
Inmt	0.944	http://api.brain-map.org/grid_data/download/68341244?include=energy
Gapvd1	0.944	http://api.brain-map.org/grid_data/download/69407582?include=energy
Cyp4a28-ps	0.944	http://api.brain-map.org/grid_data/download/70238046?include=energy
Iqsec3	0.944	http://api.brain-map.org/grid_data/download/73636153?include=energy
Lonp1	0.944	http://api.brain-map.org/grid_data/download/69817378?include=energy
Muc16	0.944	http://api.brain-map.org/grid_data/download/70200860?include=energy
Nfrkb	0.944	http://api.brain-map.org/grid_data/download/75831740?include=energy
Elf4	0.944	http://api.brain-map.org/grid_data/download/77464840?include=energy
Dlst	0.944	http://api.brain-map.org/grid_data/download/321200?include=energy
Speer1-ps1	0.944	http://api.brain-map.org/grid_data/download/69114614?include=energy
Ccdc65	0.944	http://api.brain-map.org/grid_data/download/74724776?include=energy
Ugt2a2	0.944	http://api.brain-map.org/grid_data/download/74426861?include=energy
Brca2	0.944	http://api.brain-map.org/grid_data/download/75988323?include=energy
Snapc5	0.944	http://api.brain-map.org/grid_data/download/77910785?include=energy
Krt81	0.944	http://api.brain-map.org/grid_data/download/70724906?include=energy
Olfr615	0.944	http://api.brain-map.org/grid_data/download/74734740?include=energy
Nt5dc2	0.944	http://api.brain-map.org/grid_data/download/73817984?include=energy
Glb1	0.944	http://api.brain-map.org/grid_data/download/69015703?include=energy
Psma7	0.944	http://api.brain-map.org/grid_data/download/75198064?include=energy
Ptpn7	0.944	http://api.brain-map.org/grid_data/download/71021051?include=energy
Grin3a	0.944	http://api.brain-map.org/grid_data/download/73907499?include=energy
Cep250	0.944	http://api.brain-map.org/grid_data/download/69548846?include=energy
Herc2	0.944	http://api.brain-map.org/grid_data/download/69734983?include=energy
Mt3	0.944	http://api.brain-map.org/grid_data/download/75079775?include=energy
Mapk1ip1l	0.944	http://api.brain-map.org/grid_data/download/71809133?include=energy
Vav1	0.944	http://api.brain-map.org/grid_data/download/68300866?include=energy
Cypt12	0.944	http://api.brain-map.org/grid_data/download/74427098?include=energy
Tsga10	0.944	http://api.brain-map.org/grid_data/download/77620465?include=energy
Eif4e2	0.944	http://api.brain-map.org/grid_data/download/69012855?include=energy
AW551984	0.944	http://api.brain-map.org/grid_data/download/71764065?include=energy
4931423N10Rik	0.944	http://api.brain-map.org/grid_data/download/71283541?include=energy
C230024C17	0.944	http://api.brain-map.org/grid_data/download/71574582?include=energy
Gsr	0.944	http://api.brain-map.org/grid_data/download/69734797?include=energy
Ppp1r9a	0.944	http://api.brain-map.org/grid_data/download/71064176?include=energy
Alkbh3	0.944	http://api.brain-map.org/grid_data/download/68633467?include=energy
Creg1	0.944	http://api.brain-map.org/grid_data/download/73931627?include=energy
Vps29	0.944	http://api.brain-map.org/grid_data/download/76097708?include=energy
Hcfc1r1	0.944	http://api.brain-map.org/grid_data/download/71529917?include=energy
Nmnat2	0.944	http://api.brain-map.org/grid_data/download/69782282?include=energy
Phf7	0.944	http://api.brain-map.org/grid_data/download/69028515?include=energy
Ppp1r16b	0.944	http://api.brain-map.org/grid_data/download/69289733?include=energy
6430548M08Rik	0.944	http://api.brain-map.org/grid_data/download/72008118?include=energy
D15Ertd621e	0.944	http://api.brain-map.org/grid_data/download/71723906?include=energy
Ppp1r12a	0.944	http://api.brain-map.org/grid_data/download/81600604?include=energy
Top3a	0.944	http://api.brain-map.org/grid_data/download/70191955?include=energy
LOC433701	0.944	http://api.brain-map.org/grid_data/download/70786688?include=energy
Ppp2r5b	0.944	http://api.brain-map.org/grid_data/download/74819327?include=energy
LOC434006	0.944	http://api.brain-map.org/grid_data/download/70787215?include=energy
Rnf135	0.944	http://api.brain-map.org/grid_data/download/71493032?include=energy
5730577I03Rik	0.944	http://api.brain-map.org/grid_data/download/71283553?include=energy
Scand1	0.944	http://api.brain-map.org/grid_data/download/2549?include=energy
Pde8a	0.944	http://api.brain-map.org/grid_data/download/69782939?include=energy
Gpr64	0.944	http://api.brain-map.org/grid_data/download/69257612?include=energy
Amdhd1	0.944	http://api.brain-map.org/grid_data/download/70598392?include=energy
Itgal	0.944	http://api.brain-map.org/grid_data/download/69735598?include=energy
Ubxn8	0.944	http://api.brain-map.org/grid_data/download/69173364?include=energy
Yaf2	0.944	http://api.brain-map.org/grid_data/download/70445305?include=energy
Gpr171	0.944	http://api.brain-map.org/grid_data/download/72007564?include=energy
F930048A06Rik*	0.944	http://api.brain-map.org/grid_data/download/73767424?include=energy
LOC213014	0.944	http://api.brain-map.org/grid_data/download/71147747?include=energy
Atpaf1	0.944	http://api.brain-map.org/grid_data/download/77280419?include=energy
Txn2	0.944	http://api.brain-map.org/grid_data/download/71670705?include=energy
Gstm2	0.944	http://api.brain-map.org/grid_data/download/74427062?include=energy
D730042O14Rik*	0.944	http://api.brain-map.org/grid_data/download/73906448?include=energy
C8b	0.943	http://api.brain-map.org/grid_data/download/71577962?include=energy
Amigo3	0.943	http://api.brain-map.org/grid_data/download/71281337?include=energy
Sh3bp4	0.943	http://api.brain-map.org/grid_data/download/75774110?include=energy
Mrps22	0.943	http://api.brain-map.org/grid_data/download/68743996?include=energy
Hgfac	0.943	http://api.brain-map.org/grid_data/download/68410411?include=energy
Thbd	0.943	http://api.brain-map.org/grid_data/download/75079800?include=energy
2310001A20Rik	0.943	http://api.brain-map.org/grid_data/download/69837580?include=energy
Scnn1a	0.943	http://api.brain-map.org/grid_data/download/70562125?include=energy
Gnrh1	0.943	http://api.brain-map.org/grid_data/download/75080152?include=energy
Pck2	0.943	http://api.brain-map.org/grid_data/download/71836804?include=energy
Cdyl	0.943	http://api.brain-map.org/grid_data/download/69113722?include=energy
Eif2s3y	0.943	http://api.brain-map.org/grid_data/download/69549624?include=energy
Zfp689	0.943	http://api.brain-map.org/grid_data/download/77932789?include=energy
Atxn7l3	0.943	http://api.brain-map.org/grid_data/download/71764425?include=energy
Fbxo40	0.943	http://api.brain-map.org/grid_data/download/71232661?include=energy
Arpc2	0.943	http://api.brain-map.org/grid_data/download/69526892?include=energy
Sfxn5	0.943	http://api.brain-map.org/grid_data/download/70302051?include=energy
Med17	0.943	http://api.brain-map.org/grid_data/download/583553?include=energy
Inpp5d	0.943	http://api.brain-map.org/grid_data/download/69735461?include=energy
Smchd1	0.943	http://api.brain-map.org/grid_data/download/69442428?include=energy
Olfr1179	0.943	http://api.brain-map.org/grid_data/download/74720627?include=energy
Sds	0.943	http://api.brain-map.org/grid_data/download/68302190?include=energy
March8	0.943	http://api.brain-map.org/grid_data/download/68798956?include=energy
Phkb	0.943	http://api.brain-map.org/grid_data/download/68843763?include=energy
Iws1	0.943	http://api.brain-map.org/grid_data/download/69353774?include=energy
Hspa2	0.943	http://api.brain-map.org/grid_data/download/532642?include=energy
Sox2	0.943	http://api.brain-map.org/grid_data/download/77280331?include=energy
1700086D15Rik	0.943	http://api.brain-map.org/grid_data/download/74357947?include=energy
Gm12169	0.943	http://api.brain-map.org/grid_data/download/70565909?include=energy
Tmem33	0.943	http://api.brain-map.org/grid_data/download/356284?include=energy
Prdx6b	0.943	http://api.brain-map.org/grid_data/download/74988306?include=energy
Dnajc21	0.943	http://api.brain-map.org/grid_data/download/69288572?include=energy
Lrp6	0.943	http://api.brain-map.org/grid_data/download/77454745?include=energy
Zfp217	0.943	http://api.brain-map.org/grid_data/download/70200770?include=energy
9330158H04Rik	0.943	http://api.brain-map.org/grid_data/download/308056174?include=energy
Itih4	0.943	http://api.brain-map.org/grid_data/download/69549297?include=energy
Reg1	0.943	http://api.brain-map.org/grid_data/download/68523144?include=energy
Ndufa1	0.943	http://api.brain-map.org/grid_data/download/71763492?include=energy
Gm1720	0.943	http://api.brain-map.org/grid_data/download/71764455?include=energy
Nae1	0.943	http://api.brain-map.org/grid_data/download/76098392?include=energy
Lrriq4	0.943	http://api.brain-map.org/grid_data/download/74749178?include=energy
Hmbox1	0.943	http://api.brain-map.org/grid_data/download/70301273?include=energy
Gin1	0.943	http://api.brain-map.org/grid_data/download/67792182?include=energy
Gabrg2	0.943	http://api.brain-map.org/grid_data/download/474?include=energy
Epha6	0.943	http://api.brain-map.org/grid_data/download/72472793?include=energy
Pdk3	0.943	http://api.brain-map.org/grid_data/download/2474?include=energy
Zc3h12d	0.943	http://api.brain-map.org/grid_data/download/75931576?include=energy
Wbscr17	0.943	http://api.brain-map.org/grid_data/download/1934?include=energy
Slc47a1	0.943	http://api.brain-map.org/grid_data/download/68445012?include=energy
6230427J02Rik	0.943	http://api.brain-map.org/grid_data/download/74635695?include=energy
Gas5	0.943	http://api.brain-map.org/grid_data/download/483?include=energy
Pah	0.943	http://api.brain-map.org/grid_data/download/68321674?include=energy
Arhgap29	0.943	http://api.brain-map.org/grid_data/download/77800911?include=energy
Il2ra	0.943	http://api.brain-map.org/grid_data/download/74513977?include=energy
2610204G22Rik	0.943	http://api.brain-map.org/grid_data/download/75934922?include=energy
Bre	0.943	http://api.brain-map.org/grid_data/download/74359670?include=energy
Syvn1	0.943	http://api.brain-map.org/grid_data/download/68845771?include=energy
Cd151	0.943	http://api.brain-map.org/grid_data/download/74821967?include=energy
Slc30a7	0.943	http://api.brain-map.org/grid_data/download/78534508?include=energy
Psen1	0.943	http://api.brain-map.org/grid_data/download/79760446?include=energy
LOC433764	0.943	http://api.brain-map.org/grid_data/download/70786773?include=energy
Ankrd33	0.943	http://api.brain-map.org/grid_data/download/2006?include=energy
Pcolce	0.943	http://api.brain-map.org/grid_data/download/69782867?include=energy
Ppp1r16a	0.943	http://api.brain-map.org/grid_data/download/77932807?include=energy
Tlcd2	0.943	http://api.brain-map.org/grid_data/download/72081882?include=energy
St13	0.943	http://api.brain-map.org/grid_data/download/532638?include=energy
Nin	0.943	http://api.brain-map.org/grid_data/download/76115736?include=energy
Tfb1m	0.943	http://api.brain-map.org/grid_data/download/68744558?include=energy
Ilf3	0.942	http://api.brain-map.org/grid_data/download/74425549?include=energy
Aida	0.942	http://api.brain-map.org/grid_data/download/69095963?include=energy
Twistnb	0.942	http://api.brain-map.org/grid_data/download/70302878?include=energy
BC030863	0.942	http://api.brain-map.org/grid_data/download/71574522?include=energy
2810004N23Rik	0.942	http://api.brain-map.org/grid_data/download/68795406?include=energy
1700021O21Rik	0.942	http://api.brain-map.org/grid_data/download/74357857?include=energy
Zhx1	0.942	http://api.brain-map.org/grid_data/download/68843775?include=energy
2810407C02Rik	0.942	http://api.brain-map.org/grid_data/download/68545935?include=energy
Kcnmb3	0.942	http://api.brain-map.org/grid_data/download/81600586?include=energy
Kcna5	0.942	http://api.brain-map.org/grid_data/download/75695671?include=energy
Ptpn14	0.942	http://api.brain-map.org/grid_data/download/73927769?include=energy
Snx33	0.942	http://api.brain-map.org/grid_data/download/74882525?include=energy
Zfp64	0.942	http://api.brain-map.org/grid_data/download/75695666?include=energy
Prpf8	0.942	http://api.brain-map.org/grid_data/download/68667270?include=energy
Map3k13	0.942	http://api.brain-map.org/grid_data/download/70200908?include=energy
4930448I06Rik	0.942	http://api.brain-map.org/grid_data/download/74580014?include=energy
Yif1a	0.942	http://api.brain-map.org/grid_data/download/71616832?include=energy
Bscl2	0.942	http://api.brain-map.org/grid_data/download/68798085?include=energy
Gm896	0.942	http://api.brain-map.org/grid_data/download/74359652?include=energy
G6pc2	0.942	http://api.brain-map.org/grid_data/download/69672683?include=energy
LOC620538	0.942	http://api.brain-map.org/grid_data/download/73907504?include=energy
Pcdhb22	0.942	http://api.brain-map.org/grid_data/download/69782832?include=energy
Acadm	0.942	http://api.brain-map.org/grid_data/download/71627813?include=energy
Tpmt	0.942	http://api.brain-map.org/grid_data/download/76098337?include=energy
Pnpla2	0.942	http://api.brain-map.org/grid_data/download/68743841?include=energy
Zfp111	0.942	http://api.brain-map.org/grid_data/download/80513992?include=energy
Rassf5	0.942	http://api.brain-map.org/grid_data/download/69818018?include=energy
Fbxl20	0.942	http://api.brain-map.org/grid_data/download/69080010?include=energy
LOC434131	0.942	http://api.brain-map.org/grid_data/download/70787444?include=energy

Adam30	0.942	http://api.brain-map.org/grid_data/download/76080968?include=energy
Uba2	0.942	http://api.brain-map.org/grid_data/download/69817061?include=energy
Hmx3	0.942	http://api.brain-map.org/grid_data/download/74641309?include=energy
Slc12a5	0.942	http://api.brain-map.org/grid_data/download/72001898?include=energy
Spem1	0.942	http://api.brain-map.org/grid_data/download/71920183?include=energy
Mospd4	0.942	http://api.brain-map.org/grid_data/download/74635280?include=energy
Gm3579	0.942	http://api.brain-map.org/grid_data/download/74743477?include=energy
Cdca4	0.942	http://api.brain-map.org/grid_data/download/74635493?include=energy
Gm154	0.942	http://api.brain-map.org/grid_data/download/76081018?include=energy
Sun5	0.942	http://api.brain-map.org/grid_data/download/75934509?include=energy
Grem1	0.942	http://api.brain-map.org/grid_data/download/69059946?include=energy
Cadm3	0.942	http://api.brain-map.org/grid_data/download/69169911?include=energy
8430410K20Rik	0.942	http://api.brain-map.org/grid_data/download/70524280?include=energy
Mfn2	0.942	http://api.brain-map.org/grid_data/download/74882657?include=energy
Galnt13	0.942	http://api.brain-map.org/grid_data/download/72472803?include=energy
Pde4b	0.942	http://api.brain-map.org/grid_data/download/2472?include=energy
Gtf3c5	0.942	http://api.brain-map.org/grid_data/download/67767471?include=energy
Ift172	0.942	http://api.brain-map.org/grid_data/download/71280856?include=energy
Rragd	0.942	http://api.brain-map.org/grid_data/download/73929582?include=energy
Klhl18	0.942	http://api.brain-map.org/grid_data/download/69095975?include=energy
TC1413613	0.942	http://api.brain-map.org/grid_data/download/70794013?include=energy
Has2	0.942	http://api.brain-map.org/grid_data/download/74277703?include=energy
Klhl4	0.942	http://api.brain-map.org/grid_data/download/76115731?include=energy
P2rx6	0.942	http://api.brain-map.org/grid_data/download/75551463?include=energy
Arpc4	0.942	http://api.brain-map.org/grid_data/download/69013768?include=energy
Rsph6a	0.942	http://api.brain-map.org/grid_data/download/71808828?include=energy
1110008L16Rik	0.942	http://api.brain-map.org/grid_data/download/74581068?include=energy
Tox3	0.942	http://api.brain-map.org/grid_data/download/75077172?include=energy
Phlpp2	0.942	http://api.brain-map.org/grid_data/download/69530810?include=energy
Spink12	0.942	http://api.brain-map.org/grid_data/download/100144862?include=energy
LOC433176	0.942	http://api.brain-map.org/grid_data/download/70722978?include=energy
Tpd52l2	0.942	http://api.brain-map.org/grid_data/download/413802?include=energy
Tysnd1	0.942	http://api.brain-map.org/grid_data/download/74581157?include=energy
Clpp	0.941	http://api.brain-map.org/grid_data/download/513235?include=energy
Ascl5-ps	0.941	http://api.brain-map.org/grid_data/download/74988285?include=energy
Cyct	0.941	http://api.brain-map.org/grid_data/download/75860968?include=energy
Heg1	0.941	http://api.brain-map.org/grid_data/download/74958616?include=energy
1700027I07Rik*	0.941	http://api.brain-map.org/grid_data/download/73716638?include=energy
Ppp1r15a	0.941	http://api.brain-map.org/grid_data/download/71789861?include=energy
Bmp8a	0.941	http://api.brain-map.org/grid_data/download/74431083?include=energy
Sh3bp1	0.941	http://api.brain-map.org/grid_data/download/71611753?include=energy
Srgn	0.941	http://api.brain-map.org/grid_data/download/68191294?include=energy
Tnrc6b	0.941	http://api.brain-map.org/grid_data/download/77620491?include=energy
Fam86	0.941	http://api.brain-map.org/grid_data/download/74357587?include=energy
Irf9	0.941	http://api.brain-map.org/grid_data/download/2329?include=energy
Ptov1	0.941	http://api.brain-map.org/grid_data/download/74581086?include=energy
Alms1	0.941	http://api.brain-map.org/grid_data/download/76081919?include=energy
mCG121643	0.941	http://api.brain-map.org/grid_data/download/71147852?include=energy
Ica1	0.941	http://api.brain-map.org/grid_data/download/75988482?include=energy
2810422J05Rik	0.941	http://api.brain-map.org/grid_data/download/69013143?include=energy
Trpc1	0.941	http://api.brain-map.org/grid_data/download/69288387?include=energy
Pnpla7	0.941	http://api.brain-map.org/grid_data/download/69134232?include=energy
Pam	0.941	http://api.brain-map.org/grid_data/download/73997147?include=energy
Wbscr22	0.941	http://api.brain-map.org/grid_data/download/70194964?include=energy
Vmn1r236	0.941	http://api.brain-map.org/grid_data/download/70785636?include=energy
Tmem146	0.941	http://api.brain-map.org/grid_data/download/71493699?include=energy
Itgb6	0.941	http://api.brain-map.org/grid_data/download/70546018?include=energy
Scn1b	0.941	http://api.brain-map.org/grid_data/download/77454673?include=energy
Tspan15	0.941	http://api.brain-map.org/grid_data/download/70612725?include=energy
TC1513744	0.941	http://api.brain-map.org/grid_data/download/70813101?include=energy
Pygm	0.941	http://api.brain-map.org/grid_data/download/75198214?include=energy
Tmem102	0.941	http://api.brain-map.org/grid_data/download/69407600?include=energy
Fcer2a	0.941	http://api.brain-map.org/grid_data/download/75988414?include=energy
Hck	0.941	http://api.brain-map.org/grid_data/download/68301676?include=energy
Snai2	0.941	http://api.brain-map.org/grid_data/download/100146165?include=energy
Star	0.941	http://api.brain-map.org/grid_data/download/69887325?include=energy
Aire	0.941	http://api.brain-map.org/grid_data/download/1327?include=energy
Celf4	0.941	http://api.brain-map.org/grid_data/download/73592527?include=energy
Slc22a21	0.941	http://api.brain-map.org/grid_data/download/71832800?include=energy
2210407C18Rik	0.941	http://api.brain-map.org/grid_data/download/74990506?include=energy
BC021785	0.941	http://api.brain-map.org/grid_data/download/71808643?include=energy
Actr2	0.941	http://api.brain-map.org/grid_data/download/67?include=energy
Etv6	0.941	http://api.brain-map.org/grid_data/download/1353?include=energy
Slc12a7	0.941	http://api.brain-map.org/grid_data/download/69873803?include=energy
D11Wsu99e	0.941	http://api.brain-map.org/grid_data/download/69236604?include=energy
Tssk3	0.941	http://api.brain-map.org/grid_data/download/74427048?include=energy
Pabpn1l	0.941	http://api.brain-map.org/grid_data/download/74990476?include=energy
Laptm4a	0.941	http://api.brain-map.org/grid_data/download/69736034?include=energy
Lrit2	0.941	http://api.brain-map.org/grid_data/download/75831758?include=energy
Prkrip1	0.941	http://api.brain-map.org/grid_data/download/68545539?include=energy
Ppp1cb	0.941	http://api.brain-map.org/grid_data/download/69817205?include=energy
Vps18	0.941	http://api.brain-map.org/grid_data/download/1325?include=energy
Gabra1	0.941	http://api.brain-map.org/grid_data/download/72340131?include=energy
Ms4a3	0.941	http://api.brain-map.org/grid_data/download/73635489?include=energy
Slco4a1	0.941	http://api.brain-map.org/grid_data/download/2625?include=energy
LOC433344	0.941	http://api.brain-map.org/grid_data/download/70723122?include=energy
Kin	0.941	http://api.brain-map.org/grid_data/download/68631905?include=energy
Vav2	0.941	http://api.brain-map.org/grid_data/download/74583149?include=energy
Maml2	0.941	http://api.brain-map.org/grid_data/download/100145318?include=energy
Hdac6	0.941	http://api.brain-map.org/grid_data/download/74819462?include=energy
Zfp318	0.941	http://api.brain-map.org/grid_data/download/70598371?include=energy
1810018F18Rik	0.941	http://api.brain-map.org/grid_data/download/70201564?include=energy
Appl2	0.941	http://api.brain-map.org/grid_data/download/72080165?include=energy
Gpr132	0.941	http://api.brain-map.org/grid_data/download/69857038?include=energy
A330043P19Rik*	0.941	http://api.brain-map.org/grid_data/download/75144634?include=energy
Mrps16	0.941	http://api.brain-map.org/grid_data/download/68446093?include=energy
Rad51ap1	0.94	http://api.brain-map.org/grid_data/download/632505?include=energy
Lass3	0.94	http://api.brain-map.org/grid_data/download/77620281?include=energy
Zfp639	0.94	http://api.brain-map.org/grid_data/download/69080494?include=energy
Cadm1	0.94	http://api.brain-map.org/grid_data/download/81654149?include=energy
LOC432961	0.94	http://api.brain-map.org/grid_data/download/71789957?include=energy
Helq	0.94	http://api.brain-map.org/grid_data/download/70565837?include=energy
Gm5433	0.94	http://api.brain-map.org/grid_data/download/74641294?include=energy
Ahr	0.94	http://api.brain-map.org/grid_data/download/71380375?include=energy
Zfp826	0.94	http://api.brain-map.org/grid_data/download/70231761?include=energy
A630001G21Rik	0.94	http://api.brain-map.org/grid_data/download/68744050?include=energy
Nudt22	0.94	http://api.brain-map.org/grid_data/download/671237?include=energy
Arhgef6	0.94	http://api.brain-map.org/grid_data/download/74881547?include=energy
Rasal3	0.94	http://api.brain-map.org/grid_data/download/69448668?include=energy
Polk	0.94	http://api.brain-map.org/grid_data/download/75077171?include=energy
Mycbp	0.94	http://api.brain-map.org/grid_data/download/70437792?include=energy
Rplp1	0.94	http://api.brain-map.org/grid_data/download/69818344?include=energy
Nudt15	0.94	http://api.brain-map.org/grid_data/download/76081002?include=energy
Srp54a	0.94	http://api.brain-map.org/grid_data/download/2629?include=energy
Ces1g	0.94	http://api.brain-map.org/grid_data/download/69059802?include=energy
Mm.24928	0.94	http://api.brain-map.org/grid_data/download/71456147?include=energy
Rsc1a1	0.94	http://api.brain-map.org/grid_data/download/68743733?include=energy
Ralb	0.94	http://api.brain-map.org/grid_data/download/74657981?include=energy
5031414D18Rik	0.94	http://api.brain-map.org/grid_data/download/68668012?include=energy
Spna1	0.94	http://api.brain-map.org/grid_data/download/69288333?include=energy
Ap2s1	0.94	http://api.brain-map.org/grid_data/download/71836889?include=energy
Akr1b7	0.94	http://api.brain-map.org/grid_data/download/69526528?include=energy
Eif2ak3	0.94	http://api.brain-map.org/grid_data/download/2181?include=energy
2310010M20Rik	0.94	http://api.brain-map.org/grid_data/download/71991744?include=energy
Nup35	0.94	http://api.brain-map.org/grid_data/download/75831745?include=energy
Zfp667	0.94	http://api.brain-map.org/grid_data/download/70608376?include=energy
Cab39	0.94	http://api.brain-map.org/grid_data/download/587417?include=energy
Slc25a26	0.94	http://api.brain-map.org/grid_data/download/119700?include=energy
Trim75	0.94	http://api.brain-map.org/grid_data/download/73617660?include=energy
Tmem246	0.94	http://api.brain-map.org/grid_data/download/75774671?include=energy
Hlx	0.94	http://api.brain-map.org/grid_data/download/68744134?include=energy
Whamm	0.94	http://api.brain-map.org/grid_data/download/70546138?include=energy
Kcna2	0.94	http://api.brain-map.org/grid_data/download/75038432?include=energy
Myo7a	0.94	http://api.brain-map.org/grid_data/download/69782019?include=energy
Eif5b	0.94	http://api.brain-map.org/grid_data/download/71764101?include=energy
Frs2	0.94	http://api.brain-map.org/grid_data/download/70545895?include=energy
Lpl	0.94	http://api.brain-map.org/grid_data/download/649?include=energy
Ttc21b	0.94	http://api.brain-map.org/grid_data/download/69202663?include=energy
Eftud2	0.94	http://api.brain-map.org/grid_data/download/68667760?include=energy
Myo1a	0.94	http://api.brain-map.org/grid_data/download/74635274?include=energy
Tmie	0.94	http://api.brain-map.org/grid_data/download/74957868?include=energy
Ubxn1	0.94	http://api.brain-map.org/grid_data/download/71992097?include=energy
Ldhd	0.94	http://api.brain-map.org/grid_data/download/74425554?include=energy
Hltf	0.94	http://api.brain-map.org/grid_data/download/69816894?include=energy
Lsm4	0.94	http://api.brain-map.org/grid_data/download/68637844?include=energy
Gm7489	0.94	http://api.brain-map.org/grid_data/download/73906331?include=energy
Itpr3	0.94	http://api.brain-map.org/grid_data/download/74047776?include=energy
2400001E08Rik	0.94	http://api.brain-map.org/grid_data/download/72283773?include=energy
Ubap2l	0.94	http://api.brain-map.org/grid_data/download/67767439?include=energy
Ifltd1	0.94	http://api.brain-map.org/grid_data/download/74425566?include=energy
Bri3bp	0.94	http://api.brain-map.org/grid_data/download/69059878?include=energy
Papss1	0.94	http://api.brain-map.org/grid_data/download/71280658?include=energy
Telo2	0.94	http://api.brain-map.org/grid_data/download/564765?include=energy
Hsd17b12	0.94	http://api.brain-map.org/grid_data/download/68162254?include=energy
Fmo4	0.939	http://api.brain-map.org/grid_data/download/71587679?include=energy
4930429B21Rik	0.939	http://api.brain-map.org/grid_data/download/69407947?include=energy
Prdx3	0.939	http://api.brain-map.org/grid_data/download/70743842?include=energy
Abca4	0.939	http://api.brain-map.org/grid_data/download/75831755?include=energy
6030466F02Rik	0.939	http://api.brain-map.org/grid_data/download/71809001?include=energy
Nsun6	0.939	http://api.brain-map.org/grid_data/download/69835596?include=energy
Nono	0.939	http://api.brain-map.org/grid_data/download/68443166?include=energy
Rnf149	0.939	http://api.brain-map.org/grid_data/download/898?include=energy
Zfp85-rs1	0.939	http://api.brain-map.org/grid_data/download/74743402?include=energy
Zfp839	0.939	http://api.brain-map.org/grid_data/download/70593145?include=energy
Agr2	0.939	http://api.brain-map.org/grid_data/download/76135655?include=energy
Slc35a1	0.939	http://api.brain-map.org/grid_data/download/2605?include=energy
Nr2f6	0.939	http://api.brain-map.org/grid_data/download/112645600?include=energy
Errfi1	0.939	http://api.brain-map.org/grid_data/download/71723814?include=energy
Lars	0.939	http://api.brain-map.org/grid_data/download/68443184?include=energy
9130004C02Rik	0.939	http://api.brain-map.org/grid_data/download/70946200?include=energy
Lonrf3	0.939	http://api.brain-map.org/grid_data/download/75041538?include=energy
Hnrnpul1	0.939	http://api.brain-map.org/grid_data/download/68410458?include=energy
Sdr9c7	0.939	http://api.brain-map.org/grid_data/download/69835625?include=energy
4732460I24	0.939	http://api.brain-map.org/grid_data/download/72004517?include=energy
Ccdc17	0.939	http://api.brain-map.org/grid_data/download/74300837?include=energy
Mrpl45	0.939	http://api.brain-map.org/grid_data/download/68341782?include=energy
Prl7b1	0.939	http://api.brain-map.org/grid_data/download/74581241?include=energy
Zmiz2	0.939	http://api.brain-map.org/grid_data/download/100143454?include=energy
Ppp3cb	0.939	http://api.brain-map.org/grid_data/download/71325360?include=energy
2010007H06Rik	0.939	http://api.brain-map.org/grid_data/download/74359347?include=energy
Dazap2	0.939	http://api.brain-map.org/grid_data/download/69080518?include=energy
Rab34	0.939	http://api.brain-map.org/grid_data/download/71022966?include=energy
Ppme1	0.939	http://api.brain-map.org/grid_data/download/70430702?include=energy
Adam24	0.939	http://api.brain-map.org/grid_data/download/70228723?include=energy
Cpxm1	0.939	http://api.brain-map.org/grid_data/download/75934517?include=energy
Cs	0.939	http://api.brain-map.org/grid_data/download/74658130?include=energy
Scarb1	0.939	http://api.brain-map.org/grid_data/download/2551?include=energy
Tmem209	0.939	http://api.brain-map.org/grid_data/download/68797834?include=energy
Runx1t1	0.939	http://api.brain-map.org/grid_data/download/71307232?include=energy
Paip1	0.939	http://api.brain-map.org/grid_data/download/70473939?include=energy
Eef1b2	0.939	http://api.brain-map.org/grid_data/download/75934526?include=energy
Becn1	0.939	http://api.brain-map.org/grid_data/download/70429172?include=energy
Mrpl23	0.939	http://api.brain-map.org/grid_data/download/69549672?include=energy
Tex18	0.939	http://api.brain-map.org/grid_data/download/70302467?include=energy
5330431K02Rik	0.939	http://api.brain-map.org/grid_data/download/69443379?include=energy
Taf7l	0.939	http://api.brain-map.org/grid_data/download/74580751?include=energy
Phldb2	0.939	http://api.brain-map.org/grid_data/download/74512017?include=energy
Traf2	0.939	http://api.brain-map.org/grid_data/download/2691?include=energy
Ccnk	0.939	http://api.brain-map.org/grid_data/download/70431223?include=energy
Adpgk	0.939	http://api.brain-map.org/grid_data/download/68193068?include=energy
Lonrf2	0.939	http://api.brain-map.org/grid_data/download/74988755?include=energy
4921530D09Rik	0.939	http://api.brain-map.org/grid_data/download/69407881?include=energy
Tmem109	0.939	http://api.brain-map.org/grid_data/download/74988461?include=energy
Emid1	0.939	http://api.brain-map.org/grid_data/download/69672007?include=energy
Psmg1	0.939	http://api.brain-map.org/grid_data/download/77454963?include=energy
Lyzl6	0.939	http://api.brain-map.org/grid_data/download/75851030?include=energy
Pbrm1	0.939	http://api.brain-map.org/grid_data/download/69838567?include=energy
Prkdc	0.939	http://api.brain-map.org/grid_data/download/74882717?include=energy
Taok1	0.939	http://api.brain-map.org/grid_data/download/74819235?include=energy
Slc26a7	0.939	http://api.brain-map.org/grid_data/download/2595?include=energy
Efemp2	0.939	http://api.brain-map.org/grid_data/download/2177?include=energy
Depdc7	0.939	http://api.brain-map.org/grid_data/download/70743883?include=energy
BC031353	0.939	http://api.brain-map.org/grid_data/download/77371767?include=energy
LOC433895	0.939	http://api.brain-map.org/grid_data/download/74510308?include=energy
Gm1855	0.939	http://api.brain-map.org/grid_data/download/70298214?include=energy
Ubl7	0.939	http://api.brain-map.org/grid_data/download/68443345?include=energy
Kctd18	0.939	http://api.brain-map.org/grid_data/download/70305114?include=energy
Reln	0.939	http://api.brain-map.org/grid_data/download/890?include=energy
2610028H24Rik	0.939	http://api.brain-map.org/grid_data/download/68797571?include=energy
Dleu7	0.939	http://api.brain-map.org/grid_data/download/69059913?include=energy
Ndufb2	0.939	http://api.brain-map.org/grid_data/download/70429309?include=energy
Fzr1	0.939	http://api.brain-map.org/grid_data/download/323178?include=energy
Cand1	0.939	http://api.brain-map.org/grid_data/download/69608420?include=energy
Flii	0.939	http://api.brain-map.org/grid_data/download/68193045?include=energy
Zc3h15	0.939	http://api.brain-map.org/grid_data/download/70439190?include=energy
D230035N22Rik	0.938	http://api.brain-map.org/grid_data/download/71147972?include=energy
E130018O15Rik	0.938	http://api.brain-map.org/grid_data/download/70565500?include=energy
Fez2	0.938	http://api.brain-map.org/grid_data/download/74272477?include=energy
Igsf3	0.938	http://api.brain-map.org/grid_data/download/72081517?include=energy
4922501L14Rik	0.938	http://api.brain-map.org/grid_data/download/71492856?include=energy
Nphs1	0.938	http://api.brain-map.org/grid_data/download/70300577?include=energy
Rrp9	0.938	http://api.brain-map.org/grid_data/download/69015799?include=energy
N4bp3	0.938	http://api.brain-map.org/grid_data/download/69838603?include=energy
Timm13	0.938	http://api.brain-map.org/grid_data/download/512769?include=energy
Rwdd3	0.938	http://api.brain-map.org/grid_data/download/74056138?include=energy
Bbs5	0.938	http://api.brain-map.org/grid_data/download/69116721?include=energy
Lrfn3	0.938	http://api.brain-map.org/grid_data/download/76081914?include=energy
Adamts2	0.938	http://api.brain-map.org/grid_data/download/71924385?include=energy
Wibg	0.938	http://api.brain-map.org/grid_data/download/69113895?include=energy
Kcnk1	0.938	http://api.brain-map.org/grid_data/download/67850952?include=energy
Rhot2	0.938	http://api.brain-map.org/grid_data/download/67815938?include=energy
Fbxo36	0.938	http://api.brain-map.org/grid_data/download/69114292?include=energy
Psmg3	0.938	http://api.brain-map.org/grid_data/download/275680?include=energy
Klhdc2	0.938	http://api.brain-map.org/grid_data/download/70429274?include=energy
Alg1	0.938	http://api.brain-map.org/grid_data/download/70616016?include=energy
Stt3a	0.938	http://api.brain-map.org/grid_data/download/69059865?include=energy
Ptk6	0.938	http://api.brain-map.org/grid_data/download/71377836?include=energy
Mrpl16	0.938	http://api.brain-map.org/grid_data/download/69012807?include=energy
Gtf2b	0.938	http://api.brain-map.org/grid_data/download/71616138?include=energy
Ttf1	0.938	http://api.brain-map.org/grid_data/download/71488883?include=energy
AW555464	0.938	http://api.brain-map.org/grid_data/download/68667377?include=energy
Terf2ip	0.938	http://api.brain-map.org/grid_data/download/112196711?include=energy
Map3k6	0.938	http://api.brain-map.org/grid_data/download/69752023?include=energy
Nme2	0.938	http://api.brain-map.org/grid_data/download/69782276?include=energy
Fam113b	0.938	http://api.brain-map.org/grid_data/download/68191228?include=energy
Ing4	0.938	http://api.brain-map.org/grid_data/download/357097?include=energy
Laptm5	0.938	http://api.brain-map.org/grid_data/download/100145372?include=energy
Smarcal1	0.938	http://api.brain-map.org/grid_data/download/68797495?include=energy
Usp22	0.938	http://api.brain-map.org/grid_data/download/74957911?include=energy
Map2k5	0.938	http://api.brain-map.org/grid_data/download/323585?include=energy
Dhx57	0.938	http://api.brain-map.org/grid_data/download/69288668?include=energy
D630028M02Rik*	0.938	http://api.brain-map.org/grid_data/download/73834439?include=energy
Cnbp	0.938	http://api.brain-map.org/grid_data/download/70593287?include=energy
Trpv1	0.938	http://api.brain-map.org/grid_data/download/80342023?include=energy
Ryr2	0.938	http://api.brain-map.org/grid_data/download/69288279?include=energy
Gm4869	0.938	http://api.brain-map.org/grid_data/download/70566059?include=energy
Slc30a6	0.938	http://api.brain-map.org/grid_data/download/69169307?include=energy

Bdkrb2	0.938	http://api.brain-map.org/grid_data/download/69236873?include=energy
Whsc1	0.938	http://api.brain-map.org/grid_data/download/69290728?include=energy
Hsfy2	0.938	http://api.brain-map.org/grid_data/download/76081891?include=energy
LOC433347	0.938	http://api.brain-map.org/grid_data/download/70723155?include=energy
Cdc6	0.938	http://api.brain-map.org/grid_data/download/69540647?include=energy
Olfr1043	0.938	http://api.brain-map.org/grid_data/download/74720579?include=energy
Akap1	0.938	http://api.brain-map.org/grid_data/download/75988245?include=energy
Crybb1	0.938	http://api.brain-map.org/grid_data/download/75774662?include=energy
Gm10440	0.938	http://api.brain-map.org/grid_data/download/70305664?include=energy
Man2a2	0.938	http://api.brain-map.org/grid_data/download/70523825?include=energy
Fads3	0.938	http://api.brain-map.org/grid_data/download/69672264?include=energy
P2rx1	0.938	http://api.brain-map.org/grid_data/download/75890835?include=energy
Tnip1	0.938	http://api.brain-map.org/grid_data/download/68443063?include=energy
1700061I17Rik	0.938	http://api.brain-map.org/grid_data/download/74635084?include=energy
Dhx9	0.938	http://api.brain-map.org/grid_data/download/69626879?include=energy
ORF63	0.938	http://api.brain-map.org/grid_data/download/1247?include=energy
Apba1	0.938	http://api.brain-map.org/grid_data/download/74658000?include=energy
Krtap6-1	0.938	http://api.brain-map.org/grid_data/download/70724935?include=energy
A930014P08Rik*	0.938	http://api.brain-map.org/grid_data/download/73808319?include=energy
Cideb	0.938	http://api.brain-map.org/grid_data/download/67978761?include=energy
Cdyl2	0.938	http://api.brain-map.org/grid_data/download/74641321?include=energy
Iffo2	0.938	http://api.brain-map.org/grid_data/download/322962?include=energy
Cd109	0.938	http://api.brain-map.org/grid_data/download/73931384?include=energy
Nomo1	0.938	http://api.brain-map.org/grid_data/download/1697?include=energy
Sgip1	0.938	http://api.brain-map.org/grid_data/download/638722?include=energy
Gpa33	0.938	http://api.brain-map.org/grid_data/download/100146136?include=energy
Atp6v1e1	0.938	http://api.brain-map.org/grid_data/download/655501?include=energy
A430035B10Rik	0.938	http://api.brain-map.org/grid_data/download/71809226?include=energy
LOC434565	0.938	http://api.brain-map.org/grid_data/download/74736267?include=energy
Gm5086	0.938	http://api.brain-map.org/grid_data/download/70305437?include=energy
9530020O07Rik	0.938	http://api.brain-map.org/grid_data/download/73808450?include=energy
Stk31	0.938	http://api.brain-map.org/grid_data/download/70302282?include=energy
Ralbp1	0.937	http://api.brain-map.org/grid_data/download/76081027?include=energy
Chrna1	0.937	http://api.brain-map.org/grid_data/download/75551465?include=energy
Eef1e1	0.937	http://api.brain-map.org/grid_data/download/69113614?include=energy
Ppip5k1	0.937	http://api.brain-map.org/grid_data/download/70231212?include=energy
Glod4	0.937	http://api.brain-map.org/grid_data/download/69116510?include=energy
Ttc35	0.937	http://api.brain-map.org/grid_data/download/81599618?include=energy
Txndc2	0.937	http://api.brain-map.org/grid_data/download/70565363?include=energy
1700021K19Rik	0.937	http://api.brain-map.org/grid_data/download/69353627?include=energy
Padi4	0.937	http://api.brain-map.org/grid_data/download/71161441?include=energy
Psmd7	0.937	http://api.brain-map.org/grid_data/download/71377824?include=energy
Prlhr	0.937	http://api.brain-map.org/grid_data/download/74511758?include=energy
Vmn2r66	0.937	http://api.brain-map.org/grid_data/download/75990864?include=energy
2810403A07Rik	0.937	http://api.brain-map.org/grid_data/download/71493087?include=energy
Csdc2	0.937	http://api.brain-map.org/grid_data/download/70634234?include=energy
Wbp2	0.937	http://api.brain-map.org/grid_data/download/69013750?include=energy
Glis2	0.937	http://api.brain-map.org/grid_data/download/68410470?include=energy
Ppil6	0.937	http://api.brain-map.org/grid_data/download/74359509?include=energy
Mllt1	0.937	http://api.brain-map.org/grid_data/download/68799077?include=energy
Snapc1	0.937	http://api.brain-map.org/grid_data/download/75990598?include=energy
Gripap1	0.937	http://api.brain-map.org/grid_data/download/74821691?include=energy
Rdh10	0.937	http://api.brain-map.org/grid_data/download/75831736?include=energy
C330006A16Rik	0.937	http://api.brain-map.org/grid_data/download/70596035?include=energy
Taf1d	0.937	http://api.brain-map.org/grid_data/download/1317?include=energy
Spata3	0.937	http://api.brain-map.org/grid_data/download/70526063?include=energy
Reck	0.937	http://api.brain-map.org/grid_data/download/69818066?include=energy
A930018M24Rik	0.937	http://api.brain-map.org/grid_data/download/70228633?include=energy
Ccl17	0.937	http://api.brain-map.org/grid_data/download/75990673?include=energy
Elmod3	0.937	http://api.brain-map.org/grid_data/download/75042257?include=energy
Nme4	0.937	http://api.brain-map.org/grid_data/download/68443519?include=energy
Gm11538	0.937	http://api.brain-map.org/grid_data/download/70611078?include=energy
Mterfd2	0.937	http://api.brain-map.org/grid_data/download/632515?include=energy
Ddx47	0.937	http://api.brain-map.org/grid_data/download/69169337?include=energy
Gpx7	0.937	http://api.brain-map.org/grid_data/download/67978722?include=energy
Hbb-b2	0.937	http://api.brain-map.org/grid_data/download/77371773?include=energy
Rgag1	0.937	http://api.brain-map.org/grid_data/download/75041526?include=energy
Arpc5l	0.937	http://api.brain-map.org/grid_data/download/70543029?include=energy
Gm1141	0.937	http://api.brain-map.org/grid_data/download/74562540?include=energy
Chrna9	0.937	http://api.brain-map.org/grid_data/download/75826568?include=energy
Ifngr2	0.937	http://api.brain-map.org/grid_data/download/1460?include=energy
Lyg1	0.937	http://api.brain-map.org/grid_data/download/73617552?include=energy
Fancm	0.937	http://api.brain-map.org/grid_data/download/73497507?include=energy
1700001C02Rik	0.937	http://api.brain-map.org/grid_data/download/69116871?include=energy
Cadps	0.937	http://api.brain-map.org/grid_data/download/72119581?include=energy
Slc30a2	0.937	http://api.brain-map.org/grid_data/download/74821946?include=energy
Vil1	0.937	http://api.brain-map.org/grid_data/download/1440?include=energy
4930485B16Rik	0.936	http://api.brain-map.org/grid_data/download/71380513?include=energy
Dynlrb1	0.936	http://api.brain-map.org/grid_data/download/323592?include=energy
Spire1	0.936	http://api.brain-map.org/grid_data/download/73573199?include=energy
Cdv3	0.936	http://api.brain-map.org/grid_data/download/70528521?include=energy
Sri	0.936	http://api.brain-map.org/grid_data/download/71991720?include=energy
LOC436114	0.936	http://api.brain-map.org/grid_data/download/71669003?include=energy
Hsf1	0.936	http://api.brain-map.org/grid_data/download/67810538?include=energy
Nanos2	0.936	http://api.brain-map.org/grid_data/download/70300511?include=energy
Ccne2	0.936	http://api.brain-map.org/grid_data/download/68667766?include=energy
Fam57b	0.936	http://api.brain-map.org/grid_data/download/75831762?include=energy
Bcas2	0.936	http://api.brain-map.org/grid_data/download/513003?include=energy
Nup88	0.936	http://api.brain-map.org/grid_data/download/68744695?include=energy
Actr1b	0.936	http://api.brain-map.org/grid_data/download/70919364?include=energy
Far2	0.936	http://api.brain-map.org/grid_data/download/71358652?include=energy
Psg17	0.936	http://api.brain-map.org/grid_data/download/75233775?include=energy
Rpn1	0.936	http://api.brain-map.org/grid_data/download/75774675?include=energy
8030463A06Rik	0.936	http://api.brain-map.org/grid_data/download/69446244?include=energy
Gstm3	0.936	http://api.brain-map.org/grid_data/download/68920779?include=energy
Atp9a	0.936	http://api.brain-map.org/grid_data/download/69838745?include=energy
Mybbp1a	0.936	http://api.brain-map.org/grid_data/download/68668102?include=energy
Fndc3a	0.936	http://api.brain-map.org/grid_data/download/69672551?include=energy
Snx25	0.936	http://api.brain-map.org/grid_data/download/73929868?include=energy
Stx1b	0.936	http://api.brain-map.org/grid_data/download/100144175?include=energy
Lin9	0.936	http://api.brain-map.org/grid_data/download/70542919?include=energy
Gpr142	0.936	http://api.brain-map.org/grid_data/download/71307370?include=energy
4930447A16Rik	0.936	http://api.brain-map.org/grid_data/download/74076234?include=energy
Itm2c	0.936	http://api.brain-map.org/grid_data/download/77414652?include=energy
Wdfy1	0.936	http://api.brain-map.org/grid_data/download/71992085?include=energy
Chrm4	0.936	http://api.brain-map.org/grid_data/download/261?include=energy
Anxa3	0.936	http://api.brain-map.org/grid_data/download/72283800?include=energy
Phyhipl	0.936	http://api.brain-map.org/grid_data/download/544326?include=energy
Chgb	0.936	http://api.brain-map.org/grid_data/download/70743886?include=energy
LOC433811	0.936	http://api.brain-map.org/grid_data/download/70786821?include=energy
Tnfrsf1a	0.936	http://api.brain-map.org/grid_data/download/2689?include=energy
3230401D17Rik	0.936	http://api.brain-map.org/grid_data/download/76097705?include=energy
Tmem106a	0.936	http://api.brain-map.org/grid_data/download/68498331?include=energy
Piga	0.936	http://api.brain-map.org/grid_data/download/71377672?include=energy
Sh2d2a	0.936	http://api.brain-map.org/grid_data/download/68744809?include=energy
Gm980	0.936	http://api.brain-map.org/grid_data/download/70298555?include=energy
Letm1	0.936	http://api.brain-map.org/grid_data/download/70918832?include=energy
Cd3g	0.936	http://api.brain-map.org/grid_data/download/68545533?include=energy
Tspan14	0.936	http://api.brain-map.org/grid_data/download/75828167?include=energy
Spopl	0.936	http://api.brain-map.org/grid_data/download/70205650?include=energy
Dnajb1	0.936	http://api.brain-map.org/grid_data/download/71836748?include=energy
Ptchd1	0.936	http://api.brain-map.org/grid_data/download/71249749?include=energy
Frmd5	0.936	http://api.brain-map.org/grid_data/download/71656873?include=energy
Kcnn1	0.936	http://api.brain-map.org/grid_data/download/69860427?include=energy
Uchl3	0.936	http://api.brain-map.org/grid_data/download/69116402?include=energy
Mapk10	0.936	http://api.brain-map.org/grid_data/download/70946134?include=energy
Dpp9	0.936	http://api.brain-map.org/grid_data/download/69014836?include=energy
Ubxn6	0.936	http://api.brain-map.org/grid_data/download/69015440?include=energy
Itih5	0.936	http://api.brain-map.org/grid_data/download/68745161?include=energy
Dgkz	0.936	http://api.brain-map.org/grid_data/download/69262326?include=energy
Nrxn1	0.936	http://api.brain-map.org/grid_data/download/70301083?include=energy
Amd2	0.936	http://api.brain-map.org/grid_data/download/70230927?include=energy
Ctsf	0.936	http://api.brain-map.org/grid_data/download/532571?include=energy
Hmox1	0.936	http://api.brain-map.org/grid_data/download/71617960?include=energy
B230220B15Rik	0.936	http://api.brain-map.org/grid_data/download/70231134?include=energy
Rpl32	0.936	http://api.brain-map.org/grid_data/download/75934920?include=energy
Fabp3	0.936	http://api.brain-map.org/grid_data/download/418?include=energy
Zik1	0.936	http://api.brain-map.org/grid_data/download/70609206?include=energy
Zfp647	0.936	http://api.brain-map.org/grid_data/download/75990519?include=energy
Mmab	0.936	http://api.brain-map.org/grid_data/download/68861797?include=energy
Kif15	0.936	http://api.brain-map.org/grid_data/download/1506?include=energy
Clstn1	0.936	http://api.brain-map.org/grid_data/download/73769319?include=energy
4922502D21Rik	0.936	http://api.brain-map.org/grid_data/download/72472451?include=energy
9330159F19Rik	0.936	http://api.brain-map.org/grid_data/download/69446661?include=energy
Thoc5	0.936	http://api.brain-map.org/grid_data/download/69549665?include=energy
Tgs1	0.936	http://api.brain-map.org/grid_data/download/71234533?include=energy
Mta2	0.936	http://api.brain-map.org/grid_data/download/69289423?include=energy
Tcp1	0.936	http://api.brain-map.org/grid_data/download/68191552?include=energy
Olfr1038-ps	0.936	http://api.brain-map.org/grid_data/download/74720573?include=energy
Lalba	0.935	http://api.brain-map.org/grid_data/download/70546051?include=energy
Gstm5	0.935	http://api.brain-map.org/grid_data/download/75198100?include=energy
Alg9	0.935	http://api.brain-map.org/grid_data/download/638708?include=energy
Gtl3	0.935	http://api.brain-map.org/grid_data/download/69734815?include=energy
Nlgn2	0.935	http://api.brain-map.org/grid_data/download/77656728?include=energy
Rnmtl1	0.935	http://api.brain-map.org/grid_data/download/75985973?include=energy
Atp5sl	0.935	http://api.brain-map.org/grid_data/download/75773705?include=energy
Hist2h2aa1	0.935	http://api.brain-map.org/grid_data/download/72129296?include=energy
Dhrs7c	0.935	http://api.brain-map.org/grid_data/download/68546236?include=energy
Snap91	0.935	http://api.brain-map.org/grid_data/download/984?include=energy
Asb8	0.935	http://api.brain-map.org/grid_data/download/276276?include=energy
Gm7467	0.935	http://api.brain-map.org/grid_data/download/74076060?include=energy
Ephx3	0.935	http://api.brain-map.org/grid_data/download/70228681?include=energy
LOC433602	0.935	http://api.brain-map.org/grid_data/download/74736243?include=energy
Donson	0.935	http://api.brain-map.org/grid_data/download/2157?include=energy
LOC218357	0.935	http://api.brain-map.org/grid_data/download/70724995?include=energy
Gpr50	0.935	http://api.brain-map.org/grid_data/download/74511770?include=energy
Clk1	0.935	http://api.brain-map.org/grid_data/download/71160340?include=energy
LOC235580	0.935	http://api.brain-map.org/grid_data/download/71924441?include=energy
Pde4dip	0.935	http://api.brain-map.org/grid_data/download/1994?include=energy
B230362M20Rik	0.935	http://api.brain-map.org/grid_data/download/77340460?include=energy
Foxd4	0.935	http://api.brain-map.org/grid_data/download/71280499?include=energy
Tmem245	0.935	http://api.brain-map.org/grid_data/download/71809103?include=energy
Rundc3a	0.935	http://api.brain-map.org/grid_data/download/68745366?include=energy
Sik1	0.935	http://api.brain-map.org/grid_data/download/69885576?include=energy
Zfp758	0.935	http://api.brain-map.org/grid_data/download/68445191?include=energy
Unc119b	0.935	http://api.brain-map.org/grid_data/download/69514500?include=energy
Lass4	0.935	http://api.brain-map.org/grid_data/download/176962?include=energy
Depdc1b	0.935	http://api.brain-map.org/grid_data/download/70238836?include=energy
Pspc1	0.935	http://api.brain-map.org/grid_data/download/71656122?include=energy
Usp11	0.935	http://api.brain-map.org/grid_data/download/77869130?include=energy
Olfr420	0.935	http://api.brain-map.org/grid_data/download/74724091?include=energy
Hdgfrp3	0.935	http://api.brain-map.org/grid_data/download/71656863?include=energy
Olfr730	0.935	http://api.brain-map.org/grid_data/download/74734902?include=energy
Hhipl2	0.935	http://api.brain-map.org/grid_data/download/68443459?include=energy
Ppcs	0.935	http://api.brain-map.org/grid_data/download/68341621?include=energy
Atf4	0.935	http://api.brain-map.org/grid_data/download/69288650?include=energy
Tex2	0.935	http://api.brain-map.org/grid_data/download/68798729?include=energy
Csnk2a1	0.935	http://api.brain-map.org/grid_data/download/68546488?include=energy
Arhgef1	0.935	http://api.brain-map.org/grid_data/download/74357610?include=energy
LOC432852	0.935	http://api.brain-map.org/grid_data/download/71808564?include=energy
Tfam	0.935	http://api.brain-map.org/grid_data/download/67936016?include=energy
Napa	0.935	http://api.brain-map.org/grid_data/download/532632?include=energy
Fam25c	0.935	http://api.brain-map.org/grid_data/download/73716543?include=energy
Fuca1	0.935	http://api.brain-map.org/grid_data/download/532572?include=energy
Kcng4	0.935	http://api.brain-map.org/grid_data/download/72081560?include=energy
Gm761	0.935	http://api.brain-map.org/grid_data/download/70298328?include=energy
Rnpep	0.935	http://api.brain-map.org/grid_data/download/76098385?include=energy
Plat	0.935	http://api.brain-map.org/grid_data/download/70439075?include=energy
Abhd4	0.935	http://api.brain-map.org/grid_data/download/638713?include=energy
Cd52	0.935	http://api.brain-map.org/grid_data/download/69261424?include=energy
Plekhh3	0.935	http://api.brain-map.org/grid_data/download/583849?include=energy
Atpif1	0.935	http://api.brain-map.org/grid_data/download/75774682?include=energy
Wdr37	0.935	http://api.brain-map.org/grid_data/download/70195000?include=energy
Eps15	0.935	http://api.brain-map.org/grid_data/download/72080144?include=energy
Tmprss15	0.935	http://api.brain-map.org/grid_data/download/77464910?include=energy
Zfp13	0.935	http://api.brain-map.org/grid_data/download/73584813?include=energy
Pou4f3	0.935	http://api.brain-map.org/grid_data/download/71609031?include=energy
Hsp90b1	0.935	http://api.brain-map.org/grid_data/download/75042251?include=energy
Hipk4	0.935	http://api.brain-map.org/grid_data/download/70295942?include=energy
Tspo2	0.935	http://api.brain-map.org/grid_data/download/75934522?include=energy
Ccdc99	0.935	http://api.brain-map.org/grid_data/download/69059778?include=energy
Acbd4	0.935	http://api.brain-map.org/grid_data/download/69549639?include=energy
Mxra8	0.934	http://api.brain-map.org/grid_data/download/71656072?include=energy
Mfhas1	0.934	http://api.brain-map.org/grid_data/download/70946525?include=energy
Dtx1	0.934	http://api.brain-map.org/grid_data/download/2167?include=energy
Ppp1r13b	0.934	http://api.brain-map.org/grid_data/download/68798992?include=energy
Zfp187	0.934	http://api.brain-map.org/grid_data/download/70611234?include=energy
Nrp2	0.934	http://api.brain-map.org/grid_data/download/80514091?include=energy
Bloc1s1	0.934	http://api.brain-map.org/grid_data/download/100144717?include=energy
Klra2	0.934	http://api.brain-map.org/grid_data/download/75861005?include=energy
Asb3	0.934	http://api.brain-map.org/grid_data/download/199219?include=energy
Rhox1	0.934	http://api.brain-map.org/grid_data/download/74059302?include=energy
Stx8	0.934	http://api.brain-map.org/grid_data/download/69114902?include=energy
Fgd5	0.934	http://api.brain-map.org/grid_data/download/74357548?include=energy
Tgif2	0.934	http://api.brain-map.org/grid_data/download/73584819?include=energy
Ugdh	0.934	http://api.brain-map.org/grid_data/download/77280339?include=energy
Rin3	0.934	http://api.brain-map.org/grid_data/download/71735064?include=energy
Mrps33	0.934	http://api.brain-map.org/grid_data/download/69133818?include=energy
Slc31a2	0.934	http://api.brain-map.org/grid_data/download/75773700?include=energy
Pink1	0.934	http://api.brain-map.org/grid_data/download/69783208?include=energy
9330182O14Rik	0.934	http://api.brain-map.org/grid_data/download/69446685?include=energy
Bod1l	0.934	http://api.brain-map.org/grid_data/download/69524410?include=energy
Nxph2	0.934	http://api.brain-map.org/grid_data/download/74277689?include=energy
Rara	0.934	http://api.brain-map.org/grid_data/download/1528?include=energy
Dld	0.934	http://api.brain-map.org/grid_data/download/70445700?include=energy
4930455C21Rik	0.934	http://api.brain-map.org/grid_data/download/513492?include=energy
Atl3	0.934	http://api.brain-map.org/grid_data/download/583570?include=energy
Mospd1	0.934	http://api.brain-map.org/grid_data/download/68637623?include=energy
Tmem216	0.934	http://api.brain-map.org/grid_data/download/69837170?include=energy
Icam1	0.934	http://api.brain-map.org/grid_data/download/79536461?include=energy
Smarca2	0.934	http://api.brain-map.org/grid_data/download/1972?include=energy
Them5	0.934	http://api.brain-map.org/grid_data/download/69262279?include=energy
Ccdc51	0.934	http://api.brain-map.org/grid_data/download/69443523?include=energy
P4htm	0.934	http://api.brain-map.org/grid_data/download/69443130?include=energy
Amacr	0.934	http://api.brain-map.org/grid_data/download/68300992?include=energy
Hmg20a	0.934	http://api.brain-map.org/grid_data/download/357093?include=energy
Acin1	0.934	http://api.brain-map.org/grid_data/download/100146043?include=energy
4933401L05Rik	0.934	http://api.brain-map.org/grid_data/download/71493693?include=energy
Kdelc2	0.934	http://api.brain-map.org/grid_data/download/76003354?include=energy
H2afx	0.934	http://api.brain-map.org/grid_data/download/70545961?include=energy
Zp3	0.934	http://api.brain-map.org/grid_data/download/71991995?include=energy
Kcnab2	0.934	http://api.brain-map.org/grid_data/download/1754?include=energy
F830215A15Rik*	0.934	http://api.brain-map.org/grid_data/download/73748761?include=energy
4930431F10Rik	0.934	http://api.brain-map.org/grid_data/download/73513251?include=energy
Gnb1	0.934	http://api.brain-map.org/grid_data/download/61413?include=energy
Bad	0.934	http://api.brain-map.org/grid_data/download/74881790?include=energy
Drg2	0.934	http://api.brain-map.org/grid_data/download/73606688?include=energy
Dido1	0.934	http://api.brain-map.org/grid_data/download/69262318?include=energy
Hddc2	0.934	http://api.brain-map.org/grid_data/download/71991840?include=energy
Lce1m	0.934	http://api.brain-map.org/grid_data/download/70785128?include=energy
Pcdhb14	0.934	http://api.brain-map.org/grid_data/download/79354790?include=energy
Atp2b1	0.934	http://api.brain-map.org/grid_data/download/77280449?include=energy
Grm5	0.934	http://api.brain-map.org/grid_data/download/73512423?include=energy
Tcp11l1	0.934	http://api.brain-map.org/grid_data/download/71496031?include=energy
Gng3	0.934	http://api.brain-map.org/grid_data/download/1209?include=energy
Gpx3	0.934	http://api.brain-map.org/grid_data/download/75080750?include=energy
Rdh9	0.934	http://api.brain-map.org/grid_data/download/76081906?include=energy

Cdc27	0.934	http://api.brain-map.org/grid_data/download/293466?include=energy
2410001C21Rik	0.934	http://api.brain-map.org/grid_data/download/69443481?include=energy
Vta1	0.934	http://api.brain-map.org/grid_data/download/68637814?include=energy
Zfp961	0.934	http://api.brain-map.org/grid_data/download/70566151?include=energy
Zmynd8	0.934	http://api.brain-map.org/grid_data/download/71833000?include=energy
Nell2	0.934	http://api.brain-map.org/grid_data/download/72103854?include=energy
Ttc30b	0.934	http://api.brain-map.org/grid_data/download/70303809?include=energy
Rap1a	0.934	http://api.brain-map.org/grid_data/download/75831752?include=energy
LOC433491	0.934	http://api.brain-map.org/grid_data/download/70723313?include=energy
Xpo6	0.934	http://api.brain-map.org/grid_data/download/68919904?include=energy
Prpf4	0.934	http://api.brain-map.org/grid_data/download/100146143?include=energy
Atp5e	0.934	http://api.brain-map.org/grid_data/download/69173370?include=energy
Uck2	0.934	http://api.brain-map.org/grid_data/download/71489807?include=energy
Elavl3	0.934	http://api.brain-map.org/grid_data/download/74359628?include=energy
Oas1a	0.934	http://api.brain-map.org/grid_data/download/69028408?include=energy
B230120H23Rik	0.934	http://api.brain-map.org/grid_data/download/2049?include=energy
Kdm4a	0.934	http://api.brain-map.org/grid_data/download/532714?include=energy
Gh	0.933	http://api.brain-map.org/grid_data/download/74513935?include=energy
Tuba4a	0.933	http://api.brain-map.org/grid_data/download/70616005?include=energy
Msh2	0.933	http://api.brain-map.org/grid_data/download/74657951?include=energy
Agpat3	0.933	http://api.brain-map.org/grid_data/download/85?include=energy
Uqcrc2	0.933	http://api.brain-map.org/grid_data/download/655502?include=energy
Slc7a6	0.933	http://api.brain-map.org/grid_data/download/293662?include=energy
Gorasp2	0.933	http://api.brain-map.org/grid_data/download/69015823?include=energy
Ccdc56	0.933	http://api.brain-map.org/grid_data/download/75828180?include=energy
Trim28	0.933	http://api.brain-map.org/grid_data/download/1071?include=energy
Steap2	0.933	http://api.brain-map.org/grid_data/download/71670697?include=energy
Tpcn2	0.933	http://api.brain-map.org/grid_data/download/69169839?include=energy
Vsx2	0.933	http://api.brain-map.org/grid_data/download/1687?include=energy
AI314976	0.933	http://api.brain-map.org/grid_data/download/69169767?include=energy
Cpvl	0.933	http://api.brain-map.org/grid_data/download/75931614?include=energy
Usp53	0.933	http://api.brain-map.org/grid_data/download/71211434?include=energy
Hpca	0.933	http://api.brain-map.org/grid_data/download/72129291?include=energy
Pgk2	0.933	http://api.brain-map.org/grid_data/download/71764174?include=energy
Pigq	0.933	http://api.brain-map.org/grid_data/download/356489?include=energy
Clasp1	0.933	http://api.brain-map.org/grid_data/download/73811573?include=energy
LOC434301	0.933	http://api.brain-map.org/grid_data/download/70793881?include=energy
Fthl17	0.933	http://api.brain-map.org/grid_data/download/100144392?include=energy
Higd2a	0.933	http://api.brain-map.org/grid_data/download/77910761?include=energy
Igf2r	0.933	http://api.brain-map.org/grid_data/download/69735269?include=energy
Zfp598	0.933	http://api.brain-map.org/grid_data/download/68797703?include=energy
Clec4g	0.933	http://api.brain-map.org/grid_data/download/74821778?include=energy
Nek4	0.933	http://api.brain-map.org/grid_data/download/69028336?include=energy
Ttll9	0.933	http://api.brain-map.org/grid_data/download/75934927?include=energy
Capsl	0.933	http://api.brain-map.org/grid_data/download/75079776?include=energy
LOC432685	0.933	http://api.brain-map.org/grid_data/download/70611198?include=energy
Fam125b	0.933	http://api.brain-map.org/grid_data/download/71493057?include=energy
G370126I09Rik*	0.933	http://api.brain-map.org/grid_data/download/73716674?include=energy
Dennd5b	0.933	http://api.brain-map.org/grid_data/download/74514129?include=energy
Tmem184c	0.933	http://api.brain-map.org/grid_data/download/70474047?include=energy
Fgl1	0.933	http://api.brain-map.org/grid_data/download/68410512?include=energy
8030474K03Rik	0.933	http://api.brain-map.org/grid_data/download/71809121?include=energy
Vgll3	0.933	http://api.brain-map.org/grid_data/download/72283713?include=energy
Dynlrb2	0.933	http://api.brain-map.org/grid_data/download/69236598?include=energy
Otop3	0.933	http://api.brain-map.org/grid_data/download/71210654?include=energy
Cmc1	0.933	http://api.brain-map.org/grid_data/download/75831769?include=energy
Gm5544	0.933	http://api.brain-map.org/grid_data/download/71456122?include=energy
Slc39a2	0.933	http://api.brain-map.org/grid_data/download/71210914?include=energy
Senp7	0.933	http://api.brain-map.org/grid_data/download/76081887?include=energy
Spef1	0.933	http://api.brain-map.org/grid_data/download/68443110?include=energy
Cds2	0.933	http://api.brain-map.org/grid_data/download/250?include=energy
Afap1l2	0.933	http://api.brain-map.org/grid_data/download/67764927?include=energy
F830208N12Rik*	0.933	http://api.brain-map.org/grid_data/download/73731442?include=energy
Gdap1l1	0.933	http://api.brain-map.org/grid_data/download/75774676?include=energy
Fbxl21	0.933	http://api.brain-map.org/grid_data/download/71497006?include=energy
Gbp3	0.933	http://api.brain-map.org/grid_data/download/68445215?include=energy
Sub1	0.933	http://api.brain-map.org/grid_data/download/356490?include=energy
Acacb	0.933	http://api.brain-map.org/grid_data/download/74881515?include=energy
Dusp22	0.933	http://api.brain-map.org/grid_data/download/76003520?include=energy
Cdc25a	0.933	http://api.brain-map.org/grid_data/download/75831746?include=energy
Smr3a	0.933	http://api.brain-map.org/grid_data/download/71724708?include=energy
Tsen15	0.933	http://api.brain-map.org/grid_data/download/71723833?include=energy
Ift88	0.933	http://api.brain-map.org/grid_data/download/69838761?include=energy
Gtse1	0.933	http://api.brain-map.org/grid_data/download/70545949?include=energy
Hmgn3	0.933	http://api.brain-map.org/grid_data/download/69059927?include=energy
Krba1	0.933	http://api.brain-map.org/grid_data/download/77620433?include=energy
Pcdhgb7	0.933	http://api.brain-map.org/grid_data/download/70784940?include=energy
D17H6S53E	0.933	http://api.brain-map.org/grid_data/download/70238627?include=energy
Kctd3	0.933	http://api.brain-map.org/grid_data/download/69258100?include=energy
mCG140353	0.933	http://api.brain-map.org/grid_data/download/71211483?include=energy
P4ha1	0.933	http://api.brain-map.org/grid_data/download/69080014?include=energy
Vmn2r81	0.933	http://api.brain-map.org/grid_data/download/69236825?include=energy
Hk1	0.933	http://api.brain-map.org/grid_data/download/69735054?include=energy
Gfm2	0.933	http://api.brain-map.org/grid_data/download/67787343?include=energy
Gm271	0.933	http://api.brain-map.org/grid_data/download/70296038?include=energy
Oxsm	0.933	http://api.brain-map.org/grid_data/download/69443220?include=energy
Sh3bgr	0.933	http://api.brain-map.org/grid_data/download/1416?include=energy
Ccr1	0.933	http://api.brain-map.org/grid_data/download/73718058?include=energy
Polr3gl	0.933	http://api.brain-map.org/grid_data/download/68795398?include=energy
Gpt2	0.933	http://api.brain-map.org/grid_data/download/1657?include=energy
Tmem63b	0.933	http://api.brain-map.org/grid_data/download/69014422?include=energy
Mtmr11	0.932	http://api.brain-map.org/grid_data/download/69013302?include=energy
B020031H02Rik	0.932	http://api.brain-map.org/grid_data/download/74578383?include=energy
Mboat1	0.932	http://api.brain-map.org/grid_data/download/69549150?include=energy
Npas2	0.932	http://api.brain-map.org/grid_data/download/74724764?include=energy
Kcnj5	0.932	http://api.brain-map.org/grid_data/download/72119660?include=energy
Epcam	0.932	http://api.brain-map.org/grid_data/download/76097707?include=energy
Extl2	0.932	http://api.brain-map.org/grid_data/download/70613979?include=energy
Grpel2	0.932	http://api.brain-map.org/grid_data/download/69734602?include=energy
LOC435735	0.932	http://api.brain-map.org/grid_data/download/71022573?include=energy
Sult1e1	0.932	http://api.brain-map.org/grid_data/download/68545626?include=energy
Smyd2	0.932	http://api.brain-map.org/grid_data/download/69080050?include=energy
Poteg	0.932	http://api.brain-map.org/grid_data/download/74579981?include=energy
BC034076	0.932	http://api.brain-map.org/grid_data/download/71836848?include=energy
Hsd17b13	0.932	http://api.brain-map.org/grid_data/download/68410375?include=energy
Derl2	0.932	http://api.brain-map.org/grid_data/download/386232?include=energy
1700054O13Rik	0.932	http://api.brain-map.org/grid_data/download/69117086?include=energy
Trub1	0.932	http://api.brain-map.org/grid_data/download/69202800?include=energy
Rps4y2	0.932	http://api.brain-map.org/grid_data/download/75934917?include=energy
3110043O21Rik	0.932	http://api.brain-map.org/grid_data/download/69549264?include=energy
Rasgef1a	0.932	http://api.brain-map.org/grid_data/download/69817976?include=energy
Hsd17b10	0.932	http://api.brain-map.org/grid_data/download/68545911?include=energy
Mmp28	0.932	http://api.brain-map.org/grid_data/download/71210508?include=energy
Rbp3	0.932	http://api.brain-map.org/grid_data/download/68342360?include=energy
5430416O09Rik	0.932	http://api.brain-map.org/grid_data/download/70218914?include=energy
Arhgef26	0.932	http://api.brain-map.org/grid_data/download/73931623?include=energy
Psmd11	0.932	http://api.brain-map.org/grid_data/download/74743282?include=energy
9930014A18Rik	0.932	http://api.brain-map.org/grid_data/download/69447852?include=energy
Mrpl49	0.932	http://api.brain-map.org/grid_data/download/71656065?include=energy
Tmco7	0.932	http://api.brain-map.org/grid_data/download/69527734?include=energy
F8a	0.932	http://api.brain-map.org/grid_data/download/74513953?include=energy
Usp49	0.932	http://api.brain-map.org/grid_data/download/70528431?include=energy
Chst8	0.932	http://api.brain-map.org/grid_data/download/72080187?include=energy
Gc	0.932	http://api.brain-map.org/grid_data/download/75694889?include=energy
Gkn1	0.932	http://api.brain-map.org/grid_data/download/71587727?include=energy
1700104A03Rik	0.932	http://api.brain-map.org/grid_data/download/73716710?include=energy
Hif1a	0.932	http://api.brain-map.org/grid_data/download/75828189?include=energy
Gsta3	0.932	http://api.brain-map.org/grid_data/download/68301419?include=energy
Cul3	0.932	http://api.brain-map.org/grid_data/download/75774648?include=energy
Prss58	0.932	http://api.brain-map.org/grid_data/download/69113823?include=energy
Pxn	0.932	http://api.brain-map.org/grid_data/download/1814?include=energy
Ttc36	0.932	http://api.brain-map.org/grid_data/download/74579473?include=energy
C920006O11Rik	0.932	http://api.brain-map.org/grid_data/download/74822582?include=energy
Klf3	0.932	http://api.brain-map.org/grid_data/download/69289291?include=energy
Brd1	0.932	http://api.brain-map.org/grid_data/download/75233702?include=energy
Pcyt1b	0.932	http://api.brain-map.org/grid_data/download/74641319?include=energy
Pcdhgc3	0.932	http://api.brain-map.org/grid_data/download/70784952?include=energy
Plekhf2	0.932	http://api.brain-map.org/grid_data/download/68342294?include=energy
Pbx2	0.932	http://api.brain-map.org/grid_data/download/68203470?include=energy
Cby1	0.932	http://api.brain-map.org/grid_data/download/69059941?include=energy
Col22a1	0.932	http://api.brain-map.org/grid_data/download/74635421?include=energy
Rxfp3	0.932	http://api.brain-map.org/grid_data/download/71358555?include=energy
Samd11	0.932	http://api.brain-map.org/grid_data/download/70928360?include=energy
Cxcl9	0.932	http://api.brain-map.org/grid_data/download/67979053?include=energy
LOC434825	0.932	http://api.brain-map.org/grid_data/download/71764413?include=energy
Ndufaf4	0.932	http://api.brain-map.org/grid_data/download/68523306?include=energy
Zmat2	0.931	http://api.brain-map.org/grid_data/download/69080733?include=energy
Slitrk5	0.931	http://api.brain-map.org/grid_data/download/73573193?include=energy
Snx15	0.931	http://api.brain-map.org/grid_data/download/583543?include=energy
Gcgr	0.931	http://api.brain-map.org/grid_data/download/1613?include=energy
0610009D07Rik	0.931	http://api.brain-map.org/grid_data/download/68342153?include=energy
Slc50a1	0.931	http://api.brain-map.org/grid_data/download/67870478?include=energy
Brp44	0.931	http://api.brain-map.org/grid_data/download/79393535?include=energy
F630025H21Rik*	0.931	http://api.brain-map.org/grid_data/download/73748860?include=energy
Folr4	0.931	http://api.brain-map.org/grid_data/download/69549169?include=energy
Dapl1	0.931	http://api.brain-map.org/grid_data/download/70218691?include=energy
4931417G12Rik	0.931	http://api.brain-map.org/grid_data/download/70218480?include=energy
Fzd1	0.931	http://api.brain-map.org/grid_data/download/2239?include=energy
TC1462499	0.931	http://api.brain-map.org/grid_data/download/70795829?include=energy
Ppp1r2-ps9	0.931	http://api.brain-map.org/grid_data/download/71570756?include=energy
Zfp39	0.931	http://api.brain-map.org/grid_data/download/69352700?include=energy
Vamp7	0.931	http://api.brain-map.org/grid_data/download/71724738?include=energy
Pcmtd2	0.931	http://api.brain-map.org/grid_data/download/68157388?include=energy
Vps13b	0.931	http://api.brain-map.org/grid_data/download/74359371?include=energy
Erg	0.931	http://api.brain-map.org/grid_data/download/77464970?include=energy
Cd200r2	0.931	http://api.brain-map.org/grid_data/download/71021129?include=energy
Txlng	0.931	http://api.brain-map.org/grid_data/download/74513911?include=energy
Atp8b3	0.931	http://api.brain-map.org/grid_data/download/69134462?include=energy
AI506816	0.931	http://api.brain-map.org/grid_data/download/70300192?include=energy
Hist1h2bp	0.931	http://api.brain-map.org/grid_data/download/69116889?include=energy
C2cd2l	0.931	http://api.brain-map.org/grid_data/download/73592532?include=energy
Ccl4	0.931	http://api.brain-map.org/grid_data/download/75988357?include=energy
Cml3	0.931	http://api.brain-map.org/grid_data/download/69015721?include=energy
Mars	0.931	http://api.brain-map.org/grid_data/download/76003692?include=energy
Cpsf1	0.931	http://api.brain-map.org/grid_data/download/69608028?include=energy
Olfr646	0.931	http://api.brain-map.org/grid_data/download/74734782?include=energy
Lsg1	0.931	http://api.brain-map.org/grid_data/download/68920809?include=energy
6330406I15Rik	0.931	http://api.brain-map.org/grid_data/download/69444813?include=energy
H2afy2	0.931	http://api.brain-map.org/grid_data/download/68745450?include=energy
Arf6	0.931	http://api.brain-map.org/grid_data/download/67979033?include=energy
Zfp939	0.931	http://api.brain-map.org/grid_data/download/74431673?include=energy
Aldh1a3	0.931	http://api.brain-map.org/grid_data/download/75861799?include=energy
Ddx39b	0.931	http://api.brain-map.org/grid_data/download/68148739?include=energy
Atp6v0d1	0.931	http://api.brain-map.org/grid_data/download/564647?include=energy
Atp6v0e	0.931	http://api.brain-map.org/grid_data/download/68196930?include=energy
Slc19a1	0.931	http://api.brain-map.org/grid_data/download/1282?include=energy
3110073H01Rik	0.931	http://api.brain-map.org/grid_data/download/71212123?include=energy
LOC545505	0.931	http://api.brain-map.org/grid_data/download/74822586?include=energy
Tie1	0.931	http://api.brain-map.org/grid_data/download/69262303?include=energy
Tead3	0.931	http://api.brain-map.org/grid_data/download/74513878?include=energy
Myrip	0.931	http://api.brain-map.org/grid_data/download/71789885?include=energy
Prl8a9	0.931	http://api.brain-map.org/grid_data/download/71656664?include=energy
Ccdc27	0.931	http://api.brain-map.org/grid_data/download/74562552?include=energy
Fam164b	0.931	http://api.brain-map.org/grid_data/download/71891814?include=energy
Gab3	0.931	http://api.brain-map.org/grid_data/download/69672707?include=energy
Ezh2	0.931	http://api.brain-map.org/grid_data/download/100142521?include=energy
Ccdc124	0.931	http://api.brain-map.org/grid_data/download/69174040?include=energy
Ehbp1l1	0.931	http://api.brain-map.org/grid_data/download/73817932?include=energy
Dgka	0.931	http://api.brain-map.org/grid_data/download/71148109?include=energy
LOC435374	0.931	http://api.brain-map.org/grid_data/download/71022487?include=energy
Lmx1b	0.931	http://api.brain-map.org/grid_data/download/1508?include=energy
LOC432617	0.931	http://api.brain-map.org/grid_data/download/70611126?include=energy
LOC328369	0.931	http://api.brain-map.org/grid_data/download/70595853?include=energy
Sult1b1	0.931	http://api.brain-map.org/grid_data/download/68499410?include=energy
Pank2	0.93	http://api.brain-map.org/grid_data/download/71920479?include=energy
Gdpd1	0.93	http://api.brain-map.org/grid_data/download/70429507?include=energy
Selenbp1	0.93	http://api.brain-map.org/grid_data/download/71764287?include=energy
Tsc22d3	0.93	http://api.brain-map.org/grid_data/download/74581366?include=energy
Ubxn2a	0.93	http://api.brain-map.org/grid_data/download/393255?include=energy
Pou4f1	0.93	http://api.brain-map.org/grid_data/download/74641304?include=energy
Mettl14	0.93	http://api.brain-map.org/grid_data/download/68667144?include=energy
Emilin3	0.93	http://api.brain-map.org/grid_data/download/69672013?include=energy
Olfr639	0.93	http://api.brain-map.org/grid_data/download/74734764?include=energy
Chst10	0.93	http://api.brain-map.org/grid_data/download/74657931?include=energy
Cd209a	0.93	http://api.brain-map.org/grid_data/download/74277384?include=energy
Opn1sw	0.93	http://api.brain-map.org/grid_data/download/75773699?include=energy
Aldh1b1	0.93	http://api.brain-map.org/grid_data/download/75827098?include=energy
Gm15800	0.93	http://api.brain-map.org/grid_data/download/69013450?include=energy
Megf11	0.93	http://api.brain-map.org/grid_data/download/74357580?include=energy
Actn3	0.93	http://api.brain-map.org/grid_data/download/69514696?include=energy
Pdxp	0.93	http://api.brain-map.org/grid_data/download/69782963?include=energy
B020018G13Rik*	0.93	http://api.brain-map.org/grid_data/download/74000522?include=energy
1700048O20Rik	0.93	http://api.brain-map.org/grid_data/download/74357863?include=energy
Slco1a4	0.93	http://api.brain-map.org/grid_data/download/71570751?include=energy
Cpne7	0.93	http://api.brain-map.org/grid_data/download/73817432?include=energy
Itgb8	0.93	http://api.brain-map.org/grid_data/download/73931631?include=energy
Ccdc70	0.93	http://api.brain-map.org/grid_data/download/69114992?include=energy
Mylk4	0.93	http://api.brain-map.org/grid_data/download/70566211?include=energy
Zfp622	0.93	http://api.brain-map.org/grid_data/download/71489799?include=energy
Map3k15	0.93	http://api.brain-map.org/grid_data/download/73788441?include=energy
Usp19	0.93	http://api.brain-map.org/grid_data/download/69169331?include=energy
Mtf1	0.93	http://api.brain-map.org/grid_data/download/2409?include=energy
Ier3	0.93	http://api.brain-map.org/grid_data/download/72008119?include=energy
Kpnb1	0.93	http://api.brain-map.org/grid_data/download/77340472?include=energy
4930511I11Rik	0.93	http://api.brain-map.org/grid_data/download/75850471?include=energy
D830044D21Rik	0.93	http://api.brain-map.org/grid_data/download/70238776?include=energy
Clip2	0.93	http://api.brain-map.org/grid_data/download/2137?include=energy
Actg1	0.93	http://api.brain-map.org/grid_data/download/67792203?include=energy
Pten	0.93	http://api.brain-map.org/grid_data/download/68445185?include=energy
Klhl31	0.93	http://api.brain-map.org/grid_data/download/73818826?include=energy
Aldob	0.93	http://api.brain-map.org/grid_data/download/79588294?include=energy
Nr4a2	0.93	http://api.brain-map.org/grid_data/download/732?include=energy
Hibadh	0.93	http://api.brain-map.org/grid_data/download/68076536?include=energy
Dlgap4	0.93	http://api.brain-map.org/grid_data/download/68499267?include=energy
Btn2a2	0.93	http://api.brain-map.org/grid_data/download/76081031?include=energy
Ube2o	0.93	http://api.brain-map.org/grid_data/download/69014985?include=energy
Ndufs4	0.93	http://api.brain-map.org/grid_data/download/393253?include=energy
Magi3	0.93	http://api.brain-map.org/grid_data/download/75041515?include=energy
Olfr568	0.93	http://api.brain-map.org/grid_data/download/74724199?include=energy
Zp3r	0.93	http://api.brain-map.org/grid_data/download/72004495?include=energy
Ptpn9	0.93	http://api.brain-map.org/grid_data/download/70919824?include=energy
Zic2	0.93	http://api.brain-map.org/grid_data/download/71488925?include=energy
Ttyh3	0.93	http://api.brain-map.org/grid_data/download/74988711?include=energy
Krt14	0.93	http://api.brain-map.org/grid_data/download/77620469?include=energy
Atp8a1	0.93	http://api.brain-map.org/grid_data/download/74881560?include=energy
Rhbdd1	0.93	http://api.brain-map.org/grid_data/download/100146138?include=energy
Rab1	0.93	http://api.brain-map.org/grid_data/download/878?include=energy
Zmiz1	0.93	http://api.brain-map.org/grid_data/download/74988259?include=energy
LOC434853	0.93	http://api.brain-map.org/grid_data/download/71022427?include=energy
Naa25	0.93	http://api.brain-map.org/grid_data/download/71281168?include=energy
Lpgat1	0.93	http://api.brain-map.org/grid_data/download/576419?include=energy
Asns	0.93	http://api.brain-map.org/grid_data/download/74047788?include=energy
Stx18	0.93	http://api.brain-map.org/grid_data/download/70474029?include=energy
Pdxdc1	0.93	http://api.brain-map.org/grid_data/download/77869146?include=energy
Ppm1k	0.93	http://api.brain-map.org/grid_data/download/69514452?include=energy
Sned1	0.93	http://api.brain-map.org/grid_data/download/74958587?include=energy
Mm.125279	0.93	http://api.brain-map.org/grid_data/download/71148022?include=energy

Rilpl1	0.93	http://api.brain-map.org/grid_data/download/69837493?include=energy
Trpm7	0.93	http://api.brain-map.org/grid_data/download/69288441?include=energy
Oxct1	0.93	http://api.brain-map.org/grid_data/download/655499?include=energy
Gnat1	0.93	http://api.brain-map.org/grid_data/download/2265?include=energy
Nfxl1	0.93	http://api.brain-map.org/grid_data/download/76135740?include=energy
LOC545411	0.929	http://api.brain-map.org/grid_data/download/77620839?include=energy
Wdr92	0.929	http://api.brain-map.org/grid_data/download/69526366?include=energy
4930412M03Rik	0.929	http://api.brain-map.org/grid_data/download/72182941?include=energy
A930024E05Rik	0.929	http://api.brain-map.org/grid_data/download/69514446?include=energy
Rps15	0.929	http://api.brain-map.org/grid_data/download/75774687?include=energy
Fkbp3	0.929	http://api.brain-map.org/grid_data/download/632493?include=energy
1110006G14Rik	0.929	http://api.brain-map.org/grid_data/download/74821766?include=energy
Ube2l3	0.929	http://api.brain-map.org/grid_data/download/71378583?include=energy
Esr2	0.929	http://api.brain-map.org/grid_data/download/71670737?include=energy
Ankrd52	0.929	http://api.brain-map.org/grid_data/download/69289010?include=energy
4930579G24Rik	0.929	http://api.brain-map.org/grid_data/download/69442398?include=energy
Snrnp25	0.929	http://api.brain-map.org/grid_data/download/68544997?include=energy
A230096H11Rik*	0.929	http://api.brain-map.org/grid_data/download/73949614?include=energy
Itgad	0.929	http://api.brain-map.org/grid_data/download/70596143?include=energy
Pcbp4	0.929	http://api.brain-map.org/grid_data/download/67936208?include=energy
Sra1	0.929	http://api.brain-map.org/grid_data/download/71763601?include=energy
Sec31a	0.929	http://api.brain-map.org/grid_data/download/76003605?include=energy
Ccdc60	0.929	http://api.brain-map.org/grid_data/download/70305526?include=energy
Cald1	0.929	http://api.brain-map.org/grid_data/download/70919250?include=energy
Amph	0.929	http://api.brain-map.org/grid_data/download/72340233?include=energy
Hhipl1	0.929	http://api.brain-map.org/grid_data/download/69526504?include=energy
Ap1b1	0.929	http://api.brain-map.org/grid_data/download/77869778?include=energy
Fam81b	0.929	http://api.brain-map.org/grid_data/download/70296044?include=energy
Dstyk	0.929	http://api.brain-map.org/grid_data/download/71020889?include=energy
Ncrna00086	0.929	http://api.brain-map.org/grid_data/download/69104150?include=energy
4921510H08Rik	0.929	http://api.brain-map.org/grid_data/download/70205620?include=energy
Il31ra	0.929	http://api.brain-map.org/grid_data/download/74881366?include=energy
Kif7	0.929	http://api.brain-map.org/grid_data/download/69735813?include=energy
Poll	0.929	http://api.brain-map.org/grid_data/download/67978500?include=energy
Fanci	0.929	http://api.brain-map.org/grid_data/download/68498920?include=energy
Gm773	0.929	http://api.brain-map.org/grid_data/download/71764377?include=energy
Cacna1b	0.929	http://api.brain-map.org/grid_data/download/71307202?include=energy
Susd3	0.929	http://api.brain-map.org/grid_data/download/69013122?include=energy
Nelf	0.929	http://api.brain-map.org/grid_data/download/74821712?include=energy
Flt1	0.929	http://api.brain-map.org/grid_data/download/2224?include=energy
F11r	0.929	http://api.brain-map.org/grid_data/download/75773698?include=energy
Hgs	0.929	http://api.brain-map.org/grid_data/download/69080030?include=energy
Ndst1	0.929	http://api.brain-map.org/grid_data/download/71234609?include=energy
Zmynd10	0.929	http://api.brain-map.org/grid_data/download/69114872?include=energy
Syce1	0.929	http://api.brain-map.org/grid_data/download/74265225?include=energy
Brd8	0.929	http://api.brain-map.org/grid_data/download/1673?include=energy
Xrn2	0.929	http://api.brain-map.org/grid_data/download/69080039?include=energy
Nkg7	0.929	http://api.brain-map.org/grid_data/download/75831759?include=energy
1700001P01Rik	0.929	http://api.brain-map.org/grid_data/download/69113799?include=energy
Ptar1	0.929	http://api.brain-map.org/grid_data/download/69407941?include=energy
Tmem61	0.929	http://api.brain-map.org/grid_data/download/71808861?include=energy
Mgea5	0.929	http://api.brain-map.org/grid_data/download/74821970?include=energy
Smc2	0.929	http://api.brain-map.org/grid_data/download/69874180?include=energy
Pgm5	0.929	http://api.brain-map.org/grid_data/download/71920494?include=energy
Zfml	0.929	http://api.brain-map.org/grid_data/download/69134544?include=energy
Defb42	0.929	http://api.brain-map.org/grid_data/download/73870593?include=energy
Tomm5	0.929	http://api.brain-map.org/grid_data/download/69352932?include=energy
D6Bwg1452e	0.929	http://api.brain-map.org/grid_data/download/69407319?include=energy
Trpm3	0.929	http://api.brain-map.org/grid_data/download/74431478?include=energy
C530050E15Rik	0.929	http://api.brain-map.org/grid_data/download/71496252?include=energy
C630002N23Rik	0.929	http://api.brain-map.org/grid_data/download/74513872?include=energy
Ywhah	0.929	http://api.brain-map.org/grid_data/download/1127?include=energy
Sh3bgrl3	0.929	http://api.brain-map.org/grid_data/download/69873708?include=energy
Gm5654	0.929	http://api.brain-map.org/grid_data/download/70725646?include=energy
Ankrd16	0.929	http://api.brain-map.org/grid_data/download/69526641?include=energy
Ppp1r1b	0.929	http://api.brain-map.org/grid_data/download/73732146?include=energy
Fbxl4	0.929	http://api.brain-map.org/grid_data/download/68637509?include=energy
Fhl1	0.929	http://api.brain-map.org/grid_data/download/2218?include=energy
Syngr3	0.929	http://api.brain-map.org/grid_data/download/1860?include=energy
Tsc22d1	0.929	http://api.brain-map.org/grid_data/download/1051?include=energy
Nudcd1	0.929	http://api.brain-map.org/grid_data/download/75831738?include=energy
4930435H24Rik	0.929	http://api.brain-map.org/grid_data/download/71891772?include=energy
Ap2a2	0.929	http://api.brain-map.org/grid_data/download/74819476?include=energy
Itfg3	0.929	http://api.brain-map.org/grid_data/download/69838432?include=energy
Atf7ip	0.929	http://api.brain-map.org/grid_data/download/68844331?include=energy
Plcd4	0.929	http://api.brain-map.org/grid_data/download/100144463?include=energy
4933440M02Rik	0.929	http://api.brain-map.org/grid_data/download/74821798?include=energy
1500010J02Rik	0.929	http://api.brain-map.org/grid_data/download/70813263?include=energy
Tmod1	0.929	http://api.brain-map.org/grid_data/download/75988526?include=energy
Zcchc7	0.929	http://api.brain-map.org/grid_data/download/71158306?include=energy
Cx3cr1	0.929	http://api.brain-map.org/grid_data/download/1605?include=energy
Apoc3	0.929	http://api.brain-map.org/grid_data/download/68341985?include=energy
Prpf6	0.928	http://api.brain-map.org/grid_data/download/67850946?include=energy
Ccdc28a	0.928	http://api.brain-map.org/grid_data/download/68158074?include=energy
Med27	0.928	http://api.brain-map.org/grid_data/download/583857?include=energy
Tst	0.928	http://api.brain-map.org/grid_data/download/70192111?include=energy
E030011O05Rik	0.928	http://api.brain-map.org/grid_data/download/70238961?include=energy
Tcf21	0.928	http://api.brain-map.org/grid_data/download/75831748?include=energy
Selplg	0.928	http://api.brain-map.org/grid_data/download/74988637?include=energy
Wbscr27	0.928	http://api.brain-map.org/grid_data/download/76135528?include=energy
Sdhc	0.928	http://api.brain-map.org/grid_data/download/68269302?include=energy
H47	0.928	http://api.brain-map.org/grid_data/download/69838758?include=energy
Fgf16	0.928	http://api.brain-map.org/grid_data/download/70724726?include=energy
E330014M11Rik*	0.928	http://api.brain-map.org/grid_data/download/77280559?include=energy
Ipp	0.928	http://api.brain-map.org/grid_data/download/69735514?include=energy
4930590J08Rik	0.928	http://api.brain-map.org/grid_data/download/69442416?include=energy
Gar1	0.928	http://api.brain-map.org/grid_data/download/71489802?include=energy
Plk3	0.928	http://api.brain-map.org/grid_data/download/71724563?include=energy
1700034O15Rik	0.928	http://api.brain-map.org/grid_data/download/69113716?include=energy
Tmtc3	0.928	http://api.brain-map.org/grid_data/download/69527752?include=energy
AI118078	0.928	http://api.brain-map.org/grid_data/download/69524479?include=energy
Dync1li1	0.928	http://api.brain-map.org/grid_data/download/75774684?include=energy
Slc25a23	0.928	http://api.brain-map.org/grid_data/download/71280844?include=energy
Acmsd	0.928	http://api.brain-map.org/grid_data/download/74431134?include=energy
Mtfmt	0.928	http://api.brain-map.org/grid_data/download/76097727?include=energy
Rfc5	0.928	http://api.brain-map.org/grid_data/download/74882798?include=energy
2310043J07Rik	0.928	http://api.brain-map.org/grid_data/download/72283761?include=energy
Pola1	0.928	http://api.brain-map.org/grid_data/download/75084100?include=energy
Man1b1	0.928	http://api.brain-map.org/grid_data/download/69671815?include=energy
Akr7a5	0.928	http://api.brain-map.org/grid_data/download/68498307?include=energy
Nicn1	0.928	http://api.brain-map.org/grid_data/download/75773512?include=energy
Kif21a	0.928	http://api.brain-map.org/grid_data/download/70919991?include=energy
Rpl37a	0.928	http://api.brain-map.org/grid_data/download/69818311?include=energy
A430107O13Rik	0.928	http://api.brain-map.org/grid_data/download/69448674?include=energy
Ank	0.928	http://api.brain-map.org/grid_data/download/75934916?include=energy
Vwa1	0.928	http://api.brain-map.org/grid_data/download/72007936?include=energy
1700093J21Rik	0.928	http://api.brain-map.org/grid_data/download/71232604?include=energy
Cacna1g	0.928	http://api.brain-map.org/grid_data/download/71587822?include=energy
Tsks	0.928	http://api.brain-map.org/grid_data/download/69887355?include=energy
Cldn23	0.928	http://api.brain-map.org/grid_data/download/71627833?include=energy
Nasp	0.928	http://api.brain-map.org/grid_data/download/70474779?include=energy
Aldh6a1	0.928	http://api.brain-map.org/grid_data/download/68445059?include=energy
Tmprss13	0.928	http://api.brain-map.org/grid_data/download/76080982?include=energy
Kcns3	0.928	http://api.brain-map.org/grid_data/download/77371817?include=energy
Eci1	0.928	http://api.brain-map.org/grid_data/download/68498741?include=energy
LOC226990	0.928	http://api.brain-map.org/grid_data/download/74514049?include=energy
Rce1	0.928	http://api.brain-map.org/grid_data/download/69079991?include=energy
Eif3k	0.928	http://api.brain-map.org/grid_data/download/68910971?include=energy
Megf6	0.928	http://api.brain-map.org/grid_data/download/68162266?include=energy
Themis	0.928	http://api.brain-map.org/grid_data/download/70239063?include=energy
Gemin8	0.928	http://api.brain-map.org/grid_data/download/70445628?include=energy
Celf2	0.928	http://api.brain-map.org/grid_data/download/70429202?include=energy
Mfsd2a	0.928	http://api.brain-map.org/grid_data/download/77280315?include=energy
Olfml3	0.928	http://api.brain-map.org/grid_data/download/77280333?include=energy
Dlg4	0.928	http://api.brain-map.org/grid_data/download/72109445?include=energy
1500002O20Rik	0.928	http://api.brain-map.org/grid_data/download/1954?include=energy
Dcun1d5	0.928	http://api.brain-map.org/grid_data/download/70430714?include=energy
Kcnq3	0.928	http://api.brain-map.org/grid_data/download/70562114?include=energy
LOC432426	0.928	http://api.brain-map.org/grid_data/download/70608490?include=energy
mCG146432	0.928	http://api.brain-map.org/grid_data/download/81657667?include=energy
Vmn1r71	0.928	http://api.brain-map.org/grid_data/download/73573307?include=energy
Srek1	0.928	http://api.brain-map.org/grid_data/download/69873636?include=energy
Hsd3b4	0.928	http://api.brain-map.org/grid_data/download/69059975?include=energy
Nucks1	0.928	http://api.brain-map.org/grid_data/download/68162273?include=energy
Sumo3	0.928	http://api.brain-map.org/grid_data/download/77924456?include=energy
Aagab	0.928	http://api.brain-map.org/grid_data/download/69169612?include=energy
Chpf2	0.928	http://api.brain-map.org/grid_data/download/69012822?include=energy
Med30	0.928	http://api.brain-map.org/grid_data/download/248329?include=energy
Antxrl	0.928	http://api.brain-map.org/grid_data/download/71569090?include=energy
Clec4d	0.928	http://api.brain-map.org/grid_data/download/71764721?include=energy
Scn8a	0.928	http://api.brain-map.org/grid_data/download/69863261?include=energy
Tagap	0.927	http://api.brain-map.org/grid_data/download/68162263?include=energy
Exoc3l	0.927	http://api.brain-map.org/grid_data/download/71496264?include=energy
Gm3228	0.927	http://api.brain-map.org/grid_data/download/74736117?include=energy
Slc1a1	0.927	http://api.brain-map.org/grid_data/download/71836888?include=energy
Sdf2	0.927	http://api.brain-map.org/grid_data/download/69102926?include=energy
Hibch	0.927	http://api.brain-map.org/grid_data/download/69095951?include=energy
Tbcb	0.927	http://api.brain-map.org/grid_data/download/512765?include=energy
Kcna1	0.927	http://api.brain-map.org/grid_data/download/73994714?include=energy
Raly	0.927	http://api.brain-map.org/grid_data/download/67978479?include=energy
Npr2	0.927	http://api.brain-map.org/grid_data/download/68845466?include=energy
Txnl4a	0.927	http://api.brain-map.org/grid_data/download/68443292?include=energy
Caps2	0.927	http://api.brain-map.org/grid_data/download/75988344?include=energy
LOC546378	0.927	http://api.brain-map.org/grid_data/download/71495704?include=energy
Slc27a4	0.927	http://api.brain-map.org/grid_data/download/1920?include=energy
Neurog3	0.927	http://api.brain-map.org/grid_data/download/77464994?include=energy
Zfp266	0.927	http://api.brain-map.org/grid_data/download/70227790?include=energy
Usp16	0.927	http://api.brain-map.org/grid_data/download/1886?include=energy
Ms4a2	0.927	http://api.brain-map.org/grid_data/download/74562254?include=energy
D130051D11Rik	0.927	http://api.brain-map.org/grid_data/download/74509470?include=energy
4933435G04Rik	0.927	http://api.brain-map.org/grid_data/download/74265255?include=energy
Tbc1d2b	0.927	http://api.brain-map.org/grid_data/download/69837527?include=energy
1110001J03Rik	0.927	http://api.brain-map.org/grid_data/download/69116566?include=energy
Impad1	0.927	http://api.brain-map.org/grid_data/download/68924859?include=energy
Pdpk1	0.927	http://api.brain-map.org/grid_data/download/69782957?include=energy
Thsd7b	0.927	http://api.brain-map.org/grid_data/download/71358617?include=energy
Ppl	0.927	http://api.brain-map.org/grid_data/download/69028605?include=energy
Slc39a3	0.927	http://api.brain-map.org/grid_data/download/2619?include=energy
Gnl1	0.927	http://api.brain-map.org/grid_data/download/70918922?include=energy
Tmem150a	0.927	http://api.brain-map.org/grid_data/download/68499063?include=energy
Prmt1	0.927	http://api.brain-map.org/grid_data/download/75695659?include=energy
1110002B05Rik	0.927	http://api.brain-map.org/grid_data/download/72081396?include=energy
E330023G01	0.927	http://api.brain-map.org/grid_data/download/70565524?include=energy
Supt6h	0.927	http://api.brain-map.org/grid_data/download/81600156?include=energy
Mecp2	0.927	http://api.brain-map.org/grid_data/download/79908808?include=energy
Emilin2	0.927	http://api.brain-map.org/grid_data/download/68666941?include=energy
Unc13a	0.927	http://api.brain-map.org/grid_data/download/70785218?include=energy
1700030K09Rik	0.927	http://api.brain-map.org/grid_data/download/76081930?include=energy
Itgb5	0.927	http://api.brain-map.org/grid_data/download/71920512?include=energy
Slc5a8	0.927	http://api.brain-map.org/grid_data/download/71763595?include=energy
Epha10	0.927	http://api.brain-map.org/grid_data/download/73930823?include=energy
Zfp707	0.927	http://api.brain-map.org/grid_data/download/69353278?include=energy
Galm	0.927	http://api.brain-map.org/grid_data/download/2249?include=energy
Rps19	0.927	http://api.brain-map.org/grid_data/download/72081492?include=energy
A430079D01Rik*	0.927	http://api.brain-map.org/grid_data/download/73808564?include=energy
Chd2	0.927	http://api.brain-map.org/grid_data/download/255?include=energy
Gm1302	0.927	http://api.brain-map.org/grid_data/download/74735313?include=energy
B3gat3	0.927	http://api.brain-map.org/grid_data/download/71283674?include=energy
Nr2c1	0.926	http://api.brain-map.org/grid_data/download/77455145?include=energy
Aoc3	0.926	http://api.brain-map.org/grid_data/download/71495777?include=energy
A530099J19Rik	0.926	http://api.brain-map.org/grid_data/download/69236754?include=energy
Gdnf	0.926	http://api.brain-map.org/grid_data/download/112647321?include=energy
Psma5	0.926	http://api.brain-map.org/grid_data/download/68269568?include=energy
Olfr272	0.926	http://api.brain-map.org/grid_data/download/74720946?include=energy
C1qc	0.926	http://api.brain-map.org/grid_data/download/77340474?include=energy
Arrb1	0.926	http://api.brain-map.org/grid_data/download/70305395?include=energy
Csrnp1	0.926	http://api.brain-map.org/grid_data/download/68667389?include=energy
1700018C11Rik	0.926	http://api.brain-map.org/grid_data/download/75851027?include=energy
Satl1	0.926	http://api.brain-map.org/grid_data/download/70218103?include=energy
Sec14l2	0.926	http://api.brain-map.org/grid_data/download/71325450?include=energy
Laptm4b	0.926	http://api.brain-map.org/grid_data/download/75651225?include=energy
Ppan	0.926	http://api.brain-map.org/grid_data/download/75774644?include=energy
LOC435344	0.926	http://api.brain-map.org/grid_data/download/71022469?include=energy
Pdcd1lg2	0.926	http://api.brain-map.org/grid_data/download/69782903?include=energy
Tff3	0.926	http://api.brain-map.org/grid_data/download/80514026?include=energy
Rassf10	0.926	http://api.brain-map.org/grid_data/download/69407624?include=energy
Ccdc92	0.926	http://api.brain-map.org/grid_data/download/74658111?include=energy
Gm4922	0.926	http://api.brain-map.org/grid_data/download/70305048?include=energy
Skint2	0.926	http://api.brain-map.org/grid_data/download/69262295?include=energy
Phka2	0.926	http://api.brain-map.org/grid_data/download/68667264?include=energy
Ticam2	0.926	http://api.brain-map.org/grid_data/download/70302485?include=energy
Slco6d1	0.926	http://api.brain-map.org/grid_data/download/71833040?include=energy
Mrpl34	0.926	http://api.brain-map.org/grid_data/download/69262242?include=energy
Bag5	0.926	http://api.brain-map.org/grid_data/download/70525794?include=energy
Acat2	0.926	http://api.brain-map.org/grid_data/download/77869798?include=energy
Atp10b	0.926	http://api.brain-map.org/grid_data/download/73635826?include=energy
Limd1	0.926	http://api.brain-map.org/grid_data/download/68843877?include=energy
Scn3a	0.926	http://api.brain-map.org/grid_data/download/70785043?include=energy
Pou4f2	0.926	http://api.brain-map.org/grid_data/download/71325342?include=energy
Ttc8	0.926	http://api.brain-map.org/grid_data/download/634462?include=energy
1190005I06Rik	0.926	http://api.brain-map.org/grid_data/download/68522472?include=energy
Slc24a1	0.926	http://api.brain-map.org/grid_data/download/78633998?include=energy
Trip6	0.926	http://api.brain-map.org/grid_data/download/71991846?include=energy
Rab42-ps	0.926	http://api.brain-map.org/grid_data/download/69446953?include=energy
D10Wsu52e	0.926	http://api.brain-map.org/grid_data/download/532578?include=energy
Dok2	0.926	http://api.brain-map.org/grid_data/download/2155?include=energy
9830166N24Rik*	0.926	http://api.brain-map.org/grid_data/download/75043150?include=energy
Eltd1	0.926	http://api.brain-map.org/grid_data/download/79360259?include=energy
Wdr8	0.926	http://api.brain-map.org/grid_data/download/75042258?include=energy
4930555I21Rik	0.926	http://api.brain-map.org/grid_data/download/70218366?include=energy
Ranbp10	0.926	http://api.brain-map.org/grid_data/download/71612185?include=energy
Nlrp2	0.926	http://api.brain-map.org/grid_data/download/73818829?include=energy
Srm	0.926	http://api.brain-map.org/grid_data/download/75041563?include=energy
Depdc5	0.926	http://api.brain-map.org/grid_data/download/79906725?include=energy
Rnase1	0.926	http://api.brain-map.org/grid_data/download/68522503?include=energy
Cdk8	0.926	http://api.brain-map.org/grid_data/download/75147769?include=energy
Trim54	0.926	http://api.brain-map.org/grid_data/download/71570742?include=energy
Ferd3l	0.926	http://api.brain-map.org/grid_data/download/81657659?include=energy
Impa1	0.926	http://api.brain-map.org/grid_data/download/74641325?include=energy
Serpina10	0.926	http://api.brain-map.org/grid_data/download/68638065?include=energy
Csf3r	0.926	http://api.brain-map.org/grid_data/download/77798995?include=energy
Smg5	0.926	http://api.brain-map.org/grid_data/download/67792184?include=energy
Pip4k2b	0.926	http://api.brain-map.org/grid_data/download/69012687?include=energy
Ldha	0.926	http://api.brain-map.org/grid_data/download/69736076?include=energy
Spon1	0.926	http://api.brain-map.org/grid_data/download/71836797?include=energy
Abhd16b	0.926	http://api.brain-map.org/grid_data/download/69116322?include=energy
Mab21l2	0.926	http://api.brain-map.org/grid_data/download/71381070?include=energy
1600025M17Rik	0.926	http://api.brain-map.org/grid_data/download/74581253?include=energy
Syngr1	0.926	http://api.brain-map.org/grid_data/download/70303719?include=energy
Got1l1	0.926	http://api.brain-map.org/grid_data/download/75831781?include=energy
Rer1	0.926	http://api.brain-map.org/grid_data/download/323586?include=energy
Haus4	0.926	http://api.brain-map.org/grid_data/download/67979045?include=energy
Cept1	0.926	http://api.brain-map.org/grid_data/download/68158073?include=energy
Mrps21	0.926	http://api.brain-map.org/grid_data/download/68744803?include=energy
Pea15a	0.926	http://api.brain-map.org/grid_data/download/70928292?include=energy
Blcap	0.926	http://api.brain-map.org/grid_data/download/71016617?include=energy
Olfr1106	0.926	http://api.brain-map.org/grid_data/download/74720603?include=energy

Pttg1	0.926	http://api.brain-map.org/grid_data/download/293036?include=energy
Spc24	0.926	http://api.brain-map.org/grid_data/download/100144879?include=energy
2310016G11Rik	0.925	http://api.brain-map.org/grid_data/download/74749238?include=energy
Pcyt1a	0.925	http://api.brain-map.org/grid_data/download/73731331?include=energy
Dok4	0.925	http://api.brain-map.org/grid_data/download/1486?include=energy
G830022E06Rik*	0.925	http://api.brain-map.org/grid_data/download/73716758?include=energy
Dlg1	0.925	http://api.brain-map.org/grid_data/download/68924745?include=energy
Aurkaip1	0.925	http://api.brain-map.org/grid_data/download/81599658?include=energy
D630014O11Rik	0.925	http://api.brain-map.org/grid_data/download/75080645?include=energy
Cndp2	0.925	http://api.brain-map.org/grid_data/download/68442967?include=energy
Amn	0.925	http://api.brain-map.org/grid_data/download/71487019?include=energy
Pemt	0.925	http://api.brain-map.org/grid_data/download/79756283?include=energy
Th	0.925	http://api.brain-map.org/grid_data/download/1056?include=energy
Zfp526	0.925	http://api.brain-map.org/grid_data/download/69407146?include=energy
Cramp1l	0.925	http://api.brain-map.org/grid_data/download/69028545?include=energy
Tra2b	0.925	http://api.brain-map.org/grid_data/download/75860985?include=energy
Fam19a4	0.925	http://api.brain-map.org/grid_data/download/72472752?include=energy
Slc35a3	0.925	http://api.brain-map.org/grid_data/download/2609?include=energy
Casp8	0.925	http://api.brain-map.org/grid_data/download/68193061?include=energy
Pigt	0.925	http://api.brain-map.org/grid_data/download/1810?include=energy
Tbx19	0.925	http://api.brain-map.org/grid_data/download/70593394?include=energy
Uts2d	0.925	http://api.brain-map.org/grid_data/download/75144621?include=energy
Brd4	0.925	http://api.brain-map.org/grid_data/download/67787362?include=energy
Atp6v1d	0.925	http://api.brain-map.org/grid_data/download/74047915?include=energy
Olfr735	0.925	http://api.brain-map.org/grid_data/download/74734920?include=energy
Upf2	0.925	http://api.brain-map.org/grid_data/download/70194582?include=energy
Vegfc	0.925	http://api.brain-map.org/grid_data/download/71378613?include=energy
Svop	0.925	http://api.brain-map.org/grid_data/download/69262121?include=energy
Ddx26b	0.925	http://api.brain-map.org/grid_data/download/75988610?include=energy
Atl1	0.925	http://api.brain-map.org/grid_data/download/68844044?include=energy
Psmc3ip	0.925	http://api.brain-map.org/grid_data/download/68445638?include=energy
Nr2c2	0.925	http://api.brain-map.org/grid_data/download/70593328?include=energy
5730469M10Rik	0.925	http://api.brain-map.org/grid_data/download/70436739?include=energy
Zfp292	0.925	http://api.brain-map.org/grid_data/download/72076689?include=energy
AU040320	0.925	http://api.brain-map.org/grid_data/download/532672?include=energy
Crbn	0.925	http://api.brain-map.org/grid_data/download/69608040?include=energy
Wscd2	0.925	http://api.brain-map.org/grid_data/download/69531031?include=energy
2810433D01Rik	0.925	http://api.brain-map.org/grid_data/download/73716590?include=energy
D030022P06Rik	0.925	http://api.brain-map.org/grid_data/download/70787630?include=energy
Bhlhe40	0.925	http://api.brain-map.org/grid_data/download/356488?include=energy
Dazl	0.925	http://api.brain-map.org/grid_data/download/71496411?include=energy
Mei4	0.925	http://api.brain-map.org/grid_data/download/70717359?include=energy
Gm20554	0.925	http://api.brain-map.org/grid_data/download/73834433?include=energy
Hspbp1	0.925	http://api.brain-map.org/grid_data/download/75774674?include=energy
BC018507	0.925	http://api.brain-map.org/grid_data/download/393248?include=energy
Kcnip2	0.925	http://api.brain-map.org/grid_data/download/81659533?include=energy
Ncrna00085	0.925	http://api.brain-map.org/grid_data/download/74580038?include=energy
Ikbkb	0.925	http://api.brain-map.org/grid_data/download/1750?include=energy
Angel2	0.925	http://api.brain-map.org/grid_data/download/67767464?include=energy
Tmem132c	0.925	http://api.brain-map.org/grid_data/download/71152611?include=energy
Capza3	0.925	http://api.brain-map.org/grid_data/download/75851043?include=energy
D730001G18Rik	0.925	http://api.brain-map.org/grid_data/download/71021166?include=energy
F630008L08Rik*	0.925	http://api.brain-map.org/grid_data/download/73748821?include=energy
Gjd2	0.925	http://api.brain-map.org/grid_data/download/71836902?include=energy
Arl4d	0.925	http://api.brain-map.org/grid_data/download/73817442?include=energy
Acot6	0.925	http://api.brain-map.org/grid_data/download/68076545?include=energy
Tmem206	0.925	http://api.brain-map.org/grid_data/download/75774106?include=energy
Rac1	0.925	http://api.brain-map.org/grid_data/download/68191504?include=energy
Dmc1	0.925	http://api.brain-map.org/grid_data/download/71496447?include=energy
1700052N19Rik	0.925	http://api.brain-map.org/grid_data/download/68076528?include=energy
Zfp488	0.925	http://api.brain-map.org/grid_data/download/70300061?include=energy
Npepps	0.925	http://api.brain-map.org/grid_data/download/844?include=energy
Avpr1a	0.925	http://api.brain-map.org/grid_data/download/74641316?include=energy
A330041J22Rik	0.925	http://api.brain-map.org/grid_data/download/69448020?include=energy
Setd2	0.925	http://api.brain-map.org/grid_data/download/69529053?include=energy
Krt84	0.925	http://api.brain-map.org/grid_data/download/71530001?include=energy
Tsn	0.925	http://api.brain-map.org/grid_data/download/532641?include=energy
Krt33a	0.925	http://api.brain-map.org/grid_data/download/68545300?include=energy
Ubqln4	0.925	http://api.brain-map.org/grid_data/download/70445574?include=energy
Lasp1	0.925	http://api.brain-map.org/grid_data/download/512991?include=energy
Pomp	0.925	http://api.brain-map.org/grid_data/download/75774685?include=energy
Krtap14	0.925	http://api.brain-map.org/grid_data/download/69735968?include=energy
Thap1	0.925	http://api.brain-map.org/grid_data/download/44?include=energy
Scrg1	0.925	http://api.brain-map.org/grid_data/download/71924331?include=energy
1110057K04Rik	0.925	http://api.brain-map.org/grid_data/download/69262259?include=energy
Mbl1	0.924	http://api.brain-map.org/grid_data/download/75827131?include=energy
LOC435758	0.924	http://api.brain-map.org/grid_data/download/71022579?include=energy
Dspp	0.924	http://api.brain-map.org/grid_data/download/75988390?include=energy
Gm1078	0.924	http://api.brain-map.org/grid_data/download/70299971?include=energy
Exoc3	0.924	http://api.brain-map.org/grid_data/download/532695?include=energy
Rab22a	0.924	http://api.brain-map.org/grid_data/download/71608432?include=energy
1700027D21Rik	0.924	http://api.brain-map.org/grid_data/download/71919631?include=energy
Vmn1r231	0.924	http://api.brain-map.org/grid_data/download/69442222?include=energy
Gm10549	0.924	http://api.brain-map.org/grid_data/download/74579443?include=energy
Tmem98	0.924	http://api.brain-map.org/grid_data/download/576427?include=energy
Avil	0.924	http://api.brain-map.org/grid_data/download/72340234?include=energy
LOC433959	0.924	http://api.brain-map.org/grid_data/download/70787164?include=energy
Defb50	0.924	http://api.brain-map.org/grid_data/download/73513705?include=energy
Olfr1158	0.924	http://api.brain-map.org/grid_data/download/74720615?include=energy
Prss30	0.924	http://api.brain-map.org/grid_data/download/513230?include=energy
Exosc4	0.924	http://api.brain-map.org/grid_data/download/68193052?include=energy
Tmem165	0.924	http://api.brain-map.org/grid_data/download/70191979?include=energy
Srd5a1	0.924	http://api.brain-map.org/grid_data/download/76081037?include=energy
Tmem14c	0.924	http://api.brain-map.org/grid_data/download/68151363?include=energy
Ahsa1	0.924	http://api.brain-map.org/grid_data/download/68161466?include=energy
Epm2aip1	0.924	http://api.brain-map.org/grid_data/download/583840?include=energy
Palm	0.924	http://api.brain-map.org/grid_data/download/69059956?include=energy
Vps28	0.924	http://api.brain-map.org/grid_data/download/68321662?include=energy
Pgm2	0.924	http://api.brain-map.org/grid_data/download/79536616?include=energy
R3hdm2	0.924	http://api.brain-map.org/grid_data/download/69353203?include=energy
LOC546759	0.924	http://api.brain-map.org/grid_data/download/74720525?include=energy
2310001K24Rik	0.924	http://api.brain-map.org/grid_data/download/74359359?include=energy
A530020G20Rik	0.924	http://api.brain-map.org/grid_data/download/70228472?include=energy
C230009H10Rik	0.924	http://api.brain-map.org/grid_data/download/72472761?include=energy
Tube1	0.924	http://api.brain-map.org/grid_data/download/69835622?include=energy
BC067074	0.924	http://api.brain-map.org/grid_data/download/69529238?include=energy
Ndufa3	0.924	http://api.brain-map.org/grid_data/download/73716566?include=energy
Pelp1	0.924	http://api.brain-map.org/grid_data/download/69015518?include=energy
9530080O11Rik	0.924	http://api.brain-map.org/grid_data/download/73606630?include=energy
Tk2	0.924	http://api.brain-map.org/grid_data/download/75042260?include=energy
Sntg2	0.924	http://api.brain-map.org/grid_data/download/69885600?include=energy
Inhbe	0.924	http://api.brain-map.org/grid_data/download/80341111?include=energy
Map2k4	0.924	http://api.brain-map.org/grid_data/download/276073?include=energy
Tarm1	0.924	http://api.brain-map.org/grid_data/download/71764667?include=energy
Cntnap2	0.924	http://api.brain-map.org/grid_data/download/544712?include=energy
Sh2d6	0.924	http://api.brain-map.org/grid_data/download/74580763?include=energy
E330017A01Rik	0.924	http://api.brain-map.org/grid_data/download/71809542?include=energy
Psmc5	0.924	http://api.brain-map.org/grid_data/download/67815945?include=energy
Zpbp	0.924	http://api.brain-map.org/grid_data/download/74581362?include=energy
Ldoc1	0.924	http://api.brain-map.org/grid_data/download/68667736?include=energy
Spic	0.924	http://api.brain-map.org/grid_data/download/74578299?include=energy
Morn3	0.924	http://api.brain-map.org/grid_data/download/68342135?include=energy
Trim66	0.924	http://api.brain-map.org/grid_data/download/69289273?include=energy
Mapkapk5	0.924	http://api.brain-map.org/grid_data/download/71610881?include=energy
Haus6	0.924	http://api.brain-map.org/grid_data/download/77455147?include=energy
Cercam	0.924	http://api.brain-map.org/grid_data/download/69540733?include=energy
Akap14	0.924	http://api.brain-map.org/grid_data/download/72472473?include=energy
1700001L19Rik	0.924	http://api.brain-map.org/grid_data/download/69353511?include=energy
Gm6999	0.924	http://api.brain-map.org/grid_data/download/73906095?include=energy
Zim2	0.924	http://api.brain-map.org/grid_data/download/76135811?include=energy
Pfn4	0.924	http://api.brain-map.org/grid_data/download/73716602?include=energy
Gnb3	0.924	http://api.brain-map.org/grid_data/download/71789771?include=energy
Tmprss12	0.924	http://api.brain-map.org/grid_data/download/71891796?include=energy
Fam69a	0.924	http://api.brain-map.org/grid_data/download/70609233?include=energy
Sdhd	0.924	http://api.brain-map.org/grid_data/download/69873463?include=energy
Golt1b	0.924	http://api.brain-map.org/grid_data/download/75198226?include=energy
Rnasek	0.924	http://api.brain-map.org/grid_data/download/75934529?include=energy
Trim52	0.924	http://api.brain-map.org/grid_data/download/70205626?include=energy
Dpf1	0.924	http://api.brain-map.org/grid_data/download/69104136?include=energy
Ankk1	0.924	http://api.brain-map.org/grid_data/download/69447858?include=energy
Hspb6	0.924	http://api.brain-map.org/grid_data/download/74882616?include=energy
Aspm	0.924	http://api.brain-map.org/grid_data/download/69540284?include=energy
Akap6	0.924	http://api.brain-map.org/grid_data/download/70230902?include=energy
4933402N03Rik	0.924	http://api.brain-map.org/grid_data/download/72182942?include=energy
Lrrtm2	0.924	http://api.brain-map.org/grid_data/download/69736292?include=energy
Klhl22	0.924	http://api.brain-map.org/grid_data/download/69079996?include=energy
Uspl1	0.924	http://api.brain-map.org/grid_data/download/71213029?include=energy
Snrpg	0.924	http://api.brain-map.org/grid_data/download/68744092?include=energy
Pdzd4	0.924	http://api.brain-map.org/grid_data/download/68844313?include=energy
Dgkq	0.924	http://api.brain-map.org/grid_data/download/71021168?include=energy
Ptdss2	0.923	http://api.brain-map.org/grid_data/download/68745209?include=energy
Taf13	0.923	http://api.brain-map.org/grid_data/download/2655?include=energy
Ccno	0.923	http://api.brain-map.org/grid_data/download/69540254?include=energy
Fscn1	0.923	http://api.brain-map.org/grid_data/download/74658080?include=energy
Upk2	0.923	http://api.brain-map.org/grid_data/download/70194594?include=energy
Prx	0.923	http://api.brain-map.org/grid_data/download/71210846?include=energy
Impdh1	0.923	http://api.brain-map.org/grid_data/download/69105744?include=energy
Celsr1	0.923	http://api.brain-map.org/grid_data/download/71307238?include=energy
F730035P03Rik	0.923	http://api.brain-map.org/grid_data/download/70276777?include=energy
Gdi2	0.923	http://api.brain-map.org/grid_data/download/491?include=energy
Foxo3	0.923	http://api.brain-map.org/grid_data/download/73949752?include=energy
Ppil1	0.923	http://api.brain-map.org/grid_data/download/68844134?include=energy
Tceb2	0.923	http://api.brain-map.org/grid_data/download/74822929?include=energy
Rhbg	0.923	http://api.brain-map.org/grid_data/download/78634021?include=energy
Prkcb	0.923	http://api.brain-map.org/grid_data/download/72129247?include=energy
Wdr26	0.923	http://api.brain-map.org/grid_data/download/69169499?include=energy
3830431G21Rik	0.923	http://api.brain-map.org/grid_data/download/70610378?include=energy
Foxd3	0.923	http://api.brain-map.org/grid_data/download/1496?include=energy
Cypt4	0.923	http://api.brain-map.org/grid_data/download/69116805?include=energy
Krtap31-2	0.923	http://api.brain-map.org/grid_data/download/77620841?include=energy
Dctn6	0.923	http://api.brain-map.org/grid_data/download/323597?include=energy
Gnptg	0.923	http://api.brain-map.org/grid_data/download/74357658?include=energy
Apcs	0.923	http://api.brain-map.org/grid_data/download/69526737?include=energy
Gm15229	0.923	http://api.brain-map.org/grid_data/download/70595762?include=energy
Gm608	0.923	http://api.brain-map.org/grid_data/download/70295572?include=energy
LOC546107	0.923	http://api.brain-map.org/grid_data/download/73712804?include=energy
Ceacam10	0.923	http://api.brain-map.org/grid_data/download/80514149?include=energy
Tmtc1	0.923	http://api.brain-map.org/grid_data/download/73636154?include=energy
Zfp84	0.923	http://api.brain-map.org/grid_data/download/77620305?include=energy
A230034C19Rik*	0.923	http://api.brain-map.org/grid_data/download/73949526?include=energy
Tub	0.923	http://api.brain-map.org/grid_data/download/71764449?include=energy
1700065D16Rik	0.923	http://api.brain-map.org/grid_data/download/68797780?include=energy
Prl3c1	0.923	http://api.brain-map.org/grid_data/download/71891641?include=energy
Als2cr11	0.923	http://api.brain-map.org/grid_data/download/76003373?include=energy
Adcy3	0.923	http://api.brain-map.org/grid_data/download/75?include=energy
Cacnb1	0.923	http://api.brain-map.org/grid_data/download/73497107?include=energy
Dtnbp1	0.923	http://api.brain-map.org/grid_data/download/79588292?include=energy
Flot2	0.923	http://api.brain-map.org/grid_data/download/74511805?include=energy
Pcdh18	0.923	http://api.brain-map.org/grid_data/download/71358554?include=energy
Dgkk	0.923	http://api.brain-map.org/grid_data/download/100145312?include=energy
Tbc1d21	0.923	http://api.brain-map.org/grid_data/download/71920177?include=energy
Slc7a6os	0.923	http://api.brain-map.org/grid_data/download/68667001?include=energy
Ankrd13c	0.923	http://api.brain-map.org/grid_data/download/70813185?include=energy
LOC432614	0.923	http://api.brain-map.org/grid_data/download/70611120?include=energy
Trappc6a	0.923	http://api.brain-map.org/grid_data/download/68191252?include=energy
Fam164c	0.923	http://api.brain-map.org/grid_data/download/68745185?include=energy
1700008P20Rik	0.923	http://api.brain-map.org/grid_data/download/76081047?include=energy
Pate2	0.923	http://api.brain-map.org/grid_data/download/72471803?include=energy
Gramd3	0.923	http://api.brain-map.org/grid_data/download/74988591?include=energy
Papss2	0.923	http://api.brain-map.org/grid_data/download/71920480?include=energy
Atp6v1f	0.923	http://api.brain-map.org/grid_data/download/393251?include=energy
Ccdc108	0.923	http://api.brain-map.org/grid_data/download/71495891?include=energy
LOC433886	0.923	http://api.brain-map.org/grid_data/download/75931461?include=energy
Grm4	0.923	http://api.brain-map.org/grid_data/download/71247631?include=energy
Sfpi1	0.923	http://api.brain-map.org/grid_data/download/227535?include=energy
Zfp810	0.923	http://api.brain-map.org/grid_data/download/356856?include=energy
Tsc22d4	0.923	http://api.brain-map.org/grid_data/download/1464?include=energy
4921501E09Rik	0.923	http://api.brain-map.org/grid_data/download/73617570?include=energy
Mrpl28	0.923	http://api.brain-map.org/grid_data/download/68191414?include=energy
LOC435551	0.923	http://api.brain-map.org/grid_data/download/74743489?include=energy
Plxna1	0.923	http://api.brain-map.org/grid_data/download/69289721?include=energy
Atg4a	0.923	http://api.brain-map.org/grid_data/download/75988266?include=energy
Tmem97	0.923	http://api.brain-map.org/grid_data/download/68666511?include=energy
Rmnd1	0.923	http://api.brain-map.org/grid_data/download/77371805?include=energy
2510002D24Rik	0.922	http://api.brain-map.org/grid_data/download/70303803?include=energy
6530439I21	0.922	http://api.brain-map.org/grid_data/download/70305120?include=energy
Vps33a	0.922	http://api.brain-map.org/grid_data/download/68845365?include=energy
Bag1	0.922	http://api.brain-map.org/grid_data/download/75043147?include=energy
Csk	0.922	http://api.brain-map.org/grid_data/download/75651192?include=energy
Med16	0.922	http://api.brain-map.org/grid_data/download/69262255?include=energy
Ino80	0.922	http://api.brain-map.org/grid_data/download/68745143?include=energy
4930556L07Rik	0.922	http://api.brain-map.org/grid_data/download/70218372?include=energy
Kcnk15	0.922	http://api.brain-map.org/grid_data/download/70724864?include=energy
Fam115a	0.922	http://api.brain-map.org/grid_data/download/71211695?include=energy
B130040O20Rik	0.922	http://api.brain-map.org/grid_data/download/71495855?include=energy
Kptn	0.922	http://api.brain-map.org/grid_data/download/69080065?include=energy
Svs3a	0.922	http://api.brain-map.org/grid_data/download/71892436?include=energy
Trpv2	0.922	http://api.brain-map.org/grid_data/download/71763613?include=energy
Pcdhb20	0.922	http://api.brain-map.org/grid_data/download/2468?include=energy
Elovl4	0.922	http://api.brain-map.org/grid_data/download/69059903?include=energy
Col4a3	0.922	http://api.brain-map.org/grid_data/download/75988364?include=energy
Plxdc2	0.922	http://api.brain-map.org/grid_data/download/74425563?include=energy
Aqp4	0.922	http://api.brain-map.org/grid_data/download/108?include=energy
Fam65c	0.922	http://api.brain-map.org/grid_data/download/72283342?include=energy
Cpne1	0.922	http://api.brain-map.org/grid_data/download/74735307?include=energy
Alox15	0.922	http://api.brain-map.org/grid_data/download/75831772?include=energy
Cyp2a4	0.922	http://api.brain-map.org/grid_data/download/77465046?include=energy
Loxl2	0.922	http://api.brain-map.org/grid_data/download/71670486?include=energy
Rasa4	0.922	http://api.brain-map.org/grid_data/download/74425552?include=energy
Mesdc1	0.922	http://api.brain-map.org/grid_data/download/576423?include=energy
Slc25a2	0.922	http://api.brain-map.org/grid_data/download/70302105?include=energy
2310035K24Rik	0.922	http://api.brain-map.org/grid_data/download/69013137?include=energy
Ldlrad3	0.922	http://api.brain-map.org/grid_data/download/70230825?include=energy
Trrap	0.922	http://api.brain-map.org/grid_data/download/413773?include=energy
Psme1	0.922	http://api.brain-map.org/grid_data/download/76115739?include=energy
Yme1l1	0.922	http://api.brain-map.org/grid_data/download/356849?include=energy
Marcks	0.922	http://api.brain-map.org/grid_data/download/71487124?include=energy
F630043A04Rik	0.922	http://api.brain-map.org/grid_data/download/100144951?include=energy
Vipar	0.922	http://api.brain-map.org/grid_data/download/69080004?include=energy
Chst15	0.922	http://api.brain-map.org/grid_data/download/70613978?include=energy
Cpne3	0.922	http://api.brain-map.org/grid_data/download/74988745?include=energy
Rps27l	0.922	http://api.brain-map.org/grid_data/download/75831731?include=energy
Ap3b1	0.922	http://api.brain-map.org/grid_data/download/69059806?include=energy
Gpr146	0.922	http://api.brain-map.org/grid_data/download/2059?include=energy
1700006A11Rik	0.922	http://api.brain-map.org/grid_data/download/72471734?include=energy
Ift27	0.922	http://api.brain-map.org/grid_data/download/75695629?include=energy
Zfp865	0.922	http://api.brain-map.org/grid_data/download/71493795?include=energy
Sqle	0.922	http://api.brain-map.org/grid_data/download/70231996?include=energy
Hist3h2a	0.922	http://api.brain-map.org/grid_data/download/74583150?include=energy
Rnf11	0.922	http://api.brain-map.org/grid_data/download/1276?include=energy
Rbm19	0.922	http://api.brain-map.org/grid_data/download/71016585?include=energy
Tmem8	0.922	http://api.brain-map.org/grid_data/download/386026?include=energy
4930540G01Rik*	0.922	http://api.brain-map.org/grid_data/download/73906491?include=energy
Zbtb17	0.922	http://api.brain-map.org/grid_data/download/69290734?include=energy
Nrg3	0.922	http://api.brain-map.org/grid_data/download/71920454?include=energy
Trim60	0.922	http://api.brain-map.org/grid_data/download/70301061?include=energy
Ncoa2	0.922	http://api.brain-map.org/grid_data/download/68668090?include=energy

Neurog1	0.922	http://api.brain-map.org/grid_data/download/77465058?include=energy
Pvrl2	0.922	http://api.brain-map.org/grid_data/download/119697?include=energy
Cul5	0.922	http://api.brain-map.org/grid_data/download/69170192?include=energy
Twf1	0.922	http://api.brain-map.org/grid_data/download/80342061?include=energy
Mrs2	0.922	http://api.brain-map.org/grid_data/download/71020812?include=energy
Sys1	0.922	http://api.brain-map.org/grid_data/download/69117062?include=energy
Ino80c	0.921	http://api.brain-map.org/grid_data/download/69116775?include=energy
Dock6	0.921	http://api.brain-map.org/grid_data/download/75041548?include=energy
Zp2	0.921	http://api.brain-map.org/grid_data/download/79544870?include=energy
Aco1	0.921	http://api.brain-map.org/grid_data/download/76135819?include=energy
Olfr308	0.921	http://api.brain-map.org/grid_data/download/74720967?include=energy
Pyy	0.921	http://api.brain-map.org/grid_data/download/75214925?include=energy
5730494M16Rik	0.921	http://api.brain-map.org/grid_data/download/71147834?include=energy
Sugp2	0.921	http://api.brain-map.org/grid_data/download/69549629?include=energy
Ubap1	0.921	http://api.brain-map.org/grid_data/download/74957898?include=energy
Rraga	0.921	http://api.brain-map.org/grid_data/download/69133674?include=energy
Sept14	0.921	http://api.brain-map.org/grid_data/download/75934508?include=energy
Btnl9	0.921	http://api.brain-map.org/grid_data/download/75214736?include=energy
Cct6a	0.921	http://api.brain-map.org/grid_data/download/77792633?include=energy
Gpr107	0.921	http://api.brain-map.org/grid_data/download/70230849?include=energy
Bdkrb1	0.921	http://api.brain-map.org/grid_data/download/69236867?include=energy
Gbp1	0.921	http://api.brain-map.org/grid_data/download/77464908?include=energy
Pspn	0.921	http://api.brain-map.org/grid_data/download/71377826?include=energy
Cln5	0.921	http://api.brain-map.org/grid_data/download/69549012?include=energy
Sf3a3	0.921	http://api.brain-map.org/grid_data/download/392938?include=energy
Cacna2d3	0.921	http://api.brain-map.org/grid_data/download/79591395?include=energy
Alg2	0.921	http://api.brain-map.org/grid_data/download/73931383?include=energy
Zdhhc5	0.921	http://api.brain-map.org/grid_data/download/68341770?include=energy
Nt5c3l	0.921	http://api.brain-map.org/grid_data/download/70445640?include=energy
Robo2	0.921	http://api.brain-map.org/grid_data/download/75080998?include=energy
Ssr1	0.921	http://api.brain-map.org/grid_data/download/2631?include=energy
Serpinb12	0.921	http://api.brain-map.org/grid_data/download/75831718?include=energy
D19Bwg1357e	0.921	http://api.brain-map.org/grid_data/download/68667872?include=energy
Atp6v1b2	0.921	http://api.brain-map.org/grid_data/download/129?include=energy
LOC433311	0.921	http://api.brain-map.org/grid_data/download/74988757?include=energy
3110021N24Rik	0.921	http://api.brain-map.org/grid_data/download/74359407?include=energy
Gp2	0.921	http://api.brain-map.org/grid_data/download/71587763?include=energy
Ppp4r1l-ps	0.921	http://api.brain-map.org/grid_data/download/70231845?include=energy
Ddx60	0.921	http://api.brain-map.org/grid_data/download/71616812?include=energy
LOC434406	0.921	http://api.brain-map.org/grid_data/download/70725591?include=energy
Ppt2	0.921	http://api.brain-map.org/grid_data/download/70546396?include=energy
Nkd2	0.921	http://api.brain-map.org/grid_data/download/75042259?include=energy
1700017B05Rik	0.921	http://api.brain-map.org/grid_data/download/70201289?include=energy
Med11	0.921	http://api.brain-map.org/grid_data/download/76135645?include=energy
Hsbp1	0.921	http://api.brain-map.org/grid_data/download/74581381?include=energy
Rpp25	0.921	http://api.brain-map.org/grid_data/download/75146006?include=energy
Hax1	0.921	http://api.brain-map.org/grid_data/download/2303?include=energy
Rtl1	0.921	http://api.brain-map.org/grid_data/download/74988325?include=energy
Morf4l1	0.921	http://api.brain-map.org/grid_data/download/71763798?include=energy
Dhrs1	0.921	http://api.brain-map.org/grid_data/download/100146076?include=energy
Fut9	0.921	http://api.brain-map.org/grid_data/download/70303564?include=energy
1700027J07Rik	0.921	http://api.brain-map.org/grid_data/download/75934510?include=energy
Mgat2	0.921	http://api.brain-map.org/grid_data/download/74988614?include=energy
Catsperg2	0.921	http://api.brain-map.org/grid_data/download/71492880?include=energy
Cpt1c	0.921	http://api.brain-map.org/grid_data/download/2117?include=energy
Arhgap4	0.921	http://api.brain-map.org/grid_data/download/75988286?include=energy
Actn1	0.921	http://api.brain-map.org/grid_data/download/74514369?include=energy
Ccl24	0.921	http://api.brain-map.org/grid_data/download/76080994?include=energy
Dhdds	0.921	http://api.brain-map.org/grid_data/download/70612728?include=energy
Taf5	0.921	http://api.brain-map.org/grid_data/download/69290579?include=energy
Mfsd6	0.921	http://api.brain-map.org/grid_data/download/75651167?include=energy
Nktr	0.921	http://api.brain-map.org/grid_data/download/73423868?include=energy
3010033K07Rik	0.921	http://api.brain-map.org/grid_data/download/71378676?include=energy
Hsd17b14	0.921	http://api.brain-map.org/grid_data/download/71580232?include=energy
Pnliprp2	0.921	http://api.brain-map.org/grid_data/download/69816362?include=energy
Sdhaf2	0.921	http://api.brain-map.org/grid_data/download/71160322?include=energy
LOC434781	0.921	http://api.brain-map.org/grid_data/download/71764781?include=energy
Ndufa4l2	0.92	http://api.brain-map.org/grid_data/download/73716656?include=energy
Dsg1a	0.92	http://api.brain-map.org/grid_data/download/81654153?include=energy
9530019H20Rik	0.92	http://api.brain-map.org/grid_data/download/69446923?include=energy
Tmx3	0.92	http://api.brain-map.org/grid_data/download/69028360?include=energy
Dusp19	0.92	http://api.brain-map.org/grid_data/download/80341177?include=energy
1500003O03Rik	0.92	http://api.brain-map.org/grid_data/download/71656674?include=energy
Nxph1	0.92	http://api.brain-map.org/grid_data/download/75084479?include=energy
Prr13	0.92	http://api.brain-map.org/grid_data/download/69080490?include=energy
Hexa	0.92	http://api.brain-map.org/grid_data/download/71627751?include=energy
Fgf9	0.92	http://api.brain-map.org/grid_data/download/81654115?include=energy
Stat3	0.92	http://api.brain-map.org/grid_data/download/2637?include=energy
9130221F21Rik	0.92	http://api.brain-map.org/grid_data/download/71569127?include=energy
Zeb1	0.92	http://api.brain-map.org/grid_data/download/71380441?include=energy
Tspan12	0.92	http://api.brain-map.org/grid_data/download/73997153?include=energy
Yif1b	0.92	http://api.brain-map.org/grid_data/download/69446905?include=energy
Fam123b	0.92	http://api.brain-map.org/grid_data/download/74821580?include=energy
Ces2e	0.92	http://api.brain-map.org/grid_data/download/69838558?include=energy
Spata6	0.92	http://api.brain-map.org/grid_data/download/72002078?include=energy
Cnot1	0.92	http://api.brain-map.org/grid_data/download/68546440?include=energy
9530068E07Rik	0.92	http://api.brain-map.org/grid_data/download/74988509?include=energy
2610528E23Rik	0.92	http://api.brain-map.org/grid_data/download/68545953?include=energy
F630018C04Rik*	0.92	http://api.brain-map.org/grid_data/download/73748848?include=energy
Mcpt8	0.92	http://api.brain-map.org/grid_data/download/69752143?include=energy
Mrgprb1	0.92	http://api.brain-map.org/grid_data/download/70784642?include=energy
Scn5a	0.92	http://api.brain-map.org/grid_data/download/73907501?include=energy
Atg13	0.92	http://api.brain-map.org/grid_data/download/68158078?include=energy
2610002D18Rik	0.92	http://api.brain-map.org/grid_data/download/70203507?include=energy
Stap2	0.92	http://api.brain-map.org/grid_data/download/73512501?include=energy
Fam114a2	0.92	http://api.brain-map.org/grid_data/download/1992?include=energy
Sash3	0.92	http://api.brain-map.org/grid_data/download/69549604?include=energy
Nfe2l1	0.92	http://api.brain-map.org/grid_data/download/68924787?include=energy
Fam120a	0.92	http://api.brain-map.org/grid_data/download/75080620?include=energy
Ost4	0.92	http://api.brain-map.org/grid_data/download/631702?include=energy
Rundc2a	0.92	http://api.brain-map.org/grid_data/download/70300144?include=energy
Slc27a1	0.92	http://api.brain-map.org/grid_data/download/75651228?include=energy
Mdh1b	0.92	http://api.brain-map.org/grid_data/download/75862421?include=energy
Hspa4	0.92	http://api.brain-map.org/grid_data/download/632500?include=energy
Wipi2	0.92	http://api.brain-map.org/grid_data/download/68845436?include=energy
6720419K19Rik*	0.92	http://api.brain-map.org/grid_data/download/73927658?include=energy
Dsg3	0.92	http://api.brain-map.org/grid_data/download/100145358?include=energy
Tmem85	0.92	http://api.brain-map.org/grid_data/download/68798813?include=energy
Rgma	0.92	http://api.brain-map.org/grid_data/download/68160845?include=energy
Kif4	0.92	http://api.brain-map.org/grid_data/download/74514141?include=energy
Rph3a	0.92	http://api.brain-map.org/grid_data/download/71587899?include=energy
Gria4	0.92	http://api.brain-map.org/grid_data/download/71358628?include=energy
Ptprn2	0.92	http://api.brain-map.org/grid_data/download/69236592?include=energy
6720401G13Rik	0.92	http://api.brain-map.org/grid_data/download/74958576?include=energy
Tlk1	0.92	http://api.brain-map.org/grid_data/download/70302503?include=energy
Clec2e	0.92	http://api.brain-map.org/grid_data/download/70305616?include=energy
Odc1	0.92	http://api.brain-map.org/grid_data/download/74583117?include=energy
Nudt19	0.92	http://api.brain-map.org/grid_data/download/69609073?include=energy
1700007K13Rik	0.92	http://api.brain-map.org/grid_data/download/71492850?include=energy
Sh3bgrl	0.92	http://api.brain-map.org/grid_data/download/70439141?include=energy
Gm1968	0.92	http://api.brain-map.org/grid_data/download/74430972?include=energy
Zfyve27	0.92	http://api.brain-map.org/grid_data/download/75774663?include=energy
Lrrn2	0.92	http://api.brain-map.org/grid_data/download/74819328?include=energy
Zfp105	0.92	http://api.brain-map.org/grid_data/download/69549301?include=energy
Nup98	0.92	http://api.brain-map.org/grid_data/download/71656848?include=energy
Fbxw2	0.92	http://api.brain-map.org/grid_data/download/355882?include=energy
Cdh4	0.92	http://api.brain-map.org/grid_data/download/2083?include=energy
Apol9a	0.92	http://api.brain-map.org/grid_data/download/68269574?include=energy
A230051G13Rik	0.92	http://api.brain-map.org/grid_data/download/71493657?include=energy
Wdr24	0.92	http://api.brain-map.org/grid_data/download/68162262?include=energy
Cntn1	0.92	http://api.brain-map.org/grid_data/download/414?include=energy
Fam192a	0.919	http://api.brain-map.org/grid_data/download/583573?include=energy
Loxl3	0.919	http://api.brain-map.org/grid_data/download/86370?include=energy
Adam4	0.919	http://api.brain-map.org/grid_data/download/69514774?include=energy
Mif	0.919	http://api.brain-map.org/grid_data/download/2407?include=energy
Hes7	0.919	http://api.brain-map.org/grid_data/download/2289?include=energy
Vash1	0.919	http://api.brain-map.org/grid_data/download/69672671?include=energy
Mm.158495	0.919	http://api.brain-map.org/grid_data/download/71148244?include=energy
4933427E11Rik	0.919	http://api.brain-map.org/grid_data/download/69443226?include=energy
Commd7	0.919	http://api.brain-map.org/grid_data/download/70720255?include=energy
Lrrc47	0.919	http://api.brain-map.org/grid_data/download/71808747?include=energy
Fis1	0.919	http://api.brain-map.org/grid_data/download/686210?include=energy
Pdgfrl	0.919	http://api.brain-map.org/grid_data/download/69102776?include=energy
LOC546020	0.919	http://api.brain-map.org/grid_data/download/68795465?include=energy
Ubr3	0.919	http://api.brain-map.org/grid_data/download/74988721?include=energy
Tmcc3	0.919	http://api.brain-map.org/grid_data/download/67792206?include=energy
D130058B02Rik*	0.919	http://api.brain-map.org/grid_data/download/74076030?include=energy
Mrps5	0.919	http://api.brain-map.org/grid_data/download/678682?include=energy
I830002M09Rik*	0.919	http://api.brain-map.org/grid_data/download/73767220?include=energy
Vti1a	0.919	http://api.brain-map.org/grid_data/download/74641238?include=energy
A230052G05Rik	0.919	http://api.brain-map.org/grid_data/download/74431780?include=energy
Atxn3	0.919	http://api.brain-map.org/grid_data/download/71378330?include=energy
Gpnmb	0.919	http://api.brain-map.org/grid_data/download/74581367?include=energy
1700123I01Rik	0.919	http://api.brain-map.org/grid_data/download/71920249?include=energy
Rfx4	0.919	http://api.brain-map.org/grid_data/download/71249068?include=energy
Dennd4c	0.919	http://api.brain-map.org/grid_data/download/69353741?include=energy
Ncf1	0.919	http://api.brain-map.org/grid_data/download/70546251?include=energy
D830013O20Rik	0.919	http://api.brain-map.org/grid_data/download/70238752?include=energy
D230038L04Rik*	0.919	http://api.brain-map.org/grid_data/download/73834373?include=energy
Scfd2	0.919	http://api.brain-map.org/grid_data/download/69873439?include=energy
Mboat7	0.919	http://api.brain-map.org/grid_data/download/72081696?include=energy
Cenpl	0.919	http://api.brain-map.org/grid_data/download/69202920?include=energy
6330437B10Rik*	0.919	http://api.brain-map.org/grid_data/download/73906035?include=energy
Tmem65	0.919	http://api.brain-map.org/grid_data/download/73592537?include=energy
Ppp2cb	0.919	http://api.brain-map.org/grid_data/download/69080053?include=energy
Ppib	0.919	http://api.brain-map.org/grid_data/download/74580984?include=energy
Arhgap36	0.919	http://api.brain-map.org/grid_data/download/69352834?include=energy
Myo18a	0.919	http://api.brain-map.org/grid_data/download/69782001?include=energy
Sirpa	0.919	http://api.brain-map.org/grid_data/download/74583151?include=energy
D630005B22Rik	0.919	http://api.brain-map.org/grid_data/download/71809430?include=energy
Cox7c	0.919	http://api.brain-map.org/grid_data/download/70793911?include=energy
Tgfb2	0.919	http://api.brain-map.org/grid_data/download/70927304?include=energy
Rab6a	0.919	http://api.brain-map.org/grid_data/download/74047909?include=energy
C4bp	0.919	http://api.brain-map.org/grid_data/download/68301885?include=energy
Rars2	0.919	http://api.brain-map.org/grid_data/download/68444726?include=energy
Psmc4	0.919	http://api.brain-map.org/grid_data/download/513224?include=energy
Lrmp	0.919	http://api.brain-map.org/grid_data/download/74274684?include=energy
Slc22a8	0.919	http://api.brain-map.org/grid_data/download/74657924?include=energy
Pcid2	0.919	http://api.brain-map.org/grid_data/download/69448972?include=energy
Gm810	0.919	http://api.brain-map.org/grid_data/download/70298298?include=energy
Serp1	0.919	http://api.brain-map.org/grid_data/download/631720?include=energy
Ranbp17	0.919	http://api.brain-map.org/grid_data/download/69288267?include=energy
Fbln2	0.919	http://api.brain-map.org/grid_data/download/321208?include=energy
Mm.127759	0.919	http://api.brain-map.org/grid_data/download/71148052?include=energy
Hemt1	0.919	http://api.brain-map.org/grid_data/download/74581283?include=energy
Tfec	0.919	http://api.brain-map.org/grid_data/download/69816978?include=energy
Purg	0.919	http://api.brain-map.org/grid_data/download/74882788?include=energy
Ftsj2	0.919	http://api.brain-map.org/grid_data/download/75828197?include=energy
Cnpy3	0.919	http://api.brain-map.org/grid_data/download/70429483?include=energy
Gm1564	0.919	http://api.brain-map.org/grid_data/download/71529845?include=energy
Rarres1	0.919	http://api.brain-map.org/grid_data/download/75084131?include=energy
Scn3b	0.919	http://api.brain-map.org/grid_data/download/71064082?include=energy
Lcorl	0.919	http://api.brain-map.org/grid_data/download/71281141?include=energy
Tank	0.919	http://api.brain-map.org/grid_data/download/69549246?include=energy
Zdhhc11	0.919	http://api.brain-map.org/grid_data/download/71892155?include=energy
Cmtm3	0.919	http://api.brain-map.org/grid_data/download/2099?include=energy
Gp9	0.919	http://api.brain-map.org/grid_data/download/69734518?include=energy
2310079G19Rik	0.919	http://api.brain-map.org/grid_data/download/75990428?include=energy
Gm8098	0.919	http://api.brain-map.org/grid_data/download/73906343?include=energy
Mmp14	0.919	http://api.brain-map.org/grid_data/download/74658084?include=energy
Fgfr1	0.919	http://api.brain-map.org/grid_data/download/79588290?include=energy
LOC381742	0.919	http://api.brain-map.org/grid_data/download/74988756?include=energy
LOC433683	0.919	http://api.brain-map.org/grid_data/download/71991989?include=energy
6430709H04Rik	0.919	http://api.brain-map.org/grid_data/download/74750012?include=energy
Zgpat	0.919	http://api.brain-map.org/grid_data/download/671253?include=energy
Top1mt	0.919	http://api.brain-map.org/grid_data/download/70191949?include=energy
1700042G07Rik	0.919	http://api.brain-map.org/grid_data/download/70201450?include=energy
Gpank1	0.919	http://api.brain-map.org/grid_data/download/75831768?include=energy
B230217C12Rik	0.919	http://api.brain-map.org/grid_data/download/71495879?include=energy
Ddx41	0.919	http://api.brain-map.org/grid_data/download/564637?include=energy
Gpr133	0.919	http://api.brain-map.org/grid_data/download/74511759?include=energy
Pgd	0.919	http://api.brain-map.org/grid_data/download/70918988?include=energy
Pdzd11	0.919	http://api.brain-map.org/grid_data/download/545089?include=energy
Pebp1	0.918	http://api.brain-map.org/grid_data/download/69782766?include=energy
Gcn1l1	0.918	http://api.brain-map.org/grid_data/download/69672838?include=energy
Spata9	0.918	http://api.brain-map.org/grid_data/download/75831778?include=energy
B3gat2	0.918	http://api.brain-map.org/grid_data/download/74658033?include=energy
Eif2b5	0.918	http://api.brain-map.org/grid_data/download/69059879?include=energy
A830021K08Rik	0.918	http://api.brain-map.org/grid_data/download/73811555?include=energy
A630023P12Rik	0.918	http://api.brain-map.org/grid_data/download/70228537?include=energy
Wdr43	0.918	http://api.brain-map.org/grid_data/download/69202926?include=energy
Wtip	0.918	http://api.brain-map.org/grid_data/download/80342191?include=energy
Blk	0.918	http://api.brain-map.org/grid_data/download/68544991?include=energy
Cd72	0.918	http://api.brain-map.org/grid_data/download/74431053?include=energy
Aip	0.918	http://api.brain-map.org/grid_data/download/69526456?include=energy
Sec23b	0.918	http://api.brain-map.org/grid_data/download/275685?include=energy
Serpina1d	0.918	http://api.brain-map.org/grid_data/download/68410399?include=energy
Ranbp3	0.918	http://api.brain-map.org/grid_data/download/71493044?include=energy
Lnx2	0.918	http://api.brain-map.org/grid_data/download/75041516?include=energy
LOC383507	0.918	http://api.brain-map.org/grid_data/download/70725103?include=energy
Dcaf11	0.918	http://api.brain-map.org/grid_data/download/79536535?include=energy
Nox1	0.918	http://api.brain-map.org/grid_data/download/71832830?include=energy
Phf15	0.918	http://api.brain-map.org/grid_data/download/69289709?include=energy
Adam29	0.918	http://api.brain-map.org/grid_data/download/76080967?include=energy
F730209L11Rik*	0.918	http://api.brain-map.org/grid_data/download/73767172?include=energy
Lrrc4c	0.918	http://api.brain-map.org/grid_data/download/69445355?include=energy
2310008H04Rik	0.918	http://api.brain-map.org/grid_data/download/75931444?include=energy
Cnga2	0.918	http://api.brain-map.org/grid_data/download/68745245?include=energy
Tmprss7	0.918	http://api.brain-map.org/grid_data/download/75233739?include=energy
Zranb2	0.918	http://api.brain-map.org/grid_data/download/74277436?include=energy
Setmar	0.918	http://api.brain-map.org/grid_data/download/100144500?include=energy
Taf6	0.918	http://api.brain-map.org/grid_data/download/69174553?include=energy
Mst1r	0.918	http://api.brain-map.org/grid_data/download/71020845?include=energy
Dhx8	0.918	http://api.brain-map.org/grid_data/download/67815961?include=energy
Rpl30	0.918	http://api.brain-map.org/grid_data/download/100143515?include=energy
Hprt	0.918	http://api.brain-map.org/grid_data/download/68269059?include=energy
Ppt1	0.918	http://api.brain-map.org/grid_data/download/71210049?include=energy
Ccdc93	0.918	http://api.brain-map.org/grid_data/download/69013092?include=energy
Purb	0.918	http://api.brain-map.org/grid_data/download/75080632?include=energy
Aspg	0.918	http://api.brain-map.org/grid_data/download/70717975?include=energy
M6pr	0.918	http://api.brain-map.org/grid_data/download/69134268?include=energy
Dlat	0.918	http://api.brain-map.org/grid_data/download/70743896?include=energy
LOC381575	0.918	http://api.brain-map.org/grid_data/download/70725067?include=energy
Thoc7	0.918	http://api.brain-map.org/grid_data/download/69014536?include=energy
Rragb	0.918	http://api.brain-map.org/grid_data/download/71213819?include=energy
B230340J04Rik	0.918	http://api.brain-map.org/grid_data/download/69527848?include=energy
Usp2	0.918	http://api.brain-map.org/grid_data/download/76098316?include=energy
BC024479	0.918	http://api.brain-map.org/grid_data/download/70542998?include=energy
Nsun3	0.918	http://api.brain-map.org/grid_data/download/75988297?include=energy
Igsf8	0.918	http://api.brain-map.org/grid_data/download/68797507?include=energy
Zfp629	0.918	http://api.brain-map.org/grid_data/download/69261417?include=energy
Tm9sf4	0.918	http://api.brain-map.org/grid_data/download/69169361?include=energy
Timm9	0.918	http://api.brain-map.org/grid_data/download/70543008?include=energy
LOC432459	0.918	http://api.brain-map.org/grid_data/download/71789909?include=energy
Psca	0.918	http://api.brain-map.org/grid_data/download/71377812?include=energy
Syne1	0.918	http://api.brain-map.org/grid_data/download/74640879?include=energy
Thg1l	0.918	http://api.brain-map.org/grid_data/download/69443463?include=energy

Pde3b	0.918	http://api.brain-map.org/grid_data/download/75990408?include=energy
Manf	0.918	http://api.brain-map.org/grid_data/download/69114211?include=energy
Npm2	0.918	http://api.brain-map.org/grid_data/download/72182947?include=energy
BC106179	0.918	http://api.brain-map.org/grid_data/download/71380908?include=energy
Atp1b4	0.918	http://api.brain-map.org/grid_data/download/75198124?include=energy
Mgat4c	0.918	http://api.brain-map.org/grid_data/download/70301085?include=energy
Fmod	0.918	http://api.brain-map.org/grid_data/download/68910983?include=energy
Ddb2	0.917	http://api.brain-map.org/grid_data/download/74431059?include=energy
Usp31	0.917	http://api.brain-map.org/grid_data/download/69444897?include=energy
Tigd5	0.917	http://api.brain-map.org/grid_data/download/70598363?include=energy
Hivep1	0.917	http://api.brain-map.org/grid_data/download/69289117?include=energy
Fam162a	0.917	http://api.brain-map.org/grid_data/download/68341376?include=energy
Usp45	0.917	http://api.brain-map.org/grid_data/download/68160831?include=energy
Zfp365	0.917	http://api.brain-map.org/grid_data/download/73929873?include=energy
Fmo6	0.917	http://api.brain-map.org/grid_data/download/70565999?include=energy
Rhd	0.917	http://api.brain-map.org/grid_data/download/69818155?include=energy
4833447P13Rik	0.917	http://api.brain-map.org/grid_data/download/70205602?include=energy
LOC434793	0.917	http://api.brain-map.org/grid_data/download/70725615?include=energy
Isg15	0.917	http://api.brain-map.org/grid_data/download/68563110?include=energy
Ncam2	0.917	http://api.brain-map.org/grid_data/download/682?include=energy
6530409C15Rik	0.917	http://api.brain-map.org/grid_data/download/69445391?include=energy
1110012J17Rik	0.917	http://api.brain-map.org/grid_data/download/71808705?include=energy
Lyz2	0.917	http://api.brain-map.org/grid_data/download/100055065?include=energy
LOC436095	0.917	http://api.brain-map.org/grid_data/download/74736306?include=energy
Gm805	0.917	http://api.brain-map.org/grid_data/download/71764383?include=energy
F730015K02Rik	0.917	http://api.brain-map.org/grid_data/download/69672222?include=energy
Asgr2	0.917	http://api.brain-map.org/grid_data/download/68203464?include=energy
Cpa1	0.917	http://api.brain-map.org/grid_data/download/68667497?include=energy
E330027P06Rik*	0.917	http://api.brain-map.org/grid_data/download/73834535?include=energy
Vgll1	0.917	http://api.brain-map.org/grid_data/download/71808723?include=energy
Skap1	0.917	http://api.brain-map.org/grid_data/download/70201486?include=energy
Grm8	0.917	http://api.brain-map.org/grid_data/download/73771227?include=energy
Steap4	0.917	http://api.brain-map.org/grid_data/download/79539602?include=energy
Gtpbp5	0.917	http://api.brain-map.org/grid_data/download/68545330?include=energy
Fam159b	0.917	http://api.brain-map.org/grid_data/download/69514440?include=energy
Ntn1	0.917	http://api.brain-map.org/grid_data/download/74511838?include=energy
H2-T22	0.917	http://api.brain-map.org/grid_data/download/68076531?include=energy
Cdkn2c	0.917	http://api.brain-map.org/grid_data/download/1683?include=energy
Pak3	0.917	http://api.brain-map.org/grid_data/download/71836887?include=energy
Magel2	0.917	http://api.brain-map.org/grid_data/download/79591571?include=energy
Fblim1	0.917	http://api.brain-map.org/grid_data/download/67979049?include=energy
Fyn	0.917	http://api.brain-map.org/grid_data/download/70615595?include=energy
Fndc3b	0.917	http://api.brain-map.org/grid_data/download/74988759?include=energy
Ch25h	0.917	http://api.brain-map.org/grid_data/download/68162267?include=energy
Impa2	0.917	http://api.brain-map.org/grid_data/download/68301855?include=energy
Erap1	0.917	http://api.brain-map.org/grid_data/download/68924733?include=energy
Ccdc164	0.917	http://api.brain-map.org/grid_data/download/71670726?include=energy
D16H22S680E	0.917	http://api.brain-map.org/grid_data/download/74047888?include=energy
Eif2s3x	0.917	http://api.brain-map.org/grid_data/download/69671941?include=energy
Fntb	0.917	http://api.brain-map.org/grid_data/download/69672557?include=energy
Tekt4	0.917	http://api.brain-map.org/grid_data/download/100144863?include=energy
1200016E24Rik	0.917	http://api.brain-map.org/grid_data/download/1591?include=energy
Ubr1	0.917	http://api.brain-map.org/grid_data/download/74957908?include=energy
4930539J05Rik	0.917	http://api.brain-map.org/grid_data/download/71380532?include=energy
Prpf38a	0.917	http://api.brain-map.org/grid_data/download/75831730?include=energy
Dlx4	0.917	http://api.brain-map.org/grid_data/download/74821607?include=energy
LOC268906	0.917	http://api.brain-map.org/grid_data/download/71281919?include=energy
Dock9	0.917	http://api.brain-map.org/grid_data/download/69059943?include=energy
Gadl1	0.917	http://api.brain-map.org/grid_data/download/76081195?include=energy
Acnat2	0.917	http://api.brain-map.org/grid_data/download/71627810?include=energy
Oas1e	0.917	http://api.brain-map.org/grid_data/download/75931604?include=energy
Mxd4	0.917	http://api.brain-map.org/grid_data/download/67810541?include=energy
Card11	0.917	http://api.brain-map.org/grid_data/download/76081073?include=energy
Mex3c	0.917	http://api.brain-map.org/grid_data/download/70539154?include=energy
Ifitm3	0.917	http://api.brain-map.org/grid_data/download/75198112?include=energy
9530026P05Rik	0.917	http://api.brain-map.org/grid_data/download/71494641?include=energy
Vps37d	0.917	http://api.brain-map.org/grid_data/download/69262293?include=energy
0610011L14Rik	0.917	http://api.brain-map.org/grid_data/download/601189?include=energy
Zfp335	0.917	http://api.brain-map.org/grid_data/download/70303486?include=energy
Olfr222	0.917	http://api.brain-map.org/grid_data/download/74720916?include=energy
Kif5c	0.917	http://api.brain-map.org/grid_data/download/74511818?include=energy
Zfp708	0.917	http://api.brain-map.org/grid_data/download/74735259?include=energy
LOC384546	0.917	http://api.brain-map.org/grid_data/download/70725441?include=energy
1110037F02Rik	0.917	http://api.brain-map.org/grid_data/download/74988462?include=energy
Dak	0.917	http://api.brain-map.org/grid_data/download/71627877?include=energy
Gm684	0.917	http://api.brain-map.org/grid_data/download/70298232?include=energy
2310042E22Rik	0.917	http://api.brain-map.org/grid_data/download/74357582?include=energy
Gm5167	0.917	http://api.brain-map.org/grid_data/download/68744755?include=energy
2310002L09Rik	0.916	http://api.brain-map.org/grid_data/download/71991738?include=energy
Mgst3	0.916	http://api.brain-map.org/grid_data/download/71015350?include=energy
Mr1	0.916	http://api.brain-map.org/grid_data/download/75695646?include=energy
LOC546041	0.916	http://api.brain-map.org/grid_data/download/71022367?include=energy
Cant1	0.916	http://api.brain-map.org/grid_data/download/68666493?include=energy
Pgap2	0.916	http://api.brain-map.org/grid_data/download/79360302?include=energy
Prmt3	0.916	http://api.brain-map.org/grid_data/download/69113686?include=energy
Gm1679	0.916	http://api.brain-map.org/grid_data/download/72076605?include=energy
2700078E11Rik	0.916	http://api.brain-map.org/grid_data/download/69095949?include=energy
Lrrc34	0.916	http://api.brain-map.org/grid_data/download/71377916?include=energy
Hiat1	0.916	http://api.brain-map.org/grid_data/download/69734989?include=energy
Chia	0.916	http://api.brain-map.org/grid_data/download/68631990?include=energy
Gm1553	0.916	http://api.brain-map.org/grid_data/download/70276909?include=energy
Asb14	0.916	http://api.brain-map.org/grid_data/download/70231028?include=energy
Nkain4	0.916	http://api.brain-map.org/grid_data/download/112648182?include=energy
Tubgcp4	0.916	http://api.brain-map.org/grid_data/download/71210190?include=energy
Cdh2	0.916	http://api.brain-map.org/grid_data/download/79556666?include=energy
Churc1	0.916	http://api.brain-map.org/grid_data/download/69169511?include=energy
Pabpc1	0.916	http://api.brain-map.org/grid_data/download/71616794?include=energy
Eif2ak4	0.916	http://api.brain-map.org/grid_data/download/77620788?include=energy
Gsx1	0.916	http://api.brain-map.org/grid_data/download/77800891?include=energy
C1rl	0.916	http://api.brain-map.org/grid_data/download/69530858?include=energy
Slc13a4	0.916	http://api.brain-map.org/grid_data/download/69873809?include=energy
Mef2d	0.916	http://api.brain-map.org/grid_data/download/67810540?include=energy
Mllt6	0.916	http://api.brain-map.org/grid_data/download/69816631?include=energy
Tubd1	0.916	http://api.brain-map.org/grid_data/download/69549152?include=energy
A930007A09Rik	0.916	http://api.brain-map.org/grid_data/download/71765028?include=energy
Sfmbt1	0.916	http://api.brain-map.org/grid_data/download/69446679?include=energy
Slc38a11	0.916	http://api.brain-map.org/grid_data/download/71493873?include=energy
Srsf2	0.916	http://api.brain-map.org/grid_data/download/100144112?include=energy
Mbd4	0.916	http://api.brain-map.org/grid_data/download/69169929?include=energy
6330556J12Rik*	0.916	http://api.brain-map.org/grid_data/download/73927625?include=energy
Prdm16	0.916	http://api.brain-map.org/grid_data/download/68845323?include=energy
Stox2	0.916	http://api.brain-map.org/grid_data/download/70717366?include=energy
mCG147359	0.916	http://api.brain-map.org/grid_data/download/71212536?include=energy
Bhlhe41	0.916	http://api.brain-map.org/grid_data/download/73521802?include=energy
Tm9sf2	0.916	http://api.brain-map.org/grid_data/download/513255?include=energy
Gucy2f	0.916	http://api.brain-map.org/grid_data/download/74640883?include=energy
Rnf150	0.916	http://api.brain-map.org/grid_data/download/71020891?include=energy
Poldip2	0.916	http://api.brain-map.org/grid_data/download/386018?include=energy
Tom1l2	0.916	http://api.brain-map.org/grid_data/download/74749970?include=energy
Fam69b	0.916	http://api.brain-map.org/grid_data/download/73635786?include=energy
Rtp2	0.916	http://api.brain-map.org/grid_data/download/71656715?include=energy
Cecr5	0.916	http://api.brain-map.org/grid_data/download/75831729?include=energy
Pik3r4	0.916	http://api.brain-map.org/grid_data/download/68342336?include=energy
Stard6	0.916	http://api.brain-map.org/grid_data/download/69174517?include=energy
Cetn2	0.916	http://api.brain-map.org/grid_data/download/69838701?include=energy
Plekhm1	0.916	http://api.brain-map.org/grid_data/download/75828202?include=energy
Zfp871	0.916	http://api.brain-map.org/grid_data/download/70430766?include=energy
Zfp14	0.916	http://api.brain-map.org/grid_data/download/73423669?include=energy
Fam172a	0.916	http://api.brain-map.org/grid_data/download/70200872?include=energy
Mrpl24	0.915	http://api.brain-map.org/grid_data/download/532623?include=energy
Ssh2	0.915	http://api.brain-map.org/grid_data/download/70302270?include=energy
Asb4	0.915	http://api.brain-map.org/grid_data/download/71358591?include=energy
Wdr91	0.915	http://api.brain-map.org/grid_data/download/69014446?include=energy
Elavl2	0.915	http://api.brain-map.org/grid_data/download/72340123?include=energy
Zfp664	0.915	http://api.brain-map.org/grid_data/download/70231098?include=energy
Rrn3	0.915	http://api.brain-map.org/grid_data/download/70565506?include=energy
Abcf1	0.915	http://api.brain-map.org/grid_data/download/69514618?include=energy
Lst1	0.915	http://api.brain-map.org/grid_data/download/73512528?include=energy
Pcdh17	0.915	http://api.brain-map.org/grid_data/download/73636095?include=energy
Mm.294938	0.915	http://api.brain-map.org/grid_data/download/71161459?include=energy
Fubp1	0.915	http://api.brain-map.org/grid_data/download/70813113?include=energy
Gm5127	0.915	http://api.brain-map.org/grid_data/download/70305413?include=energy
Slc35a4	0.915	http://api.brain-map.org/grid_data/download/112647750?include=energy
BC005537	0.915	http://api.brain-map.org/grid_data/download/321201?include=energy
Bank1	0.915	http://api.brain-map.org/grid_data/download/69528076?include=energy
C630028M04Rik	0.915	http://api.brain-map.org/grid_data/download/74277640?include=energy
B230359F08Rik	0.915	http://api.brain-map.org/grid_data/download/74509542?include=energy
Cpox	0.915	http://api.brain-map.org/grid_data/download/393214?include=energy
4933433P14Rik	0.915	http://api.brain-map.org/grid_data/download/70430732?include=energy
Stx7	0.915	http://api.brain-map.org/grid_data/download/69887415?include=energy
Galnt10	0.915	http://api.brain-map.org/grid_data/download/70814342?include=energy
Fam55b	0.915	http://api.brain-map.org/grid_data/download/76081053?include=energy
Fry	0.915	http://api.brain-map.org/grid_data/download/70305174?include=energy
Psme2	0.915	http://api.brain-map.org/grid_data/download/74800893?include=energy
Actl7b	0.915	http://api.brain-map.org/grid_data/download/69514690?include=energy
Capn2	0.915	http://api.brain-map.org/grid_data/download/73817430?include=energy
1200011M11Rik	0.915	http://api.brain-map.org/grid_data/download/70474821?include=energy
Prdm11	0.915	http://api.brain-map.org/grid_data/download/69445677?include=energy
Tnfrsf21	0.915	http://api.brain-map.org/grid_data/download/71836885?include=energy
Atg2b	0.915	http://api.brain-map.org/grid_data/download/71023020?include=energy
Trim42	0.915	http://api.brain-map.org/grid_data/download/71891850?include=energy
Hsd3b5	0.915	http://api.brain-map.org/grid_data/download/68301317?include=energy
Olfr1362	0.915	http://api.brain-map.org/grid_data/download/73635610?include=energy
Arap1	0.915	http://api.brain-map.org/grid_data/download/71579879?include=energy
Tspan13	0.915	http://api.brain-map.org/grid_data/download/81599660?include=energy
Nek1	0.915	http://api.brain-map.org/grid_data/download/71210532?include=energy
Duox1	0.915	http://api.brain-map.org/grid_data/download/71587607?include=energy
Sgol2	0.915	http://api.brain-map.org/grid_data/download/69873690?include=energy
Lrrc6	0.915	http://api.brain-map.org/grid_data/download/68745390?include=energy
Akap8	0.915	http://api.brain-map.org/grid_data/download/91?include=energy
Arfgef1	0.915	http://api.brain-map.org/grid_data/download/77620265?include=energy
1810012K16Rik	0.915	http://api.brain-map.org/grid_data/download/71920267?include=energy
Smg6	0.915	http://api.brain-map.org/grid_data/download/71569331?include=energy
1500026H17Rik	0.915	http://api.brain-map.org/grid_data/download/75934926?include=energy
Tmprss11d	0.915	http://api.brain-map.org/grid_data/download/68341896?include=energy
Erbb2ip	0.915	http://api.brain-map.org/grid_data/download/74634836?include=energy
Mettl9	0.915	http://api.brain-map.org/grid_data/download/69838751?include=energy
Sema6c	0.915	http://api.brain-map.org/grid_data/download/75080151?include=energy
Tbx18	0.915	http://api.brain-map.org/grid_data/download/70593393?include=energy
Ntf5	0.915	http://api.brain-map.org/grid_data/download/1393?include=energy
Gdf15	0.915	http://api.brain-map.org/grid_data/download/76081203?include=energy
Slc26a3	0.915	http://api.brain-map.org/grid_data/download/74822832?include=energy
Haus2	0.915	http://api.brain-map.org/grid_data/download/69202621?include=energy
Kifc5b	0.915	http://api.brain-map.org/grid_data/download/74658089?include=energy
Aven	0.915	http://api.brain-map.org/grid_data/download/69236843?include=energy
Nrsn1	0.915	http://api.brain-map.org/grid_data/download/71358557?include=energy
Ssbp4	0.915	http://api.brain-map.org/grid_data/download/73817987?include=energy
Ccdc34	0.915	http://api.brain-map.org/grid_data/download/71493069?include=energy
Ddx52	0.915	http://api.brain-map.org/grid_data/download/67764923?include=energy
Kdm2a	0.915	http://api.brain-map.org/grid_data/download/69816553?include=energy
Bach2	0.915	http://api.brain-map.org/grid_data/download/69816488?include=energy
Foxj3	0.915	http://api.brain-map.org/grid_data/download/68798181?include=energy
Elmo3	0.915	http://api.brain-map.org/grid_data/download/73718049?include=energy
Slc44a2	0.915	http://api.brain-map.org/grid_data/download/79756317?include=energy
Smcr8	0.915	http://api.brain-map.org/grid_data/download/70302210?include=energy
Bptf	0.915	http://api.brain-map.org/grid_data/download/68844701?include=energy
Ltn1	0.915	http://api.brain-map.org/grid_data/download/79591577?include=energy
Kif17	0.914	http://api.brain-map.org/grid_data/download/72283659?include=energy
4930447F04Rik	0.914	http://api.brain-map.org/grid_data/download/70218145?include=energy
Prss32	0.914	http://api.brain-map.org/grid_data/download/68666487?include=energy
Gdf3	0.914	http://api.brain-map.org/grid_data/download/69672844?include=energy
Slc35b1	0.914	http://api.brain-map.org/grid_data/download/75750380?include=energy
Scn4a	0.914	http://api.brain-map.org/grid_data/download/69288297?include=energy
LOC434165	0.914	http://api.brain-map.org/grid_data/download/70787531?include=energy
Hcfc1	0.914	http://api.brain-map.org/grid_data/download/68798633?include=energy
Asf1a	0.914	http://api.brain-map.org/grid_data/download/68523372?include=energy
Zfp239	0.914	http://api.brain-map.org/grid_data/download/72728?include=energy
Ripk1	0.914	http://api.brain-map.org/grid_data/download/69015224?include=energy
Lrp1b	0.914	http://api.brain-map.org/grid_data/download/71670740?include=energy
Fau	0.914	http://api.brain-map.org/grid_data/download/74580990?include=energy
Dnajb13	0.914	http://api.brain-map.org/grid_data/download/69114115?include=energy
Zfp445	0.914	http://api.brain-map.org/grid_data/download/71380381?include=energy
Zkscan6	0.914	http://api.brain-map.org/grid_data/download/69407158?include=energy
Safb	0.914	http://api.brain-map.org/grid_data/download/69816482?include=energy
Glyr1	0.914	http://api.brain-map.org/grid_data/download/100146071?include=energy
1700014D04Rik	0.914	http://api.brain-map.org/grid_data/download/76081049?include=energy
Efnb2	0.914	http://api.brain-map.org/grid_data/download/72079959?include=energy
Ppp6r3	0.914	http://api.brain-map.org/grid_data/download/356861?include=energy
Fam60a	0.914	http://api.brain-map.org/grid_data/download/75081207?include=energy
Krt79	0.914	http://api.brain-map.org/grid_data/download/68522308?include=energy
Nhej1	0.914	http://api.brain-map.org/grid_data/download/69134679?include=energy
Dcp2	0.914	http://api.brain-map.org/grid_data/download/69443511?include=energy
Pmm1	0.914	http://api.brain-map.org/grid_data/download/67936010?include=energy
5031434O11Rik	0.914	http://api.brain-map.org/grid_data/download/69443268?include=energy
Wash	0.914	http://api.brain-map.org/grid_data/download/1796?include=energy
Arhgef12	0.914	http://api.brain-map.org/grid_data/download/69133761?include=energy
Rfpl4	0.914	http://api.brain-map.org/grid_data/download/76003601?include=energy
Mrgpre	0.914	http://api.brain-map.org/grid_data/download/70946172?include=energy
8030491B10Rik*	0.914	http://api.brain-map.org/grid_data/download/73906161?include=energy
Rpl15	0.914	http://api.brain-map.org/grid_data/download/74882810?include=energy
Gdap2	0.914	http://api.brain-map.org/grid_data/download/79556736?include=energy
Gmfb	0.914	http://api.brain-map.org/grid_data/download/1726?include=energy
Rufy2	0.914	http://api.brain-map.org/grid_data/download/69873343?include=energy
Cdk11b	0.914	http://api.brain-map.org/grid_data/download/69012950?include=energy
Arl6ip5	0.914	http://api.brain-map.org/grid_data/download/75651232?include=energy
1600002H07Rik	0.914	http://api.brain-map.org/grid_data/download/69353299?include=energy
Ctso	0.914	http://api.brain-map.org/grid_data/download/76003517?include=energy
Arid4a	0.914	http://api.brain-map.org/grid_data/download/69526850?include=energy
Pgr	0.914	http://api.brain-map.org/grid_data/download/73949514?include=energy
Wnk2	0.914	http://api.brain-map.org/grid_data/download/68861954?include=energy
G6pc3	0.914	http://api.brain-map.org/grid_data/download/69672689?include=energy
LOC386486	0.914	http://api.brain-map.org/grid_data/download/71991786?include=energy
Tdpoz1	0.914	http://api.brain-map.org/grid_data/download/70302437?include=energy
Pcnt	0.914	http://api.brain-map.org/grid_data/download/1255?include=energy
A830048P05	0.914	http://api.brain-map.org/grid_data/download/70305359?include=energy
Wfs1	0.914	http://api.brain-map.org/grid_data/download/74881161?include=energy
4932431P20Rik	0.914	http://api.brain-map.org/grid_data/download/72283468?include=energy
Ahctf1	0.914	http://api.brain-map.org/grid_data/download/75931435?include=energy
Mm.78531	0.914	http://api.brain-map.org/grid_data/download/71210436?include=energy
Ripk2	0.914	http://api.brain-map.org/grid_data/download/70301037?include=energy
Cep68	0.914	http://api.brain-map.org/grid_data/download/76003468?include=energy
Zmynd11	0.914	http://api.brain-map.org/grid_data/download/70743906?include=energy
Anxa6	0.914	http://api.brain-map.org/grid_data/download/77332100?include=energy
Gne	0.914	http://api.brain-map.org/grid_data/download/75694864?include=energy
Trim72	0.914	http://api.brain-map.org/grid_data/download/70787642?include=energy
Traf3ip3	0.914	http://api.brain-map.org/grid_data/download/69134187?include=energy
Mmp7	0.914	http://api.brain-map.org/grid_data/download/74430882?include=energy
Stmn1	0.914	http://api.brain-map.org/grid_data/download/71016595?include=energy
LOC433525	0.914	http://api.brain-map.org/grid_data/download/74579449?include=energy
Ppih	0.914	http://api.brain-map.org/grid_data/download/68667282?include=energy
Gjb6	0.914	http://api.brain-map.org/grid_data/download/2261?include=energy
Pi4ka	0.914	http://api.brain-map.org/grid_data/download/69783190?include=energy
2610029I01Rik	0.914	http://api.brain-map.org/grid_data/download/69202860?include=energy
Pard6b	0.914	http://api.brain-map.org/grid_data/download/76135515?include=energy
Poldip3	0.914	http://api.brain-map.org/grid_data/download/71487151?include=energy
Fmo2	0.914	http://api.brain-map.org/grid_data/download/68444744?include=energy
Ppef2	0.914	http://api.brain-map.org/grid_data/download/68157393?include=energy

LOC432781	0.914	http://api.brain-map.org/grid_data/download/70611299?include=energy
4933413G19Rik	0.914	http://api.brain-map.org/grid_data/download/72472483?include=energy
Cldn22	0.914	http://api.brain-map.org/grid_data/download/70237950?include=energy
Ikzf5	0.914	http://api.brain-map.org/grid_data/download/70609205?include=energy
Fgd4	0.914	http://api.brain-map.org/grid_data/download/71587661?include=energy
Mm.359728	0.913	http://api.brain-map.org/grid_data/download/71209934?include=energy
Pafah1b2	0.913	http://api.brain-map.org/grid_data/download/68076554?include=energy
Ap3s1	0.913	http://api.brain-map.org/grid_data/download/73512483?include=energy
Ythdc1	0.913	http://api.brain-map.org/grid_data/download/69262335?include=energy
Nfkb2	0.913	http://api.brain-map.org/grid_data/download/76098410?include=energy
LOC434822	0.913	http://api.brain-map.org/grid_data/download/71022415?include=energy
Fgfbp3	0.913	http://api.brain-map.org/grid_data/download/74635641?include=energy
Ano9	0.913	http://api.brain-map.org/grid_data/download/68666828?include=energy
Tcerg1	0.913	http://api.brain-map.org/grid_data/download/1872?include=energy
Pgp	0.913	http://api.brain-map.org/grid_data/download/71723820?include=energy
Klf16	0.913	http://api.brain-map.org/grid_data/download/69289285?include=energy
Gpr162	0.913	http://api.brain-map.org/grid_data/download/74658160?include=energy
Cisd1	0.913	http://api.brain-map.org/grid_data/download/71627779?include=energy
LOC433071	0.913	http://api.brain-map.org/grid_data/download/71919170?include=energy
Pdia5	0.913	http://api.brain-map.org/grid_data/download/75042262?include=energy
Catsper4	0.913	http://api.brain-map.org/grid_data/download/69236981?include=energy
Nagpa	0.913	http://api.brain-map.org/grid_data/download/68162265?include=energy
Hoxc6	0.913	http://api.brain-map.org/grid_data/download/81657681?include=energy
Allc	0.913	http://api.brain-map.org/grid_data/download/74581421?include=energy
Tekt1	0.913	http://api.brain-map.org/grid_data/download/75080238?include=energy
Plekhn1	0.913	http://api.brain-map.org/grid_data/download/74822579?include=energy
Tmcc1	0.913	http://api.brain-map.org/grid_data/download/69407523?include=energy
Ipo4	0.913	http://api.brain-map.org/grid_data/download/74658174?include=energy
Rgs14	0.913	http://api.brain-map.org/grid_data/download/2529?include=energy
LOC433227	0.913	http://api.brain-map.org/grid_data/download/70723020?include=energy
Slc34a2	0.913	http://api.brain-map.org/grid_data/download/100144882?include=energy
Eif3j	0.913	http://api.brain-map.org/grid_data/download/321196?include=energy
Plac8	0.913	http://api.brain-map.org/grid_data/download/75694776?include=energy
4933407P14Rik	0.913	http://api.brain-map.org/grid_data/download/70218661?include=energy
Mpp7	0.913	http://api.brain-map.org/grid_data/download/71789843?include=energy
Guca2b	0.913	http://api.brain-map.org/grid_data/download/69173220?include=energy
Cnnm2	0.913	http://api.brain-map.org/grid_data/download/74581378?include=energy
Arhgap5	0.913	http://api.brain-map.org/grid_data/download/113?include=energy
Chtf8	0.913	http://api.brain-map.org/grid_data/download/70445520?include=energy
C1qtnf6	0.913	http://api.brain-map.org/grid_data/download/77922580?include=energy
Arih2	0.913	http://api.brain-map.org/grid_data/download/69526856?include=energy
St14	0.913	http://api.brain-map.org/grid_data/download/100146140?include=energy
Il1f8	0.913	http://api.brain-map.org/grid_data/download/71991810?include=energy
Tctn3	0.913	http://api.brain-map.org/grid_data/download/75695672?include=energy
Col2a1	0.913	http://api.brain-map.org/grid_data/download/71489805?include=energy
Aldh4a1	0.913	http://api.brain-map.org/grid_data/download/69014524?include=energy
Mocos	0.913	http://api.brain-map.org/grid_data/download/70946616?include=energy
Mbtd1	0.913	http://api.brain-map.org/grid_data/download/68341585?include=energy
G3bp1	0.913	http://api.brain-map.org/grid_data/download/68301192?include=energy
Lrrc4b	0.913	http://api.brain-map.org/grid_data/download/68845299?include=energy
Rhag	0.913	http://api.brain-map.org/grid_data/download/76081030?include=energy
Dpy19l1	0.913	http://api.brain-map.org/grid_data/download/75042226?include=energy
Egfl7	0.913	http://api.brain-map.org/grid_data/download/532705?include=energy
Copa	0.913	http://api.brain-map.org/grid_data/download/1689?include=energy
Plcxd2	0.913	http://api.brain-map.org/grid_data/download/79591579?include=energy
Sil1	0.913	http://api.brain-map.org/grid_data/download/386019?include=energy
Hspa13	0.913	http://api.brain-map.org/grid_data/download/76003326?include=energy
Khnyn	0.913	http://api.brain-map.org/grid_data/download/69446571?include=energy
Mtap	0.913	http://api.brain-map.org/grid_data/download/79568030?include=energy
Fam123a	0.913	http://api.brain-map.org/grid_data/download/74821568?include=energy
Dph5	0.913	http://api.brain-map.org/grid_data/download/75990638?include=energy
Enpp7	0.913	http://api.brain-map.org/grid_data/download/71764461?include=energy
Mia1	0.913	http://api.brain-map.org/grid_data/download/80341095?include=energy
Asprv1	0.913	http://api.brain-map.org/grid_data/download/69202322?include=energy
5930422O12Rik	0.913	http://api.brain-map.org/grid_data/download/69444735?include=energy
5730508B09Rik	0.913	http://api.brain-map.org/grid_data/download/71493771?include=energy
Adam15	0.913	http://api.brain-map.org/grid_data/download/392924?include=energy
Scx	0.913	http://api.brain-map.org/grid_data/download/74635719?include=energy
Phox2a	0.913	http://api.brain-map.org/grid_data/download/73818803?include=energy
Gtsf1l	0.913	http://api.brain-map.org/grid_data/download/69116478?include=energy
Slco1a6	0.913	http://api.brain-map.org/grid_data/download/70302192?include=energy
Krt13	0.913	http://api.brain-map.org/grid_data/download/68798328?include=energy
Cuedc2	0.913	http://api.brain-map.org/grid_data/download/79556640?include=energy
F930037H19Rik*	0.913	http://api.brain-map.org/grid_data/download/73767335?include=energy
Rnf5	0.913	http://api.brain-map.org/grid_data/download/69080047?include=energy
1110038D17Rik	0.913	http://api.brain-map.org/grid_data/download/70303737?include=energy
Napb	0.913	http://api.brain-map.org/grid_data/download/75651234?include=energy
Tgm1	0.912	http://api.brain-map.org/grid_data/download/68498445?include=energy
Rnaseh2c	0.912	http://api.brain-map.org/grid_data/download/67870083?include=energy
Pram1	0.912	http://api.brain-map.org/grid_data/download/71213806?include=energy
Cbfa2t2	0.912	http://api.brain-map.org/grid_data/download/68844725?include=energy
4930412O13Rik	0.912	http://api.brain-map.org/grid_data/download/74513917?include=energy
Gria2	0.912	http://api.brain-map.org/grid_data/download/75551462?include=energy
Plod3	0.912	http://api.brain-map.org/grid_data/download/69816308?include=energy
Mecr	0.912	http://api.brain-map.org/grid_data/download/71763522?include=energy
Pcdha8	0.912	http://api.brain-map.org/grid_data/download/79354812?include=energy
Mm.352421	0.912	http://api.brain-map.org/grid_data/download/71161153?include=energy
Asnsd1	0.912	http://api.brain-map.org/grid_data/download/75826628?include=energy
Zfp768	0.912	http://api.brain-map.org/grid_data/download/70539181?include=energy
Grtp1	0.912	http://api.brain-map.org/grid_data/download/79544828?include=energy
LOC435536	0.912	http://api.brain-map.org/grid_data/download/74822604?include=energy
Ctla4	0.912	http://api.brain-map.org/grid_data/download/75079745?include=energy
Mesp1	0.912	http://api.brain-map.org/grid_data/download/72081665?include=energy
LOC432487	0.912	http://api.brain-map.org/grid_data/download/70608565?include=energy
Dvl1	0.912	http://api.brain-map.org/grid_data/download/69671666?include=energy
Uba5	0.912	http://api.brain-map.org/grid_data/download/68797498?include=energy
Tpd52	0.912	http://api.brain-map.org/grid_data/download/69079999?include=energy
Pdcd6	0.912	http://api.brain-map.org/grid_data/download/68191144?include=energy
Deaf1	0.912	http://api.brain-map.org/grid_data/download/69262298?include=energy
1700019A02Rik	0.912	http://api.brain-map.org/grid_data/download/74357839?include=energy
Galnt5	0.912	http://api.brain-map.org/grid_data/download/76003533?include=energy
E2f2	0.912	http://api.brain-map.org/grid_data/download/100144523?include=energy
Matn3	0.912	http://api.brain-map.org/grid_data/download/69752095?include=energy
Aebp1	0.912	http://api.brain-map.org/grid_data/download/69524398?include=energy
Naa40	0.912	http://api.brain-map.org/grid_data/download/70305006?include=energy
Egfr	0.912	http://api.brain-map.org/grid_data/download/81599632?include=energy
Hoxa9	0.912	http://api.brain-map.org/grid_data/download/575?include=energy
Spata21	0.912	http://api.brain-map.org/grid_data/download/71569084?include=energy
Wdr36	0.912	http://api.brain-map.org/grid_data/download/69169570?include=energy
Idh3a	0.912	http://api.brain-map.org/grid_data/download/68632020?include=energy
Use1	0.912	http://api.brain-map.org/grid_data/download/68546242?include=energy
Commd8	0.912	http://api.brain-map.org/grid_data/download/76080997?include=energy
Grin3b	0.912	http://api.brain-map.org/grid_data/download/77869800?include=energy
LOC240093	0.912	http://api.brain-map.org/grid_data/download/74736081?include=energy
E230028L10Rik	0.912	http://api.brain-map.org/grid_data/download/74510206?include=energy
Slc1a4	0.912	http://api.brain-map.org/grid_data/download/68920797?include=energy
Cox7b2	0.912	http://api.brain-map.org/grid_data/download/69116984?include=energy
5430427O19Rik	0.912	http://api.brain-map.org/grid_data/download/70218920?include=energy
D130058E03	0.912	http://api.brain-map.org/grid_data/download/70305652?include=energy
B830028H17Rik*	0.912	http://api.brain-map.org/grid_data/download/75042245?include=energy
LOC434069	0.912	http://api.brain-map.org/grid_data/download/70725567?include=energy
Top2a	0.912	http://api.brain-map.org/grid_data/download/1301?include=energy
F930007G17Rik*	0.912	http://api.brain-map.org/grid_data/download/73767386?include=energy
Klf4	0.912	http://api.brain-map.org/grid_data/download/77792639?include=energy
Dvl2	0.912	http://api.brain-map.org/grid_data/download/112649476?include=energy
Coro2b	0.912	http://api.brain-map.org/grid_data/download/72077454?include=energy
Rtcd1	0.912	http://api.brain-map.org/grid_data/download/583850?include=energy
Ky	0.912	http://api.brain-map.org/grid_data/download/71381058?include=energy
Mkln1	0.912	http://api.brain-map.org/grid_data/download/68301622?include=energy
Jmy	0.912	http://api.brain-map.org/grid_data/download/75197666?include=energy
Rere	0.912	http://api.brain-map.org/grid_data/download/69289805?include=energy
Glg1	0.912	http://api.brain-map.org/grid_data/download/75042254?include=energy
Pabpc1l	0.912	http://api.brain-map.org/grid_data/download/70596065?include=energy
Taf2	0.912	http://api.brain-map.org/grid_data/download/73811729?include=energy
Fam50a	0.912	http://api.brain-map.org/grid_data/download/74882206?include=energy
Ptcd2	0.912	http://api.brain-map.org/grid_data/download/293037?include=energy
Zzz3	0.912	http://api.brain-map.org/grid_data/download/70200824?include=energy
LOC433951	0.912	http://api.brain-map.org/grid_data/download/70787149?include=energy
Abcb1b	0.912	http://api.brain-map.org/grid_data/download/70724511?include=energy
Catsper2	0.912	http://api.brain-map.org/grid_data/download/69236975?include=energy
Trem1	0.911	http://api.brain-map.org/grid_data/download/71489791?include=energy
6720469N11Rik	0.911	http://api.brain-map.org/grid_data/download/69445421?include=energy
Tmpo	0.911	http://api.brain-map.org/grid_data/download/77456079?include=energy
Zfand2b	0.911	http://api.brain-map.org/grid_data/download/71836831?include=energy
Rhbdd3	0.911	http://api.brain-map.org/grid_data/download/69443475?include=energy
Pygb	0.911	http://api.brain-map.org/grid_data/download/276075?include=energy
Snw1	0.911	http://api.brain-map.org/grid_data/download/77925165?include=energy
Scaf11	0.911	http://api.brain-map.org/grid_data/download/77799019?include=energy
Pcm1	0.911	http://api.brain-map.org/grid_data/download/69782850?include=energy
Adcy2	0.911	http://api.brain-map.org/grid_data/download/73?include=energy
BC030500	0.911	http://api.brain-map.org/grid_data/download/70525806?include=energy
Sh2d1b1	0.911	http://api.brain-map.org/grid_data/download/69671833?include=energy
5730403I07Rik	0.911	http://api.brain-map.org/grid_data/download/74426036?include=energy
Olfr1366	0.911	http://api.brain-map.org/grid_data/download/74720775?include=energy
Zfp157	0.911	http://api.brain-map.org/grid_data/download/70474947?include=energy
Papd4	0.911	http://api.brain-map.org/grid_data/download/76081952?include=energy
LOC436450	0.911	http://api.brain-map.org/grid_data/download/74736366?include=energy
B230316E19Rik*	0.911	http://api.brain-map.org/grid_data/download/73811579?include=energy
Pam16	0.911	http://api.brain-map.org/grid_data/download/2391?include=energy
Zcwpw1	0.911	http://api.brain-map.org/grid_data/download/74510314?include=energy
Irak4	0.911	http://api.brain-map.org/grid_data/download/69549325?include=energy
Slc6a12	0.911	http://api.brain-map.org/grid_data/download/78534516?include=energy
Mllt3	0.911	http://api.brain-map.org/grid_data/download/545114?include=energy
Kansl2	0.911	http://api.brain-map.org/grid_data/download/81599630?include=energy
H1fnt	0.911	http://api.brain-map.org/grid_data/download/76081052?include=energy
Pdpn	0.911	http://api.brain-map.org/grid_data/download/71789777?include=energy
Bhlha9	0.911	http://api.brain-map.org/grid_data/download/71380369?include=energy
Topbp1	0.911	http://api.brain-map.org/grid_data/download/70191961?include=energy
Rag1	0.911	http://api.brain-map.org/grid_data/download/76003584?include=energy
1700012B15Rik	0.911	http://api.brain-map.org/grid_data/download/71919379?include=energy
Cst11	0.911	http://api.brain-map.org/grid_data/download/76080998?include=energy
LOC436353	0.911	http://api.brain-map.org/grid_data/download/71283400?include=energy
D630044L22Rik	0.911	http://api.brain-map.org/grid_data/download/71809437?include=energy
Gm9736	0.911	http://api.brain-map.org/grid_data/download/71668373?include=energy
Fam170a	0.911	http://api.brain-map.org/grid_data/download/71587751?include=energy
Gas2l1	0.911	http://api.brain-map.org/grid_data/download/74509597?include=energy
Ecscr	0.911	http://api.brain-map.org/grid_data/download/69352878?include=energy
Fam49b	0.911	http://api.brain-map.org/grid_data/download/564781?include=energy
LOC435897	0.911	http://api.brain-map.org/grid_data/download/71022615?include=energy
Zcchc5	0.911	http://api.brain-map.org/grid_data/download/70303462?include=energy
Ube2v2	0.911	http://api.brain-map.org/grid_data/download/68844140?include=energy
Krtap3-2	0.911	http://api.brain-map.org/grid_data/download/70295458?include=energy
Ssb	0.911	http://api.brain-map.org/grid_data/download/75081397?include=energy
Mettl11a	0.911	http://api.brain-map.org/grid_data/download/68637951?include=energy
4733401D07Rik*	0.911	http://api.brain-map.org/grid_data/download/73906473?include=energy
Timm23	0.911	http://api.brain-map.org/grid_data/download/69980995?include=energy
Dsg1c	0.911	http://api.brain-map.org/grid_data/download/100145355?include=energy
1700020N01Rik	0.911	http://api.brain-map.org/grid_data/download/69816410?include=energy
Cpa4	0.911	http://api.brain-map.org/grid_data/download/75861011?include=energy
Hus1	0.911	http://api.brain-map.org/grid_data/download/69174112?include=energy
Rai12	0.911	http://api.brain-map.org/grid_data/download/68269065?include=energy
Lrrc51	0.911	http://api.brain-map.org/grid_data/download/69170162?include=energy
Arhgap33	0.911	http://api.brain-map.org/grid_data/download/74583174?include=energy
Mc3r	0.91	http://api.brain-map.org/grid_data/download/74511790?include=energy
Aif1	0.91	http://api.brain-map.org/grid_data/download/75080249?include=energy
Crb3	0.91	http://api.brain-map.org/grid_data/download/75861851?include=energy
Golph3l	0.91	http://api.brain-map.org/grid_data/download/532709?include=energy
Park7	0.91	http://api.brain-map.org/grid_data/download/112194293?include=energy
Snrpc	0.91	http://api.brain-map.org/grid_data/download/69079981?include=energy
LOC432610	0.91	http://api.brain-map.org/grid_data/download/71808472?include=energy
Acadsb	0.91	http://api.brain-map.org/grid_data/download/70528175?include=energy
Tmem35	0.91	http://api.brain-map.org/grid_data/download/71587898?include=energy
Ppm1a	0.91	http://api.brain-map.org/grid_data/download/2497?include=energy
Gldc	0.91	http://api.brain-map.org/grid_data/download/74658091?include=energy
Csnk1g1	0.91	http://api.brain-map.org/grid_data/download/69174595?include=energy
Osbpl6	0.91	http://api.brain-map.org/grid_data/download/70744325?include=energy
Grifin	0.91	http://api.brain-map.org/grid_data/download/70276963?include=energy
Trmt112	0.91	http://api.brain-map.org/grid_data/download/71654121?include=energy
Krtap15	0.91	http://api.brain-map.org/grid_data/download/69735974?include=energy
Pcdhgc4	0.91	http://api.brain-map.org/grid_data/download/70784958?include=energy
Tex13	0.91	http://api.brain-map.org/grid_data/download/74056140?include=energy
Stard13	0.91	http://api.brain-map.org/grid_data/download/71587846?include=energy
Adamts6	0.91	http://api.brain-map.org/grid_data/download/74513929?include=energy
Aggf1	0.91	http://api.brain-map.org/grid_data/download/75931434?include=energy
Cxcr6	0.91	http://api.brain-map.org/grid_data/download/69237173?include=energy
Klk8	0.91	http://api.brain-map.org/grid_data/download/72129241?include=energy
Tagln	0.91	http://api.brain-map.org/grid_data/download/68443116?include=energy
D430042O09Rik	0.91	http://api.brain-map.org/grid_data/download/67881759?include=energy
Stx4a	0.91	http://api.brain-map.org/grid_data/download/576415?include=energy
Sox2ot	0.91	http://api.brain-map.org/grid_data/download/74658020?include=energy
Zfp141	0.91	http://api.brain-map.org/grid_data/download/70787561?include=energy
Asxl1	0.91	http://api.brain-map.org/grid_data/download/69526928?include=energy
Adat1	0.91	http://api.brain-map.org/grid_data/download/638703?include=energy
Camsap3	0.91	http://api.brain-map.org/grid_data/download/68924602?include=energy
1810012P15Rik	0.91	http://api.brain-map.org/grid_data/download/73497732?include=energy
Gtpbp4	0.91	http://api.brain-map.org/grid_data/download/75828168?include=energy
B230319C09Rik	0.91	http://api.brain-map.org/grid_data/download/70231146?include=energy
A430010J10Rik	0.91	http://api.brain-map.org/grid_data/download/71735819?include=energy
Wnt10b	0.91	http://api.brain-map.org/grid_data/download/2725?include=energy
Abcf3	0.91	http://api.brain-map.org/grid_data/download/68545656?include=energy
Prmt6	0.91	http://api.brain-map.org/grid_data/download/74431679?include=energy
Hoxb1	0.91	http://api.brain-map.org/grid_data/download/69289147?include=energy
Zfp932	0.91	http://api.brain-map.org/grid_data/download/75774665?include=energy
Lrp2bp	0.91	http://api.brain-map.org/grid_data/download/69114809?include=energy
Pycrl	0.91	http://api.brain-map.org/grid_data/download/2515?include=energy
Entpd4	0.91	http://api.brain-map.org/grid_data/download/68798663?include=energy
Zfyve1	0.91	http://api.brain-map.org/grid_data/download/68667019?include=energy
Zfp532	0.91	http://api.brain-map.org/grid_data/download/69262264?include=energy
Snap23	0.91	http://api.brain-map.org/grid_data/download/69885570?include=energy
Kif18a	0.91	http://api.brain-map.org/grid_data/download/68342312?include=energy
Cdc7	0.91	http://api.brain-map.org/grid_data/download/69540653?include=energy
Leprot	0.91	http://api.brain-map.org/grid_data/download/75773649?include=energy
Vsnl1	0.91	http://api.brain-map.org/grid_data/download/1106?include=energy
Ntf3	0.91	http://api.brain-map.org/grid_data/download/2440?include=energy
mCG144692	0.91	http://api.brain-map.org/grid_data/download/71212111?include=energy
Hint1	0.91	http://api.brain-map.org/grid_data/download/73520984?include=energy
Spint1	0.91	http://api.brain-map.org/grid_data/download/70813908?include=energy
Mslnl	0.91	http://api.brain-map.org/grid_data/download/75831708?include=energy
Bag2	0.91	http://api.brain-map.org/grid_data/download/70634296?include=energy
Hexim2	0.91	http://api.brain-map.org/grid_data/download/69443112?include=energy
Fam175b	0.91	http://api.brain-map.org/grid_data/download/71612197?include=energy
Nfkbie	0.91	http://api.brain-map.org/grid_data/download/2425?include=energy
Usp28	0.91	http://api.brain-map.org/grid_data/download/70194630?include=energy
Tnfaip8l2	0.91	http://api.brain-map.org/grid_data/download/70523836?include=energy
Fam132b	0.91	http://api.brain-map.org/grid_data/download/68844128?include=energy
Capn12	0.91	http://api.brain-map.org/grid_data/download/68744504?include=energy
Erp29	0.91	http://api.brain-map.org/grid_data/download/72007751?include=energy
Mm.371778	0.91	http://api.brain-map.org/grid_data/download/71210019?include=energy
Vrk2	0.91	http://api.brain-map.org/grid_data/download/68341268?include=energy
Gm427	0.91	http://api.brain-map.org/grid_data/download/70300186?include=energy
Vwa5b2	0.91	http://api.brain-map.org/grid_data/download/71587835?include=energy
LOC433498	0.91	http://api.brain-map.org/grid_data/download/74635268?include=energy
4921515J06Rik	0.91	http://api.brain-map.org/grid_data/download/69407821?include=energy
Esyt2	0.91	http://api.brain-map.org/grid_data/download/71213707?include=energy
BC061194	0.91	http://api.brain-map.org/grid_data/download/69114878?include=energy
Pigs	0.909	http://api.brain-map.org/grid_data/download/68844223?include=energy
LOC435291	0.909	http://api.brain-map.org/grid_data/download/71456134?include=energy

Tsc2	0.909	http://api.brain-map.org/grid_data/download/1431?include=energy
Cep152	0.909	http://api.brain-map.org/grid_data/download/70230855?include=energy
Pgap1	0.909	http://api.brain-map.org/grid_data/download/73949545?include=energy
Ccdc94	0.909	http://api.brain-map.org/grid_data/download/68522326?include=energy
Rrp12	0.909	http://api.brain-map.org/grid_data/download/68667811?include=energy
Spin1	0.909	http://api.brain-map.org/grid_data/download/70429453?include=energy
Cdan1	0.909	http://api.brain-map.org/grid_data/download/69540641?include=energy
1200011I18Rik	0.909	http://api.brain-map.org/grid_data/download/77414569?include=energy
Slmap	0.909	http://api.brain-map.org/grid_data/download/76081839?include=energy
Gem	0.909	http://api.brain-map.org/grid_data/download/73817928?include=energy
Pacs1	0.909	http://api.brain-map.org/grid_data/download/76098403?include=energy
Mm.223854	0.909	http://api.brain-map.org/grid_data/download/71152503?include=energy
Dcaf17	0.909	http://api.brain-map.org/grid_data/download/68631887?include=energy
Man2b1	0.909	http://api.brain-map.org/grid_data/download/75774113?include=energy
Lrrc58	0.909	http://api.brain-map.org/grid_data/download/70231941?include=energy
Krt36	0.909	http://api.brain-map.org/grid_data/download/73514719?include=energy
Sbk1	0.909	http://api.brain-map.org/grid_data/download/71836807?include=energy
Zmym1	0.909	http://api.brain-map.org/grid_data/download/69015134?include=energy
Zfp459	0.909	http://api.brain-map.org/grid_data/download/70303504?include=energy
Ahcyl2	0.909	http://api.brain-map.org/grid_data/download/73929896?include=energy
Fam126b	0.909	http://api.brain-map.org/grid_data/download/70231749?include=energy
Gyg	0.909	http://api.brain-map.org/grid_data/download/73817930?include=energy
Slco1b2	0.909	http://api.brain-map.org/grid_data/download/78534550?include=energy
Cacng2	0.909	http://api.brain-map.org/grid_data/download/69236951?include=energy
5430419D17Rik	0.909	http://api.brain-map.org/grid_data/download/71809151?include=energy
Ap1g1	0.909	http://api.brain-map.org/grid_data/download/71280550?include=energy
Cdkl5	0.909	http://api.brain-map.org/grid_data/download/75042239?include=energy
Rbpms	0.909	http://api.brain-map.org/grid_data/download/74635298?include=energy
mCG2521.2	0.909	http://api.brain-map.org/grid_data/download/74822880?include=energy
Kctd8	0.909	http://api.brain-map.org/grid_data/download/71670722?include=energy
Cd300lb	0.909	http://api.brain-map.org/grid_data/download/75931515?include=energy
Rsph3a	0.909	http://api.brain-map.org/grid_data/download/73573145?include=energy
Ddx27	0.909	http://api.brain-map.org/grid_data/download/71610895?include=energy
Mul1	0.909	http://api.brain-map.org/grid_data/download/587440?include=energy
Pla2g4b	0.909	http://api.brain-map.org/grid_data/download/68193066?include=energy
Tgfb3	0.909	http://api.brain-map.org/grid_data/download/2667?include=energy
Hdx	0.909	http://api.brain-map.org/grid_data/download/69816524?include=energy
Mst1	0.909	http://api.brain-map.org/grid_data/download/75934514?include=energy
Nyx	0.909	http://api.brain-map.org/grid_data/download/75990556?include=energy
Zmym2	0.909	http://api.brain-map.org/grid_data/download/73925714?include=energy
Ttc9	0.909	http://api.brain-map.org/grid_data/download/70785212?include=energy
Ube2k	0.909	http://api.brain-map.org/grid_data/download/73817935?include=energy
Mxra7	0.909	http://api.brain-map.org/grid_data/download/77332080?include=energy
Zfp827	0.909	http://api.brain-map.org/grid_data/download/100144431?include=energy
2010001K21Rik	0.909	http://api.brain-map.org/grid_data/download/69201702?include=energy
V1rg10	0.909	http://api.brain-map.org/grid_data/download/69442247?include=energy
Prrg4	0.909	http://api.brain-map.org/grid_data/download/69447882?include=energy
Nenf	0.909	http://api.brain-map.org/grid_data/download/74272919?include=energy
Mm.185016	0.909	http://api.brain-map.org/grid_data/download/71152246?include=energy
Minos1	0.909	http://api.brain-map.org/grid_data/download/71212141?include=energy
Sbf2	0.909	http://api.brain-map.org/grid_data/download/77465056?include=energy
Ddx1	0.909	http://api.brain-map.org/grid_data/download/71723912?include=energy
Mccc1	0.909	http://api.brain-map.org/grid_data/download/556995?include=energy
Dock10	0.909	http://api.brain-map.org/grid_data/download/74047443?include=energy
Gm17739	0.909	http://api.brain-map.org/grid_data/download/70565488?include=energy
Mrps26	0.909	http://api.brain-map.org/grid_data/download/69113841?include=energy
Rnf168	0.909	http://api.brain-map.org/grid_data/download/75985956?include=energy
Nedd4l	0.909	http://api.brain-map.org/grid_data/download/74047904?include=energy
4931417E11Rik	0.909	http://api.brain-map.org/grid_data/download/70723716?include=energy
H2-M3	0.909	http://api.brain-map.org/grid_data/download/68631917?include=energy
Dtwd2	0.909	http://api.brain-map.org/grid_data/download/71492796?include=energy
LOC434326	0.909	http://api.brain-map.org/grid_data/download/74578395?include=energy
Tmed7	0.909	http://api.brain-map.org/grid_data/download/70813095?include=energy
Tssk4	0.909	http://api.brain-map.org/grid_data/download/71892185?include=energy
Il10ra	0.909	http://api.brain-map.org/grid_data/download/75041443?include=energy
Phf3	0.909	http://api.brain-map.org/grid_data/download/70300786?include=energy
Idua	0.909	http://api.brain-map.org/grid_data/download/69015338?include=energy
Exoc1	0.909	http://api.brain-map.org/grid_data/download/77620453?include=energy
Rbpj	0.909	http://api.brain-map.org/grid_data/download/69262324?include=energy
Ror2	0.909	http://api.brain-map.org/grid_data/download/71378507?include=energy
Tcea2	0.909	http://api.brain-map.org/grid_data/download/74988737?include=energy
Fbp1	0.909	http://api.brain-map.org/grid_data/download/68302166?include=energy
LOC242879	0.909	http://api.brain-map.org/grid_data/download/70566304?include=energy
Gpr87	0.909	http://api.brain-map.org/grid_data/download/69257660?include=energy
Ccdc8	0.909	http://api.brain-map.org/grid_data/download/70787438?include=energy
Hist1h2ag	0.908	http://api.brain-map.org/grid_data/download/77620449?include=energy
Miip	0.908	http://api.brain-map.org/grid_data/download/69080506?include=energy
Ap4e1	0.908	http://api.brain-map.org/grid_data/download/70230969?include=energy
4930479M11Rik	0.908	http://api.brain-map.org/grid_data/download/71891808?include=energy
Zscan21	0.908	http://api.brain-map.org/grid_data/download/79541418?include=energy
Fam20c	0.908	http://api.brain-map.org/grid_data/download/654929?include=energy
Dach2	0.908	http://api.brain-map.org/grid_data/download/74819331?include=energy
Klra17	0.908	http://api.brain-map.org/grid_data/download/100145302?include=energy
Pom121	0.908	http://api.brain-map.org/grid_data/download/68843811?include=energy
Pi4k2a	0.908	http://api.brain-map.org/grid_data/download/74047913?include=energy
Lmo1	0.908	http://api.brain-map.org/grid_data/download/1373?include=energy
Hif3a	0.908	http://api.brain-map.org/grid_data/download/69289111?include=energy
Apob	0.908	http://api.brain-map.org/grid_data/download/70230981?include=energy
Stat1	0.908	http://api.brain-map.org/grid_data/download/2635?include=energy
D17Wsu92e	0.908	http://api.brain-map.org/grid_data/download/71496387?include=energy
Psmb2	0.908	http://api.brain-map.org/grid_data/download/74988552?include=energy
LOC386478	0.908	http://api.brain-map.org/grid_data/download/72076411?include=energy
Col1a1	0.908	http://api.brain-map.org/grid_data/download/71992079?include=energy
Aftph	0.908	http://api.brain-map.org/grid_data/download/69835580?include=energy
Car4	0.908	http://api.brain-map.org/grid_data/download/70928238?include=energy
Akr1c12	0.908	http://api.brain-map.org/grid_data/download/100146047?include=energy
Olfr1349	0.908	http://api.brain-map.org/grid_data/download/74720741?include=energy
Dchs2	0.908	http://api.brain-map.org/grid_data/download/70946400?include=energy
Mast2	0.908	http://api.brain-map.org/grid_data/download/71656816?include=energy
Nid1	0.908	http://api.brain-map.org/grid_data/download/75084474?include=energy
Ash2l	0.908	http://api.brain-map.org/grid_data/download/513225?include=energy
Cpped1	0.908	http://api.brain-map.org/grid_data/download/70431199?include=energy
1700028J19Rik	0.908	http://api.brain-map.org/grid_data/download/73716489?include=energy
Mrpl47	0.908	http://api.brain-map.org/grid_data/download/71020791?include=energy
Eif4g2	0.908	http://api.brain-map.org/grid_data/download/70795766?include=energy
LOC432738	0.908	http://api.brain-map.org/grid_data/download/70611240?include=energy
Ogg1	0.908	http://api.brain-map.org/grid_data/download/69782563?include=energy
Hagh	0.908	http://api.brain-map.org/grid_data/download/532605?include=energy
Lrfn5	0.908	http://api.brain-map.org/grid_data/download/68798968?include=energy
Klf9	0.908	http://api.brain-map.org/grid_data/download/70593169?include=energy
Pdzd7	0.908	http://api.brain-map.org/grid_data/download/71569121?include=energy
2410004B18Rik	0.908	http://api.brain-map.org/grid_data/download/71493014?include=energy
Jkamp	0.908	http://api.brain-map.org/grid_data/download/80474706?include=energy
Ucma	0.908	http://api.brain-map.org/grid_data/download/71488974?include=energy
Mfsd6l	0.908	http://api.brain-map.org/grid_data/download/68410626?include=energy
Zkscan17	0.908	http://api.brain-map.org/grid_data/download/72182991?include=energy
Klhl17	0.908	http://api.brain-map.org/grid_data/download/69735867?include=energy
Scnn1g	0.908	http://api.brain-map.org/grid_data/download/1918?include=energy
Sestd1	0.908	http://api.brain-map.org/grid_data/download/69262280?include=energy
Derl1	0.908	http://api.brain-map.org/grid_data/download/67978472?include=energy
Ghrl	0.908	http://api.brain-map.org/grid_data/download/77465010?include=energy
Lsm14a	0.908	http://api.brain-map.org/grid_data/download/68546374?include=energy
Znrd1as	0.908	http://api.brain-map.org/grid_data/download/68441556?include=energy
Zfp661	0.908	http://api.brain-map.org/grid_data/download/68667712?include=energy
Tagln3	0.908	http://api.brain-map.org/grid_data/download/75988633?include=energy
Nts	0.908	http://api.brain-map.org/grid_data/download/73788032?include=energy
Rbm15b	0.908	http://api.brain-map.org/grid_data/download/68797918?include=energy
Mbip	0.908	http://api.brain-map.org/grid_data/download/69080024?include=energy
Foxn3	0.908	http://api.brain-map.org/grid_data/download/68522362?include=energy
LOC433987	0.908	http://api.brain-map.org/grid_data/download/74562516?include=energy
Aatf	0.908	http://api.brain-map.org/grid_data/download/75774654?include=energy
1700084P21Rik	0.908	http://api.brain-map.org/grid_data/download/74635078?include=energy
A830007P12Rik	0.908	http://api.brain-map.org/grid_data/download/68076970?include=energy
Ptp4a1	0.908	http://api.brain-map.org/grid_data/download/75079765?include=energy
Atg9b	0.908	http://api.brain-map.org/grid_data/download/69672959?include=energy
Nnat	0.908	http://api.brain-map.org/grid_data/download/77887874?include=energy
Ubtf	0.908	http://api.brain-map.org/grid_data/download/74988738?include=energy
Rimklb	0.908	http://api.brain-map.org/grid_data/download/69837406?include=energy
Fbxo48	0.908	http://api.brain-map.org/grid_data/download/70228567?include=energy
Cd209e	0.908	http://api.brain-map.org/grid_data/download/76081078?include=energy
Irgq	0.908	http://api.brain-map.org/grid_data/download/69524416?include=energy
Six5	0.908	http://api.brain-map.org/grid_data/download/81657675?include=energy
Kel	0.908	http://api.brain-map.org/grid_data/download/75931520?include=energy
5330421K23Rik*	0.908	http://api.brain-map.org/grid_data/download/75043138?include=energy
Lair1	0.908	http://api.brain-map.org/grid_data/download/68117985?include=energy
Afp	0.908	http://api.brain-map.org/grid_data/download/69524431?include=energy
Gtf2i	0.908	http://api.brain-map.org/grid_data/download/2277?include=energy
Pomt2	0.908	http://api.brain-map.org/grid_data/download/68798873?include=energy
Xcr1	0.907	http://api.brain-map.org/grid_data/download/69352718?include=energy
Meis3	0.907	http://api.brain-map.org/grid_data/download/74047845?include=energy
Oc90	0.907	http://api.brain-map.org/grid_data/download/68797870?include=energy
4833439L19Rik	0.907	http://api.brain-map.org/grid_data/download/81599638?include=energy
Etv3	0.907	http://api.brain-map.org/grid_data/download/1494?include=energy
Pebp4	0.907	http://api.brain-map.org/grid_data/download/69114192?include=energy
Atox1	0.907	http://api.brain-map.org/grid_data/download/68744498?include=energy
Ptprs	0.907	http://api.brain-map.org/grid_data/download/74882787?include=energy
Prl3a1	0.907	http://api.brain-map.org/grid_data/download/75931620?include=energy
Clk3	0.907	http://api.brain-map.org/grid_data/download/69549006?include=energy
Gkn2	0.907	http://api.brain-map.org/grid_data/download/71991732?include=energy
P2rx5	0.907	http://api.brain-map.org/grid_data/download/75551475?include=energy
A230083G16Rik	0.907	http://api.brain-map.org/grid_data/download/69447996?include=energy
Zhx2	0.907	http://api.brain-map.org/grid_data/download/68798819?include=energy
Serpina1e	0.907	http://api.brain-map.org/grid_data/download/75084134?include=energy
Shroom3	0.907	http://api.brain-map.org/grid_data/download/71377764?include=energy
Trim69	0.907	http://api.brain-map.org/grid_data/download/69116498?include=energy
Pars2	0.907	http://api.brain-map.org/grid_data/download/69059867?include=energy
5730409E04Rik	0.907	http://api.brain-map.org/grid_data/download/74988752?include=energy
4930435E12Rik	0.907	http://api.brain-map.org/grid_data/download/72182964?include=energy
Rnf4	0.907	http://api.brain-map.org/grid_data/download/513497?include=energy
Bai2	0.907	http://api.brain-map.org/grid_data/download/73584753?include=energy
Mrgprb4	0.907	http://api.brain-map.org/grid_data/download/70784648?include=energy
Zap70	0.907	http://api.brain-map.org/grid_data/download/69174010?include=energy
Plod2	0.907	http://api.brain-map.org/grid_data/download/68445119?include=energy
Ebna1bp2	0.907	http://api.brain-map.org/grid_data/download/69671845?include=energy
Sf3b4	0.907	http://api.brain-map.org/grid_data/download/69173325?include=energy
Ptdss1	0.907	http://api.brain-map.org/grid_data/download/74800904?include=energy
Zfand3	0.907	http://api.brain-map.org/grid_data/download/293694?include=energy
Usf2	0.907	http://api.brain-map.org/grid_data/download/79544866?include=energy
Upf3b	0.907	http://api.brain-map.org/grid_data/download/75862560?include=energy
Rccd1	0.907	http://api.brain-map.org/grid_data/download/80472057?include=energy
Gm1195	0.907	http://api.brain-map.org/grid_data/download/70300043?include=energy
Nrxn3	0.907	http://api.brain-map.org/grid_data/download/75042240?include=energy
4930524L23Rik	0.907	http://api.brain-map.org/grid_data/download/70218247?include=energy
Ncoa3	0.907	http://api.brain-map.org/grid_data/download/71380429?include=energy
F830205F14Rik*	0.907	http://api.brain-map.org/grid_data/download/74427108?include=energy
Bsdc1	0.907	http://api.brain-map.org/grid_data/download/74988722?include=energy
Cyr61	0.907	http://api.brain-map.org/grid_data/download/1191?include=energy
Dpep2	0.907	http://api.brain-map.org/grid_data/download/74430978?include=energy
Mtap1s	0.907	http://api.brain-map.org/grid_data/download/76003512?include=energy
Hspa12a	0.907	http://api.brain-map.org/grid_data/download/74047899?include=energy
D830024N08Rik	0.907	http://api.brain-map.org/grid_data/download/70238764?include=energy
4933407C03Rik	0.907	http://api.brain-map.org/grid_data/download/100146057?include=energy
Chchd8	0.907	http://api.brain-map.org/grid_data/download/69443349?include=energy
Nus1	0.907	http://api.brain-map.org/grid_data/download/74581265?include=energy
Tipin	0.907	http://api.brain-map.org/grid_data/download/68410195?include=energy
Tmem11	0.907	http://api.brain-map.org/grid_data/download/75043144?include=energy
Dner	0.907	http://api.brain-map.org/grid_data/download/1699?include=energy
Ap1m1	0.907	http://api.brain-map.org/grid_data/download/71610778?include=energy
Sox12	0.907	http://api.brain-map.org/grid_data/download/81657669?include=energy
Tlx3	0.907	http://api.brain-map.org/grid_data/download/71609033?include=energy
Dlg5	0.907	http://api.brain-map.org/grid_data/download/545115?include=energy
A230083A21Rik*	0.907	http://api.brain-map.org/grid_data/download/73949608?include=energy
C230060E24	0.907	http://api.brain-map.org/grid_data/download/71574594?include=energy
Pfkfb1	0.907	http://api.brain-map.org/grid_data/download/69783035?include=energy
Kcnq1	0.907	http://api.brain-map.org/grid_data/download/69014506?include=energy
Atp1a1	0.907	http://api.brain-map.org/grid_data/download/71836844?include=energy
Exog	0.907	http://api.brain-map.org/grid_data/download/69672031?include=energy
Mphosph8	0.907	http://api.brain-map.org/grid_data/download/71283528?include=energy
Ctnnd1	0.907	http://api.brain-map.org/grid_data/download/74641320?include=energy
Pde7a	0.907	http://api.brain-map.org/grid_data/download/69782927?include=energy
Aars2	0.906	http://api.brain-map.org/grid_data/download/308054705?include=energy
Lmln	0.906	http://api.brain-map.org/grid_data/download/76003557?include=energy
Chst1	0.906	http://api.brain-map.org/grid_data/download/71836872?include=energy
Ano5	0.906	http://api.brain-map.org/grid_data/download/71281671?include=energy
Tmem54	0.906	http://api.brain-map.org/grid_data/download/76097730?include=energy
B430006D22Rik	0.906	http://api.brain-map.org/grid_data/download/73712750?include=energy
4930471G03Rik	0.906	http://api.brain-map.org/grid_data/download/73513269?include=energy
4922501C03Rik	0.906	http://api.brain-map.org/grid_data/download/72283408?include=energy
G630070P10Rik*	0.906	http://api.brain-map.org/grid_data/download/73731301?include=energy
Upf3a	0.906	http://api.brain-map.org/grid_data/download/70194588?include=energy
Hcrtr2	0.906	http://api.brain-map.org/grid_data/download/80517329?include=energy
E330016A19Rik	0.906	http://api.brain-map.org/grid_data/download/70946505?include=energy
Hacl1	0.906	http://api.brain-map.org/grid_data/download/68445644?include=energy
Gng4	0.906	http://api.brain-map.org/grid_data/download/515?include=energy
AI597479	0.906	http://api.brain-map.org/grid_data/download/386254?include=energy
Plxnc1	0.906	http://api.brain-map.org/grid_data/download/820?include=energy
Pitpnm1	0.906	http://api.brain-map.org/grid_data/download/68845371?include=energy
Gadd45g	0.906	http://api.brain-map.org/grid_data/download/70634407?include=energy
LOC432872	0.906	http://api.brain-map.org/grid_data/download/74736189?include=energy
Trmt1	0.906	http://api.brain-map.org/grid_data/download/68522314?include=energy
Olfr736	0.906	http://api.brain-map.org/grid_data/download/74734926?include=energy
Pear1	0.906	http://api.brain-map.org/grid_data/download/69838068?include=energy
Olfr555	0.906	http://api.brain-map.org/grid_data/download/74724175?include=energy
Rassf7	0.906	http://api.brain-map.org/grid_data/download/68302317?include=energy
Prkacb	0.906	http://api.brain-map.org/grid_data/download/75043158?include=energy
Htra4	0.906	http://api.brain-map.org/grid_data/download/70231182?include=energy
Zfp599	0.906	http://api.brain-map.org/grid_data/download/69549315?include=energy
Wdyhv1	0.906	http://api.brain-map.org/grid_data/download/71656679?include=energy
Hsd17b7	0.906	http://api.brain-map.org/grid_data/download/71608375?include=energy
Mpv17l2	0.906	http://api.brain-map.org/grid_data/download/74988601?include=energy
Rgl1	0.906	http://api.brain-map.org/grid_data/download/70439105?include=energy
Cry2	0.906	http://api.brain-map.org/grid_data/download/302?include=energy
Cherp	0.906	http://api.brain-map.org/grid_data/download/69059949?include=energy
Cbln2	0.906	http://api.brain-map.org/grid_data/download/70231306?include=energy
Wnt9a	0.906	http://api.brain-map.org/grid_data/download/72008493?include=energy
Nt5dc1	0.906	http://api.brain-map.org/grid_data/download/76003724?include=energy
Prol1	0.906	http://api.brain-map.org/grid_data/download/72737?include=energy
1700010L13Rik	0.906	http://api.brain-map.org/grid_data/download/74359521?include=energy
Map3k9	0.906	http://api.brain-map.org/grid_data/download/74510194?include=energy
Lcn6	0.906	http://api.brain-map.org/grid_data/download/74431447?include=energy
Rbfa	0.906	http://api.brain-map.org/grid_data/download/68911003?include=energy
4933409G03Rik	0.906	http://api.brain-map.org/grid_data/download/70218667?include=energy
Mrpl37	0.906	http://api.brain-map.org/grid_data/download/100144553?include=energy
Slc7a4	0.906	http://api.brain-map.org/grid_data/download/130535?include=energy
Mrps36	0.906	http://api.brain-map.org/grid_data/download/68744570?include=energy
Tmem126b	0.906	http://api.brain-map.org/grid_data/download/70445377?include=energy
Magi2	0.906	http://api.brain-map.org/grid_data/download/74821819?include=energy
Zfp553	0.906	http://api.brain-map.org/grid_data/download/70539192?include=energy
Nol10	0.906	http://api.brain-map.org/grid_data/download/73497615?include=energy
Marveld2	0.906	http://api.brain-map.org/grid_data/download/75831744?include=energy
6720416L17Rik	0.906	http://api.brain-map.org/grid_data/download/69445409?include=energy
Sept2	0.906	http://api.brain-map.org/grid_data/download/69816834?include=energy
BC048403	0.906	http://api.brain-map.org/grid_data/download/71495956?include=energy
Ncapd3	0.906	http://api.brain-map.org/grid_data/download/69527764?include=energy
Slc37a3	0.906	http://api.brain-map.org/grid_data/download/69169325?include=energy
1810020D17Rik	0.906	http://api.brain-map.org/grid_data/download/1959?include=energy
Nat2	0.906	http://api.brain-map.org/grid_data/download/68546578?include=energy

Dus4l	0.906	http://api.brain-map.org/grid_data/download/70539233?include=energy
Htr1f	0.906	http://api.brain-map.org/grid_data/download/69859867?include=energy
Mtl5	0.906	http://api.brain-map.org/grid_data/download/75861870?include=energy
Olfm4	0.906	http://api.brain-map.org/grid_data/download/73712780?include=energy
Ankrd61	0.906	http://api.brain-map.org/grid_data/download/75831780?include=energy
Ung	0.906	http://api.brain-map.org/grid_data/download/632508?include=energy
4930519L02Rik	0.906	http://api.brain-map.org/grid_data/download/75197783?include=energy
Rab9	0.906	http://api.brain-map.org/grid_data/download/75990588?include=energy
Igdcc3	0.906	http://api.brain-map.org/grid_data/download/75831725?include=energy
Tssc1	0.906	http://api.brain-map.org/grid_data/download/68637635?include=energy
Zfp764	0.906	http://api.brain-map.org/grid_data/download/71612198?include=energy
Xist	0.906	http://api.brain-map.org/grid_data/download/80342286?include=energy
Arntl	0.906	http://api.brain-map.org/grid_data/download/2045?include=energy
XM_356036	0.906	http://api.brain-map.org/grid_data/download/70596161?include=energy
Spen	0.905	http://api.brain-map.org/grid_data/download/73606546?include=energy
Rbbp9	0.905	http://api.brain-map.org/grid_data/download/80341122?include=energy
Rbmx	0.905	http://api.brain-map.org/grid_data/download/75043154?include=energy
Dnajc3	0.905	http://api.brain-map.org/grid_data/download/100145443?include=energy
Mosc1	0.905	http://api.brain-map.org/grid_data/download/68523201?include=energy
Cdc42bpg	0.905	http://api.brain-map.org/grid_data/download/71495950?include=energy
2310045N01Rik	0.905	http://api.brain-map.org/grid_data/download/70218709?include=energy
LOC433157	0.905	http://api.brain-map.org/grid_data/download/70722946?include=energy
Ccnl2	0.905	http://api.brain-map.org/grid_data/download/211?include=energy
Arl1	0.905	http://api.brain-map.org/grid_data/download/69526862?include=energy
Git1	0.905	http://api.brain-map.org/grid_data/download/69672880?include=energy
7120438E14Rik*	0.905	http://api.brain-map.org/grid_data/download/73927670?include=energy
2310004I24Rik	0.905	http://api.brain-map.org/grid_data/download/70429106?include=energy
D830044I16Rik	0.905	http://api.brain-map.org/grid_data/download/74510134?include=energy
Mier2	0.905	http://api.brain-map.org/grid_data/download/393264?include=energy
Usp3	0.905	http://api.brain-map.org/grid_data/download/69080505?include=energy
Dapk3	0.905	http://api.brain-map.org/grid_data/download/70724661?include=energy
Sirt5	0.905	http://api.brain-map.org/grid_data/download/68632088?include=energy
Cdca8	0.905	http://api.brain-map.org/grid_data/download/69540665?include=energy
Bfsp1	0.905	http://api.brain-map.org/grid_data/download/69838255?include=energy
Hsd17b11	0.905	http://api.brain-map.org/grid_data/download/69080051?include=energy
Ncapg2	0.905	http://api.brain-map.org/grid_data/download/79536618?include=energy
Gm9895	0.905	http://api.brain-map.org/grid_data/download/72081627?include=energy
Zfp474	0.905	http://api.brain-map.org/grid_data/download/69104098?include=energy
TC1431885	0.905	http://api.brain-map.org/grid_data/download/70795430?include=energy
Nmu	0.905	http://api.brain-map.org/grid_data/download/72182969?include=energy
Kdm1a	0.905	http://api.brain-map.org/grid_data/download/69262119?include=energy
Tmem201	0.905	http://api.brain-map.org/grid_data/download/70231080?include=energy
E2f3	0.905	http://api.brain-map.org/grid_data/download/100145357?include=energy
Usp24	0.905	http://api.brain-map.org/grid_data/download/68744582?include=energy
Lrrc61	0.905	http://api.brain-map.org/grid_data/download/68444958?include=energy
Hnrnpc	0.905	http://api.brain-map.org/grid_data/download/100143529?include=energy
Cklf	0.905	http://api.brain-map.org/grid_data/download/71724478?include=energy
Gfi1	0.905	http://api.brain-map.org/grid_data/download/75990426?include=energy
Vps41	0.905	http://api.brain-map.org/grid_data/download/70194946?include=energy
Fabp12	0.905	http://api.brain-map.org/grid_data/download/69169815?include=energy
P2ry4	0.905	http://api.brain-map.org/grid_data/download/69258286?include=energy
BC053994	0.905	http://api.brain-map.org/grid_data/download/77340462?include=energy
Cetn1	0.905	http://api.brain-map.org/grid_data/download/70724643?include=energy
Ophn1	0.905	http://api.brain-map.org/grid_data/download/70539202?include=energy
Mm.274484	0.905	http://api.brain-map.org/grid_data/download/72182980?include=energy
Rhbdl3	0.905	http://api.brain-map.org/grid_data/download/71020887?include=energy
Cd24a	0.905	http://api.brain-map.org/grid_data/download/79591541?include=energy
Mfn1	0.905	http://api.brain-map.org/grid_data/download/71487139?include=energy
Ttll1	0.905	http://api.brain-map.org/grid_data/download/71610853?include=energy
Haao	0.905	http://api.brain-map.org/grid_data/download/71723808?include=energy
A930003A15Rik	0.905	http://api.brain-map.org/grid_data/download/74300783?include=energy
Pnpla3	0.905	http://api.brain-map.org/grid_data/download/68522410?include=energy
Gm5115	0.905	http://api.brain-map.org/grid_data/download/70429088?include=energy
Ubl5	0.905	http://api.brain-map.org/grid_data/download/68744600?include=energy
Meaf6	0.905	http://api.brain-map.org/grid_data/download/71991768?include=energy
Hmgcs1	0.905	http://api.brain-map.org/grid_data/download/80517057?include=energy
Stat4	0.905	http://api.brain-map.org/grid_data/download/70593391?include=energy
Mrpl35	0.905	http://api.brain-map.org/grid_data/download/68523162?include=energy
Homer2	0.905	http://api.brain-map.org/grid_data/download/71015814?include=energy
Sfn	0.905	http://api.brain-map.org/grid_data/download/2561?include=energy
LOC670796	0.905	http://api.brain-map.org/grid_data/download/77924522?include=energy
Ccdc71	0.905	http://api.brain-map.org/grid_data/download/67765707?include=energy
Fstl3	0.905	http://api.brain-map.org/grid_data/download/69672611?include=energy
Gak	0.905	http://api.brain-map.org/grid_data/download/67815935?include=energy
Lrrc27	0.905	http://api.brain-map.org/grid_data/download/69114968?include=energy
Gphn	0.905	http://api.brain-map.org/grid_data/download/1211?include=energy
Clip1	0.905	http://api.brain-map.org/grid_data/download/293260?include=energy
9630033F20Rik	0.905	http://api.brain-map.org/grid_data/download/71249748?include=energy
Gstz1	0.905	http://api.brain-map.org/grid_data/download/68632076?include=energy
Pglyrp2	0.905	http://api.brain-map.org/grid_data/download/68410357?include=energy
Hcn4	0.905	http://api.brain-map.org/grid_data/download/69257749?include=energy
Lysmd2	0.905	http://api.brain-map.org/grid_data/download/74988572?include=energy
Dnajc6	0.905	http://api.brain-map.org/grid_data/download/71358627?include=energy
Epn2	0.905	http://api.brain-map.org/grid_data/download/68632002?include=energy
Ccdc61	0.904	http://api.brain-map.org/grid_data/download/74513866?include=energy
Vmn1r21	0.904	http://api.brain-map.org/grid_data/download/75198232?include=energy
2610027F03Rik	0.904	http://api.brain-map.org/grid_data/download/74635629?include=energy
Pwwp2a	0.904	http://api.brain-map.org/grid_data/download/71380501?include=energy
Tll2	0.904	http://api.brain-map.org/grid_data/download/69981025?include=energy
Igfbp1	0.904	http://api.brain-map.org/grid_data/download/513231?include=energy
Trip12	0.904	http://api.brain-map.org/grid_data/download/67815967?include=energy
Dtna	0.904	http://api.brain-map.org/grid_data/download/77413704?include=energy
Suv420h1	0.904	http://api.brain-map.org/grid_data/download/69169427?include=energy
Dusp13	0.904	http://api.brain-map.org/grid_data/download/73716483?include=energy
Kctd11	0.904	http://api.brain-map.org/grid_data/download/76003678?include=energy
Fabp2	0.904	http://api.brain-map.org/grid_data/download/70525819?include=energy
Sdcbp	0.904	http://api.brain-map.org/grid_data/download/70744518?include=energy
Ctdspl2	0.904	http://api.brain-map.org/grid_data/download/68667425?include=energy
LOC435693	0.904	http://api.brain-map.org/grid_data/download/70784528?include=energy
Crip3	0.904	http://api.brain-map.org/grid_data/download/72183005?include=energy
Snip1	0.904	http://api.brain-map.org/grid_data/download/70303630?include=energy
Wsb2	0.904	http://api.brain-map.org/grid_data/download/71764320?include=energy
Actrt2	0.904	http://api.brain-map.org/grid_data/download/69114914?include=energy
Ctnnd2	0.904	http://api.brain-map.org/grid_data/download/69540411?include=energy
Unc5c	0.904	http://api.brain-map.org/grid_data/download/71670708?include=energy
Fam83h	0.904	http://api.brain-map.org/grid_data/download/69514506?include=energy
Pphln1	0.904	http://api.brain-map.org/grid_data/download/69080527?include=energy
2610002I17Rik	0.904	http://api.brain-map.org/grid_data/download/69202788?include=energy
B3gntl1	0.904	http://api.brain-map.org/grid_data/download/69444747?include=energy
Skiv2l	0.904	http://api.brain-map.org/grid_data/download/71210889?include=energy
Sec16a	0.904	http://api.brain-map.org/grid_data/download/71613641?include=energy
Plekhj1	0.904	http://api.brain-map.org/grid_data/download/633304?include=energy
Gcet2	0.904	http://api.brain-map.org/grid_data/download/69672814?include=energy
Acadl	0.904	http://api.brain-map.org/grid_data/download/72081416?include=energy
Man2a1	0.904	http://api.brain-map.org/grid_data/download/69201564?include=energy
Gadd45gip1	0.904	http://api.brain-map.org/grid_data/download/70545913?include=energy
D230040P08Rik*	0.904	http://api.brain-map.org/grid_data/download/74427118?include=energy
LOC432443	0.904	http://api.brain-map.org/grid_data/download/70608511?include=energy
Gm4912	0.904	http://api.brain-map.org/grid_data/download/70566181?include=energy
LOC230602	0.904	http://api.brain-map.org/grid_data/download/74514339?include=energy
Man2b2	0.904	http://api.brain-map.org/grid_data/download/77620239?include=energy
Prl4a1	0.904	http://api.brain-map.org/grid_data/download/68667073?include=energy
Lsm14b	0.904	http://api.brain-map.org/grid_data/download/71495944?include=energy
Sun3	0.904	http://api.brain-map.org/grid_data/download/71808674?include=energy
Gm5247	0.904	http://api.brain-map.org/grid_data/download/70725097?include=energy
Rad51	0.904	http://api.brain-map.org/grid_data/download/293471?include=energy
Prosapip1	0.904	http://api.brain-map.org/grid_data/download/71064051?include=energy
Slitrk2	0.904	http://api.brain-map.org/grid_data/download/69874144?include=energy
Gmpr2	0.904	http://api.brain-map.org/grid_data/download/100144758?include=energy
Anapc1	0.904	http://api.brain-map.org/grid_data/download/69028468?include=energy
Wipf2	0.904	http://api.brain-map.org/grid_data/download/69014494?include=energy
Ect2	0.904	http://api.brain-map.org/grid_data/download/68446087?include=energy
A930011G23Rik	0.904	http://api.brain-map.org/grid_data/download/69514428?include=energy
Rnase4	0.904	http://api.brain-map.org/grid_data/download/68300938?include=energy
Arpc1a	0.904	http://api.brain-map.org/grid_data/download/77280335?include=energy
1700045I19Rik	0.904	http://api.brain-map.org/grid_data/download/69353708?include=energy
1700025K23Rik	0.904	http://api.brain-map.org/grid_data/download/71919505?include=energy
Gm5752	0.904	http://api.brain-map.org/grid_data/download/74736318?include=energy
A430105I19Rik	0.904	http://api.brain-map.org/grid_data/download/70228442?include=energy
Tox4	0.904	http://api.brain-map.org/grid_data/download/70430750?include=energy
Odf4	0.904	http://api.brain-map.org/grid_data/download/77924346?include=energy
Ak3	0.904	http://api.brain-map.org/grid_data/download/70429160?include=energy
Ube2h	0.904	http://api.brain-map.org/grid_data/download/74583203?include=energy
Apbb1ip	0.904	http://api.brain-map.org/grid_data/download/68444666?include=energy
Exd2	0.904	http://api.brain-map.org/grid_data/download/70430726?include=energy
Copb2	0.904	http://api.brain-map.org/grid_data/download/1341?include=energy
Aak1	0.904	http://api.brain-map.org/grid_data/download/308055062?include=energy
Utp6	0.904	http://api.brain-map.org/grid_data/download/67767445?include=energy
Dok1	0.904	http://api.brain-map.org/grid_data/download/75042248?include=energy
Jmjd6	0.904	http://api.brain-map.org/grid_data/download/69015140?include=energy
AI848100	0.904	http://api.brain-map.org/grid_data/download/71250314?include=energy
Nrap	0.904	http://api.brain-map.org/grid_data/download/74059223?include=energy
Tmem79	0.904	http://api.brain-map.org/grid_data/download/67979039?include=energy
Phxr1	0.904	http://api.brain-map.org/grid_data/download/69783118?include=energy
F13a1	0.904	http://api.brain-map.org/grid_data/download/74882565?include=energy
Lipg	0.904	http://api.brain-map.org/grid_data/download/72182994?include=energy
Cib3	0.904	http://api.brain-map.org/grid_data/download/71021156?include=energy
Olfr676	0.904	http://api.brain-map.org/grid_data/download/74734824?include=energy
Cpxcr1	0.904	http://api.brain-map.org/grid_data/download/70300031?include=energy
Cst7	0.904	http://api.brain-map.org/grid_data/download/75988365?include=energy
Klf5	0.904	http://api.brain-map.org/grid_data/download/100146139?include=energy
Ap4m1	0.904	http://api.brain-map.org/grid_data/download/686196?include=energy
Rerg	0.904	http://api.brain-map.org/grid_data/download/76135613?include=energy
1700010D01Rik	0.904	http://api.brain-map.org/grid_data/download/69174451?include=energy
Bivm	0.904	http://api.brain-map.org/grid_data/download/75988302?include=energy
Hist1h2bg	0.904	http://api.brain-map.org/grid_data/download/73769308?include=energy
Ppm1b	0.904	http://api.brain-map.org/grid_data/download/68193051?include=energy
Fam122c	0.904	http://api.brain-map.org/grid_data/download/75931613?include=energy
Prl2b1	0.903	http://api.brain-map.org/grid_data/download/71919224?include=energy
Adcy6	0.903	http://api.brain-map.org/grid_data/download/81?include=energy
Fam124a	0.903	http://api.brain-map.org/grid_data/download/72472790?include=energy
Tle2	0.903	http://api.brain-map.org/grid_data/download/74359712?include=energy
Zfp958	0.903	http://api.brain-map.org/grid_data/download/73496911?include=energy
Trappc2l	0.903	http://api.brain-map.org/grid_data/download/68744630?include=energy
Iqcb1	0.903	http://api.brain-map.org/grid_data/download/69080729?include=energy
Gm5494	0.903	http://api.brain-map.org/grid_data/download/70722916?include=energy
Ralyl	0.903	http://api.brain-map.org/grid_data/download/72129250?include=energy
LOC433540	0.903	http://api.brain-map.org/grid_data/download/71764530?include=energy
Sik2	0.903	http://api.brain-map.org/grid_data/download/71281740?include=energy
LOC385308	0.903	http://api.brain-map.org/grid_data/download/70725471?include=energy
LOC545863	0.903	http://api.brain-map.org/grid_data/download/70787233?include=energy
Fpgs	0.903	http://api.brain-map.org/grid_data/download/68910961?include=energy
Kctd19	0.903	http://api.brain-map.org/grid_data/download/72182956?include=energy
Mip	0.903	http://api.brain-map.org/grid_data/download/71789825?include=energy
4930546C10Rik	0.903	http://api.brain-map.org/grid_data/download/70218324?include=energy
Hmgb1	0.903	http://api.brain-map.org/grid_data/download/293892?include=energy
Zic5	0.903	http://api.brain-map.org/grid_data/download/74635017?include=energy
Uggt1	0.903	http://api.brain-map.org/grid_data/download/74957909?include=energy
Usp14	0.903	http://api.brain-map.org/grid_data/download/74881157?include=energy
Rnf133	0.903	http://api.brain-map.org/grid_data/download/70301055?include=energy
Heph	0.903	http://api.brain-map.org/grid_data/download/69838555?include=energy
Cyp2c66	0.903	http://api.brain-map.org/grid_data/download/74635319?include=energy
Slc25a36	0.903	http://api.brain-map.org/grid_data/download/67881555?include=energy
Dck	0.903	http://api.brain-map.org/grid_data/download/69837461?include=energy
Slc35e2	0.903	http://api.brain-map.org/grid_data/download/74881514?include=energy
A130004G07Rik	0.903	http://api.brain-map.org/grid_data/download/69447900?include=energy
Eddm3b	0.903	http://api.brain-map.org/grid_data/download/68666559?include=energy
Ssty2	0.903	http://api.brain-map.org/grid_data/download/69885780?include=energy
Jsrp1	0.903	http://api.brain-map.org/grid_data/download/69202316?include=energy
Setd4	0.903	http://api.brain-map.org/grid_data/download/1798?include=energy
Mypn	0.903	http://api.brain-map.org/grid_data/download/68667569?include=energy
Kcnj15	0.903	http://api.brain-map.org/grid_data/download/2339?include=energy
Pcyox1l	0.903	http://api.brain-map.org/grid_data/download/71656781?include=energy
LOC432560	0.903	http://api.brain-map.org/grid_data/download/70608610?include=energy
Cdk5rap1	0.903	http://api.brain-map.org/grid_data/download/71608226?include=energy
Calm3	0.903	http://api.brain-map.org/grid_data/download/184?include=energy
Grik5	0.903	http://api.brain-map.org/grid_data/download/75551459?include=energy
Pof1b	0.903	http://api.brain-map.org/grid_data/download/74635433?include=energy
Gabre	0.903	http://api.brain-map.org/grid_data/download/75749417?include=energy
Dgcr14	0.903	http://api.brain-map.org/grid_data/download/69059822?include=energy
4732465J04Rik	0.903	http://api.brain-map.org/grid_data/download/74431354?include=energy
Odz1	0.903	http://api.brain-map.org/grid_data/download/77924494?include=energy
2810459M11Rik	0.903	http://api.brain-map.org/grid_data/download/69407307?include=energy
4933416I08Rik	0.903	http://api.brain-map.org/grid_data/download/70595817?include=energy
Tbp	0.903	http://api.brain-map.org/grid_data/download/67850950?include=energy
Ces3b	0.903	http://api.brain-map.org/grid_data/download/68445901?include=energy
Ubr7	0.903	http://api.brain-map.org/grid_data/download/69443469?include=energy
Efemp1	0.903	http://api.brain-map.org/grid_data/download/71724468?include=energy
Vmn1r24	0.903	http://api.brain-map.org/grid_data/download/70785236?include=energy
Tspan32	0.903	http://api.brain-map.org/grid_data/download/72129242?include=energy
Eid1	0.903	http://api.brain-map.org/grid_data/download/74583118?include=energy
Dgcr8	0.903	http://api.brain-map.org/grid_data/download/77620455?include=energy
LOC544793	0.903	http://api.brain-map.org/grid_data/download/73712798?include=energy
Pgm2l1	0.903	http://api.brain-map.org/grid_data/download/70615597?include=energy
2410002F23Rik	0.903	http://api.brain-map.org/grid_data/download/76098387?include=energy
Slc25a20	0.903	http://api.brain-map.org/grid_data/download/68638005?include=energy
Myl7	0.903	http://api.brain-map.org/grid_data/download/71992037?include=energy
Slc25a24	0.903	http://api.brain-map.org/grid_data/download/68668036?include=energy
Top1	0.903	http://api.brain-map.org/grid_data/download/74957897?include=energy
Arpc5	0.903	http://api.brain-map.org/grid_data/download/69526898?include=energy
Pdk2	0.903	http://api.brain-map.org/grid_data/download/68797489?include=energy
Ciapin1	0.902	http://api.brain-map.org/grid_data/download/68193065?include=energy
Nxf2	0.902	http://api.brain-map.org/grid_data/download/74580008?include=energy
Imp4	0.902	http://api.brain-map.org/grid_data/download/69608857?include=energy
Iqch	0.902	http://api.brain-map.org/grid_data/download/76003376?include=energy
Car8	0.902	http://api.brain-map.org/grid_data/download/191?include=energy
Olfr678	0.902	http://api.brain-map.org/grid_data/download/74734830?include=energy
Ogfrl1	0.902	http://api.brain-map.org/grid_data/download/73818756?include=energy
R3hcc1	0.902	http://api.brain-map.org/grid_data/download/69353614?include=energy
Dnalc4	0.902	http://api.brain-map.org/grid_data/download/74047778?include=energy
S1pr1	0.902	http://api.brain-map.org/grid_data/download/75043142?include=energy
Gm5486	0.902	http://api.brain-map.org/grid_data/download/74514288?include=energy
Gm5886	0.902	http://api.brain-map.org/grid_data/download/71161090?include=energy
Slc35a5	0.902	http://api.brain-map.org/grid_data/download/74990491?include=energy
Recql5	0.902	http://api.brain-map.org/grid_data/download/75079799?include=energy
Tnp2	0.902	http://api.brain-map.org/grid_data/download/69981401?include=energy
Apbb3	0.902	http://api.brain-map.org/grid_data/download/77371771?include=energy
Tspan6	0.902	http://api.brain-map.org/grid_data/download/74047839?include=energy
F730011J02	0.902	http://api.brain-map.org/grid_data/download/69114974?include=energy
Nsl1	0.902	http://api.brain-map.org/grid_data/download/77800913?include=energy
D7Ertd413e	0.902	http://api.brain-map.org/grid_data/download/70946390?include=energy
Zfp574	0.902	http://api.brain-map.org/grid_data/download/69262118?include=energy
Olfr1263	0.902	http://api.brain-map.org/grid_data/download/74720669?include=energy
Psmd10	0.902	http://api.brain-map.org/grid_data/download/68637455?include=energy
4933436I01Rik	0.902	http://api.brain-map.org/grid_data/download/71378687?include=energy
Kank2	0.902	http://api.brain-map.org/grid_data/download/112644736?include=energy
Dkk4	0.902	http://api.brain-map.org/grid_data/download/112647536?include=energy
Tas1r1	0.902	http://api.brain-map.org/grid_data/download/71488846?include=energy
Psmc1	0.902	http://api.brain-map.org/grid_data/download/71764207?include=energy
Psmd6	0.902	http://api.brain-map.org/grid_data/download/68269288?include=energy
C630004H02Rik	0.902	http://api.brain-map.org/grid_data/download/532674?include=energy
Mob3a	0.902	http://api.brain-map.org/grid_data/download/74059211?include=energy
Gm14140	0.902	http://api.brain-map.org/grid_data/download/70595829?include=energy
Ddx18	0.902	http://api.brain-map.org/grid_data/download/69261418?include=energy
Sirt7	0.902	http://api.brain-map.org/grid_data/download/68162272?include=energy
Dom3z	0.902	http://api.brain-map.org/grid_data/download/532640?include=energy
Gm4801	0.902	http://api.brain-map.org/grid_data/download/70565963?include=energy

Slc29a4	0.902	http://api.brain-map.org/grid_data/download/77414149?include=energy
Ccdc64	0.902	http://api.brain-map.org/grid_data/download/71492958?include=energy
Olfr975	0.902	http://api.brain-map.org/grid_data/download/74277398?include=energy
Larp4b	0.902	http://api.brain-map.org/grid_data/download/69816541?include=energy
Erc1	0.902	http://api.brain-map.org/grid_data/download/73424059?include=energy
A630095E13Rik	0.902	http://api.brain-map.org/grid_data/download/74359634?include=energy
Pcnp	0.902	http://api.brain-map.org/grid_data/download/73514797?include=energy
4930544G11Rik	0.902	http://api.brain-map.org/grid_data/download/70218294?include=energy
Kcnip1	0.902	http://api.brain-map.org/grid_data/download/1760?include=energy
Atad2b	0.902	http://api.brain-map.org/grid_data/download/77904881?include=energy
Aff4	0.902	http://api.brain-map.org/grid_data/download/2363?include=energy
Chst5	0.902	http://api.brain-map.org/grid_data/download/69548927?include=energy
Fam40a	0.902	http://api.brain-map.org/grid_data/download/71613677?include=energy
Mm.78264	0.902	http://api.brain-map.org/grid_data/download/71210430?include=energy
Samsn1	0.902	http://api.brain-map.org/grid_data/download/925?include=energy
Mfsd3	0.902	http://api.brain-map.org/grid_data/download/638700?include=energy
Taco1	0.902	http://api.brain-map.org/grid_data/download/68795389?include=energy
Htr3b	0.902	http://api.brain-map.org/grid_data/download/74641318?include=energy
Pik3r3	0.902	http://api.brain-map.org/grid_data/download/74583163?include=energy
9930111J21Rik2	0.902	http://api.brain-map.org/grid_data/download/70228292?include=energy
Gtf3c2	0.902	http://api.brain-map.org/grid_data/download/7?include=energy
Zfp593	0.902	http://api.brain-map.org/grid_data/download/100146081?include=energy
Patz1	0.902	http://api.brain-map.org/grid_data/download/69134346?include=energy
Rimbp2	0.902	http://api.brain-map.org/grid_data/download/69514470?include=energy
Pgpep1	0.902	http://api.brain-map.org/grid_data/download/68799095?include=energy
Scarf1	0.902	http://api.brain-map.org/grid_data/download/71489788?include=energy
Arl6	0.902	http://api.brain-map.org/grid_data/download/583843?include=energy
Mrps10	0.902	http://api.brain-map.org/grid_data/download/69013188?include=energy
Il31	0.902	http://api.brain-map.org/grid_data/download/71919475?include=energy
Cdca5	0.902	http://api.brain-map.org/grid_data/download/70528515?include=energy
Coq2	0.901	http://api.brain-map.org/grid_data/download/69202346?include=energy
Nsmce2	0.901	http://api.brain-map.org/grid_data/download/71209976?include=energy
Shisa7	0.901	http://api.brain-map.org/grid_data/download/70429052?include=energy
Chuk	0.901	http://api.brain-map.org/grid_data/download/1685?include=energy
F630045J19Rik*	0.901	http://api.brain-map.org/grid_data/download/73748794?include=energy
Stx12	0.901	http://api.brain-map.org/grid_data/download/70928169?include=energy
Olfr571	0.901	http://api.brain-map.org/grid_data/download/73653452?include=energy
Fbxw5	0.901	http://api.brain-map.org/grid_data/download/393240?include=energy
Csnka2ip	0.901	http://api.brain-map.org/grid_data/download/70305604?include=energy
Sart1	0.901	http://api.brain-map.org/grid_data/download/76081907?include=energy
Rrm1	0.901	http://api.brain-map.org/grid_data/download/76098380?include=energy
Zc3h12c	0.901	http://api.brain-map.org/grid_data/download/70813047?include=energy
Slc13a5	0.901	http://api.brain-map.org/grid_data/download/75931518?include=energy
Zfp658	0.901	http://api.brain-map.org/grid_data/download/73513570?include=energy
Lrfn4	0.901	http://api.brain-map.org/grid_data/download/71613644?include=energy
Olfr729	0.901	http://api.brain-map.org/grid_data/download/74734896?include=energy
Sema4c	0.901	http://api.brain-map.org/grid_data/download/74821915?include=energy
Rcor3	0.901	http://api.brain-map.org/grid_data/download/68341758?include=energy
Mpped2	0.901	http://api.brain-map.org/grid_data/download/73497744?include=energy
Tex19.2	0.901	http://api.brain-map.org/grid_data/download/70205668?include=energy
Hyi	0.901	http://api.brain-map.org/grid_data/download/74581104?include=energy
Nmt1	0.901	http://api.brain-map.org/grid_data/download/76097709?include=energy
St3gal5	0.901	http://api.brain-map.org/grid_data/download/69873785?include=energy
Fbxl18	0.901	http://api.brain-map.org/grid_data/download/74277394?include=energy
Tmem66	0.901	http://api.brain-map.org/grid_data/download/70743884?include=energy
Smarca5	0.901	http://api.brain-map.org/grid_data/download/68799155?include=energy
Gm4746	0.901	http://api.brain-map.org/grid_data/download/74822597?include=energy
Vwa2	0.901	http://api.brain-map.org/grid_data/download/76003617?include=energy
LOC433503	0.901	http://api.brain-map.org/grid_data/download/70303725?include=energy
Olfr366	0.901	http://api.brain-map.org/grid_data/download/74724061?include=energy
Arfip2	0.901	http://api.brain-map.org/grid_data/download/74990537?include=energy
Rxrb	0.901	http://api.brain-map.org/grid_data/download/1916?include=energy
Syap1	0.901	http://api.brain-map.org/grid_data/download/1858?include=energy
Gm1645	0.901	http://api.brain-map.org/grid_data/download/70298579?include=energy
Dpp4	0.901	http://api.brain-map.org/grid_data/download/79568049?include=energy
Bet1l	0.901	http://api.brain-map.org/grid_data/download/641534?include=energy
Gbf1	0.901	http://api.brain-map.org/grid_data/download/73513780?include=energy
Wdr17	0.901	http://api.brain-map.org/grid_data/download/71763649?include=energy
Gcc1	0.901	http://api.brain-map.org/grid_data/download/77620824?include=energy
Pom121l12	0.901	http://api.brain-map.org/grid_data/download/70608604?include=energy
Arf1	0.901	http://api.brain-map.org/grid_data/download/77414125?include=energy
4932443I19	0.901	http://api.brain-map.org/grid_data/download/70305042?include=energy
Spa17	0.901	http://api.brain-map.org/grid_data/download/69117152?include=energy
Ankrd13d	0.901	http://api.brain-map.org/grid_data/download/69837504?include=energy
Efhd2	0.901	http://api.brain-map.org/grid_data/download/69671911?include=energy
Kcnk4	0.901	http://api.brain-map.org/grid_data/download/71488689?include=energy
Ube2e3	0.901	http://api.brain-map.org/grid_data/download/75651166?include=energy
Mm.208903	0.901	http://api.brain-map.org/grid_data/download/71152396?include=energy
Parva	0.901	http://api.brain-map.org/grid_data/download/75077213?include=energy
Spsb3	0.901	http://api.brain-map.org/grid_data/download/69980196?include=energy
Chrm5	0.901	http://api.brain-map.org/grid_data/download/75826557?include=energy
Cd180	0.901	http://api.brain-map.org/grid_data/download/76003681?include=energy
Arhgap6	0.901	http://api.brain-map.org/grid_data/download/71487025?include=energy
Hk2	0.901	http://api.brain-map.org/grid_data/download/69013672?include=energy
Nfic	0.901	http://api.brain-map.org/grid_data/download/74988734?include=energy
Ppwd1	0.901	http://api.brain-map.org/grid_data/download/76003375?include=energy
Psmd8	0.901	http://api.brain-map.org/grid_data/download/393265?include=energy
Ak2	0.901	http://api.brain-map.org/grid_data/download/75694367?include=energy
Sumo1	0.901	http://api.brain-map.org/grid_data/download/81600154?include=energy
4930584F24Rik	0.901	http://api.brain-map.org/grid_data/download/71891982?include=energy
Lrig3	0.901	http://api.brain-map.org/grid_data/download/75988235?include=energy
Hnrnph2	0.901	http://api.brain-map.org/grid_data/download/112649906?include=energy
Chi3l7	0.901	http://api.brain-map.org/grid_data/download/74735265?include=energy
Olfr15	0.901	http://api.brain-map.org/grid_data/download/74720868?include=energy
Park2	0.901	http://api.brain-map.org/grid_data/download/2462?include=energy
Mterfd1	0.901	http://api.brain-map.org/grid_data/download/74357595?include=energy
Tbk1	0.901	http://api.brain-map.org/grid_data/download/71020898?include=energy
Cenpt	0.901	http://api.brain-map.org/grid_data/download/69672677?include=energy
2310047B19Rik	0.901	http://api.brain-map.org/grid_data/download/70303785?include=energy
Pdcd2l	0.901	http://api.brain-map.org/grid_data/download/69444753?include=energy
Rps7	0.901	http://api.brain-map.org/grid_data/download/68076526?include=energy
C130044B04Rik*	0.901	http://api.brain-map.org/grid_data/download/73811621?include=energy
Rpp40	0.901	http://api.brain-map.org/grid_data/download/69015775?include=energy
LOC432425	0.901	http://api.brain-map.org/grid_data/download/74509476?include=energy
C2cd4a	0.9	http://api.brain-map.org/grid_data/download/74736093?include=energy
Olfr1321	0.9	http://api.brain-map.org/grid_data/download/74720705?include=energy
Fdx1	0.9	http://api.brain-map.org/grid_data/download/75988415?include=energy
Tmem180	0.9	http://api.brain-map.org/grid_data/download/69103499?include=energy
Lta	0.9	http://api.brain-map.org/grid_data/download/69736298?include=energy
Mef2b	0.9	http://api.brain-map.org/grid_data/download/68745558?include=energy
Tmem232	0.9	http://api.brain-map.org/grid_data/download/73497541?include=energy
Lrp5	0.9	http://api.brain-map.org/grid_data/download/653?include=energy
Dhrs2	0.9	http://api.brain-map.org/grid_data/download/70238848?include=energy
Slc1a2	0.9	http://api.brain-map.org/grid_data/download/73521823?include=energy
Bloc1s2	0.9	http://api.brain-map.org/grid_data/download/77620441?include=energy
4931406B18Rik	0.9	http://api.brain-map.org/grid_data/download/71808808?include=energy
Pitpnb	0.9	http://api.brain-map.org/grid_data/download/69015649?include=energy
4932429P05Rik	0.9	http://api.brain-map.org/grid_data/download/74514037?include=energy
Sp100	0.9	http://api.brain-map.org/grid_data/download/74431840?include=energy
Arv1	0.9	http://api.brain-map.org/grid_data/download/71919158?include=energy
4933421E11Rik	0.9	http://api.brain-map.org/grid_data/download/69443202?include=energy
Fut7	0.9	http://api.brain-map.org/grid_data/download/69672647?include=energy
Mm.123843	0.9	http://api.brain-map.org/grid_data/download/71148010?include=energy
Myg1	0.9	http://api.brain-map.org/grid_data/download/68545887?include=energy
Ntrk2	0.9	http://api.brain-map.org/grid_data/download/746?include=energy
A830080L01Rik	0.9	http://api.brain-map.org/grid_data/download/71161189?include=energy
Decr2	0.9	http://api.brain-map.org/grid_data/download/71924363?include=energy
Ep300	0.9	http://api.brain-map.org/grid_data/download/73497135?include=energy
Fhod1	0.9	http://api.brain-map.org/grid_data/download/69672456?include=energy
Spnb1	0.9	http://api.brain-map.org/grid_data/download/75144624?include=energy
LOC433875	0.9	http://api.brain-map.org/grid_data/download/70787038?include=energy
Fam179a	0.9	http://api.brain-map.org/grid_data/download/69407636?include=energy
TC1429758	0.9	http://api.brain-map.org/grid_data/download/70795367?include=energy
D430018E03Rik	0.9	http://api.brain-map.org/grid_data/download/71021165?include=energy
Pcgf3	0.9	http://api.brain-map.org/grid_data/download/69262320?include=energy
Kpna6	0.9	http://api.brain-map.org/grid_data/download/70919477?include=energy
Gltpd1	0.9	http://api.brain-map.org/grid_data/download/69080038?include=energy
Cts3	0.9	http://api.brain-map.org/grid_data/download/75931619?include=energy
Lrrc8a	0.9	http://api.brain-map.org/grid_data/download/69170204?include=energy
Os9	0.9	http://api.brain-map.org/grid_data/download/876?include=energy
Gale	0.9	http://api.brain-map.org/grid_data/download/68545342?include=energy
Phkg1	0.9	http://api.brain-map.org/grid_data/download/69104735?include=energy
Zbtb38	0.9	http://api.brain-map.org/grid_data/download/70228627?include=energy
Myt1l	0.9	http://api.brain-map.org/grid_data/download/71735078?include=energy
Gm381	0.9	http://api.brain-map.org/grid_data/download/70295697?include=energy
Ces2c	0.9	http://api.brain-map.org/grid_data/download/71627768?include=energy
Gm5094	0.9	http://api.brain-map.org/grid_data/download/70305227?include=energy
Golga1	0.9	http://api.brain-map.org/grid_data/download/68843799?include=energy
Gm205	0.9	http://api.brain-map.org/grid_data/download/76081014?include=energy
Uhrf1bp1l	0.9	http://api.brain-map.org/grid_data/download/69169355?include=energy
Cdkn2b	0.9	http://api.brain-map.org/grid_data/download/1476?include=energy
Arl4c	0.9	http://api.brain-map.org/grid_data/download/74988455?include=energy
Nceh1	0.9	http://api.brain-map.org/grid_data/download/74881744?include=energy
P2ry12	0.9	http://api.brain-map.org/grid_data/download/112203915?include=energy
Rfc4	0.9	http://api.brain-map.org/grid_data/download/67979048?include=energy
Hnf4g	0.9	http://api.brain-map.org/grid_data/download/74821608?include=energy
Pcsk6	0.9	http://api.brain-map.org/grid_data/download/75084491?include=energy
Asah1	0.9	http://api.brain-map.org/grid_data/download/413772?include=energy
Gpt	0.9	http://api.brain-map.org/grid_data/download/68632184?include=energy
AA467197	0.9	http://api.brain-map.org/grid_data/download/69116380?include=energy
Sh3gl2	0.9	http://api.brain-map.org/grid_data/download/74581397?include=energy
Tceal8	0.9	http://api.brain-map.org/grid_data/download/67767474?include=energy
LOC432888	0.9	http://api.brain-map.org/grid_data/download/70611365?include=energy
D930026C10Rik*	0.9	http://api.brain-map.org/grid_data/download/73834469?include=energy
Snx19	0.9	http://api.brain-map.org/grid_data/download/68844660?include=energy
Ly6d	0.9	http://api.brain-map.org/grid_data/download/71724474?include=energy
Mta1	0.9	http://api.brain-map.org/grid_data/download/71380423?include=energy
Fgf21	0.9	http://api.brain-map.org/grid_data/download/436?include=energy
Pecr	0.9	http://api.brain-map.org/grid_data/download/68341406?include=energy
Hsf4	0.9	http://api.brain-map.org/grid_data/download/75214735?include=energy
Fbxl16	0.9	http://api.brain-map.org/grid_data/download/100144433?include=energy
Mfsd1	0.9	http://api.brain-map.org/grid_data/download/77800917?include=energy
Fzd10	0.9	http://api.brain-map.org/grid_data/download/77464892?include=energy
Pot1a	0.9	http://api.brain-map.org/grid_data/download/76097731?include=energy
Tusc3	0.899	http://api.brain-map.org/grid_data/download/69134474?include=energy
Clptm1	0.899	http://api.brain-map.org/grid_data/download/544901?include=energy
Mll2	0.899	http://api.brain-map.org/grid_data/download/74059201?include=energy
Kif2a	0.899	http://api.brain-map.org/grid_data/download/71610830?include=energy
Col13a1	0.899	http://api.brain-map.org/grid_data/download/68499452?include=energy
Mrpl10	0.899	http://api.brain-map.org/grid_data/download/71654122?include=energy
Aldh7a1	0.899	http://api.brain-map.org/grid_data/download/68193021?include=energy
Caprin2	0.899	http://api.brain-map.org/grid_data/download/69838739?include=energy
Slc35f1	0.899	http://api.brain-map.org/grid_data/download/75084145?include=energy
Trappc5	0.899	http://api.brain-map.org/grid_data/download/68745372?include=energy
Lgals3	0.899	http://api.brain-map.org/grid_data/download/70295488?include=energy
Igsf9	0.899	http://api.brain-map.org/grid_data/download/68196921?include=energy
Pde4d	0.899	http://api.brain-map.org/grid_data/download/1259?include=energy
Mm.30811	0.899	http://api.brain-map.org/grid_data/download/74510320?include=energy
Zfand2a	0.899	http://api.brain-map.org/grid_data/download/68798121?include=energy
Evi5l	0.899	http://api.brain-map.org/grid_data/download/69080730?include=energy
Sfrp2	0.899	http://api.brain-map.org/grid_data/download/1830?include=energy
Lat	0.899	http://api.brain-map.org/grid_data/download/2367?include=energy
Rgn	0.899	http://api.brain-map.org/grid_data/download/100144117?include=energy
D630037F22Rik	0.899	http://api.brain-map.org/grid_data/download/73712888?include=energy
Asphd1	0.899	http://api.brain-map.org/grid_data/download/69449026?include=energy
5730455P16Rik	0.899	http://api.brain-map.org/grid_data/download/69013744?include=energy
Neu2	0.899	http://api.brain-map.org/grid_data/download/75084472?include=energy
Lrit1	0.899	http://api.brain-map.org/grid_data/download/68162205?include=energy
Eif2ak1	0.899	http://api.brain-map.org/grid_data/download/71160961?include=energy
Nudt17	0.899	http://api.brain-map.org/grid_data/download/74635487?include=energy
Crygb	0.899	http://api.brain-map.org/grid_data/download/68919874?include=energy
LOC383368	0.899	http://api.brain-map.org/grid_data/download/70725091?include=energy
Ifi30	0.899	http://api.brain-map.org/grid_data/download/69014398?include=energy
Cdk9	0.899	http://api.brain-map.org/grid_data/download/81599610?include=energy
1810011H11Rik	0.899	http://api.brain-map.org/grid_data/download/71920261?include=energy
Gm686	0.899	http://api.brain-map.org/grid_data/download/70298244?include=energy
Col1a2	0.899	http://api.brain-map.org/grid_data/download/76081922?include=energy
Jrkl	0.899	http://api.brain-map.org/grid_data/download/70719032?include=energy
Elf2	0.899	http://api.brain-map.org/grid_data/download/1349?include=energy
Sirt4	0.899	http://api.brain-map.org/grid_data/download/69816864?include=energy
Llgl2	0.899	http://api.brain-map.org/grid_data/download/68443525?include=energy
Tmem82	0.899	http://api.brain-map.org/grid_data/download/68499141?include=energy
Slc22a1	0.899	http://api.brain-map.org/grid_data/download/78534490?include=energy
Chrnb1	0.899	http://api.brain-map.org/grid_data/download/75888752?include=energy
Gm315	0.899	http://api.brain-map.org/grid_data/download/74514213?include=energy
Gtsf1	0.899	http://api.brain-map.org/grid_data/download/76081046?include=energy
Ube2j2	0.899	http://api.brain-map.org/grid_data/download/77620443?include=energy
Anxa9	0.899	http://api.brain-map.org/grid_data/download/69835635?include=energy
Flnc	0.899	http://api.brain-map.org/grid_data/download/70200884?include=energy
Mrps34	0.899	http://api.brain-map.org/grid_data/download/68499302?include=energy
Dag1	0.899	http://api.brain-map.org/grid_data/download/355892?include=energy
Pik3cd	0.899	http://api.brain-map.org/grid_data/download/70429351?include=energy
Ccdc120	0.899	http://api.brain-map.org/grid_data/download/71764095?include=energy
Rfpl4b	0.899	http://api.brain-map.org/grid_data/download/73617654?include=energy
Mm.24003	0.899	http://api.brain-map.org/grid_data/download/71152629?include=energy
Strn4	0.899	http://api.brain-map.org/grid_data/download/69887403?include=energy
Cyp2c38	0.899	http://api.brain-map.org/grid_data/download/70720259?include=energy
Bpifb4	0.899	http://api.brain-map.org/grid_data/download/74514375?include=energy
Tfg	0.899	http://api.brain-map.org/grid_data/download/68632063?include=energy
Brp44l	0.899	http://api.brain-map.org/grid_data/download/2067?include=energy
4933430H15Rik	0.899	http://api.brain-map.org/grid_data/download/71493717?include=energy
Camkv	0.899	http://api.brain-map.org/grid_data/download/70743867?include=energy
Fgf12	0.899	http://api.brain-map.org/grid_data/download/79567499?include=energy
Mapk12	0.899	http://api.brain-map.org/grid_data/download/75988492?include=energy
Xab2	0.899	http://api.brain-map.org/grid_data/download/69080055?include=energy
Mylk	0.899	http://api.brain-map.org/grid_data/download/74819252?include=energy
Fam134c	0.899	http://api.brain-map.org/grid_data/download/69838762?include=energy
Rap1gap2	0.899	http://api.brain-map.org/grid_data/download/74882606?include=energy
Cxcr1	0.899	http://api.brain-map.org/grid_data/download/71281698?include=energy
Ildr1	0.899	http://api.brain-map.org/grid_data/download/100142551?include=energy
Cul4a	0.899	http://api.brain-map.org/grid_data/download/75749399?include=energy
Fgl2	0.899	http://api.brain-map.org/grid_data/download/68631996?include=energy
Cd38	0.899	http://api.brain-map.org/grid_data/download/76081081?include=energy
Gnaq	0.899	http://api.brain-map.org/grid_data/download/71280511?include=energy
Pnlip	0.899	http://api.brain-map.org/grid_data/download/76081011?include=energy
Atp6v1e2	0.898	http://api.brain-map.org/grid_data/download/69116865?include=energy
Serpini2	0.898	http://api.brain-map.org/grid_data/download/75934520?include=energy
D330025C20Rik	0.898	http://api.brain-map.org/grid_data/download/74076042?include=energy
Ccdc130	0.898	http://api.brain-map.org/grid_data/download/71610879?include=energy
Gba2	0.898	http://api.brain-map.org/grid_data/download/70795835?include=energy
X99384	0.898	http://api.brain-map.org/grid_data/download/71670681?include=energy
Cyyr1	0.898	http://api.brain-map.org/grid_data/download/69608390?include=energy
Crisp1	0.898	http://api.brain-map.org/grid_data/download/68301921?include=energy
Chek2	0.898	http://api.brain-map.org/grid_data/download/70524285?include=energy
Gprc5a	0.898	http://api.brain-map.org/grid_data/download/77414145?include=energy
Rfwd3	0.898	http://api.brain-map.org/grid_data/download/67764932?include=energy
Hiatl1	0.898	http://api.brain-map.org/grid_data/download/77414177?include=energy
Clec1b	0.898	http://api.brain-map.org/grid_data/download/71280754?include=energy
Igfbp5	0.898	http://api.brain-map.org/grid_data/download/73592530?include=energy
Dll1	0.898	http://api.brain-map.org/grid_data/download/77454727?include=energy
Ncapd2	0.898	http://api.brain-map.org/grid_data/download/71613646?include=energy
Yod1	0.898	http://api.brain-map.org/grid_data/download/73606636?include=energy
Il17d	0.898	http://api.brain-map.org/grid_data/download/75990861?include=energy
A330009N23Rik	0.898	http://api.brain-map.org/grid_data/download/70228382?include=energy
Cd320	0.898	http://api.brain-map.org/grid_data/download/67792188?include=energy
Tiaf2	0.898	http://api.brain-map.org/grid_data/download/69980358?include=energy
Ddit4	0.898	http://api.brain-map.org/grid_data/download/71496423?include=energy

Ubap2	0.898	http://api.brain-map.org/grid_data/download/79536647?include=energy
Metap1	0.898	http://api.brain-map.org/grid_data/download/74427052?include=energy
Trio	0.898	http://api.brain-map.org/grid_data/download/71147821?include=energy
Nod1	0.898	http://api.brain-map.org/grid_data/download/75831732?include=energy
LOC434820	0.898	http://api.brain-map.org/grid_data/download/74431739?include=energy
Akt3	0.898	http://api.brain-map.org/grid_data/download/77465022?include=energy
Dazap1	0.898	http://api.brain-map.org/grid_data/download/324?include=energy
Stap1	0.898	http://api.brain-map.org/grid_data/download/69838622?include=energy
Hes2	0.898	http://api.brain-map.org/grid_data/download/70303588?include=energy
A730080H06Rik	0.898	http://api.brain-map.org/grid_data/download/69449002?include=energy
Atf7	0.898	http://api.brain-map.org/grid_data/download/75990529?include=energy
4930474M22Rik	0.898	http://api.brain-map.org/grid_data/download/71891790?include=energy
A830084F11Rik*	0.898	http://api.brain-map.org/grid_data/download/74000480?include=energy
Mrps35	0.898	http://api.brain-map.org/grid_data/download/68341908?include=energy
Icos	0.898	http://api.brain-map.org/grid_data/download/68744731?include=energy
Sycp2	0.898	http://api.brain-map.org/grid_data/download/73808492?include=energy
Tex28	0.898	http://api.brain-map.org/grid_data/download/70300126?include=energy
Isg20	0.898	http://api.brain-map.org/grid_data/download/68498696?include=energy
Synpo2l	0.898	http://api.brain-map.org/grid_data/download/69887517?include=energy
Dmrtb1	0.898	http://api.brain-map.org/grid_data/download/71307274?include=energy
Nfatc4	0.898	http://api.brain-map.org/grid_data/download/71656120?include=energy
Pdss1	0.898	http://api.brain-map.org/grid_data/download/532713?include=energy
Ufd1l	0.898	http://api.brain-map.org/grid_data/download/71724750?include=energy
mCG146224	0.898	http://api.brain-map.org/grid_data/download/71212428?include=energy
Ube2q2	0.898	http://api.brain-map.org/grid_data/download/68631838?include=energy
Ezh1	0.898	http://api.brain-map.org/grid_data/download/293890?include=energy
Usp21	0.898	http://api.brain-map.org/grid_data/download/2711?include=energy
Lipt2	0.898	http://api.brain-map.org/grid_data/download/69202908?include=energy
Mettl21a	0.898	http://api.brain-map.org/grid_data/download/67815554?include=energy
4930505H01Rik	0.898	http://api.brain-map.org/grid_data/download/73927540?include=energy
A430110N23Rik	0.898	http://api.brain-map.org/grid_data/download/70305281?include=energy
Idi1	0.897	http://api.brain-map.org/grid_data/download/413789?include=energy
Ranbp2	0.897	http://api.brain-map.org/grid_data/download/71210865?include=energy
Scmh1	0.897	http://api.brain-map.org/grid_data/download/68148760?include=energy
1700013G24Rik	0.897	http://api.brain-map.org/grid_data/download/70201259?include=energy
Peo1	0.897	http://api.brain-map.org/grid_data/download/75863331?include=energy
Hddc3	0.897	http://api.brain-map.org/grid_data/download/70598379?include=energy
Khsrp	0.897	http://api.brain-map.org/grid_data/download/69735747?include=energy
Pus3	0.897	http://api.brain-map.org/grid_data/download/71763565?include=energy
Golt1a	0.897	http://api.brain-map.org/grid_data/download/69170282?include=energy
Trhr2	0.897	http://api.brain-map.org/grid_data/download/71488864?include=energy
Gli3	0.897	http://api.brain-map.org/grid_data/download/1615?include=energy
4933436C20Rik	0.897	http://api.brain-map.org/grid_data/download/71892221?include=energy
Mdk	0.897	http://api.brain-map.org/grid_data/download/1227?include=energy
Sgsm3	0.897	http://api.brain-map.org/grid_data/download/67870081?include=energy
Pmepa1	0.897	http://api.brain-map.org/grid_data/download/74635331?include=energy
Cpeb3	0.897	http://api.brain-map.org/grid_data/download/70720256?include=energy
Lcor	0.897	http://api.brain-map.org/grid_data/download/71281147?include=energy
Dmd	0.897	http://api.brain-map.org/grid_data/download/69626915?include=energy
Ttc15	0.897	http://api.brain-map.org/grid_data/download/68498836?include=energy
Lasc1	0.897	http://api.brain-map.org/grid_data/download/70304994?include=energy
Optn	0.897	http://api.brain-map.org/grid_data/download/2452?include=energy
Zmym6	0.897	http://api.brain-map.org/grid_data/download/74277665?include=energy
Rassf3	0.897	http://api.brain-map.org/grid_data/download/71836799?include=energy
Ints8	0.897	http://api.brain-map.org/grid_data/download/68667067?include=energy
Mlec	0.897	http://api.brain-map.org/grid_data/download/73930854?include=energy
B3gnt1	0.897	http://api.brain-map.org/grid_data/download/77332102?include=energy
Pdilt	0.897	http://api.brain-map.org/grid_data/download/71919278?include=energy
2610021K21Rik	0.897	http://api.brain-map.org/grid_data/download/69202842?include=energy
Ccdc12	0.897	http://api.brain-map.org/grid_data/download/74990523?include=energy
Sycp3	0.897	http://api.brain-map.org/grid_data/download/73423714?include=energy
4930412C18Rik	0.897	http://api.brain-map.org/grid_data/download/70227784?include=energy
Mrgprb5	0.897	http://api.brain-map.org/grid_data/download/73635483?include=energy
Eftud1	0.897	http://api.brain-map.org/grid_data/download/70528629?include=energy
Leng9	0.897	http://api.brain-map.org/grid_data/download/75934933?include=energy
Orai3	0.897	http://api.brain-map.org/grid_data/download/70593113?include=energy
Baz2a	0.897	http://api.brain-map.org/grid_data/download/69288686?include=energy
Emp3	0.897	http://api.brain-map.org/grid_data/download/68076548?include=energy
Ppp2r2c	0.897	http://api.brain-map.org/grid_data/download/74882698?include=energy
Rif1	0.897	http://api.brain-map.org/grid_data/download/74277651?include=energy
6030468B19Rik	0.897	http://api.brain-map.org/grid_data/download/72076713?include=energy
Abca7	0.897	http://api.brain-map.org/grid_data/download/74431128?include=energy
Gpatch2	0.897	http://api.brain-map.org/grid_data/download/69734530?include=energy
Vmac	0.897	http://api.brain-map.org/grid_data/download/69526402?include=energy
4933430N04Rik	0.897	http://api.brain-map.org/grid_data/download/71892209?include=energy
4930550C14Rik	0.897	http://api.brain-map.org/grid_data/download/74821776?include=energy
Arid2	0.897	http://api.brain-map.org/grid_data/download/68444970?include=energy
Itgb1bp2	0.897	http://api.brain-map.org/grid_data/download/71724479?include=energy
Gal	0.897	http://api.brain-map.org/grid_data/download/70231997?include=energy
LOC210619	0.897	http://api.brain-map.org/grid_data/download/71281898?include=energy
Slx4	0.897	http://api.brain-map.org/grid_data/download/69529436?include=energy
Obfc2a	0.897	http://api.brain-map.org/grid_data/download/69443541?include=energy
Olfr1026	0.897	http://api.brain-map.org/grid_data/download/74720561?include=energy
Zfp524	0.897	http://api.brain-map.org/grid_data/download/76097728?include=energy
Slc36a4	0.897	http://api.brain-map.org/grid_data/download/1597?include=energy
Amn1	0.897	http://api.brain-map.org/grid_data/download/71789927?include=energy
Nupl1	0.897	http://api.brain-map.org/grid_data/download/68637533?include=energy
LOC238766	0.897	http://api.brain-map.org/grid_data/download/70725019?include=energy
7530422B04Rik	0.897	http://api.brain-map.org/grid_data/download/72182939?include=energy
Nudt9	0.897	http://api.brain-map.org/grid_data/download/2444?include=energy
1810043G02Rik	0.897	http://api.brain-map.org/grid_data/download/68269580?include=energy
Duoxa1	0.897	http://api.brain-map.org/grid_data/download/68637527?include=energy
Ppp1r37	0.897	http://api.brain-map.org/grid_data/download/69838752?include=energy
Tmem129	0.897	http://api.brain-map.org/grid_data/download/67764908?include=energy
9030425E11Rik	0.897	http://api.brain-map.org/grid_data/download/71924353?include=energy
Erh	0.897	http://api.brain-map.org/grid_data/download/79544842?include=energy
Prkd2	0.897	http://api.brain-map.org/grid_data/download/69524503?include=energy
Bnc1	0.897	http://api.brain-map.org/grid_data/download/75934930?include=energy
Pik3r6	0.897	http://api.brain-map.org/grid_data/download/69169935?include=energy
Fga	0.897	http://api.brain-map.org/grid_data/download/68442907?include=energy
LOC434412	0.897	http://api.brain-map.org/grid_data/download/71022292?include=energy
Olfr1105	0.897	http://api.brain-map.org/grid_data/download/74720597?include=energy
Gzf1	0.897	http://api.brain-map.org/grid_data/download/69290867?include=energy
4930546K05Rik	0.897	http://api.brain-map.org/grid_data/download/72472485?include=energy
Fam185a	0.897	http://api.brain-map.org/grid_data/download/71495716?include=energy
Lrwd1	0.896	http://api.brain-map.org/grid_data/download/69838515?include=energy
Rps6ka3	0.896	http://api.brain-map.org/grid_data/download/71670727?include=energy
4930444P10Rik	0.896	http://api.brain-map.org/grid_data/download/70218139?include=energy
Mageb16	0.896	http://api.brain-map.org/grid_data/download/68667174?include=energy
Cops3	0.896	http://api.brain-map.org/grid_data/download/69568932?include=energy
Prkcg	0.896	http://api.brain-map.org/grid_data/download/73521814?include=energy
Slc28a2	0.896	http://api.brain-map.org/grid_data/download/68743769?include=energy
Bpi	0.896	http://api.brain-map.org/grid_data/download/75931540?include=energy
Tcam1	0.896	http://api.brain-map.org/grid_data/download/69113811?include=energy
Harbi1	0.896	http://api.brain-map.org/grid_data/download/69608869?include=energy
Acox3	0.896	http://api.brain-map.org/grid_data/download/69262290?include=energy
Xlr4c	0.896	http://api.brain-map.org/grid_data/download/71670714?include=energy
Zswim7	0.896	http://api.brain-map.org/grid_data/download/69202627?include=energy
Rnase10	0.896	http://api.brain-map.org/grid_data/download/70218181?include=energy
Popdc3	0.896	http://api.brain-map.org/grid_data/download/79913219?include=energy
Lfng	0.896	http://api.brain-map.org/grid_data/download/100146093?include=energy
Cep164	0.896	http://api.brain-map.org/grid_data/download/71211338?include=energy
Atp4a	0.896	http://api.brain-map.org/grid_data/download/70528258?include=energy
Best1	0.896	http://api.brain-map.org/grid_data/download/70302992?include=energy
Gm1136	0.896	http://api.brain-map.org/grid_data/download/72472472?include=energy
Ffar1	0.896	http://api.brain-map.org/grid_data/download/69257564?include=energy
LOC432416	0.896	http://api.brain-map.org/grid_data/download/75931537?include=energy
4930430A15Rik	0.896	http://api.brain-map.org/grid_data/download/71283513?include=energy
Psors1c2	0.896	http://api.brain-map.org/grid_data/download/70300953?include=energy
Fam167b	0.896	http://api.brain-map.org/grid_data/download/68445949?include=energy
Odf3	0.896	http://api.brain-map.org/grid_data/download/72081646?include=energy
Btf3l4	0.896	http://api.brain-map.org/grid_data/download/68666738?include=energy
AI450948	0.896	http://api.brain-map.org/grid_data/download/71250311?include=energy
Arl11	0.896	http://api.brain-map.org/grid_data/download/69835570?include=energy
Gm1322	0.896	http://api.brain-map.org/grid_data/download/74431260?include=energy
LOC434088	0.896	http://api.brain-map.org/grid_data/download/70787396?include=energy
Smad6	0.896	http://api.brain-map.org/grid_data/download/74581134?include=energy
Cd99	0.896	http://api.brain-map.org/grid_data/download/75198142?include=energy
Spock1	0.896	http://api.brain-map.org/grid_data/download/72340122?include=energy
Frem2	0.896	http://api.brain-map.org/grid_data/download/73513762?include=energy
P2rx4	0.896	http://api.brain-map.org/grid_data/download/75551474?include=energy
4932425I24Rik	0.896	http://api.brain-map.org/grid_data/download/70305024?include=energy
A830080D01Rik	0.896	http://api.brain-map.org/grid_data/download/72283557?include=energy
LOC436156	0.896	http://api.brain-map.org/grid_data/download/70784588?include=energy
Acot11	0.896	http://api.brain-map.org/grid_data/download/68845388?include=energy
Dppa4	0.896	http://api.brain-map.org/grid_data/download/68743853?include=energy
Fam38a	0.896	http://api.brain-map.org/grid_data/download/68443399?include=energy
1700012A16Rik	0.896	http://api.brain-map.org/grid_data/download/100144861?include=energy
Sf3b5	0.896	http://api.brain-map.org/grid_data/download/69059832?include=energy
Fam82b	0.896	http://api.brain-map.org/grid_data/download/75773702?include=energy
Atp5k	0.896	http://api.brain-map.org/grid_data/download/74426449?include=energy
Gm471	0.896	http://api.brain-map.org/grid_data/download/74735325?include=energy
Myh13	0.896	http://api.brain-map.org/grid_data/download/74514192?include=energy
B230396K10Rik	0.896	http://api.brain-map.org/grid_data/download/69527878?include=energy
Pim2	0.896	http://api.brain-map.org/grid_data/download/71608212?include=energy
Maff	0.896	http://api.brain-map.org/grid_data/download/68498343?include=energy
Ap4s1	0.896	http://api.brain-map.org/grid_data/download/513248?include=energy
Tmem115	0.896	http://api.brain-map.org/grid_data/download/601187?include=energy
Aadat	0.896	http://api.brain-map.org/grid_data/download/68301252?include=energy
Arfip1	0.896	http://api.brain-map.org/grid_data/download/71495795?include=energy
LOC433322	0.896	http://api.brain-map.org/grid_data/download/70723098?include=energy
Zc3h11a	0.895	http://api.brain-map.org/grid_data/download/68269291?include=energy
Kcnc3	0.895	http://api.brain-map.org/grid_data/download/71670481?include=energy
1700112M01Rik	0.895	http://api.brain-map.org/grid_data/download/74635150?include=energy
Rnaseh2a	0.895	http://api.brain-map.org/grid_data/download/68546119?include=energy
D930015E06Rik	0.895	http://api.brain-map.org/grid_data/download/77924472?include=energy
Olfr705	0.895	http://api.brain-map.org/grid_data/download/73653494?include=energy
4921517L17Rik	0.895	http://api.brain-map.org/grid_data/download/67779872?include=energy
Sox18	0.895	http://api.brain-map.org/grid_data/download/119681?include=energy
Myo1h	0.895	http://api.brain-map.org/grid_data/download/100145307?include=energy
LOC433109	0.895	http://api.brain-map.org/grid_data/download/70722922?include=energy
Shmt1	0.895	http://api.brain-map.org/grid_data/download/68562951?include=energy
Cyp19a1	0.895	http://api.brain-map.org/grid_data/download/71381001?include=energy
Inpp4a	0.895	http://api.brain-map.org/grid_data/download/71668413?include=energy
Arpc1b	0.895	http://api.brain-map.org/grid_data/download/67979031?include=energy
Scrn2	0.895	http://api.brain-map.org/grid_data/download/68445149?include=energy
Naga	0.895	http://api.brain-map.org/grid_data/download/68301186?include=energy
Nhp2l1	0.895	http://api.brain-map.org/grid_data/download/68633125?include=energy
Il1f10	0.895	http://api.brain-map.org/grid_data/download/76081007?include=energy
Tmem147	0.895	http://api.brain-map.org/grid_data/download/68546386?include=energy
Krt7	0.895	http://api.brain-map.org/grid_data/download/68162221?include=energy
Ttc38	0.895	http://api.brain-map.org/grid_data/download/70719011?include=energy
Uroc1	0.895	http://api.brain-map.org/grid_data/download/68410500?include=energy
Fam169a	0.895	http://api.brain-map.org/grid_data/download/70793953?include=energy
Zfp143	0.895	http://api.brain-map.org/grid_data/download/69059870?include=energy
Smyd5	0.895	http://api.brain-map.org/grid_data/download/70813389?include=energy
Trmt5	0.895	http://api.brain-map.org/grid_data/download/68546422?include=energy
Gfm1	0.895	http://api.brain-map.org/grid_data/download/386242?include=energy
Itln1	0.895	http://api.brain-map.org/grid_data/download/81655576?include=energy
LOC435908	0.895	http://api.brain-map.org/grid_data/download/71022621?include=energy
Grip2	0.895	http://api.brain-map.org/grid_data/download/112646245?include=energy
Nat6	0.895	http://api.brain-map.org/grid_data/download/1780?include=energy
Rbmxl1	0.895	http://api.brain-map.org/grid_data/download/76081868?include=energy
Tet2	0.895	http://api.brain-map.org/grid_data/download/70542956?include=energy
Btnl10	0.895	http://api.brain-map.org/grid_data/download/76003336?include=energy
C130040D06Rik	0.895	http://api.brain-map.org/grid_data/download/69530804?include=energy
Fndc1	0.895	http://api.brain-map.org/grid_data/download/73992908?include=energy
Arpc3	0.895	http://api.brain-map.org/grid_data/download/68193057?include=energy
Kcnk5	0.895	http://api.brain-map.org/grid_data/download/69028456?include=energy
Hepacam2	0.895	http://api.brain-map.org/grid_data/download/69526438?include=energy
Zfp944	0.895	http://api.brain-map.org/grid_data/download/73496899?include=energy
Ear11	0.895	http://api.brain-map.org/grid_data/download/68341776?include=energy
Coq10a	0.895	http://api.brain-map.org/grid_data/download/71211413?include=energy
Gm589	0.895	http://api.brain-map.org/grid_data/download/70295972?include=energy
Lphn2	0.895	http://api.brain-map.org/grid_data/download/1768?include=energy
Trerf1	0.895	http://api.brain-map.org/grid_data/download/75651165?include=energy
Srr	0.895	http://api.brain-map.org/grid_data/download/74357621?include=energy
Git2	0.895	http://api.brain-map.org/grid_data/download/68844325?include=energy
Kirrel3	0.895	http://api.brain-map.org/grid_data/download/71920516?include=energy
3010026O09Rik	0.895	http://api.brain-map.org/grid_data/download/75695641?include=energy
Bpifa5	0.895	http://api.brain-map.org/grid_data/download/74635388?include=energy
Zfp235	0.895	http://api.brain-map.org/grid_data/download/69290824?include=energy
Snx13	0.895	http://api.brain-map.org/grid_data/download/69885612?include=energy
Per1	0.895	http://api.brain-map.org/grid_data/download/791?include=energy
Olfr684	0.895	http://api.brain-map.org/grid_data/download/74734842?include=energy
1700024G13Rik	0.895	http://api.brain-map.org/grid_data/download/69353651?include=energy
Anks3	0.895	http://api.brain-map.org/grid_data/download/69113662?include=energy
Gtf2a1	0.895	http://api.brain-map.org/grid_data/download/70303576?include=energy
Ptgis	0.895	http://api.brain-map.org/grid_data/download/69817649?include=energy
Plekhg4	0.895	http://api.brain-map.org/grid_data/download/73514827?include=energy
4933407H18Rik	0.895	http://api.brain-map.org/grid_data/download/73606615?include=energy
Adamts10	0.895	http://api.brain-map.org/grid_data/download/71495659?include=energy
Hoxa4	0.895	http://api.brain-map.org/grid_data/download/81600124?include=energy
Zfp948	0.895	http://api.brain-map.org/grid_data/download/77932825?include=energy
Apol11b	0.895	http://api.brain-map.org/grid_data/download/70724505?include=energy
Gm136	0.895	http://api.brain-map.org/grid_data/download/70295709?include=energy
Ttc25	0.895	http://api.brain-map.org/grid_data/download/74988582?include=energy
D130084M03Rik	0.895	http://api.brain-map.org/grid_data/download/71809397?include=energy
0610040J01Rik	0.895	http://api.brain-map.org/grid_data/download/69079986?include=energy
Scn7a	0.895	http://api.brain-map.org/grid_data/download/71325432?include=energy
Ptp4a2	0.895	http://api.brain-map.org/grid_data/download/71280538?include=energy
Ocrl	0.895	http://api.brain-map.org/grid_data/download/69134334?include=energy
Trim65	0.895	http://api.brain-map.org/grid_data/download/69407666?include=energy
Lrrc18	0.895	http://api.brain-map.org/grid_data/download/69116787?include=energy
Neu4	0.895	http://api.brain-map.org/grid_data/download/100144896?include=energy
Vsig8	0.895	http://api.brain-map.org/grid_data/download/71569157?include=energy
H28	0.894	http://api.brain-map.org/grid_data/download/71723796?include=energy
Tm9sf3	0.894	http://api.brain-map.org/grid_data/download/413788?include=energy
Nek7	0.894	http://api.brain-map.org/grid_data/download/72339542?include=energy
Rab2a	0.894	http://api.brain-map.org/grid_data/download/79394344?include=energy
LOC386546	0.894	http://api.brain-map.org/grid_data/download/74736171?include=energy
Dnahc10	0.894	http://api.brain-map.org/grid_data/download/71587553?include=energy
4933428M09Rik	0.894	http://api.brain-map.org/grid_data/download/75990429?include=energy
Slpi	0.894	http://api.brain-map.org/grid_data/download/75828185?include=energy
Fbxo8	0.894	http://api.brain-map.org/grid_data/download/68545812?include=energy
Plekhg6	0.894	http://api.brain-map.org/grid_data/download/69169732?include=energy
Tmlhe	0.894	http://api.brain-map.org/grid_data/download/68302227?include=energy
4933432B09Rik	0.894	http://api.brain-map.org/grid_data/download/74580757?include=energy
Slc2a4	0.894	http://api.brain-map.org/grid_data/download/2599?include=energy
Gal3st4	0.894	http://api.brain-map.org/grid_data/download/69672731?include=energy
Cenpk	0.894	http://api.brain-map.org/grid_data/download/74635824?include=energy
Slc2a13	0.894	http://api.brain-map.org/grid_data/download/74958626?include=energy
Mll1	0.894	http://api.brain-map.org/grid_data/download/69289375?include=energy
Pdcl3	0.894	http://api.brain-map.org/grid_data/download/69782915?include=energy
Rs1	0.894	http://api.brain-map.org/grid_data/download/69262289?include=energy
Ghrh	0.894	http://api.brain-map.org/grid_data/download/496?include=energy
A230057D06Rik	0.894	http://api.brain-map.org/grid_data/download/69447942?include=energy
Vim	0.894	http://api.brain-map.org/grid_data/download/1309?include=energy
Olfr564	0.894	http://api.brain-map.org/grid_data/download/74724193?include=energy
Bzw2	0.894	http://api.brain-map.org/grid_data/download/69103487?include=energy
Itga10	0.894	http://api.brain-map.org/grid_data/download/76003676?include=energy
Gm536	0.894	http://api.brain-map.org/grid_data/download/74510224?include=energy
Actr8	0.894	http://api.brain-map.org/grid_data/download/67764913?include=energy
Tacstd2	0.894	http://api.brain-map.org/grid_data/download/69980148?include=energy
L3mbtl1	0.894	http://api.brain-map.org/grid_data/download/69289309?include=energy
Rb1cc1	0.894	http://api.brain-map.org/grid_data/download/75043159?include=energy
AW146020	0.894	http://api.brain-map.org/grid_data/download/71283662?include=energy

Dnase1l3	0.894	http://api.brain-map.org/grid_data/download/68301246?include=energy
Pex12	0.894	http://api.brain-map.org/grid_data/download/75695631?include=energy
Cd300lg	0.894	http://api.brain-map.org/grid_data/download/71580162?include=energy
Zbtb4	0.894	http://api.brain-map.org/grid_data/download/70303034?include=energy
Osbpl2	0.894	http://api.brain-map.org/grid_data/download/532701?include=energy
Ngrn	0.894	http://api.brain-map.org/grid_data/download/513242?include=energy
Hccs	0.894	http://api.brain-map.org/grid_data/download/77799007?include=energy
Iglon5	0.894	http://api.brain-map.org/grid_data/download/71380538?include=energy
Pskh1	0.894	http://api.brain-map.org/grid_data/download/68667342?include=energy
Stard7	0.894	http://api.brain-map.org/grid_data/download/74800953?include=energy
Atp6v1g1	0.894	http://api.brain-map.org/grid_data/download/73513474?include=energy
A630007B06Rik	0.894	http://api.brain-map.org/grid_data/download/72283551?include=energy
Ddr2	0.894	http://api.brain-map.org/grid_data/download/74882459?include=energy
LOC333872	0.894	http://api.brain-map.org/grid_data/download/74736111?include=energy
Clec4n	0.894	http://api.brain-map.org/grid_data/download/71656062?include=energy
G2e3	0.894	http://api.brain-map.org/grid_data/download/69444741?include=energy
Ntpcr	0.894	http://api.brain-map.org/grid_data/download/75773704?include=energy
Klc4	0.894	http://api.brain-map.org/grid_data/download/73514713?include=energy
Ngef	0.894	http://api.brain-map.org/grid_data/download/72129292?include=energy
9130017N09Rik	0.894	http://api.brain-map.org/grid_data/download/74056118?include=energy
Khdrbs2	0.894	http://api.brain-map.org/grid_data/download/74000570?include=energy
Lactb2	0.894	http://api.brain-map.org/grid_data/download/513475?include=energy
Kctd14	0.894	http://api.brain-map.org/grid_data/download/70542941?include=energy
Rbm12b	0.894	http://api.brain-map.org/grid_data/download/68798513?include=energy
Apba2	0.894	http://api.brain-map.org/grid_data/download/2031?include=energy
Dmgdh	0.894	http://api.brain-map.org/grid_data/download/69626921?include=energy
Rad18	0.893	http://api.brain-map.org/grid_data/download/564784?include=energy
Gm10517	0.893	http://api.brain-map.org/grid_data/download/73949566?include=energy
Kif3c	0.893	http://api.brain-map.org/grid_data/download/69735795?include=energy
BC068281	0.893	http://api.brain-map.org/grid_data/download/70946237?include=energy
Trp53	0.893	http://api.brain-map.org/grid_data/download/67855414?include=energy
Osbp2	0.893	http://api.brain-map.org/grid_data/download/68797487?include=energy
Rpgr	0.893	http://api.brain-map.org/grid_data/download/67936211?include=energy
B930072A22Rik*	0.893	http://api.brain-map.org/grid_data/download/74000618?include=energy
Dlx1	0.893	http://api.brain-map.org/grid_data/download/72008490?include=energy
Ube4a	0.893	http://api.brain-map.org/grid_data/download/68203459?include=energy
Tmprss5	0.893	http://api.brain-map.org/grid_data/download/74957887?include=energy
Adam17	0.893	http://api.brain-map.org/grid_data/download/75988237?include=energy
Tle1	0.893	http://api.brain-map.org/grid_data/download/1061?include=energy
Pik3cb	0.893	http://api.brain-map.org/grid_data/download/70300792?include=energy
Gm22	0.893	http://api.brain-map.org/grid_data/download/74988274?include=energy
Gm1127	0.893	http://api.brain-map.org/grid_data/download/70300001?include=energy
Ppp3ca	0.893	http://api.brain-map.org/grid_data/download/73817435?include=energy
Rhobtb3	0.893	http://api.brain-map.org/grid_data/download/74583162?include=energy
F830003B07	0.893	http://api.brain-map.org/grid_data/download/70724714?include=energy
Hnrnpa3	0.893	http://api.brain-map.org/grid_data/download/68161477?include=energy
Serpinb9d	0.893	http://api.brain-map.org/grid_data/download/72182940?include=energy
Armc7	0.893	http://api.brain-map.org/grid_data/download/67767463?include=energy
Spesp1	0.893	http://api.brain-map.org/grid_data/download/69116548?include=energy
BC027231	0.893	http://api.brain-map.org/grid_data/download/70445634?include=energy
1700023F06Rik	0.893	http://api.brain-map.org/grid_data/download/74357815?include=energy
Tars	0.893	http://api.brain-map.org/grid_data/download/68667805?include=energy
Rps17	0.893	http://api.brain-map.org/grid_data/download/71763583?include=energy
Atr	0.893	http://api.brain-map.org/grid_data/download/74562212?include=energy
Gm12474	0.893	http://api.brain-map.org/grid_data/download/75043653?include=energy
Fam175a	0.893	http://api.brain-map.org/grid_data/download/68545282?include=energy
LOC434236	0.893	http://api.brain-map.org/grid_data/download/73615572?include=energy
BC013529	0.893	http://api.brain-map.org/grid_data/download/68341513?include=energy
Guca1b	0.893	http://api.brain-map.org/grid_data/download/68341555?include=energy
Kat2a	0.893	http://api.brain-map.org/grid_data/download/70920039?include=energy
Rab11fip5	0.893	http://api.brain-map.org/grid_data/download/74059272?include=energy
Senp6	0.893	http://api.brain-map.org/grid_data/download/68499243?include=energy
Camsap2	0.893	http://api.brain-map.org/grid_data/download/70218283?include=energy
Amt	0.893	http://api.brain-map.org/grid_data/download/71211371?include=energy
Ube3c	0.893	http://api.brain-map.org/grid_data/download/71670707?include=energy
2900093K20Rik	0.893	http://api.brain-map.org/grid_data/download/74990479?include=energy
Nr2c2ap	0.893	http://api.brain-map.org/grid_data/download/74635583?include=energy
Higd1a	0.893	http://api.brain-map.org/grid_data/download/2291?include=energy
Brdt	0.893	http://api.brain-map.org/grid_data/download/74510260?include=energy
4930583K01Rik	0.893	http://api.brain-map.org/grid_data/download/70218432?include=energy
Celf1	0.893	http://api.brain-map.org/grid_data/download/631727?include=energy
Ppif	0.893	http://api.brain-map.org/grid_data/download/68269060?include=energy
C130046B21Rik	0.893	http://api.brain-map.org/grid_data/download/74277355?include=energy
Isoc2b	0.893	http://api.brain-map.org/grid_data/download/69262328?include=energy
LOC381765	0.893	http://api.brain-map.org/grid_data/download/74425521?include=energy
Ttc7	0.893	http://api.brain-map.org/grid_data/download/75831735?include=energy
A130023I24Rik	0.893	http://api.brain-map.org/grid_data/download/75693062?include=energy
LOC433758	0.893	http://api.brain-map.org/grid_data/download/70786761?include=energy
Abcg5	0.893	http://api.brain-map.org/grid_data/download/69514630?include=energy
Haus1	0.893	http://api.brain-map.org/grid_data/download/68499434?include=energy
LOC433081	0.893	http://api.brain-map.org/grid_data/download/70611497?include=energy
Nkx1-2	0.893	http://api.brain-map.org/grid_data/download/69289561?include=energy
2900006K08Rik	0.893	http://api.brain-map.org/grid_data/download/73497765?include=energy
Snapc2	0.893	http://api.brain-map.org/grid_data/download/79568055?include=energy
9830168E02Rik*	0.893	http://api.brain-map.org/grid_data/download/73906227?include=energy
Guca1a	0.893	http://api.brain-map.org/grid_data/download/68797488?include=energy
Wdr1	0.893	http://api.brain-map.org/grid_data/download/75198154?include=energy
1500011H22Rik	0.893	http://api.brain-map.org/grid_data/download/70445413?include=energy
Tmem144	0.893	http://api.brain-map.org/grid_data/download/583855?include=energy
Pramef8	0.893	http://api.brain-map.org/grid_data/download/68918703?include=energy
Dlg3	0.893	http://api.brain-map.org/grid_data/download/71496441?include=energy
Cdkal1	0.893	http://api.brain-map.org/grid_data/download/638714?include=energy
Atg16l1	0.892	http://api.brain-map.org/grid_data/download/69526761?include=energy
Cdhr3	0.892	http://api.brain-map.org/grid_data/download/74272042?include=energy
Ndrg3	0.892	http://api.brain-map.org/grid_data/download/690?include=energy
Senp5	0.892	http://api.brain-map.org/grid_data/download/74821925?include=energy
C630028L02Rik	0.892	http://api.brain-map.org/grid_data/download/76003513?include=energy
Ccl25	0.892	http://api.brain-map.org/grid_data/download/73811543?include=energy
Eif1a	0.892	http://api.brain-map.org/grid_data/download/69059848?include=energy
Ccdc101	0.892	http://api.brain-map.org/grid_data/download/69549279?include=energy
Ufm1	0.892	http://api.brain-map.org/grid_data/download/72129283?include=energy
Mfap1a	0.892	http://api.brain-map.org/grid_data/download/74988551?include=energy
Trps1	0.892	http://api.brain-map.org/grid_data/download/2697?include=energy
A630031M04Rik	0.892	http://api.brain-map.org/grid_data/download/69448924?include=energy
Stx17	0.892	http://api.brain-map.org/grid_data/download/68523174?include=energy
Hhatl	0.892	http://api.brain-map.org/grid_data/download/80514103?include=energy
Ern1	0.892	http://api.brain-map.org/grid_data/download/71021171?include=energy
Spg20	0.892	http://api.brain-map.org/grid_data/download/70919376?include=energy
Bap1	0.892	http://api.brain-map.org/grid_data/download/74881827?include=energy
Tmem131	0.892	http://api.brain-map.org/grid_data/download/74882216?include=energy
LOC546014	0.892	http://api.brain-map.org/grid_data/download/77620843?include=energy
Dbpht2	0.892	http://api.brain-map.org/grid_data/download/71496417?include=energy
Tax1bp1	0.892	http://api.brain-map.org/grid_data/download/68300890?include=energy
Prelid2	0.892	http://api.brain-map.org/grid_data/download/70231929?include=energy
Ddx21	0.892	http://api.brain-map.org/grid_data/download/71656806?include=energy
Dnaja1	0.892	http://api.brain-map.org/grid_data/download/70528611?include=energy
Kri1	0.892	http://api.brain-map.org/grid_data/download/68445658?include=energy
Shmt2	0.892	http://api.brain-map.org/grid_data/download/71724690?include=energy
Rapgefl1	0.892	http://api.brain-map.org/grid_data/download/70305419?include=energy
Clcn7	0.892	http://api.brain-map.org/grid_data/download/71307256?include=energy
Insig2	0.892	http://api.brain-map.org/grid_data/download/70525854?include=energy
Cyp2ab1	0.892	http://api.brain-map.org/grid_data/download/71587637?include=energy
Rbmx2	0.892	http://api.brain-map.org/grid_data/download/68151437?include=energy
A630005I04Rik	0.892	http://api.brain-map.org/grid_data/download/70228519?include=energy
Fam78a	0.892	http://api.brain-map.org/grid_data/download/70228370?include=energy
Kif3a	0.892	http://api.brain-map.org/grid_data/download/71529995?include=energy
A630076J17Rik	0.892	http://api.brain-map.org/grid_data/download/70228573?include=energy
Slc1a5	0.892	http://api.brain-map.org/grid_data/download/119701?include=energy
Sema4b	0.892	http://api.brain-map.org/grid_data/download/69873522?include=energy
Tdrd3	0.892	http://api.brain-map.org/grid_data/download/73817944?include=energy
Lace1	0.892	http://api.brain-map.org/grid_data/download/76003679?include=energy
Pla2g5	0.892	http://api.brain-map.org/grid_data/download/71668997?include=energy
Ncoa4	0.892	http://api.brain-map.org/grid_data/download/1782?include=energy
1110058L19Rik	0.892	http://api.brain-map.org/grid_data/download/68441574?include=energy
Eapp	0.892	http://api.brain-map.org/grid_data/download/68795424?include=energy
LOC545352	0.892	http://api.brain-map.org/grid_data/download/74988758?include=energy
Faf2	0.892	http://api.brain-map.org/grid_data/download/71492964?include=energy
Gria3	0.892	http://api.brain-map.org/grid_data/download/71247616?include=energy
Mgarp	0.892	http://api.brain-map.org/grid_data/download/68341543?include=energy
Ube2z	0.892	http://api.brain-map.org/grid_data/download/69013708?include=energy
2400003C14Rik	0.892	http://api.brain-map.org/grid_data/download/76098327?include=energy
1700056E22Rik	0.892	http://api.brain-map.org/grid_data/download/74635054?include=energy
Sall4	0.892	http://api.brain-map.org/grid_data/download/70593342?include=energy
Lmod2	0.892	http://api.brain-map.org/grid_data/download/73636019?include=energy
Tex19.1	0.892	http://api.brain-map.org/grid_data/download/68667354?include=energy
Tmem9	0.892	http://api.brain-map.org/grid_data/download/583862?include=energy
Irs1	0.892	http://api.brain-map.org/grid_data/download/69735532?include=energy
1810014F10Rik	0.892	http://api.brain-map.org/grid_data/download/69549177?include=energy
Bcar3	0.892	http://api.brain-map.org/grid_data/download/77371769?include=energy
B930086A06Rik	0.892	http://api.brain-map.org/grid_data/download/71495915?include=energy
Dcp1b	0.892	http://api.brain-map.org/grid_data/download/69528016?include=energy
Gnb1l	0.892	http://api.brain-map.org/grid_data/download/77800921?include=energy
1500011B03Rik	0.892	http://api.brain-map.org/grid_data/download/71212087?include=energy
Klhdc8b	0.892	http://api.brain-map.org/grid_data/download/69133698?include=energy
Necap2	0.892	http://api.brain-map.org/grid_data/download/68191318?include=energy
Ppara	0.892	http://api.brain-map.org/grid_data/download/77454629?include=energy
9430031J16Rik	0.892	http://api.brain-map.org/grid_data/download/71281235?include=energy
Cyp4f16	0.891	http://api.brain-map.org/grid_data/download/69173298?include=energy
Eme1	0.891	http://api.brain-map.org/grid_data/download/70305126?include=energy
Opn3	0.891	http://api.brain-map.org/grid_data/download/71020433?include=energy
Tmem204	0.891	http://api.brain-map.org/grid_data/download/73769320?include=energy
Tacc1	0.891	http://api.brain-map.org/grid_data/download/74579951?include=energy
Prl8a1	0.891	http://api.brain-map.org/grid_data/download/75931611?include=energy
Ate1	0.891	http://api.brain-map.org/grid_data/download/69549622?include=energy
4933403F05Rik	0.891	http://api.brain-map.org/grid_data/download/74819246?include=energy
Prr15	0.891	http://api.brain-map.org/grid_data/download/71496510?include=energy
Zfp36l1	0.891	http://api.brain-map.org/grid_data/download/70445341?include=energy
TC1469211	0.891	http://api.brain-map.org/grid_data/download/70795943?include=energy
Mia3	0.891	http://api.brain-map.org/grid_data/download/70228657?include=energy
Snx17	0.891	http://api.brain-map.org/grid_data/download/68910969?include=energy
Lor	0.891	http://api.brain-map.org/grid_data/download/79904796?include=energy
Rhod	0.891	http://api.brain-map.org/grid_data/download/68797930?include=energy
Spry2	0.891	http://api.brain-map.org/grid_data/download/69885720?include=energy
Glt8d1	0.891	http://api.brain-map.org/grid_data/download/69202615?include=energy
Sec16b	0.891	http://api.brain-map.org/grid_data/download/69015003?include=energy
2010321M09Rik	0.891	http://api.brain-map.org/grid_data/download/75774647?include=energy
5430437P03Rik	0.891	http://api.brain-map.org/grid_data/download/71992019?include=energy
Tsga10ip	0.891	http://api.brain-map.org/grid_data/download/100144114?include=energy
Copz1	0.891	http://api.brain-map.org/grid_data/download/385996?include=energy
Ceacam20	0.891	http://api.brain-map.org/grid_data/download/75990660?include=energy
Lpin3	0.891	http://api.brain-map.org/grid_data/download/69736203?include=energy
2410002I01Rik	0.891	http://api.brain-map.org/grid_data/download/70542865?include=energy
Osm	0.891	http://api.brain-map.org/grid_data/download/71808765?include=energy
Gsdmcl-ps	0.891	http://api.brain-map.org/grid_data/download/74580745?include=energy
Sox17	0.891	http://api.brain-map.org/grid_data/download/81657677?include=energy
Ccdc135	0.891	http://api.brain-map.org/grid_data/download/69734452?include=energy
Tmed3	0.891	http://api.brain-map.org/grid_data/download/71015196?include=energy
Stxbp2	0.891	http://api.brain-map.org/grid_data/download/75774667?include=energy
Ydjc	0.891	http://api.brain-map.org/grid_data/download/71492916?include=energy
Cdk5rap3	0.891	http://api.brain-map.org/grid_data/download/69080011?include=energy
Prps2	0.891	http://api.brain-map.org/grid_data/download/69080734?include=energy
Arfgef2	0.891	http://api.brain-map.org/grid_data/download/79912574?include=energy
Suz12	0.891	http://api.brain-map.org/grid_data/download/69261927?include=energy
C430048L16Rik	0.891	http://api.brain-map.org/grid_data/download/69531025?include=energy
Slc24a5	0.891	http://api.brain-map.org/grid_data/download/71489789?include=energy
Smc6	0.891	http://api.brain-map.org/grid_data/download/69169785?include=energy
Prpf18	0.891	http://api.brain-map.org/grid_data/download/69407290?include=energy
Prl8a2	0.891	http://api.brain-map.org/grid_data/download/75931607?include=energy
D830013H23Rik	0.891	http://api.brain-map.org/grid_data/download/70238746?include=energy
Gas2	0.891	http://api.brain-map.org/grid_data/download/71610818?include=energy
Ugt2b34	0.891	http://api.brain-map.org/grid_data/download/70528210?include=energy
Fam122a	0.891	http://api.brain-map.org/grid_data/download/68522448?include=energy
Astn1	0.891	http://api.brain-map.org/grid_data/download/71570735?include=energy
Klf7	0.891	http://api.brain-map.org/grid_data/download/112202624?include=energy
Alkbh2	0.891	http://api.brain-map.org/grid_data/download/68798406?include=energy
Btla	0.891	http://api.brain-map.org/grid_data/download/76080984?include=energy
Dcxr	0.891	http://api.brain-map.org/grid_data/download/68301993?include=energy
2900064A13Rik	0.891	http://api.brain-map.org/grid_data/download/71493129?include=energy
Fam160a2	0.891	http://api.brain-map.org/grid_data/download/71656725?include=energy
Vwde	0.891	http://api.brain-map.org/grid_data/download/70295930?include=energy
Tspyl1	0.891	http://api.brain-map.org/grid_data/download/393194?include=energy
Cep290	0.891	http://api.brain-map.org/grid_data/download/633667?include=energy
Mm.72675	0.891	http://api.brain-map.org/grid_data/download/71210412?include=energy
Arid5a	0.891	http://api.brain-map.org/grid_data/download/75042267?include=energy
Dusp9	0.891	http://api.brain-map.org/grid_data/download/69671660?include=energy
Dr1	0.891	http://api.brain-map.org/grid_data/download/81600582?include=energy
Uxt	0.891	http://api.brain-map.org/grid_data/download/75198160?include=energy
Krr1	0.891	http://api.brain-map.org/grid_data/download/69735090?include=energy
Rfc3	0.891	http://api.brain-map.org/grid_data/download/68637921?include=energy
Pcgf6	0.891	http://api.brain-map.org/grid_data/download/70634297?include=energy
LOC433197	0.891	http://api.brain-map.org/grid_data/download/70722996?include=energy
Rrp1	0.891	http://api.brain-map.org/grid_data/download/713?include=energy
Pilrb1	0.891	http://api.brain-map.org/grid_data/download/70784976?include=energy
Hexim1	0.891	http://api.brain-map.org/grid_data/download/74273119?include=energy
Lgals2	0.891	http://api.brain-map.org/grid_data/download/69549188?include=energy
Gm5087	0.89	http://api.brain-map.org/grid_data/download/70305580?include=energy
Clic1	0.89	http://api.brain-map.org/grid_data/download/67809267?include=energy
Ppy	0.89	http://api.brain-map.org/grid_data/download/71991971?include=energy
Il7r	0.89	http://api.brain-map.org/grid_data/download/69735437?include=energy
Arl9	0.89	http://api.brain-map.org/grid_data/download/73513435?include=energy
Znhit6	0.89	http://api.brain-map.org/grid_data/download/70566041?include=energy
4930588N13Rik	0.89	http://api.brain-map.org/grid_data/download/70218438?include=energy
Fabp1	0.89	http://api.brain-map.org/grid_data/download/73423693?include=energy
Ankrd22	0.89	http://api.brain-map.org/grid_data/download/71724798?include=energy
Gm4990	0.89	http://api.brain-map.org/grid_data/download/70595744?include=energy
Brf2	0.89	http://api.brain-map.org/grid_data/download/69529418?include=energy
Slc13a1	0.89	http://api.brain-map.org/grid_data/download/80473477?include=energy
Scara3	0.89	http://api.brain-map.org/grid_data/download/75042270?include=energy
Myo5c	0.89	http://api.brain-map.org/grid_data/download/75042249?include=energy
Ccdc41	0.89	http://api.brain-map.org/grid_data/download/69407887?include=energy
Zc3hc1	0.89	http://api.brain-map.org/grid_data/download/68667252?include=energy
Pak2	0.89	http://api.brain-map.org/grid_data/download/71281343?include=energy
Spata19	0.89	http://api.brain-map.org/grid_data/download/75851033?include=energy
Gm1673	0.89	http://api.brain-map.org/grid_data/download/74581181?include=energy
Gmpr	0.89	http://api.brain-map.org/grid_data/download/74581368?include=energy
Tfrc	0.89	http://api.brain-map.org/grid_data/download/68843823?include=energy
Zscan12	0.89	http://api.brain-map.org/grid_data/download/71763673?include=energy
LOC432785	0.89	http://api.brain-map.org/grid_data/download/70611305?include=energy
Tmprss6	0.89	http://api.brain-map.org/grid_data/download/68638089?include=energy
Krt25	0.89	http://api.brain-map.org/grid_data/download/68637647?include=energy
C6	0.89	http://api.brain-map.org/grid_data/download/68302257?include=energy
Gpc1	0.89	http://api.brain-map.org/grid_data/download/73994669?include=energy
Cnot6	0.89	http://api.brain-map.org/grid_data/download/72283540?include=energy
Enox2	0.89	http://api.brain-map.org/grid_data/download/70928339?include=energy
Tulp3	0.89	http://api.brain-map.org/grid_data/download/76003606?include=energy
Timeless	0.89	http://api.brain-map.org/grid_data/download/69169803?include=energy
Phc1	0.89	http://api.brain-map.org/grid_data/download/77454991?include=energy
Hip1	0.89	http://api.brain-map.org/grid_data/download/2293?include=energy
Dusp23	0.89	http://api.brain-map.org/grid_data/download/74427086?include=energy
Evc	0.89	http://api.brain-map.org/grid_data/download/556990?include=energy
4933436N17Rik	0.89	http://api.brain-map.org/grid_data/download/71378688?include=energy
Mtss1l	0.89	http://api.brain-map.org/grid_data/download/77332715?include=energy
Usp6nl	0.89	http://api.brain-map.org/grid_data/download/70565407?include=energy
Zfp94	0.89	http://api.brain-map.org/grid_data/download/80513978?include=energy
Spata2l	0.89	http://api.brain-map.org/grid_data/download/73817441?include=energy
Rab3d	0.89	http://api.brain-map.org/grid_data/download/71724023?include=energy
Tead2	0.89	http://api.brain-map.org/grid_data/download/68667115?include=energy

Sprr1b	0.89	http://api.brain-map.org/grid_data/download/74581050?include=energy
9930012K11Rik	0.89	http://api.brain-map.org/grid_data/download/70946211?include=energy
Gm5919	0.89	http://api.brain-map.org/grid_data/download/75043695?include=energy
Pcdhga12	0.89	http://api.brain-map.org/grid_data/download/770?include=energy
Ppargc1a	0.89	http://api.brain-map.org/grid_data/download/71022954?include=energy
Scgb1a1	0.89	http://api.brain-map.org/grid_data/download/70565201?include=energy
Nek5	0.89	http://api.brain-map.org/grid_data/download/68745564?include=energy
Herc3	0.89	http://api.brain-map.org/grid_data/download/68845496?include=energy
Taok3	0.89	http://api.brain-map.org/grid_data/download/70812990?include=energy
Mfsd8	0.89	http://api.brain-map.org/grid_data/download/73606609?include=energy
Mbd2	0.89	http://api.brain-map.org/grid_data/download/74822933?include=energy
Exosc2	0.89	http://api.brain-map.org/grid_data/download/68410243?include=energy
Mm.246010	0.89	http://api.brain-map.org/grid_data/download/71456139?include=energy
Popdc2	0.89	http://api.brain-map.org/grid_data/download/68798418?include=energy
Insl5	0.89	http://api.brain-map.org/grid_data/download/68301437?include=energy
Tktl1	0.89	http://api.brain-map.org/grid_data/download/73617522?include=energy
Col4a1	0.89	http://api.brain-map.org/grid_data/download/81654101?include=energy
Qpctl	0.89	http://api.brain-map.org/grid_data/download/75831771?include=energy
1700088E04Rik	0.89	http://api.brain-map.org/grid_data/download/73512567?include=energy
Maml1	0.89	http://api.brain-map.org/grid_data/download/75214946?include=energy
Gm590	0.89	http://api.brain-map.org/grid_data/download/70295978?include=energy
Gng5	0.89	http://api.brain-map.org/grid_data/download/79632238?include=energy
Copg2	0.89	http://api.brain-map.org/grid_data/download/74819259?include=energy
Dmtf1	0.89	http://api.brain-map.org/grid_data/download/68745444?include=energy
Ptpn6	0.89	http://api.brain-map.org/grid_data/download/100146055?include=energy
Rp9	0.89	http://api.brain-map.org/grid_data/download/69818287?include=energy
6030498E09Rik	0.89	http://api.brain-map.org/grid_data/download/70227890?include=energy
Rnf43	0.889	http://api.brain-map.org/grid_data/download/69407660?include=energy
Rpl12-ps1	0.889	http://api.brain-map.org/grid_data/download/74736087?include=energy
Mm.38865	0.889	http://api.brain-map.org/grid_data/download/74431786?include=energy
LOC433814	0.889	http://api.brain-map.org/grid_data/download/74580697?include=energy
Trim3	0.889	http://api.brain-map.org/grid_data/download/73732147?include=energy
Ap3d1	0.889	http://api.brain-map.org/grid_data/download/68924799?include=energy
Veph1	0.889	http://api.brain-map.org/grid_data/download/74988352?include=energy
Klk1b26	0.889	http://api.brain-map.org/grid_data/download/68632112?include=energy
Ankrd32	0.889	http://api.brain-map.org/grid_data/download/73731253?include=energy
Polr3d	0.889	http://api.brain-map.org/grid_data/download/70429363?include=energy
Tmem173	0.889	http://api.brain-map.org/grid_data/download/68861674?include=energy
1700029H14Rik	0.889	http://api.brain-map.org/grid_data/download/75860934?include=energy
Crnkl1	0.889	http://api.brain-map.org/grid_data/download/76081879?include=energy
Prss46	0.889	http://api.brain-map.org/grid_data/download/70201510?include=energy
Bicc1	0.889	http://api.brain-map.org/grid_data/download/73906442?include=energy
Tll1	0.889	http://api.brain-map.org/grid_data/download/74272905?include=energy
E330013P04Rik	0.889	http://api.brain-map.org/grid_data/download/76081003?include=energy
Cytl1	0.889	http://api.brain-map.org/grid_data/download/69407989?include=energy
Tardbp	0.889	http://api.brain-map.org/grid_data/download/68157419?include=energy
LOC382175	0.889	http://api.brain-map.org/grid_data/download/74578194?include=energy
Olfr877	0.889	http://api.brain-map.org/grid_data/download/74277396?include=energy
Gm1610	0.889	http://api.brain-map.org/grid_data/download/74743417?include=energy
Prps1l1	0.889	http://api.brain-map.org/grid_data/download/70201235?include=energy
Chn1	0.889	http://api.brain-map.org/grid_data/download/73718053?include=energy
Rb1	0.889	http://api.brain-map.org/grid_data/download/69816763?include=energy
Cxadr	0.889	http://api.brain-map.org/grid_data/download/77454980?include=energy
Tnfrsf18	0.889	http://api.brain-map.org/grid_data/download/74580930?include=energy
Slc35c2	0.889	http://api.brain-map.org/grid_data/download/2615?include=energy
Ttyh1	0.889	http://api.brain-map.org/grid_data/download/70919830?include=energy
Obfc2b	0.889	http://api.brain-map.org/grid_data/download/67815540?include=energy
LOC432444	0.889	http://api.brain-map.org/grid_data/download/70608517?include=energy
Zfp54	0.889	http://api.brain-map.org/grid_data/download/71609044?include=energy
Ribc2	0.889	http://api.brain-map.org/grid_data/download/70218426?include=energy
Mblac1	0.889	http://api.brain-map.org/grid_data/download/68920662?include=energy
Dapk1	0.889	http://api.brain-map.org/grid_data/download/72472784?include=energy
H2-Q10	0.889	http://api.brain-map.org/grid_data/download/68302281?include=energy
Prkag3	0.889	http://api.brain-map.org/grid_data/download/71210816?include=energy
Pusl1	0.889	http://api.brain-map.org/grid_data/download/70786833?include=energy
Rexo2	0.889	http://api.brain-map.org/grid_data/download/67979032?include=energy
Atad2	0.889	http://api.brain-map.org/grid_data/download/74359419?include=energy
Traf1	0.889	http://api.brain-map.org/grid_data/download/72004444?include=energy
Slco6b1	0.889	http://api.brain-map.org/grid_data/download/71919571?include=energy
Kcnk10	0.889	http://api.brain-map.org/grid_data/download/73834391?include=energy
4930417G10Rik	0.889	http://api.brain-map.org/grid_data/download/69407917?include=energy
Gm6740	0.889	http://api.brain-map.org/grid_data/download/1902?include=energy
Ptpn23	0.889	http://api.brain-map.org/grid_data/download/69817691?include=energy
Ccar1	0.889	http://api.brain-map.org/grid_data/download/69838528?include=energy
Arid5b	0.889	http://api.brain-map.org/grid_data/download/71283598?include=energy
Tmem238	0.889	http://api.brain-map.org/grid_data/download/69854151?include=energy
Slc35e3	0.889	http://api.brain-map.org/grid_data/download/68844176?include=energy
F430115D03Rik*	0.889	http://api.brain-map.org/grid_data/download/73767277?include=energy
1700067K01Rik	0.889	http://api.brain-map.org/grid_data/download/69353768?include=energy
Pgam5	0.889	http://api.brain-map.org/grid_data/download/74635668?include=energy
C730037M02Rik*	0.889	http://api.brain-map.org/grid_data/download/73906388?include=energy
D130060J02Rik	0.889	http://api.brain-map.org/grid_data/download/70238609?include=energy
4922505F16Rik*	0.889	http://api.brain-map.org/grid_data/download/73906479?include=energy
1700025H01Rik	0.889	http://api.brain-map.org/grid_data/download/71919577?include=energy
Zfp874a	0.889	http://api.brain-map.org/grid_data/download/75934931?include=energy
Rbl1	0.889	http://api.brain-map.org/grid_data/download/76003367?include=energy
Sntn	0.889	http://api.brain-map.org/grid_data/download/69448650?include=energy
Pfn2	0.889	http://api.brain-map.org/grid_data/download/1261?include=energy
Tex12	0.889	http://api.brain-map.org/grid_data/download/69174052?include=energy
Lipm	0.889	http://api.brain-map.org/grid_data/download/74272035?include=energy
Glrx3	0.889	http://api.brain-map.org/grid_data/download/71724744?include=energy
Tbca	0.889	http://api.brain-map.org/grid_data/download/68861975?include=energy
Eya2	0.889	http://api.brain-map.org/grid_data/download/70743234?include=energy
Zcchc4	0.889	http://api.brain-map.org/grid_data/download/72004461?include=energy
Abcd3	0.889	http://api.brain-map.org/grid_data/download/77799049?include=energy
Kctd20	0.889	http://api.brain-map.org/grid_data/download/69262333?include=energy
Jph3	0.889	http://api.brain-map.org/grid_data/download/69735670?include=energy
Polr3c	0.889	http://api.brain-map.org/grid_data/download/68637688?include=energy
Cpb2	0.889	http://api.brain-map.org/grid_data/download/75931617?include=energy
Galk1	0.889	http://api.brain-map.org/grid_data/download/68341627?include=energy
Nek9	0.888	http://api.brain-map.org/grid_data/download/70546269?include=energy
Ero1l	0.888	http://api.brain-map.org/grid_data/download/68545150?include=energy
Ctbp2	0.888	http://api.brain-map.org/grid_data/download/70615583?include=energy
4921534K20Rik*	0.888	http://api.brain-map.org/grid_data/download/73834547?include=energy
AI661453	0.888	http://api.brain-map.org/grid_data/download/70565987?include=energy
Cbx2	0.888	http://api.brain-map.org/grid_data/download/67814209?include=energy
Mm.359168	0.888	http://api.brain-map.org/grid_data/download/71209904?include=energy
3100002H09Rik	0.888	http://api.brain-map.org/grid_data/download/77340484?include=energy
Anp32b	0.888	http://api.brain-map.org/grid_data/download/71574416?include=energy
E330034G19Rik	0.888	http://api.brain-map.org/grid_data/download/71496528?include=energy
Dkk1	0.888	http://api.brain-map.org/grid_data/download/2149?include=energy
Nthl1	0.888	http://api.brain-map.org/grid_data/download/74431432?include=energy
1700018B24Rik	0.888	http://api.brain-map.org/grid_data/download/70526062?include=energy
Usp13	0.888	http://api.brain-map.org/grid_data/download/74957910?include=energy
Kcnmb1	0.888	http://api.brain-map.org/grid_data/download/81599616?include=energy
Fam49a	0.888	http://api.brain-map.org/grid_data/download/74425565?include=energy
Aifm2	0.888	http://api.brain-map.org/grid_data/download/71656094?include=energy
2310042D19Rik	0.888	http://api.brain-map.org/grid_data/download/69202466?include=energy
Surf4	0.888	http://api.brain-map.org/grid_data/download/69080027?include=energy
Rab5b	0.888	http://api.brain-map.org/grid_data/download/100144441?include=energy
1810063B07Rik	0.888	http://api.brain-map.org/grid_data/download/68545003?include=energy
Sfxn3	0.888	http://api.brain-map.org/grid_data/download/564760?include=energy
Serpine1	0.888	http://api.brain-map.org/grid_data/download/69549311?include=energy
2310005E17Rik	0.888	http://api.brain-map.org/grid_data/download/71991774?include=energy
D630042P16Rik	0.888	http://api.brain-map.org/grid_data/download/71808661?include=energy
Sgtb	0.888	http://api.brain-map.org/grid_data/download/71924341?include=energy
2010106G01Rik	0.888	http://api.brain-map.org/grid_data/download/1647?include=energy
Serpina6	0.888	http://api.brain-map.org/grid_data/download/68302160?include=energy
Ppp4c	0.888	http://api.brain-map.org/grid_data/download/632486?include=energy
Zc3h12a	0.888	http://api.brain-map.org/grid_data/download/71656092?include=energy
Aplnr	0.888	http://api.brain-map.org/grid_data/download/1661?include=energy
Fbxo7	0.888	http://api.brain-map.org/grid_data/download/70717351?include=energy
Haus5	0.888	http://api.brain-map.org/grid_data/download/69202400?include=energy
Gipc2	0.888	http://api.brain-map.org/grid_data/download/74658141?include=energy
Rbm14	0.888	http://api.brain-map.org/grid_data/download/100146062?include=energy
C920008G01Rik	0.888	http://api.brain-map.org/grid_data/download/75214949?include=energy
Zufsp	0.888	http://api.brain-map.org/grid_data/download/73679044?include=energy
Pkd1l1	0.888	http://api.brain-map.org/grid_data/download/75990411?include=energy
Gdf6	0.888	http://api.brain-map.org/grid_data/download/70276879?include=energy
Ltb	0.888	http://api.brain-map.org/grid_data/download/74363350?include=energy
Krt72-ps	0.888	http://api.brain-map.org/grid_data/download/70724559?include=energy
Prl3b1	0.888	http://api.brain-map.org/grid_data/download/75931483?include=energy
Upf1	0.888	http://api.brain-map.org/grid_data/download/68797715?include=energy
LOC433403	0.888	http://api.brain-map.org/grid_data/download/71765016?include=energy
Dad1	0.888	http://api.brain-map.org/grid_data/download/74819345?include=energy
Angpt2	0.888	http://api.brain-map.org/grid_data/download/68633002?include=energy
Prmt8	0.888	http://api.brain-map.org/grid_data/download/74743441?include=energy
Gm14092	0.888	http://api.brain-map.org/grid_data/download/73870692?include=energy
H1f0	0.888	http://api.brain-map.org/grid_data/download/70431343?include=energy
Olfr67	0.888	http://api.brain-map.org/grid_data/download/74734812?include=energy
Acsm5	0.888	http://api.brain-map.org/grid_data/download/71021031?include=energy
Spsb1	0.888	http://api.brain-map.org/grid_data/download/77332092?include=energy
Pdrg1	0.888	http://api.brain-map.org/grid_data/download/69549267?include=energy
1700028K03Rik	0.888	http://api.brain-map.org/grid_data/download/71919661?include=energy
Spred1	0.888	http://api.brain-map.org/grid_data/download/69028587?include=energy
Kctd15	0.888	http://api.brain-map.org/grid_data/download/67765718?include=energy
Nell1	0.888	http://api.brain-map.org/grid_data/download/74357567?include=energy
Shroom1	0.888	http://api.brain-map.org/grid_data/download/1315?include=energy
Lypd3	0.888	http://api.brain-map.org/grid_data/download/75827096?include=energy
Bcat2	0.888	http://api.brain-map.org/grid_data/download/68341310?include=energy
Igbp1	0.888	http://api.brain-map.org/grid_data/download/100142501?include=energy
Marf1	0.887	http://api.brain-map.org/grid_data/download/71281904?include=energy
Nppb	0.887	http://api.brain-map.org/grid_data/download/69174475?include=energy
Oas3	0.887	http://api.brain-map.org/grid_data/download/73423875?include=energy
Zbtb8a	0.887	http://api.brain-map.org/grid_data/download/641515?include=energy
Tmem20	0.887	http://api.brain-map.org/grid_data/download/69981079?include=energy
Cyp1a1	0.887	http://api.brain-map.org/grid_data/download/75988367?include=energy
Slc5a1	0.887	http://api.brain-map.org/grid_data/download/2621?include=energy
Rnf8	0.887	http://api.brain-map.org/grid_data/download/576452?include=energy
Abhd8	0.887	http://api.brain-map.org/grid_data/download/2012?include=energy
C1qtnf4	0.887	http://api.brain-map.org/grid_data/download/75198148?include=energy
Bmp6	0.887	http://api.brain-map.org/grid_data/download/74514174?include=energy
Fam199x	0.887	http://api.brain-map.org/grid_data/download/68563104?include=energy
Taz	0.887	http://api.brain-map.org/grid_data/download/71627800?include=energy
Hapln1	0.887	http://api.brain-map.org/grid_data/download/77332706?include=energy
Socs3	0.887	http://api.brain-map.org/grid_data/download/68911020?include=energy
Lhx3	0.887	http://api.brain-map.org/grid_data/download/77924514?include=energy
Sars2	0.887	http://api.brain-map.org/grid_data/download/100144892?include=energy
Fam190a	0.887	http://api.brain-map.org/grid_data/download/71717144?include=energy
F9	0.887	http://api.brain-map.org/grid_data/download/69672252?include=energy
Prl8a8	0.887	http://api.brain-map.org/grid_data/download/71764488?include=energy
Rwdd2b	0.887	http://api.brain-map.org/grid_data/download/1587?include=energy
Chrna5	0.887	http://api.brain-map.org/grid_data/download/75826567?include=energy
Tmem138	0.887	http://api.brain-map.org/grid_data/download/68798061?include=energy
Ppp5c	0.887	http://api.brain-map.org/grid_data/download/413805?include=energy
Olfr1030	0.887	http://api.brain-map.org/grid_data/download/74720567?include=energy
Akr1c20	0.887	http://api.brain-map.org/grid_data/download/68410548?include=energy
LOC432967	0.887	http://api.brain-map.org/grid_data/download/73818825?include=energy
Glo1	0.887	http://api.brain-map.org/grid_data/download/74047916?include=energy
LOC381788	0.887	http://api.brain-map.org/grid_data/download/71393328?include=energy
LOC329302	0.887	http://api.brain-map.org/grid_data/download/73931420?include=energy
Cenpq	0.887	http://api.brain-map.org/grid_data/download/68341675?include=energy
Cyp4f15	0.887	http://api.brain-map.org/grid_data/download/1189?include=energy
Map2k7	0.887	http://api.brain-map.org/grid_data/download/69751998?include=energy
Cngb3	0.887	http://api.brain-map.org/grid_data/download/75985880?include=energy
Spint4	0.887	http://api.brain-map.org/grid_data/download/75934518?include=energy
Mm.217348	0.887	http://api.brain-map.org/grid_data/download/71152473?include=energy
Mtf2	0.887	http://api.brain-map.org/grid_data/download/1510?include=energy
Marcksl1	0.887	http://api.brain-map.org/grid_data/download/670?include=energy
LOC435800	0.887	http://api.brain-map.org/grid_data/download/71022585?include=energy
Birc2	0.887	http://api.brain-map.org/grid_data/download/69529304?include=energy
Mm.118842	0.887	http://api.brain-map.org/grid_data/download/71147948?include=energy
Hmgn1	0.887	http://api.brain-map.org/grid_data/download/77932781?include=energy
Cenpp	0.887	http://api.brain-map.org/grid_data/download/71919565?include=energy
Arnt	0.887	http://api.brain-map.org/grid_data/download/79544892?include=energy
Hemgn	0.887	http://api.brain-map.org/grid_data/download/69114549?include=energy
Mm.203332	0.887	http://api.brain-map.org/grid_data/download/71152330?include=energy
Ept1	0.887	http://api.brain-map.org/grid_data/download/69608983?include=energy
BC051142	0.887	http://api.brain-map.org/grid_data/download/69113853?include=energy
Fastkd5	0.887	http://api.brain-map.org/grid_data/download/68667940?include=energy
Fdft1	0.887	http://api.brain-map.org/grid_data/download/75041432?include=energy
Rad9	0.887	http://api.brain-map.org/grid_data/download/69817910?include=energy
Acp6	0.887	http://api.brain-map.org/grid_data/download/68924572?include=energy
Wls	0.887	http://api.brain-map.org/grid_data/download/72077452?include=energy
Wdhd1	0.887	http://api.brain-map.org/grid_data/download/68797685?include=energy
Als2	0.887	http://api.brain-map.org/grid_data/download/67767467?include=energy
Fbxo43	0.887	http://api.brain-map.org/grid_data/download/71393304?include=energy
D3Ertd751e	0.887	http://api.brain-map.org/grid_data/download/75197624?include=energy
Zfp341	0.887	http://api.brain-map.org/grid_data/download/74058659?include=energy
Setd7	0.887	http://api.brain-map.org/grid_data/download/71016630?include=energy
Vmn1r4	0.887	http://api.brain-map.org/grid_data/download/70785242?include=energy
Sorbs1	0.887	http://api.brain-map.org/grid_data/download/74425572?include=energy
A030005K14Rik	0.887	http://api.brain-map.org/grid_data/download/71892442?include=energy
Ndufa8	0.887	http://api.brain-map.org/grid_data/download/386020?include=energy
Zfp142	0.887	http://api.brain-map.org/grid_data/download/69290800?include=energy
Abcb6	0.887	http://api.brain-map.org/grid_data/download/75749413?include=energy
Rbms1	0.887	http://api.brain-map.org/grid_data/download/74635602?include=energy
Ube2u	0.887	http://api.brain-map.org/grid_data/download/70596077?include=energy
Mbl2	0.887	http://api.brain-map.org/grid_data/download/68302392?include=energy
2810039B14Rik	0.887	http://api.brain-map.org/grid_data/download/74821590?include=energy
Rhot1	0.887	http://api.brain-map.org/grid_data/download/69262257?include=energy
Parp9	0.887	http://api.brain-map.org/grid_data/download/633670?include=energy
Lims2	0.887	http://api.brain-map.org/grid_data/download/75145999?include=energy
1700057K13Rik	0.887	http://api.brain-map.org/grid_data/download/71920165?include=energy
Bfar	0.887	http://api.brain-map.org/grid_data/download/68638077?include=energy
LOC432814	0.886	http://api.brain-map.org/grid_data/download/75991072?include=energy
2010001E11Rik	0.886	http://api.brain-map.org/grid_data/download/71920291?include=energy
Klhl36	0.886	http://api.brain-map.org/grid_data/download/77371861?include=energy
Tpo	0.886	http://api.brain-map.org/grid_data/download/75214947?include=energy
Dcaf10	0.886	http://api.brain-map.org/grid_data/download/68546470?include=energy
Tmf1	0.886	http://api.brain-map.org/grid_data/download/70295924?include=energy
Stil	0.886	http://api.brain-map.org/grid_data/download/70795949?include=energy
Fzd8	0.886	http://api.brain-map.org/grid_data/download/470?include=energy
Pa2g4	0.886	http://api.brain-map.org/grid_data/download/69262297?include=energy
9330209B10Rik*	0.886	http://api.brain-map.org/grid_data/download/73927751?include=energy
Il27	0.886	http://api.brain-map.org/grid_data/download/71764727?include=energy
Itprip	0.886	http://api.brain-map.org/grid_data/download/70231773?include=energy
BC016579	0.886	http://api.brain-map.org/grid_data/download/68341561?include=energy
Rab38	0.886	http://api.brain-map.org/grid_data/download/69817835?include=energy
Tbpl2	0.886	http://api.brain-map.org/grid_data/download/71281831?include=energy
Gm12258	0.886	http://api.brain-map.org/grid_data/download/70566199?include=energy
Agbl3	0.886	http://api.brain-map.org/grid_data/download/75863318?include=energy
Apoc1	0.886	http://api.brain-map.org/grid_data/download/68910959?include=energy
Cog5	0.886	http://api.brain-map.org/grid_data/download/71213087?include=energy
Bpnt1	0.886	http://api.brain-map.org/grid_data/download/73584789?include=energy
Tbc1d13	0.886	http://api.brain-map.org/grid_data/download/68498980?include=energy
Ndufb11	0.886	http://api.brain-map.org/grid_data/download/67764909?include=energy
Zfp511	0.886	http://api.brain-map.org/grid_data/download/76081043?include=energy
Ube2n	0.886	http://api.brain-map.org/grid_data/download/71280982?include=energy
Cdh22	0.886	http://api.brain-map.org/grid_data/download/71487073?include=energy
Slc27a2	0.886	http://api.brain-map.org/grid_data/download/70918910?include=energy
Barhl1	0.886	http://api.brain-map.org/grid_data/download/68861948?include=energy
4933402P03Rik	0.886	http://api.brain-map.org/grid_data/download/70218624?include=energy
Mybpc3	0.886	http://api.brain-map.org/grid_data/download/77455149?include=energy
Tut1	0.886	http://api.brain-map.org/grid_data/download/75750321?include=energy
Ncf2	0.886	http://api.brain-map.org/grid_data/download/74821971?include=energy
Preb	0.886	http://api.brain-map.org/grid_data/download/73584783?include=energy

Ubxn11	0.886	http://api.brain-map.org/grid_data/download/322?include=energy
Cox10	0.886	http://api.brain-map.org/grid_data/download/678696?include=energy
Qsox1	0.886	http://api.brain-map.org/grid_data/download/69817793?include=energy
Tmem175	0.886	http://api.brain-map.org/grid_data/download/112644308?include=energy
Pcdh15	0.886	http://api.brain-map.org/grid_data/download/69782784?include=energy
Mre11a	0.886	http://api.brain-map.org/grid_data/download/71020780?include=energy
Lrp1	0.886	http://api.brain-map.org/grid_data/download/69736244?include=energy
Hmx1	0.886	http://api.brain-map.org/grid_data/download/76003366?include=energy
Ctps2	0.886	http://api.brain-map.org/grid_data/download/68269061?include=energy
Scyl3	0.886	http://api.brain-map.org/grid_data/download/69353182?include=energy
Tmed9	0.886	http://api.brain-map.org/grid_data/download/513478?include=energy
LOC434359	0.886	http://api.brain-map.org/grid_data/download/70920123?include=energy
Golga2	0.886	http://api.brain-map.org/grid_data/download/392913?include=energy
Tada3	0.886	http://api.brain-map.org/grid_data/download/71724466?include=energy
Mpp6	0.886	http://api.brain-map.org/grid_data/download/73994666?include=energy
Upk3a	0.886	http://api.brain-map.org/grid_data/download/70302920?include=energy
Cyp2b19	0.886	http://api.brain-map.org/grid_data/download/69608360?include=energy
Acoxl	0.886	http://api.brain-map.org/grid_data/download/68797846?include=energy
Gatsl2	0.886	http://api.brain-map.org/grid_data/download/70545919?include=energy
Nhlrc1	0.886	http://api.brain-map.org/grid_data/download/75084473?include=energy
Rp1	0.886	http://api.brain-map.org/grid_data/download/70301067?include=energy
Wisp2	0.886	http://api.brain-map.org/grid_data/download/68523207?include=energy
Ebp	0.886	http://api.brain-map.org/grid_data/download/633685?include=energy
LOC433879	0.886	http://api.brain-map.org/grid_data/download/71764861?include=energy
Srp14	0.886	http://api.brain-map.org/grid_data/download/74750021?include=energy
Rbm11	0.886	http://api.brain-map.org/grid_data/download/1411?include=energy
Wbp4	0.886	http://api.brain-map.org/grid_data/download/68744546?include=energy
Slc4a1ap	0.886	http://api.brain-map.org/grid_data/download/68844361?include=energy
Pxmp3	0.886	http://api.brain-map.org/grid_data/download/80513994?include=energy
Cldn14	0.886	http://api.brain-map.org/grid_data/download/69548982?include=energy
Ric3	0.886	http://api.brain-map.org/grid_data/download/74514135?include=energy
Glra4	0.886	http://api.brain-map.org/grid_data/download/75551476?include=energy
BC021614	0.886	http://api.brain-map.org/grid_data/download/68499147?include=energy
Acvr1b	0.886	http://api.brain-map.org/grid_data/download/69514720?include=energy
5430411K18Rik	0.886	http://api.brain-map.org/grid_data/download/69443421?include=energy
Ganab	0.886	http://api.brain-map.org/grid_data/download/69672778?include=energy
Mepe	0.886	http://api.brain-map.org/grid_data/download/70300306?include=energy
B4galt3	0.886	http://api.brain-map.org/grid_data/download/357099?include=energy
Gm44	0.886	http://api.brain-map.org/grid_data/download/70295661?include=energy
Vmn1r225	0.886	http://api.brain-map.org/grid_data/download/70785624?include=energy
Gpr3	0.886	http://api.brain-map.org/grid_data/download/74511769?include=energy
Ktn1	0.886	http://api.brain-map.org/grid_data/download/74635451?include=energy
Ptms	0.886	http://api.brain-map.org/grid_data/download/68798711?include=energy
Foxa1	0.886	http://api.brain-map.org/grid_data/download/77869794?include=energy
Gjb2	0.886	http://api.brain-map.org/grid_data/download/1359?include=energy
TC1480207	0.886	http://api.brain-map.org/grid_data/download/70812699?include=energy
A230010G09Rik*	0.886	http://api.brain-map.org/grid_data/download/73808510?include=energy
Spg21	0.885	http://api.brain-map.org/grid_data/download/71717143?include=energy
Nr2f2	0.885	http://api.brain-map.org/grid_data/download/112646890?include=energy
Atoh8	0.885	http://api.brain-map.org/grid_data/download/81654089?include=energy
Nupr1	0.885	http://api.brain-map.org/grid_data/download/631712?include=energy
C87436	0.885	http://api.brain-map.org/grid_data/download/68637605?include=energy
Odf3l2	0.885	http://api.brain-map.org/grid_data/download/71764763?include=energy
Mob1a	0.885	http://api.brain-map.org/grid_data/download/75991092?include=energy
Fam167a	0.885	http://api.brain-map.org/grid_data/download/71924279?include=energy
Gm1088	0.885	http://api.brain-map.org/grid_data/download/77340470?include=energy
Mov10	0.885	http://api.brain-map.org/grid_data/download/68798591?include=energy
Gosr1	0.885	http://api.brain-map.org/grid_data/download/100144152?include=energy
Lrrc28	0.885	http://api.brain-map.org/grid_data/download/69736274?include=energy
2310015D24Rik	0.885	http://api.brain-map.org/grid_data/download/71393316?include=energy
4930486L24Rik	0.885	http://api.brain-map.org/grid_data/download/69117104?include=energy
Cyb5r3	0.885	http://api.brain-map.org/grid_data/download/77620794?include=energy
Tas2r120	0.885	http://api.brain-map.org/grid_data/download/70302360?include=energy
Iffo1	0.885	http://api.brain-map.org/grid_data/download/71281746?include=energy
Tmem176b	0.885	http://api.brain-map.org/grid_data/download/77866852?include=energy
Ccdc75	0.885	http://api.brain-map.org/grid_data/download/71281752?include=energy
Bub1b	0.885	http://api.brain-map.org/grid_data/download/74990525?include=energy
4933416E14Rik	0.885	http://api.brain-map.org/grid_data/download/71022906?include=energy
LOC434153	0.885	http://api.brain-map.org/grid_data/download/70787483?include=energy
Insm1	0.885	http://api.brain-map.org/grid_data/download/71393442?include=energy
LOC546415	0.885	http://api.brain-map.org/grid_data/download/74736468?include=energy
Fam125a	0.885	http://api.brain-map.org/grid_data/download/71610899?include=energy
Zscan30	0.885	http://api.brain-map.org/grid_data/download/69530947?include=energy
Nup153	0.885	http://api.brain-map.org/grid_data/download/68798549?include=energy
Pvrl4	0.885	http://api.brain-map.org/grid_data/download/2511?include=energy
Heatr2	0.885	http://api.brain-map.org/grid_data/download/68667685?include=energy
Fbxl15	0.885	http://api.brain-map.org/grid_data/download/71587643?include=energy
Aurkc	0.885	http://api.brain-map.org/grid_data/download/75988298?include=energy
Myo5b	0.885	http://api.brain-map.org/grid_data/download/73592538?include=energy
Dmwd	0.885	http://api.brain-map.org/grid_data/download/2151?include=energy
Baat	0.885	http://api.brain-map.org/grid_data/download/68301288?include=energy
Abra	0.885	http://api.brain-map.org/grid_data/download/70231875?include=energy
Fxyd2	0.885	http://api.brain-map.org/grid_data/download/68666684?include=energy
Oas2	0.885	http://api.brain-map.org/grid_data/download/70784720?include=energy
Rtn4ip1	0.885	http://api.brain-map.org/grid_data/download/68499213?include=energy
9130206I24Rik	0.885	http://api.brain-map.org/grid_data/download/69446559?include=energy
Gpr183	0.885	http://api.brain-map.org/grid_data/download/71656630?include=energy
1700109F18Rik	0.885	http://api.brain-map.org/grid_data/download/74357899?include=energy
Pramel7	0.885	http://api.brain-map.org/grid_data/download/68667575?include=energy
Gm872	0.885	http://api.brain-map.org/grid_data/download/70298375?include=energy
Ap1ar	0.885	http://api.brain-map.org/grid_data/download/69080028?include=energy
Pacrgl	0.885	http://api.brain-map.org/grid_data/download/583847?include=energy
Dnajc17	0.885	http://api.brain-map.org/grid_data/download/69059952?include=energy
St8sia2	0.885	http://api.brain-map.org/grid_data/download/77925023?include=energy
Zfp846	0.885	http://api.brain-map.org/grid_data/download/71283468?include=energy
Snupn	0.885	http://api.brain-map.org/grid_data/download/71764219?include=energy
Srd5a2	0.885	http://api.brain-map.org/grid_data/download/72471827?include=energy
Brms1	0.885	http://api.brain-map.org/grid_data/download/71763741?include=energy
Pcca	0.885	http://api.brain-map.org/grid_data/download/386263?include=energy
Hpn	0.885	http://api.brain-map.org/grid_data/download/74003382?include=energy
A430033K04Rik	0.885	http://api.brain-map.org/grid_data/download/69014458?include=energy
Zkscan4	0.885	http://api.brain-map.org/grid_data/download/71213268?include=energy
Mm.91240	0.885	http://api.brain-map.org/grid_data/download/71210466?include=energy
Col9a1	0.885	http://api.brain-map.org/grid_data/download/71064061?include=energy
Prrc2b	0.885	http://api.brain-map.org/grid_data/download/68797673?include=energy
Slc19a3	0.885	http://api.brain-map.org/grid_data/download/69873833?include=energy
Tbc1d23	0.885	http://api.brain-map.org/grid_data/download/68638125?include=energy
Fkbp14	0.885	http://api.brain-map.org/grid_data/download/70528659?include=energy
5730596B20Rik	0.884	http://api.brain-map.org/grid_data/download/69443529?include=energy
Lipe	0.884	http://api.brain-map.org/grid_data/download/69059801?include=energy
Ugt1a1	0.884	http://api.brain-map.org/grid_data/download/70302890?include=energy
Lin52	0.884	http://api.brain-map.org/grid_data/download/69444279?include=energy
Msh3	0.884	http://api.brain-map.org/grid_data/download/72076611?include=energy
Kdelr3	0.884	http://api.brain-map.org/grid_data/download/112203270?include=energy
Lima1	0.884	http://api.brain-map.org/grid_data/download/68443507?include=energy
Ldhal6b	0.884	http://api.brain-map.org/grid_data/download/69117038?include=energy
Ptpn22	0.884	http://api.brain-map.org/grid_data/download/68743763?include=energy
Csnk1a1	0.884	http://api.brain-map.org/grid_data/download/74658110?include=energy
Abr	0.884	http://api.brain-map.org/grid_data/download/70305383?include=energy
Myl2	0.884	http://api.brain-map.org/grid_data/download/70784666?include=energy
Emp1	0.884	http://api.brain-map.org/grid_data/download/77280421?include=energy
Pcdha3	0.884	http://api.brain-map.org/grid_data/download/79354806?include=energy
Gm9801	0.884	http://api.brain-map.org/grid_data/download/70305239?include=energy
Cacna1h	0.884	http://api.brain-map.org/grid_data/download/72119638?include=energy
Pla2g4a	0.884	http://api.brain-map.org/grid_data/download/68076543?include=energy
Gpr34	0.884	http://api.brain-map.org/grid_data/download/69257540?include=energy
Nsmce4a	0.884	http://api.brain-map.org/grid_data/download/72283767?include=energy
Ano7	0.884	http://api.brain-map.org/grid_data/download/69782228?include=energy
Itpr2	0.884	http://api.brain-map.org/grid_data/download/69257987?include=energy
Mthfsd	0.884	http://api.brain-map.org/grid_data/download/68799113?include=energy
Vax1	0.884	http://api.brain-map.org/grid_data/download/81655570?include=energy
Sdc3	0.884	http://api.brain-map.org/grid_data/download/71358662?include=energy
Zbtb48	0.884	http://api.brain-map.org/grid_data/download/1940?include=energy
Hist1h2be	0.884	http://api.brain-map.org/grid_data/download/69735042?include=energy
Lrrc32	0.884	http://api.brain-map.org/grid_data/download/70787591?include=energy
Smad1	0.884	http://api.brain-map.org/grid_data/download/70593359?include=energy
Clec12a	0.884	http://api.brain-map.org/grid_data/download/76003342?include=energy
2310033E01Rik	0.884	http://api.brain-map.org/grid_data/download/74359377?include=energy
Syn3	0.884	http://api.brain-map.org/grid_data/download/73930808?include=energy
Cflar	0.884	http://api.brain-map.org/grid_data/download/71212494?include=energy
Ormdl1	0.884	http://api.brain-map.org/grid_data/download/75826638?include=energy
Gulp1	0.884	http://api.brain-map.org/grid_data/download/74635755?include=energy
Ptbp2	0.884	http://api.brain-map.org/grid_data/download/71724010?include=energy
Slc7a8	0.884	http://api.brain-map.org/grid_data/download/72001998?include=energy
Supt4h1	0.884	http://api.brain-map.org/grid_data/download/69117044?include=energy
Dsel	0.884	http://api.brain-map.org/grid_data/download/71249745?include=energy
Cdk12	0.884	http://api.brain-map.org/grid_data/download/70238004?include=energy
Agpat1	0.884	http://api.brain-map.org/grid_data/download/68269682?include=energy
Snrpd1	0.884	http://api.brain-map.org/grid_data/download/70565252?include=energy
Dyrk3	0.884	http://api.brain-map.org/grid_data/download/293891?include=energy
Atp5j	0.884	http://api.brain-map.org/grid_data/download/124?include=energy
Mzt2	0.884	http://api.brain-map.org/grid_data/download/68744863?include=energy
Rltpr	0.884	http://api.brain-map.org/grid_data/download/74578182?include=energy
Lefty1	0.884	http://api.brain-map.org/grid_data/download/68667216?include=energy
Slc7a14	0.884	http://api.brain-map.org/grid_data/download/70436744?include=energy
Sptlc1	0.884	http://api.brain-map.org/grid_data/download/68745588?include=energy
Fpgt	0.884	http://api.brain-map.org/grid_data/download/69672563?include=energy
Mea1	0.884	http://api.brain-map.org/grid_data/download/70429297?include=energy
Ifi202b	0.884	http://api.brain-map.org/grid_data/download/68301742?include=energy
Dpysl4	0.884	http://api.brain-map.org/grid_data/download/1345?include=energy
Zfp786	0.884	http://api.brain-map.org/grid_data/download/70593158?include=energy
Hs6st1	0.884	http://api.brain-map.org/grid_data/download/74819258?include=energy
Ltbr	0.884	http://api.brain-map.org/grid_data/download/71378328?include=energy
LOC432692	0.884	http://api.brain-map.org/grid_data/download/70611228?include=energy
Cdh23	0.883	http://api.brain-map.org/grid_data/download/72283805?include=energy
Ckap4	0.883	http://api.brain-map.org/grid_data/download/69548964?include=energy
Emp2	0.883	http://api.brain-map.org/grid_data/download/69838242?include=energy
Mlst8	0.883	http://api.brain-map.org/grid_data/download/69059955?include=energy
March10	0.883	http://api.brain-map.org/grid_data/download/73906467?include=energy
Adcy4	0.883	http://api.brain-map.org/grid_data/download/73512495?include=energy
Ankfn1	0.883	http://api.brain-map.org/grid_data/download/74047714?include=energy
Zfp330	0.883	http://api.brain-map.org/grid_data/download/75081398?include=energy
Dedd	0.883	http://api.brain-map.org/grid_data/download/80514095?include=energy
LOC432463	0.883	http://api.brain-map.org/grid_data/download/70608541?include=energy
Atg9a	0.883	http://api.brain-map.org/grid_data/download/71495837?include=energy
Mm.205191	0.883	http://api.brain-map.org/grid_data/download/71152348?include=energy
Gykl1	0.883	http://api.brain-map.org/grid_data/download/70276891?include=energy
E230019M04Rik	0.883	http://api.brain-map.org/grid_data/download/75197576?include=energy
D6Ertd474e	0.883	http://api.brain-map.org/grid_data/download/70238716?include=energy
Ldoc1l	0.883	http://api.brain-map.org/grid_data/download/69169953?include=energy
Nek6	0.883	http://api.brain-map.org/grid_data/download/70546263?include=energy
Dennd4b	0.883	http://api.brain-map.org/grid_data/download/74431041?include=energy
S100g	0.883	http://api.brain-map.org/grid_data/download/75694876?include=energy
Vps25	0.883	http://api.brain-map.org/grid_data/download/69261415?include=energy
Gm1795	0.883	http://api.brain-map.org/grid_data/download/73513792?include=energy
Ybx1	0.883	http://api.brain-map.org/grid_data/download/743?include=energy
Dnajc7	0.883	http://api.brain-map.org/grid_data/download/68795402?include=energy
Rfx7	0.883	http://api.brain-map.org/grid_data/download/1998?include=energy
Polr2h	0.883	http://api.brain-map.org/grid_data/download/68191474?include=energy
Rabep1	0.883	http://api.brain-map.org/grid_data/download/70812877?include=energy
8030447O22Rik*	0.883	http://api.brain-map.org/grid_data/download/73927739?include=energy
Taf3	0.883	http://api.brain-map.org/grid_data/download/69290567?include=energy
Zfp189	0.883	http://api.brain-map.org/grid_data/download/79490078?include=energy
Parg	0.883	http://api.brain-map.org/grid_data/download/69782748?include=energy
Unc93b1	0.883	http://api.brain-map.org/grid_data/download/68157402?include=energy
Capn7	0.883	http://api.brain-map.org/grid_data/download/75931510?include=energy
Qrfp	0.883	http://api.brain-map.org/grid_data/download/75041585?include=energy
2310061C15Rik	0.883	http://api.brain-map.org/grid_data/download/68744916?include=energy
Sap18	0.883	http://api.brain-map.org/grid_data/download/75990507?include=energy
Lypla1	0.883	http://api.brain-map.org/grid_data/download/74047772?include=energy
Megf9	0.883	http://api.brain-map.org/grid_data/download/74658078?include=energy
Tmem38b	0.883	http://api.brain-map.org/grid_data/download/68301664?include=energy
Nrm	0.883	http://api.brain-map.org/grid_data/download/75042265?include=energy
Sass6	0.883	http://api.brain-map.org/grid_data/download/68667401?include=energy
Gm5446	0.883	http://api.brain-map.org/grid_data/download/75043605?include=energy
Pcdhga10	0.883	http://api.brain-map.org/grid_data/download/79354798?include=energy
Hrg	0.883	http://api.brain-map.org/grid_data/download/68302233?include=energy
Trim34b	0.883	http://api.brain-map.org/grid_data/download/70787597?include=energy
Mtch1	0.883	http://api.brain-map.org/grid_data/download/67764936?include=energy
Bcl2a1b	0.883	http://api.brain-map.org/grid_data/download/68523282?include=energy
Polr2f	0.883	http://api.brain-map.org/grid_data/download/75861880?include=energy
Tenc1	0.883	http://api.brain-map.org/grid_data/download/68845711?include=energy
Pdhx	0.883	http://api.brain-map.org/grid_data/download/70546347?include=energy
Svopl	0.883	http://api.brain-map.org/grid_data/download/69835609?include=energy
Iqsec1	0.883	http://api.brain-map.org/grid_data/download/77332673?include=energy
Xpc	0.883	http://api.brain-map.org/grid_data/download/70195048?include=energy
Adar	0.883	http://api.brain-map.org/grid_data/download/68845400?include=energy
Mm.207689	0.883	http://api.brain-map.org/grid_data/download/71152366?include=energy
Olfr1099	0.883	http://api.brain-map.org/grid_data/download/75214737?include=energy
Tmem86a	0.883	http://api.brain-map.org/grid_data/download/100144479?include=energy
Utp18	0.883	http://api.brain-map.org/grid_data/download/69444777?include=energy
Dcdc2a	0.883	http://api.brain-map.org/grid_data/download/68745420?include=energy
Cnot3	0.883	http://api.brain-map.org/grid_data/download/69549668?include=energy
A730037L19Rik	0.883	http://api.brain-map.org/grid_data/download/71494726?include=energy
Trpc5	0.883	http://api.brain-map.org/grid_data/download/71247658?include=energy
Cdipt	0.883	http://api.brain-map.org/grid_data/download/76098296?include=energy
Cntnap4	0.883	http://api.brain-map.org/grid_data/download/70928361?include=energy
Gm5149	0.883	http://api.brain-map.org/grid_data/download/72472462?include=energy
Mm.233461	0.883	http://api.brain-map.org/grid_data/download/71152575?include=energy
Xlr4b	0.882	http://api.brain-map.org/grid_data/download/75828174?include=energy
Ube3a	0.882	http://api.brain-map.org/grid_data/download/79591689?include=energy
C330024D21Rik	0.882	http://api.brain-map.org/grid_data/download/74300795?include=energy
BB288505	0.882	http://api.brain-map.org/grid_data/download/74514282?include=energy
Nek8	0.882	http://api.brain-map.org/grid_data/download/73818811?include=energy
C030002J06Rik	0.882	http://api.brain-map.org/grid_data/download/71809172?include=energy
LOC433829	0.882	http://api.brain-map.org/grid_data/download/70786864?include=energy
Miat	0.882	http://api.brain-map.org/grid_data/download/72340194?include=energy
Arhgef38	0.882	http://api.brain-map.org/grid_data/download/69262307?include=energy
Cdkn1c	0.882	http://api.brain-map.org/grid_data/download/75198136?include=energy
Casp6	0.882	http://api.brain-map.org/grid_data/download/79749597?include=energy
Nxph3	0.882	http://api.brain-map.org/grid_data/download/356852?include=energy
Ift81	0.882	http://api.brain-map.org/grid_data/download/71656862?include=energy
Krtap24-1	0.882	http://api.brain-map.org/grid_data/download/70296056?include=energy
Prim2	0.882	http://api.brain-map.org/grid_data/download/68795443?include=energy
Chst14	0.882	http://api.brain-map.org/grid_data/download/74657927?include=energy
Rexo1	0.882	http://api.brain-map.org/grid_data/download/69980202?include=energy
Pik3ip1	0.882	http://api.brain-map.org/grid_data/download/69014728?include=energy
Suv420h2	0.882	http://api.brain-map.org/grid_data/download/68148736?include=energy
Agk	0.882	http://api.brain-map.org/grid_data/download/70430680?include=energy
Ly9	0.882	http://api.brain-map.org/grid_data/download/76003682?include=energy
Naaladl2	0.882	http://api.brain-map.org/grid_data/download/70298591?include=energy
Socs5	0.882	http://api.brain-map.org/grid_data/download/74274685?include=energy
Cyp2t4	0.882	http://api.brain-map.org/grid_data/download/70300115?include=energy
F830002L21Rik	0.882	http://api.brain-map.org/grid_data/download/73767311?include=energy
Tnni2	0.882	http://api.brain-map.org/grid_data/download/70565333?include=energy
Rtp4	0.882	http://api.brain-map.org/grid_data/download/68444738?include=energy
LOC434348	0.882	http://api.brain-map.org/grid_data/download/70920111?include=energy
Mkl1	0.882	http://api.brain-map.org/grid_data/download/70593310?include=energy
Arcn1	0.882	http://api.brain-map.org/grid_data/download/583567?include=energy
Plxna4	0.882	http://api.brain-map.org/grid_data/download/75084090?include=energy
Rxfp4	0.882	http://api.brain-map.org/grid_data/download/69257391?include=energy
Rbm31y	0.882	http://api.brain-map.org/grid_data/download/77620253?include=energy
Heatr1	0.882	http://api.brain-map.org/grid_data/download/68410273?include=energy
Mlh3	0.882	http://api.brain-map.org/grid_data/download/69134505?include=energy
Gjb5	0.882	http://api.brain-map.org/grid_data/download/2259?include=energy
Cnn3	0.882	http://api.brain-map.org/grid_data/download/74822928?include=energy
4833413E03Rik	0.882	http://api.brain-map.org/grid_data/download/74076156?include=energy

Il4ra	0.882	http://api.brain-map.org/grid_data/download/77413692?include=energy
Sim1	0.882	http://api.brain-map.org/grid_data/download/72338698?include=energy
BC028528	0.882	http://api.brain-map.org/grid_data/download/68744636?include=energy
Wasf1	0.882	http://api.brain-map.org/grid_data/download/69059954?include=energy
4930564B18Rik	0.882	http://api.brain-map.org/grid_data/download/71891988?include=energy
Pmf1	0.882	http://api.brain-map.org/grid_data/download/74822930?include=energy
Igfbp3	0.882	http://api.brain-map.org/grid_data/download/74272920?include=energy
Olfr734	0.882	http://api.brain-map.org/grid_data/download/74734914?include=energy
Gm962	0.882	http://api.brain-map.org/grid_data/download/70298507?include=energy
Larp7	0.882	http://api.brain-map.org/grid_data/download/71022864?include=energy
Tas2r143	0.882	http://api.brain-map.org/grid_data/download/70785200?include=energy
Syn2	0.882	http://api.brain-map.org/grid_data/download/73929870?include=energy
Amac1	0.882	http://api.brain-map.org/grid_data/download/71495753?include=energy
Taar4	0.882	http://api.brain-map.org/grid_data/download/73497217?include=energy
Cdc23	0.881	http://api.brain-map.org/grid_data/download/69261958?include=energy
Hey1	0.881	http://api.brain-map.org/grid_data/download/69289081?include=energy
4933412E24Rik	0.881	http://api.brain-map.org/grid_data/download/69443166?include=energy
Syf2	0.881	http://api.brain-map.org/grid_data/download/69549608?include=energy
Brd9	0.881	http://api.brain-map.org/grid_data/download/71213093?include=energy
Klf14	0.881	http://api.brain-map.org/grid_data/download/69443343?include=energy
Kdm3b	0.881	http://api.brain-map.org/grid_data/download/71210995?include=energy
5430407P10Rik	0.881	http://api.brain-map.org/grid_data/download/70445490?include=energy
1810065E05Rik	0.881	http://api.brain-map.org/grid_data/download/68637790?include=energy
Slc35f2	0.881	http://api.brain-map.org/grid_data/download/74819683?include=energy
Recql4	0.881	http://api.brain-map.org/grid_data/download/75084132?include=energy
LOC432958	0.881	http://api.brain-map.org/grid_data/download/71808460?include=energy
Dpy19l4	0.881	http://api.brain-map.org/grid_data/download/70299893?include=energy
Sema4f	0.881	http://api.brain-map.org/grid_data/download/79544850?include=energy
Dclre1c	0.881	http://api.brain-map.org/grid_data/download/70946403?include=energy
Mid1ip1	0.881	http://api.brain-map.org/grid_data/download/68269063?include=energy
6430565L09Rik*	0.881	http://api.brain-map.org/grid_data/download/73906047?include=energy
Syngap1	0.881	http://api.brain-map.org/grid_data/download/71020897?include=energy
Slc37a1	0.881	http://api.brain-map.org/grid_data/download/78534512?include=energy
Mm.320785	0.881	http://api.brain-map.org/grid_data/download/71158873?include=energy
Bdh1	0.881	http://api.brain-map.org/grid_data/download/68861717?include=energy
Nlrp1a	0.881	http://api.brain-map.org/grid_data/download/70276903?include=energy
Ovch2	0.881	http://api.brain-map.org/grid_data/download/73818812?include=energy
Lgi4	0.881	http://api.brain-map.org/grid_data/download/69736112?include=energy
Myoz2	0.881	http://api.brain-map.org/grid_data/download/68499467?include=energy
Mthfd2l	0.881	http://api.brain-map.org/grid_data/download/71212003?include=energy
Fance	0.881	http://api.brain-map.org/grid_data/download/67767451?include=energy
Bst1	0.881	http://api.brain-map.org/grid_data/download/71496007?include=energy
Epb4.2	0.881	http://api.brain-map.org/grid_data/download/69672072?include=energy
6330444E15Rik	0.881	http://api.brain-map.org/grid_data/download/69444849?include=energy
Vmn1r171	0.881	http://api.brain-map.org/grid_data/download/74056204?include=energy
Ryr1	0.881	http://api.brain-map.org/grid_data/download/71247657?include=energy
Edc4	0.881	http://api.brain-map.org/grid_data/download/68911011?include=energy
H2-Eb1	0.881	http://api.brain-map.org/grid_data/download/73520983?include=energy
Fasl	0.881	http://api.brain-map.org/grid_data/download/68666577?include=energy
Ddx31	0.881	http://api.brain-map.org/grid_data/download/70946443?include=energy
D730002M21Rik	0.881	http://api.brain-map.org/grid_data/download/70238728?include=energy
Polr1a	0.881	http://api.brain-map.org/grid_data/download/68666900?include=energy
Pds5b	0.881	http://api.brain-map.org/grid_data/download/71283592?include=energy
Fam173a	0.881	http://api.brain-map.org/grid_data/download/70919256?include=energy
Gm5091	0.881	http://api.brain-map.org/grid_data/download/70305562?include=energy
Nebl	0.881	http://api.brain-map.org/grid_data/download/70437804?include=energy
Vstm2a	0.881	http://api.brain-map.org/grid_data/download/71836843?include=energy
Exoc6b	0.881	http://api.brain-map.org/grid_data/download/74821914?include=energy
Rnf222	0.881	http://api.brain-map.org/grid_data/download/70228286?include=energy
Bhlhb9	0.881	http://api.brain-map.org/grid_data/download/74274689?include=energy
Eif5	0.881	http://api.brain-map.org/grid_data/download/1704?include=energy
2810043O03Rik	0.881	http://api.brain-map.org/grid_data/download/70919017?include=energy
Srpk2	0.881	http://api.brain-map.org/grid_data/download/634451?include=energy
Mak16	0.881	http://api.brain-map.org/grid_data/download/70445449?include=energy
H2-Ke6	0.881	http://api.brain-map.org/grid_data/download/71529893?include=energy
D17Wsu104e	0.881	http://api.brain-map.org/grid_data/download/71280994?include=energy
Ceacam5	0.881	http://api.brain-map.org/grid_data/download/70300941?include=energy
Sirt3	0.881	http://api.brain-map.org/grid_data/download/68117984?include=energy
Fbxw11	0.881	http://api.brain-map.org/grid_data/download/532691?include=energy
Phpt1	0.881	http://api.brain-map.org/grid_data/download/68523195?include=energy
Art1	0.881	http://api.brain-map.org/grid_data/download/68798364?include=energy
C030033M19Rik	0.88	http://api.brain-map.org/grid_data/download/69013774?include=energy
Zfp184	0.88	http://api.brain-map.org/grid_data/download/68799119?include=energy
Pot1b	0.88	http://api.brain-map.org/grid_data/download/73925715?include=energy
Lep	0.88	http://api.brain-map.org/grid_data/download/69736082?include=energy
Pkhd1	0.88	http://api.brain-map.org/grid_data/download/69783232?include=energy
C130096N06Rik	0.88	http://api.brain-map.org/grid_data/download/73635978?include=energy
Fastkd2	0.88	http://api.brain-map.org/grid_data/download/69028533?include=energy
D7Ertd715e	0.88	http://api.brain-map.org/grid_data/download/73513699?include=energy
Maz	0.88	http://api.brain-map.org/grid_data/download/71488768?include=energy
Adipoq	0.88	http://api.brain-map.org/grid_data/download/68545033?include=energy
Rgs19	0.88	http://api.brain-map.org/grid_data/download/112203487?include=energy
Dstn	0.88	http://api.brain-map.org/grid_data/download/74658076?include=energy
D10Wsu102e	0.88	http://api.brain-map.org/grid_data/download/69608435?include=energy
Zcchc8	0.88	http://api.brain-map.org/grid_data/download/686206?include=energy
Fam32a	0.88	http://api.brain-map.org/grid_data/download/275684?include=energy
Bmp7	0.88	http://api.brain-map.org/grid_data/download/2065?include=energy
Gatad1	0.88	http://api.brain-map.org/grid_data/download/70546311?include=energy
4933411K16Rik	0.88	http://api.brain-map.org/grid_data/download/74357827?include=energy
Spr	0.88	http://api.brain-map.org/grid_data/download/69095979?include=energy
Alpk3	0.88	http://api.brain-map.org/grid_data/download/71574473?include=energy
Fbxl5	0.88	http://api.brain-map.org/grid_data/download/422?include=energy
Cux2	0.88	http://api.brain-map.org/grid_data/download/72128748?include=energy
Sepx1	0.88	http://api.brain-map.org/grid_data/download/69873546?include=energy
Itfg2	0.88	http://api.brain-map.org/grid_data/download/70539170?include=energy
Phf19	0.88	http://api.brain-map.org/grid_data/download/68157404?include=energy
Msl3	0.88	http://api.brain-map.org/grid_data/download/75990555?include=energy
Hpdl	0.88	http://api.brain-map.org/grid_data/download/79354762?include=energy
Pinx1	0.88	http://api.brain-map.org/grid_data/download/72283731?include=energy
Ids	0.88	http://api.brain-map.org/grid_data/download/73994671?include=energy
Dbt	0.88	http://api.brain-map.org/grid_data/download/69838563?include=energy
Zfp804b	0.88	http://api.brain-map.org/grid_data/download/73931632?include=energy
Nynrin	0.88	http://api.brain-map.org/grid_data/download/69529047?include=energy
Mx1	0.88	http://api.brain-map.org/grid_data/download/1385?include=energy
Cxcl2	0.88	http://api.brain-map.org/grid_data/download/69608309?include=energy
Serpina12	0.88	http://api.brain-map.org/grid_data/download/75831716?include=energy
LOC432690	0.88	http://api.brain-map.org/grid_data/download/70611216?include=energy
Ifi47	0.88	http://api.brain-map.org/grid_data/download/68191402?include=energy
0610037L13Rik	0.88	http://api.brain-map.org/grid_data/download/556980?include=energy
Tmc3	0.88	http://api.brain-map.org/grid_data/download/69981061?include=energy
Glce	0.88	http://api.brain-map.org/grid_data/download/74641306?include=energy
Hus1b	0.88	http://api.brain-map.org/grid_data/download/70277979?include=energy
Sin3a	0.88	http://api.brain-map.org/grid_data/download/69816852?include=energy
F830221A01Rik*	0.88	http://api.brain-map.org/grid_data/download/73748722?include=energy
Rwdd1	0.88	http://api.brain-map.org/grid_data/download/72000624?include=energy
Inppl1	0.88	http://api.brain-map.org/grid_data/download/75694364?include=energy
Pask	0.88	http://api.brain-map.org/grid_data/download/71920490?include=energy
Mapre1	0.88	http://api.brain-map.org/grid_data/download/2401?include=energy
Tas2r138	0.88	http://api.brain-map.org/grid_data/download/70302407?include=energy
Kcnj1	0.88	http://api.brain-map.org/grid_data/download/100144109?include=energy
Wars	0.88	http://api.brain-map.org/grid_data/download/70919722?include=energy
Ppfibp2	0.88	http://api.brain-map.org/grid_data/download/69817193?include=energy
Plb1	0.88	http://api.brain-map.org/grid_data/download/68162220?include=energy
Snrk	0.88	http://api.brain-map.org/grid_data/download/72182993?include=energy
Tmc7	0.88	http://api.brain-map.org/grid_data/download/71809073?include=energy
Ston1	0.88	http://api.brain-map.org/grid_data/download/70610391?include=energy
Srgap3	0.88	http://api.brain-map.org/grid_data/download/316?include=energy
Esco1	0.88	http://api.brain-map.org/grid_data/download/69169313?include=energy
Skint4	0.88	http://api.brain-map.org/grid_data/download/71378749?include=energy
Tnfrsf8	0.88	http://api.brain-map.org/grid_data/download/74431472?include=energy
Scaf4	0.88	http://api.brain-map.org/grid_data/download/73679038?include=energy
Wdtc1	0.88	http://api.brain-map.org/grid_data/download/68924425?include=energy
Lias	0.88	http://api.brain-map.org/grid_data/download/68076529?include=energy
Wfdc6a	0.88	http://api.brain-map.org/grid_data/download/70295584?include=energy
Cebpa	0.88	http://api.brain-map.org/grid_data/download/80343361?include=energy
Lrat	0.88	http://api.brain-map.org/grid_data/download/70300204?include=energy
Gm581	0.879	http://api.brain-map.org/grid_data/download/71378787?include=energy
Tgm5	0.879	http://api.brain-map.org/grid_data/download/75990420?include=energy
Tm2d1	0.879	http://api.brain-map.org/grid_data/download/69236861?include=energy
Mocs2	0.879	http://api.brain-map.org/grid_data/download/74990524?include=energy
Pcdhb12	0.879	http://api.brain-map.org/grid_data/download/79354782?include=energy
Rictor	0.879	http://api.brain-map.org/grid_data/download/69407791?include=energy
Mob3c	0.879	http://api.brain-map.org/grid_data/download/71789831?include=energy
Smc3	0.879	http://api.brain-map.org/grid_data/download/70523716?include=energy
TC1533832	0.879	http://api.brain-map.org/grid_data/download/70813335?include=energy
A430090L17Rik	0.879	http://api.brain-map.org/grid_data/download/71809253?include=energy
Oxa1l	0.879	http://api.brain-map.org/grid_data/download/532710?include=energy
LOC433706	0.879	http://api.brain-map.org/grid_data/download/74822600?include=energy
Gtf2h2	0.879	http://api.brain-map.org/grid_data/download/70634256?include=energy
Fam117b	0.879	http://api.brain-map.org/grid_data/download/69526570?include=energy
4833432P19Rik	0.879	http://api.brain-map.org/grid_data/download/69407767?include=energy
H2-DMa	0.879	http://api.brain-map.org/grid_data/download/68191438?include=energy
Olfr356	0.879	http://api.brain-map.org/grid_data/download/74724037?include=energy
Frat1	0.879	http://api.brain-map.org/grid_data/download/81654117?include=energy
Rbm7	0.879	http://api.brain-map.org/grid_data/download/69818036?include=energy
Tubgcp6	0.879	http://api.brain-map.org/grid_data/download/73834403?include=energy
Tdp1	0.879	http://api.brain-map.org/grid_data/download/641538?include=energy
Arpp19	0.879	http://api.brain-map.org/grid_data/download/74357597?include=energy
4930453N24Rik	0.879	http://api.brain-map.org/grid_data/download/68341848?include=energy
LOC435201	0.879	http://api.brain-map.org/grid_data/download/71022445?include=energy
Trib2	0.879	http://api.brain-map.org/grid_data/download/71587811?include=energy
Stradb	0.879	http://api.brain-map.org/grid_data/download/76003614?include=energy
Hoxa11	0.879	http://api.brain-map.org/grid_data/download/69289135?include=energy
Fgfrl1	0.879	http://api.brain-map.org/grid_data/download/75861795?include=energy
C2	0.879	http://api.brain-map.org/grid_data/download/68301772?include=energy
Izumo2	0.879	http://api.brain-map.org/grid_data/download/74427102?include=energy
Tmod3	0.879	http://api.brain-map.org/grid_data/download/77464990?include=energy
0910001L09Rik	0.879	http://api.brain-map.org/grid_data/download/69352814?include=energy
Nmbr	0.879	http://api.brain-map.org/grid_data/download/77332086?include=energy
Arf3	0.879	http://api.brain-map.org/grid_data/download/110?include=energy
Fbxo42	0.879	http://api.brain-map.org/grid_data/download/71232662?include=energy
Olfr112	0.879	http://api.brain-map.org/grid_data/download/69855728?include=energy
Selm	0.879	http://api.brain-map.org/grid_data/download/641541?include=energy
Tyr	0.879	http://api.brain-map.org/grid_data/download/2703?include=energy
Arr3	0.879	http://api.brain-map.org/grid_data/download/68342318?include=energy
4921530L21Rik	0.879	http://api.brain-map.org/grid_data/download/69113751?include=energy
Adcyap1r1	0.879	http://api.brain-map.org/grid_data/download/74988667?include=energy
5330432E05Rik	0.879	http://api.brain-map.org/grid_data/download/70723498?include=energy
Mri1	0.879	http://api.brain-map.org/grid_data/download/75773703?include=energy
Peli1	0.879	http://api.brain-map.org/grid_data/download/583865?include=energy
3110082J24Rik	0.879	http://api.brain-map.org/grid_data/download/74736249?include=energy
Gm5607	0.879	http://api.brain-map.org/grid_data/download/72109337?include=energy
Efna2	0.879	http://api.brain-map.org/grid_data/download/100146160?include=energy
Hint2	0.879	http://api.brain-map.org/grid_data/download/76003661?include=energy
1700112E06Rik	0.879	http://api.brain-map.org/grid_data/download/70201516?include=energy
Ooep	0.879	http://api.brain-map.org/grid_data/download/77465016?include=energy
Padi1	0.879	http://api.brain-map.org/grid_data/download/71234732?include=energy
Xpo7	0.879	http://api.brain-map.org/grid_data/download/68151347?include=energy
Rrp15	0.879	http://api.brain-map.org/grid_data/download/68795457?include=energy
Ccr3	0.879	http://api.brain-map.org/grid_data/download/69237017?include=energy
Larp1b	0.879	http://api.brain-map.org/grid_data/download/74635132?include=energy
Sgcd	0.879	http://api.brain-map.org/grid_data/download/71717142?include=energy
Zyx	0.879	http://api.brain-map.org/grid_data/download/71670710?include=energy
Tas2r140	0.879	http://api.brain-map.org/grid_data/download/70302401?include=energy
Acot2	0.879	http://api.brain-map.org/grid_data/download/71210526?include=energy
Pip5k1a	0.879	http://api.brain-map.org/grid_data/download/69079998?include=energy
Akt1s1	0.879	http://api.brain-map.org/grid_data/download/71380556?include=energy
Elac2	0.879	http://api.brain-map.org/grid_data/download/70795886?include=energy
Zfp61	0.879	http://api.brain-map.org/grid_data/download/75749409?include=energy
Cxcl15	0.879	http://api.brain-map.org/grid_data/download/75990684?include=energy
Rbp1	0.879	http://api.brain-map.org/grid_data/download/68076899?include=energy
Mest	0.879	http://api.brain-map.org/grid_data/download/71717082?include=energy
Wdr46	0.879	http://api.brain-map.org/grid_data/download/68745203?include=energy
Mylk3	0.879	http://api.brain-map.org/grid_data/download/73992942?include=energy
Tekt3	0.879	http://api.brain-map.org/grid_data/download/75990419?include=energy
Agphd1	0.879	http://api.brain-map.org/grid_data/download/70719035?include=energy
Hsd17b6	0.879	http://api.brain-map.org/grid_data/download/75990827?include=energy
Tjp2	0.879	http://api.brain-map.org/grid_data/download/68191132?include=energy
Sel1l	0.879	http://api.brain-map.org/grid_data/download/70793929?include=energy
Stom	0.879	http://api.brain-map.org/grid_data/download/69080003?include=energy
Mepce	0.879	http://api.brain-map.org/grid_data/download/67792211?include=energy
Iqck	0.879	http://api.brain-map.org/grid_data/download/74578323?include=energy
Slc9a8	0.879	http://api.brain-map.org/grid_data/download/1852?include=energy
Snx7	0.879	http://api.brain-map.org/grid_data/download/71456141?include=energy
Nt5e	0.879	http://api.brain-map.org/grid_data/download/74821722?include=energy
Zbtb34	0.878	http://api.brain-map.org/grid_data/download/76081015?include=energy
Timm50	0.878	http://api.brain-map.org/grid_data/download/68077012?include=energy
Cyp4v3	0.878	http://api.brain-map.org/grid_data/download/74882196?include=energy
6030470M02Rik	0.878	http://api.brain-map.org/grid_data/download/71380520?include=energy
Bahcc1	0.878	http://api.brain-map.org/grid_data/download/75988301?include=energy
Eif6	0.878	http://api.brain-map.org/grid_data/download/686209?include=energy
6330549D23Rik	0.878	http://api.brain-map.org/grid_data/download/69444891?include=energy
Akirin1	0.878	http://api.brain-map.org/grid_data/download/81599624?include=energy
Pkd1l2	0.878	http://api.brain-map.org/grid_data/download/69258298?include=energy
Mff	0.878	http://api.brain-map.org/grid_data/download/71486943?include=energy
LOC432979	0.878	http://api.brain-map.org/grid_data/download/70611449?include=energy
Gstcd	0.878	http://api.brain-map.org/grid_data/download/69116883?include=energy
Tlk2	0.878	http://api.brain-map.org/grid_data/download/69981019?include=energy
1110002L01Rik	0.878	http://api.brain-map.org/grid_data/download/70200848?include=energy
5330426P16Rik	0.878	http://api.brain-map.org/grid_data/download/71211314?include=energy
Dnajb5	0.878	http://api.brain-map.org/grid_data/download/69549640?include=energy
Vash2	0.878	http://api.brain-map.org/grid_data/download/1667?include=energy
Abcd4	0.878	http://api.brain-map.org/grid_data/download/68667091?include=energy
Tnfsf13	0.878	http://api.brain-map.org/grid_data/download/75934513?include=energy
Cfc1	0.878	http://api.brain-map.org/grid_data/download/81654125?include=energy
Vmn1r229	0.878	http://api.brain-map.org/grid_data/download/70785285?include=energy
LOC432406	0.878	http://api.brain-map.org/grid_data/download/70608448?include=energy
Smu1	0.878	http://api.brain-map.org/grid_data/download/74819686?include=energy
Gdap10	0.878	http://api.brain-map.org/grid_data/download/74357577?include=energy
Fert2	0.878	http://api.brain-map.org/grid_data/download/70523685?include=energy
Wnt4	0.878	http://api.brain-map.org/grid_data/download/1444?include=energy
Aanat	0.878	http://api.brain-map.org/grid_data/download/80514039?include=energy
LOC434409	0.878	http://api.brain-map.org/grid_data/download/70725597?include=energy
9930038K12Rik	0.878	http://api.brain-map.org/grid_data/download/100146046?include=energy
Lims1	0.878	http://api.brain-map.org/grid_data/download/73514731?include=energy
Atg12	0.878	http://api.brain-map.org/grid_data/download/74881536?include=energy
Myst2	0.878	http://api.brain-map.org/grid_data/download/69012971?include=energy
Nme1	0.878	http://api.brain-map.org/grid_data/download/75144617?include=energy
G6pdx	0.878	http://api.brain-map.org/grid_data/download/69672695?include=energy
Kdm5a	0.878	http://api.brain-map.org/grid_data/download/74990486?include=energy
Agtrap	0.878	http://api.brain-map.org/grid_data/download/69028581?include=energy
Slc25a34	0.878	http://api.brain-map.org/grid_data/download/72472399?include=energy
Rbm47	0.878	http://api.brain-map.org/grid_data/download/68341292?include=energy
Bsx	0.878	http://api.brain-map.org/grid_data/download/71281704?include=energy
Olfr1394	0.878	http://api.brain-map.org/grid_data/download/74720805?include=energy
Klhdc10	0.878	http://api.brain-map.org/grid_data/download/74635499?include=energy
Mm.359599	0.878	http://api.brain-map.org/grid_data/download/71209916?include=energy
Agap2	0.878	http://api.brain-map.org/grid_data/download/73513639?include=energy
Tmem27	0.878	http://api.brain-map.org/grid_data/download/69262330?include=energy
Tsnax	0.878	http://api.brain-map.org/grid_data/download/393252?include=energy
Il15	0.878	http://api.brain-map.org/grid_data/download/68158076?include=energy
Abhd14a	0.878	http://api.brain-map.org/grid_data/download/413803?include=energy
Rabif	0.878	http://api.brain-map.org/grid_data/download/75826578?include=energy
Tas2r124	0.878	http://api.brain-map.org/grid_data/download/70785182?include=energy
Brca1	0.878	http://api.brain-map.org/grid_data/download/75988313?include=energy
Ezr	0.878	http://api.brain-map.org/grid_data/download/2715?include=energy
Fbxl3	0.878	http://api.brain-map.org/grid_data/download/77465054?include=energy

Tnfrsf13c	0.878	http://api.brain-map.org/grid_data/download/72283719?include=energy
Obfc1	0.878	http://api.brain-map.org/grid_data/download/1938?include=energy
Hectd1	0.878	http://api.brain-map.org/grid_data/download/357092?include=energy
Arid1b	0.878	http://api.brain-map.org/grid_data/download/982?include=energy
Trmt12	0.878	http://api.brain-map.org/grid_data/download/75831760?include=energy
Hyal5	0.877	http://api.brain-map.org/grid_data/download/71378707?include=energy
1700040D17Rik	0.877	http://api.brain-map.org/grid_data/download/73617546?include=energy
Chpt1	0.877	http://api.brain-map.org/grid_data/download/68666565?include=energy
Fkbp5	0.877	http://api.brain-map.org/grid_data/download/70525820?include=energy
Rtn2	0.877	http://api.brain-map.org/grid_data/download/909?include=energy
LOC433353	0.877	http://api.brain-map.org/grid_data/download/70723181?include=energy
Qpct	0.877	http://api.brain-map.org/grid_data/download/583848?include=energy
Vsig1	0.877	http://api.brain-map.org/grid_data/download/70218109?include=energy
B3gnt7	0.877	http://api.brain-map.org/grid_data/download/68499473?include=energy
Rnf217	0.877	http://api.brain-map.org/grid_data/download/70595768?include=energy
Abhd16a	0.877	http://api.brain-map.org/grid_data/download/68631954?include=energy
Prr16	0.877	http://api.brain-map.org/grid_data/download/69443415?include=energy
Hsh2d	0.877	http://api.brain-map.org/grid_data/download/70277967?include=energy
Sumf1	0.877	http://api.brain-map.org/grid_data/download/68441562?include=energy
LOC434494	0.877	http://api.brain-map.org/grid_data/download/71022373?include=energy
Ctse	0.877	http://api.brain-map.org/grid_data/download/68443190?include=energy
Tiparp	0.877	http://api.brain-map.org/grid_data/download/69981007?include=energy
Mtmr10	0.877	http://api.brain-map.org/grid_data/download/68667473?include=energy
Twsg1	0.877	http://api.brain-map.org/grid_data/download/67979037?include=energy
Pde4c	0.877	http://api.brain-map.org/grid_data/download/69169779?include=energy
A430108G06Rik	0.877	http://api.brain-map.org/grid_data/download/71152228?include=energy
Zfyve19	0.877	http://api.brain-map.org/grid_data/download/68637772?include=energy
1700066B19Rik	0.877	http://api.brain-map.org/grid_data/download/73497717?include=energy
Mrpl17	0.877	http://api.brain-map.org/grid_data/download/70546191?include=energy
Wdr60	0.877	http://api.brain-map.org/grid_data/download/71147753?include=energy
2010002N04Rik	0.877	http://api.brain-map.org/grid_data/download/68301808?include=energy
Lepr	0.877	http://api.brain-map.org/grid_data/download/73930822?include=energy
F830001D05Rik*	0.877	http://api.brain-map.org/grid_data/download/73731385?include=energy
Plbd2	0.877	http://api.brain-map.org/grid_data/download/69262336?include=energy
Tex264	0.877	http://api.brain-map.org/grid_data/download/77620381?include=energy
A430089I19Rik	0.877	http://api.brain-map.org/grid_data/download/69174457?include=energy
4930505A04Rik	0.877	http://api.brain-map.org/grid_data/download/69114217?include=energy
Gm5519	0.877	http://api.brain-map.org/grid_data/download/74736219?include=energy
9130204L05Rik	0.877	http://api.brain-map.org/grid_data/download/72183002?include=energy
AB041803	0.877	http://api.brain-map.org/grid_data/download/71495197?include=energy
Mapkbp1	0.877	http://api.brain-map.org/grid_data/download/71381091?include=energy
Wdr33	0.877	http://api.brain-map.org/grid_data/download/77620231?include=energy
Noto	0.877	http://api.brain-map.org/grid_data/download/74578230?include=energy
LOC432609	0.877	http://api.brain-map.org/grid_data/download/70611108?include=energy
Kctd1	0.877	http://api.brain-map.org/grid_data/download/70744515?include=energy
Psme3	0.877	http://api.brain-map.org/grid_data/download/70429375?include=energy
Mcm3ap	0.877	http://api.brain-map.org/grid_data/download/665?include=energy
Tnfaip8	0.877	http://api.brain-map.org/grid_data/download/75038412?include=energy
TC1570131	0.877	http://api.brain-map.org/grid_data/download/70919955?include=energy
2900010M23Rik	0.877	http://api.brain-map.org/grid_data/download/68523348?include=energy
AW011738	0.877	http://api.brain-map.org/grid_data/download/70719010?include=energy
4930407I10Rik	0.877	http://api.brain-map.org/grid_data/download/70218115?include=energy
Nlrp9c	0.877	http://api.brain-map.org/grid_data/download/70784696?include=energy
Mpi	0.877	http://api.brain-map.org/grid_data/download/69079976?include=energy
Sft2d2	0.877	http://api.brain-map.org/grid_data/download/68410201?include=energy
A4galt	0.877	http://api.brain-map.org/grid_data/download/71717165?include=energy
Lrrc16a	0.877	http://api.brain-map.org/grid_data/download/75042256?include=energy
Tcf19	0.877	http://api.brain-map.org/grid_data/download/2659?include=energy
Asb9	0.877	http://api.brain-map.org/grid_data/download/69116895?include=energy
Atp2a1	0.877	http://api.brain-map.org/grid_data/download/532675?include=energy
Zscan2	0.877	http://api.brain-map.org/grid_data/download/68745191?include=energy
Gria1	0.877	http://api.brain-map.org/grid_data/download/79591719?include=energy
Apoo	0.877	http://api.brain-map.org/grid_data/download/71488353?include=energy
Ercc4	0.877	http://api.brain-map.org/grid_data/download/70528641?include=energy
Taf1	0.877	http://api.brain-map.org/grid_data/download/69527956?include=energy
Angptl7	0.877	http://api.brain-map.org/grid_data/download/81657695?include=energy
Vmn1r198	0.877	http://api.brain-map.org/grid_data/download/72472380?include=energy
BC031181	0.876	http://api.brain-map.org/grid_data/download/73718052?include=energy
Mrps12	0.876	http://api.brain-map.org/grid_data/download/68301365?include=energy
Ttc39b	0.876	http://api.brain-map.org/grid_data/download/70634139?include=energy
Zdhhc7	0.876	http://api.brain-map.org/grid_data/download/74881264?include=energy
Armc1	0.876	http://api.brain-map.org/grid_data/download/556985?include=energy
Psmb10	0.876	http://api.brain-map.org/grid_data/download/69080029?include=energy
Ddx49	0.876	http://api.brain-map.org/grid_data/download/70566145?include=energy
Rnf126	0.876	http://api.brain-map.org/grid_data/download/70473981?include=energy
Pfn1	0.876	http://api.brain-map.org/grid_data/download/73514809?include=energy
Lgals7	0.876	http://api.brain-map.org/grid_data/download/70918892?include=energy
Tbata	0.876	http://api.brain-map.org/grid_data/download/75197636?include=energy
LOC433175	0.876	http://api.brain-map.org/grid_data/download/71832937?include=energy
Ola1	0.876	http://api.brain-map.org/grid_data/download/293038?include=energy
Tek	0.876	http://api.brain-map.org/grid_data/download/68797942?include=energy
LOC434157	0.876	http://api.brain-map.org/grid_data/download/70787519?include=energy
Fam105a	0.876	http://api.brain-map.org/grid_data/download/74819326?include=energy
Dcdc5	0.876	http://api.brain-map.org/grid_data/download/71378677?include=energy
Dppa3	0.876	http://api.brain-map.org/grid_data/download/69671594?include=energy
3110002H16Rik	0.876	http://api.brain-map.org/grid_data/download/671231?include=energy
D130037M23Rik	0.876	http://api.brain-map.org/grid_data/download/70238603?include=energy
Pilra	0.876	http://api.brain-map.org/grid_data/download/75990410?include=energy
Rbfox2	0.876	http://api.brain-map.org/grid_data/download/71325402?include=energy
Hsdl2	0.876	http://api.brain-map.org/grid_data/download/74047906?include=energy
Nmur1	0.876	http://api.brain-map.org/grid_data/download/69257618?include=energy
Timm8b	0.876	http://api.brain-map.org/grid_data/download/100144401?include=energy
Mdm2	0.876	http://api.brain-map.org/grid_data/download/69752161?include=energy
Fam3a	0.876	http://api.brain-map.org/grid_data/download/69201636?include=energy
Nkiras1	0.876	http://api.brain-map.org/grid_data/download/74821972?include=energy
Ankrd6	0.876	http://api.brain-map.org/grid_data/download/71247671?include=energy
Ints10	0.876	http://api.brain-map.org/grid_data/download/81600554?include=energy
Myo1g	0.876	http://api.brain-map.org/grid_data/download/75931516?include=energy
Phactr1	0.876	http://api.brain-map.org/grid_data/download/71920511?include=energy
Psmc3	0.876	http://api.brain-map.org/grid_data/download/73512199?include=energy
Cyp2j5	0.876	http://api.brain-map.org/grid_data/download/513229?include=energy
Aqp11	0.876	http://api.brain-map.org/grid_data/download/69236831?include=energy
Bmx	0.876	http://api.brain-map.org/grid_data/download/69529388?include=energy
Vmn1r195	0.876	http://api.brain-map.org/grid_data/download/70785708?include=energy
Tcp10b	0.876	http://api.brain-map.org/grid_data/download/69116781?include=energy
Clock	0.876	http://api.brain-map.org/grid_data/download/273?include=energy
Drp2	0.876	http://api.brain-map.org/grid_data/download/69671606?include=energy
Vmn1r233	0.876	http://api.brain-map.org/grid_data/download/70785642?include=energy
Ccdc28b	0.876	http://api.brain-map.org/grid_data/download/68666529?include=energy
Ip6k1	0.876	http://api.brain-map.org/grid_data/download/69735311?include=energy
Col18a1	0.876	http://api.brain-map.org/grid_data/download/287?include=energy
Nfkbil1	0.876	http://api.brain-map.org/grid_data/download/74562576?include=energy
Galntl6	0.876	http://api.brain-map.org/grid_data/download/74750013?include=energy
Rpusd1	0.876	http://api.brain-map.org/grid_data/download/68637993?include=energy
Siglecg	0.876	http://api.brain-map.org/grid_data/download/75931590?include=energy
Tor3a	0.876	http://api.brain-map.org/grid_data/download/71489784?include=energy
Atg3	0.876	http://api.brain-map.org/grid_data/download/74988519?include=energy
A730030D03Rik*	0.876	http://api.brain-map.org/grid_data/download/73811537?include=energy
Kif1b	0.876	http://api.brain-map.org/grid_data/download/74003383?include=energy
Olfr552	0.876	http://api.brain-map.org/grid_data/download/74724163?include=energy
Numa1	0.876	http://api.brain-map.org/grid_data/download/74988615?include=energy
Gsk3a	0.876	http://api.brain-map.org/grid_data/download/70813329?include=energy
Mterf	0.876	http://api.brain-map.org/grid_data/download/70218504?include=energy
F830111B07Rik*	0.876	http://api.brain-map.org/grid_data/download/73748656?include=energy
Snx31	0.876	http://api.brain-map.org/grid_data/download/75081003?include=energy
Gpr141	0.876	http://api.brain-map.org/grid_data/download/71381568?include=energy
Vgll2	0.876	http://api.brain-map.org/grid_data/download/71488889?include=energy
Amotl2	0.876	http://api.brain-map.org/grid_data/download/68162203?include=energy
LOC436410	0.876	http://api.brain-map.org/grid_data/download/71283376?include=energy
Crx	0.876	http://api.brain-map.org/grid_data/download/77454639?include=energy
4732471D19Rik	0.876	http://api.brain-map.org/grid_data/download/69407690?include=energy
Cenpj	0.876	http://api.brain-map.org/grid_data/download/69548813?include=energy
Tpbg	0.876	http://api.brain-map.org/grid_data/download/76085743?include=energy
7030419G21Rik	0.876	http://api.brain-map.org/grid_data/download/70300487?include=energy
Drd4	0.876	http://api.brain-map.org/grid_data/download/112650336?include=energy
Aig1	0.875	http://api.brain-map.org/grid_data/download/68797499?include=energy
Bod1	0.875	http://api.brain-map.org/grid_data/download/69202406?include=energy
4930558G23Rik*	0.875	http://api.brain-map.org/grid_data/download/73927546?include=energy
Lrrc30	0.875	http://api.brain-map.org/grid_data/download/70296062?include=energy
Cd3d	0.875	http://api.brain-map.org/grid_data/download/69540593?include=energy
2610318N02Rik	0.875	http://api.brain-map.org/grid_data/download/68546266?include=energy
Col6a2	0.875	http://api.brain-map.org/grid_data/download/1177?include=energy
AI428936	0.875	http://api.brain-map.org/grid_data/download/68498842?include=energy
1700040F15Rik	0.875	http://api.brain-map.org/grid_data/download/72182943?include=energy
Mreg	0.875	http://api.brain-map.org/grid_data/download/73573331?include=energy
Psma1	0.875	http://api.brain-map.org/grid_data/download/413778?include=energy
Samd10	0.875	http://api.brain-map.org/grid_data/download/70565195?include=energy
Evx1	0.875	http://api.brain-map.org/grid_data/download/70303558?include=energy
Ccl3	0.875	http://api.brain-map.org/grid_data/download/71380965?include=energy
Aaas	0.875	http://api.brain-map.org/grid_data/download/68637443?include=energy
Sfrp1	0.875	http://api.brain-map.org/grid_data/download/2563?include=energy
Fam118a	0.875	http://api.brain-map.org/grid_data/download/71656068?include=energy
Gtf2h3	0.875	http://api.brain-map.org/grid_data/download/79360251?include=energy
Lrrc8c	0.875	http://api.brain-map.org/grid_data/download/71723924?include=energy
Exosc1	0.875	http://api.brain-map.org/grid_data/download/2203?include=energy
Pcdhb7	0.875	http://api.brain-map.org/grid_data/download/70723595?include=energy
Ehmt2	0.875	http://api.brain-map.org/grid_data/download/71283720?include=energy
Brwd1	0.875	http://api.brain-map.org/grid_data/download/1109?include=energy
LOC277506	0.875	http://api.brain-map.org/grid_data/download/71158282?include=energy
Col19a1	0.875	http://api.brain-map.org/grid_data/download/74658066?include=energy
Cryaa	0.875	http://api.brain-map.org/grid_data/download/2127?include=energy
Cnnm4	0.875	http://api.brain-map.org/grid_data/download/69568806?include=energy
Slc27a3	0.875	http://api.brain-map.org/grid_data/download/69873857?include=energy
Btbd1	0.875	http://api.brain-map.org/grid_data/download/71723869?include=energy
Myof	0.875	http://api.brain-map.org/grid_data/download/69672414?include=energy
Pak1ip1	0.875	http://api.brain-map.org/grid_data/download/69782688?include=energy
Lrr1	0.875	http://api.brain-map.org/grid_data/download/71991858?include=energy
Cd177	0.875	http://api.brain-map.org/grid_data/download/70474803?include=energy
Ceacam9	0.875	http://api.brain-map.org/grid_data/download/69540719?include=energy
Spz1	0.875	http://api.brain-map.org/grid_data/download/69114664?include=energy
4931432E15Rik	0.875	http://api.brain-map.org/grid_data/download/71892053?include=energy
Rrp36	0.875	http://api.brain-map.org/grid_data/download/68302035?include=energy
2010110P09Rik	0.875	http://api.brain-map.org/grid_data/download/69202232?include=energy
Tmed4	0.875	http://api.brain-map.org/grid_data/download/68148749?include=energy
Lcp2	0.875	http://api.brain-map.org/grid_data/download/70546063?include=energy
Gucy1b3	0.875	http://api.brain-map.org/grid_data/download/69734845?include=energy
Arl5b	0.875	http://api.brain-map.org/grid_data/download/74277354?include=energy
Ncoa7	0.875	http://api.brain-map.org/grid_data/download/77340496?include=energy
Dcaf6	0.875	http://api.brain-map.org/grid_data/download/71283450?include=energy
Fcgr1	0.875	http://api.brain-map.org/grid_data/download/74277652?include=energy
1110034A24Rik	0.875	http://api.brain-map.org/grid_data/download/69838257?include=energy
F730117H04Rik*	0.875	http://api.brain-map.org/grid_data/download/73767193?include=energy
Hdac9	0.875	http://api.brain-map.org/grid_data/download/73497168?include=energy
Mcpt4	0.875	http://api.brain-map.org/grid_data/download/75828181?include=energy
Grxcr1	0.875	http://api.brain-map.org/grid_data/download/70787083?include=energy
Dis3l2	0.875	http://api.brain-map.org/grid_data/download/176778?include=energy
Cdk5	0.875	http://api.brain-map.org/grid_data/download/244?include=energy
LOC433757	0.875	http://api.brain-map.org/grid_data/download/70786755?include=energy
Slc35d1	0.875	http://api.brain-map.org/grid_data/download/72001995?include=energy
Ssrp1	0.875	http://api.brain-map.org/grid_data/download/74819346?include=energy
Pla2g2e	0.875	http://api.brain-map.org/grid_data/download/68545465?include=energy
Gm1550	0.875	http://api.brain-map.org/grid_data/download/100144937?include=energy
Tmem179b	0.875	http://api.brain-map.org/grid_data/download/71920285?include=energy
Zbtb6	0.875	http://api.brain-map.org/grid_data/download/70785762?include=energy
Zfp386	0.875	http://api.brain-map.org/grid_data/download/70445347?include=energy
Dirc2	0.875	http://api.brain-map.org/grid_data/download/72339397?include=energy
Kdm6a	0.875	http://api.brain-map.org/grid_data/download/68798567?include=energy
Prdx6	0.875	http://api.brain-map.org/grid_data/download/686199?include=energy
Dnmt3b	0.875	http://api.brain-map.org/grid_data/download/71587577?include=energy
LOC433065	0.875	http://api.brain-map.org/grid_data/download/70611479?include=energy
Grin2c	0.875	http://api.brain-map.org/grid_data/download/75888748?include=energy
Ephb4	0.875	http://api.brain-map.org/grid_data/download/79544894?include=energy
Baz2b	0.874	http://api.brain-map.org/grid_data/download/68845615?include=energy
Hpcal1	0.874	http://api.brain-map.org/grid_data/download/74581395?include=energy
Spaca4	0.874	http://api.brain-map.org/grid_data/download/75851023?include=energy
Eif3m	0.874	http://api.brain-map.org/grid_data/download/68300974?include=energy
LOC434631	0.874	http://api.brain-map.org/grid_data/download/73512348?include=energy
B230354K17Rik	0.874	http://api.brain-map.org/grid_data/download/67787341?include=energy
Spryd4	0.874	http://api.brain-map.org/grid_data/download/67767442?include=energy
LOC434101	0.874	http://api.brain-map.org/grid_data/download/74430966?include=energy
Krtap16-7	0.874	http://api.brain-map.org/grid_data/download/70724923?include=energy
Ascl3	0.874	http://api.brain-map.org/grid_data/download/70723363?include=energy
Timp1	0.874	http://api.brain-map.org/grid_data/download/77790730?include=energy
Itga8	0.874	http://api.brain-map.org/grid_data/download/69735586?include=energy
Sh2b3	0.874	http://api.brain-map.org/grid_data/download/1633?include=energy
Gm5577	0.874	http://api.brain-map.org/grid_data/download/70787257?include=energy
9530003J23Rik	0.874	http://api.brain-map.org/grid_data/download/70228173?include=energy
5830419E12Rik*	0.874	http://api.brain-map.org/grid_data/download/73927595?include=energy
Ppil3	0.874	http://api.brain-map.org/grid_data/download/69015272?include=energy
4632428N05Rik	0.874	http://api.brain-map.org/grid_data/download/75695652?include=energy
A630046I20Rik*	0.874	http://api.brain-map.org/grid_data/download/73949773?include=energy
Fbxo24	0.874	http://api.brain-map.org/grid_data/download/71307304?include=energy
Dzip1l	0.874	http://api.brain-map.org/grid_data/download/69202944?include=energy
Med20	0.874	http://api.brain-map.org/grid_data/download/69817067?include=energy
5730446D14Rik	0.874	http://api.brain-map.org/grid_data/download/71493765?include=energy
Rnf215	0.874	http://api.brain-map.org/grid_data/download/69352736?include=energy
Grk6	0.874	http://api.brain-map.org/grid_data/download/69734566?include=energy
Cspg4	0.874	http://api.brain-map.org/grid_data/download/69608160?include=energy
Chpf	0.874	http://api.brain-map.org/grid_data/download/71580180?include=energy
LOC432415	0.874	http://api.brain-map.org/grid_data/download/74579479?include=energy
Tax1bp3	0.874	http://api.brain-map.org/grid_data/download/69980160?include=energy
Clint1	0.874	http://api.brain-map.org/grid_data/download/70230843?include=energy
Pex19	0.874	http://api.brain-map.org/grid_data/download/671224?include=energy
4930528F23Rik	0.874	http://api.brain-map.org/grid_data/download/71891898?include=energy
Sod3	0.874	http://api.brain-map.org/grid_data/download/74273308?include=energy
Scube2	0.874	http://api.brain-map.org/grid_data/download/71924235?include=energy
Gm1527	0.874	http://api.brain-map.org/grid_data/download/74562510?include=energy
Obox6	0.874	http://api.brain-map.org/grid_data/download/81657689?include=energy
Olfr466	0.874	http://api.brain-map.org/grid_data/download/74724121?include=energy
Dhrs11	0.874	http://api.brain-map.org/grid_data/download/68632948?include=energy
Nr6a1	0.874	http://api.brain-map.org/grid_data/download/70593329?include=energy
Timp4	0.874	http://api.brain-map.org/grid_data/download/70565327?include=energy
Tmem55a	0.874	http://api.brain-map.org/grid_data/download/71610880?include=energy
Arvcf	0.874	http://api.brain-map.org/grid_data/download/75214941?include=energy
Tiam2	0.874	http://api.brain-map.org/grid_data/download/75041513?include=energy
4930469G21Rik	0.874	http://api.brain-map.org/grid_data/download/74076228?include=energy
Med1	0.874	http://api.brain-map.org/grid_data/download/75931440?include=energy
Ammecr1l	0.874	http://api.brain-map.org/grid_data/download/68441538?include=energy
Amz1	0.874	http://api.brain-map.org/grid_data/download/69445379?include=energy
Fosl1	0.874	http://api.brain-map.org/grid_data/download/68667922?include=energy
Prr15l	0.874	http://api.brain-map.org/grid_data/download/74819231?include=energy
Chml	0.874	http://api.brain-map.org/grid_data/download/74047442?include=energy
9330159H11Rik	0.874	http://api.brain-map.org/grid_data/download/71210196?include=energy
Ankrd23	0.874	http://api.brain-map.org/grid_data/download/69169899?include=energy
Pgm3	0.874	http://api.brain-map.org/grid_data/download/69783053?include=energy
Susd4	0.874	http://api.brain-map.org/grid_data/download/75774678?include=energy
A530058O07Rik	0.874	http://api.brain-map.org/grid_data/download/71160271?include=energy
Omg	0.874	http://api.brain-map.org/grid_data/download/2450?include=energy
Zfp691	0.874	http://api.brain-map.org/grid_data/download/69288542?include=energy
4921508M14Rik	0.874	http://api.brain-map.org/grid_data/download/74076174?include=energy
LOC433110	0.874	http://api.brain-map.org/grid_data/download/71283364?include=energy
Eepd1	0.874	http://api.brain-map.org/grid_data/download/70805895?include=energy
Ostm1	0.874	http://api.brain-map.org/grid_data/download/69014961?include=energy
Atoh1	0.874	http://api.brain-map.org/grid_data/download/75826683?include=energy
Dnahc6	0.874	http://api.brain-map.org/grid_data/download/70228585?include=energy
A930033H14Rik	0.873	http://api.brain-map.org/grid_data/download/69514476?include=energy
Pik3c2g	0.873	http://api.brain-map.org/grid_data/download/73514815?include=energy

Eif2a	0.873	http://api.brain-map.org/grid_data/download/73497535?include=energy
Rhoj	0.873	http://api.brain-map.org/grid_data/download/69818176?include=energy
Dpm2	0.873	http://api.brain-map.org/grid_data/download/69079984?include=energy
2610008E11Rik	0.873	http://api.brain-map.org/grid_data/download/73617558?include=energy
1700029I01Rik	0.873	http://api.brain-map.org/grid_data/download/69134250?include=energy
Grik4	0.873	http://api.brain-map.org/grid_data/download/75551478?include=energy
Ptger4	0.873	http://api.brain-map.org/grid_data/download/112203056?include=energy
Dohh	0.873	http://api.brain-map.org/grid_data/download/413812?include=energy
Prosc	0.873	http://api.brain-map.org/grid_data/download/76115741?include=energy
Cpd	0.873	http://api.brain-map.org/grid_data/download/69568998?include=energy
LOC381872	0.873	http://api.brain-map.org/grid_data/download/70596125?include=energy
Tmem132a	0.873	http://api.brain-map.org/grid_data/download/70436749?include=energy
A930002H24Rik	0.873	http://api.brain-map.org/grid_data/download/70722934?include=energy
Klhl25	0.873	http://api.brain-map.org/grid_data/download/74359455?include=energy
D6Mm5e	0.873	http://api.brain-map.org/grid_data/download/71580210?include=energy
Etfb	0.873	http://api.brain-map.org/grid_data/download/69262325?include=energy
Lsm11	0.873	http://api.brain-map.org/grid_data/download/71488756?include=energy
l7Rn6	0.873	http://api.brain-map.org/grid_data/download/75198082?include=energy
Mavs	0.873	http://api.brain-map.org/grid_data/download/69261395?include=energy
Carf	0.873	http://api.brain-map.org/grid_data/download/76003365?include=energy
Cenpb	0.873	http://api.brain-map.org/grid_data/download/76081088?include=energy
Slc16a10	0.873	http://api.brain-map.org/grid_data/download/74000666?include=energy
9630013P03Rik*	0.873	http://api.brain-map.org/grid_data/download/77280434?include=energy
LOC434463	0.873	http://api.brain-map.org/grid_data/download/74635635?include=energy
Ranbp9	0.873	http://api.brain-map.org/grid_data/download/69817940?include=energy
Osbpl3	0.873	http://api.brain-map.org/grid_data/download/70231995?include=energy
4930562F07Rik	0.873	http://api.brain-map.org/grid_data/download/69442359?include=energy
Itgav	0.873	http://api.brain-map.org/grid_data/download/74658082?include=energy
A330021E22Rik	0.873	http://api.brain-map.org/grid_data/download/69448008?include=energy
Exoc3l2	0.873	http://api.brain-map.org/grid_data/download/69443178?include=energy
Grhl2	0.873	http://api.brain-map.org/grid_data/download/68798031?include=energy
Zfp637	0.873	http://api.brain-map.org/grid_data/download/71281528?include=energy
F430201E05Rik*	0.873	http://api.brain-map.org/grid_data/download/73767284?include=energy
Trak1	0.873	http://api.brain-map.org/grid_data/download/68924823?include=energy
Serpina1a	0.873	http://api.brain-map.org/grid_data/download/68301228?include=energy
Fbxw9	0.873	http://api.brain-map.org/grid_data/download/69080023?include=energy
Mrpl44	0.873	http://api.brain-map.org/grid_data/download/71020790?include=energy
2310021P13Rik	0.873	http://api.brain-map.org/grid_data/download/73834511?include=energy
1700020D05Rik	0.873	http://api.brain-map.org/grid_data/download/69353608?include=energy
Txndc12	0.873	http://api.brain-map.org/grid_data/download/68269289?include=energy
Zfp513	0.873	http://api.brain-map.org/grid_data/download/69549613?include=energy
Usp34	0.873	http://api.brain-map.org/grid_data/download/74821956?include=energy
Zbtb25	0.873	http://api.brain-map.org/grid_data/download/72471814?include=energy
Rbm41	0.873	http://api.brain-map.org/grid_data/download/69549671?include=energy
Pcgf1	0.873	http://api.brain-map.org/grid_data/download/68744701?include=energy
Nkx2-2	0.873	http://api.brain-map.org/grid_data/download/70593314?include=energy
Slc7a1	0.873	http://api.brain-map.org/grid_data/download/69874036?include=energy
Ecd	0.873	http://api.brain-map.org/grid_data/download/70430744?include=energy
4930422G04Rik	0.873	http://api.brain-map.org/grid_data/download/68445967?include=energy
Luzp1	0.873	http://api.brain-map.org/grid_data/download/69736328?include=energy
Dars2	0.873	http://api.brain-map.org/grid_data/download/69444711?include=energy
Ap2b1	0.873	http://api.brain-map.org/grid_data/download/112648612?include=energy
Hmha1	0.873	http://api.brain-map.org/grid_data/download/68797888?include=energy
LOC547320	0.873	http://api.brain-map.org/grid_data/download/77620259?include=energy
Apoa1	0.873	http://api.brain-map.org/grid_data/download/75826649?include=energy
Tgif1	0.873	http://api.brain-map.org/grid_data/download/67850949?include=energy
Slc38a5	0.873	http://api.brain-map.org/grid_data/download/69873941?include=energy
Hfm1	0.872	http://api.brain-map.org/grid_data/download/71808442?include=energy
BC052688	0.872	http://api.brain-map.org/grid_data/download/74736183?include=energy
Pld4	0.872	http://api.brain-map.org/grid_data/download/75831776?include=energy
Map3k7	0.872	http://api.brain-map.org/grid_data/download/2393?include=energy
Ppp1r1c	0.872	http://api.brain-map.org/grid_data/download/76081019?include=energy
Tmem60	0.872	http://api.brain-map.org/grid_data/download/68160848?include=energy
Dqx1	0.872	http://api.brain-map.org/grid_data/download/69134316?include=energy
4930458A03Rik	0.872	http://api.brain-map.org/grid_data/download/72182950?include=energy
LOC434871	0.872	http://api.brain-map.org/grid_data/download/74514351?include=energy
1700022F17Rik	0.872	http://api.brain-map.org/grid_data/download/71377997?include=energy
Agpat5	0.872	http://api.brain-map.org/grid_data/download/68637479?include=energy
Gm5089	0.872	http://api.brain-map.org/grid_data/download/74510296?include=energy
Wfdc13	0.872	http://api.brain-map.org/grid_data/download/74277321?include=energy
Slc25a29	0.872	http://api.brain-map.org/grid_data/download/78534484?include=energy
Rfx3	0.872	http://api.brain-map.org/grid_data/download/70634235?include=energy
Hspa1a	0.872	http://api.brain-map.org/grid_data/download/69816607?include=energy
Tmem87b	0.872	http://api.brain-map.org/grid_data/download/73635419?include=energy
Gpr135	0.872	http://api.brain-map.org/grid_data/download/74562236?include=energy
Blzf1	0.872	http://api.brain-map.org/grid_data/download/70430910?include=energy
Mtif3	0.872	http://api.brain-map.org/grid_data/download/70303815?include=energy
4930451E10Rik	0.872	http://api.brain-map.org/grid_data/download/74579993?include=energy
Mm.235026	0.872	http://api.brain-map.org/grid_data/download/71152599?include=energy
Ccnd1	0.872	http://api.brain-map.org/grid_data/download/69540507?include=energy
Gemin5	0.872	http://api.brain-map.org/grid_data/download/74003379?include=energy
BC028799	0.872	http://api.brain-map.org/grid_data/download/75988299?include=energy
A430092C21Rik*	0.872	http://api.brain-map.org/grid_data/download/73808582?include=energy
Mrpl39	0.872	http://api.brain-map.org/grid_data/download/673?include=energy
Ankrd39	0.872	http://api.brain-map.org/grid_data/download/69446595?include=energy
Tbl2	0.872	http://api.brain-map.org/grid_data/download/1868?include=energy
Arid3a	0.872	http://api.brain-map.org/grid_data/download/69288638?include=energy
LOC432938	0.872	http://api.brain-map.org/grid_data/download/70611413?include=energy
Slmo1	0.872	http://api.brain-map.org/grid_data/download/71627845?include=energy
2610303G11Rik	0.872	http://api.brain-map.org/grid_data/download/74635647?include=energy
Ccdc67	0.872	http://api.brain-map.org/grid_data/download/70717362?include=energy
Shisa5	0.872	http://api.brain-map.org/grid_data/download/81599650?include=energy
9530020I12Rik	0.872	http://api.brain-map.org/grid_data/download/70228185?include=energy
Adrbk1	0.872	http://api.brain-map.org/grid_data/download/71569304?include=energy
Cyp26a1	0.872	http://api.brain-map.org/grid_data/download/68301425?include=energy
LOC268939	0.872	http://api.brain-map.org/grid_data/download/70595775?include=energy
Slc7a5	0.872	http://api.brain-map.org/grid_data/download/68546482?include=energy
Mina	0.872	http://api.brain-map.org/grid_data/download/68410602?include=energy
Sfrp5	0.872	http://api.brain-map.org/grid_data/download/77454965?include=energy
Ephb6	0.872	http://api.brain-map.org/grid_data/download/2191?include=energy
Khk	0.872	http://api.brain-map.org/grid_data/download/68341483?include=energy
Vmn2r-ps88	0.872	http://api.brain-map.org/grid_data/download/70724971?include=energy
Fam47c	0.872	http://api.brain-map.org/grid_data/download/70205608?include=energy
Pdzk1	0.872	http://api.brain-map.org/grid_data/download/68321898?include=energy
Homer3	0.872	http://api.brain-map.org/grid_data/download/356087?include=energy
Gorab	0.872	http://api.brain-map.org/grid_data/download/69524578?include=energy
Parp3	0.872	http://api.brain-map.org/grid_data/download/75694429?include=energy
Vstm2b	0.872	http://api.brain-map.org/grid_data/download/72128753?include=energy
1700021F05Rik	0.872	http://api.brain-map.org/grid_data/download/68341489?include=energy
H6pd	0.872	http://api.brain-map.org/grid_data/download/68845353?include=energy
3010003L21Rik	0.872	http://api.brain-map.org/grid_data/download/71991947?include=energy
2700081O15Rik	0.872	http://api.brain-map.org/grid_data/download/69202999?include=energy
Il17a	0.872	http://api.brain-map.org/grid_data/download/76003675?include=energy
Mphosph9	0.872	http://api.brain-map.org/grid_data/download/70300403?include=energy
Eda	0.872	http://api.brain-map.org/grid_data/download/69671881?include=energy
Tex21	0.872	http://api.brain-map.org/grid_data/download/71280739?include=energy
Nt5c	0.872	http://api.brain-map.org/grid_data/download/68269279?include=energy
Serpina3n	0.872	http://api.brain-map.org/grid_data/download/75043155?include=energy
Ndufb4	0.872	http://api.brain-map.org/grid_data/download/68745006?include=energy
Ighg	0.872	http://api.brain-map.org/grid_data/download/77924364?include=energy
Farsb	0.872	http://api.brain-map.org/grid_data/download/69015643?include=energy
Vasp	0.871	http://api.brain-map.org/grid_data/download/69080503?include=energy
LOC270764	0.871	http://api.brain-map.org/grid_data/download/71924481?include=energy
Ddx56	0.871	http://api.brain-map.org/grid_data/download/68193020?include=energy
Ormdl2	0.871	http://api.brain-map.org/grid_data/download/71763837?include=energy
Gan	0.871	http://api.brain-map.org/grid_data/download/77464882?include=energy
Pigv	0.871	http://api.brain-map.org/grid_data/download/69838553?include=energy
Gstm4	0.871	http://api.brain-map.org/grid_data/download/70743872?include=energy
Cenpn	0.871	http://api.brain-map.org/grid_data/download/68798091?include=energy
Zscan4a	0.871	http://api.brain-map.org/grid_data/download/68667234?include=energy
Mvp	0.871	http://api.brain-map.org/grid_data/download/321202?include=energy
Treml2	0.871	http://api.brain-map.org/grid_data/download/71574467?include=energy
8030411F24Rik	0.871	http://api.brain-map.org/grid_data/download/74580853?include=energy
Sat2	0.871	http://api.brain-map.org/grid_data/download/69873415?include=energy
Ulk3	0.871	http://api.brain-map.org/grid_data/download/75233603?include=energy
Pnkp	0.871	http://api.brain-map.org/grid_data/download/69015093?include=energy
9430038I01Rik	0.871	http://api.brain-map.org/grid_data/download/74580901?include=energy
Slc10a7	0.871	http://api.brain-map.org/grid_data/download/100144877?include=energy
Dda1	0.871	http://api.brain-map.org/grid_data/download/70539149?include=energy
BC017643	0.871	http://api.brain-map.org/grid_data/download/76098402?include=energy
Gm1045	0.871	http://api.brain-map.org/grid_data/download/76081020?include=energy
Cradd	0.871	http://api.brain-map.org/grid_data/download/79539642?include=energy
Dnahc14	0.871	http://api.brain-map.org/grid_data/download/69447990?include=energy
Prl5a1	0.871	http://api.brain-map.org/grid_data/download/68667109?include=energy
Hoxd8	0.871	http://api.brain-map.org/grid_data/download/70813008?include=energy
Gm4879	0.871	http://api.brain-map.org/grid_data/download/74736069?include=energy
G730001M10Rik*	0.871	http://api.brain-map.org/grid_data/download/73716764?include=energy
Lrrc69	0.871	http://api.brain-map.org/grid_data/download/70201420?include=energy
Ddx28	0.871	http://api.brain-map.org/grid_data/download/69626735?include=energy
Polr2k	0.871	http://api.brain-map.org/grid_data/download/75198106?include=energy
BC066135	0.871	http://api.brain-map.org/grid_data/download/77620243?include=energy
Esrp1	0.871	http://api.brain-map.org/grid_data/download/73731235?include=energy
Zfp112	0.871	http://api.brain-map.org/grid_data/download/70303660?include=energy
Gm1574	0.871	http://api.brain-map.org/grid_data/download/68744152?include=energy
mCG140369.1	0.871	http://api.brain-map.org/grid_data/download/71211489?include=energy
Hspbap1	0.871	http://api.brain-map.org/grid_data/download/70277973?include=energy
Dcaf15	0.871	http://api.brain-map.org/grid_data/download/71656791?include=energy
Cisd3	0.871	http://api.brain-map.org/grid_data/download/74580972?include=energy
Zfp326	0.871	http://api.brain-map.org/grid_data/download/70474071?include=energy
Lgals12	0.871	http://api.brain-map.org/grid_data/download/75828172?include=energy
Avpr2	0.871	http://api.brain-map.org/grid_data/download/74509500?include=energy
U2af2	0.871	http://api.brain-map.org/grid_data/download/386012?include=energy
Ttc5	0.871	http://api.brain-map.org/grid_data/download/69838750?include=energy
Siglech	0.871	http://api.brain-map.org/grid_data/download/77332094?include=energy
Kcne2	0.871	http://api.brain-map.org/grid_data/download/1758?include=energy
Stx5a	0.871	http://api.brain-map.org/grid_data/download/67978462?include=energy
Ccbl1	0.871	http://api.brain-map.org/grid_data/download/68798717?include=energy
BC017612	0.871	http://api.brain-map.org/grid_data/download/71654134?include=energy
Vpreb3	0.871	http://api.brain-map.org/grid_data/download/68744140?include=energy
Sema3e	0.871	http://api.brain-map.org/grid_data/download/70436736?include=energy
Zfp422	0.871	http://api.brain-map.org/grid_data/download/386028?include=energy
Cetn4	0.871	http://api.brain-map.org/grid_data/download/69116841?include=energy
Rhbdf1	0.871	http://api.brain-map.org/grid_data/download/2533?include=energy
Slamf8	0.871	http://api.brain-map.org/grid_data/download/75861835?include=energy
1700037C18Rik	0.871	http://api.brain-map.org/grid_data/download/74635707?include=energy
Ift74	0.871	http://api.brain-map.org/grid_data/download/75862573?include=energy
4930597A21Rik	0.871	http://api.brain-map.org/grid_data/download/71891958?include=energy
Foxn1	0.871	http://api.brain-map.org/grid_data/download/71307328?include=energy
Plcz1	0.871	http://api.brain-map.org/grid_data/download/74988296?include=energy
Coq6	0.871	http://api.brain-map.org/grid_data/download/71021162?include=energy
Il1rapl2	0.871	http://api.brain-map.org/grid_data/download/73994672?include=energy
Tyw3	0.871	http://api.brain-map.org/grid_data/download/69443286?include=energy
Csn1s1	0.871	http://api.brain-map.org/grid_data/download/69079987?include=energy
Gpr30	0.871	http://api.brain-map.org/grid_data/download/80342021?include=energy
Cxcl12	0.871	http://api.brain-map.org/grid_data/download/308?include=energy
LOC328316	0.871	http://api.brain-map.org/grid_data/download/70595847?include=energy
Tspan17	0.871	http://api.brain-map.org/grid_data/download/74990526?include=energy
Klhl29	0.871	http://api.brain-map.org/grid_data/download/69735682?include=energy
Sf3a2	0.871	http://api.brain-map.org/grid_data/download/74882916?include=energy
Dbc1	0.871	http://api.brain-map.org/grid_data/download/72109467?include=energy
Zfp174	0.871	http://api.brain-map.org/grid_data/download/70725477?include=energy
Gtpbp10	0.87	http://api.brain-map.org/grid_data/download/74580032?include=energy
Tstd1	0.87	http://api.brain-map.org/grid_data/download/70566005?include=energy
B2m	0.87	http://api.brain-map.org/grid_data/download/71924291?include=energy
Mrps18c	0.87	http://api.brain-map.org/grid_data/download/68523318?include=energy
Usp36	0.87	http://api.brain-map.org/grid_data/download/70194648?include=energy
Ccnc	0.87	http://api.brain-map.org/grid_data/download/75988358?include=energy
Kcna6	0.87	http://api.brain-map.org/grid_data/download/74819554?include=energy
Tob2	0.87	http://api.brain-map.org/grid_data/download/77340500?include=energy
Tmem108	0.87	http://api.brain-map.org/grid_data/download/71358589?include=energy
Wdr77	0.87	http://api.brain-map.org/grid_data/download/413776?include=energy
Tmem37	0.87	http://api.brain-map.org/grid_data/download/68666750?include=energy
Cdc45	0.87	http://api.brain-map.org/grid_data/download/68522404?include=energy
Mucl1	0.87	http://api.brain-map.org/grid_data/download/68632032?include=energy
Gabra3	0.87	http://api.brain-map.org/grid_data/download/70562043?include=energy
Erlin2	0.87	http://api.brain-map.org/grid_data/download/68148694?include=energy
Prmt7	0.87	http://api.brain-map.org/grid_data/download/68203511?include=energy
Rbm44	0.87	http://api.brain-map.org/grid_data/download/71668563?include=energy
Etf1	0.87	http://api.brain-map.org/grid_data/download/70429244?include=energy
Ociad1	0.87	http://api.brain-map.org/grid_data/download/73512529?include=energy
Slc2a9	0.87	http://api.brain-map.org/grid_data/download/78534486?include=energy
3425401B19Rik	0.87	http://api.brain-map.org/grid_data/download/70611329?include=energy
Angpt1	0.87	http://api.brain-map.org/grid_data/download/69526629?include=energy
B230206H07Rik	0.87	http://api.brain-map.org/grid_data/download/71495867?include=energy
1700019G17Rik	0.87	http://api.brain-map.org/grid_data/download/76098404?include=energy
Ccnb2	0.87	http://api.brain-map.org/grid_data/download/633285?include=energy
Nrgn	0.87	http://api.brain-map.org/grid_data/download/69153759?include=energy
Zbtb1	0.87	http://api.brain-map.org/grid_data/download/74990493?include=energy
Abhd2	0.87	http://api.brain-map.org/grid_data/download/68148692?include=energy
H2-Ob	0.87	http://api.brain-map.org/grid_data/download/71764643?include=energy
Senp8	0.87	http://api.brain-map.org/grid_data/download/77620798?include=energy
D2hgdh	0.87	http://api.brain-map.org/grid_data/download/67792186?include=energy
Tab3	0.87	http://api.brain-map.org/grid_data/download/70610413?include=energy
Pml	0.87	http://api.brain-map.org/grid_data/download/68301264?include=energy
Sec24c	0.87	http://api.brain-map.org/grid_data/download/69015506?include=energy
6330409N04Rik	0.87	http://api.brain-map.org/grid_data/download/73512559?include=energy
Wfdc12	0.87	http://api.brain-map.org/grid_data/download/68498702?include=energy
Capn13	0.87	http://api.brain-map.org/grid_data/download/74578266?include=energy
9230108O11Rik*	0.87	http://api.brain-map.org/grid_data/download/73906191?include=energy
Cand2	0.87	http://api.brain-map.org/grid_data/download/69202262?include=energy
Pfkfb2	0.87	http://api.brain-map.org/grid_data/download/1399?include=energy
Trim56	0.87	http://api.brain-map.org/grid_data/download/68845645?include=energy
0610011F06Rik	0.87	http://api.brain-map.org/grid_data/download/74581002?include=energy
Celsr3	0.87	http://api.brain-map.org/grid_data/download/74724639?include=energy
Atp6v1b1	0.87	http://api.brain-map.org/grid_data/download/68342270?include=energy
Ppp6r2	0.87	http://api.brain-map.org/grid_data/download/67787331?include=energy
Senp3	0.87	http://api.brain-map.org/grid_data/download/81599636?include=energy
Ncald	0.87	http://api.brain-map.org/grid_data/download/71924321?include=energy
Oprd1	0.87	http://api.brain-map.org/grid_data/download/112646675?include=energy
Ppp1r14b	0.87	http://api.brain-map.org/grid_data/download/73512186?include=energy
Pomt1	0.87	http://api.brain-map.org/grid_data/download/69080732?include=energy
Serf1	0.87	http://api.brain-map.org/grid_data/download/75198166?include=energy
Pqlc2	0.87	http://api.brain-map.org/grid_data/download/68161456?include=energy
Zfp28	0.87	http://api.brain-map.org/grid_data/download/69262113?include=energy
Nr2f1	0.87	http://api.brain-map.org/grid_data/download/720?include=energy
D630039A03Rik	0.87	http://api.brain-map.org/grid_data/download/67881749?include=energy
1110017D15Rik	0.87	http://api.brain-map.org/grid_data/download/69174607?include=energy
4931428F04Rik	0.87	http://api.brain-map.org/grid_data/download/68845251?include=energy
1700011L22Rik	0.87	http://api.brain-map.org/grid_data/download/71920135?include=energy
Tmem199	0.87	http://api.brain-map.org/grid_data/download/69524497?include=energy
Adamtsl5	0.87	http://api.brain-map.org/grid_data/download/71063847?include=energy
Darc	0.87	http://api.brain-map.org/grid_data/download/74581383?include=energy
Cse1l	0.87	http://api.brain-map.org/grid_data/download/71378309?include=energy
Mon1b	0.87	http://api.brain-map.org/grid_data/download/74958566?include=energy
Rps5	0.87	http://api.brain-map.org/grid_data/download/77868800?include=energy
Dock1	0.87	http://api.brain-map.org/grid_data/download/100144855?include=energy
1700040I03Rik	0.87	http://api.brain-map.org/grid_data/download/69353687?include=energy
Gm1715	0.87	http://api.brain-map.org/grid_data/download/77924420?include=energy
Usp50	0.87	http://api.brain-map.org/grid_data/download/69116811?include=energy
Gm234	0.869	http://api.brain-map.org/grid_data/download/70295721?include=energy
Atf3	0.869	http://api.brain-map.org/grid_data/download/70814344?include=energy
C330027C09Rik	0.869	http://api.brain-map.org/grid_data/download/69531001?include=energy
Fech	0.869	http://api.brain-map.org/grid_data/download/75990389?include=energy
Card6	0.869	http://api.brain-map.org/grid_data/download/71021118?include=energy
Hook1	0.869	http://api.brain-map.org/grid_data/download/68745108?include=energy
Maea	0.869	http://api.brain-map.org/grid_data/download/70919525?include=energy
2310003L06Rik	0.869	http://api.brain-map.org/grid_data/download/70203471?include=energy

D030040J03Rik*	0.869	http://api.brain-map.org/grid_data/download/73811723?include=energy
Sugp1	0.869	http://api.brain-map.org/grid_data/download/532702?include=energy
4930578G10Rik	0.869	http://api.brain-map.org/grid_data/download/74265207?include=energy
Ddx11	0.869	http://api.brain-map.org/grid_data/download/69626723?include=energy
Cluap1	0.869	http://api.brain-map.org/grid_data/download/68443483?include=energy
Zfp799	0.869	http://api.brain-map.org/grid_data/download/71281515?include=energy
Tbc1d14	0.869	http://api.brain-map.org/grid_data/download/71020391?include=energy
Kng2	0.869	http://api.brain-map.org/grid_data/download/76081916?include=energy
Utp23	0.869	http://api.brain-map.org/grid_data/download/71924008?include=energy
Adap1	0.869	http://api.brain-map.org/grid_data/download/74658055?include=energy
Bcl2l11	0.869	http://api.brain-map.org/grid_data/download/74658243?include=energy
Galnt1	0.869	http://api.brain-map.org/grid_data/download/68666977?include=energy
Lrch1	0.869	http://api.brain-map.org/grid_data/download/71021149?include=energy
Prss35	0.869	http://api.brain-map.org/grid_data/download/74511839?include=energy
Nab1	0.869	http://api.brain-map.org/grid_data/download/70743878?include=energy
Dhrs4	0.869	http://api.brain-map.org/grid_data/download/71992043?include=energy
Morn4	0.869	http://api.brain-map.org/grid_data/download/68844062?include=energy
Trdmt1	0.869	http://api.brain-map.org/grid_data/download/69627033?include=energy
Ptrf	0.869	http://api.brain-map.org/grid_data/download/73521818?include=energy
St6galnac3	0.869	http://api.brain-map.org/grid_data/download/75077242?include=energy
Etnk2	0.869	http://api.brain-map.org/grid_data/download/69672174?include=energy
Gm9	0.869	http://api.brain-map.org/grid_data/download/71764494?include=energy
Csrnp3	0.869	http://api.brain-map.org/grid_data/download/68861933?include=energy
Celf3	0.869	http://api.brain-map.org/grid_data/download/74641324?include=energy
Pim3	0.869	http://api.brain-map.org/grid_data/download/641525?include=energy
Pak4	0.869	http://api.brain-map.org/grid_data/download/69549633?include=energy
Dusp15	0.869	http://api.brain-map.org/grid_data/download/70238949?include=energy
Cd226	0.869	http://api.brain-map.org/grid_data/download/68744068?include=energy
Mapk13	0.869	http://api.brain-map.org/grid_data/download/2397?include=energy
Coq10b	0.869	http://api.brain-map.org/grid_data/download/70200938?include=energy
Exosc10	0.869	http://api.brain-map.org/grid_data/download/68161479?include=energy
A530010L16Rik	0.869	http://api.brain-map.org/grid_data/download/72183004?include=energy
LOC238379	0.869	http://api.brain-map.org/grid_data/download/70566205?include=energy
Ttll11	0.869	http://api.brain-map.org/grid_data/download/71919026?include=energy
Apba3	0.869	http://api.brain-map.org/grid_data/download/68442913?include=energy
LOC434456	0.869	http://api.brain-map.org/grid_data/download/71022355?include=energy
Lrrc20	0.869	http://api.brain-map.org/grid_data/download/68797500?include=energy
Macrod2	0.869	http://api.brain-map.org/grid_data/download/70528005?include=energy
Ttc26	0.869	http://api.brain-map.org/grid_data/download/71148160?include=energy
Plekha3	0.869	http://api.brain-map.org/grid_data/download/76081901?include=energy
Nat15	0.869	http://api.brain-map.org/grid_data/download/67979034?include=energy
Tanc2	0.869	http://api.brain-map.org/grid_data/download/73949403?include=energy
Ptges3l	0.869	http://api.brain-map.org/grid_data/download/71492898?include=energy
Zfp383	0.869	http://api.brain-map.org/grid_data/download/69352858?include=energy
Cnpy4	0.869	http://api.brain-map.org/grid_data/download/69202824?include=energy
Uba1	0.869	http://api.brain-map.org/grid_data/download/70194486?include=energy
Stk35	0.869	http://api.brain-map.org/grid_data/download/69887367?include=energy
Degs1	0.869	http://api.brain-map.org/grid_data/download/73512562?include=energy
Mipol1	0.869	http://api.brain-map.org/grid_data/download/70946547?include=energy
LOC433932	0.869	http://api.brain-map.org/grid_data/download/71669001?include=energy
Ccna1	0.869	http://api.brain-map.org/grid_data/download/72182960?include=energy
Adck5	0.869	http://api.brain-map.org/grid_data/download/75990662?include=energy
Mybphl	0.869	http://api.brain-map.org/grid_data/download/70598382?include=energy
Dlx2	0.869	http://api.brain-map.org/grid_data/download/1482?include=energy
Stard3nl	0.869	http://api.brain-map.org/grid_data/download/67978662?include=energy
Zfp521	0.869	http://api.brain-map.org/grid_data/download/71015801?include=energy
Abca15	0.869	http://api.brain-map.org/grid_data/download/78534522?include=energy
Acer1	0.869	http://api.brain-map.org/grid_data/download/71393298?include=energy
Usp10	0.868	http://api.brain-map.org/grid_data/download/100144168?include=energy
Rdh7	0.868	http://api.brain-map.org/grid_data/download/68638059?include=energy
Adck1	0.868	http://api.brain-map.org/grid_data/download/80514097?include=energy
0610012G03Rik	0.868	http://api.brain-map.org/grid_data/download/70429100?include=energy
Fam35a	0.868	http://api.brain-map.org/grid_data/download/70609809?include=energy
Atp11b	0.868	http://api.brain-map.org/grid_data/download/70744324?include=energy
Fitm1	0.868	http://api.brain-map.org/grid_data/download/72081615?include=energy
LOC433394	0.868	http://api.brain-map.org/grid_data/download/70723211?include=energy
Olfr1370	0.868	http://api.brain-map.org/grid_data/download/74720799?include=energy
C1qa	0.868	http://api.brain-map.org/grid_data/download/77371775?include=energy
Ptk7	0.868	http://api.brain-map.org/grid_data/download/74882765?include=energy
Slc22a5	0.868	http://api.brain-map.org/grid_data/download/69173376?include=energy
Adamts3	0.868	http://api.brain-map.org/grid_data/download/73834559?include=energy
Zfp652	0.868	http://api.brain-map.org/grid_data/download/71213298?include=energy
C1qtnf1	0.868	http://api.brain-map.org/grid_data/download/68924461?include=energy
Dock3	0.868	http://api.brain-map.org/grid_data/download/71587583?include=energy
LOC433114	0.868	http://api.brain-map.org/grid_data/download/68845783?include=energy
Ceacam11	0.868	http://api.brain-map.org/grid_data/download/75931616?include=energy
Lrrc40	0.868	http://api.brain-map.org/grid_data/download/632516?include=energy
Styk1	0.868	http://api.brain-map.org/grid_data/download/71250308?include=energy
A230059C12Rik*	0.868	http://api.brain-map.org/grid_data/download/73906292?include=energy
Msc	0.868	http://api.brain-map.org/grid_data/download/69289411?include=energy
Ccl6	0.868	http://api.brain-map.org/grid_data/download/71489804?include=energy
Herc6	0.868	http://api.brain-map.org/grid_data/download/74635138?include=energy
Cd302	0.868	http://api.brain-map.org/grid_data/download/68546428?include=energy
Hoxc10	0.868	http://api.brain-map.org/grid_data/download/70431373?include=energy
Ccdc106	0.868	http://api.brain-map.org/grid_data/download/583852?include=energy
Wdr76	0.868	http://api.brain-map.org/grid_data/download/70227842?include=energy
Ly6c1	0.868	http://api.brain-map.org/grid_data/download/100144900?include=energy
Ucn3	0.868	http://api.brain-map.org/grid_data/download/1091?include=energy
Ccdc55	0.868	http://api.brain-map.org/grid_data/download/69526432?include=energy
Mrps2	0.868	http://api.brain-map.org/grid_data/download/68798115?include=energy
Ilf2	0.868	http://api.brain-map.org/grid_data/download/67815541?include=energy
Sorbs2	0.868	http://api.brain-map.org/grid_data/download/67787333?include=energy
Kcnq5	0.868	http://api.brain-map.org/grid_data/download/72128745?include=energy
Vapa	0.868	http://api.brain-map.org/grid_data/download/75695663?include=energy
Samd7	0.868	http://api.brain-map.org/grid_data/download/100144927?include=energy
Lgals8	0.868	http://api.brain-map.org/grid_data/download/68546293?include=energy
Csrp3	0.868	http://api.brain-map.org/grid_data/download/77332671?include=energy
Slc15a5	0.868	http://api.brain-map.org/grid_data/download/71569139?include=energy
LOC435219	0.868	http://api.brain-map.org/grid_data/download/70725628?include=energy
Nsa2	0.868	http://api.brain-map.org/grid_data/download/69443487?include=energy
Med8	0.868	http://api.brain-map.org/grid_data/download/112196048?include=energy
Ptpre	0.868	http://api.brain-map.org/grid_data/download/69817733?include=energy
Tmprss2	0.868	http://api.brain-map.org/grid_data/download/100144482?include=energy
Timm17a	0.868	http://api.brain-map.org/grid_data/download/80471955?include=energy
Gm10482	0.868	http://api.brain-map.org/grid_data/download/74822599?include=energy
Utp20	0.868	http://api.brain-map.org/grid_data/download/70610231?include=energy
Spaca3	0.868	http://api.brain-map.org/grid_data/download/75934511?include=energy
Zdhhc1	0.868	http://api.brain-map.org/grid_data/download/71488895?include=energy
Rbp7	0.868	http://api.brain-map.org/grid_data/download/68545057?include=energy
1700025F22Rik	0.868	http://api.brain-map.org/grid_data/download/69116942?include=energy
Tceal1	0.868	http://api.brain-map.org/grid_data/download/68193054?include=energy
N6amt2	0.868	http://api.brain-map.org/grid_data/download/69837159?include=energy
Zfp41	0.868	http://api.brain-map.org/grid_data/download/70445353?include=energy
Ccdc148	0.868	http://api.brain-map.org/grid_data/download/69837984?include=energy
Htr4	0.868	http://api.brain-map.org/grid_data/download/72119658?include=energy
Hyou1	0.868	http://api.brain-map.org/grid_data/download/671242?include=energy
4930438A08Rik	0.868	http://api.brain-map.org/grid_data/download/73513257?include=energy
Psmd3	0.868	http://api.brain-map.org/grid_data/download/393258?include=energy
Sall2	0.867	http://api.brain-map.org/grid_data/download/69290435?include=energy
Gyk	0.867	http://api.brain-map.org/grid_data/download/68191516?include=energy
9030418K01Rik	0.867	http://api.brain-map.org/grid_data/download/73767139?include=energy
Arsk	0.867	http://api.brain-map.org/grid_data/download/68844283?include=energy
Tfap2d	0.867	http://api.brain-map.org/grid_data/download/69290633?include=energy
Kera	0.867	http://api.brain-map.org/grid_data/download/71232908?include=energy
Bloc1s3	0.867	http://api.brain-map.org/grid_data/download/74822609?include=energy
Casp2	0.867	http://api.brain-map.org/grid_data/download/74562594?include=energy
Dnajb4	0.867	http://api.brain-map.org/grid_data/download/638712?include=energy
Abi3	0.867	http://api.brain-map.org/grid_data/download/68744994?include=energy
Elfn1	0.867	http://api.brain-map.org/grid_data/download/71587930?include=energy
Pxmp4	0.867	http://api.brain-map.org/grid_data/download/68499225?include=energy
Nop14	0.867	http://api.brain-map.org/grid_data/download/70430674?include=energy
Pus7l	0.867	http://api.brain-map.org/grid_data/download/69407403?include=energy
Zwilch	0.867	http://api.brain-map.org/grid_data/download/69261408?include=energy
Clec7a	0.867	http://api.brain-map.org/grid_data/download/81654145?include=energy
Tnfsf8	0.867	http://api.brain-map.org/grid_data/download/69981213?include=energy
Cntnap5b	0.867	http://api.brain-map.org/grid_data/download/70305550?include=energy
Ptprz1	0.867	http://api.brain-map.org/grid_data/download/77371825?include=energy
Ftsj3	0.867	http://api.brain-map.org/grid_data/download/80514028?include=energy
G6b	0.867	http://api.brain-map.org/grid_data/download/73513774?include=energy
Tff1	0.867	http://api.brain-map.org/grid_data/download/1049?include=energy
A130088B03Rik	0.867	http://api.brain-map.org/grid_data/download/70611437?include=energy
Rab40c	0.867	http://api.brain-map.org/grid_data/download/68196918?include=energy
Atf6b	0.867	http://api.brain-map.org/grid_data/download/69059953?include=energy
BC005655	0.867	http://api.brain-map.org/grid_data/download/75826583?include=energy
Cutc	0.867	http://api.brain-map.org/grid_data/download/75695647?include=energy
4930427A07Rik	0.867	http://api.brain-map.org/grid_data/download/69407935?include=energy
LOC434368	0.867	http://api.brain-map.org/grid_data/download/74819253?include=energy
2900092E17Rik	0.867	http://api.brain-map.org/grid_data/download/68076546?include=energy
Nctc1	0.867	http://api.brain-map.org/grid_data/download/71809145?include=energy
Pik3c2a	0.867	http://api.brain-map.org/grid_data/download/69783178?include=energy
Kcnk9	0.867	http://api.brain-map.org/grid_data/download/100142561?include=energy
Gm128	0.867	http://api.brain-map.org/grid_data/download/70295888?include=energy
Zkscan1	0.867	http://api.brain-map.org/grid_data/download/70430772?include=energy
Fpr2	0.867	http://api.brain-map.org/grid_data/download/70724732?include=energy
Prrt3	0.867	http://api.brain-map.org/grid_data/download/72081547?include=energy
Ccr7	0.867	http://api.brain-map.org/grid_data/download/69237041?include=energy
Atp6v1a	0.867	http://api.brain-map.org/grid_data/download/74641315?include=energy
2610001J05Rik	0.867	http://api.brain-map.org/grid_data/download/69202782?include=energy
Pcdhb16	0.867	http://api.brain-map.org/grid_data/download/72340179?include=energy
Slc39a8	0.867	http://api.brain-map.org/grid_data/download/69873959?include=energy
0610010F05Rik	0.867	http://api.brain-map.org/grid_data/download/74957923?include=energy
Cited1	0.867	http://api.brain-map.org/grid_data/download/74641326?include=energy
Slc37a4	0.867	http://api.brain-map.org/grid_data/download/69873935?include=energy
Tnfrsf22	0.867	http://api.brain-map.org/grid_data/download/74635713?include=energy
Krt27	0.867	http://api.brain-map.org/grid_data/download/70724900?include=energy
Dym	0.867	http://api.brain-map.org/grid_data/download/564764?include=energy
Zfp653	0.867	http://api.brain-map.org/grid_data/download/68191246?include=energy
Chmp6	0.867	http://api.brain-map.org/grid_data/download/71147797?include=energy
Rbm3	0.867	http://api.brain-map.org/grid_data/download/100142528?include=energy
Prok2	0.867	http://api.brain-map.org/grid_data/download/70300929?include=energy
Pip5k1c	0.867	http://api.brain-map.org/grid_data/download/71836809?include=energy
S1pr3	0.867	http://api.brain-map.org/grid_data/download/100144871?include=energy
Ccl19	0.867	http://api.brain-map.org/grid_data/download/68744008?include=energy
Kif23	0.867	http://api.brain-map.org/grid_data/download/68924691?include=energy
LOC433138	0.867	http://api.brain-map.org/grid_data/download/72471719?include=energy
Egr1	0.867	http://api.brain-map.org/grid_data/download/71247672?include=energy
Soat2	0.867	http://api.brain-map.org/grid_data/download/74425515?include=energy
Azgp1	0.867	http://api.brain-map.org/grid_data/download/69028617?include=energy
LOC277385	0.867	http://api.brain-map.org/grid_data/download/74359688?include=energy
LOC545350	0.867	http://api.brain-map.org/grid_data/download/74736432?include=energy
Mcts1	0.867	http://api.brain-map.org/grid_data/download/70723589?include=energy
A730020E08Rik	0.867	http://api.brain-map.org/grid_data/download/70228591?include=energy
Polr1c	0.867	http://api.brain-map.org/grid_data/download/70918886?include=energy
Olfr223	0.867	http://api.brain-map.org/grid_data/download/74720922?include=energy
Fam18a	0.867	http://api.brain-map.org/grid_data/download/74736129?include=energy
Klhdc4	0.867	http://api.brain-map.org/grid_data/download/67978784?include=energy
Cenpe	0.867	http://api.brain-map.org/grid_data/download/69548807?include=energy
H2-T24	0.867	http://api.brain-map.org/grid_data/download/76003546?include=energy
Fam151a	0.866	http://api.brain-map.org/grid_data/download/69261405?include=energy
Fezf1	0.866	http://api.brain-map.org/grid_data/download/72283796?include=energy
Klk14	0.866	http://api.brain-map.org/grid_data/download/70295434?include=energy
Cds1	0.866	http://api.brain-map.org/grid_data/download/72103808?include=energy
Ahrr	0.866	http://api.brain-map.org/grid_data/download/75988244?include=energy
Dnaic2	0.866	http://api.brain-map.org/grid_data/download/70611114?include=energy
Gm4894	0.866	http://api.brain-map.org/grid_data/download/70305275?include=energy
Btbd16	0.866	http://api.brain-map.org/grid_data/download/72182953?include=energy
0610010O12Rik	0.866	http://api.brain-map.org/grid_data/download/75079788?include=energy
2310050C09Rik	0.866	http://api.brain-map.org/grid_data/download/70218715?include=energy
Zfp119a	0.866	http://api.brain-map.org/grid_data/download/69290788?include=energy
Dab1	0.866	http://api.brain-map.org/grid_data/download/71924090?include=energy
Slco1c1	0.866	http://api.brain-map.org/grid_data/download/69874090?include=energy
LOC433250	0.866	http://api.brain-map.org/grid_data/download/74736225?include=energy
Gemin4	0.866	http://api.brain-map.org/grid_data/download/74818624?include=energy
Rnf185	0.866	http://api.brain-map.org/grid_data/download/67979020?include=energy
Loxl4	0.866	http://api.brain-map.org/grid_data/download/71209898?include=energy
Nbea	0.866	http://api.brain-map.org/grid_data/download/100144407?include=energy
Mm.29402	0.866	http://api.brain-map.org/grid_data/download/71161453?include=energy
LOC434058	0.866	http://api.brain-map.org/grid_data/download/70787227?include=energy
Gigyf1	0.866	http://api.brain-map.org/grid_data/download/69782993?include=energy
Gm5108	0.866	http://api.brain-map.org/grid_data/download/72182955?include=energy
Sytl3	0.866	http://api.brain-map.org/grid_data/download/2653?include=energy
Gm20555	0.866	http://api.brain-map.org/grid_data/download/73834427?include=energy
Fam196a	0.866	http://api.brain-map.org/grid_data/download/75042243?include=energy
Nudt13	0.866	http://api.brain-map.org/grid_data/download/69013444?include=energy
Tatdn1	0.866	http://api.brain-map.org/grid_data/download/69133617?include=energy
Romo1	0.866	http://api.brain-map.org/grid_data/download/70303761?include=energy
Rrp7a	0.866	http://api.brain-map.org/grid_data/download/68269706?include=energy
Mmp17	0.866	http://api.brain-map.org/grid_data/download/68799059?include=energy
1700010M22Rik	0.866	http://api.brain-map.org/grid_data/download/70201223?include=energy
Gorasp1	0.866	http://api.brain-map.org/grid_data/download/81599662?include=energy
Zscan4c	0.866	http://api.brain-map.org/grid_data/download/77924436?include=energy
Selp	0.866	http://api.brain-map.org/grid_data/download/76081032?include=energy
4931408A02Rik	0.866	http://api.brain-map.org/grid_data/download/112645384?include=energy
BC089491	0.866	http://api.brain-map.org/grid_data/download/72182961?include=energy
Ccnh	0.866	http://api.brain-map.org/grid_data/download/68546209?include=energy
Olfr572	0.866	http://api.brain-map.org/grid_data/download/74724205?include=energy
Mapkapk2	0.866	http://api.brain-map.org/grid_data/download/68844038?include=energy
Pbx1	0.866	http://api.brain-map.org/grid_data/download/71763546?include=energy
Olfr225	0.866	http://api.brain-map.org/grid_data/download/74720928?include=energy
Crygs	0.866	http://api.brain-map.org/grid_data/download/68919952?include=energy
Fam26e	0.866	http://api.brain-map.org/grid_data/download/70928307?include=energy
Obp1a	0.866	http://api.brain-map.org/grid_data/download/69782545?include=energy
Foxn2	0.866	http://api.brain-map.org/grid_data/download/69816559?include=energy
Agpat2	0.866	http://api.brain-map.org/grid_data/download/76080970?include=energy
Pelo	0.866	http://api.brain-map.org/grid_data/download/789?include=energy
LOC381876	0.866	http://api.brain-map.org/grid_data/download/71489815?include=energy
Nhlh1	0.866	http://api.brain-map.org/grid_data/download/75077191?include=energy
2310057M21Rik	0.866	http://api.brain-map.org/grid_data/download/69838403?include=energy
Lig4	0.866	http://api.brain-map.org/grid_data/download/69736130?include=energy
Ccdc66	0.866	http://api.brain-map.org/grid_data/download/69671786?include=energy
1700008O03Rik	0.866	http://api.brain-map.org/grid_data/download/75934506?include=energy
Sdccag8	0.866	http://api.brain-map.org/grid_data/download/323593?include=energy
Pou5f2	0.866	http://api.brain-map.org/grid_data/download/100145348?include=energy
Psen2	0.866	http://api.brain-map.org/grid_data/download/70539161?include=energy
Naa15	0.866	http://api.brain-map.org/grid_data/download/68667055?include=energy
Myh9	0.866	http://api.brain-map.org/grid_data/download/69781983?include=energy
Zfp59	0.866	http://api.brain-map.org/grid_data/download/69407164?include=energy
Mlkl	0.866	http://api.brain-map.org/grid_data/download/68498848?include=energy
Chka	0.866	http://api.brain-map.org/grid_data/download/75863292?include=energy
LOC227109	0.866	http://api.brain-map.org/grid_data/download/70566011?include=energy
Prickle3	0.866	http://api.brain-map.org/grid_data/download/69262322?include=energy
Pcnx	0.866	http://api.brain-map.org/grid_data/download/73818801?include=energy
Dpysl3	0.866	http://api.brain-map.org/grid_data/download/654928?include=energy
Lcn8	0.866	http://api.brain-map.org/grid_data/download/69114492?include=energy
Cdk5rap2	0.866	http://api.brain-map.org/grid_data/download/70303546?include=energy
Emr1	0.866	http://api.brain-map.org/grid_data/download/69256622?include=energy
Zfp275	0.866	http://api.brain-map.org/grid_data/download/69169493?include=energy
2410003K15Rik	0.866	http://api.brain-map.org/grid_data/download/70542875?include=energy
Iqce	0.865	http://api.brain-map.org/grid_data/download/70474851?include=energy
3426406K10Rik*	0.865	http://api.brain-map.org/grid_data/download/73906455?include=energy
Olfr633	0.865	http://api.brain-map.org/grid_data/download/74734758?include=energy
D830029A09Rik	0.865	http://api.brain-map.org/grid_data/download/72472778?include=energy
1700018A04Rik	0.865	http://api.brain-map.org/grid_data/download/74265249?include=energy
Capn3	0.865	http://api.brain-map.org/grid_data/download/69540351?include=energy
Eif1ax	0.865	http://api.brain-map.org/grid_data/download/74988539?include=energy
LOC436351	0.865	http://api.brain-map.org/grid_data/download/70300493?include=energy
Diras2	0.865	http://api.brain-map.org/grid_data/download/100143519?include=energy

Lamb1	0.865	http://api.brain-map.org/grid_data/download/71670482?include=energy
Accsl	0.865	http://api.brain-map.org/grid_data/download/74578224?include=energy
Zbtb11	0.865	http://api.brain-map.org/grid_data/download/73808285?include=energy
Dctn5	0.865	http://api.brain-map.org/grid_data/download/68203483?include=energy
Rpl21	0.865	http://api.brain-map.org/grid_data/download/74882831?include=energy
Atp13a3	0.865	http://api.brain-map.org/grid_data/download/70295769?include=energy
Smarcc1	0.865	http://api.brain-map.org/grid_data/download/69874174?include=energy
Pcdhb2	0.865	http://api.brain-map.org/grid_data/download/74743271?include=energy
Ankzf1	0.865	http://api.brain-map.org/grid_data/download/69549649?include=energy
Gm819	0.865	http://api.brain-map.org/grid_data/download/75931514?include=energy
Hspb11	0.865	http://api.brain-map.org/grid_data/download/70609244?include=energy
2810403G07Rik	0.865	http://api.brain-map.org/grid_data/download/71161018?include=energy
Vmn1r237	0.865	http://api.brain-map.org/grid_data/download/75934925?include=energy
1700006J14Rik	0.865	http://api.brain-map.org/grid_data/download/71919248?include=energy
Cotl1	0.865	http://api.brain-map.org/grid_data/download/72472769?include=energy
Gja3	0.865	http://api.brain-map.org/grid_data/download/69257325?include=energy
Habp2	0.865	http://api.brain-map.org/grid_data/download/68445083?include=energy
Gpatch8	0.865	http://api.brain-map.org/grid_data/download/73834421?include=energy
Procr	0.865	http://api.brain-map.org/grid_data/download/68523366?include=energy
Mark2	0.865	http://api.brain-map.org/grid_data/download/74076084?include=energy
Cyb5d2	0.865	http://api.brain-map.org/grid_data/download/70228089?include=energy
Shh	0.865	http://api.brain-map.org/grid_data/download/1418?include=energy
Chic2	0.865	http://api.brain-map.org/grid_data/download/75197504?include=energy
Ankfy1	0.865	http://api.brain-map.org/grid_data/download/69288614?include=energy
Sprr1a	0.865	http://api.brain-map.org/grid_data/download/1534?include=energy
Alx1	0.865	http://api.brain-map.org/grid_data/download/68798555?include=energy
C3	0.865	http://api.brain-map.org/grid_data/download/70528425?include=energy
Abca13	0.865	http://api.brain-map.org/grid_data/download/73513371?include=energy
Ak5	0.865	http://api.brain-map.org/grid_data/download/71808699?include=energy
Lancl3	0.865	http://api.brain-map.org/grid_data/download/73925716?include=energy
Klk1b1	0.865	http://api.brain-map.org/grid_data/download/69169761?include=energy
Apc	0.865	http://api.brain-map.org/grid_data/download/74881517?include=energy
Tmed8	0.865	http://api.brain-map.org/grid_data/download/69981073?include=energy
Galnt11	0.865	http://api.brain-map.org/grid_data/download/68301506?include=energy
Kbtbd2	0.865	http://api.brain-map.org/grid_data/download/68196939?include=energy
Btrc	0.865	http://api.brain-map.org/grid_data/download/74047905?include=energy
Nrg4	0.865	http://api.brain-map.org/grid_data/download/68545965?include=energy
4833439C01Rik*	0.865	http://api.brain-map.org/grid_data/download/73870713?include=energy
Ppapdc3	0.865	http://api.brain-map.org/grid_data/download/75695630?include=energy
C1s	0.865	http://api.brain-map.org/grid_data/download/75826672?include=energy
Crlf3	0.865	http://api.brain-map.org/grid_data/download/112195365?include=energy
Mpp2	0.865	http://api.brain-map.org/grid_data/download/68798980?include=energy
0610007P14Rik	0.865	http://api.brain-map.org/grid_data/download/70927828?include=energy
Yes1	0.865	http://api.brain-map.org/grid_data/download/70717352?include=energy
Abhd15	0.865	http://api.brain-map.org/grid_data/download/100144878?include=energy
4932411N23Rik	0.865	http://api.brain-map.org/grid_data/download/71808796?include=energy
Ssbp1	0.865	http://api.brain-map.org/grid_data/download/68522423?include=energy
Olfr556	0.865	http://api.brain-map.org/grid_data/download/74724181?include=energy
Dhx34	0.865	http://api.brain-map.org/grid_data/download/71280862?include=energy
Zfp608	0.865	http://api.brain-map.org/grid_data/download/73584711?include=energy
Insc	0.865	http://api.brain-map.org/grid_data/download/70610376?include=energy
Sla	0.865	http://api.brain-map.org/grid_data/download/2569?include=energy
Npvf	0.865	http://api.brain-map.org/grid_data/download/74882808?include=energy
4930451I11Rik	0.865	http://api.brain-map.org/grid_data/download/75851059?include=energy
Fbxl12	0.865	http://api.brain-map.org/grid_data/download/386244?include=energy
Rbm26	0.865	http://api.brain-map.org/grid_data/download/70474839?include=energy
BC043934	0.865	http://api.brain-map.org/grid_data/download/70231743?include=energy
Tnpo3	0.865	http://api.brain-map.org/grid_data/download/68798187?include=energy
Sec63	0.865	http://api.brain-map.org/grid_data/download/67815549?include=energy
Cd86	0.865	http://api.brain-map.org/grid_data/download/68301802?include=energy
Nme8	0.865	http://api.brain-map.org/grid_data/download/69114555?include=energy
Slc30a8	0.865	http://api.brain-map.org/grid_data/download/70302142?include=energy
Bgn	0.865	http://api.brain-map.org/grid_data/download/68443178?include=energy
4930579G22Rik	0.865	http://api.brain-map.org/grid_data/download/71891952?include=energy
Mdp1	0.865	http://api.brain-map.org/grid_data/download/68924697?include=energy
Pou5f1	0.865	http://api.brain-map.org/grid_data/download/71325348?include=energy
Uhrf1bp1	0.865	http://api.brain-map.org/grid_data/download/69672246?include=energy
Pafah1b3	0.865	http://api.brain-map.org/grid_data/download/69782676?include=energy
Zfp628	0.865	http://api.brain-map.org/grid_data/download/74822608?include=energy
LOC434360	0.865	http://api.brain-map.org/grid_data/download/71211320?include=energy
Serpina1b	0.865	http://api.brain-map.org/grid_data/download/72182995?include=energy
Znfx1	0.865	http://api.brain-map.org/grid_data/download/69524632?include=energy
Cxxc1	0.864	http://api.brain-map.org/grid_data/download/68498645?include=energy
Chrdl2	0.864	http://api.brain-map.org/grid_data/download/641539?include=energy
Tm4sf5	0.864	http://api.brain-map.org/grid_data/download/393244?include=energy
Ndufb3	0.864	http://api.brain-map.org/grid_data/download/70473927?include=energy
LOC433682	0.864	http://api.brain-map.org/grid_data/download/74513965?include=energy
Top3b	0.864	http://api.brain-map.org/grid_data/download/67815547?include=energy
Stard5	0.864	http://api.brain-map.org/grid_data/download/70813909?include=energy
Btbd9	0.864	http://api.brain-map.org/grid_data/download/69015194?include=energy
Timm44	0.864	http://api.brain-map.org/grid_data/download/71489781?include=energy
Pcdha2	0.864	http://api.brain-map.org/grid_data/download/70784745?include=energy
Lancl1	0.864	http://api.brain-map.org/grid_data/download/74881159?include=energy
1700123M08Rik	0.864	http://api.brain-map.org/grid_data/download/71232606?include=energy
Mtch2	0.864	http://api.brain-map.org/grid_data/download/72080084?include=energy
Awat1	0.864	http://api.brain-map.org/grid_data/download/71489814?include=energy
Cnn1	0.864	http://api.brain-map.org/grid_data/download/75988363?include=energy
Thumpd1	0.864	http://api.brain-map.org/grid_data/download/67978696?include=energy
Rhov	0.864	http://api.brain-map.org/grid_data/download/68445095?include=energy
Rpl12	0.864	http://api.brain-map.org/grid_data/download/70813317?include=energy
Olfm1	0.864	http://api.brain-map.org/grid_data/download/760?include=energy
Klk1b8	0.864	http://api.brain-map.org/grid_data/download/68632118?include=energy
Ephx4	0.864	http://api.brain-map.org/grid_data/download/69672922?include=energy
Tcf3	0.864	http://api.brain-map.org/grid_data/download/67850951?include=energy
Mtif2	0.864	http://api.brain-map.org/grid_data/download/677?include=energy
Hrc	0.864	http://api.brain-map.org/grid_data/download/75694706?include=energy
Usp47	0.864	http://api.brain-map.org/grid_data/download/70302950?include=energy
C530025M09Rik	0.864	http://api.brain-map.org/grid_data/download/69531037?include=energy
Sgce	0.864	http://api.brain-map.org/grid_data/download/68301168?include=energy
Mfsd2b	0.864	http://api.brain-map.org/grid_data/download/74514025?include=energy
Cars	0.864	http://api.brain-map.org/grid_data/download/68844385?include=energy
Grin2b	0.864	http://api.brain-map.org/grid_data/download/74988710?include=energy
Celf6	0.864	http://api.brain-map.org/grid_data/download/71358616?include=energy
LOC385492	0.864	http://api.brain-map.org/grid_data/download/71456096?include=energy
Htr5b	0.864	http://api.brain-map.org/grid_data/download/71247644?include=energy
Acads	0.864	http://api.brain-map.org/grid_data/download/68342390?include=energy
Bpifb2	0.864	http://api.brain-map.org/grid_data/download/74581217?include=energy
Rcbtb2	0.864	http://api.brain-map.org/grid_data/download/67978710?include=energy
Eif4ebp3	0.864	http://api.brain-map.org/grid_data/download/69116751?include=energy
9630011N22Rik	0.864	http://api.brain-map.org/grid_data/download/69447781?include=energy
Htatip2	0.864	http://api.brain-map.org/grid_data/download/512749?include=energy
Prr12	0.864	http://api.brain-map.org/grid_data/download/68080698?include=energy
Fam59b	0.864	http://api.brain-map.org/grid_data/download/69672952?include=energy
Hoxb13	0.864	http://api.brain-map.org/grid_data/download/2299?include=energy
Rab2b	0.864	http://api.brain-map.org/grid_data/download/69838513?include=energy
Dcst1	0.864	http://api.brain-map.org/grid_data/download/71764849?include=energy
9230104L09Rik	0.864	http://api.brain-map.org/grid_data/download/70228059?include=energy
Bbs1	0.864	http://api.brain-map.org/grid_data/download/70231230?include=energy
Grip1	0.864	http://api.brain-map.org/grid_data/download/74988749?include=energy
Neil1	0.864	http://api.brain-map.org/grid_data/download/68861692?include=energy
Cnnm3	0.864	http://api.brain-map.org/grid_data/download/76003515?include=energy
Anxa8	0.864	http://api.brain-map.org/grid_data/download/71616349?include=energy
Zfx	0.863	http://api.brain-map.org/grid_data/download/71280637?include=energy
Gpr77	0.863	http://api.brain-map.org/grid_data/download/74510176?include=energy
Kcmf1	0.863	http://api.brain-map.org/grid_data/download/68920767?include=energy
Ube2dnl1	0.863	http://api.brain-map.org/grid_data/download/75851056?include=energy
Npl	0.863	http://api.brain-map.org/grid_data/download/67815970?include=energy
Cldn7	0.863	http://api.brain-map.org/grid_data/download/71280709?include=energy
Ubtfl1	0.863	http://api.brain-map.org/grid_data/download/75043689?include=energy
Scn10a	0.863	http://api.brain-map.org/grid_data/download/69288285?include=energy
Spdya	0.863	http://api.brain-map.org/grid_data/download/69407833?include=energy
Gm12295	0.863	http://api.brain-map.org/grid_data/download/70305329?include=energy
Ankrd54	0.863	http://api.brain-map.org/grid_data/download/1677?include=energy
1700025B11Rik	0.863	http://api.brain-map.org/grid_data/download/71919625?include=energy
LOC433775	0.863	http://api.brain-map.org/grid_data/download/70786800?include=energy
Gdpd3	0.863	http://api.brain-map.org/grid_data/download/75694405?include=energy
Elovl7	0.863	http://api.brain-map.org/grid_data/download/71487106?include=energy
Adamts9	0.863	http://api.brain-map.org/grid_data/download/75214740?include=energy
Onecut2	0.863	http://api.brain-map.org/grid_data/download/69289645?include=energy
Nbeal2	0.863	http://api.brain-map.org/grid_data/download/71574534?include=energy
Nfia	0.863	http://api.brain-map.org/grid_data/download/1239?include=energy
Ms4a6d	0.863	http://api.brain-map.org/grid_data/download/68637802?include=energy
2700049A03Rik	0.863	http://api.brain-map.org/grid_data/download/69202986?include=energy
Kat8	0.863	http://api.brain-map.org/grid_data/download/2417?include=energy
Ube2m	0.863	http://api.brain-map.org/grid_data/download/69028246?include=energy
LOC433351	0.863	http://api.brain-map.org/grid_data/download/71808570?include=energy
Nrd1	0.863	http://api.brain-map.org/grid_data/download/67765720?include=energy
Theg	0.863	http://api.brain-map.org/grid_data/download/75861001?include=energy
D2Ertd750e	0.863	http://api.brain-map.org/grid_data/download/68498289?include=energy
A030011F13Rik*	0.863	http://api.brain-map.org/grid_data/download/73808291?include=energy
Parp8	0.863	http://api.brain-map.org/grid_data/download/74819242?include=energy
Cdc5l	0.863	http://api.brain-map.org/grid_data/download/77904707?include=energy
Dclk1	0.863	http://api.brain-map.org/grid_data/download/69169654?include=energy
Elfn2	0.863	http://api.brain-map.org/grid_data/download/70305090?include=energy
Nkx2-3	0.863	http://api.brain-map.org/grid_data/download/81654157?include=energy
Umodl1	0.863	http://api.brain-map.org/grid_data/download/71580174?include=energy
Lcn2	0.863	http://api.brain-map.org/grid_data/download/77799009?include=energy
Ifnz	0.863	http://api.brain-map.org/grid_data/download/70565861?include=energy
Gpr113	0.863	http://api.brain-map.org/grid_data/download/69257403?include=energy
A830023I12Rik	0.863	http://api.brain-map.org/grid_data/download/70717978?include=energy
Lman1l	0.863	http://api.brain-map.org/grid_data/download/81659535?include=energy
Csnk1g2	0.863	http://api.brain-map.org/grid_data/download/67979028?include=energy
Bzw1	0.863	http://api.brain-map.org/grid_data/download/324215?include=energy
Myadml2	0.863	http://api.brain-map.org/grid_data/download/70598374?include=energy
Tmem89	0.863	http://api.brain-map.org/grid_data/download/71378056?include=energy
Rftn1	0.863	http://api.brain-map.org/grid_data/download/71920515?include=energy
Itgbl1	0.863	http://api.brain-map.org/grid_data/download/74988458?include=energy
Orm2	0.863	http://api.brain-map.org/grid_data/download/75831770?include=energy
Ssu72	0.863	http://api.brain-map.org/grid_data/download/70474827?include=energy
Ldlr	0.863	http://api.brain-map.org/grid_data/download/1766?include=energy
E4f1	0.863	http://api.brain-map.org/grid_data/download/1199?include=energy
Ehd3	0.863	http://api.brain-map.org/grid_data/download/71836903?include=energy
LOC434053	0.863	http://api.brain-map.org/grid_data/download/70787221?include=energy
Six4	0.863	http://api.brain-map.org/grid_data/download/70593347?include=energy
Phf2	0.863	http://api.brain-map.org/grid_data/download/68798543?include=energy
Pim1	0.863	http://api.brain-map.org/grid_data/download/73423968?include=energy
Spef2	0.863	http://api.brain-map.org/grid_data/download/69530923?include=energy
Ndst2	0.863	http://api.brain-map.org/grid_data/download/71234619?include=energy
Exoc7	0.863	http://api.brain-map.org/grid_data/download/67815544?include=energy
Zfp819	0.863	http://api.brain-map.org/grid_data/download/68667150?include=energy
Map4k5	0.863	http://api.brain-map.org/grid_data/download/71022738?include=energy
6330578E17Rik	0.863	http://api.brain-map.org/grid_data/download/70445526?include=energy
1810013D15Rik	0.863	http://api.brain-map.org/grid_data/download/74359323?include=energy
Gm4125	0.863	http://api.brain-map.org/grid_data/download/75991108?include=energy
Cldn25	0.863	http://api.brain-map.org/grid_data/download/71656904?include=energy
Gm13490	0.862	http://api.brain-map.org/grid_data/download/74581211?include=energy
Trafd1	0.862	http://api.brain-map.org/grid_data/download/68563037?include=energy
Dnajc28	0.862	http://api.brain-map.org/grid_data/download/583551?include=energy
4930502E18Rik	0.862	http://api.brain-map.org/grid_data/download/70717356?include=energy
Prss28	0.862	http://api.brain-map.org/grid_data/download/73514845?include=energy
Fxyd6	0.862	http://api.brain-map.org/grid_data/download/73592534?include=energy
Igsf21	0.862	http://api.brain-map.org/grid_data/download/74357578?include=energy
Tmem151b	0.862	http://api.brain-map.org/grid_data/download/70295614?include=energy
Hebp1	0.862	http://api.brain-map.org/grid_data/download/74988456?include=energy
Mkrn2	0.862	http://api.brain-map.org/grid_data/download/68161474?include=energy
Loxl1	0.862	http://api.brain-map.org/grid_data/download/70805934?include=energy
2310003H01Rik	0.862	http://api.brain-map.org/grid_data/download/72080185?include=energy
Ssr4	0.862	http://api.brain-map.org/grid_data/download/69885768?include=energy
Atrx	0.862	http://api.brain-map.org/grid_data/download/74881733?include=energy
Lce1d	0.862	http://api.brain-map.org/grid_data/download/75198220?include=energy
Ankhd1	0.862	http://api.brain-map.org/grid_data/download/74265352?include=energy
Npat	0.862	http://api.brain-map.org/grid_data/download/71234634?include=energy
Syt8	0.862	http://api.brain-map.org/grid_data/download/71991696?include=energy
Tmem220	0.862	http://api.brain-map.org/grid_data/download/69448984?include=energy
Epb4.1l5	0.862	http://api.brain-map.org/grid_data/download/68076883?include=energy
Fcgr3	0.862	http://api.brain-map.org/grid_data/download/68667599?include=energy
D2Wsu81e	0.862	http://api.brain-map.org/grid_data/download/70919657?include=energy
Sgpl1	0.862	http://api.brain-map.org/grid_data/download/71656096?include=energy
Fut2	0.862	http://api.brain-map.org/grid_data/download/69672635?include=energy
Hipk2	0.862	http://api.brain-map.org/grid_data/download/68196925?include=energy
Fchsd1	0.862	http://api.brain-map.org/grid_data/download/68919910?include=energy
Gpx5	0.862	http://api.brain-map.org/grid_data/download/76003657?include=energy
E030007A22Rik	0.862	http://api.brain-map.org/grid_data/download/74988748?include=energy
B330016D10Rik	0.862	http://api.brain-map.org/grid_data/download/75197540?include=energy
Rbks	0.862	http://api.brain-map.org/grid_data/download/68441484?include=energy
8430427H17Rik	0.862	http://api.brain-map.org/grid_data/download/69446292?include=energy
Acaca	0.862	http://api.brain-map.org/grid_data/download/70228693?include=energy
Stoml1	0.862	http://api.brain-map.org/grid_data/download/74881134?include=energy
G530102J04Rik*	0.862	http://api.brain-map.org/grid_data/download/73808153?include=energy
2210016L21Rik	0.862	http://api.brain-map.org/grid_data/download/69173319?include=energy
Casp3	0.862	http://api.brain-map.org/grid_data/download/70431217?include=energy
Gm1140	0.862	http://api.brain-map.org/grid_data/download/70300025?include=energy
Gfra3	0.862	http://api.brain-map.org/grid_data/download/77620435?include=energy
LOC436046	0.862	http://api.brain-map.org/grid_data/download/70784576?include=energy
Prph	0.862	http://api.brain-map.org/grid_data/download/70232000?include=energy
Cdk13	0.862	http://api.brain-map.org/grid_data/download/71021144?include=energy
Zfp418	0.862	http://api.brain-map.org/grid_data/download/67881753?include=energy
Itpr1	0.862	http://api.brain-map.org/grid_data/download/72119596?include=energy
I830018F20Rik*	0.862	http://api.brain-map.org/grid_data/download/73767241?include=energy
Ugt1a6a	0.862	http://api.brain-map.org/grid_data/download/70194564?include=energy
Mbd3l1	0.862	http://api.brain-map.org/grid_data/download/75860975?include=energy
1190007I07Rik	0.862	http://api.brain-map.org/grid_data/download/74581116?include=energy
Igtp	0.862	http://api.brain-map.org/grid_data/download/69735305?include=energy
Pole	0.862	http://api.brain-map.org/grid_data/download/75931431?include=energy
Foxq1	0.862	http://api.brain-map.org/grid_data/download/100144492?include=energy
Nmd3	0.862	http://api.brain-map.org/grid_data/download/81599628?include=energy
Dicer1	0.862	http://api.brain-map.org/grid_data/download/74988272?include=energy
Clec1a	0.862	http://api.brain-map.org/grid_data/download/75990658?include=energy
Pacsin2	0.862	http://api.brain-map.org/grid_data/download/74047920?include=energy
Klhl38	0.862	http://api.brain-map.org/grid_data/download/69446256?include=energy
Tpp1	0.862	http://api.brain-map.org/grid_data/download/68148756?include=energy
Kdm5d	0.862	http://api.brain-map.org/grid_data/download/70303606?include=energy
Myo9a	0.862	http://api.brain-map.org/grid_data/download/69407672?include=energy
Kcng3	0.862	http://api.brain-map.org/grid_data/download/71717451?include=energy
Tstd2	0.862	http://api.brain-map.org/grid_data/download/69028701?include=energy
Ccdc110	0.862	http://api.brain-map.org/grid_data/download/70295655?include=energy
Noc2l	0.862	http://api.brain-map.org/grid_data/download/583872?include=energy
Wdr16	0.862	http://api.brain-map.org/grid_data/download/71377947?include=energy
Fam188b	0.862	http://api.brain-map.org/grid_data/download/75990481?include=energy
F930021L06Rik*	0.862	http://api.brain-map.org/grid_data/download/73767317?include=energy
Mcfd2	0.862	http://api.brain-map.org/grid_data/download/513474?include=energy
6720456H20Rik	0.862	http://api.brain-map.org/grid_data/download/69837850?include=energy
Eif2b4	0.862	http://api.brain-map.org/grid_data/download/68637730?include=energy
Inha	0.862	http://api.brain-map.org/grid_data/download/69028653?include=energy
Dcps	0.861	http://api.brain-map.org/grid_data/download/583560?include=energy
Sost	0.861	http://api.brain-map.org/grid_data/download/70302234?include=energy
Trmu	0.861	http://api.brain-map.org/grid_data/download/72471708?include=energy
Mipep	0.861	http://api.brain-map.org/grid_data/download/70946546?include=energy
Usp4	0.861	http://api.brain-map.org/grid_data/download/564646?include=energy
Plin2	0.861	http://api.brain-map.org/grid_data/download/75988239?include=energy
Mrgprx1	0.861	http://api.brain-map.org/grid_data/download/70784660?include=energy
2410002O22Rik	0.861	http://api.brain-map.org/grid_data/download/73423675?include=energy
2310030G06Rik	0.861	http://api.brain-map.org/grid_data/download/68545207?include=energy
Ankrd27	0.861	http://api.brain-map.org/grid_data/download/75931429?include=energy
Slc36a1	0.861	http://api.brain-map.org/grid_data/download/75041491?include=energy
4921536K21Rik	0.861	http://api.brain-map.org/grid_data/download/69113584?include=energy
Gpr65	0.861	http://api.brain-map.org/grid_data/download/79541425?include=energy

Adamts18	0.861	http://api.brain-map.org/grid_data/download/75651128?include=energy
Chmp1a	0.861	http://api.brain-map.org/grid_data/download/76081951?include=energy
Exoc4	0.861	http://api.brain-map.org/grid_data/download/74658239?include=energy
Fam63b	0.861	http://api.brain-map.org/grid_data/download/73716752?include=energy
D430019H16Rik	0.861	http://api.brain-map.org/grid_data/download/70795509?include=energy
Igh-VS107	0.861	http://api.brain-map.org/grid_data/download/76003669?include=energy
LOC217573	0.861	http://api.brain-map.org/grid_data/download/74735373?include=energy
BC004004	0.861	http://api.brain-map.org/grid_data/download/70430842?include=energy
Commd10	0.861	http://api.brain-map.org/grid_data/download/68545824?include=energy
Fbn1	0.861	http://api.brain-map.org/grid_data/download/69672300?include=energy
4930579K19Rik	0.861	http://api.brain-map.org/grid_data/download/69442410?include=energy
Tceb3	0.861	http://api.brain-map.org/grid_data/download/75043141?include=energy
Dab2	0.861	http://api.brain-map.org/grid_data/download/68443015?include=energy
1300002K09Rik	0.861	http://api.brain-map.org/grid_data/download/74581140?include=energy
Fgf15	0.861	http://api.brain-map.org/grid_data/download/1715?include=energy
Rabgap1	0.861	http://api.brain-map.org/grid_data/download/70473969?include=energy
Dpf2	0.861	http://api.brain-map.org/grid_data/download/68301353?include=energy
Hesx1	0.861	http://api.brain-map.org/grid_data/download/81657691?include=energy
Gprc5c	0.861	http://api.brain-map.org/grid_data/download/79632236?include=energy
Aqp5	0.861	http://api.brain-map.org/grid_data/download/71280556?include=energy
Rps6ka6	0.861	http://api.brain-map.org/grid_data/download/68667246?include=energy
Tmem208	0.861	http://api.brain-map.org/grid_data/download/69015745?include=energy
Mettl3	0.861	http://api.brain-map.org/grid_data/download/564756?include=energy
D730006F09Rik*	0.861	http://api.brain-map.org/grid_data/download/73906430?include=energy
Trim16	0.861	http://api.brain-map.org/grid_data/download/73931416?include=energy
Fam179b	0.861	http://api.brain-map.org/grid_data/download/69262152?include=energy
Rock1	0.861	http://api.brain-map.org/grid_data/download/71570744?include=energy
LOC545890	0.861	http://api.brain-map.org/grid_data/download/74736450?include=energy
Sp2	0.861	http://api.brain-map.org/grid_data/download/70439165?include=energy
Wasl	0.861	http://api.brain-map.org/grid_data/download/69013615?include=energy
Kbtbd10	0.861	http://api.brain-map.org/grid_data/download/70278117?include=energy
7420422E11Rik*	0.861	http://api.brain-map.org/grid_data/download/73927688?include=energy
Bdp1	0.861	http://api.brain-map.org/grid_data/download/71808711?include=energy
Sike1	0.861	http://api.brain-map.org/grid_data/download/70445508?include=energy
Rps6ka1	0.861	http://api.brain-map.org/grid_data/download/69873307?include=energy
Lrrc8e	0.861	http://api.brain-map.org/grid_data/download/70201612?include=energy
Mx2	0.861	http://api.brain-map.org/grid_data/download/1778?include=energy
LOC381529	0.861	http://api.brain-map.org/grid_data/download/70596071?include=energy
5830472F04Rik	0.861	http://api.brain-map.org/grid_data/download/74265333?include=energy
Dcaf4	0.861	http://api.brain-map.org/grid_data/download/2717?include=energy
Stambp	0.861	http://api.brain-map.org/grid_data/download/413793?include=energy
Sdf2l1	0.861	http://api.brain-map.org/grid_data/download/71656749?include=energy
Catsper3	0.861	http://api.brain-map.org/grid_data/download/75931612?include=energy
2010107G23Rik	0.861	http://api.brain-map.org/grid_data/download/67787342?include=energy
Mmrn2	0.861	http://api.brain-map.org/grid_data/download/69134181?include=energy
Sigirr	0.86	http://api.brain-map.org/grid_data/download/79541442?include=energy
Gabbr2	0.86	http://api.brain-map.org/grid_data/download/71247614?include=energy
Dbp	0.86	http://api.brain-map.org/grid_data/download/1193?include=energy
Rpap2	0.86	http://api.brain-map.org/grid_data/download/583846?include=energy
Ogfod1	0.86	http://api.brain-map.org/grid_data/download/74047922?include=energy
Rufy1	0.86	http://api.brain-map.org/grid_data/download/69873337?include=energy
Dffb	0.86	http://api.brain-map.org/grid_data/download/68799101?include=energy
Mpp5	0.86	http://api.brain-map.org/grid_data/download/71020776?include=energy
9530077C05Rik	0.86	http://api.brain-map.org/grid_data/download/68545989?include=energy
Ccdc15	0.86	http://api.brain-map.org/grid_data/download/76080956?include=energy
AU020206	0.86	http://api.brain-map.org/grid_data/download/70813203?include=energy
Necab1	0.86	http://api.brain-map.org/grid_data/download/72472791?include=energy
Olfr665	0.86	http://api.brain-map.org/grid_data/download/73653482?include=energy
Pde12	0.86	http://api.brain-map.org/grid_data/download/69837964?include=energy
Synj2	0.86	http://api.brain-map.org/grid_data/download/71064267?include=energy
Hlcs	0.86	http://api.brain-map.org/grid_data/download/68667103?include=energy
Cpt2	0.86	http://api.brain-map.org/grid_data/download/69608034?include=energy
4930430O22Rik	0.86	http://api.brain-map.org/grid_data/download/74076198?include=energy
Rcc1	0.86	http://api.brain-map.org/grid_data/download/68410315?include=energy
Kif2c	0.86	http://api.brain-map.org/grid_data/download/386252?include=energy
Tarbp2	0.86	http://api.brain-map.org/grid_data/download/75695657?include=energy
Mtap7d3	0.86	http://api.brain-map.org/grid_data/download/72471720?include=energy
Ralgapa2	0.86	http://api.brain-map.org/grid_data/download/74277699?include=energy
Lzts1	0.86	http://api.brain-map.org/grid_data/download/73994716?include=energy
Keg1	0.86	http://api.brain-map.org/grid_data/download/75694890?include=energy
3200002M19Rik	0.86	http://api.brain-map.org/grid_data/download/76097706?include=energy
A530021J07Rik	0.86	http://api.brain-map.org/grid_data/download/71569175?include=energy
Slc39a5	0.86	http://api.brain-map.org/grid_data/download/1645?include=energy
Gpr126	0.86	http://api.brain-map.org/grid_data/download/73520982?include=energy
Ntm	0.86	http://api.brain-map.org/grid_data/download/77866868?include=energy
Naif1	0.86	http://api.brain-map.org/grid_data/download/68844797?include=energy
Ccr5	0.86	http://api.brain-map.org/grid_data/download/69237029?include=energy
Sart3	0.86	http://api.brain-map.org/grid_data/download/74819691?include=energy
Dcaf7	0.86	http://api.brain-map.org/grid_data/download/68080703?include=energy
Tcirg1	0.86	http://api.brain-map.org/grid_data/download/69980214?include=energy
9930024G20Rik*	0.86	http://api.brain-map.org/grid_data/download/73808498?include=energy
Myo10	0.86	http://api.brain-map.org/grid_data/download/74743255?include=energy
Nfkbiz	0.86	http://api.brain-map.org/grid_data/download/69028450?include=energy
Prl7a1	0.86	http://api.brain-map.org/grid_data/download/68667061?include=energy
BC003331	0.86	http://api.brain-map.org/grid_data/download/70445604?include=energy
Gm9866	0.86	http://api.brain-map.org/grid_data/download/73808325?include=energy
Slc17a4	0.86	http://api.brain-map.org/grid_data/download/78634031?include=energy
Safb2	0.86	http://api.brain-map.org/grid_data/download/69816822?include=energy
Lonrf1	0.86	http://api.brain-map.org/grid_data/download/70595720?include=energy
Dusp12	0.86	http://api.brain-map.org/grid_data/download/70946492?include=energy
Sftpa1	0.86	http://api.brain-map.org/grid_data/download/70302045?include=energy
P2rx2	0.86	http://api.brain-map.org/grid_data/download/75551473?include=energy
Bmp2k	0.86	http://api.brain-map.org/grid_data/download/73712912?include=energy
Olfr1230	0.86	http://api.brain-map.org/grid_data/download/74720645?include=energy
Lap3	0.86	http://api.brain-map.org/grid_data/download/583844?include=energy
Gm106	0.86	http://api.brain-map.org/grid_data/download/71021177?include=energy
Nol3	0.86	http://api.brain-map.org/grid_data/download/67767468?include=energy
Ccdc88b	0.86	http://api.brain-map.org/grid_data/download/68545947?include=energy
Fgfr4	0.86	http://api.brain-map.org/grid_data/download/1581?include=energy
Efcab9	0.86	http://api.brain-map.org/grid_data/download/70598396?include=energy
Eif2d	0.86	http://api.brain-map.org/grid_data/download/323588?include=energy
Zfp955a	0.86	http://api.brain-map.org/grid_data/download/71210971?include=energy
Zfp354c	0.86	http://api.brain-map.org/grid_data/download/69352688?include=energy
Snx22	0.86	http://api.brain-map.org/grid_data/download/70299989?include=energy
Tnfrsf19	0.86	http://api.brain-map.org/grid_data/download/74425524?include=energy
1700093K21Rik	0.86	http://api.brain-map.org/grid_data/download/69116416?include=energy
Chm	0.86	http://api.brain-map.org/grid_data/download/68845472?include=energy
C8a	0.86	http://api.brain-map.org/grid_data/download/68795463?include=energy
Raph1	0.86	http://api.brain-map.org/grid_data/download/69446893?include=energy
Layn	0.86	http://api.brain-map.org/grid_data/download/73520977?include=energy
Gm5938	0.86	http://api.brain-map.org/grid_data/download/74736462?include=energy
Irx4	0.86	http://api.brain-map.org/grid_data/download/69289231?include=energy
Map2k1	0.86	http://api.brain-map.org/grid_data/download/71381076?include=energy
Fars2	0.86	http://api.brain-map.org/grid_data/download/68076538?include=energy
Fubp3	0.86	http://api.brain-map.org/grid_data/download/74359724?include=energy
Gbp5	0.86	http://api.brain-map.org/grid_data/download/69015344?include=energy
Tnfrsf10b	0.86	http://api.brain-map.org/grid_data/download/70302551?include=energy
Dnahc8	0.86	http://api.brain-map.org/grid_data/download/69626945?include=energy
Noxa1	0.859	http://api.brain-map.org/grid_data/download/75831737?include=energy
0610007L01Rik	0.859	http://api.brain-map.org/grid_data/download/67787332?include=energy
Cttn	0.859	http://api.brain-map.org/grid_data/download/70616018?include=energy
Braf	0.859	http://api.brain-map.org/grid_data/download/74579398?include=energy
Katnal2	0.859	http://api.brain-map.org/grid_data/download/69407457?include=energy
Golga5	0.859	http://api.brain-map.org/grid_data/download/68632930?include=energy
Rab12	0.859	http://api.brain-map.org/grid_data/download/74988626?include=energy
Camk1	0.859	http://api.brain-map.org/grid_data/download/70445646?include=energy
Flot1	0.859	http://api.brain-map.org/grid_data/download/67752111?include=energy
BC048355	0.859	http://api.brain-map.org/grid_data/download/69262317?include=energy
Ppp1r3c	0.859	http://api.brain-map.org/grid_data/download/70546390?include=energy
LOC432691	0.859	http://api.brain-map.org/grid_data/download/70611222?include=energy
Arfgap1	0.859	http://api.brain-map.org/grid_data/download/68845281?include=energy
Il15ra	0.859	http://api.brain-map.org/grid_data/download/74277718?include=energy
Qsox2	0.859	http://api.brain-map.org/grid_data/download/71656073?include=energy
Ctgf	0.859	http://api.brain-map.org/grid_data/download/1183?include=energy
Ube2f	0.859	http://api.brain-map.org/grid_data/download/638723?include=energy
Mmd	0.859	http://api.brain-map.org/grid_data/download/583559?include=energy
Tinag	0.859	http://api.brain-map.org/grid_data/download/77910721?include=energy
Fam154b	0.859	http://api.brain-map.org/grid_data/download/70305646?include=energy
Smad5	0.859	http://api.brain-map.org/grid_data/download/75831749?include=energy
Dpp10	0.859	http://api.brain-map.org/grid_data/download/72104183?include=energy
1700057D05Rik*	0.859	http://api.brain-map.org/grid_data/download/74359538?include=energy
Tle3	0.859	http://api.brain-map.org/grid_data/download/2681?include=energy
Gpsm3	0.859	http://api.brain-map.org/grid_data/download/71587769?include=energy
Kif20a	0.859	http://api.brain-map.org/grid_data/download/69735771?include=energy
Alkbh7	0.859	http://api.brain-map.org/grid_data/download/68441580?include=energy
Nrk	0.859	http://api.brain-map.org/grid_data/download/75084477?include=energy
Shbg	0.859	http://api.brain-map.org/grid_data/download/71991911?include=energy
Acot13	0.859	http://api.brain-map.org/grid_data/download/75042252?include=energy
Mm.308721	0.859	http://api.brain-map.org/grid_data/download/71161501?include=energy
Serpinb6c	0.859	http://api.brain-map.org/grid_data/download/68798394?include=energy
Pccb	0.859	http://api.brain-map.org/grid_data/download/70539167?include=energy
1700016H13Rik	0.859	http://api.brain-map.org/grid_data/download/74581337?include=energy
3110052M02Rik	0.859	http://api.brain-map.org/grid_data/download/69407481?include=energy
Ccdc96	0.859	http://api.brain-map.org/grid_data/download/69407815?include=energy
Mfap3l	0.859	http://api.brain-map.org/grid_data/download/69752406?include=energy
Bend7	0.859	http://api.brain-map.org/grid_data/download/76003521?include=energy
Chrna10	0.859	http://api.brain-map.org/grid_data/download/100147507?include=energy
Cdk15	0.859	http://api.brain-map.org/grid_data/download/75990875?include=energy
C130074G19Rik	0.859	http://api.brain-map.org/grid_data/download/71496025?include=energy
Fip1l1	0.859	http://api.brain-map.org/grid_data/download/532635?include=energy
Myh3	0.859	http://api.brain-map.org/grid_data/download/70723510?include=energy
Ddx55	0.859	http://api.brain-map.org/grid_data/download/70946471?include=energy
Yeats2	0.859	http://api.brain-map.org/grid_data/download/74988249?include=energy
Tsr1	0.859	http://api.brain-map.org/grid_data/download/67764944?include=energy
P2rx3	0.859	http://api.brain-map.org/grid_data/download/75888751?include=energy
Mlycd	0.859	http://api.brain-map.org/grid_data/download/67978514?include=energy
Map1lc3a	0.859	http://api.brain-map.org/grid_data/download/69751973?include=energy
F830004M19Rik	0.859	http://api.brain-map.org/grid_data/download/70276783?include=energy
Phf23	0.859	http://api.brain-map.org/grid_data/download/68632996?include=energy
Sgca	0.859	http://api.brain-map.org/grid_data/download/69873666?include=energy
Pnliprp1	0.859	http://api.brain-map.org/grid_data/download/69816356?include=energy
Gm11818	0.859	http://api.brain-map.org/grid_data/download/75990478?include=energy
Rp1l1	0.859	http://api.brain-map.org/grid_data/download/69169977?include=energy
Ampd1	0.859	http://api.brain-map.org/grid_data/download/70717992?include=energy
Otud3	0.859	http://api.brain-map.org/grid_data/download/70203627?include=energy
Psmd13	0.859	http://api.brain-map.org/grid_data/download/74882718?include=energy
BC018242	0.859	http://api.brain-map.org/grid_data/download/71836832?include=energy
Fkrp	0.859	http://api.brain-map.org/grid_data/download/68798741?include=energy
Smg1	0.858	http://api.brain-map.org/grid_data/download/74735092?include=energy
Nudt4	0.858	http://api.brain-map.org/grid_data/download/71836882?include=energy
Hps6	0.858	http://api.brain-map.org/grid_data/download/67815958?include=energy
Rrbp1	0.858	http://api.brain-map.org/grid_data/download/70812681?include=energy
2310036O22Rik	0.858	http://api.brain-map.org/grid_data/download/67814203?include=energy
Wdr59	0.858	http://api.brain-map.org/grid_data/download/71764855?include=energy
Fam178a	0.858	http://api.brain-map.org/grid_data/download/68844713?include=energy
LOC432586	0.858	http://api.brain-map.org/grid_data/download/70611060?include=energy
Foxj2	0.858	http://api.brain-map.org/grid_data/download/79632206?include=energy
Nom1	0.858	http://api.brain-map.org/grid_data/download/70787026?include=energy
Slco1a5	0.858	http://api.brain-map.org/grid_data/download/67850945?include=energy
Cebpd	0.858	http://api.brain-map.org/grid_data/download/76115722?include=energy
Wnt5b	0.858	http://api.brain-map.org/grid_data/download/77454631?include=energy
Tmem5	0.858	http://api.brain-map.org/grid_data/download/70919412?include=energy
Mm.359971	0.858	http://api.brain-map.org/grid_data/download/71209961?include=energy
Tceb1	0.858	http://api.brain-map.org/grid_data/download/68744564?include=energy
Fras1	0.858	http://api.brain-map.org/grid_data/download/73994667?include=energy
Commd4	0.858	http://api.brain-map.org/grid_data/download/68924437?include=energy
Klhl28	0.858	http://api.brain-map.org/grid_data/download/68162261?include=energy
Dab2ip	0.858	http://api.brain-map.org/grid_data/download/71496405?include=energy
Ccbl2	0.858	http://api.brain-map.org/grid_data/download/69529083?include=energy
Asb15	0.858	http://api.brain-map.org/grid_data/download/75851053?include=energy
Tfap2c	0.858	http://api.brain-map.org/grid_data/download/74047843?include=energy
Nhlrc2	0.858	http://api.brain-map.org/grid_data/download/70300535?include=energy
Ostc	0.858	http://api.brain-map.org/grid_data/download/1649?include=energy
E430033K04Rik	0.858	http://api.brain-map.org/grid_data/download/69671821?include=energy
Dnase1l2	0.858	http://api.brain-map.org/grid_data/download/69626991?include=energy
Rnf32	0.858	http://api.brain-map.org/grid_data/download/75080209?include=energy
Cdc34	0.858	http://api.brain-map.org/grid_data/download/68637796?include=energy
Gm2176	0.858	http://api.brain-map.org/grid_data/download/70228430?include=energy
4930563E18Rik	0.858	http://api.brain-map.org/grid_data/download/74821777?include=energy
Ptgs1	0.858	http://api.brain-map.org/grid_data/download/74800973?include=energy
Sqrdl	0.858	http://api.brain-map.org/grid_data/download/74047907?include=energy
Mm.375223	0.858	http://api.brain-map.org/grid_data/download/71210148?include=energy
LOC432660	0.858	http://api.brain-map.org/grid_data/download/70611174?include=energy
Galnt14	0.858	http://api.brain-map.org/grid_data/download/72472804?include=energy
LOC433859	0.858	http://api.brain-map.org/grid_data/download/70787014?include=energy
Htr1d	0.858	http://api.brain-map.org/grid_data/download/71393418?include=energy
Mphosph10	0.858	http://api.brain-map.org/grid_data/download/69262337?include=energy
Osbp	0.858	http://api.brain-map.org/grid_data/download/69095962?include=energy
Faim	0.858	http://api.brain-map.org/grid_data/download/70723583?include=energy
Mrps24	0.858	http://api.brain-map.org/grid_data/download/70546209?include=energy
Sdk1	0.858	http://api.brain-map.org/grid_data/download/69873469?include=energy
Tmem190	0.858	http://api.brain-map.org/grid_data/download/71891976?include=energy
Eif2s2	0.858	http://api.brain-map.org/grid_data/download/512771?include=energy
2210013O21Rik	0.858	http://api.brain-map.org/grid_data/download/72283725?include=energy
Tpcn1	0.858	http://api.brain-map.org/grid_data/download/69262231?include=energy
D130007C19Rik	0.858	http://api.brain-map.org/grid_data/download/69608477?include=energy
9030624G23Rik	0.858	http://api.brain-map.org/grid_data/download/71892334?include=energy
mCG58700.2	0.858	http://api.brain-map.org/grid_data/download/71213701?include=energy
Nudt2	0.858	http://api.brain-map.org/grid_data/download/77414917?include=energy
0610025J13Rik	0.858	http://api.brain-map.org/grid_data/download/72081715?include=energy
Gdf7	0.858	http://api.brain-map.org/grid_data/download/81790674?include=energy
Sidt2	0.858	http://api.brain-map.org/grid_data/download/68161475?include=energy
4833403I15Rik	0.858	http://api.brain-map.org/grid_data/download/71833052?include=energy
Wnt2b	0.858	http://api.brain-map.org/grid_data/download/1589?include=energy
Il22ra2	0.858	http://api.brain-map.org/grid_data/download/74990478?include=energy
Phyhip	0.858	http://api.brain-map.org/grid_data/download/544328?include=energy
Cox6b2	0.858	http://api.brain-map.org/grid_data/download/69838649?include=energy
Cnksr1	0.858	http://api.brain-map.org/grid_data/download/69568788?include=energy
Ghdc	0.858	http://api.brain-map.org/grid_data/download/69608471?include=energy
Anxa13	0.857	http://api.brain-map.org/grid_data/download/68302386?include=energy
Mad2l2	0.857	http://api.brain-map.org/grid_data/download/564638?include=energy
C030046I01Rik	0.857	http://api.brain-map.org/grid_data/download/74881890?include=energy
Tymp	0.857	http://api.brain-map.org/grid_data/download/587439?include=energy
Nav1	0.857	http://api.brain-map.org/grid_data/download/77280430?include=energy
Prl7d1	0.857	http://api.brain-map.org/grid_data/download/69783316?include=energy
Hist1h1d	0.857	http://api.brain-map.org/grid_data/download/69735036?include=energy
Klhl24	0.857	http://api.brain-map.org/grid_data/download/1982?include=energy
Crtc3	0.857	http://api.brain-map.org/grid_data/download/76097724?include=energy
Cckar	0.857	http://api.brain-map.org/grid_data/download/203?include=energy
Sec22c	0.857	http://api.brain-map.org/grid_data/download/68924829?include=energy
Isy1	0.857	http://api.brain-map.org/grid_data/download/583874?include=energy
Klk10	0.857	http://api.brain-map.org/grid_data/download/70928269?include=energy
Rsl24d1	0.857	http://api.brain-map.org/grid_data/download/69134583?include=energy
Med14	0.857	http://api.brain-map.org/grid_data/download/74578377?include=energy
Tceanc	0.857	http://api.brain-map.org/grid_data/download/73712840?include=energy
Spata20	0.857	http://api.brain-map.org/grid_data/download/69113787?include=energy
Gm3716	0.857	http://api.brain-map.org/grid_data/download/70299941?include=energy
Naip5	0.857	http://api.brain-map.org/grid_data/download/69529298?include=energy
Dppa5a	0.857	http://api.brain-map.org/grid_data/download/69671600?include=energy
Zer1	0.857	http://api.brain-map.org/grid_data/download/72283569?include=energy
Trappc3	0.857	http://api.brain-map.org/grid_data/download/513234?include=energy
Smoc1	0.857	http://api.brain-map.org/grid_data/download/71717592?include=energy
Sp4	0.857	http://api.brain-map.org/grid_data/download/75985932?include=energy
C230073O14Rik	0.857	http://api.brain-map.org/grid_data/download/71152509?include=energy
D130067C23Rik	0.857	http://api.brain-map.org/grid_data/download/73870653?include=energy
Mcl1	0.857	http://api.brain-map.org/grid_data/download/112647104?include=energy
Polr3h	0.857	http://api.brain-map.org/grid_data/download/81599664?include=energy

BC005561	0.857	http://api.brain-map.org/grid_data/download/74988761?include=energy
Olfr96	0.857	http://api.brain-map.org/grid_data/download/74735003?include=energy
Il13	0.857	http://api.brain-map.org/grid_data/download/69735359?include=energy
Nat14	0.857	http://api.brain-map.org/grid_data/download/69012932?include=energy
Ptprd	0.857	http://api.brain-map.org/grid_data/download/855?include=energy
Ccr4	0.857	http://api.brain-map.org/grid_data/download/69237023?include=energy
Il12rb1	0.857	http://api.brain-map.org/grid_data/download/69028390?include=energy
1700021P04Rik	0.857	http://api.brain-map.org/grid_data/download/71919535?include=energy
4930509E16Rik	0.857	http://api.brain-map.org/grid_data/download/70218199?include=energy
1700030F18Rik	0.857	http://api.brain-map.org/grid_data/download/69114223?include=energy
Cdh24	0.857	http://api.brain-map.org/grid_data/download/70231307?include=energy
Tada1	0.857	http://api.brain-map.org/grid_data/download/413833?include=energy
Pfas	0.857	http://api.brain-map.org/grid_data/download/69783017?include=energy
Tnfrsf13b	0.857	http://api.brain-map.org/grid_data/download/71919164?include=energy
Tollip	0.857	http://api.brain-map.org/grid_data/download/68798244?include=energy
Wdr4	0.857	http://api.brain-map.org/grid_data/download/79632232?include=energy
Fam65a	0.857	http://api.brain-map.org/grid_data/download/75774668?include=energy
Farsa	0.857	http://api.brain-map.org/grid_data/download/71723930?include=energy
Iqub	0.857	http://api.brain-map.org/grid_data/download/70218528?include=energy
Gk2	0.857	http://api.brain-map.org/grid_data/download/76081900?include=energy
Prelp	0.857	http://api.brain-map.org/grid_data/download/68637832?include=energy
Phtf2	0.857	http://api.brain-map.org/grid_data/download/68667509?include=energy
Slc27a6	0.857	http://api.brain-map.org/grid_data/download/69873863?include=energy
Bhmt2	0.857	http://api.brain-map.org/grid_data/download/75694877?include=energy
Diablo	0.857	http://api.brain-map.org/grid_data/download/68667085?include=energy
Nrn1	0.857	http://api.brain-map.org/grid_data/download/75147760?include=energy
Gpr18	0.857	http://api.brain-map.org/grid_data/download/71015809?include=energy
Kcnip3	0.857	http://api.brain-map.org/grid_data/download/71587887?include=energy
1500011K16Rik	0.857	http://api.brain-map.org/grid_data/download/70598394?include=energy
2410089E03Rik	0.857	http://api.brain-map.org/grid_data/download/68919842?include=energy
Lig3	0.857	http://api.brain-map.org/grid_data/download/69262348?include=energy
E2f7	0.857	http://api.brain-map.org/grid_data/download/70239033?include=energy
Brd2	0.857	http://api.brain-map.org/grid_data/download/70305496?include=energy
Piwil4	0.857	http://api.brain-map.org/grid_data/download/71281692?include=energy
Cabyr	0.857	http://api.brain-map.org/grid_data/download/69540260?include=energy
Kdelr1	0.857	http://api.brain-map.org/grid_data/download/130912?include=energy
Spink5	0.857	http://api.brain-map.org/grid_data/download/75931558?include=energy
Sez6l	0.857	http://api.brain-map.org/grid_data/download/73929583?include=energy
3110001D03Rik	0.857	http://api.brain-map.org/grid_data/download/67815953?include=energy
Kcnmb2	0.857	http://api.brain-map.org/grid_data/download/74635689?include=energy
2300002M23Rik	0.857	http://api.brain-map.org/grid_data/download/70203447?include=energy
Foxb1	0.857	http://api.brain-map.org/grid_data/download/79908799?include=energy
Gal3st3	0.857	http://api.brain-map.org/grid_data/download/100145393?include=energy
Kcna3	0.857	http://api.brain-map.org/grid_data/download/70723477?include=energy
Max	0.857	http://api.brain-map.org/grid_data/download/69289333?include=energy
6720456B07Rik	0.856	http://api.brain-map.org/grid_data/download/74988744?include=energy
Cyb561d1	0.856	http://api.brain-map.org/grid_data/download/75988366?include=energy
Cenpm	0.856	http://api.brain-map.org/grid_data/download/68269562?include=energy
Coq9	0.856	http://api.brain-map.org/grid_data/download/68523300?include=energy
Bnip3l	0.856	http://api.brain-map.org/grid_data/download/75988303?include=energy
Ppm1f	0.856	http://api.brain-map.org/grid_data/download/68845382?include=energy
Tmem86b	0.856	http://api.brain-map.org/grid_data/download/72472763?include=energy
Pcdhga8	0.856	http://api.brain-map.org/grid_data/download/70784904?include=energy
Prkce	0.856	http://api.brain-map.org/grid_data/download/75991096?include=energy
1700034H15Rik	0.856	http://api.brain-map.org/grid_data/download/71920117?include=energy
Cog6	0.856	http://api.brain-map.org/grid_data/download/71610877?include=energy
Hpd	0.856	http://api.brain-map.org/grid_data/download/356492?include=energy
9430015G10Rik	0.856	http://api.brain-map.org/grid_data/download/71610789?include=energy
Ighmbp2	0.856	http://api.brain-map.org/grid_data/download/74514255?include=energy
Pkp1	0.856	http://api.brain-map.org/grid_data/download/76081010?include=energy
9330175E14Rik	0.856	http://api.brain-map.org/grid_data/download/70228101?include=energy
Zfp263	0.856	http://api.brain-map.org/grid_data/download/68667204?include=energy
Oaz3	0.856	http://api.brain-map.org/grid_data/download/69782539?include=energy
4930563D23Rik	0.856	http://api.brain-map.org/grid_data/download/70218390?include=energy
Phc2	0.856	http://api.brain-map.org/grid_data/download/68844492?include=energy
Btbd7	0.856	http://api.brain-map.org/grid_data/download/69549612?include=energy
5031438A03Rik	0.856	http://api.brain-map.org/grid_data/download/74510272?include=energy
B230218O03	0.856	http://api.brain-map.org/grid_data/download/71574485?include=energy
Snrnp200	0.856	http://api.brain-map.org/grid_data/download/68844510?include=energy
Eif2c3	0.856	http://api.brain-map.org/grid_data/download/70724708?include=energy
E030044B06Rik	0.856	http://api.brain-map.org/grid_data/download/72076515?include=energy
Plce1	0.856	http://api.brain-map.org/grid_data/download/70784982?include=energy
Kcp	0.856	http://api.brain-map.org/grid_data/download/71529863?include=energy
Fam84b	0.856	http://api.brain-map.org/grid_data/download/75081395?include=energy
Ralgps1	0.856	http://api.brain-map.org/grid_data/download/71920506?include=energy
Syt4	0.856	http://api.brain-map.org/grid_data/download/2651?include=energy
Pirt	0.856	http://api.brain-map.org/grid_data/download/72283798?include=energy
B230342D01Rik*	0.856	http://api.brain-map.org/grid_data/download/74000576?include=energy
4931440F15Rik	0.856	http://api.brain-map.org/grid_data/download/69442455?include=energy
Cpn1	0.856	http://api.brain-map.org/grid_data/download/68666631?include=energy
Serinc4	0.856	http://api.brain-map.org/grid_data/download/74277634?include=energy
Fam46b	0.856	http://api.brain-map.org/grid_data/download/68162238?include=energy
D4Ertd22e	0.856	http://api.brain-map.org/grid_data/download/75197510?include=energy
Ap3s2	0.856	http://api.brain-map.org/grid_data/download/71495789?include=energy
Rab23	0.856	http://api.brain-map.org/grid_data/download/68161464?include=energy
Tmem39b	0.856	http://api.brain-map.org/grid_data/download/68797810?include=energy
Osr2	0.856	http://api.brain-map.org/grid_data/download/72182996?include=energy
3300002A11Rik	0.856	http://api.brain-map.org/grid_data/download/73716620?include=energy
Rarg	0.856	http://api.brain-map.org/grid_data/download/130913?include=energy
Szt2	0.856	http://api.brain-map.org/grid_data/download/1446?include=energy
D130012J16Rik*	0.856	http://api.brain-map.org/grid_data/download/73811735?include=energy
Atg7	0.856	http://api.brain-map.org/grid_data/download/68745048?include=energy
Dok3	0.856	http://api.brain-map.org/grid_data/download/76003519?include=energy
Angptl1	0.856	http://api.brain-map.org/grid_data/download/70230933?include=energy
BC016608	0.856	http://api.brain-map.org/grid_data/download/68443489?include=energy
4632419I22Rik	0.856	http://api.brain-map.org/grid_data/download/69407642?include=energy
Tnfaip3	0.856	http://api.brain-map.org/grid_data/download/75084101?include=energy
Rabggta	0.856	http://api.brain-map.org/grid_data/download/70429381?include=energy
Clybl	0.856	http://api.brain-map.org/grid_data/download/68441508?include=energy
2410016O06Rik	0.856	http://api.brain-map.org/grid_data/download/69202639?include=energy
Soat1	0.856	http://api.brain-map.org/grid_data/download/69885642?include=energy
Endou	0.856	http://api.brain-map.org/grid_data/download/69817175?include=energy
P2ry6	0.856	http://api.brain-map.org/grid_data/download/1800?include=energy
Muc5b	0.856	http://api.brain-map.org/grid_data/download/70300439?include=energy
Itga7	0.856	http://api.brain-map.org/grid_data/download/73520991?include=energy
Ikbke	0.856	http://api.brain-map.org/grid_data/download/68162236?include=energy
Clp1	0.856	http://api.brain-map.org/grid_data/download/532573?include=energy
Pign	0.856	http://api.brain-map.org/grid_data/download/513018?include=energy
A230072I06Rik	0.856	http://api.brain-map.org/grid_data/download/73906251?include=energy
Rps15a	0.856	http://api.brain-map.org/grid_data/download/74357560?include=energy
1700055D18Rik	0.856	http://api.brain-map.org/grid_data/download/69353720?include=energy
Galns	0.856	http://api.brain-map.org/grid_data/download/70612726?include=energy
Tmem168	0.856	http://api.brain-map.org/grid_data/download/75774112?include=energy
Ruvbl2	0.856	http://api.brain-map.org/grid_data/download/69816816?include=energy
LOC433420	0.856	http://api.brain-map.org/grid_data/download/70723235?include=energy
Fbxo11	0.856	http://api.brain-map.org/grid_data/download/71529779?include=energy
Atf7ip2	0.856	http://api.brain-map.org/grid_data/download/70305407?include=energy
Nol9	0.855	http://api.brain-map.org/grid_data/download/69407630?include=energy
Stoml3	0.855	http://api.brain-map.org/grid_data/download/69887373?include=energy
Acbd3	0.855	http://api.brain-map.org/grid_data/download/70717986?include=energy
Gle1	0.855	http://api.brain-map.org/grid_data/download/68637945?include=energy
A730096I18Rik*	0.855	http://api.brain-map.org/grid_data/download/75144645?include=energy
Mtus1	0.855	http://api.brain-map.org/grid_data/download/73712762?include=energy
Psme4	0.855	http://api.brain-map.org/grid_data/download/69817444?include=energy
Uqcr10	0.855	http://api.brain-map.org/grid_data/download/71763957?include=energy
Gm371	0.855	http://api.brain-map.org/grid_data/download/70296008?include=energy
Nkpd1	0.855	http://api.brain-map.org/grid_data/download/70300547?include=energy
Ldlrap1	0.855	http://api.brain-map.org/grid_data/download/2010?include=energy
Ppp2r5e	0.855	http://api.brain-map.org/grid_data/download/71280682?include=energy
Pop4	0.855	http://api.brain-map.org/grid_data/download/357096?include=energy
Stxbp5l	0.855	http://api.brain-map.org/grid_data/download/69887433?include=energy
Gm1131	0.855	http://api.brain-map.org/grid_data/download/74509590?include=energy
Syce3	0.855	http://api.brain-map.org/grid_data/download/73497693?include=energy
Etv4	0.855	http://api.brain-map.org/grid_data/download/71307298?include=energy
Chsy1	0.855	http://api.brain-map.org/grid_data/download/69548939?include=energy
Tnfaip8l1	0.855	http://api.brain-map.org/grid_data/download/68161459?include=energy
Nlgn1	0.855	http://api.brain-map.org/grid_data/download/70300553?include=energy
Ypel1	0.855	http://api.brain-map.org/grid_data/download/69114352?include=energy
Pnck	0.855	http://api.brain-map.org/grid_data/download/827?include=energy
Aox3l1	0.855	http://api.brain-map.org/grid_data/download/73513417?include=energy
Spred3	0.855	http://api.brain-map.org/grid_data/download/69608507?include=energy
Skp2	0.855	http://api.brain-map.org/grid_data/download/69134468?include=energy
Tmem71	0.855	http://api.brain-map.org/grid_data/download/69526396?include=energy
A4gnt	0.855	http://api.brain-map.org/grid_data/download/71569169?include=energy
Mmp12	0.855	http://api.brain-map.org/grid_data/download/68445179?include=energy
Stab1	0.855	http://api.brain-map.org/grid_data/download/68745102?include=energy
Slc5a6	0.855	http://api.brain-map.org/grid_data/download/73617510?include=energy
Ddx3x	0.855	http://api.brain-map.org/grid_data/download/337?include=energy
Ovca2	0.855	http://api.brain-map.org/grid_data/download/71210753?include=energy
Efcab5	0.855	http://api.brain-map.org/grid_data/download/73584825?include=energy
Nup188	0.855	http://api.brain-map.org/grid_data/download/69782485?include=energy
Mgat5	0.855	http://api.brain-map.org/grid_data/download/73931638?include=energy
Ucn	0.855	http://api.brain-map.org/grid_data/download/1435?include=energy
Dll3	0.855	http://api.brain-map.org/grid_data/download/81657663?include=energy
Sft2d1	0.855	http://api.brain-map.org/grid_data/download/79536367?include=energy
Oxnad1	0.855	http://api.brain-map.org/grid_data/download/641537?include=energy
mCG61405.1	0.855	http://api.brain-map.org/grid_data/download/71213722?include=energy
Pet112l	0.855	http://api.brain-map.org/grid_data/download/70437852?include=energy
Gucy1a2	0.855	http://api.brain-map.org/grid_data/download/74431661?include=energy
Bmi1	0.855	http://api.brain-map.org/grid_data/download/68667934?include=energy
LOC434713	0.855	http://api.brain-map.org/grid_data/download/71022403?include=energy
Hes1	0.855	http://api.brain-map.org/grid_data/download/556?include=energy
Bckdha	0.855	http://api.brain-map.org/grid_data/download/68443080?include=energy
Zfp518b	0.855	http://api.brain-map.org/grid_data/download/69445427?include=energy
Gm2447	0.855	http://api.brain-map.org/grid_data/download/70305287?include=energy
Ssr3	0.855	http://api.brain-map.org/grid_data/download/68301670?include=energy
Kctd16	0.855	http://api.brain-map.org/grid_data/download/73636009?include=energy
Gls2	0.855	http://api.brain-map.org/grid_data/download/69289069?include=energy
Rnf31	0.855	http://api.brain-map.org/grid_data/download/68861810?include=energy
B4galnt4	0.855	http://api.brain-map.org/grid_data/download/71211764?include=energy
1700042B14Rik	0.855	http://api.brain-map.org/grid_data/download/71920123?include=energy
Ush1c	0.855	http://api.brain-map.org/grid_data/download/68341340?include=energy
A330069K06Rik	0.855	http://api.brain-map.org/grid_data/download/71494684?include=energy
Cc2d1a	0.855	http://api.brain-map.org/grid_data/download/70430852?include=energy
Cep57	0.855	http://api.brain-map.org/grid_data/download/71764029?include=energy
LOC545666	0.855	http://api.brain-map.org/grid_data/download/70608466?include=energy
Lrrfip1	0.855	http://api.brain-map.org/grid_data/download/71920431?include=energy
Prim1	0.855	http://api.brain-map.org/grid_data/download/77464986?include=energy
Fam136a	0.855	http://api.brain-map.org/grid_data/download/1964?include=energy
Nphs2	0.855	http://api.brain-map.org/grid_data/download/76003714?include=energy
Caskin1	0.855	http://api.brain-map.org/grid_data/download/69288836?include=energy
Cacna1e	0.855	http://api.brain-map.org/grid_data/download/74988669?include=energy
Arhgap9	0.855	http://api.brain-map.org/grid_data/download/67881767?include=energy
Lin7c	0.855	http://api.brain-map.org/grid_data/download/74822936?include=energy
Ankrd35	0.855	http://api.brain-map.org/grid_data/download/70205440?include=energy
Rcan1	0.855	http://api.brain-map.org/grid_data/download/362?include=energy
Dscr3	0.855	http://api.brain-map.org/grid_data/download/413830?include=energy
Cdc42se1	0.855	http://api.brain-map.org/grid_data/download/69116877?include=energy
LOC432422	0.855	http://api.brain-map.org/grid_data/download/71808373?include=energy
9430016H08Rik	0.854	http://api.brain-map.org/grid_data/download/70445550?include=energy
Htra2	0.854	http://api.brain-map.org/grid_data/download/69262306?include=energy
E230016K23Rik	0.854	http://api.brain-map.org/grid_data/download/70239021?include=energy
Dpcd	0.854	http://api.brain-map.org/grid_data/download/68545527?include=energy
Peli3	0.854	http://api.brain-map.org/grid_data/download/67814218?include=energy
Gabrb1	0.854	http://api.brain-map.org/grid_data/download/2247?include=energy
Iqcg	0.854	http://api.brain-map.org/grid_data/download/70218739?include=energy
Fam63a	0.854	http://api.brain-map.org/grid_data/download/70717357?include=energy
Fam53a	0.854	http://api.brain-map.org/grid_data/download/583842?include=energy
Ptges	0.854	http://api.brain-map.org/grid_data/download/70565141?include=energy
Btg4	0.854	http://api.brain-map.org/grid_data/download/75988334?include=energy
Adamts12	0.854	http://api.brain-map.org/grid_data/download/69514786?include=energy
Fam171b	0.854	http://api.brain-map.org/grid_data/download/69608953?include=energy
Lin7b	0.854	http://api.brain-map.org/grid_data/download/73617678?include=energy
Lmcd1	0.854	http://api.brain-map.org/grid_data/download/68445688?include=energy
C730047G04Rik*	0.854	http://api.brain-map.org/grid_data/download/74000654?include=energy
Spata24	0.854	http://api.brain-map.org/grid_data/download/75931478?include=energy
Irgm2	0.854	http://api.brain-map.org/grid_data/download/69549149?include=energy
Ssx2ip	0.854	http://api.brain-map.org/grid_data/download/73817985?include=energy
Efcab3	0.854	http://api.brain-map.org/grid_data/download/72129254?include=energy
Pxk	0.854	http://api.brain-map.org/grid_data/download/71487172?include=energy
Ptplb	0.854	http://api.brain-map.org/grid_data/download/75695645?include=energy
4930583I09Rik	0.854	http://api.brain-map.org/grid_data/download/74580709?include=energy
F830108O16Rik*	0.854	http://api.brain-map.org/grid_data/download/73748683?include=energy
Prg2	0.854	http://api.brain-map.org/grid_data/download/76081023?include=energy
Usp20	0.854	http://api.brain-map.org/grid_data/download/72104170?include=energy
Nqo1	0.854	http://api.brain-map.org/grid_data/download/74800915?include=energy
Rnf214	0.854	http://api.brain-map.org/grid_data/download/69608501?include=energy
Opn5	0.854	http://api.brain-map.org/grid_data/download/69258226?include=energy
A530095I07Rik	0.854	http://api.brain-map.org/grid_data/download/70228507?include=energy
A530030B07Rik*	0.854	http://api.brain-map.org/grid_data/download/73808576?include=energy
F2	0.854	http://api.brain-map.org/grid_data/download/68302245?include=energy
Ankrd40	0.854	http://api.brain-map.org/grid_data/download/70793941?include=energy
Srek1ip1	0.854	http://api.brain-map.org/grid_data/download/69407463?include=energy
Sync	0.854	http://api.brain-map.org/grid_data/download/70302312?include=energy
Strap	0.854	http://api.brain-map.org/grid_data/download/70795527?include=energy
Slco5a1	0.854	http://api.brain-map.org/grid_data/download/69874108?include=energy
Ccr9	0.854	http://api.brain-map.org/grid_data/download/69237053?include=energy
Olfr984	0.854	http://api.brain-map.org/grid_data/download/74735021?include=energy
Afm	0.854	http://api.brain-map.org/grid_data/download/74431098?include=energy
A230066D03Rik	0.854	http://api.brain-map.org/grid_data/download/71992115?include=energy
C130047K18Rik*	0.854	http://api.brain-map.org/grid_data/download/73811627?include=energy
4930549C01Rik	0.854	http://api.brain-map.org/grid_data/download/69113590?include=energy
Prss39	0.854	http://api.brain-map.org/grid_data/download/74749944?include=energy
Ttbk1	0.854	http://api.brain-map.org/grid_data/download/69530965?include=energy
Bcar1	0.854	http://api.brain-map.org/grid_data/download/71656769?include=energy
2900005J15Rik	0.854	http://api.brain-map.org/grid_data/download/69407325?include=energy
Ldb3	0.854	http://api.brain-map.org/grid_data/download/2369?include=energy
4931406H21Rik	0.854	http://api.brain-map.org/grid_data/download/70218462?include=energy
Enpp1	0.854	http://api.brain-map.org/grid_data/download/71381028?include=energy
Nxph4	0.854	http://api.brain-map.org/grid_data/download/73521000?include=energy
Mafg	0.854	http://api.brain-map.org/grid_data/download/631716?include=energy
Map2k3	0.854	http://api.brain-map.org/grid_data/download/79536697?include=energy
Leprel2	0.854	http://api.brain-map.org/grid_data/download/74047912?include=energy
Ar	0.854	http://api.brain-map.org/grid_data/download/2039?include=energy
Tprg	0.854	http://api.brain-map.org/grid_data/download/71808415?include=energy
Clrn1	0.854	http://api.brain-map.org/grid_data/download/74743390?include=energy
Irx6	0.854	http://api.brain-map.org/grid_data/download/100146098?include=energy
Bsnd	0.854	http://api.brain-map.org/grid_data/download/68191210?include=energy
Rnf166	0.854	http://api.brain-map.org/grid_data/download/71020892?include=energy
Trappc8	0.854	http://api.brain-map.org/grid_data/download/70218878?include=energy
Krt77	0.854	http://api.brain-map.org/grid_data/download/71213812?include=energy
Tubb1	0.854	http://api.brain-map.org/grid_data/download/74736444?include=energy
Oog2	0.854	http://api.brain-map.org/grid_data/download/74514261?include=energy
Arhgdig	0.854	http://api.brain-map.org/grid_data/download/79556628?include=energy
Tmco1	0.854	http://api.brain-map.org/grid_data/download/386233?include=energy
Chchd7	0.854	http://api.brain-map.org/grid_data/download/70445664?include=energy
Trmt61a	0.854	http://api.brain-map.org/grid_data/download/69013116?include=energy
Fam3c	0.854	http://api.brain-map.org/grid_data/download/75259443?include=energy
Tmem191c	0.854	http://api.brain-map.org/grid_data/download/71580168?include=energy
Myog	0.854	http://api.brain-map.org/grid_data/download/69289453?include=energy
Cdcp2	0.854	http://api.brain-map.org/grid_data/download/71809373?include=energy
Gm216	0.854	http://api.brain-map.org/grid_data/download/74578311?include=energy
St18	0.854	http://api.brain-map.org/grid_data/download/75038443?include=energy
Solh	0.853	http://api.brain-map.org/grid_data/download/70303636?include=energy
Ankrd42	0.853	http://api.brain-map.org/grid_data/download/71283547?include=energy
Elavl1	0.853	http://api.brain-map.org/grid_data/download/71496553?include=energy
Nosip	0.853	http://api.brain-map.org/grid_data/download/69782324?include=energy

Srl	0.853	http://api.brain-map.org/grid_data/download/69885744?include=energy
5830416P10Rik	0.853	http://api.brain-map.org/grid_data/download/74579410?include=energy
6330581N18Rik	0.853	http://api.brain-map.org/grid_data/download/70717370?include=energy
LOC432671	0.853	http://api.brain-map.org/grid_data/download/70611180?include=energy
Tsen2	0.853	http://api.brain-map.org/grid_data/download/69262153?include=energy
Ciz1	0.853	http://api.brain-map.org/grid_data/download/583870?include=energy
Btg3	0.853	http://api.brain-map.org/grid_data/download/72008120?include=energy
1500012K07Rik	0.853	http://api.brain-map.org/grid_data/download/71492832?include=energy
Zfp668	0.853	http://api.brain-map.org/grid_data/download/70525795?include=energy
Ankrd50	0.853	http://api.brain-map.org/grid_data/download/71670721?include=energy
Mrpl18	0.853	http://api.brain-map.org/grid_data/download/68269748?include=energy
LOC434213	0.853	http://api.brain-map.org/grid_data/download/70787585?include=energy
Cenpi	0.853	http://api.brain-map.org/grid_data/download/70545901?include=energy
4933437F05Rik	0.853	http://api.brain-map.org/grid_data/download/69443256?include=energy
Kcnh6	0.853	http://api.brain-map.org/grid_data/download/74431751?include=energy
Lztr1	0.853	http://api.brain-map.org/grid_data/download/74047877?include=energy
Tbc1d22b	0.853	http://api.brain-map.org/grid_data/download/68845526?include=energy
Cc2d1b	0.853	http://api.brain-map.org/grid_data/download/70717980?include=energy
D930046H04Rik	0.853	http://api.brain-map.org/grid_data/download/70238830?include=energy
Slc41a2	0.853	http://api.brain-map.org/grid_data/download/69447918?include=energy
Cyth1	0.853	http://api.brain-map.org/grid_data/download/69015206?include=energy
Cdkn2aip	0.853	http://api.brain-map.org/grid_data/download/70303887?include=energy
Tmem62	0.853	http://api.brain-map.org/grid_data/download/71495909?include=energy
Olfr1180	0.853	http://api.brain-map.org/grid_data/download/73635544?include=energy
Yipf1	0.853	http://api.brain-map.org/grid_data/download/74881163?include=energy
Adam12	0.853	http://api.brain-map.org/grid_data/download/75988236?include=energy
Baiap2	0.853	http://api.brain-map.org/grid_data/download/73520966?include=energy
mCG20113.1	0.853	http://api.brain-map.org/grid_data/download/71211997?include=energy
Stk38l	0.853	http://api.brain-map.org/grid_data/download/71064288?include=energy
Snd1	0.853	http://api.brain-map.org/grid_data/download/77869128?include=energy
Rdbp	0.853	http://api.brain-map.org/grid_data/download/68744910?include=energy
Triap1	0.853	http://api.brain-map.org/grid_data/download/74658242?include=energy
D6Wsu116e	0.853	http://api.brain-map.org/grid_data/download/68918733?include=energy
Fmo5	0.853	http://api.brain-map.org/grid_data/download/70528671?include=energy
Rsrc2	0.853	http://api.brain-map.org/grid_data/download/73497678?include=energy
Gltscr2	0.853	http://api.brain-map.org/grid_data/download/68193067?include=energy
Arsj	0.853	http://api.brain-map.org/grid_data/download/71249746?include=energy
Ssna1	0.853	http://api.brain-map.org/grid_data/download/69262344?include=energy
Gapdhs	0.853	http://api.brain-map.org/grid_data/download/69672784?include=energy
Rsbn1	0.853	http://api.brain-map.org/grid_data/download/69873325?include=energy
TC1560604	0.853	http://api.brain-map.org/grid_data/download/70919406?include=energy
Zfp280b	0.853	http://api.brain-map.org/grid_data/download/70593392?include=energy
Mllt10	0.853	http://api.brain-map.org/grid_data/download/69289393?include=energy
Gimap4	0.853	http://api.brain-map.org/grid_data/download/72283611?include=energy
Sh2d1a	0.853	http://api.brain-map.org/grid_data/download/68744164?include=energy
Cdc40	0.853	http://api.brain-map.org/grid_data/download/69174076?include=energy
Nacc1	0.853	http://api.brain-map.org/grid_data/download/69530672?include=energy
Eid2b	0.853	http://api.brain-map.org/grid_data/download/70787501?include=energy
Irs4	0.853	http://api.brain-map.org/grid_data/download/71717079?include=energy
Strn3	0.853	http://api.brain-map.org/grid_data/download/69887397?include=energy
1700018B08Rik	0.853	http://api.brain-map.org/grid_data/download/71919499?include=energy
Vamp4	0.853	http://api.brain-map.org/grid_data/download/70194892?include=energy
Rbm8a	0.853	http://api.brain-map.org/grid_data/download/68844379?include=energy
TC1459816	0.853	http://api.brain-map.org/grid_data/download/70795730?include=energy
LOC434823	0.853	http://api.brain-map.org/grid_data/download/71022421?include=energy
Ccdc43	0.852	http://api.brain-map.org/grid_data/download/68862144?include=energy
Zdhhc6	0.852	http://api.brain-map.org/grid_data/download/70445311?include=energy
Med21	0.852	http://api.brain-map.org/grid_data/download/70946171?include=energy
Bcl3	0.852	http://api.brain-map.org/grid_data/download/75934929?include=energy
Auts2	0.852	http://api.brain-map.org/grid_data/download/79591687?include=energy
Gypc	0.852	http://api.brain-map.org/grid_data/download/68522350?include=energy
Il11	0.852	http://api.brain-map.org/grid_data/download/76003674?include=energy
Prorsd1	0.852	http://api.brain-map.org/grid_data/download/70203435?include=energy
Klf12	0.852	http://api.brain-map.org/grid_data/download/69289279?include=energy
Pdx1	0.852	http://api.brain-map.org/grid_data/download/69289213?include=energy
Setd5	0.852	http://api.brain-map.org/grid_data/download/37?include=energy
Pigw	0.852	http://api.brain-map.org/grid_data/download/69783166?include=energy
Cmah	0.852	http://api.brain-map.org/grid_data/download/68743751?include=energy
Tshz1	0.852	http://api.brain-map.org/grid_data/download/72129289?include=energy
Dpp7	0.852	http://api.brain-map.org/grid_data/download/532673?include=energy
Thoc1	0.852	http://api.brain-map.org/grid_data/download/70445257?include=energy
Nprl2	0.852	http://api.brain-map.org/grid_data/download/2701?include=energy
Echdc1	0.852	http://api.brain-map.org/grid_data/download/69838747?include=energy
2210016F16Rik	0.852	http://api.brain-map.org/grid_data/download/70795497?include=energy
3930402G23Rik	0.852	http://api.brain-map.org/grid_data/download/69407576?include=energy
Rprd1b	0.852	http://api.brain-map.org/grid_data/download/70523835?include=energy
Dlx5	0.852	http://api.brain-map.org/grid_data/download/1450?include=energy
Cgrrf1	0.852	http://api.brain-map.org/grid_data/download/678708?include=energy
Olfr1316	0.852	http://api.brain-map.org/grid_data/download/73635586?include=energy
Tom1	0.852	http://api.brain-map.org/grid_data/download/513013?include=energy
Epha3	0.852	http://api.brain-map.org/grid_data/download/72472792?include=energy
Dnase2b	0.852	http://api.brain-map.org/grid_data/download/71587565?include=energy
Nup210	0.852	http://api.brain-map.org/grid_data/download/71234693?include=energy
Anks1	0.852	http://api.brain-map.org/grid_data/download/68862126?include=energy
Parp10	0.852	http://api.brain-map.org/grid_data/download/73808420?include=energy
Sema7a	0.852	http://api.brain-map.org/grid_data/download/955?include=energy
Slc30a1	0.852	http://api.brain-map.org/grid_data/download/69873893?include=energy
Naa30	0.852	http://api.brain-map.org/grid_data/download/71656689?include=energy
Jmjd1c	0.852	http://api.brain-map.org/grid_data/download/69289261?include=energy
Itih2	0.852	http://api.brain-map.org/grid_data/download/69549292?include=energy
Upp1	0.852	http://api.brain-map.org/grid_data/download/71836877?include=energy
Cdk2ap1	0.852	http://api.brain-map.org/grid_data/download/633273?include=energy
Mcm7	0.852	http://api.brain-map.org/grid_data/download/77464988?include=energy
Rnf114	0.852	http://api.brain-map.org/grid_data/download/69013648?include=energy
LOC432906	0.852	http://api.brain-map.org/grid_data/download/70611377?include=energy
Stx3	0.852	http://api.brain-map.org/grid_data/download/74047924?include=energy
Hoxb6	0.852	http://api.brain-map.org/grid_data/download/323827?include=energy
Kdelc1	0.852	http://api.brain-map.org/grid_data/download/67787328?include=energy
Cpsf7	0.852	http://api.brain-map.org/grid_data/download/71210223?include=energy
C030003M13Rik*	0.852	http://api.brain-map.org/grid_data/download/73808367?include=energy
Taok2	0.852	http://api.brain-map.org/grid_data/download/72081744?include=energy
1700001J03Rik	0.852	http://api.brain-map.org/grid_data/download/75860912?include=energy
Wdr70	0.852	http://api.brain-map.org/grid_data/download/70611389?include=energy
Rufy4	0.852	http://api.brain-map.org/grid_data/download/75041573?include=energy
LOC433569	0.852	http://api.brain-map.org/grid_data/download/70785798?include=energy
Sox7	0.852	http://api.brain-map.org/grid_data/download/70593370?include=energy
Gm196	0.852	http://api.brain-map.org/grid_data/download/71670725?include=energy
Gm14015	0.852	http://api.brain-map.org/grid_data/download/75990476?include=energy
Sebox	0.852	http://api.brain-map.org/grid_data/download/756?include=energy
F630028O10Rik*	0.852	http://api.brain-map.org/grid_data/download/73748890?include=energy
Ppp1r1a	0.852	http://api.brain-map.org/grid_data/download/75773706?include=energy
Cdk14	0.852	http://api.brain-map.org/grid_data/download/71670684?include=energy
Usp38	0.852	http://api.brain-map.org/grid_data/download/68798001?include=energy
Atp6v1g3	0.852	http://api.brain-map.org/grid_data/download/70231058?include=energy
Csn3	0.852	http://api.brain-map.org/grid_data/download/75831753?include=energy
2210416O15Rik	0.852	http://api.brain-map.org/grid_data/download/74635325?include=energy
1110054O05Rik	0.852	http://api.brain-map.org/grid_data/download/71570752?include=energy
Sstr5	0.852	http://api.brain-map.org/grid_data/download/69288345?include=energy
2310079F23Rik	0.852	http://api.brain-map.org/grid_data/download/70539254?include=energy
Mrps28	0.852	http://api.brain-map.org/grid_data/download/75259441?include=energy
Rreb1	0.852	http://api.brain-map.org/grid_data/download/72340134?include=energy
Sclt1	0.851	http://api.brain-map.org/grid_data/download/69262120?include=energy
Otog	0.851	http://api.brain-map.org/grid_data/download/71920469?include=energy
A230035D19Rik*	0.851	http://api.brain-map.org/grid_data/download/73906257?include=energy
Taf1c	0.851	http://api.brain-map.org/grid_data/download/69262254?include=energy
Dhcr7	0.851	http://api.brain-map.org/grid_data/download/633278?include=energy
A330087I24	0.851	http://api.brain-map.org/grid_data/download/73513359?include=energy
Sst	0.851	http://api.brain-map.org/grid_data/download/1001?include=energy
2310003F16Rik	0.851	http://api.brain-map.org/grid_data/download/70717354?include=energy
Hoxb2	0.851	http://api.brain-map.org/grid_data/download/100144102?include=energy
Dnmt3a	0.851	http://api.brain-map.org/grid_data/download/71724864?include=energy
Tcta	0.851	http://api.brain-map.org/grid_data/download/68633377?include=energy
Itgax	0.851	http://api.brain-map.org/grid_data/download/69028324?include=energy
C030034I22Rik	0.851	http://api.brain-map.org/grid_data/download/72472751?include=energy
Pet2	0.851	http://api.brain-map.org/grid_data/download/69782999?include=energy
Bckdk	0.851	http://api.brain-map.org/grid_data/download/68745347?include=energy
9330161L09Rik	0.851	http://api.brain-map.org/grid_data/download/74300657?include=energy
Ddx46	0.851	http://api.brain-map.org/grid_data/download/73870761?include=energy
Cwh43	0.851	http://api.brain-map.org/grid_data/download/74819236?include=energy
S100a9	0.851	http://api.brain-map.org/grid_data/download/68744510?include=energy
Ceacam18	0.851	http://api.brain-map.org/grid_data/download/69262285?include=energy
9830001H06Rik	0.851	http://api.brain-map.org/grid_data/download/77340458?include=energy
Thoc2	0.851	http://api.brain-map.org/grid_data/download/69444837?include=energy
LOC433627	0.851	http://api.brain-map.org/grid_data/download/70785840?include=energy
Gm5742	0.851	http://api.brain-map.org/grid_data/download/71456108?include=energy
Gbgt1	0.851	http://api.brain-map.org/grid_data/download/71587703?include=energy
D1Bwg0212e	0.851	http://api.brain-map.org/grid_data/download/69116437?include=energy
Dph1	0.851	http://api.brain-map.org/grid_data/download/71789897?include=energy
Rpp14	0.851	http://api.brain-map.org/grid_data/download/71612223?include=energy
Arhgap22	0.851	http://api.brain-map.org/grid_data/download/678695?include=energy
E2f4	0.851	http://api.brain-map.org/grid_data/download/1195?include=energy
Arhgef11	0.851	http://api.brain-map.org/grid_data/download/70231005?include=energy
Olfr13	0.851	http://api.brain-map.org/grid_data/download/73635574?include=energy
Ap3m2	0.851	http://api.brain-map.org/grid_data/download/70430808?include=energy
Gpr173	0.851	http://api.brain-map.org/grid_data/download/74724531?include=energy
Lass5	0.851	http://api.brain-map.org/grid_data/download/75990553?include=energy
Nipsnap3b	0.851	http://api.brain-map.org/grid_data/download/68341920?include=energy
C3ar1	0.851	http://api.brain-map.org/grid_data/download/79360304?include=energy
Olfr1358	0.851	http://api.brain-map.org/grid_data/download/74720537?include=energy
Prkab2	0.851	http://api.brain-map.org/grid_data/download/69013603?include=energy
4631423B10Rik	0.851	http://api.brain-map.org/grid_data/download/69407588?include=energy
Eomes	0.851	http://api.brain-map.org/grid_data/download/397?include=energy
Ykt6	0.851	http://api.brain-map.org/grid_data/download/71380453?include=energy
Ankrd9	0.851	http://api.brain-map.org/grid_data/download/70445586?include=energy
Gm807	0.851	http://api.brain-map.org/grid_data/download/70298292?include=energy
4930563E22Rik	0.851	http://api.brain-map.org/grid_data/download/73513296?include=energy
4921528O07Rik	0.851	http://api.brain-map.org/grid_data/download/71891629?include=energy
Alox12	0.851	http://api.brain-map.org/grid_data/download/69526558?include=energy
Ralgapb	0.851	http://api.brain-map.org/grid_data/download/69527842?include=energy
Zfp711	0.851	http://api.brain-map.org/grid_data/download/73808522?include=energy
Selk	0.851	http://api.brain-map.org/grid_data/download/74800903?include=energy
Tom1l1	0.851	http://api.brain-map.org/grid_data/download/74359389?include=energy
Cyth3	0.851	http://api.brain-map.org/grid_data/download/74357632?include=energy
Eda2r	0.851	http://api.brain-map.org/grid_data/download/69671887?include=energy
Lce1c	0.851	http://api.brain-map.org/grid_data/download/75198130?include=energy
Pop5	0.851	http://api.brain-map.org/grid_data/download/67815954?include=energy
Sprr2a1	0.851	http://api.brain-map.org/grid_data/download/75694866?include=energy
Tmem80	0.851	http://api.brain-map.org/grid_data/download/71493753?include=energy
Plaa	0.851	http://api.brain-map.org/grid_data/download/69783280?include=energy
LOC545086	0.851	http://api.brain-map.org/grid_data/download/100146079?include=energy
Casc4	0.851	http://api.brain-map.org/grid_data/download/70305658?include=energy
BC046404	0.851	http://api.brain-map.org/grid_data/download/70528354?include=energy
Mfrp	0.85	http://api.brain-map.org/grid_data/download/70300312?include=energy
Urb2	0.85	http://api.brain-map.org/grid_data/download/73513404?include=energy
Rpusd4	0.85	http://api.brain-map.org/grid_data/download/70919191?include=energy
Utp15	0.85	http://api.brain-map.org/grid_data/download/76098382?include=energy
Ptprm	0.85	http://api.brain-map.org/grid_data/download/864?include=energy
Wdr31	0.85	http://api.brain-map.org/grid_data/download/2719?include=energy
Mdfic	0.85	http://api.brain-map.org/grid_data/download/71232889?include=energy
Pex11c	0.85	http://api.brain-map.org/grid_data/download/69783005?include=energy
Gpr112	0.85	http://api.brain-map.org/grid_data/download/73497023?include=energy
Lum	0.85	http://api.brain-map.org/grid_data/download/324012?include=energy
Ceacam15	0.85	http://api.brain-map.org/grid_data/download/71021010?include=energy
Dnajb9	0.85	http://api.brain-map.org/grid_data/download/77454993?include=energy
Myo1e	0.85	http://api.brain-map.org/grid_data/download/69013167?include=energy
Atpaf2	0.85	http://api.brain-map.org/grid_data/download/77904923?include=energy
Ints12	0.85	http://api.brain-map.org/grid_data/download/68924805?include=energy
Tbl1xr1	0.85	http://api.brain-map.org/grid_data/download/69446250?include=energy
Samd12	0.85	http://api.brain-map.org/grid_data/download/71809346?include=energy
1600012F09Rik	0.85	http://api.brain-map.org/grid_data/download/69113602?include=energy
C230066G23Rik	0.85	http://api.brain-map.org/grid_data/download/69530911?include=energy
Gdf2	0.85	http://api.brain-map.org/grid_data/download/70276873?include=energy
1700101I19Rik	0.85	http://api.brain-map.org/grid_data/download/74359072?include=energy
Il5ra	0.85	http://api.brain-map.org/grid_data/download/69735419?include=energy
Marveld1	0.85	http://api.brain-map.org/grid_data/download/75831756?include=energy
Serpini1	0.85	http://api.brain-map.org/grid_data/download/963?include=energy
Nudt16l1	0.85	http://api.brain-map.org/grid_data/download/68161470?include=energy
Mpp4	0.85	http://api.brain-map.org/grid_data/download/68744970?include=energy
Chst7	0.85	http://api.brain-map.org/grid_data/download/556996?include=energy
Il17rd	0.85	http://api.brain-map.org/grid_data/download/74958537?include=energy
Pvt1	0.85	http://api.brain-map.org/grid_data/download/74359718?include=energy
F830006F03Rik*	0.85	http://api.brain-map.org/grid_data/download/73731487?include=energy
H2-Aa	0.85	http://api.brain-map.org/grid_data/download/68633131?include=energy
Gpbp1l1	0.85	http://api.brain-map.org/grid_data/download/70445484?include=energy
Cd44	0.85	http://api.brain-map.org/grid_data/download/79556644?include=energy
D3Ertd254e	0.85	http://api.brain-map.org/grid_data/download/71489813?include=energy
Usp51	0.85	http://api.brain-map.org/grid_data/download/71213761?include=energy
4930417O22Rik	0.85	http://api.brain-map.org/grid_data/download/75934516?include=energy
Akr1b3	0.85	http://api.brain-map.org/grid_data/download/70813922?include=energy
C230086J09Rik	0.85	http://api.brain-map.org/grid_data/download/71735076?include=energy
Cdh3	0.85	http://api.brain-map.org/grid_data/download/1337?include=energy
Irf1	0.85	http://api.brain-map.org/grid_data/download/79539604?include=energy
Enpp4	0.85	http://api.brain-map.org/grid_data/download/74357719?include=energy
6430598A04Rik	0.85	http://api.brain-map.org/grid_data/download/69549614?include=energy
Ss18l1	0.85	http://api.brain-map.org/grid_data/download/68799125?include=energy
Lman1	0.85	http://api.brain-map.org/grid_data/download/69015254?include=energy
Zscan10	0.85	http://api.brain-map.org/grid_data/download/70231797?include=energy
Calb1	0.85	http://api.brain-map.org/grid_data/download/71717640?include=energy
Pddc1	0.85	http://api.brain-map.org/grid_data/download/68158081?include=energy
Ccdc80	0.85	http://api.brain-map.org/grid_data/download/74821579?include=energy
Anp32a	0.85	http://api.brain-map.org/grid_data/download/94?include=energy
Bola3	0.85	http://api.brain-map.org/grid_data/download/69201666?include=energy
Spinlw1	0.85	http://api.brain-map.org/grid_data/download/69114679?include=energy
Wdr74	0.85	http://api.brain-map.org/grid_data/download/68341943?include=energy
Igsf9b	0.849	http://api.brain-map.org/grid_data/download/75147761?include=energy
Atg2a	0.849	http://api.brain-map.org/grid_data/download/69837590?include=energy
Foxp3	0.849	http://api.brain-map.org/grid_data/download/69289004?include=energy
Atf5	0.849	http://api.brain-map.org/grid_data/download/73716662?include=energy
Rbm45	0.849	http://api.brain-map.org/grid_data/download/69028288?include=energy
9330190M22Rik*	0.849	http://api.brain-map.org/grid_data/download/73906209?include=energy
Serf2	0.849	http://api.brain-map.org/grid_data/download/69873558?include=energy
Mmp2	0.849	http://api.brain-map.org/grid_data/download/70946591?include=energy
Heatr6	0.849	http://api.brain-map.org/grid_data/download/631705?include=energy
Art3	0.849	http://api.brain-map.org/grid_data/download/70231017?include=energy
Pcna	0.849	http://api.brain-map.org/grid_data/download/75826584?include=energy
1700073E17Rik	0.849	http://api.brain-map.org/grid_data/download/70525773?include=energy
Fndc8	0.849	http://api.brain-map.org/grid_data/download/71891725?include=energy
Mms22l	0.849	http://api.brain-map.org/grid_data/download/74000630?include=energy
4930547C10Rik	0.849	http://api.brain-map.org/grid_data/download/70218330?include=energy
2010005H15Rik	0.849	http://api.brain-map.org/grid_data/download/71991678?include=energy
Ccdc84	0.849	http://api.brain-map.org/grid_data/download/71808668?include=energy
Gnb2l1	0.849	http://api.brain-map.org/grid_data/download/71021172?include=energy
Crcp	0.849	http://api.brain-map.org/grid_data/download/76097713?include=energy
C430049E01Rik*	0.849	http://api.brain-map.org/grid_data/download/74000636?include=energy
Mcoln1	0.849	http://api.brain-map.org/grid_data/download/69258148?include=energy
4933422H20Rik	0.849	http://api.brain-map.org/grid_data/download/71832793?include=energy
Bai3	0.849	http://api.brain-map.org/grid_data/download/71247597?include=energy
Ssh1	0.849	http://api.brain-map.org/grid_data/download/69672928?include=energy
Gm889	0.849	http://api.brain-map.org/grid_data/download/71832951?include=energy
Deptor	0.849	http://api.brain-map.org/grid_data/download/75749773?include=energy
Gstt2	0.849	http://api.brain-map.org/grid_data/download/100146056?include=energy
Nfyc	0.849	http://api.brain-map.org/grid_data/download/112197140?include=energy
1500031L02Rik	0.849	http://api.brain-map.org/grid_data/download/68157411?include=energy
2010012O05Rik	0.849	http://api.brain-map.org/grid_data/download/68545687?include=energy
Cask	0.849	http://api.brain-map.org/grid_data/download/70918868?include=energy
Htr1a	0.849	http://api.brain-map.org/grid_data/download/79556616?include=energy
Hoxb4	0.849	http://api.brain-map.org/grid_data/download/76081918?include=energy

Pla2g6	0.849	http://api.brain-map.org/grid_data/download/80341093?include=energy
Tomm34	0.849	http://api.brain-map.org/grid_data/download/75042253?include=energy
Golga3	0.849	http://api.brain-map.org/grid_data/download/68799131?include=energy
Phlda2	0.849	http://api.brain-map.org/grid_data/download/68446057?include=energy
Tmem237	0.849	http://api.brain-map.org/grid_data/download/70298537?include=energy
Ermap	0.849	http://api.brain-map.org/grid_data/download/68844875?include=energy
Clnk	0.849	http://api.brain-map.org/grid_data/download/75934515?include=energy
Adam3	0.849	http://api.brain-map.org/grid_data/download/75860980?include=energy
Prl8a6	0.849	http://api.brain-map.org/grid_data/download/71919254?include=energy
Mettl5	0.849	http://api.brain-map.org/grid_data/download/74821968?include=energy
Dnajc5g	0.849	http://api.brain-map.org/grid_data/download/72182962?include=energy
Prrx1	0.849	http://api.brain-map.org/grid_data/download/71161507?include=energy
Nars2	0.849	http://api.brain-map.org/grid_data/download/68523231?include=energy
Cyp4a12a	0.849	http://api.brain-map.org/grid_data/download/69015751?include=energy
Gramd2	0.849	http://api.brain-map.org/grid_data/download/73712906?include=energy
Col16a1	0.849	http://api.brain-map.org/grid_data/download/74273122?include=energy
B3gnt8	0.849	http://api.brain-map.org/grid_data/download/68410584?include=energy
Crhr2	0.849	http://api.brain-map.org/grid_data/download/79907934?include=energy
Anapc11	0.849	http://api.brain-map.org/grid_data/download/70445580?include=energy
Mecom	0.849	http://api.brain-map.org/grid_data/download/77924536?include=energy
Zfp444	0.849	http://api.brain-map.org/grid_data/download/70946183?include=energy
Dyrk1a	0.849	http://api.brain-map.org/grid_data/download/79907941?include=energy
BC053749	0.849	http://api.brain-map.org/grid_data/download/73423781?include=energy
Bace2	0.849	http://api.brain-map.org/grid_data/download/143?include=energy
Loxhd1	0.849	http://api.brain-map.org/grid_data/download/73514737?include=energy
A230032E15Rik*	0.849	http://api.brain-map.org/grid_data/download/73949572?include=energy
Phactr2	0.849	http://api.brain-map.org/grid_data/download/73521004?include=energy
Pias3	0.849	http://api.brain-map.org/grid_data/download/67764911?include=energy
Xcl1	0.849	http://api.brain-map.org/grid_data/download/68744116?include=energy
Slc13a3	0.849	http://api.brain-map.org/grid_data/download/2575?include=energy
Fam96a	0.849	http://api.brain-map.org/grid_data/download/77904919?include=energy
Arl13a	0.848	http://api.brain-map.org/grid_data/download/71574428?include=energy
Morc2a	0.848	http://api.brain-map.org/grid_data/download/68844833?include=energy
Il21	0.848	http://api.brain-map.org/grid_data/download/76081012?include=energy
Trak2	0.848	http://api.brain-map.org/grid_data/download/75990672?include=energy
Ascc2	0.848	http://api.brain-map.org/grid_data/download/686207?include=energy
Ttc7b	0.848	http://api.brain-map.org/grid_data/download/72004450?include=energy
Hdhd1a	0.848	http://api.brain-map.org/grid_data/download/70201540?include=energy
Ptpdc1	0.848	http://api.brain-map.org/grid_data/download/72472738?include=energy
Mynn	0.848	http://api.brain-map.org/grid_data/download/69134673?include=energy
Pkp3	0.848	http://api.brain-map.org/grid_data/download/69783262?include=energy
Slc6a19	0.848	http://api.brain-map.org/grid_data/download/73617516?include=energy
Zic1	0.848	http://api.brain-map.org/grid_data/download/72103843?include=energy
Hao2	0.848	http://api.brain-map.org/grid_data/download/556991?include=energy
Stk32b	0.848	http://api.brain-map.org/grid_data/download/69887361?include=energy
Kprp	0.848	http://api.brain-map.org/grid_data/download/70785828?include=energy
Zfp369	0.848	http://api.brain-map.org/grid_data/download/75197570?include=energy
Neu1	0.848	http://api.brain-map.org/grid_data/download/532607?include=energy
5430405G05Rik	0.848	http://api.brain-map.org/grid_data/download/69443409?include=energy
A230097P14Rik*	0.848	http://api.brain-map.org/grid_data/download/75042236?include=energy
Zfp192	0.848	http://api.brain-map.org/grid_data/download/69133731?include=energy
Lck	0.848	http://api.brain-map.org/grid_data/download/68301999?include=energy
Epn1	0.848	http://api.brain-map.org/grid_data/download/76003523?include=energy
Txndc17	0.848	http://api.brain-map.org/grid_data/download/75828179?include=energy
Ttc24	0.848	http://api.brain-map.org/grid_data/download/69116351?include=energy
Slc9a5	0.848	http://api.brain-map.org/grid_data/download/74562600?include=energy
Ggps1	0.848	http://api.brain-map.org/grid_data/download/71723936?include=energy
1810032O08Rik	0.848	http://api.brain-map.org/grid_data/download/68861927?include=energy
Tmtc2	0.848	http://api.brain-map.org/grid_data/download/69446298?include=energy
A230044A09Rik*	0.848	http://api.brain-map.org/grid_data/download/73906286?include=energy
Lysmd1	0.848	http://api.brain-map.org/grid_data/download/70474953?include=energy
Krt6a	0.848	http://api.brain-map.org/grid_data/download/71991834?include=energy
BC030307	0.848	http://api.brain-map.org/grid_data/download/678688?include=energy
Msrb2	0.848	http://api.brain-map.org/grid_data/download/76135795?include=energy
Kiss1r	0.848	http://api.brain-map.org/grid_data/download/80343334?include=energy
Stk33	0.848	http://api.brain-map.org/grid_data/download/74641163?include=energy
Kcnj4	0.848	http://api.brain-map.org/grid_data/download/74357568?include=energy
Lcp1	0.848	http://api.brain-map.org/grid_data/download/71612196?include=energy
Kcnq2	0.848	http://api.brain-map.org/grid_data/download/73496947?include=energy
Mgat4b	0.848	http://api.brain-map.org/grid_data/download/71489801?include=energy
LOC434771	0.848	http://api.brain-map.org/grid_data/download/70725609?include=energy
Rsbn1l	0.848	http://api.brain-map.org/grid_data/download/71147815?include=energy
Rnf157	0.848	http://api.brain-map.org/grid_data/download/74562224?include=energy
2310015B20Rik	0.848	http://api.brain-map.org/grid_data/download/74635364?include=energy
Trove2	0.848	http://api.brain-map.org/grid_data/download/71656917?include=energy
Jph2	0.848	http://api.brain-map.org/grid_data/download/68498534?include=energy
Plaur	0.848	http://api.brain-map.org/grid_data/download/79632246?include=energy
2310047M10Rik	0.848	http://api.brain-map.org/grid_data/download/69854162?include=energy
Api5	0.848	http://api.brain-map.org/grid_data/download/68443104?include=energy
Gm6653	0.848	http://api.brain-map.org/grid_data/download/73870680?include=energy
Tcf7l1	0.848	http://api.brain-map.org/grid_data/download/1424?include=energy
Hoxd4	0.848	http://api.brain-map.org/grid_data/download/74514147?include=energy
Zfp87	0.848	http://api.brain-map.org/grid_data/download/68637874?include=energy
Ccni	0.848	http://api.brain-map.org/grid_data/download/74988529?include=energy
mCG140870	0.848	http://api.brain-map.org/grid_data/download/76003319?include=energy
Gpr22	0.848	http://api.brain-map.org/grid_data/download/74635731?include=energy
Spink10	0.848	http://api.brain-map.org/grid_data/download/73818813?include=energy
Emx2	0.848	http://api.brain-map.org/grid_data/download/100142559?include=energy
Tmem40	0.847	http://api.brain-map.org/grid_data/download/76098295?include=energy
Eps8	0.847	http://api.brain-map.org/grid_data/download/1705?include=energy
Vps4a	0.847	http://api.brain-map.org/grid_data/download/68300956?include=energy
2700046A07Rik	0.847	http://api.brain-map.org/grid_data/download/70542920?include=energy
Hmgxb3	0.847	http://api.brain-map.org/grid_data/download/69288584?include=energy
Crabp1	0.847	http://api.brain-map.org/grid_data/download/1179?include=energy
Fancl	0.847	http://api.brain-map.org/grid_data/download/77620247?include=energy
4833420G11Rik	0.847	http://api.brain-map.org/grid_data/download/71456132?include=energy
Tcf23	0.847	http://api.brain-map.org/grid_data/download/70302431?include=energy
Gm4776	0.847	http://api.brain-map.org/grid_data/download/70303911?include=energy
2010109K11Rik	0.847	http://api.brain-map.org/grid_data/download/70303767?include=energy
2610034B18Rik	0.847	http://api.brain-map.org/grid_data/download/68157399?include=energy
Phgr1	0.847	http://api.brain-map.org/grid_data/download/68666726?include=energy
Liph	0.847	http://api.brain-map.org/grid_data/download/77280417?include=energy
Krt28	0.847	http://api.brain-map.org/grid_data/download/70723682?include=energy
Gm1627	0.847	http://api.brain-map.org/grid_data/download/72472396?include=energy
Chst12	0.847	http://api.brain-map.org/grid_data/download/74881144?include=energy
Izumo3	0.847	http://api.brain-map.org/grid_data/download/72081652?include=energy
Vax2	0.847	http://api.brain-map.org/grid_data/download/69290722?include=energy
Lrrc15	0.847	http://api.brain-map.org/grid_data/download/75831710?include=energy
Nprl3	0.847	http://api.brain-map.org/grid_data/download/69261427?include=energy
St6galnac4	0.847	http://api.brain-map.org/grid_data/download/71280607?include=energy
LOC433187	0.847	http://api.brain-map.org/grid_data/download/70722984?include=energy
Klhl5	0.847	http://api.brain-map.org/grid_data/download/67815553?include=energy
D3Bwg0562e	0.847	http://api.brain-map.org/grid_data/download/73992941?include=energy
Mcm5	0.847	http://api.brain-map.org/grid_data/download/2403?include=energy
Myo3a	0.847	http://api.brain-map.org/grid_data/download/69289441?include=energy
Sulf2	0.847	http://api.brain-map.org/grid_data/download/72007935?include=energy
Kpna1	0.847	http://api.brain-map.org/grid_data/download/2357?include=energy
Gprin2	0.847	http://api.brain-map.org/grid_data/download/70611335?include=energy
9230112E08Rik	0.847	http://api.brain-map.org/grid_data/download/70717376?include=energy
Stab2	0.847	http://api.brain-map.org/grid_data/download/69887307?include=energy
Ablim1	0.847	http://api.brain-map.org/grid_data/download/77620820?include=energy
1110038F14Rik	0.847	http://api.brain-map.org/grid_data/download/68546155?include=energy
Caml	0.847	http://api.brain-map.org/grid_data/download/79568047?include=energy
Serpinb9e	0.847	http://api.brain-map.org/grid_data/download/68667306?include=energy
Galnt2	0.847	http://api.brain-map.org/grid_data/download/68911014?include=energy
Mlc1	0.847	http://api.brain-map.org/grid_data/download/1770?include=energy
Tas1r2	0.847	http://api.brain-map.org/grid_data/download/74056139?include=energy
Spam1	0.847	http://api.brain-map.org/grid_data/download/69885666?include=energy
Ociad2	0.847	http://api.brain-map.org/grid_data/download/75041527?include=energy
B230214G05	0.847	http://api.brain-map.org/grid_data/download/70305431?include=energy
1700019N19Rik	0.847	http://api.brain-map.org/grid_data/download/69114465?include=energy
Dnajb3	0.847	http://api.brain-map.org/grid_data/download/69626957?include=energy
Phf20	0.847	http://api.brain-map.org/grid_data/download/69816446?include=energy
Hoxc9	0.847	http://api.brain-map.org/grid_data/download/74635683?include=energy
Rac2	0.847	http://api.brain-map.org/grid_data/download/79534949?include=energy
Hinfp	0.847	http://api.brain-map.org/grid_data/download/67767461?include=energy
Rngtt	0.847	http://api.brain-map.org/grid_data/download/68920695?include=energy
Cacng1	0.847	http://api.brain-map.org/grid_data/download/69236945?include=energy
D030059C06Rik	0.847	http://api.brain-map.org/grid_data/download/71161471?include=energy
Clec4a3	0.847	http://api.brain-map.org/grid_data/download/71991953?include=energy
Vprbp	0.847	http://api.brain-map.org/grid_data/download/69527986?include=energy
Ccdc159	0.847	http://api.brain-map.org/grid_data/download/69854587?include=energy
Urm1	0.847	http://api.brain-map.org/grid_data/download/70429124?include=energy
Kcnu1	0.847	http://api.brain-map.org/grid_data/download/74431610?include=energy
Tsga8	0.847	http://api.brain-map.org/grid_data/download/74276888?include=energy
Nat10	0.847	http://api.brain-map.org/grid_data/download/69015470?include=energy
LOC545403	0.847	http://api.brain-map.org/grid_data/download/75043635?include=energy
Pnpt1	0.847	http://api.brain-map.org/grid_data/download/69838568?include=energy
4933411K20Rik	0.847	http://api.brain-map.org/grid_data/download/69262232?include=energy
Emr4	0.847	http://api.brain-map.org/grid_data/download/70593138?include=energy
Gm6583	0.847	http://api.brain-map.org/grid_data/download/73716704?include=energy
Sell	0.847	http://api.brain-map.org/grid_data/download/68667787?include=energy
Dtx2	0.846	http://api.brain-map.org/grid_data/download/67764935?include=energy
Tyms-ps	0.846	http://api.brain-map.org/grid_data/download/70192189?include=energy
D630030B08Rik	0.846	http://api.brain-map.org/grid_data/download/71148142?include=energy
Hist2h3c2	0.846	http://api.brain-map.org/grid_data/download/71627718?include=energy
Actr10	0.846	http://api.brain-map.org/grid_data/download/64?include=energy
Ndufc1	0.846	http://api.brain-map.org/grid_data/download/69782151?include=energy
Olfr1441	0.846	http://api.brain-map.org/grid_data/download/74720832?include=energy
Necab3	0.846	http://api.brain-map.org/grid_data/download/73771235?include=energy
4632415K11Rik	0.846	http://api.brain-map.org/grid_data/download/68498621?include=energy
Ccdc11	0.846	http://api.brain-map.org/grid_data/download/70218780?include=energy
Olfr868	0.846	http://api.brain-map.org/grid_data/download/74734985?include=energy
Trappc2	0.846	http://api.brain-map.org/grid_data/download/68744940?include=energy
2300009A05Rik	0.846	http://api.brain-map.org/grid_data/download/69202334?include=energy
Oit1	0.846	http://api.brain-map.org/grid_data/download/308055433?include=energy
Pax9	0.846	http://api.brain-map.org/grid_data/download/2466?include=energy
Trmt2a	0.846	http://api.brain-map.org/grid_data/download/68745257?include=energy
Gm5105	0.846	http://api.brain-map.org/grid_data/download/70429082?include=energy
Oxsr1	0.846	http://api.brain-map.org/grid_data/download/69782593?include=energy
Junb	0.846	http://api.brain-map.org/grid_data/download/2335?include=energy
Gm4887	0.846	http://api.brain-map.org/grid_data/download/73931418?include=energy
2210017I01Rik	0.846	http://api.brain-map.org/grid_data/download/75043647?include=energy
Gart	0.846	http://api.brain-map.org/grid_data/download/77455064?include=energy
LOC433206	0.846	http://api.brain-map.org/grid_data/download/70723014?include=energy
Nmi	0.846	http://api.brain-map.org/grid_data/download/413813?include=energy
Nav2	0.846	http://api.brain-map.org/grid_data/download/74641113?include=energy
4732490P12	0.846	http://api.brain-map.org/grid_data/download/70303851?include=energy
Tspan10	0.846	http://api.brain-map.org/grid_data/download/68341967?include=energy
Pigh	0.846	http://api.brain-map.org/grid_data/download/69783148?include=energy
LOC432727	0.846	http://api.brain-map.org/grid_data/download/70725501?include=energy
Mlx	0.846	http://api.brain-map.org/grid_data/download/77414147?include=energy
Rpn2	0.846	http://api.brain-map.org/grid_data/download/906?include=energy
A730045E13Rik	0.846	http://api.brain-map.org/grid_data/download/71494732?include=energy
Acot8	0.846	http://api.brain-map.org/grid_data/download/633297?include=energy
Pdcl	0.846	http://api.brain-map.org/grid_data/download/324213?include=energy
Sema3c	0.846	http://api.brain-map.org/grid_data/download/939?include=energy
Gm9805	0.846	http://api.brain-map.org/grid_data/download/74735361?include=energy
Tmem50a	0.846	http://api.brain-map.org/grid_data/download/69407505?include=energy
LOC214488	0.846	http://api.brain-map.org/grid_data/download/100144943?include=energy
1700029M20Rik	0.846	http://api.brain-map.org/grid_data/download/71891611?include=energy
Sema3d	0.846	http://api.brain-map.org/grid_data/download/74581376?include=energy
Pla2g3	0.846	http://api.brain-map.org/grid_data/download/69783274?include=energy
LOC434205	0.846	http://api.brain-map.org/grid_data/download/70787579?include=energy
Ccdc79	0.846	http://api.brain-map.org/grid_data/download/71493230?include=energy
Saa2	0.846	http://api.brain-map.org/grid_data/download/68631881?include=energy
Dnajc5b	0.846	http://api.brain-map.org/grid_data/download/70525775?include=energy
Mfsd12	0.846	http://api.brain-map.org/grid_data/download/69407911?include=energy
LOC432689	0.846	http://api.brain-map.org/grid_data/download/70611210?include=energy
Gpr85	0.846	http://api.brain-map.org/grid_data/download/1738?include=energy
6330563N19Rik*	0.846	http://api.brain-map.org/grid_data/download/73906041?include=energy
1190003J15Rik	0.846	http://api.brain-map.org/grid_data/download/68744038?include=energy
Clec12b	0.846	http://api.brain-map.org/grid_data/download/70218792?include=energy
Eefsec	0.846	http://api.brain-map.org/grid_data/download/67767448?include=energy
Gm5897	0.846	http://api.brain-map.org/grid_data/download/77621678?include=energy
Accn1	0.846	http://api.brain-map.org/grid_data/download/73930838?include=energy
Sspo	0.846	http://api.brain-map.org/grid_data/download/71281295?include=energy
Nuf2	0.846	http://api.brain-map.org/grid_data/download/228?include=energy
Ino80e	0.846	http://api.brain-map.org/grid_data/download/67815968?include=energy
LOC432771	0.846	http://api.brain-map.org/grid_data/download/74514243?include=energy
E330020C20Rik*	0.846	http://api.brain-map.org/grid_data/download/74076126?include=energy
Xrcc4	0.845	http://api.brain-map.org/grid_data/download/68161457?include=energy
LOC434231	0.845	http://api.brain-map.org/grid_data/download/74514123?include=energy
Casr	0.845	http://api.brain-map.org/grid_data/download/72338697?include=energy
Klrg2	0.845	http://api.brain-map.org/grid_data/download/74635394?include=energy
Clk4	0.845	http://api.brain-map.org/grid_data/download/532637?include=energy
Atp12a	0.845	http://api.brain-map.org/grid_data/download/76003622?include=energy
E2f5	0.845	http://api.brain-map.org/grid_data/download/77454578?include=energy
Wnk4	0.845	http://api.brain-map.org/grid_data/download/69817348?include=energy
Ifitm5	0.845	http://api.brain-map.org/grid_data/download/71919098?include=energy
Rapsn	0.845	http://api.brain-map.org/grid_data/download/76081028?include=energy
Rae1	0.845	http://api.brain-map.org/grid_data/download/69262116?include=energy
Pex26	0.845	http://api.brain-map.org/grid_data/download/556993?include=energy
Tmem104	0.845	http://api.brain-map.org/grid_data/download/69531061?include=energy
Srsf9	0.845	http://api.brain-map.org/grid_data/download/75043152?include=energy
Cd163	0.845	http://api.brain-map.org/grid_data/download/76003514?include=energy
1810033F07Rik*	0.845	http://api.brain-map.org/grid_data/download/74359568?include=energy
Rab18	0.845	http://api.brain-map.org/grid_data/download/70303612?include=energy
Rabl3	0.845	http://api.brain-map.org/grid_data/download/68666965?include=energy
Arnt2	0.845	http://api.brain-map.org/grid_data/download/71358590?include=energy
Sycn	0.845	http://api.brain-map.org/grid_data/download/68342069?include=energy
Mm.25549	0.845	http://api.brain-map.org/grid_data/download/71735068?include=energy
Ttpal	0.845	http://api.brain-map.org/grid_data/download/74641323?include=energy
LOC436358	0.845	http://api.brain-map.org/grid_data/download/72472394?include=energy
Wdr47	0.845	http://api.brain-map.org/grid_data/download/74819248?include=energy
Glt1d1	0.845	http://api.brain-map.org/grid_data/download/70218950?include=energy
Dupd1	0.845	http://api.brain-map.org/grid_data/download/71022493?include=energy
Adcyap1	0.845	http://api.brain-map.org/grid_data/download/74511882?include=energy
G370141D04Rik*	0.845	http://api.brain-map.org/grid_data/download/73716698?include=energy
Txlnb	0.845	http://api.brain-map.org/grid_data/download/71492982?include=energy
LOC432901	0.845	http://api.brain-map.org/grid_data/download/70611371?include=energy
Tmem121	0.845	http://api.brain-map.org/grid_data/download/68920683?include=energy
Ucp3	0.845	http://api.brain-map.org/grid_data/download/70194546?include=energy
Enpep	0.845	http://api.brain-map.org/grid_data/download/73769289?include=energy
Krtap4-7	0.845	http://api.brain-map.org/grid_data/download/70724929?include=energy
Nlrp4b	0.845	http://api.brain-map.org/grid_data/download/75931603?include=energy
Hs3st3a1	0.845	http://api.brain-map.org/grid_data/download/69735102?include=energy
Brd7	0.845	http://api.brain-map.org/grid_data/download/69529412?include=energy
Dnmt1	0.845	http://api.brain-map.org/grid_data/download/68798885?include=energy
E130309F12Rik	0.845	http://api.brain-map.org/grid_data/download/69671768?include=energy
Sema5b	0.845	http://api.brain-map.org/grid_data/download/953?include=energy
Katnb1	0.845	http://api.brain-map.org/grid_data/download/68920731?include=energy
Cstf3	0.845	http://api.brain-map.org/grid_data/download/532600?include=energy
Chi3l3	0.845	http://api.brain-map.org/grid_data/download/69117128?include=energy
Pdzrn4	0.845	http://api.brain-map.org/grid_data/download/70300738?include=energy
Htr2a	0.845	http://api.brain-map.org/grid_data/download/81671344?include=energy
Zfp750	0.845	http://api.brain-map.org/grid_data/download/70228316?include=energy
Clcn1	0.845	http://api.brain-map.org/grid_data/download/71307250?include=energy
Per2	0.845	http://api.brain-map.org/grid_data/download/793?include=energy
Fam171a1	0.845	http://api.brain-map.org/grid_data/download/72283797?include=energy
8430408G22Rik	0.845	http://api.brain-map.org/grid_data/download/68546323?include=energy
Zfp719	0.845	http://api.brain-map.org/grid_data/download/71021028?include=energy
Crtac1	0.845	http://api.brain-map.org/grid_data/download/75749739?include=energy
Acbd5	0.845	http://api.brain-map.org/grid_data/download/70717987?include=energy
Ston2	0.845	http://api.brain-map.org/grid_data/download/70717363?include=energy
Brox	0.845	http://api.brain-map.org/grid_data/download/68203496?include=energy
Rab11fip1	0.845	http://api.brain-map.org/grid_data/download/69407744?include=energy

Rbm17	0.845	http://api.brain-map.org/grid_data/download/68744934?include=energy
Chd1l	0.845	http://api.brain-map.org/grid_data/download/69548903?include=energy
Zbtb33	0.845	http://api.brain-map.org/grid_data/download/69817097?include=energy
Tsc22d2	0.845	http://api.brain-map.org/grid_data/download/74958463?include=energy
Prkrir	0.844	http://api.brain-map.org/grid_data/download/69169773?include=energy
Hnrnpr	0.844	http://api.brain-map.org/grid_data/download/71723961?include=energy
Aifm3	0.844	http://api.brain-map.org/grid_data/download/74724774?include=energy
Exoc8	0.844	http://api.brain-map.org/grid_data/download/69262234?include=energy
Dock2	0.844	http://api.brain-map.org/grid_data/download/75988389?include=energy
Spc25	0.844	http://api.brain-map.org/grid_data/download/68148733?include=energy
4930557F10Rik	0.844	http://api.brain-map.org/grid_data/download/72076665?include=energy
Agbl2	0.844	http://api.brain-map.org/grid_data/download/75931555?include=energy
Usp7	0.844	http://api.brain-map.org/grid_data/download/70194666?include=energy
Wap	0.844	http://api.brain-map.org/grid_data/download/71656123?include=energy
Rnf165	0.844	http://api.brain-map.org/grid_data/download/70295799?include=energy
Olfr371	0.844	http://api.brain-map.org/grid_data/download/73653398?include=energy
Runx1	0.844	http://api.brain-map.org/grid_data/download/80342171?include=energy
LOC434005	0.844	http://api.brain-map.org/grid_data/download/70787209?include=energy
Ace3	0.844	http://api.brain-map.org/grid_data/download/70565969?include=energy
Prmt5	0.844	http://api.brain-map.org/grid_data/download/68161465?include=energy
Defb22	0.844	http://api.brain-map.org/grid_data/download/75197600?include=energy
Nucb1	0.844	http://api.brain-map.org/grid_data/download/69782467?include=energy
Apex1	0.844	http://api.brain-map.org/grid_data/download/69526749?include=energy
LOC432625	0.844	http://api.brain-map.org/grid_data/download/70611132?include=energy
Hook3	0.844	http://api.brain-map.org/grid_data/download/1197?include=energy
9230101K24Rik	0.844	http://api.brain-map.org/grid_data/download/70228047?include=energy
Pole4	0.844	http://api.brain-map.org/grid_data/download/75651230?include=energy
Hspg2	0.844	http://api.brain-map.org/grid_data/download/74359694?include=energy
Melk	0.844	http://api.brain-map.org/grid_data/download/69752173?include=energy
4930403N07Rik	0.844	http://api.brain-map.org/grid_data/download/74076192?include=energy
Mcee	0.844	http://api.brain-map.org/grid_data/download/68546113?include=energy
Ggcx	0.844	http://api.brain-map.org/grid_data/download/76003536?include=energy
Acss3	0.844	http://api.brain-map.org/grid_data/download/70227956?include=energy
Sh2d4b	0.844	http://api.brain-map.org/grid_data/download/69608396?include=energy
Tmprss11e	0.844	http://api.brain-map.org/grid_data/download/70228340?include=energy
March11	0.844	http://api.brain-map.org/grid_data/download/70305204?include=energy
Nlrp4c	0.844	http://api.brain-map.org/grid_data/download/70785018?include=energy
Fas	0.844	http://api.brain-map.org/grid_data/download/69981151?include=energy
Fbxo28	0.844	http://api.brain-map.org/grid_data/download/70794043?include=energy
Cnr2	0.844	http://api.brain-map.org/grid_data/download/2111?include=energy
Slamf1	0.844	http://api.brain-map.org/grid_data/download/80342050?include=energy
Atp13a1	0.844	http://api.brain-map.org/grid_data/download/70718597?include=energy
Bcl2l13	0.844	http://api.brain-map.org/grid_data/download/68444690?include=energy
Zmat5	0.844	http://api.brain-map.org/grid_data/download/68632214?include=energy
Tusc1	0.844	http://api.brain-map.org/grid_data/download/70302872?include=energy
Mrrf	0.844	http://api.brain-map.org/grid_data/download/69080514?include=energy
Tcte1	0.844	http://api.brain-map.org/grid_data/download/69980226?include=energy
Fmn2	0.844	http://api.brain-map.org/grid_data/download/72182988?include=energy
Olfr1428	0.844	http://api.brain-map.org/grid_data/download/74720817?include=energy
March7	0.844	http://api.brain-map.org/grid_data/download/79760444?include=energy
4933402D24Rik	0.844	http://api.brain-map.org/grid_data/download/70218612?include=energy
Ccdc138	0.844	http://api.brain-map.org/grid_data/download/70305078?include=energy
4933403G14Rik	0.844	http://api.brain-map.org/grid_data/download/69443118?include=energy
Pgbd1	0.844	http://api.brain-map.org/grid_data/download/73497101?include=energy
Atxn7l3b	0.844	http://api.brain-map.org/grid_data/download/75985993?include=energy
Mthfd2	0.844	http://api.brain-map.org/grid_data/download/601185?include=energy
1700097I19Rik*	0.844	http://api.brain-map.org/grid_data/download/74359562?include=energy
Gpr63	0.844	http://api.brain-map.org/grid_data/download/74277675?include=energy
Creb3l3	0.844	http://api.brain-map.org/grid_data/download/68341477?include=energy
Zfp407	0.844	http://api.brain-map.org/grid_data/download/69445361?include=energy
Pop7	0.844	http://api.brain-map.org/grid_data/download/686220?include=energy
Ly6e	0.844	http://api.brain-map.org/grid_data/download/79587728?include=energy
Ctdnep1	0.844	http://api.brain-map.org/grid_data/download/70473867?include=energy
4921507L20Rik	0.844	http://api.brain-map.org/grid_data/download/74076168?include=energy
Gmfg	0.844	http://api.brain-map.org/grid_data/download/68203468?include=energy
E130309D02Rik	0.844	http://api.brain-map.org/grid_data/download/69671762?include=energy
Mlxip	0.844	http://api.brain-map.org/grid_data/download/71574491?include=energy
Dpm1	0.844	http://api.brain-map.org/grid_data/download/69671588?include=energy
Fuca2	0.844	http://api.brain-map.org/grid_data/download/75990609?include=energy
Lrrn4	0.844	http://api.brain-map.org/grid_data/download/69527938?include=energy
Mrpl2	0.844	http://api.brain-map.org/grid_data/download/68795410?include=energy
Rftn2	0.844	http://api.brain-map.org/grid_data/download/71493135?include=energy
Nphp1	0.844	http://api.brain-map.org/grid_data/download/76003567?include=energy
Fat3	0.844	http://api.brain-map.org/grid_data/download/74047713?include=energy
Casp12	0.844	http://api.brain-map.org/grid_data/download/71656059?include=energy
D630028G08Rik	0.843	http://api.brain-map.org/grid_data/download/70946249?include=energy
Dnttip1	0.843	http://api.brain-map.org/grid_data/download/73718056?include=energy
Snx24	0.843	http://api.brain-map.org/grid_data/download/68797637?include=energy
Evc2	0.843	http://api.brain-map.org/grid_data/download/71496571?include=energy
Cd33	0.843	http://api.brain-map.org/grid_data/download/75043153?include=energy
Nusap1	0.843	http://api.brain-map.org/grid_data/download/70919501?include=energy
Pcdha11	0.843	http://api.brain-map.org/grid_data/download/79354796?include=energy
Prf1	0.843	http://api.brain-map.org/grid_data/download/76081021?include=energy
Setdb2	0.843	http://api.brain-map.org/grid_data/download/70296050?include=energy
Rprd2	0.843	http://api.brain-map.org/grid_data/download/70946186?include=energy
Mm.259863	0.843	http://api.brain-map.org/grid_data/download/71735073?include=energy
Gm239	0.843	http://api.brain-map.org/grid_data/download/71764437?include=energy
4930455F23Rik	0.843	http://api.brain-map.org/grid_data/download/68797553?include=energy
Cep170	0.843	http://api.brain-map.org/grid_data/download/77620273?include=energy
Nme7	0.843	http://api.brain-map.org/grid_data/download/69262339?include=energy
Ccdc74a	0.843	http://api.brain-map.org/grid_data/download/71212247?include=energy
Car5a	0.843	http://api.brain-map.org/grid_data/download/68446039?include=energy
Gnpda2	0.843	http://api.brain-map.org/grid_data/download/71152533?include=energy
Gm5532	0.843	http://api.brain-map.org/grid_data/download/74430924?include=energy
Irf2	0.843	http://api.brain-map.org/grid_data/download/68203461?include=energy
Agbl4	0.843	http://api.brain-map.org/grid_data/download/70218510?include=energy
D730048I06Rik	0.843	http://api.brain-map.org/grid_data/download/71656093?include=energy
Cep72	0.843	http://api.brain-map.org/grid_data/download/74988742?include=energy
S100a7a	0.843	http://api.brain-map.org/grid_data/download/71489786?include=energy
Nobox	0.843	http://api.brain-map.org/grid_data/download/69816661?include=energy
LOC382193	0.843	http://api.brain-map.org/grid_data/download/70596188?include=energy
Cirbp	0.843	http://api.brain-map.org/grid_data/download/69548958?include=energy
Slc26a1	0.843	http://api.brain-map.org/grid_data/download/78534518?include=energy
Vkorc1	0.843	http://api.brain-map.org/grid_data/download/68633152?include=energy
Med26	0.843	http://api.brain-map.org/grid_data/download/73992934?include=energy
BC049635	0.843	http://api.brain-map.org/grid_data/download/69114962?include=energy
Phf21a	0.843	http://api.brain-map.org/grid_data/download/71307190?include=energy
TC1439292	0.843	http://api.brain-map.org/grid_data/download/70795478?include=energy
Gm830	0.843	http://api.brain-map.org/grid_data/download/71764613?include=energy
Lmf1	0.843	http://api.brain-map.org/grid_data/download/583876?include=energy
Tspan8	0.843	http://api.brain-map.org/grid_data/download/71608210?include=energy
Ppp1r11	0.843	http://api.brain-map.org/grid_data/download/532738?include=energy
Rabl2	0.843	http://api.brain-map.org/grid_data/download/678693?include=energy
Nuak1	0.843	http://api.brain-map.org/grid_data/download/73497621?include=energy
Med13	0.843	http://api.brain-map.org/grid_data/download/70302479?include=energy
Krt17	0.843	http://api.brain-map.org/grid_data/download/75991022?include=energy
Steap3	0.843	http://api.brain-map.org/grid_data/download/74881275?include=energy
Lgals4	0.843	http://api.brain-map.org/grid_data/download/75931432?include=energy
Dpy30	0.843	http://api.brain-map.org/grid_data/download/68269062?include=energy
Skint7	0.843	http://api.brain-map.org/grid_data/download/70231863?include=energy
Unc13d	0.843	http://api.brain-map.org/grid_data/download/69838393?include=energy
Asxl3	0.843	http://api.brain-map.org/grid_data/download/74988269?include=energy
Gm10057	0.843	http://api.brain-map.org/grid_data/download/71891551?include=energy
Akr1c13	0.843	http://api.brain-map.org/grid_data/download/68410524?include=energy
Bhlha15	0.843	http://api.brain-map.org/grid_data/download/68301987?include=energy
D130009I18Rik	0.843	http://api.brain-map.org/grid_data/download/71021164?include=energy
Naa10	0.843	http://api.brain-map.org/grid_data/download/68633345?include=energy
Olfr1391	0.843	http://api.brain-map.org/grid_data/download/73653284?include=energy
Usp37	0.843	http://api.brain-map.org/grid_data/download/75931527?include=energy
Btbd3	0.843	http://api.brain-map.org/grid_data/download/71016618?include=energy
Fancc	0.843	http://api.brain-map.org/grid_data/download/68162253?include=energy
Lars2	0.843	http://api.brain-map.org/grid_data/download/69736046?include=energy
Slc6a8	0.843	http://api.brain-map.org/grid_data/download/69874030?include=energy
Ppp1r3b	0.843	http://api.brain-map.org/grid_data/download/75084111?include=energy
Olfr421-ps1	0.843	http://api.brain-map.org/grid_data/download/74724097?include=energy
Scamp2	0.843	http://api.brain-map.org/grid_data/download/69015805?include=energy
2310047K21Rik	0.843	http://api.brain-map.org/grid_data/download/75934921?include=energy
7120411E18Rik*	0.843	http://api.brain-map.org/grid_data/download/73906065?include=energy
Dcc	0.843	http://api.brain-map.org/grid_data/download/72472785?include=energy
Bcl2a1a	0.843	http://api.brain-map.org/grid_data/download/76080983?include=energy
Lats1	0.843	http://api.brain-map.org/grid_data/download/73514725?include=energy
Gm648	0.842	http://api.brain-map.org/grid_data/download/74430918?include=energy
LOC231914	0.842	http://api.brain-map.org/grid_data/download/68666547?include=energy
Gm1834	0.842	http://api.brain-map.org/grid_data/download/70295870?include=energy
Tchhl1	0.842	http://api.brain-map.org/grid_data/download/71489787?include=energy
Tbc1d9	0.842	http://api.brain-map.org/grid_data/download/72079987?include=energy
TC1419762	0.842	http://api.brain-map.org/grid_data/download/70795337?include=energy
LOC436548	0.842	http://api.brain-map.org/grid_data/download/74736378?include=energy
Syk	0.842	http://api.brain-map.org/grid_data/download/71280625?include=energy
Astl	0.842	http://api.brain-map.org/grid_data/download/76080981?include=energy
Gm11458	0.842	http://api.brain-map.org/grid_data/download/71022834?include=energy
Dhrsx	0.842	http://api.brain-map.org/grid_data/download/71211791?include=energy
Lrtm1	0.842	http://api.brain-map.org/grid_data/download/75931572?include=energy
Pkn3	0.842	http://api.brain-map.org/grid_data/download/67881752?include=energy
Gatad2a	0.842	http://api.brain-map.org/grid_data/download/68162200?include=energy
LOC432744	0.842	http://api.brain-map.org/grid_data/download/74579497?include=energy
2810428I15Rik	0.842	http://api.brain-map.org/grid_data/download/68744797?include=energy
Panx2	0.842	http://api.brain-map.org/grid_data/download/74581443?include=energy
Foxf2	0.842	http://api.brain-map.org/grid_data/download/72472440?include=energy
Rnf7	0.842	http://api.brain-map.org/grid_data/download/72000622?include=energy
2810043G22Rik	0.842	http://api.brain-map.org/grid_data/download/70566023?include=energy
Abi1	0.842	http://api.brain-map.org/grid_data/download/532610?include=energy
F730214C23Rik*	0.842	http://api.brain-map.org/grid_data/download/73767154?include=energy
Tcte2	0.842	http://api.brain-map.org/grid_data/download/69980232?include=energy
1700031F05Rik	0.842	http://api.brain-map.org/grid_data/download/73512568?include=energy
Stk11ip	0.842	http://api.brain-map.org/grid_data/download/68744952?include=energy
Prox2	0.842	http://api.brain-map.org/grid_data/download/69353735?include=energy
Fcgr4	0.842	http://api.brain-map.org/grid_data/download/79632202?include=energy
Cd2ap	0.842	http://api.brain-map.org/grid_data/download/69540581?include=energy
A630069G11Rik*	0.842	http://api.brain-map.org/grid_data/download/73949767?include=energy
Crisp2	0.842	http://api.brain-map.org/grid_data/download/69114268?include=energy
Sorl1	0.842	http://api.brain-map.org/grid_data/download/74511924?include=energy
Zbtb41	0.842	http://api.brain-map.org/grid_data/download/69838418?include=energy
Itsn2	0.842	http://api.brain-map.org/grid_data/download/69873714?include=energy
AF366264	0.842	http://api.brain-map.org/grid_data/download/70724553?include=energy
Cdk10	0.842	http://api.brain-map.org/grid_data/download/74735277?include=energy
Nme3	0.842	http://api.brain-map.org/grid_data/download/68744839?include=energy
Nuak2	0.842	http://api.brain-map.org/grid_data/download/71612195?include=energy
Cfi	0.842	http://api.brain-map.org/grid_data/download/69548858?include=energy
Efcab4a	0.842	http://api.brain-map.org/grid_data/download/69529023?include=energy
Amhr2	0.842	http://api.brain-map.org/grid_data/download/81654097?include=energy
Calu	0.842	http://api.brain-map.org/grid_data/download/69013426?include=energy
1600002K03Rik	0.842	http://api.brain-map.org/grid_data/download/70200961?include=energy
Setd3	0.842	http://api.brain-map.org/grid_data/download/74047910?include=energy
Ppfia4	0.842	http://api.brain-map.org/grid_data/download/69817187?include=energy
Hrh2	0.842	http://api.brain-map.org/grid_data/download/74724750?include=energy
Psd	0.842	http://api.brain-map.org/grid_data/download/72128746?include=energy
C230015M18Rik*	0.842	http://api.brain-map.org/grid_data/download/73811669?include=energy
4930412L05Rik	0.842	http://api.brain-map.org/grid_data/download/100147506?include=energy
Syt14	0.842	http://api.brain-map.org/grid_data/download/69887523?include=energy
4930547N16Rik	0.842	http://api.brain-map.org/grid_data/download/70218336?include=energy
Fiz1	0.842	http://api.brain-map.org/grid_data/download/69288974?include=energy
LOC436488	0.842	http://api.brain-map.org/grid_data/download/71022702?include=energy
Armc5	0.842	http://api.brain-map.org/grid_data/download/68499004?include=energy
Fbxw19	0.842	http://api.brain-map.org/grid_data/download/70724679?include=energy
Slc35e4	0.842	http://api.brain-map.org/grid_data/download/532730?include=energy
Muc3	0.842	http://api.brain-map.org/grid_data/download/75862443?include=energy
Cxxc4	0.842	http://api.brain-map.org/grid_data/download/74277639?include=energy
Ugcg	0.842	http://api.brain-map.org/grid_data/download/70194552?include=energy
Hectd3	0.842	http://api.brain-map.org/grid_data/download/69134432?include=energy
Chd8	0.842	http://api.brain-map.org/grid_data/download/70920099?include=energy
Ccdc123	0.842	http://api.brain-map.org/grid_data/download/69080031?include=energy
Vezt	0.842	http://api.brain-map.org/grid_data/download/74819329?include=energy
Fam48a	0.842	http://api.brain-map.org/grid_data/download/70238663?include=energy
Fgd3	0.842	http://api.brain-map.org/grid_data/download/72472800?include=energy
Naa35	0.841	http://api.brain-map.org/grid_data/download/68844349?include=energy
Clca1	0.841	http://api.brain-map.org/grid_data/download/2105?include=energy
Parp14	0.841	http://api.brain-map.org/grid_data/download/76081008?include=energy
B4galnt2	0.841	http://api.brain-map.org/grid_data/download/69672742?include=energy
1700011H14Rik	0.841	http://api.brain-map.org/grid_data/download/68499440?include=energy
Crebbp	0.841	http://api.brain-map.org/grid_data/download/69816518?include=energy
Serpinb6a	0.841	http://api.brain-map.org/grid_data/download/74425564?include=energy
Zfp866	0.841	http://api.brain-map.org/grid_data/download/70305676?include=energy
Paqr3	0.841	http://api.brain-map.org/grid_data/download/69782724?include=energy
Fate1	0.841	http://api.brain-map.org/grid_data/download/70228077?include=energy
Rad52	0.841	http://api.brain-map.org/grid_data/download/71610867?include=energy
St3gal6	0.841	http://api.brain-map.org/grid_data/download/73732154?include=energy
Abcb1a	0.841	http://api.brain-map.org/grid_data/download/78534536?include=energy
LOC432419	0.841	http://api.brain-map.org/grid_data/download/71456097?include=energy
4930519F16Rik	0.841	http://api.brain-map.org/grid_data/download/69442302?include=energy
1700074P13Rik	0.841	http://api.brain-map.org/grid_data/download/69116715?include=energy
Nmt2	0.841	http://api.brain-map.org/grid_data/download/686192?include=energy
mCG1036067	0.841	http://api.brain-map.org/grid_data/download/71022888?include=energy
Mtrf1l	0.841	http://api.brain-map.org/grid_data/download/71892346?include=energy
Glis1	0.841	http://api.brain-map.org/grid_data/download/76081016?include=energy
Mogat1	0.841	http://api.brain-map.org/grid_data/download/68666762?include=energy
Slc4a8	0.841	http://api.brain-map.org/grid_data/download/1849?include=energy
Zhx3	0.841	http://api.brain-map.org/grid_data/download/68918982?include=energy
Srsf7	0.841	http://api.brain-map.org/grid_data/download/71724684?include=energy
F930119H24Rik*	0.841	http://api.brain-map.org/grid_data/download/73767290?include=energy
Timm10	0.841	http://api.brain-map.org/grid_data/download/73521825?include=energy
Asgr1	0.841	http://api.brain-map.org/grid_data/download/2047?include=energy
Tbck	0.841	http://api.brain-map.org/grid_data/download/79567760?include=energy
Tbc1d24	0.841	http://api.brain-map.org/grid_data/download/69531049?include=energy
9930023K05Rik	0.841	http://api.brain-map.org/grid_data/download/69013420?include=energy
5830403M04Rik	0.841	http://api.brain-map.org/grid_data/download/69443535?include=energy
Nudt12	0.841	http://api.brain-map.org/grid_data/download/69014668?include=energy
LOC432968	0.841	http://api.brain-map.org/grid_data/download/71764805?include=energy
A430057O09	0.841	http://api.brain-map.org/grid_data/download/73512556?include=energy
Chd5	0.841	http://api.brain-map.org/grid_data/download/72283806?include=energy
Cpsf6	0.841	http://api.brain-map.org/grid_data/download/70300162?include=energy
Tlr1	0.841	http://api.brain-map.org/grid_data/download/70302509?include=energy
Ireb2	0.841	http://api.brain-map.org/grid_data/download/68845831?include=energy
Pcdha4	0.841	http://api.brain-map.org/grid_data/download/79354792?include=energy
Opa3	0.841	http://api.brain-map.org/grid_data/download/69609025?include=energy
Dffa	0.841	http://api.brain-map.org/grid_data/download/74819257?include=energy
Serpina3k	0.841	http://api.brain-map.org/grid_data/download/72081514?include=energy
Fcamr	0.841	http://api.brain-map.org/grid_data/download/71587649?include=energy
Plau	0.841	http://api.brain-map.org/grid_data/download/69783286?include=energy
Tmem145	0.841	http://api.brain-map.org/grid_data/download/74511987?include=energy
Med18	0.841	http://api.brain-map.org/grid_data/download/69407236?include=energy
Slc22a6	0.841	http://api.brain-map.org/grid_data/download/100144121?include=energy
Dera	0.841	http://api.brain-map.org/grid_data/download/68342234?include=energy
Krit1	0.841	http://api.brain-map.org/grid_data/download/69540495?include=energy
Slc35f5	0.841	http://api.brain-map.org/grid_data/download/77792775?include=energy
Dhx37	0.841	http://api.brain-map.org/grid_data/download/69626873?include=energy
F830115B05Rik*	0.841	http://api.brain-map.org/grid_data/download/73731412?include=energy
Sec14l4	0.841	http://api.brain-map.org/grid_data/download/68498301?include=energy
Ryk	0.841	http://api.brain-map.org/grid_data/download/2541?include=energy
Scd4	0.841	http://api.brain-map.org/grid_data/download/75863344?include=energy
Rspo4	0.841	http://api.brain-map.org/grid_data/download/69514464?include=energy
Mm.329473	0.841	http://api.brain-map.org/grid_data/download/71160265?include=energy
Fos	0.841	http://api.brain-map.org/grid_data/download/79912554?include=energy
Chrna2	0.841	http://api.brain-map.org/grid_data/download/75551460?include=energy
Ifitm1	0.841	http://api.brain-map.org/grid_data/download/77793159?include=energy
Dcun1d3	0.84	http://api.brain-map.org/grid_data/download/70430882?include=energy
Vmn1r39	0.84	http://api.brain-map.org/grid_data/download/74736498?include=energy

Cav3	0.84	http://api.brain-map.org/grid_data/download/68499332?include=energy
Cnnm1	0.84	http://api.brain-map.org/grid_data/download/73992930?include=energy
LOC544728	0.84	http://api.brain-map.org/grid_data/download/71147846?include=energy
Fgd2	0.84	http://api.brain-map.org/grid_data/download/68410279?include=energy
Rpl35	0.84	http://api.brain-map.org/grid_data/download/68441496?include=energy
Numb	0.84	http://api.brain-map.org/grid_data/download/69095946?include=energy
Cspg5	0.84	http://api.brain-map.org/grid_data/download/73931385?include=energy
Morn5	0.84	http://api.brain-map.org/grid_data/download/69116528?include=energy
Plk2	0.84	http://api.brain-map.org/grid_data/download/74743272?include=energy
Ctdp1	0.84	http://api.brain-map.org/grid_data/download/70919949?include=energy
Lins	0.84	http://api.brain-map.org/grid_data/download/76003339?include=energy
Nudt14	0.84	http://api.brain-map.org/grid_data/download/75749421?include=energy
Zic4	0.84	http://api.brain-map.org/grid_data/download/71486925?include=energy
Ihh	0.84	http://api.brain-map.org/grid_data/download/68924727?include=energy
Fgfr2	0.84	http://api.brain-map.org/grid_data/download/445?include=energy
Zfp655	0.84	http://api.brain-map.org/grid_data/download/77925743?include=energy
Ttc27	0.84	http://api.brain-map.org/grid_data/download/74819241?include=energy
C230071H18Rik	0.84	http://api.brain-map.org/grid_data/download/72283803?include=energy
Fut10	0.84	http://api.brain-map.org/grid_data/download/68162206?include=energy
A730061H03Rik	0.84	http://api.brain-map.org/grid_data/download/73811531?include=energy
Gpr55	0.84	http://api.brain-map.org/grid_data/download/79591399?include=energy
Siah1a	0.84	http://api.brain-map.org/grid_data/download/71764314?include=energy
LOC434382	0.84	http://api.brain-map.org/grid_data/download/70920153?include=energy
Tmeff1	0.84	http://api.brain-map.org/grid_data/download/1299?include=energy
4932414N04Rik	0.84	http://api.brain-map.org/grid_data/download/71892107?include=energy
Pdcd5	0.84	http://api.brain-map.org/grid_data/download/68844319?include=energy
F430010L17Rik*	0.84	http://api.brain-map.org/grid_data/download/73767362?include=energy
Lypla2	0.84	http://api.brain-map.org/grid_data/download/69736376?include=energy
Wif1	0.84	http://api.brain-map.org/grid_data/download/74511885?include=energy
LOC432408	0.84	http://api.brain-map.org/grid_data/download/74581080?include=energy
Asap2	0.84	http://api.brain-map.org/grid_data/download/74635791?include=energy
Pde9a	0.84	http://api.brain-map.org/grid_data/download/785?include=energy
Zfp710	0.84	http://api.brain-map.org/grid_data/download/71380357?include=energy
Igbp1b	0.84	http://api.brain-map.org/grid_data/download/70724846?include=energy
Olfr731	0.84	http://api.brain-map.org/grid_data/download/74734908?include=energy
Rbbp5	0.84	http://api.brain-map.org/grid_data/download/69014770?include=energy
Alg14	0.84	http://api.brain-map.org/grid_data/download/75081004?include=energy
Map3k11	0.84	http://api.brain-map.org/grid_data/download/68924721?include=energy
Ppp1r3f	0.84	http://api.brain-map.org/grid_data/download/69817229?include=energy
Sgsm2	0.84	http://api.brain-map.org/grid_data/download/69873355?include=energy
Gspt2	0.84	http://api.brain-map.org/grid_data/download/69734791?include=energy
Flt3l	0.84	http://api.brain-map.org/grid_data/download/79912546?include=energy
Csn1s2a	0.84	http://api.brain-map.org/grid_data/download/77798999?include=energy
Fstl5	0.84	http://api.brain-map.org/grid_data/download/72283812?include=energy
Ankib1	0.84	http://api.brain-map.org/grid_data/download/69288620?include=energy
1700072H12Rik	0.84	http://api.brain-map.org/grid_data/download/74357941?include=energy
Cd28	0.84	http://api.brain-map.org/grid_data/download/70528497?include=energy
Ccdc76	0.84	http://api.brain-map.org/grid_data/download/70305257?include=energy
Zfp646	0.84	http://api.brain-map.org/grid_data/download/70305132?include=energy
Atp6v0d2	0.84	http://api.brain-map.org/grid_data/download/70231046?include=energy
Chd9	0.84	http://api.brain-map.org/grid_data/download/69548915?include=energy
Pmpca	0.84	http://api.brain-map.org/grid_data/download/74357590?include=energy
LOC433494	0.839	http://api.brain-map.org/grid_data/download/74509591?include=energy
4931422A03Rik	0.839	http://api.brain-map.org/grid_data/download/74510242?include=energy
Apom	0.839	http://api.brain-map.org/grid_data/download/68410530?include=energy
Asb6	0.839	http://api.brain-map.org/grid_data/download/1599?include=energy
Nr0b2	0.839	http://api.brain-map.org/grid_data/download/77454633?include=energy
Nbr1	0.839	http://api.brain-map.org/grid_data/download/1235?include=energy
Olfr279	0.839	http://api.brain-map.org/grid_data/download/73653350?include=energy
C230011D21Rik*	0.839	http://api.brain-map.org/grid_data/download/73811657?include=energy
Ibtk	0.839	http://api.brain-map.org/grid_data/download/69289189?include=energy
Mdc1	0.839	http://api.brain-map.org/grid_data/download/71161393?include=energy
Dusp4	0.839	http://api.brain-map.org/grid_data/download/72472788?include=energy
4930548G14Rik	0.839	http://api.brain-map.org/grid_data/download/71891904?include=energy
Smarcd2	0.839	http://api.brain-map.org/grid_data/download/70539156?include=energy
Cfl1	0.839	http://api.brain-map.org/grid_data/download/70719651?include=energy
Tram1l1	0.839	http://api.brain-map.org/grid_data/download/71161024?include=energy
Nf1	0.839	http://api.brain-map.org/grid_data/download/69782210?include=energy
Zcchc6	0.839	http://api.brain-map.org/grid_data/download/68158090?include=energy
Slc25a47	0.839	http://api.brain-map.org/grid_data/download/69524485?include=energy
LOC100040019	0.839	http://api.brain-map.org/grid_data/download/74578329?include=energy
Hsd3b1	0.839	http://api.brain-map.org/grid_data/download/68667637?include=energy
TC1445412	0.839	http://api.brain-map.org/grid_data/download/70795521?include=energy
Gnaz	0.839	http://api.brain-map.org/grid_data/download/1730?include=energy
Mgl2	0.839	http://api.brain-map.org/grid_data/download/73423793?include=energy
TC1420744	0.839	http://api.brain-map.org/grid_data/download/70795343?include=energy
Cd68	0.839	http://api.brain-map.org/grid_data/download/77904937?include=energy
4930594M22Rik	0.839	http://api.brain-map.org/grid_data/download/71210265?include=energy
Rcor2	0.839	http://api.brain-map.org/grid_data/download/69261922?include=energy
Lifr	0.839	http://api.brain-map.org/grid_data/download/71016573?include=energy
Mov10l1	0.839	http://api.brain-map.org/grid_data/download/72076446?include=energy
Acox2	0.839	http://api.brain-map.org/grid_data/download/68445985?include=energy
1700062G21Rik*	0.839	http://api.brain-map.org/grid_data/download/74357935?include=energy
Mis18bp1	0.839	http://api.brain-map.org/grid_data/download/77620758?include=energy
Podn	0.839	http://api.brain-map.org/grid_data/download/75988568?include=energy
Ttll4	0.839	http://api.brain-map.org/grid_data/download/71670702?include=energy
Sar1a	0.839	http://api.brain-map.org/grid_data/download/321204?include=energy
Ppp1r3g	0.839	http://api.brain-map.org/grid_data/download/71492844?include=energy
Spata7	0.839	http://api.brain-map.org/grid_data/download/68745287?include=energy
Ccnl1	0.839	http://api.brain-map.org/grid_data/download/69134646?include=energy
Man1a2	0.839	http://api.brain-map.org/grid_data/download/68918274?include=energy
2010003K11Rik	0.839	http://api.brain-map.org/grid_data/download/68499231?include=energy
Egfem1	0.839	http://api.brain-map.org/grid_data/download/71892310?include=energy
LOC432877	0.839	http://api.brain-map.org/grid_data/download/70611359?include=energy
Letmd1	0.839	http://api.brain-map.org/grid_data/download/1635?include=energy
Fem1b	0.839	http://api.brain-map.org/grid_data/download/69288962?include=energy
D930028M14Rik	0.839	http://api.brain-map.org/grid_data/download/73512190?include=energy
Slc5a5	0.839	http://api.brain-map.org/grid_data/download/69874006?include=energy
Cdh7	0.839	http://api.brain-map.org/grid_data/download/73520973?include=energy
Sec14l1	0.839	http://api.brain-map.org/grid_data/download/74988453?include=energy
Blnk	0.839	http://api.brain-map.org/grid_data/download/74819692?include=energy
Dnajc10	0.839	http://api.brain-map.org/grid_data/download/68196955?include=energy
Dixdc1	0.839	http://api.brain-map.org/grid_data/download/70238878?include=energy
Sdcbp2	0.839	http://api.brain-map.org/grid_data/download/69080500?include=energy
Pkd2l2	0.839	http://api.brain-map.org/grid_data/download/74431278?include=energy
Wdr12	0.839	http://api.brain-map.org/grid_data/download/532601?include=energy
Pex14	0.839	http://api.brain-map.org/grid_data/download/1397?include=energy
Snn	0.839	http://api.brain-map.org/grid_data/download/70523790?include=energy
Kcnj11	0.838	http://api.brain-map.org/grid_data/download/69258035?include=energy
Elof1	0.838	http://api.brain-map.org/grid_data/download/67815555?include=energy
Parp16	0.838	http://api.brain-map.org/grid_data/download/69014872?include=energy
Pappa	0.838	http://api.brain-map.org/grid_data/download/74581444?include=energy
Vkorc1l1	0.838	http://api.brain-map.org/grid_data/download/76081920?include=energy
Pkn1	0.838	http://api.brain-map.org/grid_data/download/71656704?include=energy
1810009A15Rik	0.838	http://api.brain-map.org/grid_data/download/68845532?include=energy
Gpbp1	0.838	http://api.brain-map.org/grid_data/download/532735?include=energy
Qtrt1	0.838	http://api.brain-map.org/grid_data/download/68920821?include=energy
Erf	0.838	http://api.brain-map.org/grid_data/download/2195?include=energy
Pcdhb5	0.838	http://api.brain-map.org/grid_data/download/79354788?include=energy
Cmtm7	0.838	http://api.brain-map.org/grid_data/download/68546185?include=energy
Numbl	0.838	http://api.brain-map.org/grid_data/download/71920456?include=energy
1300018I17Rik	0.838	http://api.brain-map.org/grid_data/download/68844624?include=energy
Taf8	0.838	http://api.brain-map.org/grid_data/download/69015230?include=energy
Sdc4	0.838	http://api.brain-map.org/grid_data/download/633291?include=energy
Galnt12	0.838	http://api.brain-map.org/grid_data/download/100144528?include=energy
Elovl3	0.838	http://api.brain-map.org/grid_data/download/68302305?include=energy
Zfp109	0.838	http://api.brain-map.org/grid_data/download/69817115?include=energy
LOC433055	0.838	http://api.brain-map.org/grid_data/download/70611473?include=energy
Hbb-b1	0.838	http://api.brain-map.org/grid_data/download/67936011?include=energy
Tgif2lx1	0.838	http://api.brain-map.org/grid_data/download/76003340?include=energy
Cyp1b1	0.838	http://api.brain-map.org/grid_data/download/68667222?include=energy
Gli1	0.838	http://api.brain-map.org/grid_data/download/69289057?include=energy
Tmem18	0.838	http://api.brain-map.org/grid_data/download/68499207?include=energy
Lyzl4	0.838	http://api.brain-map.org/grid_data/download/68444750?include=energy
9630013D21Rik	0.838	http://api.brain-map.org/grid_data/download/71020993?include=energy
Fshb	0.838	http://api.brain-map.org/grid_data/download/69174427?include=energy
D10Bwg1379e	0.838	http://api.brain-map.org/grid_data/download/75988646?include=energy
Cpsf3l	0.838	http://api.brain-map.org/grid_data/download/74357611?include=energy
Tm2d2	0.838	http://api.brain-map.org/grid_data/download/68797502?include=energy
Pcdhga6	0.838	http://api.brain-map.org/grid_data/download/70784892?include=energy
Zfp428	0.838	http://api.brain-map.org/grid_data/download/71493026?include=energy
Tgm4	0.838	http://api.brain-map.org/grid_data/download/69980328?include=energy
Cldn1	0.838	http://api.brain-map.org/grid_data/download/70612723?include=energy
2610101N10Rik	0.838	http://api.brain-map.org/grid_data/download/70920075?include=energy
Xrcc6bp1	0.838	http://api.brain-map.org/grid_data/download/70474923?include=energy
Ccdc162	0.838	http://api.brain-map.org/grid_data/download/74580787?include=energy
Mrpl50	0.838	http://api.brain-map.org/grid_data/download/68445682?include=energy
Parm1	0.838	http://api.brain-map.org/grid_data/download/75749783?include=energy
Smap1	0.838	http://api.brain-map.org/grid_data/download/413824?include=energy
Mast4	0.838	http://api.brain-map.org/grid_data/download/73929897?include=energy
Olfr461	0.838	http://api.brain-map.org/grid_data/download/74724115?include=energy
Tfap2b	0.838	http://api.brain-map.org/grid_data/download/70598361?include=energy
Chrna7	0.838	http://api.brain-map.org/grid_data/download/74511738?include=energy
Fam54a	0.838	http://api.brain-map.org/grid_data/download/68445167?include=energy
Smpd2	0.838	http://api.brain-map.org/grid_data/download/68301323?include=energy
Dram1	0.838	http://api.brain-map.org/grid_data/download/68445632?include=energy
Fam174a	0.838	http://api.brain-map.org/grid_data/download/70429495?include=energy
Zfp760	0.838	http://api.brain-map.org/grid_data/download/75197582?include=energy
Tprkb	0.838	http://api.brain-map.org/grid_data/download/69549180?include=energy
Nudt6	0.838	http://api.brain-map.org/grid_data/download/67764912?include=energy
March9	0.838	http://api.brain-map.org/grid_data/download/69528136?include=energy
Mum1l1	0.838	http://api.brain-map.org/grid_data/download/74357565?include=energy
Mm.220632	0.838	http://api.brain-map.org/grid_data/download/71152491?include=energy
Cnot7	0.838	http://api.brain-map.org/grid_data/download/321203?include=energy
Tapbp	0.838	http://api.brain-map.org/grid_data/download/70919185?include=energy
Pggt1b	0.838	http://api.brain-map.org/grid_data/download/70300768?include=energy
Gm11545	0.838	http://api.brain-map.org/grid_data/download/70228418?include=energy
3110026E04Rik*	0.838	http://api.brain-map.org/grid_data/download/74359598?include=energy
Stambpl1	0.838	http://api.brain-map.org/grid_data/download/68637880?include=energy
Smpd4	0.838	http://api.brain-map.org/grid_data/download/75651226?include=energy
Magea4	0.838	http://api.brain-map.org/grid_data/download/70784624?include=energy
Fcnb	0.838	http://api.brain-map.org/grid_data/download/71587655?include=energy
Alkbh5	0.838	http://api.brain-map.org/grid_data/download/68798998?include=energy
Fam89b	0.838	http://api.brain-map.org/grid_data/download/1898?include=energy
Mm.100021	0.838	http://api.brain-map.org/grid_data/download/71147876?include=energy
4632417D23Rik	0.838	http://api.brain-map.org/grid_data/download/70303845?include=energy
Aldh3a1	0.838	http://api.brain-map.org/grid_data/download/75988265?include=energy
C1d	0.837	http://api.brain-map.org/grid_data/download/74990481?include=energy
Gfer	0.837	http://api.brain-map.org/grid_data/download/71587721?include=energy
Spag11b	0.837	http://api.brain-map.org/grid_data/download/75043677?include=energy
Lrp12	0.837	http://api.brain-map.org/grid_data/download/69549627?include=energy
Alg10b	0.837	http://api.brain-map.org/grid_data/download/72472397?include=energy
Socs2	0.837	http://api.brain-map.org/grid_data/download/74431634?include=energy
Sprr2b	0.837	http://api.brain-map.org/grid_data/download/69885708?include=energy
Nfe2	0.837	http://api.brain-map.org/grid_data/download/70593312?include=energy
Fer1l4	0.837	http://api.brain-map.org/grid_data/download/74580877?include=energy
Chodl	0.837	http://api.brain-map.org/grid_data/download/71380977?include=energy
Elk3	0.837	http://api.brain-map.org/grid_data/download/81654105?include=energy
Mrpl27	0.837	http://api.brain-map.org/grid_data/download/68744785?include=energy
Cdkn2d	0.837	http://api.brain-map.org/grid_data/download/1603?include=energy
Gabpb2	0.837	http://api.brain-map.org/grid_data/download/69672713?include=energy
Tctn2	0.837	http://api.brain-map.org/grid_data/download/68919085?include=energy
C77080	0.837	http://api.brain-map.org/grid_data/download/69531121?include=energy
Rprm	0.837	http://api.brain-map.org/grid_data/download/71836875?include=energy
Ncbp2	0.837	http://api.brain-map.org/grid_data/download/69782067?include=energy
Olfr266	0.837	http://api.brain-map.org/grid_data/download/74720934?include=energy
Pcnxl2	0.837	http://api.brain-map.org/grid_data/download/70239051?include=energy
1810027O10Rik	0.837	http://api.brain-map.org/grid_data/download/69407110?include=energy
Inhbb	0.837	http://api.brain-map.org/grid_data/download/73636087?include=energy
Aif1l	0.837	http://api.brain-map.org/grid_data/download/67815543?include=energy
Mtor	0.837	http://api.brain-map.org/grid_data/download/68845741?include=energy
Zbp1	0.837	http://api.brain-map.org/grid_data/download/68341806?include=energy
1600014C23Rik	0.837	http://api.brain-map.org/grid_data/download/70200996?include=energy
Psmb4	0.837	http://api.brain-map.org/grid_data/download/68196974?include=energy
Traf3ip1	0.837	http://api.brain-map.org/grid_data/download/74821743?include=energy
Fbxw8	0.837	http://api.brain-map.org/grid_data/download/69672378?include=energy
Cysltr1	0.837	http://api.brain-map.org/grid_data/download/75651227?include=energy
LOC382271	0.837	http://api.brain-map.org/grid_data/download/74736123?include=energy
Me3	0.837	http://api.brain-map.org/grid_data/download/69353593?include=energy
Pipox	0.837	http://api.brain-map.org/grid_data/download/68341346?include=energy
3110062M04Rik	0.837	http://api.brain-map.org/grid_data/download/70203670?include=energy
Pgc	0.837	http://api.brain-map.org/grid_data/download/70300762?include=energy
Ttc34	0.837	http://api.brain-map.org/grid_data/download/71669006?include=energy
Tmem87a	0.837	http://api.brain-map.org/grid_data/download/68444952?include=energy
Ccdc32	0.837	http://api.brain-map.org/grid_data/download/68667551?include=energy
Fam58b	0.837	http://api.brain-map.org/grid_data/download/68151450?include=energy
March4	0.837	http://api.brain-map.org/grid_data/download/73514749?include=energy
Gpr152	0.837	http://api.brain-map.org/grid_data/download/69257474?include=energy
Ppyr1	0.837	http://api.brain-map.org/grid_data/download/69258316?include=energy
Cd37	0.837	http://api.brain-map.org/grid_data/download/70528503?include=energy
Gm9946	0.837	http://api.brain-map.org/grid_data/download/70305574?include=energy
Trim37	0.837	http://api.brain-map.org/grid_data/download/74749996?include=energy
Casz1	0.837	http://api.brain-map.org/grid_data/download/70429058?include=energy
Lao1	0.837	http://api.brain-map.org/grid_data/download/68342246?include=energy
1700003E16Rik	0.837	http://api.brain-map.org/grid_data/download/70303749?include=energy
Nubp1	0.837	http://api.brain-map.org/grid_data/download/74819687?include=energy
L1td1	0.837	http://api.brain-map.org/grid_data/download/74359676?include=energy
Adprhl2	0.837	http://api.brain-map.org/grid_data/download/68920719?include=energy
4933431E20Rik	0.837	http://api.brain-map.org/grid_data/download/71569096?include=energy
Lmbrd2	0.837	http://api.brain-map.org/grid_data/download/70228274?include=energy
Pla2g12a	0.837	http://api.brain-map.org/grid_data/download/1518?include=energy
Tra2a	0.836	http://api.brain-map.org/grid_data/download/69549647?include=energy
Otol1	0.836	http://api.brain-map.org/grid_data/download/71764751?include=energy
Apobec3	0.836	http://api.brain-map.org/grid_data/download/67979041?include=energy
Txndc11	0.836	http://api.brain-map.org/grid_data/download/68637933?include=energy
Ppp1r14c	0.836	http://api.brain-map.org/grid_data/download/75831722?include=energy
Stxbp5	0.836	http://api.brain-map.org/grid_data/download/69887427?include=energy
F830222I21Rik*	0.836	http://api.brain-map.org/grid_data/download/73748749?include=energy
Vcpip1	0.836	http://api.brain-map.org/grid_data/download/70528097?include=energy
LOC433501	0.836	http://api.brain-map.org/grid_data/download/71213849?include=energy
Rpain	0.836	http://api.brain-map.org/grid_data/download/68798603?include=energy
Anapc13	0.836	http://api.brain-map.org/grid_data/download/68744773?include=energy
Mm.354411	0.836	http://api.brain-map.org/grid_data/download/71209823?include=energy
Gtpbp2	0.836	http://api.brain-map.org/grid_data/download/69734833?include=energy
Cbx3	0.836	http://api.brain-map.org/grid_data/download/68633306?include=energy
Gpr89	0.836	http://api.brain-map.org/grid_data/download/67881554?include=energy
Rhebl1	0.836	http://api.brain-map.org/grid_data/download/583845?include=energy
Uxs1	0.836	http://api.brain-map.org/grid_data/download/71763634?include=energy
Ubd	0.836	http://api.brain-map.org/grid_data/download/75828183?include=energy
Stub1	0.836	http://api.brain-map.org/grid_data/download/79912552?include=energy
Zfp207	0.836	http://api.brain-map.org/grid_data/download/68203477?include=energy
Glt6d1	0.836	http://api.brain-map.org/grid_data/download/71892065?include=energy
Trim9	0.836	http://api.brain-map.org/grid_data/download/1074?include=energy
2010001M09Rik	0.836	http://api.brain-map.org/grid_data/download/69835568?include=energy
4930488N24Rik	0.836	http://api.brain-map.org/grid_data/download/70218187?include=energy
Cd14	0.836	http://api.brain-map.org/grid_data/download/69028521?include=energy
Jak3	0.836	http://api.brain-map.org/grid_data/download/69735658?include=energy
Paox	0.836	http://api.brain-map.org/grid_data/download/75863296?include=energy
Gm14543	0.836	http://api.brain-map.org/grid_data/download/70784594?include=energy
Chrm1	0.836	http://api.brain-map.org/grid_data/download/73907497?include=energy
1700102P08Rik	0.836	http://api.brain-map.org/grid_data/download/69116793?include=energy
Rnf216	0.836	http://api.brain-map.org/grid_data/download/71280988?include=energy
Lsm1	0.836	http://api.brain-map.org/grid_data/download/74822938?include=energy
Rqcd1	0.836	http://api.brain-map.org/grid_data/download/68799107?include=energy
Muc15	0.836	http://api.brain-map.org/grid_data/download/75988378?include=energy
Parp1	0.836	http://api.brain-map.org/grid_data/download/70429154?include=energy
Herc1	0.836	http://api.brain-map.org/grid_data/download/632501?include=energy

Galnt9	0.836	http://api.brain-map.org/grid_data/download/73817422?include=energy
mCG9749.3	0.836	http://api.brain-map.org/grid_data/download/71211746?include=energy
Neto1	0.836	http://api.brain-map.org/grid_data/download/72129248?include=energy
Hyal2	0.836	http://api.brain-map.org/grid_data/download/69735186?include=energy
Ubn1	0.836	http://api.brain-map.org/grid_data/download/70303654?include=energy
Llph	0.836	http://api.brain-map.org/grid_data/download/74427076?include=energy
LOC434220	0.836	http://api.brain-map.org/grid_data/download/70787603?include=energy
Notch2	0.836	http://api.brain-map.org/grid_data/download/69837872?include=energy
Zfp317	0.836	http://api.brain-map.org/grid_data/download/70539089?include=energy
2810040C05Rik	0.836	http://api.brain-map.org/grid_data/download/75991174?include=energy
9030617O03Rik	0.836	http://api.brain-map.org/grid_data/download/70445544?include=energy
Zfp280c	0.836	http://api.brain-map.org/grid_data/download/1669?include=energy
Trim23	0.836	http://api.brain-map.org/grid_data/download/74749983?include=energy
Pth1r	0.836	http://api.brain-map.org/grid_data/download/112202195?include=energy
Nlrp6	0.836	http://api.brain-map.org/grid_data/download/69173238?include=energy
9130008F23Rik	0.836	http://api.brain-map.org/grid_data/download/100144864?include=energy
Ccdc151	0.836	http://api.brain-map.org/grid_data/download/68843955?include=energy
Abcc9	0.835	http://api.brain-map.org/grid_data/download/76003609?include=energy
Sprr2k	0.835	http://api.brain-map.org/grid_data/download/70785122?include=energy
Adi1	0.835	http://api.brain-map.org/grid_data/download/633688?include=energy
Cd300a	0.835	http://api.brain-map.org/grid_data/download/71809358?include=energy
Cd2bp2	0.835	http://api.brain-map.org/grid_data/download/80514013?include=energy
Dusp21	0.835	http://api.brain-map.org/grid_data/download/70201492?include=energy
Psmb3	0.835	http://api.brain-map.org/grid_data/download/634449?include=energy
Otc	0.835	http://api.brain-map.org/grid_data/download/74581369?include=energy
Xkr8	0.835	http://api.brain-map.org/grid_data/download/68843919?include=energy
Tex24	0.835	http://api.brain-map.org/grid_data/download/74743501?include=energy
Ccdc88a	0.835	http://api.brain-map.org/grid_data/download/71723845?include=energy
Cts8	0.835	http://api.brain-map.org/grid_data/download/76081180?include=energy
N4bp1	0.835	http://api.brain-map.org/grid_data/download/73906053?include=energy
Gpr62	0.835	http://api.brain-map.org/grid_data/download/71213189?include=energy
Adam1a	0.835	http://api.brain-map.org/grid_data/download/69837201?include=energy
Lamc1	0.835	http://api.brain-map.org/grid_data/download/77868319?include=energy
D530008I23	0.835	http://api.brain-map.org/grid_data/download/74513884?include=energy
Chrng	0.835	http://api.brain-map.org/grid_data/download/75551466?include=energy
Zfp52	0.835	http://api.brain-map.org/grid_data/download/76098326?include=energy
Pomgnt1	0.835	http://api.brain-map.org/grid_data/download/532651?include=energy
Cd3e	0.835	http://api.brain-map.org/grid_data/download/75988359?include=energy
4930471M23Rik	0.835	http://api.brain-map.org/grid_data/download/638707?include=energy
Rap2b	0.835	http://api.brain-map.org/grid_data/download/75934918?include=energy
Mm.201305	0.835	http://api.brain-map.org/grid_data/download/71152318?include=energy
Gpr81	0.835	http://api.brain-map.org/grid_data/download/77464856?include=energy
Naip6	0.835	http://api.brain-map.org/grid_data/download/70795472?include=energy
Rph3al	0.835	http://api.brain-map.org/grid_data/download/100144929?include=energy
LOC434146	0.835	http://api.brain-map.org/grid_data/download/70787459?include=energy
Tmco5	0.835	http://api.brain-map.org/grid_data/download/69114205?include=energy
Camkk2	0.835	http://api.brain-map.org/grid_data/download/70743899?include=energy
Mcpt2	0.835	http://api.brain-map.org/grid_data/download/70784636?include=energy
March3	0.835	http://api.brain-map.org/grid_data/download/71020994?include=energy
Dagla	0.835	http://api.brain-map.org/grid_data/download/71281813?include=energy
Fibin	0.835	http://api.brain-map.org/grid_data/download/68443477?include=energy
Dis3l	0.835	http://api.brain-map.org/grid_data/download/68844152?include=energy
Snx10	0.835	http://api.brain-map.org/grid_data/download/70429435?include=energy
C130026L21Rik	0.835	http://api.brain-map.org/grid_data/download/71380938?include=energy
4932411G14Rik	0.835	http://api.brain-map.org/grid_data/download/70218552?include=energy
Orai1	0.835	http://api.brain-map.org/grid_data/download/68444672?include=energy
LOC432627	0.835	http://api.brain-map.org/grid_data/download/70611138?include=energy
Zdhhc3	0.835	http://api.brain-map.org/grid_data/download/71763655?include=energy
LOC434308	0.835	http://api.brain-map.org/grid_data/download/70793869?include=energy
Whrn	0.835	http://api.brain-map.org/grid_data/download/77371813?include=energy
Alox12b	0.835	http://api.brain-map.org/grid_data/download/72128779?include=energy
A630095N17Rik	0.835	http://api.brain-map.org/grid_data/download/71494708?include=energy
Golm1	0.835	http://api.brain-map.org/grid_data/download/69080045?include=energy
Axin1	0.835	http://api.brain-map.org/grid_data/download/2053?include=energy
Rel	0.835	http://api.brain-map.org/grid_data/download/69289793?include=energy
Etv2	0.835	http://api.brain-map.org/grid_data/download/69288950?include=energy
Erbb4	0.835	http://api.brain-map.org/grid_data/download/72472797?include=energy
Cysltr2	0.835	http://api.brain-map.org/grid_data/download/69856452?include=energy
Pax7	0.834	http://api.brain-map.org/grid_data/download/766?include=energy
Ncoa5	0.834	http://api.brain-map.org/grid_data/download/79360261?include=energy
Ptpn21	0.834	http://api.brain-map.org/grid_data/download/77799015?include=energy
4930542C12Rik	0.834	http://api.brain-map.org/grid_data/download/71892167?include=energy
Mrap	0.834	http://api.brain-map.org/grid_data/download/68545663?include=energy
Tshr	0.834	http://api.brain-map.org/grid_data/download/80471957?include=energy
Cabin1	0.834	http://api.brain-map.org/grid_data/download/68861988?include=energy
Slc25a15	0.834	http://api.brain-map.org/grid_data/download/69117140?include=energy
Ppp1r15b	0.834	http://api.brain-map.org/grid_data/download/70523739?include=energy
Mrc2	0.834	http://api.brain-map.org/grid_data/download/71020779?include=energy
Glod5	0.834	http://api.brain-map.org/grid_data/download/71920297?include=energy
Rhpn1	0.834	http://api.brain-map.org/grid_data/download/68799010?include=energy
Pgls	0.834	http://api.brain-map.org/grid_data/download/70546354?include=energy
Hoxc11	0.834	http://api.brain-map.org/grid_data/download/81790682?include=energy
Cpt1b	0.834	http://api.brain-map.org/grid_data/download/68443357?include=energy
Golim4	0.834	http://api.brain-map.org/grid_data/download/71281307?include=energy
N4bp2l1	0.834	http://api.brain-map.org/grid_data/download/68638017?include=energy
2610002M06Rik	0.834	http://api.brain-map.org/grid_data/download/69080489?include=energy
Nos3	0.834	http://api.brain-map.org/grid_data/download/75831750?include=energy
9230007O13Rik*	0.834	http://api.brain-map.org/grid_data/download/73906155?include=energy
Vps72	0.834	http://api.brain-map.org/grid_data/download/80341089?include=energy
Fam70b	0.834	http://api.brain-map.org/grid_data/download/71924145?include=energy
Tssk5	0.834	http://api.brain-map.org/grid_data/download/71920141?include=energy
Gtf2e1	0.834	http://api.brain-map.org/grid_data/download/70431337?include=energy
Irf5	0.834	http://api.brain-map.org/grid_data/download/69289225?include=energy
Toe1	0.834	http://api.brain-map.org/grid_data/download/532669?include=energy
Hormad1	0.834	http://api.brain-map.org/grid_data/download/69113967?include=energy
Phf16	0.834	http://api.brain-map.org/grid_data/download/69783083?include=energy
Mtfp1	0.834	http://api.brain-map.org/grid_data/download/75774673?include=energy
Mrpl40	0.834	http://api.brain-map.org/grid_data/download/68523150?include=energy
D8Ertd738e	0.834	http://api.brain-map.org/grid_data/download/68632631?include=energy
Dmrt1	0.834	http://api.brain-map.org/grid_data/download/69288896?include=energy
Aard	0.834	http://api.brain-map.org/grid_data/download/71487007?include=energy
P2rx7	0.834	http://api.brain-map.org/grid_data/download/75551477?include=energy
Polm	0.834	http://api.brain-map.org/grid_data/download/71656135?include=energy
D030044L04Rik	0.834	http://api.brain-map.org/grid_data/download/70238058?include=energy
Adam9	0.834	http://api.brain-map.org/grid_data/download/76003388?include=energy
Fbxo5	0.834	http://api.brain-map.org/grid_data/download/68797804?include=energy
Aarsd1	0.834	http://api.brain-map.org/grid_data/download/633286?include=energy
Cwc27	0.834	http://api.brain-map.org/grid_data/download/545110?include=energy
C230036H16Rik	0.834	http://api.brain-map.org/grid_data/download/70793899?include=energy
Rpl41	0.834	http://api.brain-map.org/grid_data/download/70812660?include=energy
Cog4	0.834	http://api.brain-map.org/grid_data/download/532680?include=energy
Zmynd15	0.834	http://api.brain-map.org/grid_data/download/75043701?include=energy
A930013B10Rik	0.834	http://api.brain-map.org/grid_data/download/70228621?include=energy
Csnk2a2	0.834	http://api.brain-map.org/grid_data/download/70524283?include=energy
Dnase1l1	0.834	http://api.brain-map.org/grid_data/download/68445071?include=energy
Zfp704	0.834	http://api.brain-map.org/grid_data/download/70565599?include=energy
Cdh18	0.834	http://api.brain-map.org/grid_data/download/74658022?include=energy
Abi2	0.834	http://api.brain-map.org/grid_data/download/69514636?include=energy
Gpr45	0.834	http://api.brain-map.org/grid_data/download/69257576?include=energy
Gbp2	0.834	http://api.brain-map.org/grid_data/download/71608199?include=energy
Ctsz	0.834	http://api.brain-map.org/grid_data/download/71015377?include=energy
G430023E01Rik*	0.834	http://api.brain-map.org/grid_data/download/73716740?include=energy
Dcaf8	0.834	http://api.brain-map.org/grid_data/download/70919304?include=energy
Smek1	0.834	http://api.brain-map.org/grid_data/download/69134298?include=energy
Ipo13	0.834	http://api.brain-map.org/grid_data/download/68632178?include=energy
B230220N19Rik	0.834	http://api.brain-map.org/grid_data/download/70231140?include=energy
Gipc1	0.834	http://api.brain-map.org/grid_data/download/67755429?include=energy
1700106J16Rik	0.834	http://api.brain-map.org/grid_data/download/70201498?include=energy
3830403N18Rik	0.834	http://api.brain-map.org/grid_data/download/70609810?include=energy
St6gal1	0.834	http://api.brain-map.org/grid_data/download/74658241?include=energy
Lsamp	0.833	http://api.brain-map.org/grid_data/download/625?include=energy
Defb48	0.833	http://api.brain-map.org/grid_data/download/74514219?include=energy
Cabp2	0.833	http://api.brain-map.org/grid_data/download/70724619?include=energy
Prkx	0.833	http://api.brain-map.org/grid_data/download/70946160?include=energy
Sap30bp	0.833	http://api.brain-map.org/grid_data/download/671230?include=energy
Appbp2	0.833	http://api.brain-map.org/grid_data/download/583841?include=energy
Pigm	0.833	http://api.brain-map.org/grid_data/download/71920496?include=energy
Gm757	0.833	http://api.brain-map.org/grid_data/download/70298316?include=energy
Tarsl2	0.833	http://api.brain-map.org/grid_data/download/70430784?include=energy
Olfr1496	0.833	http://api.brain-map.org/grid_data/download/74720862?include=energy
Zfp595	0.833	http://api.brain-map.org/grid_data/download/75197546?include=energy
Rab40b	0.833	http://api.brain-map.org/grid_data/download/69817874?include=energy
Src	0.833	http://api.brain-map.org/grid_data/download/385779?include=energy
Dip2c	0.833	http://api.brain-map.org/grid_data/download/70946185?include=energy
Ascc1	0.833	http://api.brain-map.org/grid_data/download/69169821?include=energy
Gabpa	0.833	http://api.brain-map.org/grid_data/download/77455038?include=energy
Hes5	0.833	http://api.brain-map.org/grid_data/download/81657687?include=energy
C230074J23Rik*	0.833	http://api.brain-map.org/grid_data/download/73811699?include=energy
Cyp2a12	0.833	http://api.brain-map.org/grid_data/download/75828192?include=energy
Dgkh	0.833	http://api.brain-map.org/grid_data/download/73992945?include=energy
Ankrd49	0.833	http://api.brain-map.org/grid_data/download/68633273?include=energy
Mfsd5	0.833	http://api.brain-map.org/grid_data/download/392923?include=energy
Ier3ip1	0.833	http://api.brain-map.org/grid_data/download/671252?include=energy
Crtam	0.833	http://api.brain-map.org/grid_data/download/71496336?include=energy
Zfp260	0.833	http://api.brain-map.org/grid_data/download/75080996?include=energy
LOC381400	0.833	http://api.brain-map.org/grid_data/download/70596053?include=energy
Scg3	0.833	http://api.brain-map.org/grid_data/download/73718057?include=energy
Vwce	0.833	http://api.brain-map.org/grid_data/download/68920761?include=energy
Gm9734	0.833	http://api.brain-map.org/grid_data/download/70609221?include=energy
4932411K12Rik	0.833	http://api.brain-map.org/grid_data/download/70218558?include=energy
Plch2	0.833	http://api.brain-map.org/grid_data/download/74724786?include=energy
2310002L13Rik	0.833	http://api.brain-map.org/grid_data/download/72283743?include=energy
A330019P12Rik*	0.833	http://api.brain-map.org/grid_data/download/73808540?include=energy
Cnih4	0.833	http://api.brain-map.org/grid_data/download/68910997?include=energy
Plxnd1	0.833	http://api.brain-map.org/grid_data/download/73521005?include=energy
Calm4	0.833	http://api.brain-map.org/grid_data/download/68798322?include=energy
Adamtsl1	0.833	http://api.brain-map.org/grid_data/download/73811567?include=energy
Arhgap20	0.833	http://api.brain-map.org/grid_data/download/70230999?include=energy
BC024760	0.833	http://api.brain-map.org/grid_data/download/73513552?include=energy
Nlrx1	0.833	http://api.brain-map.org/grid_data/download/68667210?include=energy
Nupl2	0.833	http://api.brain-map.org/grid_data/download/68633416?include=energy
TC1560474	0.833	http://api.brain-map.org/grid_data/download/70919400?include=energy
Ptgir	0.833	http://api.brain-map.org/grid_data/download/69863249?include=energy
7120432I05Rik*	0.833	http://api.brain-map.org/grid_data/download/73906083?include=energy
Cxcl3	0.833	http://api.brain-map.org/grid_data/download/70276915?include=energy
Sowahc	0.833	http://api.brain-map.org/grid_data/download/69288512?include=energy
Cmtm1	0.833	http://api.brain-map.org/grid_data/download/70237926?include=energy
Defb41	0.833	http://api.brain-map.org/grid_data/download/71892388?include=energy
Cln6	0.833	http://api.brain-map.org/grid_data/download/69549018?include=energy
Fscn2	0.833	http://api.brain-map.org/grid_data/download/76003647?include=energy
Ccl9	0.833	http://api.brain-map.org/grid_data/download/69540464?include=energy
Nck2	0.833	http://api.brain-map.org/grid_data/download/68301634?include=energy
Omp	0.833	http://api.brain-map.org/grid_data/download/73712864?include=energy
2310039H08Rik	0.833	http://api.brain-map.org/grid_data/download/68546001?include=energy
Mm.332803	0.833	http://api.brain-map.org/grid_data/download/71160328?include=energy
Stat5b	0.833	http://api.brain-map.org/grid_data/download/71016584?include=energy
Mm.371966	0.833	http://api.brain-map.org/grid_data/download/71210037?include=energy
Ptafr	0.833	http://api.brain-map.org/grid_data/download/69863248?include=energy
3110056O03Rik	0.833	http://api.brain-map.org/grid_data/download/69407487?include=energy
A630038E17Rik	0.833	http://api.brain-map.org/grid_data/download/69170174?include=energy
Ercc3	0.833	http://api.brain-map.org/grid_data/download/67765706?include=energy
Rab33b	0.833	http://api.brain-map.org/grid_data/download/69817823?include=energy
Myb	0.833	http://api.brain-map.org/grid_data/download/1512?include=energy
Ambra1	0.833	http://api.brain-map.org/grid_data/download/70528575?include=energy
Casp4	0.833	http://api.brain-map.org/grid_data/download/75861031?include=energy
Eno1	0.833	http://api.brain-map.org/grid_data/download/74822850?include=energy
Cd96	0.833	http://api.brain-map.org/grid_data/download/68668120?include=energy
LOC436061	0.833	http://api.brain-map.org/grid_data/download/74562218?include=energy
Plac1	0.833	http://api.brain-map.org/grid_data/download/68667180?include=energy
Sec61a2	0.833	http://api.brain-map.org/grid_data/download/112643861?include=energy
Tas2r139	0.833	http://api.brain-map.org/grid_data/download/70302413?include=energy
Fbln5	0.833	http://api.brain-map.org/grid_data/download/69672294?include=energy
1700009N14Rik	0.833	http://api.brain-map.org/grid_data/download/74822594?include=energy
Lass1	0.833	http://api.brain-map.org/grid_data/download/69736052?include=energy
Alg12	0.833	http://api.brain-map.org/grid_data/download/556988?include=energy
LOC432619	0.833	http://api.brain-map.org/grid_data/download/74509566?include=energy
1810013D10Rik	0.832	http://api.brain-map.org/grid_data/download/71492910?include=energy
D19Ertd386e	0.832	http://api.brain-map.org/grid_data/download/70238639?include=energy
Zfp60	0.832	http://api.brain-map.org/grid_data/download/68631942?include=energy
Sult6b1	0.832	http://api.brain-map.org/grid_data/download/74635562?include=energy
Xpnpep1	0.832	http://api.brain-map.org/grid_data/download/70195054?include=energy
Snx32	0.832	http://api.brain-map.org/grid_data/download/71211572?include=energy
Zbtb43	0.832	http://api.brain-map.org/grid_data/download/69290854?include=energy
Cnih2	0.832	http://api.brain-map.org/grid_data/download/2109?include=energy
Adra2a	0.832	http://api.brain-map.org/grid_data/download/73615550?include=energy
Acot12	0.832	http://api.brain-map.org/grid_data/download/76081035?include=energy
Fth1	0.832	http://api.brain-map.org/grid_data/download/70795961?include=energy
Lmod1	0.832	http://api.brain-map.org/grid_data/download/70295500?include=energy
Zcrb1	0.832	http://api.brain-map.org/grid_data/download/68862150?include=energy
Bmp15	0.832	http://api.brain-map.org/grid_data/download/68667455?include=energy
Ncoa6	0.832	http://api.brain-map.org/grid_data/download/70539109?include=energy
Mex3d	0.832	http://api.brain-map.org/grid_data/download/75041562?include=energy
F830110H03Rik*	0.832	http://api.brain-map.org/grid_data/download/73731397?include=energy
Cbx6	0.832	http://api.brain-map.org/grid_data/download/73513615?include=energy
Gm5420	0.832	http://api.brain-map.org/grid_data/download/71213825?include=energy
Prdm4	0.832	http://api.brain-map.org/grid_data/download/69289751?include=energy
5830418K08Rik	0.832	http://api.brain-map.org/grid_data/download/71493777?include=energy
Fjx1	0.832	http://api.brain-map.org/grid_data/download/1203?include=energy
Helz	0.832	http://api.brain-map.org/grid_data/download/69816583?include=energy
Car6	0.832	http://api.brain-map.org/grid_data/download/75831763?include=energy
9430023L20Rik	0.832	http://api.brain-map.org/grid_data/download/69080044?include=energy
Cxcl16	0.832	http://api.brain-map.org/grid_data/download/68341794?include=energy
Robo3	0.832	http://api.brain-map.org/grid_data/download/72000623?include=energy
Tbrg1	0.832	http://api.brain-map.org/grid_data/download/71280820?include=energy
Hamp2	0.832	http://api.brain-map.org/grid_data/download/69201690?include=energy
Hrh4	0.832	http://api.brain-map.org/grid_data/download/69257767?include=energy
Gm5447	0.832	http://api.brain-map.org/grid_data/download/72182968?include=energy
Asrgl1	0.832	http://api.brain-map.org/grid_data/download/641521?include=energy
Zc3h18	0.832	http://api.brain-map.org/grid_data/download/67978512?include=energy
Zfyve26	0.832	http://api.brain-map.org/grid_data/download/71210977?include=energy
Sept9	0.832	http://api.brain-map.org/grid_data/download/69262288?include=energy
Hps1	0.832	http://api.brain-map.org/grid_data/download/69735084?include=energy
4930515G13Rik	0.832	http://api.brain-map.org/grid_data/download/70218223?include=energy
LOC436515	0.832	http://api.brain-map.org/grid_data/download/71022720?include=energy
Tapt1	0.832	http://api.brain-map.org/grid_data/download/69442467?include=energy
Trim10	0.832	http://api.brain-map.org/grid_data/download/70302818?include=energy
Shq1	0.832	http://api.brain-map.org/grid_data/download/68667527?include=energy
LOC669998	0.832	http://api.brain-map.org/grid_data/download/73949746?include=energy
Prm1	0.832	http://api.brain-map.org/grid_data/download/69174505?include=energy
Ggt5	0.832	http://api.brain-map.org/grid_data/download/69672874?include=energy
Ccdc134	0.832	http://api.brain-map.org/grid_data/download/69202472?include=energy
Cav1	0.832	http://api.brain-map.org/grid_data/download/68667952?include=energy
Fam113a	0.832	http://api.brain-map.org/grid_data/download/68910986?include=energy
Serpinb9c	0.832	http://api.brain-map.org/grid_data/download/71280613?include=energy
Aoc2	0.832	http://api.brain-map.org/grid_data/download/69526683?include=energy
Pyhin1	0.832	http://api.brain-map.org/grid_data/download/71764715?include=energy
Gfral	0.832	http://api.brain-map.org/grid_data/download/70565611?include=energy
Rgs9bp	0.832	http://api.brain-map.org/grid_data/download/71020885?include=energy
Gps2	0.832	http://api.brain-map.org/grid_data/download/68301658?include=energy
Prss21	0.832	http://api.brain-map.org/grid_data/download/71919511?include=energy
Olfr845	0.832	http://api.brain-map.org/grid_data/download/74734967?include=energy
Spats2l	0.832	http://api.brain-map.org/grid_data/download/583564?include=energy
7530416G11Rik	0.832	http://api.brain-map.org/grid_data/download/74510212?include=energy
BC024139	0.832	http://api.brain-map.org/grid_data/download/68843961?include=energy
Gm132	0.832	http://api.brain-map.org/grid_data/download/70295643?include=energy
Ctcf	0.832	http://api.brain-map.org/grid_data/download/79760450?include=energy
Kdelr2	0.832	http://api.brain-map.org/grid_data/download/79536685?include=energy

Yipf6	0.832	http://api.brain-map.org/grid_data/download/68797691?include=energy
Fam78b	0.831	http://api.brain-map.org/grid_data/download/72472750?include=energy
Gata6	0.831	http://api.brain-map.org/grid_data/download/69289033?include=energy
A230065H16Rik	0.831	http://api.brain-map.org/grid_data/download/71250292?include=energy
Ebf1	0.831	http://api.brain-map.org/grid_data/download/100142552?include=energy
Serpina5	0.831	http://api.brain-map.org/grid_data/download/69873576?include=energy
Uba7	0.831	http://api.brain-map.org/grid_data/download/70194480?include=energy
LOC226538	0.831	http://api.brain-map.org/grid_data/download/71021175?include=energy
Ptplad1	0.831	http://api.brain-map.org/grid_data/download/67765732?include=energy
Ube2a	0.831	http://api.brain-map.org/grid_data/download/68563019?include=energy
Plekha1	0.831	http://api.brain-map.org/grid_data/download/583860?include=energy
Ott	0.831	http://api.brain-map.org/grid_data/download/71234713?include=energy
Tbpl1	0.831	http://api.brain-map.org/grid_data/download/68744606?include=energy
Cxcr2	0.831	http://api.brain-map.org/grid_data/download/68667336?include=energy
4732494K09Rik*	0.831	http://api.brain-map.org/grid_data/download/73808246?include=energy
Amz2	0.831	http://api.brain-map.org/grid_data/download/67881775?include=energy
Recql	0.831	http://api.brain-map.org/grid_data/download/69015524?include=energy
LOC544683	0.831	http://api.brain-map.org/grid_data/download/73712936?include=energy
Bcl7b	0.831	http://api.brain-map.org/grid_data/download/1601?include=energy
Prdm6	0.831	http://api.brain-map.org/grid_data/download/74514085?include=energy
Dusp10	0.831	http://api.brain-map.org/grid_data/download/73771239?include=energy
Rfwd2	0.831	http://api.brain-map.org/grid_data/download/75080751?include=energy
4933413A10Rik	0.831	http://api.brain-map.org/grid_data/download/73513302?include=energy
Cd300e	0.831	http://api.brain-map.org/grid_data/download/68162270?include=energy
Krt76	0.831	http://api.brain-map.org/grid_data/download/71378666?include=energy
B4galnt3	0.831	http://api.brain-map.org/grid_data/download/73636090?include=energy
D730045B01Rik	0.831	http://api.brain-map.org/grid_data/download/73712738?include=energy
Notch1	0.831	http://api.brain-map.org/grid_data/download/70593326?include=energy
Mrgpra2b	0.831	http://api.brain-map.org/grid_data/download/68744128?include=energy
Polr2j	0.831	http://api.brain-map.org/grid_data/download/70946133?include=energy
LOC383848	0.831	http://api.brain-map.org/grid_data/download/70608364?include=energy
Ttl	0.831	http://api.brain-map.org/grid_data/download/583871?include=energy
Phf6	0.831	http://api.brain-map.org/grid_data/download/72080044?include=energy
Tmem57	0.831	http://api.brain-map.org/grid_data/download/69352890?include=energy
Ltc4s	0.831	http://api.brain-map.org/grid_data/download/68545015?include=energy
LOC434415	0.831	http://api.brain-map.org/grid_data/download/71022304?include=energy
Mcpt1	0.831	http://api.brain-map.org/grid_data/download/70784630?include=energy
Tppp2	0.831	http://api.brain-map.org/grid_data/download/70295757?include=energy
Ccdc85a	0.831	http://api.brain-map.org/grid_data/download/75081006?include=energy
Tmem239	0.831	http://api.brain-map.org/grid_data/download/69114652?include=energy
Iars2	0.831	http://api.brain-map.org/grid_data/download/74958491?include=energy
Klra8	0.831	http://api.brain-map.org/grid_data/download/68744182?include=energy
Rpph1	0.831	http://api.brain-map.org/grid_data/download/73573139?include=energy
Abpb	0.831	http://api.brain-map.org/grid_data/download/70724517?include=energy
Myst3	0.831	http://api.brain-map.org/grid_data/download/71764326?include=energy
Trib3	0.831	http://api.brain-map.org/grid_data/download/68301688?include=energy
Exoc2	0.831	http://api.brain-map.org/grid_data/download/70593344?include=energy
A830054O04Rik	0.831	http://api.brain-map.org/grid_data/download/69514386?include=energy
Rxfp2	0.831	http://api.brain-map.org/grid_data/download/73907500?include=energy
Fzd2	0.831	http://api.brain-map.org/grid_data/download/81654103?include=energy
Abcd2	0.831	http://api.brain-map.org/grid_data/download/70616004?include=energy
Slc16a11	0.831	http://api.brain-map.org/grid_data/download/69873815?include=energy
Stx2	0.831	http://api.brain-map.org/grid_data/download/68797504?include=energy
Ms4a14	0.831	http://api.brain-map.org/grid_data/download/70300079?include=energy
Hsd17b2	0.831	http://api.brain-map.org/grid_data/download/68301395?include=energy
Med23	0.831	http://api.brain-map.org/grid_data/download/112649261?include=energy
Lcn5	0.831	http://api.brain-map.org/grid_data/download/71892454?include=energy
Ogdhl	0.831	http://api.brain-map.org/grid_data/download/70725025?include=energy
Stx11	0.831	http://api.brain-map.org/grid_data/download/71020895?include=energy
Mrpl36	0.831	http://api.brain-map.org/grid_data/download/68269532?include=energy
Mettl7b	0.83	http://api.brain-map.org/grid_data/download/68666756?include=energy
Kdm5b	0.83	http://api.brain-map.org/grid_data/download/69289243?include=energy
Bmyc	0.83	http://api.brain-map.org/grid_data/download/71307196?include=energy
Trim29	0.83	http://api.brain-map.org/grid_data/download/70565351?include=energy
Fgg	0.83	http://api.brain-map.org/grid_data/download/576438?include=energy
Gm732	0.83	http://api.brain-map.org/grid_data/download/72472461?include=energy
AI480653	0.83	http://api.brain-map.org/grid_data/download/68631844?include=energy
Skint9	0.83	http://api.brain-map.org/grid_data/download/71765004?include=energy
Ttc39c	0.83	http://api.brain-map.org/grid_data/download/70743885?include=energy
Mrpl43	0.83	http://api.brain-map.org/grid_data/download/73818779?include=energy
Actl7a	0.83	http://api.brain-map.org/grid_data/download/70228699?include=energy
Ncbp1	0.83	http://api.brain-map.org/grid_data/download/70786694?include=energy
Prps1	0.83	http://api.brain-map.org/grid_data/download/69817372?include=energy
Nup85	0.83	http://api.brain-map.org/grid_data/download/69782856?include=energy
4921504E06Rik	0.83	http://api.brain-map.org/grid_data/download/71891557?include=energy
Rad54l	0.83	http://api.brain-map.org/grid_data/download/68562938?include=energy
Agbl5	0.83	http://api.brain-map.org/grid_data/download/68862096?include=energy
Slx1b	0.83	http://api.brain-map.org/grid_data/download/74819688?include=energy
Spata16	0.83	http://api.brain-map.org/grid_data/download/75931606?include=energy
Plcd1	0.83	http://api.brain-map.org/grid_data/download/1812?include=energy
Gck	0.83	http://api.brain-map.org/grid_data/download/68269269?include=energy
Sertad2	0.83	http://api.brain-map.org/grid_data/download/70524281?include=energy
Gpr124	0.83	http://api.brain-map.org/grid_data/download/69257421?include=energy
Ppp1r8	0.83	http://api.brain-map.org/grid_data/download/68160833?include=energy
Otud7b	0.83	http://api.brain-map.org/grid_data/download/71147858?include=energy
E130309D14Rik	0.83	http://api.brain-map.org/grid_data/download/74743465?include=energy
Zbtb42	0.83	http://api.brain-map.org/grid_data/download/70596200?include=energy
C78339	0.83	http://api.brain-map.org/grid_data/download/71213235?include=energy
Msln	0.83	http://api.brain-map.org/grid_data/download/75826582?include=energy
Pms2	0.83	http://api.brain-map.org/grid_data/download/69816344?include=energy
Gpr157	0.83	http://api.brain-map.org/grid_data/download/69256646?include=energy
Sox4	0.83	http://api.brain-map.org/grid_data/download/81654151?include=energy
2510039O18Rik	0.83	http://api.brain-map.org/grid_data/download/71610842?include=energy
Olfr309	0.83	http://api.brain-map.org/grid_data/download/74720973?include=energy
4932438A13Rik	0.83	http://api.brain-map.org/grid_data/download/74427107?include=energy
Lphn1	0.83	http://api.brain-map.org/grid_data/download/71211689?include=energy
Rnase11	0.83	http://api.brain-map.org/grid_data/download/74743360?include=energy
Abca1	0.83	http://api.brain-map.org/grid_data/download/69514524?include=energy
Mgat3	0.83	http://api.brain-map.org/grid_data/download/70946536?include=energy
Mex3a	0.83	http://api.brain-map.org/grid_data/download/73497759?include=energy
Gm15104	0.83	http://api.brain-map.org/grid_data/download/70595907?include=energy
5930437A14Rik	0.83	http://api.brain-map.org/grid_data/download/74562278?include=energy
Myo19	0.83	http://api.brain-map.org/grid_data/download/77620303?include=energy
F2rl2	0.83	http://api.brain-map.org/grid_data/download/75934923?include=energy
Stag1	0.83	http://api.brain-map.org/grid_data/download/69816924?include=energy
Ttc13	0.83	http://api.brain-map.org/grid_data/download/634471?include=energy
Dut	0.83	http://api.brain-map.org/grid_data/download/76081899?include=energy
Nr1h5	0.83	http://api.brain-map.org/grid_data/download/70300583?include=energy
Adrbk2	0.829	http://api.brain-map.org/grid_data/download/74724787?include=energy
Hnrnpul2	0.829	http://api.brain-map.org/grid_data/download/71232571?include=energy
Magi1	0.829	http://api.brain-map.org/grid_data/download/73519705?include=energy
Nckipsd	0.829	http://api.brain-map.org/grid_data/download/69782079?include=energy
LOC434407	0.829	http://api.brain-map.org/grid_data/download/71764389?include=energy
Wdr27	0.829	http://api.brain-map.org/grid_data/download/72471721?include=energy
Ptgfr	0.829	http://api.brain-map.org/grid_data/download/72283795?include=energy
Dmxl2	0.829	http://api.brain-map.org/grid_data/download/70238991?include=energy
AF529169	0.829	http://api.brain-map.org/grid_data/download/73636152?include=energy
Fam59a	0.829	http://api.brain-map.org/grid_data/download/70276933?include=energy
Ccdc137	0.829	http://api.brain-map.org/grid_data/download/1978?include=energy
Ptgdr	0.829	http://api.brain-map.org/grid_data/download/75233665?include=energy
Grm3	0.829	http://api.brain-map.org/grid_data/download/539?include=energy
Myh7	0.829	http://api.brain-map.org/grid_data/download/75197558?include=energy
Btd	0.829	http://api.brain-map.org/grid_data/download/2069?include=energy
Xylb	0.829	http://api.brain-map.org/grid_data/download/70195078?include=energy
2410124H12Rik	0.829	http://api.brain-map.org/grid_data/download/71991864?include=energy
Polr3g	0.829	http://api.brain-map.org/grid_data/download/68545483?include=energy
Ncln	0.829	http://api.brain-map.org/grid_data/download/576439?include=energy
Ikzf2	0.829	http://api.brain-map.org/grid_data/download/69407206?include=energy
Obox5	0.829	http://api.brain-map.org/grid_data/download/68667667?include=energy
Ccdc59	0.829	http://api.brain-map.org/grid_data/download/68522320?include=energy
B430217B02Rik	0.829	http://api.brain-map.org/grid_data/download/70231200?include=energy
Grb14	0.829	http://api.brain-map.org/grid_data/download/73997150?include=energy
1810030O07Rik	0.829	http://api.brain-map.org/grid_data/download/69442290?include=energy
Il2rb	0.829	http://api.brain-map.org/grid_data/download/68632130?include=energy
Ebf2	0.829	http://api.brain-map.org/grid_data/download/75990543?include=energy
Tbx6	0.829	http://api.brain-map.org/grid_data/download/71280631?include=energy
Scml4	0.829	http://api.brain-map.org/grid_data/download/75147763?include=energy
Mesdc2	0.829	http://api.brain-map.org/grid_data/download/72081556?include=energy
Cxcl14	0.829	http://api.brain-map.org/grid_data/download/74272041?include=energy
Zfp40	0.829	http://api.brain-map.org/grid_data/download/71488907?include=energy
Serpinb13	0.829	http://api.brain-map.org/grid_data/download/75931583?include=energy
Cd34	0.829	http://api.brain-map.org/grid_data/download/74047898?include=energy
Samd15	0.829	http://api.brain-map.org/grid_data/download/70296032?include=energy
Vrk3	0.829	http://api.brain-map.org/grid_data/download/68196941?include=energy
Usp18	0.829	http://api.brain-map.org/grid_data/download/70194618?include=energy
Slc35b3	0.829	http://api.brain-map.org/grid_data/download/69059799?include=energy
Cabp7	0.829	http://api.brain-map.org/grid_data/download/73930835?include=energy
Aen	0.829	http://api.brain-map.org/grid_data/download/70445467?include=energy
Ehf	0.829	http://api.brain-map.org/grid_data/download/79534956?include=energy
Psmf1	0.829	http://api.brain-map.org/grid_data/download/77910645?include=energy
Bpifb5	0.829	http://api.brain-map.org/grid_data/download/75826684?include=energy
Cbln4	0.829	http://api.brain-map.org/grid_data/download/72283804?include=energy
Gja5	0.829	http://api.brain-map.org/grid_data/download/68799029?include=energy
Cbr4	0.829	http://api.brain-map.org/grid_data/download/69528118?include=energy
4922502B01Rik	0.829	http://api.brain-map.org/grid_data/download/74359473?include=energy
Ttc37	0.828	http://api.brain-map.org/grid_data/download/69526480?include=energy
Tmem151a	0.828	http://api.brain-map.org/grid_data/download/69527722?include=energy
Tas2r126	0.828	http://api.brain-map.org/grid_data/download/70302366?include=energy
1520401A03Rik	0.828	http://api.brain-map.org/grid_data/download/71492838?include=energy
1700007B14Rik	0.828	http://api.brain-map.org/grid_data/download/71377906?include=energy
Cntd1	0.828	http://api.brain-map.org/grid_data/download/69080499?include=energy
Zfp943	0.828	http://api.brain-map.org/grid_data/download/69407971?include=energy
LOC435625	0.828	http://api.brain-map.org/grid_data/download/70725673?include=energy
Mll5	0.828	http://api.brain-map.org/grid_data/download/69289387?include=energy
Polg	0.828	http://api.brain-map.org/grid_data/download/73617474?include=energy
Pcdhgb2	0.828	http://api.brain-map.org/grid_data/download/70784916?include=energy
Plxdc1	0.828	http://api.brain-map.org/grid_data/download/814?include=energy
Cntn3	0.828	http://api.brain-map.org/grid_data/download/79591567?include=energy
Tmub1	0.828	http://api.brain-map.org/grid_data/download/601186?include=energy
Kcna10	0.828	http://api.brain-map.org/grid_data/download/69257993?include=energy
2610020H08Rik	0.828	http://api.brain-map.org/grid_data/download/68797733?include=energy
Olfr399	0.828	http://api.brain-map.org/grid_data/download/73653410?include=energy
Tchp	0.828	http://api.brain-map.org/grid_data/download/68861636?include=energy
Ccdc53	0.828	http://api.brain-map.org/grid_data/download/68522344?include=energy
Ctdsp1	0.828	http://api.brain-map.org/grid_data/download/73584759?include=energy
LOC436244	0.828	http://api.brain-map.org/grid_data/download/74514363?include=energy
Zbtb12	0.828	http://api.brain-map.org/grid_data/download/68798442?include=energy
Cul9	0.828	http://api.brain-map.org/grid_data/download/69782736?include=energy
9230112D13Rik	0.828	http://api.brain-map.org/grid_data/download/72081586?include=energy
Klk1	0.828	http://api.brain-map.org/grid_data/download/80342189?include=energy
Ap1m2	0.828	http://api.brain-map.org/grid_data/download/79749604?include=energy
Hyal4	0.828	http://api.brain-map.org/grid_data/download/70277985?include=energy
Bin3	0.828	http://api.brain-map.org/grid_data/download/71724470?include=energy
Tia1	0.828	http://api.brain-map.org/grid_data/download/68160841?include=energy
Cd1d2	0.828	http://api.brain-map.org/grid_data/download/69028485?include=energy
Tpr	0.828	http://api.brain-map.org/grid_data/download/72104172?include=energy
Gm5482	0.828	http://api.brain-map.org/grid_data/download/71283358?include=energy
Ppil4	0.828	http://api.brain-map.org/grid_data/download/69134481?include=energy
Lingo2	0.828	http://api.brain-map.org/grid_data/download/72128919?include=energy
Cytip	0.828	http://api.brain-map.org/grid_data/download/1270?include=energy
Zc3h3	0.828	http://api.brain-map.org/grid_data/download/70523662?include=energy
Fem1a	0.828	http://api.brain-map.org/grid_data/download/2212?include=energy
Ube2g2	0.828	http://api.brain-map.org/grid_data/download/1547?include=energy
Bcor	0.828	http://api.brain-map.org/grid_data/download/70303540?include=energy
Cmtm4	0.828	http://api.brain-map.org/grid_data/download/2101?include=energy
Dnaja4	0.828	http://api.brain-map.org/grid_data/download/70744391?include=energy
Mcoln3	0.828	http://api.brain-map.org/grid_data/download/75934924?include=energy
Olfr24	0.828	http://api.brain-map.org/grid_data/download/73653356?include=energy
Rnf24	0.828	http://api.brain-map.org/grid_data/download/71570741?include=energy
Ly86	0.828	http://api.brain-map.org/grid_data/download/69736358?include=energy
Mutyh	0.828	http://api.brain-map.org/grid_data/download/69028551?include=energy
Nphp4	0.828	http://api.brain-map.org/grid_data/download/71234655?include=energy
Plcb1	0.828	http://api.brain-map.org/grid_data/download/75084969?include=energy
Lcmt1	0.828	http://api.brain-map.org/grid_data/download/68077015?include=energy
Hace1	0.828	http://api.brain-map.org/grid_data/download/69734899?include=energy
Tc2n	0.828	http://api.brain-map.org/grid_data/download/70300433?include=energy
Tecpr2	0.828	http://api.brain-map.org/grid_data/download/70218420?include=energy
1700017D01Rik	0.828	http://api.brain-map.org/grid_data/download/71919481?include=energy
4933426M11Rik	0.828	http://api.brain-map.org/grid_data/download/76098390?include=energy
Ralgapa1	0.828	http://api.brain-map.org/grid_data/download/73496917?include=energy
Hmcn1	0.828	http://api.brain-map.org/grid_data/download/70296074?include=energy
Mfsd10	0.828	http://api.brain-map.org/grid_data/download/68196933?include=energy
Cep55	0.828	http://api.brain-map.org/grid_data/download/70474815?include=energy
Chmp5	0.828	http://api.brain-map.org/grid_data/download/413815?include=energy
Slc4a10	0.827	http://api.brain-map.org/grid_data/download/1922?include=energy
Fam83f	0.827	http://api.brain-map.org/grid_data/download/68443465?include=energy
Prmt10	0.827	http://api.brain-map.org/grid_data/download/70230879?include=energy
1700025E21Rik	0.827	http://api.brain-map.org/grid_data/download/74581319?include=energy
Lpar4	0.827	http://api.brain-map.org/grid_data/download/69257510?include=energy
Odf2l	0.827	http://api.brain-map.org/grid_data/download/68341746?include=energy
Foxb2	0.827	http://api.brain-map.org/grid_data/download/1609?include=energy
Ccdc126	0.827	http://api.brain-map.org/grid_data/download/69444819?include=energy
2510003E04Rik	0.827	http://api.brain-map.org/grid_data/download/77620796?include=energy
6330403A02Rik	0.827	http://api.brain-map.org/grid_data/download/72340183?include=energy
Hspa12b	0.827	http://api.brain-map.org/grid_data/download/70429268?include=energy
Zc3h8	0.827	http://api.brain-map.org/grid_data/download/68797852?include=energy
1810010K12Rik	0.827	http://api.brain-map.org/grid_data/download/74359317?include=energy
mCG7931.2	0.827	http://api.brain-map.org/grid_data/download/73512515?include=energy
BC032203	0.827	http://api.brain-map.org/grid_data/download/77867708?include=energy
Cyp27b1	0.827	http://api.brain-map.org/grid_data/download/69608354?include=energy
Rhox4e	0.827	http://api.brain-map.org/grid_data/download/69015296?include=energy
Gm1567	0.827	http://api.brain-map.org/grid_data/download/70298393?include=energy
Slc3a2	0.827	http://api.brain-map.org/grid_data/download/69873971?include=energy
LOC433405	0.827	http://api.brain-map.org/grid_data/download/70723223?include=energy
Pou2f3	0.827	http://api.brain-map.org/grid_data/download/74578341?include=energy
Zfp161	0.827	http://api.brain-map.org/grid_data/download/1136?include=energy
Sfswap	0.827	http://api.brain-map.org/grid_data/download/68080701?include=energy
Glcci1	0.827	http://api.brain-map.org/grid_data/download/74425568?include=energy
C330011M18Rik	0.827	http://api.brain-map.org/grid_data/download/70231935?include=energy
Ewsr1	0.827	http://api.brain-map.org/grid_data/download/408?include=energy
Fam178b	0.827	http://api.brain-map.org/grid_data/download/68667228?include=energy
Lama1	0.827	http://api.brain-map.org/grid_data/download/71381064?include=energy
Snx20	0.827	http://api.brain-map.org/grid_data/download/68545983?include=energy
Wrnip1	0.827	http://api.brain-map.org/grid_data/download/564643?include=energy
Trpc7	0.827	http://api.brain-map.org/grid_data/download/69863263?include=energy
Setdb1	0.827	http://api.brain-map.org/grid_data/download/69134640?include=energy
1700001F09Rik	0.827	http://api.brain-map.org/grid_data/download/69116733?include=energy
Bmp4	0.827	http://api.brain-map.org/grid_data/download/79556702?include=energy
2410137M14Rik	0.827	http://api.brain-map.org/grid_data/download/71991876?include=energy
Prkd3	0.827	http://api.brain-map.org/grid_data/download/71152587?include=energy
Tpbpa	0.827	http://api.brain-map.org/grid_data/download/70191985?include=energy
Npff	0.827	http://api.brain-map.org/grid_data/download/74514321?include=energy
D4Bwg0951e	0.827	http://api.brain-map.org/grid_data/download/68157410?include=energy
Wfikkn1	0.827	http://api.brain-map.org/grid_data/download/70303010?include=energy
Psmd12	0.827	http://api.brain-map.org/grid_data/download/532629?include=energy
Fndc7	0.827	http://api.brain-map.org/grid_data/download/71496522?include=energy
Epha5	0.827	http://api.brain-map.org/grid_data/download/71587625?include=energy
Prtg	0.827	http://api.brain-map.org/grid_data/download/75931594?include=energy
Mdm1	0.827	http://api.brain-map.org/grid_data/download/70303713?include=energy
Txnl4b	0.827	http://api.brain-map.org/grid_data/download/70192159?include=energy
Tigd3	0.827	http://api.brain-map.org/grid_data/download/68844749?include=energy
Mm.149980	0.827	http://api.brain-map.org/grid_data/download/71148196?include=energy
Parp6	0.827	http://api.brain-map.org/grid_data/download/68845263?include=energy
Uap1l1	0.827	http://api.brain-map.org/grid_data/download/71378582?include=energy
LOC382098	0.827	http://api.brain-map.org/grid_data/download/71489816?include=energy
Ppp1r35	0.827	http://api.brain-map.org/grid_data/download/75198196?include=energy
Cyp8b1	0.827	http://api.brain-map.org/grid_data/download/81599654?include=energy

Pex11a	0.827	http://api.brain-map.org/grid_data/download/68441472?include=energy
Cnpy2	0.827	http://api.brain-map.org/grid_data/download/70539157?include=energy
Ctxn1	0.827	http://api.brain-map.org/grid_data/download/74586669?include=energy
9230113P08Rik	0.827	http://api.brain-map.org/grid_data/download/70228065?include=energy
5730494N06Rik	0.827	http://api.brain-map.org/grid_data/download/70429130?include=energy
LOC436014	0.826	http://api.brain-map.org/grid_data/download/73584723?include=energy
Il1a	0.826	http://api.brain-map.org/grid_data/download/77923179?include=energy
Mtdh	0.826	http://api.brain-map.org/grid_data/download/71789855?include=energy
Cdkn1b	0.826	http://api.brain-map.org/grid_data/download/112204566?include=energy
Ccdc13	0.826	http://api.brain-map.org/grid_data/download/70609234?include=energy
P2ry10	0.826	http://api.brain-map.org/grid_data/download/68667742?include=energy
Sis	0.826	http://api.brain-map.org/grid_data/download/70203429?include=energy
Atp6v1c2	0.826	http://api.brain-map.org/grid_data/download/74881265?include=energy
Ccdc69	0.826	http://api.brain-map.org/grid_data/download/75988377?include=energy
Lipi	0.826	http://api.brain-map.org/grid_data/download/70238824?include=energy
Ssbp3	0.826	http://api.brain-map.org/grid_data/download/74635614?include=energy
Foxi1	0.826	http://api.brain-map.org/grid_data/download/2232?include=energy
LOC433254	0.826	http://api.brain-map.org/grid_data/download/72108822?include=energy
D430024I10Rik*	0.826	http://api.brain-map.org/grid_data/download/73834409?include=energy
Accs	0.826	http://api.brain-map.org/grid_data/download/68546088?include=energy
Cdhr1	0.826	http://api.brain-map.org/grid_data/download/72104186?include=energy
Phf5a	0.826	http://api.brain-map.org/grid_data/download/68545378?include=energy
Ap1g2	0.826	http://api.brain-map.org/grid_data/download/69526701?include=energy
LOC433079	0.826	http://api.brain-map.org/grid_data/download/70611491?include=energy
Ier5l	0.826	http://api.brain-map.org/grid_data/download/70430686?include=energy
Cyp2u1	0.826	http://api.brain-map.org/grid_data/download/71496375?include=energy
G930045G22Rik	0.826	http://api.brain-map.org/grid_data/download/73731241?include=energy
Bicd2	0.826	http://api.brain-map.org/grid_data/download/68161483?include=energy
2810453I06Rik	0.826	http://api.brain-map.org/grid_data/download/392926?include=energy
9130404H23Rik	0.826	http://api.brain-map.org/grid_data/download/71892352?include=energy
Neurl4	0.826	http://api.brain-map.org/grid_data/download/69352756?include=energy
Haus8	0.826	http://api.brain-map.org/grid_data/download/68076895?include=energy
Myod1	0.826	http://api.brain-map.org/grid_data/download/69289447?include=energy
4831426I19Rik	0.826	http://api.brain-map.org/grid_data/download/71493159?include=energy
Tirap	0.826	http://api.brain-map.org/grid_data/download/70302497?include=energy
Lrrc38	0.826	http://api.brain-map.org/grid_data/download/72340184?include=energy
Wdr48	0.826	http://api.brain-map.org/grid_data/download/69446262?include=energy
Pdzk1ip1	0.826	http://api.brain-map.org/grid_data/download/68341501?include=energy
F530213H18Rik*	0.826	http://api.brain-map.org/grid_data/download/73767400?include=energy
Cops7b	0.826	http://api.brain-map.org/grid_data/download/356493?include=energy
Depdc1a	0.826	http://api.brain-map.org/grid_data/download/68499010?include=energy
Slc39a9	0.826	http://api.brain-map.org/grid_data/download/69873965?include=energy
Wdpcp	0.826	http://api.brain-map.org/grid_data/download/69012956?include=energy
4933406M09Rik	0.826	http://api.brain-map.org/grid_data/download/71808815?include=energy
Vasn	0.826	http://api.brain-map.org/grid_data/download/68845508?include=energy
2410018L13Rik	0.826	http://api.brain-map.org/grid_data/download/68918976?include=energy
Fzd6	0.826	http://api.brain-map.org/grid_data/download/74047775?include=energy
Rdh16	0.826	http://api.brain-map.org/grid_data/download/69818060?include=energy
Slc2a6	0.826	http://api.brain-map.org/grid_data/download/71670696?include=energy
Stox1	0.826	http://api.brain-map.org/grid_data/download/69407684?include=energy
Tyw1	0.826	http://api.brain-map.org/grid_data/download/70946223?include=energy
Nup107	0.826	http://api.brain-map.org/grid_data/download/68844576?include=energy
1700028I16Rik	0.826	http://api.brain-map.org/grid_data/download/74357869?include=energy
St5	0.826	http://api.brain-map.org/grid_data/download/76135635?include=energy
Slc22a13b	0.826	http://api.brain-map.org/grid_data/download/71892394?include=energy
B530045E10Rik	0.826	http://api.brain-map.org/grid_data/download/69527962?include=energy
Uox	0.826	http://api.brain-map.org/grid_data/download/68410422?include=energy
Atp2a3	0.826	http://api.brain-map.org/grid_data/download/71608195?include=energy
Nptx2	0.826	http://api.brain-map.org/grid_data/download/74047773?include=energy
Gpn3	0.826	http://api.brain-map.org/grid_data/download/67978708?include=energy
Fam98b	0.826	http://api.brain-map.org/grid_data/download/71212446?include=energy
Agtpbp1	0.826	http://api.brain-map.org/grid_data/download/69524461?include=energy
4930548H24Rik	0.826	http://api.brain-map.org/grid_data/download/71892077?include=energy
Engase	0.826	http://api.brain-map.org/grid_data/download/69608881?include=energy
Fitm2	0.826	http://api.brain-map.org/grid_data/download/70238800?include=energy
Klhl6	0.826	http://api.brain-map.org/grid_data/download/70295428?include=energy
Ctsg	0.826	http://api.brain-map.org/grid_data/download/69608226?include=energy
Ltbp1	0.826	http://api.brain-map.org/grid_data/download/74580889?include=energy
Mon2	0.826	http://api.brain-map.org/grid_data/download/71493063?include=energy
Gm725	0.826	http://api.brain-map.org/grid_data/download/74821842?include=energy
Crb1	0.826	http://api.brain-map.org/grid_data/download/77924540?include=energy
Slit3	0.826	http://api.brain-map.org/grid_data/download/73929858?include=energy
F830305A12Rik*	0.826	http://api.brain-map.org/grid_data/download/73748788?include=energy
Gm5124	0.826	http://api.brain-map.org/grid_data/download/70595901?include=energy
Ogn	0.826	http://api.brain-map.org/grid_data/download/2446?include=energy
Ncstn	0.826	http://api.brain-map.org/grid_data/download/638705?include=energy
Eps8l3	0.826	http://api.brain-map.org/grid_data/download/404?include=energy
Zc4h2	0.825	http://api.brain-map.org/grid_data/download/70717991?include=energy
Svil	0.825	http://api.brain-map.org/grid_data/download/77332748?include=energy
LOC432754	0.825	http://api.brain-map.org/grid_data/download/70611270?include=energy
Tert	0.825	http://api.brain-map.org/grid_data/download/69290662?include=energy
Zfp202	0.825	http://api.brain-map.org/grid_data/download/68797900?include=energy
AI846148	0.825	http://api.brain-map.org/grid_data/download/71492892?include=energy
Dchs1	0.825	http://api.brain-map.org/grid_data/download/72472786?include=energy
Slc8a1	0.825	http://api.brain-map.org/grid_data/download/73635975?include=energy
Rhox11	0.825	http://api.brain-map.org/grid_data/download/69113710?include=energy
Sucnr1	0.825	http://api.brain-map.org/grid_data/download/69288351?include=energy
Noa1	0.825	http://api.brain-map.org/grid_data/download/634470?include=energy
A530098C11Rik	0.825	http://api.brain-map.org/grid_data/download/70723068?include=energy
Pds5a	0.825	http://api.brain-map.org/grid_data/download/532739?include=energy
LOC434424	0.825	http://api.brain-map.org/grid_data/download/71022316?include=energy
Mpv17l	0.825	http://api.brain-map.org/grid_data/download/70546185?include=energy
Tspan1	0.825	http://api.brain-map.org/grid_data/download/67979035?include=energy
A530026G17	0.825	http://api.brain-map.org/grid_data/download/70305293?include=energy
BC013712	0.825	http://api.brain-map.org/grid_data/download/69528130?include=energy
C330019G07Rik	0.825	http://api.brain-map.org/grid_data/download/69530995?include=energy
Pcf11	0.825	http://api.brain-map.org/grid_data/download/69134310?include=energy
Atad3a	0.825	http://api.brain-map.org/grid_data/download/68919091?include=energy
Eif4e3	0.825	http://api.brain-map.org/grid_data/download/71656063?include=energy
2310058D17Rik	0.825	http://api.brain-map.org/grid_data/download/71148211?include=energy
Csda	0.825	http://api.brain-map.org/grid_data/download/69174577?include=energy
Zfp516	0.825	http://api.brain-map.org/grid_data/download/77620451?include=energy
Syt7	0.825	http://api.brain-map.org/grid_data/download/1035?include=energy
Foxr1	0.825	http://api.brain-map.org/grid_data/download/74578365?include=energy
Nat3	0.825	http://api.brain-map.org/grid_data/download/70784708?include=energy
Ppp6c	0.825	http://api.brain-map.org/grid_data/download/75695649?include=energy
H13	0.825	http://api.brain-map.org/grid_data/download/70545955?include=energy
4931429I11Rik	0.825	http://api.brain-map.org/grid_data/download/70218486?include=energy
Mbd3l2	0.825	http://api.brain-map.org/grid_data/download/68743781?include=energy
Mm.119051	0.825	http://api.brain-map.org/grid_data/download/71147954?include=energy
LOC546096	0.825	http://api.brain-map.org/grid_data/download/75043683?include=energy
Psd4	0.825	http://api.brain-map.org/grid_data/download/69817396?include=energy
Per3	0.825	http://api.brain-map.org/grid_data/download/75084530?include=energy
LOC381754	0.825	http://api.brain-map.org/grid_data/download/70725073?include=energy
Rp2h	0.825	http://api.brain-map.org/grid_data/download/69113835?include=energy
Fgf5	0.825	http://api.brain-map.org/grid_data/download/69672438?include=energy
Clec2g	0.825	http://api.brain-map.org/grid_data/download/75197672?include=energy
Tmem95	0.825	http://api.brain-map.org/grid_data/download/70725495?include=energy
2010317E24Rik	0.825	http://api.brain-map.org/grid_data/download/73584729?include=energy
Grik3	0.825	http://api.brain-map.org/grid_data/download/75749418?include=energy
Btbd10	0.825	http://api.brain-map.org/grid_data/download/70524295?include=energy
Gm4881	0.825	http://api.brain-map.org/grid_data/download/70566112?include=energy
A730036I17Rik	0.825	http://api.brain-map.org/grid_data/download/69448960?include=energy
Car15	0.825	http://api.brain-map.org/grid_data/download/69080485?include=energy
Akt2	0.825	http://api.brain-map.org/grid_data/download/70528216?include=energy
Fut11	0.825	http://api.brain-map.org/grid_data/download/69672629?include=energy
1700015E13Rik	0.825	http://api.brain-map.org/grid_data/download/71380489?include=energy
Ddx20	0.824	http://api.brain-map.org/grid_data/download/69626729?include=energy
Grn	0.824	http://api.brain-map.org/grid_data/download/77371839?include=energy
Tecrl	0.824	http://api.brain-map.org/grid_data/download/69885738?include=energy
1300015D01Rik	0.824	http://api.brain-map.org/grid_data/download/71992116?include=energy
Spry1	0.824	http://api.brain-map.org/grid_data/download/68563074?include=energy
Cd274	0.824	http://api.brain-map.org/grid_data/download/100144558?include=energy
E130114P18Rik	0.824	http://api.brain-map.org/grid_data/download/71809455?include=energy
Defb4	0.824	http://api.brain-map.org/grid_data/download/71991726?include=energy
Katna1	0.824	http://api.brain-map.org/grid_data/download/74357608?include=energy
Cd46	0.824	http://api.brain-map.org/grid_data/download/73512566?include=energy
Lpcat3	0.824	http://api.brain-map.org/grid_data/download/100146058?include=energy
LOC433165	0.824	http://api.brain-map.org/grid_data/download/70722958?include=energy
Gm17747	0.824	http://api.brain-map.org/grid_data/download/74430906?include=energy
Nfu1	0.824	http://api.brain-map.org/grid_data/download/68076991?include=energy
Pik3cg	0.824	http://api.brain-map.org/grid_data/download/69013528?include=energy
Wfdc16	0.824	http://api.brain-map.org/grid_data/download/76081029?include=energy
Megf10	0.824	http://api.brain-map.org/grid_data/download/71249082?include=energy
A930009E05Rik	0.824	http://api.brain-map.org/grid_data/download/72340221?include=energy
Acsbg1	0.824	http://api.brain-map.org/grid_data/download/369?include=energy
Gas1	0.824	http://api.brain-map.org/grid_data/download/2251?include=energy
Nck1	0.824	http://api.brain-map.org/grid_data/download/69134589?include=energy
4933405O20Rik	0.824	http://api.brain-map.org/grid_data/download/69116859?include=energy
Fam154a	0.824	http://api.brain-map.org/grid_data/download/76080932?include=energy
Med10	0.824	http://api.brain-map.org/grid_data/download/70523705?include=energy
LOC547045	0.824	http://api.brain-map.org/grid_data/download/74736516?include=energy
Krt20	0.824	http://api.brain-map.org/grid_data/download/69015260?include=energy
Ddost	0.824	http://api.brain-map.org/grid_data/download/70920093?include=energy
Nup205	0.824	http://api.brain-map.org/grid_data/download/71234692?include=energy
Lamtor3	0.824	http://api.brain-map.org/grid_data/download/69751986?include=energy
Rbp2	0.824	http://api.brain-map.org/grid_data/download/69818042?include=energy
Spata25	0.824	http://api.brain-map.org/grid_data/download/71919607?include=energy
Asph	0.824	http://api.brain-map.org/grid_data/download/69169433?include=energy
Acp2	0.824	http://api.brain-map.org/grid_data/download/68117992?include=energy
Kcnc4	0.824	http://api.brain-map.org/grid_data/download/1756?include=energy
Slc25a16	0.824	http://api.brain-map.org/grid_data/download/70565240?include=energy
Cbln3	0.824	http://api.brain-map.org/grid_data/download/69540429?include=energy
Oxt	0.824	http://api.brain-map.org/grid_data/download/112648396?include=energy
Cmtm2b	0.824	http://api.brain-map.org/grid_data/download/72081640?include=energy
Ism1	0.824	http://api.brain-map.org/grid_data/download/71249741?include=energy
Orc4	0.824	http://api.brain-map.org/grid_data/download/70429339?include=energy
Spaca1	0.824	http://api.brain-map.org/grid_data/download/68797515?include=energy
Pcbd2	0.824	http://api.brain-map.org/grid_data/download/68523219?include=energy
6030445D17Rik	0.824	http://api.brain-map.org/grid_data/download/70227872?include=energy
Pls3	0.824	http://api.brain-map.org/grid_data/download/355886?include=energy
Zfp868	0.823	http://api.brain-map.org/grid_data/download/386029?include=energy
Capg	0.823	http://api.brain-map.org/grid_data/download/67978490?include=energy
C230029M16	0.823	http://api.brain-map.org/grid_data/download/70305532?include=energy
4932411E22Rik	0.823	http://api.brain-map.org/grid_data/download/70218546?include=energy
Memo1	0.823	http://api.brain-map.org/grid_data/download/74990492?include=energy
Alkbh1	0.823	http://api.brain-map.org/grid_data/download/71495747?include=energy
Sec22b	0.823	http://api.brain-map.org/grid_data/download/69873481?include=energy
Stxbp6	0.823	http://api.brain-map.org/grid_data/download/70744323?include=energy
4933400C05Rik	0.823	http://api.brain-map.org/grid_data/download/70218600?include=energy
Ano10	0.823	http://api.brain-map.org/grid_data/download/69526378?include=energy
Cage1	0.823	http://api.brain-map.org/grid_data/download/72472771?include=energy
Mgam	0.823	http://api.brain-map.org/grid_data/download/70300318?include=energy
4933401B06Rik	0.823	http://api.brain-map.org/grid_data/download/70724445?include=energy
Nsa2-ps1	0.823	http://api.brain-map.org/grid_data/download/70723032?include=energy
Qrich1	0.823	http://api.brain-map.org/grid_data/download/74359413?include=energy
Eya1	0.823	http://api.brain-map.org/grid_data/download/73818759?include=energy
Rapgef3	0.823	http://api.brain-map.org/grid_data/download/74635376?include=energy
Unc119	0.823	http://api.brain-map.org/grid_data/download/67978723?include=energy
Iqcf5	0.823	http://api.brain-map.org/grid_data/download/71919284?include=energy
Npas4	0.823	http://api.brain-map.org/grid_data/download/71281129?include=energy
Mm.336072	0.823	http://api.brain-map.org/grid_data/download/71160955?include=energy
Ispd	0.823	http://api.brain-map.org/grid_data/download/69442383?include=energy
Dgkg	0.823	http://api.brain-map.org/grid_data/download/343?include=energy
Icosl	0.823	http://api.brain-map.org/grid_data/download/1217?include=energy
Prkci	0.823	http://api.brain-map.org/grid_data/download/68546554?include=energy
Pitrm1	0.823	http://api.brain-map.org/grid_data/download/74273117?include=energy
Gm5079	0.823	http://api.brain-map.org/grid_data/download/71574588?include=energy
Gm14492	0.823	http://api.brain-map.org/grid_data/download/73870617?include=energy
Oaz2	0.823	http://api.brain-map.org/grid_data/download/748?include=energy
Lrfn1	0.823	http://api.brain-map.org/grid_data/download/532583?include=energy
D030072B18Rik	0.823	http://api.brain-map.org/grid_data/download/71488931?include=energy
Dap	0.823	http://api.brain-map.org/grid_data/download/79393541?include=energy
Pcdha7	0.823	http://api.brain-map.org/grid_data/download/79354810?include=energy
Gm5401	0.823	http://api.brain-map.org/grid_data/download/74736165?include=energy
Zfp449	0.823	http://api.brain-map.org/grid_data/download/76003325?include=energy
Erbb2	0.823	http://api.brain-map.org/grid_data/download/1709?include=energy
Sdccag3	0.823	http://api.brain-map.org/grid_data/download/2075?include=energy
LOC435668	0.823	http://api.brain-map.org/grid_data/download/74822605?include=energy
Art5	0.823	http://api.brain-map.org/grid_data/download/76003457?include=energy
Abhd13	0.823	http://api.brain-map.org/grid_data/download/68861686?include=energy
LOC435508	0.823	http://api.brain-map.org/grid_data/download/71022510?include=energy
Pcdhb9	0.823	http://api.brain-map.org/grid_data/download/73512187?include=energy
Hmgcr	0.823	http://api.brain-map.org/grid_data/download/75079789?include=energy
Irx1	0.823	http://api.brain-map.org/grid_data/download/2325?include=energy
F630049H13Rik*	0.823	http://api.brain-map.org/grid_data/download/73748809?include=energy
Sepn1	0.823	http://api.brain-map.org/grid_data/download/68861766?include=energy
LOC545313	0.823	http://api.brain-map.org/grid_data/download/77620822?include=energy
Kcnf1	0.823	http://api.brain-map.org/grid_data/download/71358578?include=energy
Zfp771	0.823	http://api.brain-map.org/grid_data/download/74359754?include=energy
Lrig2	0.823	http://api.brain-map.org/grid_data/download/70300210?include=energy
Mall	0.823	http://api.brain-map.org/grid_data/download/77910685?include=energy
Epha7	0.822	http://api.brain-map.org/grid_data/download/402?include=energy
1700019M22Rik	0.822	http://api.brain-map.org/grid_data/download/69113871?include=energy
Mpo	0.822	http://api.brain-map.org/grid_data/download/68667515?include=energy
Oxgr1	0.822	http://api.brain-map.org/grid_data/download/69860841?include=energy
Lrrc25	0.822	http://api.brain-map.org/grid_data/download/76003680?include=energy
Nr1h4	0.822	http://api.brain-map.org/grid_data/download/77455032?include=energy
Ldb2	0.822	http://api.brain-map.org/grid_data/download/74641311?include=energy
Igf2bp3	0.822	http://api.brain-map.org/grid_data/download/68745468?include=energy
Add3	0.822	http://api.brain-map.org/grid_data/download/68795422?include=energy
Wipf1	0.822	http://api.brain-map.org/grid_data/download/68666864?include=energy
Aqr	0.822	http://api.brain-map.org/grid_data/download/71209928?include=energy
Hoxa3	0.822	http://api.brain-map.org/grid_data/download/100145394?include=energy
Sec23ip	0.822	http://api.brain-map.org/grid_data/download/69873487?include=energy
Mrps11	0.822	http://api.brain-map.org/grid_data/download/68162218?include=energy
Svip	0.822	http://api.brain-map.org/grid_data/download/73931622?include=energy
Creb3l1	0.822	http://api.brain-map.org/grid_data/download/112197858?include=energy
LOC278757	0.822	http://api.brain-map.org/grid_data/download/70725037?include=energy
6330545A04Rik	0.822	http://api.brain-map.org/grid_data/download/74510278?include=energy
Dlgap2	0.822	http://api.brain-map.org/grid_data/download/73992720?include=energy
Lrrc50	0.822	http://api.brain-map.org/grid_data/download/69116472?include=energy
Ctbs	0.822	http://api.brain-map.org/grid_data/download/70238022?include=energy
C1ql2	0.822	http://api.brain-map.org/grid_data/download/71587929?include=energy
Noxo1	0.822	http://api.brain-map.org/grid_data/download/69080483?include=energy
Prkag1	0.822	http://api.brain-map.org/grid_data/download/69817300?include=energy
Plcb2	0.822	http://api.brain-map.org/grid_data/download/73514821?include=energy
Triml1	0.822	http://api.brain-map.org/grid_data/download/71574558?include=energy
Mtmr9	0.822	http://api.brain-map.org/grid_data/download/70795385?include=energy
Tnn	0.822	http://api.brain-map.org/grid_data/download/70302581?include=energy
Cfl2	0.822	http://api.brain-map.org/grid_data/download/74357607?include=energy
B230032P10Rik*	0.822	http://api.brain-map.org/grid_data/download/74000534?include=energy
Zfp3	0.822	http://api.brain-map.org/grid_data/download/69288602?include=energy
Zfp37	0.822	http://api.brain-map.org/grid_data/download/72182987?include=energy
Snx5	0.822	http://api.brain-map.org/grid_data/download/70813107?include=energy
Gstm6	0.822	http://api.brain-map.org/grid_data/download/68632148?include=energy
Prss38	0.822	http://api.brain-map.org/grid_data/download/71764357?include=energy
Itga11	0.822	http://api.brain-map.org/grid_data/download/74882626?include=energy
Pacsin3	0.822	http://api.brain-map.org/grid_data/download/513493?include=energy
Smndc1	0.822	http://api.brain-map.org/grid_data/download/75831712?include=energy
Ptk2	0.822	http://api.brain-map.org/grid_data/download/75774672?include=energy
Orc2	0.822	http://api.brain-map.org/grid_data/download/70429333?include=energy
Cldn21	0.822	http://api.brain-map.org/grid_data/download/70295966?include=energy
Zfp688	0.822	http://api.brain-map.org/grid_data/download/69549174?include=energy
Inadl	0.822	http://api.brain-map.org/grid_data/download/71148178?include=energy
Tex9	0.822	http://api.brain-map.org/grid_data/download/74635313?include=energy
Smc1a	0.822	http://api.brain-map.org/grid_data/download/74635114?include=energy

Gsc	0.822	http://api.brain-map.org/grid_data/download/81655566?include=energy
Wee1	0.822	http://api.brain-map.org/grid_data/download/70429471?include=energy
Jak2	0.822	http://api.brain-map.org/grid_data/download/74510182?include=energy
Trmt2b	0.822	http://api.brain-map.org/grid_data/download/71656759?include=energy
Dpys	0.821	http://api.brain-map.org/grid_data/download/76135529?include=energy
C1ra	0.821	http://api.brain-map.org/grid_data/download/70813896?include=energy
4732440D04Rik	0.821	http://api.brain-map.org/grid_data/download/71152479?include=energy
Ggn	0.821	http://api.brain-map.org/grid_data/download/100144949?include=energy
Fsd2	0.821	http://api.brain-map.org/grid_data/download/70228245?include=energy
4930449I24Rik	0.821	http://api.brain-map.org/grid_data/download/76080931?include=energy
Klhl34	0.821	http://api.brain-map.org/grid_data/download/70298172?include=energy
Arhgef16	0.821	http://api.brain-map.org/grid_data/download/76003619?include=energy
Slc26a2	0.821	http://api.brain-map.org/grid_data/download/68562944?include=energy
Galntl1	0.821	http://api.brain-map.org/grid_data/download/71717305?include=energy
Tlr12	0.821	http://api.brain-map.org/grid_data/download/69981037?include=energy
Slc22a13	0.821	http://api.brain-map.org/grid_data/download/1663?include=energy
Arl6ip4	0.821	http://api.brain-map.org/grid_data/download/69262235?include=energy
Slc39a10	0.821	http://api.brain-map.org/grid_data/download/69873947?include=energy
Satb1	0.821	http://api.brain-map.org/grid_data/download/2545?include=energy
Polr3e	0.821	http://api.brain-map.org/grid_data/download/678715?include=energy
Magix	0.821	http://api.brain-map.org/grid_data/download/74581446?include=energy
LOC380905	0.821	http://api.brain-map.org/grid_data/download/70595996?include=energy
1700008A04Rik	0.821	http://api.brain-map.org/grid_data/download/74581331?include=energy
B4galt2	0.821	http://api.brain-map.org/grid_data/download/71280715?include=energy
Gcfc1	0.821	http://api.brain-map.org/grid_data/download/71724786?include=energy
Cad	0.821	http://api.brain-map.org/grid_data/download/74635481?include=energy
Prdm15	0.821	http://api.brain-map.org/grid_data/download/73514833?include=energy
Tbccd1	0.821	http://api.brain-map.org/grid_data/download/70305060?include=energy
Tecpr1	0.821	http://api.brain-map.org/grid_data/download/68161468?include=energy
Slc25a44	0.821	http://api.brain-map.org/grid_data/download/70528288?include=energy
Nipal2	0.821	http://api.brain-map.org/grid_data/download/641522?include=energy
Ager	0.821	http://api.brain-map.org/grid_data/download/75988242?include=energy
Slc12a9	0.821	http://api.brain-map.org/grid_data/download/68924835?include=energy
A930012O16Rik	0.821	http://api.brain-map.org/grid_data/download/71809055?include=energy
Smyd3	0.821	http://api.brain-map.org/grid_data/download/69874204?include=energy
Cuzd1	0.821	http://api.brain-map.org/grid_data/download/74635192?include=energy
F630201J21Rik*	0.821	http://api.brain-map.org/grid_data/download/73748944?include=energy
Fxyd1	0.821	http://api.brain-map.org/grid_data/download/68632918?include=energy
Krt10	0.821	http://api.brain-map.org/grid_data/download/69735921?include=energy
4930503L19Rik	0.821	http://api.brain-map.org/grid_data/download/69015152?include=energy
Mpp3	0.821	http://api.brain-map.org/grid_data/download/71920520?include=energy
2210015D19Rik	0.821	http://api.brain-map.org/grid_data/download/71211513?include=energy
F830221E22Rik*	0.821	http://api.brain-map.org/grid_data/download/77924366?include=energy
R74862	0.821	http://api.brain-map.org/grid_data/download/67936009?include=energy
Mlf1	0.821	http://api.brain-map.org/grid_data/download/68744904?include=energy
Map2k2	0.821	http://api.brain-map.org/grid_data/download/73514743?include=energy
Ugt2a3	0.821	http://api.brain-map.org/grid_data/download/68499165?include=energy
LOC434398	0.821	http://api.brain-map.org/grid_data/download/74510098?include=energy
6430548G04	0.821	http://api.brain-map.org/grid_data/download/71489794?include=energy
Olfm2	0.821	http://api.brain-map.org/grid_data/download/71717155?include=energy
Clec16a	0.821	http://api.brain-map.org/grid_data/download/74580739?include=energy
4930532C19Rik*	0.821	http://api.brain-map.org/grid_data/download/73870719?include=energy
Entpd1	0.821	http://api.brain-map.org/grid_data/download/393?include=energy
Il34	0.821	http://api.brain-map.org/grid_data/download/72007750?include=energy
Pecam1	0.821	http://api.brain-map.org/grid_data/download/68443238?include=energy
Tyms	0.821	http://api.brain-map.org/grid_data/download/75990848?include=energy
Tfdp1	0.82	http://api.brain-map.org/grid_data/download/70919161?include=energy
Tmem170	0.82	http://api.brain-map.org/grid_data/download/69446328?include=energy
Zfp287	0.82	http://api.brain-map.org/grid_data/download/69290848?include=energy
Kctd6	0.82	http://api.brain-map.org/grid_data/download/74357550?include=energy
Sema6b	0.82	http://api.brain-map.org/grid_data/download/72001866?include=energy
Ppp2r3c	0.82	http://api.brain-map.org/grid_data/download/71656060?include=energy
Cox8c	0.82	http://api.brain-map.org/grid_data/download/74581325?include=energy
Dph2	0.82	http://api.brain-map.org/grid_data/download/2161?include=energy
Adam26a	0.82	http://api.brain-map.org/grid_data/download/70230771?include=energy
Bmpr1b	0.82	http://api.brain-map.org/grid_data/download/69529376?include=energy
6430527G18Rik	0.82	http://api.brain-map.org/grid_data/download/69444927?include=energy
1700020I14Rik	0.82	http://api.brain-map.org/grid_data/download/71380495?include=energy
Rnpc3	0.82	http://api.brain-map.org/grid_data/download/583856?include=energy
Cyp4a14	0.82	http://api.brain-map.org/grid_data/download/71580150?include=energy
St6galnac6	0.82	http://api.brain-map.org/grid_data/download/74047902?include=energy
6030452D12Rik	0.82	http://api.brain-map.org/grid_data/download/70227884?include=energy
4930432K21Rik	0.82	http://api.brain-map.org/grid_data/download/69407965?include=energy
4833423E24Rik	0.82	http://api.brain-map.org/grid_data/download/70205584?include=energy
Fbxw14	0.82	http://api.brain-map.org/grid_data/download/70724720?include=energy
Amigo1	0.82	http://api.brain-map.org/grid_data/download/77932819?include=energy
Hils1	0.82	http://api.brain-map.org/grid_data/download/69174487?include=energy
Syncrip	0.82	http://api.brain-map.org/grid_data/download/1391?include=energy
Zfp770	0.82	http://api.brain-map.org/grid_data/download/69288548?include=energy
Fgb	0.82	http://api.brain-map.org/grid_data/download/68638095?include=energy
Wnt8b	0.82	http://api.brain-map.org/grid_data/download/1890?include=energy
Mrpl15	0.82	http://api.brain-map.org/grid_data/download/70918778?include=energy
9430088L24Rik	0.82	http://api.brain-map.org/grid_data/download/71494635?include=energy
Setd1a	0.82	http://api.brain-map.org/grid_data/download/69258214?include=energy
9530056K15Rik	0.82	http://api.brain-map.org/grid_data/download/71494647?include=energy
Ormdl3	0.82	http://api.brain-map.org/grid_data/download/68745492?include=energy
Prss51	0.82	http://api.brain-map.org/grid_data/download/74581313?include=energy
Zfp414	0.82	http://api.brain-map.org/grid_data/download/68920701?include=energy
Apaf1	0.82	http://api.brain-map.org/grid_data/download/68745275?include=energy
Gpr21	0.82	http://api.brain-map.org/grid_data/download/69857607?include=energy
Atm	0.82	http://api.brain-map.org/grid_data/download/1329?include=energy
Acap3	0.82	http://api.brain-map.org/grid_data/download/70593277?include=energy
Fads1	0.82	http://api.brain-map.org/grid_data/download/678684?include=energy
Pde1c	0.82	http://api.brain-map.org/grid_data/download/1804?include=energy
Mup-ps15	0.82	http://api.brain-map.org/grid_data/download/70725127?include=energy
AY702102	0.82	http://api.brain-map.org/grid_data/download/74822862?include=energy
Rims1	0.82	http://api.brain-map.org/grid_data/download/73514899?include=energy
Ccdc77	0.82	http://api.brain-map.org/grid_data/download/68637485?include=energy
Aox4	0.82	http://api.brain-map.org/grid_data/download/69526695?include=energy
Olfr1500	0.82	http://api.brain-map.org/grid_data/download/74720874?include=energy
Gm1650	0.82	http://api.brain-map.org/grid_data/download/70298585?include=energy
LOC433258	0.82	http://api.brain-map.org/grid_data/download/73931636?include=energy
Srprb	0.82	http://api.brain-map.org/grid_data/download/1536?include=energy
Fam181b	0.82	http://api.brain-map.org/grid_data/download/68861760?include=energy
Thap2	0.82	http://api.brain-map.org/grid_data/download/68744791?include=energy
Myom2	0.82	http://api.brain-map.org/grid_data/download/71234528?include=energy
Reg3g	0.82	http://api.brain-map.org/grid_data/download/69117116?include=energy
Inpp5e	0.82	http://api.brain-map.org/grid_data/download/69735467?include=energy
mCG132591	0.82	http://api.brain-map.org/grid_data/download/71212048?include=energy
Sh3bp5l	0.82	http://api.brain-map.org/grid_data/download/75695651?include=energy
Odam	0.819	http://api.brain-map.org/grid_data/download/74635370?include=energy
Polr2a	0.819	http://api.brain-map.org/grid_data/download/69816685?include=energy
Fut8	0.819	http://api.brain-map.org/grid_data/download/72007749?include=energy
Kif27	0.819	http://api.brain-map.org/grid_data/download/70295416?include=energy
Epb4.1l2	0.819	http://api.brain-map.org/grid_data/download/70431433?include=energy
0610031J06Rik	0.819	http://api.brain-map.org/grid_data/download/68342348?include=energy
Bak1	0.819	http://api.brain-map.org/grid_data/download/75831757?include=energy
Krt12	0.819	http://api.brain-map.org/grid_data/download/69735927?include=energy
Astn2	0.819	http://api.brain-map.org/grid_data/download/74047748?include=energy
Rax	0.819	http://api.brain-map.org/grid_data/download/1821?include=energy
Scamp4	0.819	http://api.brain-map.org/grid_data/download/68269749?include=energy
Tmx1	0.819	http://api.brain-map.org/grid_data/download/68795409?include=energy
Trf	0.819	http://api.brain-map.org/grid_data/download/68795444?include=energy
2310022A10Rik	0.819	http://api.brain-map.org/grid_data/download/68080690?include=energy
Slc44a4	0.819	http://api.brain-map.org/grid_data/download/68321886?include=energy
Pik3r5	0.819	http://api.brain-map.org/grid_data/download/75988403?include=energy
Smagp	0.819	http://api.brain-map.org/grid_data/download/70528312?include=energy
Dtymk	0.819	http://api.brain-map.org/grid_data/download/75990559?include=energy
Kcnh5	0.819	http://api.brain-map.org/grid_data/download/77620826?include=energy
Cisd2	0.819	http://api.brain-map.org/grid_data/download/68745030?include=energy
Slitrk4	0.819	http://api.brain-map.org/grid_data/download/74882938?include=energy
2810432D09Rik	0.819	http://api.brain-map.org/grid_data/download/68845538?include=energy
Naa16	0.819	http://api.brain-map.org/grid_data/download/75828195?include=energy
Rapgef1	0.819	http://api.brain-map.org/grid_data/download/69817946?include=energy
Fgf2	0.819	http://api.brain-map.org/grid_data/download/100145369?include=energy
Trim61	0.819	http://api.brain-map.org/grid_data/download/68910988?include=energy
Prss16	0.819	http://api.brain-map.org/grid_data/download/75931615?include=energy
E230025N22Rik	0.819	http://api.brain-map.org/grid_data/download/71587619?include=energy
Olfr95	0.819	http://api.brain-map.org/grid_data/download/74734997?include=energy
Pcdh1	0.819	http://api.brain-map.org/grid_data/download/75934527?include=energy
Zfp72	0.819	http://api.brain-map.org/grid_data/download/70785738?include=energy
Mcm6	0.819	http://api.brain-map.org/grid_data/download/71358638?include=energy
Slc30a4	0.819	http://api.brain-map.org/grid_data/download/71280652?include=energy
Fbxl19	0.819	http://api.brain-map.org/grid_data/download/68845203?include=energy
Mnx1	0.819	http://api.brain-map.org/grid_data/download/100145354?include=energy
Zbed3	0.819	http://api.brain-map.org/grid_data/download/72076599?include=energy
Pih1d1	0.819	http://api.brain-map.org/grid_data/download/73606582?include=energy
Zfp397	0.819	http://api.brain-map.org/grid_data/download/68445913?include=energy
Zfp295	0.819	http://api.brain-map.org/grid_data/download/71613645?include=energy
1700108F19Rik	0.819	http://api.brain-map.org/grid_data/download/71919601?include=energy
Rrp1b	0.819	http://api.brain-map.org/grid_data/download/71022924?include=energy
Gm10283	0.819	http://api.brain-map.org/grid_data/download/71808680?include=energy
Scarf2	0.819	http://api.brain-map.org/grid_data/download/74514357?include=energy
Ahdc1	0.819	http://api.brain-map.org/grid_data/download/74357718?include=energy
Rrm2b	0.819	http://api.brain-map.org/grid_data/download/68919818?include=energy
Shroom2	0.819	http://api.brain-map.org/grid_data/download/69526773?include=energy
Rala	0.819	http://api.brain-map.org/grid_data/download/67765730?include=energy
Gm5475	0.819	http://api.brain-map.org/grid_data/download/74578347?include=energy
Vnn1	0.819	http://api.brain-map.org/grid_data/download/68445979?include=energy
Avpr1b	0.819	http://api.brain-map.org/grid_data/download/79488955?include=energy
BC048502	0.819	http://api.brain-map.org/grid_data/download/69113883?include=energy
Gas2l3	0.819	http://api.brain-map.org/grid_data/download/69672802?include=energy
Sphk2	0.818	http://api.brain-map.org/grid_data/download/69079997?include=energy
Dvl3	0.818	http://api.brain-map.org/grid_data/download/70724673?include=energy
Cyba	0.818	http://api.brain-map.org/grid_data/download/75828191?include=energy
1190007F08Rik	0.818	http://api.brain-map.org/grid_data/download/70946184?include=energy
Adck3	0.818	http://api.brain-map.org/grid_data/download/67881751?include=energy
Cth	0.818	http://api.brain-map.org/grid_data/download/75042264?include=energy
Trpv6	0.818	http://api.brain-map.org/grid_data/download/100144410?include=energy
A530046M15	0.818	http://api.brain-map.org/grid_data/download/71569181?include=energy
Man1c1	0.818	http://api.brain-map.org/grid_data/download/69736448?include=energy
Olfr985	0.818	http://api.brain-map.org/grid_data/download/74277400?include=energy
Disc1	0.818	http://api.brain-map.org/grid_data/download/79394158?include=energy
Rpe	0.818	http://api.brain-map.org/grid_data/download/545112?include=energy
Car5b	0.818	http://api.brain-map.org/grid_data/download/68545360?include=energy
Tmem43	0.818	http://api.brain-map.org/grid_data/download/70539180?include=energy
Nbl1	0.818	http://api.brain-map.org/grid_data/download/69782061?include=energy
Adam21	0.818	http://api.brain-map.org/grid_data/download/70228717?include=energy
B230304F11Rik*	0.818	http://api.brain-map.org/grid_data/download/74000540?include=energy
A530053M12Rik	0.818	http://api.brain-map.org/grid_data/download/74277717?include=energy
Srsf10	0.818	http://api.brain-map.org/grid_data/download/69059861?include=energy
Tmem116	0.818	http://api.brain-map.org/grid_data/download/68446021?include=energy
Smurf2	0.818	http://api.brain-map.org/grid_data/download/69874198?include=energy
Rab3ip	0.818	http://api.brain-map.org/grid_data/download/71717136?include=energy
Gm826	0.818	http://api.brain-map.org/grid_data/download/70298322?include=energy
Xrcc1	0.818	http://api.brain-map.org/grid_data/download/69014746?include=energy
Diap3	0.818	http://api.brain-map.org/grid_data/download/70429226?include=energy
TC1447552	0.818	http://api.brain-map.org/grid_data/download/74578248?include=energy
A830039N20Rik	0.818	http://api.brain-map.org/grid_data/download/72340208?include=energy
Eif2c1	0.818	http://api.brain-map.org/grid_data/download/70724702?include=energy
Lenep	0.818	http://api.brain-map.org/grid_data/download/633287?include=energy
BC017158	0.818	http://api.brain-map.org/grid_data/download/70445616?include=energy
LOC436508	0.818	http://api.brain-map.org/grid_data/download/70300499?include=energy
Rbbp8	0.818	http://api.brain-map.org/grid_data/download/68666822?include=energy
Myf6	0.818	http://api.brain-map.org/grid_data/download/69289435?include=energy
Smyd4	0.818	http://api.brain-map.org/grid_data/download/70302216?include=energy
Cenph	0.818	http://api.brain-map.org/grid_data/download/68522332?include=energy
Serping1	0.818	http://api.brain-map.org/grid_data/download/68076550?include=energy
LOC432674	0.818	http://api.brain-map.org/grid_data/download/70611186?include=energy
LOC432644	0.818	http://api.brain-map.org/grid_data/download/74510230?include=energy
Nr5a2	0.818	http://api.brain-map.org/grid_data/download/74822611?include=energy
F830001M15Rik*	0.818	http://api.brain-map.org/grid_data/download/73731475?include=energy
4930419B13Rik*	0.818	http://api.brain-map.org/grid_data/download/73808213?include=energy
Ddx25	0.818	http://api.brain-map.org/grid_data/download/68157390?include=energy
Nipbl	0.818	http://api.brain-map.org/grid_data/download/69133773?include=energy
Tnfsf15	0.818	http://api.brain-map.org/grid_data/download/74056143?include=energy
Arl8b	0.818	http://api.brain-map.org/grid_data/download/68193050?include=energy
9130019P16Rik	0.818	http://api.brain-map.org/grid_data/download/70228022?include=energy
Dnase2a	0.818	http://api.brain-map.org/grid_data/download/68745066?include=energy
Fahd1	0.818	http://api.brain-map.org/grid_data/download/67814215?include=energy
Ears2	0.818	http://api.brain-map.org/grid_data/download/68151344?include=energy
Fggy	0.818	http://api.brain-map.org/grid_data/download/69549278?include=energy
Fkbpl	0.818	http://api.brain-map.org/grid_data/download/69012861?include=energy
Esf1	0.817	http://api.brain-map.org/grid_data/download/72283378?include=energy
Vsig2	0.817	http://api.brain-map.org/grid_data/download/75214944?include=energy
Tmem135	0.817	http://api.brain-map.org/grid_data/download/532699?include=energy
S100a5	0.817	http://api.brain-map.org/grid_data/download/69873379?include=energy
Galr3	0.817	http://api.brain-map.org/grid_data/download/100146166?include=energy
Pak1	0.817	http://api.brain-map.org/grid_data/download/73521001?include=energy
Cdc42se2	0.817	http://api.brain-map.org/grid_data/download/70527981?include=energy
Kcnk18	0.817	http://api.brain-map.org/grid_data/download/100144867?include=energy
Mypop	0.817	http://api.brain-map.org/grid_data/download/73423887?include=energy
Ppargc1b	0.817	http://api.brain-map.org/grid_data/download/71325354?include=energy
Gdf9	0.817	http://api.brain-map.org/grid_data/download/68667360?include=energy
Unk	0.817	http://api.brain-map.org/grid_data/download/69013684?include=energy
Abcb8	0.817	http://api.brain-map.org/grid_data/download/71627755?include=energy
Klf1	0.817	http://api.brain-map.org/grid_data/download/71488728?include=energy
C80913	0.817	http://api.brain-map.org/grid_data/download/70429178?include=energy
Prdm1	0.817	http://api.brain-map.org/grid_data/download/75985912?include=energy
Pf4	0.817	http://api.brain-map.org/grid_data/download/69117134?include=energy
Arl4a	0.817	http://api.brain-map.org/grid_data/download/100144398?include=energy
LOC433231	0.817	http://api.brain-map.org/grid_data/download/70723038?include=energy
Tomm6	0.817	http://api.brain-map.org/grid_data/download/678717?include=energy
Mier3	0.817	http://api.brain-map.org/grid_data/download/74513947?include=energy
E030010N08Rik	0.817	http://api.brain-map.org/grid_data/download/70238955?include=energy
Dnalc1	0.817	http://api.brain-map.org/grid_data/download/74427101?include=energy
Plg	0.817	http://api.brain-map.org/grid_data/download/2485?include=energy
Apoc2	0.817	http://api.brain-map.org/grid_data/download/68632196?include=energy
Klhl26	0.817	http://api.brain-map.org/grid_data/download/71616113?include=energy
Pcdhgb4	0.817	http://api.brain-map.org/grid_data/download/70784922?include=energy
Tab1	0.817	http://api.brain-map.org/grid_data/download/69013666?include=energy
Zfp277	0.817	http://api.brain-map.org/grid_data/download/68920803?include=energy
Sh2d7	0.817	http://api.brain-map.org/grid_data/download/70717367?include=energy
Arhgef25	0.817	http://api.brain-map.org/grid_data/download/69013642?include=energy
Prpf4b	0.817	http://api.brain-map.org/grid_data/download/69134493?include=energy
Zbtb45	0.817	http://api.brain-map.org/grid_data/download/71764395?include=energy
Rlbp1	0.817	http://api.brain-map.org/grid_data/download/638716?include=energy
Frmpd1	0.817	http://api.brain-map.org/grid_data/download/73520978?include=energy
Atp5g1	0.817	http://api.brain-map.org/grid_data/download/73513453?include=energy
Ndfip2	0.817	http://api.brain-map.org/grid_data/download/76003564?include=energy
Nmur2	0.817	http://api.brain-map.org/grid_data/download/81600118?include=energy
Fhl5	0.817	http://api.brain-map.org/grid_data/download/75851046?include=energy
4930565A17	0.817	http://api.brain-map.org/grid_data/download/72005996?include=energy
Actl6a	0.817	http://api.brain-map.org/grid_data/download/70919334?include=energy
Sox5	0.817	http://api.brain-map.org/grid_data/download/1284?include=energy
Uap1	0.817	http://api.brain-map.org/grid_data/download/70743895?include=energy
Vmn1r82	0.817	http://api.brain-map.org/grid_data/download/69442260?include=energy
Papolg	0.817	http://api.brain-map.org/grid_data/download/69014878?include=energy
4632404H12Rik	0.817	http://api.brain-map.org/grid_data/download/69407606?include=energy
Grk4	0.817	http://api.brain-map.org/grid_data/download/70724762?include=energy
Immp2l	0.817	http://api.brain-map.org/grid_data/download/70278069?include=energy
Fam100b	0.817	http://api.brain-map.org/grid_data/download/69352908?include=energy
Hoxa2	0.816	http://api.brain-map.org/grid_data/download/77464936?include=energy
Ifngr1	0.816	http://api.brain-map.org/grid_data/download/69735257?include=energy
1700052K11Rik	0.816	http://api.brain-map.org/grid_data/download/100144891?include=energy
LOC239679	0.816	http://api.brain-map.org/grid_data/download/75990865?include=energy
Sbds	0.816	http://api.brain-map.org/grid_data/download/68269276?include=energy

Ly96	0.816	http://api.brain-map.org/grid_data/download/70300252?include=energy
C230078M08Rik	0.816	http://api.brain-map.org/grid_data/download/77904879?include=energy
AI316807	0.816	http://api.brain-map.org/grid_data/download/71213081?include=energy
A930012M21Rik	0.816	http://api.brain-map.org/grid_data/download/74300699?include=energy
Olfr866	0.816	http://api.brain-map.org/grid_data/download/74734973?include=energy
E230023K05Rik	0.816	http://api.brain-map.org/grid_data/download/74510200?include=energy
LOC432748	0.816	http://api.brain-map.org/grid_data/download/71924502?include=energy
A630018G05Rik	0.816	http://api.brain-map.org/grid_data/download/70228531?include=energy
Wdr69	0.816	http://api.brain-map.org/grid_data/download/71493663?include=energy
Trim40	0.816	http://api.brain-map.org/grid_data/download/74743384?include=energy
Slc33a1	0.816	http://api.brain-map.org/grid_data/download/68745137?include=energy
Trappc9	0.816	http://api.brain-map.org/grid_data/download/68441604?include=energy
Cts7	0.816	http://api.brain-map.org/grid_data/download/68910985?include=energy
Pdlim3	0.816	http://api.brain-map.org/grid_data/download/69782951?include=energy
Tbc1d8b	0.816	http://api.brain-map.org/grid_data/download/73513245?include=energy
Serpina3f	0.816	http://api.brain-map.org/grid_data/download/71574552?include=energy
Osbpl11	0.816	http://api.brain-map.org/grid_data/download/76135504?include=energy
1810011O10Rik	0.816	http://api.brain-map.org/grid_data/download/68341573?include=energy
Cic	0.816	http://api.brain-map.org/grid_data/download/71307244?include=energy
Rbpjl	0.816	http://api.brain-map.org/grid_data/download/69289781?include=energy
Gpr160	0.816	http://api.brain-map.org/grid_data/download/69857040?include=energy
Tle4	0.816	http://api.brain-map.org/grid_data/download/73521809?include=energy
1700066M21Rik	0.816	http://api.brain-map.org/grid_data/download/69353747?include=energy
Cys1	0.816	http://api.brain-map.org/grid_data/download/74635725?include=energy
Olfr607	0.816	http://api.brain-map.org/grid_data/download/74720531?include=energy
9330185C12Rik	0.816	http://api.brain-map.org/grid_data/download/73513353?include=energy
Yy1	0.816	http://api.brain-map.org/grid_data/download/75831764?include=energy
Bsn	0.816	http://api.brain-map.org/grid_data/download/73992923?include=energy
Snrpb2	0.816	http://api.brain-map.org/grid_data/download/68633404?include=energy
4930523C07Rik	0.816	http://api.brain-map.org/grid_data/download/73808600?include=energy
Spdyb	0.816	http://api.brain-map.org/grid_data/download/71891755?include=energy
2310040G24Rik	0.816	http://api.brain-map.org/grid_data/download/74635415?include=energy
Mrgprf	0.816	http://api.brain-map.org/grid_data/download/79541444?include=energy
Hnf4a	0.816	http://api.brain-map.org/grid_data/download/1746?include=energy
Adss	0.816	http://api.brain-map.org/grid_data/download/76003369?include=energy
Riok3	0.816	http://api.brain-map.org/grid_data/download/68637808?include=energy
Ighd	0.816	http://api.brain-map.org/grid_data/download/74578200?include=energy
Efcab2	0.816	http://api.brain-map.org/grid_data/download/378544?include=energy
LOC433092	0.816	http://api.brain-map.org/grid_data/download/74431733?include=energy
Mkrn3	0.816	http://api.brain-map.org/grid_data/download/69289369?include=energy
Kdm5c	0.816	http://api.brain-map.org/grid_data/download/69289255?include=energy
Rsf1	0.816	http://api.brain-map.org/grid_data/download/71211677?include=energy
C230040D14	0.815	http://api.brain-map.org/grid_data/download/70305538?include=energy
Xpr1	0.815	http://api.brain-map.org/grid_data/download/70195066?include=energy
Crym	0.815	http://api.brain-map.org/grid_data/download/73592526?include=energy
Vhl	0.815	http://api.brain-map.org/grid_data/download/71378632?include=energy
Ccdc21	0.815	http://api.brain-map.org/grid_data/download/68797496?include=energy
1700034I23Rik	0.815	http://api.brain-map.org/grid_data/download/77799035?include=energy
Chac1	0.815	http://api.brain-map.org/grid_data/download/75828178?include=energy
Ccng2	0.815	http://api.brain-map.org/grid_data/download/69540519?include=energy
Cdx2	0.815	http://api.brain-map.org/grid_data/download/75934932?include=energy
Trim55	0.815	http://api.brain-map.org/grid_data/download/74510146?include=energy
1700019L03Rik	0.815	http://api.brain-map.org/grid_data/download/69353587?include=energy
Camk2a	0.815	http://api.brain-map.org/grid_data/download/79490122?include=energy
Tsen54	0.815	http://api.brain-map.org/grid_data/download/69352788?include=energy
Hnrpll	0.815	http://api.brain-map.org/grid_data/download/69169343?include=energy
Mnd1	0.815	http://api.brain-map.org/grid_data/download/68445664?include=energy
D930042J21Rik*	0.815	http://api.brain-map.org/grid_data/download/73834481?include=energy
Vmn1r228	0.815	http://api.brain-map.org/grid_data/download/70785331?include=energy
Hbs1l	0.815	http://api.brain-map.org/grid_data/download/68300902?include=energy
Gm1320	0.815	http://api.brain-map.org/grid_data/download/70300091?include=energy
Snx30	0.815	http://api.brain-map.org/grid_data/download/69407702?include=energy
Crmp1	0.815	http://api.brain-map.org/grid_data/download/73512561?include=energy
Olfr603	0.815	http://api.brain-map.org/grid_data/download/74724247?include=energy
Ldlrad2	0.815	http://api.brain-map.org/grid_data/download/71022591?include=energy
Rcor1	0.815	http://api.brain-map.org/grid_data/download/74819517?include=energy
Th1l	0.815	http://api.brain-map.org/grid_data/download/513226?include=energy
Sh3tc2	0.815	http://api.brain-map.org/grid_data/download/69608965?include=energy
Fbxl17	0.815	http://api.brain-map.org/grid_data/download/71212440?include=energy
Hn1	0.815	http://api.brain-map.org/grid_data/download/70431361?include=energy
Trub2	0.815	http://api.brain-map.org/grid_data/download/393246?include=energy
LOC383041	0.815	http://api.brain-map.org/grid_data/download/71281925?include=energy
Rbm43	0.815	http://api.brain-map.org/grid_data/download/67978672?include=energy
Zfp775	0.815	http://api.brain-map.org/grid_data/download/70593180?include=energy
3110079O15Rik	0.815	http://api.brain-map.org/grid_data/download/70723676?include=energy
Slc6a15	0.815	http://api.brain-map.org/grid_data/download/73929609?include=energy
A630078I01Rik*	0.815	http://api.brain-map.org/grid_data/download/73808624?include=energy
Zfp131	0.815	http://api.brain-map.org/grid_data/download/69028462?include=energy
Gja4	0.815	http://api.brain-map.org/grid_data/download/69028503?include=energy
Gtf2h4	0.815	http://api.brain-map.org/grid_data/download/1458?include=energy
Atxn7l1	0.815	http://api.brain-map.org/grid_data/download/71486982?include=energy
4930544O15Rik	0.815	http://api.brain-map.org/grid_data/download/70218312?include=energy
Rhobtb1	0.815	http://api.brain-map.org/grid_data/download/69290423?include=energy
Ms4a7	0.815	http://api.brain-map.org/grid_data/download/68499428?include=energy
Fzd5	0.815	http://api.brain-map.org/grid_data/download/1500?include=energy
Cerk	0.815	http://api.brain-map.org/grid_data/download/72472765?include=energy
Lnx1	0.815	http://api.brain-map.org/grid_data/download/74277745?include=energy
Syt13	0.815	http://api.brain-map.org/grid_data/download/1864?include=energy
Epc2	0.815	http://api.brain-map.org/grid_data/download/70239111?include=energy
Gmppa	0.815	http://api.brain-map.org/grid_data/download/73818757?include=energy
2810405K02Rik	0.815	http://api.brain-map.org/grid_data/download/74047901?include=energy
Prg3	0.815	http://api.brain-map.org/grid_data/download/75931519?include=energy
Zfp687	0.815	http://api.brain-map.org/grid_data/download/71380351?include=energy
Bub1	0.815	http://api.brain-map.org/grid_data/download/68563068?include=energy
1700001L05Rik	0.815	http://api.brain-map.org/grid_data/download/73870725?include=energy
4930570N19Rik*	0.815	http://api.brain-map.org/grid_data/download/73870557?include=energy
Ptpn13	0.814	http://api.brain-map.org/grid_data/download/69028432?include=energy
Slc52a2	0.814	http://api.brain-map.org/grid_data/download/71016711?include=energy
Mos	0.814	http://api.brain-map.org/grid_data/download/71020763?include=energy
Kcnb1	0.814	http://api.brain-map.org/grid_data/download/74047856?include=energy
Zfp940	0.814	http://api.brain-map.org/grid_data/download/74514043?include=energy
Slc16a7	0.814	http://api.brain-map.org/grid_data/download/72001956?include=energy
Pxmp2	0.814	http://api.brain-map.org/grid_data/download/69015146?include=energy
Gm1833	0.814	http://api.brain-map.org/grid_data/download/70295853?include=energy
Yrdc	0.814	http://api.brain-map.org/grid_data/download/70430870?include=energy
Il1rap	0.814	http://api.brain-map.org/grid_data/download/1462?include=energy
LOC435066	0.814	http://api.brain-map.org/grid_data/download/71022439?include=energy
BC028450	0.814	http://api.brain-map.org/grid_data/download/69529035?include=energy
Vps45	0.814	http://api.brain-map.org/grid_data/download/513015?include=energy
Oxr1	0.814	http://api.brain-map.org/grid_data/download/72129295?include=energy
Ces1c	0.814	http://api.brain-map.org/grid_data/download/68632154?include=energy
Nipa2	0.814	http://api.brain-map.org/grid_data/download/75041549?include=energy
Poln	0.814	http://api.brain-map.org/grid_data/download/73423975?include=energy
Mycl1	0.814	http://api.brain-map.org/grid_data/download/74881158?include=energy
Pcdhgb5	0.814	http://api.brain-map.org/grid_data/download/70784928?include=energy
Gimap5	0.814	http://api.brain-map.org/grid_data/download/70239027?include=energy
Ccdc83	0.814	http://api.brain-map.org/grid_data/download/75694308?include=energy
Rnf183	0.814	http://api.brain-map.org/grid_data/download/75828173?include=energy
Zfp787	0.814	http://api.brain-map.org/grid_data/download/71378665?include=energy
Cdca2	0.814	http://api.brain-map.org/grid_data/download/73520972?include=energy
Cdk5r1	0.814	http://api.brain-map.org/grid_data/download/247?include=energy
Ppp2r2b	0.814	http://api.brain-map.org/grid_data/download/1655?include=energy
Pknox1	0.814	http://api.brain-map.org/grid_data/download/1401?include=energy
Gm17745	0.814	http://api.brain-map.org/grid_data/download/70608571?include=energy
Olfr449	0.814	http://api.brain-map.org/grid_data/download/74724103?include=energy
Lats2	0.814	http://api.brain-map.org/grid_data/download/73931411?include=energy
Cst10	0.814	http://api.brain-map.org/grid_data/download/75831751?include=energy
Lipk	0.814	http://api.brain-map.org/grid_data/download/69014925?include=energy
Insm2	0.814	http://api.brain-map.org/grid_data/download/71609030?include=energy
Oxct2a	0.814	http://api.brain-map.org/grid_data/download/71210768?include=energy
4930560A13Rik*	0.814	http://api.brain-map.org/grid_data/download/73927552?include=energy
Rdh12	0.814	http://api.brain-map.org/grid_data/download/75214939?include=energy
Cwc25	0.814	http://api.brain-map.org/grid_data/download/73929874?include=energy
6430571L13Rik	0.814	http://api.brain-map.org/grid_data/download/71493813?include=energy
D6Wsu163e	0.814	http://api.brain-map.org/grid_data/download/68443333?include=energy
Krt24	0.814	http://api.brain-map.org/grid_data/download/70218721?include=energy
Cdh10	0.814	http://api.brain-map.org/grid_data/download/73592529?include=energy
2410012M07Rik	0.814	http://api.brain-map.org/grid_data/download/70203501?include=energy
Ppp2r1b	0.814	http://api.brain-map.org/grid_data/download/69835562?include=energy
Ighe	0.814	http://api.brain-map.org/grid_data/download/69734458?include=energy
Uts2r	0.814	http://api.brain-map.org/grid_data/download/69288471?include=energy
Spata5	0.814	http://api.brain-map.org/grid_data/download/69885672?include=energy
Cxcr4	0.814	http://api.brain-map.org/grid_data/download/100144881?include=energy
Gm5551	0.814	http://api.brain-map.org/grid_data/download/70787020?include=energy
4930474N05Rik	0.814	http://api.brain-map.org/grid_data/download/70723694?include=energy
Nos1	0.813	http://api.brain-map.org/grid_data/download/75147762?include=energy
Tbc1d7	0.813	http://api.brain-map.org/grid_data/download/69980172?include=energy
LOC330599	0.813	http://api.brain-map.org/grid_data/download/70595877?include=energy
Chrnd	0.813	http://api.brain-map.org/grid_data/download/74988680?include=energy
Pcdhgb6	0.813	http://api.brain-map.org/grid_data/download/70784934?include=energy
Fmo3	0.813	http://api.brain-map.org/grid_data/download/69672515?include=energy
Cpb1	0.813	http://api.brain-map.org/grid_data/download/74749237?include=energy
Wee2	0.813	http://api.brain-map.org/grid_data/download/68667661?include=energy
Krt40	0.813	http://api.brain-map.org/grid_data/download/72283641?include=energy
Ift46	0.813	http://api.brain-map.org/grid_data/download/74357609?include=energy
Il12rb2	0.813	http://api.brain-map.org/grid_data/download/69735353?include=energy
Zfp35	0.813	http://api.brain-map.org/grid_data/download/70598372?include=energy
A530060J09Rik*	0.813	http://api.brain-map.org/grid_data/download/73808588?include=energy
9430018F04Rik*	0.813	http://api.brain-map.org/grid_data/download/73927757?include=energy
Ddx54	0.813	http://api.brain-map.org/grid_data/download/71280874?include=energy
Rsu1	0.813	http://api.brain-map.org/grid_data/download/632495?include=energy
Efna1	0.813	http://api.brain-map.org/grid_data/download/308056322?include=energy
Vmn2r29	0.813	http://api.brain-map.org/grid_data/download/69445373?include=energy
Cyb5r1	0.813	http://api.brain-map.org/grid_data/download/74988452?include=energy
Plekhh2	0.813	http://api.brain-map.org/grid_data/download/74581448?include=energy
Vps13a	0.813	http://api.brain-map.org/grid_data/download/69608929?include=energy
Angptl4	0.813	http://api.brain-map.org/grid_data/download/71723863?include=energy
Slc12a8	0.813	http://api.brain-map.org/grid_data/download/1836?include=energy
4933413J09Rik	0.813	http://api.brain-map.org/grid_data/download/70218751?include=energy
Usp25	0.813	http://api.brain-map.org/grid_data/download/2713?include=energy
Bicd1	0.813	http://api.brain-map.org/grid_data/download/70430904?include=energy
Pla2g2c	0.813	http://api.brain-map.org/grid_data/download/68632691?include=energy
LOC432566	0.813	http://api.brain-map.org/grid_data/download/74578353?include=energy
Dnmt3l	0.813	http://api.brain-map.org/grid_data/download/73716572?include=energy
Dhx40	0.813	http://api.brain-map.org/grid_data/download/73592533?include=energy
Hpgds	0.813	http://api.brain-map.org/grid_data/download/69817637?include=energy
Chrne	0.813	http://api.brain-map.org/grid_data/download/75888738?include=energy
Gpr20	0.813	http://api.brain-map.org/grid_data/download/69857606?include=energy
Cnga4	0.813	http://api.brain-map.org/grid_data/download/76081091?include=energy
8430432A02Rik	0.813	http://api.brain-map.org/grid_data/download/72081567?include=energy
Pou6f2	0.813	http://api.brain-map.org/grid_data/download/71249067?include=energy
Cox16	0.813	http://api.brain-map.org/grid_data/download/633690?include=energy
Rab3b	0.813	http://api.brain-map.org/grid_data/download/70436738?include=energy
Aldh3a2	0.813	http://api.brain-map.org/grid_data/download/68269266?include=energy
Slc20a1	0.813	http://api.brain-map.org/grid_data/download/1571?include=energy
LOC433369	0.813	http://api.brain-map.org/grid_data/download/70723193?include=energy
Casp14	0.813	http://api.brain-map.org/grid_data/download/69540393?include=energy
Prg4	0.813	http://api.brain-map.org/grid_data/download/76081025?include=energy
Serpina3c	0.813	http://api.brain-map.org/grid_data/download/74511905?include=energy
Rab39	0.813	http://api.brain-map.org/grid_data/download/68666870?include=energy
Smpd1	0.813	http://api.brain-map.org/grid_data/download/77413684?include=energy
Cldn8	0.813	http://api.brain-map.org/grid_data/download/631722?include=energy
Abca6	0.813	http://api.brain-map.org/grid_data/download/69514548?include=energy
Mm.246775	0.813	http://api.brain-map.org/grid_data/download/71456142?include=energy
Npr1	0.813	http://api.brain-map.org/grid_data/download/71670678?include=energy
Tcof1	0.813	http://api.brain-map.org/grid_data/download/68919045?include=energy
Mosc2	0.813	http://api.brain-map.org/grid_data/download/68269301?include=energy
Usp15	0.813	http://api.brain-map.org/grid_data/download/68666983?include=energy
LOC433635	0.813	http://api.brain-map.org/grid_data/download/71456123?include=energy
Rabep2	0.813	http://api.brain-map.org/grid_data/download/1970?include=energy
Ttc39d	0.813	http://api.brain-map.org/grid_data/download/70218378?include=energy
Rpgrip1l	0.813	http://api.brain-map.org/grid_data/download/69353714?include=energy
LOC545838	0.813	http://api.brain-map.org/grid_data/download/74277637?include=energy
Ccdc72	0.813	http://api.brain-map.org/grid_data/download/71763951?include=energy
Nkiras2	0.813	http://api.brain-map.org/grid_data/download/2429?include=energy
Gpr137b	0.812	http://api.brain-map.org/grid_data/download/67979043?include=energy
Cog3	0.812	http://api.brain-map.org/grid_data/download/70445670?include=energy
Kif26a	0.812	http://api.brain-map.org/grid_data/download/71529989?include=energy
4930562C15Rik	0.812	http://api.brain-map.org/grid_data/download/72283444?include=energy
Fhit	0.812	http://api.brain-map.org/grid_data/download/75990503?include=energy
Ccrl1	0.812	http://api.brain-map.org/grid_data/download/69237059?include=energy
Repin1	0.812	http://api.brain-map.org/grid_data/download/69289799?include=energy
Cadm2	0.812	http://api.brain-map.org/grid_data/download/73635999?include=energy
Ppm1m	0.812	http://api.brain-map.org/grid_data/download/68798205?include=energy
Zfp580	0.812	http://api.brain-map.org/grid_data/download/71488913?include=energy
D030016E14Rik	0.812	http://api.brain-map.org/grid_data/download/74359616?include=energy
Nrarp	0.812	http://api.brain-map.org/grid_data/download/70593339?include=energy
Helt	0.812	http://api.brain-map.org/grid_data/download/81654155?include=energy
Dio1	0.812	http://api.brain-map.org/grid_data/download/71213901?include=energy
Gpd1l	0.812	http://api.brain-map.org/grid_data/download/74988763?include=energy
Shc4	0.812	http://api.brain-map.org/grid_data/download/71493789?include=energy
Mm.349531	0.812	http://api.brain-map.org/grid_data/download/71161096?include=energy
E130010M05Rik	0.812	http://api.brain-map.org/grid_data/download/70238979?include=energy
1700013H16Rik	0.812	http://api.brain-map.org/grid_data/download/76081048?include=energy
Pde6b	0.812	http://api.brain-map.org/grid_data/download/69133641?include=energy
D130032N22Rik*	0.812	http://api.brain-map.org/grid_data/download/73834349?include=energy
Zfp438	0.812	http://api.brain-map.org/grid_data/download/69288674?include=energy
Ctsj	0.812	http://api.brain-map.org/grid_data/download/75931621?include=energy
Sstr1	0.812	http://api.brain-map.org/grid_data/download/71488819?include=energy
4930522H14Rik	0.812	http://api.brain-map.org/grid_data/download/75931624?include=energy
Plekhm3	0.812	http://api.brain-map.org/grid_data/download/68798352?include=energy
Cyp3a11	0.812	http://api.brain-map.org/grid_data/download/68302178?include=energy
Ndst3	0.812	http://api.brain-map.org/grid_data/download/75041473?include=energy
Rras	0.812	http://api.brain-map.org/grid_data/download/69873319?include=energy
Dock7	0.812	http://api.brain-map.org/grid_data/download/68845633?include=energy
Srrm4	0.812	http://api.brain-map.org/grid_data/download/77620790?include=energy
7120492P10Rik*	0.812	http://api.brain-map.org/grid_data/download/73906089?include=energy
Rnf186	0.812	http://api.brain-map.org/grid_data/download/75694863?include=energy
Palm3	0.812	http://api.brain-map.org/grid_data/download/74958547?include=energy
Zfp281	0.812	http://api.brain-map.org/grid_data/download/81599626?include=energy
Samd4b	0.812	http://api.brain-map.org/grid_data/download/70528348?include=energy
Gm41	0.812	http://api.brain-map.org/grid_data/download/70725031?include=energy
Tasp1	0.812	http://api.brain-map.org/grid_data/download/68637886?include=energy
C130039O16Rik	0.812	http://api.brain-map.org/grid_data/download/71232648?include=energy
Slc8a2	0.812	http://api.brain-map.org/grid_data/download/74581450?include=energy
Armc8	0.812	http://api.brain-map.org/grid_data/download/68546230?include=energy
Pcdhga7	0.812	http://api.brain-map.org/grid_data/download/70784898?include=energy
Rnf141	0.812	http://api.brain-map.org/grid_data/download/564645?include=energy
Olfr148	0.812	http://api.brain-map.org/grid_data/download/74720850?include=energy
Ptplad2	0.812	http://api.brain-map.org/grid_data/download/587424?include=energy
Zfp606	0.812	http://api.brain-map.org/grid_data/download/69407176?include=energy
A530006G24Rik	0.812	http://api.brain-map.org/grid_data/download/71809265?include=energy
4932430N15Rik*	0.812	http://api.brain-map.org/grid_data/download/73927577?include=energy
Fabp6	0.812	http://api.brain-map.org/grid_data/download/73513735?include=energy
Zfp597	0.812	http://api.brain-map.org/grid_data/download/70303672?include=energy
Mm.347812	0.811	http://api.brain-map.org/grid_data/download/71161078?include=energy
Kbtbd7	0.811	http://api.brain-map.org/grid_data/download/70295637?include=energy
1300018J18Rik	0.811	http://api.brain-map.org/grid_data/download/70200932?include=energy
B3galnt2	0.811	http://api.brain-map.org/grid_data/download/69608887?include=energy
Kcnn4	0.811	http://api.brain-map.org/grid_data/download/130911?include=energy
Pcdhga9	0.811	http://api.brain-map.org/grid_data/download/74277422?include=energy
Paxip1	0.811	http://api.brain-map.org/grid_data/download/71668994?include=energy
Negr1	0.811	http://api.brain-map.org/grid_data/download/692?include=energy
Dpep3	0.811	http://api.brain-map.org/grid_data/download/75831779?include=energy
Iqcf4	0.811	http://api.brain-map.org/grid_data/download/70474869?include=energy
Mylpf	0.811	http://api.brain-map.org/grid_data/download/70784678?include=energy
Slc22a18	0.811	http://api.brain-map.org/grid_data/download/78534520?include=energy

2400009B08Rik	0.811	http://api.brain-map.org/grid_data/download/70303797?include=energy
Dcakd	0.811	http://api.brain-map.org/grid_data/download/68861698?include=energy
LOC434154	0.811	http://api.brain-map.org/grid_data/download/70787489?include=energy
Ifit1	0.811	http://api.brain-map.org/grid_data/download/632514?include=energy
Lpar3	0.811	http://api.brain-map.org/grid_data/download/69237227?include=energy
Stc2	0.811	http://api.brain-map.org/grid_data/download/80343330?include=energy
Ifnk	0.811	http://api.brain-map.org/grid_data/download/70278009?include=energy
Msh5	0.811	http://api.brain-map.org/grid_data/download/71020823?include=energy
Sdc1	0.811	http://api.brain-map.org/grid_data/download/634448?include=energy
Wdr85	0.811	http://api.brain-map.org/grid_data/download/68546311?include=energy
Exosc8	0.811	http://api.brain-map.org/grid_data/download/74658079?include=energy
4933411G11Rik	0.811	http://api.brain-map.org/grid_data/download/72006002?include=energy
Eml5	0.811	http://api.brain-map.org/grid_data/download/74513941?include=energy
Slitrk1	0.811	http://api.brain-map.org/grid_data/download/74882937?include=energy
Sept5	0.811	http://api.brain-map.org/grid_data/download/958?include=energy
Hrnr	0.811	http://api.brain-map.org/grid_data/download/75990416?include=energy
Cmklr1	0.811	http://api.brain-map.org/grid_data/download/72340129?include=energy
Rest	0.811	http://api.brain-map.org/grid_data/download/112645169?include=energy
Tesk1	0.811	http://api.brain-map.org/grid_data/download/69980268?include=energy
Lrriq3	0.811	http://api.brain-map.org/grid_data/download/70218631?include=energy
Magea10	0.811	http://api.brain-map.org/grid_data/download/70566175?include=energy
Spt1	0.811	http://api.brain-map.org/grid_data/download/71724720?include=energy
Trip13	0.811	http://api.brain-map.org/grid_data/download/71488870?include=energy
4930404H11Rik	0.811	http://api.brain-map.org/grid_data/download/74579975?include=energy
Rilp	0.811	http://api.brain-map.org/grid_data/download/71569375?include=energy
Dclre1b	0.811	http://api.brain-map.org/grid_data/download/564642?include=energy
Sdk2	0.811	http://api.brain-map.org/grid_data/download/77332746?include=energy
Aqp8	0.811	http://api.brain-map.org/grid_data/download/77454616?include=energy
E130008D07Rik	0.811	http://api.brain-map.org/grid_data/download/73513717?include=energy
Gjb4	0.811	http://api.brain-map.org/grid_data/download/77793179?include=energy
H19	0.811	http://api.brain-map.org/grid_data/download/71723955?include=energy
Polr1d	0.811	http://api.brain-map.org/grid_data/download/68499368?include=energy
Papln	0.811	http://api.brain-map.org/grid_data/download/73423911?include=energy
Hnf1b	0.811	http://api.brain-map.org/grid_data/download/1874?include=energy
Vac14	0.811	http://api.brain-map.org/grid_data/download/74357721?include=energy
Smad9	0.811	http://api.brain-map.org/grid_data/download/69816882?include=energy
Ift52	0.81	http://api.brain-map.org/grid_data/download/678709?include=energy
Aprt	0.81	http://api.brain-map.org/grid_data/download/633282?include=energy
Slc1a6	0.81	http://api.brain-map.org/grid_data/download/69873845?include=energy
Gmeb1	0.81	http://api.brain-map.org/grid_data/download/68924775?include=energy
Nxt1	0.81	http://api.brain-map.org/grid_data/download/68445155?include=energy
Gpr180	0.81	http://api.brain-map.org/grid_data/download/69671702?include=energy
Pou2f2	0.81	http://api.brain-map.org/grid_data/download/81657683?include=energy
Zscan18	0.81	http://api.brain-map.org/grid_data/download/70566106?include=energy
Nmnat1	0.81	http://api.brain-map.org/grid_data/download/71210544?include=energy
N6amt1	0.81	http://api.brain-map.org/grid_data/download/1233?include=energy
Xpa	0.81	http://api.brain-map.org/grid_data/download/70474743?include=energy
Mat2b	0.81	http://api.brain-map.org/grid_data/download/67978386?include=energy
Tor2a	0.81	http://api.brain-map.org/grid_data/download/70542968?include=energy
Pgr15l	0.81	http://api.brain-map.org/grid_data/download/73615804?include=energy
Grap2	0.81	http://api.brain-map.org/grid_data/download/68743949?include=energy
Dot1l	0.81	http://api.brain-map.org/grid_data/download/74821681?include=energy
Nccrp1	0.81	http://api.brain-map.org/grid_data/download/72081633?include=energy
Ptch1	0.81	http://api.brain-map.org/grid_data/download/69817625?include=energy
Fbxo34	0.81	http://api.brain-map.org/grid_data/download/74882585?include=energy
Col12a1	0.81	http://api.brain-map.org/grid_data/download/73817431?include=energy
Has1	0.81	http://api.brain-map.org/grid_data/download/71393346?include=energy
Gtf2a2	0.81	http://api.brain-map.org/grid_data/download/68666911?include=energy
Trappc10	0.81	http://api.brain-map.org/grid_data/download/1063?include=energy
Slc4a11	0.81	http://api.brain-map.org/grid_data/download/69873994?include=energy
Npy5r	0.81	http://api.brain-map.org/grid_data/download/69258202?include=energy
Coil	0.81	http://api.brain-map.org/grid_data/download/69173304?include=energy
Fbxo46	0.81	http://api.brain-map.org/grid_data/download/69672366?include=energy
Alpk1	0.81	http://api.brain-map.org/grid_data/download/76080943?include=energy
Wdfy4	0.81	http://api.brain-map.org/grid_data/download/74277302?include=energy
Tcf15	0.81	http://api.brain-map.org/grid_data/download/69549193?include=energy
Pi16	0.81	http://api.brain-map.org/grid_data/download/73423938?include=energy
G630064H08Rik	0.81	http://api.brain-map.org/grid_data/download/71529809?include=energy
Commd9	0.81	http://api.brain-map.org/grid_data/download/69568926?include=energy
D730003I15Rik	0.81	http://api.brain-map.org/grid_data/download/74300747?include=energy
Ppil2	0.81	http://api.brain-map.org/grid_data/download/69549249?include=energy
mCG1030693.1	0.81	http://api.brain-map.org/grid_data/download/71022852?include=energy
Clpb	0.81	http://api.brain-map.org/grid_data/download/70593358?include=energy
1700023L04Rik	0.81	http://api.brain-map.org/grid_data/download/71492862?include=energy
Asb17	0.81	http://api.brain-map.org/grid_data/download/69116457?include=energy
Relb	0.81	http://api.brain-map.org/grid_data/download/2525?include=energy
Oasl2	0.809	http://api.brain-map.org/grid_data/download/68546317?include=energy
Cd300lf	0.809	http://api.brain-map.org/grid_data/download/75831767?include=energy
0610040B10Rik	0.809	http://api.brain-map.org/grid_data/download/69352802?include=energy
Fam65b	0.809	http://api.brain-map.org/grid_data/download/73635806?include=energy
Ddx58	0.809	http://api.brain-map.org/grid_data/download/69626825?include=energy
Cdkn2aipnl	0.809	http://api.brain-map.org/grid_data/download/69608453?include=energy
Igkv19-93	0.809	http://api.brain-map.org/grid_data/download/69735287?include=energy
Camsap1	0.809	http://api.brain-map.org/grid_data/download/69540327?include=energy
Eny2	0.809	http://api.brain-map.org/grid_data/download/68924431?include=energy
Nup210l	0.809	http://api.brain-map.org/grid_data/download/74580093?include=energy
Cst6	0.809	http://api.brain-map.org/grid_data/download/75041560?include=energy
Htr2b	0.809	http://api.brain-map.org/grid_data/download/79556708?include=energy
Anubl1	0.809	http://api.brain-map.org/grid_data/download/69114980?include=energy
Podnl1	0.809	http://api.brain-map.org/grid_data/download/100146162?include=energy
Zfp62	0.809	http://api.brain-map.org/grid_data/download/71763667?include=energy
Zfp518a	0.809	http://api.brain-map.org/grid_data/download/70203567?include=energy
1520402A15Rik	0.809	http://api.brain-map.org/grid_data/download/71211671?include=energy
Acvr2b	0.809	http://api.brain-map.org/grid_data/download/69514732?include=energy
B930023M13Rik	0.809	http://api.brain-map.org/grid_data/download/74822577?include=energy
1700122O11Rik	0.809	http://api.brain-map.org/grid_data/download/74359299?include=energy
Treml1	0.809	http://api.brain-map.org/grid_data/download/71489792?include=energy
Mm.375697	0.809	http://api.brain-map.org/grid_data/download/71210160?include=energy
Schip1	0.809	http://api.brain-map.org/grid_data/download/72077455?include=energy
Tfe3	0.809	http://api.brain-map.org/grid_data/download/69290639?include=energy
Scgn	0.809	http://api.brain-map.org/grid_data/download/583549?include=energy
Ccdc40	0.809	http://api.brain-map.org/grid_data/download/69527998?include=energy
Col5a1	0.809	http://api.brain-map.org/grid_data/download/75147768?include=energy
LOC328602	0.809	http://api.brain-map.org/grid_data/download/74579467?include=energy
Tlx1	0.809	http://api.brain-map.org/grid_data/download/76097725?include=energy
Cox15	0.809	http://api.brain-map.org/grid_data/download/80525718?include=energy
Reg3a	0.809	http://api.brain-map.org/grid_data/download/74635262?include=energy
Scn2a1	0.809	http://api.brain-map.org/grid_data/download/100145300?include=energy
1810008A18Rik	0.809	http://api.brain-map.org/grid_data/download/75828177?include=energy
Wdr38	0.809	http://api.brain-map.org/grid_data/download/74635156?include=energy
Il9r	0.809	http://api.brain-map.org/grid_data/download/69735443?include=energy
5930403N24Rik	0.809	http://api.brain-map.org/grid_data/download/69444717?include=energy
Tmem214	0.809	http://api.brain-map.org/grid_data/download/70445395?include=energy
2010300C02Rik	0.809	http://api.brain-map.org/grid_data/download/70743898?include=energy
Ppdpf	0.809	http://api.brain-map.org/grid_data/download/68919880?include=energy
Prm2	0.809	http://api.brain-map.org/grid_data/download/75860928?include=energy
Gm9732	0.809	http://api.brain-map.org/grid_data/download/73512569?include=energy
Slc25a14	0.809	http://api.brain-map.org/grid_data/download/69835597?include=energy
Gabrp	0.809	http://api.brain-map.org/grid_data/download/75888749?include=energy
Klrb1c	0.809	http://api.brain-map.org/grid_data/download/69117110?include=energy
Nox3	0.809	http://api.brain-map.org/grid_data/download/70300571?include=energy
Dars	0.809	http://api.brain-map.org/grid_data/download/71656067?include=energy
Dmbx1	0.809	http://api.brain-map.org/grid_data/download/1484?include=energy
6030458C11Rik	0.809	http://api.brain-map.org/grid_data/download/68545045?include=energy
Myf5	0.809	http://api.brain-map.org/grid_data/download/73606522?include=energy
1700011A15Rik	0.809	http://api.brain-map.org/grid_data/download/69114235?include=energy
Gpr161	0.809	http://api.brain-map.org/grid_data/download/70562045?include=energy
9330159N22Rik	0.809	http://api.brain-map.org/grid_data/download/74580883?include=energy
Shisa6	0.809	http://api.brain-map.org/grid_data/download/74821852?include=energy
Rab36	0.809	http://api.brain-map.org/grid_data/download/69817829?include=energy
Ccl2	0.809	http://api.brain-map.org/grid_data/download/70526067?include=energy
Ampd2	0.809	http://api.brain-map.org/grid_data/download/69134244?include=energy
Polr3a	0.809	http://api.brain-map.org/grid_data/download/68843787?include=energy
Olfr1120	0.809	http://api.brain-map.org/grid_data/download/73635532?include=energy
Tlr2	0.809	http://api.brain-map.org/grid_data/download/77792773?include=energy
D630010B17Rik	0.809	http://api.brain-map.org/grid_data/download/74510128?include=energy
Ift80	0.809	http://api.brain-map.org/grid_data/download/67815966?include=energy
Kalrn	0.809	http://api.brain-map.org/grid_data/download/73930821?include=energy
5330438I03Rik	0.809	http://api.brain-map.org/grid_data/download/71493735?include=energy
Psph	0.809	http://api.brain-map.org/grid_data/download/68269067?include=energy
Zfp36l2	0.809	http://api.brain-map.org/grid_data/download/70812948?include=energy
Bace1	0.809	http://api.brain-map.org/grid_data/download/73520965?include=energy
Pth2r	0.809	http://api.brain-map.org/grid_data/download/69863250?include=energy
Agps	0.808	http://api.brain-map.org/grid_data/download/74819325?include=energy
Ptrh2	0.808	http://api.brain-map.org/grid_data/download/68633318?include=energy
B230317F23Rik	0.808	http://api.brain-map.org/grid_data/download/69527824?include=energy
1190005F20Rik	0.808	http://api.brain-map.org/grid_data/download/69133647?include=energy
Midn	0.808	http://api.brain-map.org/grid_data/download/71670739?include=energy
Pag1	0.808	http://api.brain-map.org/grid_data/download/71280964?include=energy
3110070M22Rik	0.808	http://api.brain-map.org/grid_data/download/69407499?include=energy
LOC433584	0.808	http://api.brain-map.org/grid_data/download/74635809?include=energy
Trdv2-2	0.808	http://api.brain-map.org/grid_data/download/71494714?include=energy
1700011F14Rik	0.808	http://api.brain-map.org/grid_data/download/69113859?include=energy
4930579C12Rik	0.808	http://api.brain-map.org/grid_data/download/71808853?include=energy
Mm.214315	0.808	http://api.brain-map.org/grid_data/download/71152467?include=energy
Gpsm1	0.808	http://api.brain-map.org/grid_data/download/69169827?include=energy
Pctp	0.808	http://api.brain-map.org/grid_data/download/76081009?include=energy
6330405D24Rik	0.808	http://api.brain-map.org/grid_data/download/73513320?include=energy
Pcdhb13	0.808	http://api.brain-map.org/grid_data/download/74743259?include=energy
Trp53inp2	0.808	http://api.brain-map.org/grid_data/download/70192081?include=energy
Gm5126	0.808	http://api.brain-map.org/grid_data/download/70595889?include=energy
Prl	0.808	http://api.brain-map.org/grid_data/download/75861792?include=energy
Tmem223	0.808	http://api.brain-map.org/grid_data/download/69352724?include=energy
Fnip2	0.808	http://api.brain-map.org/grid_data/download/74882227?include=energy
Pus7	0.808	http://api.brain-map.org/grid_data/download/79556638?include=energy
BC060267	0.808	http://api.brain-map.org/grid_data/download/68845269?include=energy
Reep6	0.808	http://api.brain-map.org/grid_data/download/68638053?include=energy
Tes	0.808	http://api.brain-map.org/grid_data/download/413801?include=energy
1700071A11Rik	0.808	http://api.brain-map.org/grid_data/download/71492886?include=energy
Fam43a	0.808	http://api.brain-map.org/grid_data/download/72472748?include=energy
Fut1	0.808	http://api.brain-map.org/grid_data/download/69672623?include=energy
Dpf3	0.808	http://api.brain-map.org/grid_data/download/72129252?include=energy
Fbxo33	0.808	http://api.brain-map.org/grid_data/download/71381034?include=energy
Prss45	0.808	http://api.brain-map.org/grid_data/download/72182966?include=energy
Gm4792	0.808	http://api.brain-map.org/grid_data/download/75990858?include=energy
Tab2	0.808	http://api.brain-map.org/grid_data/download/68191336?include=energy
AF251705	0.808	http://api.brain-map.org/grid_data/download/76097704?include=energy
9930022D16Rik	0.808	http://api.brain-map.org/grid_data/download/70228263?include=energy
Tm6sf1	0.808	http://api.brain-map.org/grid_data/download/71836883?include=energy
Ccdc90a	0.808	http://api.brain-map.org/grid_data/download/69444771?include=energy
Acad10	0.808	http://api.brain-map.org/grid_data/download/68499022?include=energy
Stat2	0.808	http://api.brain-map.org/grid_data/download/74425571?include=energy
Rapgef2	0.808	http://api.brain-map.org/grid_data/download/70300995?include=energy
Nkapl	0.808	http://api.brain-map.org/grid_data/download/69407785?include=energy
2510012J08Rik	0.808	http://api.brain-map.org/grid_data/download/71147840?include=energy
Pde4a	0.808	http://api.brain-map.org/grid_data/download/1806?include=energy
Krcc1	0.808	http://api.brain-map.org/grid_data/download/633270?include=energy
LOC433495	0.807	http://api.brain-map.org/grid_data/download/70723325?include=energy
Mep1a	0.807	http://api.brain-map.org/grid_data/download/68341448?include=energy
Tbx2	0.807	http://api.brain-map.org/grid_data/download/70593396?include=energy
Olfr1361	0.807	http://api.brain-map.org/grid_data/download/73635604?include=energy
Zfp362	0.807	http://api.brain-map.org/grid_data/download/152?include=energy
Ltbp4	0.807	http://api.brain-map.org/grid_data/download/76085748?include=energy
Fuk	0.807	http://api.brain-map.org/grid_data/download/385778?include=energy
Tox	0.807	http://api.brain-map.org/grid_data/download/71670691?include=energy
Egln1	0.807	http://api.brain-map.org/grid_data/download/77340476?include=energy
2610015P09Rik	0.807	http://api.brain-map.org/grid_data/download/70295649?include=energy
Olfr796	0.807	http://api.brain-map.org/grid_data/download/74734944?include=energy
Crisp3	0.807	http://api.brain-map.org/grid_data/download/67815965?include=energy
Cog7	0.807	http://api.brain-map.org/grid_data/download/71764873?include=energy
Efna4	0.807	http://api.brain-map.org/grid_data/download/77925721?include=energy
Vmn1r85	0.807	http://api.brain-map.org/grid_data/download/70302980?include=energy
Tnfrsf9	0.807	http://api.brain-map.org/grid_data/download/68545471?include=energy
Sephs2	0.807	http://api.brain-map.org/grid_data/download/68845544?include=energy
Eif3b	0.807	http://api.brain-map.org/grid_data/download/68637856?include=energy
Hnmt	0.807	http://api.brain-map.org/grid_data/download/68744845?include=energy
Acn9	0.807	http://api.brain-map.org/grid_data/download/69514666?include=energy
Ccrn4l	0.807	http://api.brain-map.org/grid_data/download/69540537?include=energy
1700113P08Rik	0.807	http://api.brain-map.org/grid_data/download/71211326?include=energy
5430440L12Rik	0.807	http://api.brain-map.org/grid_data/download/71147912?include=energy
Chrdl1	0.807	http://api.brain-map.org/grid_data/download/71021153?include=energy
Uchl4	0.807	http://api.brain-map.org/grid_data/download/70302884?include=energy
Morc4	0.807	http://api.brain-map.org/grid_data/download/72004483?include=energy
Fgfr3	0.807	http://api.brain-map.org/grid_data/download/448?include=energy
Msh6	0.807	http://api.brain-map.org/grid_data/download/70546221?include=energy
6820421L05Rik*	0.807	http://api.brain-map.org/grid_data/download/73906059?include=energy
D11Wsu47e	0.807	http://api.brain-map.org/grid_data/download/74819575?include=energy
D14Ertd449e	0.807	http://api.brain-map.org/grid_data/download/68638131?include=energy
Tmem198b	0.807	http://api.brain-map.org/grid_data/download/68795423?include=energy
Adamts8	0.807	http://api.brain-map.org/grid_data/download/73925719?include=energy
Sntg1	0.807	http://api.brain-map.org/grid_data/download/72002025?include=energy
Cox17	0.807	http://api.brain-map.org/grid_data/download/74000678?include=energy
Lpar6	0.807	http://api.brain-map.org/grid_data/download/69258292?include=energy
Dcun1d1	0.807	http://api.brain-map.org/grid_data/download/71612208?include=energy
Arsb	0.807	http://api.brain-map.org/grid_data/download/71023008?include=energy
Sept6	0.807	http://api.brain-map.org/grid_data/download/532648?include=energy
Pygo2	0.807	http://api.brain-map.org/grid_data/download/68845627?include=energy
Cblc	0.807	http://api.brain-map.org/grid_data/download/68924715?include=energy
Fxyd7	0.807	http://api.brain-map.org/grid_data/download/73592536?include=energy
Mysm1	0.807	http://api.brain-map.org/grid_data/download/69530828?include=energy
Htatsf1	0.807	http://api.brain-map.org/grid_data/download/69059923?include=energy
Gm1758	0.807	http://api.brain-map.org/grid_data/download/74735319?include=energy
Vmn1r234	0.807	http://api.brain-map.org/grid_data/download/69442235?include=energy
Lypd5	0.807	http://api.brain-map.org/grid_data/download/71991702?include=energy
Rdm1	0.807	http://api.brain-map.org/grid_data/download/69015733?include=energy
Sybu	0.807	http://api.brain-map.org/grid_data/download/70301086?include=energy
6430601O08Rik*	0.807	http://api.brain-map.org/grid_data/download/73870635?include=energy
Zfp512	0.807	http://api.brain-map.org/grid_data/download/69407140?include=energy
Pfkl	0.807	http://api.brain-map.org/grid_data/download/795?include=energy
Tmem189	0.807	http://api.brain-map.org/grid_data/download/69735986?include=energy
4930541O19Rik	0.807	http://api.brain-map.org/grid_data/download/74510248?include=energy
A330053M17Rik*	0.806	http://api.brain-map.org/grid_data/download/73949626?include=energy
Mtmr3	0.806	http://api.brain-map.org/grid_data/download/70546227?include=energy
Afap1	0.806	http://api.brain-map.org/grid_data/download/74425569?include=energy
Lrrc19	0.806	http://api.brain-map.org/grid_data/download/75986090?include=energy
Nip7	0.806	http://api.brain-map.org/grid_data/download/513470?include=energy
LOC545785	0.806	http://api.brain-map.org/grid_data/download/71158958?include=energy
Lect2	0.806	http://api.brain-map.org/grid_data/download/68446075?include=energy
Klre1	0.806	http://api.brain-map.org/grid_data/download/72283665?include=energy
Tssc4	0.806	http://api.brain-map.org/grid_data/download/71020951?include=energy
AI837181	0.806	http://api.brain-map.org/grid_data/download/634459?include=energy
Ccnb3	0.806	http://api.brain-map.org/grid_data/download/76081076?include=energy
4833420G17Rik	0.806	http://api.brain-map.org/grid_data/download/74047840?include=energy
Aqp12	0.806	http://api.brain-map.org/grid_data/download/69855894?include=energy
Zw10	0.806	http://api.brain-map.org/grid_data/download/69549655?include=energy
LOC546676	0.806	http://api.brain-map.org/grid_data/download/74736474?include=energy
Mc1r	0.806	http://api.brain-map.org/grid_data/download/69258124?include=energy
Irf2bp1	0.806	http://api.brain-map.org/grid_data/download/70431403?include=energy
Aass	0.806	http://api.brain-map.org/grid_data/download/77464956?include=energy
Kdm4c	0.806	http://api.brain-map.org/grid_data/download/641526?include=energy
Hira	0.806	http://api.brain-map.org/grid_data/download/73497174?include=energy
Tnfsf13b	0.806	http://api.brain-map.org/grid_data/download/73617534?include=energy
Kcnma1	0.806	http://api.brain-map.org/grid_data/download/74578206?include=energy
Gfra2	0.806	http://api.brain-map.org/grid_data/download/72472805?include=energy
LOC385403	0.806	http://api.brain-map.org/grid_data/download/74736153?include=energy
Sdr16c5	0.806	http://api.brain-map.org/grid_data/download/71281758?include=energy
Rit1	0.806	http://api.brain-map.org/grid_data/download/70795898?include=energy

Plcg1	0.806	http://api.brain-map.org/grid_data/download/74300789?include=energy
Mettl21d	0.806	http://api.brain-map.org/grid_data/download/74359730?include=energy
Gm505	0.806	http://api.brain-map.org/grid_data/download/71764655?include=energy
Rps10	0.806	http://api.brain-map.org/grid_data/download/70473987?include=energy
Dnajb14	0.806	http://api.brain-map.org/grid_data/download/69443493?include=energy
BC049702	0.806	http://api.brain-map.org/grid_data/download/70525770?include=energy
Wfdc3	0.806	http://api.brain-map.org/grid_data/download/70609173?include=energy
Pcdha9	0.806	http://api.brain-map.org/grid_data/download/79354814?include=energy
Klhl23	0.806	http://api.brain-map.org/grid_data/download/69530834?include=energy
4933415F23Rik	0.806	http://api.brain-map.org/grid_data/download/70218774?include=energy
Palb2	0.806	http://api.brain-map.org/grid_data/download/69134340?include=energy
2310003C23Rik	0.806	http://api.brain-map.org/grid_data/download/68845209?include=energy
Olfr1101	0.806	http://api.brain-map.org/grid_data/download/73635520?include=energy
Zfp182	0.806	http://api.brain-map.org/grid_data/download/68862036?include=energy
LOC432567	0.806	http://api.brain-map.org/grid_data/download/74635743?include=energy
Plbd1	0.806	http://api.brain-map.org/grid_data/download/68410387?include=energy
Bola2	0.806	http://api.brain-map.org/grid_data/download/74427089?include=energy
Car7	0.806	http://api.brain-map.org/grid_data/download/74511736?include=energy
Il1f6	0.806	http://api.brain-map.org/grid_data/download/71919134?include=energy
B930042J22Rik*	0.806	http://api.brain-map.org/grid_data/download/74000606?include=energy
Gm1082	0.806	http://api.brain-map.org/grid_data/download/69672910?include=energy
Rgs18	0.806	http://api.brain-map.org/grid_data/download/75990414?include=energy
Ift140	0.806	http://api.brain-map.org/grid_data/download/71495710?include=energy
Ocel1	0.806	http://api.brain-map.org/grid_data/download/74580895?include=energy
Heatr5a	0.806	http://api.brain-map.org/grid_data/download/69626588?include=energy
1810043H04Rik	0.806	http://api.brain-map.org/grid_data/download/71211290?include=energy
Ubl4	0.806	http://api.brain-map.org/grid_data/download/75774670?include=energy
Tmem213	0.806	http://api.brain-map.org/grid_data/download/70238692?include=energy
Apol6	0.806	http://api.brain-map.org/grid_data/download/69816422?include=energy
Mgat4a	0.806	http://api.brain-map.org/grid_data/download/70300324?include=energy
F730043M19Rik	0.806	http://api.brain-map.org/grid_data/download/73731247?include=energy
Wdr25	0.806	http://api.brain-map.org/grid_data/download/69528022?include=energy
Tbx10	0.806	http://api.brain-map.org/grid_data/download/100144890?include=energy
Degs2	0.806	http://api.brain-map.org/grid_data/download/68666643?include=energy
Eif1ad	0.805	http://api.brain-map.org/grid_data/download/68445077?include=energy
Frmpd4	0.805	http://api.brain-map.org/grid_data/download/73931421?include=energy
4933414I15Rik	0.805	http://api.brain-map.org/grid_data/download/70218768?include=energy
A530016L24Rik	0.805	http://api.brain-map.org/grid_data/download/75828171?include=energy
Syne2	0.805	http://api.brain-map.org/grid_data/download/74640880?include=energy
Rabggtb	0.805	http://api.brain-map.org/grid_data/download/80473485?include=energy
Thsd4	0.805	http://api.brain-map.org/grid_data/download/69527776?include=energy
Rfc2	0.805	http://api.brain-map.org/grid_data/download/70473975?include=energy
Lyn	0.805	http://api.brain-map.org/grid_data/download/68563122?include=energy
Psmc6	0.805	http://api.brain-map.org/grid_data/download/73512205?include=energy
Sec24d	0.805	http://api.brain-map.org/grid_data/download/69262332?include=energy
4930541J03Rik*	0.805	http://api.brain-map.org/grid_data/download/73906497?include=energy
Snrnp40	0.805	http://api.brain-map.org/grid_data/download/68844194?include=energy
LOC436510	0.805	http://api.brain-map.org/grid_data/download/71022708?include=energy
Eif1b	0.805	http://api.brain-map.org/grid_data/download/75651231?include=energy
LOC435882	0.805	http://api.brain-map.org/grid_data/download/71022603?include=energy
Rai2	0.805	http://api.brain-map.org/grid_data/download/69117092?include=energy
Mrps17	0.805	http://api.brain-map.org/grid_data/download/68191480?include=energy
Kcnv2	0.805	http://api.brain-map.org/grid_data/download/1764?include=energy
Supt3h	0.805	http://api.brain-map.org/grid_data/download/69816948?include=energy
1700036A12Rik	0.805	http://api.brain-map.org/grid_data/download/77799037?include=energy
Bhlhe23	0.805	http://api.brain-map.org/grid_data/download/70231815?include=energy
Spatc1	0.805	http://api.brain-map.org/grid_data/download/74635096?include=energy
Cdc14b	0.805	http://api.brain-map.org/grid_data/download/76081085?include=energy
Cd2	0.805	http://api.brain-map.org/grid_data/download/68910982?include=energy
Gpatch1	0.805	http://api.brain-map.org/grid_data/download/69734524?include=energy
Shoc2	0.805	http://api.brain-map.org/grid_data/download/73519850?include=energy
Mm.330624	0.805	http://api.brain-map.org/grid_data/download/71160277?include=energy
1810062G17Rik	0.805	http://api.brain-map.org/grid_data/download/74359329?include=energy
Ptpla	0.805	http://api.brain-map.org/grid_data/download/69817667?include=energy
Prdm10	0.805	http://api.brain-map.org/grid_data/download/70300905?include=energy
Gm5510	0.805	http://api.brain-map.org/grid_data/download/74736207?include=energy
9130229H14Rik	0.805	http://api.brain-map.org/grid_data/download/70717373?include=energy
4921522P10Rik	0.805	http://api.brain-map.org/grid_data/download/76080930?include=energy
Cyb5	0.805	http://api.brain-map.org/grid_data/download/75043146?include=energy
Mamstr	0.805	http://api.brain-map.org/grid_data/download/100144868?include=energy
Orm1	0.805	http://api.brain-map.org/grid_data/download/68301294?include=energy
Shc2	0.805	http://api.brain-map.org/grid_data/download/71152563?include=energy
Izumo1	0.805	http://api.brain-map.org/grid_data/download/73716507?include=energy
Nfya	0.805	http://api.brain-map.org/grid_data/download/70919215?include=energy
Lilrb3	0.805	http://api.brain-map.org/grid_data/download/71280595?include=energy
Fap	0.805	http://api.brain-map.org/grid_data/download/70473891?include=energy
H3f3b	0.805	http://api.brain-map.org/grid_data/download/70545976?include=energy
Bms1	0.805	http://api.brain-map.org/grid_data/download/68918922?include=energy
Rnf125	0.805	http://api.brain-map.org/grid_data/download/75990415?include=energy
Rnf151	0.805	http://api.brain-map.org/grid_data/download/75851062?include=energy
5730419I09Rik	0.805	http://api.brain-map.org/grid_data/download/68795407?include=energy
Clca3	0.805	http://api.brain-map.org/grid_data/download/68563031?include=energy
Muc1	0.805	http://api.brain-map.org/grid_data/download/68443214?include=energy
Mfi2	0.805	http://api.brain-map.org/grid_data/download/68563098?include=energy
Uhrf2	0.805	http://api.brain-map.org/grid_data/download/70445287?include=energy
Il17f	0.804	http://api.brain-map.org/grid_data/download/70278027?include=energy
Arl13b	0.804	http://api.brain-map.org/grid_data/download/71892448?include=energy
Bcl6	0.804	http://api.brain-map.org/grid_data/download/75651139?include=energy
Gm5885	0.804	http://api.brain-map.org/grid_data/download/70566094?include=energy
Cga	0.804	http://api.brain-map.org/grid_data/download/69548876?include=energy
Pan2	0.804	http://api.brain-map.org/grid_data/download/70194654?include=energy
LOC432942	0.804	http://api.brain-map.org/grid_data/download/74736195?include=energy
Il5	0.804	http://api.brain-map.org/grid_data/download/69735413?include=energy
C1qbp	0.804	http://api.brain-map.org/grid_data/download/70528408?include=energy
Pi4k2b	0.804	http://api.brain-map.org/grid_data/download/73423953?include=energy
Anapc10	0.804	http://api.brain-map.org/grid_data/download/68637551?include=energy
Rnf138	0.804	http://api.brain-map.org/grid_data/download/75990838?include=energy
Mm.258279	0.804	http://api.brain-map.org/grid_data/download/71735071?include=energy
Akr1c6	0.804	http://api.brain-map.org/grid_data/download/70946159?include=energy
Ranbp6	0.804	http://api.brain-map.org/grid_data/download/73497648?include=energy
4933427D06Rik	0.804	http://api.brain-map.org/grid_data/download/70218804?include=energy
5830428M24Rik	0.804	http://api.brain-map.org/grid_data/download/72076683?include=energy
Crip2	0.804	http://api.brain-map.org/grid_data/download/70612727?include=energy
Rd3	0.804	http://api.brain-map.org/grid_data/download/69407517?include=energy
Slfn1	0.804	http://api.brain-map.org/grid_data/download/100146156?include=energy
Akr1b8	0.804	http://api.brain-map.org/grid_data/download/68300944?include=energy
Gpr97	0.804	http://api.brain-map.org/grid_data/download/69257672?include=energy
Ddx51	0.804	http://api.brain-map.org/grid_data/download/70946450?include=energy
LOC432517	0.804	http://api.brain-map.org/grid_data/download/74514306?include=energy
Gm606	0.804	http://api.brain-map.org/grid_data/download/73617648?include=energy
Ifi27l2a	0.804	http://api.brain-map.org/grid_data/download/70218727?include=energy
Golph3	0.804	http://api.brain-map.org/grid_data/download/70794037?include=energy
8030423F21Rik	0.804	http://api.brain-map.org/grid_data/download/69445623?include=energy
Tet1	0.804	http://api.brain-map.org/grid_data/download/70238034?include=energy
Samm50	0.804	http://api.brain-map.org/grid_data/download/70598377?include=energy
Rps6ka2	0.804	http://api.brain-map.org/grid_data/download/70301276?include=energy
Ptpn1	0.804	http://api.brain-map.org/grid_data/download/76003580?include=energy
Olfr714	0.804	http://api.brain-map.org/grid_data/download/74734878?include=energy
Rin1	0.804	http://api.brain-map.org/grid_data/download/67870468?include=energy
Il23a	0.804	http://api.brain-map.org/grid_data/download/587404?include=energy
Ctdsp2	0.804	http://api.brain-map.org/grid_data/download/68498986?include=energy
Gfod1	0.804	http://api.brain-map.org/grid_data/download/69526426?include=energy
Foxp4	0.804	http://api.brain-map.org/grid_data/download/70946123?include=energy
Herpud2	0.804	http://api.brain-map.org/grid_data/download/68196928?include=energy
Casc3	0.804	http://api.brain-map.org/grid_data/download/69540387?include=energy
Cpne2	0.804	http://api.brain-map.org/grid_data/download/70743844?include=energy
2310081J21Rik	0.804	http://api.brain-map.org/grid_data/download/70542864?include=energy
LOC436200	0.804	http://api.brain-map.org/grid_data/download/74736324?include=energy
LOC432405	0.804	http://api.brain-map.org/grid_data/download/70300150?include=energy
LOC433499	0.804	http://api.brain-map.org/grid_data/download/70723331?include=energy
Olfr332	0.804	http://api.brain-map.org/grid_data/download/74721012?include=energy
Olfr613	0.804	http://api.brain-map.org/grid_data/download/74734734?include=energy
Ints2	0.804	http://api.brain-map.org/grid_data/download/69837985?include=energy
Rpl22	0.804	http://api.brain-map.org/grid_data/download/68445143?include=energy
Clca6	0.804	http://api.brain-map.org/grid_data/download/71486995?include=energy
Cnot4	0.804	http://api.brain-map.org/grid_data/download/68745054?include=energy
Pnrc2	0.804	http://api.brain-map.org/grid_data/download/2491?include=energy
4933403O08Rik	0.804	http://api.brain-map.org/grid_data/download/70717364?include=energy
1700054A03Rik	0.803	http://api.brain-map.org/grid_data/download/71920129?include=energy
LOC433699	0.803	http://api.brain-map.org/grid_data/download/70725555?include=energy
Gm4358	0.803	http://api.brain-map.org/grid_data/download/70295948?include=energy
Npw	0.803	http://api.brain-map.org/grid_data/download/74635785?include=energy
Sirt6	0.803	http://api.brain-map.org/grid_data/download/68798501?include=energy
Gm14200	0.803	http://api.brain-map.org/grid_data/download/71148154?include=energy
Eif2s1	0.803	http://api.brain-map.org/grid_data/download/67765729?include=energy
Slc16a14	0.803	http://api.brain-map.org/grid_data/download/2577?include=energy
A930018P22Rik	0.803	http://api.brain-map.org/grid_data/download/72081592?include=energy
Pou2f1	0.803	http://api.brain-map.org/grid_data/download/1563?include=energy
6030499A19Rik*	0.803	http://api.brain-map.org/grid_data/download/73808279?include=energy
Cyp2c37	0.803	http://api.brain-map.org/grid_data/download/69028366?include=energy
Sprr4	0.803	http://api.brain-map.org/grid_data/download/73573205?include=energy
Fcf1	0.803	http://api.brain-map.org/grid_data/download/68523360?include=energy
Rsph10b2	0.803	http://api.brain-map.org/grid_data/download/69442329?include=energy
Tmem134	0.803	http://api.brain-map.org/grid_data/download/70474917?include=energy
Iqcd	0.803	http://api.brain-map.org/grid_data/download/70218828?include=energy
Mm.211000	0.803	http://api.brain-map.org/grid_data/download/71669005?include=energy
Cdk20	0.803	http://api.brain-map.org/grid_data/download/68151445?include=energy
Zglp1	0.803	http://api.brain-map.org/grid_data/download/100144104?include=energy
Tti1	0.803	http://api.brain-map.org/grid_data/download/68911029?include=energy
Zfp146	0.803	http://api.brain-map.org/grid_data/download/69290806?include=energy
2810055G20Rik	0.803	http://api.brain-map.org/grid_data/download/70303827?include=energy
Setbp1	0.803	http://api.brain-map.org/grid_data/download/1532?include=energy
Stau1	0.803	http://api.brain-map.org/grid_data/download/68301258?include=energy
Lrrc52	0.803	http://api.brain-map.org/grid_data/download/72283420?include=energy
Gm5111	0.803	http://api.brain-map.org/grid_data/download/71569196?include=energy
Rhoq	0.803	http://api.brain-map.org/grid_data/download/68924455?include=energy
D330012F22Rik	0.803	http://api.brain-map.org/grid_data/download/68191198?include=energy
Azin1	0.803	http://api.brain-map.org/grid_data/download/74047917?include=energy
Ggt1	0.803	http://api.brain-map.org/grid_data/download/68301210?include=energy
Hist1h3f	0.803	http://api.brain-map.org/grid_data/download/69816589?include=energy
Tmem210	0.803	http://api.brain-map.org/grid_data/download/70218408?include=energy
Akr1b10	0.803	http://api.brain-map.org/grid_data/download/678697?include=energy
Lmbr1l	0.803	http://api.brain-map.org/grid_data/download/68080686?include=energy
Abcg4	0.803	http://api.brain-map.org/grid_data/download/634455?include=energy
Zxda	0.803	http://api.brain-map.org/grid_data/download/75985952?include=energy
Matn1	0.803	http://api.brain-map.org/grid_data/download/68745576?include=energy
Zzef1	0.803	http://api.brain-map.org/grid_data/download/70200818?include=energy
A630012P03Rik	0.803	http://api.brain-map.org/grid_data/download/70228525?include=energy
Dcbld1	0.803	http://api.brain-map.org/grid_data/download/67814206?include=energy
Gm4874	0.803	http://api.brain-map.org/grid_data/download/70566088?include=energy
2310065F04Rik	0.803	http://api.brain-map.org/grid_data/download/72283360?include=energy
Abcb11	0.803	http://api.brain-map.org/grid_data/download/76003608?include=energy
Ifitm7	0.803	http://api.brain-map.org/grid_data/download/70278003?include=energy
Gm9758	0.803	http://api.brain-map.org/grid_data/download/69113817?include=energy
6430502G17Rik	0.803	http://api.brain-map.org/grid_data/download/71147942?include=energy
Ufc1	0.803	http://api.brain-map.org/grid_data/download/1637?include=energy
Dusp1	0.803	http://api.brain-map.org/grid_data/download/324212?include=energy
Rab26	0.803	http://api.brain-map.org/grid_data/download/69817817?include=energy
Phrf1	0.803	http://api.brain-map.org/grid_data/download/71495173?include=energy
Adnp	0.803	http://api.brain-map.org/grid_data/download/70919764?include=energy
Znrf2	0.802	http://api.brain-map.org/grid_data/download/76003370?include=energy
Bach1	0.802	http://api.brain-map.org/grid_data/download/77455015?include=energy
Vps26b	0.802	http://api.brain-map.org/grid_data/download/68745263?include=energy
Ubtd1	0.802	http://api.brain-map.org/grid_data/download/71654133?include=energy
Ptk2b	0.802	http://api.brain-map.org/grid_data/download/74357569?include=energy
Evpl	0.802	http://api.brain-map.org/grid_data/download/74635120?include=energy
Crh	0.802	http://api.brain-map.org/grid_data/download/292?include=energy
BC062185	0.802	http://api.brain-map.org/grid_data/download/70719030?include=energy
Lipc	0.802	http://api.brain-map.org/grid_data/download/671236?include=energy
4931409K22Rik	0.802	http://api.brain-map.org/grid_data/download/74431272?include=energy
Rnf44	0.802	http://api.brain-map.org/grid_data/download/378545?include=energy
Chd7	0.802	http://api.brain-map.org/grid_data/download/71836884?include=energy
Ankrd7	0.802	http://api.brain-map.org/grid_data/download/69114498?include=energy
Gngt1	0.802	http://api.brain-map.org/grid_data/download/74822826?include=energy
Lcat	0.802	http://api.brain-map.org/grid_data/download/75042269?include=energy
Fam164a	0.802	http://api.brain-map.org/grid_data/download/71281274?include=energy
Fam108a	0.802	http://api.brain-map.org/grid_data/download/69549674?include=energy
Ighg2a	0.802	http://api.brain-map.org/grid_data/download/77620257?include=energy
Efr3a	0.802	http://api.brain-map.org/grid_data/download/79568028?include=energy
Gm11733	0.802	http://api.brain-map.org/grid_data/download/71213837?include=energy
Eps8l1	0.802	http://api.brain-map.org/grid_data/download/1707?include=energy
TC1451722	0.802	http://api.brain-map.org/grid_data/download/72472384?include=energy
1110032A03Rik	0.802	http://api.brain-map.org/grid_data/download/68631923?include=energy
Slc9a2	0.802	http://api.brain-map.org/grid_data/download/74749943?include=energy
Lrrn3	0.802	http://api.brain-map.org/grid_data/download/75041464?include=energy
Gins2	0.802	http://api.brain-map.org/grid_data/download/69407756?include=energy
Svs5	0.802	http://api.brain-map.org/grid_data/download/69887499?include=energy
2310007L24Rik	0.802	http://api.brain-map.org/grid_data/download/71991756?include=energy
Hivep2	0.802	http://api.brain-map.org/grid_data/download/70795922?include=energy
LOC432973	0.802	http://api.brain-map.org/grid_data/download/74514180?include=energy
Qser1	0.802	http://api.brain-map.org/grid_data/download/69407714?include=energy
Nr1d1	0.802	http://api.brain-map.org/grid_data/download/1243?include=energy
Agxt	0.802	http://api.brain-map.org/grid_data/download/68499075?include=energy
Accn3	0.802	http://api.brain-map.org/grid_data/download/75831754?include=energy
Tmem194b	0.802	http://api.brain-map.org/grid_data/download/71493729?include=energy
C230096K16Rik	0.802	http://api.brain-map.org/grid_data/download/73811693?include=energy
Mrpl1	0.802	http://api.brain-map.org/grid_data/download/70813269?include=energy
Tufm	0.802	http://api.brain-map.org/grid_data/download/70919651?include=energy
Kcnj9	0.802	http://api.brain-map.org/grid_data/download/72119580?include=energy
Hdac7	0.802	http://api.brain-map.org/grid_data/download/75077262?include=energy
Gvin1	0.802	http://api.brain-map.org/grid_data/download/70528121?include=energy
Ces4a	0.802	http://api.brain-map.org/grid_data/download/68498513?include=energy
Psd2	0.802	http://api.brain-map.org/grid_data/download/73712870?include=energy
Fam187b	0.802	http://api.brain-map.org/grid_data/download/71919182?include=energy
Alg3	0.801	http://api.brain-map.org/grid_data/download/68442847?include=energy
Zbtb10	0.801	http://api.brain-map.org/grid_data/download/70195096?include=energy
Polr3k	0.801	http://api.brain-map.org/grid_data/download/70439087?include=energy
Ptpn20	0.801	http://api.brain-map.org/grid_data/download/69817679?include=energy
LOC675709	0.801	http://api.brain-map.org/grid_data/download/74277437?include=energy
Pdlim5	0.801	http://api.brain-map.org/grid_data/download/68191216?include=energy
Txndc15	0.801	http://api.brain-map.org/grid_data/download/69835610?include=energy
A730055L17Rik	0.801	http://api.brain-map.org/grid_data/download/69448990?include=energy
Lrrc8b	0.801	http://api.brain-map.org/grid_data/download/70787095?include=energy
Cplx4	0.801	http://api.brain-map.org/grid_data/download/68342258?include=energy
Trp53tg5	0.801	http://api.brain-map.org/grid_data/download/71492904?include=energy
Pum2	0.801	http://api.brain-map.org/grid_data/download/68845514?include=energy
Timd2	0.801	http://api.brain-map.org/grid_data/download/68545276?include=energy
Lsm12	0.801	http://api.brain-map.org/grid_data/download/75692954?include=energy
Tnfsf12	0.801	http://api.brain-map.org/grid_data/download/69981175?include=energy
LOC434786	0.801	http://api.brain-map.org/grid_data/download/74736273?include=energy
Sel1l2	0.801	http://api.brain-map.org/grid_data/download/72472442?include=energy
Gas7	0.801	http://api.brain-map.org/grid_data/download/73992909?include=energy
Fdxr	0.801	http://api.brain-map.org/grid_data/download/73512553?include=energy
Nkx3-1	0.801	http://api.brain-map.org/grid_data/download/75934937?include=energy
Fer1l5	0.801	http://api.brain-map.org/grid_data/download/74562534?include=energy
Hbp1	0.801	http://api.brain-map.org/grid_data/download/69015452?include=energy
Rnf41	0.801	http://api.brain-map.org/grid_data/download/70439117?include=energy
Fn1	0.801	http://api.brain-map.org/grid_data/download/72119593?include=energy
1810029B16Rik	0.801	http://api.brain-map.org/grid_data/download/68744898?include=energy
4930557A04Rik	0.801	http://api.brain-map.org/grid_data/download/73513290?include=energy
Aoah	0.801	http://api.brain-map.org/grid_data/download/69526677?include=energy
Pak6	0.801	http://api.brain-map.org/grid_data/download/75084501?include=energy
Tead4	0.801	http://api.brain-map.org/grid_data/download/70812799?include=energy
G930032B20Rik*	0.801	http://api.brain-map.org/grid_data/download/73716746?include=energy
Cldn10	0.801	http://api.brain-map.org/grid_data/download/68148695?include=energy
Klhdc5	0.801	http://api.brain-map.org/grid_data/download/70787414?include=energy
LOC434292	0.801	http://api.brain-map.org/grid_data/download/75991088?include=energy

Lypd4	0.801	http://api.brain-map.org/grid_data/download/69114286?include=energy
Olfr1323	0.801	http://api.brain-map.org/grid_data/download/74720717?include=energy
Kctd21	0.801	http://api.brain-map.org/grid_data/download/74000660?include=energy
B130024G19Rik	0.801	http://api.brain-map.org/grid_data/download/74277748?include=energy
Svs1	0.801	http://api.brain-map.org/grid_data/download/70302294?include=energy
Rab11fip2	0.801	http://api.brain-map.org/grid_data/download/70300977?include=energy
Pcdha5	0.8	http://api.brain-map.org/grid_data/download/79354808?include=energy
Olfr576	0.8	http://api.brain-map.org/grid_data/download/74724217?include=energy
Capn9	0.8	http://api.brain-map.org/grid_data/download/68745072?include=energy
Abtb1	0.8	http://api.brain-map.org/grid_data/download/1659?include=energy
Cntrob	0.8	http://api.brain-map.org/grid_data/download/68862102?include=energy
Rapgef4	0.8	http://api.brain-map.org/grid_data/download/74749895?include=energy
Noc4l	0.8	http://api.brain-map.org/grid_data/download/532677?include=energy
LOC433962	0.8	http://api.brain-map.org/grid_data/download/70787170?include=energy
Apobr	0.8	http://api.brain-map.org/grid_data/download/71724804?include=energy
Gpr61	0.8	http://api.brain-map.org/grid_data/download/69257606?include=energy
Slc30a10	0.8	http://api.brain-map.org/grid_data/download/72283599?include=energy
Tdrd6	0.8	http://api.brain-map.org/grid_data/download/75990417?include=energy
Bri3	0.8	http://api.brain-map.org/grid_data/download/70528396?include=energy
F830201C10Rik*	0.8	http://api.brain-map.org/grid_data/download/74427105?include=energy
Ly6g5b	0.8	http://api.brain-map.org/grid_data/download/71992103?include=energy
Sepsecs	0.8	http://api.brain-map.org/grid_data/download/69608971?include=energy
C530043A13Rik	0.8	http://api.brain-map.org/grid_data/download/71021025?include=energy
Gemin7	0.8	http://api.brain-map.org/grid_data/download/70545925?include=energy
Spink6	0.8	http://api.brain-map.org/grid_data/download/71891989?include=energy
Lgals9	0.8	http://api.brain-map.org/grid_data/download/69059800?include=energy
Palm2	0.8	http://api.brain-map.org/grid_data/download/74581441?include=energy
AA792892	0.8	http://api.brain-map.org/grid_data/download/70228669?include=energy
Ccnf	0.8	http://api.brain-map.org/grid_data/download/68795429?include=energy
Casp7	0.8	http://api.brain-map.org/grid_data/download/68443092?include=energy
Folr2	0.8	http://api.brain-map.org/grid_data/download/1717?include=energy
Slc31a1	0.8	http://api.brain-map.org/grid_data/download/2603?include=energy
Klf17	0.8	http://api.brain-map.org/grid_data/download/69352706?include=energy
Edem3	0.8	http://api.brain-map.org/grid_data/download/70539223?include=energy
Isx	0.8	http://api.brain-map.org/grid_data/download/71377730?include=energy
Etv5	0.8	http://api.brain-map.org/grid_data/download/70613989?include=energy
A230059G12Rik	0.8	http://api.brain-map.org/grid_data/download/70717563?include=energy
1810030N24Rik	0.8	http://api.brain-map.org/grid_data/download/68744875?include=energy
Sema5a	0.8	http://api.brain-map.org/grid_data/download/950?include=energy
Grpel1	0.8	http://api.brain-map.org/grid_data/download/413800?include=energy
Kctd2	0.8	http://api.brain-map.org/grid_data/download/71281807?include=energy
Panx1	0.8	http://api.brain-map.org/grid_data/download/69782712?include=energy
Rab27b	0.8	http://api.brain-map.org/grid_data/download/74641314?include=energy
Ermp1	0.8	http://api.brain-map.org/grid_data/download/68444791?include=energy
LOC546352	0.8	http://api.brain-map.org/grid_data/download/75214739?include=energy
Sertad3	0.8	http://api.brain-map.org/grid_data/download/69549610?include=energy
Lhx6	0.8	http://api.brain-map.org/grid_data/download/635?include=energy
Chad	0.8	http://api.brain-map.org/grid_data/download/68301335?include=energy
Itpripl2	0.8	http://api.brain-map.org/grid_data/download/73811651?include=energy
Nkx1-1	0.8	http://api.brain-map.org/grid_data/download/81790666?include=energy
Slc2a10	0.8	http://api.brain-map.org/grid_data/download/100145322?include=energy
2310001H17Rik	0.8	http://api.brain-map.org/grid_data/download/69202340?include=energy
Afap1l1	0.8	http://api.brain-map.org/grid_data/download/71158919?include=energy
Vsig10	0.799	http://api.brain-map.org/grid_data/download/70719028?include=energy
Ramp2	0.799	http://api.brain-map.org/grid_data/download/71378504?include=energy
Cox11	0.799	http://api.brain-map.org/grid_data/download/70528551?include=energy
Unc13b	0.799	http://api.brain-map.org/grid_data/download/72004454?include=energy
Hrk	0.799	http://api.brain-map.org/grid_data/download/73513576?include=energy
Rabl5	0.799	http://api.brain-map.org/grid_data/download/70439093?include=energy
BC038156	0.799	http://api.brain-map.org/grid_data/download/69529089?include=energy
Rnf115	0.799	http://api.brain-map.org/grid_data/download/70743900?include=energy
1810074P20Rik	0.799	http://api.brain-map.org/grid_data/download/15?include=energy
Siglece	0.799	http://api.brain-map.org/grid_data/download/1832?include=energy
Zfp354a	0.799	http://api.brain-map.org/grid_data/download/68666816?include=energy
A830073O21Rik	0.799	http://api.brain-map.org/grid_data/download/74819036?include=energy
Psmd9	0.799	http://api.brain-map.org/grid_data/download/69262237?include=energy
Apex2	0.799	http://api.brain-map.org/grid_data/download/70528234?include=energy
LOC434468	0.799	http://api.brain-map.org/grid_data/download/71022361?include=energy
Gsg1	0.799	http://api.brain-map.org/grid_data/download/68441610?include=energy
Gpr15	0.799	http://api.brain-map.org/grid_data/download/71378727?include=energy
4933434M16Rik	0.799	http://api.brain-map.org/grid_data/download/71892179?include=energy
Olfr1410	0.799	http://api.brain-map.org/grid_data/download/73653314?include=energy
Hpx	0.799	http://api.brain-map.org/grid_data/download/75826671?include=energy
Cd276	0.799	http://api.brain-map.org/grid_data/download/68633351?include=energy
Zbtb3	0.799	http://api.brain-map.org/grid_data/download/69290740?include=energy
Nlrp3	0.799	http://api.brain-map.org/grid_data/download/71021155?include=energy
Gm5753	0.799	http://api.brain-map.org/grid_data/download/71281973?include=energy
Trappc1	0.799	http://api.brain-map.org/grid_data/download/71670701?include=energy
Dnahc5	0.799	http://api.brain-map.org/grid_data/download/69626939?include=energy
Cyp20a1	0.799	http://api.brain-map.org/grid_data/download/68797589?include=energy
Krt5	0.799	http://api.brain-map.org/grid_data/download/68795455?include=energy
Amelx	0.799	http://api.brain-map.org/grid_data/download/100146164?include=energy
Htr7	0.799	http://api.brain-map.org/grid_data/download/71393436?include=energy
Dennd1c	0.799	http://api.brain-map.org/grid_data/download/68498241?include=energy
Plekho2	0.799	http://api.brain-map.org/grid_data/download/68498220?include=energy
Pzp	0.799	http://api.brain-map.org/grid_data/download/69015212?include=energy
1110008J03Rik	0.799	http://api.brain-map.org/grid_data/download/68445694?include=energy
1700011J10Rik	0.799	http://api.brain-map.org/grid_data/download/74821754?include=energy
Rasgrf2	0.799	http://api.brain-map.org/grid_data/download/73992916?include=energy
Cyp7a1	0.799	http://api.brain-map.org/grid_data/download/75197612?include=energy
Pcbd1	0.799	http://api.brain-map.org/grid_data/download/1802?include=energy
Zfhx2as	0.799	http://api.brain-map.org/grid_data/download/74363340?include=energy
Tmc4	0.799	http://api.brain-map.org/grid_data/download/71789933?include=energy
Zfr2	0.799	http://api.brain-map.org/grid_data/download/70717372?include=energy
Gm13420	0.799	http://api.brain-map.org/grid_data/download/75693178?include=energy
mCG147059	0.799	http://api.brain-map.org/grid_data/download/73635407?include=energy
Gnai1	0.799	http://api.brain-map.org/grid_data/download/72472806?include=energy
Olfr1188	0.799	http://api.brain-map.org/grid_data/download/74720633?include=energy
Clec3b	0.799	http://api.brain-map.org/grid_data/download/68545039?include=energy
C130022M03Rik	0.799	http://api.brain-map.org/grid_data/download/69530780?include=energy
Krt23	0.799	http://api.brain-map.org/grid_data/download/68341955?include=energy
B630005N14Rik	0.799	http://api.brain-map.org/grid_data/download/69527968?include=energy
Cux1	0.799	http://api.brain-map.org/grid_data/download/79556618?include=energy
Prl2c2	0.798	http://api.brain-map.org/grid_data/download/75077152?include=energy
Ms4a4c	0.798	http://api.brain-map.org/grid_data/download/68744642?include=energy
Calhm3	0.798	http://api.brain-map.org/grid_data/download/71489810?include=energy
Erlec1	0.798	http://api.brain-map.org/grid_data/download/583854?include=energy
Slc41a3	0.798	http://api.brain-map.org/grid_data/download/73512484?include=energy
Zfp715	0.798	http://api.brain-map.org/grid_data/download/71612181?include=energy
Nmrk2	0.798	http://api.brain-map.org/grid_data/download/70278099?include=energy
BC027092	0.798	http://api.brain-map.org/grid_data/download/71724834?include=energy
Csmd1	0.798	http://api.brain-map.org/grid_data/download/77798997?include=energy
Zfp27	0.798	http://api.brain-map.org/grid_data/download/70445335?include=energy
Slc7a7	0.798	http://api.brain-map.org/grid_data/download/74431002?include=energy
Tdh	0.798	http://api.brain-map.org/grid_data/download/68667025?include=energy
Ppm1e	0.798	http://api.brain-map.org/grid_data/download/100144389?include=energy
8430408J09Rik	0.798	http://api.brain-map.org/grid_data/download/71209835?include=energy
Hnrnpf	0.798	http://api.brain-map.org/grid_data/download/68637503?include=energy
Fam171a2	0.798	http://api.brain-map.org/grid_data/download/70528324?include=energy
Hs3st2	0.798	http://api.brain-map.org/grid_data/download/74988765?include=energy
Ints5	0.798	http://api.brain-map.org/grid_data/download/67815936?include=energy
Cdh11	0.798	http://api.brain-map.org/grid_data/download/232?include=energy
Vdr	0.798	http://api.brain-map.org/grid_data/download/112204135?include=energy
Cdh20	0.798	http://api.brain-map.org/grid_data/download/77464878?include=energy
Higd1c	0.798	http://api.brain-map.org/grid_data/download/73617666?include=energy
Rnf182	0.798	http://api.brain-map.org/grid_data/download/70719034?include=energy
LOC547026	0.798	http://api.brain-map.org/grid_data/download/71211043?include=energy
Gm9776	0.798	http://api.brain-map.org/grid_data/download/73949593?include=energy
Pdcd4	0.798	http://api.brain-map.org/grid_data/download/73931413?include=energy
4930529M08Rik	0.798	http://api.brain-map.org/grid_data/download/69442335?include=energy
B020004C17Rik	0.798	http://api.brain-map.org/grid_data/download/70725519?include=energy
Fam177a	0.798	http://api.brain-map.org/grid_data/download/71280907?include=energy
Lage3	0.798	http://api.brain-map.org/grid_data/download/70918808?include=energy
Phf13	0.798	http://api.brain-map.org/grid_data/download/69289703?include=energy
Arhgap30	0.798	http://api.brain-map.org/grid_data/download/73513429?include=energy
Gm2a	0.798	http://api.brain-map.org/grid_data/download/74988549?include=energy
Mcc	0.798	http://api.brain-map.org/grid_data/download/70298304?include=energy
Krtap16-8	0.798	http://api.brain-map.org/grid_data/download/75198118?include=energy
Slc25a30	0.798	http://api.brain-map.org/grid_data/download/69095952?include=energy
Ubash3a	0.798	http://api.brain-map.org/grid_data/download/1086?include=energy
Baiap2l2	0.798	http://api.brain-map.org/grid_data/download/74431104?include=energy
Clca4	0.798	http://api.brain-map.org/grid_data/download/70593135?include=energy
Tcl1	0.798	http://api.brain-map.org/grid_data/download/68667730?include=energy
A130095K04Rik*	0.798	http://api.brain-map.org/grid_data/download/73808516?include=energy
Gm5590	0.798	http://api.brain-map.org/grid_data/download/70787546?include=energy
Usp12	0.798	http://api.brain-map.org/grid_data/download/77620806?include=energy
C1galt1	0.798	http://api.brain-map.org/grid_data/download/68743775?include=energy
Vps16	0.798	http://api.brain-map.org/grid_data/download/631711?include=energy
Cr1l	0.798	http://api.brain-map.org/grid_data/download/2125?include=energy
Piwil2	0.798	http://api.brain-map.org/grid_data/download/70300810?include=energy
Elac1	0.798	http://api.brain-map.org/grid_data/download/641545?include=energy
Calml3	0.798	http://api.brain-map.org/grid_data/download/79490064?include=energy
Fam26f	0.798	http://api.brain-map.org/grid_data/download/70228579?include=energy
LOC245498	0.798	http://api.brain-map.org/grid_data/download/70595738?include=energy
Cdc42ep1	0.797	http://api.brain-map.org/grid_data/download/70814345?include=energy
Lsm3	0.797	http://api.brain-map.org/grid_data/download/69838556?include=energy
Rc3h2	0.797	http://api.brain-map.org/grid_data/download/70946614?include=energy
Nkx6-1	0.797	http://api.brain-map.org/grid_data/download/81657661?include=energy
Olig3	0.797	http://api.brain-map.org/grid_data/download/71656779?include=energy
Tmem93	0.797	http://api.brain-map.org/grid_data/download/68410518?include=energy
Zfp9	0.797	http://api.brain-map.org/grid_data/download/70445365?include=energy
Rpap1	0.797	http://api.brain-map.org/grid_data/download/69818293?include=energy
Zmat3	0.797	http://api.brain-map.org/grid_data/download/70785726?include=energy
Gm11961	0.797	http://api.brain-map.org/grid_data/download/71569226?include=energy
Alox5ap	0.797	http://api.brain-map.org/grid_data/download/68744534?include=energy
Catsper1	0.797	http://api.brain-map.org/grid_data/download/71307226?include=energy
Ypel3	0.797	http://api.brain-map.org/grid_data/download/75214942?include=energy
Hoxc12	0.797	http://api.brain-map.org/grid_data/download/71393394?include=energy
Panx3	0.797	http://api.brain-map.org/grid_data/download/68668114?include=energy
Polq	0.797	http://api.brain-map.org/grid_data/download/76081017?include=energy
Rab8a	0.797	http://api.brain-map.org/grid_data/download/71325390?include=energy
Rnft2	0.797	http://api.brain-map.org/grid_data/download/68918964?include=energy
Fetub	0.797	http://api.brain-map.org/grid_data/download/68638119?include=energy
Pla2g4f	0.797	http://api.brain-map.org/grid_data/download/69407696?include=energy
Tas2r109	0.797	http://api.brain-map.org/grid_data/download/70785158?include=energy
Cdc14a	0.797	http://api.brain-map.org/grid_data/download/77464968?include=energy
Rag2	0.797	http://api.brain-map.org/grid_data/download/69817916?include=energy
Pcdhga3	0.797	http://api.brain-map.org/grid_data/download/70784872?include=energy
Orai2	0.797	http://api.brain-map.org/grid_data/download/73925718?include=energy
Tead1	0.797	http://api.brain-map.org/grid_data/download/75043139?include=energy
Egr2	0.797	http://api.brain-map.org/grid_data/download/72128749?include=energy
Strada	0.797	http://api.brain-map.org/grid_data/download/74819330?include=energy
LOC436511	0.797	http://api.brain-map.org/grid_data/download/71022714?include=energy
Slc25a40	0.797	http://api.brain-map.org/grid_data/download/69527812?include=energy
Treml4	0.797	http://api.brain-map.org/grid_data/download/75990425?include=energy
Cntnap3	0.797	http://api.brain-map.org/grid_data/download/71924451?include=energy
Olfr1178	0.797	http://api.brain-map.org/grid_data/download/74720621?include=energy
Tmem140	0.797	http://api.brain-map.org/grid_data/download/68341800?include=energy
2410080I02Rik	0.797	http://api.brain-map.org/grid_data/download/74635520?include=energy
Dctn4	0.797	http://api.brain-map.org/grid_data/download/67764937?include=energy
BC046331	0.797	http://api.brain-map.org/grid_data/download/68924817?include=energy
Spry3	0.797	http://api.brain-map.org/grid_data/download/70295990?include=energy
Tnk2	0.797	http://api.brain-map.org/grid_data/download/74056144?include=energy
Sirt1	0.797	http://api.brain-map.org/grid_data/download/69816858?include=energy
Nlrc3	0.797	http://api.brain-map.org/grid_data/download/74513983?include=energy
Pdcd10	0.797	http://api.brain-map.org/grid_data/download/74581295?include=energy
Htr5a	0.797	http://api.brain-map.org/grid_data/download/71393430?include=energy
Ntrk3	0.797	http://api.brain-map.org/grid_data/download/76115738?include=energy
Gm10382	0.797	http://api.brain-map.org/grid_data/download/73906515?include=energy
Slc7a15	0.797	http://api.brain-map.org/grid_data/download/74359335?include=energy
Ubash3b	0.797	http://api.brain-map.org/grid_data/download/74581370?include=energy
Pisd-ps3	0.797	http://api.brain-map.org/grid_data/download/71211701?include=energy
Znrd1	0.797	http://api.brain-map.org/grid_data/download/75985942?include=energy
Slco2b1	0.797	http://api.brain-map.org/grid_data/download/69874096?include=energy
Mesp2	0.797	http://api.brain-map.org/grid_data/download/69289345?include=energy
Sash1	0.797	http://api.brain-map.org/grid_data/download/71924225?include=energy
Btk	0.797	http://api.brain-map.org/grid_data/download/68667928?include=energy
Ncapg	0.797	http://api.brain-map.org/grid_data/download/71020961?include=energy
Mixl1	0.797	http://api.brain-map.org/grid_data/download/81657685?include=energy
Arhgap1	0.797	http://api.brain-map.org/grid_data/download/69526797?include=energy
Tnrc18	0.797	http://api.brain-map.org/grid_data/download/73424495?include=energy
Pcdhac2	0.797	http://api.brain-map.org/grid_data/download/71456119?include=energy
Krt1	0.797	http://api.brain-map.org/grid_data/download/69735950?include=energy
Pyroxd1	0.797	http://api.brain-map.org/grid_data/download/71612418?include=energy
Lsm2	0.797	http://api.brain-map.org/grid_data/download/68301114?include=energy
Gpd1	0.796	http://api.brain-map.org/grid_data/download/74586667?include=energy
D830035M03Rik	0.796	http://api.brain-map.org/grid_data/download/70238770?include=energy
Psg28	0.796	http://api.brain-map.org/grid_data/download/74277432?include=energy
1500001M20Rik	0.796	http://api.brain-map.org/grid_data/download/70598393?include=energy
Wdr62	0.796	http://api.brain-map.org/grid_data/download/71213075?include=energy
3110047P20Rik	0.796	http://api.brain-map.org/grid_data/download/73771233?include=energy
Upk1b	0.796	http://api.brain-map.org/grid_data/download/68745269?include=energy
Efha2	0.796	http://api.brain-map.org/grid_data/download/71023032?include=energy
Siae	0.796	http://api.brain-map.org/grid_data/download/324008?include=energy
Myocd	0.796	http://api.brain-map.org/grid_data/download/76081911?include=energy
Olfr599	0.796	http://api.brain-map.org/grid_data/download/74724235?include=energy
Slc16a3	0.796	http://api.brain-map.org/grid_data/download/72080085?include=energy
E330009J07Rik	0.796	http://api.brain-map.org/grid_data/download/74658077?include=energy
LOC434061	0.796	http://api.brain-map.org/grid_data/download/70787239?include=energy
Shisa4	0.796	http://api.brain-map.org/grid_data/download/72283504?include=energy
Glmn	0.796	http://api.brain-map.org/grid_data/download/67978475?include=energy
Cxcl17	0.796	http://api.brain-map.org/grid_data/download/68499491?include=energy
Gabrb3	0.796	http://api.brain-map.org/grid_data/download/75551468?include=energy
Wdr89	0.796	http://api.brain-map.org/grid_data/download/69854734?include=energy
Gla	0.796	http://api.brain-map.org/grid_data/download/71723943?include=energy
Get4	0.796	http://api.brain-map.org/grid_data/download/601190?include=energy
AI467606	0.796	http://api.brain-map.org/grid_data/download/71656675?include=energy
C230096C10Rik	0.796	http://api.brain-map.org/grid_data/download/69530941?include=energy
Xpnpep3	0.796	http://api.brain-map.org/grid_data/download/75988400?include=energy
Med12	0.796	http://api.brain-map.org/grid_data/download/68844098?include=energy
Acsm3	0.796	http://api.brain-map.org/grid_data/download/393238?include=energy
LOC544977	0.796	http://api.brain-map.org/grid_data/download/74736402?include=energy
Bbox1	0.796	http://api.brain-map.org/grid_data/download/68410393?include=energy
Zfp663	0.796	http://api.brain-map.org/grid_data/download/73497568?include=energy
Hmx2	0.796	http://api.brain-map.org/grid_data/download/68203440?include=energy
Snrpd3	0.796	http://api.brain-map.org/grid_data/download/69885588?include=energy
Slfnl1	0.796	http://api.brain-map.org/grid_data/download/72182957?include=energy
Slc35d2	0.796	http://api.brain-map.org/grid_data/download/71764697?include=energy
4930459I23Rik	0.796	http://api.brain-map.org/grid_data/download/100144872?include=energy
C330024E10Rik	0.796	http://api.brain-map.org/grid_data/download/74300735?include=energy
4921519B16Rik*	0.796	http://api.brain-map.org/grid_data/download/73870527?include=energy
F730219E24Rik*	0.796	http://api.brain-map.org/grid_data/download/73767187?include=energy
Bean1	0.796	http://api.brain-map.org/grid_data/download/70305490?include=energy
Snrnp27	0.796	http://api.brain-map.org/grid_data/download/68545848?include=energy
Zfp251	0.796	http://api.brain-map.org/grid_data/download/74580871?include=energy
Zfp867	0.796	http://api.brain-map.org/grid_data/download/70528366?include=energy
2310007J06Rik	0.796	http://api.brain-map.org/grid_data/download/71022936?include=energy
Eml3	0.796	http://api.brain-map.org/grid_data/download/71015805?include=energy
Atpbd4	0.796	http://api.brain-map.org/grid_data/download/68151410?include=energy
Pif1	0.796	http://api.brain-map.org/grid_data/download/68924685?include=energy
5031434C07Rik	0.796	http://api.brain-map.org/grid_data/download/70218872?include=energy
Tcap	0.796	http://api.brain-map.org/grid_data/download/69261409?include=energy
Kctd7	0.796	http://api.brain-map.org/grid_data/download/71488722?include=energy
Mela	0.796	http://api.brain-map.org/grid_data/download/74658083?include=energy
Mertk	0.796	http://api.brain-map.org/grid_data/download/69752185?include=energy
Mm.375144	0.796	http://api.brain-map.org/grid_data/download/71210130?include=energy
Fgd1	0.795	http://api.brain-map.org/grid_data/download/70429250?include=energy

Itpkc	0.795	http://api.brain-map.org/grid_data/download/69549300?include=energy
Cdh16	0.795	http://api.brain-map.org/grid_data/download/79541429?include=energy
Rnpepl1	0.795	http://api.brain-map.org/grid_data/download/67792185?include=energy
Trp53rk	0.795	http://api.brain-map.org/grid_data/download/68342372?include=energy
Tada2b	0.795	http://api.brain-map.org/grid_data/download/71213665?include=energy
Ythdf1	0.795	http://api.brain-map.org/grid_data/download/69835624?include=energy
Olfr1350	0.795	http://api.brain-map.org/grid_data/download/74720747?include=energy
C230003A10Rik*	0.795	http://api.brain-map.org/grid_data/download/74000624?include=energy
Cd27	0.795	http://api.brain-map.org/grid_data/download/69981157?include=energy
Lrriq1	0.795	http://api.brain-map.org/grid_data/download/73584795?include=energy
Wt1	0.795	http://api.brain-map.org/grid_data/download/77464976?include=energy
Plcb3	0.795	http://api.brain-map.org/grid_data/download/68632942?include=energy
Lrrc26	0.795	http://api.brain-map.org/grid_data/download/69095966?include=energy
LOC380945	0.795	http://api.brain-map.org/grid_data/download/68546179?include=energy
Aipl1	0.795	http://api.brain-map.org/grid_data/download/79360276?include=energy
Neurod6	0.795	http://api.brain-map.org/grid_data/download/698?include=energy
Rcvrn	0.795	http://api.brain-map.org/grid_data/download/69818054?include=energy
1700067P10Rik	0.795	http://api.brain-map.org/grid_data/download/69113698?include=energy
Edar	0.795	http://api.brain-map.org/grid_data/download/71280562?include=energy
Lrrc14b	0.795	http://api.brain-map.org/grid_data/download/70611293?include=energy
Chek1	0.795	http://api.brain-map.org/grid_data/download/75988361?include=energy
Map3k14	0.795	http://api.brain-map.org/grid_data/download/71808729?include=energy
Mybl2	0.795	http://api.brain-map.org/grid_data/download/68798489?include=energy
Unc5d	0.795	http://api.brain-map.org/grid_data/download/71924239?include=energy
Acpl2	0.795	http://api.brain-map.org/grid_data/download/80343348?include=energy
Snrpe	0.795	http://api.brain-map.org/grid_data/download/68844606?include=energy
Aph1b	0.795	http://api.brain-map.org/grid_data/download/71281527?include=energy
Gba	0.795	http://api.brain-map.org/grid_data/download/1611?include=energy
Wdfy2	0.795	http://api.brain-map.org/grid_data/download/72109399?include=energy
Gm9991	0.795	http://api.brain-map.org/grid_data/download/74277701?include=energy
4932443I19Rik	0.795	http://api.brain-map.org/grid_data/download/73674078?include=energy
Mrpl9	0.795	http://api.brain-map.org/grid_data/download/71020802?include=energy
Gm340	0.795	http://api.brain-map.org/grid_data/download/70298513?include=energy
Nol11	0.795	http://api.brain-map.org/grid_data/download/67787338?include=energy
5430425J12Rik	0.795	http://api.brain-map.org/grid_data/download/74580781?include=energy
1700024P04Rik	0.795	http://api.brain-map.org/grid_data/download/73716477?include=energy
D330041B21Rik	0.795	http://api.brain-map.org/grid_data/download/73513684?include=energy
C130022K22Rik	0.795	http://api.brain-map.org/grid_data/download/70528402?include=energy
Serpina3b	0.795	http://api.brain-map.org/grid_data/download/70228304?include=energy
Rnf144b	0.795	http://api.brain-map.org/grid_data/download/71836839?include=energy
Frmpd3	0.795	http://api.brain-map.org/grid_data/download/70305466?include=energy
Ctage5	0.795	http://api.brain-map.org/grid_data/download/67792199?include=energy
Mitd1	0.795	http://api.brain-map.org/grid_data/download/70445419?include=energy
LOC545181	0.794	http://api.brain-map.org/grid_data/download/74736420?include=energy
Ms4a18	0.794	http://api.brain-map.org/grid_data/download/71892274?include=energy
Pvrl1	0.794	http://api.brain-map.org/grid_data/download/74363351?include=energy
Grp	0.794	http://api.brain-map.org/grid_data/download/1363?include=energy
8430431K14Rik	0.794	http://api.brain-map.org/grid_data/download/74822576?include=energy
Ttc28	0.794	http://api.brain-map.org/grid_data/download/72283336?include=energy
Aacs	0.794	http://api.brain-map.org/grid_data/download/74988454?include=energy
Mm.108566	0.794	http://api.brain-map.org/grid_data/download/71147900?include=energy
Rbpms2	0.794	http://api.brain-map.org/grid_data/download/73512496?include=energy
Golga7b	0.794	http://api.brain-map.org/grid_data/download/76097699?include=energy
K230012H24Rik*	0.794	http://api.brain-map.org/grid_data/download/73767271?include=energy
Fam46a	0.794	http://api.brain-map.org/grid_data/download/73520967?include=energy
Cdk6	0.794	http://api.brain-map.org/grid_data/download/69540695?include=energy
Arglu1	0.794	http://api.brain-map.org/grid_data/download/70528139?include=energy
Prr3	0.794	http://api.brain-map.org/grid_data/download/512753?include=energy
Ncf4	0.794	http://api.brain-map.org/grid_data/download/68498932?include=energy
Asb2	0.794	http://api.brain-map.org/grid_data/download/71283613?include=energy
0610030E20Rik	0.794	http://api.brain-map.org/grid_data/download/69352782?include=energy
Gstm1	0.794	http://api.brain-map.org/grid_data/download/68443202?include=energy
Hopx	0.794	http://api.brain-map.org/grid_data/download/79556716?include=energy
Tdgf1	0.794	http://api.brain-map.org/grid_data/download/68667679?include=energy
Gm5432	0.794	http://api.brain-map.org/grid_data/download/70611150?include=energy
LOC433928	0.794	http://api.brain-map.org/grid_data/download/70787101?include=energy
6430500J09Rik*	0.794	http://api.brain-map.org/grid_data/download/73927631?include=energy
Cggbp1	0.794	http://api.brain-map.org/grid_data/download/68919958?include=energy
Tbcel	0.794	http://api.brain-map.org/grid_data/download/69671708?include=energy
Ubac1	0.794	http://api.brain-map.org/grid_data/download/69080513?include=energy
F10	0.794	http://api.brain-map.org/grid_data/download/632503?include=energy
Mcm8	0.794	http://api.brain-map.org/grid_data/download/69837488?include=energy
6330512M04Rik	0.794	http://api.brain-map.org/grid_data/download/70528115?include=energy
Odf2	0.794	http://api.brain-map.org/grid_data/download/68918952?include=energy
4931407G18Rik	0.794	http://api.brain-map.org/grid_data/download/70525780?include=energy
Zfp820	0.794	http://api.brain-map.org/grid_data/download/68445895?include=energy
1700024B05Rik	0.794	http://api.brain-map.org/grid_data/download/71378046?include=energy
Tbrg4	0.794	http://api.brain-map.org/grid_data/download/68151444?include=energy
Tcea3	0.794	http://api.brain-map.org/grid_data/download/1870?include=energy
Dhx33	0.794	http://api.brain-map.org/grid_data/download/68666852?include=energy
Eya3	0.794	http://api.brain-map.org/grid_data/download/68844588?include=energy
Col4a2	0.794	http://api.brain-map.org/grid_data/download/73592540?include=energy
Hspb1	0.794	http://api.brain-map.org/grid_data/download/74363357?include=energy
2310011J03Rik	0.794	http://api.brain-map.org/grid_data/download/68080683?include=energy
Serpinb3b	0.794	http://api.brain-map.org/grid_data/download/74821945?include=energy
Thnsl2	0.794	http://api.brain-map.org/grid_data/download/68498890?include=energy
Camkk1	0.794	http://api.brain-map.org/grid_data/download/73930840?include=energy
S100a14	0.794	http://api.brain-map.org/grid_data/download/68160847?include=energy
Retn	0.794	http://api.brain-map.org/grid_data/download/68743972?include=energy
Sf3b1	0.794	http://api.brain-map.org/grid_data/download/69873612?include=energy
Wwc1	0.794	http://api.brain-map.org/grid_data/download/71020388?include=energy
Naalad2	0.794	http://api.brain-map.org/grid_data/download/71280901?include=energy
Rad51c	0.794	http://api.brain-map.org/grid_data/download/70300989?include=energy
AF085738	0.794	http://api.brain-map.org/grid_data/download/70230813?include=energy
Ppp1r2-ps7	0.794	http://api.brain-map.org/grid_data/download/71378036?include=energy
Dpyd	0.794	http://api.brain-map.org/grid_data/download/68191204?include=energy
A430072C10Rik	0.794	http://api.brain-map.org/grid_data/download/70228424?include=energy
Gpr26	0.794	http://api.brain-map.org/grid_data/download/70562057?include=energy
Gins1	0.794	http://api.brain-map.org/grid_data/download/75828186?include=energy
Robo4	0.794	http://api.brain-map.org/grid_data/download/69169481?include=energy
Rab11fip4	0.794	http://api.brain-map.org/grid_data/download/74300777?include=energy
BC049762	0.794	http://api.brain-map.org/grid_data/download/69116409?include=energy
Map3k2	0.794	http://api.brain-map.org/grid_data/download/69752011?include=energy
S100a10	0.794	http://api.brain-map.org/grid_data/download/70560277?include=energy
0610037P05Rik	0.794	http://api.brain-map.org/grid_data/download/68193049?include=energy
4933407E24Rik	0.794	http://api.brain-map.org/grid_data/download/74265243?include=energy
Crtc2	0.793	http://api.brain-map.org/grid_data/download/67787327?include=energy
Loh12cr1	0.793	http://api.brain-map.org/grid_data/download/68196940?include=energy
C130075H21Rik*	0.793	http://api.brain-map.org/grid_data/download/73811645?include=energy
Gpc6	0.793	http://api.brain-map.org/grid_data/download/1736?include=energy
G630041M05Rik	0.793	http://api.brain-map.org/grid_data/download/69289021?include=energy
Tas2r114	0.793	http://api.brain-map.org/grid_data/download/74277433?include=energy
Gdf1	0.793	http://api.brain-map.org/grid_data/download/75081205?include=energy
Fam5b	0.793	http://api.brain-map.org/grid_data/download/71249743?include=energy
Bud13	0.793	http://api.brain-map.org/grid_data/download/70717353?include=energy
E330013L01Rik*	0.793	http://api.brain-map.org/grid_data/download/74427121?include=energy
Tspyl3	0.793	http://api.brain-map.org/grid_data/download/69104156?include=energy
Rilpl2	0.793	http://api.brain-map.org/grid_data/download/67978511?include=energy
Samd8	0.793	http://api.brain-map.org/grid_data/download/70813155?include=energy
Trp53i11	0.793	http://api.brain-map.org/grid_data/download/73521826?include=energy
Map4k1	0.793	http://api.brain-map.org/grid_data/download/68203509?include=energy
Apoa5	0.793	http://api.brain-map.org/grid_data/download/75826660?include=energy
Cryl1	0.793	http://api.brain-map.org/grid_data/download/77620762?include=energy
Olfr982	0.793	http://api.brain-map.org/grid_data/download/74277399?include=energy
Tlr7	0.793	http://api.brain-map.org/grid_data/download/70302533?include=energy
Ing3	0.793	http://api.brain-map.org/grid_data/download/73679026?include=energy
Rtbdn	0.793	http://api.brain-map.org/grid_data/download/68498830?include=energy
Rpa1	0.793	http://api.brain-map.org/grid_data/download/68193041?include=energy
LOC435618	0.793	http://api.brain-map.org/grid_data/download/71022543?include=energy
F830118D09Rik*	0.793	http://api.brain-map.org/grid_data/download/73748671?include=energy
Eef1d	0.793	http://api.brain-map.org/grid_data/download/73497547?include=energy
Gatad2b	0.793	http://api.brain-map.org/grid_data/download/71021020?include=energy
Tmem45b	0.793	http://api.brain-map.org/grid_data/download/68499296?include=energy
LOC434598	0.793	http://api.brain-map.org/grid_data/download/71022385?include=energy
4930442G15Rik	0.793	http://api.brain-map.org/grid_data/download/71891707?include=energy
Macrod1	0.793	http://api.brain-map.org/grid_data/download/633277?include=energy
Pla2g12b	0.793	http://api.brain-map.org/grid_data/download/68499153?include=energy
Tubal3	0.793	http://api.brain-map.org/grid_data/download/72471792?include=energy
Zbed4	0.793	http://api.brain-map.org/grid_data/download/68666923?include=energy
Cwc15	0.793	http://api.brain-map.org/grid_data/download/633672?include=energy
mCG145422	0.793	http://api.brain-map.org/grid_data/download/71212168?include=energy
Gen1	0.793	http://api.brain-map.org/grid_data/download/71493783?include=energy
mCG1049722.1	0.793	http://api.brain-map.org/grid_data/download/74750076?include=energy
Ipo8	0.793	http://api.brain-map.org/grid_data/download/69735508?include=energy
Mm.246494	0.793	http://api.brain-map.org/grid_data/download/71456140?include=energy
Pqlc1	0.793	http://api.brain-map.org/grid_data/download/70927829?include=energy
Runx2	0.793	http://api.brain-map.org/grid_data/download/1278?include=energy
Dhx15	0.793	http://api.brain-map.org/grid_data/download/69134499?include=energy
Impg1	0.793	http://api.brain-map.org/grid_data/download/2321?include=energy
Tnf	0.793	http://api.brain-map.org/grid_data/download/100142565?include=energy
B3gnt6	0.792	http://api.brain-map.org/grid_data/download/69529101?include=energy
Zfp623	0.792	http://api.brain-map.org/grid_data/download/68637981?include=energy
Gm17746	0.792	http://api.brain-map.org/grid_data/download/73931423?include=energy
Spert	0.792	http://api.brain-map.org/grid_data/download/71210556?include=energy
Taf4b	0.792	http://api.brain-map.org/grid_data/download/70302324?include=energy
Snf8	0.792	http://api.brain-map.org/grid_data/download/70474767?include=energy
Mcmbp	0.792	http://api.brain-map.org/grid_data/download/68444988?include=energy
Vmn1r227	0.792	http://api.brain-map.org/grid_data/download/70785630?include=energy
Crtap	0.792	http://api.brain-map.org/grid_data/download/68745179?include=energy
Ipo5	0.792	http://api.brain-map.org/grid_data/download/76115732?include=energy
Mmaa	0.792	http://api.brain-map.org/grid_data/download/1772?include=energy
Stt3b	0.792	http://api.brain-map.org/grid_data/download/71020390?include=energy
Gm4876	0.792	http://api.brain-map.org/grid_data/download/70305460?include=energy
Heatr3	0.792	http://api.brain-map.org/grid_data/download/69013161?include=energy
Cby3	0.792	http://api.brain-map.org/grid_data/download/70201534?include=energy
Nedd1	0.792	http://api.brain-map.org/grid_data/download/71234621?include=energy
Ube2r2	0.792	http://api.brain-map.org/grid_data/download/70474053?include=energy
Rom1	0.792	http://api.brain-map.org/grid_data/download/68443363?include=energy
Ada	0.792	http://api.brain-map.org/grid_data/download/70528192?include=energy
Fam103a1	0.792	http://api.brain-map.org/grid_data/download/68919934?include=energy
Plin1	0.792	http://api.brain-map.org/grid_data/download/74431408?include=energy
Pdgfb	0.792	http://api.brain-map.org/grid_data/download/71587782?include=energy
Trim38	0.792	http://api.brain-map.org/grid_data/download/70295679?include=energy
Mrps6	0.792	http://api.brain-map.org/grid_data/download/68443429?include=energy
Ctsr	0.792	http://api.brain-map.org/grid_data/download/75931622?include=energy
Jdp2	0.792	http://api.brain-map.org/grid_data/download/73930842?include=energy
Tmem63c	0.792	http://api.brain-map.org/grid_data/download/76115724?include=energy
Rptor	0.792	http://api.brain-map.org/grid_data/download/74562284?include=energy
Pigf	0.792	http://api.brain-map.org/grid_data/download/68563062?include=energy
Fkbp10	0.792	http://api.brain-map.org/grid_data/download/2220?include=energy
Raf1	0.792	http://api.brain-map.org/grid_data/download/1816?include=energy
A930038B10Rik	0.792	http://api.brain-map.org/grid_data/download/69514488?include=energy
B3gat1	0.792	http://api.brain-map.org/grid_data/download/73818755?include=energy
Olfr355	0.792	http://api.brain-map.org/grid_data/download/74724031?include=energy
Ikbkap	0.792	http://api.brain-map.org/grid_data/download/69013714?include=energy
Adad2	0.792	http://api.brain-map.org/grid_data/download/74579969?include=energy
Med7	0.792	http://api.brain-map.org/grid_data/download/68301766?include=energy
Cyp2g1	0.792	http://api.brain-map.org/grid_data/download/69835608?include=energy
Dydc2	0.792	http://api.brain-map.org/grid_data/download/69443238?include=energy
Trap1a	0.792	http://api.brain-map.org/grid_data/download/68666971?include=energy
Coq4	0.792	http://api.brain-map.org/grid_data/download/77792747?include=energy
C230012O17Rik	0.792	http://api.brain-map.org/grid_data/download/69530876?include=energy
1700016M24Rik	0.792	http://api.brain-map.org/grid_data/download/70201283?include=energy
Syt12	0.792	http://api.brain-map.org/grid_data/download/1291?include=energy
Jag2	0.792	http://api.brain-map.org/grid_data/download/69735652?include=energy
Arrdc2	0.792	http://api.brain-map.org/grid_data/download/68523330?include=energy
Pcdhga11	0.792	http://api.brain-map.org/grid_data/download/79354776?include=energy
F630040K05Rik	0.792	http://api.brain-map.org/grid_data/download/71281937?include=energy
Eed	0.792	http://api.brain-map.org/grid_data/download/74990485?include=energy
3830408C21Rik	0.792	http://api.brain-map.org/grid_data/download/73712810?include=energy
Cbl	0.791	http://api.brain-map.org/grid_data/download/69540423?include=energy
Ms4a13	0.791	http://api.brain-map.org/grid_data/download/71920171?include=energy
Cpeb1	0.791	http://api.brain-map.org/grid_data/download/72472770?include=energy
2410075B13Rik	0.791	http://api.brain-map.org/grid_data/download/68193015?include=energy
6430590A10Rik	0.791	http://api.brain-map.org/grid_data/download/69445367?include=energy
Poc5	0.791	http://api.brain-map.org/grid_data/download/686186?include=energy
Il18	0.791	http://api.brain-map.org/grid_data/download/68499255?include=energy
Olfr374	0.791	http://api.brain-map.org/grid_data/download/74724073?include=energy
Gm831	0.791	http://api.brain-map.org/grid_data/download/71764401?include=energy
Dmxl1	0.791	http://api.brain-map.org/grid_data/download/69531067?include=energy
Nexn	0.791	http://api.brain-map.org/grid_data/download/74581098?include=energy
Fam149b	0.791	http://api.brain-map.org/grid_data/download/68080706?include=energy
Lrrtm3	0.791	http://api.brain-map.org/grid_data/download/73931637?include=energy
LOC545070	0.791	http://api.brain-map.org/grid_data/download/74736408?include=energy
D130043K22Rik	0.791	http://api.brain-map.org/grid_data/download/69608495?include=energy
Lamc2	0.791	http://api.brain-map.org/grid_data/download/74988766?include=energy
BC053393	0.791	http://api.brain-map.org/grid_data/download/68667691?include=energy
Tnp1	0.791	http://api.brain-map.org/grid_data/download/69174280?include=energy
Tas2r116	0.791	http://api.brain-map.org/grid_data/download/73573241?include=energy
Zfp212	0.791	http://api.brain-map.org/grid_data/download/71608218?include=energy
Jagn1	0.791	http://api.brain-map.org/grid_data/download/68162259?include=energy
Entpd6	0.791	http://api.brain-map.org/grid_data/download/68546197?include=energy
Plxna2	0.791	http://api.brain-map.org/grid_data/download/70431431?include=energy
D1Pas1	0.791	http://api.brain-map.org/grid_data/download/70724655?include=energy
Ap2a1	0.791	http://api.brain-map.org/grid_data/download/75991159?include=energy
Zfp128	0.791	http://api.brain-map.org/grid_data/download/69290794?include=energy
Mamdc2	0.791	http://api.brain-map.org/grid_data/download/69262286?include=energy
Pknox2	0.791	http://api.brain-map.org/grid_data/download/76135815?include=energy
Il20	0.791	http://api.brain-map.org/grid_data/download/70278051?include=energy
Mib1	0.791	http://api.brain-map.org/grid_data/download/70546179?include=energy
Lrrc2	0.791	http://api.brain-map.org/grid_data/download/74821911?include=energy
Srpx2	0.791	http://api.brain-map.org/grid_data/download/68443387?include=energy
Ern2	0.791	http://api.brain-map.org/grid_data/download/72472798?include=energy
Bcorl1	0.791	http://api.brain-map.org/grid_data/download/69816500?include=energy
Gm6013	0.791	http://api.brain-map.org/grid_data/download/74736510?include=energy
Ccnjl	0.791	http://api.brain-map.org/grid_data/download/71832982?include=energy
Pld3	0.791	http://api.brain-map.org/grid_data/download/808?include=energy
Cpsf4	0.791	http://api.brain-map.org/grid_data/download/68843996?include=energy
Nif3l1	0.791	http://api.brain-map.org/grid_data/download/69782252?include=energy
Gm9796	0.791	http://api.brain-map.org/grid_data/download/75043611?include=energy
Dhrs7b	0.791	http://api.brain-map.org/grid_data/download/67978461?include=energy
Smok4a	0.791	http://api.brain-map.org/grid_data/download/70218217?include=energy
BC025446	0.791	http://api.brain-map.org/grid_data/download/68445931?include=energy
4933406J08Rik	0.791	http://api.brain-map.org/grid_data/download/70218655?include=energy
Pcdhac1	0.79	http://api.brain-map.org/grid_data/download/73512189?include=energy
Scap	0.79	http://api.brain-map.org/grid_data/download/71381109?include=energy
Pdha2	0.79	http://api.brain-map.org/grid_data/download/69782945?include=energy
Dennd3	0.79	http://api.brain-map.org/grid_data/download/69671678?include=energy
Tnpo1	0.79	http://api.brain-map.org/grid_data/download/68667910?include=energy
TC1410835	0.79	http://api.brain-map.org/grid_data/download/70793917?include=energy
Trim24	0.79	http://api.brain-map.org/grid_data/download/68843913?include=energy
Rpp30	0.79	http://api.brain-map.org/grid_data/download/69818369?include=energy
Cd19	0.79	http://api.brain-map.org/grid_data/download/69540555?include=energy
Morc1	0.79	http://api.brain-map.org/grid_data/download/68498762?include=energy
Kcnj3	0.79	http://api.brain-map.org/grid_data/download/72340132?include=energy
1110012L19Rik	0.79	http://api.brain-map.org/grid_data/download/70474797?include=energy
Pafah2	0.79	http://api.brain-map.org/grid_data/download/68077013?include=energy
Zadh2	0.79	http://api.brain-map.org/grid_data/download/69169857?include=energy
LOC432835	0.79	http://api.brain-map.org/grid_data/download/70611323?include=energy
Fam131b	0.79	http://api.brain-map.org/grid_data/download/72128774?include=energy
Mcm9	0.79	http://api.brain-map.org/grid_data/download/69752131?include=energy
Pramel3	0.79	http://api.brain-map.org/grid_data/download/75990412?include=energy
Htr6	0.79	http://api.brain-map.org/grid_data/download/69257981?include=energy
Taf12	0.79	http://api.brain-map.org/grid_data/download/641529?include=energy
Nap1l1	0.79	http://api.brain-map.org/grid_data/download/75198178?include=energy
4933406G16Rik	0.79	http://api.brain-map.org/grid_data/download/71892095?include=energy
Sgta	0.79	http://api.brain-map.org/grid_data/download/68076557?include=energy

Wfdc15b	0.79	http://api.brain-map.org/grid_data/download/69116745?include=energy
Slc35c1	0.79	http://api.brain-map.org/grid_data/download/70565246?include=energy
Zfr	0.79	http://api.brain-map.org/grid_data/download/69134616?include=energy
Gstt1	0.79	http://api.brain-map.org/grid_data/download/68302005?include=energy
Tmem240	0.79	http://api.brain-map.org/grid_data/download/75990890?include=energy
Slc6a1	0.79	http://api.brain-map.org/grid_data/download/476?include=energy
Lrguk	0.79	http://api.brain-map.org/grid_data/download/69407875?include=energy
Pbsn	0.79	http://api.brain-map.org/grid_data/download/74514231?include=energy
Rsph1	0.79	http://api.brain-map.org/grid_data/download/1078?include=energy
D430041D05Rik	0.79	http://api.brain-map.org/grid_data/download/72472777?include=energy
Dsc1	0.79	http://api.brain-map.org/grid_data/download/69671612?include=energy
4930524N10Rik	0.79	http://api.brain-map.org/grid_data/download/71891874?include=energy
Lhfpl2	0.79	http://api.brain-map.org/grid_data/download/72007934?include=energy
Clps	0.79	http://api.brain-map.org/grid_data/download/74822932?include=energy
Vmn1r81	0.79	http://api.brain-map.org/grid_data/download/70785696?include=energy
5530601H04Rik	0.79	http://api.brain-map.org/grid_data/download/71022858?include=energy
Nfix	0.79	http://api.brain-map.org/grid_data/download/2423?include=energy
Ust	0.79	http://api.brain-map.org/grid_data/download/71670734?include=energy
1700057G04Rik	0.79	http://api.brain-map.org/grid_data/download/72472463?include=energy
Cst9	0.79	http://api.brain-map.org/grid_data/download/69114797?include=energy
Grpr	0.79	http://api.brain-map.org/grid_data/download/81600602?include=energy
Txnrd2	0.79	http://api.brain-map.org/grid_data/download/68076977?include=energy
Cnrip1	0.79	http://api.brain-map.org/grid_data/download/71211578?include=energy
Lgals1	0.79	http://api.brain-map.org/grid_data/download/70634417?include=energy
Lrrc41	0.789	http://api.brain-map.org/grid_data/download/68441550?include=energy
Tmod4	0.789	http://api.brain-map.org/grid_data/download/76081038?include=energy
Mkrn1	0.789	http://api.brain-map.org/grid_data/download/67978685?include=energy
Epyc	0.789	http://api.brain-map.org/grid_data/download/513011?include=energy
Them4	0.789	http://api.brain-map.org/grid_data/download/69407803?include=energy
Nlrp10	0.789	http://api.brain-map.org/grid_data/download/71234530?include=energy
AA415398	0.789	http://api.brain-map.org/grid_data/download/68744122?include=energy
Fam115c	0.789	http://api.brain-map.org/grid_data/download/68193062?include=energy
1810009J06Rik	0.789	http://api.brain-map.org/grid_data/download/75991006?include=energy
LOC433474	0.789	http://api.brain-map.org/grid_data/download/70723283?include=energy
Gca	0.789	http://api.brain-map.org/grid_data/download/74641159?include=energy
4930592A05Rik	0.789	http://api.brain-map.org/grid_data/download/74580721?include=energy
Glrx	0.789	http://api.brain-map.org/grid_data/download/73931409?include=energy
Igj	0.789	http://api.brain-map.org/grid_data/download/68442961?include=energy
Zfp69	0.789	http://api.brain-map.org/grid_data/download/73497672?include=energy
Zfp92	0.789	http://api.brain-map.org/grid_data/download/69407200?include=energy
Celsr2	0.789	http://api.brain-map.org/grid_data/download/69237065?include=energy
Igsf6	0.789	http://api.brain-map.org/grid_data/download/69028444?include=energy
Ntn3	0.789	http://api.brain-map.org/grid_data/download/70300589?include=energy
Nup54	0.789	http://api.brain-map.org/grid_data/download/71234694?include=energy
Ankrd34a	0.789	http://api.brain-map.org/grid_data/download/74743507?include=energy
Cacna1d	0.789	http://api.brain-map.org/grid_data/download/68798037?include=energy
Vill	0.789	http://api.brain-map.org/grid_data/download/71015194?include=energy
Tmem196	0.789	http://api.brain-map.org/grid_data/download/74511946?include=energy
Umod	0.789	http://api.brain-map.org/grid_data/download/513233?include=energy
Rbm15	0.789	http://api.brain-map.org/grid_data/download/73617480?include=energy
Slc11a2	0.789	http://api.brain-map.org/grid_data/download/2571?include=energy
Terf2	0.789	http://api.brain-map.org/grid_data/download/69012717?include=energy
Nxnl1	0.789	http://api.brain-map.org/grid_data/download/68342282?include=energy
Ikbkg	0.789	http://api.brain-map.org/grid_data/download/71610883?include=energy
Nufip2	0.789	http://api.brain-map.org/grid_data/download/71380459?include=energy
Serpinf2	0.789	http://api.brain-map.org/grid_data/download/68498313?include=energy
Rpap3	0.789	http://api.brain-map.org/grid_data/download/513477?include=energy
C130050O18Rik	0.789	http://api.brain-map.org/grid_data/download/69530816?include=energy
LOC433280	0.789	http://api.brain-map.org/grid_data/download/70723050?include=energy
Crls1	0.789	http://api.brain-map.org/grid_data/download/1148?include=energy
Otud5	0.789	http://api.brain-map.org/grid_data/download/583864?include=energy
Nbn	0.789	http://api.brain-map.org/grid_data/download/69262299?include=energy
Napg	0.789	http://api.brain-map.org/grid_data/download/74822941?include=energy
Slmo2	0.789	http://api.brain-map.org/grid_data/download/67814216?include=energy
H2-T23	0.789	http://api.brain-map.org/grid_data/download/75749388?include=energy
Cdc42ep5	0.789	http://api.brain-map.org/grid_data/download/68300872?include=energy
Ilkap	0.789	http://api.brain-map.org/grid_data/download/67815550?include=energy
Tshb	0.789	http://api.brain-map.org/grid_data/download/100144122?include=energy
1700013D24Rik	0.789	http://api.brain-map.org/grid_data/download/74581355?include=energy
Top2b	0.789	http://api.brain-map.org/grid_data/download/100146153?include=energy
Slfn3	0.789	http://api.brain-map.org/grid_data/download/69874120?include=energy
5730437O09Rik*	0.788	http://api.brain-map.org/grid_data/download/73870563?include=energy
Gm1965	0.788	http://api.brain-map.org/grid_data/download/70787263?include=energy
Gosr2	0.788	http://api.brain-map.org/grid_data/download/67855413?include=energy
Asah2	0.788	http://api.brain-map.org/grid_data/download/69526910?include=energy
D830039M14Rik	0.788	http://api.brain-map.org/grid_data/download/69626558?include=energy
4930402F06Rik	0.788	http://api.brain-map.org/grid_data/download/70218079?include=energy
Siah3	0.788	http://api.brain-map.org/grid_data/download/70298435?include=energy
Glb1l3	0.788	http://api.brain-map.org/grid_data/download/71833026?include=energy
Alx3	0.788	http://api.brain-map.org/grid_data/download/77464960?include=energy
Xrn1	0.788	http://api.brain-map.org/grid_data/download/70195072?include=energy
Cndp1	0.788	http://api.brain-map.org/grid_data/download/69838254?include=energy
Tacr2	0.788	http://api.brain-map.org/grid_data/download/77464860?include=energy
Arhgef19	0.788	http://api.brain-map.org/grid_data/download/70528246?include=energy
3110001I22Rik	0.788	http://api.brain-map.org/grid_data/download/68546131?include=energy
Pabpc6	0.788	http://api.brain-map.org/grid_data/download/70218594?include=energy
Cep135	0.788	http://api.brain-map.org/grid_data/download/74821659?include=energy
Hoxc4	0.788	http://api.brain-map.org/grid_data/download/73606503?include=energy
Ctnna2	0.788	http://api.brain-map.org/grid_data/download/74363355?include=energy
Rab7l1	0.788	http://api.brain-map.org/grid_data/download/75827120?include=energy
Cobl	0.788	http://api.brain-map.org/grid_data/download/71380983?include=energy
E330018D03Rik	0.788	http://api.brain-map.org/grid_data/download/73712744?include=energy
Ube2d2b	0.788	http://api.brain-map.org/grid_data/download/69114715?include=energy
A630033H20Rik	0.788	http://api.brain-map.org/grid_data/download/69855729?include=energy
Tnr	0.788	http://api.brain-map.org/grid_data/download/73930819?include=energy
Tmem167b	0.788	http://api.brain-map.org/grid_data/download/69549644?include=energy
Bmp1	0.788	http://api.brain-map.org/grid_data/download/69529352?include=energy
Vcan	0.788	http://api.brain-map.org/grid_data/download/73520975?include=energy
Cxcl11	0.788	http://api.brain-map.org/grid_data/download/76081898?include=energy
Zfp828	0.788	http://api.brain-map.org/grid_data/download/70431271?include=energy
S100a3	0.788	http://api.brain-map.org/grid_data/download/73617498?include=energy
Ndufb6	0.788	http://api.brain-map.org/grid_data/download/70295894?include=energy
Poglut1	0.788	http://api.brain-map.org/grid_data/download/532688?include=energy
Nudt8	0.788	http://api.brain-map.org/grid_data/download/68919946?include=energy
3110021A11Rik	0.788	http://api.brain-map.org/grid_data/download/73716608?include=energy
Dzip3	0.788	http://api.brain-map.org/grid_data/download/72283348?include=energy
E530001K10Rik	0.788	http://api.brain-map.org/grid_data/download/74000552?include=energy
1700057H15Rik	0.788	http://api.brain-map.org/grid_data/download/74635060?include=energy
Acp1	0.788	http://api.brain-map.org/grid_data/download/69549669?include=energy
Bmp10	0.788	http://api.brain-map.org/grid_data/download/76003510?include=energy
Sec11a	0.788	http://api.brain-map.org/grid_data/download/69885678?include=energy
Clcn4-2	0.788	http://api.brain-map.org/grid_data/download/73512560?include=energy
Trpm5	0.787	http://api.brain-map.org/grid_data/download/69288429?include=energy
Pogk	0.787	http://api.brain-map.org/grid_data/download/71064277?include=energy
Hmgcll1	0.787	http://api.brain-map.org/grid_data/download/68196935?include=energy
Znhit2	0.787	http://api.brain-map.org/grid_data/download/70474083?include=energy
Dntt	0.787	http://api.brain-map.org/grid_data/download/75988379?include=energy
Rhbdf2	0.787	http://api.brain-map.org/grid_data/download/68797864?include=energy
BC055324	0.787	http://api.brain-map.org/grid_data/download/71764946?include=energy
Amdhd2	0.787	http://api.brain-map.org/grid_data/download/678714?include=energy
Ptcra	0.787	http://api.brain-map.org/grid_data/download/100144888?include=energy
Kcne3	0.787	http://api.brain-map.org/grid_data/download/2337?include=energy
C030046E11Rik	0.787	http://api.brain-map.org/grid_data/download/73906137?include=energy
B3galt1	0.787	http://api.brain-map.org/grid_data/download/69527896?include=energy
LOC435768	0.787	http://api.brain-map.org/grid_data/download/70784534?include=energy
Cbll1	0.787	http://api.brain-map.org/grid_data/download/70813245?include=energy
Meox1	0.787	http://api.brain-map.org/grid_data/download/86372?include=energy
Rad17	0.787	http://api.brain-map.org/grid_data/download/71381139?include=energy
Sgcg	0.787	http://api.brain-map.org/grid_data/download/74749941?include=energy
Mcart6	0.787	http://api.brain-map.org/grid_data/download/70609211?include=energy
Trmt11	0.787	http://api.brain-map.org/grid_data/download/68666953?include=energy
BC055004	0.787	http://api.brain-map.org/grid_data/download/69012888?include=energy
Cdh17	0.787	http://api.brain-map.org/grid_data/download/69838100?include=energy
BC022713	0.787	http://api.brain-map.org/grid_data/download/68498747?include=energy
Rbm22	0.787	http://api.brain-map.org/grid_data/download/74059292?include=energy
A930002I21Rik	0.787	http://api.brain-map.org/grid_data/download/70228609?include=energy
B930095G15Rik	0.787	http://api.brain-map.org/grid_data/download/69528028?include=energy
Apoa4	0.787	http://api.brain-map.org/grid_data/download/68341298?include=energy
Tomm22	0.787	http://api.brain-map.org/grid_data/download/69262238?include=energy
Hfe	0.787	http://api.brain-map.org/grid_data/download/70277943?include=energy
Qrsl1	0.787	http://api.brain-map.org/grid_data/download/69817787?include=energy
Adcy9	0.787	http://api.brain-map.org/grid_data/download/83?include=energy
Srebf2	0.787	http://api.brain-map.org/grid_data/download/70439177?include=energy
1700030J22Rik	0.787	http://api.brain-map.org/grid_data/download/69114658?include=energy
Sec61a1	0.787	http://api.brain-map.org/grid_data/download/512530?include=energy
Cldn26	0.787	http://api.brain-map.org/grid_data/download/71493216?include=energy
Zfp26	0.787	http://api.brain-map.org/grid_data/download/74431628?include=energy
Gsx2	0.787	http://api.brain-map.org/grid_data/download/69289075?include=energy
Bysl	0.787	http://api.brain-map.org/grid_data/download/68910958?include=energy
9230002O09Rik*	0.787	http://api.brain-map.org/grid_data/download/73949418?include=energy
1700019B03Rik	0.787	http://api.brain-map.org/grid_data/download/70525771?include=energy
Tfip11	0.787	http://api.brain-map.org/grid_data/download/70526028?include=energy
Ell	0.787	http://api.brain-map.org/grid_data/download/532704?include=energy
Mm.216517	0.787	http://api.brain-map.org/grid_data/download/74510188?include=energy
4930565D16Rik	0.787	http://api.brain-map.org/grid_data/download/71892005?include=energy
Mm.184971	0.787	http://api.brain-map.org/grid_data/download/71152240?include=energy
Dapp1	0.787	http://api.brain-map.org/grid_data/download/68443327?include=energy
Krtap6-2	0.787	http://api.brain-map.org/grid_data/download/70295464?include=energy
Prss43	0.787	http://api.brain-map.org/grid_data/download/71281837?include=energy
Hadh	0.787	http://api.brain-map.org/grid_data/download/71381046?include=energy
Clec4f	0.786	http://api.brain-map.org/grid_data/download/68203465?include=energy
Tmem123	0.786	http://api.brain-map.org/grid_data/download/68080674?include=energy
Mki67ip	0.786	http://api.brain-map.org/grid_data/download/68798211?include=energy
Tlcd1	0.786	http://api.brain-map.org/grid_data/download/386240?include=energy
Taf11	0.786	http://api.brain-map.org/grid_data/download/69816954?include=energy
Edc3	0.786	http://api.brain-map.org/grid_data/download/80343346?include=energy
Cebpe	0.786	http://api.brain-map.org/grid_data/download/71021147?include=energy
Chchd5	0.786	http://api.brain-map.org/grid_data/download/68444678?include=energy
Casq2	0.786	http://api.brain-map.org/grid_data/download/76115726?include=energy
1700125H20Rik	0.786	http://api.brain-map.org/grid_data/download/69114956?include=energy
Zranb3	0.786	http://api.brain-map.org/grid_data/download/71809379?include=energy
Elovl2	0.786	http://api.brain-map.org/grid_data/download/69671995?include=energy
Mga	0.786	http://api.brain-map.org/grid_data/download/69816625?include=energy
Gmcl1	0.786	http://api.brain-map.org/grid_data/download/69672826?include=energy
Suclg2	0.786	http://api.brain-map.org/grid_data/download/69887445?include=energy
Adck2	0.786	http://api.brain-map.org/grid_data/download/70717988?include=energy
Rims4	0.786	http://api.brain-map.org/grid_data/download/71020888?include=energy
Nln	0.786	http://api.brain-map.org/grid_data/download/638735?include=energy
Rassf6	0.786	http://api.brain-map.org/grid_data/download/75041561?include=energy
BC002118	0.786	http://api.brain-map.org/grid_data/download/77792905?include=energy
LOC546051	0.786	http://api.brain-map.org/grid_data/download/71211452?include=energy
LOC546815	0.786	http://api.brain-map.org/grid_data/download/74736486?include=energy
Ugt1a2	0.786	http://api.brain-map.org/grid_data/download/68410369?include=energy
Bahd1	0.786	http://api.brain-map.org/grid_data/download/69528064?include=energy
Adra2c	0.786	http://api.brain-map.org/grid_data/download/70723357?include=energy
Elmod2	0.786	http://api.brain-map.org/grid_data/download/69447834?include=energy
3110099E03Rik	0.786	http://api.brain-map.org/grid_data/download/70203682?include=energy
Rab4b	0.786	http://api.brain-map.org/grid_data/download/71724654?include=energy
Pla2g10	0.786	http://api.brain-map.org/grid_data/download/68666744?include=energy
Lcn10	0.786	http://api.brain-map.org/grid_data/download/72081580?include=energy
Specc1l	0.786	http://api.brain-map.org/grid_data/download/71152192?include=energy
Hsd17b4	0.786	http://api.brain-map.org/grid_data/download/68269055?include=energy
Fbxo41	0.786	http://api.brain-map.org/grid_data/download/70946252?include=energy
Padi6	0.786	http://api.brain-map.org/grid_data/download/68667467?include=energy
Mlxipl	0.786	http://api.brain-map.org/grid_data/download/1932?include=energy
Sp8	0.786	http://api.brain-map.org/grid_data/download/74272904?include=energy
Thop1	0.786	http://api.brain-map.org/grid_data/download/68638023?include=energy
D030040B21Rik	0.786	http://api.brain-map.org/grid_data/download/70305640?include=energy
Mcart1	0.786	http://api.brain-map.org/grid_data/download/69752107?include=energy
Lysmd3	0.785	http://api.brain-map.org/grid_data/download/70528300?include=energy
Mms19	0.785	http://api.brain-map.org/grid_data/download/68797709?include=energy
Alppl2	0.785	http://api.brain-map.org/grid_data/download/68667545?include=energy
Ptpro	0.785	http://api.brain-map.org/grid_data/download/72340109?include=energy
4931408C20Rik	0.785	http://api.brain-map.org/grid_data/download/70565915?include=energy
St8sia4	0.785	http://api.brain-map.org/grid_data/download/69873773?include=energy
Spib	0.785	http://api.brain-map.org/grid_data/download/77904755?include=energy
Klhl8	0.785	http://api.brain-map.org/grid_data/download/69735879?include=energy
Mxd3	0.785	http://api.brain-map.org/grid_data/download/68798879?include=energy
G530016P13Rik*	0.785	http://api.brain-map.org/grid_data/download/73767323?include=energy
Rbl2	0.785	http://api.brain-map.org/grid_data/download/70525831?include=energy
Svep1	0.785	http://api.brain-map.org/grid_data/download/71280808?include=energy
Pfn3	0.785	http://api.brain-map.org/grid_data/download/71212677?include=energy
Slc38a7	0.785	http://api.brain-map.org/grid_data/download/68637969?include=energy
Gm1292	0.785	http://api.brain-map.org/grid_data/download/70300085?include=energy
Pycr1	0.785	http://api.brain-map.org/grid_data/download/70473957?include=energy
LOC329428	0.785	http://api.brain-map.org/grid_data/download/70725049?include=energy
Mmp16	0.785	http://api.brain-map.org/grid_data/download/72129244?include=energy
Hdhd3	0.785	http://api.brain-map.org/grid_data/download/69838764?include=energy
Dbil5	0.785	http://api.brain-map.org/grid_data/download/69114447?include=energy
Sh3rf1	0.785	http://api.brain-map.org/grid_data/download/74581451?include=energy
Irgc1	0.785	http://api.brain-map.org/grid_data/download/76081005?include=energy
Kif12	0.785	http://api.brain-map.org/grid_data/download/68632673?include=energy
Cep70	0.785	http://api.brain-map.org/grid_data/download/71496019?include=energy
D130051O21Rik*	0.785	http://api.brain-map.org/grid_data/download/73834361?include=energy
Tcfl5	0.785	http://api.brain-map.org/grid_data/download/71381121?include=energy
Vps13c	0.785	http://api.brain-map.org/grid_data/download/74058646?include=energy
Zmym4	0.785	http://api.brain-map.org/grid_data/download/70598370?include=energy
LOC434305	0.785	http://api.brain-map.org/grid_data/download/70793863?include=energy
BC094916	0.785	http://api.brain-map.org/grid_data/download/74430942?include=energy
BC051070	0.785	http://api.brain-map.org/grid_data/download/68797613?include=energy
Ehmt1	0.785	http://api.brain-map.org/grid_data/download/71808582?include=energy
Lhcgr	0.785	http://api.brain-map.org/grid_data/download/72283794?include=energy
Slc26a5	0.785	http://api.brain-map.org/grid_data/download/73424005?include=energy
5830427D03Rik	0.785	http://api.brain-map.org/grid_data/download/70227848?include=energy
1700025G04Rik	0.785	http://api.brain-map.org/grid_data/download/75774649?include=energy
Gm40	0.785	http://api.brain-map.org/grid_data/download/70295542?include=energy
Il1rl2	0.785	http://api.brain-map.org/grid_data/download/75931598?include=energy
Ak1	0.785	http://api.brain-map.org/grid_data/download/77414110?include=energy
Cxxc5	0.785	http://api.brain-map.org/grid_data/download/69080510?include=energy
Gm14644	0.785	http://api.brain-map.org/grid_data/download/70724959?include=energy
Spty2d1	0.785	http://api.brain-map.org/grid_data/download/70227854?include=energy
Colec12	0.785	http://api.brain-map.org/grid_data/download/69015314?include=energy
Ddx19b	0.785	http://api.brain-map.org/grid_data/download/77414162?include=energy
Trim31	0.785	http://api.brain-map.org/grid_data/download/68499416?include=energy
4921523A10Rik	0.785	http://api.brain-map.org/grid_data/download/70303893?include=energy
Srp68	0.785	http://api.brain-map.org/grid_data/download/70474023?include=energy
Snta1	0.785	http://api.brain-map.org/grid_data/download/70474011?include=energy
Grcc10	0.785	http://api.brain-map.org/grid_data/download/386251?include=energy
Mgst1	0.785	http://api.brain-map.org/grid_data/download/68269646?include=energy
Vmn1r-ps8	0.785	http://api.brain-map.org/grid_data/download/70785254?include=energy
Gm890	0.784	http://api.brain-map.org/grid_data/download/70298399?include=energy
Adamtsl2	0.784	http://api.brain-map.org/grid_data/download/74881352?include=energy
D17H6S56E-5	0.784	http://api.brain-map.org/grid_data/download/67787346?include=energy
Tmco4	0.784	http://api.brain-map.org/grid_data/download/68341280?include=energy
Gnmt	0.784	http://api.brain-map.org/grid_data/download/68301413?include=energy
Magef1	0.784	http://api.brain-map.org/grid_data/download/71493819?include=energy
Cyp2a5	0.784	http://api.brain-map.org/grid_data/download/70238040?include=energy
Gon4l	0.784	http://api.brain-map.org/grid_data/download/392939?include=energy
Sgsm1	0.784	http://api.brain-map.org/grid_data/download/73521821?include=energy
Snx8	0.784	http://api.brain-map.org/grid_data/download/678713?include=energy
Syna	0.784	http://api.brain-map.org/grid_data/download/70295691?include=energy
Gabra6	0.784	http://api.brain-map.org/grid_data/download/75551467?include=energy
BC031781	0.784	http://api.brain-map.org/grid_data/download/73712852?include=energy
A730037C10Rik	0.784	http://api.brain-map.org/grid_data/download/74277734?include=energy
5730522E02Rik	0.784	http://api.brain-map.org/grid_data/download/71892292?include=energy
Naprt1	0.784	http://api.brain-map.org/grid_data/download/68745078?include=energy
Nsd1	0.784	http://api.brain-map.org/grid_data/download/74743429?include=energy

Ercc6l	0.784	http://api.brain-map.org/grid_data/download/70528306?include=energy
Nagk	0.784	http://api.brain-map.org/grid_data/download/69080025?include=energy
Hdgfl1	0.784	http://api.brain-map.org/grid_data/download/69817781?include=energy
F830018J01Rik*	0.784	http://api.brain-map.org/grid_data/download/73748644?include=energy
Flad1	0.784	http://api.brain-map.org/grid_data/download/68499028?include=energy
9430007A20Rik	0.784	http://api.brain-map.org/grid_data/download/71020979?include=energy
Gm5608	0.784	http://api.brain-map.org/grid_data/download/70793833?include=energy
Mapk4	0.784	http://api.brain-map.org/grid_data/download/73994721?include=energy
Arhgef7	0.784	http://api.brain-map.org/grid_data/download/76003447?include=energy
Larp1	0.784	http://api.brain-map.org/grid_data/download/68666894?include=energy
Dnttip2	0.784	http://api.brain-map.org/grid_data/download/69080708?include=energy
Wnt16	0.784	http://api.brain-map.org/grid_data/download/100145362?include=energy
Il1rn	0.784	http://api.brain-map.org/grid_data/download/75831739?include=energy
Ifi204	0.783	http://api.brain-map.org/grid_data/download/576424?include=energy
1700060C20Rik	0.783	http://api.brain-map.org/grid_data/download/70201468?include=energy
Ybx2	0.783	http://api.brain-map.org/grid_data/download/75080250?include=energy
Tbc1d4	0.783	http://api.brain-map.org/grid_data/download/100144442?include=energy
Itch	0.783	http://api.brain-map.org/grid_data/download/74580847?include=energy
Rgs11	0.783	http://api.brain-map.org/grid_data/download/74359353?include=energy
Ccdc154	0.783	http://api.brain-map.org/grid_data/download/74514031?include=energy
Slfn2	0.783	http://api.brain-map.org/grid_data/download/69874114?include=energy
Mob4	0.783	http://api.brain-map.org/grid_data/download/70565123?include=energy
Acpp	0.783	http://api.brain-map.org/grid_data/download/69514672?include=energy
Nr4a1	0.783	http://api.brain-map.org/grid_data/download/1792?include=energy
Iqcc	0.783	http://api.brain-map.org/grid_data/download/70430876?include=energy
A530085O15Rik	0.783	http://api.brain-map.org/grid_data/download/71808466?include=energy
Rpusd2	0.783	http://api.brain-map.org/grid_data/download/69407779?include=energy
4833424O15Rik	0.783	http://api.brain-map.org/grid_data/download/70744326?include=energy
4921513I03Rik	0.783	http://api.brain-map.org/grid_data/download/70205632?include=energy
Ttll3	0.783	http://api.brain-map.org/grid_data/download/633275?include=energy
Gpr182	0.783	http://api.brain-map.org/grid_data/download/69236783?include=energy
Wdr3	0.783	http://api.brain-map.org/grid_data/download/68798274?include=energy
Jtb	0.783	http://api.brain-map.org/grid_data/download/69134661?include=energy
Ube2g1	0.783	http://api.brain-map.org/grid_data/download/70194510?include=energy
4930578N16Rik	0.783	http://api.brain-map.org/grid_data/download/69442323?include=energy
Hcrt	0.783	http://api.brain-map.org/grid_data/download/79913316?include=energy
Pax4	0.783	http://api.brain-map.org/grid_data/download/69289667?include=energy
BC051628	0.783	http://api.brain-map.org/grid_data/download/69114253?include=energy
F830016C15Rik*	0.783	http://api.brain-map.org/grid_data/download/74427106?include=energy
Ndnl2	0.783	http://api.brain-map.org/grid_data/download/71764162?include=energy
Trh	0.783	http://api.brain-map.org/grid_data/download/71016631?include=energy
Otp	0.783	http://api.brain-map.org/grid_data/download/2454?include=energy
Sdpr	0.783	http://api.brain-map.org/grid_data/download/68441544?include=energy
Slc47a2	0.783	http://api.brain-map.org/grid_data/download/70595946?include=energy
Parn	0.783	http://api.brain-map.org/grid_data/download/587426?include=energy
Il1rapl1	0.783	http://api.brain-map.org/grid_data/download/70278045?include=energy
2310009B15Rik	0.783	http://api.brain-map.org/grid_data/download/72283330?include=energy
2410042D21Rik	0.783	http://api.brain-map.org/grid_data/download/67978661?include=energy
Mgll	0.783	http://api.brain-map.org/grid_data/download/72129243?include=energy
Uso1	0.783	http://api.brain-map.org/grid_data/download/638711?include=energy
Derl3	0.783	http://api.brain-map.org/grid_data/download/69201684?include=energy
LOC434660	0.783	http://api.brain-map.org/grid_data/download/71022397?include=energy
Plrg1	0.783	http://api.brain-map.org/grid_data/download/392932?include=energy
Tnfsf9	0.783	http://api.brain-map.org/grid_data/download/69981233?include=energy
Sec61b	0.783	http://api.brain-map.org/grid_data/download/76081908?include=energy
Irak2	0.783	http://api.brain-map.org/grid_data/download/69735520?include=energy
Hyal3	0.783	http://api.brain-map.org/grid_data/download/68341651?include=energy
1700087M12Rik*	0.782	http://api.brain-map.org/grid_data/download/74359556?include=energy
1700029J11Rik	0.782	http://api.brain-map.org/grid_data/download/70201391?include=energy
Yipf4	0.782	http://api.brain-map.org/grid_data/download/68076885?include=energy
Mfsd11	0.782	http://api.brain-map.org/grid_data/download/74821569?include=energy
Ccdc146	0.782	http://api.brain-map.org/grid_data/download/74265153?include=energy
LOC381739	0.782	http://api.brain-map.org/grid_data/download/70596101?include=energy
Mm.350536	0.782	http://api.brain-map.org/grid_data/download/71161117?include=energy
Chchd1	0.782	http://api.brain-map.org/grid_data/download/68862162?include=energy
Cartpt	0.782	http://api.brain-map.org/grid_data/download/72077479?include=energy
Cck	0.782	http://api.brain-map.org/grid_data/download/200?include=energy
Tpk1	0.782	http://api.brain-map.org/grid_data/download/70525821?include=energy
Pabpc2	0.782	http://api.brain-map.org/grid_data/download/71763843?include=energy
A930104D05Rik	0.782	http://api.brain-map.org/grid_data/download/72471695?include=energy
Pla2g4d	0.782	http://api.brain-map.org/grid_data/download/71808717?include=energy
Cela3b	0.782	http://api.brain-map.org/grid_data/download/69671983?include=energy
4930455F16Rik	0.782	http://api.brain-map.org/grid_data/download/74581349?include=energy
Doxl2	0.782	http://api.brain-map.org/grid_data/download/74430894?include=energy
AA960436	0.782	http://api.brain-map.org/grid_data/download/68666655?include=energy
Egr3	0.782	http://api.brain-map.org/grid_data/download/74581377?include=energy
1700007G11Rik	0.782	http://api.brain-map.org/grid_data/download/74427100?include=energy
C1qtnf7	0.782	http://api.brain-map.org/grid_data/download/70231899?include=energy
A730027B03Rik	0.782	http://api.brain-map.org/grid_data/download/69548795?include=energy
Fam54b	0.782	http://api.brain-map.org/grid_data/download/68193029?include=energy
Gramd1c	0.782	http://api.brain-map.org/grid_data/download/70528023?include=energy
Prph2	0.782	http://api.brain-map.org/grid_data/download/75990505?include=energy
Gm5918	0.782	http://api.brain-map.org/grid_data/download/71211797?include=energy
Prex2	0.782	http://api.brain-map.org/grid_data/download/100144925?include=energy
Sccpdh	0.782	http://api.brain-map.org/grid_data/download/73635772?include=energy
6330416G13Rik	0.782	http://api.brain-map.org/grid_data/download/68498558?include=energy
Pfdn1	0.782	http://api.brain-map.org/grid_data/download/68632057?include=energy
9130221H12Rik	0.782	http://api.brain-map.org/grid_data/download/67815934?include=energy
Lypd1	0.782	http://api.brain-map.org/grid_data/download/72008305?include=energy
Wdr61	0.782	http://api.brain-map.org/grid_data/download/70445455?include=energy
Pck1	0.782	http://api.brain-map.org/grid_data/download/77923598?include=energy
Efcab7	0.782	http://api.brain-map.org/grid_data/download/68341866?include=energy
Bpifc	0.782	http://api.brain-map.org/grid_data/download/70231821?include=energy
Rgs1	0.782	http://api.brain-map.org/grid_data/download/68744516?include=energy
1600029D21Rik	0.782	http://api.brain-map.org/grid_data/download/68342240?include=energy
Rdh11	0.782	http://api.brain-map.org/grid_data/download/69549651?include=energy
Arl5a	0.782	http://api.brain-map.org/grid_data/download/74274688?include=energy
1700128E19Rik	0.782	http://api.brain-map.org/grid_data/download/74359305?include=energy
Sowahb	0.782	http://api.brain-map.org/grid_data/download/69816440?include=energy
Ccdc73	0.782	http://api.brain-map.org/grid_data/download/70303917?include=energy
Hecw1	0.782	http://api.brain-map.org/grid_data/download/74882685?include=energy
4930526D03Rik	0.782	http://api.brain-map.org/grid_data/download/70218253?include=energy
Gp6	0.782	http://api.brain-map.org/grid_data/download/70276945?include=energy
Art2b	0.782	http://api.brain-map.org/grid_data/download/70724577?include=energy
Wdsub1	0.782	http://api.brain-map.org/grid_data/download/68798217?include=energy
Ppm1h	0.782	http://api.brain-map.org/grid_data/download/71809259?include=energy
Ndel1	0.782	http://api.brain-map.org/grid_data/download/1514?include=energy
Dock5	0.781	http://api.brain-map.org/grid_data/download/73513711?include=energy
Crem	0.781	http://api.brain-map.org/grid_data/download/68545971?include=energy
Abhd1	0.781	http://api.brain-map.org/grid_data/download/68321976?include=energy
1700063H04Rik	0.781	http://api.brain-map.org/grid_data/download/70201480?include=energy
Lrg1	0.781	http://api.brain-map.org/grid_data/download/68499183?include=energy
Slc2a8	0.781	http://api.brain-map.org/grid_data/download/69873887?include=energy
AI606473	0.781	http://api.brain-map.org/grid_data/download/1468?include=energy
Klhl14	0.781	http://api.brain-map.org/grid_data/download/69444801?include=energy
LOC433015	0.781	http://api.brain-map.org/grid_data/download/77621722?include=energy
Itpa	0.781	http://api.brain-map.org/grid_data/download/67881762?include=energy
Mm.350954	0.781	http://api.brain-map.org/grid_data/download/71161123?include=energy
LOC432747	0.781	http://api.brain-map.org/grid_data/download/70611258?include=energy
Pex11b	0.781	http://api.brain-map.org/grid_data/download/70439069?include=energy
Ing1	0.781	http://api.brain-map.org/grid_data/download/70431397?include=energy
Zfp169	0.781	http://api.brain-map.org/grid_data/download/69353663?include=energy
Slc34a3	0.781	http://api.brain-map.org/grid_data/download/73818808?include=energy
BC056474	0.781	http://api.brain-map.org/grid_data/download/68919057?include=energy
Pias1	0.781	http://api.brain-map.org/grid_data/download/69013194?include=energy
Zeb2	0.781	http://api.brain-map.org/grid_data/download/71924066?include=energy
Esx1	0.781	http://api.brain-map.org/grid_data/download/100145356?include=energy
4932435O22Rik	0.781	http://api.brain-map.org/grid_data/download/70218576?include=energy
Bud31	0.781	http://api.brain-map.org/grid_data/download/70724613?include=energy
Lnp	0.781	http://api.brain-map.org/grid_data/download/69028438?include=energy
Ski	0.781	http://api.brain-map.org/grid_data/download/71325474?include=energy
Tat	0.781	http://api.brain-map.org/grid_data/download/2657?include=energy
Col27a1	0.781	http://api.brain-map.org/grid_data/download/74272916?include=energy
LOC433871	0.781	http://api.brain-map.org/grid_data/download/70787032?include=energy
Ly6h	0.781	http://api.brain-map.org/grid_data/download/71924388?include=energy
Pcdhb1	0.781	http://api.brain-map.org/grid_data/download/70300690?include=energy
Adamts7	0.781	http://api.brain-map.org/grid_data/download/70724535?include=energy
Gpr119	0.781	http://api.brain-map.org/grid_data/download/69257415?include=energy
Trp53inp1	0.781	http://api.brain-map.org/grid_data/download/77464972?include=energy
Pld1	0.781	http://api.brain-map.org/grid_data/download/69783298?include=energy
Nr3c2	0.781	http://api.brain-map.org/grid_data/download/730?include=energy
Stra6	0.781	http://api.brain-map.org/grid_data/download/75041492?include=energy
Letm2	0.781	http://api.brain-map.org/grid_data/download/69837839?include=energy
Whsc2	0.781	http://api.brain-map.org/grid_data/download/68162237?include=energy
Barx1	0.781	http://api.brain-map.org/grid_data/download/71283687?include=energy
Timm17b	0.781	http://api.brain-map.org/grid_data/download/68269514?include=energy
D630045J12Rik	0.781	http://api.brain-map.org/grid_data/download/69609019?include=energy
Egln2	0.781	http://api.brain-map.org/grid_data/download/67815539?include=energy
Hephl1	0.781	http://api.brain-map.org/grid_data/download/74562272?include=energy
Dusp2	0.781	http://api.brain-map.org/grid_data/download/74514097?include=energy
Tnfrsf1b	0.781	http://api.brain-map.org/grid_data/download/69981145?include=energy
Fam71f1	0.781	http://api.brain-map.org/grid_data/download/71281871?include=energy
MGC7717	0.781	http://api.brain-map.org/grid_data/download/79538290?include=energy
Sh2d4a	0.78	http://api.brain-map.org/grid_data/download/70302063?include=energy
Rnf38	0.78	http://api.brain-map.org/grid_data/download/68844743?include=energy
Ptger1	0.78	http://api.brain-map.org/grid_data/download/71325378?include=energy
BC051076	0.78	http://api.brain-map.org/grid_data/download/71574564?include=energy
Pxdn	0.78	http://api.brain-map.org/grid_data/download/70539244?include=energy
Xpot	0.78	http://api.brain-map.org/grid_data/download/70195060?include=energy
Nab2	0.78	http://api.brain-map.org/grid_data/download/68745462?include=energy
Kif3b	0.78	http://api.brain-map.org/grid_data/download/70919842?include=energy
Gpsm2	0.78	http://api.brain-map.org/grid_data/download/71610884?include=energy
Wdr82	0.78	http://api.brain-map.org/grid_data/download/71494623?include=energy
Cyb561	0.78	http://api.brain-map.org/grid_data/download/71020402?include=energy
Ccl21a	0.78	http://api.brain-map.org/grid_data/download/68546025?include=energy
Srbd1	0.78	http://api.brain-map.org/grid_data/download/68546215?include=energy
Brd3	0.78	http://api.brain-map.org/grid_data/download/67787358?include=energy
Lsp1	0.78	http://api.brain-map.org/grid_data/download/100144100?include=energy
Lpin2	0.78	http://api.brain-map.org/grid_data/download/378554?include=energy
Dhx38	0.78	http://api.brain-map.org/grid_data/download/69838420?include=energy
Gm887	0.78	http://api.brain-map.org/grid_data/download/74735331?include=energy
Pts	0.78	http://api.brain-map.org/grid_data/download/68148753?include=energy
Ccdc37	0.78	http://api.brain-map.org/grid_data/download/74581372?include=energy
Pcdhgb8	0.78	http://api.brain-map.org/grid_data/download/70784946?include=energy
Ttll12	0.78	http://api.brain-map.org/grid_data/download/68667982?include=energy
Gm666	0.78	http://api.brain-map.org/grid_data/download/70298190?include=energy
Galr2	0.78	http://api.brain-map.org/grid_data/download/80517343?include=energy
Ppfia1	0.78	http://api.brain-map.org/grid_data/download/71377793?include=energy
Pigo	0.78	http://api.brain-map.org/grid_data/download/69783160?include=energy
Cbfa2t3	0.78	http://api.brain-map.org/grid_data/download/75038485?include=energy
Parvg	0.78	http://api.brain-map.org/grid_data/download/70813925?include=energy
Slc2a3	0.78	http://api.brain-map.org/grid_data/download/1840?include=energy
Sltm	0.78	http://api.brain-map.org/grid_data/download/601192?include=energy
C330006D17Rik	0.78	http://api.brain-map.org/grid_data/download/74300729?include=energy
Ftsjd2	0.78	http://api.brain-map.org/grid_data/download/67792202?include=energy
Lcn9	0.78	http://api.brain-map.org/grid_data/download/71892400?include=energy
LOC243965	0.78	http://api.brain-map.org/grid_data/download/72076623?include=energy
Ncan	0.779	http://api.brain-map.org/grid_data/download/69608154?include=energy
1700049G17Rik	0.779	http://api.brain-map.org/grid_data/download/73497705?include=energy
Mettl10	0.779	http://api.brain-map.org/grid_data/download/71492934?include=energy
Krtap13	0.779	http://api.brain-map.org/grid_data/download/68798430?include=energy
Hic1	0.779	http://api.brain-map.org/grid_data/download/69289099?include=energy
Mical1	0.779	http://api.brain-map.org/grid_data/download/68443435?include=energy
2210021J22Rik	0.779	http://api.brain-map.org/grid_data/download/68441448?include=energy
Zdhhc4	0.779	http://api.brain-map.org/grid_data/download/601191?include=energy
Myl1	0.779	http://api.brain-map.org/grid_data/download/69781989?include=energy
Mmp1a	0.779	http://api.brain-map.org/grid_data/download/70300391?include=energy
1700055N04Rik	0.779	http://api.brain-map.org/grid_data/download/70201462?include=energy
Las1l	0.779	http://api.brain-map.org/grid_data/download/68203481?include=energy
Neurog2	0.779	http://api.brain-map.org/grid_data/download/74579503?include=energy
Hoxa10	0.779	http://api.brain-map.org/grid_data/download/70431367?include=energy
Oraov1	0.779	http://api.brain-map.org/grid_data/download/80525705?include=energy
Otud4	0.779	http://api.brain-map.org/grid_data/download/69626570?include=energy
Rcl1	0.779	http://api.brain-map.org/grid_data/download/69079978?include=energy
Jmjd4	0.779	http://api.brain-map.org/grid_data/download/69288662?include=energy
Ccdc121	0.779	http://api.brain-map.org/grid_data/download/73513341?include=energy
Pde6a	0.779	http://api.brain-map.org/grid_data/download/68632960?include=energy
Narfl	0.779	http://api.brain-map.org/grid_data/download/68667485?include=energy
1110008F13Rik	0.779	http://api.brain-map.org/grid_data/download/70474791?include=energy
Srpx	0.779	http://api.brain-map.org/grid_data/download/80472068?include=energy
7420444D23Rik*	0.779	http://api.brain-map.org/grid_data/download/73927718?include=energy
A630043P06	0.779	http://api.brain-map.org/grid_data/download/70305311?include=energy
Mgmt	0.779	http://api.brain-map.org/grid_data/download/71612271?include=energy
Itga2b	0.779	http://api.brain-map.org/grid_data/download/76003677?include=energy
LOC433969	0.779	http://api.brain-map.org/grid_data/download/70787197?include=energy
H2-Ab1	0.779	http://api.brain-map.org/grid_data/download/68269616?include=energy
Otub2	0.779	http://api.brain-map.org/grid_data/download/74821973?include=energy
5330403J18Rik*	0.779	http://api.brain-map.org/grid_data/download/73808258?include=energy
D18Ertd653e	0.779	http://api.brain-map.org/grid_data/download/73817443?include=energy
Fzd9	0.779	http://api.brain-map.org/grid_data/download/73907498?include=energy
C330005M16Rik	0.779	http://api.brain-map.org/grid_data/download/74272930?include=energy
Adam10	0.779	http://api.brain-map.org/grid_data/download/69514738?include=energy
4931403F16Rik	0.779	http://api.brain-map.org/grid_data/download/69236730?include=energy
Gm947	0.779	http://api.brain-map.org/grid_data/download/70298501?include=energy
LOC433542	0.779	http://api.brain-map.org/grid_data/download/70785774?include=energy
Mc2r	0.779	http://api.brain-map.org/grid_data/download/69860839?include=energy
C86695	0.779	http://api.brain-map.org/grid_data/download/72471781?include=energy
Yipf2	0.779	http://api.brain-map.org/grid_data/download/70445407?include=energy
Scn9a	0.779	http://api.brain-map.org/grid_data/download/74749261?include=energy
Bag3	0.778	http://api.brain-map.org/grid_data/download/69528058?include=energy
Tcerg1l	0.778	http://api.brain-map.org/grid_data/download/75041511?include=energy
Pde8b	0.778	http://api.brain-map.org/grid_data/download/71020389?include=energy
Smcp	0.778	http://api.brain-map.org/grid_data/download/70546117?include=energy
Mpzl2	0.778	http://api.brain-map.org/grid_data/download/68301282?include=energy
Nop58	0.778	http://api.brain-map.org/grid_data/download/73617456?include=energy
Cdc123	0.778	http://api.brain-map.org/grid_data/download/671227?include=energy
Cdk3-ps	0.778	http://api.brain-map.org/grid_data/download/71021146?include=energy
Nos2	0.778	http://api.brain-map.org/grid_data/download/69028647?include=energy
Pcdhb18	0.778	http://api.brain-map.org/grid_data/download/74581445?include=energy
Sec23a	0.778	http://api.brain-map.org/grid_data/download/70919262?include=energy
Ticam1	0.778	http://api.brain-map.org/grid_data/download/72081545?include=energy
Lactb	0.778	http://api.brain-map.org/grid_data/download/68924515?include=energy
Abt1	0.778	http://api.brain-map.org/grid_data/download/69514642?include=energy
Cox8b	0.778	http://api.brain-map.org/grid_data/download/68744779?include=energy
Fam184a	0.778	http://api.brain-map.org/grid_data/download/74363348?include=energy
Fam100a	0.778	http://api.brain-map.org/grid_data/download/71213011?include=energy
Mettl6	0.778	http://api.brain-map.org/grid_data/download/678690?include=energy
Cd6	0.778	http://api.brain-map.org/grid_data/download/112205643?include=energy
LOC435481	0.778	http://api.brain-map.org/grid_data/download/71281943?include=energy
Seh1l	0.778	http://api.brain-map.org/grid_data/download/68443513?include=energy
4931440L10Rik	0.778	http://api.brain-map.org/grid_data/download/71892083?include=energy
Rab43	0.778	http://api.brain-map.org/grid_data/download/73929886?include=energy
Gbp7	0.778	http://api.brain-map.org/grid_data/download/68076910?include=energy
A630073D07Rik	0.778	http://api.brain-map.org/grid_data/download/74822856?include=energy
Pde6h	0.778	http://api.brain-map.org/grid_data/download/71393334?include=energy
Olfr513	0.778	http://api.brain-map.org/grid_data/download/74724133?include=energy
Pcdhgc5	0.778	http://api.brain-map.org/grid_data/download/70784964?include=energy
Zbtb39	0.778	http://api.brain-map.org/grid_data/download/71493825?include=energy
Mrp63	0.778	http://api.brain-map.org/grid_data/download/69114581?include=energy
Chst4	0.778	http://api.brain-map.org/grid_data/download/69015332?include=energy
Ccz1	0.778	http://api.brain-map.org/grid_data/download/70528270?include=energy
Tmc6	0.778	http://api.brain-map.org/grid_data/download/70928250?include=energy
Bglap-rs1	0.778	http://api.brain-map.org/grid_data/download/68631978?include=energy
Mfap4	0.778	http://api.brain-map.org/grid_data/download/68498639?include=energy
AA986860	0.778	http://api.brain-map.org/grid_data/download/69514518?include=energy
Gstt3	0.777	http://api.brain-map.org/grid_data/download/68443196?include=energy
LOC236469	0.777	http://api.brain-map.org/grid_data/download/70566169?include=energy

Fam55d	0.777	http://api.brain-map.org/grid_data/download/74822581?include=energy
Sgsh	0.777	http://api.brain-map.org/grid_data/download/69873696?include=energy
Gimap8	0.777	http://api.brain-map.org/grid_data/download/75197648?include=energy
Olfr479	0.777	http://api.brain-map.org/grid_data/download/74724127?include=energy
Slc5a4b	0.777	http://api.brain-map.org/grid_data/download/70785092?include=energy
Ppm1l	0.777	http://api.brain-map.org/grid_data/download/71924215?include=energy
Dmbt1	0.777	http://api.brain-map.org/grid_data/download/68924757?include=energy
Xrcc3	0.777	http://api.brain-map.org/grid_data/download/69817085?include=energy
Lmbrd1	0.777	http://api.brain-map.org/grid_data/download/68151447?include=energy
LOC434003	0.777	http://api.brain-map.org/grid_data/download/70787203?include=energy
Gtf2e2	0.777	http://api.brain-map.org/grid_data/download/583853?include=energy
Pkd1l3	0.777	http://api.brain-map.org/grid_data/download/74509524?include=energy
Clptm1l	0.777	http://api.brain-map.org/grid_data/download/1675?include=energy
Jhdm1d	0.777	http://api.brain-map.org/grid_data/download/74514186?include=energy
6430550D23Rik	0.777	http://api.brain-map.org/grid_data/download/71493807?include=energy
Krtap5-5	0.777	http://api.brain-map.org/grid_data/download/70228298?include=energy
Cd207	0.777	http://api.brain-map.org/grid_data/download/73512557?include=energy
Tbc1d20	0.777	http://api.brain-map.org/grid_data/download/68191162?include=energy
Pdzd3	0.777	http://api.brain-map.org/grid_data/download/75693104?include=energy
Lrch2	0.777	http://api.brain-map.org/grid_data/download/70546075?include=energy
1700010B08Rik	0.777	http://api.brain-map.org/grid_data/download/77799027?include=energy
Tbc1d15	0.777	http://api.brain-map.org/grid_data/download/70565294?include=energy
Ccl8	0.777	http://api.brain-map.org/grid_data/download/69540463?include=energy
Padi3	0.777	http://api.brain-map.org/grid_data/download/69782664?include=energy
Gfpt2	0.777	http://api.brain-map.org/grid_data/download/74425526?include=energy
9930021D14Rik	0.777	http://api.brain-map.org/grid_data/download/70228257?include=energy
Gm806	0.777	http://api.brain-map.org/grid_data/download/72471732?include=energy
Npffr2	0.777	http://api.brain-map.org/grid_data/download/70562070?include=energy
Mstn	0.777	http://api.brain-map.org/grid_data/download/69672850?include=energy
D930031A20Rik	0.777	http://api.brain-map.org/grid_data/download/71809461?include=energy
Traf6	0.777	http://api.brain-map.org/grid_data/download/68844534?include=energy
Slc5a4a	0.777	http://api.brain-map.org/grid_data/download/70785086?include=energy
Ccdc115	0.777	http://api.brain-map.org/grid_data/download/68633014?include=energy
Urb1	0.777	http://api.brain-map.org/grid_data/download/70303881?include=energy
LOC433113	0.777	http://api.brain-map.org/grid_data/download/71281892?include=energy
Cript	0.777	http://api.brain-map.org/grid_data/download/68196938?include=energy
Ceacam3	0.777	http://api.brain-map.org/grid_data/download/70608370?include=energy
Nepn	0.777	http://api.brain-map.org/grid_data/download/70218968?include=energy
4930589L23Rik	0.777	http://api.brain-map.org/grid_data/download/74580715?include=energy
Slc20a2	0.777	http://api.brain-map.org/grid_data/download/70231308?include=energy
Pcbp3	0.777	http://api.brain-map.org/grid_data/download/768?include=energy
Nwd1	0.777	http://api.brain-map.org/grid_data/download/69447966?include=energy
2010204K13Rik	0.776	http://api.brain-map.org/grid_data/download/70303773?include=energy
LOC432575	0.776	http://api.brain-map.org/grid_data/download/70725489?include=energy
D930030D11Rik	0.776	http://api.brain-map.org/grid_data/download/71496399?include=energy
Igf1	0.776	http://api.brain-map.org/grid_data/download/75197528?include=energy
Dpcr1	0.776	http://api.brain-map.org/grid_data/download/70298208?include=energy
Mrpl32	0.776	http://api.brain-map.org/grid_data/download/68667121?include=energy
Mctp1	0.776	http://api.brain-map.org/grid_data/download/75985954?include=energy
Srsf6	0.776	http://api.brain-map.org/grid_data/download/413829?include=energy
Tusc2	0.776	http://api.brain-map.org/grid_data/download/71280727?include=energy
Gm57	0.776	http://api.brain-map.org/grid_data/download/70295536?include=energy
Tyro3	0.776	http://api.brain-map.org/grid_data/download/72119607?include=energy
Ankra2	0.776	http://api.brain-map.org/grid_data/download/75985901?include=energy
Zcchc3	0.776	http://api.brain-map.org/grid_data/download/70785744?include=energy
Fam53b	0.776	http://api.brain-map.org/grid_data/download/678692?include=energy
Zbtb44	0.776	http://api.brain-map.org/grid_data/download/71283559?include=energy
Prickle1	0.776	http://api.brain-map.org/grid_data/download/69817294?include=energy
Csnk1d	0.776	http://api.brain-map.org/grid_data/download/70918766?include=energy
Slc25a19	0.776	http://api.brain-map.org/grid_data/download/68797497?include=energy
Psmb8	0.776	http://api.brain-map.org/grid_data/download/68269544?include=energy
Figf	0.776	http://api.brain-map.org/grid_data/download/68911007?include=energy
Pin1	0.776	http://api.brain-map.org/grid_data/download/69783196?include=energy
Hoxa6	0.776	http://api.brain-map.org/grid_data/download/69816595?include=energy
Gpr150	0.776	http://api.brain-map.org/grid_data/download/69257462?include=energy
Tlr5	0.776	http://api.brain-map.org/grid_data/download/70302527?include=energy
Dcaf12	0.776	http://api.brain-map.org/grid_data/download/68158072?include=energy
4932415G12Rik	0.776	http://api.brain-map.org/grid_data/download/70218570?include=energy
Mettl7a1	0.776	http://api.brain-map.org/grid_data/download/70919931?include=energy
Mfge8	0.776	http://api.brain-map.org/grid_data/download/71763474?include=energy
Mon1a	0.776	http://api.brain-map.org/grid_data/download/69262345?include=energy
Ccdc117	0.776	http://api.brain-map.org/grid_data/download/68498277?include=energy
Tmem139	0.776	http://api.brain-map.org/grid_data/download/70228645?include=energy
Ostb	0.776	http://api.brain-map.org/grid_data/download/74427088?include=energy
Meis1	0.776	http://api.brain-map.org/grid_data/download/74047866?include=energy
Armc2	0.776	http://api.brain-map.org/grid_data/download/74988750?include=energy
Bcl2l10	0.776	http://api.brain-map.org/grid_data/download/68667449?include=energy
L2hgdh	0.776	http://api.brain-map.org/grid_data/download/68342252?include=energy
Ptprj	0.776	http://api.brain-map.org/grid_data/download/73521803?include=energy
Oas1f	0.776	http://api.brain-map.org/grid_data/download/70784714?include=energy
Vmn1r89	0.775	http://api.brain-map.org/grid_data/download/70598373?include=energy
Tmem202	0.775	http://api.brain-map.org/grid_data/download/69113937?include=energy
Tmem169	0.775	http://api.brain-map.org/grid_data/download/70717977?include=energy
Tbl1x	0.775	http://api.brain-map.org/grid_data/download/70919609?include=energy
F530002G13Rik*	0.775	http://api.brain-map.org/grid_data/download/73767406?include=energy
Cml1	0.775	http://api.brain-map.org/grid_data/download/74056108?include=energy
Ypel4	0.775	http://api.brain-map.org/grid_data/download/71213204?include=energy
Gabra2	0.775	http://api.brain-map.org/grid_data/download/74988690?include=energy
BC089597	0.775	http://api.brain-map.org/grid_data/download/70524270?include=energy
Vmn1r15	0.775	http://api.brain-map.org/grid_data/download/70785267?include=energy
LOC433963	0.775	http://api.brain-map.org/grid_data/download/70787176?include=energy
LOC434300	0.775	http://api.brain-map.org/grid_data/download/75988611?include=energy
Rfx2	0.775	http://api.brain-map.org/grid_data/download/74265297?include=energy
Hpse2	0.775	http://api.brain-map.org/grid_data/download/70298519?include=energy
B430212C06Rik	0.775	http://api.brain-map.org/grid_data/download/100144924?include=energy
Slc22a12	0.775	http://api.brain-map.org/grid_data/download/68632697?include=energy
Tha1	0.775	http://api.brain-map.org/grid_data/download/70598391?include=energy
Mpz	0.775	http://api.brain-map.org/grid_data/download/71280532?include=energy
4930453L07Rik	0.775	http://api.brain-map.org/grid_data/download/70218163?include=energy
LOC435906	0.775	http://api.brain-map.org/grid_data/download/70784552?include=energy
Zfp280d	0.775	http://api.brain-map.org/grid_data/download/67881771?include=energy
BC049688	0.775	http://api.brain-map.org/grid_data/download/73716632?include=energy
Mm.360391	0.775	http://api.brain-map.org/grid_data/download/71209988?include=energy
Cldn17	0.775	http://api.brain-map.org/grid_data/download/70237938?include=energy
AI504432	0.775	http://api.brain-map.org/grid_data/download/69524633?include=energy
9130011J15Rik	0.775	http://api.brain-map.org/grid_data/download/69446352?include=energy
C130079G13Rik	0.775	http://api.brain-map.org/grid_data/download/72183006?include=energy
A230004C22Rik*	0.775	http://api.brain-map.org/grid_data/download/73949539?include=energy
Snx12	0.775	http://api.brain-map.org/grid_data/download/68269270?include=energy
Chtf18	0.775	http://api.brain-map.org/grid_data/download/68499237?include=energy
Klf8	0.775	http://api.brain-map.org/grid_data/download/69289297?include=energy
2010011I20Rik	0.775	http://api.brain-map.org/grid_data/download/641524?include=energy
Irx5	0.775	http://api.brain-map.org/grid_data/download/74822916?include=energy
4932429F07Rik*	0.775	http://api.brain-map.org/grid_data/download/73870686?include=energy
Ankrd45	0.775	http://api.brain-map.org/grid_data/download/69113877?include=energy
Tns4	0.775	http://api.brain-map.org/grid_data/download/69838570?include=energy
Fancd2	0.775	http://api.brain-map.org/grid_data/download/73635979?include=energy
LOC381556	0.775	http://api.brain-map.org/grid_data/download/74514300?include=energy
D030056L22Rik	0.775	http://api.brain-map.org/grid_data/download/587413?include=energy
Gucy2e	0.775	http://api.brain-map.org/grid_data/download/69015356?include=energy
Polr2c	0.775	http://api.brain-map.org/grid_data/download/76081912?include=energy
Ikzf4	0.775	http://api.brain-map.org/grid_data/download/74430990?include=energy
Mrpl55	0.775	http://api.brain-map.org/grid_data/download/68545477?include=energy
Kctd13	0.775	http://api.brain-map.org/grid_data/download/71488716?include=energy
Tcf12	0.775	http://api.brain-map.org/grid_data/download/69549664?include=energy
Esrp2	0.775	http://api.brain-map.org/grid_data/download/69549294?include=energy
Mmp25	0.775	http://api.brain-map.org/grid_data/download/70946593?include=energy
Galntl2	0.775	http://api.brain-map.org/grid_data/download/69672772?include=energy
Grrp1	0.774	http://api.brain-map.org/grid_data/download/69855307?include=energy
Etfdh	0.774	http://api.brain-map.org/grid_data/download/68269652?include=energy
Zfp191	0.774	http://api.brain-map.org/grid_data/download/1894?include=energy
Zfp322a	0.774	http://api.brain-map.org/grid_data/download/70785756?include=energy
Abca12	0.774	http://api.brain-map.org/grid_data/download/72471693?include=energy
A630052C17Rik	0.774	http://api.brain-map.org/grid_data/download/69448936?include=energy
Fam129b	0.774	http://api.brain-map.org/grid_data/download/68499016?include=energy
1700029G01Rik	0.774	http://api.brain-map.org/grid_data/download/69114109?include=energy
Crp	0.774	http://api.brain-map.org/grid_data/download/68302221?include=energy
Jakmip2	0.774	http://api.brain-map.org/grid_data/download/72076593?include=energy
C130070B15Rik	0.774	http://api.brain-map.org/grid_data/download/70231881?include=energy
E130102H24Rik	0.774	http://api.brain-map.org/grid_data/download/74580954?include=energy
Gtf2h1	0.774	http://api.brain-map.org/grid_data/download/68667419?include=energy
Tcl1b1	0.774	http://api.brain-map.org/grid_data/download/68667413?include=energy
Eif2b2	0.774	http://api.brain-map.org/grid_data/download/67978649?include=energy
Bcl11a	0.774	http://api.brain-map.org/grid_data/download/71836837?include=energy
Kcnj14	0.774	http://api.brain-map.org/grid_data/download/67853369?include=energy
Pln	0.774	http://api.brain-map.org/grid_data/download/74357563?include=energy
Dhtkd1	0.774	http://api.brain-map.org/grid_data/download/100145309?include=energy
Ces2a	0.774	http://api.brain-map.org/grid_data/download/75861873?include=energy
Tsga14	0.774	http://api.brain-map.org/grid_data/download/68151448?include=energy
Rfc1	0.774	http://api.brain-map.org/grid_data/download/69816780?include=energy
Amot	0.774	http://api.brain-map.org/grid_data/download/74425553?include=energy
LOC381815	0.774	http://api.brain-map.org/grid_data/download/70725079?include=energy
Mm.37454	0.774	http://api.brain-map.org/grid_data/download/71210091?include=energy
LOC381357	0.774	http://api.brain-map.org/grid_data/download/70596047?include=energy
Tspan4	0.774	http://api.brain-map.org/grid_data/download/80471945?include=energy
Prkaa1	0.774	http://api.brain-map.org/grid_data/download/74635797?include=energy
Abhd11	0.774	http://api.brain-map.org/grid_data/download/1577?include=energy
Lrp8	0.774	http://api.brain-map.org/grid_data/download/74047715?include=energy
Stim2	0.774	http://api.brain-map.org/grid_data/download/73929869?include=energy
Pygo1	0.774	http://api.brain-map.org/grid_data/download/69289769?include=energy
Ankrd12	0.774	http://api.brain-map.org/grid_data/download/73513410?include=energy
Prokr1	0.774	http://api.brain-map.org/grid_data/download/74724728?include=energy
Utf1	0.774	http://api.brain-map.org/grid_data/download/69817073?include=energy
Snx4	0.774	http://api.brain-map.org/grid_data/download/70474017?include=energy
Nrf1	0.774	http://api.brain-map.org/grid_data/download/2438?include=energy
Adora1	0.774	http://api.brain-map.org/grid_data/download/73496905?include=energy
Ccr6	0.774	http://api.brain-map.org/grid_data/download/69237035?include=energy
Sec24b	0.774	http://api.brain-map.org/grid_data/download/68920407?include=energy
Zfp42	0.774	http://api.brain-map.org/grid_data/download/68667581?include=energy
Hao1	0.774	http://api.brain-map.org/grid_data/download/70277919?include=energy
Serpinb5	0.774	http://api.brain-map.org/grid_data/download/68443172?include=energy
Usp44	0.774	http://api.brain-map.org/grid_data/download/70239057?include=energy
Fcho2	0.774	http://api.brain-map.org/grid_data/download/68667593?include=energy
Zdhhc25	0.774	http://api.brain-map.org/grid_data/download/69114950?include=energy
2210404O07Rik	0.774	http://api.brain-map.org/grid_data/download/69202268?include=energy
Gm4955	0.774	http://api.brain-map.org/grid_data/download/70566280?include=energy
Csnk2b	0.773	http://api.brain-map.org/grid_data/download/68076534?include=energy
Wdr81	0.773	http://api.brain-map.org/grid_data/download/69838749?include=energy
LOC432874	0.773	http://api.brain-map.org/grid_data/download/74431014?include=energy
Aspn	0.773	http://api.brain-map.org/grid_data/download/68744677?include=energy
Fam166b	0.773	http://api.brain-map.org/grid_data/download/70205590?include=energy
Crispld1	0.773	http://api.brain-map.org/grid_data/download/72077456?include=energy
4631405J19Rik	0.773	http://api.brain-map.org/grid_data/download/70303839?include=energy
Oas1d	0.773	http://api.brain-map.org/grid_data/download/68667697?include=energy
1500015L24Rik	0.773	http://api.brain-map.org/grid_data/download/70598395?include=energy
Taar2	0.773	http://api.brain-map.org/grid_data/download/73573229?include=energy
Fam120c	0.773	http://api.brain-map.org/grid_data/download/74581430?include=energy
Slc25a21	0.773	http://api.brain-map.org/grid_data/download/69015416?include=energy
Spag11a	0.773	http://api.brain-map.org/grid_data/download/70302240?include=energy
Lca5l	0.773	http://api.brain-map.org/grid_data/download/70303899?include=energy
Ms4a5	0.773	http://api.brain-map.org/grid_data/download/69116387?include=energy
Btnl2	0.773	http://api.brain-map.org/grid_data/download/77620269?include=energy
Pycard	0.773	http://api.brain-map.org/grid_data/download/68269285?include=energy
Ndufaf1	0.773	http://api.brain-map.org/grid_data/download/583861?include=energy
Polh	0.773	http://api.brain-map.org/grid_data/download/69817151?include=energy
Cldn15	0.773	http://api.brain-map.org/grid_data/download/68441478?include=energy
Igfn1	0.773	http://api.brain-map.org/grid_data/download/75988547?include=energy
4930503B20Rik	0.773	http://api.brain-map.org/grid_data/download/69114441?include=energy
Zfc3h1	0.773	http://api.brain-map.org/grid_data/download/70231725?include=energy
Muc4	0.773	http://api.brain-map.org/grid_data/download/73423862?include=energy
5330437I02Rik	0.773	http://api.brain-map.org/grid_data/download/69443391?include=energy
Pcmt1	0.773	http://api.brain-map.org/grid_data/download/73423926?include=energy
LOC382133	0.773	http://api.brain-map.org/grid_data/download/69114884?include=energy
Lmx1a	0.773	http://api.brain-map.org/grid_data/download/74277747?include=energy
Baiap2l1	0.773	http://api.brain-map.org/grid_data/download/68076990?include=energy
Rsad2	0.773	http://api.brain-map.org/grid_data/download/69015123?include=energy
Atf6	0.773	http://api.brain-map.org/grid_data/download/69288656?include=energy
Lyl1	0.773	http://api.brain-map.org/grid_data/download/386239?include=energy
F930043J12Rik*	0.773	http://api.brain-map.org/grid_data/download/73808135?include=energy
Gpr35	0.773	http://api.brain-map.org/grid_data/download/112649690?include=energy
Cpne6	0.773	http://api.brain-map.org/grid_data/download/73769322?include=energy
Prr14	0.773	http://api.brain-map.org/grid_data/download/413775?include=energy
A730085E03Rik	0.773	http://api.brain-map.org/grid_data/download/71494738?include=energy
Adck4	0.773	http://api.brain-map.org/grid_data/download/70743880?include=energy
Pip5k1b	0.773	http://api.brain-map.org/grid_data/download/74425520?include=energy
6230409E13Rik	0.773	http://api.brain-map.org/grid_data/download/68845215?include=energy
Cyp21a1	0.773	http://api.brain-map.org/grid_data/download/75931487?include=energy
Slc44a3	0.772	http://api.brain-map.org/grid_data/download/68076911?include=energy
LOC269472	0.772	http://api.brain-map.org/grid_data/download/68744713?include=energy
Rab10	0.772	http://api.brain-map.org/grid_data/download/69817799?include=energy
2810422O20Rik	0.772	http://api.brain-map.org/grid_data/download/68666989?include=energy
Nop2	0.772	http://api.brain-map.org/grid_data/download/532574?include=energy
Zfp346	0.772	http://api.brain-map.org/grid_data/download/68667817?include=energy
Ckap2l	0.772	http://api.brain-map.org/grid_data/download/68797882?include=energy
Tbx3	0.772	http://api.brain-map.org/grid_data/download/70303642?include=energy
Gatsl3	0.772	http://api.brain-map.org/grid_data/download/68546149?include=energy
Ptpn2	0.772	http://api.brain-map.org/grid_data/download/77413686?include=energy
Vgll4	0.772	http://api.brain-map.org/grid_data/download/69262349?include=energy
BC061237	0.772	http://api.brain-map.org/grid_data/download/70526065?include=energy
Pdgfc	0.772	http://api.brain-map.org/grid_data/download/79360310?include=energy
Nadk	0.772	http://api.brain-map.org/grid_data/download/532589?include=energy
Slc38a9	0.772	http://api.brain-map.org/grid_data/download/69113943?include=energy
Lsm6	0.772	http://api.brain-map.org/grid_data/download/68162214?include=energy
Celf5	0.772	http://api.brain-map.org/grid_data/download/73513597?include=energy
Kctd17	0.772	http://api.brain-map.org/grid_data/download/72340180?include=energy
Ccdc9	0.772	http://api.brain-map.org/grid_data/download/634460?include=energy
Fgr	0.772	http://api.brain-map.org/grid_data/download/75831773?include=energy
Cdkn1a	0.772	http://api.brain-map.org/grid_data/download/80343383?include=energy
Scel	0.772	http://api.brain-map.org/grid_data/download/69873433?include=energy
Cd70	0.772	http://api.brain-map.org/grid_data/download/69981194?include=energy
2810030E01Rik	0.772	http://api.brain-map.org/grid_data/download/68797912?include=energy
Mrpl46	0.772	http://api.brain-map.org/grid_data/download/69116769?include=energy
Nup62	0.772	http://api.brain-map.org/grid_data/download/70546305?include=energy
Atp8b5	0.772	http://api.brain-map.org/grid_data/download/69407923?include=energy
2210408I21Rik	0.772	http://api.brain-map.org/grid_data/download/74000648?include=energy
Atp6v0a4	0.772	http://api.brain-map.org/grid_data/download/68745582?include=energy
Ifit2	0.772	http://api.brain-map.org/grid_data/download/74988457?include=energy
Pprc1	0.772	http://api.brain-map.org/grid_data/download/513021?include=energy
Sel1l3	0.772	http://api.brain-map.org/grid_data/download/74988657?include=energy
P4ha3	0.772	http://api.brain-map.org/grid_data/download/69782634?include=energy
Dennd1a	0.772	http://api.brain-map.org/grid_data/download/68157417?include=energy
Cabp5	0.772	http://api.brain-map.org/grid_data/download/68443321?include=energy
Hn1l	0.772	http://api.brain-map.org/grid_data/download/69262252?include=energy
Alg6	0.772	http://api.brain-map.org/grid_data/download/77456081?include=energy
Hpse	0.771	http://api.brain-map.org/grid_data/download/77340468?include=energy
Dkkl1	0.771	http://api.brain-map.org/grid_data/download/70634395?include=energy
Zfp300	0.771	http://api.brain-map.org/grid_data/download/70593299?include=energy
Thumpd2	0.771	http://api.brain-map.org/grid_data/download/71213144?include=energy
Col5a3	0.771	http://api.brain-map.org/grid_data/download/74272917?include=energy
Mpeg1	0.771	http://api.brain-map.org/grid_data/download/71020765?include=energy
Rasef	0.771	http://api.brain-map.org/grid_data/download/71735823?include=energy
Ctrb1	0.771	http://api.brain-map.org/grid_data/download/69608214?include=energy
Dis3	0.771	http://api.brain-map.org/grid_data/download/73512564?include=energy
Akr1d1	0.771	http://api.brain-map.org/grid_data/download/68637659?include=energy
Galntl4	0.771	http://api.brain-map.org/grid_data/download/70928328?include=energy
Brf1	0.771	http://api.brain-map.org/grid_data/download/68845191?include=energy
Egfl6	0.771	http://api.brain-map.org/grid_data/download/70239093?include=energy

Hnrnpk	0.771	http://api.brain-map.org/grid_data/download/68443003?include=energy
Chl1	0.771	http://api.brain-map.org/grid_data/download/74272476?include=energy
Znf512b	0.771	http://api.brain-map.org/grid_data/download/69734440?include=energy
Zfp82	0.771	http://api.brain-map.org/grid_data/download/71488919?include=energy
Sytl4	0.771	http://api.brain-map.org/grid_data/download/75651223?include=energy
Mphosph6	0.771	http://api.brain-map.org/grid_data/download/68269052?include=energy
Gng10	0.771	http://api.brain-map.org/grid_data/download/71280814?include=energy
AI428898	0.771	http://api.brain-map.org/grid_data/download/71212563?include=energy
Scpep1	0.771	http://api.brain-map.org/grid_data/download/67979030?include=energy
B3gnt5	0.771	http://api.brain-map.org/grid_data/download/73992711?include=energy
Kdm2b	0.771	http://api.brain-map.org/grid_data/download/68844630?include=energy
Edn2	0.771	http://api.brain-map.org/grid_data/download/68162247?include=energy
Gm10635	0.771	http://api.brain-map.org/grid_data/download/75144655?include=energy
TC1430172	0.771	http://api.brain-map.org/grid_data/download/70795391?include=energy
Rimbp3	0.771	http://api.brain-map.org/grid_data/download/70566268?include=energy
Itgam	0.771	http://api.brain-map.org/grid_data/download/69735604?include=energy
Vmn2r-ps105	0.771	http://api.brain-map.org/grid_data/download/71789915?include=energy
Zcchc2	0.771	http://api.brain-map.org/grid_data/download/70303040?include=energy
Lilrb4	0.771	http://api.brain-map.org/grid_data/download/68795454?include=energy
Zfp607	0.771	http://api.brain-map.org/grid_data/download/73497792?include=energy
1700026N04Rik	0.771	http://api.brain-map.org/grid_data/download/71919529?include=energy
Tnfrsf25	0.771	http://api.brain-map.org/grid_data/download/121096?include=energy
Olfr132	0.771	http://api.brain-map.org/grid_data/download/74720693?include=energy
Prnd	0.771	http://api.brain-map.org/grid_data/download/67881780?include=energy
Alb	0.771	http://api.brain-map.org/grid_data/download/68444982?include=energy
Olfr690	0.771	http://api.brain-map.org/grid_data/download/74734860?include=energy
Srebf1	0.771	http://api.brain-map.org/grid_data/download/68919848?include=energy
Tas2r108	0.771	http://api.brain-map.org/grid_data/download/70302330?include=energy
Mustn1	0.771	http://api.brain-map.org/grid_data/download/73635495?include=energy
Cilp	0.77	http://api.brain-map.org/grid_data/download/71021157?include=energy
Zfp648	0.77	http://api.brain-map.org/grid_data/download/100144945?include=energy
Sobp	0.77	http://api.brain-map.org/grid_data/download/71920521?include=energy
Hspa1l	0.77	http://api.brain-map.org/grid_data/download/75041442?include=energy
C230049M14Rik*	0.77	http://api.brain-map.org/grid_data/download/73811663?include=energy
Pex5	0.77	http://api.brain-map.org/grid_data/download/68546287?include=energy
Tas1r3	0.77	http://api.brain-map.org/grid_data/download/69863262?include=energy
Tspan18	0.77	http://api.brain-map.org/grid_data/download/74357573?include=energy
Ugt2b5	0.77	http://api.brain-map.org/grid_data/download/68442883?include=energy
Zfp248	0.77	http://api.brain-map.org/grid_data/download/71380447?include=energy
Mrps25	0.77	http://api.brain-map.org/grid_data/download/70546215?include=energy
Adig	0.77	http://api.brain-map.org/grid_data/download/69835567?include=energy
Meig1	0.77	http://api.brain-map.org/grid_data/download/80527163?include=energy
Bai1	0.77	http://api.brain-map.org/grid_data/download/71283681?include=energy
Pcdhb10	0.77	http://api.brain-map.org/grid_data/download/79354816?include=energy
Pde6g	0.77	http://api.brain-map.org/grid_data/download/72104173?include=energy
Zmat1	0.77	http://api.brain-map.org/grid_data/download/70231224?include=energy
Fam133b	0.77	http://api.brain-map.org/grid_data/download/68157396?include=energy
Anks6	0.77	http://api.brain-map.org/grid_data/download/71492976?include=energy
Lyst	0.77	http://api.brain-map.org/grid_data/download/73994715?include=energy
2810025M15Rik	0.77	http://api.brain-map.org/grid_data/download/70524258?include=energy
Gm1698	0.77	http://api.brain-map.org/grid_data/download/74562570?include=energy
Cplx3	0.77	http://api.brain-map.org/grid_data/download/70928340?include=energy
Lrrc42	0.77	http://api.brain-map.org/grid_data/download/68151420?include=energy
Mat1a	0.77	http://api.brain-map.org/grid_data/download/75826650?include=energy
Lin7a	0.77	http://api.brain-map.org/grid_data/download/73520997?include=energy
Ska1	0.77	http://api.brain-map.org/grid_data/download/71627816?include=energy
Fosl2	0.77	http://api.brain-map.org/grid_data/download/1720?include=energy
Ttc23	0.77	http://api.brain-map.org/grid_data/download/70445425?include=energy
Fam69c	0.77	http://api.brain-map.org/grid_data/download/73636086?include=energy
Aqp2	0.77	http://api.brain-map.org/grid_data/download/112195601?include=energy
B3gnt4	0.77	http://api.brain-map.org/grid_data/download/70231176?include=energy
Patl1	0.77	http://api.brain-map.org/grid_data/download/68919012?include=energy
Col4a6	0.77	http://api.brain-map.org/grid_data/download/72103841?include=energy
Osgep	0.77	http://api.brain-map.org/grid_data/download/413823?include=energy
Ccny	0.77	http://api.brain-map.org/grid_data/download/67881782?include=energy
Cdc25b	0.77	http://api.brain-map.org/grid_data/download/74274686?include=energy
Fam71d	0.77	http://api.brain-map.org/grid_data/download/69113728?include=energy
Mllt4	0.769	http://api.brain-map.org/grid_data/download/73423817?include=energy
1700012B07Rik	0.769	http://api.brain-map.org/grid_data/download/72472474?include=energy
Kmo	0.769	http://api.brain-map.org/grid_data/download/68666768?include=energy
Srms	0.769	http://api.brain-map.org/grid_data/download/71280619?include=energy
A230081H15Rik	0.769	http://api.brain-map.org/grid_data/download/70608523?include=energy
Dpagt1	0.769	http://api.brain-map.org/grid_data/download/631706?include=energy
Zfp748	0.769	http://api.brain-map.org/grid_data/download/68667724?include=energy
Kctd5	0.769	http://api.brain-map.org/grid_data/download/1629?include=energy
Fcer1g	0.769	http://api.brain-map.org/grid_data/download/100144165?include=energy
Ugt2a1	0.769	http://api.brain-map.org/grid_data/download/75831713?include=energy
Pcdha6	0.769	http://api.brain-map.org/grid_data/download/70784824?include=energy
Oxtr	0.769	http://api.brain-map.org/grid_data/download/75081001?include=energy
Cacfd1	0.769	http://api.brain-map.org/grid_data/download/74431804?include=energy
Hunk	0.769	http://api.brain-map.org/grid_data/download/2307?include=energy
Odz4	0.769	http://api.brain-map.org/grid_data/download/754?include=energy
Scfd1	0.769	http://api.brain-map.org/grid_data/download/71763870?include=energy
Ckmt2	0.769	http://api.brain-map.org/grid_data/download/70543062?include=energy
1110005A03Rik	0.769	http://api.brain-map.org/grid_data/download/68498283?include=energy
Gm20557	0.769	http://api.brain-map.org/grid_data/download/70305036?include=energy
Fam57a	0.769	http://api.brain-map.org/grid_data/download/69202496?include=energy
Dcbld2	0.769	http://api.brain-map.org/grid_data/download/73992944?include=energy
Dpp3	0.769	http://api.brain-map.org/grid_data/download/532634?include=energy
Phf8	0.769	http://api.brain-map.org/grid_data/download/73906113?include=energy
2210012G02Rik	0.769	http://api.brain-map.org/grid_data/download/69549184?include=energy
Ccnj	0.769	http://api.brain-map.org/grid_data/download/69540525?include=energy
LOC435287	0.769	http://api.brain-map.org/grid_data/download/71022457?include=energy
Gata1	0.769	http://api.brain-map.org/grid_data/download/77454718?include=energy
Abcc1	0.769	http://api.brain-map.org/grid_data/download/69514594?include=energy
Nnmt	0.769	http://api.brain-map.org/grid_data/download/68523168?include=energy
Gm4774	0.769	http://api.brain-map.org/grid_data/download/70724977?include=energy
Gpr111	0.769	http://api.brain-map.org/grid_data/download/71281967?include=energy
Whsc1l1	0.769	http://api.brain-map.org/grid_data/download/74562588?include=energy
Fcrla	0.769	http://api.brain-map.org/grid_data/download/69528088?include=energy
Nudt16	0.769	http://api.brain-map.org/grid_data/download/75828199?include=energy
Itgb3	0.769	http://api.brain-map.org/grid_data/download/69735628?include=energy
Hdac4	0.769	http://api.brain-map.org/grid_data/download/70218534?include=energy
Nhlrc3	0.769	http://api.brain-map.org/grid_data/download/71493831?include=energy
C330046G03Rik	0.769	http://api.brain-map.org/grid_data/download/70231947?include=energy
Nploc4	0.769	http://api.brain-map.org/grid_data/download/69526498?include=energy
Mm.316532	0.769	http://api.brain-map.org/grid_data/download/71162004?include=energy
Epha4	0.769	http://api.brain-map.org/grid_data/download/2187?include=energy
Tmem159	0.769	http://api.brain-map.org/grid_data/download/76135828?include=energy
Sh3gl1	0.768	http://api.brain-map.org/grid_data/download/72001869?include=energy
Kcne1l	0.768	http://api.brain-map.org/grid_data/download/68196920?include=energy
C230015M01Rik*	0.768	http://api.brain-map.org/grid_data/download/73811675?include=energy
Rps13	0.768	http://api.brain-map.org/grid_data/download/68301867?include=energy
Klf13	0.768	http://api.brain-map.org/grid_data/download/74581020?include=energy
9330188P03Rik	0.768	http://api.brain-map.org/grid_data/download/71494617?include=energy
Runx3	0.768	http://api.brain-map.org/grid_data/download/79544838?include=energy
Pm20d1	0.768	http://api.brain-map.org/grid_data/download/69407678?include=energy
Gimap6	0.768	http://api.brain-map.org/grid_data/download/68523270?include=energy
Sun1	0.768	http://api.brain-map.org/grid_data/download/74425548?include=energy
Pter	0.768	http://api.brain-map.org/grid_data/download/71763849?include=energy
Ptpmt1	0.768	http://api.brain-map.org/grid_data/download/74581056?include=energy
Gm378	0.768	http://api.brain-map.org/grid_data/download/70295673?include=energy
Mpdz	0.768	http://api.brain-map.org/grid_data/download/71020764?include=energy
St3gal2	0.768	http://api.brain-map.org/grid_data/download/70920051?include=energy
Ankle2	0.768	http://api.brain-map.org/grid_data/download/71283412?include=energy
Gm561	0.768	http://api.brain-map.org/grid_data/download/70295876?include=energy
Dmrta1	0.768	http://api.brain-map.org/grid_data/download/69288902?include=energy
Slc22a4	0.768	http://api.brain-map.org/grid_data/download/78534492?include=energy
Fam53c	0.768	http://api.brain-map.org/grid_data/download/69855160?include=energy
E2f8	0.768	http://api.brain-map.org/grid_data/download/70946182?include=energy
Ranbp3l	0.768	http://api.brain-map.org/grid_data/download/69530792?include=energy
Sntb2	0.768	http://api.brain-map.org/grid_data/download/69885594?include=energy
Cd209b	0.768	http://api.brain-map.org/grid_data/download/70724631?include=energy
Pno1	0.768	http://api.brain-map.org/grid_data/download/587421?include=energy
Hyal6	0.768	http://api.brain-map.org/grid_data/download/70218588?include=energy
Tap1	0.768	http://api.brain-map.org/grid_data/download/68301551?include=energy
Adat2	0.768	http://api.brain-map.org/grid_data/download/68745173?include=energy
Skint3	0.768	http://api.brain-map.org/grid_data/download/74509446?include=energy
Klhl32	0.768	http://api.brain-map.org/grid_data/download/74578218?include=energy
Tpsg1	0.768	http://api.brain-map.org/grid_data/download/576444?include=energy
Ptprg	0.768	http://api.brain-map.org/grid_data/download/74430888?include=energy
Tmem194	0.768	http://api.brain-map.org/grid_data/download/68162216?include=energy
Insr	0.768	http://api.brain-map.org/grid_data/download/69735484?include=energy
Zbtb22	0.768	http://api.brain-map.org/grid_data/download/67814219?include=energy
Rheb	0.768	http://api.brain-map.org/grid_data/download/564750?include=energy
Gja8	0.768	http://api.brain-map.org/grid_data/download/69257337?include=energy
Mrpl11	0.768	http://api.brain-map.org/grid_data/download/74003384?include=energy
LOC434100	0.768	http://api.brain-map.org/grid_data/download/70787408?include=energy
Cstf2t	0.768	http://api.brain-map.org/grid_data/download/68196929?include=energy
Rnft1	0.767	http://api.brain-map.org/grid_data/download/69114620?include=energy
Gatc	0.767	http://api.brain-map.org/grid_data/download/71492922?include=energy
LOC435849	0.767	http://api.brain-map.org/grid_data/download/70784546?include=energy
9130024F11Rik	0.767	http://api.brain-map.org/grid_data/download/71?include=energy
Dlk2	0.767	http://api.brain-map.org/grid_data/download/69261390?include=energy
Rnf181	0.767	http://api.brain-map.org/grid_data/download/70474941?include=energy
Sigmar1	0.767	http://api.brain-map.org/grid_data/download/112196265?include=energy
Fam110a	0.767	http://api.brain-map.org/grid_data/download/68633410?include=energy
LOC434408	0.767	http://api.brain-map.org/grid_data/download/71022280?include=energy
Tmem188	0.767	http://api.brain-map.org/grid_data/download/69443274?include=energy
Olfr450	0.767	http://api.brain-map.org/grid_data/download/74724109?include=energy
Plekhg5	0.767	http://api.brain-map.org/grid_data/download/74881855?include=energy
4930579C15Rik	0.767	http://api.brain-map.org/grid_data/download/71493669?include=energy
Clec2h	0.767	http://api.brain-map.org/grid_data/download/75827130?include=energy
Olfr1320	0.767	http://api.brain-map.org/grid_data/download/74720699?include=energy
C630004L07Rik	0.767	http://api.brain-map.org/grid_data/download/73808385?include=energy
Myl12a	0.767	http://api.brain-map.org/grid_data/download/68076973?include=energy
Tmem211	0.767	http://api.brain-map.org/grid_data/download/70595895?include=energy
Snhg6	0.767	http://api.brain-map.org/grid_data/download/71212416?include=energy
Orc1	0.767	http://api.brain-map.org/grid_data/download/70546323?include=energy
Etl4	0.767	http://api.brain-map.org/grid_data/download/70920069?include=energy
Prune	0.767	http://api.brain-map.org/grid_data/download/68861803?include=energy
LOC640610	0.767	http://api.brain-map.org/grid_data/download/72283653?include=energy
Kit	0.767	http://api.brain-map.org/grid_data/download/73520994?include=energy
Prr5	0.767	http://api.brain-map.org/grid_data/download/68151343?include=energy
Smarce1	0.767	http://api.brain-map.org/grid_data/download/70439153?include=energy
Slc25a18	0.767	http://api.brain-map.org/grid_data/download/71210908?include=energy
Stx6	0.767	http://api.brain-map.org/grid_data/download/71724732?include=energy
Sult1c1	0.767	http://api.brain-map.org/grid_data/download/68745498?include=energy
Serpinb9b	0.767	http://api.brain-map.org/grid_data/download/70302033?include=energy
Ugt2b35	0.767	http://api.brain-map.org/grid_data/download/71735067?include=energy
Zbtb24	0.767	http://api.brain-map.org/grid_data/download/73592542?include=energy
Kcnk13	0.767	http://api.brain-map.org/grid_data/download/1762?include=energy
Taf1b	0.767	http://api.brain-map.org/grid_data/download/69816960?include=energy
B430306N03Rik	0.767	http://api.brain-map.org/grid_data/download/70231206?include=energy
Tnmd	0.767	http://api.brain-map.org/grid_data/download/71924343?include=energy
Rpp38	0.767	http://api.brain-map.org/grid_data/download/68667793?include=energy
Wfdc1	0.767	http://api.brain-map.org/grid_data/download/70195018?include=energy
Zbtb8os	0.767	http://api.brain-map.org/grid_data/download/70305389?include=energy
AF067063	0.767	http://api.brain-map.org/grid_data/download/70724541?include=energy
Cdx4	0.767	http://api.brain-map.org/grid_data/download/69288848?include=energy
Rgnef	0.767	http://api.brain-map.org/grid_data/download/74272892?include=energy
4930433N12Rik	0.767	http://api.brain-map.org/grid_data/download/73617576?include=energy
Nme6	0.767	http://api.brain-map.org/grid_data/download/68191528?include=energy
Gnpda1	0.767	http://api.brain-map.org/grid_data/download/69734488?include=energy
Fkbp8	0.767	http://api.brain-map.org/grid_data/download/71487112?include=energy
Det1	0.767	http://api.brain-map.org/grid_data/download/68632026?include=energy
Wrap53	0.767	http://api.brain-map.org/grid_data/download/69080482?include=energy
Gm12429	0.767	http://api.brain-map.org/grid_data/download/77340488?include=energy
Cd300ld	0.767	http://api.brain-map.org/grid_data/download/75828019?include=energy
D930007P13Rik	0.766	http://api.brain-map.org/grid_data/download/71283319?include=energy
Arg1	0.766	http://api.brain-map.org/grid_data/download/68301276?include=energy
Rtp1	0.766	http://api.brain-map.org/grid_data/download/69734434?include=energy
Fam83a	0.766	http://api.brain-map.org/grid_data/download/69261420?include=energy
Gm1667	0.766	http://api.brain-map.org/grid_data/download/70299911?include=energy
Slc14a2	0.766	http://api.brain-map.org/grid_data/download/78534528?include=energy
C920012J21Rik*	0.766	http://api.brain-map.org/grid_data/download/73811705?include=energy
Vrtn	0.766	http://api.brain-map.org/grid_data/download/70611192?include=energy
Utp3	0.766	http://api.brain-map.org/grid_data/download/68196936?include=energy
Adamts13	0.766	http://api.brain-map.org/grid_data/download/74430936?include=energy
Nanp	0.766	http://api.brain-map.org/grid_data/download/583866?include=energy
TC1445293	0.766	http://api.brain-map.org/grid_data/download/70795515?include=energy
Snap29	0.766	http://api.brain-map.org/grid_data/download/68545717?include=energy
Usp32	0.766	http://api.brain-map.org/grid_data/download/70302938?include=energy
Taar1	0.766	http://api.brain-map.org/grid_data/download/69288369?include=energy
Tas2r136	0.766	http://api.brain-map.org/grid_data/download/74277434?include=energy
Cxcr3	0.766	http://api.brain-map.org/grid_data/download/70593137?include=energy
Dst	0.766	http://api.brain-map.org/grid_data/download/70238943?include=energy
2210018M11Rik	0.766	http://api.brain-map.org/grid_data/download/68191156?include=energy
Mc4r	0.766	http://api.brain-map.org/grid_data/download/79556630?include=energy
Ugt2b1	0.766	http://api.brain-map.org/grid_data/download/68445006?include=energy
Elk1	0.766	http://api.brain-map.org/grid_data/download/2185?include=energy
Dnd1	0.766	http://api.brain-map.org/grid_data/download/68545941?include=energy
Lyg2	0.766	http://api.brain-map.org/grid_data/download/74562260?include=energy
G630055F21Rik*	0.766	http://api.brain-map.org/grid_data/download/73731319?include=energy
Ensa	0.766	http://api.brain-map.org/grid_data/download/73513729?include=energy
Mrpl53	0.766	http://api.brain-map.org/grid_data/download/68499177?include=energy
Scara5	0.766	http://api.brain-map.org/grid_data/download/68342342?include=energy
Smc5	0.766	http://api.brain-map.org/grid_data/download/67792201?include=energy
Dnajb11	0.766	http://api.brain-map.org/grid_data/download/686194?include=energy
Fam20a	0.766	http://api.brain-map.org/grid_data/download/70744517?include=energy
8030462N17Rik	0.766	http://api.brain-map.org/grid_data/download/69446238?include=energy
Xrcc6	0.766	http://api.brain-map.org/grid_data/download/68631893?include=energy
Dolk	0.765	http://api.brain-map.org/grid_data/download/68196932?include=energy
Rpl18a	0.765	http://api.brain-map.org/grid_data/download/678716?include=energy
Kcna7	0.765	http://api.brain-map.org/grid_data/download/69258005?include=energy
Zfp1	0.765	http://api.brain-map.org/grid_data/download/70598368?include=energy
Brcc3	0.765	http://api.brain-map.org/grid_data/download/70431205?include=energy
4430402I18Rik	0.765	http://api.brain-map.org/grid_data/download/70204111?include=energy
Gm5737	0.765	http://api.brain-map.org/grid_data/download/71022639?include=energy
Pi4kb	0.765	http://api.brain-map.org/grid_data/download/69262117?include=energy
4931406C07Rik	0.765	http://api.brain-map.org/grid_data/download/68269520?include=energy
C130080K17Rik*	0.765	http://api.brain-map.org/grid_data/download/73811639?include=energy
Zfp503	0.765	http://api.brain-map.org/grid_data/download/138?include=energy
Slc25a32	0.765	http://api.brain-map.org/grid_data/download/67815538?include=energy
Cd200r3	0.765	http://api.brain-map.org/grid_data/download/71021130?include=energy
Crip1	0.765	http://api.brain-map.org/grid_data/download/68632643?include=energy
Khdrbs3	0.765	http://api.brain-map.org/grid_data/download/73769321?include=energy
Ifne	0.765	http://api.brain-map.org/grid_data/download/71281349?include=energy
Cirh1a	0.765	http://api.brain-map.org/grid_data/download/77925143?include=energy
C330049O21Rik	0.765	http://api.brain-map.org/grid_data/download/71021009?include=energy
Zkscan3	0.765	http://api.brain-map.org/grid_data/download/68442991?include=energy
Dse	0.765	http://api.brain-map.org/grid_data/download/68798837?include=energy
Sbno1	0.765	http://api.brain-map.org/grid_data/download/930?include=energy
LOC545142	0.765	http://api.brain-map.org/grid_data/download/74736414?include=energy
Wwox	0.765	http://api.brain-map.org/grid_data/download/72077451?include=energy
4930596D02Rik	0.765	http://api.brain-map.org/grid_data/download/70566250?include=energy
Chst9	0.765	http://api.brain-map.org/grid_data/download/69548933?include=energy
Gnl3	0.765	http://api.brain-map.org/grid_data/download/68191306?include=energy
Ubxn2b	0.765	http://api.brain-map.org/grid_data/download/69407433?include=energy
BC020535	0.765	http://api.brain-map.org/grid_data/download/70445622?include=energy
Fam101a	0.765	http://api.brain-map.org/grid_data/download/68797762?include=energy
Gm94	0.765	http://api.brain-map.org/grid_data/download/74635767?include=energy
A730049N16Rik	0.765	http://api.brain-map.org/grid_data/download/69448978?include=energy
Pomc	0.765	http://api.brain-map.org/grid_data/download/2493?include=energy
Pla2g15	0.765	http://api.brain-map.org/grid_data/download/68637545?include=energy
Lepre1	0.765	http://api.brain-map.org/grid_data/download/2371?include=energy
Wfikkn2	0.765	http://api.brain-map.org/grid_data/download/74359425?include=energy

Osta	0.765	http://api.brain-map.org/grid_data/download/68151348?include=energy
Ddx10	0.765	http://api.brain-map.org/grid_data/download/69626717?include=energy
Ptprh	0.765	http://api.brain-map.org/grid_data/download/75043671?include=energy
Echdc3	0.765	http://api.brain-map.org/grid_data/download/631725?include=energy
Rnf169	0.765	http://api.brain-map.org/grid_data/download/74359479?include=energy
Rrh	0.765	http://api.brain-map.org/grid_data/download/69012674?include=energy
Ednrb	0.765	http://api.brain-map.org/grid_data/download/74750017?include=energy
Mpv17	0.765	http://api.brain-map.org/grid_data/download/70525818?include=energy
Srd5a3	0.765	http://api.brain-map.org/grid_data/download/71393268?include=energy
Olfr283	0.765	http://api.brain-map.org/grid_data/download/73653368?include=energy
Polr2e	0.765	http://api.brain-map.org/grid_data/download/71211734?include=energy
Fbxo16	0.765	http://api.brain-map.org/grid_data/download/69672324?include=energy
B230373P09Rik	0.764	http://api.brain-map.org/grid_data/download/73520818?include=energy
Tmem119	0.764	http://api.brain-map.org/grid_data/download/68161453?include=energy
Gm14047	0.764	http://api.brain-map.org/grid_data/download/70305192?include=energy
4832428G11Rik	0.764	http://api.brain-map.org/grid_data/download/72182949?include=energy
A430080K08Rik*	0.764	http://api.brain-map.org/grid_data/download/73808570?include=energy
Cuedc1	0.764	http://api.brain-map.org/grid_data/download/532736?include=energy
1700034H14Rik	0.764	http://api.brain-map.org/grid_data/download/68498319?include=energy
Wnt5a	0.764	http://api.brain-map.org/grid_data/download/79556660?include=energy
Etaa1	0.764	http://api.brain-map.org/grid_data/download/67792210?include=energy
Bbip1	0.764	http://api.brain-map.org/grid_data/download/71211507?include=energy
Tgfbrap1	0.764	http://api.brain-map.org/grid_data/download/70203621?include=energy
Glp2r	0.764	http://api.brain-map.org/grid_data/download/69134304?include=energy
4933430I17Rik	0.764	http://api.brain-map.org/grid_data/download/72182959?include=energy
Suox	0.764	http://api.brain-map.org/grid_data/download/532698?include=energy
Uevld	0.764	http://api.brain-map.org/grid_data/download/69527024?include=energy
Zfp763	0.764	http://api.brain-map.org/grid_data/download/68667240?include=energy
Ngf	0.764	http://api.brain-map.org/grid_data/download/74581384?include=energy
Ier2	0.764	http://api.brain-map.org/grid_data/download/71836849?include=energy
Retnlg	0.764	http://api.brain-map.org/grid_data/download/70301013?include=energy
Uimc1	0.764	http://api.brain-map.org/grid_data/download/69873367?include=energy
Tmem183a	0.764	http://api.brain-map.org/grid_data/download/67978659?include=energy
4930528H21Rik	0.764	http://api.brain-map.org/grid_data/download/70218265?include=energy
Krt80	0.764	http://api.brain-map.org/grid_data/download/74724762?include=energy
D630004N19Rik	0.764	http://api.brain-map.org/grid_data/download/69608989?include=energy
Lin28a	0.764	http://api.brain-map.org/grid_data/download/69816619?include=energy
Rnf213	0.764	http://api.brain-map.org/grid_data/download/73949430?include=energy
Hoxb5	0.764	http://api.brain-map.org/grid_data/download/71380417?include=energy
Phf21b	0.764	http://api.brain-map.org/grid_data/download/69288596?include=energy
1110067D22Rik	0.764	http://api.brain-map.org/grid_data/download/532758?include=energy
Marveld3	0.764	http://api.brain-map.org/grid_data/download/70300282?include=energy
Shprh	0.764	http://api.brain-map.org/grid_data/download/71147918?include=energy
Sectm1b	0.764	http://api.brain-map.org/grid_data/download/68341436?include=energy
C5ar1	0.764	http://api.brain-map.org/grid_data/download/69236885?include=energy
BC067068	0.764	http://api.brain-map.org/grid_data/download/70231791?include=energy
3010065I17Rik*	0.764	http://api.brain-map.org/grid_data/download/74359580?include=energy
Acap2	0.764	http://api.brain-map.org/grid_data/download/71021148?include=energy
A1cf	0.764	http://api.brain-map.org/grid_data/download/70201618?include=energy
Smr2	0.764	http://api.brain-map.org/grid_data/download/69874186?include=energy
Col14a1	0.763	http://api.brain-map.org/grid_data/download/74658056?include=energy
Ccdc19	0.763	http://api.brain-map.org/grid_data/download/71210538?include=energy
2010305A19Rik	0.763	http://api.brain-map.org/grid_data/download/386031?include=energy
Sectm1a	0.763	http://api.brain-map.org/grid_data/download/68076971?include=energy
Axl	0.763	http://api.brain-map.org/grid_data/download/68798199?include=energy
Il4	0.763	http://api.brain-map.org/grid_data/download/68545899?include=energy
Crtc1	0.763	http://api.brain-map.org/grid_data/download/71250309?include=energy
D630042F21Rik	0.763	http://api.brain-map.org/grid_data/download/70238710?include=energy
Rap2a	0.763	http://api.brain-map.org/grid_data/download/71016692?include=energy
Wdr20a	0.763	http://api.brain-map.org/grid_data/download/70919268?include=energy
Foxn4	0.763	http://api.brain-map.org/grid_data/download/71307334?include=energy
Hoxb9	0.763	http://api.brain-map.org/grid_data/download/71393388?include=energy
Lmnb2	0.763	http://api.brain-map.org/grid_data/download/68845460?include=energy
Ints9	0.763	http://api.brain-map.org/grid_data/download/68151443?include=energy
Tmem69	0.763	http://api.brain-map.org/grid_data/download/70228561?include=energy
Rbp4	0.763	http://api.brain-map.org/grid_data/download/71016583?include=energy
Fam118b	0.763	http://api.brain-map.org/grid_data/download/68797511?include=energy
Rab5c	0.763	http://api.brain-map.org/grid_data/download/71486970?include=energy
Mm.360169	0.763	http://api.brain-map.org/grid_data/download/71209982?include=energy
Tex22	0.763	http://api.brain-map.org/grid_data/download/69114860?include=energy
Eif4b	0.763	http://api.brain-map.org/grid_data/download/70919489?include=energy
Hoxb8	0.763	http://api.brain-map.org/grid_data/download/100146157?include=energy
Fbxo45	0.763	http://api.brain-map.org/grid_data/download/68631862?include=energy
Trim45	0.763	http://api.brain-map.org/grid_data/download/71656937?include=energy
Srsf11	0.763	http://api.brain-map.org/grid_data/download/73808302?include=energy
Pgm1	0.763	http://api.brain-map.org/grid_data/download/68666947?include=energy
Topors	0.763	http://api.brain-map.org/grid_data/download/69549623?include=energy
Pcp2	0.763	http://api.brain-map.org/grid_data/download/655115?include=energy
Mageh1	0.763	http://api.brain-map.org/grid_data/download/17?include=energy
Nos1ap	0.763	http://api.brain-map.org/grid_data/download/77280574?include=energy
Gm280	0.763	http://api.brain-map.org/grid_data/download/73513807?include=energy
2900052N01Rik	0.763	http://api.brain-map.org/grid_data/download/70203591?include=energy
Trp53bp2	0.763	http://api.brain-map.org/grid_data/download/69838069?include=energy
Frem1	0.763	http://api.brain-map.org/grid_data/download/71281677?include=energy
9130022E12Rik*	0.763	http://api.brain-map.org/grid_data/download/73808198?include=energy
Slc22a20	0.763	http://api.brain-map.org/grid_data/download/68745378?include=energy
Olfr398	0.763	http://api.brain-map.org/grid_data/download/74724079?include=energy
Ces2g	0.763	http://api.brain-map.org/grid_data/download/68445053?include=energy
4933433C11Rik	0.763	http://api.brain-map.org/grid_data/download/71808834?include=energy
Bmp2	0.762	http://api.brain-map.org/grid_data/download/76003511?include=energy
Cacng3	0.762	http://api.brain-map.org/grid_data/download/73615560?include=energy
Cxcr5	0.762	http://api.brain-map.org/grid_data/download/69835573?include=energy
Gab2	0.762	http://api.brain-map.org/grid_data/download/69672701?include=energy
2310033P09Rik	0.762	http://api.brain-map.org/grid_data/download/631710?include=energy
Cyp4f14	0.762	http://api.brain-map.org/grid_data/download/68795449?include=energy
Med6	0.762	http://api.brain-map.org/grid_data/download/68269700?include=energy
Ak4	0.762	http://api.brain-map.org/grid_data/download/74641312?include=energy
Bcl9l	0.762	http://api.brain-map.org/grid_data/download/67979042?include=energy
Tmem70	0.762	http://api.brain-map.org/grid_data/download/564751?include=energy
Cap2	0.762	http://api.brain-map.org/grid_data/download/70436737?include=energy
Nipal1	0.762	http://api.brain-map.org/grid_data/download/70204105?include=energy
Gpr125	0.762	http://api.brain-map.org/grid_data/download/74988700?include=energy
Nubp2	0.762	http://api.brain-map.org/grid_data/download/513014?include=energy
Trim33	0.762	http://api.brain-map.org/grid_data/download/74277439?include=energy
9530009G21Rik	0.762	http://api.brain-map.org/grid_data/download/70228179?include=energy
Arhgap26	0.762	http://api.brain-map.org/grid_data/download/75080997?include=energy
Kcnh2	0.762	http://api.brain-map.org/grid_data/download/71393466?include=energy
1700080O16Rik	0.762	http://api.brain-map.org/grid_data/download/71920153?include=energy
Fam170b	0.762	http://api.brain-map.org/grid_data/download/70218061?include=energy
Foxf1	0.762	http://api.brain-map.org/grid_data/download/73731391?include=energy
Lrrk1	0.762	http://api.brain-map.org/grid_data/download/69169648?include=energy
Igf2bp1	0.762	http://api.brain-map.org/grid_data/download/73811717?include=energy
mCG18431.2	0.762	http://api.brain-map.org/grid_data/download/71210989?include=energy
LOC381854	0.762	http://api.brain-map.org/grid_data/download/70596113?include=energy
Lrrc8d	0.762	http://api.brain-map.org/grid_data/download/385997?include=energy
9430077A04Rik	0.762	http://api.brain-map.org/grid_data/download/70228161?include=energy
Tmem184a	0.762	http://api.brain-map.org/grid_data/download/68410267?include=energy
2610027L16Rik	0.762	http://api.brain-map.org/grid_data/download/583859?include=energy
Stk17b	0.762	http://api.brain-map.org/grid_data/download/79488957?include=energy
Gabra5	0.762	http://api.brain-map.org/grid_data/download/72077453?include=energy
Zc3h7b	0.762	http://api.brain-map.org/grid_data/download/72001863?include=energy
Dbx2	0.761	http://api.brain-map.org/grid_data/download/81657665?include=energy
Larp6	0.761	http://api.brain-map.org/grid_data/download/69443427?include=energy
Ube2b	0.761	http://api.brain-map.org/grid_data/download/74750009?include=energy
Zcchc9	0.761	http://api.brain-map.org/grid_data/download/68077014?include=energy
Zkscan2	0.761	http://api.brain-map.org/grid_data/download/74821605?include=energy
2410006H16Rik	0.761	http://api.brain-map.org/grid_data/download/74635445?include=energy
1700001O22Rik	0.761	http://api.brain-map.org/grid_data/download/70474833?include=energy
Htra3	0.761	http://api.brain-map.org/grid_data/download/69735180?include=energy
Thap7	0.761	http://api.brain-map.org/grid_data/download/69015428?include=energy
B230209E15Rik	0.761	http://api.brain-map.org/grid_data/download/71495873?include=energy
Notch4	0.761	http://api.brain-map.org/grid_data/download/73818802?include=energy
Gm5265	0.761	http://api.brain-map.org/grid_data/download/70725109?include=energy
1810048J11Rik	0.761	http://api.brain-map.org/grid_data/download/69013004?include=energy
Gspt1	0.761	http://api.brain-map.org/grid_data/download/71763786?include=energy
Rlf	0.761	http://api.brain-map.org/grid_data/download/71283574?include=energy
Nlrp5	0.761	http://api.brain-map.org/grid_data/download/68667431?include=energy
Ugt2b38	0.761	http://api.brain-map.org/grid_data/download/68321946?include=energy
Wdr19	0.761	http://api.brain-map.org/grid_data/download/70194988?include=energy
Sh3yl1	0.761	http://api.brain-map.org/grid_data/download/392927?include=energy
Tnxb	0.761	http://api.brain-map.org/grid_data/download/74749957?include=energy
Fancg	0.761	http://api.brain-map.org/grid_data/download/69672288?include=energy
Tbx15	0.761	http://api.brain-map.org/grid_data/download/69290591?include=energy
Col17a1	0.761	http://api.brain-map.org/grid_data/download/73818807?include=energy
Ccdc3	0.761	http://api.brain-map.org/grid_data/download/75651160?include=energy
G630008E18	0.761	http://api.brain-map.org/grid_data/download/70565575?include=energy
Slc5a9	0.761	http://api.brain-map.org/grid_data/download/68445670?include=energy
Bcl7a	0.761	http://api.brain-map.org/grid_data/download/583569?include=energy
Cyp2e1	0.761	http://api.brain-map.org/grid_data/download/68341531?include=energy
Bola1	0.761	http://api.brain-map.org/grid_data/download/71211566?include=energy
Snx2	0.761	http://api.brain-map.org/grid_data/download/68269283?include=energy
Abcc3	0.761	http://api.brain-map.org/grid_data/download/69514606?include=energy
Rem1	0.761	http://api.brain-map.org/grid_data/download/69818078?include=energy
Uncx	0.761	http://api.brain-map.org/grid_data/download/100144532?include=energy
Spata22	0.761	http://api.brain-map.org/grid_data/download/72472486?include=energy
Mm.270304	0.76	http://api.brain-map.org/grid_data/download/71735821?include=energy
Dhdh	0.76	http://api.brain-map.org/grid_data/download/71580231?include=energy
Atad1	0.76	http://api.brain-map.org/grid_data/download/68193071?include=energy
Gm6753	0.76	http://api.brain-map.org/grid_data/download/77340486?include=energy
Lyrm2	0.76	http://api.brain-map.org/grid_data/download/68545557?include=energy
Ttc30a1	0.76	http://api.brain-map.org/grid_data/download/75197684?include=energy
Gm5368	0.76	http://api.brain-map.org/grid_data/download/74822598?include=energy
Polr1b	0.76	http://api.brain-map.org/grid_data/download/71377753?include=energy
Ugt3a2	0.76	http://api.brain-map.org/grid_data/download/71654144?include=energy
6330527O06Rik	0.76	http://api.brain-map.org/grid_data/download/70927827?include=energy
Lingo1	0.76	http://api.brain-map.org/grid_data/download/77620459?include=energy
Gmds	0.76	http://api.brain-map.org/grid_data/download/68631972?include=energy
L3mbtl3	0.76	http://api.brain-map.org/grid_data/download/70295476?include=energy
Snrpa	0.76	http://api.brain-map.org/grid_data/download/68076524?include=energy
Lemd3	0.76	http://api.brain-map.org/grid_data/download/74579422?include=energy
Atp2b4	0.76	http://api.brain-map.org/grid_data/download/74735253?include=energy
Dusp28	0.76	http://api.brain-map.org/grid_data/download/69352808?include=energy
Synpo	0.76	http://api.brain-map.org/grid_data/download/74640881?include=energy
Il11ra2	0.76	http://api.brain-map.org/grid_data/download/75041444?include=energy
Ppapdc2	0.76	http://api.brain-map.org/grid_data/download/73512365?include=energy
Lcmt2	0.76	http://api.brain-map.org/grid_data/download/69736058?include=energy
Crk	0.76	http://api.brain-map.org/grid_data/download/70919232?include=energy
Mnt	0.76	http://api.brain-map.org/grid_data/download/68798897?include=energy
Ctdspl	0.76	http://api.brain-map.org/grid_data/download/70720257?include=energy
Col5a2	0.76	http://api.brain-map.org/grid_data/download/71016666?include=energy
Plcg2	0.76	http://api.brain-map.org/grid_data/download/68160832?include=energy
Mm.59167	0.76	http://api.brain-map.org/grid_data/download/71210382?include=energy
Lrrc29	0.76	http://api.brain-map.org/grid_data/download/69261392?include=energy
Chmp7	0.76	http://api.brain-map.org/grid_data/download/68196908?include=energy
Krt39	0.76	http://api.brain-map.org/grid_data/download/72283635?include=energy
Ankrd26	0.76	http://api.brain-map.org/grid_data/download/70231242?include=energy
4930518I15Rik	0.76	http://api.brain-map.org/grid_data/download/74265135?include=energy
Slc25a22	0.76	http://api.brain-map.org/grid_data/download/1639?include=energy
Ppp1r17	0.759	http://api.brain-map.org/grid_data/download/73817988?include=energy
Rgs12	0.759	http://api.brain-map.org/grid_data/download/70613990?include=energy
Gls	0.759	http://api.brain-map.org/grid_data/download/500?include=energy
Ube2d3	0.759	http://api.brain-map.org/grid_data/download/69015386?include=energy
Fam163b	0.759	http://api.brain-map.org/grid_data/download/73520970?include=energy
Fgf8	0.759	http://api.brain-map.org/grid_data/download/68798561?include=energy
Tnfaip2	0.759	http://api.brain-map.org/grid_data/download/76081039?include=energy
Snai3	0.759	http://api.brain-map.org/grid_data/download/71488795?include=energy
Cdkl2	0.759	http://api.brain-map.org/grid_data/download/74735283?include=energy
Gm815	0.759	http://api.brain-map.org/grid_data/download/72471801?include=energy
0610009L18Rik	0.759	http://api.brain-map.org/grid_data/download/74300825?include=energy
Prokr2	0.759	http://api.brain-map.org/grid_data/download/74511780?include=energy
Map4k3	0.759	http://api.brain-map.org/grid_data/download/657?include=energy
1600012P17Rik	0.759	http://api.brain-map.org/grid_data/download/70200982?include=energy
Tfap2e	0.759	http://api.brain-map.org/grid_data/download/69816972?include=energy
Cdt1	0.759	http://api.brain-map.org/grid_data/download/68797577?include=energy
Sorcs3	0.759	http://api.brain-map.org/grid_data/download/75988635?include=energy
Dclre1a	0.759	http://api.brain-map.org/grid_data/download/71152641?include=energy
Chchd4	0.759	http://api.brain-map.org/grid_data/download/70431235?include=energy
Tex14	0.759	http://api.brain-map.org/grid_data/download/69980292?include=energy
2810417H13Rik	0.759	http://api.brain-map.org/grid_data/download/70429118?include=energy
Gapt	0.759	http://api.brain-map.org/grid_data/download/70305210?include=energy
Slit1	0.759	http://api.brain-map.org/grid_data/download/73788105?include=energy
Tubgcp2	0.759	http://api.brain-map.org/grid_data/download/392915?include=energy
Gm5431	0.759	http://api.brain-map.org/grid_data/download/70725483?include=energy
Lyzl1	0.759	http://api.brain-map.org/grid_data/download/75860919?include=energy
LOC546269	0.759	http://api.brain-map.org/grid_data/download/74736456?include=energy
Kcnv1	0.759	http://api.brain-map.org/grid_data/download/71232860?include=energy
Cobll1	0.759	http://api.brain-map.org/grid_data/download/71021160?include=energy
D530031E19Rik*	0.759	http://api.brain-map.org/grid_data/download/73808414?include=energy
Lztfl1	0.759	http://api.brain-map.org/grid_data/download/532700?include=energy
LOC433640	0.759	http://api.brain-map.org/grid_data/download/71456125?include=energy
Olfr1231	0.759	http://api.brain-map.org/grid_data/download/74720651?include=energy
Mm.373807	0.759	http://api.brain-map.org/grid_data/download/71210085?include=energy
Ell3	0.759	http://api.brain-map.org/grid_data/download/69549164?include=energy
Vps36	0.759	http://api.brain-map.org/grid_data/download/686218?include=energy
Actr6	0.759	http://api.brain-map.org/grid_data/download/69514708?include=energy
Ficd	0.759	http://api.brain-map.org/grid_data/download/75197534?include=energy
Jarid2	0.759	http://api.brain-map.org/grid_data/download/605?include=energy
Cdkn3	0.759	http://api.brain-map.org/grid_data/download/69113955?include=energy
K230010J24Rik	0.759	http://api.brain-map.org/grid_data/download/70611431?include=energy
Siah2	0.759	http://api.brain-map.org/grid_data/download/68843889?include=energy
Zfp110	0.758	http://api.brain-map.org/grid_data/download/71763661?include=energy
C77370	0.758	http://api.brain-map.org/grid_data/download/69531127?include=energy
Ces2b	0.758	http://api.brain-map.org/grid_data/download/68744946?include=energy
Olfr1352	0.758	http://api.brain-map.org/grid_data/download/73635598?include=energy
Papd5	0.758	http://api.brain-map.org/grid_data/download/71147803?include=energy
Pus10	0.758	http://api.brain-map.org/grid_data/download/68161476?include=energy
Daglb	0.758	http://api.brain-map.org/grid_data/download/641520?include=energy
Fam76a	0.758	http://api.brain-map.org/grid_data/download/69549656?include=energy
Gxylt2	0.758	http://api.brain-map.org/grid_data/download/73931405?include=energy
Mrpl52	0.758	http://api.brain-map.org/grid_data/download/71020801?include=energy
Gm11536	0.758	http://api.brain-map.org/grid_data/download/73712900?include=energy
Armc10	0.758	http://api.brain-map.org/grid_data/download/68924769?include=energy
Creb3l2	0.758	http://api.brain-map.org/grid_data/download/68920431?include=energy
Bard1	0.758	http://api.brain-map.org/grid_data/download/75197486?include=energy
Rmnd5b	0.758	http://api.brain-map.org/grid_data/download/638726?include=energy
1810010H24Rik	0.758	http://api.brain-map.org/grid_data/download/69407092?include=energy
Mgst2	0.758	http://api.brain-map.org/grid_data/download/69549189?include=energy
Ofd1	0.758	http://api.brain-map.org/grid_data/download/69782557?include=energy
A730018C14Rik	0.758	http://api.brain-map.org/grid_data/download/69448954?include=energy
Plscr2	0.758	http://api.brain-map.org/grid_data/download/75990837?include=energy
Cd79a	0.758	http://api.brain-map.org/grid_data/download/68744492?include=energy
Cyp24a1	0.758	http://api.brain-map.org/grid_data/download/69608348?include=energy
Vps26a	0.758	http://api.brain-map.org/grid_data/download/68442901?include=energy
Efcab4b	0.758	http://api.brain-map.org/grid_data/download/70299965?include=energy
C430042M11Rik	0.758	http://api.brain-map.org/grid_data/download/73513609?include=energy
LOC433310	0.758	http://api.brain-map.org/grid_data/download/71808686?include=energy
Luc7l3	0.758	http://api.brain-map.org/grid_data/download/69407511?include=energy
H2-DMb1	0.758	http://api.brain-map.org/grid_data/download/68076897?include=energy
AI464131	0.758	http://api.brain-map.org/grid_data/download/69524572?include=energy
Fmnl1	0.758	http://api.brain-map.org/grid_data/download/75041433?include=energy
9430076C15Rik	0.758	http://api.brain-map.org/grid_data/download/70228155?include=energy
E030013D07Rik*	0.758	http://api.brain-map.org/grid_data/download/73834487?include=energy
Proc	0.758	http://api.brain-map.org/grid_data/download/68301383?include=energy
Zfp780b	0.758	http://api.brain-map.org/grid_data/download/71380387?include=energy
Hsd11b2	0.758	http://api.brain-map.org/grid_data/download/69015787?include=energy
Zdhhc24	0.758	http://api.brain-map.org/grid_data/download/69289321?include=energy
Prpf3	0.758	http://api.brain-map.org/grid_data/download/76081905?include=energy

Zfp282	0.758	http://api.brain-map.org/grid_data/download/68162195?include=energy
LOC432809	0.758	http://api.brain-map.org/grid_data/download/74822591?include=energy
Pigg	0.757	http://api.brain-map.org/grid_data/download/70787107?include=energy
Nras	0.757	http://api.brain-map.org/grid_data/download/68745000?include=energy
Entpd8	0.757	http://api.brain-map.org/grid_data/download/77932829?include=energy
6430573F11Rik	0.757	http://api.brain-map.org/grid_data/download/75651150?include=energy
Klhl30	0.757	http://api.brain-map.org/grid_data/download/69407594?include=energy
Arap2	0.757	http://api.brain-map.org/grid_data/download/74881937?include=energy
Fcrls	0.757	http://api.brain-map.org/grid_data/download/1896?include=energy
Myc	0.757	http://api.brain-map.org/grid_data/download/112194721?include=energy
Ocstamp	0.757	http://api.brain-map.org/grid_data/download/587407?include=energy
AI606181	0.757	http://api.brain-map.org/grid_data/download/69526384?include=energy
Ablim3	0.757	http://api.brain-map.org/grid_data/download/73992943?include=energy
Fat1	0.757	http://api.brain-map.org/grid_data/download/71496994?include=energy
Arntl2	0.757	http://api.brain-map.org/grid_data/download/100145442?include=energy
Alpk2	0.757	http://api.brain-map.org/grid_data/download/74988275?include=energy
Aff1	0.757	http://api.brain-map.org/grid_data/download/73870509?include=energy
LOC433156	0.757	http://api.brain-map.org/grid_data/download/70795491?include=energy
Prrx2	0.757	http://api.brain-map.org/grid_data/download/1522?include=energy
Map3k5	0.757	http://api.brain-map.org/grid_data/download/73994720?include=energy
LOC432842	0.757	http://api.brain-map.org/grid_data/download/70611341?include=energy
Il18bp	0.757	http://api.brain-map.org/grid_data/download/68638011?include=energy
Creg2	0.757	http://api.brain-map.org/grid_data/download/74882195?include=energy
Alkbh8	0.757	http://api.brain-map.org/grid_data/download/68797679?include=energy
Metap2	0.757	http://api.brain-map.org/grid_data/download/74277730?include=energy
Dhodh	0.757	http://api.brain-map.org/grid_data/download/587435?include=energy
Mrpl14	0.757	http://api.brain-map.org/grid_data/download/68441622?include=energy
Zfp148	0.757	http://api.brain-map.org/grid_data/download/68546566?include=energy
A830061E14Rik	0.757	http://api.brain-map.org/grid_data/download/69514392?include=energy
Sec22a	0.757	http://api.brain-map.org/grid_data/download/386245?include=energy
Fbxo4	0.756	http://api.brain-map.org/grid_data/download/68191150?include=energy
Dkc1	0.756	http://api.brain-map.org/grid_data/download/70305484?include=energy
Irak3	0.756	http://api.brain-map.org/grid_data/download/70278087?include=energy
Prss22	0.756	http://api.brain-map.org/grid_data/download/69014752?include=energy
LOC434289	0.756	http://api.brain-map.org/grid_data/download/70793839?include=energy
Edem2	0.756	http://api.brain-map.org/grid_data/download/413814?include=energy
Clns1a	0.756	http://api.brain-map.org/grid_data/download/67881553?include=energy
LOC433374	0.756	http://api.brain-map.org/grid_data/download/71808510?include=energy
Tex13a	0.756	http://api.brain-map.org/grid_data/download/69353657?include=energy
Gm364	0.756	http://api.brain-map.org/grid_data/download/74562564?include=energy
Tjp1	0.756	http://api.brain-map.org/grid_data/download/69981013?include=energy
Fcer1a	0.756	http://api.brain-map.org/grid_data/download/69672384?include=energy
Yipf3	0.756	http://api.brain-map.org/grid_data/download/671235?include=energy
Tcte3	0.756	http://api.brain-map.org/grid_data/download/69174499?include=energy
Cpsf4l	0.756	http://api.brain-map.org/grid_data/download/75827109?include=energy
1700026J04Rik	0.756	http://api.brain-map.org/grid_data/download/74357833?include=energy
Acsm1	0.756	http://api.brain-map.org/grid_data/download/68499123?include=energy
Brap	0.756	http://api.brain-map.org/grid_data/download/74822607?include=energy
Kdm6b	0.756	http://api.brain-map.org/grid_data/download/70278111?include=energy
4933406B17Rik	0.756	http://api.brain-map.org/grid_data/download/71892101?include=energy
Mxi1	0.756	http://api.brain-map.org/grid_data/download/74819343?include=energy
Islr2	0.756	http://api.brain-map.org/grid_data/download/142?include=energy
Zfp367	0.756	http://api.brain-map.org/grid_data/download/69352694?include=energy
Olfr186	0.756	http://api.brain-map.org/grid_data/download/73653338?include=energy
Rnf146	0.756	http://api.brain-map.org/grid_data/download/69114198?include=energy
B4galt7	0.756	http://api.brain-map.org/grid_data/download/532708?include=energy
LOC432876	0.756	http://api.brain-map.org/grid_data/download/74514276?include=energy
Zfp609	0.756	http://api.brain-map.org/grid_data/download/69407182?include=energy
LOC383692	0.756	http://api.brain-map.org/grid_data/download/70725115?include=energy
Cdc37	0.756	http://api.brain-map.org/grid_data/download/71496288?include=energy
Kcnk6	0.756	http://api.brain-map.org/grid_data/download/69860425?include=energy
Pkp4	0.755	http://api.brain-map.org/grid_data/download/69134562?include=energy
Fyco1	0.755	http://api.brain-map.org/grid_data/download/69672653?include=energy
Prrg1	0.755	http://api.brain-map.org/grid_data/download/71378075?include=energy
Pla2g2f	0.755	http://api.brain-map.org/grid_data/download/70300822?include=energy
Zfp426	0.755	http://api.brain-map.org/grid_data/download/68161461?include=energy
Rpp21	0.755	http://api.brain-map.org/grid_data/download/68744928?include=energy
Tnfsf4	0.755	http://api.brain-map.org/grid_data/download/69981181?include=energy
Slain2	0.755	http://api.brain-map.org/grid_data/download/70528085?include=energy
Igfbp4	0.755	http://api.brain-map.org/grid_data/download/71924311?include=energy
Cep76	0.755	http://api.brain-map.org/grid_data/download/69444783?include=energy
LOC546222	0.755	http://api.brain-map.org/grid_data/download/74822580?include=energy
Lin54	0.755	http://api.brain-map.org/grid_data/download/69524566?include=energy
LOC433417	0.755	http://api.brain-map.org/grid_data/download/74430912?include=energy
Nvl	0.755	http://api.brain-map.org/grid_data/download/5?include=energy
Tfap4	0.755	http://api.brain-map.org/grid_data/download/69012711?include=energy
Greb1	0.755	http://api.brain-map.org/grid_data/download/71924412?include=energy
Zcchc16	0.755	http://api.brain-map.org/grid_data/download/73906203?include=energy
A130017F12Rik*	0.755	http://api.brain-map.org/grid_data/download/73949520?include=energy
Fsd1l	0.755	http://api.brain-map.org/grid_data/download/69608094?include=energy
Pgf	0.755	http://api.brain-map.org/grid_data/download/77869838?include=energy
Tmc2	0.755	http://api.brain-map.org/grid_data/download/74056142?include=energy
Spinkl	0.755	http://api.brain-map.org/grid_data/download/70228167?include=energy
Farp1	0.755	http://api.brain-map.org/grid_data/download/70919519?include=energy
Zdhhc19	0.755	http://api.brain-map.org/grid_data/download/69114932?include=energy
LOC432658	0.754	http://api.brain-map.org/grid_data/download/70611168?include=energy
Atrip	0.754	http://api.brain-map.org/grid_data/download/70946198?include=energy
Fam71e2	0.754	http://api.brain-map.org/grid_data/download/74821755?include=energy
Gm1586	0.754	http://api.brain-map.org/grid_data/download/70298429?include=energy
Rps19bp1	0.754	http://api.brain-map.org/grid_data/download/69854175?include=energy
LOC435443	0.754	http://api.brain-map.org/grid_data/download/74736279?include=energy
Fam109a	0.754	http://api.brain-map.org/grid_data/download/69448002?include=energy
Ptprr	0.754	http://api.brain-map.org/grid_data/download/74882784?include=energy
Myadm	0.754	http://api.brain-map.org/grid_data/download/74658086?include=energy
Tomm40l	0.754	http://api.brain-map.org/grid_data/download/74359437?include=energy
9830163H01Rik	0.754	http://api.brain-map.org/grid_data/download/70228251?include=energy
Cpne4	0.754	http://api.brain-map.org/grid_data/download/74579932?include=energy
Lama4	0.754	http://api.brain-map.org/grid_data/download/76003553?include=energy
Gramd1b	0.754	http://api.brain-map.org/grid_data/download/71495161?include=energy
0610009O20Rik	0.754	http://api.brain-map.org/grid_data/download/74359443?include=energy
Sox30	0.754	http://api.brain-map.org/grid_data/download/69816906?include=energy
Efcab6	0.754	http://api.brain-map.org/grid_data/download/74819465?include=energy
Fpr1	0.754	http://api.brain-map.org/grid_data/download/70723387?include=energy
Cr2	0.754	http://api.brain-map.org/grid_data/download/74427072?include=energy
Sp3	0.754	http://api.brain-map.org/grid_data/download/79556710?include=energy
Epgn	0.754	http://api.brain-map.org/grid_data/download/71021170?include=energy
Vmn1r50	0.754	http://api.brain-map.org/grid_data/download/70785224?include=energy
Nutf2	0.754	http://api.brain-map.org/grid_data/download/631715?include=energy
E030047D23Rik*	0.754	http://api.brain-map.org/grid_data/download/73906424?include=energy
Mm.204306	0.754	http://api.brain-map.org/grid_data/download/71152336?include=energy
Ube2e2	0.754	http://api.brain-map.org/grid_data/download/70539166?include=energy
Fxyd5	0.754	http://api.brain-map.org/grid_data/download/74819694?include=energy
Abcb7	0.754	http://api.brain-map.org/grid_data/download/78534538?include=energy
Gm104	0.754	http://api.brain-map.org/grid_data/download/70295835?include=energy
Slc19a2	0.754	http://api.brain-map.org/grid_data/download/2585?include=energy
Slc25a45	0.754	http://api.brain-map.org/grid_data/download/68162231?include=energy
1700001K23Rik	0.754	http://api.brain-map.org/grid_data/download/74581247?include=energy
2810007J24Rik	0.754	http://api.brain-map.org/grid_data/download/68666523?include=energy
Cspp1	0.754	http://api.brain-map.org/grid_data/download/70231767?include=energy
Tusc5	0.754	http://api.brain-map.org/grid_data/download/70192147?include=energy
B230398E24Rik*	0.753	http://api.brain-map.org/grid_data/download/74000564?include=energy
Mthfd1l	0.753	http://api.brain-map.org/grid_data/download/678691?include=energy
Ccdc64b	0.753	http://api.brain-map.org/grid_data/download/68191222?include=energy
Tmem117	0.753	http://api.brain-map.org/grid_data/download/70528294?include=energy
Ptpn12	0.753	http://api.brain-map.org/grid_data/download/68798861?include=energy
D230037D09Rik	0.753	http://api.brain-map.org/grid_data/download/69608893?include=energy
R3hdm1	0.753	http://api.brain-map.org/grid_data/download/69133668?include=energy
Mll3	0.753	http://api.brain-map.org/grid_data/download/69289381?include=energy
Otx1	0.753	http://api.brain-map.org/grid_data/download/79360320?include=energy
Erich1	0.753	http://api.brain-map.org/grid_data/download/74430984?include=energy
Pdlim2	0.753	http://api.brain-map.org/grid_data/download/2476?include=energy
Mill1	0.753	http://api.brain-map.org/grid_data/download/70300342?include=energy
Tril	0.753	http://api.brain-map.org/grid_data/download/71380471?include=energy
Map3k10	0.753	http://api.brain-map.org/grid_data/download/71212596?include=energy
Vars2	0.753	http://api.brain-map.org/grid_data/download/68843925?include=energy
2810021B07Rik	0.753	http://api.brain-map.org/grid_data/download/69855305?include=energy
Ss18	0.753	http://api.brain-map.org/grid_data/download/69885762?include=energy
St8sia5	0.753	http://api.brain-map.org/grid_data/download/72081445?include=energy
Fam71e1	0.753	http://api.brain-map.org/grid_data/download/70201331?include=energy
Gpkow	0.753	http://api.brain-map.org/grid_data/download/69816571?include=energy
Zbtb5	0.753	http://api.brain-map.org/grid_data/download/68844582?include=energy
Gzmn	0.753	http://api.brain-map.org/grid_data/download/70724780?include=energy
Trim11	0.753	http://api.brain-map.org/grid_data/download/587427?include=energy
Mmp9	0.753	http://api.brain-map.org/grid_data/download/68745402?include=energy
Adamts17	0.753	http://api.brain-map.org/grid_data/download/71924431?include=energy
Stk10	0.753	http://api.brain-map.org/grid_data/download/71020894?include=energy
Scai	0.753	http://api.brain-map.org/grid_data/download/74581420?include=energy
Greb1l	0.753	http://api.brain-map.org/grid_data/download/70596002?include=energy
Hipk3	0.753	http://api.brain-map.org/grid_data/download/71381052?include=energy
Best2	0.753	http://api.brain-map.org/grid_data/download/1930?include=energy
Tmem221	0.753	http://api.brain-map.org/grid_data/download/70793893?include=energy
Sprr2h	0.753	http://api.brain-map.org/grid_data/download/74743378?include=energy
Tmem100	0.753	http://api.brain-map.org/grid_data/download/68744833?include=energy
Gm5664	0.753	http://api.brain-map.org/grid_data/download/71456135?include=energy
Dsc3	0.752	http://api.brain-map.org/grid_data/download/81655564?include=energy
1110034G24Rik	0.752	http://api.brain-map.org/grid_data/download/70598380?include=energy
Qrfpr	0.752	http://api.brain-map.org/grid_data/download/71717619?include=energy
Gpr114	0.752	http://api.brain-map.org/grid_data/download/74562266?include=energy
D930029H24Rik*	0.752	http://api.brain-map.org/grid_data/download/73834475?include=energy
Nlk	0.752	http://api.brain-map.org/grid_data/download/76085742?include=energy
Zfp236	0.752	http://api.brain-map.org/grid_data/download/69290830?include=energy
Luzp2	0.752	http://api.brain-map.org/grid_data/download/69736334?include=energy
Cyp2d13	0.752	http://api.brain-map.org/grid_data/download/68666475?include=energy
Trhde	0.752	http://api.brain-map.org/grid_data/download/75774669?include=energy
Ren1	0.752	http://api.brain-map.org/grid_data/download/69818084?include=energy
Def6	0.752	http://api.brain-map.org/grid_data/download/69626831?include=energy
LOC434302	0.752	http://api.brain-map.org/grid_data/download/70793857?include=energy
Sit1	0.752	http://api.brain-map.org/grid_data/download/74277716?include=energy
Gm1966	0.752	http://api.brain-map.org/grid_data/download/70725579?include=energy
Gpr44	0.752	http://api.brain-map.org/grid_data/download/69257570?include=energy
1700011M02Rik	0.752	http://api.brain-map.org/grid_data/download/70201241?include=energy
Gbp8	0.752	http://api.brain-map.org/grid_data/download/68443423?include=energy
Ccdc23	0.752	http://api.brain-map.org/grid_data/download/632512?include=energy
Gnai3	0.752	http://api.brain-map.org/grid_data/download/68845723?include=energy
Ppox	0.752	http://api.brain-map.org/grid_data/download/68076525?include=energy
Zfp677	0.752	http://api.brain-map.org/grid_data/download/74822844?include=energy
Gm7723	0.752	http://api.brain-map.org/grid_data/download/80342303?include=energy
Olfr867	0.752	http://api.brain-map.org/grid_data/download/74734979?include=energy
Gadd45b	0.752	http://api.brain-map.org/grid_data/download/70928374?include=energy
Pklr	0.752	http://api.brain-map.org/grid_data/download/69783244?include=energy
9530053A07Rik	0.752	http://api.brain-map.org/grid_data/download/71809139?include=energy
Cbx5	0.752	http://api.brain-map.org/grid_data/download/198?include=energy
LOC432647	0.752	http://api.brain-map.org/grid_data/download/74431558?include=energy
Hat1	0.752	http://api.brain-map.org/grid_data/download/69014734?include=energy
Phip	0.752	http://api.brain-map.org/grid_data/download/69783089?include=energy
Tgfb1i1	0.752	http://api.brain-map.org/grid_data/download/631719?include=energy
Oxct2b	0.752	http://api.brain-map.org/grid_data/download/72182984?include=energy
2410127L17Rik	0.752	http://api.brain-map.org/grid_data/download/68920791?include=energy
LOC434590	0.752	http://api.brain-map.org/grid_data/download/70725603?include=energy
Prkab1	0.752	http://api.brain-map.org/grid_data/download/68076974?include=energy
7420464K22Rik*	0.752	http://api.brain-map.org/grid_data/download/73906143?include=energy
9530085L02Rik	0.752	http://api.brain-map.org/grid_data/download/74821799?include=energy
Faim3	0.751	http://api.brain-map.org/grid_data/download/69407134?include=energy
Armcx3	0.751	http://api.brain-map.org/grid_data/download/564641?include=energy
Rgag4	0.751	http://api.brain-map.org/grid_data/download/69444909?include=energy
Mapk8	0.751	http://api.brain-map.org/grid_data/download/71063715?include=energy
Thap11	0.751	http://api.brain-map.org/grid_data/download/69014800?include=energy
Nat9	0.751	http://api.brain-map.org/grid_data/download/69838760?include=energy
Ephx2	0.751	http://api.brain-map.org/grid_data/download/70813923?include=energy
Zfp560	0.751	http://api.brain-map.org/grid_data/download/71213874?include=energy
Neurl1a	0.751	http://api.brain-map.org/grid_data/download/73769327?include=energy
Poc1a	0.751	http://api.brain-map.org/grid_data/download/68545806?include=energy
BC088983	0.751	http://api.brain-map.org/grid_data/download/71211560?include=energy
Atg14	0.751	http://api.brain-map.org/grid_data/download/69608525?include=energy
Dctd	0.751	http://api.brain-map.org/grid_data/download/68633383?include=energy
Alkbh4	0.751	http://api.brain-map.org/grid_data/download/71492928?include=energy
Cep120	0.751	http://api.brain-map.org/grid_data/download/68797697?include=energy
Gm5785	0.751	http://api.brain-map.org/grid_data/download/75043617?include=energy
3300002P13Rik	0.751	http://api.brain-map.org/grid_data/download/73716626?include=energy
Lrfn2	0.751	http://api.brain-map.org/grid_data/download/75041454?include=energy
Xdh	0.751	http://api.brain-map.org/grid_data/download/70195030?include=energy
Atf1	0.751	http://api.brain-map.org/grid_data/download/71724810?include=energy
Gm13177	0.751	http://api.brain-map.org/grid_data/download/70784540?include=energy
LOC434432	0.751	http://api.brain-map.org/grid_data/download/71022328?include=energy
Cdc20	0.751	http://api.brain-map.org/grid_data/download/67979044?include=energy
Filip1l	0.751	http://api.brain-map.org/grid_data/download/74363347?include=energy
Gopc	0.751	http://api.brain-map.org/grid_data/download/68797513?include=energy
Manbal	0.751	http://api.brain-map.org/grid_data/download/678686?include=energy
Cmya5	0.751	http://api.brain-map.org/grid_data/download/71021159?include=energy
Dio2	0.751	http://api.brain-map.org/grid_data/download/77924476?include=energy
Arsi	0.75	http://api.brain-map.org/grid_data/download/77620267?include=energy
Agmat	0.75	http://api.brain-map.org/grid_data/download/73818841?include=energy
Baz1a	0.75	http://api.brain-map.org/grid_data/download/73635977?include=energy
Atg4c	0.75	http://api.brain-map.org/grid_data/download/70523729?include=energy
2310005G13Rik	0.75	http://api.brain-map.org/grid_data/download/70203477?include=energy
Heyl	0.75	http://api.brain-map.org/grid_data/download/69289093?include=energy
Ctla2a	0.75	http://api.brain-map.org/grid_data/download/68544979?include=energy
Kpna3	0.75	http://api.brain-map.org/grid_data/download/73512563?include=energy
Ip6k3	0.75	http://api.brain-map.org/grid_data/download/70238740?include=energy
Rnf167	0.75	http://api.brain-map.org/grid_data/download/686202?include=energy
Tspan11	0.75	http://api.brain-map.org/grid_data/download/70436731?include=energy
Abca8b	0.75	http://api.brain-map.org/grid_data/download/69514554?include=energy
Herc4	0.75	http://api.brain-map.org/grid_data/download/69028677?include=energy
Mmadhc	0.75	http://api.brain-map.org/grid_data/download/68269064?include=energy
Sesn1	0.75	http://api.brain-map.org/grid_data/download/70231304?include=energy
Odz2	0.75	http://api.brain-map.org/grid_data/download/79591631?include=energy
Oas1b	0.75	http://api.brain-map.org/grid_data/download/68301861?include=energy
Larp4	0.75	http://api.brain-map.org/grid_data/download/70238645?include=energy
Prkag2	0.75	http://api.brain-map.org/grid_data/download/29?include=energy
Olfr728	0.75	http://api.brain-map.org/grid_data/download/74734890?include=energy
Ccdc132	0.75	http://api.brain-map.org/grid_data/download/70474863?include=energy
2610201A13Rik	0.75	http://api.brain-map.org/grid_data/download/71456133?include=energy
Fhl4	0.75	http://api.brain-map.org/grid_data/download/70543052?include=energy
Bpifb9a	0.75	http://api.brain-map.org/grid_data/download/70528091?include=energy
Slc16a2	0.75	http://api.brain-map.org/grid_data/download/73929597?include=energy
Sms	0.75	http://api.brain-map.org/grid_data/download/74819260?include=energy
Pan3	0.75	http://api.brain-map.org/grid_data/download/68745155?include=energy
Epc1	0.75	http://api.brain-map.org/grid_data/download/71213907?include=energy
Adnp2	0.75	http://api.brain-map.org/grid_data/download/68196915?include=energy
Pbx4	0.749	http://api.brain-map.org/grid_data/download/68522517?include=energy
Scube1	0.749	http://api.brain-map.org/grid_data/download/74272894?include=energy
Mars2	0.749	http://api.brain-map.org/grid_data/download/69531103?include=energy
Acvr1	0.749	http://api.brain-map.org/grid_data/download/69514714?include=energy
Mcrs1	0.749	http://api.brain-map.org/grid_data/download/68076881?include=energy
4930509N14Rik*	0.749	http://api.brain-map.org/grid_data/download/73906503?include=energy
1810063B05Rik	0.749	http://api.brain-map.org/grid_data/download/70474887?include=energy
Lrrc57	0.749	http://api.brain-map.org/grid_data/download/68744815?include=energy
5730405G21Rik	0.749	http://api.brain-map.org/grid_data/download/70218938?include=energy
Gp1ba	0.749	http://api.brain-map.org/grid_data/download/71232740?include=energy
Spry4	0.749	http://api.brain-map.org/grid_data/download/69885726?include=energy
Tmem167	0.749	http://api.brain-map.org/grid_data/download/69134444?include=energy
Fbxl6	0.749	http://api.brain-map.org/grid_data/download/68797972?include=energy
Ptcd3	0.749	http://api.brain-map.org/grid_data/download/70609222?include=energy
5330427O13Rik	0.749	http://api.brain-map.org/grid_data/download/74265279?include=energy
Ube2q1	0.749	http://api.brain-map.org/grid_data/download/69133857?include=energy
Zfp57	0.749	http://api.brain-map.org/grid_data/download/72103842?include=energy
Gm10044	0.749	http://api.brain-map.org/grid_data/download/70725001?include=energy

Zc3h14	0.749	http://api.brain-map.org/grid_data/download/76003368?include=energy
Rprd1a	0.749	http://api.brain-map.org/grid_data/download/70794001?include=energy
Suds3	0.749	http://api.brain-map.org/grid_data/download/74635439?include=energy
Mageb3	0.749	http://api.brain-map.org/grid_data/download/71022732?include=energy
Adh7	0.749	http://api.brain-map.org/grid_data/download/68745384?include=energy
Gm13749	0.749	http://api.brain-map.org/grid_data/download/70723086?include=energy
Art4	0.749	http://api.brain-map.org/grid_data/download/68745480?include=energy
Hormad2	0.749	http://api.brain-map.org/grid_data/download/70218271?include=energy
BC022687	0.749	http://api.brain-map.org/grid_data/download/67815962?include=energy
LOC434015	0.749	http://api.brain-map.org/grid_data/download/74562492?include=energy
Tnpo2	0.749	http://api.brain-map.org/grid_data/download/513243?include=energy
Med25	0.749	http://api.brain-map.org/grid_data/download/545109?include=energy
Klri2	0.749	http://api.brain-map.org/grid_data/download/72471707?include=energy
LOC381907	0.749	http://api.brain-map.org/grid_data/download/70596137?include=energy
Sval1	0.749	http://api.brain-map.org/grid_data/download/70785134?include=energy
Tmem160	0.749	http://api.brain-map.org/grid_data/download/74635144?include=energy
Mlf1ip	0.749	http://api.brain-map.org/grid_data/download/70946569?include=energy
LOC272661	0.749	http://api.brain-map.org/grid_data/download/70595799?include=energy
Gm166	0.749	http://api.brain-map.org/grid_data/download/69672934?include=energy
4921524L21Rik	0.748	http://api.brain-map.org/grid_data/download/69113763?include=energy
Alox12e	0.748	http://api.brain-map.org/grid_data/download/68076979?include=energy
Bend5	0.748	http://api.brain-map.org/grid_data/download/74583139?include=energy
Rev3l	0.748	http://api.brain-map.org/grid_data/download/69818090?include=energy
Espnl	0.748	http://api.brain-map.org/grid_data/download/100144939?include=energy
Arfgap3	0.748	http://api.brain-map.org/grid_data/download/68269273?include=energy
Ppm1d	0.748	http://api.brain-map.org/grid_data/download/68080695?include=energy
C630050I24Rik	0.748	http://api.brain-map.org/grid_data/download/74300759?include=energy
Stam	0.748	http://api.brain-map.org/grid_data/download/73931415?include=energy
Gm10499	0.748	http://api.brain-map.org/grid_data/download/72183008?include=energy
Gcat	0.748	http://api.brain-map.org/grid_data/download/68499135?include=energy
Tmem161b	0.748	http://api.brain-map.org/grid_data/download/68667192?include=energy
G930020L02Rik*	0.748	http://api.brain-map.org/grid_data/download/73716788?include=energy
Nfyb	0.748	http://api.brain-map.org/grid_data/download/68546305?include=energy
Pwp2	0.748	http://api.brain-map.org/grid_data/download/112645814?include=energy
Tex10	0.748	http://api.brain-map.org/grid_data/download/80342215?include=energy
Nup50	0.748	http://api.brain-map.org/grid_data/download/68844558?include=energy
Xirp1	0.748	http://api.brain-map.org/grid_data/download/75988362?include=energy
5033430I15Rik	0.748	http://api.brain-map.org/grid_data/download/74265291?include=energy
Pea15b	0.748	http://api.brain-map.org/grid_data/download/73513480?include=energy
Papd7	0.748	http://api.brain-map.org/grid_data/download/69817169?include=energy
Dlx1as	0.748	http://api.brain-map.org/grid_data/download/79906735?include=energy
Tlr8	0.748	http://api.brain-map.org/grid_data/download/70302539?include=energy
Ighm	0.748	http://api.brain-map.org/grid_data/download/72109389?include=energy
Arhgap17	0.748	http://api.brain-map.org/grid_data/download/1413?include=energy
Nle1	0.748	http://api.brain-map.org/grid_data/download/68633008?include=energy
Sct	0.748	http://api.brain-map.org/grid_data/download/74357731?include=energy
Il25	0.748	http://api.brain-map.org/grid_data/download/72283623?include=energy
Tshz3	0.748	http://api.brain-map.org/grid_data/download/73521811?include=energy
Snca	0.748	http://api.brain-map.org/grid_data/download/79908848?include=energy
Cstad	0.748	http://api.brain-map.org/grid_data/download/75990482?include=energy
Rps6kc1	0.748	http://api.brain-map.org/grid_data/download/68918280?include=energy
1810019J16Rik	0.747	http://api.brain-map.org/grid_data/download/633292?include=energy
Cnot6l	0.747	http://api.brain-map.org/grid_data/download/68341699?include=energy
Map3k3	0.747	http://api.brain-map.org/grid_data/download/68157403?include=energy
Gm324	0.747	http://api.brain-map.org/grid_data/download/70295787?include=energy
Cryzl1	0.747	http://api.brain-map.org/grid_data/download/304?include=energy
U2af1l4	0.747	http://api.brain-map.org/grid_data/download/74822898?include=energy
Ipo7	0.747	http://api.brain-map.org/grid_data/download/69735502?include=energy
Net1	0.747	http://api.brain-map.org/grid_data/download/74581396?include=energy
Abtb2	0.747	http://api.brain-map.org/grid_data/download/69013762?include=energy
Kng1	0.747	http://api.brain-map.org/grid_data/download/68637653?include=energy
Srsf4	0.747	http://api.brain-map.org/grid_data/download/641542?include=energy
1810019D21Rik	0.747	http://api.brain-map.org/grid_data/download/70201570?include=energy
Fn3krp	0.747	http://api.brain-map.org/grid_data/download/74819239?include=energy
Sh3tc1	0.747	http://api.brain-map.org/grid_data/download/68844182?include=energy
Taf6l	0.747	http://api.brain-map.org/grid_data/download/70445251?include=energy
Ror1	0.747	http://api.brain-map.org/grid_data/download/69818260?include=energy
Bcl2l14	0.747	http://api.brain-map.org/grid_data/download/68498872?include=energy
B230325K18Rik	0.747	http://api.brain-map.org/grid_data/download/69527830?include=energy
Dedd2	0.747	http://api.brain-map.org/grid_data/download/68162246?include=energy
C730014E05Rik	0.747	http://api.brain-map.org/grid_data/download/71021030?include=energy
Plekhf1	0.747	http://api.brain-map.org/grid_data/download/632484?include=energy
Pcdh19	0.747	http://api.brain-map.org/grid_data/download/81655211?include=energy
Ubxn7	0.747	http://api.brain-map.org/grid_data/download/73513377?include=energy
Zfp133-ps	0.747	http://api.brain-map.org/grid_data/download/73906185?include=energy
Boll	0.747	http://api.brain-map.org/grid_data/download/71891886?include=energy
Dnase1	0.747	http://api.brain-map.org/grid_data/download/74431203?include=energy
Lsr	0.747	http://api.brain-map.org/grid_data/download/513247?include=energy
Cdhr5	0.747	http://api.brain-map.org/grid_data/download/69013660?include=energy
Ehd1	0.747	http://api.brain-map.org/grid_data/download/69671929?include=energy
Snai1	0.747	http://api.brain-map.org/grid_data/download/70439159?include=energy
Fundc1	0.747	http://api.brain-map.org/grid_data/download/512531?include=energy
Lhfpl1	0.747	http://api.brain-map.org/grid_data/download/68797619?include=energy
LOC434313	0.746	http://api.brain-map.org/grid_data/download/70795955?include=energy
Ccdc90b	0.746	http://api.brain-map.org/grid_data/download/70429489?include=energy
Ftmt	0.746	http://api.brain-map.org/grid_data/download/70276831?include=energy
D830036C21Rik	0.746	http://api.brain-map.org/grid_data/download/69626552?include=energy
Zfp830	0.746	http://api.brain-map.org/grid_data/download/100142512?include=energy
1810037I17Rik	0.746	http://api.brain-map.org/grid_data/download/67978735?include=energy
Ccrl2	0.746	http://api.brain-map.org/grid_data/download/68546191?include=energy
6430706K06Rik*	0.746	http://api.brain-map.org/grid_data/download/74300819?include=energy
Cblb	0.746	http://api.brain-map.org/grid_data/download/74988451?include=energy
mCG142183	0.746	http://api.brain-map.org/grid_data/download/71211823?include=energy
Cars2	0.746	http://api.brain-map.org/grid_data/download/71808421?include=energy
Fam83b	0.746	http://api.brain-map.org/grid_data/download/75214948?include=energy
Hif1an	0.746	http://api.brain-map.org/grid_data/download/69734995?include=energy
C730049P21Rik*	0.746	http://api.brain-map.org/grid_data/download/73808402?include=energy
Trim41	0.746	http://api.brain-map.org/grid_data/download/587432?include=energy
LOC433601	0.746	http://api.brain-map.org/grid_data/download/70725549?include=energy
Fam194a	0.746	http://api.brain-map.org/grid_data/download/80471943?include=energy
Klhl20	0.746	http://api.brain-map.org/grid_data/download/68193059?include=energy
Wdr55	0.746	http://api.brain-map.org/grid_data/download/68743889?include=energy
Usp43	0.746	http://api.brain-map.org/grid_data/download/671248?include=energy
Mtr	0.746	http://api.brain-map.org/grid_data/download/74431745?include=energy
4930592C13Rik	0.746	http://api.brain-map.org/grid_data/download/69442422?include=energy
Unc50	0.746	http://api.brain-map.org/grid_data/download/587429?include=energy
Chi3l1	0.746	http://api.brain-map.org/grid_data/download/68443232?include=energy
LOC544913	0.746	http://api.brain-map.org/grid_data/download/72283498?include=energy
Vmn1r13	0.746	http://api.brain-map.org/grid_data/download/70785260?include=energy
Mcm10	0.746	http://api.brain-map.org/grid_data/download/69752119?include=energy
Ikbip	0.745	http://api.brain-map.org/grid_data/download/68545638?include=energy
Gpr143	0.745	http://api.brain-map.org/grid_data/download/69257456?include=energy
6530411M01Rik	0.745	http://api.brain-map.org/grid_data/download/73870569?include=energy
Kcng2	0.745	http://api.brain-map.org/grid_data/download/74431065?include=energy
Gypa	0.745	http://api.brain-map.org/grid_data/download/69734857?include=energy
Rhox4b	0.745	http://api.brain-map.org/grid_data/download/69288932?include=energy
Hnrpdl	0.745	http://api.brain-map.org/grid_data/download/571?include=energy
2410088K16Rik	0.745	http://api.brain-map.org/grid_data/download/71991888?include=energy
Fam98a	0.745	http://api.brain-map.org/grid_data/download/71493093?include=energy
Jazf1	0.745	http://api.brain-map.org/grid_data/download/69113931?include=energy
Scyl2	0.745	http://api.brain-map.org/grid_data/download/68845490?include=energy
Polrmt	0.745	http://api.brain-map.org/grid_data/download/69816697?include=energy
Wfdc15a	0.745	http://api.brain-map.org/grid_data/download/71378655?include=energy
Tacc2	0.745	http://api.brain-map.org/grid_data/download/68191396?include=energy
Olfr270	0.745	http://api.brain-map.org/grid_data/download/74720940?include=energy
Dcun1d4	0.745	http://api.brain-map.org/grid_data/download/74357720?include=energy
Pin4	0.745	http://api.brain-map.org/grid_data/download/69783202?include=energy
9330102E08Rik	0.745	http://api.brain-map.org/grid_data/download/69446613?include=energy
Efnb1	0.745	http://api.brain-map.org/grid_data/download/100142516?include=energy
Cryz	0.745	http://api.brain-map.org/grid_data/download/632491?include=energy
Ndp	0.745	http://api.brain-map.org/grid_data/download/71234544?include=energy
Ear14	0.745	http://api.brain-map.org/grid_data/download/70785012?include=energy
9430024E24Rik	0.745	http://api.brain-map.org/grid_data/download/71892376?include=energy
Bpifa2	0.745	http://api.brain-map.org/grid_data/download/79544890?include=energy
Twist1	0.745	http://api.brain-map.org/grid_data/download/70445281?include=energy
Olfr70	0.745	http://api.brain-map.org/grid_data/download/68745486?include=energy
Gpx2	0.745	http://api.brain-map.org/grid_data/download/74819652?include=energy
Mm.54145	0.745	http://api.brain-map.org/grid_data/download/71210334?include=energy
Angel1	0.745	http://api.brain-map.org/grid_data/download/545126?include=energy
2310039L15Rik	0.745	http://api.brain-map.org/grid_data/download/71991804?include=energy
Tmem231	0.745	http://api.brain-map.org/grid_data/download/70717360?include=energy
Wdr18	0.744	http://api.brain-map.org/grid_data/download/1551?include=energy
Sae1	0.744	http://api.brain-map.org/grid_data/download/70194534?include=energy
4930449I04Rik	0.744	http://api.brain-map.org/grid_data/download/74076216?include=energy
Krt82	0.744	http://api.brain-map.org/grid_data/download/70295452?include=energy
Trmt6	0.744	http://api.brain-map.org/grid_data/download/68667563?include=energy
Il17ra	0.744	http://api.brain-map.org/grid_data/download/73520990?include=energy
Cks1b	0.744	http://api.brain-map.org/grid_data/download/69548976?include=energy
Mtbp	0.744	http://api.brain-map.org/grid_data/download/69837129?include=energy
1700065I17Rik	0.744	http://api.brain-map.org/grid_data/download/71920207?include=energy
Ubiad1	0.744	http://api.brain-map.org/grid_data/download/68341388?include=energy
Pcsk4	0.744	http://api.brain-map.org/grid_data/download/71920492?include=energy
Pcdha10	0.744	http://api.brain-map.org/grid_data/download/1253?include=energy
Gm749	0.744	http://api.brain-map.org/grid_data/download/74578260?include=energy
Akap13	0.744	http://api.brain-map.org/grid_data/download/74581373?include=energy
Ccnb1	0.744	http://api.brain-map.org/grid_data/download/68301825?include=energy
Zfp238	0.744	http://api.brain-map.org/grid_data/download/2741?include=energy
Cldn6	0.744	http://api.brain-map.org/grid_data/download/633269?include=energy
Prss29	0.744	http://api.brain-map.org/grid_data/download/71280970?include=energy
Mterfd3	0.744	http://api.brain-map.org/grid_data/download/70231999?include=energy
3830417A13Rik	0.744	http://api.brain-map.org/grid_data/download/74579509?include=energy
4933437N03Rik	0.744	http://api.brain-map.org/grid_data/download/71892227?include=energy
Lat2	0.744	http://api.brain-map.org/grid_data/download/68269670?include=energy
1700019O17Rik	0.744	http://api.brain-map.org/grid_data/download/71377957?include=energy
Cyp3a25	0.744	http://api.brain-map.org/grid_data/download/71656070?include=energy
Mfap2	0.744	http://api.brain-map.org/grid_data/download/68117990?include=energy
Olfr65	0.744	http://api.brain-map.org/grid_data/download/74734770?include=energy
Mro	0.744	http://api.brain-map.org/grid_data/download/71789849?include=energy
Cdc42ep4	0.744	http://api.brain-map.org/grid_data/download/71723875?include=energy
Rasl12	0.744	http://api.brain-map.org/grid_data/download/69818012?include=energy
4933439F18Rik	0.744	http://api.brain-map.org/grid_data/download/68845454?include=energy
1110059G10Rik	0.744	http://api.brain-map.org/grid_data/download/70445401?include=energy
Hoxd3	0.744	http://api.brain-map.org/grid_data/download/69289183?include=energy
Vmn1r84	0.744	http://api.brain-map.org/grid_data/download/69442254?include=energy
Tmem111	0.744	http://api.brain-map.org/grid_data/download/67978660?include=energy
Mageb5	0.744	http://api.brain-map.org/grid_data/download/70300264?include=energy
Ulbp1	0.744	http://api.brain-map.org/grid_data/download/70228454?include=energy
Kpna7	0.744	http://api.brain-map.org/grid_data/download/74514168?include=energy
9030414G15Rik*	0.744	http://api.brain-map.org/grid_data/download/73808219?include=energy
Rnf128	0.744	http://api.brain-map.org/grid_data/download/1914?include=energy
Lrrc31	0.743	http://api.brain-map.org/grid_data/download/70239015?include=energy
Fbxo27	0.743	http://api.brain-map.org/grid_data/download/69672330?include=energy
Ccdc54	0.743	http://api.brain-map.org/grid_data/download/69114785?include=energy
Igkv4-92	0.743	http://api.brain-map.org/grid_data/download/70725361?include=energy
Slc15a3	0.743	http://api.brain-map.org/grid_data/download/68799016?include=energy
Ascc3	0.743	http://api.brain-map.org/grid_data/download/80471940?include=energy
Gpr101	0.743	http://api.brain-map.org/grid_data/download/79591639?include=energy
TC1558560	0.743	http://api.brain-map.org/grid_data/download/70919244?include=energy
E330037P12Rik*	0.743	http://api.brain-map.org/grid_data/download/74427120?include=energy
Prrc1	0.743	http://api.brain-map.org/grid_data/download/68269048?include=energy
Cnih3	0.743	http://api.brain-map.org/grid_data/download/39?include=energy
Tdrd12	0.743	http://api.brain-map.org/grid_data/download/70203495?include=energy
1700034J04Rik	0.743	http://api.brain-map.org/grid_data/download/74357887?include=energy
LOC223397	0.743	http://api.brain-map.org/grid_data/download/70725007?include=energy
Tmem90b	0.743	http://api.brain-map.org/grid_data/download/73931426?include=energy
Cd5l	0.743	http://api.brain-map.org/grid_data/download/633687?include=energy
Zar1	0.743	http://api.brain-map.org/grid_data/download/70195090?include=energy
Boc	0.743	http://api.brain-map.org/grid_data/download/74819594?include=energy
4833408A19Rik	0.743	http://api.brain-map.org/grid_data/download/69407738?include=energy
Pde11a	0.743	http://api.brain-map.org/grid_data/download/75081007?include=energy
4930433I11Rik	0.743	http://api.brain-map.org/grid_data/download/70218133?include=energy
Elp2	0.743	http://api.brain-map.org/grid_data/download/70919531?include=energy
S1pr4	0.743	http://api.brain-map.org/grid_data/download/69237221?include=energy
Pcif1	0.743	http://api.brain-map.org/grid_data/download/70528647?include=energy
Krt75	0.743	http://api.brain-map.org/grid_data/download/70295470?include=energy
Yeats4	0.743	http://api.brain-map.org/grid_data/download/587423?include=energy
Olfr1426	0.743	http://api.brain-map.org/grid_data/download/68798145?include=energy
Abce1	0.743	http://api.brain-map.org/grid_data/download/512784?include=energy
Cnksr2	0.742	http://api.brain-map.org/grid_data/download/74431564?include=energy
Doc2a	0.742	http://api.brain-map.org/grid_data/download/76135829?include=energy
Zbtb26	0.742	http://api.brain-map.org/grid_data/download/70228543?include=energy
Prkg1	0.742	http://api.brain-map.org/grid_data/download/73521817?include=energy
1200009I06Rik	0.742	http://api.brain-map.org/grid_data/download/71492802?include=energy
Rbak	0.742	http://api.brain-map.org/grid_data/download/70303618?include=energy
Apof	0.742	http://api.brain-map.org/grid_data/download/68321988?include=energy
Thbs2	0.742	http://api.brain-map.org/grid_data/download/73929578?include=energy
LOC433781	0.742	http://api.brain-map.org/grid_data/download/70786815?include=energy
4930420K17Rik	0.742	http://api.brain-map.org/grid_data/download/71210277?include=energy
Tet3	0.742	http://api.brain-map.org/grid_data/download/71574528?include=energy
Dsc2	0.742	http://api.brain-map.org/grid_data/download/75831766?include=energy
Hecw2	0.742	http://api.brain-map.org/grid_data/download/70300529?include=energy
Mbnl3	0.742	http://api.brain-map.org/grid_data/download/69838620?include=energy
Igsf1	0.742	http://api.brain-map.org/grid_data/download/76003550?include=energy
Kcnn2	0.742	http://api.brain-map.org/grid_data/download/77371815?include=energy
Vmn2r107	0.742	http://api.brain-map.org/grid_data/download/70302986?include=energy
Coq3	0.742	http://api.brain-map.org/grid_data/download/69568950?include=energy
Dcaf12l1	0.742	http://api.brain-map.org/grid_data/download/70195006?include=energy
Slc35a2	0.742	http://api.brain-map.org/grid_data/download/2607?include=energy
Plagl1	0.742	http://api.brain-map.org/grid_data/download/71249057?include=energy
Ubox5	0.742	http://api.brain-map.org/grid_data/download/69261416?include=energy
Extl1	0.742	http://api.brain-map.org/grid_data/download/69672186?include=energy
D930048N14Rik	0.742	http://api.brain-map.org/grid_data/download/69626600?include=energy
Serinc2	0.742	http://api.brain-map.org/grid_data/download/75828193?include=energy
Jmjd8	0.742	http://api.brain-map.org/grid_data/download/686213?include=energy
LOC436436	0.742	http://api.brain-map.org/grid_data/download/71022690?include=energy
Tnni3	0.742	http://api.brain-map.org/grid_data/download/1065?include=energy
Zfp334	0.741	http://api.brain-map.org/grid_data/download/68193069?include=energy
Gldn	0.741	http://api.brain-map.org/grid_data/download/70720252?include=energy
Zfp777	0.741	http://api.brain-map.org/grid_data/download/71283483?include=energy
E130001F20Rik*	0.741	http://api.brain-map.org/grid_data/download/73834517?include=energy
F830031D20Rik	0.741	http://api.brain-map.org/grid_data/download/70276795?include=energy
Ubqln3	0.741	http://api.brain-map.org/grid_data/download/70218606?include=energy
Ucp1	0.741	http://api.brain-map.org/grid_data/download/100146053?include=energy
Clstn2	0.741	http://api.brain-map.org/grid_data/download/73636091?include=energy
Agtr1b	0.741	http://api.brain-map.org/grid_data/download/74724627?include=energy
Olfr1367	0.741	http://api.brain-map.org/grid_data/download/74720781?include=energy
Entpd2	0.741	http://api.brain-map.org/grid_data/download/73732144?include=energy
Fam165b	0.741	http://api.brain-map.org/grid_data/download/633288?include=energy
Csf2ra	0.741	http://api.brain-map.org/grid_data/download/74578212?include=energy
Neurod2	0.741	http://api.brain-map.org/grid_data/download/75651126?include=energy
2700050L05Rik	0.741	http://api.brain-map.org/grid_data/download/67764910?include=energy
LOC433727	0.741	http://api.brain-map.org/grid_data/download/74363344?include=energy
Paqr6	0.741	http://api.brain-map.org/grid_data/download/73712816?include=energy
Rnps1	0.741	http://api.brain-map.org/grid_data/download/68076539?include=energy
Cnot8	0.741	http://api.brain-map.org/grid_data/download/513467?include=energy
2310014L17Rik	0.741	http://api.brain-map.org/grid_data/download/69202376?include=energy
9130019O22Rik	0.741	http://api.brain-map.org/grid_data/download/74431697?include=energy
Dok5	0.741	http://api.brain-map.org/grid_data/download/80343394?include=energy
Rasa3	0.741	http://api.brain-map.org/grid_data/download/69817970?include=energy
9030625A04Rik	0.741	http://api.brain-map.org/grid_data/download/70946199?include=energy
Ifit3	0.741	http://api.brain-map.org/grid_data/download/74047911?include=energy
2900060B14Rik	0.741	http://api.brain-map.org/grid_data/download/74300813?include=energy
Wdr41	0.741	http://api.brain-map.org/grid_data/download/70195012?include=energy
Pigk	0.741	http://api.brain-map.org/grid_data/download/74821733?include=energy
LOC436530	0.741	http://api.brain-map.org/grid_data/download/71283406?include=energy
BC016548	0.741	http://api.brain-map.org/grid_data/download/68341633?include=energy
Thyn1	0.741	http://api.brain-map.org/grid_data/download/67765703?include=energy

Zfp46	0.741	http://api.brain-map.org/grid_data/download/68203460?include=energy
Kcnrg	0.741	http://api.brain-map.org/grid_data/download/70593125?include=energy
Homer1	0.741	http://api.brain-map.org/grid_data/download/1748?include=energy
6430510M02Rik	0.741	http://api.brain-map.org/grid_data/download/74431339?include=energy
Mks1	0.741	http://api.brain-map.org/grid_data/download/70595952?include=energy
Dhx29	0.741	http://api.brain-map.org/grid_data/download/70528593?include=energy
Kctd12	0.741	http://api.brain-map.org/grid_data/download/73520993?include=energy
1700104B16Rik	0.741	http://api.brain-map.org/grid_data/download/74359073?include=energy
Vps54	0.741	http://api.brain-map.org/grid_data/download/68410578?include=energy
4930597O21Rik	0.741	http://api.brain-map.org/grid_data/download/74580703?include=energy
Sertad1	0.741	http://api.brain-map.org/grid_data/download/71610896?include=energy
Pop1	0.74	http://api.brain-map.org/grid_data/download/67881768?include=energy
Etd	0.74	http://api.brain-map.org/grid_data/download/69116430?include=energy
Abhd6	0.74	http://api.brain-map.org/grid_data/download/74047923?include=energy
Kif1c	0.74	http://api.brain-map.org/grid_data/download/69735765?include=energy
Cbwd1	0.74	http://api.brain-map.org/grid_data/download/583873?include=energy
Scaper	0.74	http://api.brain-map.org/grid_data/download/74277342?include=energy
Ros1	0.74	http://api.brain-map.org/grid_data/download/69818281?include=energy
Qtrtd1	0.74	http://api.brain-map.org/grid_data/download/71654123?include=energy
Lyrm1	0.74	http://api.brain-map.org/grid_data/download/564754?include=energy
4930521A18Rik	0.74	http://api.brain-map.org/grid_data/download/70218229?include=energy
Ibsp	0.74	http://api.brain-map.org/grid_data/download/70546000?include=energy
Exosc6	0.74	http://api.brain-map.org/grid_data/download/71496988?include=energy
Fam131a	0.74	http://api.brain-map.org/grid_data/download/71493117?include=energy
Gcm2	0.74	http://api.brain-map.org/grid_data/download/69289051?include=energy
Btg2	0.74	http://api.brain-map.org/grid_data/download/72283802?include=energy
Mknk2	0.74	http://api.brain-map.org/grid_data/download/74821702?include=energy
Med22	0.74	http://api.brain-map.org/grid_data/download/71763900?include=energy
Ankrd1	0.74	http://api.brain-map.org/grid_data/download/79360314?include=energy
Ipo11	0.74	http://api.brain-map.org/grid_data/download/68196916?include=energy
Slc39a1	0.74	http://api.brain-map.org/grid_data/download/69134685?include=energy
4933402E13Rik	0.74	http://api.brain-map.org/grid_data/download/70218618?include=energy
Wnt1	0.74	http://api.brain-map.org/grid_data/download/112644522?include=energy
Fuz	0.74	http://api.brain-map.org/grid_data/download/68845520?include=energy
Dlg2	0.74	http://api.brain-map.org/grid_data/download/73817444?include=energy
LOC640526	0.74	http://api.brain-map.org/grid_data/download/74580966?include=energy
Csn2	0.74	http://api.brain-map.org/grid_data/download/77464974?include=energy
Scnn1b	0.74	http://api.brain-map.org/grid_data/download/69288321?include=energy
9330181H08Rik*	0.74	http://api.brain-map.org/grid_data/download/73808426?include=energy
Ptx3	0.74	http://api.brain-map.org/grid_data/download/100146051?include=energy
Evx2	0.74	http://api.brain-map.org/grid_data/download/68918958?include=energy
Mtap9	0.74	http://api.brain-map.org/grid_data/download/70218890?include=energy
Adrb2	0.74	http://api.brain-map.org/grid_data/download/68744522?include=energy
6430503K07Rik	0.74	http://api.brain-map.org/grid_data/download/74265339?include=energy
Mmgt2	0.739	http://api.brain-map.org/grid_data/download/71212024?include=energy
Hspa14	0.739	http://api.brain-map.org/grid_data/download/632494?include=energy
Hmga2	0.739	http://api.brain-map.org/grid_data/download/71393370?include=energy
4933400F03Rik	0.739	http://api.brain-map.org/grid_data/download/73731307?include=energy
Rab39b	0.739	http://api.brain-map.org/grid_data/download/69817841?include=energy
6530401N04Rik	0.739	http://api.brain-map.org/grid_data/download/69445385?include=energy
Acrv1	0.739	http://api.brain-map.org/grid_data/download/69514678?include=energy
LOC277923	0.739	http://api.brain-map.org/grid_data/download/74743453?include=energy
Serpinb9	0.739	http://api.brain-map.org/grid_data/download/70919513?include=energy
Olfr630	0.739	http://api.brain-map.org/grid_data/download/74734752?include=energy
Sp110	0.739	http://api.brain-map.org/grid_data/download/68443250?include=energy
Pou6f1	0.739	http://api.brain-map.org/grid_data/download/74357559?include=energy
Xkr7	0.739	http://api.brain-map.org/grid_data/download/74277332?include=energy
Irf7	0.739	http://api.brain-map.org/grid_data/download/71393448?include=energy
9530008L14Rik	0.739	http://api.brain-map.org/grid_data/download/68445991?include=energy
Tbc1d17	0.739	http://api.brain-map.org/grid_data/download/634454?include=energy
H2-Ke2	0.739	http://api.brain-map.org/grid_data/download/68196924?include=energy
1700011F03Rik	0.739	http://api.brain-map.org/grid_data/download/80474704?include=energy
Pole3	0.739	http://api.brain-map.org/grid_data/download/70813383?include=energy
Sag	0.739	http://api.brain-map.org/grid_data/download/68443375?include=energy
Fbxw7	0.739	http://api.brain-map.org/grid_data/download/72128750?include=energy
Cmbl	0.739	http://api.brain-map.org/grid_data/download/1651?include=energy
9830147P19Rik	0.739	http://api.brain-map.org/grid_data/download/70228239?include=energy
2610109H07Rik	0.739	http://api.brain-map.org/grid_data/download/74272038?include=energy
Spata13	0.739	http://api.brain-map.org/grid_data/download/73997152?include=energy
Kcnj16	0.739	http://api.brain-map.org/grid_data/download/2341?include=energy
Gm19570	0.739	http://api.brain-map.org/grid_data/download/70295763?include=energy
Rpl3l	0.739	http://api.brain-map.org/grid_data/download/70785042?include=energy
Piwil1	0.738	http://api.brain-map.org/grid_data/download/70300804?include=energy
F630102L10Rik*	0.738	http://api.brain-map.org/grid_data/download/75144622?include=energy
Cass4	0.738	http://api.brain-map.org/grid_data/download/71809157?include=energy
4930404A10Rik	0.738	http://api.brain-map.org/grid_data/download/69407905?include=energy
Susd5	0.738	http://api.brain-map.org/grid_data/download/74562200?include=energy
Ly75	0.738	http://api.brain-map.org/grid_data/download/69736346?include=energy
Pcdhgb1	0.738	http://api.brain-map.org/grid_data/download/70784910?include=energy
Tgm6	0.738	http://api.brain-map.org/grid_data/download/74431459?include=energy
9130017K11Rik	0.738	http://api.brain-map.org/grid_data/download/74359467?include=energy
Siglec1	0.738	http://api.brain-map.org/grid_data/download/76081034?include=energy
Zfp654	0.738	http://api.brain-map.org/grid_data/download/70201158?include=energy
Tpm2	0.738	http://api.brain-map.org/grid_data/download/71486976?include=energy
Gxylt1	0.738	http://api.brain-map.org/grid_data/download/74359700?include=energy
Masp1	0.738	http://api.brain-map.org/grid_data/download/73615782?include=energy
4930599N23Rik	0.738	http://api.brain-map.org/grid_data/download/70218450?include=energy
Zc3h6	0.738	http://api.brain-map.org/grid_data/download/70528011?include=energy
Brpf3	0.738	http://api.brain-map.org/grid_data/download/69113769?include=energy
Unc5a	0.738	http://api.brain-map.org/grid_data/download/69262251?include=energy
Zfp286	0.738	http://api.brain-map.org/grid_data/download/68196910?include=energy
Ccdc45	0.738	http://api.brain-map.org/grid_data/download/72182952?include=energy
A430078G23Rik	0.738	http://api.brain-map.org/grid_data/download/74277713?include=energy
Atp4b	0.738	http://api.brain-map.org/grid_data/download/70719009?include=energy
Spag5	0.738	http://api.brain-map.org/grid_data/download/72081516?include=energy
2610528J11Rik	0.738	http://api.brain-map.org/grid_data/download/70439189?include=energy
Cyp26b1	0.738	http://api.brain-map.org/grid_data/download/72081548?include=energy
Cacnb3	0.737	http://api.brain-map.org/grid_data/download/70560340?include=energy
Fxyd4	0.737	http://api.brain-map.org/grid_data/download/70276837?include=energy
Haus3	0.737	http://api.brain-map.org/grid_data/download/68499548?include=energy
Abcg2	0.737	http://api.brain-map.org/grid_data/download/68667878?include=energy
Fam117a	0.737	http://api.brain-map.org/grid_data/download/71656874?include=energy
Fam135a	0.737	http://api.brain-map.org/grid_data/download/70205680?include=energy
Gimap9	0.737	http://api.brain-map.org/grid_data/download/76080955?include=energy
Prdm14	0.737	http://api.brain-map.org/grid_data/download/72472395?include=energy
9130011E15Rik	0.737	http://api.brain-map.org/grid_data/download/68843967?include=energy
Olfr1102	0.737	http://api.brain-map.org/grid_data/download/73635526?include=energy
Paics	0.737	http://api.brain-map.org/grid_data/download/68637611?include=energy
Pcdhga1	0.737	http://api.brain-map.org/grid_data/download/74277411?include=energy
Mmp11	0.737	http://api.brain-map.org/grid_data/download/68667994?include=energy
Serac1	0.737	http://api.brain-map.org/grid_data/download/69873552?include=energy
Gm906	0.737	http://api.brain-map.org/grid_data/download/72472484?include=energy
Mettl22	0.737	http://api.brain-map.org/grid_data/download/68148735?include=energy
D430013K02	0.737	http://api.brain-map.org/grid_data/download/71580192?include=energy
Pdik1l	0.737	http://api.brain-map.org/grid_data/download/68148757?include=energy
Fktn	0.737	http://api.brain-map.org/grid_data/download/70474089?include=energy
Stx19	0.737	http://api.brain-map.org/grid_data/download/68441502?include=energy
Tnnt2	0.737	http://api.brain-map.org/grid_data/download/74580996?include=energy
Olfr368	0.736	http://api.brain-map.org/grid_data/download/74724067?include=energy
Ces3a	0.736	http://api.brain-map.org/grid_data/download/69028623?include=energy
Apol8	0.736	http://api.brain-map.org/grid_data/download/74581422?include=energy
Gm16223	0.736	http://api.brain-map.org/grid_data/download/70787059?include=energy
Porcn	0.736	http://api.brain-map.org/grid_data/download/71763559?include=energy
Vsig4	0.736	http://api.brain-map.org/grid_data/download/68545288?include=energy
Adsl	0.736	http://api.brain-map.org/grid_data/download/641513?include=energy
Tnks2	0.736	http://api.brain-map.org/grid_data/download/69443433?include=energy
Agfg2	0.736	http://api.brain-map.org/grid_data/download/67978673?include=energy
Ambp	0.736	http://api.brain-map.org/grid_data/download/71723857?include=energy
Faah	0.736	http://api.brain-map.org/grid_data/download/513002?include=energy
Xpo5	0.736	http://api.brain-map.org/grid_data/download/69014967?include=energy
Tfpi2	0.736	http://api.brain-map.org/grid_data/download/80514015?include=energy
Accn5	0.736	http://api.brain-map.org/grid_data/download/69236771?include=energy
Mios	0.736	http://api.brain-map.org/grid_data/download/68633146?include=energy
Zfp879	0.736	http://api.brain-map.org/grid_data/download/69288566?include=energy
Mxd1	0.736	http://api.brain-map.org/grid_data/download/69549192?include=energy
9330119M13Rik	0.736	http://api.brain-map.org/grid_data/download/69446619?include=energy
Fbxo38	0.736	http://api.brain-map.org/grid_data/download/71232659?include=energy
B3galt2	0.736	http://api.brain-map.org/grid_data/download/71064081?include=energy
Zc3h7a	0.736	http://api.brain-map.org/grid_data/download/68196950?include=energy
LOC432545	0.736	http://api.brain-map.org/grid_data/download/74736177?include=energy
Gprc5d	0.736	http://api.brain-map.org/grid_data/download/69257689?include=energy
Eif4ebp2	0.736	http://api.brain-map.org/grid_data/download/67978760?include=energy
Wdr53	0.736	http://api.brain-map.org/grid_data/download/68637461?include=energy
LOC435719	0.736	http://api.brain-map.org/grid_data/download/72472393?include=energy
Syngr4	0.736	http://api.brain-map.org/grid_data/download/69114709?include=energy
B230215A15Rik*	0.736	http://api.brain-map.org/grid_data/download/73808355?include=energy
Olfr16	0.736	http://api.brain-map.org/grid_data/download/74720892?include=energy
Dhh	0.736	http://api.brain-map.org/grid_data/download/69835576?include=energy
Scrn3	0.736	http://api.brain-map.org/grid_data/download/71735824?include=energy
Gdf10	0.736	http://api.brain-map.org/grid_data/download/71587709?include=energy
Hivep3	0.736	http://api.brain-map.org/grid_data/download/71393358?include=energy
Pnma2	0.736	http://api.brain-map.org/grid_data/download/69816374?include=energy
5430435G22Rik	0.736	http://api.brain-map.org/grid_data/download/68633357?include=energy
Klf11	0.736	http://api.brain-map.org/grid_data/download/70598362?include=energy
Aldoart2	0.736	http://api.brain-map.org/grid_data/download/70919179?include=energy
D330010C22Rik	0.736	http://api.brain-map.org/grid_data/download/72283575?include=energy
Cd40lg	0.735	http://api.brain-map.org/grid_data/download/69981187?include=energy
Usp42	0.735	http://api.brain-map.org/grid_data/download/74277728?include=energy
Epsti1	0.735	http://api.brain-map.org/grid_data/download/634447?include=energy
Mrpl33	0.735	http://api.brain-map.org/grid_data/download/69116817?include=energy
Dgke	0.735	http://api.brain-map.org/grid_data/download/74431484?include=energy
Mm.372587	0.735	http://api.brain-map.org/grid_data/download/74513959?include=energy
Id2	0.735	http://api.brain-map.org/grid_data/download/71836806?include=energy
Ube2ql1	0.735	http://api.brain-map.org/grid_data/download/71249083?include=energy
Tbrg3	0.735	http://api.brain-map.org/grid_data/download/70302425?include=energy
Appl1	0.735	http://api.brain-map.org/grid_data/download/69407373?include=energy
Rasal2	0.735	http://api.brain-map.org/grid_data/download/71670719?include=energy
Mm.26272	0.735	http://api.brain-map.org/grid_data/download/74640884?include=energy
LOC432686	0.735	http://api.brain-map.org/grid_data/download/70611204?include=energy
Opcml	0.735	http://api.brain-map.org/grid_data/download/73992920?include=energy
Xkr4	0.735	http://api.brain-map.org/grid_data/download/74277322?include=energy
Adcy1	0.735	http://api.brain-map.org/grid_data/download/71212473?include=energy
Cpa5	0.735	http://api.brain-map.org/grid_data/download/76003516?include=energy
Tmem217	0.735	http://api.brain-map.org/grid_data/download/70218762?include=energy
Plxna3	0.735	http://api.brain-map.org/grid_data/download/1403?include=energy
Lmf2	0.735	http://api.brain-map.org/grid_data/download/69524554?include=energy
Il24	0.735	http://api.brain-map.org/grid_data/download/72283629?include=energy
Ptx4	0.735	http://api.brain-map.org/grid_data/download/72081607?include=energy
Iigp1	0.735	http://api.brain-map.org/grid_data/download/70546012?include=energy
N4bp2	0.735	http://api.brain-map.org/grid_data/download/74363354?include=energy
Madcam1	0.735	http://api.brain-map.org/grid_data/download/2389?include=energy
1700013F07Rik	0.734	http://api.brain-map.org/grid_data/download/71211440?include=energy
Il21r	0.734	http://api.brain-map.org/grid_data/download/69028384?include=energy
Faf1	0.734	http://api.brain-map.org/grid_data/download/69672276?include=energy
Crkl	0.734	http://api.brain-map.org/grid_data/download/69608058?include=energy
Mettl1	0.734	http://api.brain-map.org/grid_data/download/71763792?include=energy
Cbln1	0.734	http://api.brain-map.org/grid_data/download/100145395?include=energy
D330046F09Rik	0.734	http://api.brain-map.org/grid_data/download/74276866?include=energy
Frrs1	0.734	http://api.brain-map.org/grid_data/download/2557?include=energy
Mfsd4	0.734	http://api.brain-map.org/grid_data/download/75831775?include=energy
Rai14	0.734	http://api.brain-map.org/grid_data/download/73521806?include=energy
Mup4	0.734	http://api.brain-map.org/grid_data/download/68301969?include=energy
Decr1	0.734	http://api.brain-map.org/grid_data/download/74425550?include=energy
Thra	0.734	http://api.brain-map.org/grid_data/download/79912542?include=energy
Birc5	0.734	http://api.brain-map.org/grid_data/download/633663?include=energy
Aga	0.734	http://api.brain-map.org/grid_data/download/100144170?include=energy
Zbtb46	0.734	http://api.brain-map.org/grid_data/download/73808192?include=energy
Zfp735	0.734	http://api.brain-map.org/grid_data/download/70201247?include=energy
C1qtnf9	0.734	http://api.brain-map.org/grid_data/download/70228041?include=energy
Chsy3	0.734	http://api.brain-map.org/grid_data/download/75985983?include=energy
Ube3b	0.734	http://api.brain-map.org/grid_data/download/67815937?include=energy
Ankar	0.734	http://api.brain-map.org/grid_data/download/69288524?include=energy
Polr2b	0.734	http://api.brain-map.org/grid_data/download/686189?include=energy
Thrsp	0.734	http://api.brain-map.org/grid_data/download/71587845?include=energy
Pdc	0.734	http://api.brain-map.org/grid_data/download/68341549?include=energy
LOC433467	0.734	http://api.brain-map.org/grid_data/download/70723271?include=energy
Syt17	0.734	http://api.brain-map.org/grid_data/download/73616035?include=energy
Tspan5	0.734	http://api.brain-map.org/grid_data/download/74657928?include=energy
Cyb5rl	0.734	http://api.brain-map.org/grid_data/download/68861730?include=energy
Zfp259	0.734	http://api.brain-map.org/grid_data/download/67792180?include=energy
Echdc2	0.734	http://api.brain-map.org/grid_data/download/71836871?include=energy
Cdc42bpb	0.734	http://api.brain-map.org/grid_data/download/71579855?include=energy
Sec13	0.734	http://api.brain-map.org/grid_data/download/68191432?include=energy
Arhgef37	0.734	http://api.brain-map.org/grid_data/download/69443244?include=energy
B230399H06Rik	0.733	http://api.brain-map.org/grid_data/download/69527890?include=energy
Plekhg2	0.733	http://api.brain-map.org/grid_data/download/68862138?include=energy
Mpg	0.733	http://api.brain-map.org/grid_data/download/67765728?include=energy
Ppp1r12b	0.733	http://api.brain-map.org/grid_data/download/76003611?include=energy
Mras	0.733	http://api.brain-map.org/grid_data/download/72119592?include=energy
Csf3	0.733	http://api.brain-map.org/grid_data/download/69608118?include=energy
Gm5146	0.733	http://api.brain-map.org/grid_data/download/74430960?include=energy
Myo9b	0.733	http://api.brain-map.org/grid_data/download/71456109?include=energy
Adam1b	0.733	http://api.brain-map.org/grid_data/download/69514756?include=energy
Mogat2	0.733	http://api.brain-map.org/grid_data/download/68667533?include=energy
Nfil3	0.733	http://api.brain-map.org/grid_data/download/70743904?include=energy
Nfatc2ip	0.733	http://api.brain-map.org/grid_data/download/76003703?include=energy
Fbxw21	0.733	http://api.brain-map.org/grid_data/download/72471790?include=energy
Sv2b	0.733	http://api.brain-map.org/grid_data/download/72104171?include=energy
Tbc1d5	0.733	http://api.brain-map.org/grid_data/download/70565300?include=energy
Tor1b	0.733	http://api.brain-map.org/grid_data/download/70812723?include=energy
Zfp938	0.733	http://api.brain-map.org/grid_data/download/68632094?include=energy
Cd7	0.733	http://api.brain-map.org/grid_data/download/68499308?include=energy
Gpha2	0.733	http://api.brain-map.org/grid_data/download/69734548?include=energy
Phospho1	0.733	http://api.brain-map.org/grid_data/download/71670685?include=energy
BC003965	0.732	http://api.brain-map.org/grid_data/download/513254?include=energy
Ell2	0.732	http://api.brain-map.org/grid_data/download/71213659?include=energy
Htra1	0.732	http://api.brain-map.org/grid_data/download/68321650?include=energy
Fam83e	0.732	http://api.brain-map.org/grid_data/download/73731283?include=energy
Foxs1	0.732	http://api.brain-map.org/grid_data/download/69288980?include=energy
Tas2r110	0.732	http://api.brain-map.org/grid_data/download/70785164?include=energy
Camta2	0.732	http://api.brain-map.org/grid_data/download/69540339?include=energy
Plscr1	0.732	http://api.brain-map.org/grid_data/download/632487?include=energy
LOC436456	0.732	http://api.brain-map.org/grid_data/download/71022696?include=energy
Dlx6as1	0.732	http://api.brain-map.org/grid_data/download/2000?include=energy
Efhb	0.732	http://api.brain-map.org/grid_data/download/70610402?include=energy
Fam123c	0.732	http://api.brain-map.org/grid_data/download/70228149?include=energy
Atp9b	0.732	http://api.brain-map.org/grid_data/download/74272466?include=energy
Gm4825	0.732	http://api.brain-map.org/grid_data/download/74736057?include=energy
Carm1	0.732	http://api.brain-map.org/grid_data/download/686190?include=energy
Gclm	0.732	http://api.brain-map.org/grid_data/download/69672832?include=energy
4833444G19Rik	0.732	http://api.brain-map.org/grid_data/download/70205596?include=energy
Lgi1	0.732	http://api.brain-map.org/grid_data/download/71670484?include=energy
Pcdhb6	0.732	http://api.brain-map.org/grid_data/download/80341087?include=energy
Cfp	0.732	http://api.brain-map.org/grid_data/download/69783023?include=energy
Pik3r1	0.732	http://api.brain-map.org/grid_data/download/74359574?include=energy
Trim62	0.732	http://api.brain-map.org/grid_data/download/69444825?include=energy
Tas2r134	0.732	http://api.brain-map.org/grid_data/download/70785194?include=energy
D0H4S114	0.732	http://api.brain-map.org/grid_data/download/72472773?include=energy
Mm.375159	0.732	http://api.brain-map.org/grid_data/download/71210142?include=energy
Mapkapk3	0.732	http://api.brain-map.org/grid_data/download/69549253?include=energy
E430025E21Rik	0.732	http://api.brain-map.org/grid_data/download/68191114?include=energy
Gm853	0.732	http://api.brain-map.org/grid_data/download/75931517?include=energy
Tmem44	0.732	http://api.brain-map.org/grid_data/download/71152258?include=energy
Rasgef1c	0.732	http://api.brain-map.org/grid_data/download/69817982?include=energy
Dmrtc2	0.732	http://api.brain-map.org/grid_data/download/69816547?include=energy
Csgalnact1	0.732	http://api.brain-map.org/grid_data/download/69407654?include=energy
Wdr13	0.732	http://api.brain-map.org/grid_data/download/70194982?include=energy

Abcc8	0.731	http://api.brain-map.org/grid_data/download/70919388?include=energy
Itga4	0.731	http://api.brain-map.org/grid_data/download/77924520?include=energy
Krt35	0.731	http://api.brain-map.org/grid_data/download/72283677?include=energy
Stx1a	0.731	http://api.brain-map.org/grid_data/download/2645?include=energy
Sh3rf3	0.731	http://api.brain-map.org/grid_data/download/69838316?include=energy
Agrp	0.731	http://api.brain-map.org/grid_data/download/72283799?include=energy
Nek2	0.731	http://api.brain-map.org/grid_data/download/75042263?include=energy
Mcf2l	0.731	http://api.brain-map.org/grid_data/download/71789807?include=energy
Blvrb	0.731	http://api.brain-map.org/grid_data/download/634437?include=energy
Tfr2	0.731	http://api.brain-map.org/grid_data/download/79541439?include=energy
Ctag2	0.731	http://api.brain-map.org/grid_data/download/75831777?include=energy
Adh6b	0.731	http://api.brain-map.org/grid_data/download/70566029?include=energy
Vps8	0.731	http://api.brain-map.org/grid_data/download/68162202?include=energy
Ikzf3	0.731	http://api.brain-map.org/grid_data/download/69817127?include=energy
Dhx58	0.731	http://api.brain-map.org/grid_data/download/71763933?include=energy
Zfp740	0.731	http://api.brain-map.org/grid_data/download/68162215?include=energy
Rbm10	0.731	http://api.brain-map.org/grid_data/download/513249?include=energy
Etv1	0.731	http://api.brain-map.org/grid_data/download/73512203?include=energy
Npnt	0.731	http://api.brain-map.org/grid_data/download/71670677?include=energy
Tmem203	0.731	http://api.brain-map.org/grid_data/download/67815941?include=energy
Zfp408	0.731	http://api.brain-map.org/grid_data/download/72472441?include=energy
Mapk14	0.731	http://api.brain-map.org/grid_data/download/2399?include=energy
B930071L05	0.731	http://api.brain-map.org/grid_data/download/75990477?include=energy
Mm.48719	0.731	http://api.brain-map.org/grid_data/download/71210310?include=energy
Tram2	0.731	http://api.brain-map.org/grid_data/download/68633300?include=energy
Trat1	0.731	http://api.brain-map.org/grid_data/download/71281819?include=energy
Slc2a12	0.731	http://api.brain-map.org/grid_data/download/74581449?include=energy
Sept8	0.731	http://api.brain-map.org/grid_data/download/69549619?include=energy
Bmpr1a	0.731	http://api.brain-map.org/grid_data/download/68845568?include=energy
Mfsd7b	0.731	http://api.brain-map.org/grid_data/download/70430778?include=energy
D6Ertd160e	0.731	http://api.brain-map.org/grid_data/download/70946250?include=energy
Pibf1	0.731	http://api.brain-map.org/grid_data/download/69608531?include=energy
LOC432483	0.731	http://api.brain-map.org/grid_data/download/70608559?include=energy
mCG1048826	0.73	http://api.brain-map.org/grid_data/download/74510254?include=energy
Zswim3	0.73	http://api.brain-map.org/grid_data/download/67792187?include=energy
Mtpn	0.73	http://api.brain-map.org/grid_data/download/2411?include=energy
1190002N15Rik	0.73	http://api.brain-map.org/grid_data/download/77332088?include=energy
GA_x5J8B7TQSKC-412-5	0.73	http://api.brain-map.org/grid_data/download/69014716?include=energy
Kirrel2	0.73	http://api.brain-map.org/grid_data/download/68797506?include=energy
3110082D06Rik	0.73	http://api.brain-map.org/grid_data/download/74581400?include=energy
Dao	0.73	http://api.brain-map.org/grid_data/download/70429220?include=energy
Rspry1	0.73	http://api.brain-map.org/grid_data/download/70528029?include=energy
Csf2	0.73	http://api.brain-map.org/grid_data/download/73512543?include=energy
Nlrp4e	0.73	http://api.brain-map.org/grid_data/download/70784702?include=energy
Atp2c2	0.73	http://api.brain-map.org/grid_data/download/74635162?include=energy
Tinf2	0.73	http://api.brain-map.org/grid_data/download/77414587?include=energy
Six6	0.73	http://api.brain-map.org/grid_data/download/70593357?include=energy
Hoxd1	0.73	http://api.brain-map.org/grid_data/download/69289171?include=energy
Zfp455	0.73	http://api.brain-map.org/grid_data/download/69352712?include=energy
Nppc	0.73	http://api.brain-map.org/grid_data/download/71920453?include=energy
Ptprc	0.73	http://api.brain-map.org/grid_data/download/71378437?include=energy
F7	0.73	http://api.brain-map.org/grid_data/download/69672216?include=energy
Hps4	0.73	http://api.brain-map.org/grid_data/download/68843895?include=energy
Cdh15	0.73	http://api.brain-map.org/grid_data/download/77869780?include=energy
Zc3hav1l	0.73	http://api.brain-map.org/grid_data/download/70231128?include=energy
BC017647	0.73	http://api.brain-map.org/grid_data/download/634467?include=energy
Foxg1	0.73	http://api.brain-map.org/grid_data/download/2230?include=energy
Klk7	0.73	http://api.brain-map.org/grid_data/download/68498914?include=energy
C130071C03Rik	0.73	http://api.brain-map.org/grid_data/download/69530840?include=energy
Sema3g	0.73	http://api.brain-map.org/grid_data/download/69526474?include=energy
Ptges2	0.73	http://api.brain-map.org/grid_data/download/67978412?include=energy
Calhm2	0.73	http://api.brain-map.org/grid_data/download/68445173?include=energy
Adam25	0.73	http://api.brain-map.org/grid_data/download/70228729?include=energy
Mrgprg	0.73	http://api.brain-map.org/grid_data/download/69258166?include=energy
Brsk2	0.73	http://api.brain-map.org/grid_data/download/73513582?include=energy
Nop16	0.73	http://api.brain-map.org/grid_data/download/68301646?include=energy
Psd3	0.73	http://api.brain-map.org/grid_data/download/73636030?include=energy
Lrrc39	0.73	http://api.brain-map.org/grid_data/download/69446899?include=energy
A530064D06Rik	0.729	http://api.brain-map.org/grid_data/download/70228490?include=energy
Klhl3	0.729	http://api.brain-map.org/grid_data/download/71152390?include=energy
Wasf2	0.729	http://api.brain-map.org/grid_data/download/70194958?include=energy
Tmem185b	0.729	http://api.brain-map.org/grid_data/download/68633074?include=energy
Gpn1	0.729	http://api.brain-map.org/grid_data/download/583552?include=energy
Pfkfb4	0.729	http://api.brain-map.org/grid_data/download/69261924?include=energy
LOC434158	0.729	http://api.brain-map.org/grid_data/download/70787525?include=energy
Zbtb37	0.729	http://api.brain-map.org/grid_data/download/70593298?include=energy
Mrpl48	0.729	http://api.brain-map.org/grid_data/download/70919298?include=energy
2300005B03Rik	0.729	http://api.brain-map.org/grid_data/download/70203453?include=energy
Sprr2i	0.729	http://api.brain-map.org/grid_data/download/70785110?include=energy
Ppid	0.729	http://api.brain-map.org/grid_data/download/100145375?include=energy
Kif2b	0.729	http://api.brain-map.org/grid_data/download/70295422?include=energy
Rock2	0.729	http://api.brain-map.org/grid_data/download/75988621?include=energy
Tmem51	0.729	http://api.brain-map.org/grid_data/download/633676?include=energy
Dscam	0.729	http://api.brain-map.org/grid_data/download/71212398?include=energy
Ptgr1	0.729	http://api.brain-map.org/grid_data/download/68269592?include=energy
Gm1716	0.729	http://api.brain-map.org/grid_data/download/70300019?include=energy
Tmem101	0.729	http://api.brain-map.org/grid_data/download/67979017?include=energy
Ccl22	0.729	http://api.brain-map.org/grid_data/download/68301341?include=energy
Hcn1	0.729	http://api.brain-map.org/grid_data/download/77280561?include=energy
Rnf121	0.728	http://api.brain-map.org/grid_data/download/67815556?include=energy
Gmps	0.728	http://api.brain-map.org/grid_data/download/1361?include=energy
Gtpbp6	0.728	http://api.brain-map.org/grid_data/download/68161473?include=energy
Kcnmb4	0.728	http://api.brain-map.org/grid_data/download/72283793?include=energy
9330120H11Rik	0.728	http://api.brain-map.org/grid_data/download/74822584?include=energy
Ctf1	0.728	http://api.brain-map.org/grid_data/download/1695?include=energy
LOC433045	0.728	http://api.brain-map.org/grid_data/download/70611467?include=energy
Hbegf	0.728	http://api.brain-map.org/grid_data/download/73584735?include=energy
Slc30a3	0.728	http://api.brain-map.org/grid_data/download/73788094?include=energy
Creb1	0.728	http://api.brain-map.org/grid_data/download/71022996?include=energy
Msra	0.728	http://api.brain-map.org/grid_data/download/69015536?include=energy
Suv39h2	0.728	http://api.brain-map.org/grid_data/download/1538?include=energy
Gins4	0.728	http://api.brain-map.org/grid_data/download/68076542?include=energy
Tlr13	0.728	http://api.brain-map.org/grid_data/download/70302521?include=energy
Incenp	0.728	http://api.brain-map.org/grid_data/download/71529953?include=energy
4930524B15Rik	0.728	http://api.brain-map.org/grid_data/download/70218235?include=energy
1810013L24Rik	0.728	http://api.brain-map.org/grid_data/download/70201552?include=energy
Vmn2r1	0.728	http://api.brain-map.org/grid_data/download/75991076?include=energy
Gemin6	0.728	http://api.brain-map.org/grid_data/download/71764107?include=energy
3110033I20Rik*	0.727	http://api.brain-map.org/grid_data/download/74359604?include=energy
4933434I20Rik	0.727	http://api.brain-map.org/grid_data/download/71891946?include=energy
2810038L03Rik	0.727	http://api.brain-map.org/grid_data/download/71493081?include=energy
Gm223	0.727	http://api.brain-map.org/grid_data/download/70296020?include=energy
LOC224330	0.727	http://api.brain-map.org/grid_data/download/74431426?include=energy
4922505E12Rik	0.727	http://api.brain-map.org/grid_data/download/70723199?include=energy
Batf2	0.727	http://api.brain-map.org/grid_data/download/70542942?include=energy
LOC432590	0.727	http://api.brain-map.org/grid_data/download/70611072?include=energy
9330199L01Rik*	0.727	http://api.brain-map.org/grid_data/download/73808438?include=energy
Farp2	0.727	http://api.brain-map.org/grid_data/download/68269628?include=energy
Hscb	0.727	http://api.brain-map.org/grid_data/download/69549171?include=energy
LOC436246	0.727	http://api.brain-map.org/grid_data/download/70784606?include=energy
Slc25a38	0.727	http://api.brain-map.org/grid_data/download/393191?include=energy
Mto1	0.727	http://api.brain-map.org/grid_data/download/68844564?include=energy
Rap1b	0.727	http://api.brain-map.org/grid_data/download/74819347?include=energy
Tap2	0.727	http://api.brain-map.org/grid_data/download/68300878?include=energy
Hgf	0.727	http://api.brain-map.org/grid_data/download/558?include=energy
Osgin1	0.727	http://api.brain-map.org/grid_data/download/68410542?include=energy
Crhr1	0.727	http://api.brain-map.org/grid_data/download/297?include=energy
BC049265	0.727	http://api.brain-map.org/grid_data/download/74431077?include=energy
Cyfip1	0.726	http://api.brain-map.org/grid_data/download/74819653?include=energy
Csf2rb2	0.726	http://api.brain-map.org/grid_data/download/73992937?include=energy
Olfr691	0.726	http://api.brain-map.org/grid_data/download/74734866?include=energy
Cldn4	0.726	http://api.brain-map.org/grid_data/download/70719652?include=energy
Lass6	0.726	http://api.brain-map.org/grid_data/download/69014997?include=energy
Plcd3	0.726	http://api.brain-map.org/grid_data/download/77414915?include=energy
Fam188a	0.726	http://api.brain-map.org/grid_data/download/532676?include=energy
Ptpra	0.726	http://api.brain-map.org/grid_data/download/71378408?include=energy
D14Abb1e	0.726	http://api.brain-map.org/grid_data/download/74277636?include=energy
Hoxa1	0.726	http://api.brain-map.org/grid_data/download/71380411?include=energy
Rtn4rl1	0.726	http://api.brain-map.org/grid_data/download/1826?include=energy
Cdcp1	0.726	http://api.brain-map.org/grid_data/download/69169833?include=energy
Zfp7	0.726	http://api.brain-map.org/grid_data/download/386030?include=energy
Fgfr1op2	0.726	http://api.brain-map.org/grid_data/download/70743887?include=energy
Wiz	0.726	http://api.brain-map.org/grid_data/download/68798909?include=energy
Lrrc66	0.726	http://api.brain-map.org/grid_data/download/70528330?include=energy
9330112K21Rik*	0.726	http://api.brain-map.org/grid_data/download/73906179?include=energy
Tatdn3	0.726	http://api.brain-map.org/grid_data/download/71492826?include=energy
Gm7265	0.726	http://api.brain-map.org/grid_data/download/73834529?include=energy
Syt5	0.726	http://api.brain-map.org/grid_data/download/1029?include=energy
Gm1914	0.726	http://api.brain-map.org/grid_data/download/70300067?include=energy
Mmp24	0.726	http://api.brain-map.org/grid_data/download/70946592?include=energy
Ccnyl1	0.726	http://api.brain-map.org/grid_data/download/74562308?include=energy
D16Ertd472e	0.726	http://api.brain-map.org/grid_data/download/318?include=energy
Car10	0.726	http://api.brain-map.org/grid_data/download/71587832?include=energy
4930444A02Rik	0.726	http://api.brain-map.org/grid_data/download/67787334?include=energy
Avl9	0.726	http://api.brain-map.org/grid_data/download/71020965?include=energy
Gtf3c4	0.725	http://api.brain-map.org/grid_data/download/70526066?include=energy
A3galt2	0.725	http://api.brain-map.org/grid_data/download/74562242?include=energy
Unc93a	0.725	http://api.brain-map.org/grid_data/download/74056156?include=energy
St3gal1	0.725	http://api.brain-map.org/grid_data/download/73616023?include=energy
Akap7	0.725	http://api.brain-map.org/grid_data/download/100147502?include=energy
D830014E11Rik	0.725	http://api.brain-map.org/grid_data/download/70238758?include=energy
Wdr90	0.725	http://api.brain-map.org/grid_data/download/68861711?include=energy
4930515G01Rik	0.725	http://api.brain-map.org/grid_data/download/71493222?include=energy
Cry1	0.725	http://api.brain-map.org/grid_data/download/73712882?include=energy
Slc4a1	0.725	http://api.brain-map.org/grid_data/download/68666882?include=energy
Mfsd7a	0.725	http://api.brain-map.org/grid_data/download/69407708?include=energy
Itpka	0.725	http://api.brain-map.org/grid_data/download/79589520?include=energy
Apol10a	0.725	http://api.brain-map.org/grid_data/download/70227974?include=energy
Ralgps2	0.725	http://api.brain-map.org/grid_data/download/68667503?include=energy
Gmip	0.725	http://api.brain-map.org/grid_data/download/68843853?include=energy
Ankrd60	0.725	http://api.brain-map.org/grid_data/download/71919667?include=energy
Tmem132d	0.725	http://api.brain-map.org/grid_data/download/71161066?include=energy
Skint11	0.725	http://api.brain-map.org/grid_data/download/71808502?include=energy
Nptx1	0.725	http://api.brain-map.org/grid_data/download/73520998?include=energy
Nfkbid	0.725	http://api.brain-map.org/grid_data/download/69527746?include=energy
F2rl3	0.725	http://api.brain-map.org/grid_data/download/69256640?include=energy
G6pc	0.725	http://api.brain-map.org/grid_data/download/68341507?include=energy
H2-M1	0.725	http://api.brain-map.org/grid_data/download/70724786?include=energy
Ccs	0.725	http://api.brain-map.org/grid_data/download/68546260?include=energy
Fbxw17	0.725	http://api.brain-map.org/grid_data/download/68743913?include=energy
Ankrd33b	0.725	http://api.brain-map.org/grid_data/download/71232638?include=energy
LOC432476	0.725	http://api.brain-map.org/grid_data/download/70608553?include=energy
Bspry	0.724	http://api.brain-map.org/grid_data/download/632488?include=energy
C630049E18Rik*	0.724	http://api.brain-map.org/grid_data/download/74749260?include=energy
Cntf	0.724	http://api.brain-map.org/grid_data/download/68545669?include=energy
Nxnl2	0.724	http://api.brain-map.org/grid_data/download/70528055?include=energy
Tas2r123	0.724	http://api.brain-map.org/grid_data/download/70785176?include=energy
Serpinb11	0.724	http://api.brain-map.org/grid_data/download/68269538?include=energy
Smok2a	0.724	http://api.brain-map.org/grid_data/download/72002011?include=energy
1700016D06Rik	0.724	http://api.brain-map.org/grid_data/download/69114926?include=energy
Itgb3bp	0.724	http://api.brain-map.org/grid_data/download/69113805?include=energy
Asb5	0.724	http://api.brain-map.org/grid_data/download/69288644?include=energy
Gm3392	0.724	http://api.brain-map.org/grid_data/download/70295685?include=energy
Zfp2	0.724	http://api.brain-map.org/grid_data/download/71609043?include=energy
Gprin1	0.724	http://api.brain-map.org/grid_data/download/75077243?include=energy
Rbm42	0.724	http://api.brain-map.org/grid_data/download/392929?include=energy
Med15	0.724	http://api.brain-map.org/grid_data/download/69816673?include=energy
Ptchd2	0.724	http://api.brain-map.org/grid_data/download/77620810?include=energy
Proz	0.724	http://api.brain-map.org/grid_data/download/678700?include=energy
Alpi	0.724	http://api.brain-map.org/grid_data/download/70201624?include=energy
2410141K09Rik	0.724	http://api.brain-map.org/grid_data/download/71991882?include=energy
Cdc26	0.724	http://api.brain-map.org/grid_data/download/392912?include=energy
Efna3	0.724	http://api.brain-map.org/grid_data/download/381?include=energy
Ttc9b	0.724	http://api.brain-map.org/grid_data/download/71249081?include=energy
LOC432574	0.724	http://api.brain-map.org/grid_data/download/74431718?include=energy
Nkap	0.724	http://api.brain-map.org/grid_data/download/69549265?include=energy
Pitx3	0.724	http://api.brain-map.org/grid_data/download/81790676?include=energy
Hyls1	0.724	http://api.brain-map.org/grid_data/download/74359515?include=energy
7420426K07Rik	0.723	http://api.brain-map.org/grid_data/download/73906131?include=energy
BC051665	0.723	http://api.brain-map.org/grid_data/download/68667348?include=energy
Vmn1r69	0.723	http://api.brain-map.org/grid_data/download/70302974?include=energy
4921517D21Rik	0.723	http://api.brain-map.org/grid_data/download/70205638?include=energy
LOC434102	0.723	http://api.brain-map.org/grid_data/download/70725573?include=energy
Ddit4l	0.723	http://api.brain-map.org/grid_data/download/71836878?include=energy
Tceal5	0.723	http://api.brain-map.org/grid_data/download/70723571?include=energy
Mapkap1	0.723	http://api.brain-map.org/grid_data/download/68301329?include=energy
Eif4enif1	0.723	http://api.brain-map.org/grid_data/download/68148734?include=energy
Olfr152	0.723	http://api.brain-map.org/grid_data/download/74720886?include=energy
Irf4	0.723	http://api.brain-map.org/grid_data/download/69289219?include=energy
Foxc1	0.723	http://api.brain-map.org/grid_data/download/70431319?include=energy
Sprn	0.723	http://api.brain-map.org/grid_data/download/73512367?include=energy
Fam188b2	0.723	http://api.brain-map.org/grid_data/download/69443385?include=energy
Cdh9	0.723	http://api.brain-map.org/grid_data/download/72472764?include=energy
Tal2	0.723	http://api.brain-map.org/grid_data/download/69290585?include=energy
Aldh16a1	0.723	http://api.brain-map.org/grid_data/download/68321880?include=energy
Eid2	0.723	http://api.brain-map.org/grid_data/download/70723381?include=energy
Sh3bp2	0.723	http://api.brain-map.org/grid_data/download/513020?include=energy
Sctr	0.723	http://api.brain-map.org/grid_data/download/71487191?include=energy
Hapln3	0.723	http://api.brain-map.org/grid_data/download/69734911?include=energy
G630016D24Rik	0.723	http://api.brain-map.org/grid_data/download/73731313?include=energy
Zdhhc16	0.723	http://api.brain-map.org/grid_data/download/686216?include=energy
Bnip1	0.723	http://api.brain-map.org/grid_data/download/69133845?include=energy
Brpf1	0.723	http://api.brain-map.org/grid_data/download/68845562?include=energy
Fmr1	0.723	http://api.brain-map.org/grid_data/download/73606703?include=energy
Grb2	0.723	http://api.brain-map.org/grid_data/download/69734584?include=energy
Pold3	0.723	http://api.brain-map.org/grid_data/download/69817145?include=energy
Cryba4	0.722	http://api.brain-map.org/grid_data/download/68919862?include=energy
4930448F12Rik	0.722	http://api.brain-map.org/grid_data/download/71891683?include=energy
Zfp930	0.722	http://api.brain-map.org/grid_data/download/71380399?include=energy
Efna5	0.722	http://api.brain-map.org/grid_data/download/383?include=energy
Rorb	0.722	http://api.brain-map.org/grid_data/download/79556597?include=energy
Ctu1	0.722	http://api.brain-map.org/grid_data/download/70927830?include=energy
Tram1	0.722	http://api.brain-map.org/grid_data/download/513019?include=energy
Akap17b	0.722	http://api.brain-map.org/grid_data/download/69527836?include=energy
Slamf7	0.722	http://api.brain-map.org/grid_data/download/68301945?include=energy
Oog3	0.722	http://api.brain-map.org/grid_data/download/68667491?include=energy
Smarca4	0.722	http://api.brain-map.org/grid_data/download/71488774?include=energy
Gm5533	0.722	http://api.brain-map.org/grid_data/download/74277715?include=energy
Ints7	0.722	http://api.brain-map.org/grid_data/download/68666935?include=energy
Tespa1	0.722	http://api.brain-map.org/grid_data/download/71020964?include=energy
Icam5	0.722	http://api.brain-map.org/grid_data/download/73520989?include=energy
Hs6st3	0.722	http://api.brain-map.org/grid_data/download/73520986?include=energy
8430417E04Rik*	0.721	http://api.brain-map.org/grid_data/download/73927694?include=energy
Tor1a	0.721	http://api.brain-map.org/grid_data/download/686200?include=energy
Rorc	0.721	http://api.brain-map.org/grid_data/download/80525643?include=energy
Mm.145224	0.721	http://api.brain-map.org/grid_data/download/71148166?include=energy
Dusp3	0.721	http://api.brain-map.org/grid_data/download/73512497?include=energy
Aicda	0.721	http://api.brain-map.org/grid_data/download/70230885?include=energy
Gstm7	0.721	http://api.brain-map.org/grid_data/download/71656927?include=energy
Fstl4	0.721	http://api.brain-map.org/grid_data/download/71717187?include=energy
2210011C24Rik	0.721	http://api.brain-map.org/grid_data/download/74635286?include=energy
Kcnt2	0.721	http://api.brain-map.org/grid_data/download/81600588?include=energy
4930402K13Rik	0.721	http://api.brain-map.org/grid_data/download/70218091?include=energy
Masp2	0.721	http://api.brain-map.org/grid_data/download/68301389?include=energy
Bcl2l12	0.721	http://api.brain-map.org/grid_data/download/71064289?include=energy
Ulk2	0.721	http://api.brain-map.org/grid_data/download/70194570?include=energy
C130036L24Rik	0.721	http://api.brain-map.org/grid_data/download/71212671?include=energy

LOC212476	0.721	http://api.brain-map.org/grid_data/download/70724983?include=energy
Cyp4f18	0.721	http://api.brain-map.org/grid_data/download/70445682?include=energy
Hmgcl	0.721	http://api.brain-map.org/grid_data/download/556994?include=energy
L3mbtl2	0.721	http://api.brain-map.org/grid_data/download/67815942?include=energy
Adam7	0.721	http://api.brain-map.org/grid_data/download/76003378?include=energy
Sphkap	0.72	http://api.brain-map.org/grid_data/download/76085749?include=energy
4930526I15Rik	0.72	http://api.brain-map.org/grid_data/download/71213099?include=energy
Zbtb7c	0.72	http://api.brain-map.org/grid_data/download/70528276?include=energy
Gprc6a	0.72	http://api.brain-map.org/grid_data/download/69859864?include=energy
Krt4	0.72	http://api.brain-map.org/grid_data/download/69735956?include=energy
Ggta1	0.72	http://api.brain-map.org/grid_data/download/70545931?include=energy
Fut4	0.72	http://api.brain-map.org/grid_data/download/69672641?include=energy
Rnf139	0.72	http://api.brain-map.org/grid_data/download/68667754?include=energy
Fam107a	0.72	http://api.brain-map.org/grid_data/download/74431465?include=energy
Gmeb2	0.72	http://api.brain-map.org/grid_data/download/69734464?include=energy
Cdon	0.72	http://api.brain-map.org/grid_data/download/69540707?include=energy
C630030K01Rik*	0.72	http://api.brain-map.org/grid_data/download/73808379?include=energy
Tmem178	0.72	http://api.brain-map.org/grid_data/download/73992709?include=energy
Dtwd1	0.72	http://api.brain-map.org/grid_data/download/587420?include=energy
Clec4a2	0.72	http://api.brain-map.org/grid_data/download/80341097?include=energy
Frg1	0.72	http://api.brain-map.org/grid_data/download/631718?include=energy
D330045A20Rik	0.72	http://api.brain-map.org/grid_data/download/70238651?include=energy
F530007B04Rik*	0.72	http://api.brain-map.org/grid_data/download/73767329?include=energy
Dgcr6	0.72	http://api.brain-map.org/grid_data/download/73592535?include=energy
Tepp	0.72	http://api.brain-map.org/grid_data/download/74635048?include=energy
Mm.371905	0.72	http://api.brain-map.org/grid_data/download/71210031?include=energy
Olfr1357	0.72	http://api.brain-map.org/grid_data/download/74720769?include=energy
Mm.321917	0.719	http://api.brain-map.org/grid_data/download/71158888?include=energy
Dnajc16	0.719	http://api.brain-map.org/grid_data/download/870?include=energy
2310007B03Rik	0.719	http://api.brain-map.org/grid_data/download/71991780?include=energy
Zfp385c	0.719	http://api.brain-map.org/grid_data/download/71569220?include=energy
6430531B16Rik	0.719	http://api.brain-map.org/grid_data/download/71493801?include=energy
Fam84a	0.719	http://api.brain-map.org/grid_data/download/70927811?include=energy
Tmed5	0.719	http://api.brain-map.org/grid_data/download/587410?include=energy
Bcl6b	0.719	http://api.brain-map.org/grid_data/download/69288704?include=energy
1700020A23Rik	0.719	http://api.brain-map.org/grid_data/download/71377976?include=energy
Stk16	0.719	http://api.brain-map.org/grid_data/download/68080710?include=energy
BC100451	0.719	http://api.brain-map.org/grid_data/download/73615805?include=energy
Fam189a1	0.719	http://api.brain-map.org/grid_data/download/72340182?include=energy
4930535E06Rik*	0.719	http://api.brain-map.org/grid_data/download/73927565?include=energy
Slc44a5	0.719	http://api.brain-map.org/grid_data/download/74514162?include=energy
Fgf7	0.719	http://api.brain-map.org/grid_data/download/77454720?include=energy
Cmtm8	0.719	http://api.brain-map.org/grid_data/download/68744552?include=energy
Tmprss11bnl	0.719	http://api.brain-map.org/grid_data/download/75931587?include=energy
Fbxl22	0.719	http://api.brain-map.org/grid_data/download/71380465?include=energy
Ubxn4	0.719	http://api.brain-map.org/grid_data/download/641544?include=energy
Rpl7l1	0.719	http://api.brain-map.org/grid_data/download/70919675?include=energy
Il1r2	0.718	http://api.brain-map.org/grid_data/download/68523258?include=energy
Inhbc	0.718	http://api.brain-map.org/grid_data/download/68546542?include=energy
Gm270	0.718	http://api.brain-map.org/grid_data/download/70295751?include=energy
Ang	0.718	http://api.brain-map.org/grid_data/download/70724571?include=energy
Inhba	0.718	http://api.brain-map.org/grid_data/download/70431432?include=energy
Uqcrq	0.718	http://api.brain-map.org/grid_data/download/68161463?include=energy
Gm5636	0.718	http://api.brain-map.org/grid_data/download/71022409?include=energy
Sstr2	0.718	http://api.brain-map.org/grid_data/download/77371821?include=energy
A830010M20Rik	0.718	http://api.brain-map.org/grid_data/download/74277735?include=energy
H2-M2	0.718	http://api.brain-map.org/grid_data/download/70276982?include=energy
Hrh1	0.718	http://api.brain-map.org/grid_data/download/72283792?include=energy
Ehhadh	0.718	http://api.brain-map.org/grid_data/download/393210?include=energy
Fgf4	0.718	http://api.brain-map.org/grid_data/download/70919394?include=energy
Arc	0.718	http://api.brain-map.org/grid_data/download/74273120?include=energy
Csrnp2	0.718	http://api.brain-map.org/grid_data/download/74047914?include=energy
Rpia	0.718	http://api.brain-map.org/grid_data/download/69013200?include=energy
Lhx2	0.718	http://api.brain-map.org/grid_data/download/2375?include=energy
Sipa1l1	0.718	http://api.brain-map.org/grid_data/download/74511986?include=energy
Gm5793	0.718	http://api.brain-map.org/grid_data/download/69113775?include=energy
Shpk	0.718	http://api.brain-map.org/grid_data/download/71380944?include=energy
Lxn	0.718	http://api.brain-map.org/grid_data/download/72340108?include=energy
A630091E08Rik	0.718	http://api.brain-map.org/grid_data/download/72471722?include=energy
Cdrt4	0.718	http://api.brain-map.org/grid_data/download/75851036?include=energy
Clec11a	0.717	http://api.brain-map.org/grid_data/download/112195815?include=energy
Mylip	0.717	http://api.brain-map.org/grid_data/download/77278965?include=energy
Slc10a3	0.717	http://api.brain-map.org/grid_data/download/74047876?include=energy
2510049J12Rik	0.717	http://api.brain-map.org/grid_data/download/72283372?include=energy
Gpd2	0.717	http://api.brain-map.org/grid_data/download/70615598?include=energy
1700101E01Rik	0.717	http://api.brain-map.org/grid_data/download/100144858?include=energy
Slamf9	0.717	http://api.brain-map.org/grid_data/download/68342123?include=energy
Fam129a	0.717	http://api.brain-map.org/grid_data/download/70300541?include=energy
Wnt10a	0.717	http://api.brain-map.org/grid_data/download/81654087?include=energy
Aqp9	0.717	http://api.brain-map.org/grid_data/download/2037?include=energy
Gnptab	0.717	http://api.brain-map.org/grid_data/download/71213831?include=energy
6.33E+19	0.717	http://api.brain-map.org/grid_data/download/70305084?include=energy
4732456N10Rik	0.717	http://api.brain-map.org/grid_data/download/70205560?include=energy
Tbc1d1	0.717	http://api.brain-map.org/grid_data/download/71670698?include=energy
Npy	0.717	http://api.brain-map.org/grid_data/download/717?include=energy
9330155M09Rik	0.717	http://api.brain-map.org/grid_data/download/69446649?include=energy
Gm13889	0.717	http://api.brain-map.org/grid_data/download/77280557?include=energy
Olfr1402	0.717	http://api.brain-map.org/grid_data/download/73653302?include=energy
Otud1	0.717	http://api.brain-map.org/grid_data/download/74822944?include=energy
9030624J02Rik	0.716	http://api.brain-map.org/grid_data/download/68920755?include=energy
A730013G03Rik	0.716	http://api.brain-map.org/grid_data/download/69448942?include=energy
Tbc1d19	0.716	http://api.brain-map.org/grid_data/download/587411?include=energy
A830005F24Rik	0.716	http://api.brain-map.org/grid_data/download/70228597?include=energy
Dnajb7	0.716	http://api.brain-map.org/grid_data/download/69626963?include=energy
Moxd1	0.716	http://api.brain-map.org/grid_data/download/74357562?include=energy
LOC434281	0.716	http://api.brain-map.org/grid_data/download/70787735?include=energy
Slc9a4	0.716	http://api.brain-map.org/grid_data/download/69874072?include=energy
Lclat1	0.716	http://api.brain-map.org/grid_data/download/70295793?include=energy
Peg12	0.716	http://api.brain-map.org/grid_data/download/70784970?include=energy
4930594M17Rik	0.716	http://api.brain-map.org/grid_data/download/72283456?include=energy
Myst4	0.716	http://api.brain-map.org/grid_data/download/69816637?include=energy
Sfr1	0.716	http://api.brain-map.org/grid_data/download/71763717?include=energy
Olah	0.716	http://api.brain-map.org/grid_data/download/68441466?include=energy
Ccl11	0.716	http://api.brain-map.org/grid_data/download/70528461?include=energy
Gsg2	0.716	http://api.brain-map.org/grid_data/download/69734785?include=energy
E330021D16Rik	0.716	http://api.brain-map.org/grid_data/download/70239045?include=energy
Ddhd2	0.716	http://api.brain-map.org/grid_data/download/69626693?include=energy
Limch1	0.716	http://api.brain-map.org/grid_data/download/71249084?include=energy
Myot	0.716	http://api.brain-map.org/grid_data/download/75827110?include=energy
Pth	0.716	http://api.brain-map.org/grid_data/download/74821913?include=energy
2310008H09Rik	0.716	http://api.brain-map.org/grid_data/download/69549645?include=energy
1810055G02Rik	0.716	http://api.brain-map.org/grid_data/download/601188?include=energy
Dhps	0.716	http://api.brain-map.org/grid_data/download/69116516?include=energy
Foxred2	0.715	http://api.brain-map.org/grid_data/download/74363342?include=energy
1700019N12Rik	0.715	http://api.brain-map.org/grid_data/download/73718050?include=energy
Eml6	0.715	http://api.brain-map.org/grid_data/download/68151438?include=energy
Arhgap25	0.715	http://api.brain-map.org/grid_data/download/72128789?include=energy
Tspyl5	0.715	http://api.brain-map.org/grid_data/download/73497080?include=energy
Fxc1	0.715	http://api.brain-map.org/grid_data/download/68269658?include=energy
5730559C18Rik	0.715	http://api.brain-map.org/grid_data/download/72283486?include=energy
Zfp472	0.715	http://api.brain-map.org/grid_data/download/67787337?include=energy
Brip1	0.715	http://api.brain-map.org/grid_data/download/71380926?include=energy
Igkv5-37	0.715	http://api.brain-map.org/grid_data/download/70725371?include=energy
Msgn1	0.715	http://api.brain-map.org/grid_data/download/70300421?include=energy
Akr1c18	0.715	http://api.brain-map.org/grid_data/download/74425542?include=energy
Kcng1	0.715	http://api.brain-map.org/grid_data/download/77340480?include=energy
Hoxa7	0.715	http://api.brain-map.org/grid_data/download/71891539?include=energy
Muc13	0.715	http://api.brain-map.org/grid_data/download/68632709?include=energy
Klhdc1	0.715	http://api.brain-map.org/grid_data/download/69735843?include=energy
Pcdhga5	0.715	http://api.brain-map.org/grid_data/download/72338695?include=energy
LOC432983	0.715	http://api.brain-map.org/grid_data/download/71281931?include=energy
Ncaph	0.715	http://api.brain-map.org/grid_data/download/227754?include=energy
Fbxl8	0.715	http://api.brain-map.org/grid_data/download/68498926?include=energy
Cpn2	0.714	http://api.brain-map.org/grid_data/download/70303743?include=energy
Capn6	0.714	http://api.brain-map.org/grid_data/download/77798989?include=energy
Tnfrsf12a	0.714	http://api.brain-map.org/grid_data/download/2687?include=energy
Crim1	0.714	http://api.brain-map.org/grid_data/download/72283807?include=energy
Cacna1i	0.714	http://api.brain-map.org/grid_data/download/74512007?include=energy
Pex3	0.714	http://api.brain-map.org/grid_data/download/71152419?include=energy
Fam26d	0.714	http://api.brain-map.org/grid_data/download/70205530?include=energy
Ulk1	0.714	http://api.brain-map.org/grid_data/download/68845335?include=energy
Pion	0.714	http://api.brain-map.org/grid_data/download/69448918?include=energy
Spice1	0.714	http://api.brain-map.org/grid_data/download/69608543?include=energy
LOC545810	0.714	http://api.brain-map.org/grid_data/download/71924492?include=energy
E130012A19Rik	0.714	http://api.brain-map.org/grid_data/download/74363349?include=energy
Aste1	0.714	http://api.brain-map.org/grid_data/download/69352828?include=energy
Dlgap1	0.714	http://api.brain-map.org/grid_data/download/71924386?include=energy
Jph1	0.714	http://api.brain-map.org/grid_data/download/69735664?include=energy
Dyrk4	0.714	http://api.brain-map.org/grid_data/download/68797625?include=energy
Nkx2-5	0.714	http://api.brain-map.org/grid_data/download/100147508?include=energy
Micall1	0.714	http://api.brain-map.org/grid_data/download/74076240?include=energy
Glipr1	0.714	http://api.brain-map.org/grid_data/download/74586665?include=energy
Rspo3	0.714	http://api.brain-map.org/grid_data/download/69980352?include=energy
Rps6kb1	0.713	http://api.brain-map.org/grid_data/download/678706?include=energy
D230039L06Rik	0.713	http://api.brain-map.org/grid_data/download/74510110?include=energy
Mrps14	0.713	http://api.brain-map.org/grid_data/download/68924626?include=energy
1700023E05Rik	0.713	http://api.brain-map.org/grid_data/download/69113793?include=energy
Dusp18	0.713	http://api.brain-map.org/grid_data/download/70634129?include=energy
Cdk2ap2	0.713	http://api.brain-map.org/grid_data/download/68498974?include=energy
Atg4d	0.713	http://api.brain-map.org/grid_data/download/67815943?include=energy
Tmem42	0.713	http://api.brain-map.org/grid_data/download/69352776?include=energy
Cwf19l1	0.713	http://api.brain-map.org/grid_data/download/68162242?include=energy
Elk4	0.713	http://api.brain-map.org/grid_data/download/69288938?include=energy
Nradd	0.713	http://api.brain-map.org/grid_data/download/71991917?include=energy
2610044O15Rik	0.713	http://api.brain-map.org/grid_data/download/68160846?include=energy
Calr4	0.713	http://api.brain-map.org/grid_data/download/70218637?include=energy
Glt28d2	0.713	http://api.brain-map.org/grid_data/download/69407720?include=energy
Ntng2	0.713	http://api.brain-map.org/grid_data/download/73615803?include=energy
Isg20l2	0.713	http://api.brain-map.org/grid_data/download/69540242?include=energy
Cxcl10	0.713	http://api.brain-map.org/grid_data/download/68744881?include=energy
Wdr83	0.713	http://api.brain-map.org/grid_data/download/68162271?include=energy
Klk1b16	0.713	http://api.brain-map.org/grid_data/download/73514707?include=energy
1700086L19Rik	0.713	http://api.brain-map.org/grid_data/download/1643?include=energy
Pwp1	0.713	http://api.brain-map.org/grid_data/download/67787339?include=energy
Snord123	0.713	http://api.brain-map.org/grid_data/download/74427061?include=energy
Polr2g	0.713	http://api.brain-map.org/grid_data/download/392911?include=energy
Med24	0.713	http://api.brain-map.org/grid_data/download/2675?include=energy
Ccng1	0.712	http://api.brain-map.org/grid_data/download/324210?include=energy
Gm711	0.712	http://api.brain-map.org/grid_data/download/69114567?include=energy
Mbtps2	0.712	http://api.brain-map.org/grid_data/download/69015631?include=energy
LOC432500	0.712	http://api.brain-map.org/grid_data/download/70608577?include=energy
Mtpap	0.712	http://api.brain-map.org/grid_data/download/69014710?include=energy
Tnfrsf11b	0.712	http://api.brain-map.org/grid_data/download/68666929?include=energy
Ghrhr	0.712	http://api.brain-map.org/grid_data/download/75931533?include=energy
B4galt4	0.712	http://api.brain-map.org/grid_data/download/71487031?include=energy
Atg10	0.712	http://api.brain-map.org/grid_data/download/68117991?include=energy
Slc26a8	0.712	http://api.brain-map.org/grid_data/download/2597?include=energy
Pcdhga4	0.712	http://api.brain-map.org/grid_data/download/70784878?include=energy
Jrk	0.712	http://api.brain-map.org/grid_data/download/68193017?include=energy
LOC433561	0.712	http://api.brain-map.org/grid_data/download/70785786?include=energy
F630113I08Rik*	0.712	http://api.brain-map.org/grid_data/download/73748938?include=energy
Pon3	0.712	http://api.brain-map.org/grid_data/download/74357564?include=energy
Olfr720	0.712	http://api.brain-map.org/grid_data/download/74734884?include=energy
Cables1	0.711	http://api.brain-map.org/grid_data/download/71021063?include=energy
Gns	0.711	http://api.brain-map.org/grid_data/download/69734494?include=energy
Tm4sf4	0.711	http://api.brain-map.org/grid_data/download/68341304?include=energy
A830093I24Rik	0.711	http://api.brain-map.org/grid_data/download/70717982?include=energy
A930038C07Rik	0.711	http://api.brain-map.org/grid_data/download/72080134?include=energy
A730013O20Rik	0.711	http://api.brain-map.org/grid_data/download/73512542?include=energy
Pramel6	0.711	http://api.brain-map.org/grid_data/download/76003577?include=energy
6530418L21Rik	0.711	http://api.brain-map.org/grid_data/download/74819651?include=energy
Mtmr1	0.711	http://api.brain-map.org/grid_data/download/68918928?include=energy
LOC433948	0.711	http://api.brain-map.org/grid_data/download/70787143?include=energy
Pax8	0.711	http://api.brain-map.org/grid_data/download/74990504?include=energy
Hoxd10	0.711	http://api.brain-map.org/grid_data/download/80341133?include=energy
4632433K11Rik	0.711	http://api.brain-map.org/grid_data/download/70593156?include=energy
B430209F14Rik	0.711	http://api.brain-map.org/grid_data/download/71495903?include=energy
Foxo4	0.711	http://api.brain-map.org/grid_data/download/69289399?include=energy
Ceacam2	0.711	http://api.brain-map.org/grid_data/download/68797486?include=energy
1110008E08Rik	0.711	http://api.brain-map.org/grid_data/download/74300855?include=energy
Leo1	0.711	http://api.brain-map.org/grid_data/download/73497603?include=energy
Aqp7	0.711	http://api.brain-map.org/grid_data/download/68632703?include=energy
Col20a1	0.711	http://api.brain-map.org/grid_data/download/71654111?include=energy
Dhx16	0.711	http://api.brain-map.org/grid_data/download/512766?include=energy
Dyrk2	0.711	http://api.brain-map.org/grid_data/download/72081810?include=energy
Mbd1	0.711	http://api.brain-map.org/grid_data/download/69752101?include=energy
Vmn1r70	0.711	http://api.brain-map.org/grid_data/download/74277463?include=energy
Prex1	0.71	http://api.brain-map.org/grid_data/download/73925725?include=energy
Cmtm2a	0.71	http://api.brain-map.org/grid_data/download/75851049?include=energy
Gpn2	0.71	http://api.brain-map.org/grid_data/download/68637927?include=energy
Gm12541	0.71	http://api.brain-map.org/grid_data/download/71569241?include=energy
C230050L11	0.71	http://api.brain-map.org/grid_data/download/70305544?include=energy
Gng13	0.71	http://api.brain-map.org/grid_data/download/73615561?include=energy
Cml2	0.71	http://api.brain-map.org/grid_data/download/68191168?include=energy
Tas2r107	0.71	http://api.brain-map.org/grid_data/download/70785152?include=energy
Ms4a4b	0.71	http://api.brain-map.org/grid_data/download/75828182?include=energy
Cacna2d1	0.71	http://api.brain-map.org/grid_data/download/72119649?include=energy
Egr4	0.71	http://api.brain-map.org/grid_data/download/74800827?include=energy
St6galnac5	0.71	http://api.brain-map.org/grid_data/download/73787946?include=energy
Lrp2	0.71	http://api.brain-map.org/grid_data/download/70723247?include=energy
Cd8a	0.709	http://api.brain-map.org/grid_data/download/70528509?include=energy
Sema3a	0.709	http://api.brain-map.org/grid_data/download/79591337?include=energy
Gm1562	0.709	http://api.brain-map.org/grid_data/download/70611096?include=energy
Dscaml1	0.709	http://api.brain-map.org/grid_data/download/75041575?include=energy
Dusp6	0.709	http://api.brain-map.org/grid_data/download/75650864?include=energy
Pcdh7	0.709	http://api.brain-map.org/grid_data/download/73521003?include=energy
Tbx4	0.709	http://api.brain-map.org/grid_data/download/71381115?include=energy
9030612E09Rik	0.709	http://api.brain-map.org/grid_data/download/71892364?include=energy
Wnt7a	0.709	http://api.brain-map.org/grid_data/download/1936?include=energy
Lgsn	0.709	http://api.brain-map.org/grid_data/download/70276897?include=energy
Mlh1	0.709	http://api.brain-map.org/grid_data/download/576418?include=energy
2310061N02Rik	0.709	http://api.brain-map.org/grid_data/download/100144381?include=energy
LOC434854	0.709	http://api.brain-map.org/grid_data/download/71022433?include=energy
Clca2	0.708	http://api.brain-map.org/grid_data/download/70301278?include=energy
Fbrsl1	0.708	http://api.brain-map.org/grid_data/download/71213042?include=energy
Crhbp	0.708	http://api.brain-map.org/grid_data/download/80516310?include=energy
1700113H08Rik	0.708	http://api.brain-map.org/grid_data/download/70201522?include=energy
Klhl12	0.708	http://api.brain-map.org/grid_data/download/69289303?include=energy
Nup133	0.708	http://api.brain-map.org/grid_data/download/68795418?include=energy
Lrch4	0.708	http://api.brain-map.org/grid_data/download/72283689?include=energy
Smarcad1	0.708	http://api.brain-map.org/grid_data/download/70795724?include=energy
Wbp11	0.708	http://api.brain-map.org/grid_data/download/641536?include=energy
Zfp316	0.708	http://api.brain-map.org/grid_data/download/68843871?include=energy
Gm5250	0.708	http://api.brain-map.org/grid_data/download/74736135?include=energy
4930578I06Rik	0.708	http://api.brain-map.org/grid_data/download/69113961?include=energy
Gpr39	0.708	http://api.brain-map.org/grid_data/download/69859296?include=energy
Ppfia2	0.708	http://api.brain-map.org/grid_data/download/71496504?include=energy
Fam82a2	0.708	http://api.brain-map.org/grid_data/download/71492808?include=energy
Zbtb20	0.708	http://api.brain-map.org/grid_data/download/1143?include=energy
Osbpl10	0.708	http://api.brain-map.org/grid_data/download/71015387?include=energy
Tmem128	0.708	http://api.brain-map.org/grid_data/download/68080707?include=energy
Lipn	0.708	http://api.brain-map.org/grid_data/download/74635337?include=energy
Ptprt	0.708	http://api.brain-map.org/grid_data/download/72008491?include=energy
Lyar	0.707	http://api.brain-map.org/grid_data/download/69736370?include=energy
Daxx	0.707	http://api.brain-map.org/grid_data/download/69626630?include=energy
1700021C14Rik	0.707	http://api.brain-map.org/grid_data/download/74581364?include=energy
Olfr357	0.707	http://api.brain-map.org/grid_data/download/74724043?include=energy

Gm14461	0.707	http://api.brain-map.org/grid_data/download/71569214?include=energy
Hmgxb4	0.707	http://api.brain-map.org/grid_data/download/68798627?include=energy
Lcn12	0.707	http://api.brain-map.org/grid_data/download/70228053?include=energy
Twf2	0.707	http://api.brain-map.org/grid_data/download/73732148?include=energy
LOC277795	0.707	http://api.brain-map.org/grid_data/download/70595811?include=energy
LOC433288	0.707	http://api.brain-map.org/grid_data/download/70723062?include=energy
Ano4	0.707	http://api.brain-map.org/grid_data/download/73497660?include=energy
LOC432762	0.707	http://api.brain-map.org/grid_data/download/71764476?include=energy
Irak1bp1	0.707	http://api.brain-map.org/grid_data/download/74635427?include=energy
Gm7461	0.707	http://api.brain-map.org/grid_data/download/73870551?include=energy
Hsd11b1	0.707	http://api.brain-map.org/grid_data/download/73520987?include=energy
Hoxc8	0.707	http://api.brain-map.org/grid_data/download/72471723?include=energy
Cd40	0.707	http://api.brain-map.org/grid_data/download/68442889?include=energy
C130052G03Rik	0.707	http://api.brain-map.org/grid_data/download/69530822?include=energy
Raver2	0.707	http://api.brain-map.org/grid_data/download/72128776?include=energy
Fdxacb1	0.706	http://api.brain-map.org/grid_data/download/70596173?include=energy
Apol9b	0.706	http://api.brain-map.org/grid_data/download/69014907?include=energy
Srfbp1	0.706	http://api.brain-map.org/grid_data/download/1653?include=energy
Thbs3	0.706	http://api.brain-map.org/grid_data/download/74657923?include=energy
4833442J19Rik	0.706	http://api.brain-map.org/grid_data/download/73712846?include=energy
Gtf2h5	0.706	http://api.brain-map.org/grid_data/download/69261414?include=energy
Arhgap39	0.706	http://api.brain-map.org/grid_data/download/73992939?include=energy
Gm694	0.706	http://api.brain-map.org/grid_data/download/70298250?include=energy
Rasal1	0.706	http://api.brain-map.org/grid_data/download/75774111?include=energy
Fadd	0.706	http://api.brain-map.org/grid_data/download/68191366?include=energy
Yap1	0.706	http://api.brain-map.org/grid_data/download/68546464?include=energy
Lrrtm4	0.706	http://api.brain-map.org/grid_data/download/70437762?include=energy
Arhgap31	0.706	http://api.brain-map.org/grid_data/download/73512366?include=energy
Slc22a2	0.706	http://api.brain-map.org/grid_data/download/78534502?include=energy
LOC546889	0.706	http://api.brain-map.org/grid_data/download/74736492?include=energy
Apobec4	0.706	http://api.brain-map.org/grid_data/download/70218822?include=energy
Usp46	0.706	http://api.brain-map.org/grid_data/download/70805955?include=energy
Krt31	0.706	http://api.brain-map.org/grid_data/download/70724876?include=energy
Large	0.706	http://api.brain-map.org/grid_data/download/71670483?include=energy
4930578C19Rik	0.706	http://api.brain-map.org/grid_data/download/69442371?include=energy
Galnt7	0.706	http://api.brain-map.org/grid_data/download/70276849?include=energy
Ift20	0.705	http://api.brain-map.org/grid_data/download/73712822?include=energy
Trim30a	0.705	http://api.brain-map.org/grid_data/download/67936391?include=energy
D930020B18Rik	0.705	http://api.brain-map.org/grid_data/download/70238806?include=energy
Rhof	0.705	http://api.brain-map.org/grid_data/download/72000164?include=energy
LOC436367	0.705	http://api.brain-map.org/grid_data/download/71283370?include=energy
A130084B18Rik*	0.705	http://api.brain-map.org/grid_data/download/73949560?include=energy
Tac2	0.705	http://api.brain-map.org/grid_data/download/72339556?include=energy
Ptpn18	0.705	http://api.brain-map.org/grid_data/download/2509?include=energy
Rab17	0.705	http://api.brain-map.org/grid_data/download/68269556?include=energy
Fam107b	0.705	http://api.brain-map.org/grid_data/download/71015800?include=energy
Ing5	0.705	http://api.brain-map.org/grid_data/download/68844409?include=energy
Zfp933	0.705	http://api.brain-map.org/grid_data/download/74058871?include=energy
A730011L01Rik	0.705	http://api.brain-map.org/grid_data/download/68843859?include=energy
4933405L10Rik	0.705	http://api.brain-map.org/grid_data/download/70218649?include=energy
F930106M21Rik*	0.705	http://api.brain-map.org/grid_data/download/73808147?include=energy
Nlrp9a	0.705	http://api.brain-map.org/grid_data/download/70784690?include=energy
Srf	0.705	http://api.brain-map.org/grid_data/download/68798849?include=energy
Rbbp4	0.705	http://api.brain-map.org/grid_data/download/69818024?include=energy
Supt7l	0.705	http://api.brain-map.org/grid_data/download/68795460?include=energy
Zbtb40	0.705	http://api.brain-map.org/grid_data/download/68843835?include=energy
Pcdha12	0.705	http://api.brain-map.org/grid_data/download/70784744?include=energy
Mical2	0.704	http://api.brain-map.org/grid_data/download/71249740?include=energy
A230085B16Rik	0.704	http://api.brain-map.org/grid_data/download/74821557?include=energy
Fam36a	0.704	http://api.brain-map.org/grid_data/download/71456150?include=energy
Galt	0.704	http://api.brain-map.org/grid_data/download/513017?include=energy
Col7a1	0.704	http://api.brain-map.org/grid_data/download/70720250?include=energy
AY702103	0.704	http://api.brain-map.org/grid_data/download/73513492?include=energy
Pole2	0.704	http://api.brain-map.org/grid_data/download/68798465?include=energy
Vamp2	0.704	http://api.brain-map.org/grid_data/download/1092?include=energy
Slc23a3	0.704	http://api.brain-map.org/grid_data/download/72001969?include=energy
Adamts5	0.704	http://api.brain-map.org/grid_data/download/77931973?include=energy
Aadac	0.704	http://api.brain-map.org/grid_data/download/68341932?include=energy
Sip1	0.704	http://api.brain-map.org/grid_data/download/69549611?include=energy
Dapk2	0.704	http://api.brain-map.org/grid_data/download/68301180?include=energy
Kif20b	0.704	http://api.brain-map.org/grid_data/download/71020775?include=energy
Nt5c1a	0.704	http://api.brain-map.org/grid_data/download/70295900?include=energy
4930466O16Rik*	0.704	http://api.brain-map.org/grid_data/download/73927558?include=energy
Usp1	0.704	http://api.brain-map.org/grid_data/download/68633324?include=energy
Esm1	0.703	http://api.brain-map.org/grid_data/download/69080066?include=energy
1300014I06Rik	0.703	http://api.brain-map.org/grid_data/download/68191312?include=energy
Slc26a6	0.703	http://api.brain-map.org/grid_data/download/68637826?include=energy
Chit1	0.703	http://api.brain-map.org/grid_data/download/74635349?include=energy
Utp14a	0.703	http://api.brain-map.org/grid_data/download/70302962?include=energy
Dytn	0.703	http://api.brain-map.org/grid_data/download/74431230?include=energy
Gm5095	0.703	http://api.brain-map.org/grid_data/download/71569202?include=energy
Ngp	0.703	http://api.brain-map.org/grid_data/download/73997145?include=energy
LOC380720	0.703	http://api.brain-map.org/grid_data/download/73931422?include=energy
R3hdml	0.703	http://api.brain-map.org/grid_data/download/100144126?include=energy
Ramp1	0.703	http://api.brain-map.org/grid_data/download/1912?include=energy
Enc1	0.703	http://api.brain-map.org/grid_data/download/390?include=energy
Zdhhc13	0.703	http://api.brain-map.org/grid_data/download/68745215?include=energy
Pkn2	0.703	http://api.brain-map.org/grid_data/download/68798915?include=energy
Spns2	0.703	http://api.brain-map.org/grid_data/download/73512500?include=energy
B3galt6	0.703	http://api.brain-map.org/grid_data/download/69527908?include=energy
Dem1	0.702	http://api.brain-map.org/grid_data/download/68151346?include=energy
Sh3bp5	0.702	http://api.brain-map.org/grid_data/download/68797739?include=energy
Slit2	0.702	http://api.brain-map.org/grid_data/download/79591683?include=energy
0610008F07Rik	0.702	http://api.brain-map.org/grid_data/download/68441532?include=energy
Chrm3	0.702	http://api.brain-map.org/grid_data/download/2095?include=energy
Snx11	0.702	http://api.brain-map.org/grid_data/download/68666571?include=energy
Osgin2	0.702	http://api.brain-map.org/grid_data/download/70445610?include=energy
E130119H09Rik	0.702	http://api.brain-map.org/grid_data/download/70238985?include=energy
Mtfr1	0.702	http://api.brain-map.org/grid_data/download/587437?include=energy
Gm4763	0.702	http://api.brain-map.org/grid_data/download/68546254?include=energy
2900092D14Rik	0.702	http://api.brain-map.org/grid_data/download/71212199?include=energy
Ccnt1	0.702	http://api.brain-map.org/grid_data/download/69540531?include=energy
Rtf1	0.702	http://api.brain-map.org/grid_data/download/71210983?include=energy
Tg	0.702	http://api.brain-map.org/grid_data/download/69980334?include=energy
Ceacam19	0.702	http://api.brain-map.org/grid_data/download/70231851?include=energy
Actn4	0.702	http://api.brain-map.org/grid_data/download/68301533?include=energy
Tnk1	0.702	http://api.brain-map.org/grid_data/download/68637850?include=energy
Nup43	0.702	http://api.brain-map.org/grid_data/download/68301527?include=energy
1700129C05Rik	0.702	http://api.brain-map.org/grid_data/download/69114986?include=energy
Rpusd3	0.702	http://api.brain-map.org/grid_data/download/69526360?include=energy
Gramd4	0.702	http://api.brain-map.org/grid_data/download/69528553?include=energy
C230057M02Rik	0.701	http://api.brain-map.org/grid_data/download/70231905?include=energy
LOC433697	0.701	http://api.brain-map.org/grid_data/download/70786667?include=energy
Acr	0.701	http://api.brain-map.org/grid_data/download/71495209?include=energy
Mmp20	0.701	http://api.brain-map.org/grid_data/download/70300397?include=energy
Prss12	0.701	http://api.brain-map.org/grid_data/download/71836879?include=energy
Krtap8-1	0.701	http://api.brain-map.org/grid_data/download/73927583?include=energy
Vopp1	0.701	http://api.brain-map.org/grid_data/download/70928373?include=energy
Nlrp4g	0.701	http://api.brain-map.org/grid_data/download/70725013?include=energy
LOC385069	0.701	http://api.brain-map.org/grid_data/download/70608403?include=energy
Cdh26	0.701	http://api.brain-map.org/grid_data/download/68797509?include=energy
Lce3f	0.701	http://api.brain-map.org/grid_data/download/71656726?include=energy
Osr1	0.701	http://api.brain-map.org/grid_data/download/70546317?include=energy
Tnfaip8l3	0.701	http://api.brain-map.org/grid_data/download/76097700?include=energy
2810408A11Rik	0.701	http://api.brain-map.org/grid_data/download/71493099?include=energy
Pdp2	0.701	http://api.brain-map.org/grid_data/download/70299983?include=energy
Slc12a6	0.701	http://api.brain-map.org/grid_data/download/71210901?include=energy
Slc36a3	0.701	http://api.brain-map.org/grid_data/download/70539119?include=energy
Cul7	0.701	http://api.brain-map.org/grid_data/download/67814205?include=energy
1200016B10Rik	0.7	http://api.brain-map.org/grid_data/download/69014416?include=energy
Dimt1	0.7	http://api.brain-map.org/grid_data/download/72283318?include=energy
Pcdh8	0.7	http://api.brain-map.org/grid_data/download/74750019?include=energy
Slc46a3	0.7	http://api.brain-map.org/grid_data/download/386255?include=energy
2810006K23Rik	0.7	http://api.brain-map.org/grid_data/download/69012867?include=energy
Dpm3	0.7	http://api.brain-map.org/grid_data/download/68546107?include=energy
Cyp3a13	0.7	http://api.brain-map.org/grid_data/download/70724649?include=energy
Taar6	0.7	http://api.brain-map.org/grid_data/download/70724989?include=energy
Tcf4	0.7	http://api.brain-map.org/grid_data/download/1044?include=energy
Emg1	0.7	http://api.brain-map.org/grid_data/download/631713?include=energy
4930568D16Rik	0.7	http://api.brain-map.org/grid_data/download/71892029?include=energy
4932442L08Rik	0.7	http://api.brain-map.org/grid_data/download/70218582?include=energy
Nit1	0.7	http://api.brain-map.org/grid_data/download/71723997?include=energy
Ankrd17	0.7	http://api.brain-map.org/grid_data/download/71148136?include=energy
1110008P14Rik	0.7	http://api.brain-map.org/grid_data/download/71015816?include=energy
Gse1	0.7	http://api.brain-map.org/grid_data/download/70276975?include=energy
Vmn1r177	0.699	http://api.brain-map.org/grid_data/download/70785279?include=energy
Zdhhc14	0.699	http://api.brain-map.org/grid_data/download/71209886?include=energy
Foxk2	0.699	http://api.brain-map.org/grid_data/download/71283434?include=energy
LOC432429	0.699	http://api.brain-map.org/grid_data/download/70608496?include=energy
Zfp583	0.699	http://api.brain-map.org/grid_data/download/71380393?include=energy
Slc5a11	0.699	http://api.brain-map.org/grid_data/download/68666613?include=energy
Bcl2a1d	0.699	http://api.brain-map.org/grid_data/download/77904713?include=energy
Nrcam	0.699	http://api.brain-map.org/grid_data/download/172?include=energy
Slc27a5	0.699	http://api.brain-map.org/grid_data/download/68203458?include=energy
Myct1	0.699	http://api.brain-map.org/grid_data/download/70300451?include=energy
Flrt2	0.699	http://api.brain-map.org/grid_data/download/73931417?include=energy
Sprr2j-ps	0.699	http://api.brain-map.org/grid_data/download/70785116?include=energy
Sfrp4	0.699	http://api.brain-map.org/grid_data/download/77465014?include=energy
Cdh12	0.699	http://api.brain-map.org/grid_data/download/73513627?include=energy
Ptpru	0.699	http://api.brain-map.org/grid_data/download/858?include=energy
Evi5	0.699	http://api.brain-map.org/grid_data/download/71670743?include=energy
Aplf	0.699	http://api.brain-map.org/grid_data/download/631707?include=energy
mCG113116.1	0.699	http://api.brain-map.org/grid_data/download/71022978?include=energy
Banp	0.699	http://api.brain-map.org/grid_data/download/68196962?include=energy
9930013L23Rik	0.699	http://api.brain-map.org/grid_data/download/72128775?include=energy
Gpr176	0.699	http://api.brain-map.org/grid_data/download/74511881?include=energy
Airn	0.698	http://api.brain-map.org/grid_data/download/71991929?include=energy
Srp9	0.698	http://api.brain-map.org/grid_data/download/68861736?include=energy
Kdm4d	0.698	http://api.brain-map.org/grid_data/download/70305018?include=energy
Fabp9	0.698	http://api.brain-map.org/grid_data/download/69114453?include=energy
Cacnb4	0.698	http://api.brain-map.org/grid_data/download/1679?include=energy
Defa-rs1	0.698	http://api.brain-map.org/grid_data/download/71991708?include=energy
Smyd1	0.698	http://api.brain-map.org/grid_data/download/71488780?include=energy
Ythdf2	0.698	http://api.brain-map.org/grid_data/download/68300851?include=energy
LOC434324	0.698	http://api.brain-map.org/grid_data/download/70793887?include=energy
Amica1	0.698	http://api.brain-map.org/grid_data/download/68667324?include=energy
Ddn	0.698	http://api.brain-map.org/grid_data/download/73636031?include=energy
Cct6b	0.698	http://api.brain-map.org/grid_data/download/74510266?include=energy
Clcc1	0.698	http://api.brain-map.org/grid_data/download/633659?include=energy
LOC383319	0.698	http://api.brain-map.org/grid_data/download/70596234?include=energy
Duxbl	0.697	http://api.brain-map.org/grid_data/download/68844516?include=energy
Hcfc2	0.697	http://api.brain-map.org/grid_data/download/69407074?include=energy
Rbm39	0.697	http://api.brain-map.org/grid_data/download/71325420?include=energy
2610301G19Rik	0.697	http://api.brain-map.org/grid_data/download/68193063?include=energy
Cinp	0.697	http://api.brain-map.org/grid_data/download/583875?include=energy
Smad3	0.697	http://api.brain-map.org/grid_data/download/70593360?include=energy
Adrb1	0.697	http://api.brain-map.org/grid_data/download/77340494?include=energy
Ms4a10	0.697	http://api.brain-map.org/grid_data/download/74635247?include=energy
Slc24a4	0.697	http://api.brain-map.org/grid_data/download/72001982?include=energy
Pabpc5	0.697	http://api.brain-map.org/grid_data/download/69782640?include=energy
A130028J20Rik	0.696	http://api.brain-map.org/grid_data/download/70298166?include=energy
Sipa1l3	0.696	http://api.brain-map.org/grid_data/download/74822585?include=energy
Abpz	0.696	http://api.brain-map.org/grid_data/download/70724601?include=energy
Mcts2	0.696	http://api.brain-map.org/grid_data/download/68545632?include=energy
Bpifa1	0.696	http://api.brain-map.org/grid_data/download/68797978?include=energy
Osgepl1	0.696	http://api.brain-map.org/grid_data/download/69028689?include=energy
Grid2	0.696	http://api.brain-map.org/grid_data/download/71247617?include=energy
Mkx	0.696	http://api.brain-map.org/grid_data/download/69288554?include=energy
Prpf39	0.696	http://api.brain-map.org/grid_data/download/74427050?include=energy
C530043K16Rik	0.696	http://api.brain-map.org/grid_data/download/71021026?include=energy
Fmo1	0.696	http://api.brain-map.org/grid_data/download/72008121?include=energy
Bambi	0.696	http://api.brain-map.org/grid_data/download/68410333?include=energy
Cypt3	0.695	http://api.brain-map.org/grid_data/download/80474702?include=energy
Krt42	0.695	http://api.brain-map.org/grid_data/download/74635514?include=energy
Sall3	0.695	http://api.brain-map.org/grid_data/download/74363352?include=energy
Pcsk5	0.695	http://api.brain-map.org/grid_data/download/70616019?include=energy
Wdr45l	0.695	http://api.brain-map.org/grid_data/download/68269265?include=energy
Mrpl30	0.695	http://api.brain-map.org/grid_data/download/68191324?include=energy
Zfp185	0.695	http://api.brain-map.org/grid_data/download/70785750?include=energy
Ints6	0.695	http://api.brain-map.org/grid_data/download/68844486?include=energy
Ext1	0.695	http://api.brain-map.org/grid_data/download/633664?include=energy
Mab21l3	0.695	http://api.brain-map.org/grid_data/download/68795420?include=energy
Gm7073	0.695	http://api.brain-map.org/grid_data/download/73906101?include=energy
Gdf5	0.695	http://api.brain-map.org/grid_data/download/81654111?include=energy
Nkain2	0.695	http://api.brain-map.org/grid_data/download/73513326?include=energy
2310067E19Rik	0.694	http://api.brain-map.org/grid_data/download/70218733?include=energy
D8Ertd82e	0.694	http://api.brain-map.org/grid_data/download/73931630?include=energy
2610507I01Rik	0.694	http://api.brain-map.org/grid_data/download/74265273?include=energy
Rab27a	0.694	http://api.brain-map.org/grid_data/download/68076980?include=energy
Rybp	0.694	http://api.brain-map.org/grid_data/download/68843847?include=energy
Tnip3	0.694	http://api.brain-map.org/grid_data/download/70227992?include=energy
Mtm1	0.694	http://api.brain-map.org/grid_data/download/71570738?include=energy
Nudcd2	0.694	http://api.brain-map.org/grid_data/download/68300815?include=energy
Cabp1	0.694	http://api.brain-map.org/grid_data/download/74514333?include=energy
Ctsk	0.694	http://api.brain-map.org/grid_data/download/68745293?include=energy
LOC433455	0.694	http://api.brain-map.org/grid_data/download/70723253?include=energy
Mm.202219	0.694	http://api.brain-map.org/grid_data/download/71152324?include=energy
Kdsr	0.694	http://api.brain-map.org/grid_data/download/68158091?include=energy
C130079K02Rik*	0.694	http://api.brain-map.org/grid_data/download/73811633?include=energy
4.93E+23	0.694	http://api.brain-map.org/grid_data/download/72005999?include=energy
S100a4	0.694	http://api.brain-map.org/grid_data/download/69873373?include=energy
Prss57	0.694	http://api.brain-map.org/grid_data/download/69817384?include=energy
Nampt	0.694	http://api.brain-map.org/grid_data/download/68191390?include=energy
F930115I02Rik*	0.694	http://api.brain-map.org/grid_data/download/73808123?include=energy
Zdhhc21	0.694	http://api.brain-map.org/grid_data/download/69817103?include=energy
Mdfi	0.693	http://api.brain-map.org/grid_data/download/112648831?include=energy
Armcx5	0.693	http://api.brain-map.org/grid_data/download/74743495?include=energy
Ccdc68	0.693	http://api.brain-map.org/grid_data/download/69012771?include=energy
Dgkd	0.693	http://api.brain-map.org/grid_data/download/70723104?include=energy
Tmem171	0.693	http://api.brain-map.org/grid_data/download/70298411?include=energy
Sp6	0.693	http://api.brain-map.org/grid_data/download/69816912?include=energy
Wbscr16	0.693	http://api.brain-map.org/grid_data/download/67815552?include=energy
Lhpp	0.693	http://api.brain-map.org/grid_data/download/72340126?include=energy
Vmn1r226	0.693	http://api.brain-map.org/grid_data/download/70785321?include=energy
Gm10619	0.693	http://api.brain-map.org/grid_data/download/70305030?include=energy
4930588J15Rik	0.693	http://api.brain-map.org/grid_data/download/74265189?include=energy
1700007K09Rik	0.693	http://api.brain-map.org/grid_data/download/70609147?include=energy
Vps4b	0.693	http://api.brain-map.org/grid_data/download/513252?include=energy
Gm5546	0.693	http://api.brain-map.org/grid_data/download/70786598?include=energy
Zfp619	0.693	http://api.brain-map.org/grid_data/download/70203597?include=energy
Lgr5	0.693	http://api.brain-map.org/grid_data/download/72283791?include=energy
BC048679	0.693	http://api.brain-map.org/grid_data/download/70295602?include=energy
Serp2	0.693	http://api.brain-map.org/grid_data/download/70303821?include=energy
LOC386484	0.693	http://api.brain-map.org/grid_data/download/70795712?include=energy
Pglyrp3	0.693	http://api.brain-map.org/grid_data/download/100144092?include=energy
LOC432984	0.693	http://api.brain-map.org/grid_data/download/71282566?include=energy
4930432K09Rik	0.693	http://api.brain-map.org/grid_data/download/72283426?include=energy
4930449E01Rik	0.692	http://api.brain-map.org/grid_data/download/71891695?include=energy
Gdpd4	0.692	http://api.brain-map.org/grid_data/download/72472488?include=energy
Zfp946	0.692	http://api.brain-map.org/grid_data/download/68498998?include=energy
C230013L11Rik	0.692	http://api.brain-map.org/grid_data/download/69530882?include=energy
Rab35	0.692	http://api.brain-map.org/grid_data/download/68844289?include=energy
Slc17a7	0.692	http://api.brain-map.org/grid_data/download/75081210?include=energy
4930591A17Rik	0.692	http://api.brain-map.org/grid_data/download/71892023?include=energy
3010085J16Rik*	0.692	http://api.brain-map.org/grid_data/download/74359586?include=energy
1700022I11Rik	0.692	http://api.brain-map.org/grid_data/download/71377998?include=energy
Nnt	0.692	http://api.brain-map.org/grid_data/download/68443262?include=energy

Mapk11	0.692	http://api.brain-map.org/grid_data/download/74357574?include=energy
Itgb2l	0.692	http://api.brain-map.org/grid_data/download/69735622?include=energy
Cd22	0.692	http://api.brain-map.org/grid_data/download/69540561?include=energy
Mapk7	0.692	http://api.brain-map.org/grid_data/download/69752053?include=energy
Hs1bp3	0.692	http://api.brain-map.org/grid_data/download/74509452?include=energy
D930048G16Rik	0.691	http://api.brain-map.org/grid_data/download/70611503?include=energy
Vmn1r51	0.691	http://api.brain-map.org/grid_data/download/69169295?include=energy
Ankle1	0.691	http://api.brain-map.org/grid_data/download/73512541?include=energy
Chac2	0.691	http://api.brain-map.org/grid_data/download/67881779?include=energy
Ccdc111	0.691	http://api.brain-map.org/grid_data/download/71147870?include=energy
Ccdc57	0.691	http://api.brain-map.org/grid_data/download/68843949?include=energy
Tle6	0.691	http://api.brain-map.org/grid_data/download/69262347?include=energy
Olfr672	0.691	http://api.brain-map.org/grid_data/download/74734818?include=energy
Baalc	0.691	http://api.brain-map.org/grid_data/download/73520964?include=energy
Tada2a	0.691	http://api.brain-map.org/grid_data/download/678699?include=energy
LOC432913	0.691	http://api.brain-map.org/grid_data/download/69114091?include=energy
Ccl5	0.691	http://api.brain-map.org/grid_data/download/76135505?include=energy
Zfp446	0.691	http://api.brain-map.org/grid_data/download/69288578?include=energy
Thrb	0.691	http://api.brain-map.org/grid_data/download/71249069?include=energy
Mrpl38	0.691	http://api.brain-map.org/grid_data/download/68191534?include=energy
Irs2	0.691	http://api.brain-map.org/grid_data/download/74363343?include=energy
Nr5a1	0.691	http://api.brain-map.org/grid_data/download/734?include=energy
C430049B03Rik	0.691	http://api.brain-map.org/grid_data/download/71021023?include=energy
Fam126a	0.691	http://api.brain-map.org/grid_data/download/70528617?include=energy
Mtmr14	0.691	http://api.brain-map.org/grid_data/download/576417?include=energy
LOC434420	0.691	http://api.brain-map.org/grid_data/download/71022310?include=energy
Intu	0.69	http://api.brain-map.org/grid_data/download/73514803?include=energy
Maml3	0.69	http://api.brain-map.org/grid_data/download/68666917?include=energy
Rab19	0.69	http://api.brain-map.org/grid_data/download/68744588?include=energy
Spp2	0.69	http://api.brain-map.org/grid_data/download/68545551?include=energy
Krt73	0.69	http://api.brain-map.org/grid_data/download/74750042?include=energy
BC052486	0.69	http://api.brain-map.org/grid_data/download/74277732?include=energy
3110040M04Rik	0.69	http://api.brain-map.org/grid_data/download/70203633?include=energy
1700023A16Rik	0.69	http://api.brain-map.org/grid_data/download/71919517?include=energy
Clcn2	0.69	http://api.brain-map.org/grid_data/download/72119656?include=energy
Rnf20	0.69	http://api.brain-map.org/grid_data/download/73424190?include=energy
Olfr1449	0.69	http://api.brain-map.org/grid_data/download/74720844?include=energy
Jmjd5	0.69	http://api.brain-map.org/grid_data/download/68148683?include=energy
Mm.360771	0.69	http://api.brain-map.org/grid_data/download/71210001?include=energy
Sox6	0.69	http://api.brain-map.org/grid_data/download/71488807?include=energy
1700034F02Rik	0.69	http://api.brain-map.org/grid_data/download/70201403?include=energy
F730049H03Rik*	0.69	http://api.brain-map.org/grid_data/download/73731259?include=energy
Nckap1l	0.69	http://api.brain-map.org/grid_data/download/68162256?include=energy
Ccdc36	0.69	http://api.brain-map.org/grid_data/download/71022334?include=energy
LOC433997	0.69	http://api.brain-map.org/grid_data/download/72471828?include=energy
F830017M08Rik*	0.69	http://api.brain-map.org/grid_data/download/73748638?include=energy
Frzb	0.69	http://api.brain-map.org/grid_data/download/79556658?include=energy
LOC381412	0.689	http://api.brain-map.org/grid_data/download/74430900?include=energy
1700054N08Rik	0.689	http://api.brain-map.org/grid_data/download/70743908?include=energy
Csprs	0.689	http://api.brain-map.org/grid_data/download/69734738?include=energy
Slc5a2	0.689	http://api.brain-map.org/grid_data/download/68632936?include=energy
D230002A01Rik	0.689	http://api.brain-map.org/grid_data/download/69608863?include=energy
Abcb10	0.689	http://api.brain-map.org/grid_data/download/74881353?include=energy
Hoxc5	0.689	http://api.brain-map.org/grid_data/download/69289165?include=energy
Zscan20	0.689	http://api.brain-map.org/grid_data/download/67792183?include=energy
TC1496710	0.689	http://api.brain-map.org/grid_data/download/70812865?include=energy
Chn2	0.689	http://api.brain-map.org/grid_data/download/74881162?include=energy
Il11ra1	0.689	http://api.brain-map.org/grid_data/download/2315?include=energy
Myl12b	0.689	http://api.brain-map.org/grid_data/download/70784672?include=energy
Adh6a	0.689	http://api.brain-map.org/grid_data/download/70230807?include=energy
A730020M07Rik	0.689	http://api.brain-map.org/grid_data/download/69548789?include=energy
Rnf40	0.688	http://api.brain-map.org/grid_data/download/68632038?include=energy
Fezf2	0.688	http://api.brain-map.org/grid_data/download/75651163?include=energy
Slc25a35	0.688	http://api.brain-map.org/grid_data/download/68342021?include=energy
Zdhhc17	0.688	http://api.brain-map.org/grid_data/download/74300771?include=energy
Fkbp7	0.688	http://api.brain-map.org/grid_data/download/68545027?include=energy
Gcc2	0.688	http://api.brain-map.org/grid_data/download/67787335?include=energy
5033411D12Rik	0.688	http://api.brain-map.org/grid_data/download/69838601?include=energy
Tbc1d10a	0.688	http://api.brain-map.org/grid_data/download/532663?include=energy
Zkscan5	0.688	http://api.brain-map.org/grid_data/download/68844170?include=energy
Dock8	0.688	http://api.brain-map.org/grid_data/download/70919495?include=energy
Il6	0.688	http://api.brain-map.org/grid_data/download/71280931?include=energy
Aup1	0.688	http://api.brain-map.org/grid_data/download/68300950?include=energy
A330099J10Rik*	0.688	http://api.brain-map.org/grid_data/download/73949659?include=energy
March1	0.688	http://api.brain-map.org/grid_data/download/70203573?include=energy
Msl2	0.688	http://api.brain-map.org/grid_data/download/71213162?include=energy
Klf15	0.688	http://api.brain-map.org/grid_data/download/393192?include=energy
Tmem64	0.688	http://api.brain-map.org/grid_data/download/77414589?include=energy
Ccl12	0.688	http://api.brain-map.org/grid_data/download/68744922?include=energy
Smek2	0.687	http://api.brain-map.org/grid_data/download/386256?include=energy
Ms4a1	0.687	http://api.brain-map.org/grid_data/download/68545372?include=energy
Zfp451	0.687	http://api.brain-map.org/grid_data/download/587436?include=energy
Il1r1	0.687	http://api.brain-map.org/grid_data/download/69735383?include=energy
Ggt6	0.687	http://api.brain-map.org/grid_data/download/69028665?include=energy
Lipt1	0.687	http://api.brain-map.org/grid_data/download/100144376?include=energy
Furin	0.687	http://api.brain-map.org/grid_data/download/74822935?include=energy
Egln3	0.687	http://api.brain-map.org/grid_data/download/68196934?include=energy
Fabp7	0.687	http://api.brain-map.org/grid_data/download/72104184?include=energy
C530030P08Rik	0.687	http://api.brain-map.org/grid_data/download/73870623?include=energy
Dsn1	0.687	http://api.brain-map.org/grid_data/download/68522356?include=energy
Nudt5	0.687	http://api.brain-map.org/grid_data/download/68191330?include=energy
AI317395	0.686	http://api.brain-map.org/grid_data/download/71656124?include=energy
Ppp4r2	0.686	http://api.brain-map.org/grid_data/download/74511850?include=energy
3110006O06Rik	0.686	http://api.brain-map.org/grid_data/download/69407451?include=energy
AW209491	0.686	http://api.brain-map.org/grid_data/download/634472?include=energy
Dhfr	0.686	http://api.brain-map.org/grid_data/download/633296?include=energy
Kdm1b	0.686	http://api.brain-map.org/grid_data/download/68161482?include=energy
A230004M16Rik	0.686	http://api.brain-map.org/grid_data/download/74431758?include=energy
Zfp120	0.686	http://api.brain-map.org/grid_data/download/70445323?include=energy
Apoa2	0.686	http://api.brain-map.org/grid_data/download/68632190?include=energy
A530032D15Rik	0.686	http://api.brain-map.org/grid_data/download/70228478?include=energy
Gm5083	0.686	http://api.brain-map.org/grid_data/download/73930833?include=energy
Tmem136	0.686	http://api.brain-map.org/grid_data/download/70295960?include=energy
Slc9a3	0.686	http://api.brain-map.org/grid_data/download/70227998?include=energy
Tmem225	0.686	http://api.brain-map.org/grid_data/download/73512565?include=energy
Acot4	0.686	http://api.brain-map.org/grid_data/download/71210858?include=energy
Rrnad1	0.686	http://api.brain-map.org/grid_data/download/68160843?include=energy
Fastkd3	0.685	http://api.brain-map.org/grid_data/download/68844612?include=energy
Ercc2	0.685	http://api.brain-map.org/grid_data/download/532721?include=energy
Itih1	0.685	http://api.brain-map.org/grid_data/download/68499040?include=energy
Ccdc129	0.685	http://api.brain-map.org/grid_data/download/70238686?include=energy
Hdac1	0.685	http://api.brain-map.org/grid_data/download/69734953?include=energy
Krt34	0.685	http://api.brain-map.org/grid_data/download/70724894?include=energy
Pola2	0.685	http://api.brain-map.org/grid_data/download/68196964?include=energy
Stk4	0.685	http://api.brain-map.org/grid_data/download/68843769?include=energy
2310046A06Rik	0.685	http://api.brain-map.org/grid_data/download/73931408?include=energy
2610017I09Rik	0.685	http://api.brain-map.org/grid_data/download/77280584?include=energy
Vmn1r72	0.685	http://api.brain-map.org/grid_data/download/70785648?include=energy
Sf3b3	0.685	http://api.brain-map.org/grid_data/download/392910?include=energy
Rod1	0.685	http://api.brain-map.org/grid_data/download/73424196?include=energy
4930533L02Rik	0.685	http://api.brain-map.org/grid_data/download/74265159?include=energy
9030224M15Rik	0.685	http://api.brain-map.org/grid_data/download/74581410?include=energy
Nfib	0.685	http://api.brain-map.org/grid_data/download/1555?include=energy
Zfp382	0.685	http://api.brain-map.org/grid_data/download/72471780?include=energy
Tubg1	0.685	http://api.brain-map.org/grid_data/download/68269035?include=energy
Rab32	0.685	http://api.brain-map.org/grid_data/download/69549652?include=energy
1700020O03Rik	0.685	http://api.brain-map.org/grid_data/download/69028671?include=energy
D930030O05Rik	0.684	http://api.brain-map.org/grid_data/download/70238818?include=energy
Slc1a3	0.684	http://api.brain-map.org/grid_data/download/77620233?include=energy
5430417C01Rik	0.684	http://api.brain-map.org/grid_data/download/74265303?include=energy
Zfp90	0.684	http://api.brain-map.org/grid_data/download/74431333?include=energy
A330004N04Rik*	0.684	http://api.brain-map.org/grid_data/download/73808534?include=energy
Slc26a4	0.684	http://api.brain-map.org/grid_data/download/70302117?include=energy
Dennd2a	0.684	http://api.brain-map.org/grid_data/download/69528034?include=energy
Elf3	0.684	http://api.brain-map.org/grid_data/download/71380405?include=energy
Cyp2c44	0.684	http://api.brain-map.org/grid_data/download/70528336?include=energy
Casp8ap2	0.684	http://api.brain-map.org/grid_data/download/69540399?include=energy
Smox	0.684	http://api.brain-map.org/grid_data/download/67881784?include=energy
Rnf19b	0.684	http://api.brain-map.org/grid_data/download/70277991?include=energy
Serpinc1	0.684	http://api.brain-map.org/grid_data/download/68498433?include=energy
Nubpl	0.684	http://api.brain-map.org/grid_data/download/74359395?include=energy
Anxa11	0.684	http://api.brain-map.org/grid_data/download/2029?include=energy
Hsd3b7	0.684	http://api.brain-map.org/grid_data/download/633272?include=energy
AY512931	0.684	http://api.brain-map.org/grid_data/download/70793875?include=energy
Oprk1	0.684	http://api.brain-map.org/grid_data/download/80517217?include=energy
Fam124b	0.683	http://api.brain-map.org/grid_data/download/70717981?include=energy
Prkca	0.683	http://api.brain-map.org/grid_data/download/840?include=energy
Dusp14	0.683	http://api.brain-map.org/grid_data/download/70927831?include=energy
Tmem229a	0.683	http://api.brain-map.org/grid_data/download/74822904?include=energy
Rras2	0.683	http://api.brain-map.org/grid_data/download/70565189?include=energy
Fbn2	0.683	http://api.brain-map.org/grid_data/download/72472799?include=energy
8430406I07Rik	0.683	http://api.brain-map.org/grid_data/download/70429142?include=energy
LOC433093	0.683	http://api.brain-map.org/grid_data/download/71924514?include=energy
Aldh8a1	0.683	http://api.brain-map.org/grid_data/download/68191240?include=energy
Hand1	0.683	http://api.brain-map.org/grid_data/download/100142509?include=energy
Rab20	0.683	http://api.brain-map.org/grid_data/download/69817811?include=energy
Dkk3	0.682	http://api.brain-map.org/grid_data/download/79556605?include=energy
Ccdc86	0.682	http://api.brain-map.org/grid_data/download/68920650?include=energy
4933409K07Rik	0.682	http://api.brain-map.org/grid_data/download/69443148?include=energy
Smcr7l	0.682	http://api.brain-map.org/grid_data/download/69524560?include=energy
Dmrtc1a	0.682	http://api.brain-map.org/grid_data/download/70610381?include=energy
Gm8656	0.682	http://api.brain-map.org/grid_data/download/74359736?include=energy
Gm10037	0.682	http://api.brain-map.org/grid_data/download/70725043?include=energy
9930005F22Rik	0.682	http://api.brain-map.org/grid_data/download/71022597?include=energy
Msh4	0.682	http://api.brain-map.org/grid_data/download/70300427?include=energy
Foxd1	0.682	http://api.brain-map.org/grid_data/download/81790670?include=energy
Mmp3	0.682	http://api.brain-map.org/grid_data/download/68300809?include=energy
Plekho1	0.682	http://api.brain-map.org/grid_data/download/69407248?include=energy
Ccdc14	0.682	http://api.brain-map.org/grid_data/download/70545907?include=energy
Pgbd5	0.681	http://api.brain-map.org/grid_data/download/73931642?include=energy
Cybasc3	0.681	http://api.brain-map.org/grid_data/download/70946226?include=energy
Bdnf	0.681	http://api.brain-map.org/grid_data/download/79587720?include=energy
Synrg	0.681	http://api.brain-map.org/grid_data/download/71495783?include=energy
Cct8l1	0.681	http://api.brain-map.org/grid_data/download/69114646?include=energy
Xkr5	0.681	http://api.brain-map.org/grid_data/download/70218932?include=energy
Stac	0.681	http://api.brain-map.org/grid_data/download/73788116?include=energy
Ltk	0.681	http://api.brain-map.org/grid_data/download/75988666?include=energy
Gmppb	0.681	http://api.brain-map.org/grid_data/download/69734470?include=energy
5430401F13Rik	0.681	http://api.brain-map.org/grid_data/download/70218902?include=energy
Gm777	0.681	http://api.brain-map.org/grid_data/download/74514079?include=energy
2410018M08Rik	0.681	http://api.brain-map.org/grid_data/download/75985869?include=energy
Hspb2	0.681	http://api.brain-map.org/grid_data/download/69735150?include=energy
Lyplal1	0.68	http://api.brain-map.org/grid_data/download/68444702?include=energy
9130023H24Rik	0.68	http://api.brain-map.org/grid_data/download/73513347?include=energy
Glt8d2	0.68	http://api.brain-map.org/grid_data/download/71249079?include=energy
Pde1a	0.68	http://api.brain-map.org/grid_data/download/782?include=energy
Lrba	0.68	http://api.brain-map.org/grid_data/download/69736204?include=energy
Bcl9	0.68	http://api.brain-map.org/grid_data/download/641531?include=energy
9530064J02	0.68	http://api.brain-map.org/grid_data/download/70305198?include=energy
Ddx19a	0.68	http://api.brain-map.org/grid_data/download/71992031?include=energy
Gpx6	0.68	http://api.brain-map.org/grid_data/download/686205?include=energy
Mad2l1bp	0.679	http://api.brain-map.org/grid_data/download/68269297?include=energy
LOC381394	0.679	http://api.brain-map.org/grid_data/download/74635773?include=energy
Kbtbd13	0.679	http://api.brain-map.org/grid_data/download/69443439?include=energy
Stard3	0.679	http://api.brain-map.org/grid_data/download/67978498?include=energy
Ltf	0.679	http://api.brain-map.org/grid_data/download/633694?include=energy
Sypl2	0.679	http://api.brain-map.org/grid_data/download/74357803?include=energy
Wisp1	0.679	http://api.brain-map.org/grid_data/download/68667799?include=energy
1700020L24Rik	0.679	http://api.brain-map.org/grid_data/download/73818856?include=energy
C8g	0.679	http://api.brain-map.org/grid_data/download/587431?include=energy
Aym1	0.679	http://api.brain-map.org/grid_data/download/73513507?include=energy
Rpa3	0.679	http://api.brain-map.org/grid_data/download/68545681?include=energy
Adamts20	0.679	http://api.brain-map.org/grid_data/download/69514804?include=energy
Thpo	0.679	http://api.brain-map.org/grid_data/download/2671?include=energy
Kif21b	0.678	http://api.brain-map.org/grid_data/download/69735777?include=energy
Aasdh	0.678	http://api.brain-map.org/grid_data/download/69447960?include=energy
Fbxo17	0.678	http://api.brain-map.org/grid_data/download/69549163?include=energy
Lrp4	0.678	http://api.brain-map.org/grid_data/download/71670487?include=energy
6030405A18Rik	0.678	http://api.brain-map.org/grid_data/download/71920518?include=energy
Il16	0.678	http://api.brain-map.org/grid_data/download/71064290?include=energy
Muc6	0.678	http://api.brain-map.org/grid_data/download/70300445?include=energy
Dtx4	0.678	http://api.brain-map.org/grid_data/download/69671642?include=energy
Mm.86217	0.678	http://api.brain-map.org/grid_data/download/74641161?include=energy
Adam18	0.678	http://api.brain-map.org/grid_data/download/70228711?include=energy
Itga6	0.678	http://api.brain-map.org/grid_data/download/68919830?include=energy
Mrps23	0.677	http://api.brain-map.org/grid_data/download/638706?include=energy
Btaf1	0.677	http://api.brain-map.org/grid_data/download/70239075?include=energy
Glyctk	0.677	http://api.brain-map.org/grid_data/download/68499046?include=energy
Ccdc147	0.677	http://api.brain-map.org/grid_data/download/70298525?include=energy
A830036E02Rik	0.677	http://api.brain-map.org/grid_data/download/74512028?include=energy
Ddah2	0.677	http://api.brain-map.org/grid_data/download/67978684?include=energy
4921511E18Rik	0.677	http://api.brain-map.org/grid_data/download/71152342?include=energy
Tas2r135	0.677	http://api.brain-map.org/grid_data/download/70302390?include=energy
Rspo1	0.676	http://api.brain-map.org/grid_data/download/73636101?include=energy
Fanca	0.676	http://api.brain-map.org/grid_data/download/69672282?include=energy
Dpp8	0.676	http://api.brain-map.org/grid_data/download/73512513?include=energy
2810442I21Rik	0.676	http://api.brain-map.org/grid_data/download/71764370?include=energy
Zc3h4	0.676	http://api.brain-map.org/grid_data/download/70528384?include=energy
Six1	0.676	http://api.brain-map.org/grid_data/download/1834?include=energy
D030063F01Rik	0.676	http://api.brain-map.org/grid_data/download/73931628?include=energy
Peli2	0.676	http://api.brain-map.org/grid_data/download/68148696?include=energy
Acot3	0.675	http://api.brain-map.org/grid_data/download/71210852?include=energy
Ube2dnl2	0.675	http://api.brain-map.org/grid_data/download/71570760?include=energy
Tsta3	0.675	http://api.brain-map.org/grid_data/download/70919125?include=energy
Tbx20	0.675	http://api.brain-map.org/grid_data/download/73497232?include=energy
Col24a1	0.675	http://api.brain-map.org/grid_data/download/73636093?include=energy
Syt16	0.675	http://api.brain-map.org/grid_data/download/71358663?include=energy
Neil2	0.675	http://api.brain-map.org/grid_data/download/80525367?include=energy
Kbtbd12	0.675	http://api.brain-map.org/grid_data/download/70218816?include=energy
Tmem219	0.675	http://api.brain-map.org/grid_data/download/68080705?include=energy
Gm16519	0.675	http://api.brain-map.org/grid_data/download/74736480?include=energy
Serpinb9g	0.675	http://api.brain-map.org/grid_data/download/68910984?include=energy
2310047D07Rik	0.675	http://api.brain-map.org/grid_data/download/72283354?include=energy
Stk19	0.675	http://api.brain-map.org/grid_data/download/67815955?include=energy
Neurod1	0.675	http://api.brain-map.org/grid_data/download/75650865?include=energy
Gm525	0.675	http://api.brain-map.org/grid_data/download/70295727?include=energy
LOC433823	0.675	http://api.brain-map.org/grid_data/download/70786858?include=energy
Zfp229	0.675	http://api.brain-map.org/grid_data/download/69288692?include=energy
Pgap3	0.675	http://api.brain-map.org/grid_data/download/69782987?include=energy
Rasl10a	0.674	http://api.brain-map.org/grid_data/download/73636038?include=energy
Tpp2	0.674	http://api.brain-map.org/grid_data/download/68797995?include=energy
Adam2	0.674	http://api.brain-map.org/grid_data/download/69514762?include=energy
Btg1	0.674	http://api.brain-map.org/grid_data/download/73718576?include=energy
Lmna	0.674	http://api.brain-map.org/grid_data/download/73512494?include=energy
Radil	0.674	http://api.brain-map.org/grid_data/download/68843973?include=energy
O3far1	0.674	http://api.brain-map.org/grid_data/download/69838559?include=energy
5730455O13Rik	0.674	http://api.brain-map.org/grid_data/download/71992049?include=energy
Klrk1	0.674	http://api.brain-map.org/grid_data/download/69549653?include=energy
TC1412975	0.674	http://api.brain-map.org/grid_data/download/70793971?include=energy
Kbtbd5	0.673	http://api.brain-map.org/grid_data/download/69289267?include=energy
Slc17a5	0.673	http://api.brain-map.org/grid_data/download/68798013?include=energy
C030025P15Rik	0.673	http://api.brain-map.org/grid_data/download/70946238?include=energy
Gpr174	0.673	http://api.brain-map.org/grid_data/download/69257373?include=energy
LOC433588	0.673	http://api.brain-map.org/grid_data/download/70785810?include=energy
A930037D12Rik*	0.673	http://api.brain-map.org/grid_data/download/73808343?include=energy
Npc1l1	0.673	http://api.brain-map.org/grid_data/download/77924414?include=energy
Tmem200a	0.673	http://api.brain-map.org/grid_data/download/79591403?include=energy

Uggt2	0.673	http://api.brain-map.org/grid_data/download/71211626?include=energy
Rangrf	0.673	http://api.brain-map.org/grid_data/download/68162257?include=energy
Pbk	0.673	http://api.brain-map.org/grid_data/download/70784726?include=energy
Trim27	0.673	http://api.brain-map.org/grid_data/download/67979051?include=energy
9130015A21Rik	0.673	http://api.brain-map.org/grid_data/download/70228016?include=energy
Trcg1	0.673	http://api.brain-map.org/grid_data/download/74276887?include=energy
Tspan31	0.673	http://api.brain-map.org/grid_data/download/70473993?include=energy
Mudeng	0.672	http://api.brain-map.org/grid_data/download/67787329?include=energy
Ccdc33	0.672	http://api.brain-map.org/grid_data/download/69442353?include=energy
Cpne8	0.672	http://api.brain-map.org/grid_data/download/73520974?include=energy
H2-M10.1	0.672	http://api.brain-map.org/grid_data/download/70724792?include=energy
Nsmce1	0.672	http://api.brain-map.org/grid_data/download/68637515?include=energy
Stk40	0.672	http://api.brain-map.org/grid_data/download/69012668?include=energy
Adam32	0.672	http://api.brain-map.org/grid_data/download/74581193?include=energy
Vmn1r11	0.672	http://api.brain-map.org/grid_data/download/70785248?include=energy
Acot9	0.672	http://api.brain-map.org/grid_data/download/641546?include=energy
Colq	0.672	http://api.brain-map.org/grid_data/download/74514091?include=energy
Prss23	0.672	http://api.brain-map.org/grid_data/download/70634118?include=energy
Hoxb3	0.672	http://api.brain-map.org/grid_data/download/69289153?include=energy
Dph3	0.672	http://api.brain-map.org/grid_data/download/68191174?include=energy
Gulo	0.671	http://api.brain-map.org/grid_data/download/68498427?include=energy
Pik3ap1	0.671	http://api.brain-map.org/grid_data/download/71377691?include=energy
Pitpnm3	0.671	http://api.brain-map.org/grid_data/download/71494678?include=energy
Gpc4	0.671	http://api.brain-map.org/grid_data/download/1617?include=energy
Nckap5	0.671	http://api.brain-map.org/grid_data/download/69608483?include=energy
Cldn12	0.671	http://api.brain-map.org/grid_data/download/2107?include=energy
E230015B07Rik	0.671	http://api.brain-map.org/grid_data/download/69671780?include=energy
Pigx	0.671	http://api.brain-map.org/grid_data/download/71610788?include=energy
Terf1	0.671	http://api.brain-map.org/grid_data/download/68545923?include=energy
F930003F07Rik*	0.671	http://api.brain-map.org/grid_data/download/73767412?include=energy
Palmd	0.671	http://api.brain-map.org/grid_data/download/74581442?include=energy
0610007P08Rik	0.671	http://api.brain-map.org/grid_data/download/68523288?include=energy
BC048943	0.67	http://api.brain-map.org/grid_data/download/69529149?include=energy
Cldn9	0.67	http://api.brain-map.org/grid_data/download/75831774?include=energy
LOC432471	0.67	http://api.brain-map.org/grid_data/download/74509530?include=energy
Mpped1	0.67	http://api.brain-map.org/grid_data/download/70231303?include=energy
Mkks	0.67	http://api.brain-map.org/grid_data/download/70919209?include=energy
Chmp2b	0.67	http://api.brain-map.org/grid_data/download/70474809?include=energy
D430020J02Rik	0.67	http://api.brain-map.org/grid_data/download/72283581?include=energy
Fam81a	0.67	http://api.brain-map.org/grid_data/download/73732150?include=energy
Mtag2	0.67	http://api.brain-map.org/grid_data/download/73712924?include=energy
Egf	0.67	http://api.brain-map.org/grid_data/download/80341155?include=energy
Gm88	0.67	http://api.brain-map.org/grid_data/download/70295781?include=energy
Hpcal4	0.67	http://api.brain-map.org/grid_data/download/73520985?include=energy
Tmed6	0.67	http://api.brain-map.org/grid_data/download/70201558?include=energy
Vip	0.669	http://api.brain-map.org/grid_data/download/77371835?include=energy
Galnt4	0.669	http://api.brain-map.org/grid_data/download/69015380?include=energy
LOC433459	0.669	http://api.brain-map.org/grid_data/download/70723265?include=energy
Adra1d	0.669	http://api.brain-map.org/grid_data/download/72119628?include=energy
Fam135b	0.669	http://api.brain-map.org/grid_data/download/74581399?include=energy
Ipmk	0.669	http://api.brain-map.org/grid_data/download/69735496?include=energy
Kcnh1	0.669	http://api.brain-map.org/grid_data/download/73636099?include=energy
Cebpz	0.669	http://api.brain-map.org/grid_data/download/68157406?include=energy
Hsd3b6	0.669	http://api.brain-map.org/grid_data/download/68667186?include=energy
Gm5585	0.669	http://api.brain-map.org/grid_data/download/70787420?include=energy
Pclo	0.669	http://api.brain-map.org/grid_data/download/71670682?include=energy
Aldh1l2	0.668	http://api.brain-map.org/grid_data/download/68631984?include=energy
Bpifa3	0.668	http://api.brain-map.org/grid_data/download/74635066?include=energy
Hsf2	0.668	http://api.brain-map.org/grid_data/download/68637909?include=energy
Gm1257	0.668	http://api.brain-map.org/grid_data/download/70300073?include=energy
Lmtk2	0.668	http://api.brain-map.org/grid_data/download/69736148?include=energy
Zfp697	0.668	http://api.brain-map.org/grid_data/download/70230837?include=energy
B930041F14Rik	0.668	http://api.brain-map.org/grid_data/download/69528010?include=energy
Plekha2	0.668	http://api.brain-map.org/grid_data/download/70743843?include=energy
Tgfb1	0.668	http://api.brain-map.org/grid_data/download/80343319?include=energy
Nkx6-3	0.668	http://api.brain-map.org/grid_data/download/69816458?include=energy
Doc2b	0.668	http://api.brain-map.org/grid_data/download/72283810?include=energy
Ly6g5c	0.668	http://api.brain-map.org/grid_data/download/70300246?include=energy
Mboat2	0.668	http://api.brain-map.org/grid_data/download/545123?include=energy
Oip5	0.668	http://api.brain-map.org/grid_data/download/70218974?include=energy
Fosb	0.668	http://api.brain-map.org/grid_data/download/69288986?include=energy
Gm5535	0.668	http://api.brain-map.org/grid_data/download/70723295?include=energy
Foxk1	0.667	http://api.brain-map.org/grid_data/download/73497147?include=energy
Ksr1	0.667	http://api.brain-map.org/grid_data/download/69735980?include=energy
Cdkn2a	0.667	http://api.brain-map.org/grid_data/download/74430996?include=energy
Skor1	0.667	http://api.brain-map.org/grid_data/download/69530929?include=energy
LOC546577	0.667	http://api.brain-map.org/grid_data/download/73712828?include=energy
Paqr9	0.667	http://api.brain-map.org/grid_data/download/69782730?include=energy
LOC433173	0.667	http://api.brain-map.org/grid_data/download/70722964?include=energy
Ncor2	0.667	http://api.brain-map.org/grid_data/download/69782091?include=energy
Lrrc63	0.666	http://api.brain-map.org/grid_data/download/71283498?include=energy
Sec31b	0.666	http://api.brain-map.org/grid_data/download/74431209?include=energy
Ccbp2	0.666	http://api.brain-map.org/grid_data/download/74724637?include=energy
Lix1	0.666	http://api.brain-map.org/grid_data/download/643?include=energy
Arl14	0.666	http://api.brain-map.org/grid_data/download/70228010?include=energy
Gm4956	0.666	http://api.brain-map.org/grid_data/download/69117098?include=energy
Tmem9b	0.666	http://api.brain-map.org/grid_data/download/631723?include=energy
4833414E09Rik	0.666	http://api.brain-map.org/grid_data/download/73521828?include=energy
Tdg	0.666	http://api.brain-map.org/grid_data/download/71020392?include=energy
Stk36	0.666	http://api.brain-map.org/grid_data/download/71152497?include=energy
Apold1	0.666	http://api.brain-map.org/grid_data/download/71213677?include=energy
Fam160a1	0.666	http://api.brain-map.org/grid_data/download/74363337?include=energy
5830469L07Rik*	0.666	http://api.brain-map.org/grid_data/download/73927601?include=energy
Nr4a3	0.666	http://api.brain-map.org/grid_data/download/73615793?include=energy
Pdk4	0.665	http://api.brain-map.org/grid_data/download/68546548?include=energy
Sipa1l2	0.665	http://api.brain-map.org/grid_data/download/72118960?include=energy
Tmem181a	0.665	http://api.brain-map.org/grid_data/download/74509560?include=energy
Met	0.665	http://api.brain-map.org/grid_data/download/79591401?include=energy
Vnn3	0.665	http://api.brain-map.org/grid_data/download/70194940?include=energy
Cln3	0.664	http://api.brain-map.org/grid_data/download/68745084?include=energy
4930447K03Rik	0.664	http://api.brain-map.org/grid_data/download/70725507?include=energy
Gins3	0.664	http://api.brain-map.org/grid_data/download/69549605?include=energy
Grwd1	0.664	http://api.brain-map.org/grid_data/download/67792214?include=energy
Mm.352566	0.664	http://api.brain-map.org/grid_data/download/71161159?include=energy
Mm.152121	0.663	http://api.brain-map.org/grid_data/download/71148223?include=energy
9830147E19Rik	0.663	http://api.brain-map.org/grid_data/download/70593192?include=energy
Lpar5	0.663	http://api.brain-map.org/grid_data/download/69257666?include=energy
Pde5a	0.663	http://api.brain-map.org/grid_data/download/73931641?include=energy
Mfsd9	0.663	http://api.brain-map.org/grid_data/download/69014722?include=energy
Plekha4	0.663	http://api.brain-map.org/grid_data/download/67881772?include=energy
D1Ertd622e	0.663	http://api.brain-map.org/grid_data/download/68410327?include=energy
Klhl15	0.663	http://api.brain-map.org/grid_data/download/686185?include=energy
Rgmb	0.662	http://api.brain-map.org/grid_data/download/68162223?include=energy
Creb3l4	0.662	http://api.brain-map.org/grid_data/download/69549151?include=energy
Mtus2	0.662	http://api.brain-map.org/grid_data/download/70431434?include=energy
Mas1	0.662	http://api.brain-map.org/grid_data/download/73636100?include=energy
Ets2	0.662	http://api.brain-map.org/grid_data/download/1202?include=energy
Pdss2	0.662	http://api.brain-map.org/grid_data/download/74265309?include=energy
Fibcd1	0.662	http://api.brain-map.org/grid_data/download/72472801?include=energy
Ubtd2	0.661	http://api.brain-map.org/grid_data/download/69838317?include=energy
Nrp1	0.661	http://api.brain-map.org/grid_data/download/74272479?include=energy
Zg16	0.661	http://api.brain-map.org/grid_data/download/68632924?include=energy
Lrrc4	0.661	http://api.brain-map.org/grid_data/download/75041463?include=energy
Gif	0.66	http://api.brain-map.org/grid_data/download/72182974?include=energy
Enoph1	0.66	http://api.brain-map.org/grid_data/download/545107?include=energy
Gcsh	0.66	http://api.brain-map.org/grid_data/download/69015500?include=energy
Cckbr	0.66	http://api.brain-map.org/grid_data/download/70560342?include=energy
Sarm1	0.66	http://api.brain-map.org/grid_data/download/68920419?include=energy
Olfr592	0.66	http://api.brain-map.org/grid_data/download/74724223?include=energy
Tex261	0.66	http://api.brain-map.org/grid_data/download/74047919?include=energy
1600014K23Rik	0.66	http://api.brain-map.org/grid_data/download/70201050?include=energy
Lrrc56	0.66	http://api.brain-map.org/grid_data/download/68162198?include=energy
Rapgef5	0.66	http://api.brain-map.org/grid_data/download/70301084?include=energy
B930007M17Rik	0.66	http://api.brain-map.org/grid_data/download/69527992?include=energy
Amh	0.66	http://api.brain-map.org/grid_data/download/69526577?include=energy
LOC436044	0.659	http://api.brain-map.org/grid_data/download/70784570?include=energy
Mmd2	0.659	http://api.brain-map.org/grid_data/download/74562206?include=energy
Cadps2	0.659	http://api.brain-map.org/grid_data/download/73817429?include=energy
Med13l	0.659	http://api.brain-map.org/grid_data/download/74056141?include=energy
Vtcn1	0.659	http://api.brain-map.org/grid_data/download/68545644?include=energy
Zfp809	0.659	http://api.brain-map.org/grid_data/download/71307184?include=energy
Scgb3a1	0.659	http://api.brain-map.org/grid_data/download/71210883?include=energy
Syt10	0.659	http://api.brain-map.org/grid_data/download/79556609?include=energy
4932441B19Rik	0.659	http://api.brain-map.org/grid_data/download/70566229?include=energy
Fgf6	0.659	http://api.brain-map.org/grid_data/download/69672444?include=energy
Lman2l	0.658	http://api.brain-map.org/grid_data/download/68745227?include=energy
Mfng	0.658	http://api.brain-map.org/grid_data/download/68301096?include=energy
Tbc1d30	0.658	http://api.brain-map.org/grid_data/download/72283432?include=energy
Cenpo	0.658	http://api.brain-map.org/grid_data/download/71763679?include=energy
Hs2st1	0.658	http://api.brain-map.org/grid_data/download/556981?include=energy
Mtrf1	0.658	http://api.brain-map.org/grid_data/download/68632637?include=energy
Cdc73	0.658	http://api.brain-map.org/grid_data/download/67792181?include=energy
Bet3l	0.658	http://api.brain-map.org/grid_data/download/100144090?include=energy
Mup2	0.658	http://api.brain-map.org/grid_data/download/680?include=energy
Pabpc4	0.658	http://api.brain-map.org/grid_data/download/68300896?include=energy
Gm4872	0.658	http://api.brain-map.org/grid_data/download/70305682?include=energy
Aldh9a1	0.658	http://api.brain-map.org/grid_data/download/633680?include=energy
4930544L04Rik	0.657	http://api.brain-map.org/grid_data/download/73927571?include=energy
BC042761	0.657	http://api.brain-map.org/grid_data/download/71574540?include=energy
Hkdc1	0.657	http://api.brain-map.org/grid_data/download/70634299?include=energy
Tmem247	0.657	http://api.brain-map.org/grid_data/download/72081683?include=energy
Mmp19	0.657	http://api.brain-map.org/grid_data/download/70300385?include=energy
Tas2r130	0.657	http://api.brain-map.org/grid_data/download/70302378?include=energy
Rgr	0.657	http://api.brain-map.org/grid_data/download/73712732?include=energy
Mm.323455	0.657	http://api.brain-map.org/grid_data/download/74430930?include=energy
Cchcr1	0.657	http://api.brain-map.org/grid_data/download/67787336?include=energy
Strm	0.657	http://api.brain-map.org/grid_data/download/69816942?include=energy
1700013J13Rik*	0.656	http://api.brain-map.org/grid_data/download/74357809?include=energy
Cacng8	0.656	http://api.brain-map.org/grid_data/download/72108823?include=energy
LOC432407	0.656	http://api.brain-map.org/grid_data/download/70608454?include=energy
Plscr4	0.656	http://api.brain-map.org/grid_data/download/68744456?include=energy
Asap1	0.656	http://api.brain-map.org/grid_data/download/73512377?include=energy
1700065J11Rik	0.656	http://api.brain-map.org/grid_data/download/71920147?include=energy
D630045M09Rik	0.656	http://api.brain-map.org/grid_data/download/70611287?include=energy
Fbxo6	0.656	http://api.brain-map.org/grid_data/download/638725?include=energy
Ms4a4d	0.656	http://api.brain-map.org/grid_data/download/71020822?include=energy
Bag4	0.655	http://api.brain-map.org/grid_data/download/68844241?include=energy
Krtap26-1	0.655	http://api.brain-map.org/grid_data/download/70203465?include=energy
A130034H22Rik*	0.655	http://api.brain-map.org/grid_data/download/73949499?include=energy
Yipf5	0.655	http://api.brain-map.org/grid_data/download/67978648?include=energy
4933427D14Rik	0.655	http://api.brain-map.org/grid_data/download/74272053?include=energy
Guf1	0.655	http://api.brain-map.org/grid_data/download/74562230?include=energy
Slc25a46	0.654	http://api.brain-map.org/grid_data/download/587418?include=energy
LOC433965	0.654	http://api.brain-map.org/grid_data/download/74635186?include=energy
Rxfp1	0.654	http://api.brain-map.org/grid_data/download/70562124?include=energy
Gsdmcl1	0.654	http://api.brain-map.org/grid_data/download/71378017?include=energy
A830018L16Rik	0.654	http://api.brain-map.org/grid_data/download/73818758?include=energy
2010109I03Rik	0.654	http://api.brain-map.org/grid_data/download/69202226?include=energy
Gna15	0.654	http://api.brain-map.org/grid_data/download/68203478?include=energy
0610009B22Rik	0.654	http://api.brain-map.org/grid_data/download/71763939?include=energy
Serpinb1b	0.654	http://api.brain-map.org/grid_data/download/544327?include=energy
Mug1	0.654	http://api.brain-map.org/grid_data/download/69013432?include=energy
BC049715	0.654	http://api.brain-map.org/grid_data/download/69113704?include=energy
Gm1625	0.654	http://api.brain-map.org/grid_data/download/71668662?include=energy
Dact1	0.654	http://api.brain-map.org/grid_data/download/69626612?include=energy
Trpc4	0.653	http://api.brain-map.org/grid_data/download/1305?include=energy
Slc45a2	0.653	http://api.brain-map.org/grid_data/download/70300288?include=energy
4930432F04Rik	0.653	http://api.brain-map.org/grid_data/download/74076204?include=energy
Ercc8	0.653	http://api.brain-map.org/grid_data/download/67792198?include=energy
Akr1c14	0.653	http://api.brain-map.org/grid_data/download/68321958?include=energy
mCG147104	0.653	http://api.brain-map.org/grid_data/download/71212518?include=energy
LOC385649	0.652	http://api.brain-map.org/grid_data/download/74431570?include=energy
Dennd2d	0.652	http://api.brain-map.org/grid_data/download/68269286?include=energy
Eri1	0.652	http://api.brain-map.org/grid_data/download/69838406?include=energy
Gpatch3	0.652	http://api.brain-map.org/grid_data/download/69626576?include=energy
Dusp5	0.652	http://api.brain-map.org/grid_data/download/73931633?include=energy
Rnaseh1	0.652	http://api.brain-map.org/grid_data/download/671240?include=energy
Grin2a	0.652	http://api.brain-map.org/grid_data/download/75081000?include=energy
4933424G06Rik	0.651	http://api.brain-map.org/grid_data/download/69443208?include=energy
Leprel1	0.651	http://api.brain-map.org/grid_data/download/70295482?include=energy
Olfr1490	0.651	http://api.brain-map.org/grid_data/download/74720856?include=energy
Zkscan14	0.651	http://api.brain-map.org/grid_data/download/80471971?include=energy
Kras	0.651	http://api.brain-map.org/grid_data/download/67979046?include=energy
Rfesd	0.65	http://api.brain-map.org/grid_data/download/68162219?include=energy
Zfp91	0.65	http://api.brain-map.org/grid_data/download/71152623?include=energy
LOC433228	0.65	http://api.brain-map.org/grid_data/download/73788454?include=energy
Poli	0.65	http://api.brain-map.org/grid_data/download/69817157?include=energy
Dmrt2	0.65	http://api.brain-map.org/grid_data/download/68544967?include=energy
Klf10	0.65	http://api.brain-map.org/grid_data/download/70744519?include=energy
Hey2	0.65	http://api.brain-map.org/grid_data/download/69289087?include=energy
Trib1	0.65	http://api.brain-map.org/grid_data/download/71015818?include=energy
I920196L20Rik*	0.649	http://api.brain-map.org/grid_data/download/73731358?include=energy
Nufip1	0.649	http://api.brain-map.org/grid_data/download/68667330?include=energy
Il23r	0.649	http://api.brain-map.org/grid_data/download/70278063?include=energy
Gm1070	0.649	http://api.brain-map.org/grid_data/download/70299959?include=energy
Cps1	0.649	http://api.brain-map.org/grid_data/download/69608022?include=energy
Rhoh	0.649	http://api.brain-map.org/grid_data/download/71210871?include=energy
Plac1l	0.649	http://api.brain-map.org/grid_data/download/70295811?include=energy
Magt1	0.649	http://api.brain-map.org/grid_data/download/71212557?include=energy
Rab8b	0.649	http://api.brain-map.org/grid_data/download/68844618?include=energy
Zdhhc8	0.649	http://api.brain-map.org/grid_data/download/71211359?include=energy
Rtn4rl2	0.649	http://api.brain-map.org/grid_data/download/74581375?include=energy
Tesk2	0.649	http://api.brain-map.org/grid_data/download/556979?include=energy
Mitf	0.649	http://api.brain-map.org/grid_data/download/71325258?include=energy
Spred2	0.648	http://api.brain-map.org/grid_data/download/71724714?include=energy
Iqgap2	0.648	http://api.brain-map.org/grid_data/download/73615573?include=energy
Tas2r125	0.648	http://api.brain-map.org/grid_data/download/70785188?include=energy
2610301B20Rik	0.648	http://api.brain-map.org/grid_data/download/68745060?include=energy
Figla	0.648	http://api.brain-map.org/grid_data/download/81790686?include=energy
Ephb3	0.648	http://api.brain-map.org/grid_data/download/1492?include=energy
Abi3bp	0.647	http://api.brain-map.org/grid_data/download/71152276?include=energy
Kcnh7	0.647	http://api.brain-map.org/grid_data/download/70562103?include=energy
Synpr	0.647	http://api.brain-map.org/grid_data/download/1862?include=energy
AU015836	0.647	http://api.brain-map.org/grid_data/download/74359658?include=energy
Col10a1	0.647	http://api.brain-map.org/grid_data/download/74003411?include=energy
Tnni3k	0.647	http://api.brain-map.org/grid_data/download/74276886?include=energy
LOC544771	0.647	http://api.brain-map.org/grid_data/download/77620814?include=energy
Gimap7	0.647	http://api.brain-map.org/grid_data/download/69549172?include=energy
Zfp811	0.647	http://api.brain-map.org/grid_data/download/70528378?include=energy
Adam33	0.646	http://api.brain-map.org/grid_data/download/75147767?include=energy
Scp2	0.646	http://api.brain-map.org/grid_data/download/68300926?include=energy
Tbx21	0.646	http://api.brain-map.org/grid_data/download/71488852?include=energy
Fbxl14	0.646	http://api.brain-map.org/grid_data/download/532747?include=energy
Acsm4	0.646	http://api.brain-map.org/grid_data/download/68797512?include=energy
Tmprss11f	0.646	http://api.brain-map.org/grid_data/download/70205346?include=energy
Fpr-rs3	0.646	http://api.brain-map.org/grid_data/download/70723393?include=energy
Gm5478	0.646	http://api.brain-map.org/grid_data/download/70546144?include=energy
Cort	0.646	http://api.brain-map.org/grid_data/download/72472766?include=energy
LOC432907	0.646	http://api.brain-map.org/grid_data/download/74509554?include=energy
4931431C16Rik	0.645	http://api.brain-map.org/grid_data/download/70218492?include=energy
Rab44	0.645	http://api.brain-map.org/grid_data/download/72283522?include=energy
Abhd5	0.645	http://api.brain-map.org/grid_data/download/686187?include=energy
Nrip1	0.645	http://api.brain-map.org/grid_data/download/739?include=energy
Saal1	0.645	http://api.brain-map.org/grid_data/download/73513308?include=energy
Lgr4	0.645	http://api.brain-map.org/grid_data/download/71307382?include=energy
Pm20d2	0.645	http://api.brain-map.org/grid_data/download/74359742?include=energy
Hmgb3	0.644	http://api.brain-map.org/grid_data/download/564752?include=energy

Timd4	0.644	http://api.brain-map.org/grid_data/download/68523213?include=energy
2010111I01Rik	0.644	http://api.brain-map.org/grid_data/download/71717630?include=energy
Pramel1	0.644	http://api.brain-map.org/grid_data/download/73423990?include=energy
Prkaa2	0.644	http://api.brain-map.org/grid_data/download/71212129?include=energy
Uchl5	0.644	http://api.brain-map.org/grid_data/download/413774?include=energy
Mfap3	0.644	http://api.brain-map.org/grid_data/download/386025?include=energy
Foxa3	0.644	http://api.brain-map.org/grid_data/download/80514161?include=energy
Elmo2	0.643	http://api.brain-map.org/grid_data/download/71015802?include=energy
Wnt7b	0.643	http://api.brain-map.org/grid_data/download/1113?include=energy
Tmem241	0.643	http://api.brain-map.org/grid_data/download/70227878?include=energy
Mael	0.643	http://api.brain-map.org/grid_data/download/69114543?include=energy
Camk1g	0.642	http://api.brain-map.org/grid_data/download/70743868?include=energy
Hspb3	0.642	http://api.brain-map.org/grid_data/download/73520988?include=energy
4930552P12Rik	0.642	http://api.brain-map.org/grid_data/download/70218354?include=energy
Adra1a	0.642	http://api.brain-map.org/grid_data/download/75042247?include=energy
Ptprk	0.642	http://api.brain-map.org/grid_data/download/74743303?include=energy
Mm.185993	0.642	http://api.brain-map.org/grid_data/download/74509548?include=energy
Adam19	0.641	http://api.brain-map.org/grid_data/download/74272054?include=energy
D830050J10Rik	0.641	http://api.brain-map.org/grid_data/download/74514019?include=energy
Syt6	0.641	http://api.brain-map.org/grid_data/download/1032?include=energy
Pxt1	0.641	http://api.brain-map.org/grid_data/download/70300971?include=energy
LOC433620	0.641	http://api.brain-map.org/grid_data/download/70785834?include=energy
LOC434010	0.641	http://api.brain-map.org/grid_data/download/70919077?include=energy
C920025C15Rik*	0.641	http://api.brain-map.org/grid_data/download/73811711?include=energy
Lysmd4	0.641	http://api.brain-map.org/grid_data/download/69014572?include=energy
Pdzrn3	0.64	http://api.brain-map.org/grid_data/download/71836787?include=energy
Prrg3	0.64	http://api.brain-map.org/grid_data/download/69672946?include=energy
Exoc6	0.64	http://api.brain-map.org/grid_data/download/73817986?include=energy
6430704M03Rik	0.64	http://api.brain-map.org/grid_data/download/74431765?include=energy
Duoxa2	0.64	http://api.brain-map.org/grid_data/download/68499280?include=energy
Yjefn3	0.64	http://api.brain-map.org/grid_data/download/74750064?include=energy
Efhd1	0.64	http://api.brain-map.org/grid_data/download/638709?include=energy
Slc52a3	0.64	http://api.brain-map.org/grid_data/download/634468?include=energy
Olfr211	0.64	http://api.brain-map.org/grid_data/download/74720904?include=energy
Fam5c	0.64	http://api.brain-map.org/grid_data/download/70927812?include=energy
Nol4	0.639	http://api.brain-map.org/grid_data/download/76115737?include=energy
Naa20	0.639	http://api.brain-map.org/grid_data/download/512770?include=energy
Tti2	0.639	http://api.brain-map.org/grid_data/download/587425?include=energy
Asb12	0.639	http://api.brain-map.org/grid_data/download/69816476?include=energy
Cldn19	0.639	http://api.brain-map.org/grid_data/download/69548988?include=energy
0610007P22Rik	0.639	http://api.brain-map.org/grid_data/download/68546094?include=energy
Hhat	0.639	http://api.brain-map.org/grid_data/download/68300833?include=energy
Zxdc	0.639	http://api.brain-map.org/grid_data/download/67978686?include=energy
Sirpb1a	0.638	http://api.brain-map.org/grid_data/download/72283528?include=energy
Fzd3	0.638	http://api.brain-map.org/grid_data/download/466?include=energy
Ankrd5	0.638	http://api.brain-map.org/grid_data/download/69816464?include=energy
Pax2	0.638	http://api.brain-map.org/grid_data/download/69816667?include=energy
Pla2g2d	0.637	http://api.brain-map.org/grid_data/download/69783268?include=energy
Olfr324	0.637	http://api.brain-map.org/grid_data/download/74720997?include=energy
Fam92a	0.637	http://api.brain-map.org/grid_data/download/587419?include=energy
Ccl27a	0.637	http://api.brain-map.org/grid_data/download/70474755?include=energy
Pvrl3	0.637	http://api.brain-map.org/grid_data/download/73521804?include=energy
Dact2	0.637	http://api.brain-map.org/grid_data/download/71064219?include=energy
Lancl2	0.637	http://api.brain-map.org/grid_data/download/638715?include=energy
B9d2	0.637	http://api.brain-map.org/grid_data/download/68522435?include=energy
Slc25a31	0.637	http://api.brain-map.org/grid_data/download/69114821?include=energy
A2m	0.637	http://api.brain-map.org/grid_data/download/100146086?include=energy
Epx	0.637	http://api.brain-map.org/grid_data/download/68667521?include=energy
Tbxa2r	0.637	http://api.brain-map.org/grid_data/download/69288363?include=energy
4930512B01Rik	0.636	http://api.brain-map.org/grid_data/download/70218211?include=energy
Pofut1	0.636	http://api.brain-map.org/grid_data/download/71209859?include=energy
A130029G22Rik*	0.636	http://api.brain-map.org/grid_data/download/73808504?include=energy
4930569F06Rik	0.636	http://api.brain-map.org/grid_data/download/74265165?include=energy
Prss52	0.636	http://api.brain-map.org/grid_data/download/69113734?include=energy
Gucy1b2	0.636	http://api.brain-map.org/grid_data/download/69734839?include=energy
Vwa3b	0.636	http://api.brain-map.org/grid_data/download/70525781?include=energy
Krtap13-1	0.636	http://api.brain-map.org/grid_data/download/68744767?include=energy
Kcnj6	0.636	http://api.brain-map.org/grid_data/download/609?include=energy
BC023105	0.635	http://api.brain-map.org/grid_data/download/67787348?include=energy
LOC546247	0.635	http://api.brain-map.org/grid_data/download/74431819?include=energy
Il17b	0.635	http://api.brain-map.org/grid_data/download/632511?include=energy
Rtn4r	0.635	http://api.brain-map.org/grid_data/download/72080145?include=energy
Fermt3	0.635	http://api.brain-map.org/grid_data/download/69549181?include=energy
LOC433335	0.635	http://api.brain-map.org/grid_data/download/70723110?include=energy
Dnajc22	0.635	http://api.brain-map.org/grid_data/download/68861723?include=energy
Ndufaf3	0.635	http://api.brain-map.org/grid_data/download/68545723?include=energy
E030026I10Rik	0.635	http://api.brain-map.org/grid_data/download/70238967?include=energy
Hoxc13	0.635	http://api.brain-map.org/grid_data/download/71377742?include=energy
BC048546	0.635	http://api.brain-map.org/grid_data/download/70524296?include=energy
1700041G16Rik	0.634	http://api.brain-map.org/grid_data/download/71378653?include=energy
Slc13a2	0.634	http://api.brain-map.org/grid_data/download/78534494?include=energy
Sstr4	0.634	http://api.brain-map.org/grid_data/download/73636037?include=energy
Ncor1	0.634	http://api.brain-map.org/grid_data/download/686?include=energy
Mtx1	0.634	http://api.brain-map.org/grid_data/download/68196942?include=energy
4930426L09Rik	0.634	http://api.brain-map.org/grid_data/download/74076186?include=energy
Nlrp9b	0.634	http://api.brain-map.org/grid_data/download/73635501?include=energy
Sdr16c6	0.633	http://api.brain-map.org/grid_data/download/72182951?include=energy
Cox4i2	0.633	http://api.brain-map.org/grid_data/download/69113680?include=energy
Zdhhc2	0.633	http://api.brain-map.org/grid_data/download/72128751?include=energy
Gm4952	0.632	http://api.brain-map.org/grid_data/download/71152300?include=energy
Tomm40	0.632	http://api.brain-map.org/grid_data/download/69549194?include=energy
5930412G12Rik	0.632	http://api.brain-map.org/grid_data/download/73767436?include=energy
Abl2	0.632	http://api.brain-map.org/grid_data/download/70228687?include=energy
B430203M17Rik	0.632	http://api.brain-map.org/grid_data/download/69527950?include=energy
Dcx	0.632	http://api.brain-map.org/grid_data/download/70946414?include=energy
LOC434392	0.632	http://api.brain-map.org/grid_data/download/71022274?include=energy
4732479B03Rik*	0.632	http://api.brain-map.org/grid_data/download/73808252?include=energy
3110035E14Rik	0.631	http://api.brain-map.org/grid_data/download/73817427?include=energy
Wfdc5	0.631	http://api.brain-map.org/grid_data/download/74058649?include=energy
Fhl2	0.631	http://api.brain-map.org/grid_data/download/70743903?include=energy
Dnajc19	0.631	http://api.brain-map.org/grid_data/download/70474881?include=energy
Trpm8	0.631	http://api.brain-map.org/grid_data/download/69863284?include=energy
Rbms2	0.631	http://api.brain-map.org/grid_data/download/513016?include=energy
Htr3a	0.63	http://api.brain-map.org/grid_data/download/74724760?include=energy
Endog	0.63	http://api.brain-map.org/grid_data/download/71992025?include=energy
Bhmt	0.63	http://api.brain-map.org/grid_data/download/68162239?include=energy
Eya4	0.63	http://api.brain-map.org/grid_data/download/77280436?include=energy
Gata4	0.63	http://api.brain-map.org/grid_data/download/69289027?include=energy
Cetn3	0.63	http://api.brain-map.org/grid_data/download/68076549?include=energy
Med9	0.63	http://api.brain-map.org/grid_data/download/71487046?include=energy
6530402F18Rik	0.63	http://api.brain-map.org/grid_data/download/74265351?include=energy
Wars2	0.63	http://api.brain-map.org/grid_data/download/70194952?include=energy
Olfr1299	0.63	http://api.brain-map.org/grid_data/download/73635568?include=energy
Fscn3	0.629	http://api.brain-map.org/grid_data/download/69114561?include=energy
Spata2	0.629	http://api.brain-map.org/grid_data/download/68196913?include=energy
Gcm1	0.629	http://api.brain-map.org/grid_data/download/69289045?include=energy
Phf20l1	0.629	http://api.brain-map.org/grid_data/download/69288908?include=energy
Impg2	0.629	http://api.brain-map.org/grid_data/download/74819536?include=energy
Diexf	0.629	http://api.brain-map.org/grid_data/download/545120?include=energy
Gm16486	0.628	http://api.brain-map.org/grid_data/download/72283366?include=energy
Lama3	0.628	http://api.brain-map.org/grid_data/download/71232927?include=energy
BC006965	0.628	http://api.brain-map.org/grid_data/download/68269296?include=energy
Gmcl1l	0.628	http://api.brain-map.org/grid_data/download/70201295?include=energy
Taf5l	0.627	http://api.brain-map.org/grid_data/download/686215?include=energy
Slc16a8	0.627	http://api.brain-map.org/grid_data/download/74431453?include=energy
LOC433884	0.627	http://api.brain-map.org/grid_data/download/70787065?include=energy
Ttll6	0.627	http://api.brain-map.org/grid_data/download/74363338?include=energy
Gm885	0.627	http://api.brain-map.org/grid_data/download/72471815?include=energy
Acap1	0.626	http://api.brain-map.org/grid_data/download/68162225?include=energy
Tnik	0.626	http://api.brain-map.org/grid_data/download/74431792?include=energy
Trim6	0.626	http://api.brain-map.org/grid_data/download/68795464?include=energy
4930466F19Rik	0.626	http://api.brain-map.org/grid_data/download/74076222?include=energy
Mafb	0.626	http://api.brain-map.org/grid_data/download/73771240?include=energy
Mtmr12	0.626	http://api.brain-map.org/grid_data/download/70431435?include=energy
9630028B13Rik	0.626	http://api.brain-map.org/grid_data/download/69447798?include=energy
Igfbp6	0.626	http://api.brain-map.org/grid_data/download/70928248?include=energy
Kcnh3	0.626	http://api.brain-map.org/grid_data/download/74641310?include=energy
TC1548724	0.626	http://api.brain-map.org/grid_data/download/70919035?include=energy
C330018D20Rik	0.626	http://api.brain-map.org/grid_data/download/69530983?include=energy
Pum1	0.626	http://api.brain-map.org/grid_data/download/73424038?include=energy
Dnajc14	0.626	http://api.brain-map.org/grid_data/download/564785?include=energy
4921511C20Rik	0.625	http://api.brain-map.org/grid_data/download/70723688?include=energy
9930021J03Rik	0.625	http://api.brain-map.org/grid_data/download/70717418?include=energy
Zfp874b	0.625	http://api.brain-map.org/grid_data/download/70724499?include=energy
Napepld	0.625	http://api.brain-map.org/grid_data/download/70300294?include=energy
Ftcd	0.625	http://api.brain-map.org/grid_data/download/68321892?include=energy
Senp1	0.625	http://api.brain-map.org/grid_data/download/68196948?include=energy
Hoxd13	0.625	http://api.brain-map.org/grid_data/download/71393400?include=energy
LOC433610	0.624	http://api.brain-map.org/grid_data/download/70785816?include=energy
Prpf31	0.624	http://api.brain-map.org/grid_data/download/564748?include=energy
Lcn13	0.624	http://api.brain-map.org/grid_data/download/68545569?include=energy
Glb1l	0.624	http://api.brain-map.org/grid_data/download/74076144?include=energy
Mal2	0.624	http://api.brain-map.org/grid_data/download/73994717?include=energy
LOC386468	0.624	http://api.brain-map.org/grid_data/download/70608421?include=energy
Cox7a1	0.624	http://api.brain-map.org/grid_data/download/74509440?include=energy
Coro1a	0.624	http://api.brain-map.org/grid_data/download/67978734?include=energy
Ddx3y	0.624	http://api.brain-map.org/grid_data/download/70528587?include=energy
LOC546959	0.624	http://api.brain-map.org/grid_data/download/74736504?include=energy
Ntn4	0.623	http://api.brain-map.org/grid_data/download/69782443?include=energy
6820433M10Rik*	0.623	http://api.brain-map.org/grid_data/download/73927637?include=energy
4932422M17Rik	0.622	http://api.brain-map.org/grid_data/download/74265237?include=energy
Zdhhc23	0.622	http://api.brain-map.org/grid_data/download/74363345?include=energy
Exph5	0.622	http://api.brain-map.org/grid_data/download/75080619?include=energy
Ier5	0.622	http://api.brain-map.org/grid_data/download/73931410?include=energy
Gm1118	0.622	http://api.brain-map.org/grid_data/download/74514249?include=energy
Hal	0.622	http://api.brain-map.org/grid_data/download/69014614?include=energy
4930564D02Rik	0.622	http://api.brain-map.org/grid_data/download/74265177?include=energy
2310057J18Rik	0.622	http://api.brain-map.org/grid_data/download/68342129?include=energy
Fat4	0.621	http://api.brain-map.org/grid_data/download/74363356?include=energy
4930483J18Rik	0.621	http://api.brain-map.org/grid_data/download/71892149?include=energy
1600014C10Rik	0.621	http://api.brain-map.org/grid_data/download/564749?include=energy
Tnrc6c	0.621	http://api.brain-map.org/grid_data/download/71487001?include=energy
Olfr598	0.62	http://api.brain-map.org/grid_data/download/74724229?include=energy
Ing2	0.62	http://api.brain-map.org/grid_data/download/69289201?include=energy
B3galnt1	0.62	http://api.brain-map.org/grid_data/download/68076555?include=energy
4930522N08Rik	0.619	http://api.brain-map.org/grid_data/download/74265141?include=energy
Epor	0.619	http://api.brain-map.org/grid_data/download/74822934?include=energy
Chd1	0.619	http://api.brain-map.org/grid_data/download/69548897?include=energy
Pak7	0.619	http://api.brain-map.org/grid_data/download/75988567?include=energy
Olfr829	0.619	http://api.brain-map.org/grid_data/download/74734956?include=energy
B3gnt3	0.619	http://api.brain-map.org/grid_data/download/67815957?include=energy
Orc6	0.619	http://api.brain-map.org/grid_data/download/69782575?include=energy
Eif4ebp1	0.619	http://api.brain-map.org/grid_data/download/68191444?include=energy
Hsd3b2	0.618	http://api.brain-map.org/grid_data/download/68445018?include=energy
Sult2b1	0.618	http://api.brain-map.org/grid_data/download/71763894?include=energy
Slc9a9	0.618	http://api.brain-map.org/grid_data/download/71670730?include=energy
Smpx	0.618	http://api.brain-map.org/grid_data/download/72002024?include=energy
LOC381557	0.618	http://api.brain-map.org/grid_data/download/71670744?include=energy
Bet1	0.618	http://api.brain-map.org/grid_data/download/68300932?include=energy
Mybpc2	0.617	http://api.brain-map.org/grid_data/download/67787360?include=energy
Fam198b	0.617	http://api.brain-map.org/grid_data/download/70523728?include=energy
E430033C13Rik*	0.617	http://api.brain-map.org/grid_data/download/74300765?include=energy
LOC436032	0.617	http://api.brain-map.org/grid_data/download/71022645?include=energy
Art2a-ps	0.617	http://api.brain-map.org/grid_data/download/71892370?include=energy
LOC434441	0.617	http://api.brain-map.org/grid_data/download/71022349?include=energy
AW112010	0.616	http://api.brain-map.org/grid_data/download/73767256?include=energy
Zdhhc18	0.616	http://api.brain-map.org/grid_data/download/74822874?include=energy
Trhr	0.616	http://api.brain-map.org/grid_data/download/1303?include=energy
Golga4	0.616	http://api.brain-map.org/grid_data/download/517?include=energy
Oas1c	0.616	http://api.brain-map.org/grid_data/download/68667407?include=energy
Slc6a5	0.616	http://api.brain-map.org/grid_data/download/69874024?include=energy
Xrcc2	0.616	http://api.brain-map.org/grid_data/download/68157394?include=energy
Esr1	0.615	http://api.brain-map.org/grid_data/download/79591677?include=energy
2210409E12Rik	0.615	http://api.brain-map.org/grid_data/download/71991714?include=energy
Esyt3	0.615	http://api.brain-map.org/grid_data/download/72472780?include=energy
Slc4a7	0.615	http://api.brain-map.org/grid_data/download/74431576?include=energy
Pdk1	0.614	http://api.brain-map.org/grid_data/download/70437846?include=energy
Arhgap15	0.614	http://api.brain-map.org/grid_data/download/68545729?include=energy
Sytl2	0.614	http://api.brain-map.org/grid_data/download/73520979?include=energy
Gsdma	0.614	http://api.brain-map.org/grid_data/download/70276969?include=energy
Gda	0.614	http://api.brain-map.org/grid_data/download/74047444?include=energy
Tas2r113	0.614	http://api.brain-map.org/grid_data/download/70302336?include=energy
LOC432506	0.613	http://api.brain-map.org/grid_data/download/74822922?include=energy
Slamf6	0.613	http://api.brain-map.org/grid_data/download/68544985?include=energy
Tnnc1	0.612	http://api.brain-map.org/grid_data/download/73817440?include=energy
Pamr1	0.612	http://api.brain-map.org/grid_data/download/70744322?include=energy
AA987161	0.612	http://api.brain-map.org/grid_data/download/70228675?include=energy
Dio3	0.612	http://api.brain-map.org/grid_data/download/71717188?include=energy
Ptgs2	0.612	http://api.brain-map.org/grid_data/download/71016664?include=energy
Trem3	0.612	http://api.brain-map.org/grid_data/download/70302605?include=energy
Smoc2	0.611	http://api.brain-map.org/grid_data/download/74357576?include=energy
Upp2	0.611	http://api.brain-map.org/grid_data/download/68445024?include=energy
D630023F18Rik	0.61	http://api.brain-map.org/grid_data/download/69609007?include=energy
Pcsk1	0.61	http://api.brain-map.org/grid_data/download/72008494?include=energy
Pitpnm2	0.61	http://api.brain-map.org/grid_data/download/73997148?include=energy
Naa38	0.61	http://api.brain-map.org/grid_data/download/587430?include=energy
Chchd6	0.61	http://api.brain-map.org/grid_data/download/67979019?include=energy
Slc26a10	0.61	http://api.brain-map.org/grid_data/download/74273116?include=energy
Vipr1	0.61	http://api.brain-map.org/grid_data/download/73927619?include=energy
Nfe2l2	0.609	http://api.brain-map.org/grid_data/download/700?include=energy
4933426D04Rik	0.609	http://api.brain-map.org/grid_data/download/74265267?include=energy
Rnf2	0.609	http://api.brain-map.org/grid_data/download/68341878?include=energy
Ppm1j	0.608	http://api.brain-map.org/grid_data/download/71280868?include=energy
Mmp21	0.608	http://api.brain-map.org/grid_data/download/73423847?include=energy
Rnf19a	0.608	http://api.brain-map.org/grid_data/download/73931414?include=energy
Arl15	0.608	http://api.brain-map.org/grid_data/download/74363341?include=energy
Nov	0.608	http://api.brain-map.org/grid_data/download/69120620?include=energy
Prl2a1	0.608	http://api.brain-map.org/grid_data/download/71919236?include=energy
Ecm2	0.607	http://api.brain-map.org/grid_data/download/70719031?include=energy
Ahsp	0.607	http://api.brain-map.org/grid_data/download/70239129?include=energy
Asb10	0.607	http://api.brain-map.org/grid_data/download/69816470?include=energy
Tmem198	0.607	http://api.brain-map.org/grid_data/download/68845305?include=energy
Metrnl	0.606	http://api.brain-map.org/grid_data/download/68633363?include=energy
Ppp1r14d	0.606	http://api.brain-map.org/grid_data/download/68499320?include=energy
5430411C19Rik	0.605	http://api.brain-map.org/grid_data/download/74265285?include=energy
Slco6c1	0.605	http://api.brain-map.org/grid_data/download/70302198?include=energy
Mef2c	0.605	http://api.brain-map.org/grid_data/download/667?include=energy
Ahsg	0.605	http://api.brain-map.org/grid_data/download/68301306?include=energy
Grm2	0.605	http://api.brain-map.org/grid_data/download/79591611?include=energy
Col23a1	0.605	http://api.brain-map.org/grid_data/download/73636092?include=energy
3110099E13Rik*	0.604	http://api.brain-map.org/grid_data/download/74359610?include=energy
Nxf7	0.604	http://api.brain-map.org/grid_data/download/75988557?include=energy
F830045P16Rik	0.604	http://api.brain-map.org/grid_data/download/70276801?include=energy
Ly6g6e	0.603	http://api.brain-map.org/grid_data/download/77280582?include=energy
Fbxo30	0.603	http://api.brain-map.org/grid_data/download/68162228?include=energy
BC039966	0.602	http://api.brain-map.org/grid_data/download/73513564?include=energy
Matn2	0.602	http://api.brain-map.org/grid_data/download/73817421?include=energy
Kcnd1	0.601	http://api.brain-map.org/grid_data/download/74300867?include=energy
Slurp1	0.601	http://api.brain-map.org/grid_data/download/70598376?include=energy
Slc12a1	0.601	http://api.brain-map.org/grid_data/download/78534498?include=energy
4930517O19Rik	0.6	http://api.brain-map.org/grid_data/download/74076246?include=energy
Dctpp1	0.6	http://api.brain-map.org/grid_data/download/393254?include=energy
Gm1604b	0.6	http://api.brain-map.org/grid_data/download/70298471?include=energy
BC048671	0.599	http://api.brain-map.org/grid_data/download/70528360?include=energy
Rep15	0.599	http://api.brain-map.org/grid_data/download/68499249?include=energy
Tas2r119	0.599	http://api.brain-map.org/grid_data/download/70302354?include=energy
4933427G17Rik	0.598	http://api.brain-map.org/grid_data/download/70218810?include=energy

A130010J15Rik	0.598	http://api.brain-map.org/grid_data/download/69014560?include=energy
Cav2	0.597	http://api.brain-map.org/grid_data/download/68444620?include=energy
Doc2g	0.597	http://api.brain-map.org/grid_data/download/74881341?include=energy
Cecr6	0.597	http://api.brain-map.org/grid_data/download/69288854?include=energy
Mybpc1	0.596	http://api.brain-map.org/grid_data/download/71920513?include=energy
Phxr4	0.596	http://api.brain-map.org/grid_data/download/69783124?include=energy
Psma8	0.596	http://api.brain-map.org/grid_data/download/75985879?include=energy
Olfr1509	0.596	http://api.brain-map.org/grid_data/download/74720880?include=energy
B130006D01Rik	0.596	http://api.brain-map.org/grid_data/download/71212578?include=energy
Ppp2r5a	0.595	http://api.brain-map.org/grid_data/download/68148738?include=energy
Rasl11b	0.595	http://api.brain-map.org/grid_data/download/71717137?include=energy
Abp1	0.595	http://api.brain-map.org/grid_data/download/80341197?include=energy
6720475J19Rik	0.595	http://api.brain-map.org/grid_data/download/71023026?include=energy
Ccdc42	0.594	http://api.brain-map.org/grid_data/download/70228466?include=energy
LOC433898	0.594	http://api.brain-map.org/grid_data/download/70787077?include=energy
Satb2	0.594	http://api.brain-map.org/grid_data/download/73992708?include=energy
Gk5	0.594	http://api.brain-map.org/grid_data/download/69530989?include=energy
A930031F24Rik*	0.594	http://api.brain-map.org/grid_data/download/73808349?include=energy
Npr3	0.594	http://api.brain-map.org/grid_data/download/73931412?include=energy
Zcchc13	0.593	http://api.brain-map.org/grid_data/download/75197690?include=energy
Tcp11l2	0.592	http://api.brain-map.org/grid_data/download/68196911?include=energy
Polr2d	0.59	http://api.brain-map.org/grid_data/download/413780?include=energy
BC125332	0.589	http://api.brain-map.org/grid_data/download/71210571?include=energy
Slc6a14	0.589	http://api.brain-map.org/grid_data/download/70302174?include=energy
Stra13	0.589	http://api.brain-map.org/grid_data/download/71992055?include=energy
2900026A02Rik	0.588	http://api.brain-map.org/grid_data/download/71670713?include=energy
Prss36	0.588	http://api.brain-map.org/grid_data/download/69530959?include=energy
4930519G04Rik	0.588	http://api.brain-map.org/grid_data/download/69442308?include=energy
Acvr2a	0.588	http://api.brain-map.org/grid_data/download/74509464?include=energy
Vmn1r206	0.588	http://api.brain-map.org/grid_data/download/69442266?include=energy
Tmem74	0.587	http://api.brain-map.org/grid_data/download/74881364?include=energy
Fbxw4	0.587	http://api.brain-map.org/grid_data/download/68441616?include=energy
Dfnb59	0.587	http://api.brain-map.org/grid_data/download/70298573?include=energy
Bmp3	0.586	http://api.brain-map.org/grid_data/download/72472749?include=energy
Smtnl1	0.586	http://api.brain-map.org/grid_data/download/68191498?include=energy
Gpbar1	0.585	http://api.brain-map.org/grid_data/download/69856893?include=energy
F630306D02Rik*	0.585	http://api.brain-map.org/grid_data/download/73748815?include=energy
Ube2l6	0.585	http://api.brain-map.org/grid_data/download/68269295?include=energy
Dsp	0.584	http://api.brain-map.org/grid_data/download/69201504?include=energy
LOC432741	0.584	http://api.brain-map.org/grid_data/download/71152521?include=energy
Taf9b	0.584	http://api.brain-map.org/grid_data/download/71380920?include=energy
H2afy3	0.583	http://api.brain-map.org/grid_data/download/70276981?include=energy
LOC436050	0.581	http://api.brain-map.org/grid_data/download/71022651?include=energy
Orc5	0.581	http://api.brain-map.org/grid_data/download/633289?include=energy
Tigd4	0.58	http://api.brain-map.org/grid_data/download/70302491?include=energy
Fam18b	0.58	http://api.brain-map.org/grid_data/download/69407128?include=energy
Bcdin3d	0.58	http://api.brain-map.org/grid_data/download/638736?include=energy
Lrrc55	0.579	http://api.brain-map.org/grid_data/download/73931406?include=energy
Ext2	0.579	http://api.brain-map.org/grid_data/download/392918?include=energy
Arhgap8	0.579	http://api.brain-map.org/grid_data/download/633299?include=energy
Tmem215	0.579	http://api.brain-map.org/grid_data/download/73636150?include=energy
Gm1747	0.579	http://api.brain-map.org/grid_data/download/70608415?include=energy
Gphb5	0.578	http://api.brain-map.org/grid_data/download/70276957?include=energy
Grhl1	0.578	http://api.brain-map.org/grid_data/download/68076900?include=energy
Bik	0.578	http://api.brain-map.org/grid_data/download/632496?include=energy
Sidt1	0.578	http://api.brain-map.org/grid_data/download/71587812?include=energy
Clec4a1	0.578	http://api.brain-map.org/grid_data/download/68797508?include=energy
Gnb4	0.577	http://api.brain-map.org/grid_data/download/74047771?include=energy
Npy1r	0.577	http://api.brain-map.org/grid_data/download/72103807?include=energy
Lipf	0.577	http://api.brain-map.org/grid_data/download/70295494?include=energy
Tctex1d2	0.576	http://api.brain-map.org/grid_data/download/71763945?include=energy
Tnip2	0.576	http://api.brain-map.org/grid_data/download/74425540?include=energy
Srrm3	0.576	http://api.brain-map.org/grid_data/download/74359503?include=energy
LOC545904	0.575	http://api.brain-map.org/grid_data/download/74277702?include=energy
Grxcr2	0.575	http://api.brain-map.org/grid_data/download/70298358?include=energy
Hoxa5	0.574	http://api.brain-map.org/grid_data/download/544903?include=energy
Siva1	0.574	http://api.brain-map.org/grid_data/download/71763882?include=energy
Sos2	0.573	http://api.brain-map.org/grid_data/download/71212488?include=energy
Odz3	0.572	http://api.brain-map.org/grid_data/download/752?include=energy
Lrrn4cl	0.572	http://api.brain-map.org/grid_data/download/80472029?include=energy
Zfpl1	0.571	http://api.brain-map.org/grid_data/download/68269056?include=energy
Zbtb16	0.57	http://api.brain-map.org/grid_data/download/71717125?include=energy
Nhedc1	0.57	http://api.brain-map.org/grid_data/download/72283474?include=energy
Gm850	0.567	http://api.brain-map.org/grid_data/download/70298352?include=energy
Slc43a1	0.566	http://api.brain-map.org/grid_data/download/68797906?include=energy
Mei1	0.564	http://api.brain-map.org/grid_data/download/70300300?include=energy
Fam45a	0.564	http://api.brain-map.org/grid_data/download/686212?include=energy
Tbc1d12	0.564	http://api.brain-map.org/grid_data/download/70474041?include=energy
Ltbp2	0.563	http://api.brain-map.org/grid_data/download/69736304?include=energy
Igkv5-39	0.563	http://api.brain-map.org/grid_data/download/70300109?include=energy
Hoxd12	0.563	http://api.brain-map.org/grid_data/download/69289177?include=energy
Lemd1	0.562	http://api.brain-map.org/grid_data/download/72283683?include=energy
Camp	0.562	http://api.brain-map.org/grid_data/download/69540321?include=energy
Tec	0.562	http://api.brain-map.org/grid_data/download/68162243?include=energy
Btbd19	0.562	http://api.brain-map.org/grid_data/download/72283516?include=energy
Eras	0.561	http://api.brain-map.org/grid_data/download/70239135?include=energy
4930579F01Rik	0.561	http://api.brain-map.org/grid_data/download/71892017?include=energy
Ankdd1b	0.559	http://api.brain-map.org/grid_data/download/70946201?include=energy
Fhod3	0.559	http://api.brain-map.org/grid_data/download/71924400?include=energy
Klk4	0.558	http://api.brain-map.org/grid_data/download/69735885?include=energy
2810055F11Rik	0.558	http://api.brain-map.org/grid_data/download/633679?include=energy
Pcdh20	0.556	http://api.brain-map.org/grid_data/download/71670738?include=energy
Rasa2	0.555	http://api.brain-map.org/grid_data/download/73512192?include=energy
Odf3l1	0.555	http://api.brain-map.org/grid_data/download/69113781?include=energy
Kcnb2	0.554	http://api.brain-map.org/grid_data/download/74581424?include=energy
Prpsap1	0.554	http://api.brain-map.org/grid_data/download/73424023?include=energy
1700031A10Rik	0.553	http://api.brain-map.org/grid_data/download/73512570?include=energy
4930449A18Rik	0.552	http://api.brain-map.org/grid_data/download/71891713?include=energy
Clec18a	0.552	http://api.brain-map.org/grid_data/download/74562498?include=energy
2610206C17Rik	0.549	http://api.brain-map.org/grid_data/download/70203537?include=energy
Fst	0.549	http://api.brain-map.org/grid_data/download/72283811?include=energy
Iqcf1	0.548	http://api.brain-map.org/grid_data/download/70474875?include=energy
Magea9-ps	0.548	http://api.brain-map.org/grid_data/download/70300258?include=energy
Myoz1	0.547	http://api.brain-map.org/grid_data/download/67792213?include=energy
Chrd	0.547	http://api.brain-map.org/grid_data/download/257?include=energy
Trpc6	0.545	http://api.brain-map.org/grid_data/download/70562126?include=energy
Mzf1	0.544	http://api.brain-map.org/grid_data/download/74431372?include=energy
Tmem14a	0.544	http://api.brain-map.org/grid_data/download/77414600?include=energy
Slc46a1	0.544	http://api.brain-map.org/grid_data/download/69028372?include=energy
Cnpy1	0.544	http://api.brain-map.org/grid_data/download/70228209?include=energy
C330007P06Rik	0.543	http://api.brain-map.org/grid_data/download/68196967?include=energy
Lrrc33	0.542	http://api.brain-map.org/grid_data/download/70528623?include=energy
Hars2	0.542	http://api.brain-map.org/grid_data/download/633681?include=energy
9430029A11Rik	0.542	http://api.brain-map.org/grid_data/download/70228137?include=energy
Kctd4	0.54	http://api.brain-map.org/grid_data/download/71063818?include=energy
4930566N20Rik	0.54	http://api.brain-map.org/grid_data/download/74265201?include=energy
Cln8	0.54	http://api.brain-map.org/grid_data/download/70429196?include=energy
Psrc1	0.54	http://api.brain-map.org/grid_data/download/71015388?include=energy
Slc39a6	0.538	http://api.brain-map.org/grid_data/download/73930852?include=energy
1110020C17Rik	0.537	http://api.brain-map.org/grid_data/download/74300861?include=energy
C330011F03	0.534	http://api.brain-map.org/grid_data/download/74431582?include=energy
Rg9mtd2	0.533	http://api.brain-map.org/grid_data/download/70301019?include=energy
Apool	0.532	http://api.brain-map.org/grid_data/download/70528145?include=energy
Nmb	0.531	http://api.brain-map.org/grid_data/download/71836874?include=energy
Stard8	0.531	http://api.brain-map.org/grid_data/download/73521808?include=energy
Sdr42e1	0.531	http://api.brain-map.org/grid_data/download/68162232?include=energy
Gtdc1	0.531	http://api.brain-map.org/grid_data/download/74425541?include=energy
Fam102b	0.531	http://api.brain-map.org/grid_data/download/73520819?include=energy
Olfr1087	0.526	http://api.brain-map.org/grid_data/download/73635508?include=energy
Frmd6	0.526	http://api.brain-map.org/grid_data/download/73931382?include=energy
Slc22a14	0.526	http://api.brain-map.org/grid_data/download/71832787?include=energy
2310069G16Rik	0.525	http://api.brain-map.org/grid_data/download/71212404?include=energy
Rgsl1	0.524	http://api.brain-map.org/grid_data/download/73424177?include=energy
Hebp2	0.522	http://api.brain-map.org/grid_data/download/386227?include=energy
Dnahc11	0.52	http://api.brain-map.org/grid_data/download/73520976?include=energy
Islr	0.52	http://api.brain-map.org/grid_data/download/68300827?include=energy
4930578I07Rik	0.519	http://api.brain-map.org/grid_data/download/74265195?include=energy
Lgr6	0.519	http://api.brain-map.org/grid_data/download/71488750?include=energy
Kank3	0.518	http://api.brain-map.org/grid_data/download/69838595?include=energy
Myl4	0.518	http://api.brain-map.org/grid_data/download/72129251?include=energy
Ppp3cc	0.516	http://api.brain-map.org/grid_data/download/70784994?include=energy
Abcc6	0.511	http://api.brain-map.org/grid_data/download/69514612?include=energy
Synb	0.511	http://api.brain-map.org/grid_data/download/70238812?include=energy
Gsc2	0.51	http://api.brain-map.org/grid_data/download/81790664?include=energy
Rspo2	0.509	http://api.brain-map.org/grid_data/download/71016632?include=energy
Tdo2	0.509	http://api.brain-map.org/grid_data/download/71717618?include=energy
Glis3	0.507	http://api.brain-map.org/grid_data/download/71587733?include=energy
Cyp7b1	0.502	http://api.brain-map.org/grid_data/download/70927813?include=energy
Clvs2	0.489	http://api.brain-map.org/grid_data/download/71924374?include=energy
4930597L12Rik	0.488	http://api.brain-map.org/grid_data/download/74265171?include=energy
Trdn	0.487	http://api.brain-map.org/grid_data/download/70302593?include=energy
Efha1	0.485	http://api.brain-map.org/grid_data/download/1946?include=energy
Dppa2	0.484	http://api.brain-map.org/grid_data/download/70724667?include=energy
Atp10a	0.482	http://api.brain-map.org/grid_data/download/72128790?include=energy
Sp7	0.475	http://api.brain-map.org/grid_data/download/71488813?include=energy
Hcn3	0.473	http://api.brain-map.org/grid_data/download/71393352?include=energy
Shisa3	0.472	http://api.brain-map.org/grid_data/download/70238734?include=energy
4933425L06Rik	0.471	http://api.brain-map.org/grid_data/download/70218798?include=energy
Adam8	0.464	http://api.brain-map.org/grid_data/download/68498992?include=energy
Sall1	0.462	http://api.brain-map.org/grid_data/download/69290429?include=energy
Jam2	0.461	http://api.brain-map.org/grid_data/download/602?include=energy
Prdm5	0.455	http://api.brain-map.org/grid_data/download/69289757?include=energy
Lct	0.451	http://api.brain-map.org/grid_data/download/73931621?include=energy
Cpa6	0.403	http://api.brain-map.org/grid_data/download/75197606?include=energy
Tubb6	0.397	http://api.brain-map.org/grid_data/download/68191570?include=energy
Map3k1	0.394	http://api.brain-map.org/grid_data/download/69752004?include=energy
Dpp6	0.388	http://api.brain-map.org/grid_data/download/73520981?include=energy
Myo16	0.333	http://api.brain-map.org/grid_data/download/73520969?include=energy
Otop2	0.324	http://api.brain-map.org/grid_data/download/71210635?include=energy
Crlf1	0.309	http://api.brain-map.org/grid_data/download/72283808?include=energy
Gcnt1	0.293	http://api.brain-map.org/grid_data/download/68546476?include=energy
Prox1	0.245	http://api.brain-map.org/grid_data/download/73520980?include=energy
Ush1g	0.213	http://api.brain-map.org/grid_data/download/70302926?include=energy

Supplementary Table 6: Clusters of MS-associated gene expression throughout neurodevelopment of brain regions affected by immune dysfunction.

Gene	Age	Z-score	Cluster	URL
Adcyap1	p04	1.367	1	http://api.brain-map.org/grid_data/download/100056232?include=energy
Adnp	p04	0.970	1	http://api.brain-map.org/grid_data/download/100081607?include=energy
Adra2b	p04	1.090	1	http://api.brain-map.org/grid_data/download/100056127?include=energy
Ahi1	p04	1.203	1	http://api.brain-map.org/grid_data/download/100081430?include=energy
Alcam	p04	0.601	1	http://api.brain-map.org/grid_data/download/100084414?include=energy
Angpt2	p04	1.199	1	http://api.brain-map.org/grid_data/download/100092225?include=energy
Angptl4	p04	1.499	1	http://api.brain-map.org/grid_data/download/100072198?include=energy
Apc	p04	0.495	1	http://api.brain-map.org/grid_data/download/100091889?include=energy
Axl	p04	0.894	1	http://api.brain-map.org/grid_data/download/100056010?include=energy
Bmpr2	p04	0.923	1	http://api.brain-map.org/grid_data/download/100057296?include=energy
Cacna1h	p04	1.400	1	http://api.brain-map.org/grid_data/download/100092168?include=energy
Casp3	p04	1.359	1	http://api.brain-map.org/grid_data/download/100084424?include=energy
Cdh1	p04	1.497	1	http://api.brain-map.org/grid_data/download/100057286?include=energy
Cntn1	p04	0.915	1	http://api.brain-map.org/grid_data/download/100091878?include=energy
Cxcl12	p04	1.073	1	http://api.brain-map.org/grid_data/download/100084454?include=energy
Cxcr4	p04	0.772	1	http://api.brain-map.org/grid_data/download/100084355?include=energy
Ddr1	p04	0.727	1	http://api.brain-map.org/grid_data/download/100057305?include=energy
Ephb2	p04	1.466	1	http://api.brain-map.org/grid_data/download/100057311?include=energy
Fbxw4	p04	1.311	1	http://api.brain-map.org/grid_data/download/100055996?include=energy
Fgf23	p04	1.292	1	http://api.brain-map.org/grid_data/download/100074941?include=energy
Fmr1	p04	1.015	1	http://api.brain-map.org/grid_data/download/100072184?include=energy
Fn1	p04	0.598	1	http://api.brain-map.org/grid_data/download/100056091?include=energy
Fyn	p04	1.257	1	http://api.brain-map.org/grid_data/download/100079217?include=energy
Gap43	p04	0.874	1	http://api.brain-map.org/grid_data/download/100054842?include=energy
Gata3	p04	1.457	1	http://api.brain-map.org/grid_data/download/100078485?include=energy
Ghsr	p04	0.944	1	http://api.brain-map.org/grid_data/download/100056136?include=energy
Gria2	p04	0.762	1	http://api.brain-map.org/grid_data/download/100056084?include=energy
Grm3	p04	1.455	1	http://api.brain-map.org/grid_data/download/100074223?include=energy
Grm8	p04	1.149	1	http://api.brain-map.org/grid_data/download/100056121?include=energy
Hfe	p04	1.314	1	http://api.brain-map.org/grid_data/download/100084418?include=energy
Hrh1	p04	1.493	1	http://api.brain-map.org/grid_data/download/100056341?include=energy
Mc4r	p04	1.299	1	http://api.brain-map.org/grid_data/download/100073395?include=energy
Ncam1	p04	1.371	1	http://api.brain-map.org/grid_data/download/100072208?include=energy
Nfkbia	p04	1.339	1	http://api.brain-map.org/grid_data/download/100057360?include=energy
Nfya	p04	0.996	1	http://api.brain-map.org/grid_data/download/100077680?include=energy
Nos1	p04	1.282	1	http://api.brain-map.org/grid_data/download/100057322?include=energy
Notch3	p04	1.278	1	http://api.brain-map.org/grid_data/download/100073389?include=energy
Npy	p04	1.418	1	http://api.brain-map.org/grid_data/download/100073378?include=energy
Nr4a3	p04	1.467	1	http://api.brain-map.org/grid_data/download/100077675?include=energy
Nrxn1	p04	1.079	1	http://api.brain-map.org/grid_data/download/100056069?include=energy
Ntf3	p04	1.499	1	http://api.brain-map.org/grid_data/download/100056619?include=energy
Ntn1	p04	1.455	1	http://api.brain-map.org/grid_data/download/100057341?include=energy
Plxna2	p04	1.086	1	http://api.brain-map.org/grid_data/download/100092169?include=energy
Plxna3	p04	1.370	1	http://api.brain-map.org/grid_data/download/100074188?include=energy
Polr1d	p04	0.550	1	http://api.brain-map.org/grid_data/download/161149818?include=energy
Rara	p04	0.943	1	http://api.brain-map.org/grid_data/download/100079223?include=energy
Reln	p04	1.165	1	http://api.brain-map.org/grid_data/download/100073399?include=energy
Rtn4r	p04	1.067	1	http://api.brain-map.org/grid_data/download/100056070?include=energy
Sema3a	p04	0.659	1	http://api.brain-map.org/grid_data/download/100072228?include=energy
Sema4a	p04	0.975	1	http://api.brain-map.org/grid_data/download/100056000?include=energy
Slc1a3	p04	1.392	1	http://api.brain-map.org/grid_data/download/100084400?include=energy
Slc1a4	p04	1.496	1	http://api.brain-map.org/grid_data/download/100084403?include=energy
Slc6a4	p04	1.280	1	http://api.brain-map.org/grid_data/download/100056202?include=energy
Sp3	p04	0.916	1	http://api.brain-map.org/grid_data/download/100074969?include=energy
Ssrp1	p04	0.960	1	http://api.brain-map.org/grid_data/download/100081393?include=energy
Stx1a	p04	0.630	1	http://api.brain-map.org/grid_data/download/100073372?include=energy
Trpv1	p04	1.202	1	http://api.brain-map.org/grid_data/download/100056185?include=energy
Vegfa	p04	0.901	1	http://api.brain-map.org/grid_data/download/100084416?include=energy
Ache	p04	-0.994	2	http://api.brain-map.org/grid_data/download/100020324?include=energy
Aff3	p04	-0.607	2	http://api.brain-map.org/grid_data/download/100075001?include=energy
Agt	p04	-0.005	2	http://api.brain-map.org/grid_data/download/100092244?include=energy
Amh	p04	-0.473	2	http://api.brain-map.org/grid_data/download/100056338?include=energy
App	p04	-0.362	2	http://api.brain-map.org/grid_data/download/100072246?include=energy
Aqp4	p04	-1.051	2	http://api.brain-map.org/grid_data/download/100054925?include=energy
Bach2	p04	-0.347	2	http://api.brain-map.org/grid_data/download/100076666?include=energy
Bmp2	p04	-0.561	2	http://api.brain-map.org/grid_data/download/100072192?include=energy
Bmp4	p04	-0.646	2	http://api.brain-map.org/grid_data/download/100054907?include=energy
C1qa	p04	0.416	2	http://api.brain-map.org/grid_data/download/100093000?include=energy
Cd44	p04	-0.823	2	http://api.brain-map.org/grid_data/download/100054861?include=energy
Cdh3	p04	-0.636	2	http://api.brain-map.org/grid_data/download/100077682?include=energy
Cic	p04	-0.053	2	http://api.brain-map.org/grid_data/download/100081625?include=energy
Cldn5	p04	-0.406	2	http://api.brain-map.org/grid_data/download/100092250?include=energy
Clock	p04	-0.478	2	http://api.brain-map.org/grid_data/download/100074203?include=energy
Clstn2	p04	-1.071	2	http://api.brain-map.org/grid_data/download/100091841?include=energy
Clu	p04	0.012	2	http://api.brain-map.org/grid_data/download/100073408?include=energy
Col11a2	p04	-0.793	2	http://api.brain-map.org/grid_data/download/100078870?include=energy
Col2a1	p04	-0.709	2	http://api.brain-map.org/grid_data/download/100077687?include=energy
Creb1	p04	-0.891	2	http://api.brain-map.org/grid_data/download/100054962?include=energy
Crhr1	p04	-1.122	2	http://api.brain-map.org/grid_data/download/100074479?include=energy
Csf1r	p04	0.168	2	http://api.brain-map.org/grid_data/download/100057280?include=energy
Ctnnb1	p04	0.002	2	http://api.brain-map.org/grid_data/download/100073374?include=energy
Cyp7b1	p04	-1.143	2	http://api.brain-map.org/grid_data/download/100081820?include=energy
Dbp	p04	-0.913	2	http://api.brain-map.org/grid_data/download/100078487?include=energy
Dll1	p04	-0.722	2	http://api.brain-map.org/grid_data/download/100032091?include=energy
Dll4	p04	-0.466	2	http://api.brain-map.org/grid_data/download/100056016?include=energy
Ebf1	p04	-0.173	2	http://api.brain-map.org/grid_data/download/100092103?include=energy
Ednrb	p04	-0.300	2	http://api.brain-map.org/grid_data/download/100021561?include=energy
Egf	p04	-0.598	2	http://api.brain-map.org/grid_data/download/100101332?include=energy
Egfr	p04	-0.460	2	http://api.brain-map.org/grid_data/download/100021570?include=energy
Epha4	p04	-1.053	2	http://api.brain-map.org/grid_data/download/100093835?include=energy
Erbb3	p04	-0.864	2	http://api.brain-map.org/grid_data/download/100072219?include=energy
Ets1	p04	0.489	2	http://api.brain-map.org/grid_data/download/100074708?include=energy
Fgf1	p04	-0.896	2	http://api.brain-map.org/grid_data/download/100091892?include=energy
Foxc1	p04	-1.059	2	http://api.brain-map.org/grid_data/download/100074278?include=energy
Foxo3	p04	-0.789	2	http://api.brain-map.org/grid_data/download/100101240?include=energy
Fzd4	p04	-0.852	2	http://api.brain-map.org/grid_data/download/100091931?include=energy
Gal	p04	0.034	2	http://api.brain-map.org/grid_data/download/100056067?include=energy
Gdf15	p04	-1.089	2	http://api.brain-map.org/grid_data/download/100072233?include=energy
Gfap	p04	-0.298	2	http://api.brain-map.org/grid_data/download/100073403?include=energy
Gja1	p04	-0.944	2	http://api.brain-map.org/grid_data/download/100054851?include=energy
Gli1	p04	-0.880	2	http://api.brain-map.org/grid_data/download/100059122?include=energy
Glis3	p04	-0.550	2	http://api.brain-map.org/grid_data/download/100093012?include=energy
Grm1	p04	-0.987	2	http://api.brain-map.org/grid_data/download/100072270?include=energy
Hcrt	p04	-0.529	2	http://api.brain-map.org/grid_data/download/100073410?include=energy
Hspb8	p04	-0.876	2	http://api.brain-map.org/grid_data/download/100055017?include=energy
Igf1	p04	-0.081	2	http://api.brain-map.org/grid_data/download/100072243?include=energy
Igfbp1	p04	-0.643	2	http://api.brain-map.org/grid_data/download/100092127?include=energy
Igfbp3	p04	0.410	2	http://api.brain-map.org/grid_data/download/100072209?include=energy
Ikzf1	p04	-0.642	2	http://api.brain-map.org/grid_data/download/100078557?include=energy
Insrr	p04	-1.131	2	http://api.brain-map.org/grid_data/download/100093113?include=energy
Irf1	p04	-0.991	2	http://api.brain-map.org/grid_data/download/100092128?include=energy
Itga4	p04	-0.559	2	http://api.brain-map.org/grid_data/download/100078406?include=energy
Itgam	p04	-0.515	2	http://api.brain-map.org/grid_data/download/100081850?include=energy
Jag1	p04	-0.390	2	http://api.brain-map.org/grid_data/download/100059159?include=energy
Jazf1	p04	-0.336	2	http://api.brain-map.org/grid_data/download/100075693?include=energy
Kdr	p04	-0.400	2	http://api.brain-map.org/grid_data/download/100054882?include=energy
Lgals1	p04	-0.968	2	http://api.brain-map.org/grid_data/download/100055177?include=energy
Lpl	p04	-0.481	2	http://api.brain-map.org/grid_data/download/100055156?include=energy
Mapk8	p04	-0.978	2	http://api.brain-map.org/grid_data/download/100054996?include=energy
Maz	p04	-1.120	2	http://api.brain-map.org/grid_data/download/100078523?include=energy
Mertk	p04	-0.857	2	http://api.brain-map.org/grid_data/download/100091918?include=energy
Mst1	p04	-0.846	2	http://api.brain-map.org/grid_data/download/100091926?include=energy
Mtor	p04	-1.188	2	http://api.brain-map.org/grid_data/download/100091856?include=energy
Mycn	p04	-0.015	2	http://api.brain-map.org/grid_data/download/100077648?include=energy
Myt1	p04	-0.756	2	http://api.brain-map.org/grid_data/download/100074985?include=energy
Nfe2l2	p04	-0.696	2	http://api.brain-map.org/grid_data/download/100075637?include=energy
Nfkb1	p04	-0.990	2	http://api.brain-map.org/grid_data/download/100075676?include=energy
Nog	p04	-0.712	2	http://api.brain-map.org/grid_data/download/100076644?include=energy
Notch2	p04	-0.262	2	http://api.brain-map.org/grid_data/download/100055011?include=energy
Notch4	p04	-0.974	2	http://api.brain-map.org/grid_data/download/100074263?include=energy
Nr1h3	p04	-0.597	2	http://api.brain-map.org/grid_data/download/100081834?include=energy
Nr3c2	p04	-0.394	2	http://api.brain-map.org/grid_data/download/100078561?include=energy
Olig1	p04	-1.144	2	http://api.brain-map.org/grid_data/download/100077689?include=energy
P2rx7	p04	-0.714	2	http://api.brain-map.org/grid_data/download/100072206?include=energy
Pml	p04	-0.716	2	http://api.brain-map.org/grid_data/download/100101193?include=energy
Pou5f1	p04	-1.039	2	http://api.brain-map.org/grid_data/download/100081480?include=energy
Ppargc1a	p04	-0.683	2	http://api.brain-map.org/grid_data/download/100077679?include=energy
Psen1	p04	-0.307	2	http://api.brain-map.org/grid_data/download/100056015?include=energy
Rbpj	p04	-0.648	2	http://api.brain-map.org/grid_data/download/100056045?include=energy
Robo3	p04	-0.653	2	http://api.brain-map.org/grid_data/download/100074272?include=energy
Sall1	p04	-0.615	2	http://api.brain-map.org/grid_data/download/161151415?include=energy
Selplg	p04	-1.177	2	http://api.brain-map.org/grid_data/download/100092211?include=energy
Sema7a	p04	0.337	2	http://api.brain-map.org/grid_data/download/100056087?include=energy
Serpina3k	p04	-0.964	2	http://api.brain-map.org/grid_data/download/100055158?include=energy
Slc16a2	p04	-0.842	2	http://api.brain-map.org/grid_data/download/100091833?include=energy
Slc40a1	p04	0.097	2	http://api.brain-map.org/grid_data/download/100081836?include=energy
Snca	p04	0.271	2	http://api.brain-map.org/grid_data/download/100091932?include=energy
Sox8	p04	-1.197	2	http://api.brain-map.org/grid_data/download/100075736?include=energy
Sstr4	p04	-0.933	2	http://api.brain-map.org/grid_data/download/100072177?include=energy
Stat4	p04	-0.794	2	http://api.brain-map.org/grid_data/download/100075735?include=energy
Tbx21	p04	-0.543	2	http://api.brain-map.org/grid_data/download/100093010?include=energy
Tgfa	p04	-0.329	2	http://api.brain-map.org/grid_data/download/100057402?include=energy
Tgfb1	p04	-0.148	2	http://api.brain-map.org/grid_data/download/100054863?include=energy
Tgfb2	p04	-1.110	2	http://api.brain-map.org/grid_data/download/100054850?include=energy
Tgfbr1	p04	-0.011	2	http://api.brain-map.org/grid_data/download/100054964?include=energy
Th	p04	0.077	2	http://api.brain-map.org/grid_data/download/100073400?include=energy
Tmem119	p04	-1.137	2	http://api.brain-map.org/grid_data/download/100090894?include=energy
Tph2	p04	-0.500	2	http://api.brain-map.org/grid_data/download/100057403?include=energy
Trib2	p04	0.385	2	http://api.brain-map.org/grid_data/download/100055166?include=energy
Trp53	p04	-0.289	2	http://api.brain-map.org/grid_data/download/100092249?include=energy
Vdr	p04	-0.572	2	http://api.brain-map.org/grid_data/download/100074713?include=energy
Vipr2	p04	-0.983	2	http://api.brain-map.org/grid_data/download/100081862?include=energy
Wnt3	p04	-0.412	2	http://api.brain-map.org/grid_data/download/100073412?include=energy
Xbp1	p04	-0.990	2	http://api.brain-map.org/grid_data/download/100075682?include=energy
Zfp36l1	p04	-0.595	2	http://api.brain-map.org/grid_data/download/161151333?include=energy
A2m	p04	-0.575	3	http://api.brain-map.org/grid_data/download/100084373?include=energy
Abl1	p04	-1.438	3	http://api.brain-map.org/grid_data/download/100084364?include=energy
Ace	p04	-0.937	3	http://api.brain-map.org/grid_data/download/100084361?include=energy
Actb	p04	-1.449	3	http://api.brain-map.org/grid_data/download/100056178?include=energy
Anxa2	p04	-1.315	3	http://api.brain-map.org/grid_data/download/100074264?include=energy
Arnt2	p04	-1.235	3	http://api.brain-map.org/grid_data/download/100074988?include=energy
Atf4	p04	-1.311	3	http://api.brain-map.org/grid_data/download/100075690?include=energy
Bdnf	p04	-1.232	3	http://api.brain-map.org/grid_data/download/100091864?include=energy
Bmp1	p04	-1.456	3	http://api.brain-map.org/grid_data/download/100092330?include=energy
Bmp7	p04	-1.022	3	http://api.brain-map.org/grid_data/download/100074464?include=energy
Car2	p04	-1.035	3	http://api.brain-map.org/grid_data/download/100081431?include=energy
Cartpt	p04	-1.335	3	http://api.brain-map.org/grid_data/download/100055006?include=energy
Cldn11	p04	-1.445	3	http://api.brain-map.org/grid_data/download/100072261?include=energy
Cnp	p04	-1.341	3	http://api.brain-map.org/grid_data/download/100081837?include=energy
Comt	p04	-1.434	3	http://api.brain-map.org/grid_data/download/100021509?include=energy
Cx3cl1	p04	-1.426	3	http://api.brain-map.org/grid_data/download/100074726?include=energy
Dcn	p04	-1.449	3	http://api.brain-map.org/grid_data/download/100040925?include=energy
Dlk1	p04	-1.444	3	http://api.brain-map.org/grid_data/download/100056345?include=energy
Drd2	p04	-1.474	3	http://api.brain-map.org/grid_data/download/100021512?include=energy
Egr1	p04	-1.317	3	http://api.brain-map.org/grid_data/download/100092154?include=energy
Eomes	p04	-0.287	3	http://api.brain-map.org/grid_data/download/100092540?include=energy
Epas1	p04	-1.340	3	http://api.brain-map.org/grid_data/download/100075697?include=energy
Fgfr1	p04	-1.128	3	http://api.brain-map.org/grid_data/download/100055157?include=energy
Fos	p04	-1.322	3	http://api.brain-map.org/grid_data/download/100056004?include=energy
Gad1	p04	-1.152	3	http://api.brain-map.org/grid_data/download/100054917?include=energy
Gad2	p04	-1.475	3	http://api.brain-map.org/grid_data/download/100092175?include=energy
Gpr37	p04	-1.168	3	http://api.brain-map.org/grid_data/download/100054915?include=energy
Gria3	p04	-1.335	3	http://api.brain-map.org/grid_data/download/100092161?include=energy
Gria4	p04	-1.231	3	http://api.brain-map.org/grid_data/download/100092177?include=energy
Grin2b	p04	-1.460	3	http://api.brain-map.org/grid_data/download/100092159?include=energy
Grm5	p04	-1.270	3	http://api.brain-map.org/grid_data/download/100092137?include=energy
Hif1a	p04	-1.390	3	http://api.brain-map.org/grid_data/download/100075639?include=energy
Icam5	p04	-1.310	3	http://api.brain-map.org/grid_data/download/100076661?include=energy
Inpp4b	p04	-1.134	3	http://api.brain-map.org/grid_data/download/100054988?include=energy
Lepr	p04	-0.962	3	http://api.brain-map.org/grid_data/download/100091939?include=energy
Lrrk2	p04	-0.974	3	http://api.brain-map.org/grid_data/download/100072183?include=energy
Mog	p04	-1.435	3	http://api.brain-map.org/grid_data/download/100084460?include=energy
Ncoa2	p04	-1.178	3	http://api.brain-map.org/grid_data/download/100075004?include=energy
Nfasc	p04	-1.405	3	http://api.brain-map.org/grid_data/download/100074987?include=energy
Ngf	p04	-1.281	3	http://api.brain-map.org/grid_data/download/100074232?include=energy
Nr3c1	p04	-0.739	3	http://api.brain-map.org/grid_data/download/100074192?include=energy
Nr4a1	p04	-1.460	3	http://api.brain-map.org/grid_data/download/100054849?include=energy
Ostf1	p04	-1.119	3	http://api.brain-map.org/grid_data/download/100075741?include=energy
Plp1	p04	-1.283	3	http://api.brain-map.org/grid_data/download/100054916?include=energy
Pomc	p04	-1.205	3	http://api.brain-map.org/grid_data/download/100074250?include=energy
Prlr	p04	-1.493	3	http://api.brain-map.org/grid_data/download/100054848?include=energy
Prnp	p04	-1.480	3	http://api.brain-map.org/grid_data/download/100054845?include=energy
Rac2	p04	-0.947	3	http://api.brain-map.org/grid_data/download/100094443?include=energy
Rora	p04	-1.485	3	http://api.brain-map.org/grid_data/download/100054927?include=energy
Rreb1	p04	-1.380	3	http://api.brain-map.org/grid_data/download/100056170?include=energy
S100b	p04	-1.308	3	http://api.brain-map.org/grid_data/download/100074954?include=energy
Slc1a2	p04	-1.364	3	http://api.brain-map.org/grid_data/download/100057363?include=energy
Smad4	p04	-1.124	3	http://api.brain-map.org/grid_data/download/100074270?include=energy
Sorl1	p04	-1.334	3	http://api.brain-map.org/grid_data/download/100084345?include=energy
Sox10	p04	-1.305	3	http://api.brain-map.org/grid_data/download/100054866?include=energy
Sox2	p04	-1.076	3	http://api.brain-map.org/grid_data/download/100074249?include=energy
Spp1	p04	-1.381	3	http://api.brain-map.org/grid_data/download/100056075?include=energy
Stat1	p04	-1.302	3	http://api.brain-map.org/grid_data/download/100074964?include=energy
Stat5b	p04	-0.579	3	http://api.brain-map.org/grid_data/download/100075638?include=energy
Syn1	p04	-1.279	3	http://api.brain-map.org/grid_data/download/100054911?include=energy
Syt1	p04	-1.389	3	http://api.brain-map.org/grid_data/download/100072264?include=energy
Tac1	p04	-1.487	3	http://api.brain-map.org/grid_data/download/100074468?include=energy
Tac2	p04	-1.332	3	http://api.brain-map.org/grid_data/download/100054971?include=energy
Tef	p04	-1.439	3	http://api.brain-map.org/grid_data/download/100075648?include=energy
Trp63	p04	-1.191	3	http://api.brain-map.org/grid_data/download/100092340?include=energy
Vip	p04	-1.474	3	http://api.brain-map.org/grid_data/download/100074480?include=energy
Adam17	p04	-0.746	4	http://api.brain-map.org/grid_data/download/100091923?include=energy
Agtr1a	p04	-0.079	4	http://api.brain-map.org/grid_data/download/100020263?include=energy
Ahr	p04	-0.961	4	http://api.brain-map.org/grid_data/download/100081403?include=energy
Akt1	p04	-0.509	4	http://api.brain-map.org/grid_data/download/100081424?include=energy
Apod	p04	-1.144	4	http://api.brain-map.org/grid_data/download/100084378?include=energy
Apoe	p04	-0.885	4	http://api.brain-map.org/grid_data/download/100054922?include=energy
Ar	p04	-0.611	4	http://api.brain-map.org/grid_data/download/100020262?include=energy
Arntl	p04	0.000	4	http://api.brain-map.org/grid_data/download/100074704?include=energy
Ascl2	p04	-0.525	4	http://api.brain-map.org/grid_data/download/100092123?include=energy
Atf3	p04	-1.043	4	http://api.brain-map.org/grid_data/download/100091971?include=energy
Bche	p04	-0.713	4	http://api.brain-map.org/grid_data/download/100020261?include=energy
Bcl2	p04	-0.343	4	http://api.brain-map.org/grid_data/download/100076639?include=energy
Bmp5	p04	-1.086	4	http://api.brain-map.org/grid_data/download/100091920?include=energy
Brca1	p04	-0.693	4	http://api.brain-map.org/grid_data/download/161155276?include=energy
Cd47	p04	0.508	4	http://api.brain-map.org/grid_data/download/100074716?include=energy
Cdh17	p04	-0.685	4	http://api.brain-map.org/grid_data/download/100075713?include=energy
Cdx2	p04	-0.880	4	http://api.brain-map.org/grid_data/download/100081405?include=energy
Chat	p04	-0.581	4	http://api.brain-map.org/grid_data/download/100059117?include=energy
Cnr1	p04	-0.838	4	http://api.brain-map.org/grid_data/download/100051838?include=energy
Cnr2	p04	-0.786	4	http://api.brain-map.org/grid_data/download/100054924?include=energy
Cst3	p04	-0.237	4	http://api.brain-map.org/grid_data/download/100056013?include=energy
Cux2	p04	0.090	4	http://api.brain-map.org/grid_data/download/100056187?include=energy
Ddit3	p04	-0.548	4	http://api.brain-map.org/grid_data/download/100078501?include=energy
Erg	p04	0.328	4	http://api.brain-map.org/grid_data/download/100078551?include=energy
Esr1	p04	-1.060	4	http://api.brain-map.org/grid_data/download/100021757?include=energy
Esr2	p04	-0.712	4	http://api.brain-map.org/grid_data/download/100021758?include=energy
Evi5	p04	-0.732	4	http://api.brain-map.org/grid_data/download/100091937?include=energy
Fas	p04	-0.046	4	http://api.brain-map.org/grid_data/download/100084344?include=energy
Foxp3	p04	-0.411	4	http://api.brain-map.org/grid_data/download/100081410?include=energy
Gabpa	p04	0.392	4	http://api.brain-map.org/grid_data/download/100078476?include=energy
Gabra3	p04	-0.876	4	http://api.brain-map.org/grid_data/download/100021625?include=energy
Galr2	p04	0.160	4	http://api.brain-map.org/grid_data/download/100092251?include=energy
Grm4	p04	-0.399	4	http://api.brain-map.org/grid_data/download/100056120?include=energy

Hap1	p04	0.453	4	http://api.brain-map.org/grid_data/download/100054910?include=energy
Hoxb4	p04	-0.708	4	http://api.brain-map.org/grid_data/download/100078519?include=energy
Hrh3	p04	-1.103	4	http://api.brain-map.org/grid_data/download/100101304?include=energy
Hspd1	p04	-0.120	4	http://api.brain-map.org/grid_data/download/100084410?include=energy
Igf1r	p04	0.500	4	http://api.brain-map.org/grid_data/download/100057336?include=energy
Il16	p04	-0.989	4	http://api.brain-map.org/grid_data/download/100055173?include=energy
Il6st	p04	-0.499	4	http://api.brain-map.org/grid_data/download/100057337?include=energy
Itln1	p04	-0.919	4	http://api.brain-map.org/grid_data/download/100093122?include=energy
Kcnh7	p04	-0.941	4	http://api.brain-map.org/grid_data/download/100040938?include=energy
Lrp1	p04	0.690	4	http://api.brain-map.org/grid_data/download/100084381?include=energy
Mapt	p04	-0.044	4	http://api.brain-map.org/grid_data/download/100054902?include=energy
Mbp	p04	-1.139	4	http://api.brain-map.org/grid_data/download/100054892?include=energy
Mef2b	p04	-0.819	4	http://api.brain-map.org/grid_data/download/100101307?include=energy
Mmp9	p04	-0.493	4	http://api.brain-map.org/grid_data/download/100094463?include=energy
Msc	p04	-1.108	4	http://api.brain-map.org/grid_data/download/100081417?include=energy
Mst1r	p04	-0.178	4	http://api.brain-map.org/grid_data/download/100074185?include=energy
Nfatc2	p04	-0.668	4	http://api.brain-map.org/grid_data/download/100073381?include=energy
Nos3	p04	-0.405	4	http://api.brain-map.org/grid_data/download/100084358?include=energy
Notch1	p04	0.745	4	http://api.brain-map.org/grid_data/download/100056172?include=energy
Nr0b2	p04	-0.917	4	http://api.brain-map.org/grid_data/download/100081821?include=energy
Nr1i2	p04	-0.302	4	http://api.brain-map.org/grid_data/download/100091842?include=energy
Nr4a2	p04	-0.264	4	http://api.brain-map.org/grid_data/download/100032294?include=energy
P2rx4	p04	-0.500	4	http://api.brain-map.org/grid_data/download/100090940?include=energy
Pik3cb	p04	-0.790	4	http://api.brain-map.org/grid_data/download/100076636?include=energy
Ppara	p04	-0.925	4	http://api.brain-map.org/grid_data/download/100094442?include=energy
Pparg	p04	-0.883	4	http://api.brain-map.org/grid_data/download/100054867?include=energy
Prkcb	p04	-1.159	4	http://api.brain-map.org/grid_data/download/100081781?include=energy
Ptgs2	p04	-0.507	4	http://api.brain-map.org/grid_data/download/100040915?include=energy
Pthlh	p04	-1.112	4	http://api.brain-map.org/grid_data/download/100101156?include=energy
Ptprc	p04	-0.511	4	http://api.brain-map.org/grid_data/download/100076670?include=energy
Pvalb	p04	-1.140	4	http://api.brain-map.org/grid_data/download/100054926?include=energy
Rela	p04	-0.601	4	http://api.brain-map.org/grid_data/download/100074693?include=energy
Ros1	p04	-0.438	4	http://api.brain-map.org/grid_data/download/100057754?include=energy
S1pr1	p04	-0.888	4	http://api.brain-map.org/grid_data/download/100092179?include=energy
Scn10a	p04	-0.488	4	http://api.brain-map.org/grid_data/download/100092197?include=energy
Sgk1	p04	-0.604	4	http://api.brain-map.org/grid_data/download/100054992?include=energy
Shh	p04	0.150	4	http://api.brain-map.org/grid_data/download/100056242?include=energy
Slc1a1	p04	-1.022	4	http://api.brain-map.org/grid_data/download/100054970?include=energy
Slc9a9	p04	-0.781	4	http://api.brain-map.org/grid_data/download/100084342?include=energy
Smad7	p04	-1.017	4	http://api.brain-map.org/grid_data/download/100056177?include=energy
Sort1	p04	-0.786	4	http://api.brain-map.org/grid_data/download/100084391?include=energy
Stat3	p04	-0.920	4	http://api.brain-map.org/grid_data/download/100075013?include=energy
Stat5a	p04	-0.254	4	http://api.brain-map.org/grid_data/download/100078884?include=energy
Tdo2	p04	-0.866	4	http://api.brain-map.org/grid_data/download/100081485?include=energy
Tgfb3	p04	-0.711	4	http://api.brain-map.org/grid_data/download/100054868?include=energy
Tlr2	p04	0.766	4	http://api.brain-map.org/grid_data/download/100084408?include=energy
Trp73	p04	-0.259	4	http://api.brain-map.org/grid_data/download/100076669?include=energy
Tspan31	p04	-0.795	4	http://api.brain-map.org/grid_data/download/100094473?include=energy
Tyro3	p04	-0.655	4	http://api.brain-map.org/grid_data/download/100056093?include=energy
Ubtf	p04	-1.017	4	http://api.brain-map.org/grid_data/download/100081602?include=energy
Vav2	p04	0.152	4	http://api.brain-map.org/grid_data/download/100040893?include=energy
Zeb1	p04	-0.653	4	http://api.brain-map.org/grid_data/download/100075726?include=energy
Zic1	p04	-0.708	4	http://api.brain-map.org/grid_data/download/100054957?include=energy
Adcyap1	p14	-0.181	1	http://api.brain-map.org/grid_data/download/100015429?include=energy
Adnp	p14	-0.032	1	http://api.brain-map.org/grid_data/download/100071299?include=energy
Adra2b	p14	-1.032	1	http://api.brain-map.org/grid_data/download/100033664?include=energy
Ahi1	p14	0.129	1	http://api.brain-map.org/grid_data/download/100070249?include=energy
Alcam	p14	1.048	1	http://api.brain-map.org/grid_data/download/100084480?include=energy
Angpt2	p14	0.422	1	http://api.brain-map.org/grid_data/download/100044887?include=energy
Angptl4	p14	-0.505	1	http://api.brain-map.org/grid_data/download/100093756?include=energy
Apc	p14	0.535	1	http://api.brain-map.org/grid_data/download/100042423?include=energy
Axl	p14	0.563	1	http://api.brain-map.org/grid_data/download/100032174?include=energy
Bmpr2	p14	-0.910	1	http://api.brain-map.org/grid_data/download/100042432?include=energy
Cacna1h	p14	0.002	1	http://api.brain-map.org/grid_data/download/100033366?include=energy
Casp3	p14	-0.889	1	http://api.brain-map.org/grid_data/download/100093948?include=energy
Cdh1	p14	-0.410	1	http://api.brain-map.org/grid_data/download/100042342?include=energy
Cntn1	p14	0.460	1	http://api.brain-map.org/grid_data/download/100093747?include=energy
Cxcl12	p14	0.589	1	http://api.brain-map.org/grid_data/download/100093951?include=energy
Cxcr4	p14	0.939	1	http://api.brain-map.org/grid_data/download/100084487?include=energy
Ddr1	p14	0.726	1	http://api.brain-map.org/grid_data/download/100038944?include=energy
Ephb2	p14	-0.570	1	http://api.brain-map.org/grid_data/download/100083108?include=energy
Fbxw4	p14	0.249	1	http://api.brain-map.org/grid_data/download/100041067?include=energy
Fgf23	p14	-0.774	1	http://api.brain-map.org/grid_data/download/100074100?include=energy
Fmr1	p14	0.603	1	http://api.brain-map.org/grid_data/download/100045248?include=energy
Fn1	p14	1.087	1	http://api.brain-map.org/grid_data/download/100041500?include=energy
Fyn	p14	-0.989	1	http://api.brain-map.org/grid_data/download/100070223?include=energy
Gap43	p14	0.781	1	http://api.brain-map.org/grid_data/download/100034571?include=energy
Gata3	p14	-0.215	1	http://api.brain-map.org/grid_data/download/100093750?include=energy
Ghsr	p14	0.764	1	http://api.brain-map.org/grid_data/download/100036607?include=energy
Gria2	p14	-0.232	1	http://api.brain-map.org/grid_data/download/100041812?include=energy
Grm3	p14	-0.241	1	http://api.brain-map.org/grid_data/download/100035528?include=energy
Grm8	p14	0.526	1	http://api.brain-map.org/grid_data/download/100041445?include=energy
Hfe	p14	-0.613	1	http://api.brain-map.org/grid_data/download/100084545?include=energy
Hrh1	p14	-0.464	1	http://api.brain-map.org/grid_data/download/100015324?include=energy
Mc4r	p14	0.246	1	http://api.brain-map.org/grid_data/download/100039224?include=energy
Ncam1	p14	0.105	1	http://api.brain-map.org/grid_data/download/100045356?include=energy
Nfkbia	p14	-0.737	1	http://api.brain-map.org/grid_data/download/100083102?include=energy
Nfya	p14	0.708	1	http://api.brain-map.org/grid_data/download/100053494?include=energy
Nos1	p14	-0.390	1	http://api.brain-map.org/grid_data/download/100083115?include=energy
Notch3	p14	0.311	1	http://api.brain-map.org/grid_data/download/100039231?include=energy
Npy	p14	-0.016	1	http://api.brain-map.org/grid_data/download/100035172?include=energy
Nr4a3	p14	-0.594	1	http://api.brain-map.org/grid_data/download/100093759?include=energy
Nrxn1	p14	0.409	1	http://api.brain-map.org/grid_data/download/100076230?include=energy
Ntf3	p14	-0.499	1	http://api.brain-map.org/grid_data/download/100015433?include=energy
Ntn1	p14	-0.142	1	http://api.brain-map.org/grid_data/download/100042403?include=energy
Plxna2	p14	0.291	1	http://api.brain-map.org/grid_data/download/100041608?include=energy
Plxna3	p14	-0.334	1	http://api.brain-map.org/grid_data/download/100035020?include=energy
Polr1d	p14	-0.422	1	http://api.brain-map.org/grid_data/download/166003379?include=energy
Rara	p14	-0.469	1	http://api.brain-map.org/grid_data/download/100070217?include=energy
Reln	p14	0.412	1	http://api.brain-map.org/grid_data/download/100038872?include=energy
Rtn4r	p14	-0.377	1	http://api.brain-map.org/grid_data/download/100041780?include=energy
Sema3a	p14	1.042	1	http://api.brain-map.org/grid_data/download/100038195?include=energy
Sema4a	p14	0.717	1	http://api.brain-map.org/grid_data/download/100036969?include=energy
Slc1a3	p14	0.058	1	http://api.brain-map.org/grid_data/download/100093911?include=energy
Slc1a4	p14	-0.420	1	http://api.brain-map.org/grid_data/download/100084493?include=energy
Slc6a4	p14	0.272	1	http://api.brain-map.org/grid_data/download/100045306?include=energy
Sp3	p14	-0.012	1	http://api.brain-map.org/grid_data/download/100076143?include=energy
Ssrp1	p14	-0.576	1	http://api.brain-map.org/grid_data/download/100070260?include=energy
Stx1a	p14	0.981	1	http://api.brain-map.org/grid_data/download/100035668?include=energy
Trpv1	p14	0.421	1	http://api.brain-map.org/grid_data/download/100041505?include=energy
Vegfa	p14	-1.099	1	http://api.brain-map.org/grid_data/download/100091088?include=energy
Ache	p14	1.339	2	http://api.brain-map.org/grid_data/download/100015310?include=energy
Aff3	p14	1.366	2	http://api.brain-map.org/grid_data/download/100052987?include=energy
Agt	p14	1.322	2	http://api.brain-map.org/grid_data/download/100039268?include=energy
Amh	p14	1.416	2	http://api.brain-map.org/grid_data/download/100042462?include=energy
App	p14	1.257	2	http://api.brain-map.org/grid_data/download/100038222?include=energy
Aqp4	p14	1.355	2	http://api.brain-map.org/grid_data/download/100030645?include=energy
Bach2	p14	1.488	2	http://api.brain-map.org/grid_data/download/100053393?include=energy
Bmp2	p14	1.023	2	http://api.brain-map.org/grid_data/download/100045252?include=energy
Bmp4	p14	1.491	2	http://api.brain-map.org/grid_data/download/100030605?include=energy
C1qa	p14	1.175	2	http://api.brain-map.org/grid_data/download/100084472?include=energy
Cd44	p14	1.323	2	http://api.brain-map.org/grid_data/download/100039207?include=energy
Cdh3	p14	1.488	2	http://api.brain-map.org/grid_data/download/100053473?include=energy
Cic	p14	1.282	2	http://api.brain-map.org/grid_data/download/100071341?include=energy
Cldn5	p14	1.492	2	http://api.brain-map.org/grid_data/download/100039354?include=energy
Clock	p14	1.492	2	http://api.brain-map.org/grid_data/download/100083163?include=energy
Clstn2	p14	1.133	2	http://api.brain-map.org/grid_data/download/100053594?include=energy
Clu	p14	0.762	2	http://api.brain-map.org/grid_data/download/100045519?include=energy
Col11a2	p14	1.448	2	http://api.brain-map.org/grid_data/download/100083208?include=energy
Col2a1	p14	1.478	2	http://api.brain-map.org/grid_data/download/100053489?include=energy
Creb1	p14	1.386	2	http://api.brain-map.org/grid_data/download/100035706?include=energy
Crhr1	p14	1.312	2	http://api.brain-map.org/grid_data/download/100056838?include=energy
Csf1r	p14	0.826	2	http://api.brain-map.org/grid_data/download/100042150?include=energy
Ctnnb1	p14	1.224	2	http://api.brain-map.org/grid_data/download/100053231?include=energy
Cyp7b1	p14	1.258	2	http://api.brain-map.org/grid_data/download/100070136?include=energy
Dbp	p14	1.350	2	http://api.brain-map.org/grid_data/download/100056425?include=energy
Dll1	p14	1.371	2	http://api.brain-map.org/grid_data/download/100047806?include=energy
Dll4	p14	1.304	2	http://api.brain-map.org/grid_data/download/100041605?include=energy
Ebf1	p14	1.458	2	http://api.brain-map.org/grid_data/download/100045623?include=energy
Ednrb	p14	1.478	2	http://api.brain-map.org/grid_data/download/100015301?include=energy
Egf	p14	1.489	2	http://api.brain-map.org/grid_data/download/100044878?include=energy
Egfr	p14	1.230	2	http://api.brain-map.org/grid_data/download/100015300?include=energy
Epha4	p14	1.228	2	http://api.brain-map.org/grid_data/download/100042452?include=energy
Erbb3	p14	1.396	2	http://api.brain-map.org/grid_data/download/100045555?include=energy
Ets1	p14	1.170	2	http://api.brain-map.org/grid_data/download/100052897?include=energy
Fgf1	p14	0.963	2	http://api.brain-map.org/grid_data/download/100030613?include=energy
Foxc1	p14	1.351	2	http://api.brain-map.org/grid_data/download/100045344?include=energy
Foxo3	p14	0.920	2	http://api.brain-map.org/grid_data/download/100056493?include=energy
Fzd4	p14	1.428	2	http://api.brain-map.org/grid_data/download/100034542?include=energy
Gal	p14	1.381	2	http://api.brain-map.org/grid_data/download/100041736?include=energy
Gdf15	p14	1.319	2	http://api.brain-map.org/grid_data/download/100045617?include=energy
Gfap	p14	1.448	2	http://api.brain-map.org/grid_data/download/100045585?include=energy
Gja1	p14	1.237	2	http://api.brain-map.org/grid_data/download/100039164?include=energy
Gli1	p14	1.306	2	http://api.brain-map.org/grid_data/download/100045257?include=energy
Glis3	p14	1.479	2	http://api.brain-map.org/grid_data/download/100053620?include=energy
Grm1	p14	1.379	2	http://api.brain-map.org/grid_data/download/100038271?include=energy
Hcrt	p14	1.494	2	http://api.brain-map.org/grid_data/download/100045705?include=energy
Hspb8	p14	1.003	2	http://api.brain-map.org/grid_data/download/100076179?include=energy
Igf1	p14	1.434	2	http://api.brain-map.org/grid_data/download/100045685?include=energy
Igfbp1	p14	1.473	2	http://api.brain-map.org/grid_data/download/100034630?include=energy
Igfbp3	p14	1.222	2	http://api.brain-map.org/grid_data/download/100045205?include=energy
Ikzf1	p14	1.422	2	http://api.brain-map.org/grid_data/download/100056492?include=energy
Insrr	p14	1.076	2	http://api.brain-map.org/grid_data/download/100056844?include=energy
Irf1	p14	1.302	2	http://api.brain-map.org/grid_data/download/100051764?include=energy
Itga4	p14	1.498	2	http://api.brain-map.org/grid_data/download/100056397?include=energy
Itgam	p14	1.496	2	http://api.brain-map.org/grid_data/download/100075158?include=energy
Jag1	p14	1.465	2	http://api.brain-map.org/grid_data/download/100045431?include=energy
Jazf1	p14	1.209	2	http://api.brain-map.org/grid_data/download/100052623?include=energy
Kdr	p14	1.494	2	http://api.brain-map.org/grid_data/download/100034635?include=energy
Lgals1	p14	1.339	2	http://api.brain-map.org/grid_data/download/100040815?include=energy
Lpl	p14	1.493	2	http://api.brain-map.org/grid_data/download/100040819?include=energy
Mapk8	p14	1.261	2	http://api.brain-map.org/grid_data/download/100076158?include=energy
Maz	p14	1.211	2	http://api.brain-map.org/grid_data/download/100053617?include=energy
Mertk	p14	1.416	2	http://api.brain-map.org/grid_data/download/100042401?include=energy
Mst1	p14	0.980	2	http://api.brain-map.org/grid_data/download/100045628?include=energy
Mtor	p14	1.258	2	http://api.brain-map.org/grid_data/download/100041658?include=energy
Mycn	p14	1.419	2	http://api.brain-map.org/grid_data/download/100053530?include=energy
Myt1	p14	1.396	2	http://api.brain-map.org/grid_data/download/100053016?include=energy
Nfe2l2	p14	1.341	2	http://api.brain-map.org/grid_data/download/100053113?include=energy
Nfkb1	p14	1.385	2	http://api.brain-map.org/grid_data/download/100052805?include=energy
Nog	p14	1.472	2	http://api.brain-map.org/grid_data/download/100053406?include=energy
Notch2	p14	1.474	2	http://api.brain-map.org/grid_data/download/100040950?include=energy
Notch4	p14	1.285	2	http://api.brain-map.org/grid_data/download/100049619?include=energy
Nr1h3	p14	1.449	2	http://api.brain-map.org/grid_data/download/100070097?include=energy
Nr3c2	p14	0.482	2	http://api.brain-map.org/grid_data/download/100083240?include=energy
Olig1	p14	1.024	2	http://api.brain-map.org/grid_data/download/100053470?include=energy
P2rx7	p14	1.475	2	http://api.brain-map.org/grid_data/download/100045354?include=energy
Pml	p14	1.437	2	http://api.brain-map.org/grid_data/download/100053042?include=energy
Pou5f1	p14	1.358	2	http://api.brain-map.org/grid_data/download/100071919?include=energy
Ppargc1a	p14	1.447	2	http://api.brain-map.org/grid_data/download/100053495?include=energy
Psen1	p14	1.336	2	http://api.brain-map.org/grid_data/download/100041559?include=energy
Rbpj	p14	1.434	2	http://api.brain-map.org/grid_data/download/100041643?include=energy
Robo3	p14	1.420	2	http://api.brain-map.org/grid_data/download/100049618?include=energy
Sall1	p14	1.459	2	http://api.brain-map.org/grid_data/download/166003601?include=energy
Selplg	p14	1.269	2	http://api.brain-map.org/grid_data/download/100053491?include=energy
Sema7a	p14	0.962	2	http://api.brain-map.org/grid_data/download/100041773?include=energy
Serpina3k	p14	1.096	2	http://api.brain-map.org/grid_data/download/100040758?include=energy
Slc16a2	p14	1.369	2	http://api.brain-map.org/grid_data/download/100045139?include=energy
Slc40a1	p14	1.379	2	http://api.brain-map.org/grid_data/download/100070094?include=energy
Snca	p14	0.718	2	http://api.brain-map.org/grid_data/download/100083167?include=energy
Sox8	p14	1.141	2	http://api.brain-map.org/grid_data/download/100053333?include=energy
Sstr4	p14	1.404	2	http://api.brain-map.org/grid_data/download/100045479?include=energy
Stat4	p14	1.462	2	http://api.brain-map.org/grid_data/download/100053319?include=energy
Tbx21	p14	1.495	2	http://api.brain-map.org/grid_data/download/100053618?include=energy
Tgfa	p14	1.473	2	http://api.brain-map.org/grid_data/download/100045147?include=energy
Tgfb1	p14	1.368	2	http://api.brain-map.org/grid_data/download/100039228?include=energy
Tgfb2	p14	1.253	2	http://api.brain-map.org/grid_data/download/100039162?include=energy
Tgfbr1	p14	1.397	2	http://api.brain-map.org/grid_data/download/100035737?include=energy
Th	p14	1.056	2	http://api.brain-map.org/grid_data/download/100038194?include=energy
Tmem119	p14	1.294	2	http://api.brain-map.org/grid_data/download/100090854?include=energy
Tph2	p14	1.497	2	http://api.brain-map.org/grid_data/download/100045270?include=energy
Trib2	p14	1.225	2	http://api.brain-map.org/grid_data/download/100037862?include=energy
Trp53	p14	1.432	2	http://api.brain-map.org/grid_data/download/100051765?include=energy
Vdr	p14	1.493	2	http://api.brain-map.org/grid_data/download/100052889?include=energy
Vipr2	p14	1.178	2	http://api.brain-map.org/grid_data/download/100075160?include=energy
Wnt3	p14	1.492	2	http://api.brain-map.org/grid_data/download/100045696?include=energy
Xbp1	p14	1.084	2	http://api.brain-map.org/grid_data/download/100053132?include=energy
Zfp36l1	p14	1.209	2	http://api.brain-map.org/grid_data/download/166003008?include=energy
A2m	p14	-1.100	3	http://api.brain-map.org/grid_data/download/100084468?include=energy
Abl1	p14	0.811	3	http://api.brain-map.org/grid_data/download/100084478?include=energy
Ace	p14	-0.634	3	http://api.brain-map.org/grid_data/download/100084475?include=energy
Actb	p14	0.217	3	http://api.brain-map.org/grid_data/download/100041480?include=energy
Anxa2	p14	0.690	3	http://api.brain-map.org/grid_data/download/100039598?include=energy
Arnt2	p14	0.002	3	http://api.brain-map.org/grid_data/download/100076210?include=energy
Atf4	p14	-0.247	3	http://api.brain-map.org/grid_data/download/100083049?include=energy
Bdnf	p14	1.040	3	http://api.brain-map.org/grid_data/download/100045103?include=energy
Bmp1	p14	0.684	3	http://api.brain-map.org/grid_data/download/100042416?include=energy
Bmp7	p14	0.473	3	http://api.brain-map.org/grid_data/download/100074098?include=energy
Car2	p14	-0.654	3	http://api.brain-map.org/grid_data/download/100070321?include=energy
Cartpt	p14	0.293	3	http://api.brain-map.org/grid_data/download/100040706?include=energy
Cldn11	p14	0.109	3	http://api.brain-map.org/grid_data/download/100045575?include=energy
Cnp	p14	-0.045	3	http://api.brain-map.org/grid_data/download/100070093?include=energy
Comt	p14	0.533	3	http://api.brain-map.org/grid_data/download/100015318?include=energy
Cx3cl1	p14	0.038	3	http://api.brain-map.org/grid_data/download/100051760?include=energy
Dcn	p14	0.687	3	http://api.brain-map.org/grid_data/download/100015428?include=energy
Dlk1	p14	0.120	3	http://api.brain-map.org/grid_data/download/100032188?include=energy
Drd2	p14	0.752	3	http://api.brain-map.org/grid_data/download/100015332?include=energy
Egr1	p14	-0.049	3	http://api.brain-map.org/grid_data/download/100041502?include=energy
Eomes	p14	-0.326	3	http://api.brain-map.org/grid_data/download/100093540?include=energy
Epas1	p14	1.075	3	http://api.brain-map.org/grid_data/download/100052859?include=energy
Fgfr1	p14	0.879	3	http://api.brain-map.org/grid_data/download/100040823?include=energy
Fos	p14	0.194	3	http://api.brain-map.org/grid_data/download/100076184?include=energy
Gad1	p14	0.968	3	http://api.brain-map.org/grid_data/download/100039430?include=energy
Gad2	p14	0.725	3	http://api.brain-map.org/grid_data/download/100040789?include=energy
Gpr37	p14	0.720	3	http://api.brain-map.org/grid_data/download/100075321?include=energy
Gria3	p14	-0.094	3	http://api.brain-map.org/grid_data/download/100041458?include=energy
Gria4	p14	0.236	3	http://api.brain-map.org/grid_data/download/100041040?include=energy
Grin2b	p14	0.487	3	http://api.brain-map.org/grid_data/download/100041301?include=energy
Grm5	p14	0.746	3	http://api.brain-map.org/grid_data/download/100038015?include=energy
Hif1a	p14	0.603	3	http://api.brain-map.org/grid_data/download/100053088?include=energy
Icam5	p14	1.102	3	http://api.brain-map.org/grid_data/download/100053382?include=energy
Inpp4b	p14	0.898	3	http://api.brain-map.org/grid_data/download/100040906?include=energy
Lepr	p14	-0.308	3	http://api.brain-map.org/grid_data/download/100093765?include=energy
Lrrk2	p14	-0.326	3	http://api.brain-map.org/grid_data/download/100045207?include=energy
Mog	p14	0.232	3	http://api.brain-map.org/grid_data/download/100093885?include=energy
Ncoa2	p14	0.621	3	http://api.brain-map.org/grid_data/download/100052984?include=energy
Nfasc	p14	0.741	3	http://api.brain-map.org/grid_data/download/100053028?include=energy
Ngf	p14	-0.058	3	http://api.brain-map.org/grid_data/download/100034617?include=energy
Nr3c1	p14	0.518	3	http://api.brain-map.org/grid_data/download/100035015?include=energy
Nr4a1	p14	0.762	3	http://api.brain-map.org/grid_data/download/100039165?include=energy
Ostf1	p14	1.022	3	http://api.brain-map.org/grid_data/download/100053318?include=energy
Plp1	p14	0.054	3	http://api.brain-map.org/grid_data/download/100076144?include=energy
Pomc	p14	0.663	3	http://api.brain-map.org/grid_data/download/100049616?include=energy
Prlr	p14	0.406	3	http://api.brain-map.org/grid_data/download/100075322?include=energy
Prnp	p14	0.309	3	http://api.brain-map.org/grid_data/download/100075307?include=energy
Rac2	p14	-0.441	3	http://api.brain-map.org/grid_data/download/100084482?include=energy
Rora	p14	0.693	3	http://api.brain-map.org/grid_data/download/100039419?include=energy

Rreb1	p14	0.146	3	http://api.brain-map.org/grid_data/download/100041470?include=energy
S100b	p14	0.066	3	http://api.brain-map.org/grid_data/download/100045455?include=energy
Slc1a2	p14	0.683	3	http://api.brain-map.org/grid_data/download/100045141?include=energy
Smad4	p14	0.862	3	http://api.brain-map.org/grid_data/download/100045600?include=energy
Sorl1	p14	0.195	3	http://api.brain-map.org/grid_data/download/100084507?include=energy
Sox10	p14	0.808	3	http://api.brain-map.org/grid_data/download/100034592?include=energy
Sox2	p14	0.639	3	http://api.brain-map.org/grid_data/download/100076206?include=energy
Spp1	p14	0.523	3	http://api.brain-map.org/grid_data/download/100039384?include=energy
Stat1	p14	0.585	3	http://api.brain-map.org/grid_data/download/100052934?include=energy
Stat5b	p14	-0.873	3	http://api.brain-map.org/grid_data/download/100093787?include=energy
Syn1	p14	-0.148	3	http://api.brain-map.org/grid_data/download/100075289?include=energy
Syt1	p14	0.609	3	http://api.brain-map.org/grid_data/download/100045576?include=energy
Tac1	p14	0.459	3	http://api.brain-map.org/grid_data/download/100056850?include=energy
Tac2	p14	1.055	3	http://api.brain-map.org/grid_data/download/100040657?include=energy
Tef	p14	0.180	3	http://api.brain-map.org/grid_data/download/100076175?include=energy
Trp63	p14	0.966	3	http://api.brain-map.org/grid_data/download/100037885?include=energy
Vip	p14	0.510	3	http://api.brain-map.org/grid_data/download/100049610?include=energy
Adam17	p14	-0.074	4	http://api.brain-map.org/grid_data/download/100041523?include=energy
Agtr1a	p14	-0.362	4	http://api.brain-map.org/grid_data/download/100076142?include=energy
Ahr	p14	0.181	4	http://api.brain-map.org/grid_data/download/100071139?include=energy
Akt1	p14	-1.133	4	http://api.brain-map.org/grid_data/download/100071211?include=energy
Apod	p14	-0.313	4	http://api.brain-map.org/grid_data/download/100084522?include=energy
Apoe	p14	0.371	4	http://api.brain-map.org/grid_data/download/100039428?include=energy
Ar	p14	-0.423	4	http://api.brain-map.org/grid_data/download/100015312?include=energy
Arntl	p14	-0.790	4	http://api.brain-map.org/grid_data/download/100075283?include=energy
Ascl2	p14	-0.462	4	http://api.brain-map.org/grid_data/download/100070212?include=energy
Atf3	p14	0.577	4	http://api.brain-map.org/grid_data/download/100070213?include=energy
Bche	p14	-0.088	4	http://api.brain-map.org/grid_data/download/100015317?include=energy
Bcl2	p14	0.225	4	http://api.brain-map.org/grid_data/download/100053378?include=energy
Bmp5	p14	0.343	4	http://api.brain-map.org/grid_data/download/100042426?include=energy
Brca1	p14	0.733	4	http://api.brain-map.org/grid_data/download/165999176?include=energy
Cd47	p14	-1.426	4	http://api.brain-map.org/grid_data/download/100052886?include=energy
Cdh17	p14	-1.002	4	http://api.brain-map.org/grid_data/download/100076183?include=energy
Cdx2	p14	-0.008	4	http://api.brain-map.org/grid_data/download/100071140?include=energy
Chat	p14	-0.625	4	http://api.brain-map.org/grid_data/download/100026524?include=energy
Cnr1	p14	0.624	4	http://api.brain-map.org/grid_data/download/100015330?include=energy
Cnr2	p14	0.234	4	http://api.brain-map.org/grid_data/download/100030633?include=energy
Cst3	p14	-0.635	4	http://api.brain-map.org/grid_data/download/100041547?include=energy
Cux2	p14	-0.105	4	http://api.brain-map.org/grid_data/download/100041487?include=energy
Ddit3	p14	-0.389	4	http://api.brain-map.org/grid_data/download/100076190?include=energy
Erg	p14	-0.484	4	http://api.brain-map.org/grid_data/download/100093716?include=energy
Esr1	p14	-0.145	4	http://api.brain-map.org/grid_data/download/100083166?include=energy
Esr2	p14	-0.350	4	http://api.brain-map.org/grid_data/download/100083180?include=energy
Evi5	p14	-0.443	4	http://api.brain-map.org/grid_data/download/100083170?include=energy
Fas	p14	-0.883	4	http://api.brain-map.org/grid_data/download/100084506?include=energy
Foxp3	p14	-0.462	4	http://api.brain-map.org/grid_data/download/100071142?include=energy
Gabpa	p14	-1.095	4	http://api.brain-map.org/grid_data/download/100093696?include=energy
Gabra3	p14	0.069	4	http://api.brain-map.org/grid_data/download/100083053?include=energy
Galr2	p14	-0.763	4	http://api.brain-map.org/grid_data/download/100093896?include=energy
Grm4	p14	-0.492	4	http://api.brain-map.org/grid_data/download/100041439?include=energy
Hap1	p14	0.141	4	http://api.brain-map.org/grid_data/download/100075345?include=energy
Hoxb4	p14	-0.721	4	http://api.brain-map.org/grid_data/download/100093820?include=energy
Hrh3	p14	-0.050	4	http://api.brain-map.org/grid_data/download/100101353?include=energy
Hspd1	p14	-0.446	4	http://api.brain-map.org/grid_data/download/100084497?include=energy
Igf1r	p14	-0.447	4	http://api.brain-map.org/grid_data/download/100041856?include=energy
Il16	p14	-0.252	4	http://api.brain-map.org/grid_data/download/100044875?include=energy
Il6st	p14	-1.146	4	http://api.brain-map.org/grid_data/download/100041976?include=energy
Itln1	p14	-0.127	4	http://api.brain-map.org/grid_data/download/100074085?include=energy
Kcnh7	p14	-0.314	4	http://api.brain-map.org/grid_data/download/100015451?include=energy
Lrp1	p14	-0.874	4	http://api.brain-map.org/grid_data/download/100084518?include=energy
Mapt	p14	-0.262	4	http://api.brain-map.org/grid_data/download/100030593?include=energy
Mbp	p14	-0.160	4	http://api.brain-map.org/grid_data/download/100034560?include=energy
Mmp9	p14	-0.435	4	http://api.brain-map.org/grid_data/download/100093809?include=energy
Msc	p14	0.504	4	http://api.brain-map.org/grid_data/download/100071276?include=energy
Mst1r	p14	-0.608	4	http://api.brain-map.org/grid_data/download/100093724?include=energy
Nfatc2	p14	-0.184	4	http://api.brain-map.org/grid_data/download/100035394?include=energy
Nos3	p14	0.527	4	http://api.brain-map.org/grid_data/download/100084491?include=energy
Notch1	p14	-0.723	4	http://api.brain-map.org/grid_data/download/100094341?include=energy
Nr0b2	p14	0.608	4	http://api.brain-map.org/grid_data/download/100070123?include=energy
Nr1i2	p14	0.442	4	http://api.brain-map.org/grid_data/download/100053550?include=energy
Nr4a2	p14	-0.865	4	http://api.brain-map.org/grid_data/download/100026602?include=energy
P2rx4	p14	-0.488	4	http://api.brain-map.org/grid_data/download/100101347?include=energy
Pik3cb	p14	-0.124	4	http://api.brain-map.org/grid_data/download/100053464?include=energy
Pou2af1	p14	-0.569	4	http://api.brain-map.org/grid_data/download/100070139?include=energy
Ppara	p14	0.561	4	http://api.brain-map.org/grid_data/download/100070100?include=energy
Pparg	p14	-0.243	4	http://api.brain-map.org/grid_data/download/100034625?include=energy
Prkcb	p14	0.016	4	http://api.brain-map.org/grid_data/download/100070312?include=energy
Ptgs2	p14	-0.761	4	http://api.brain-map.org/grid_data/download/100010062?include=energy
Pthlh	p14	0.495	4	http://api.brain-map.org/grid_data/download/100093723?include=energy
Ptprc	p14	-0.692	4	http://api.brain-map.org/grid_data/download/100083184?include=energy
Pvalb	p14	-0.228	4	http://api.brain-map.org/grid_data/download/100039445?include=energy
Rela	p14	-0.293	4	http://api.brain-map.org/grid_data/download/100052774?include=energy
Ros1	p14	-0.533	4	http://api.brain-map.org/grid_data/download/100083250?include=energy
S1pr1	p14	0.657	4	http://api.brain-map.org/grid_data/download/100041790?include=energy
Scn10a	p14	-0.503	4	http://api.brain-map.org/grid_data/download/100010060?include=energy
Sgk1	p14	0.615	4	http://api.brain-map.org/grid_data/download/100056877?include=energy
Shh	p14	-0.758	4	http://api.brain-map.org/grid_data/download/100093645?include=energy
Slc1a1	p14	0.157	4	http://api.brain-map.org/grid_data/download/100040650?include=energy
Slc9a9	p14	-0.868	4	http://api.brain-map.org/grid_data/download/100084542?include=energy
Smad7	p14	0.255	4	http://api.brain-map.org/grid_data/download/100037883?include=energy
Sort1	p14	-0.369	4	http://api.brain-map.org/grid_data/download/100084529?include=energy
Stat3	p14	-0.187	4	http://api.brain-map.org/grid_data/download/100052936?include=energy
Stat5a	p14	-1.309	4	http://api.brain-map.org/grid_data/download/100101201?include=energy
Tdo2	p14	0.398	4	http://api.brain-map.org/grid_data/download/100071321?include=energy
Tgfb3	p14	-0.300	4	http://api.brain-map.org/grid_data/download/100034628?include=energy
Tlr2	p14	-0.985	4	http://api.brain-map.org/grid_data/download/100084496?include=energy
Trp73	p14	-0.760	4	http://api.brain-map.org/grid_data/download/100101249?include=energy
Tspan31	p14	0.631	4	http://api.brain-map.org/grid_data/download/100093851?include=energy
Tyro3	p14	-0.404	4	http://api.brain-map.org/grid_data/download/100041507?include=energy
Ubtf	p14	-0.469	4	http://api.brain-map.org/grid_data/download/100071210?include=energy
Vav2	p14	0.353	4	http://api.brain-map.org/grid_data/download/100015574?include=energy
Zeb1	p14	-0.291	4	http://api.brain-map.org/grid_data/download/100053311?include=energy
Zic1	p14	-0.588	4	http://api.brain-map.org/grid_data/download/100093741?include=energy
Adcyap1	p28	-1.038	1	http://api.brain-map.org/grid_data/download/100011597?include=energy
Adnp	p28	0.430	1	http://api.brain-map.org/grid_data/download/100071328?include=energy
Adra2b	p28	0.582	1	http://api.brain-map.org/grid_data/download/100074072?include=energy
Ahi1	p28	-1.235	1	http://api.brain-map.org/grid_data/download/100070253?include=energy
Alcam	p28	-1.127	1	http://api.brain-map.org/grid_data/download/100083003?include=energy
Angpt2	p28	-1.001	1	http://api.brain-map.org/grid_data/download/100036574?include=energy
Angptl4	p28	-0.448	1	http://api.brain-map.org/grid_data/download/100045247?include=energy
Apc	p28	-1.499	1	http://api.brain-map.org/grid_data/download/100042271?include=energy
Axl	p28	-1.370	1	http://api.brain-map.org/grid_data/download/100037824?include=energy
Bmpr2	p28	-0.819	1	http://api.brain-map.org/grid_data/download/100042306?include=energy
Cacna1h	p28	-0.869	1	http://api.brain-map.org/grid_data/download/100101227?include=energy
Casp3	p28	-0.591	1	http://api.brain-map.org/grid_data/download/100083566?include=energy
Cdh1	p28	-0.552	1	http://api.brain-map.org/grid_data/download/100042112?include=energy
Cntn1	p28	0.021	1	http://api.brain-map.org/grid_data/download/100035735?include=energy
Cxcl12	p28	-0.587	1	http://api.brain-map.org/grid_data/download/100093853?include=energy
Cxcr4	p28	-1.026	1	http://api.brain-map.org/grid_data/download/100083007?include=energy
Ddr1	p28	-0.060	1	http://api.brain-map.org/grid_data/download/100039052?include=energy
Ephb2	p28	-0.698	1	http://api.brain-map.org/grid_data/download/100038883?include=energy
Fbxw4	p28	-0.825	1	http://api.brain-map.org/grid_data/download/100040904?include=energy
Fgf23	p28	-0.805	1	http://api.brain-map.org/grid_data/download/100074091?include=energy
Fmr1	p28	-1.194	1	http://api.brain-map.org/grid_data/download/100045482?include=energy
Fn1	p28	-0.694	1	http://api.brain-map.org/grid_data/download/100041187?include=energy
Fyn	p28	-0.584	1	http://api.brain-map.org/grid_data/download/100070241?include=energy
Gap43	p28	-1.187	1	http://api.brain-map.org/grid_data/download/100034930?include=energy
Gata3	p28	-0.792	1	http://api.brain-map.org/grid_data/download/100093821?include=energy
Ghsr	p28	-1.035	1	http://api.brain-map.org/grid_data/download/100036357?include=energy
Gria2	p28	-1.320	1	http://api.brain-map.org/grid_data/download/100041810?include=energy
Grm3	p28	-0.816	1	http://api.brain-map.org/grid_data/download/100039786?include=energy
Grm8	p28	-0.855	1	http://api.brain-map.org/grid_data/download/100041536?include=energy
Hfe	p28	-0.921	1	http://api.brain-map.org/grid_data/download/100083087?include=energy
Hrh1	p28	-0.629	1	http://api.brain-map.org/grid_data/download/100014110?include=energy
Mc4r	p28	-0.599	1	http://api.brain-map.org/grid_data/download/100039197?include=energy
Ncam1	p28	-0.634	1	http://api.brain-map.org/grid_data/download/100045214?include=energy
Nfkbia	p28	-0.792	1	http://api.brain-map.org/grid_data/download/100042447?include=energy
Nfya	p28	-0.686	1	http://api.brain-map.org/grid_data/download/100053481?include=energy
Nos1	p28	-1.081	1	http://api.brain-map.org/grid_data/download/100042449?include=energy
Notch3	p28	-0.740	1	http://api.brain-map.org/grid_data/download/100039204?include=energy
Npy	p28	-0.642	1	http://api.brain-map.org/grid_data/download/100035670?include=energy
Nr4a3	p28	-0.198	1	http://api.brain-map.org/grid_data/download/100053516?include=energy
Nrxn1	p28	-1.274	1	http://api.brain-map.org/grid_data/download/100041824?include=energy
Ntf3	p28	-0.448	1	http://api.brain-map.org/grid_data/download/100056888?include=energy
Ntn1	p28	-0.693	1	http://api.brain-map.org/grid_data/download/100042417?include=energy
Plxna2	p28	-1.317	1	http://api.brain-map.org/grid_data/download/100042115?include=energy
Plxna3	p28	-0.030	1	http://api.brain-map.org/grid_data/download/100082513?include=energy
Polr1d	p28	1.057	1	http://api.brain-map.org/grid_data/download/166338612?include=energy
Rara	p28	-1.180	1	http://api.brain-map.org/grid_data/download/100070235?include=energy
Reln	p28	-1.128	1	http://api.brain-map.org/grid_data/download/100038825?include=energy
Rtn4r	p28	-1.206	1	http://api.brain-map.org/grid_data/download/100041832?include=energy
Sema3a	p28	-0.979	1	http://api.brain-map.org/grid_data/download/100038791?include=energy
Sema4a	p28	-0.619	1	http://api.brain-map.org/grid_data/download/100036011?include=energy
Slc1a3	p28	-0.796	1	http://api.brain-map.org/grid_data/download/100083582?include=energy
Slc1a4	p28	-0.604	1	http://api.brain-map.org/grid_data/download/100083013?include=energy
Slc6a4	p28	-0.574	1	http://api.brain-map.org/grid_data/download/100045653?include=energy
Sp3	p28	0.484	1	http://api.brain-map.org/grid_data/download/100052965?include=energy
Ssrp1	p28	-1.106	1	http://api.brain-map.org/grid_data/download/100070278?include=energy
Stx1a	p28	-1.219	1	http://api.brain-map.org/grid_data/download/100074404?include=energy
Trpv1	p28	-0.638	1	http://api.brain-map.org/grid_data/download/100041081?include=energy
Vegfa	p28	-0.594	1	http://api.brain-map.org/grid_data/download/100083535?include=energy
Ache	p28	0.109	2	http://api.brain-map.org/grid_data/download/100010054?include=energy
Aff3	p28	-0.868	2	http://api.brain-map.org/grid_data/download/100093846?include=energy
Agt	p28	-0.220	2	http://api.brain-map.org/grid_data/download/100039262?include=energy
Amh	p28	-0.067	2	http://api.brain-map.org/grid_data/download/100042191?include=energy
App	p28	-1.114	2	http://api.brain-map.org/grid_data/download/100038836?include=energy
Aqp4	p28	-0.072	2	http://api.brain-map.org/grid_data/download/100031644?include=energy
Bach2	p28	-0.490	2	http://api.brain-map.org/grid_data/download/100053392?include=energy
Bmp2	p28	-1.107	2	http://api.brain-map.org/grid_data/download/100042632?include=energy
Bmp4	p28	-0.467	2	http://api.brain-map.org/grid_data/download/100031512?include=energy
C1qa	p28	-0.497	2	http://api.brain-map.org/grid_data/download/100083015?include=energy
Cd44	p28	-0.722	2	http://api.brain-map.org/grid_data/download/100039259?include=energy
Cdh3	p28	-0.520	2	http://api.brain-map.org/grid_data/download/100053504?include=energy
Cic	p28	-1.161	2	http://api.brain-map.org/grid_data/download/100071346?include=energy
Cldn5	p28	-0.640	2	http://api.brain-map.org/grid_data/download/100039328?include=energy
Clock	p28	-0.636	2	http://api.brain-map.org/grid_data/download/100035732?include=energy
Clstn2	p28	-0.564	2	http://api.brain-map.org/grid_data/download/100053609?include=energy
Clu	p28	0.643	2	http://api.brain-map.org/grid_data/download/100045701?include=energy
Col11a2	p28	-0.153	2	http://api.brain-map.org/grid_data/download/100056570?include=energy
Col2a1	p28	-0.480	2	http://api.brain-map.org/grid_data/download/100053499?include=energy
Creb1	p28	-0.533	2	http://api.brain-map.org/grid_data/download/100036364?include=energy
Crhr1	p28	-0.059	2	http://api.brain-map.org/grid_data/download/100056857?include=energy
Csf1r	p28	-1.444	2	http://api.brain-map.org/grid_data/download/100041825?include=energy
Ctnnb1	p28	0.000	2	http://api.brain-map.org/grid_data/download/100035648?include=energy
Cyp7b1	p28	-0.285	2	http://api.brain-map.org/grid_data/download/100070148?include=energy
Dbp	p28	0.133	2	http://api.brain-map.org/grid_data/download/100056443?include=energy
Dll1	p28	-0.765	2	http://api.brain-map.org/grid_data/download/100093638?include=energy
Dll4	p28	-1.023	2	http://api.brain-map.org/grid_data/download/100041705?include=energy
Ebf1	p28	-0.734	2	http://api.brain-map.org/grid_data/download/100093811?include=energy
Ednrb	p28	-0.717	2	http://api.brain-map.org/grid_data/download/100010080?include=energy
Egf	p28	-0.329	2	http://api.brain-map.org/grid_data/download/100034748?include=energy
Egfr	p28	-1.085	2	http://api.brain-map.org/grid_data/download/100010081?include=energy
Epha4	p28	0.341	2	http://api.brain-map.org/grid_data/download/100045552?include=energy
Erbb3	p28	0.018	2	http://api.brain-map.org/grid_data/download/100045501?include=energy
Ets1	p28	-0.913	2	http://api.brain-map.org/grid_data/download/100056319?include=energy
Fgf1	p28	-0.829	2	http://api.brain-map.org/grid_data/download/100056893?include=energy
Foxc1	p28	-0.216	2	http://api.brain-map.org/grid_data/download/100045288?include=energy
Foxo3	p28	0.807	2	http://api.brain-map.org/grid_data/download/100056481?include=energy
Fzd4	p28	-0.474	2	http://api.brain-map.org/grid_data/download/100034867?include=energy
Gal	p28	-0.921	2	http://api.brain-map.org/grid_data/download/100041788?include=energy
Gdf15	p28	-0.270	2	http://api.brain-map.org/grid_data/download/100045567?include=energy
Gfap	p28	-0.852	2	http://api.brain-map.org/grid_data/download/100045591?include=energy
Gja1	p28	0.372	2	http://api.brain-map.org/grid_data/download/100039245?include=energy
Gli1	p28	-0.676	2	http://api.brain-map.org/grid_data/download/100045606?include=energy
Glis3	p28	-0.661	2	http://api.brain-map.org/grid_data/download/100053610?include=energy
Grm1	p28	-0.347	2	http://api.brain-map.org/grid_data/download/100038888?include=energy
Hcrt	p28	-0.374	2	http://api.brain-map.org/grid_data/download/100045534?include=energy
Hspb8	p28	0.717	2	http://api.brain-map.org/grid_data/download/100040813?include=energy
Igf1	p28	-0.784	2	http://api.brain-map.org/grid_data/download/100045632?include=energy
Igfbp1	p28	-0.226	2	http://api.brain-map.org/grid_data/download/100034861?include=energy
Igfbp3	p28	-0.877	2	http://api.brain-map.org/grid_data/download/100074360?include=energy
Ikzf1	p28	-0.028	2	http://api.brain-map.org/grid_data/download/100056527?include=energy
Insrr	p28	-0.502	2	http://api.brain-map.org/grid_data/download/100057737?include=energy
Irf1	p28	0.214	2	http://api.brain-map.org/grid_data/download/100051746?include=energy
Itga4	p28	-0.436	2	http://api.brain-map.org/grid_data/download/100056415?include=energy
Itgam	p28	-0.398	2	http://api.brain-map.org/grid_data/download/100075140?include=energy
Jag1	p28	-0.786	2	http://api.brain-map.org/grid_data/download/100045669?include=energy
Jazf1	p28	0.286	2	http://api.brain-map.org/grid_data/download/100052604?include=energy
Kdr	p28	-0.478	2	http://api.brain-map.org/grid_data/download/100034932?include=energy
Lgals1	p28	-0.503	2	http://api.brain-map.org/grid_data/download/100040756?include=energy
Lpl	p28	-0.391	2	http://api.brain-map.org/grid_data/download/100040778?include=energy
Mapk8	p28	-0.596	2	http://api.brain-map.org/grid_data/download/100031944?include=energy
Maz	p28	-0.416	2	http://api.brain-map.org/grid_data/download/100053604?include=energy
Mertk	p28	-0.508	2	http://api.brain-map.org/grid_data/download/100042420?include=energy
Mst1	p28	-0.878	2	http://api.brain-map.org/grid_data/download/100074424?include=energy
Mtor	p28	-0.079	2	http://api.brain-map.org/grid_data/download/100041696?include=energy
Mycn	p28	-0.700	2	http://api.brain-map.org/grid_data/download/100053539?include=energy
Myt1	p28	0.049	2	http://api.brain-map.org/grid_data/download/100053011?include=energy
Nfe2l2	p28	0.182	2	http://api.brain-map.org/grid_data/download/100053098?include=energy
Nfkb1	p28	-0.088	2	http://api.brain-map.org/grid_data/download/100052823?include=energy
Nog	p28	-0.518	2	http://api.brain-map.org/grid_data/download/100053396?include=energy
Notch2	p28	-0.714	2	http://api.brain-map.org/grid_data/download/100101152?include=energy
Notch4	p28	-0.576	2	http://api.brain-map.org/grid_data/download/100049601?include=energy
Nr1h3	p28	-0.125	2	http://api.brain-map.org/grid_data/download/100070104?include=energy
Nr3c2	p28	1.098	2	http://api.brain-map.org/grid_data/download/100056520?include=energy
Olig1	p28	0.624	2	http://api.brain-map.org/grid_data/download/100053477?include=energy
P2rx7	p28	-0.496	2	http://api.brain-map.org/grid_data/download/100045245?include=energy
Pml	p28	-0.075	2	http://api.brain-map.org/grid_data/download/100053060?include=energy
Pou5f1	p28	-0.049	2	http://api.brain-map.org/grid_data/download/100071901?include=energy
Ppargc1a	p28	-0.654	2	http://api.brain-map.org/grid_data/download/100053506?include=energy
Psen1	p28	0.028	2	http://api.brain-map.org/grid_data/download/100041685?include=energy
Rbpj	p28	-0.722	2	http://api.brain-map.org/grid_data/download/100041717?include=energy
Robo3	p28	-0.020	2	http://api.brain-map.org/grid_data/download/100049600?include=energy
Sall1	p28	-0.159	2	http://api.brain-map.org/grid_data/download/166341395?include=energy
Selplg	p28	-0.052	2	http://api.brain-map.org/grid_data/download/100053501?include=energy
Sema7a	p28	0.098	2	http://api.brain-map.org/grid_data/download/100041784?include=energy
Serpina3k	p28	-0.722	2	http://api.brain-map.org/grid_data/download/100040801?include=energy
Slc16a2	p28	-0.637	2	http://api.brain-map.org/grid_data/download/100082516?include=energy
Slc40a1	p28	-0.761	2	http://api.brain-map.org/grid_data/download/100070353?include=energy
Snca	p28	0.485	2	http://api.brain-map.org/grid_data/download/100039221?include=energy
Sox8	p28	0.390	2	http://api.brain-map.org/grid_data/download/100053313?include=energy
Sstr4	p28	-0.390	2	http://api.brain-map.org/grid_data/download/100045254?include=energy
Stat4	p28	-0.404	2	http://api.brain-map.org/grid_data/download/100053337?include=energy
Tbx21	p28	-0.568	2	http://api.brain-map.org/grid_data/download/100053606?include=energy
Tgfa	p28	-0.756	2	http://api.brain-map.org/grid_data/download/100042512?include=energy
Tgfb1	p28	-1.035	2	http://api.brain-map.org/grid_data/download/100039721?include=energy
Tgfb2	p28	-0.378	2	http://api.brain-map.org/grid_data/download/100039237?include=energy
Tgfbr1	p28	-0.484	2	http://api.brain-map.org/grid_data/download/100036399?include=energy
Th	p28	-1.353	2	http://api.brain-map.org/grid_data/download/100038912?include=energy
Tmem119	p28	0.020	2	http://api.brain-map.org/grid_data/download/100090794?include=energy
Tph2	p28	-0.420	2	http://api.brain-map.org/grid_data/download/100045170?include=energy
Trib2	p28	-0.641	2	http://api.brain-map.org/grid_data/download/100035748?include=energy
Trp53	p28	-0.247	2	http://api.brain-map.org/grid_data/download/100051747?include=energy
Vdr	p28	-0.557	2	http://api.brain-map.org/grid_data/download/100073994?include=energy
Vipr2	p28	0.463	2	http://api.brain-map.org/grid_data/download/100075143?include=energy
Wnt3	p28	-0.436	2	http://api.brain-map.org/grid_data/download/100045592?include=energy
Xbp1	p28	0.601	2	http://api.brain-map.org/grid_data/download/100053125?include=energy

Zfp36l1	p28	0.399	2	http://api.brain-map.org/grid_data/download/166340204?include=energy
A2m	p28	1.004	3	http://api.brain-map.org/grid_data/download/100082996?include=energy
Abl1	p28	0.511	3	http://api.brain-map.org/grid_data/download/100083022?include=energy
Ace	p28	1.279	3	http://api.brain-map.org/grid_data/download/100083019?include=energy
Actb	p28	0.399	3	http://api.brain-map.org/grid_data/download/100045117?include=energy
Anxa2	p28	-0.235	3	http://api.brain-map.org/grid_data/download/100039353?include=energy
Arnt2	p28	0.019	3	http://api.brain-map.org/grid_data/download/100053039?include=energy
Atf4	p28	0.832	3	http://api.brain-map.org/grid_data/download/100052608?include=energy
Bdnf	p28	-0.341	3	http://api.brain-map.org/grid_data/download/100040834?include=energy
Bmp1	p28	0.145	3	http://api.brain-map.org/grid_data/download/100042304?include=energy
Bmp7	p28	1.164	3	http://api.brain-map.org/grid_data/download/100074090?include=energy
Car2	p28	0.653	3	http://api.brain-map.org/grid_data/download/100070336?include=energy
Cartpt	p28	1.064	3	http://api.brain-map.org/grid_data/download/100074009?include=energy
Cldn11	p28	0.724	3	http://api.brain-map.org/grid_data/download/100045631?include=energy
Cnp	p28	0.347	3	http://api.brain-map.org/grid_data/download/100070352?include=energy
Comt	p28	0.807	3	http://api.brain-map.org/grid_data/download/100010068?include=energy
Cx3cl1	p28	0.657	3	http://api.brain-map.org/grid_data/download/100051742?include=energy
Dcn	p28	0.118	3	http://api.brain-map.org/grid_data/download/100011593?include=energy
Dlk1	p28	0.771	3	http://api.brain-map.org/grid_data/download/100074049?include=energy
Drd2	p28	0.375	3	http://api.brain-map.org/grid_data/download/100010075?include=energy
Egr1	p28	0.276	3	http://api.brain-map.org/grid_data/download/100041604?include=energy
Eomes	p28	1.452	3	http://api.brain-map.org/grid_data/download/100056416?include=energy
Epas1	p28	0.054	3	http://api.brain-map.org/grid_data/download/100052877?include=energy
Fgfr1	p28	-0.555	3	http://api.brain-map.org/grid_data/download/100040785?include=energy
Fos	p28	0.026	3	http://api.brain-map.org/grid_data/download/100040860?include=energy
Gad1	p28	-0.508	3	http://api.brain-map.org/grid_data/download/100039955?include=energy
Gad2	p28	0.279	3	http://api.brain-map.org/grid_data/download/100040752?include=energy
Gpr37	p28	0.937	3	http://api.brain-map.org/grid_data/download/100039821?include=energy
Gria3	p28	0.409	3	http://api.brain-map.org/grid_data/download/100041178?include=energy
Gria4	p28	-0.185	3	http://api.brain-map.org/grid_data/download/100040936?include=energy
Grin2b	p28	0.207	3	http://api.brain-map.org/grid_data/download/100041435?include=energy
Grm5	p28	-0.327	3	http://api.brain-map.org/grid_data/download/100039417?include=energy
Hif1a	p28	0.837	3	http://api.brain-map.org/grid_data/download/100053117?include=energy
Icam5	p28	0.258	3	http://api.brain-map.org/grid_data/download/100053410?include=energy
Inpp4b	p28	-0.545	3	http://api.brain-map.org/grid_data/download/100041057?include=energy
Lepr	p28	1.401	3	http://api.brain-map.org/grid_data/download/100045347?include=energy
Lrrk2	p28	1.392	3	http://api.brain-map.org/grid_data/download/100075414?include=energy
Mog	p28	0.888	3	http://api.brain-map.org/grid_data/download/100083554?include=energy
Ncoa2	p28	-0.453	3	http://api.brain-map.org/grid_data/download/100052942?include=energy
Nfasc	p28	-0.025	3	http://api.brain-map.org/grid_data/download/100053017?include=energy
Ngf	p28	0.191	3	http://api.brain-map.org/grid_data/download/100034862?include=energy
Nr3c1	p28	1.150	3	http://api.brain-map.org/grid_data/download/100040050?include=energy
Nr4a1	p28	0.203	3	http://api.brain-map.org/grid_data/download/100039229?include=energy
Ostf1	p28	-0.543	3	http://api.brain-map.org/grid_data/download/100053329?include=energy
Plp1	p28	0.068	3	http://api.brain-map.org/grid_data/download/100039826?include=energy
Pomc	p28	-0.422	3	http://api.brain-map.org/grid_data/download/100074416?include=energy
Prlr	p28	0.626	3	http://api.brain-map.org/grid_data/download/100034996?include=energy
Prnp	p28	0.463	3	http://api.brain-map.org/grid_data/download/100034640?include=energy
Rac2	p28	1.382	3	http://api.brain-map.org/grid_data/download/100082991?include=energy
Rora	p28	0.405	3	http://api.brain-map.org/grid_data/download/100040008?include=energy
Rreb1	p28	0.220	3	http://api.brain-map.org/grid_data/download/100041532?include=energy
S100b	p28	0.114	3	http://api.brain-map.org/grid_data/download/100045237?include=energy
Slc1a2	p28	-0.129	3	http://api.brain-map.org/grid_data/download/100042555?include=energy
Smad4	p28	-0.562	3	http://api.brain-map.org/grid_data/download/100045536?include=energy
Sorl1	p28	0.053	3	http://api.brain-map.org/grid_data/download/100083078?include=energy
Sox10	p28	0.759	3	http://api.brain-map.org/grid_data/download/100034749?include=energy
Sox2	p28	1.037	3	http://api.brain-map.org/grid_data/download/100049605?include=energy
Spp1	p28	-0.047	3	http://api.brain-map.org/grid_data/download/100039260?include=energy
Stat1	p28	-0.235	3	http://api.brain-map.org/grid_data/download/100052971?include=energy
Stat5b	p28	1.377	3	http://api.brain-map.org/grid_data/download/100053110?include=energy
Syn1	p28	0.312	3	http://api.brain-map.org/grid_data/download/100039727?include=energy
Syt1	p28	-0.056	3	http://api.brain-map.org/grid_data/download/100045511?include=energy
Tac1	p28	0.358	3	http://api.brain-map.org/grid_data/download/100056865?include=energy
Tac2	p28	-0.057	3	http://api.brain-map.org/grid_data/download/100040644?include=energy
Tef	p28	0.858	3	http://api.brain-map.org/grid_data/download/100055193?include=energy
Trp63	p28	-0.446	3	http://api.brain-map.org/grid_data/download/100074116?include=energy
Vip	p28	0.707	3	http://api.brain-map.org/grid_data/download/100049592?include=energy
Adam17	p28	-0.615	4	http://api.brain-map.org/grid_data/download/100041641?include=energy
Agtr1a	p28	-0.955	4	http://api.brain-map.org/grid_data/download/100010072?include=energy
Ahr	p28	-0.542	4	http://api.brain-map.org/grid_data/download/100071147?include=energy
Akt1	p28	0.587	4	http://api.brain-map.org/grid_data/download/100071194?include=energy
Apod	p28	0.214	4	http://api.brain-map.org/grid_data/download/100083095?include=energy
Apoe	p28	-0.730	4	http://api.brain-map.org/grid_data/download/100039959?include=energy
Ar	p28	-0.461	4	http://api.brain-map.org/grid_data/download/100010088?include=energy
Arntl	p28	-0.620	4	http://api.brain-map.org/grid_data/download/100056318?include=energy
Ascl2	p28	-0.513	4	http://api.brain-map.org/grid_data/download/100070230?include=energy
Atf3	p28	-0.624	4	http://api.brain-map.org/grid_data/download/100070231?include=energy
Bche	p28	-0.639	4	http://api.brain-map.org/grid_data/download/100010083?include=energy
Bcl2	p28	-1.130	4	http://api.brain-map.org/grid_data/download/100074121?include=energy
Bmp5	p28	-0.473	4	http://api.brain-map.org/grid_data/download/100042301?include=energy
Brca1	p28	-1.015	4	http://api.brain-map.org/grid_data/download/166423327?include=energy
Cd47	p28	0.080	4	http://api.brain-map.org/grid_data/download/100052904?include=energy
Cdh17	p28	0.613	4	http://api.brain-map.org/grid_data/download/100074055?include=energy
Cdx2	p28	-0.513	4	http://api.brain-map.org/grid_data/download/100071125?include=energy
Chat	p28	-0.275	4	http://api.brain-map.org/grid_data/download/100045750?include=energy
Cnr1	p28	-0.864	4	http://api.brain-map.org/grid_data/download/100010069?include=energy
Cnr2	p28	-0.767	4	http://api.brain-map.org/grid_data/download/100031589?include=energy
Cst3	p28	-0.603	4	http://api.brain-map.org/grid_data/download/100041664?include=energy
Cux2	p28	-1.213	4	http://api.brain-map.org/grid_data/download/100041541?include=energy
Ddit3	p28	-0.558	4	http://api.brain-map.org/grid_data/download/100074321?include=energy
Erg	p28	-1.072	4	http://api.brain-map.org/grid_data/download/100056479?include=energy
Esr1	p28	-0.150	4	http://api.brain-map.org/grid_data/download/100010074?include=energy
Esr2	p28	-0.420	4	http://api.brain-map.org/grid_data/download/100010087?include=energy
Evi5	p28	-0.301	4	http://api.brain-map.org/grid_data/download/100045187?include=energy
Fas	p28	-0.481	4	http://api.brain-map.org/grid_data/download/100083093?include=energy
Foxp3	p28	-0.621	4	http://api.brain-map.org/grid_data/download/100071126?include=energy
Gabpa	p28	-0.485	4	http://api.brain-map.org/grid_data/download/100074422?include=energy
Gabra3	p28	-0.572	4	http://api.brain-map.org/grid_data/download/100010085?include=energy
Galr2	p28	-0.750	4	http://api.brain-map.org/grid_data/download/100083590?include=energy
Grm4	p28	-0.604	4	http://api.brain-map.org/grid_data/download/100041527?include=energy
Hap1	p28	-1.437	4	http://api.brain-map.org/grid_data/download/100039446?include=energy
Hoxb4	p28	0.022	4	http://api.brain-map.org/grid_data/download/100053558?include=energy
Hrh3	p28	-0.171	4	http://api.brain-map.org/grid_data/download/100101272?include=energy
Hspd1	p28	-0.863	4	http://api.brain-map.org/grid_data/download/100083630?include=energy
Igf1r	p28	-1.155	4	http://api.brain-map.org/grid_data/download/100042543?include=energy
Il16	p28	-0.150	4	http://api.brain-map.org/grid_data/download/100036511?include=energy
Il6st	p28	0.619	4	http://api.brain-map.org/grid_data/download/100074129?include=energy
Itln1	p28	-0.370	4	http://api.brain-map.org/grid_data/download/100074106?include=energy
Kcnh7	p28	-0.155	4	http://api.brain-map.org/grid_data/download/100011611?include=energy
Lrp1	p28	-0.841	4	http://api.brain-map.org/grid_data/download/100083071?include=energy
Mapt	p28	-1.047	4	http://api.brain-map.org/grid_data/download/100074001?include=energy
Mbp	p28	0.004	4	http://api.brain-map.org/grid_data/download/100035003?include=energy
Mef2b	p28	-0.295	4	http://api.brain-map.org/grid_data/download/100074043?include=energy
Mmp9	p28	-0.570	4	http://api.brain-map.org/grid_data/download/100090846?include=energy
Msc	p28	-0.516	4	http://api.brain-map.org/grid_data/download/100071255?include=energy
Mst1r	p28	-0.677	4	http://api.brain-map.org/grid_data/download/100040090?include=energy
Nfatc2	p28	-0.612	4	http://api.brain-map.org/grid_data/download/100035659?include=energy
Nos3	p28	-1.190	4	http://api.brain-map.org/grid_data/download/100083011?include=energy
Notch1	p28	-0.991	4	http://api.brain-map.org/grid_data/download/100037893?include=energy
Nr0b2	p28	-0.779	4	http://api.brain-map.org/grid_data/download/100070152?include=energy
Nr1i2	p28	-1.233	4	http://api.brain-map.org/grid_data/download/100053603?include=energy
Nr4a2	p28	-0.315	4	http://api.brain-map.org/grid_data/download/100075410?include=energy
P2rx4	p28	-0.511	4	http://api.brain-map.org/grid_data/download/100090884?include=energy
Pik3cb	p28	-0.529	4	http://api.brain-map.org/grid_data/download/100053448?include=energy
Pou2af1	p28	-0.586	4	http://api.brain-map.org/grid_data/download/100070158?include=energy
Ppara	p28	-0.757	4	http://api.brain-map.org/grid_data/download/100070109?include=energy
Pparg	p28	-0.310	4	http://api.brain-map.org/grid_data/download/100034863?include=energy
Prkcb	p28	-0.137	4	http://api.brain-map.org/grid_data/download/100070339?include=energy
Ptgs2	p28	-0.191	4	http://api.brain-map.org/grid_data/download/100014096?include=energy
Pthlh	p28	-0.506	4	http://api.brain-map.org/grid_data/download/100041789?include=energy
Ptprc	p28	-0.276	4	http://api.brain-map.org/grid_data/download/100053441?include=energy
Pvalb	p28	0.087	4	http://api.brain-map.org/grid_data/download/100039969?include=energy
Rela	p28	-0.591	4	http://api.brain-map.org/grid_data/download/100052750?include=energy
Ros1	p28	-0.527	4	http://api.brain-map.org/grid_data/download/100093852?include=energy
S1pr1	p28	-0.821	4	http://api.brain-map.org/grid_data/download/100041849?include=energy
Scn10a	p28	-0.509	4	http://api.brain-map.org/grid_data/download/100014149?include=energy
Sgk1	p28	-1.068	4	http://api.brain-map.org/grid_data/download/100040799?include=energy
Shh	p28	-0.749	4	http://api.brain-map.org/grid_data/download/100101226?include=energy
Slc1a1	p28	-0.450	4	http://api.brain-map.org/grid_data/download/100040708?include=energy
Slc9a9	p28	0.453	4	http://api.brain-map.org/grid_data/download/100083092?include=energy
Smad7	p28	-0.517	4	http://api.brain-map.org/grid_data/download/100037870?include=energy
Sort1	p28	-0.311	4	http://api.brain-map.org/grid_data/download/100083072?include=energy
Stat3	p28	-0.314	4	http://api.brain-map.org/grid_data/download/100052973?include=energy
Stat5a	p28	0.752	4	http://api.brain-map.org/grid_data/download/100074323?include=energy
Tdo2	p28	-0.761	4	http://api.brain-map.org/grid_data/download/100071310?include=energy
Tgfb3	p28	-0.468	4	http://api.brain-map.org/grid_data/download/100034931?include=energy
Tlr2	p28	-0.733	4	http://api.brain-map.org/grid_data/download/100083628?include=energy
Trp73	p28	-0.448	4	http://api.brain-map.org/grid_data/download/100074375?include=energy
Tspan31	p28	-0.907	4	http://api.brain-map.org/grid_data/download/100090878?include=energy
Tyro3	p28	-0.431	4	http://api.brain-map.org/grid_data/download/100041200?include=energy
Ubtf	p28	0.176	4	http://api.brain-map.org/grid_data/download/100071204?include=energy
Vav2	p28	-1.420	4	http://api.brain-map.org/grid_data/download/100011621?include=energy
Zeb1	p28	-0.539	4	http://api.brain-map.org/grid_data/download/100053330?include=energy
Zic1	p28	-0.162	4	http://api.brain-map.org/grid_data/download/100035750?include=energy
Adcyap1	p56	-0.148	1	http://api.brain-map.org/grid_data/download/74511882?include=energy
Adnp	p56	-1.368	1	http://api.brain-map.org/grid_data/download/70919764?include=energy
Adra2b	p56	-0.639	1	http://api.brain-map.org/grid_data/download/74724532?include=energy
Ahi1	p56	-0.097	1	http://api.brain-map.org/grid_data/download/69549642?include=energy
Alcam	p56	-0.521	1	http://api.brain-map.org/grid_data/download/70928271?include=energy
Angpt2	p56	-0.620	1	http://api.brain-map.org/grid_data/download/68633002?include=energy
Angptl4	p56	-0.546	1	http://api.brain-map.org/grid_data/download/71723863?include=energy
Apc	p56	0.469	1	http://api.brain-map.org/grid_data/download/74881517?include=energy
Axl	p56	-0.087	1	http://api.brain-map.org/grid_data/download/68798199?include=energy
Bmpr2	p56	0.806	1	http://api.brain-map.org/grid_data/download/69529382?include=energy
Cacna1h	p56	-0.534	1	http://api.brain-map.org/grid_data/download/72119638?include=energy
Casp3	p56	0.121	1	http://api.brain-map.org/grid_data/download/70431217?include=energy
Cdh1	p56	-0.535	1	http://api.brain-map.org/grid_data/download/69540671?include=energy
Cntn1	p56	-1.397	1	http://api.brain-map.org/grid_data/download/414?include=energy
Cxcl12	p56	-1.076	1	http://api.brain-map.org/grid_data/download/308?include=energy
Cxcr4	p56	-0.685	1	http://api.brain-map.org/grid_data/download/100144881?include=energy
Ddr1	p56	-1.393	1	http://api.brain-map.org/grid_data/download/69626705?include=energy
Ephb2	p56	-0.198	1	http://api.brain-map.org/grid_data/download/69672096?include=energy
Fbxw4	p56	-0.734	1	http://api.brain-map.org/grid_data/download/68441616?include=energy
Fgf23	p56	0.287	1	http://api.brain-map.org/grid_data/download/81655572?include=energy
Fmr1	p56	-0.424	1	http://api.brain-map.org/grid_data/download/73606703?include=energy
Fn1	p56	-0.990	1	http://api.brain-map.org/grid_data/download/72119593?include=energy
Fyn	p56	0.315	1	http://api.brain-map.org/grid_data/download/70615595?include=energy
Gap43	p56	-0.467	1	http://api.brain-map.org/grid_data/download/70928270?include=energy
Gata3	p56	-0.451	1	http://api.brain-map.org/grid_data/download/73931427?include=energy
Ghsr	p56	-0.674	1	http://api.brain-map.org/grid_data/download/74511739?include=energy
Gria2	p56	0.790	1	http://api.brain-map.org/grid_data/download/75551462?include=energy
Grm3	p56	-0.398	1	http://api.brain-map.org/grid_data/download/539?include=energy
Grm8	p56	-0.820	1	http://api.brain-map.org/grid_data/download/73771227?include=energy
Hfe	p56	0.220	1	http://api.brain-map.org/grid_data/download/70277943?include=energy
Hrh1	p56	-0.399	1	http://api.brain-map.org/grid_data/download/72283792?include=energy
Mc4r	p56	-0.946	1	http://api.brain-map.org/grid_data/download/79556630?include=energy
Ncam1	p56	-0.842	1	http://api.brain-map.org/grid_data/download/80342134?include=energy
Nfkbia	p56	0.191	1	http://api.brain-map.org/grid_data/download/69782222?include=energy
Nfya	p56	-1.018	1	http://api.brain-map.org/grid_data/download/71763504?include=energy
Nos1	p56	0.188	1	http://api.brain-map.org/grid_data/download/75147762?include=energy
Notch3	p56	-0.850	1	http://api.brain-map.org/grid_data/download/1789?include=energy
Npy	p56	-0.760	1	http://api.brain-map.org/grid_data/download/717?include=energy
Nr4a3	p56	-0.675	1	http://api.brain-map.org/grid_data/download/73615793?include=energy
Nrxn1	p56	-0.215	1	http://api.brain-map.org/grid_data/download/70301083?include=energy
Ntf3	p56	-0.552	1	http://api.brain-map.org/grid_data/download/2440?include=energy
Ntn1	p56	-0.619	1	http://api.brain-map.org/grid_data/download/74511838?include=energy
Plxna2	p56	-0.060	1	http://api.brain-map.org/grid_data/download/70431431?include=energy
Plxna3	p56	-1.006	1	http://api.brain-map.org/grid_data/download/1403?include=energy
Polr1d	p56	-1.185	1	http://api.brain-map.org/grid_data/download/68499368?include=energy
Rara	p56	0.707	1	http://api.brain-map.org/grid_data/download/1529?include=energy
Reln	p56	-0.449	1	http://api.brain-map.org/grid_data/download/890?include=energy
Rtn4r	p56	0.515	1	http://api.brain-map.org/grid_data/download/72080145?include=energy
Sema3a	p56	-0.722	1	http://api.brain-map.org/grid_data/download/938?include=energy
Sema4a	p56	-1.073	1	http://api.brain-map.org/grid_data/download/942?include=energy
Slc1a3	p56	-0.653	1	http://api.brain-map.org/grid_data/download/77620233?include=energy
Slc1a4	p56	-0.471	1	http://api.brain-map.org/grid_data/download/68920797?include=energy
Slc6a4	p56	-0.979	1	http://api.brain-map.org/grid_data/download/2624?include=energy
Sp3	p56	-1.388	1	http://api.brain-map.org/grid_data/download/74822606?include=energy
Ssrp1	p56	0.723	1	http://api.brain-map.org/grid_data/download/69838048?include=energy
Stx1a	p56	-0.392	1	http://api.brain-map.org/grid_data/download/2645?include=energy
Trpv1	p56	-0.985	1	http://api.brain-map.org/grid_data/download/80342023?include=energy
Vegfa	p56	0.792	1	http://api.brain-map.org/grid_data/download/74988747?include=energy
Ache	p56	-0.454	2	http://api.brain-map.org/grid_data/download/70301082?include=energy
Aff3	p56	0.109	2	http://api.brain-map.org/grid_data/download/71656693?include=energy
Agt	p56	-1.097	2	http://api.brain-map.org/grid_data/download/87?include=energy
Amh	p56	-0.876	2	http://api.brain-map.org/grid_data/download/69526577?include=energy
App	p56	0.220	2	http://api.brain-map.org/grid_data/download/107?include=energy
Aqp4	p56	-0.232	2	http://api.brain-map.org/grid_data/download/108?include=energy
Bach2	p56	-0.652	2	http://api.brain-map.org/grid_data/download/69816488?include=energy
Bmp2	p56	0.644	2	http://api.brain-map.org/grid_data/download/76003511?include=energy
Bmp4	p56	-0.377	2	http://api.brain-map.org/grid_data/download/79669531?include=energy
C1qa	p56	-1.095	2	http://api.brain-map.org/grid_data/download/68191456?include=energy
Cd44	p56	0.222	2	http://api.brain-map.org/grid_data/download/224?include=energy
Cdh3	p56	-0.332	2	http://api.brain-map.org/grid_data/download/1337?include=energy
Cic	p56	-0.068	2	http://api.brain-map.org/grid_data/download/71307244?include=energy
Cldn5	p56	-0.446	2	http://api.brain-map.org/grid_data/download/79490120?include=energy
Clock	p56	-0.378	2	http://api.brain-map.org/grid_data/download/273?include=energy
Clstn2	p56	0.502	2	http://api.brain-map.org/grid_data/download/73636091?include=energy
Clu	p56	-1.416	2	http://api.brain-map.org/grid_data/download/275?include=energy
Col11a2	p56	-0.502	2	http://api.brain-map.org/grid_data/download/74581187?include=energy
Col2a1	p56	-0.289	2	http://api.brain-map.org/grid_data/download/71489805?include=energy
Creb1	p56	0.039	2	http://api.brain-map.org/grid_data/download/69549618?include=energy
Crhr1	p56	-0.130	2	http://api.brain-map.org/grid_data/download/297?include=energy
Csf1r	p56	0.450	2	http://api.brain-map.org/grid_data/download/69608106?include=energy
Ctnnb1	p56	-1.226	2	http://api.brain-map.org/grid_data/download/196?include=energy
Cyp7b1	p56	0.170	2	http://api.brain-map.org/grid_data/download/70927813?include=energy
Dbp	p56	-0.571	2	http://api.brain-map.org/grid_data/download/1193?include=energy
Dll1	p56	0.116	2	http://api.brain-map.org/grid_data/download/77454727?include=energy
Dll4	p56	0.185	2	http://api.brain-map.org/grid_data/download/100144503?include=energy
Ebf1	p56	-0.551	2	http://api.brain-map.org/grid_data/download/100142552?include=energy
Ednrb	p56	-0.461	2	http://api.brain-map.org/grid_data/download/74750017?include=energy
Egf	p56	-0.563	2	http://api.brain-map.org/grid_data/download/80341155?include=energy
Egfr	p56	0.315	2	http://api.brain-map.org/grid_data/download/81599632?include=energy
Epha4	p56	-0.516	2	http://api.brain-map.org/grid_data/download/2187?include=energy
Erbb3	p56	-0.550	2	http://api.brain-map.org/grid_data/download/71496565?include=energy
Ets1	p56	-0.746	2	http://api.brain-map.org/grid_data/download/2199?include=energy
Fgf1	p56	0.763	2	http://api.brain-map.org/grid_data/download/79556599?include=energy
Foxc1	p56	-0.076	2	http://api.brain-map.org/grid_data/download/70431319?include=energy
Foxo3	p56	-0.938	2	http://api.brain-map.org/grid_data/download/73949752?include=energy
Fzd4	p56	-0.102	2	http://api.brain-map.org/grid_data/download/2241?include=energy
Gal	p56	-0.494	2	http://api.brain-map.org/grid_data/download/70231997?include=energy
Gdf15	p56	0.040	2	http://api.brain-map.org/grid_data/download/76081203?include=energy
Gfap	p56	-0.298	2	http://api.brain-map.org/grid_data/download/1357?include=energy
Gja1	p56	-0.665	2	http://api.brain-map.org/grid_data/download/2255?include=energy
Gli1	p56	0.250	2	http://api.brain-map.org/grid_data/download/69289057?include=energy
Glis3	p56	-0.268	2	http://api.brain-map.org/grid_data/download/71587733?include=energy
Grm1	p56	-0.045	2	http://api.brain-map.org/grid_data/download/537?include=energy
Hcrt	p56	-0.591	2	http://api.brain-map.org/grid_data/download/79913316?include=energy
Hspb8	p56	-0.844	2	http://api.brain-map.org/grid_data/download/1181?include=energy
Igf1	p56	-0.568	2	http://api.brain-map.org/grid_data/download/75197528?include=energy
Igfbp1	p56	-0.604	2	http://api.brain-map.org/grid_data/download/513231?include=energy
Igfbp3	p56	-0.755	2	http://api.brain-map.org/grid_data/download/74272920?include=energy
Ikzf1	p56	-0.752	2	http://api.brain-map.org/grid_data/download/1144?include=energy
Insrr	p56	0.558	2	http://api.brain-map.org/grid_data/download/71670480?include=energy
Irf1	p56	-0.526	2	http://api.brain-map.org/grid_data/download/79539604?include=energy
Itga4	p56	-0.503	2	http://api.brain-map.org/grid_data/download/77924520?include=energy
Itgam	p56	-0.583	2	http://api.brain-map.org/grid_data/download/69735604?include=energy

Jag1	p56	-0.289	2	http://api.brain-map.org/grid_data/download/69735646?include=energy
Jazf1	p56	-1.159	2	http://api.brain-map.org/grid_data/download/69113931?include=energy
Kdr	p56	-0.616	2	http://api.brain-map.org/grid_data/download/2348?include=energy
Lgals1	p56	0.132	2	http://api.brain-map.org/grid_data/download/70634417?include=energy
Lpl	p56	-0.621	2	http://api.brain-map.org/grid_data/download/649?include=energy
Mapk8	p56	0.314	2	http://api.brain-map.org/grid_data/download/71063715?include=energy
Maz	p56	0.325	2	http://api.brain-map.org/grid_data/download/71488768?include=energy
Mertk	p56	-0.051	2	http://api.brain-map.org/grid_data/download/69752185?include=energy
Mst1	p56	0.744	2	http://api.brain-map.org/grid_data/download/75934514?include=energy
Mtor	p56	0.010	2	http://api.brain-map.org/grid_data/download/68845741?include=energy
Mycn	p56	-0.704	2	http://api.brain-map.org/grid_data/download/79762720?include=energy
Myt1	p56	-0.690	2	http://api.brain-map.org/grid_data/download/71924077?include=energy
Nfe2l2	p56	-0.827	2	http://api.brain-map.org/grid_data/download/700?include=energy
Nfkb1	p56	-0.307	2	http://api.brain-map.org/grid_data/download/77454688?include=energy
Nog	p56	-0.241	2	http://api.brain-map.org/grid_data/download/75084476?include=energy
Notch2	p56	-0.499	2	http://api.brain-map.org/grid_data/download/69837872?include=energy
Notch4	p56	0.265	2	http://api.brain-map.org/grid_data/download/73818802?include=energy
Nr1h3	p56	-0.727	2	http://api.brain-map.org/grid_data/download/2432?include=energy
Nr3c2	p56	-1.186	2	http://api.brain-map.org/grid_data/download/731?include=energy
Olig1	p56	-0.504	2	http://api.brain-map.org/grid_data/download/70437828?include=energy
P2rx7	p56	-0.265	2	http://api.brain-map.org/grid_data/download/75551477?include=energy
Pml	p56	-0.646	2	http://api.brain-map.org/grid_data/download/68301264?include=energy
Pou5f1	p56	-0.270	2	http://api.brain-map.org/grid_data/download/71325348?include=energy
Ppargc1a	p56	-0.111	2	http://api.brain-map.org/grid_data/download/71022954?include=energy
Psen1	p56	-1.058	2	http://api.brain-map.org/grid_data/download/79760446?include=energy
Rbpj	p56	-0.064	2	http://api.brain-map.org/grid_data/download/69262324?include=energy
Robo3	p56	-0.746	2	http://api.brain-map.org/grid_data/download/72000623?include=energy
Sall1	p56	-0.685	2	http://api.brain-map.org/grid_data/download/69290429?include=energy
Selplg	p56	-0.040	2	http://api.brain-map.org/grid_data/download/74988637?include=energy
Sema7a	p56	-1.397	2	http://api.brain-map.org/grid_data/download/955?include=energy
Serpina3k	p56	0.590	2	http://api.brain-map.org/grid_data/download/72081514?include=energy
Slc16a2	p56	0.110	2	http://api.brain-map.org/grid_data/download/73929597?include=energy
Slc40a1	p56	-0.714	2	http://api.brain-map.org/grid_data/download/79904732?include=energy
Snca	p56	-1.475	2	http://api.brain-map.org/grid_data/download/79904550?include=energy
Sox8	p56	-0.335	2	http://api.brain-map.org/grid_data/download/74988736?include=energy
Sstr4	p56	-0.081	2	http://api.brain-map.org/grid_data/download/73636037?include=energy
Stat4	p56	-0.263	2	http://api.brain-map.org/grid_data/download/70593391?include=energy
Tbx21	p56	-0.385	2	http://api.brain-map.org/grid_data/download/71488852?include=energy
Tgfa	p56	-0.388	2	http://api.brain-map.org/grid_data/download/69980304?include=energy
Tgfb1	p56	-0.185	2	http://api.brain-map.org/grid_data/download/80343319?include=energy
Tgfb2	p56	0.235	2	http://api.brain-map.org/grid_data/download/70927304?include=energy
Tgfbr1	p56	-0.902	2	http://api.brain-map.org/grid_data/download/1054?include=energy
Th	p56	0.220	2	http://api.brain-map.org/grid_data/download/1058?include=energy
Tmem119	p56	-0.177	2	http://api.brain-map.org/grid_data/download/68161453?include=energy
Tph2	p56	-0.577	2	http://api.brain-map.org/grid_data/download/74511925?include=energy
Trib2	p56	-0.969	2	http://api.brain-map.org/grid_data/download/68151385?include=energy
Trp53	p56	-0.897	2	http://api.brain-map.org/grid_data/download/67855414?include=energy
Vdr	p56	-0.364	2	http://api.brain-map.org/grid_data/download/1929?include=energy
Vipr2	p56	-0.658	2	http://api.brain-map.org/grid_data/download/1104?include=energy
Wnt3	p56	-0.644	2	http://api.brain-map.org/grid_data/download/2732?include=energy
Xbp1	p56	-0.695	2	http://api.brain-map.org/grid_data/download/1119?include=energy
Zfp36l1	p56	-1.012	2	http://api.brain-map.org/grid_data/download/70445341?include=energy
A2m	p56	0.672	3	http://api.brain-map.org/grid_data/download/100146086?include=energy
Abl1	p56	0.116	3	http://api.brain-map.org/grid_data/download/68844474?include=energy
Ace	p56	0.291	3	http://api.brain-map.org/grid_data/download/72109489?include=energy
Actb	p56	0.833	3	http://api.brain-map.org/grid_data/download/74881516?include=energy
Anxa2	p56	0.860	3	http://api.brain-map.org/grid_data/download/70928384?include=energy
Arnt2	p56	1.214	3	http://api.brain-map.org/grid_data/download/71358590?include=energy
Atf4	p56	0.727	3	http://api.brain-map.org/grid_data/download/69288650?include=energy
Bdnf	p56	0.534	3	http://api.brain-map.org/grid_data/download/79587720?include=energy
Bmp1	p56	0.626	3	http://api.brain-map.org/grid_data/download/69529352?include=energy
Bmp7	p56	-0.615	3	http://api.brain-map.org/grid_data/download/2065?include=energy
Car2	p56	1.036	3	http://api.brain-map.org/grid_data/download/69014434?include=energy
Cartpt	p56	-0.022	3	http://api.brain-map.org/grid_data/download/72077479?include=energy
Cldn11	p56	0.613	3	http://api.brain-map.org/grid_data/download/77280580?include=energy
Cnp	p56	1.039	3	http://api.brain-map.org/grid_data/download/77371841?include=energy
Comt	p56	0.094	3	http://api.brain-map.org/grid_data/download/68301371?include=energy
Cx3cl1	p56	0.730	3	http://api.brain-map.org/grid_data/download/2135?include=energy
Dcn	p56	0.644	3	http://api.brain-map.org/grid_data/download/329?include=energy
Dlk1	p56	0.553	3	http://api.brain-map.org/grid_data/download/71587885?include=energy
Drd2	p56	0.348	3	http://api.brain-map.org/grid_data/download/357?include=energy
Egr1	p56	1.090	3	http://api.brain-map.org/grid_data/download/71247672?include=energy
Eomes	p56	-0.839	3	http://api.brain-map.org/grid_data/download/80516770?include=energy
Epas1	p56	0.212	3	http://api.brain-map.org/grid_data/download/69015278?include=energy
Fgfr1	p56	0.804	3	http://api.brain-map.org/grid_data/download/79588290?include=energy
Fos	p56	1.102	3	http://api.brain-map.org/grid_data/download/459?include=energy
Gad1	p56	0.692	3	http://api.brain-map.org/grid_data/download/479?include=energy
Gad2	p56	0.471	3	http://api.brain-map.org/grid_data/download/79903740?include=energy
Gpr37	p56	-0.489	3	http://api.brain-map.org/grid_data/download/2271?include=energy
Gria3	p56	1.021	3	http://api.brain-map.org/grid_data/download/69257701?include=energy
Gria4	p56	1.181	3	http://api.brain-map.org/grid_data/download/71358628?include=energy
Grin2b	p56	0.766	3	http://api.brain-map.org/grid_data/download/74988710?include=energy
Grm5	p56	0.851	3	http://api.brain-map.org/grid_data/download/73512423?include=energy
Hif1a	p56	-0.050	3	http://api.brain-map.org/grid_data/download/75828189?include=energy
Icam5	p56	-0.050	3	http://api.brain-map.org/grid_data/download/73520989?include=energy
Inpp4b	p56	0.781	3	http://api.brain-map.org/grid_data/download/72080054?include=energy
Lepr	p56	-0.131	3	http://api.brain-map.org/grid_data/download/73930822?include=energy
Lrrk2	p56	-0.092	3	http://api.brain-map.org/grid_data/download/73497609?include=energy
Mog	p56	0.315	3	http://api.brain-map.org/grid_data/download/70946633?include=energy
Ncoa2	p56	1.010	3	http://api.brain-map.org/grid_data/download/68668090?include=energy
Nfasc	p56	0.690	3	http://api.brain-map.org/grid_data/download/68844086?include=energy
Ngf	p56	1.148	3	http://api.brain-map.org/grid_data/download/74581384?include=energy
Nr3c1	p56	-0.929	3	http://api.brain-map.org/grid_data/download/727?include=energy
Nr4a1	p56	0.496	3	http://api.brain-map.org/grid_data/download/1792?include=energy
Ostf1	p56	0.640	3	http://api.brain-map.org/grid_data/download/73907505?include=energy
Plp1	p56	1.161	3	http://api.brain-map.org/grid_data/download/75457514?include=energy
Pomc	p56	0.964	3	http://api.brain-map.org/grid_data/download/2493?include=energy
Prlr	p56	0.461	3	http://api.brain-map.org/grid_data/download/1268?include=energy
Prnp	p56	0.708	3	http://api.brain-map.org/grid_data/download/1405?include=energy
Rac2	p56	0.006	3	http://api.brain-map.org/grid_data/download/79534949?include=energy
Rora	p56	0.387	3	http://api.brain-map.org/grid_data/download/2537?include=energy
Rreb1	p56	1.013	3	http://api.brain-map.org/grid_data/download/72340134?include=energy
S100b	p56	1.128	3	http://api.brain-map.org/grid_data/download/923?include=energy
Slc1a2	p56	0.810	3	http://api.brain-map.org/grid_data/download/69873839?include=energy
Smad4	p56	0.824	3	http://api.brain-map.org/grid_data/download/71656698?include=energy
Sorl1	p56	1.086	3	http://api.brain-map.org/grid_data/download/74511924?include=energy
Sox10	p56	-0.262	3	http://api.brain-map.org/grid_data/download/75694718?include=energy
Sox2	p56	-0.600	3	http://api.brain-map.org/grid_data/download/77280331?include=energy
Spp1	p56	0.904	3	http://api.brain-map.org/grid_data/download/70436740?include=energy
Stat1	p56	0.952	3	http://api.brain-map.org/grid_data/download/2635?include=energy
Stat5b	p56	0.076	3	http://api.brain-map.org/grid_data/download/71016584?include=energy
Syn1	p56	1.116	3	http://api.brain-map.org/grid_data/download/71724559?include=energy
Syt1	p56	0.835	3	http://api.brain-map.org/grid_data/download/1014?include=energy
Tac1	p56	0.671	3	http://api.brain-map.org/grid_data/download/1038?include=energy
Tac2	p56	0.333	3	http://api.brain-map.org/grid_data/download/72339556?include=energy
Tef	p56	0.401	3	http://api.brain-map.org/grid_data/download/393201?include=energy
Trp63	p56	0.670	3	http://api.brain-map.org/grid_data/download/69290692?include=energy
Vip	p56	0.257	3	http://api.brain-map.org/grid_data/download/77371835?include=energy
Adam17	p56	1.435	4	http://api.brain-map.org/grid_data/download/75988237?include=energy
Agtr1a	p56	1.396	4	http://api.brain-map.org/grid_data/download/2021?include=energy
Ahr	p56	1.323	4	http://api.brain-map.org/grid_data/download/71380375?include=energy
Akt1	p56	1.055	4	http://api.brain-map.org/grid_data/download/75988255?include=energy
Apod	p56	1.244	4	http://api.brain-map.org/grid_data/download/71380893?include=energy
Apoe	p56	1.244	4	http://api.brain-map.org/grid_data/download/79490110?include=energy
Ar	p56	1.495	4	http://api.brain-map.org/grid_data/download/2039?include=energy
Arntl	p56	1.411	4	http://api.brain-map.org/grid_data/download/2045?include=energy
Ascl2	p56	1.499	4	http://api.brain-map.org/grid_data/download/1156?include=energy
Atf3	p56	1.091	4	http://api.brain-map.org/grid_data/download/70814344?include=energy
Bche	p56	1.441	4	http://api.brain-map.org/grid_data/download/74881889?include=energy
Bcl2	p56	1.247	4	http://api.brain-map.org/grid_data/download/69529274?include=energy
Bmp5	p56	1.216	4	http://api.brain-map.org/grid_data/download/73817428?include=energy
Brca1	p56	0.976	4	http://api.brain-map.org/grid_data/download/75988313?include=energy
Cd47	p56	0.837	4	http://api.brain-map.org/grid_data/download/357100?include=energy
Cdh17	p56	1.073	4	http://api.brain-map.org/grid_data/download/69838100?include=energy
Cdx2	p56	1.401	4	http://api.brain-map.org/grid_data/download/75934932?include=energy
Chat	p56	1.482	4	http://api.brain-map.org/grid_data/download/252?include=energy
Cnr1	p56	1.078	4	http://api.brain-map.org/grid_data/download/283?include=energy
Cnr2	p56	1.319	4	http://api.brain-map.org/grid_data/download/2111?include=energy
Cst3	p56	1.475	4	http://api.brain-map.org/grid_data/download/69120555?include=energy
Cux2	p56	1.228	4	http://api.brain-map.org/grid_data/download/72128748?include=energy
Ddit3	p56	1.496	4	http://api.brain-map.org/grid_data/download/564779?include=energy
Erg	p56	1.229	4	http://api.brain-map.org/grid_data/download/77464970?include=energy
Esr1	p56	1.354	4	http://api.brain-map.org/grid_data/download/79591677?include=energy
Esr2	p56	1.482	4	http://api.brain-map.org/grid_data/download/70303552?include=energy
Evi5	p56	1.476	4	http://api.brain-map.org/grid_data/download/70565545?include=energy
Fas	p56	1.410	4	http://api.brain-map.org/grid_data/download/69981151?include=energy
Foxp3	p56	1.494	4	http://api.brain-map.org/grid_data/download/69289004?include=energy
Gabpa	p56	1.189	4	http://api.brain-map.org/grid_data/download/77455038?include=energy
Gabra3	p56	1.379	4	http://api.brain-map.org/grid_data/download/70562043?include=energy
Galr2	p56	1.353	4	http://api.brain-map.org/grid_data/download/80517343?include=energy
Grm4	p56	1.495	4	http://api.brain-map.org/grid_data/download/71247631?include=energy
Hap1	p56	0.843	4	http://api.brain-map.org/grid_data/download/72007561?include=energy
Hoxb4	p56	1.407	4	http://api.brain-map.org/grid_data/download/76081918?include=energy
Hrh3	p56	1.324	4	http://api.brain-map.org/grid_data/download/73636034?include=energy
Hspd1	p56	1.429	4	http://api.brain-map.org/grid_data/download/69169385?include=energy
Igf1r	p56	1.102	4	http://api.brain-map.org/grid_data/download/69735263?include=energy
Il16	p56	1.391	4	http://api.brain-map.org/grid_data/download/71064290?include=energy
Il6st	p56	1.026	4	http://api.brain-map.org/grid_data/download/69735431?include=energy
Itln1	p56	1.415	4	http://api.brain-map.org/grid_data/download/81655576?include=energy
Kcnh7	p56	1.411	4	http://api.brain-map.org/grid_data/download/70562103?include=energy
Lrp1	p56	1.026	4	http://api.brain-map.org/grid_data/download/69736244?include=energy
Mapt	p56	1.354	4	http://api.brain-map.org/grid_data/download/79556696?include=energy
Mbp	p56	1.295	4	http://api.brain-map.org/grid_data/download/112202838?include=energy
Mef2b	p56	1.114	4	http://api.brain-map.org/grid_data/download/68745558?include=energy
Mmp9	p56	1.498	4	http://api.brain-map.org/grid_data/download/68745402?include=energy
Msc	p56	1.119	4	http://api.brain-map.org/grid_data/download/69289411?include=energy
Mst1r	p56	1.463	4	http://api.brain-map.org/grid_data/download/71020845?include=energy
Nfatc2	p56	1.465	4	http://api.brain-map.org/grid_data/download/70437816?include=energy
Nos3	p56	1.068	4	http://api.brain-map.org/grid_data/download/75831750?include=energy
Notch1	p56	0.969	4	http://api.brain-map.org/grid_data/download/70593326?include=energy
Nr0b2	p56	1.087	4	http://api.brain-map.org/grid_data/download/77454633?include=energy
Nr1i2	p56	1.093	4	http://api.brain-map.org/grid_data/download/71325288?include=energy
Nr4a2	p56	1.443	4	http://api.brain-map.org/grid_data/download/732?include=energy
P2rx4	p56	1.500	4	http://api.brain-map.org/grid_data/download/75551474?include=energy
Pik3cb	p56	1.443	4	http://api.brain-map.org/grid_data/download/70300792?include=energy
Pou2af1	p56	1.155	4	http://api.brain-map.org/grid_data/download/68743739?include=energy
Ppara	p56	1.121	4	http://api.brain-map.org/grid_data/download/77454629?include=energy
Pparg	p56	1.437	4	http://api.brain-map.org/grid_data/download/2496?include=energy
Prkcb	p56	1.280	4	http://api.brain-map.org/grid_data/download/72129247?include=energy
Ptgs2	p56	1.459	4	http://api.brain-map.org/grid_data/download/71016664?include=energy
Pthlh	p56	1.123	4	http://api.brain-map.org/grid_data/download/73592531?include=energy
Ptprc	p56	1.478	4	http://api.brain-map.org/grid_data/download/71378437?include=energy
Pvalb	p56	1.281	4	http://api.brain-map.org/grid_data/download/75496597?include=energy
Rela	p56	1.485	4	http://api.brain-map.org/grid_data/download/2523?include=energy
Ros1	p56	1.499	4	http://api.brain-map.org/grid_data/download/69818281?include=energy
S1pr1	p56	1.052	4	http://api.brain-map.org/grid_data/download/75043142?include=energy
Scn10a	p56	1.500	4	http://api.brain-map.org/grid_data/download/69288285?include=energy
Sgk1	p56	1.056	4	http://api.brain-map.org/grid_data/download/79903782?include=energy
Shh	p56	1.357	4	http://api.brain-map.org/grid_data/download/1418?include=energy
Slc1a1	p56	1.315	4	http://api.brain-map.org/grid_data/download/71836888?include=energy
Slc9a9	p56	1.196	4	http://api.brain-map.org/grid_data/download/71670730?include=energy
Smad7	p56	1.278	4	http://api.brain-map.org/grid_data/download/69290501?include=energy
Sort1	p56	1.466	4	http://api.brain-map.org/grid_data/download/69288327?include=energy
Stat3	p56	1.421	4	http://api.brain-map.org/grid_data/download/2637?include=energy
Stat5a	p56	0.811	4	http://api.brain-map.org/grid_data/download/77413690?include=energy
Tdo2	p56	1.230	4	http://api.brain-map.org/grid_data/download/71717618?include=energy
Tgfb3	p56	1.478	4	http://api.brain-map.org/grid_data/download/2667?include=energy
Tlr2	p56	0.952	4	http://api.brain-map.org/grid_data/download/77792773?include=energy
Trp73	p56	1.468	4	http://api.brain-map.org/grid_data/download/73929579?include=energy
Tspan31	p56	1.071	4	http://api.brain-map.org/grid_data/download/70473993?include=energy
Tyro3	p56	1.490	4	http://api.brain-map.org/grid_data/download/69201612?include=energy
Ubtf	p56	1.310	4	http://api.brain-map.org/grid_data/download/74988738?include=energy
Vav2	p56	0.915	4	http://api.brain-map.org/grid_data/download/74583149?include=energy
Zeb1	p56	1.483	4	http://api.brain-map.org/grid_data/download/71380441?include=energy
Zic1	p56	1.458	4	http://api.brain-map.org/grid_data/download/72103843?include=energy

Supplementary Table 7: Disease ontology terms enriched in genes preferentially expressed in brain regions highly-sensitive to immune dysfunction.

Disease	P-value	FDR q-value	Genes
Parkinson Disease	1.05E-09	1.01E-05	[illegible]
Parkinsonian Disorders	7.58E-10	1.01E-05	[illegible]
Neurodegenerative Disorders	2.89E-09	1.86E-05	[illegible]
Alzheimer's Disease	4.25E-09	2.05E-05	[illegible]
Leukodystrophy	1.47E-07	5.66E-04	[illegible]
Familial (FPAH)	2.28E-07	7.32E-04	[illegible]
Dystonia	4.33E-07	1.19E-03	[illegible]
nervous system disorder	6.86E-07	1.65E-03	[illegible]
Epilepsy	1.40E-06	3.01E-03	[illegible]
Renal Cell Carcinoma	3.96E-06	7.64E-03	[illegible]
Dysarthria	5.50E-06	9.64E-03	[illegible]
Animal Mammary Neoplasms	8.72E-06	1.21E-02	[illegible]
ANOPHTHALMIA AND PULMONARY HYPOPLASIA	1.18E-05	1.21E-02	[illegible]
Decreased nerve conduction velocity	9.62E-06	1.21E-02	[illegible]
Glioblastoma Multiforme	1.11E-05	1.21E-02	[illegible]
Mental Depression	8.15E-06	1.21E-02	[illegible]
Pain	1.08E-05	1.21E-02	[illegible]
Pancreatic Neoplasm	1.19E-05	1.21E-02	[illegible]
Tumor Cell Invasion	1.10E-05	1.21E-02	[illegible]
Carcinoma	1.41E-05	1.36E-02	[illegible]
Glioblastoma	1.49E-05	1.37E-02	[illegible]
Dementia	1.73E-05	1.52E-02	[illegible]
Mammary Carcinoma, Animal	1.82E-05	1.52E-02	[illegible]
Multiple Sclerosis	1.90E-05	1.53E-02	[illegible]
Tremor	2.03E-05	1.57E-02	[illegible]
Psychotic Disorders	2.49E-05	1.85E-02	[illegible]
Aganglionosis, Colonic	3.52E-05	2.20E-02	[illegible]
Deglutition Disorders	3.28E-05	2.20E-02	[illegible]
Malignant neoplasm of ovary	3.38E-05	2.20E-02	[illegible]
Polyneuropathy	3.53E-05	2.20E-02	[illegible]
Schizophrenia	3.50E-05	2.20E-02	[illegible]
Depressive disorder	4.12E-05	2.48E-02	[illegible]
Conventional (Clear Cell) Renal Cell Carcinoma	4.43E-05	2.52E-02	[illegible]
Schizophreniform Disorders	4.44E-05	2.52E-02	[illegible]
ovarian neoplasm	4.89E-05	2.69E-02	[illegible]
Neoplasms	5.71E-05	3.05E-02	[illegible]
Peripheral Nervous System Diseases	5.86E-05	3.05E-02	[illegible]
Dystonia Disorders	6.18E-05	3.13E-02	[illegible]
Amyloidosis	6.66E-05	3.29E-02	[illegible]
Adult Glioblastoma	7.76E-05	3.48E-02	[illegible]
Ataxia	7.74E-05	3.48E-02	[illegible]
Childhood Glioblastoma	7.28E-05	3.48E-02	[illegible]
Neurologic Symptoms	7.60E-05	3.48E-02	[illegible]
Carcinoma, Spindle-Cell	8.92E-05	3.74E-02	[illegible]
Leukoencephalopathy	8.91E-05	3.74E-02	[illegible]
Talipes cavus	8.63E-05	3.74E-02	[illegible]
Migraine with Aura	9.31E-05	3.82E-02	[illegible]
Carcinomatosis	9.74E-05	3.91E-02	[illegible]
Depressed mood	1.10E-04	4.07E-02	[illegible]
Impaired cognition	1.10E-04	4.07E-02	[illegible]
Lung Neoplasms	1.07E-04	4.07E-02	[illegible]
Mood swings	1.07E-04	4.07E-02	[illegible]
Collecting Duct Carcinoma of the Kidney	1.17E-04	4.25E-02	[illegible]

Supplementary Table 8: Gene ontology terms enriched in genes preferentially expressed in brain regions highly-sensitive to immune dysfunction.

GO term	Description	P-value	FDR q-value	Genes
GO:0050877	nervous system process	7.21E-12	1.12E-07	Npas3, Myh14, Ntrk1, Olfr143, Olfr1324, Olfr1271, Olfr125, Ntsr1, Abl1, Gabrq, Olfr653, Cbr3, Olfr981, Tas2r129, Olfr655, Slc6a3, Olfr629, Olfr640, Tas2r121, Ace, [illegible]
GO:0003008	system process	1.00E-10	7.74E-07	Myh14, Olfr143, Gsn, Olfr1271, Abl1, Gabrq, Olfr653, Tas2r129, Olfr655, Slc6a3, Olfr629, Olfr640, Tas2r121, Ace, Olfr1184, Mag, Tas2r106, Olfr923, Tas2r104, [illegible]
GO:0007600	sensory perception	5.04E-10	2.60E-06	Myh14, Olfr143, Ntrk1, Olfr1324, Olfr1271, Olfr125, Olfr653, Olfr981, Tas2r129, Slc6a3, Olfr655, Olfr629, Tas2r121, Olfr640, Ace, Olfr1184, Olfr1356, Tas2r106, [illegible]
GO:0007626	locomotory behavior	2.27E-09	8.77E-06	Npas3, Penk, En1, Abca2, Inpp5f, Ntsr1, Sez6l2, Gbx1, Drd2, Drd1a, Fxn, Apoe, Slc6a3, Rasd2, Lrrtm1, Fshr, Klhl1, Uchl1, Nkx2-1, Slitrk6, Naglu, Chrna4, Alk, [illegible]
GO:0007272	ensheathment of neurons	1.53E-07	3.37E-04	Bcas1, B4galt6, Cldn11, Slc8a3, Ppard, Qk, Itgb4, Myoc, Mal, Pard3, Myo5a, Plp1, Nkx6-2, Sirt2, Gnpat, Acer3, Jam3, Ugt8a, Serinc5, Mag, Myrf, Id4, Pmp22, Mbp, [illegible]
GO:0008366	axon ensheathment	1.53E-07	3.93E-04	Bcas1, B4galt6, Cldn11, Slc8a3, Ppard, Qk, Itgb4, Myoc, Mal, Pard3, Plp1, Myo5a, Nkx6-2, Sirt2, Gnpat, Acer3, Ugt8a, Jam3, Serinc5, Mag, Myrf, Id4, Pmp22, Mbp, [illegible]
GO:0007186	G protein-coupled receptor signaling pathway	1.33E-07	4.10E-04	Rgs13, Olfr143, Olfr1324, Olfr125, Olfr1271, Pthlh, Ntsr1, Gabrq, Olfr653, Olfr981, Ntsr2, Marco, Tas2r129, Olfr655, Olfr629, Olfr640, Tas2r121, Olfr1184, Olfr1356, [illegible]
GO:0007610	behavior	2.88E-07	5.57E-04	Penk, Npas3, Myh14, Inpp5f, Ntrk1, Abca2, Gatm, Ntsr1, Drd5, Ap1s2, Sez6l2, Csnk1e, Drd2, Drd1a, Apoe, Ahcy, Slc6a3, Ace, Prkcz, Uchl1, Dach1, Musk, Slitrk6, [illegible]
GO:1903793	positive regulation of anion transport	6.81E-07	1.17E-03	Edn1, Slc36a2, Ntsr1, Il1b, Agt, Trpa1, Abat, P2ry2, [illegible]
GO:0042552	myelination	7.74E-07	1.20E-03	Bcas1, B4galt6, Slc8a3, Qk, Itgb4, Myoc, Mal, [illegible]
GO:0009636	response to toxic substance	2.50E-06	3.51E-03	Bcl2, Slc23a1, Bcl2l1, Ntrk1, Stk24, Pon2, Kcnc2, [illegible]
GO:0032501	multicellular organismal process	4.88E-06	5.81E-03	Phactr4, Ikzf1, Olfr143, Olfr1271, Abl1, Pthlh, [illegible]
GO:0044070	regulation of anion transport	4.65E-06	5.99E-03	Eprs, Ntsr1, Il1b, Agt, Agtr2, Apoe, P2ry2, Akt1, [illegible]
GO:0007608	sensory perception of smell	1.05E-05	1.09E-02	Olfr610, Olfr609, Olfr143, Olfr354, Olfr1324, Olfr76, [illegible]
GO:0015893	drug transport	9.99E-06	1.10E-02	Slc25a17, Folr1, Slc22a3, Slc25a10, Slc7a10, [illegible]
GO:0030913	paranodal junction assembly	1.32E-05	1.27E-02	Epb4.1l3, Cd9, Gnpat, Ugt8a, Nfasc, Cntnap1, [illegible]
GO:0001505	regulation of neurotransmitter levels	2.94E-05	2.67E-02	Slc22a3, Ntsr1, Rora, Prkaca, Maoa, Drd2, Drd1a, [illegible]
GO:0030534	adult behavior	4.58E-05	3.93E-02	Unc79, En1, Inpp5f, Ntsr1, Sez6l2, Drd2, Gbx1, [illegible]
GO:0090659	walking behavior	5.04E-05	4.10E-02	Glra1, Glrb, Spg11, Cend1, Kcnj10, Cacna1a, Drd2, [illegible]
GO:0007010	cytoskeleton organization	5.36E-05	4.14E-02	Phactr4, Myh14, Rhobtb2, Gsn, Abl1, Nudc, [illegible]
GO:0006928	movement of cell or subcellular component	5.71E-05	4.21E-02	Robo1, Phactr4, Myh14, Vav3, Ntrk1, Mak, Gsn, [illegible]
GO:0032890	regulation of organic acid transport	6.83E-05	4.59E-02	Edn1, Stk39, Eprs, Slc36a2, Acsl4, Ntsr1, Il1b, Agt, [illegible]
GO:0008344	adult locomotory behavior	7.20E-05	4.64E-02	Glra1, Glrb, En1, Gigyf2, Inpp5f, Ntsr1, Sncg, [illegible]
GO:2001023	regulation of response to drug	6.74E-05	4.74E-02	Stk39, Slc36a2, Galr1, Syt11, Agt, Pawr, Sncg, Drd2, Pcp4, Abat, Syt9, Gpr37, Cnr1, Foxp1, Gabbr1, Slc18a1, Adora2a, Rgs2, Hsph1, Htr1b, Itgb1, Chrna4, Chrna6, Syt3, Ngb, Chrnb2, Htr2c, Npy2r, Reg3b, Cacna1a, Rtn4, 2610018G03Rik, Psap, Ptpn11, Chrna3, Txn1, Ptpn5, Lrrk2, [illegible]
GO:0051952	regulation of amine transport	7.79E-05	4.82E-02	Stk39, Slc36a2, Syt11, Ntsr1, Agt, Il1b, Agtr2, Sncg, Drd2, Pcp4, Abat, Syt9, P2ry1, Cnr1, Gabbr1, Rab3gap1, Slc43a2, Slc15a1, Stxbp1, Slc18a1, Sv2a, Adora2a, Rgs2, Htr1b, Itgb1, Chrna4, Chrna6, Syt3, Chrnb2, Htr2c, Npy2r, Cacna1a, Avp, Rtn4, Chrna3, Ghsr, Dpysl2, Rab3a, Chga, [illegible]
GO:0032892	positive regulation of organic acid transport	8.85E-05	5.27E-02	Edn1, Itgb1, Slc36a2, Acsl6, Htr2c, Il1b, Ntsr1, Agt, Mapk9, Tnfsf11, Pla2r1, Avp, Abat, P2ry2, Gabbr1, Rab3gap1, Acsl1, Stxbp1, Dpysl2, Adora2a, Map2k6, Arl6ip1, Nr3c1, Slc12a2
GO:0010941	regulation of cell death	9.81E-05	5.62E-02	Mal, Gsn, Pip5kl1, Abl1, Ascl1, Wdr73, Pcsk9, Ace, Tomm70a, Stxbp1, Mag, Sgms1, Acvr1c, Gbe1, Madd, Aurka, Tmbim4, Zfp36, Cep63, Dbh, 2610018G03Rik, Psap, Ndufc2, Ghsr, Stat5a, Cd248, Qk, Hdac3, Ctsb, Ctsc, Hcls1, Cst3, Pawr, Adora2a, Il20ra, Adm, Bclaf1, Parp2, Uaca, [illegible]
GO:0006820	anion transport	1.05E-04	5.82E-02	Slc38a2, Slc2a2, Clcnka, Slc2a1, Slc25a17, Pitpna, Slc23a1, Abcg8, Clcn5, Slc22a3, Slc7a10, Slc25a10, Ntsr1, Gabrq, Clca5, Drd2, Apoe, Slc25a41, Clcn6, Slc10a6, B230216G23Rik, Ace, Prkcd, Slc4a2, Atp11c, Slc4a3, Slc3a1, Abcb4, Slc37a2, Acsl6, Sfxn1, Atp11a, Atp8a2, Fabp5, [illegible]
GO:0007606	sensory perception of chemical stimulus	1.17E-04	6.04E-02	Olfr610, Olfr609, Olfr143, Olfr1324, Olfr354, Olfr76, Olfr1271, Olfr125, Olfr685, Olfr656, Olfr653, Olfr981, Olfr412, Drd2, Olfr547, Tas2r129, Olfr1344, Olfr365, Olfr655, Slc6a3, Olfr616, Olfr629, Olfr522, Olfr640, Tas2r121, Reep2, Olfr1184, Olfr1356, Olfr558, Tas2r106, Olfr923, Olfr820, [illegible] Olfr1387, Olfr649, Olfr214, Olfr686, Olfr1104, Olfr123, Olfr1396, Olfr6, Olfr124
GO:0006869	lipid transport	1.15E-04	6.16E-02	Ppard, Slc2a1, Slc25a17, Abcg8, Pitpna, Abca14, Abca2, Pparg, Slc22a22, Agtr2, Drd2, Apoe, Apoh, Apod, Slc10a6, B230216G23Rik, Ace, Osbpl5, Atp11c, Got2, Apoc4, Sgpp1, Gltp, Stard4, Npc2, Abcb4, Plekha8, Pitpnc1, Gpihbp1, Acsl6, Atp11a, Atp8a2, Fabp5, Abca3, Abcd1, Acsl3, [illegible]
GO:0042069	regulation of catecholamine metabolic process	1.27E-04	6.32E-02	Tacr3, Chrnb2, Htr2c, Drd1a, Abat, Gpr37, Slc6a3, Comt, Epas1, Maob, Pde1b, Atp7a, Pnkd
GO:0009712	catechol-containing compound metabolic process	1.35E-04	6.35E-02	Sult1d1, Sult1a1, Sncaip, Kl, Agtr2, Gata3, Maoa, Dbh, Drd2, Moxd2, Slc6a3, Gpr37, Comt, Rnf180, Epas1, Ddc, Gch1, Atp7a, Hdc
GO:0099177	regulation of trans-synaptic signaling	1.41E-04	6.39E-02	Ntrk1, Abl1, Ntsr1, Drd5, Prkaca, Drd2, Drd1a, Apoe, Rab11a, N28178, Eif4a3, Stxbp1, Prkcz, Car2, Musk, Nsg1, Htr1b, Tprgl, Chrna4, Chrna6, Map1a, Htr2c, Chrnb2, Npy2r, F2r, Fabp5, Ntng1, Ckap5, Cacna1a, Dbi, Cacna2d2, Bcr, Mapk1, Chat, Ghsr, Tubb2b, Snap47, Pate4, Slc6a4, [illegible] Tacr1, Grid1, Grik3, Dgkb, Cntn2, Slc6a6, Zdhhc12, Rab3a, Nr3c1, Grm1, Edn1, Kdr, Glul, Edn3, Rims3, Nptn, Myo6, Sncg, Abat, Lgmn, Cel, Kif5b, Pnkd, Igsf11, Gip, Cdh1, Spg11, Fcgr2b, Glra3, Ppp3r1, Kcnj10, Begain, Eif4e, Chrnb3, Pmch, Efnb3, Gfap, Prkar2b, Ywhag, Grm6
GO:0006584	catecholamine metabolic process	1.35E-04	6.54E-02	Sult1d1, Sult1a1, Sncaip, Kl, Agtr2, Gata3, Maoa, Dbh, Drd2, Moxd2, Slc6a3, Gpr37, Comt, Rnf180, Epas1, Ddc, Gch1, Atp7a, Hdc
GO:0061564	axon development	1.48E-04	6.54E-02	Bcl2, L1cam, Folr1, Nefh, Plp1, Bex1, Trim32, Jun, Mag, Tnc, Nefm, Tspan2, Ddr1, Gap43, Pmp22
GO:0018958	phenol-containing compound metabolic process	2.04E-04	7.87E-02	Bcl2, Ctsb, Pmel, Sncaip, Fah, Agtr2, Myo5a, Maoa, Tph2, Drd2, Gpr37, Slc6a3, Rnf180, Gch1, Pde1b, Oca2, Ctsl, Cpq, Sult1d1, Sult1a1, Kl, Gata3, Dbh, Moxd2, Comt, Ctns, Iyd, Epas1, Ddc, Atp7a, Hdc
GO:0042053	regulation of dopamine metabolic process	2.02E-04	8.00E-02	Slc6a3, Gpr37, Comt, Tacr3, Htr2c, Chrnb2, Epas1, Maob, Pde1b, Pnkd, Drd1a, Abat
GO:0040018	positive regulation of multicellular organism growth	2.01E-04	8.18E-02	Bcl2, Gpam, Ikzf1, Pou1f1, Atp8a2, Agt, Drd2, Sh3pxd2b, Slc6a3, Flt3, Pou3f2, Ghsr, Bbs2, Pls1, Stat5a, Igf2, Sptbn4, Bbs4, Csf1
GO:0050804	modulation of chemical synaptic transmission	1.91E-04	8.21E-02	Ntrk1, Abl1, Ntsr1, Drd5, Prkaca, Drd2, Drd1a, Apoe, Rab11a, Eif4a3, N28178, Stxbp1, Prkcz, Car2, Musk, Nsg1, Htr1b, Tprgl, Chrna4, Chrna6, Htr2c, Map1a, Chrnb2, Npy2r, F2r, Ntng1, Ckap5, Cacna1a, Cacna2d2, Dbi, Mapk1, Bcr, Chat, Ghsr, Tubb2b, Snap47, Pate4, Slc6a4, Slc12a2, [illegible] Grid1, Grik3, Dgkb, Cntn2, Slc6a6, Zdhhc12, Rab3a, Nr3c1, Grm1, Edn1, Kdr, Glul, Edn3, Rims3, Myo6, Nptn, Sncg, Abat, Lgmn, Cel, Kif5b, Pnkd, Igsf11, Gip, Cdh1, Spg11, Fcgr2b, Glra3, Ppp3r1, Begain, Kcnj10, Eif4e, Chrnb3, Pmch, Efnb3, Gfap, Prkar2b, Ywhag, Grm6
GO:0002088	lens development in camera-type eye	2.01E-04	8.40E-02	Crybb2, Crybb3, Crygc, Crygd, Gje1, Six3, Gata3, Cryba1, Cryba2, Crybg3, Gja1, Cryab, Pax6, Ctns, Tgfbr2, Crygf, Cryge, Birc7, Slitrk6
GO:0046189	phenol-containing compound biosynthetic process	2.28E-04	8.61E-02	Pmel, Kl, Agtr2, Myo5a, Gata3, Tph2, Dbh, Slc6a3, Gpr37, Moxd2, Ctns, Epas1, Ddc, Gch1, Oca2, Atp7a, Hdc
GO:0051957	positive regulation of amino acid transport	2.41E-04	8.68E-02	Itgb1, Slc36a2, Htr2c, Ntsr1, Agt, Avp, Abat, Gabbr1, Rab3gap1, Stxbp1, Dpysl2, Adora2a, Nr3c1, Arl6ip1, Slc12a2
GO:0022010	central nervous system myelination	2.56E-04	8.81E-02	B4galt6, Mag, Cntn2, Myrf, Plp1, Kcnj10, Sox10, Cntnap1, Id4, Nkx6-2
GO:0044281	small molecule metabolic process	2.40E-04	8.85E-02	Fasn, Hadha, Cyp51, Surf1, Abca2, Idh3b, Cyp4a10, Ntsr1, Fah, Cyp2j6, Rora, Cbr3, Cyp2d9, Sucla2, Cyp2f2, Acadvl, Ahcy, Chchd10, Me2, Phgdh, Pcsk9, Gpx4, Aco2, Car2, Hoga1, Acox1, Fbp2, Retsat, Ptgds, Car1, Cyp2c50, Slc37a2, Tmem55b, Sfxn1, Dcn, Gsto2, Cygb, Carns1, [illegible]
GO:0007628	adult walking behavior	2.66E-04	8.95E-02	Glra1, Glrb, Cend1, Kcnj10, Cacna1a, Gbx1, Drd2, Drd1a, Cacna1c, Chat, Fxn, Efnb3, Ctns, Klhl1, Uchl1, Scn1a, Cntn2, Sptbn4, Npc1, Pmp22
GO:0032291	axon ensheathment in central nervous system	2.56E-04	9.01E-02	B4galt6, Mag, Myrf, Cntn2, Plp1, Kcnj10, Sox10, Cntnap1, Id4, Nkx6-2
GO:0046942	carboxylic acid transport	3.31E-04	1.00E-01	Slc38a2, Ppard, Slc2a1, Slc25a17, Slc23a1, Pparg, Folr1, Slc22a3, Slc25a10, Slc7a10, Slc22a22, Ntsr1, Slc25a12, Drd2, Apoe, Slc16a13, Slc10a6, B230216G23Rik, Ace, Prkcd, Slc16a12, Emb, Slc9a3r1, Slc3a1, Abcb4, Sfxn2, Acsl6, Sfxn1, Fabp5, Slc23a2, Abcd1, Acsl3, Cacna1a, Psap, [illegible]
GO:0032879	regulation of localization	3.28E-04	1.01E-01	Xpo4, Cyp51, Abca2, Gsn, Abl1, Pip5kl1, Lrsam1, Efcab1, Nup214, Nkain3, Tmsb10, Stxbp4, Coro1b, Ptbp1, Tomm70a, Pcsk9, Ace, Apoc4, Stxbp1, Acvr1c, Dach1, Wnt8a, Nsun2, Chrna4, Chrna6, Madd, Dcn, Cygb, Hc, Zfp384, Chrnb2, Elp3, Ccl28, Zfp36, Dnajb2, Gnl3l, Acsl3, [illegible] Rgs9, Rit2, Slc43a2, Slc15a1, Bbs2, Trpm2, Sv2a, Kif5b, Rgs2, Pnkd, Csf1, Gclc, Hip1r, Arap3, Gip, Tmsb15l, Oaz1, Rabgap1l, Cltc, Kcnj12, Kcnj10, Kcnj2, Cryab, Gja1, Gfap, Scn4b, Rfx6, Dpysl2, B4galt1, Ocm
GO:0002175	protein localization to paranode region of axon	3.27E-04	1.03E-01	Epb4.1l3, Ugt8a, Mal, Nfasc, Cntnap1
GO:0001504	neurotransmitter uptake	3.65E-04	1.03E-01	Slc29a1, Slc22a3, Slc17a6, Slc6a9, Ntsr1, Atp1a2, Kcnj10, Slc1a7, Slc6a11, Slc7a11, Slc6a3, Slc6a2, Slc18a2, Slc18a1, Slc6a4, Grm1
GO:0032222	regulation of synaptic transmission, cholinergic	3.95E-04	1.03E-01	Tac1, Ngfr, Chrna3, Ifng, Htr2c, Tacr1, Adora2a, Musk, Cacna1a
GO:0098700	neurotransmitter loading into synaptic vesicle	3.23E-04	1.04E-01	Slc18a3, Slc32a1, Slc17a8, Slc18a2, Slc18a1, Slc17a6, Ddc
GO:0007165	signal transduction	3.64E-04	1.04E-01	Phactr4, Aatk, Olfr143, Olfr1271, Abl1, Pthlh, Plch1, Diras1, Coro1b, Olfr655, Olfr629, Ache, Olfr640, Olfr1184, Acvr1c, Ptf1a, Nek11, Ptger2, Olfr923, Csnk1g3, Chrna4, Chrna6, Chrnb2, Rnd3, Zfp36, Neurl2, Glra2, Psap, Ghsr, Acvrl1, Olfr1395, Ric8b, Dand5, Adcy7, Olfr131, Rassf9, [illegible]
GO:0015849	organic acid transport	3.76E-04	1.04E-01	Slc38a2, Ppard, Slc2a1, Slc25a17, Slc23a1, Pparg, Folr1, Slc22a3, Slc25a10, Slc7a10, Slc22a22, Ntsr1, Slc25a12, Drd2, Apoe, Slc16a13, Slc10a6, B230216G23Rik, Ace, Prkcd, Slc16a12, Emb, Slc9a3r1, Slc3a1, Abcb4, Sfxn2, Acsl6, Sfxn1, Fabp5, Slc23a2, Abcd1, Acsl3, Cacna1a, Psap, [illegible]
GO:0035813	regulation of renal sodium excretion	3.84E-04	1.04E-01	Edn1, Tac1, Tacr1, Agt, Agtr2, Drd2, Avp, Anpep, Comt, B230216G23Rik, Ace, Adora2a, Corin
GO:0007212	dopamine receptor signaling pathway	3.89E-04	1.04E-01	Gnb5, Adcy5, Caly, Drd5, Drd2, Drd1a, Rgs9, Oprm1, Gnal, Htt, Gnao1, Slc9a3r1, Nsg2, Nsg1
GO:0032224	positive regulation of synaptic transmission, cholinergic	3.23E-04	1.06E-01	Tac1, Ngfr, Ifng, Htr2c, Tacr1, Adora2a, Musk
GO:0009713	catechol-containing compound biosynthetic process	3.63E-04	1.06E-01	Moxd2, Slc6a3, Gpr37, Epas1, Kl, Gch1, Ddc, Agtr2, Gata3, Atp7a, Dbh, Hdc
GO:0042423	catecholamine biosynthetic process	3.63E-04	1.08E-01	Gpr37, Slc6a3, Moxd2, Epas1, Kl, Ddc, Gch1, Agtr2, Gata3, Atp7a, Dbh, Hdc
GO:0040012	regulation of locomotion	4.22E-04	1.09E-01	Robo1, Stk24, Abl1, Pip5kl1, Efcab1, Tmsb10, Igsf10, Coro1b, Ace, Fbln1, Acvr1c, Dach1, Dcn, Elp3, Hc, Ccl28, Cygb, F2r, Slc24a6, Wdr44, 2610018G03Rik, Ghsr, Acta2, Prcp, F2rl1, Acvrl1, Stat5a, Prr5l, Scrt1, Itga2, Tfap2a, Itga5, Kank1, Itga3, Rap2c, Pawr, Osbpl8, Adamts1, Il33, Evl, [illegible]
GO:0019233	sensory perception of pain	5.25E-04	1.18E-01	Edn1, Penk, Ntrk1, Ednra, Trpa1, P2ry2, P2ry1, Fam134b, Cnr1, Aqp1, Ace, Uchl1, Mme, Ndn, Tac1, Ngfr, Gip, Chrna4, Ccr2, Chrnb2, Tacr1, Mapk3, Cacna1a, Aloxe3, Mapk1, Oprm1, Oprl1, Grm1
GO:2000377	regulation of reactive oxygen species metabolic process	5.23E-04	1.19E-01	Bcl2, Slc25a33, Slc5a3, Il1b, Agt, Plin5, Sirt2, Tlr6, Hspd1, Akt1, Hsp90aa1, Prkcd, Nox4, Gch1, Acp5, Ddah1, Cxcl1, Klf2, Foxm1, Aldh2, Mapk9, Abcd1, Smpd3, Mmp8, Wdr35, Bco2, Pdgfrb, Bcr, Coq7, Ndufc2, Oprm1, Cd36, Pikfyve, Cd47, Prcp, H60a, F2rl1, Agxt2, Tfap2a, Mycn, Eef1a1, [illegible]
GO:0033238	regulation of cellular amine metabolic process	5.06E-04	1.20E-01	Tacr3, Htr2c, Chrnb2, Ins2, Cacna1a, Drd1a, Abat, Slc7a11, Gpr37, Slc6a3, Comt, Fshr, Epas1, Maob, Pde1b, Atp7a, Pnkd
GO:0006836	neurotransmitter transport	5.21E-04	1.20E-01	Edn1, Slc29a1, Slc36a2, Slc32a1, Slc5a7, Ywhaz, Ednra, Rims3, Slc22a3, Slc6a9, Slc6a13, Slc17a6, Ntsr1, Slc25a12, Slc1a7, Prkaca, Slc6a11, Slc6a3, Slc7a13, Aqp1, Slc6a2, Slc6a20a, Stxbp1, Slc18a1, Cplx2, Sv2a, Slc44a1, Slc9a3r1, Slc3a1, Slc25a13, Xbp1, Cplx1, Sv2c, Atp1a2, [illegible]
GO:0098801	regulation of renal system process	5.06E-04	1.22E-01	Edn1, Tac1, Tacr1, F2r, Agt, Agtr2, Drd2, Avp, Anpep, [illegible]
GO:0017144	drug metabolic process	5.19E-04	1.22E-01	Cyp2j9, Surf1, Idh3b, Gatm, Cyp4a10, Hspa8, [illegible]
GO:0043270	positive regulation of ion transport	4.87E-04	1.23E-01	Cd4, Prss8, Kcnc2, Agt, Il1b, Ntsr1, Abl1, Drd2, [illegible]
GO:0040013	negative regulation of locomotion	5.03E-04	1.23E-01	Bcl2, Robo1, Ppard, Srgap2, Mid2, Jag1, Pparg, [illegible]
GO:0055085	transmembrane transport	4.99E-04	1.25E-01	Abca2, Slc25a10, Ntsr1, Gabrq, Slc22a15, Slc6a3, [illegible]
GO:0043648	dicarboxylic acid metabolic process	5.73E-04	1.27E-01	Idh3b, Glul, Folr1, Gad2, Sucla2, Me2, Got1, [illegible]
GO:0032233	positive regulation of actin filament bundle assembly	6.36E-04	1.31E-01	Id1, Myoc, Lpar1, Abl1, Wnt11, Limk1, Sh3pxd2b, [illegible]
GO:0051954	positive regulation of amine transport	6.34E-04	1.32E-01	Itgb1, Slc36a2, Htr2c, Chrnb2, Npy2r, Ntsr1, Agt, [illegible]
GO:0035094	response to nicotine	6.20E-04	1.33E-01	Chrnb3, Bcl2, Chrna4, Nfkb1, Cnr1, Ntrk1, Chrnb2, [illegible]
GO:0015711	organic anion transport	6.26E-04	1.33E-01	Slc38a2, Slc2a2, Slc2a1, Slc25a17, Slc23a1, [illegible]
GO:1901566	organonitrogen compound biosynthetic process	6.15E-04	1.34E-01	Abca2, Eif1, Phgdh, Slc6a3, Eif3f, Extl3, Sgms1, [illegible]
GO:0031175	neuron projection development	6.69E-04	1.36E-01	Dip2a, Bcl2, Ntrk1, Folr1, Gprin3, Rasgrf1, Triobp, [illegible]
GO:0060548	negative regulation of cell death	6.76E-04	1.36E-01	Card14, Ntrk1, Abl1, Ntsr1, Ascl1, Wdr73, Pde3a, [illegible]
GO:0048468	cell development	7.23E-04	1.43E-01	Robo1, Fasn, Ntrk1, Pou1f1, Pthlh, Pitx2, Tjp3, [illegible]
GO:0051955	regulation of amino acid transport	7.44E-04	1.44E-01	Htr1b, Stk39, Itgb1, Slc36a2, Htr2c, Agt, Il1b, Ntsr1, [illegible]
GO:0007017	microtubule-based process	7.41E-04	1.45E-01	Mak, Hspa8, Poc1b, Nudc, Wdr73, Dnaic2, Ccp110, [illegible]
GO:0030030	cell projection organization	7.68E-04	1.47E-01	Vav3, Mak, Ntrk1, Gsn, Poc1b, Abl1, Dnaic2, [illegible]
GO:0065008	regulation of biological quality	7.85E-04	1.48E-01	Abca2, Abl1, Pthlh, Steap1, Tmsb10, Acadvl, [illegible]
GO:2000145	regulation of cell motility	7.93E-04	1.48E-01	Robo1, Stk24, Abl1, Pip5kl1, Efcab1, Tmsb10, [illegible]
GO:0042221	response to chemical	8.43E-04	1.55E-01	Vav3, Man1a, Gsn, Prdm2, Abl1, Cyp2f2, Stxbp4, [illegible]
GO:0006811	ion transport	8.74E-04	1.59E-01	Abcg8, Tmem63a, Mmgt1, Slc25a10, Ntsr1, Gabrq, [illegible]
GO:0030334	regulation of cell migration	8.97E-04	1.59E-01	Robo1, Stk24, Abl1, Pip5kl1, Tmsb10, Igsf10, [illegible]
GO:0099612	protein localization to axon	8.88E-04	1.60E-01	Epb4.1l3, Ugt8a, Mal, Nfasc, Cope, Cntn2, Cntnap1, [illegible]
GO:0030029	actin filament-based process	9.58E-04	1.63E-01	Phactr4, Myh14, Amotl1, Gsn, Abl1, Tmsb10, [illegible] Apbb1, Sun2, Rhou, Micalcl, Palld, Ablim2
GO:0048484	enteric nervous system development	9.42E-04	1.64E-01	Phactr4, Sox8, Arx, Ednra, Rhox10, Phox2b, Ascl1, [illegible]
GO:0015908	fatty acid transport	9.57E-04	1.64E-01	Ppard, Slc2a1, Crot, Slc25a17, Acsl6, Slco2a1, [illegible]
GO:0042391	regulation of membrane potential	9.84E-04	1.65E-01	Myh14, Pip5kl1, Ntsr1, Gabrq, Drd1a, Ntsr2, [illegible]
GO:0048265	response to pain	9.42E-04	1.66E-01	Tac1, Comt, Slc6a2, Tacr1, Gch1, Calca, Trpa1, [illegible]
